# Intrinsically disordered proteins (IDPs) in trypanosomatids

**DOI:** 10.1186/1471-2164-15-1100

**Published:** 2014-12-13

**Authors:** Patrícia de Cássia Ruy, Raul Torrieri, Juliano Simões Toledo, Viviane de Souza Alves, Angela Kaysel Cruz, Jeronimo Conceição Ruiz

**Affiliations:** Informática de Biossistemas, Centro de Pesquisas René Rachou – Fundação Oswaldo Cruz (FIOCRUZ), Belo Horizonte, MG Brasil; Faculdade de Medicina de Ribeirão Preto, Universidade de São Paulo, Ribeirão Preto, SP Brasil; Instituto de Ciências Biológicas, Universidade Federal de Minas Gerais, Belo Horizonte, MG Brasil

**Keywords:** Intrinsically disordered proteins (IDPs), Trypanosomatids, IDP prediction

## Abstract

**Background:**

Proteins are composed of one or more amino acid chains and exhibit several structure levels. IDPs (intrinsically disordered proteins) represent a class of proteins that do not fold into any particular conformation and exist as dynamic ensembles in their native state. Due to their intrinsic adaptability, IDPs participate in many regulatory biological processes, including parasite immune escape. Using the information from trypanosomatids proteomes, we developed a pipeline for the identification, characterization and analysis of IDPs. The pipeline employs six disorder prediction methodologies and integrates structural and functional annotation information, subcellular location prediction and physicochemical properties. At the core of the IDP pipeline, there is a relational database that describes the protein disorder knowledge in a logically consistent manner.

**Results:**

The results obtained from the IDP pipeline showed that *Leishmania* and *Trypanosoma* species have approximately 70% and 55% IDPs, respectively. Our results indicate that IDPs in trypanosomatids contain disorder-promoting amino acids and order-promoting amino acids. The functional annotation analysis demonstrated enrichment of selected Gene Ontology terms. A relevant association was observed between the disordered residue numbers within predicted IDPs and their subcellular location, lack of transmembrane domains and lack of predicted function. We validated our computational findings with 2D electrophoresis designed for IDP identification and found that 100% of the identified protein spots were predicted *in silico*.

**Conclusions:**

Because there is no pipeline or database addressing IDPs in trypanosomatids, the pipeline described here represents the first attempt to establish possible correlations between protein function and structural disorder in these eukaryotes. Interestingly, all significant associations detected in the contingency analysis were observed when the protein disorder content reached approximately 40%. The exploratory data analysis allowed us to develop hypotheses regarding the IDPs’ association with key biological features of these parasites, including transcription and transcriptional regulation, RNA processing and splicing, and cytoskeleton.

**Electronic supplementary material:**

The online version of this article (doi:10.1186/1471-2164-15-1100) contains supplementary material, which is available to authorized users.

## Background

### Intrinsically disordered proteins

The traditional view of protein structure/function connection holds as one of its principles the concept that the biological function of a protein is critically dependent on a well-defined 3D conformational structure.

Despite the existence of studies that date back over 20 years [1, 2] demonstrating the existence of proteins or protein domains without a defined structure, the generality of this phenomenon was reported in 1999 [3] and has only recently been extensively studied (see Additional file 1).

In the early 1990s, the existence of functionally active proteins lacking a stable conformation under physiological conditions was evidenced by several studies. These proteins, currently known as IDPs (intrinsically disordered proteins), were identified in both prokaryotes and eukaryotes, including mammals. Some IDPs are either fully disordered or completely structured, but most have intrinsically disordered regions (IDRs) that comprise unstructured segments whose amino acid composition prevents autonomous folding [4].

Only 32% of the crystallized structures available in the PDB (Protein Data Bank) [5] are completely devoid of disorder, indicating that a stable 3D fold represents the exception rather than the rule [6]. Some authors have even suggested that intrinsic protein disorder may require a reassessment of the protein-structure-function paradigm [7].

The number of IDPs and IDRs in various proteomes is very large. For example, nearly 75% of the signaling proteins in mammals are predicted to contain long disordered regions of more than 30 residues, approximately half of their total proteins are predicted to contain such long disordered regions, and approximately 25% of their proteins are predicted to be fully disordered. The results observed by Dunker and colleagues [8] show that for Eukaryota genomes such as *Drosophila melanogaster* and *Homo sapiens*, the percentage of proteins with contiguous disordered segments with lengths greater than 30 amino acids is 36.6% and 35.2%, respectively. In contrast, it has been reported [9] that general patterns distinguishing disordered regions and amino acid compositions present in non-parasitic protozoa such as *T. thermophila* are lacking. For instance, parasitic protozoan datasets are significantly depleted in tryptophan (W) and enriched in lysine (K) [9]. IDPs and IDRs show an amazing structural variability as well as a very wide variety of functions, although the unfoldome and unfoldomics concepts were only recently introduced [10].

A peculiar feature of IDPs is that as a consequence of their “lack” of a defined structure, they are associated with high accessibility of their polypeptide chains. Thus, this class of proteins is prone to extensive post-translational modifications, such as phosphorylation, acetylation, and ubiquitination, that can modulate their biological activities [11–13].

The growing list of features attributed to disordered regions and proteins suggests that they act as molecular rheostats to support a continuum of conformational states and transitions. These features enable IDRs and IDPs to mediate highly specific interactions with multiple binding partners [4]. IDPs require interaction/binding with other biomolecules. This process involves a disorder-to-order transition that favors the participation of IDPs in different cellular pathways, including regulatory cascades [14–16]. In this context, IDPs and IDRs play varied roles in regulating the function of their binding partners and in promoting the assembly of supramolecular complexes [10].

Interestingly, the proteins involved in host/parasite interaction processes in *Plasmodium falciparum* have been described as IDPs. The structural plasticity of IDPs allows promiscuous interactions and may contribute in different ways to parasite invasion and survival within the host, either by inhibiting the generation of effective antibody mediated-responses or by facilitating interaction with host molecules necessary for a successful infection [17].

### Model organism

The Family Trypanosomatidae includes organisms of the genera *Leishmania*, *Phytomonas*, *Crithidia*, *Blastocrithidia*, *Herpetomonas* and *Trypanosoma*. Two members of these genera are medically important (*Leishmania* and *Trypanosoma*).

The African trypanosomes (*Trypanosoma brucei*, *T. congolense* and *T. vivax*) are endemic in rural areas in sub-Saharan Africa and cause sleeping sickness in humans. This disease can be fatal if untreated [18, 19]. *Trypanosoma cruzi* is endemic in South and Central America and is the etiological agent of Chagas disease [18, 20].

Parasites of the genus *Leishmania* cause leishmaniasis, a multifaceted disease presenting a wide spectrum of clinical manifestations. In humans, disease presentation varies from self-healing cutaneous lesions to visceral disorders that may lead to death if untreated [18].

The range of drugs available for treatment is limited, and drug resistance has been observed [21].

Due to the important medical and public health aspects of trypanosomatids, this work investigated the genomic information on *L. braziliensis*, *L. major*, *L. infantum*, *L. mexicana*, *L. tarentolae*, *T. cruzi* and *T. brucei* in a wide *in silico* comparative analysis for the identification and characterization of IDPs.

### IDP prediction

Due to the functional plasticity of IDPs [3, 22–27] and the problems associated with their recognition, several different computational methodologies and analysis tools have been developed to enable the systematic identification of the structural disorder of proteins from different predicted proteomes [28].

Despite the existence of numerous algorithms, the computational identification of IDPs still represents a major challenge, mainly because of the absence of a proper consensus definition on how to identify an IDP [3, 29–33].

As general features, proteins classified as IDPs possess low-complexity regions, low hydrophobic amino acid content, and therefore highly polar characteristics and charged amino acids [34, 35]. These attributes are consistent with the inability of these proteins to adopt a globular conformation and have motivated the evolution of several algorithms designed to predict disordered regions in proteins [34, 36–40]. In addition to these features, other peculiarities have been employed in the identification of protein structural disorders, such as the preference for specific amino acids and the high variability of these amino acids in sequences [32, 37, 41]. Another major factor is related to the length of disordered regions required for identification as an IDP, as prediction accuracy is higher for long disordered regions (larger than 40 base pairs) [42].

Given the wide range of features, multifactorial computational approaches are still scarce and unable to predict all the characteristics described above *in silico*. Notably, specific methodologies have been described [43] that perform better with certain protein characteristics. Pryor et al. [43] evaluated the performance of 13 disorder predictor programs to predict disordered regions located in integral membrane proteins. As a result, it has become clear that combinatorial approaches should provide more complete answers to the problem of predicting IDPs [38].

NMR (nuclear magnetic resonance) studies have shown that the degree of disorder in proteins can vary greatly, ranging from the total absence of secondary and tertiary structure [44, 45] to the presence of partial secondary structures [3, 27, 46], which further complicates predictions. Recently, single-cell experiments with IDPs have provided new insights into the biophysics and complexity of these proteins. These studies promise to better illuminate the critical roles of the biophysics underlying protein disorder [47].

Single-molecule experiments address three broad classes of structural and functional complexity: a) the conformational features and dynamics of monomeric IDPs; b) IDP interactions with binding partners and concomitant folding; and c) the more complex behavior of IDPs, with a specific focus on binding-modulated function by interaction with multiple partners.

Due to the complexity in predicting these regions, we used the ROC (receiver operating characteristic) curve [48] methodology to identify the best combination of structural disorder predictors, which was then used in a computational pipeline applying *ab initio* techniques for genome-scale prediction in trypanosomatids.

## Methods

The IDP pipeline was developed to run in a Linux environment implemented in the Perl language using the MySQL Database Management System. Version 2.3 of the *Leishmania braziliensis*, *Leishmania major*, *Leishmania infantum*, *Trypanosoma brucei* and *Trypanosoma cruzi* proteomes and version 4.0 of the *Leishmania mexicana* and *Leishmania tarentolae* proteomes were downloaded from TriTrypDB (http://tritrypdb.org/tritrypdb/).

### IDP pipeline

Of note, protein disorder can be defined in many ways depending on the research focus and experimental method used. In this work, we considered an IDP as a given predicted protein that has a region of at least 40 consecutive disordered residues. This definition is not ideal, and this issue represents a field of intense debate that has already been reported in the paper “Assessment of protein disorder region prediction in CASP 10” [48].

**Figure 1:**
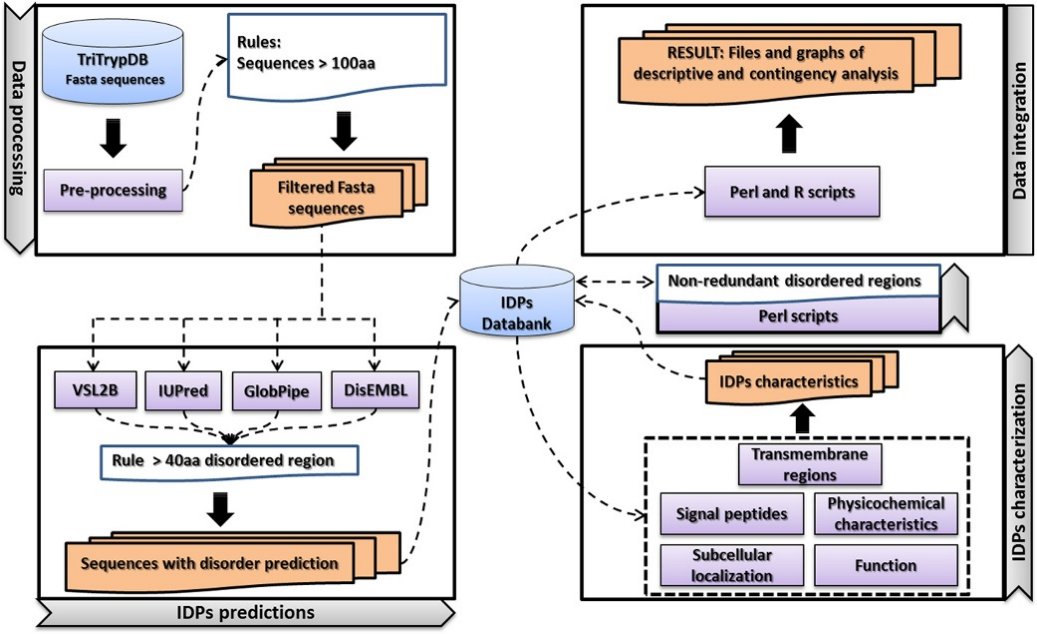
**IDP pipeline.** The IDP pipeline starts with the pre-processing of FASTA sequences with verification of the sequence length and possible annotation errors. The IDP prediction was made using four different programs. Sequences with regions of at least 40 amino acids were predicted as disordered. The identified IDPs were characterized according to different features. Next, the non-redundant disordered regions were considered for each possible combination of disorder prediction programs. Finally, the descriptive and contingency analyses were conducted. The data generated by the IDP pipeline were stored in a MySQL database for analysis during the process and for future requests.

The automated pipeline was developed employing different methods of prediction, enabling the identification and characterization of IDPs in different organisms (Figure 1). The predicted proteomes of the studied organisms were the only input to the IDP pipeline; as a result, it generated a MySQL database with IDP characterization information, two files with the analysis of the results (descriptive and contingency analysis), and a set of directories containing the outputs from each algorithm.

### Pre-processing sequences

Sequences that have possible annotation errors such as an absence of initial methionine, internal stop codons and illegal characters (*, X, B, Z and U), most likely inserted during the process of automatic annotation, were removed.

Because the prediction accuracy is higher for long disordered regions (larger than 40 base pairs), a sequence length cutoff of 100 bp (base pairs) was applied.

### IDP prediction

Disorder predictors that were publically available for download and local execution were selected. Four predictors were selected, spanning six different methodologies of disorder prediction: DisEMBL (implements three methods) [37], GlobPipe [49], IUPred [50] and VSL2B [51].

### IDP feature prediction

The Phobius algorithm [52] was used to predict transmembrane regions and signal peptides.

Physicochemical predictions were made by the program pepstats from the EMBOSS package (The European Molecular Biology Open Software Suite).

WoLF PSORT was used for the prediction of subcellular locations [53].

The functional annotation of predicted IDPs was based on the Gene Ontology vocabulary of functional classification. The program chosen to perform functional annotation was Blast2GO Pipeline Version B2G4Pipe [54].

The classification of proteins as “hypothetical” or “with predicted function” was based on sequence similarity searches using BLAST (Basic Local Alignment Search Tool) [55] against the non-redundant database (nr) of proteins from NCBI (National Center for Biotechnology Information). The *e*-*value* cutoff applied was 1.0 × 10^-6^.

### Functional enrichment analysis

The Perl library GO::TermFinder was used to identify the enrichment of functional terms in IDPs [56]. This algorithm compares genes that code for IDPs to a list of previously functionally annotated genes. A statistical test was used to check for association strength. The hypergeometric distribution and the Bonferroni correction for multiple hypotheses were employed to correct *p*-*values*. We defined 0.05 as the minimum *p*-*value* for the functional annotation of IDP genes.

### Prediction of consensus regions

The protein disorder consensus regions were obtained considering a) the overlap degree and b) the consecutiveness of the regions. In the first case, the regions were grouped, generating a single consensus region. In the second case, an overlap of the subsequent regions was necessary within a margin of 10% of their length around the region boundaries (see Additional file 2).

### Localization of protein disorder in a given protein

Each disordered region was classified by its localization along the whole protein sequence length using the following terms: a) N terminal, b) C terminal, c) intermediate, and d) spanning from the C to N end. The N and C terminal regions were defined as 15% of sequence ends. The number and percentage of disordered residues were also calculated.

### Descriptive analysis

The descriptive analysis summarizes some key information that resulted from the IDP pipeline. This analysis comprises thirteen descriptive results, including the number of proteins larger than the minimum length chosen by the user, the number of proteins that start with methionine and contain no annotation errors, and the number of IDPs predicted by the best combination of predictors. For optimal viewing of this information, graphs were generated for each of the items described above.

### Calculating amino acid frequencies

To analyze the frequency of amino acids present in disordered regions compared with globular regions, the method described by Romero and colleagues was employed [34]. For amino acid frequency estimation in globular regions, the dataset PDB_S25 version May 2010 [57], available at http://bioinfo.tg.fh-giessen.de/pdbselect/, was used. The PDB_S25 dataset consists of non-redundant protein chains, where any two chains share more than 25% sequence identity.

### Contingency analysis

The contingency analysis evaluated the association between two variables and predicted whether the frequency of related variables was above or below an expected value obtained through the likelihood ratio chi-square test [58]. We defined 0.05 as the maximum *p*-*value* to consider an association to be significant. The analysis was performed in the R environment (http://www.r-project.org/) using the VCD (Visualizing Categorical Data) package. For each found association, an association plot was created [59].

### Best combination of structural disorder predictors

To assess the performance of structural disorder prediction algorithms in trypanosomatids, individual and combined algorithm predictions were evaluated. Thus, when we refer to a predictor, we are quoting either an individual predictor or a given combination of predictors.

Considering multiple predictions, the “consensus disorder prediction” was obtained by taking into account the longest overlapped prediction region. A minimum of one amino acid overlap was required to join two different predictions.

ROC analysis [48] was performed using single regions or “consensus disorder predictions” to determine the best combination of predictors.

The positive control dataset was obtained from the Disprot database (version 4.9) (http://www.disprot.org), which stores experimental information about protein disorders. Considering that in this database, each disordered region can be identified by different experimental techniques and that experimentally validated regions can be superposed, consensus information was created to remove positive and negative prediction redundancy (see Additional file 2).

A true positive (TP) was defined every time a prediction was fully inserted (10% tolerance) within the coordinates defined by the Disprot database. A true negative (TN) was defined whenever a prediction was not generated for an ordered (structured) region. A false positive (FP) occurred every time a prediction was generated for an ordered region. Finally, a false negative (FN) was generated whenever a prediction was not generated for a disordered region (see Additional file 3). These values made it possible to calculate two essential metrics of classification performance: True Positive Rate (TPR) = TP/(TP+FN) and False Positive Rate (FPR) = FP/(FP + TN).

### Ethics statement

Experiments were performed in compliance with the Ethical Principles in Animal Research adopted by Brazilian College of Animal Experimentation (COBEA) and was approved by the College of Medicine of Ribeirão Preto of the University of São Paulo – Ethical Commission of Ethics in Animal Research (CETEA) under protocol number 118/2008.

### Experimental analysis

To validate the computational predictions, we employed two different experimental approaches described in the literature and developed specifically for IDP identification [60, 61].

The first one, Csizmók et al. (an unconventional two-dimensional electrophoresis (2-DE)), was applied to protein extracts of *L. major*. Proteins were resolved on a 7.5% polyacrylamide native gel for the first dimension (heating for 10 min at 100°C) and on a 5-20% large-format (18 × 16 cm) polyacrylamide gel containing 8 M urea for the second dimension. The two-dimensional gel was stained with colloidal Coomassie (Coomassie blue G-250) for 30 hours. The IDPs were identified near the diagonal line.

The second one, Galea et al. (an IDP enrichment methodology to protein extracts), was applied in *L. major* and *L. braziliensis* protein extracts. The purified proteins enriched by heating at 100°C for one hour were subjected to conventional 2-DE. Proteins were focalized in pH 3-5.6 (*L. major*) and pH 4-7 (*L. braziliensis*) strips (13 cm, nonlinear gradient), and SDS-PAGE was performed using 12.5% polyacrylamide gels stained with colloidal Coomassie for 30 hours.

## Results and discussion

Because these pathogenic organisms are ancestral eukaryotes, the relevance of their disordered proteins in terms of their participation in different biological processes is applicable to higher organisms. Thus, our main objective was to analyze the IDP content in trypanosomatids. Our study approach adopted the development of an automatic pipeline that can be applied not only for trypanosomatids but for any organism. The developed IDP pipeline was applied to seven trypanosomatids proteomes (*L. major*, *L. braziliensis*, *L. infantum*, *L. mexicana*, *L. tarentolae*, *T. cruzi* and *T. brucei*).

### The best combinatorial approach for IDP prediction in trypanosomatid genomes

**Figure 2:**
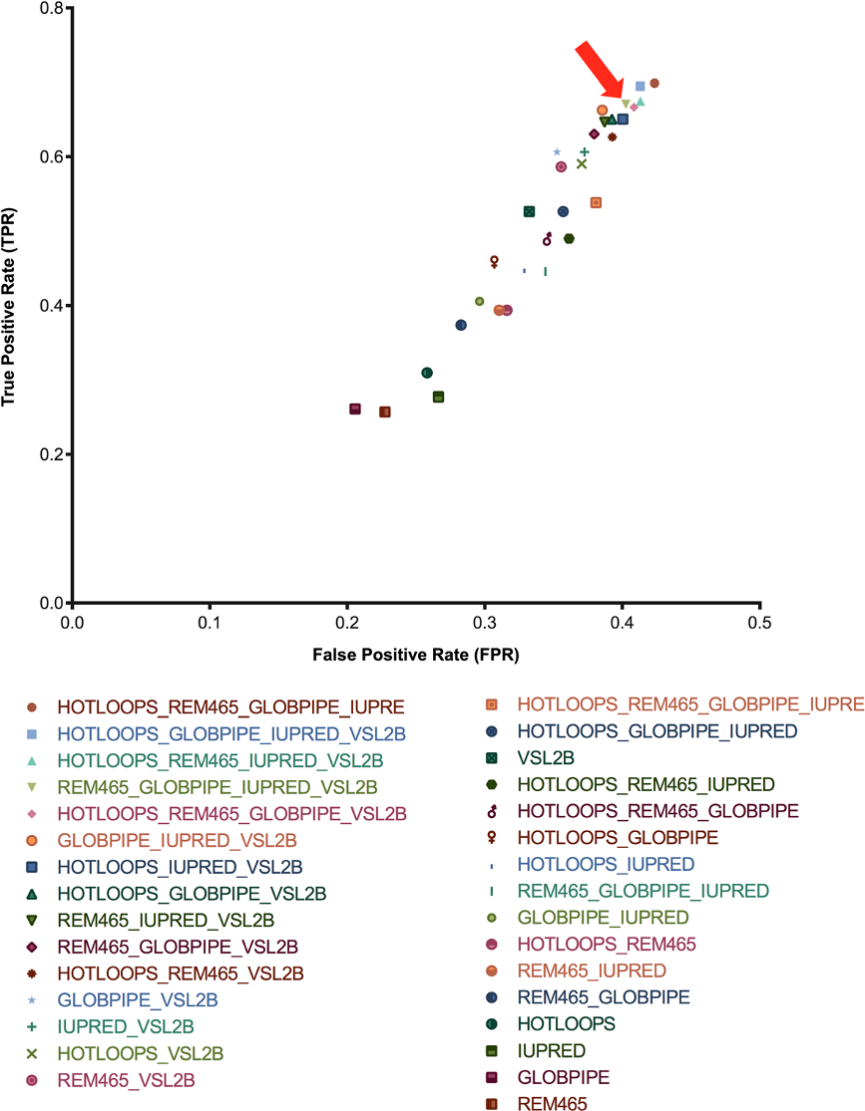
**ROC graph.** The Disprot database was used to establish the positive and negative datasets to find the most efficient algorithm combination. The TPR and FPR values of all possible combinations among the selected disordered predictors are presented. The chosen combination is indicated with a red arrow. The chosen algorithm combination for structural disorder prediction included REM465, GlobPipe, IUPred and VSL2B (True Positive Rate = 0.670 and False Positive Rate = 0.402). This particular combination presented the lowest count of false positives and a high count of false negatives among the five best predictor combinations.

As discussed above, a number of approaches can be used to predict protein disorder, each presenting its own strengths and weaknesses. To determine which algorithm combination presented the best performance, we built gold standard positive and negative datasets with sequences from the Disprot database (http://www.disprot.org). The performance evaluation was estimated using the ROC graphs approach to select the most efficient algorithm combination. Our results (Figure 2) indicated that the best algorithm combination for structural disorder prediction included REM465, GlobPipe, IUPred and VSL2B (TPR=0.670 and FPR=0.402). This particular combination presented the lowest number of false positives and a high number of false negatives among the five best predictor combinations. Therefore, this combination does not make misclassifications but tends to classify regions with a high certainty of being disorderly. In this respect, it is especially important to combine predictors because the combination can override the specific weaknesses of each predictor alone. As a direct consequence, the presence of FP classifications might result in erroneous profiling of proteins in the association analysis between disordered regions and other features. We observed that among the five best-performing combinations, those with the highest count of FP predictions included the Hotloops predictor. The individual performance of this predictor showed that Hotloops predictions had a higher level of FPs compared with the other predictors. Therefore, we chose an algorithm combination that did not include Hotloops.

Thus, despite not having the largest TPR among the five best performing combinations, we believe that this combination is ideal for our analysis.

### IDPs in trypanosomatids

Disordered proteins commonly exist without a stable three-dimensional structure and are characterized by highly flexible conformations. Analysis of protein disorder in several genomic sequences has shown that the occurrence of disordered regions of substantial size (more than 40 residues) is surprisingly common in functional proteins [23, 62]. The existence of disordered functional proteins such as hormones [63] and the experimental observation of such proteins in normal cells [64] demonstrates the crucial role of this protein class.

Disordered proteins play an important role in many biological processes, such as the regulation of transcription, translation and signal transduction [3, 25, 65, 66], and their existence is a strong argument for a reassessment of the structure-function paradigm [7].

Our results indicated that approximately 70% and 55% of *Leishmania* and *Trypanosoma* protein content, respectively, could be classified as disordered (protein disordered regions greater than or equal to 40 consecutive residues) after the pre-processing filtering step (see Additional files 4 and 5). Such high protein disorder content is not unexpected because similar results were found in other protozoan parasites such as *Toxoplasma gondii* (almost 70%) and *Plasmodium falciparum* (almost 50%) [17]. It is important to highlight that similar to trypanosomatids, three of the four apicomplexan species in which IDPs were found to be most abundant (*P. falciparum*, *P. vivax*, and *T. gondii*) represent major and widely distributed human pathogens [17]. Taken together, these results suggest a potential correlation between protein disorder and the genomic plasticity required to interact with different hosts, as previously described by Feng et al. [17].

Another interesting finding is the correlation between functional annotation and protein disorder. In this context, it is important to note that approximately 60% of trypanosomatid genomes consist of hypothetical proteins. Our results indicate that 60-70% of proteins predicted as IDPs have no predicted function (see Additional file 6) inferred by functional sequence similarity annotation methodologies. Considering that the predominantly used methodologies for automatic genome annotation (sequence similarity) have the low-complexity filter enabled as a default setting and matrices that are used as training dataset proteins with different composition profiles (most are globular proteins), our results suggest that this approach is inefficient for the functional annotation of disordered proteins.

A recent publication by Oates and colleagues (D^2^P^2^: database of disordered protein predictions) [67] included predictions for several trypanosomatid genomes and implemented a distinctive approach to estimating prediction agreement among the evaluated algorithms.

In contrast to the D^2^P^2^ database that evaluated consensus predictions using agreement among the different predictions, our methodology employed gold standard positive and negative datasets obtained from broad mining in public domain databases, including the PDB and Disprot, for the organisms studied. This approach added an essential confidence layer pertaining to the accuracy of predictions for trypanosomatids that is impossible to achieve when considering the 1765 complete proteomes included in D^2^P^2^.

### IDPs attributes in trypanosomatids

Considering that only slight differences were observed in sequence attributes among the analyzed predicted proteomes, we summarize the most essential ones here.

**Figure 3:**
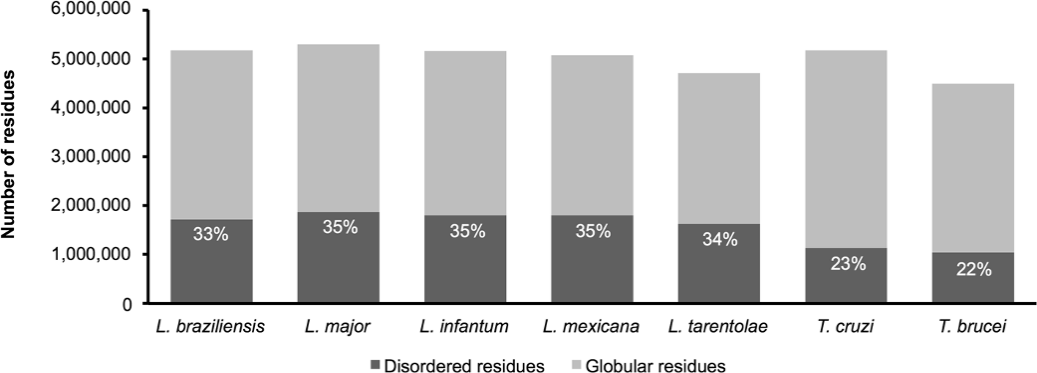
**Number of disordered residues.** The considered number of disordered residues was predicted by the chosen combination of disorder prediction programs (REM465, GlobPipe, IUPred and VSL2B).

In *Leishmania* species, almost 34% of residues were disordered, whereas in *Trypanosoma* species, 22% of residues were disordered (Figure 3). In comparison, Feng and collaborators [17] found that 10.3% of residues were disordered in *P. falciparum*. The observed differences may be related to differences in GC content (approximately 60% for trypanosomatids and approximately 20% for *P. falciparum*) and/or to the use of a prediction approach that involved just one predictor (Hot-loops of DisEMBL) in the work with the Apicomplexa genome and four predictors (REM465, GlobPipe, IUPred and VSL2B) for trypanosomatid genomes. For *Leishmania* and *Trypanosoma* species, the great majority (70%) of IDPs contained less than 50% disordered residues (see Additional file 7). Almost 30% of disordered regions ranged from 40 to 60 amino acids in length, with almost 50% of all disordered regions reaching 80 amino acids in length (see Additional file 8).

**Figure 4:**
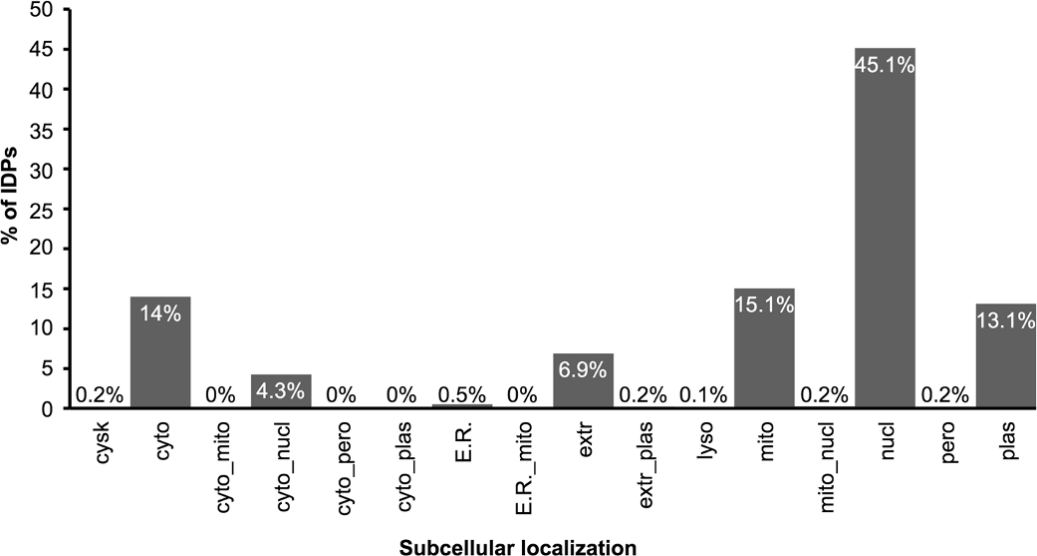
**Subcellular localization of IDPs in** ***L. braziliensis.*** The IDP subcellular localization prediction was performed using the algorithm WolfPSort with the terms cysk (cytoskeleton), cyto (cytosol), cyto_mito (cytosol or mitochondria), cyto_nucl (cytosolic or nuclear), cyto_pero (cytosol or peroxisome) cyto_plas (cytosol or plasma membrane), ER (endoplasmic reticulum), ER_mito (endoplasmic reticulum or mitochondria), extr (extracellular), extr_plas (extracellular or plasma membrane), lyso (lysosome), myth (mitochondria), mito_nucl (mitochondria or nuclear), nuclear (nuclear), pero (peroxisome) and plasma (plasma membrane).

Signal peptide analysis (Figure 4) showed that 45% of the IDPs presented signal peptides for the nucleus, followed by 15% for the mitochondria, 14% for the cytoplasm, 13% for the plasma membrane and 6% for the extracellular space. A previous study with the yeast proteome reported that proteins containing disorder are often located in the cell nucleus [68], suggesting that this occurs because the nucleus represents physical protection for unfolded structures against the cytoplasmic degradation apparatus.

Transmembrane domain predictions for *Leishmania* and *Trypanosoma* IDPs showed that approximately 50% and 65% have up to three transmembrane domains, respectively.

**Figure 5:**
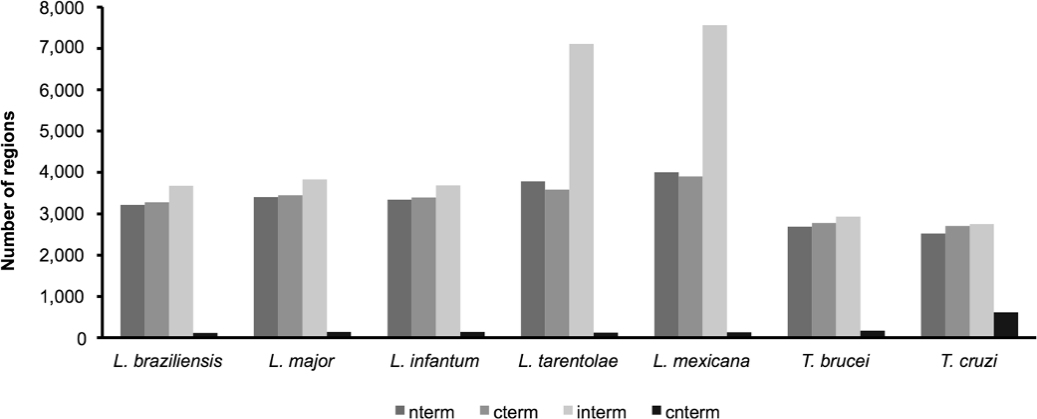
**Location of disordered regions in proteins.** The disordered regions were classified by the following terms: nterm (N terminal), cterm (C terminal), interm (intermediate) and cnterm (spanning from the C to N end). The N and C terminal regions were defined as 15% of sequence ends. The X-axis represents the name of the organism. The Y-axis indicates the number of disordered regions.

As can be observed in Figure 5, there is a tendency for the disordered regions to be located in the intermediate portion of the sequences (35%), followed by the C-terminal end (31%). *L. mexicana* and *L. tarentolae* represent outliers of this general profile, and we speculate that the obtained results are associated with the preliminary annotation status of the version of the genome used in this analysis. Additionally, *T. cruzi* had a much higher number of disordered regions (612 regions) spanning from the C to N-terminal region compared with the other six organisms. We speculate that this bias is associated with the fact that over 50% of the *T. cruzi* genome consists of repeated sequences [69].

### Amino acid frequencies

A comparative analysis of amino acid frequencies in globular protein regions was performed based on PDB_S25 data (http://bioinfo.tg.fh-giessen.de/pdbselect/). Our results suggest a general rule for trypanosomatids IDPs: a) P, Q, E, R and S amino acids are enriched, and b) W, Y, F, V, I, L and C amino acids are depleted (see Additional file 9).

These disordered regions are depleted in hydrophobic amino acids (I, L and V) and aromatic amino acids (W, Y and F), which typically constitute the hydrophobic core of globular proteins [10]. The decreased content of cysteine (C) in disordered regions supports the lack of formation of hydrophobic cores of globular proteins because this amino acid frequently occurs in sites of activating or stabilizing disulfide bridges important for maintaining protein structure in proteins and are thus not necessary in IDPs [25]. Consequently, L, I, F, V, W, Y and C can be considered order promoters.

Amino acids that function as disorder promoters in trypanosomatids are P, Q, E, R and S. For example, proline (P) enrichment is related to the lack of structure because this amino acid is known to oppose the formation of rigid secondary structures. P is actively involved in protein-protein interaction regions [70] and shows a strong preference for open conformation regions, which suggests a functional dimension for the prevalence of this amino acid in IDPs that strongly depends on the target recognition [25].

Disordered proteins in trypanosomatid genomes have low concentrations of aromatic (F, Y and W) and hydrophobic (I, L, F, W and V) amino acids and high concentrations of polar (S, T, Q, R, D and E) and charged (R, D and E) amino acids. Minor variations in the overall behavior may also depend on the experimental method used to identify the region (NMR, X-ray crystallography and circular dichroism) [71], the disordered region size [72], the structural disorder predictor [10] and the location of the disordered region along the sequences (N-terminal, C-terminal and intermediate) [73].

### IDP functions in trypanosomatids

**Figure 6:**
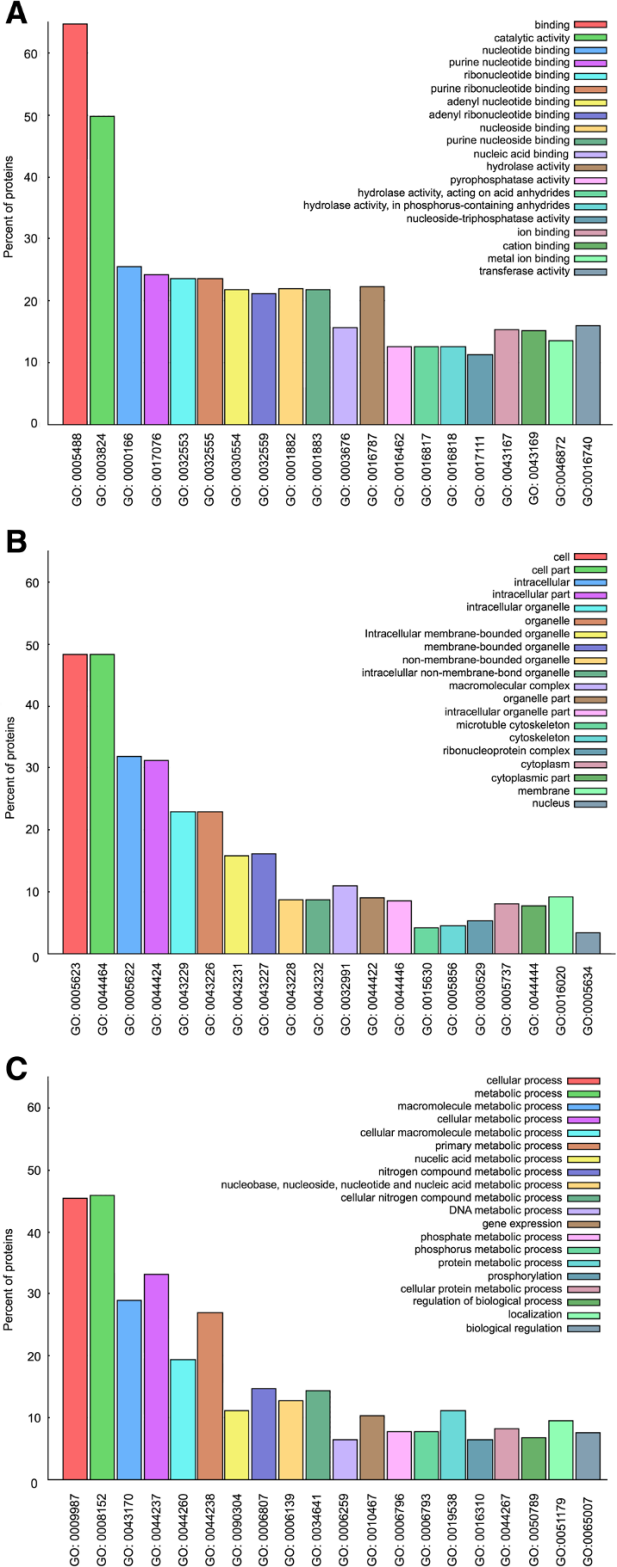
**GO enrichment analysis.** The Perl library GO::TermFinder was used for the enrichment analysis. A *p*-*value* ≤ 0.05 was considered significant. The X-axis indicates the GO number; a description of each is included in the legend. The Y-axis indicates the percentage of proteins. The first 20 enriched GO terms of *L. braziliensis* IDPs are included for **A)** molecular function, **B)** cellular component and **C)** biological process.

Figure 6A shows that the terms ‘*binding*’ (corrected *p*-*value*: 0) and ‘*catalytic activity*’ (corrected *p*-*value*: 2.14e^-221^) are the most common among the 20 most highly enriched terms in the GO molecular function category. GO defines the term ‘*binding*’ as the selective, non-covalent, often stoichiometric interaction of a molecule with one or more specific sites on another molecule. GO defines ‘*catalytic activity*’ as the catalysis of a biochemical reaction at physiological temperatures in a biologically catalyzed reaction. The reactants are known as substrates and the catalysts as enzymes.

For cellular component category analysis (Figure 6B), we observed that the terms ‘*part of the cell*’ (corrected *p*-*value*: 3.72e^-240^) and ‘*intracellular*’ (corrected *p*-*value*: 1.38e^-132^) were the most common. According to the GO definition, ‘*part of the cell*’ is any constituent part of a cell, defined as the basic structure and functional unit of all organisms. ‘*Intracellular*’ is the live content of a cell, which is contained by (but not including) the plasma membrane, usually excluding large vacuoles and masses of material ingested or secreted.

For the biological process category (Figure 6C), the terms ‘*metabolic process*’ (corrected *p*-*value*: 6.33e^-202^) and ‘*cellular process*’ (corrected *p*-*value*: 7.91e^-229^) were highlighted. According to the GO definition, ‘*metabolic processes*’ are chemical reactions and pathways including anabolism and catabolism by which living organisms transform chemicals. The definition of ‘*cellular process*’ is any process performed at the cellular level but not necessarily restricted to a single cell; for example, cellular communication is a cellular process.

Experimental data have revealed that for many proteins, the performed function depends on the degree of unstructuration instead of the degree of protein structuration [74, 75]. Taking this into account, among the many GO terms that we have shown to be significantly enriched (*p*-*value* less than 0.05) in IDPs and given their relevance for trypanosomatids, we chose to highlight the following:

- Transcription and transcriptional regulation: DNA-protein and protein-protein interactions are the core processes during the transcription process. Several examples of IDPs involved in transcriptional regulation have been reported [15, 22]. For example, the activation domain C-terminus of the proto-oncoprotein (bZIP) is unstructured and flexible and effectively suppresses transcription *in vitro* [76]. In trypanosomatids, post-transcriptional control is the predominant means by which gene expression is regulated. In fact, it is thought that genes are transcribed and processed continuously and then regulated, either by selective transport to the cytoplasm, mRNA stability or the selection of mRNA sequences for translation. mRNA stability has been widely studied in trypanosomatids, with most data focusing on demonstrating the role of the 3’UTR (3’ untranslated region) and interacting proteins. Much of the field of regulation of gene expression in trypanosomatids needs to be elucidated and investigated. In this context, we suggest that IDPs may play an important role.
- RNA processing and splicing: In trypanosomatids, trans-splicing is responsible for processing the polycistronic pre-mRNA, resulting in individual messages. Thus, each mRNA contains the spliced leader sequence (SL) at the 5’ end and the poly-A tail at the 3’ end. Pre-mRNA processing in trypanosomatids is catalyzed by the spliceosome, a high-molecular-weight machinery comprising ribonucleoproteins (RNPs) and other proteins. Proteins involved in spliceosome “assembly” (by facilitating the recruitment of its components) are rich in serine and arginine residues, two amino acids enriched in trypanosomatids IDPs.
- Cytoskeleton: A set of protein structures, filaments and microtubules determine the structure and shape of the cell and contribute to the cytoskeleton. IDPs play crucial roles in the assembly and function of the cytoskeleton. The degree of disorder of cytoskeleton proteins is comparable to those observed in proteins involved in cell signaling and regulation [65].
- Flagellum: *Leishmania* and *Trypanosoma* are flagellate protozoa whose life cycle involves the sequential infection of vector and mammalian hosts. During these processes, complex morphological and biochemical changes occur successively. These processes are particularly important for *Leishmania*, which possesses both an extracellular flagellated form and an intracellular form without flagella in the mammalian host.

In bacteria, the main flagellum component is a filament called flagellin, ranging from 12 to 25 nm in diameter. Structural disorder plays a crucial role in the assembly of the bacterial flagella [24]. Based on these observations, we suggest that protein terminal disorder is associated with the flagellum in trypanosomatids, and this disorder may play important roles in a manner similar to that occurring in flagellin.

### Contingency analysis

With the predictions made from different disorder algorithms and several other analyses, each predicted IDP loaded in the database showed 56 associated variables. To verify the existence of significant associations between protein disorder and other identified biological features, we performed a multivariate investigation using the contingency analysis strategy approach. This approach was applied to thirteen chosen features (protein length, protein annotation, protein localization, protein molecular weight, protein charge, isoelectric point, number of C-terminal disordered regions, number of N-terminal disordered regions, number of intermediate disordered regions, number of CN-terminal disordered regions, percent of disordered residues, number of transmembrane domains and number of disordered regions) to generate a contingency table. The characteristics that were significantly associated with the percentage of disordered residues were a) predicted/hypothetical, b) isoelectric point, c) location and d) transmembrane domains.

A very interesting inverse behavior was observed in all significant associations (*p*-*value* less than 0.05) when the protein disorder content reached approximately 40%.

**Figure 7:**
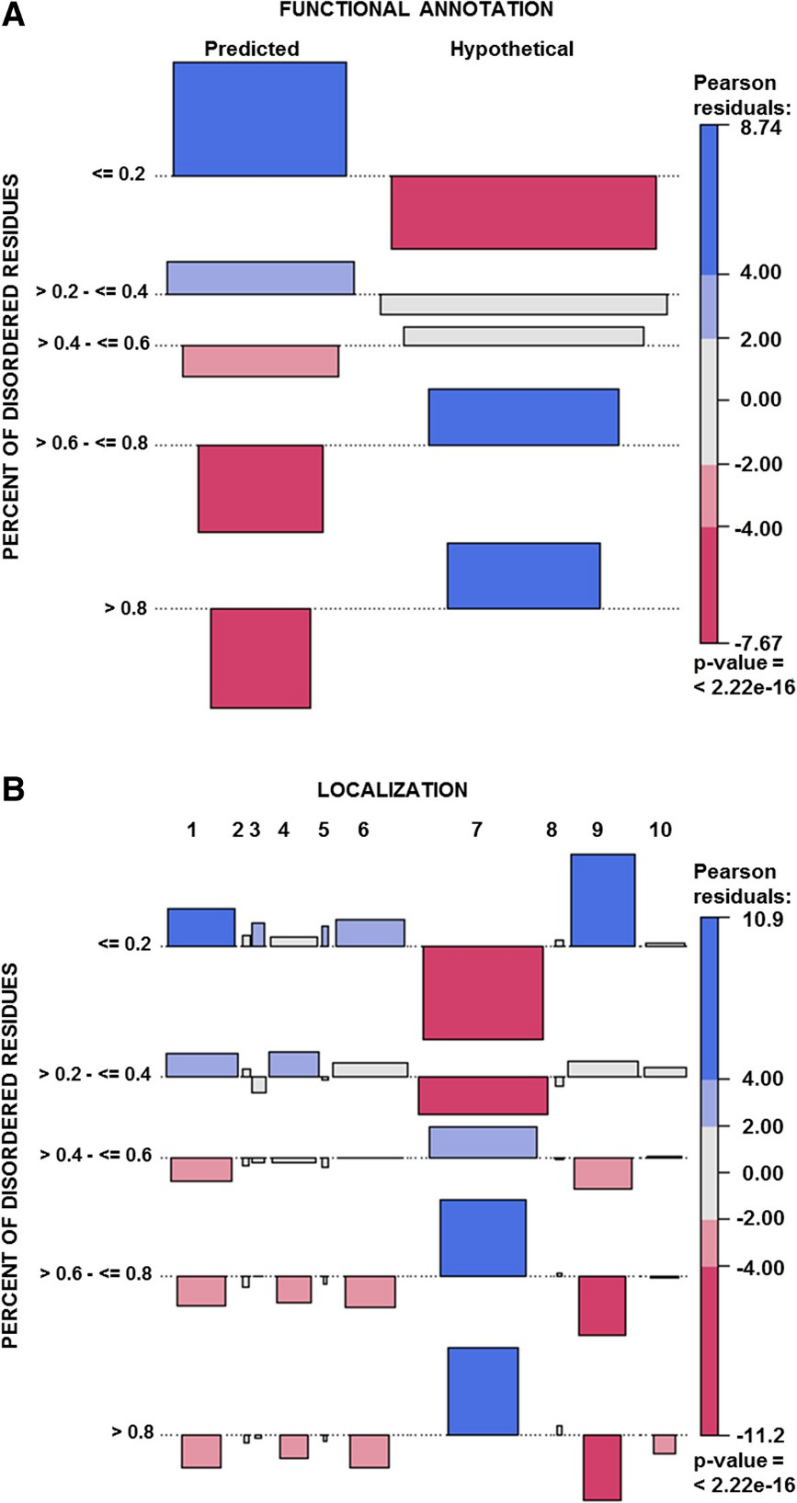
**Associations with** ***L. braziliensis*** **IDPs.** The VCD package was used, considering a *p*-*value* ≤0.05 as a significant association. The colors represent whether the frequency is higher (blue) or lower (pink) than expected. The numbers represent the categories of attributes. The association between the percentage of disordered residues and **A)** the functional annotation of the protein (predicted or hypothetical) and **B)** the predicted location as 1- cytosol; 2- cytoskeleton; 3- endoplasmic reticulum; 4- extracellular; 5- lysosome; 6- mitochondria; 7- nuclear; 8- peroxisome; 9- plasma membrane and 10- without prediction.

One of these significant associations correlates with two classification terms: a) the percentage of disordered residues and b) functional protein annotation (predicted or hypothetical) (Figure 7A).

As the disorder content increased up to 40%, the predicted function annotation became more frequent than expected. For values greater than 40%, we observed a higher hypothetical protein annotation frequency than expected. Approximately 60% of trypanosomatid genomes consisted of hypothetical proteins with no significant similarity with public domain databases. Considering the current scenario of genome automatic annotation, we suggest a direct association of our findings, specifically the increasing of structural disorder percentage, with the high content of unpredicted protein functions in trypanosomatid genomes.

Another significant association correlated the classification term isoelectric point and the percentage of disordered residues. In more than 40% of disordered content, a higher than expected frequency of very basic proteins (pH>9) was observed (see Additional file 10).

The classification term subcellular location also revealed a significant association with the percentage of disordered residues (Figure 7B). When the disordered residue content reached 40%, a higher frequency than expected occurred in the classes plasma membrane, cytosol and mitochondria. In contrast, when the percent of disordered residues was above 40%, a higher increase in the nuclear location class was observed.

The last significant association correlated the classification term transmembrane domain and the percentage of disordered residues. IDPs with more than 40% disordered content tended to contain transmembrane domains at a lower frequency than expected (see Additional file 11).

### Experimental analysis

To confirm the computational predictions, two special gel electrophoresis methods were employed to separate and/or enrich protein preparations with IDPs (one was developed by Csizmók et al. and the other by Galea et al.). The first methodology involved a special two-dimensional electrophoresis and was performed only for the *L. major* proteome (see Additional file 12A) because it required a large amount of parasite culture to obtain a sufficient protein extract. The second approach, based on previous IDPs enrichment, was applied to the *L. major* and *L. braziliensis* proteomes (see Additional file 12B and C).

After IDP fractionation, 17 protein spots were selected from the gels and identified by mass spectrometry (MALDI-TOF/TOF analyzer). The results (see Additional file 13) indicated that 100% (15/15) of the identified proteins were recognized as disordered proteins *in silico* by the best combination of structural disorder predictors (REM465, GlobPipe, IUPRED and VSL2B).

This finding indicated that the pipeline met its goal of integrative IDP prediction in addition to data integration with a safe, automatic and organized computational characterization.

## Conclusions

In this work, a central pipeline for the identification, characterization and analysis of IDPs in trypanosomatids was developed. Predictions were experimentally validated, and the pipeline met its goal of data integration by performing a safe, automatic, organized and integrative IDP prediction and computational characterization. The results presented highlight the following: a) the high content of IDPs, approximately 70% for *Leishmania* and 50% for *Trypanosoma* species; b) that different features such as functional annotation (predicted/hypothetical), isoelectric point, location (nucleus) and transmembrane domains are significantly associated with the percentage of disordered residues, as confirmed by the contingency analysis; and c) that the terms ‘*binding*’ and ‘*catalytic activity*’ >are the most common ones for molecular function category analysis. Thus, the IDP scenario in trypanosomatids may prove to be relevant to the understanding of host-parasite interaction and the peculiarities of pathogens’ biology. In addition, the information regarding the IDP content in trypanosomatids may drive new studies of gene function and the evolution of these ancestral and pathogenic eukaryotes. The aim of this work is not to develop a web server but to perform a deep analysis of IDP content in trypanosomatid genomes. The database and developed pipeline are available to the research community upon request.

### Availability of supporting data

The dataset supporting the results of this article is included within the article (and its additional files).

## Electronic supplementary material

Additional file 1: **Number of publications related to IDPs.** The following terms were searched: intrinsically disordered proteins, intrinsically unfolded proteins, intrinsically unstructured proteins and natively unfolded proteins. (PDF 11 KB)

Additional file 2: **Consensus disorder prediction.** Consensus of disordered prediction. (PDF 28 KB)

Additional file 3: **Considered hits and errors.** Considered hits and errors. (PDF 31 KB)

Additional file 4: **Pre-processing results.** Number and percentage of sequences that went into each step of the IDP pipeline pre-processing. (PDF 10 KB)

Additional file 5: **IDPs information.** Identified IDPs for each organism. (PDF 3 MB)

Additional file 6: **IDPs functional annotation.** Classification of the identified IDPs (hypothetical or with predicted function). (PDF 10 KB)

Additional file 7: **Percent of disordered residues in** ***L. braziliensis.*** Percentage of disordered residues in *L. braziliensis*. (PDF 11 KB)

Additional file 8: **Percent of disordered regions in** ***L. braziliensis.*** Percentage of disordered regions in *L. braziliensis*. (PDF 11 KB)

Additional file 9: **Frequency of IDP amino acids relative to globular amino acids in** ***L. braziliensis.*** Frequency of IDP amino acids relative to globular amino acids in *L. braziliensis.* (PDF 12 KB)

Additional file 10: **Association between the percentage of disordered residues and isoelectric points in** ***L. braziliensis.*** The colors represent whether the frequency is higher (blue) or lower (pink) than expected. The numbers represent the categories of attributes. (PDF 30 KB)

Additional file 11: **Association between the percentage of disordered residues and transmembrane domains in** ***L. braziliensis.*** The colors represent whether the frequency is higher (blue) or lower (pink) than expected. The numbers represent the categories of attributes. (PDF 31 KB)

Additional file 12: **IDPs identification gels.** A) *L. major* 2D electrophoresis gel; the IDPs are located near the diagonal line; B) *L. major* 2D electrophoresis with IDP enrichment; and C) *L. braziliensis* 2D electrophoresis with IDP enrichment. (PDF 268 KB)

Additional file 13: **Experimental validation.** The IDP spot number is provided in **Figure S8A**, **S8B** and **S8C**. (PDF 14 KB)
