# Supplementary figures and images for: Intrinsically disordered proteins (IDPs) in trypanosomatids
Source: BMC Genomics. 2014 Dec 13;15(1):1100. doi: 10.1186/1471-2164-15-1100 (PMC4378006; doi:10.1186/1471-2164-15-1100)

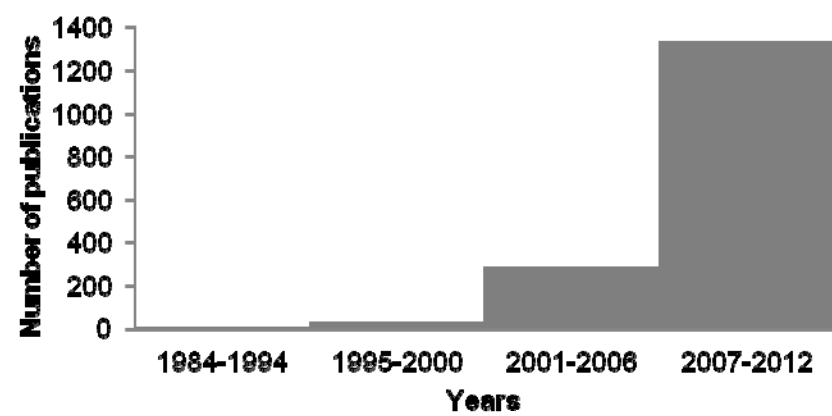

Supplement: Supplementary file 1 — Additional file 1: Number of publications related to IDPs. The following terms were searched: intrinsically disordered proteins, intrinsically unfolded proteins, intrinsically unstructured proteins and natively unfolded proteins. (PDF 11 KB) [file 12864_2014_6918_MOESM1_ESM.pdf]

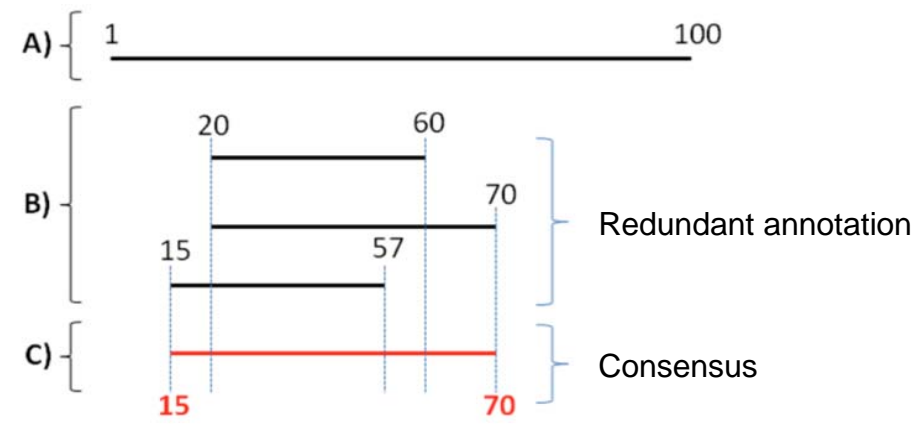

Supplement: Supplementary file 2 — Additional file 2: Consensus disorder prediction. Consensus of disordered prediction. (PDF 28 KB) [file 12864_2014_6918_MOESM2_ESM.pdf]

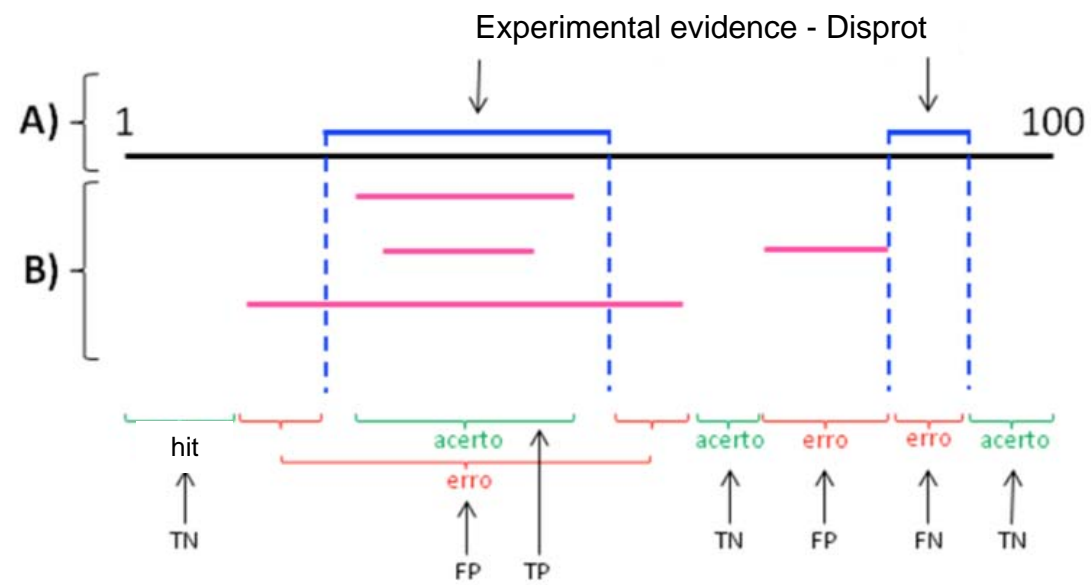

Supplement: Supplementary file 3 — Additional file 3: Considered hits and errors. Considered hits and errors. (PDF 31 KB) [file 12864_2014_6918_MOESM3_ESM.pdf]

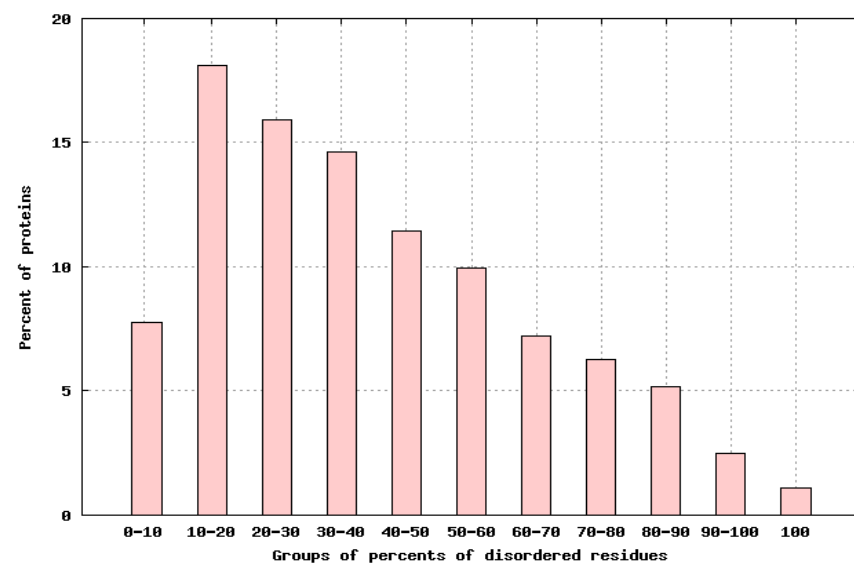

Supplement: Supplementary file 7 — Additional file 7: Percent of disordered residues in L. braziliensis. Percentage of disordered residues in L. braziliensis. (PDF 11 KB) [file 12864_2014_6918_MOESM7_ESM.pdf]

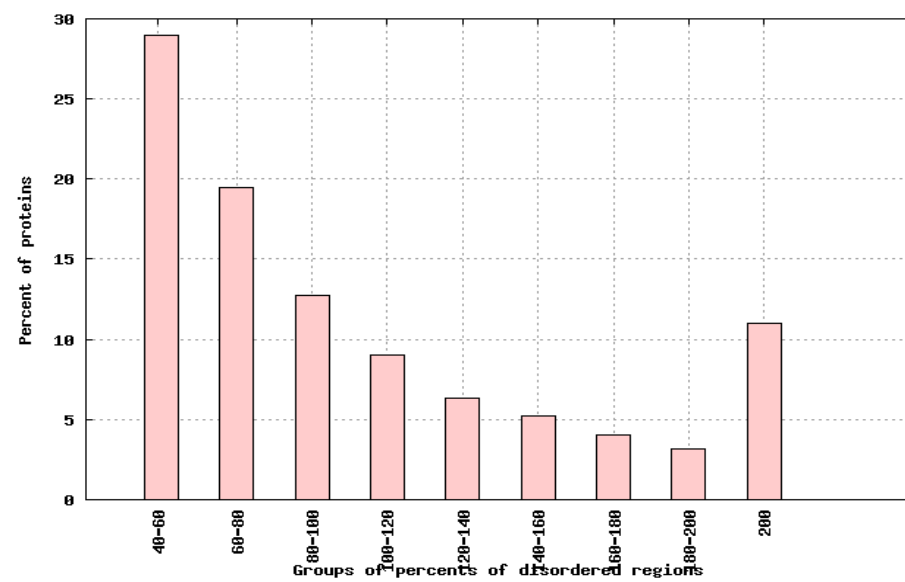

Supplement: Supplementary file 8 — Additional file 8: Percent of disordered regions in L. braziliensis. Percentage of disordered regions in L. braziliensis. (PDF 11 KB) [file 12864_2014_6918_MOESM8_ESM.pdf]

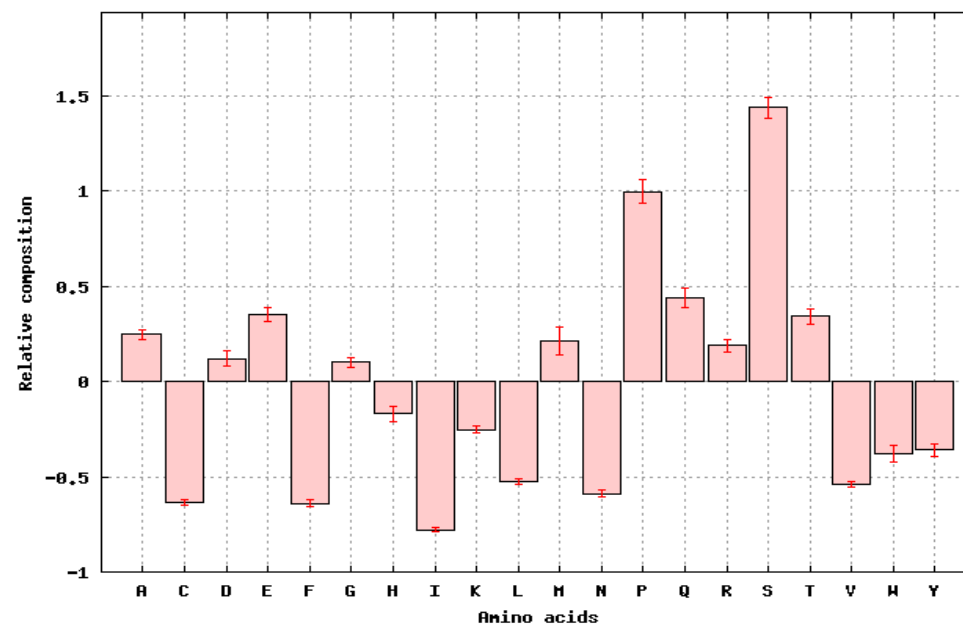

Supplement: Supplementary file 9 — Additional file 9: Frequency of IDP amino acids relative to globular amino acids in L. braziliensis. Frequency of IDP amino acids relative to globular amino acids in L. braziliensis. (PDF 12 KB) [file 12864_2014_6918_MOESM9_ESM.pdf]

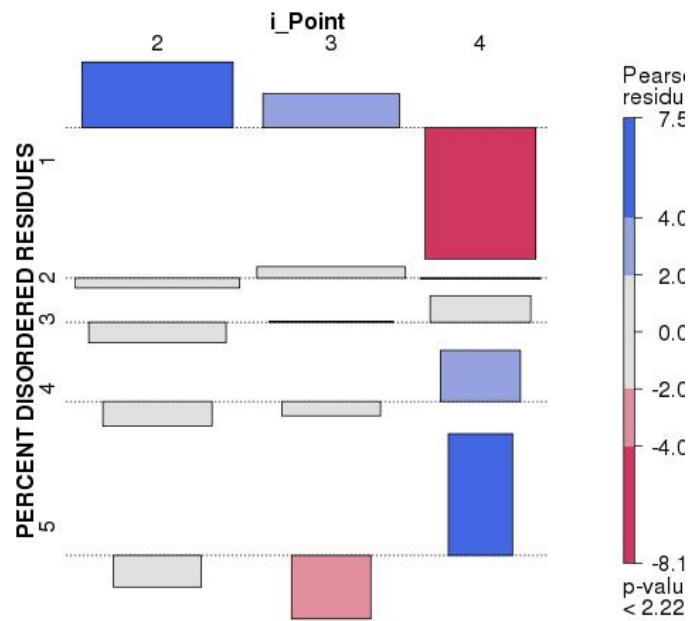

| I_POINT                        |   |
|--------------------------------|---|
| <= 3                           | 1 |
| > 3 AND <= 7                   | 2 |
| > 7 AND <= 9                   | 3 |
| > 9                            | 4 |
|                                |   |
| PERCENT OF DISORDERED RESIDUES |   |
| > 0 AND <= 0.2                 | 1 |
| > 0.2 AND <= 0.4               | 2 |
| > 0.4 AND <= 0.6               | 3 |
| > 0.6 AND <= 0.8               | 4 |
| > 0.8                          | 5 |

Supplement: Supplementary file 10 — Additional file 10: Association between the percentage of disordered residues and isoelectric points in L. braziliensis. The colors represent whether the frequency is higher (blue) or lower (pink) than expected. The numbers represent the categories of attributes. (PDF 30 KB) [file 12864_2014_6918_MOESM10_ESM.pdf]

**A**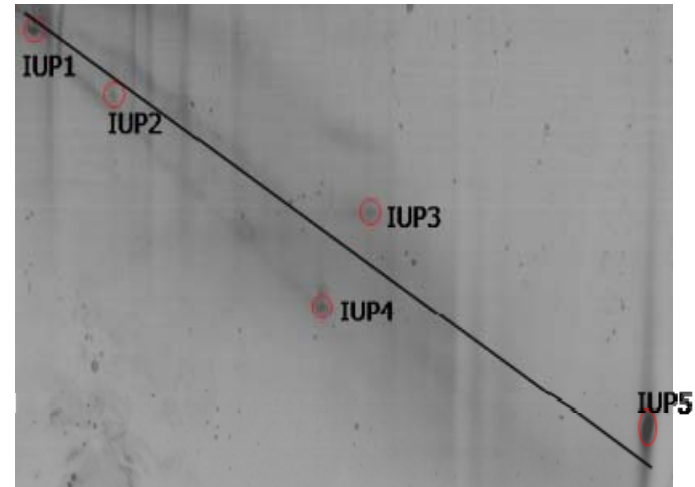**B**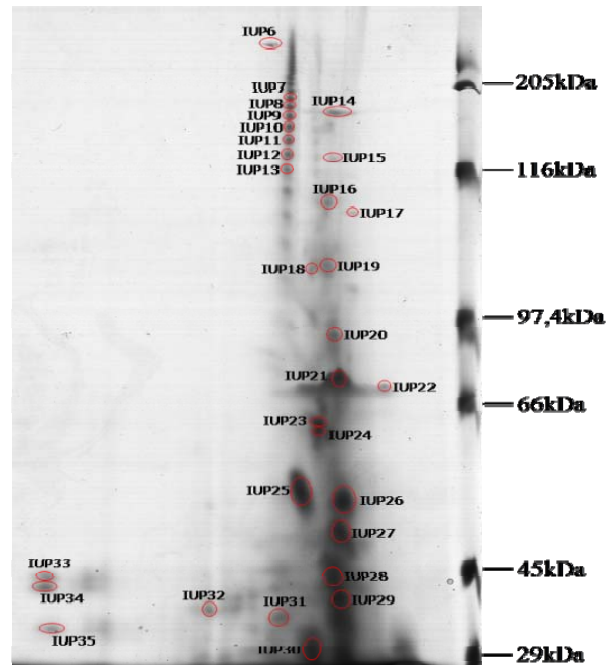**C**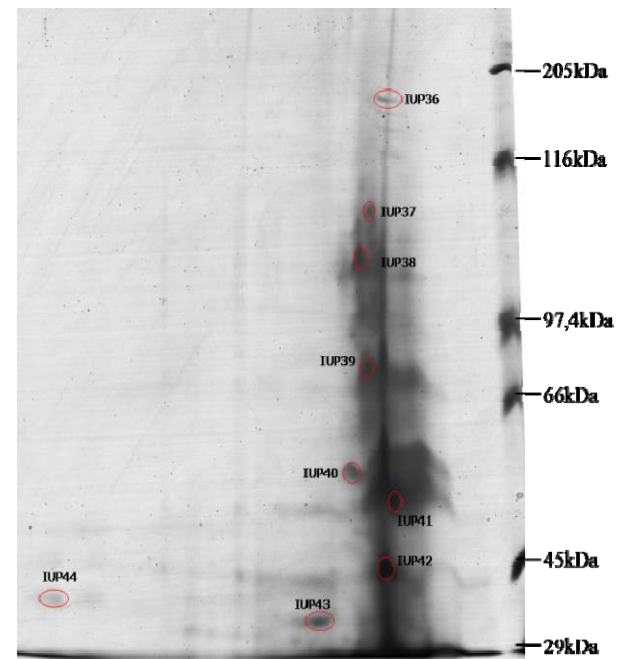

Supplement: Supplementary file 12 — Additional file 12: IDPs identification gels. A) L. major 2D electrophoresis gel; the IDPs are located near the diagonal line; B) L. major 2D electrophoresis with IDP enrichment; and C) L. braziliensis 2D electrophoresis with IDP enrichment. (PDF 268 KB) [file 12864_2014_6918_MOESM12_ESM.pdf]
