# Supplementary material for: Intrinsically disordered proteins (IDPs) in trypanosomatids
Source: BMC Genomics. 2014 Dec 13;15(1):1100. doi: 10.1186/1471-2164-15-1100 (PMC4378006; doi:10.1186/1471-2164-15-1100)
Supplement: Supplementary file 4 — Additional file 4: Pre-processing results. Number and percentage of sequences that went into each step of the IDP pipeline pre-processing. (PDF 10 KB) [file 12864_2014_6918_MOESM4_ESM.pdf]

Organism	Sequences larger than 100 amino acids	Sequences with initial Methionine	Sequences with initial Methionine and don’t have annotation errors
L. braziliensis	98.2% (8165 proteins)	97.2% (8084 proteins)	95.8% (7965 proteins)
L. major	98.1% (8253 proteins)	97.7% (8222 proteins)	97.1% (8171 proteins)
L. infantum	98.2% (8072 proteins)	97.7% (8034 proteins)	95.3% (7835 proteins)
L. mexicana	100% (7952 proteins)	100% (7952 proteins)	100% (7952 proteins)
L. tarentolae	100% (7465 proteins)	100% (7465 proteins)	100% (7465 proteins)
T. cruzi	97.8% (10096 proteins)	86.4% (8921 proteins)	79.2% (8178 proteins)
T. brucei	97.0% (9599 proteins)	96.3% (9537 proteins)	95.7% (9472 proteins)
