# Supplementary material for: Intrinsically disordered proteins (IDPs) in trypanosomatids
Source: BMC Genomics. 2014 Dec 13;15(1):1100. doi: 10.1186/1471-2164-15-1100 (PMC4378006; doi:10.1186/1471-2164-15-1100)
Supplement: Supplementary file 6 — Additional file 6: IDPs functional annotation. Classification of the identified IDPs (hypothetical or with predicted function). (PDF 10 KB) [file 12864_2014_6918_MOESM6_ESM.pdf]

Organism	Number of analyzed sequences	Number of predicted IDPs	Percent of IDPs with predicted function	Percent of hypothetical IDPs
L. braziliensis	7965 (95.8%)	5725 (68.9%)	28.9%	70.9%
L. major	8171 (97.2%)	5959 (70.8%)	29.1%	70.6%
L. infantum	7835 (95.3%)	5797 (70.5%)	28.7%	71%
L. mexicana	7952 (100%)	5774 (72.6%)	29.5%	70.4%
L. tarentolae	7465 (100%)	5483 (73.4%)	26.9%	72.5%
T. cruzi	8178 (79.2%)	5565 (53.9%).	38.5%	61.5%
T. brucei	9472 (95.7%)	5510 (55.6%)	32%	68%
