# Supplementary material for: Intrinsically disordered proteins (IDPs) in trypanosomatids
Source: BMC Genomics. 2014 Dec 13;15(1):1100. doi: 10.1186/1471-2164-15-1100 (PMC4378006; doi:10.1186/1471-2164-15-1100)
Supplement: Supplementary file 11 — Additional file 11: Association between the percentage of disordered residues and transmembrane domains in L. braziliensis. The colors represent whether the frequency is higher (blue) or lower (pink) than expected. The numbers represent the categories of attributes. (PDF 31 KB) [file 12864_2014_6918_MOESM11_ESM.pdf]

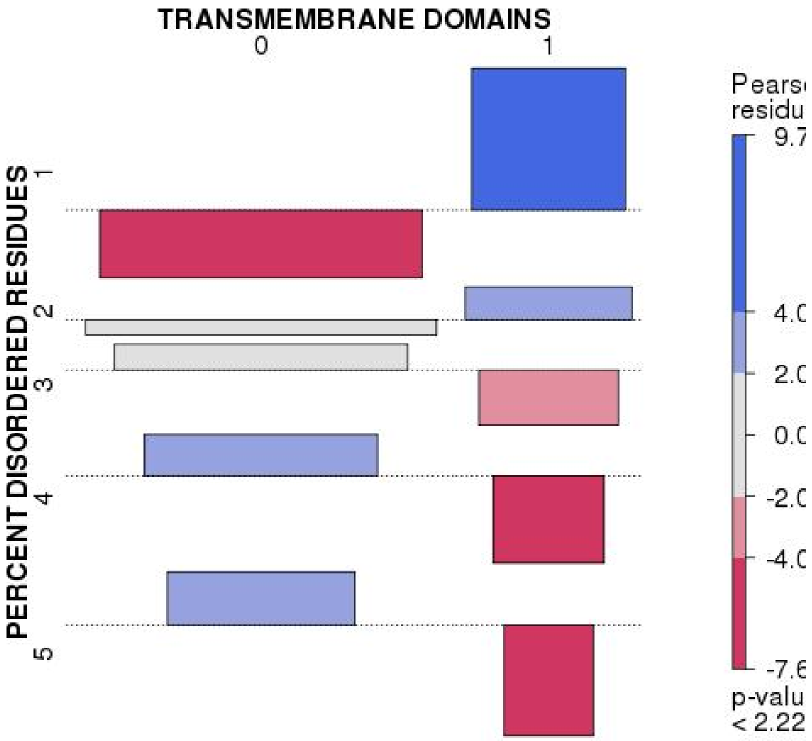

TRANSMEMBRANE DOMAINS	
without transmembrane regions	0
with transmembrane regions	1
**PERCENT OF DISORDERED RESIDUES**	
> 0 AND <= 0.2	1
> 0.2 AND <= 0.4	2
> 0.4 AND <= 0.6	3
> 0.6 AND <= 0.8	4
> 0.8	5
