# Supplementary material for: Intrinsically disordered proteins (IDPs) in trypanosomatids
Source: BMC Genomics. 2014 Dec 13;15(1):1100. doi: 10.1186/1471-2164-15-1100 (PMC4378006; doi:10.1186/1471-2164-15-1100)
Supplement: Supplementary file 13 — Additional file 13: Experimental validation. The IDP spot number is provided in Figure S8A, S8B and S8C. (PDF 14 KB) [file 12864_2014_6918_MOESM13_ESM.pdf]

IDP spot	Organism	Protein ID	Function	Score	Best Combination
					REM465_GLOBPIPE_IUPRED_VSL2B
IUP1	*L. major*	LmjF27.0240	kinetoplast-associated protein-like protein	285	X
IUP4	*L. major*	LmjF32.2520	hypothetical protein, unknown function	31	X
IUP5	*L. major*	LmjF09.0910	calmodulin, putative	505	X
IUP6	*L. major*	LmjF27.0240	kinetoplast-associated protein-like protein	435	X
IUP8	*L. major*	LmjF05.0380	microtubule-associated protein, putative	188	X
IUP10	*L. major*	LmjF05.0380	microtubule-associated protein, putative	449	X
IUP11	*L. major*	LmjF05.0380	microtubule-associated protein, putative	228	X
IUP25	*L. major*	LmjF23.1020	hypothetical protein, unknown function	1973	X
IUP26a	*L. major*	LmjF04.0770	nascent polypeptide associated complex subunit-like protein	1093	X
IUP26b	*L. major*	LmjF19.1160	hypothetical protein, conserved	1054	X
IUP27	*L. major*	LmjF04.0770	nascent polypeptide associated complex subunit-like protein	1854	X
IUP32	*L. major*	LmjF28.2770	heat-shock protein hsp70, putative	608	X
IUP34	*L. major*	LmjF30.2460	heat shock 70-related protein 1, mitochondrial precursor, putative	1591	X
IUP37	*L. braziliensis*	no hits	-	-	
IUP40	*L. braziliensis*	no hits	-	-	
IUP42	*L. braziliensis*	LbrM25_V2.0580	eukaryotic initiation factor 5a, putative	2160	X
IUP43	*L. braziliensis*	LbrM28_V2.1300	glucose-regulated protein 78, putative	363	X
