# Supplementary material for: Intrinsically disordered proteins (IDPs) in trypanosomatids
Source: BMC Genomics. 2014 Dec 13;15(1):1100. doi: 10.1186/1471-2164-15-1100 (PMC4378006; doi:10.1186/1471-2164-15-1100)
Supplement: Supplementary file 5 — Additional file 5: IDPs information. Identified IDPs for each organism. (PDF 3 MB) [file 12864_2014_6918_MOESM5_ESM.pdf]

Protein ID	Organism	Length	Start coord	End coord
LbrM01_V2.0010	*L. braziliensis*	197	1	197
LbrM01_V2.0010	*L. braziliensis*	64	315	378
LbrM01_V2.0010	*L. braziliensis*	408	412	819
LbrM01_V2.0010	*L. braziliensis*	65	849	913
LbrM01_V2.0020	*L. braziliensis*	146	288	433
LbrM01_V2.0020	*L. braziliensis*	156	462	617
LbrM01_V2.0030	*L. braziliensis*	61	97	157
LbrM01_V2.0030	*L. braziliensis*	68	312	379
LbrM01_V2.0030	*L. braziliensis*	68	312	379
LbrM01_V2.0040	*L. braziliensis*	57	276	332
LbrM01_V2.0050	*L. braziliensis*	44	45	88
LbrM01_V2.0060	*L. braziliensis*	58	70	127
LbrM01_V2.0060	*L. braziliensis*	124	446	569
LbrM01_V2.0070	*L. braziliensis*	54	1	54
LbrM01_V2.0090	*L. braziliensis*	114	125	238
LbrM01_V2.0100	*L. braziliensis*	75	70	144
LbrM01_V2.0100	*L. braziliensis*	62	240	301
LbrM01_V2.0100	*L. braziliensis*	94	521	614
LbrM01_V2.0140	*L. braziliensis*	58	23	80
LbrM01_V2.0150	*L. braziliensis*	42	1	42
LbrM01_V2.0150	*L. braziliensis*	106	79	184
LbrM01_V2.0150	*L. braziliensis*	45	204	248
LbrM01_V2.0150	*L. braziliensis*	291	283	573
LbrM01_V2.0160	*L. braziliensis*	186	179	364
LbrM01_V2.0180	*L. braziliensis*	44	63	106
LbrM01_V2.0200	*L. braziliensis*	73	1	73
LbrM01_V2.0200	*L. braziliensis*	78	130	207
LbrM01_V2.0200	*L. braziliensis*	158	296	453
LbrM01_V2.0210	*L. braziliensis*	51	718	768
LbrM01_V2.0220	*L. braziliensis*	66	23	88
LbrM01_V2.0220	*L. braziliensis*	81	112	192
LbrM01_V2.0230	*L. braziliensis*	42	87	128
LbrM01_V2.0240	*L. braziliensis*	63	42	104
LbrM01_V2.0240	*L. braziliensis*	142	190	331
LbrM01_V2.0240	*L. braziliensis*	85	388	472
LbrM01_V2.0240	*L. braziliensis*	54	653	706
LbrM01_V2.0250	*L. braziliensis*	54	50	103
LbrM01_V2.0260	*L. braziliensis*	71	58	128
LbrM01_V2.0260	*L. braziliensis*	84	163	246
LbrM01_V2.0260	*L. braziliensis*	128	329	456
LbrM01_V2.0280	*L. braziliensis*	46	273	318
LbrM01_V2.0280	*L. braziliensis*	70	512	581
LbrM01_V2.0280	*L. braziliensis*	136	596	731

LbrM01_V2.0290	*L. braziliensis*	186	42	227
LbrM01_V2.0290	*L. braziliensis*	287	263	549
LbrM01_V2.0310	*L. braziliensis*	54	1	54
LbrM01_V2.0390	*L. braziliensis*	110	1	110
LbrM01_V2.0400	*L. braziliensis*	220	369	588
LbrM01_V2.0400	*L. braziliensis*	134	606	739
LbrM01_V2.0420	*L. braziliensis*	42	147	188
LbrM01_V2.0430	*L. braziliensis*	45	556	600
LbrM01_V2.0430	*L. braziliensis*	92	683	774
LbrM01_V2.0440	*L. braziliensis*	109	1	109
LbrM01_V2.0440	*L. braziliensis*	87	220	306
LbrM01_V2.0440	*L. braziliensis*	119	485	603
LbrM01_V2.0440	*L. braziliensis*	54	761	814
LbrM01_V2.0450	*L. braziliensis*	53	100	152
LbrM01_V2.0450	*L. braziliensis*	147	233	379
LbrM01_V2.0450	*L. braziliensis*	43	500	542
LbrM01_V2.0460	*L. braziliensis*	85	1	85
LbrM01_V2.0470	*L. braziliensis*	43	1	43
LbrM01_V2.0480	*L. braziliensis*	137	130	266
LbrM01_V2.0480	*L. braziliensis*	56	390	445
LbrM01_V2.0480	*L. braziliensis*	67	485	551
LbrM01_V2.0480	*L. braziliensis*	56	580	635
LbrM01_V2.0490	*L. braziliensis*	49	279	327
LbrM01_V2.0510	*L. braziliensis*	299	1	299
LbrM01_V2.0510	*L. braziliensis*	46	734	779
LbrM01_V2.0510	*L. braziliensis*	210	877	1086
LbrM01_V2.0510	*L. braziliensis*	252	1111	1362
LbrM01_V2.0550	*L. braziliensis*	52	1	52
LbrM01_V2.0560	*L. braziliensis*	206	1	206
LbrM01_V2.0560	*L. braziliensis*	44	250	293
LbrM01_V2.0570	*L. braziliensis*	130	1	130
LbrM01_V2.0570	*L. braziliensis*	61	206	266
LbrM01_V2.0570	*L. braziliensis*	289	381	669
LbrM01_V2.0580	*L. braziliensis*	42	1	42
LbrM01_V2.0580	*L. braziliensis*	52	65	116
LbrM01_V2.0580	*L. braziliensis*	76	189	264
LbrM01_V2.0580	*L. braziliensis*	234	294	527
LbrM01_V2.0590	*L. braziliensis*	109	1	109
LbrM01_V2.0590	*L. braziliensis*	83	211	293
LbrM01_V2.0610	*L. braziliensis*	74	1	74
LbrM01_V2.0610	*L. braziliensis*	104	242	345
LbrM01_V2.0630	*L. braziliensis*	40	1	40
LbrM01_V2.0630	*L. braziliensis*	133	90	222
LbrM01_V2.0630	*L. braziliensis*	42	506	547
LbrM01_V2.0630	*L. braziliensis*	44	570	613
LbrM01_V2.0630	*L. braziliensis*	47	644	690
LbrM01_V2.0630	*L. braziliensis*	97	739	835

LbrM01_V2.0630	*L. braziliensis*	49	1464	1512
LbrM01_V2.0630	*L. braziliensis*	67	1631	1697
LbrM01_V2.0650	*L. braziliensis*	41	23	63
LbrM01_V2.0650	*L. braziliensis*	182	99	280
LbrM01_V2.0660	*L. braziliensis*	499	1	499
LbrM01_V2.0660	*L. braziliensis*	40	550	589
LbrM01_V2.0660	*L. braziliensis*	189	621	809
LbrM01_V2.0680	*L. braziliensis*	55	207	261
LbrM01_V2.0680	*L. braziliensis*	154	277	430
LbrM01_V2.0680	*L. braziliensis*	154	277	430
LbrM01_V2.0690	*L. braziliensis*	45	272	316
LbrM01_V2.0700	*L. braziliensis*	236	1	236
LbrM01_V2.0700	*L. braziliensis*	76	362	437
LbrM01_V2.0700	*L. braziliensis*	46	754	799
LbrM01_V2.0700	*L. braziliensis*	49	838	886
LbrM01_V2.0700	*L. braziliensis*	59	1428	1486
LbrM01_V2.0700	*L. braziliensis*	57	1533	1589
LbrM01_V2.0710	*L. braziliensis*	149	119	267
LbrM01_V2.0710	*L. braziliensis*	175	378	552
LbrM01_V2.0730	*L. braziliensis*	236	181	416
LbrM01_V2.0750	*L. braziliensis*	167	1	167
LbrM01_V2.0750	*L. braziliensis*	171	170	340
LbrM01_V2.0750	*L. braziliensis*	58	359	416
LbrM01_V2.0750	*L. braziliensis*	40	457	496
LbrM01_V2.0760	*L. braziliensis*	224	1	224
LbrM01_V2.0770	*L. braziliensis*	121	565	685
LbrM01_V2.0780	*L. braziliensis*	133	679	811
LbrM01_V2.0790	*L. braziliensis*	49	1	49
LbrM01_V2.0790	*L. braziliensis*	49	132	180
LbrM01_V2.0790	*L. braziliensis*	40	248	287
LbrM01_V2.0790	*L. braziliensis*	76	304	379
LbrM01_V2.0790	*L. braziliensis*	148	865	1012
LbrM02_V2.0020	*L. braziliensis*	42	1	42
LbrM02_V2.0050	*L. braziliensis*	42	1	42
LbrM02_V2.0080	*L. braziliensis*	43	88	130
LbrM02_V2.0090	*L. braziliensis*	47	351	397
LbrM02_V2.0100	*L. braziliensis*	42	160	201
LbrM02_V2.0100	*L. braziliensis*	65	754	818
LbrM02_V2.0110	*L. braziliensis*	365	1	365
LbrM02_V2.0110	*L. braziliensis*	158	377	534
LbrM02_V2.0110	*L. braziliensis*	40	882	921
LbrM02_V2.0120	*L. braziliensis*	72	759	830
LbrM02_V2.0130	*L. braziliensis*	40	112	151
LbrM02_V2.0130	*L. braziliensis*	92	156	247
LbrM02_V2.0130	*L. braziliensis*	49	515	563
LbrM02_V2.0130	*L. braziliensis*	158	638	795
LbrM02_V2.0130	*L. braziliensis*	51	1127	1177

LbrM02_V2.0130	*L. braziliensis*	57	1291	1347
LbrM02_V2.0130	*L. braziliensis*	60	1449	1508
LbrM02_V2.0130	*L. braziliensis*	50	2109	2158
LbrM02_V2.0130	*L. braziliensis*	60	2405	2464
LbrM02_V2.0130	*L. braziliensis*	55	2645	2699
LbrM02_V2.0130	*L. braziliensis*	65	2742	2806
LbrM02_V2.0130	*L. braziliensis*	64	3420	3483
LbrM02_V2.0130	*L. braziliensis*	105	3534	3638
LbrM02_V2.0130	*L. braziliensis*	66	4148	4213
LbrM02_V2.0130	*L. braziliensis*	52	4834	4885
LbrM02_V2.0140	*L. braziliensis*	63	54	116
LbrM02_V2.0150	*L. braziliensis*	171	140	310
LbrM02_V2.0150	*L. braziliensis*	41	580	620
LbrM02_V2.0150	*L. braziliensis*	70	766	835
LbrM02_V2.0180	*L. braziliensis*	122	75	196
LbrM02_V2.0180	*L. braziliensis*	76	250	325
LbrM02_V2.0180	*L. braziliensis*	110	588	697
LbrM02_V2.0180	*L. braziliensis*	47	791	837
LbrM02_V2.0180	*L. braziliensis*	41	869	909
LbrM02_V2.0180	*L. braziliensis*	46	942	987
LbrM02_V2.0180	*L. braziliensis*	96	1098	1193
LbrM02_V2.0180	*L. braziliensis*	67	1208	1274
LbrM02_V2.0180	*L. braziliensis*	41	1295	1335
LbrM02_V2.0180	*L. braziliensis*	63	1522	1584
LbrM02_V2.0180	*L. braziliensis*	54	1657	1710
LbrM02_V2.0180	*L. braziliensis*	148	1744	1891
LbrM02_V2.0180	*L. braziliensis*	49	1894	1942
LbrM02_V2.0190	*L. braziliensis*	156	1	156
LbrM02_V2.0190	*L. braziliensis*	56	298	353
LbrM02_V2.0200	*L. braziliensis*	97	40	136
LbrM02_V2.0200	*L. braziliensis*	43	143	185
LbrM02_V2.0200	*L. braziliensis*	61	187	247
LbrM02_V2.0210	*L. braziliensis*	60	30	89
LbrM02_V2.0240	*L. braziliensis*	75	51	125
LbrM02_V2.0240	*L. braziliensis*	66	196	261
LbrM02_V2.0240	*L. braziliensis*	91	533	623
LbrM02_V2.0240	*L. braziliensis*	65	739	803
LbrM02_V2.0250	*L. braziliensis*	40	549	588
LbrM02_V2.0260	*L. braziliensis*	86	24	109
LbrM02_V2.0260	*L. braziliensis*	52	430	481
LbrM02_V2.0260	*L. braziliensis*	42	599	640
LbrM02_V2.0270	*L. braziliensis*	40	96	135
LbrM02_V2.0270	*L. braziliensis*	59	187	245
LbrM02_V2.0270	*L. braziliensis*	41	288	328
LbrM02_V2.0270	*L. braziliensis*	41	521	561
LbrM02_V2.0280	*L. braziliensis*	46	48	93
LbrM02_V2.0280	*L. braziliensis*	41	280	320

LbrM02_V2.0280	*L. braziliensis*	45	528	572
LbrM02_V2.0280	*L. braziliensis*	107	669	775
LbrM02_V2.0280	*L. braziliensis*	40	796	835
LbrM02_V2.0280	*L. braziliensis*	80	858	937
LbrM02_V2.0280	*L. braziliensis*	42	1039	1080
LbrM02_V2.0320	*L. braziliensis*	94	205	298
LbrM02_V2.0320	*L. braziliensis*	68	393	460
LbrM02_V2.0320	*L. braziliensis*	155	543	697
LbrM02_V2.0320	*L. braziliensis*	61	963	1023
LbrM02_V2.0330	*L. braziliensis*	96	72	167
LbrM02_V2.0340	*L. braziliensis*	110	18	127
LbrM02_V2.0340	*L. braziliensis*	41	455	495
LbrM02_V2.0350	*L. braziliensis*	99	1	99
LbrM02_V2.0350	*L. braziliensis*	54	450	503
LbrM02_V2.0350	*L. braziliensis*	78	774	851
LbrM02_V2.0350	*L. braziliensis*	190	1224	1413
LbrM02_V2.0350	*L. braziliensis*	65	1851	1915
LbrM02_V2.0360	*L. braziliensis*	78	1	78
LbrM02_V2.0360	*L. braziliensis*	49	108	156
LbrM02_V2.0360	*L. braziliensis*	100	168	267
LbrM02_V2.0360	*L. braziliensis*	51	456	506
LbrM02_V2.0370	*L. braziliensis*	51	1	51
LbrM02_V2.0370	*L. braziliensis*	98	113	210
LbrM02_V2.0370	*L. braziliensis*	125	304	428
LbrM02_V2.0370	*L. braziliensis*	68	564	631
LbrM02_V2.0370	*L. braziliensis*	80	679	758
LbrM02_V2.0370	*L. braziliensis*	60	823	882
LbrM02_V2.0380	*L. braziliensis*	77	1	77
LbrM02_V2.0380	*L. braziliensis*	339	110	448
LbrM02_V2.0380	*L. braziliensis*	50	477	526
LbrM02_V2.0380	*L. braziliensis*	54	554	607
LbrM02_V2.0380	*L. braziliensis*	65	829	893
LbrM02_V2.0380	*L. braziliensis*	130	1026	1155
LbrM02_V2.0400	*L. braziliensis*	57	476	532
LbrM02_V2.0410	*L. braziliensis*	46	122	167
LbrM02_V2.0410	*L. braziliensis*	99	260	358
LbrM02_V2.0410	*L. braziliensis*	64	394	457
LbrM02_V2.0420	*L. braziliensis*	59	43	101
LbrM02_V2.0430	*L. braziliensis*	66	1	66
LbrM02_V2.0430	*L. braziliensis*	120	104	223
LbrM02_V2.0430	*L. braziliensis*	121	255	375
LbrM02_V2.0430	*L. braziliensis*	156	434	589
LbrM02_V2.0430	*L. braziliensis*	76	626	701
LbrM02_V2.0430	*L. braziliensis*	51	815	865
LbrM02_V2.0450	*L. braziliensis*	366	1	366
LbrM02_V2.0450	*L. braziliensis*	368	393	760
LbrM02_V2.0450	*L. braziliensis*	63	774	836

LbrM02_V2.0450	*L. braziliensis*	558	890	1447
LbrM02_V2.0450	*L. braziliensis*	128	1464	1591
LbrM02_V2.0450	*L. braziliensis*	354	1605	1958
LbrM02_V2.0450	*L. braziliensis*	106	2054	2159
LbrM02_V2.0450	*L. braziliensis*	342	2177	2518
LbrM02_V2.0450	*L. braziliensis*	166	2543	2708
LbrM02_V2.0450	*L. braziliensis*	113	2731	2843
LbrM02_V2.0460	*L. braziliensis*	146	1	146
LbrM02_V2.0470	*L. braziliensis*	49	32	80
LbrM02_V2.0470	*L. braziliensis*	46	110	155
LbrM02_V2.0470	*L. braziliensis*	61	175	235
LbrM02_V2.0470	*L. braziliensis*	207	256	462
LbrM02_V2.0470	*L. braziliensis*	141	508	648
LbrM02_V2.0470	*L. braziliensis*	66	681	746
LbrM02_V2.0470	*L. braziliensis*	128	800	927
LbrM02_V2.0470	*L. braziliensis*	89	996	1084
LbrM02_V2.0480	*L. braziliensis*	104	1	104
LbrM02_V2.0480	*L. braziliensis*	57	140	196
LbrM02_V2.0480	*L. braziliensis*	78	513	590
LbrM02_V2.0490	*L. braziliensis*	137	34	170
LbrM02_V2.0500	*L. braziliensis*	122	64	185
LbrM02_V2.0500	*L. braziliensis*	95	232	326
LbrM02_V2.0500	*L. braziliensis*	77	359	435
LbrM02_V2.0500	*L. braziliensis*	62	469	530
LbrM02_V2.0500	*L. braziliensis*	172	546	717
LbrM02_V2.0510	*L. braziliensis*	61	161	221
LbrM02_V2.0510	*L. braziliensis*	50	251	300
LbrM02_V2.0510	*L. braziliensis*	48	463	510
LbrM02_V2.0520	*L. braziliensis*	87	19	105
LbrM02_V2.0530	*L. braziliensis*	68	1	68
LbrM02_V2.0530	*L. braziliensis*	75	436	510
LbrM02_V2.0530	*L. braziliensis*	284	552	835
LbrM02_V2.0540	*L. braziliensis*	180	1	180
LbrM02_V2.0540	*L. braziliensis*	50	205	254
LbrM02_V2.0540	*L. braziliensis*	157	260	416
LbrM02_V2.0540	*L. braziliensis*	125	656	780
LbrM02_V2.0540	*L. braziliensis*	82	804	885
LbrM02_V2.0540	*L. braziliensis*	56	1006	1061
LbrM02_V2.0540	*L. braziliensis*	57	1075	1131
LbrM02_V2.0540	*L. braziliensis*	86	1363	1448
LbrM02_V2.0550	*L. braziliensis*	193	283	475
LbrM02_V2.0550	*L. braziliensis*	63	498	560
LbrM02_V2.0550	*L. braziliensis*	136	600	735
LbrM02_V2.0560	*L. braziliensis*	53	826	878
LbrM02_V2.0560	*L. braziliensis*	90	923	1012
LbrM02_V2.0560	*L. braziliensis*	91	1104	1194
LbrM02_V2.0560	*L. braziliensis*	45	1622	1666

LbrM02_V2.0560	*L. braziliensis*	85	1777	1861
LbrM02_V2.0560	*L. braziliensis*	195	2029	2223
LbrM02_V2.0560	*L. braziliensis*	55	2241	2295
LbrM02_V2.0560	*L. braziliensis*	120	2528	2647
LbrM02_V2.0560	*L. braziliensis*	67	2650	2716
LbrM02_V2.0560	*L. braziliensis*	71	2927	2997
LbrM02_V2.0560	*L. braziliensis*	40	3018	3057
LbrM02_V2.0560	*L. braziliensis*	64	3149	3212
LbrM02_V2.0560	*L. braziliensis*	47	3344	3390
LbrM02_V2.0560	*L. braziliensis*	263	3425	3687
LbrM02_V2.0570	*L. braziliensis*	148	1	148
LbrM02_V2.0570	*L. braziliensis*	101	269	369
LbrM02_V2.0570	*L. braziliensis*	128	378	505
LbrM02_V2.0590	*L. braziliensis*	84	1	84
LbrM02_V2.0590	*L. braziliensis*	217	105	321
LbrM02_V2.0600	*L. braziliensis*	77	18	94
LbrM02_V2.0610	*L. braziliensis*	162	62	223
LbrM02_V2.0610	*L. braziliensis*	55	286	340
LbrM02_V2.0620	*L. braziliensis*	76	85	160
LbrM02_V2.0620	*L. braziliensis*	171	263	433
LbrM02_V2.0620	*L. braziliensis*	40	1366	1405
LbrM02_V2.0630	*L. braziliensis*	44	1	44
LbrM02_V2.0630	*L. braziliensis*	79	108	186
LbrM02_V2.0640	*L. braziliensis*	417	1	417
LbrM02_V2.0640	*L. braziliensis*	75	426	500
LbrM02_V2.0640	*L. braziliensis*	144	763	906
LbrM02_V2.0660	*L. braziliensis*	115	1	115
LbrM02_V2.0660	*L. braziliensis*	53	253	305
LbrM02_V2.0680	*L. braziliensis*	59	1	59
LbrM02_V2.0680	*L. braziliensis*	103	68	170
LbrM02_V2.0680	*L. braziliensis*	93	176	268
LbrM02_V2.0680	*L. braziliensis*	64	302	365
LbrM02_V2.0680	*L. braziliensis*	104	411	514
LbrM02_V2.0690	*L. braziliensis*	65	1	65
LbrM02_V2.0690	*L. braziliensis*	133	395	527
LbrM02_V2.0700	*L. braziliensis*	45	17	61
LbrM02_V2.0700	*L. braziliensis*	67	97	163
LbrM02_V2.0700	*L. braziliensis*	72	186	257
LbrM02_V2.0730	*L. braziliensis*	87	1	87
LbrM02_V2.0730	*L. braziliensis*	52	133	184
LbrM02_V2.0730	*L. braziliensis*	203	189	391
LbrM02_V2.0730	*L. braziliensis*	603	403	1005
LbrM02_V2.0730	*L. braziliensis*	685	1019	1703
LbrM02_V2.0730	*L. braziliensis*	139	1827	1965
LbrM02_V2.0740	*L. braziliensis*	364	1	364
LbrM02_V2.0740	*L. braziliensis*	157	378	534
LbrM02_V2.0750	*L. braziliensis*	64	1	64

LbrM02_V2.0750	*L. braziliensis*	41	106	146
LbrM02_V2.0750	*L. braziliensis*	59	190	248
LbrM02_V2.0750	*L. braziliensis*	57	271	327
LbrM02_V2.0750	*L. braziliensis*	77	368	444
LbrM02_V2.0750	*L. braziliensis*	73	466	538
LbrM02_V2.0750	*L. braziliensis*	82	560	641
LbrM02_V2.0750	*L. braziliensis*	56	679	734
LbrM02_V2.0750	*L. braziliensis*	77	760	836
LbrM02_V2.0750	*L. braziliensis*	191	1166	1356
LbrM02_V2.0750	*L. braziliensis*	66	1379	1444
LbrM02_V2.0750	*L. braziliensis*	142	1475	1616
LbrM02_V2.0760	*L. braziliensis*	152	287	438
LbrM02_V2.0770	*L. braziliensis*	45	17	61
LbrM02_V2.0770	*L. braziliensis*	58	107	164
LbrM02_V2.0770	*L. braziliensis*	73	185	257
LbrM02_V2.0770	*L. braziliensis*	86	283	368
LbrM03_V2.0010	*L. braziliensis*	76	195	270
LbrM03_V2.0020	*L. braziliensis*	42	1	42
LbrM03_V2.0020	*L. braziliensis*	79	86	164
LbrM03_V2.0020	*L. braziliensis*	79	86	164
LbrM03_V2.0040	*L. braziliensis*	93	17	109
LbrM03_V2.0090	*L. braziliensis*	108	1	108
LbrM03_V2.0100	*L. braziliensis*	59	83	141
LbrM03_V2.0100	*L. braziliensis*	40	528	567
LbrM03_V2.0100	*L. braziliensis*	40	528	567
LbrM03_V2.0110	*L. braziliensis*	50	11	60
LbrM03_V2.0120	*L. braziliensis*	337	172	508
LbrM03_V2.0120	*L. braziliensis*	86	589	674
LbrM03_V2.0120	*L. braziliensis*	193	706	898
LbrM03_V2.0130	*L. braziliensis*	485	162	646
LbrM03_V2.0130	*L. braziliensis*	141	729	869
LbrM03_V2.0150	*L. braziliensis*	95	45	139
LbrM03_V2.0170	*L. braziliensis*	108	91	198
LbrM03_V2.0170	*L. braziliensis*	44	438	481
LbrM03_V2.0170	*L. braziliensis*	44	438	481
LbrM03_V2.0180	*L. braziliensis*	112	58	169
LbrM03_V2.0190	*L. braziliensis*	193	1	193
LbrM03_V2.0190	*L. braziliensis*	48	368	415
LbrM03_V2.0190	*L. braziliensis*	48	368	415
LbrM03_V2.0200	*L. braziliensis*	52	159	210
LbrM03_V2.0230	*L. braziliensis*	47	43	89
LbrM03_V2.0270	*L. braziliensis*	52	1	52
LbrM03_V2.0280	*L. braziliensis*	61	87	147
LbrM03_V2.0280	*L. braziliensis*	727	150	876
LbrM03_V2.0280	*L. braziliensis*	154	961	1114
LbrM03_V2.0280	*L. braziliensis*	97	1130	1226
LbrM03_V2.0280	*L. braziliensis*	96	1240	1335

LbrM03_V2.0280	*L. braziliensis*	75	1365	1439
LbrM03_V2.0290	*L. braziliensis*	43	1	43
LbrM03_V2.0290	*L. braziliensis*	55	50	104
LbrM03_V2.0290	*L. braziliensis*	149	141	289
LbrM03_V2.0290	*L. braziliensis*	124	382	505
LbrM03_V2.0290	*L. braziliensis*	92	653	744
LbrM03_V2.0290	*L. braziliensis*	179	803	981
LbrM03_V2.0300	*L. braziliensis*	80	1	80
LbrM03_V2.0300	*L. braziliensis*	230	94	323
LbrM03_V2.0300	*L. braziliensis*	265	334	598
LbrM03_V2.0300	*L. braziliensis*	200	621	820
LbrM03_V2.0300	*L. braziliensis*	341	833	1173
LbrM03_V2.0300	*L. braziliensis*	702	1186	1887
LbrM03_V2.0310	*L. braziliensis*	100	1	100
LbrM03_V2.0310	*L. braziliensis*	299	132	430
LbrM03_V2.0310	*L. braziliensis*	149	439	587
LbrM03_V2.0310	*L. braziliensis*	102	718	819
LbrM03_V2.0310	*L. braziliensis*	119	828	946
LbrM03_V2.0320	*L. braziliensis*	41	44	84
LbrM03_V2.0320	*L. braziliensis*	74	238	311
LbrM03_V2.0320	*L. braziliensis*	62	387	448
LbrM03_V2.0320	*L. braziliensis*	45	635	679
LbrM03_V2.0330	*L. braziliensis*	209	31	239
LbrM03_V2.0340	*L. braziliensis*	363	1	363
LbrM03_V2.0340	*L. braziliensis*	69	424	492
LbrM03_V2.0340	*L. braziliensis*	188	599	786
LbrM03_V2.0340	*L. braziliensis*	59	956	1014
LbrM03_V2.0340	*L. braziliensis*	85	1022	1106
LbrM03_V2.0340	*L. braziliensis*	77	1125	1201
LbrM03_V2.0340	*L. braziliensis*	87	1295	1381
LbrM03_V2.0340	*L. braziliensis*	102	1407	1508
LbrM03_V2.0350	*L. braziliensis*	63	1	63
LbrM03_V2.0360	*L. braziliensis*	135	1	135
LbrM03_V2.0370	*L. braziliensis*	47	36	82
LbrM03_V2.0370	*L. braziliensis*	110	289	398
LbrM03_V2.0370	*L. braziliensis*	48	640	687
LbrM03_V2.0380	*L. braziliensis*	213	1	213
LbrM03_V2.0380	*L. braziliensis*	79	383	461
LbrM03_V2.0380	*L. braziliensis*	106	487	592
LbrM03_V2.0380	*L. braziliensis*	56	638	693
LbrM03_V2.0380	*L. braziliensis*	52	782	833
LbrM03_V2.0380	*L. braziliensis*	40	976	1015
LbrM03_V2.0380	*L. braziliensis*	43	1149	1191
LbrM03_V2.0380	*L. braziliensis*	121	1312	1432
LbrM03_V2.0380	*L. braziliensis*	103	1493	1595
LbrM03_V2.0380	*L. braziliensis*	65	1690	1754
LbrM03_V2.0390	*L. braziliensis*	55	52	106

LbrM03_V2.0400	*L. braziliensis*	56	52	107
LbrM03_V2.0410	*L. braziliensis*	124	74	197
LbrM03_V2.0410	*L. braziliensis*	173	316	488
LbrM03_V2.0410	*L. braziliensis*	66	530	595
LbrM03_V2.0410	*L. braziliensis*	87	748	834
LbrM03_V2.0410	*L. braziliensis*	199	860	1058
LbrM03_V2.0410	*L. braziliensis*	52	1127	1178
LbrM03_V2.0410	*L. braziliensis*	69	1213	1281
LbrM03_V2.0410	*L. braziliensis*	48	1307	1354
LbrM03_V2.0410	*L. braziliensis*	54	1436	1489
LbrM03_V2.0420	*L. braziliensis*	101	1	101
LbrM03_V2.0420	*L. braziliensis*	795	126	920
LbrM03_V2.0420	*L. braziliensis*	546	950	1495
LbrM03_V2.0420	*L. braziliensis*	68	1567	1634
LbrM03_V2.0420	*L. braziliensis*	80	1659	1738
LbrM03_V2.0420	*L. braziliensis*	77	1819	1895
LbrM03_V2.0420	*L. braziliensis*	81	1898	1978
LbrM03_V2.0420	*L. braziliensis*	65	2058	2122
LbrM03_V2.0420	*L. braziliensis*	45	2207	2251
LbrM03_V2.0430	*L. braziliensis*	341	1	341
LbrM03_V2.0430	*L. braziliensis*	42	524	565
LbrM03_V2.0430	*L. braziliensis*	45	637	681
LbrM03_V2.0430	*L. braziliensis*	88	745	832
LbrM03_V2.0440	*L. braziliensis*	60	92	151
LbrM03_V2.0440	*L. braziliensis*	67	798	864
LbrM03_V2.0440	*L. braziliensis*	45	914	958
LbrM03_V2.0440	*L. braziliensis*	62	970	1031
LbrM03_V2.0440	*L. braziliensis*	76	1064	1139
LbrM03_V2.0440	*L. braziliensis*	65	1180	1244
LbrM03_V2.0440	*L. braziliensis*	80	1283	1362
LbrM03_V2.0440	*L. braziliensis*	60	1471	1530
LbrM03_V2.0440	*L. braziliensis*	96	1624	1719
LbrM03_V2.0450	*L. braziliensis*	50	1	50
LbrM03_V2.0460	*L. braziliensis*	81	1	81
LbrM03_V2.0460	*L. braziliensis*	51	231	281
LbrM03_V2.0460	*L. braziliensis*	43	352	394
LbrM03_V2.0460	*L. braziliensis*	90	420	509
LbrM03_V2.0460	*L. braziliensis*	391	710	1100
LbrM03_V2.0460	*L. braziliensis*	86	1293	1378
LbrM03_V2.0470	*L. braziliensis*	89	1	89
LbrM03_V2.0470	*L. braziliensis*	276	119	394
LbrM03_V2.0470	*L. braziliensis*	89	449	537
LbrM03_V2.0470	*L. braziliensis*	51	564	614
LbrM03_V2.0490	*L. braziliensis*	100	81	180
LbrM03_V2.0500	*L. braziliensis*	71	24	94
LbrM03_V2.0500	*L. braziliensis*	335	126	460
LbrM03_V2.0500	*L. braziliensis*	106	563	668

LbrM03_V2.0500	*L. braziliensis*	61	839	899
LbrM03_V2.0520	*L. braziliensis*	53	124	176
LbrM03_V2.0520	*L. braziliensis*	49	549	597
LbrM03_V2.0520	*L. braziliensis*	50	633	682
LbrM03_V2.0540	*L. braziliensis*	57	53	109
LbrM03_V2.0540	*L. braziliensis*	54	212	265
LbrM03_V2.0550	*L. braziliensis*	43	14	56
LbrM03_V2.0550	*L. braziliensis*	136	62	197
LbrM03_V2.0550	*L. braziliensis*	61	487	547
LbrM03_V2.0550	*L. braziliensis*	72	701	772
LbrM03_V2.0560	*L. braziliensis*	104	8	111
LbrM03_V2.0560	*L. braziliensis*	63	136	198
LbrM03_V2.0560	*L. braziliensis*	57	514	570
LbrM03_V2.0590	*L. braziliensis*	43	29	71
LbrM03_V2.0590	*L. braziliensis*	61	284	344
LbrM03_V2.0590	*L. braziliensis*	67	709	775
LbrM03_V2.0590	*L. braziliensis*	57	1444	1500
LbrM03_V2.0590	*L. braziliensis*	65	1549	1613
LbrM03_V2.0600	*L. braziliensis*	58	21	78
LbrM03_V2.0600	*L. braziliensis*	179	148	326
LbrM03_V2.0600	*L. braziliensis*	51	450	500
LbrM03_V2.0600	*L. braziliensis*	53	564	616
LbrM03_V2.0600	*L. braziliensis*	148	640	787
LbrM03_V2.0600	*L. braziliensis*	63	800	862
LbrM03_V2.0600	*L. braziliensis*	70	920	989
LbrM03_V2.0600	*L. braziliensis*	52	1029	1080
LbrM03_V2.0600	*L. braziliensis*	59	1162	1220
LbrM03_V2.0600	*L. braziliensis*	67	1350	1416
LbrM03_V2.0600	*L. braziliensis*	70	1465	1534
LbrM03_V2.0600	*L. braziliensis*	65	1682	1746
LbrM03_V2.0600	*L. braziliensis*	90	1755	1844
LbrM03_V2.0600	*L. braziliensis*	57	1970	2026
LbrM03_V2.0600	*L. braziliensis*	133	2191	2323
LbrM03_V2.0600	*L. braziliensis*	68	2361	2428
LbrM03_V2.0600	*L. braziliensis*	128	2450	2577
LbrM03_V2.0600	*L. braziliensis*	47	2648	2694
LbrM03_V2.0610	*L. braziliensis*	59	43	101
LbrM03_V2.0610	*L. braziliensis*	41	214	254
LbrM03_V2.0610	*L. braziliensis*	41	214	254
LbrM03_V2.0620	*L. braziliensis*	163	1	163
LbrM03_V2.0620	*L. braziliensis*	56	274	329
LbrM03_V2.0620	*L. braziliensis*	65	402	466
LbrM03_V2.0620	*L. braziliensis*	138	611	748
LbrM03_V2.0620	*L. braziliensis*	96	968	1063
LbrM03_V2.0630	*L. braziliensis*	125	40	164
LbrM03_V2.0630	*L. braziliensis*	43	230	272
LbrM03_V2.0630	*L. braziliensis*	54	354	407

LbrM03_V2.0630	*L. braziliensis*	52	439	490
LbrM03_V2.0650	*L. braziliensis*	79	1	79
LbrM03_V2.0650	*L. braziliensis*	45	377	421
LbrM03_V2.0650	*L. braziliensis*	286	497	782
LbrM03_V2.0680	*L. braziliensis*	209	32	240
LbrM03_V2.0680	*L. braziliensis*	221	262	482
LbrM03_V2.0680	*L. braziliensis*	46	566	611
LbrM03_V2.0680	*L. braziliensis*	216	689	904
LbrM03_V2.0680	*L. braziliensis*	84	971	1054
LbrM03_V2.0680	*L. braziliensis*	428	1064	1491
LbrM03_V2.0680	*L. braziliensis*	527	1523	2049
LbrM03_V2.0680	*L. braziliensis*	51	2058	2108
LbrM03_V2.0680	*L. braziliensis*	48	2134	2181
LbrM03_V2.0680	*L. braziliensis*	295	2202	2496
LbrM03_V2.0690	*L. braziliensis*	84	20	103
LbrM03_V2.0690	*L. braziliensis*	164	246	409
LbrM03_V2.0700	*L. braziliensis*	175	1	175
LbrM03_V2.0700	*L. braziliensis*	142	196	337
LbrM03_V2.0700	*L. braziliensis*	330	435	764
LbrM03_V2.0700	*L. braziliensis*	391	813	1203
LbrM03_V2.0700	*L. braziliensis*	161	1504	1664
LbrM03_V2.0700	*L. braziliensis*	52	1709	1760
LbrM03_V2.0700	*L. braziliensis*	75	1898	1972
LbrM03_V2.0720	*L. braziliensis*	123	1	123
LbrM03_V2.0720	*L. braziliensis*	146	167	312
LbrM03_V2.0720	*L. braziliensis*	204	330	533
LbrM03_V2.0720	*L. braziliensis*	41	563	603
LbrM03_V2.0720	*L. braziliensis*	134	757	890
LbrM03_V2.0720	*L. braziliensis*	74	927	1000
LbrM03_V2.0720	*L. braziliensis*	47	1053	1099
LbrM03_V2.0720	*L. braziliensis*	100	1175	1274
LbrM03_V2.0730	*L. braziliensis*	249	1	249
LbrM03_V2.0730	*L. braziliensis*	101	282	382
LbrM03_V2.0730	*L. braziliensis*	251	386	636
LbrM03_V2.0730	*L. braziliensis*	111	661	771
LbrM03_V2.0730	*L. braziliensis*	60	808	867
LbrM03_V2.0730	*L. braziliensis*	666	878	1543
LbrM03_V2.0730	*L. braziliensis*	74	1642	1715
LbrM03_V2.0730	*L. braziliensis*	95	1780	1874
LbrM03_V2.0730	*L. braziliensis*	170	1953	2122
LbrM03_V2.0730	*L. braziliensis*	417	2213	2629
LbrM03_V2.0730	*L. braziliensis*	229	2642	2870
LbrM03_V2.0740	*L. braziliensis*	52	46	97
LbrM03_V2.0750	*L. braziliensis*	90	55	144
LbrM03_V2.0750	*L. braziliensis*	59	225	283
LbrM03_V2.0750	*L. braziliensis*	80	494	573
LbrM03_V2.0750	*L. braziliensis*	44	711	754

LbrM03_V2.0750	*L. braziliensis*	114	865	978
LbrM03_V2.0760	*L. braziliensis*	90	1	90
LbrM03_V2.0760	*L. braziliensis*	540	121	660
LbrM03_V2.0760	*L. braziliensis*	60	671	730
LbrM03_V2.0760	*L. braziliensis*	57	743	799
LbrM03_V2.0760	*L. braziliensis*	49	823	871
LbrM03_V2.0760	*L. braziliensis*	54	933	986
LbrM03_V2.0760	*L. braziliensis*	288	1145	1432
LbrM03_V2.0760	*L. braziliensis*	76	1458	1533
LbrM03_V2.0760	*L. braziliensis*	224	1538	1761
LbrM03_V2.0760	*L. braziliensis*	95	1853	1947
LbrM03_V2.0760	*L. braziliensis*	280	1951	2230
LbrM03_V2.0770	*L. braziliensis*	116	39	154
LbrM03_V2.0770	*L. braziliensis*	57	353	409
LbrM03_V2.0770	*L. braziliensis*	87	713	799
LbrM03_V2.0770	*L. braziliensis*	100	1022	1121
LbrM03_V2.0780	*L. braziliensis*	276	159	434
LbrM03_V2.0780	*L. braziliensis*	200	694	893
LbrM03_V2.0780	*L. braziliensis*	326	895	1220
LbrM03_V2.0790	*L. braziliensis*	111	159	269
LbrM03_V2.0790	*L. braziliensis*	40	402	441
LbrM03_V2.0790	*L. braziliensis*	97	521	617
LbrM03_V2.0790	*L. braziliensis*	46	703	748
LbrM03_V2.0790	*L. braziliensis*	50	756	805
LbrM03_V2.0790	*L. braziliensis*	55	1246	1300
LbrM03_V2.0790	*L. braziliensis*	51	1726	1776
LbrM03_V2.0800	*L. braziliensis*	103	62	164
LbrM03_V2.0800	*L. braziliensis*	98	203	300
LbrM03_V2.0800	*L. braziliensis*	144	429	572
LbrM03_V2.0800	*L. braziliensis*	108	603	710
LbrM03_V2.0810	*L. braziliensis*	49	211	259
LbrM03_V2.0810	*L. braziliensis*	79	294	372
LbrM03_V2.0850	*L. braziliensis*	138	8	145
LbrM03_V2.0890	*L. braziliensis*	60	354	413
LbrM03_V2.0920	*L. braziliensis*	43	32	74
LbrM03_V2.0950	*L. braziliensis*	210	1	210
LbrM03_V2.0950	*L. braziliensis*	155	244	398
LbrM03_V2.0950	*L. braziliensis*	42	417	458
LbrM03_V2.0950	*L. braziliensis*	60	460	519
LbrM03_V2.0950	*L. braziliensis*	68	549	616
LbrM03_V2.0950	*L. braziliensis*	98	637	734
LbrM03_V2.0950	*L. braziliensis*	183	828	1010
LbrM03_V2.0960	*L. braziliensis*	47	1	47
LbrM03_V2.0960	*L. braziliensis*	48	366	413
LbrM03_V2.0960	*L. braziliensis*	51	732	782
LbrM03_V2.0970	*L. braziliensis*	173	186	358
LbrM03_V2.0970	*L. braziliensis*	67	1097	1163

LbrM03_V2.0970	*L. braziliensis*	114	1474	1587
LbrM03_V2.0970	*L. braziliensis*	59	1798	1856
LbrM03_V2.0970	*L. braziliensis*	92	1897	1988
LbrM03_V2.0970	*L. braziliensis*	40	2019	2058
LbrM04_V2.0050	*L. braziliensis*	45	218	262
LbrM04_V2.0070	*L. braziliensis*	86	1	86
LbrM04_V2.0070	*L. braziliensis*	80	501	580
LbrM04_V2.0070	*L. braziliensis*	53	692	744
LbrM04_V2.0080	*L. braziliensis*	64	40	103
LbrM04_V2.0080	*L. braziliensis*	45	171	215
LbrM04_V2.0100	*L. braziliensis*	202	1	202
LbrM04_V2.0100	*L. braziliensis*	127	250	376
LbrM04_V2.0100	*L. braziliensis*	143	407	549
LbrM04_V2.0110	*L. braziliensis*	191	1	191
LbrM04_V2.0130	*L. braziliensis*	59	1	59
LbrM04_V2.0130	*L. braziliensis*	78	77	154
LbrM04_V2.0130	*L. braziliensis*	78	77	154
LbrM04_V2.0140	*L. braziliensis*	50	1027	1076
LbrM04_V2.0140	*L. braziliensis*	63	1261	1323
LbrM04_V2.0140	*L. braziliensis*	63	1261	1323
LbrM04_V2.0150	*L. braziliensis*	101	57	157
LbrM04_V2.0150	*L. braziliensis*	113	207	319
LbrM04_V2.0150	*L. braziliensis*	119	478	596
LbrM04_V2.0160	*L. braziliensis*	71	77	147
LbrM04_V2.0160	*L. braziliensis*	111	235	345
LbrM04_V2.0180	*L. braziliensis*	134	25	158
LbrM04_V2.0190	*L. braziliensis*	103	1	103
LbrM04_V2.0190	*L. braziliensis*	138	124	261
LbrM04_V2.0190	*L. braziliensis*	41	428	468
LbrM04_V2.0200	*L. braziliensis*	80	11	90
LbrM04_V2.0200	*L. braziliensis*	98	134	231
LbrM04_V2.0200	*L. braziliensis*	98	134	231
LbrM04_V2.0210	*L. braziliensis*	67	1	67
LbrM04_V2.0210	*L. braziliensis*	46	110	155
LbrM04_V2.0210	*L. braziliensis*	46	110	155
LbrM04_V2.0220	*L. braziliensis*	47	1	47
LbrM04_V2.0220	*L. braziliensis*	48	104	151
LbrM04_V2.0220	*L. braziliensis*	107	425	531
LbrM04_V2.0230	*L. braziliensis*	97	373	469
LbrM04_V2.0240	*L. braziliensis*	45	53	97
LbrM04_V2.0240	*L. braziliensis*	77	251	327
LbrM04_V2.0240	*L. braziliensis*	71	372	442
LbrM04_V2.0250	*L. braziliensis*	43	55	97
LbrM04_V2.0250	*L. braziliensis*	77	251	327
LbrM04_V2.0250	*L. braziliensis*	70	373	442
LbrM04_V2.0260	*L. braziliensis*	163	230	392
LbrM04_V2.0270	*L. braziliensis*	66	293	358

LbrM04_V2.0280	*L. braziliensis*	110	21	130
LbrM04_V2.0290	*L. braziliensis*	135	223	357
LbrM04_V2.0290	*L. braziliensis*	75	493	567
LbrM04_V2.0290	*L. braziliensis*	84	619	702
LbrM04_V2.0290	*L. braziliensis*	119	745	863
LbrM04_V2.0290	*L. braziliensis*	58	897	954
LbrM04_V2.0300	*L. braziliensis*	42	1	42
LbrM04_V2.0300	*L. braziliensis*	54	80	133
LbrM04_V2.0300	*L. braziliensis*	48	228	275
LbrM04_V2.0310	*L. braziliensis*	43	69	111
LbrM04_V2.0310	*L. braziliensis*	61	235	295
LbrM04_V2.0320	*L. braziliensis*	265	1	265
LbrM04_V2.0320	*L. braziliensis*	62	545	606
LbrM04_V2.0320	*L. braziliensis*	62	939	1000
LbrM04_V2.0320	*L. braziliensis*	50	1276	1325
LbrM04_V2.0320	*L. braziliensis*	254	1689	1942
LbrM04_V2.0350	*L. braziliensis*	49	442	490
LbrM04_V2.0360	*L. braziliensis*	40	17	56
LbrM04_V2.0360	*L. braziliensis*	42	360	401
LbrM04_V2.0360	*L. braziliensis*	42	425	466
LbrM04_V2.0380	*L. braziliensis*	214	1	214
LbrM04_V2.0380	*L. braziliensis*	73	218	290
LbrM04_V2.0380	*L. braziliensis*	358	372	729
LbrM04_V2.0380	*L. braziliensis*	139	740	878
LbrM04_V2.0380	*L. braziliensis*	83	924	1006
LbrM04_V2.0380	*L. braziliensis*	167	1126	1292
LbrM04_V2.0380	*L. braziliensis*	54	1318	1371
LbrM04_V2.0380	*L. braziliensis*	62	1429	1490
LbrM04_V2.0380	*L. braziliensis*	49	1560	1608
LbrM04_V2.0380	*L. braziliensis*	50	1734	1783
LbrM04_V2.0380	*L. braziliensis*	58	1989	2046
LbrM04_V2.0390	*L. braziliensis*	58	63	120
LbrM04_V2.0390	*L. braziliensis*	103	189	291
LbrM04_V2.0410	*L. braziliensis*	49	1	49
LbrM04_V2.0410	*L. braziliensis*	77	281	357
LbrM04_V2.0420	*L. braziliensis*	50	1	50
LbrM04_V2.0420	*L. braziliensis*	77	282	358
LbrM04_V2.0430	*L. braziliensis*	71	189	259
LbrM04_V2.0440	*L. braziliensis*	50	1	50
LbrM04_V2.0440	*L. braziliensis*	80	172	251
LbrM04_V2.0450	*L. braziliensis*	294	1	294
LbrM04_V2.0450	*L. braziliensis*	135	307	441
LbrM04_V2.0450	*L. braziliensis*	308	534	841
LbrM04_V2.0450	*L. braziliensis*	58	875	932
LbrM04_V2.0450	*L. braziliensis*	54	1003	1056
LbrM04_V2.0460	*L. braziliensis*	57	292	348
LbrM04_V2.0480	*L. braziliensis*	227	1	227

LbrM04_V2.0480	*L. braziliensis*	61	230	290
LbrM04_V2.0480	*L. braziliensis*	164	304	467
LbrM04_V2.0480	*L. braziliensis*	344	514	857
LbrM04_V2.0480	*L. braziliensis*	49	905	953
LbrM04_V2.0480	*L. braziliensis*	83	970	1052
LbrM04_V2.0480	*L. braziliensis*	45	1194	1238
LbrM04_V2.0490	*L. braziliensis*	145	1	145
LbrM04_V2.0510	*L. braziliensis*	44	1	44
LbrM04_V2.0510	*L. braziliensis*	222	98	319
LbrM04_V2.0510	*L. braziliensis*	60	665	724
LbrM04_V2.0510	*L. braziliensis*	64	757	820
LbrM04_V2.0520	*L. braziliensis*	50	139	188
LbrM04_V2.0540	*L. braziliensis*	83	1	83
LbrM04_V2.0540	*L. braziliensis*	206	120	325
LbrM04_V2.0550	*L. braziliensis*	587	1	587
LbrM04_V2.0550	*L. braziliensis*	435	619	1053
LbrM04_V2.0550	*L. braziliensis*	257	1140	1396
LbrM04_V2.0550	*L. braziliensis*	209	1433	1641
LbrM04_V2.0550	*L. braziliensis*	71	1763	1833
LbrM04_V2.0550	*L. braziliensis*	252	1933	2184
LbrM04_V2.0550	*L. braziliensis*	397	2304	2700
LbrM04_V2.0550	*L. braziliensis*	119	2750	2868
LbrM04_V2.0550	*L. braziliensis*	66	2870	2935
LbrM04_V2.0550	*L. braziliensis*	110	2946	3055
LbrM04_V2.0560	*L. braziliensis*	184	1	184
LbrM04_V2.0560	*L. braziliensis*	261	260	520
LbrM04_V2.0560	*L. braziliensis*	346	667	1012
LbrM04_V2.0560	*L. braziliensis*	69	1252	1320
LbrM04_V2.0570	*L. braziliensis*	57	94	150
LbrM04_V2.0570	*L. braziliensis*	79	295	373
LbrM04_V2.0570	*L. braziliensis*	43	452	494
LbrM04_V2.0570	*L. braziliensis*	97	871	967
LbrM04_V2.0580	*L. braziliensis*	95	1	95
LbrM04_V2.0580	*L. braziliensis*	125	164	288
LbrM04_V2.0580	*L. braziliensis*	57	316	372
LbrM04_V2.0590	*L. braziliensis*	69	42	110
LbrM04_V2.0600	*L. braziliensis*	42	904	945
LbrM04_V2.0600	*L. braziliensis*	42	1179	1220
LbrM04_V2.0600	*L. braziliensis*	70	1278	1347
LbrM04_V2.0610	*L. braziliensis*	88	200	287
LbrM04_V2.0620	*L. braziliensis*	149	1	149
LbrM04_V2.0620	*L. braziliensis*	69	531	599
LbrM04_V2.0620	*L. braziliensis*	41	619	659
LbrM04_V2.0620	*L. braziliensis*	49	693	741
LbrM04_V2.0620	*L. braziliensis*	70	772	841
LbrM04_V2.0620	*L. braziliensis*	58	1024	1081
LbrM04_V2.0620	*L. braziliensis*	73	1115	1187

LbrM04_V2.0620	*L. braziliensis*	54	1202	1255
LbrM04_V2.0620	*L. braziliensis*	168	1327	1494
LbrM04_V2.0620	*L. braziliensis*	96	1505	1600
LbrM04_V2.0640	*L. braziliensis*	56	116	171
LbrM04_V2.0670	*L. braziliensis*	77	40	116
LbrM04_V2.0670	*L. braziliensis*	79	256	334
LbrM04_V2.0670	*L. braziliensis*	92	468	559
LbrM04_V2.0680	*L. braziliensis*	107	1	107
LbrM04_V2.0680	*L. braziliensis*	80	243	322
LbrM04_V2.0680	*L. braziliensis*	46	489	534
LbrM04_V2.0700	*L. braziliensis*	131	62	192
LbrM04_V2.0710	*L. braziliensis*	91	1	91
LbrM04_V2.0710	*L. braziliensis*	145	148	292
LbrM04_V2.0710	*L. braziliensis*	207	303	509
LbrM04_V2.0730	*L. braziliensis*	130	85	214
LbrM04_V2.0730	*L. braziliensis*	91	249	339
LbrM04_V2.0730	*L. braziliensis*	91	249	339
LbrM04_V2.0750	*L. braziliensis*	40	1	40
LbrM04_V2.0750	*L. braziliensis*	70	90	159
LbrM04_V2.0750	*L. braziliensis*	70	90	159
LbrM04_V2.0760	*L. braziliensis*	58	221	278
LbrM04_V2.0760	*L. braziliensis*	101	494	594
LbrM04_V2.0770	*L. braziliensis*	461	393	853
LbrM04_V2.0770	*L. braziliensis*	239	859	1097
LbrM04_V2.0770	*L. braziliensis*	41	1099	1139
LbrM04_V2.0780	*L. braziliensis*	57	257	313
LbrM04_V2.0780	*L. braziliensis*	216	455	670
LbrM04_V2.0790	*L. braziliensis*	588	1	588
LbrM04_V2.0790	*L. braziliensis*	251	644	894
LbrM04_V2.0790	*L. braziliensis*	266	911	1176
LbrM04_V2.0800	*L. braziliensis*	124	1	124
LbrM04_V2.0800	*L. braziliensis*	245	157	401
LbrM04_V2.0800	*L. braziliensis*	80	596	675
LbrM04_V2.0800	*L. braziliensis*	158	693	850
LbrM04_V2.0810	*L. braziliensis*	75	1	75
LbrM04_V2.0820	*L. braziliensis*	67	97	163
LbrM04_V2.0820	*L. braziliensis*	57	263	319
LbrM04_V2.0820	*L. braziliensis*	57	263	319
LbrM04_V2.0830	*L. braziliensis*	319	1	319
LbrM04_V2.0840	*L. braziliensis*	164	48	211
LbrM04_V2.0840	*L. braziliensis*	40	215	254
LbrM04_V2.0850	*L. braziliensis*	259	1	259
LbrM04_V2.0850	*L. braziliensis*	899	264	1162
LbrM04_V2.0850	*L. braziliensis*	45	1167	1211
LbrM04_V2.0860	*L. braziliensis*	48	1	48
LbrM04_V2.0860	*L. braziliensis*	110	53	162
LbrM04_V2.0860	*L. braziliensis*	79	202	280

LbrM04_V2.0860	*L. braziliensis*	45	347	391
LbrM04_V2.0860	*L. braziliensis*	51	537	587
LbrM04_V2.0860	*L. braziliensis*	96	600	695
LbrM04_V2.0860	*L. braziliensis*	134	1003	1136
LbrM04_V2.0870	*L. braziliensis*	67	1	67
LbrM04_V2.0890	*L. braziliensis*	138	97	234
LbrM04_V2.0890	*L. braziliensis*	95	483	577
LbrM04_V2.0890	*L. braziliensis*	46	691	736
LbrM04_V2.0900	*L. braziliensis*	220	1	220
LbrM04_V2.0900	*L. braziliensis*	75	261	335
LbrM04_V2.0900	*L. braziliensis*	184	459	642
LbrM04_V2.0900	*L. braziliensis*	48	688	735
LbrM04_V2.0900	*L. braziliensis*	50	738	787
LbrM04_V2.0900	*L. braziliensis*	63	888	950
LbrM04_V2.0900	*L. braziliensis*	52	998	1049
LbrM04_V2.0910	*L. braziliensis*	252	71	322
LbrM04_V2.0910	*L. braziliensis*	105	354	458
LbrM04_V2.0910	*L. braziliensis*	99	585	683
LbrM04_V2.0920	*L. braziliensis*	434	1	434
LbrM04_V2.0920	*L. braziliensis*	176	445	620
LbrM04_V2.0930	*L. braziliensis*	49	1	49
LbrM04_V2.0930	*L. braziliensis*	64	66	129
LbrM04_V2.0930	*L. braziliensis*	47	433	479
LbrM04_V2.0940	*L. braziliensis*	51	1	51
LbrM04_V2.0940	*L. braziliensis*	240	123	362
LbrM04_V2.0940	*L. braziliensis*	368	466	833
LbrM04_V2.0970	*L. braziliensis*	250	41	290
LbrM04_V2.0980	*L. braziliensis*	66	34	99
LbrM04_V2.0980	*L. braziliensis*	57	155	211
LbrM04_V2.0980	*L. braziliensis*	47	230	276
LbrM04_V2.0980	*L. braziliensis*	118	281	398
LbrM04_V2.0990	*L. braziliensis*	250	1	250
LbrM04_V2.0990	*L. braziliensis*	50	292	341
LbrM04_V2.0990	*L. braziliensis*	52	439	490
LbrM04_V2.0990	*L. braziliensis*	113	740	852
LbrM04_V2.0990	*L. braziliensis*	62	998	1059
LbrM04_V2.0990	*L. braziliensis*	50	1134	1183
LbrM04_V2.1000	*L. braziliensis*	169	67	235
LbrM04_V2.1000	*L. braziliensis*	59	238	296
LbrM04_V2.1010	*L. braziliensis*	49	713	761
LbrM04_V2.1010	*L. braziliensis*	232	954	1185
LbrM04_V2.1020	*L. braziliensis*	223	1	223
LbrM04_V2.1030	*L. braziliensis*	43	145	187
LbrM04_V2.1030	*L. braziliensis*	77	205	281
LbrM04_V2.1050	*L. braziliensis*	68	270	337
LbrM04_V2.1060	*L. braziliensis*	68	288	355
LbrM04_V2.1070	*L. braziliensis*	291	179	469

LbrM04_V2.1090	*L. braziliensis*	149	1	149
LbrM04_V2.1090	*L. braziliensis*	41	477	517
LbrM04_V2.1090	*L. braziliensis*	42	617	658
LbrM04_V2.1090	*L. braziliensis*	82	713	794
LbrM04_V2.1100	*L. braziliensis*	601	1	601
LbrM04_V2.1100	*L. braziliensis*	435	608	1042
LbrM04_V2.1100	*L. braziliensis*	211	1085	1295
LbrM04_V2.1130	*L. braziliensis*	157	40	196
LbrM04_V2.1130	*L. braziliensis*	73	215	287
LbrM04_V2.1130	*L. braziliensis*	337	331	667
LbrM04_V2.1140	*L. braziliensis*	77	1	77
LbrM04_V2.1150	*L. braziliensis*	303	1	303
LbrM04_V2.1160	*L. braziliensis*	47	122	168
LbrM04_V2.1190	*L. braziliensis*	94	1	94
LbrM04_V2.1190	*L. braziliensis*	46	270	315
LbrM04_V2.1200	*L. braziliensis*	121	1241	1361
LbrM04_V2.1200	*L. braziliensis*	376	1369	1744
LbrM04_V2.1200	*L. braziliensis*	51	1759	1809
LbrM04_V2.1200	*L. braziliensis*	120	1845	1964
LbrM04_V2.1200	*L. braziliensis*	251	1973	2223
LbrM04_V2.1200	*L. braziliensis*	83	2250	2332
LbrM04_V2.1230	*L. braziliensis*	43	326	368
LbrM04_V2.1240	*L. braziliensis*	49	1	49
LbrM04_V2.1240	*L. braziliensis*	372	112	483
LbrM04_V2.1240	*L. braziliensis*	52	982	1033
LbrM04_V2.1260	*L. braziliensis*	67	76	142
LbrM04_V2.1260	*L. braziliensis*	107	193	299
LbrM04_V2.1260	*L. braziliensis*	107	193	299
LbrM04_V2.1270	*L. braziliensis*	141	41	181
LbrM04_V2.1280	*L. braziliensis*	128	212	339
LbrM04_V2.1290	*L. braziliensis*	46	1	46
LbrM04_V2.1290	*L. braziliensis*	1148	124	1271
LbrM04_V2.1300	*L. braziliensis*	164	1	164
LbrM04_V2.1320	*L. braziliensis*	158	103	260
LbrM04_V2.1330	*L. braziliensis*	69	41	109
LbrM04_V2.1340	*L. braziliensis*	158	40	197
LbrM05_V2.0010	*L. braziliensis*	122	1	122
LbrM05_V2.0010	*L. braziliensis*	41	145	185
LbrM05_V2.0010	*L. braziliensis*	198	226	423
LbrM05_V2.0010	*L. braziliensis*	67	431	497
LbrM05_V2.0010	*L. braziliensis*	113	511	623
LbrM05_V2.0010	*L. braziliensis*	161	680	840
LbrM05_V2.0040	*L. braziliensis*	98	1	98
LbrM05_V2.0040	*L. braziliensis*	261	106	366
LbrM05_V2.0040	*L. braziliensis*	92	382	473
LbrM05_V2.0040	*L. braziliensis*	45	485	529
LbrM05_V2.0050	*L. braziliensis*	113	45	157

LbrM05_V2.0050	*L. braziliensis*	78	194	271
LbrM05_V2.0050	*L. braziliensis*	65	368	432
LbrM05_V2.0050	*L. braziliensis*	44	623	666
LbrM05_V2.0050	*L. braziliensis*	51	770	820
LbrM05_V2.0060	*L. braziliensis*	155	621	775
LbrM05_V2.0080	*L. braziliensis*	112	1	112
LbrM05_V2.0080	*L. braziliensis*	44	159	202
LbrM05_V2.0080	*L. braziliensis*	44	159	202
LbrM05_V2.0100	*L. braziliensis*	44	146	189
LbrM05_V2.0100	*L. braziliensis*	53	226	278
LbrM05_V2.0100	*L. braziliensis*	53	226	278
LbrM05_V2.0130	*L. braziliensis*	50	177	226
LbrM05_V2.0130	*L. braziliensis*	99	334	432
LbrM05_V2.0130	*L. braziliensis*	99	334	432
LbrM05_V2.0140	*L. braziliensis*	85	1	85
LbrM05_V2.0140	*L. braziliensis*	77	613	689
LbrM05_V2.0150	*L. braziliensis*	50	393	442
LbrM05_V2.0160	*L. braziliensis*	194	1	194
LbrM05_V2.0160	*L. braziliensis*	62	197	258
LbrM05_V2.0160	*L. braziliensis*	254	293	546
LbrM05_V2.0170	*L. braziliensis*	54	1	54
LbrM05_V2.0170	*L. braziliensis*	672	77	748
LbrM05_V2.0180	*L. braziliensis*	62	115	176
LbrM05_V2.0190	*L. braziliensis*	44	1	44
LbrM05_V2.0190	*L. braziliensis*	233	117	349
LbrM05_V2.0200	*L. braziliensis*	48	1	48
LbrM05_V2.0200	*L. braziliensis*	127	254	380
LbrM05_V2.0200	*L. braziliensis*	82	591	672
LbrM05_V2.0200	*L. braziliensis*	114	675	788
LbrM05_V2.0210	*L. braziliensis*	88	28	115
LbrM05_V2.0210	*L. braziliensis*	84	172	255
LbrM05_V2.0210	*L. braziliensis*	42	996	1037
LbrM05_V2.0210	*L. braziliensis*	56	1165	1220
LbrM05_V2.0210	*L. braziliensis*	183	1290	1472
LbrM05_V2.0220	*L. braziliensis*	47	122	168
LbrM05_V2.0220	*L. braziliensis*	85	330	414
LbrM05_V2.0220	*L. braziliensis*	61	504	564
LbrM05_V2.0240	*L. braziliensis*	45	85	129
LbrM05_V2.0250	*L. braziliensis*	52	28	79
LbrM05_V2.0250	*L. braziliensis*	81	237	317
LbrM05_V2.0260	*L. braziliensis*	49	1	49
LbrM05_V2.0260	*L. braziliensis*	111	212	322
LbrM05_V2.0260	*L. braziliensis*	111	212	322
LbrM05_V2.0290	*L. braziliensis*	101	1	101
LbrM05_V2.0300	*L. braziliensis*	103	1	103
LbrM05_V2.0300	*L. braziliensis*	63	317	379
LbrM05_V2.0300	*L. braziliensis*	54	544	597

LbrM05_V2.0310	*L. braziliensis*	60	1	60
LbrM05_V2.0320	*L. braziliensis*	95	378	472
LbrM05_V2.0320	*L. braziliensis*	40	811	850
LbrM05_V2.0320	*L. braziliensis*	40	811	850
LbrM05_V2.0330	*L. braziliensis*	107	1	107
LbrM05_V2.0330	*L. braziliensis*	49	138	186
LbrM05_V2.0340	*L. braziliensis*	130	1	130
LbrM05_V2.0340	*L. braziliensis*	103	138	240
LbrM05_V2.0340	*L. braziliensis*	476	398	873
LbrM05_V2.0360	*L. braziliensis*	66	179	244
LbrM05_V2.0360	*L. braziliensis*	45	261	305
LbrM05_V2.0360	*L. braziliensis*	152	478	629
LbrM05_V2.0370	*L. braziliensis*	56	73	128
LbrM05_V2.0380	*L. braziliensis*	624	1	624
LbrM05_V2.0380	*L. braziliensis*	216	640	855
LbrM05_V2.0390	*L. braziliensis*	1903	1	1903
LbrM05_V2.0400	*L. braziliensis*	100	1	100
LbrM05_V2.0400	*L. braziliensis*	77	320	396
LbrM05_V2.0410	*L. braziliensis*	318	177	494
LbrM05_V2.0410	*L. braziliensis*	248	693	940
LbrM05_V2.0410	*L. braziliensis*	47	970	1016
LbrM05_V2.0420	*L. braziliensis*	50	109	158
LbrM05_V2.0420	*L. braziliensis*	42	221	262
LbrM05_V2.0420	*L. braziliensis*	95	331	425
LbrM05_V2.0420	*L. braziliensis*	57	459	515
LbrM05_V2.0440	*L. braziliensis*	53	42	94
LbrM05_V2.0450	*L. braziliensis*	78	82	159
LbrM05_V2.0450	*L. braziliensis*	44	437	480
LbrM05_V2.0450	*L. braziliensis*	84	716	799
LbrM05_V2.0470	*L. braziliensis*	58	1	58
LbrM05_V2.0470	*L. braziliensis*	54	141	194
LbrM05_V2.0470	*L. braziliensis*	165	476	640
LbrM05_V2.0480	*L. braziliensis*	154	44	197
LbrM05_V2.0490	*L. braziliensis*	93	211	303
LbrM05_V2.0540	*L. braziliensis*	207	1	207
LbrM05_V2.0540	*L. braziliensis*	57	301	357
LbrM05_V2.0540	*L. braziliensis*	259	372	630
LbrM05_V2.0540	*L. braziliensis*	77	657	733
LbrM05_V2.0540	*L. braziliensis*	44	770	813
LbrM05_V2.0540	*L. braziliensis*	62	854	915
LbrM05_V2.0540	*L. braziliensis*	133	944	1076
LbrM05_V2.0540	*L. braziliensis*	134	1080	1213
LbrM05_V2.0540	*L. braziliensis*	203	1226	1428
LbrM05_V2.0540	*L. braziliensis*	125	1451	1575
LbrM05_V2.0540	*L. braziliensis*	169	1649	1817
LbrM05_V2.0540	*L. braziliensis*	68	1945	2012
LbrM05_V2.0550	*L. braziliensis*	85	1	85

LbrM05_V2.0550	*L. braziliensis*	45	134	178
LbrM05_V2.0550	*L. braziliensis*	45	134	178
LbrM05_V2.0570	*L. braziliensis*	52	66	117
LbrM05_V2.0570	*L. braziliensis*	126	205	330
LbrM05_V2.0590	*L. braziliensis*	42	1	42
LbrM05_V2.0590	*L. braziliensis*	243	79	321
LbrM05_V2.0590	*L. braziliensis*	155	341	495
LbrM05_V2.0590	*L. braziliensis*	231	534	764
LbrM05_V2.0600	*L. braziliensis*	96	1	96
LbrM05_V2.0600	*L. braziliensis*	54	149	202
LbrM05_V2.0600	*L. braziliensis*	54	149	202
LbrM05_V2.0610	*L. braziliensis*	55	39	93
LbrM05_V2.0620	*L. braziliensis*	90	1	90
LbrM05_V2.0630	*L. braziliensis*	56	52	107
LbrM05_V2.0630	*L. braziliensis*	60	193	252
LbrM05_V2.0640	*L. braziliensis*	114	1	114
LbrM05_V2.0640	*L. braziliensis*	71	312	382
LbrM05_V2.0640	*L. braziliensis*	242	537	778
LbrM05_V2.0650	*L. braziliensis*	47	1	47
LbrM05_V2.0660	*L. braziliensis*	576	1	576
LbrM05_V2.0660	*L. braziliensis*	767	583	1349
LbrM05_V2.0660	*L. braziliensis*	64	1385	1448
LbrM05_V2.0660	*L. braziliensis*	79	1454	1532
LbrM05_V2.0660	*L. braziliensis*	661	1556	2216
LbrM05_V2.0660	*L. braziliensis*	188	2241	2428
LbrM05_V2.0670	*L. braziliensis*	48	1	48
LbrM05_V2.0680	*L. braziliensis*	42	47	88
LbrM05_V2.0680	*L. braziliensis*	62	181	242
LbrM05_V2.0680	*L. braziliensis*	217	254	470
LbrM05_V2.0680	*L. braziliensis*	359	516	874
LbrM05_V2.0680	*L. braziliensis*	41	942	982
LbrM05_V2.0680	*L. braziliensis*	45	1079	1123
LbrM05_V2.0680	*L. braziliensis*	110	1153	1262
LbrM05_V2.0680	*L. braziliensis*	71	1336	1406
LbrM05_V2.0680	*L. braziliensis*	345	1474	1818
LbrM05_V2.0680	*L. braziliensis*	100	1837	1936
LbrM05_V2.0680	*L. braziliensis*	56	1968	2023
LbrM05_V2.0680	*L. braziliensis*	66	2029	2094
LbrM05_V2.0680	*L. braziliensis*	264	2122	2385
LbrM05_V2.0680	*L. braziliensis*	139	2401	2539
LbrM05_V2.0680	*L. braziliensis*	86	2562	2647
LbrM05_V2.0680	*L. braziliensis*	215	2658	2872
LbrM05_V2.0690	*L. braziliensis*	1252	1	1252
LbrM05_V2.0690	*L. braziliensis*	165	1268	1432
LbrM05_V2.0690	*L. braziliensis*	42	1443	1484
LbrM05_V2.0690	*L. braziliensis*	105	1623	1727
LbrM05_V2.0700	*L. braziliensis*	144	1	144

LbrM05_V2.0700	*L. braziliensis*	101	154	254
LbrM05_V2.0700	*L. braziliensis*	62	268	329
LbrM05_V2.0700	*L. braziliensis*	254	337	590
LbrM05_V2.0700	*L. braziliensis*	73	716	788
LbrM05_V2.0700	*L. braziliensis*	389	842	1230
LbrM05_V2.0710	*L. braziliensis*	536	81	616
LbrM05_V2.0710	*L. braziliensis*	54	628	681
LbrM05_V2.0720	*L. braziliensis*	179	154	332
LbrM05_V2.0730	*L. braziliensis*	57	48	104
LbrM05_V2.0730	*L. braziliensis*	138	294	431
LbrM05_V2.0740	*L. braziliensis*	147	32	178
LbrM05_V2.0740	*L. braziliensis*	144	198	341
LbrM05_V2.0740	*L. braziliensis*	111	346	456
LbrM05_V2.0740	*L. braziliensis*	61	459	519
LbrM05_V2.0740	*L. braziliensis*	99	527	625
LbrM05_V2.0740	*L. braziliensis*	254	794	1047
LbrM05_V2.0740	*L. braziliensis*	45	1211	1255
LbrM05_V2.0740	*L. braziliensis*	128	1295	1422
LbrM05_V2.0740	*L. braziliensis*	133	1487	1619
LbrM05_V2.0750	*L. braziliensis*	207	102	308
LbrM05_V2.0750	*L. braziliensis*	633	310	942
LbrM05_V2.0750	*L. braziliensis*	43	946	988
LbrM05_V2.0750	*L. braziliensis*	448	1012	1459
LbrM05_V2.0770	*L. braziliensis*	61	1	61
LbrM05_V2.0770	*L. braziliensis*	113	63	175
LbrM05_V2.0770	*L. braziliensis*	44	187	230
LbrM05_V2.0770	*L. braziliensis*	45	251	295
LbrM05_V2.0780	*L. braziliensis*	74	174	247
LbrM05_V2.0790	*L. braziliensis*	53	1	53
LbrM05_V2.0790	*L. braziliensis*	49	197	245
LbrM05_V2.0800	*L. braziliensis*	48	259	306
LbrM05_V2.0810	*L. braziliensis*	715	49	763
LbrM05_V2.0810	*L. braziliensis*	701	796	1496
LbrM05_V2.0820	*L. braziliensis*	124	1	124
LbrM05_V2.0820	*L. braziliensis*	340	161	500
LbrM05_V2.0820	*L. braziliensis*	148	520	667
LbrM05_V2.0830	*L. braziliensis*	94	62	155
LbrM05_V2.0840	*L. braziliensis*	65	1	65
LbrM05_V2.0840	*L. braziliensis*	134	172	305
LbrM05_V2.0840	*L. braziliensis*	73	386	458
LbrM05_V2.0840	*L. braziliensis*	66	550	615
LbrM05_V2.0840	*L. braziliensis*	1033	631	1663
LbrM05_V2.0840	*L. braziliensis*	114	1666	1779
LbrM05_V2.0840	*L. braziliensis*	45	1781	1825
LbrM05_V2.0850	*L. braziliensis*	55	1	55
LbrM05_V2.0850	*L. braziliensis*	50	155	204
LbrM05_V2.0850	*L. braziliensis*	50	155	204

LbrM05_V2.0860	*L. braziliensis*	67	245	311
LbrM05_V2.0860	*L. braziliensis*	193	614	806
LbrM05_V2.0860	*L. braziliensis*	64	845	908
LbrM05_V2.0860	*L. braziliensis*	51	1057	1107
LbrM05_V2.0860	*L. braziliensis*	51	1269	1319
LbrM05_V2.0860	*L. braziliensis*	64	1530	1593
LbrM05_V2.0880	*L. braziliensis*	60	192	251
LbrM05_V2.0880	*L. braziliensis*	65	286	350
LbrM05_V2.0880	*L. braziliensis*	74	407	480
LbrM05_V2.0880	*L. braziliensis*	57	664	720
LbrM05_V2.0890	*L. braziliensis*	459	1	459
LbrM05_V2.0890	*L. braziliensis*	166	480	645
LbrM05_V2.0890	*L. braziliensis*	57	810	866
LbrM05_V2.0890	*L. braziliensis*	75	913	987
LbrM05_V2.0890	*L. braziliensis*	348	989	1336
LbrM05_V2.0890	*L. braziliensis*	64	1352	1415
LbrM05_V2.0900	*L. braziliensis*	121	1	121
LbrM05_V2.0900	*L. braziliensis*	82	145	226
LbrM05_V2.0900	*L. braziliensis*	67	381	447
LbrM05_V2.0900	*L. braziliensis*	50	476	525
LbrM05_V2.0900	*L. braziliensis*	185	706	890
LbrM05_V2.0900	*L. braziliensis*	46	898	943
LbrM05_V2.0910	*L. braziliensis*	240	167	406
LbrM05_V2.0920	*L. braziliensis*	522	69	590
LbrM05_V2.0920	*L. braziliensis*	187	630	816
LbrM05_V2.0920	*L. braziliensis*	252	847	1098
LbrM05_V2.0920	*L. braziliensis*	67	1132	1198
LbrM05_V2.0930	*L. braziliensis*	45	77	121
LbrM05_V2.0930	*L. braziliensis*	44	239	282
LbrM05_V2.0930	*L. braziliensis*	51	325	375
LbrM05_V2.0930	*L. braziliensis*	40	399	438
LbrM05_V2.1000	*L. braziliensis*	57	1	57
LbrM05_V2.1010	*L. braziliensis*	138	484	621
LbrM05_V2.1020	*L. braziliensis*	69	110	178
LbrM05_V2.1020	*L. braziliensis*	44	263	306
LbrM05_V2.1020	*L. braziliensis*	55	377	431
LbrM05_V2.1020	*L. braziliensis*	71	501	571
LbrM05_V2.1020	*L. braziliensis*	48	744	791
LbrM05_V2.1030	*L. braziliensis*	47	28	74
LbrM05_V2.1040	*L. braziliensis*	58	1	58
LbrM05_V2.1050	*L. braziliensis*	69	72	140
LbrM05_V2.1050	*L. braziliensis*	43	210	252
LbrM05_V2.1050	*L. braziliensis*	43	210	252
LbrM05_V2.1060	*L. braziliensis*	40	145	184
LbrM05_V2.1060	*L. braziliensis*	68	411	478
LbrM05_V2.1060	*L. braziliensis*	68	411	478
LbrM05_V2.1070	*L. braziliensis*	80	166	245

LbrM05_V2.1080	*L. braziliensis*	42	20	61
LbrM05_V2.1080	*L. braziliensis*	61	141	201
LbrM05_V2.1080	*L. braziliensis*	48	535	582
LbrM05_V2.1080	*L. braziliensis*	99	627	725
LbrM05_V2.1090	*L. braziliensis*	115	197	311
LbrM05_V2.1090	*L. braziliensis*	45	320	364
LbrM05_V2.1090	*L. braziliensis*	115	398	512
LbrM05_V2.1110	*L. braziliensis*	45	1	45
LbrM05_V2.1120	*L. braziliensis*	132	1	132
LbrM05_V2.1120	*L. braziliensis*	70	238	307
LbrM05_V2.1120	*L. braziliensis*	74	431	504
LbrM05_V2.1120	*L. braziliensis*	213	703	915
LbrM05_V2.1120	*L. braziliensis*	87	1059	1145
LbrM05_V2.1130	*L. braziliensis*	91	1	91
LbrM05_V2.1150	*L. braziliensis*	49	44	92
LbrM05_V2.1150	*L. braziliensis*	41	162	202
LbrM05_V2.1150	*L. braziliensis*	71	218	288
LbrM05_V2.1150	*L. braziliensis*	65	385	449
LbrM05_V2.1150	*L. braziliensis*	69	479	547
LbrM05_V2.1150	*L. braziliensis*	179	564	742
LbrM05_V2.1150	*L. braziliensis*	96	775	870
LbrM05_V2.1170	*L. braziliensis*	96	1	96
LbrM05_V2.1170	*L. braziliensis*	45	100	144
LbrM05_V2.1170	*L. braziliensis*	51	311	361
LbrM05_V2.1170	*L. braziliensis*	133	390	522
LbrM05_V2.1180	*L. braziliensis*	65	1	65
LbrM05_V2.1180	*L. braziliensis*	48	115	162
LbrM05_V2.1180	*L. braziliensis*	95	182	276
LbrM05_V2.1180	*L. braziliensis*	62	343	404
LbrM05_V2.1180	*L. braziliensis*	58	455	512
LbrM05_V2.1180	*L. braziliensis*	114	547	660
LbrM05_V2.1180	*L. braziliensis*	59	849	907
LbrM05_V2.1180	*L. braziliensis*	59	979	1037
LbrM05_V2.1180	*L. braziliensis*	78	1230	1307
LbrM05_V2.1180	*L. braziliensis*	229	1325	1553
LbrM05_V2.1180	*L. braziliensis*	337	1645	1981
LbrM05_V2.1190	*L. braziliensis*	57	1	57
LbrM05_V2.1190	*L. braziliensis*	48	90	137
LbrM05_V2.1190	*L. braziliensis*	48	90	137
LbrM05_V2.1200	*L. braziliensis*	73	14	86
LbrM05_V2.1210	*L. braziliensis*	48	215	262
LbrM05_V2.1230	*L. braziliensis*	403	1	403
LbrM05_V2.1240	*L. braziliensis*	172	97	268
LbrM05_V2.1240	*L. braziliensis*	115	448	562
LbrM05_V2.1240	*L. braziliensis*	86	577	662
LbrM05_V2.1240	*L. braziliensis*	169	676	844
LbrM05_V2.1240	*L. braziliensis*	58	1349	1406

LbrM06_V2.0010	*L. braziliensis*	75	1	75
LbrM06_V2.0030	*L. braziliensis*	79	48	126
LbrM06_V2.0030	*L. braziliensis*	46	165	210
LbrM06_V2.0050	*L. braziliensis*	155	1	155
LbrM06_V2.0060	*L. braziliensis*	106	42	147
LbrM06_V2.0060	*L. braziliensis*	120	215	334
LbrM06_V2.0060	*L. braziliensis*	61	367	427
LbrM06_V2.0080	*L. braziliensis*	80	1	80
LbrM06_V2.0090	*L. braziliensis*	148	105	252
LbrM06_V2.0090	*L. braziliensis*	45	254	298
LbrM06_V2.0090	*L. braziliensis*	98	344	441
LbrM06_V2.0110	*L. braziliensis*	46	145	190
LbrM06_V2.0140	*L. braziliensis*	48	368	415
LbrM06_V2.0150	*L. braziliensis*	41	54	94
LbrM06_V2.0160	*L. braziliensis*	113	1	113
LbrM06_V2.0160	*L. braziliensis*	68	373	440
LbrM06_V2.0160	*L. braziliensis*	118	528	645
LbrM06_V2.0170	*L. braziliensis*	73	57	129
LbrM06_V2.0170	*L. braziliensis*	59	232	290
LbrM06_V2.0170	*L. braziliensis*	65	330	394
LbrM06_V2.0170	*L. braziliensis*	151	451	601
LbrM06_V2.0180	*L. braziliensis*	73	1	73
LbrM06_V2.0180	*L. braziliensis*	253	79	331
LbrM06_V2.0190	*L. braziliensis*	60	31	90
LbrM06_V2.0200	*L. braziliensis*	74	212	285
LbrM06_V2.0220	*L. braziliensis*	92	48	139
LbrM06_V2.0220	*L. braziliensis*	43	359	401
LbrM06_V2.0220	*L. braziliensis*	50	696	745
LbrM06_V2.0220	*L. braziliensis*	42	795	836
LbrM06_V2.0220	*L. braziliensis*	87	855	941
LbrM06_V2.0230	*L. braziliensis*	144	113	256
LbrM06_V2.0240	*L. braziliensis*	69	1061	1129
LbrM06_V2.0240	*L. braziliensis*	284	1179	1462
LbrM06_V2.0250	*L. braziliensis*	67	102	168
LbrM06_V2.0260	*L. braziliensis*	68	75	142
LbrM06_V2.0260	*L. braziliensis*	47	347	393
LbrM06_V2.0260	*L. braziliensis*	94	529	622
LbrM06_V2.0260	*L. braziliensis*	83	745	827
LbrM06_V2.0300	*L. braziliensis*	417	1	417
LbrM06_V2.0300	*L. braziliensis*	85	431	515
LbrM06_V2.0300	*L. braziliensis*	43	600	642
LbrM06_V2.0300	*L. braziliensis*	198	672	869
LbrM06_V2.0310	*L. braziliensis*	144	311	454
LbrM06_V2.0310	*L. braziliensis*	250	793	1042
LbrM06_V2.0310	*L. braziliensis*	44	1165	1208
LbrM06_V2.0320	*L. braziliensis*	47	35	81
LbrM06_V2.0320	*L. braziliensis*	50	856	905

LbrM06_V2.0360	*L. braziliensis*	45	1	45
LbrM06_V2.0360	*L. braziliensis*	47	299	345
LbrM06_V2.0360	*L. braziliensis*	47	299	345
LbrM06_V2.0370	*L. braziliensis*	61	371	431
LbrM06_V2.0370	*L. braziliensis*	47	477	523
LbrM06_V2.0370	*L. braziliensis*	47	477	523
LbrM06_V2.0390	*L. braziliensis*	64	52	115
LbrM06_V2.0390	*L. braziliensis*	119	132	250
LbrM06_V2.0400	*L. braziliensis*	145	35	179
LbrM06_V2.0410	*L. braziliensis*	64	52	115
LbrM06_V2.0410	*L. braziliensis*	140	132	271
LbrM06_V2.0420	*L. braziliensis*	140	37	176
LbrM06_V2.0430	*L. braziliensis*	71	1	71
LbrM06_V2.0430	*L. braziliensis*	424	143	566
LbrM06_V2.0440	*L. braziliensis*	112	1	112
LbrM06_V2.0440	*L. braziliensis*	53	163	215
LbrM06_V2.0440	*L. braziliensis*	222	463	684
LbrM06_V2.0450	*L. braziliensis*	82	34	115
LbrM06_V2.0450	*L. braziliensis*	413	416	828
LbrM06_V2.0450	*L. braziliensis*	91	868	958
LbrM06_V2.0460	*L. braziliensis*	121	1	121
LbrM06_V2.0460	*L. braziliensis*	49	182	230
LbrM06_V2.0460	*L. braziliensis*	82	275	356
LbrM06_V2.0460	*L. braziliensis*	82	395	476
LbrM06_V2.0470	*L. braziliensis*	92	32	123
LbrM06_V2.0470	*L. braziliensis*	57	130	186
LbrM06_V2.0470	*L. braziliensis*	175	301	475
LbrM06_V2.0470	*L. braziliensis*	345	499	843
LbrM06_V2.0470	*L. braziliensis*	70	913	982
LbrM06_V2.0480	*L. braziliensis*	47	1	47
LbrM06_V2.0480	*L. braziliensis*	87	169	255
LbrM06_V2.0480	*L. braziliensis*	253	300	552
LbrM06_V2.0480	*L. braziliensis*	102	596	697
LbrM06_V2.0480	*L. braziliensis*	58	785	842
LbrM06_V2.0480	*L. braziliensis*	95	855	949
LbrM06_V2.0480	*L. braziliensis*	96	994	1089
LbrM06_V2.0500	*L. braziliensis*	147	1	147
LbrM06_V2.0510	*L. braziliensis*	53	53	105
LbrM06_V2.0510	*L. braziliensis*	60	120	179
LbrM06_V2.0510	*L. braziliensis*	70	344	413
LbrM06_V2.0510	*L. braziliensis*	67	445	511
LbrM06_V2.0510	*L. braziliensis*	43	543	585
LbrM06_V2.0520	*L. braziliensis*	55	168	222
LbrM06_V2.0520	*L. braziliensis*	78	399	476
LbrM06_V2.0520	*L. braziliensis*	66	611	676
LbrM06_V2.0530	*L. braziliensis*	147	63	209
LbrM06_V2.0530	*L. braziliensis*	148	218	365

LbrM06_V2.0530	*L. braziliensis*	42	386	427
LbrM06_V2.0530	*L. braziliensis*	95	462	556
LbrM06_V2.0530	*L. braziliensis*	161	702	862
LbrM06_V2.0530	*L. braziliensis*	253	892	1144
LbrM06_V2.0540	*L. braziliensis*	52	97	148
LbrM06_V2.0550	*L. braziliensis*	64	14	77
LbrM06_V2.0550	*L. braziliensis*	111	155	265
LbrM06_V2.0550	*L. braziliensis*	85	500	584
LbrM06_V2.0550	*L. braziliensis*	71	606	676
LbrM06_V2.0550	*L. braziliensis*	101	694	794
LbrM06_V2.0560	*L. braziliensis*	126	1	126
LbrM06_V2.0560	*L. braziliensis*	71	219	289
LbrM06_V2.0560	*L. braziliensis*	64	295	358
LbrM06_V2.0560	*L. braziliensis*	55	371	425
LbrM06_V2.0560	*L. braziliensis*	58	573	630
LbrM06_V2.0560	*L. braziliensis*	61	753	813
LbrM06_V2.0560	*L. braziliensis*	87	841	927
LbrM06_V2.0560	*L. braziliensis*	41	1144	1184
LbrM06_V2.0560	*L. braziliensis*	52	1188	1239
LbrM06_V2.0560	*L. braziliensis*	46	1547	1592
LbrM06_V2.0560	*L. braziliensis*	52	1651	1702
LbrM06_V2.0580	*L. braziliensis*	69	1	69
LbrM06_V2.0590	*L. braziliensis*	70	1	70
LbrM06_V2.0600	*L. braziliensis*	73	82	154
LbrM06_V2.0600	*L. braziliensis*	58	246	303
LbrM06_V2.0600	*L. braziliensis*	47	444	490
LbrM06_V2.0600	*L. braziliensis*	67	551	617
LbrM06_V2.0600	*L. braziliensis*	229	634	862
LbrM06_V2.0600	*L. braziliensis*	349	881	1229
LbrM06_V2.0600	*L. braziliensis*	252	1245	1496
LbrM06_V2.0610	*L. braziliensis*	59	165	223
LbrM06_V2.0610	*L. braziliensis*	260	260	519
LbrM06_V2.0610	*L. braziliensis*	73	593	665
LbrM06_V2.0610	*L. braziliensis*	63	855	917
LbrM06_V2.0610	*L. braziliensis*	156	946	1101
LbrM06_V2.0610	*L. braziliensis*	220	1163	1382
LbrM06_V2.0620	*L. braziliensis*	153	1	153
LbrM06_V2.0640	*L. braziliensis*	186	1	186
LbrM06_V2.0640	*L. braziliensis*	98	476	573
LbrM06_V2.0640	*L. braziliensis*	146	651	796
LbrM06_V2.0640	*L. braziliensis*	103	883	985
LbrM06_V2.0640	*L. braziliensis*	121	1092	1212
LbrM06_V2.0640	*L. braziliensis*	141	1250	1390
LbrM06_V2.0640	*L. braziliensis*	147	1420	1566
LbrM06_V2.0640	*L. braziliensis*	200	1587	1786
LbrM06_V2.0640	*L. braziliensis*	372	1810	2181
LbrM06_V2.0640	*L. braziliensis*	156	2186	2341

LbrM06_V2.0640	*L. braziliensis*	47	2375	2421
LbrM06_V2.0640	*L. braziliensis*	110	2484	2593
LbrM06_V2.0640	*L. braziliensis*	41	2755	2795
LbrM06_V2.0640	*L. braziliensis*	126	2878	3003
LbrM06_V2.0650	*L. braziliensis*	45	1	45
LbrM06_V2.0650	*L. braziliensis*	50	339	388
LbrM06_V2.0650	*L. braziliensis*	50	339	388
LbrM06_V2.0660	*L. braziliensis*	365	1	365
LbrM06_V2.0660	*L. braziliensis*	167	370	536
LbrM06_V2.0660	*L. braziliensis*	69	539	607
LbrM06_V2.0670	*L. braziliensis*	528	1	528
LbrM06_V2.0670	*L. braziliensis*	508	538	1045
LbrM06_V2.0670	*L. braziliensis*	78	1047	1124
LbrM06_V2.0670	*L. braziliensis*	131	1142	1272
LbrM06_V2.0680	*L. braziliensis*	44	56	99
LbrM06_V2.0690	*L. braziliensis*	128	27	154
LbrM06_V2.0690	*L. braziliensis*	52	207	258
LbrM06_V2.0690	*L. braziliensis*	74	280	353
LbrM06_V2.0700	*L. braziliensis*	126	1	126
LbrM06_V2.0700	*L. braziliensis*	92	206	297
LbrM06_V2.0700	*L. braziliensis*	90	333	422
LbrM06_V2.0700	*L. braziliensis*	68	583	650
LbrM06_V2.0700	*L. braziliensis*	46	670	715
LbrM06_V2.0700	*L. braziliensis*	48	878	925
LbrM06_V2.0700	*L. braziliensis*	182	1173	1354
LbrM06_V2.0700	*L. braziliensis*	71	1585	1655
LbrM06_V2.0700	*L. braziliensis*	48	1929	1976
LbrM06_V2.0700	*L. braziliensis*	76	2347	2422
LbrM06_V2.0700	*L. braziliensis*	191	2447	2637
LbrM06_V2.0710	*L. braziliensis*	466	77	542
LbrM06_V2.0710	*L. braziliensis*	105	561	665
LbrM06_V2.0710	*L. braziliensis*	102	684	785
LbrM06_V2.0710	*L. braziliensis*	82	832	913
LbrM06_V2.0710	*L. braziliensis*	49	991	1039
LbrM06_V2.0710	*L. braziliensis*	204	1071	1274
LbrM06_V2.0710	*L. braziliensis*	86	1342	1427
LbrM06_V2.0710	*L. braziliensis*	53	1598	1650
LbrM06_V2.0730	*L. braziliensis*	553	1	553
LbrM06_V2.0730	*L. braziliensis*	110	561	670
LbrM06_V2.0730	*L. braziliensis*	76	746	821
LbrM06_V2.0740	*L. braziliensis*	59	1	59
LbrM06_V2.0740	*L. braziliensis*	119	142	260
LbrM06_V2.0740	*L. braziliensis*	75	388	462
LbrM06_V2.0740	*L. braziliensis*	69	540	608
LbrM06_V2.0750	*L. braziliensis*	70	1	70
LbrM06_V2.0750	*L. braziliensis*	67	168	234
LbrM06_V2.0750	*L. braziliensis*	71	294	364

LbrM06_V2.0750	*L. braziliensis*	79	403	481
LbrM06_V2.0750	*L. braziliensis*	586	486	1071
LbrM06_V2.0750	*L. braziliensis*	94	1076	1169
LbrM06_V2.0760	*L. braziliensis*	391	1	391
LbrM06_V2.0760	*L. braziliensis*	348	418	765
LbrM06_V2.0760	*L. braziliensis*	317	822	1138
LbrM06_V2.0760	*L. braziliensis*	126	1230	1355
LbrM06_V2.0760	*L. braziliensis*	50	1404	1453
LbrM06_V2.0760	*L. braziliensis*	60	1468	1527
LbrM06_V2.0760	*L. braziliensis*	208	1543	1750
LbrM06_V2.0760	*L. braziliensis*	52	1782	1833
LbrM06_V2.0770	*L. braziliensis*	199	34	232
LbrM06_V2.0770	*L. braziliensis*	57	262	318
LbrM06_V2.0770	*L. braziliensis*	128	352	479
LbrM06_V2.0770	*L. braziliensis*	199	546	744
LbrM06_V2.0770	*L. braziliensis*	104	789	892
LbrM06_V2.0770	*L. braziliensis*	68	946	1013
LbrM06_V2.0770	*L. braziliensis*	58	1146	1203
LbrM06_V2.0780	*L. braziliensis*	199	47	245
LbrM06_V2.0780	*L. braziliensis*	84	290	373
LbrM06_V2.0780	*L. braziliensis*	122	437	558
LbrM06_V2.0780	*L. braziliensis*	73	687	759
LbrM06_V2.0780	*L. braziliensis*	102	786	887
LbrM06_V2.0780	*L. braziliensis*	70	995	1064
LbrM06_V2.0780	*L. braziliensis*	73	1118	1190
LbrM06_V2.0780	*L. braziliensis*	153	1381	1533
LbrM06_V2.0780	*L. braziliensis*	47	1551	1597
LbrM06_V2.0780	*L. braziliensis*	45	1650	1694
LbrM06_V2.0780	*L. braziliensis*	100	1894	1993
LbrM06_V2.0780	*L. braziliensis*	65	2127	2191
LbrM06_V2.0780	*L. braziliensis*	80	2346	2425
LbrM06_V2.0780	*L. braziliensis*	49	2432	2480
LbrM06_V2.0780	*L. braziliensis*	60	2724	2783
LbrM06_V2.0780	*L. braziliensis*	68	2876	2943
LbrM06_V2.0780	*L. braziliensis*	54	3165	3218
LbrM06_V2.0780	*L. braziliensis*	106	3258	3363
LbrM06_V2.0780	*L. braziliensis*	48	3511	3558
LbrM06_V2.0790	*L. braziliensis*	186	1	186
LbrM06_V2.0790	*L. braziliensis*	85	225	309
LbrM06_V2.0800	*L. braziliensis*	45	92	136
LbrM06_V2.0800	*L. braziliensis*	118	152	269
LbrM06_V2.0800	*L. braziliensis*	56	372	427
LbrM06_V2.0800	*L. braziliensis*	118	455	572
LbrM06_V2.0800	*L. braziliensis*	209	831	1039
LbrM06_V2.0800	*L. braziliensis*	316	1092	1407
LbrM06_V2.0810	*L. braziliensis*	46	72	117
LbrM06_V2.0810	*L. braziliensis*	111	128	238

LbrM06_V2.0820	*L. braziliensis*	351	72	422
LbrM06_V2.0820	*L. braziliensis*	53	427	479
LbrM06_V2.0820	*L. braziliensis*	270	518	787
LbrM06_V2.0820	*L. braziliensis*	54	862	915
LbrM06_V2.0820	*L. braziliensis*	96	985	1080
LbrM06_V2.0820	*L. braziliensis*	92	1123	1214
LbrM06_V2.0820	*L. braziliensis*	127	1219	1345
LbrM06_V2.0820	*L. braziliensis*	83	1373	1455
LbrM06_V2.0820	*L. braziliensis*	89	1503	1591
LbrM06_V2.0820	*L. braziliensis*	686	1671	2356
LbrM06_V2.0820	*L. braziliensis*	171	2389	2559
LbrM06_V2.0820	*L. braziliensis*	76	2579	2654
LbrM06_V2.0820	*L. braziliensis*	107	2714	2820
LbrM06_V2.0820	*L. braziliensis*	48	2926	2973
LbrM06_V2.0820	*L. braziliensis*	185	3156	3340
LbrM06_V2.0840	*L. braziliensis*	73	1	73
LbrM06_V2.0840	*L. braziliensis*	57	370	426
LbrM06_V2.0840	*L. braziliensis*	57	370	426
LbrM06_V2.0850	*L. braziliensis*	118	1	118
LbrM06_V2.0860	*L. braziliensis*	93	24	116
LbrM06_V2.0860	*L. braziliensis*	105	205	309
LbrM06_V2.0860	*L. braziliensis*	186	518	703
LbrM06_V2.0880	*L. braziliensis*	136	1	136
LbrM06_V2.0880	*L. braziliensis*	80	155	234
LbrM06_V2.0880	*L. braziliensis*	69	267	335
LbrM06_V2.0880	*L. braziliensis*	48	349	396
LbrM06_V2.0880	*L. braziliensis*	246	442	687
LbrM06_V2.0880	*L. braziliensis*	177	730	906
LbrM06_V2.0880	*L. braziliensis*	116	1028	1143
LbrM06_V2.0880	*L. braziliensis*	59	1256	1314
LbrM06_V2.0880	*L. braziliensis*	106	1341	1446
LbrM06_V2.0880	*L. braziliensis*	51	1493	1543
LbrM06_V2.0880	*L. braziliensis*	63	1605	1667
LbrM06_V2.0880	*L. braziliensis*	167	1752	1918
LbrM06_V2.0880	*L. braziliensis*	76	1999	2074
LbrM06_V2.0900	*L. braziliensis*	71	245	315
LbrM06_V2.0900	*L. braziliensis*	71	319	389
LbrM06_V2.0900	*L. braziliensis*	56	413	468
LbrM06_V2.0920	*L. braziliensis*	44	75	118
LbrM06_V2.0920	*L. braziliensis*	55	205	259
LbrM06_V2.0920	*L. braziliensis*	160	301	460
LbrM06_V2.0950	*L. braziliensis*	70	445	514
LbrM06_V2.0950	*L. braziliensis*	120	544	663
LbrM06_V2.0950	*L. braziliensis*	80	705	784
LbrM06_V2.0950	*L. braziliensis*	104	1229	1332
LbrM06_V2.0950	*L. braziliensis*	108	1382	1489
LbrM06_V2.0960	*L. braziliensis*	56	231	286

LbrM06_V2.0960	*L. braziliensis*	76	733	808
LbrM06_V2.0960	*L. braziliensis*	76	733	808
LbrM06_V2.0970	*L. braziliensis*	103	1	103
LbrM06_V2.0970	*L. braziliensis*	77	174	250
LbrM06_V2.0970	*L. braziliensis*	101	760	860
LbrM06_V2.0970	*L. braziliensis*	162	877	1038
LbrM06_V2.0980	*L. braziliensis*	89	79	167
LbrM06_V2.0980	*L. braziliensis*	50	204	253
LbrM06_V2.0980	*L. braziliensis*	50	204	253
LbrM06_V2.0990	*L. braziliensis*	66	116	181
LbrM06_V2.0990	*L. braziliensis*	42	283	324
LbrM06_V2.0990	*L. braziliensis*	42	708	749
LbrM06_V2.1000	*L. braziliensis*	50	1	50
LbrM06_V2.1000	*L. braziliensis*	146	85	230
LbrM06_V2.1010	*L. braziliensis*	49	150	198
LbrM06_V2.1010	*L. braziliensis*	76	382	457
LbrM06_V2.1010	*L. braziliensis*	157	536	692
LbrM06_V2.1010	*L. braziliensis*	126	707	832
LbrM06_V2.1020	*L. braziliensis*	190	1	190
LbrM06_V2.1020	*L. braziliensis*	138	236	373
LbrM06_V2.1020	*L. braziliensis*	105	441	545
LbrM06_V2.1020	*L. braziliensis*	89	629	717
LbrM06_V2.1040	*L. braziliensis*	43	271	313
LbrM06_V2.1070	*L. braziliensis*	143	1	143
LbrM06_V2.1070	*L. braziliensis*	86	185	270
LbrM06_V2.1070	*L. braziliensis*	48	281	328
LbrM06_V2.1070	*L. braziliensis*	97	517	613
LbrM06_V2.1090	*L. braziliensis*	44	64	107
LbrM06_V2.1090	*L. braziliensis*	234	134	367
LbrM06_V2.1090	*L. braziliensis*	408	467	874
LbrM06_V2.1090	*L. braziliensis*	231	902	1132
LbrM06_V2.1090	*L. braziliensis*	44	1141	1184
LbrM06_V2.1090	*L. braziliensis*	44	1221	1264
LbrM06_V2.1120	*L. braziliensis*	66	40	105
LbrM06_V2.1120	*L. braziliensis*	41	173	213
LbrM06_V2.1120	*L. braziliensis*	63	338	400
LbrM06_V2.1120	*L. braziliensis*	98	416	513
LbrM06_V2.1120	*L. braziliensis*	51	734	784
LbrM06_V2.1130	*L. braziliensis*	165	48	212
LbrM06_V2.1130	*L. braziliensis*	57	269	325
LbrM06_V2.1130	*L. braziliensis*	63	459	521
LbrM06_V2.1140	*L. braziliensis*	47	62	108
LbrM06_V2.1140	*L. braziliensis*	64	138	201
LbrM06_V2.1140	*L. braziliensis*	48	495	542
LbrM06_V2.1140	*L. braziliensis*	51	546	596
LbrM06_V2.1140	*L. braziliensis*	40	791	830
LbrM06_V2.1160	*L. braziliensis*	42	1	42

LbrM06_V2.1160	*L. braziliensis*	51	225	275
LbrM06_V2.1160	*L. braziliensis*	45	411	455
LbrM06_V2.1180	*L. braziliensis*	215	94	308
LbrM06_V2.1190	*L. braziliensis*	81	1	81
LbrM06_V2.1190	*L. braziliensis*	75	134	208
LbrM06_V2.1190	*L. braziliensis*	103	225	327
LbrM06_V2.1200	*L. braziliensis*	41	91	131
LbrM06_V2.1200	*L. braziliensis*	46	512	557
LbrM06_V2.1200	*L. braziliensis*	46	512	557
LbrM06_V2.1210	*L. braziliensis*	67	77	143
LbrM06_V2.1210	*L. braziliensis*	82	357	438
LbrM06_V2.1210	*L. braziliensis*	304	451	754
LbrM06_V2.1230	*L. braziliensis*	57	1	57
LbrM06_V2.1230	*L. braziliensis*	59	129	187
LbrM06_V2.1230	*L. braziliensis*	59	129	187
LbrM06_V2.1240	*L. braziliensis*	51	1	51
LbrM06_V2.1290	*L. braziliensis*	71	1	71
LbrM06_V2.1290	*L. braziliensis*	126	123	248
LbrM06_V2.1290	*L. braziliensis*	55	257	311
LbrM06_V2.1290	*L. braziliensis*	551	321	871
LbrM06_V2.1290	*L. braziliensis*	141	880	1020
LbrM06_V2.1290	*L. braziliensis*	282	1052	1333
LbrM06_V2.1290	*L. braziliensis*	292	1371	1662
LbrM06_V2.1290	*L. braziliensis*	336	1772	2107
LbrM06_V2.1290	*L. braziliensis*	147	2156	2302
LbrM06_V2.1290	*L. braziliensis*	325	2312	2636
LbrM06_V2.1290	*L. braziliensis*	106	2691	2796
LbrM06_V2.1300	*L. braziliensis*	463	132	594
LbrM07_V2.0020	*L. braziliensis*	55	1	55
LbrM07_V2.0020	*L. braziliensis*	107	119	225
LbrM07_V2.0040	*L. braziliensis*	43	172	214
LbrM07_V2.0040	*L. braziliensis*	74	241	314
LbrM07_V2.0040	*L. braziliensis*	74	241	314
LbrM07_V2.0050	*L. braziliensis*	60	654	713
LbrM07_V2.0060	*L. braziliensis*	45	73	117
LbrM07_V2.0100	*L. braziliensis*	97	480	576
LbrM07_V2.0120	*L. braziliensis*	49	1	49
LbrM07_V2.0120	*L. braziliensis*	63	532	594
LbrM07_V2.0120	*L. braziliensis*	45	1359	1403
LbrM07_V2.0120	*L. braziliensis*	50	1863	1912
LbrM07_V2.0120	*L. braziliensis*	46	2003	2048
LbrM07_V2.0140	*L. braziliensis*	89	261	349
LbrM07_V2.0150	*L. braziliensis*	63	20	82
LbrM07_V2.0160	*L. braziliensis*	573	244	816
LbrM07_V2.0170	*L. braziliensis*	142	188	329
LbrM07_V2.0170	*L. braziliensis*	288	467	754
LbrM07_V2.0170	*L. braziliensis*	157	838	994

LbrM07_V2.0180	*L. braziliensis*	109	1	109
LbrM07_V2.0180	*L. braziliensis*	85	164	248
LbrM07_V2.0180	*L. braziliensis*	43	267	309
LbrM07_V2.0180	*L. braziliensis*	103	324	426
LbrM07_V2.0180	*L. braziliensis*	40	471	510
LbrM07_V2.0180	*L. braziliensis*	49	529	577
LbrM07_V2.0180	*L. braziliensis*	52	601	652
LbrM07_V2.0180	*L. braziliensis*	81	783	863
LbrM07_V2.0180	*L. braziliensis*	74	872	945
LbrM07_V2.0180	*L. braziliensis*	314	963	1276
LbrM07_V2.0180	*L. braziliensis*	114	1347	1460
LbrM07_V2.0180	*L. braziliensis*	57	1508	1564
LbrM07_V2.0180	*L. braziliensis*	92	1619	1710
LbrM07_V2.0180	*L. braziliensis*	226	1752	1977
LbrM07_V2.0180	*L. braziliensis*	45	2023	2067
LbrM07_V2.0200	*L. braziliensis*	49	38	86
LbrM07_V2.0200	*L. braziliensis*	70	209	278
LbrM07_V2.0200	*L. braziliensis*	79	379	457
LbrM07_V2.0210	*L. braziliensis*	43	1	43
LbrM07_V2.0220	*L. braziliensis*	72	44	115
LbrM07_V2.0220	*L. braziliensis*	44	160	203
LbrM07_V2.0230	*L. braziliensis*	91	1	91
LbrM07_V2.0240	*L. braziliensis*	81	1	81
LbrM07_V2.0240	*L. braziliensis*	57	131	187
LbrM07_V2.0240	*L. braziliensis*	57	131	187
LbrM07_V2.0250	*L. braziliensis*	78	150	227
LbrM07_V2.0250	*L. braziliensis*	90	371	460
LbrM07_V2.0250	*L. braziliensis*	42	467	508
LbrM07_V2.0260	*L. braziliensis*	398	1	398
LbrM07_V2.0260	*L. braziliensis*	200	406	605
LbrM07_V2.0260	*L. braziliensis*	190	644	833
LbrM07_V2.0260	*L. braziliensis*	316	863	1178
LbrM07_V2.0260	*L. braziliensis*	42	1208	1249
LbrM07_V2.0260	*L. braziliensis*	136	1369	1504
LbrM07_V2.0260	*L. braziliensis*	136	1626	1761
LbrM07_V2.0290	*L. braziliensis*	61	171	231
LbrM07_V2.0290	*L. braziliensis*	212	347	558
LbrM07_V2.0290	*L. braziliensis*	49	620	668
LbrM07_V2.0290	*L. braziliensis*	182	816	997
LbrM07_V2.0290	*L. braziliensis*	60	1040	1099
LbrM07_V2.0290	*L. braziliensis*	141	1289	1429
LbrM07_V2.0290	*L. braziliensis*	41	1623	1663
LbrM07_V2.0290	*L. braziliensis*	89	1746	1834
LbrM07_V2.0290	*L. braziliensis*	208	1968	2175
LbrM07_V2.0290	*L. braziliensis*	99	2243	2341
LbrM07_V2.0290	*L. braziliensis*	124	2544	2667
LbrM07_V2.0290	*L. braziliensis*	248	2922	3169

LbrM07_V2.0290	*L. braziliensis*	72	3200	3271
LbrM07_V2.0290	*L. braziliensis*	86	3437	3522
LbrM07_V2.0290	*L. braziliensis*	58	3676	3733
LbrM07_V2.0290	*L. braziliensis*	71	3804	3874
LbrM07_V2.0290	*L. braziliensis*	101	3968	4068
LbrM07_V2.0290	*L. braziliensis*	437	4070	4506
LbrM07_V2.0290	*L. braziliensis*	260	4529	4788
LbrM07_V2.0290	*L. braziliensis*	81	4880	4960
LbrM07_V2.0290	*L. braziliensis*	114	5065	5178
LbrM07_V2.0290	*L. braziliensis*	58	5252	5309
LbrM07_V2.0290	*L. braziliensis*	44	5541	5584
LbrM07_V2.0290	*L. braziliensis*	77	5756	5832
LbrM07_V2.0310	*L. braziliensis*	218	80	297
LbrM07_V2.0310	*L. braziliensis*	46	431	476
LbrM07_V2.0310	*L. braziliensis*	98	535	632
LbrM07_V2.0320	*L. braziliensis*	97	1	97
LbrM07_V2.0320	*L. braziliensis*	46	249	294
LbrM07_V2.0320	*L. braziliensis*	55	577	631
LbrM07_V2.0320	*L. braziliensis*	86	658	743
LbrM07_V2.0330	*L. braziliensis*	65	63	127
LbrM07_V2.0330	*L. braziliensis*	296	574	869
LbrM07_V2.0340	*L. braziliensis*	58	139	196
LbrM07_V2.0340	*L. braziliensis*	102	244	345
LbrM07_V2.0340	*L. braziliensis*	97	437	533
LbrM07_V2.0350	*L. braziliensis*	80	41	120
LbrM07_V2.0350	*L. braziliensis*	71	289	359
LbrM07_V2.0350	*L. braziliensis*	46	425	470
LbrM07_V2.0350	*L. braziliensis*	130	605	734
LbrM07_V2.0350	*L. braziliensis*	63	767	829
LbrM07_V2.0360	*L. braziliensis*	90	1	90
LbrM07_V2.0360	*L. braziliensis*	72	531	602
LbrM07_V2.0370	*L. braziliensis*	263	243	505
LbrM07_V2.0370	*L. braziliensis*	74	572	645
LbrM07_V2.0370	*L. braziliensis*	74	572	645
LbrM07_V2.0390	*L. braziliensis*	45	1	45
LbrM07_V2.0390	*L. braziliensis*	155	64	218
LbrM07_V2.0390	*L. braziliensis*	60	346	405
LbrM07_V2.0390	*L. braziliensis*	79	493	571
LbrM07_V2.0390	*L. braziliensis*	86	600	685
LbrM07_V2.0400	*L. braziliensis*	41	59	99
LbrM07_V2.0400	*L. braziliensis*	128	152	279
LbrM07_V2.0400	*L. braziliensis*	90	387	476
LbrM07_V2.0400	*L. braziliensis*	95	593	687
LbrM07_V2.0400	*L. braziliensis*	67	848	914
LbrM07_V2.0400	*L. braziliensis*	60	958	1017
LbrM07_V2.0400	*L. braziliensis*	92	1563	1654
LbrM07_V2.0400	*L. braziliensis*	99	2070	2168

LbrM07_V2.0400	*L. braziliensis*	123	2208	2330
LbrM07_V2.0400	*L. braziliensis*	127	2434	2560
LbrM07_V2.0400	*L. braziliensis*	41	2704	2744
LbrM07_V2.0400	*L. braziliensis*	53	2821	2873
LbrM07_V2.0400	*L. braziliensis*	56	2991	3046
LbrM07_V2.0400	*L. braziliensis*	56	3413	3468
LbrM07_V2.0410	*L. braziliensis*	73	113	185
LbrM07_V2.0410	*L. braziliensis*	62	423	484
LbrM07_V2.0410	*L. braziliensis*	62	423	484
LbrM07_V2.0420	*L. braziliensis*	192	124	315
LbrM07_V2.0440	*L. braziliensis*	51	59	109
LbrM07_V2.0450	*L. braziliensis*	72	40	111
LbrM07_V2.0490	*L. braziliensis*	66	1	66
LbrM07_V2.0500	*L. braziliensis*	169	1	169
LbrM07_V2.0500	*L. braziliensis*	56	249	304
LbrM07_V2.0500	*L. braziliensis*	106	315	420
LbrM07_V2.0500	*L. braziliensis*	161	635	795
LbrM07_V2.0500	*L. braziliensis*	120	960	1079
LbrM07_V2.0500	*L. braziliensis*	58	1870	1927
LbrM07_V2.0520	*L. braziliensis*	52	28	79
LbrM07_V2.0540	*L. braziliensis*	52	1974	2025
LbrM07_V2.0540	*L. braziliensis*	50	2112	2161
LbrM07_V2.0540	*L. braziliensis*	44	2385	2428
LbrM07_V2.0540	*L. braziliensis*	101	2479	2579
LbrM07_V2.0540	*L. braziliensis*	43	2714	2756
LbrM07_V2.0540	*L. braziliensis*	62	3401	3462
LbrM07_V2.0540	*L. braziliensis*	56	3666	3721
LbrM07_V2.0540	*L. braziliensis*	54	3799	3852
LbrM07_V2.0540	*L. braziliensis*	48	3917	3964
LbrM07_V2.0540	*L. braziliensis*	41	4223	4263
LbrM07_V2.0540	*L. braziliensis*	45	4398	4442
LbrM07_V2.0550	*L. braziliensis*	44	1	44
LbrM07_V2.0550	*L. braziliensis*	50	116	165
LbrM07_V2.0550	*L. braziliensis*	90	210	299
LbrM07_V2.0550	*L. braziliensis*	93	303	395
LbrM07_V2.0560	*L. braziliensis*	75	60	134
LbrM07_V2.0570	*L. braziliensis*	75	60	134
LbrM07_V2.0580	*L. braziliensis*	147	32	178
LbrM07_V2.0590	*L. braziliensis*	64	1	64
LbrM07_V2.0600	*L. braziliensis*	160	1	160
LbrM07_V2.0600	*L. braziliensis*	158	230	387
LbrM07_V2.0600	*L. braziliensis*	226	400	625
LbrM07_V2.0610	*L. braziliensis*	245	95	339
LbrM07_V2.0610	*L. braziliensis*	153	628	780
LbrM07_V2.0610	*L. braziliensis*	72	800	871
LbrM07_V2.0610	*L. braziliensis*	119	897	1015
LbrM07_V2.0610	*L. braziliensis*	52	1067	1118

LbrM07_V2.0610	*L. braziliensis*	48	1152	1199
LbrM07_V2.0610	*L. braziliensis*	78	1237	1314
LbrM07_V2.0610	*L. braziliensis*	65	1545	1609
LbrM07_V2.0620	*L. braziliensis*	57	169	225
LbrM07_V2.0630	*L. braziliensis*	134	1	134
LbrM07_V2.0630	*L. braziliensis*	219	251	469
LbrM07_V2.0640	*L. braziliensis*	448	76	523
LbrM07_V2.0640	*L. braziliensis*	43	600	642
LbrM07_V2.0640	*L. braziliensis*	61	739	799
LbrM07_V2.0640	*L. braziliensis*	94	997	1090
LbrM07_V2.0640	*L. braziliensis*	89	1123	1211
LbrM07_V2.0650	*L. braziliensis*	539	79	617
LbrM07_V2.0650	*L. braziliensis*	48	728	775
LbrM07_V2.0650	*L. braziliensis*	79	1012	1090
LbrM07_V2.0670	*L. braziliensis*	45	109	153
LbrM07_V2.0670	*L. braziliensis*	40	158	197
LbrM07_V2.0670	*L. braziliensis*	40	158	197
LbrM07_V2.0690	*L. braziliensis*	122	21	142
LbrM07_V2.0690	*L. braziliensis*	129	144	272
LbrM07_V2.0690	*L. braziliensis*	159	290	448
LbrM07_V2.0730	*L. braziliensis*	122	1	122
LbrM07_V2.0740	*L. braziliensis*	127	1	127
LbrM07_V2.0760	*L. braziliensis*	69	1	69
LbrM07_V2.0760	*L. braziliensis*	100	87	186
LbrM07_V2.0760	*L. braziliensis*	67	213	279
LbrM07_V2.0760	*L. braziliensis*	538	283	820
LbrM07_V2.0760	*L. braziliensis*	197	877	1073
LbrM07_V2.0760	*L. braziliensis*	112	1076	1187
LbrM07_V2.0760	*L. braziliensis*	182	1295	1476
LbrM07_V2.0760	*L. braziliensis*	41	1528	1568
LbrM07_V2.0760	*L. braziliensis*	173	1661	1833
LbrM07_V2.0770	*L. braziliensis*	78	1	78
LbrM07_V2.0770	*L. braziliensis*	187	107	293
LbrM07_V2.0770	*L. braziliensis*	57	308	364
LbrM07_V2.0770	*L. braziliensis*	46	616	661
LbrM07_V2.0770	*L. braziliensis*	136	757	892
LbrM07_V2.0780	*L. braziliensis*	50	57	106
LbrM07_V2.0780	*L. braziliensis*	44	148	191
LbrM07_V2.0780	*L. braziliensis*	44	148	191
LbrM07_V2.0790	*L. braziliensis*	64	31	94
LbrM07_V2.0790	*L. braziliensis*	105	99	203
LbrM07_V2.0790	*L. braziliensis*	186	218	403
LbrM07_V2.0790	*L. braziliensis*	149	432	580
LbrM07_V2.0800	*L. braziliensis*	92	865	956
LbrM07_V2.0810	*L. braziliensis*	43	180	222
LbrM07_V2.0820	*L. braziliensis*	137	1	137
LbrM07_V2.0820	*L. braziliensis*	105	173	277

LbrM07_V2.0820	*L. braziliensis*	77	316	392
LbrM07_V2.0820	*L. braziliensis*	131	436	566
LbrM07_V2.0820	*L. braziliensis*	40	597	636
LbrM07_V2.0820	*L. braziliensis*	49	770	818
LbrM07_V2.0820	*L. braziliensis*	106	965	1070
LbrM07_V2.0820	*L. braziliensis*	79	1128	1206
LbrM07_V2.0820	*L. braziliensis*	48	1445	1492
LbrM07_V2.0820	*L. braziliensis*	103	1707	1809
LbrM07_V2.0820	*L. braziliensis*	48	1829	1876
LbrM07_V2.0820	*L. braziliensis*	53	1957	2009
LbrM07_V2.0820	*L. braziliensis*	118	2111	2228
LbrM07_V2.0820	*L. braziliensis*	73	2359	2431
LbrM07_V2.0820	*L. braziliensis*	66	2654	2719
LbrM07_V2.0820	*L. braziliensis*	139	2742	2880
LbrM07_V2.0820	*L. braziliensis*	155	3038	3192
LbrM07_V2.0820	*L. braziliensis*	108	3234	3341
LbrM07_V2.0820	*L. braziliensis*	65	3486	3550
LbrM07_V2.0820	*L. braziliensis*	58	3570	3627
LbrM07_V2.0820	*L. braziliensis*	235	3665	3899
LbrM07_V2.0830	*L. braziliensis*	141	124	264
LbrM07_V2.0840	*L. braziliensis*	228	1	228
LbrM07_V2.0840	*L. braziliensis*	131	323	453
LbrM07_V2.0840	*L. braziliensis*	267	533	799
LbrM07_V2.0840	*L. braziliensis*	82	925	1006
LbrM07_V2.0850	*L. braziliensis*	49	1	49
LbrM07_V2.0850	*L. braziliensis*	138	131	268
LbrM07_V2.0860	*L. braziliensis*	111	74	184
LbrM07_V2.0860	*L. braziliensis*	200	231	430
LbrM07_V2.0870	*L. braziliensis*	172	34	205
LbrM07_V2.0890	*L. braziliensis*	42	748	789
LbrM07_V2.0890	*L. braziliensis*	47	1429	1475
LbrM07_V2.0890	*L. braziliensis*	63	1877	1939
LbrM07_V2.0890	*L. braziliensis*	92	2014	2105
LbrM07_V2.0890	*L. braziliensis*	164	2110	2273
LbrM07_V2.0890	*L. braziliensis*	89	2287	2375
LbrM07_V2.0900	*L. braziliensis*	50	1	50
LbrM07_V2.0910	*L. braziliensis*	47	427	473
LbrM07_V2.0910	*L. braziliensis*	51	1840	1890
LbrM07_V2.0910	*L. braziliensis*	51	1840	1890
LbrM07_V2.0920.A	*L. braziliensis*	47	424	470
LbrM07_V2.0920.A	*L. braziliensis*	40	622	661
LbrM07_V2.0920.A	*L. braziliensis*	40	622	661
LbrM07_V2.0930	*L. braziliensis*	786	95	880
LbrM07_V2.0930	*L. braziliensis*	133	906	1038
LbrM07_V2.0930	*L. braziliensis*	228	1066	1293
LbrM07_V2.0940	*L. braziliensis*	73	1	73
LbrM07_V2.0940	*L. braziliensis*	44	132	175

LbrM07_V2.0940	*L. braziliensis*	133	244	376
LbrM07_V2.0950	*L. braziliensis*	236	1	236
LbrM07_V2.0950	*L. braziliensis*	52	270	321
LbrM07_V2.0950	*L. braziliensis*	45	353	397
LbrM07_V2.0950	*L. braziliensis*	44	413	456
LbrM07_V2.0950	*L. braziliensis*	201	533	733
LbrM07_V2.0970	*L. braziliensis*	228	1	228
LbrM07_V2.0970	*L. braziliensis*	43	339	381
LbrM07_V2.0970	*L. braziliensis*	91	529	619
LbrM07_V2.0970	*L. braziliensis*	88	814	901
LbrM07_V2.0970	*L. braziliensis*	254	940	1193
LbrM07_V2.0970	*L. braziliensis*	88	1204	1291
LbrM07_V2.0970	*L. braziliensis*	81	1350	1430
LbrM07_V2.0970	*L. braziliensis*	88	1490	1577
LbrM07_V2.0970	*L. braziliensis*	288	1641	1928
LbrM07_V2.0970	*L. braziliensis*	44	2064	2107
LbrM07_V2.0980	*L. braziliensis*	76	102	177
LbrM07_V2.0990	*L. braziliensis*	51	1	51
LbrM07_V2.0990	*L. braziliensis*	75	379	453
LbrM07_V2.0990	*L. braziliensis*	43	502	544
LbrM07_V2.0990	*L. braziliensis*	77	583	659
LbrM07_V2.0990	*L. braziliensis*	61	1215	1275
LbrM07_V2.0990	*L. braziliensis*	103	1784	1886
LbrM07_V2.0990	*L. braziliensis*	49	2306	2354
LbrM07_V2.1000	*L. braziliensis*	97	1	97
LbrM07_V2.1000	*L. braziliensis*	45	138	182
LbrM07_V2.1000	*L. braziliensis*	495	185	679
LbrM07_V2.1000	*L. braziliensis*	101	720	820
LbrM07_V2.1000	*L. braziliensis*	120	891	1010
LbrM07_V2.1000	*L. braziliensis*	145	1118	1262
LbrM07_V2.1000	*L. braziliensis*	41	1283	1323
LbrM07_V2.1000	*L. braziliensis*	42	1456	1497
LbrM07_V2.1000	*L. braziliensis*	208	1577	1784
LbrM07_V2.1000	*L. braziliensis*	89	1914	2002
LbrM07_V2.1000	*L. braziliensis*	173	2050	2222
LbrM07_V2.1000	*L. braziliensis*	48	2304	2351
LbrM07_V2.1010	*L. braziliensis*	1095	1	1095
LbrM07_V2.1020	*L. braziliensis*	75	46	120
LbrM07_V2.1020	*L. braziliensis*	165	164	328
LbrM07_V2.1020	*L. braziliensis*	122	693	814
LbrM07_V2.1040	*L. braziliensis*	240	88	327
LbrM07_V2.1050	*L. braziliensis*	46	1	46
LbrM07_V2.1050	*L. braziliensis*	275	184	458
LbrM07_V2.1060	*L. braziliensis*	44	1	44
LbrM07_V2.1070	*L. braziliensis*	365	1	365
LbrM07_V2.1070	*L. braziliensis*	47	588	634
LbrM07_V2.1070	*L. braziliensis*	141	912	1052

LbrM07_V2.1080	*L. braziliensis*	57	14	70
LbrM07_V2.1100	*L. braziliensis*	164	1	164
LbrM07_V2.1100	*L. braziliensis*	41	193	233
LbrM07_V2.1110	*L. braziliensis*	122	870	991
LbrM07_V2.1120	*L. braziliensis*	47	1	47
LbrM07_V2.1130	*L. braziliensis*	99	22	120
LbrM07_V2.1140	*L. braziliensis*	94	105	198
LbrM07_V2.1140	*L. braziliensis*	77	217	293
LbrM07_V2.1150	*L. braziliensis*	195	343	537
LbrM07_V2.1150	*L. braziliensis*	43	657	699
LbrM07_V2.1170	*L. braziliensis*	73	1	73
LbrM07_V2.1170	*L. braziliensis*	138	167	304
LbrM07_V2.1190	*L. braziliensis*	40	35	74
LbrM07_V2.1190	*L. braziliensis*	76	234	309
LbrM07_V2.1190	*L. braziliensis*	76	234	309
LbrM07_V2.1200	*L. braziliensis*	149	191	339
LbrM07_V2.1230	*L. braziliensis*	136	54	189
LbrM07_V2.1230	*L. braziliensis*	47	1103	1149
LbrM07_V2.1230	*L. braziliensis*	50	1271	1320
LbrM07_V2.1240	*L. braziliensis*	81	1	81
LbrM07_V2.1260	*L. braziliensis*	700	53	752
LbrM07_V2.1260	*L. braziliensis*	114	859	972
LbrM07_V2.1260	*L. braziliensis*	45	1148	1192
LbrM07_V2.1270	*L. braziliensis*	86	1	86
LbrM07_V2.1270	*L. braziliensis*	45	296	340
LbrM07_V2.1270	*L. braziliensis*	44	1021	1064
LbrM07_V2.1270	*L. braziliensis*	84	1140	1223
LbrM08_V2.0010	*L. braziliensis*	52	172	223
LbrM08_V2.0010	*L. braziliensis*	273	240	512
LbrM08_V2.0010	*L. braziliensis*	118	714	831
LbrM08_V2.0010	*L. braziliensis*	84	853	936
LbrM08_V2.0040	*L. braziliensis*	59	47	105
LbrM08_V2.0070	*L. braziliensis*	73	75	147
LbrM08_V2.0070	*L. braziliensis*	80	200	279
LbrM08_V2.0070	*L. braziliensis*	121	289	409
LbrM08_V2.0080	*L. braziliensis*	156	1	156
LbrM08_V2.0080	*L. braziliensis*	45	602	646
LbrM08_V2.0080	*L. braziliensis*	65	656	720
LbrM08_V2.0090	*L. braziliensis*	194	659	852
LbrM08_V2.0100	*L. braziliensis*	48	1	48
LbrM08_V2.0100	*L. braziliensis*	86	254	339
LbrM08_V2.0100	*L. braziliensis*	64	387	450
LbrM08_V2.0110	*L. braziliensis*	40	120	159
LbrM08_V2.0110	*L. braziliensis*	196	193	388
LbrM08_V2.0110	*L. braziliensis*	266	489	754
LbrM08_V2.0130	*L. braziliensis*	62	15	76
LbrM08_V2.0130	*L. braziliensis*	182	147	328

LbrM08_V2.0130	*L. braziliensis*	799	349	1147
LbrM08_V2.0150	*L. braziliensis*	50	303	352
LbrM08_V2.0150	*L. braziliensis*	57	525	581
LbrM08_V2.0150	*L. braziliensis*	83	683	765
LbrM08_V2.0150	*L. braziliensis*	45	857	901
LbrM08_V2.0150	*L. braziliensis*	65	961	1025
LbrM08_V2.0160	*L. braziliensis*	72	1	72
LbrM08_V2.0160	*L. braziliensis*	55	148	202
LbrM08_V2.0160	*L. braziliensis*	59	283	341
LbrM08_V2.0170	*L. braziliensis*	88	15	102
LbrM08_V2.0180	*L. braziliensis*	111	235	345
LbrM08_V2.0190	*L. braziliensis*	61	234	294
LbrM08_V2.0200	*L. braziliensis*	63	1	63
LbrM08_V2.0200	*L. braziliensis*	53	122	174
LbrM08_V2.0200	*L. braziliensis*	53	122	174
LbrM08_V2.0210	*L. braziliensis*	63	534	596
LbrM08_V2.0220	*L. braziliensis*	80	39	118
LbrM08_V2.0220	*L. braziliensis*	94	150	243
LbrM08_V2.0220	*L. braziliensis*	163	262	424
LbrM08_V2.0230	*L. braziliensis*	100	88	187
LbrM08_V2.0230	*L. braziliensis*	54	465	518
LbrM08_V2.0250	*L. braziliensis*	50	213	262
LbrM08_V2.0260	*L. braziliensis*	50	64	113
LbrM08_V2.0270	*L. braziliensis*	188	1	188
LbrM08_V2.0270	*L. braziliensis*	432	211	642
LbrM08_V2.0280	*L. braziliensis*	142	43	184
LbrM08_V2.0280	*L. braziliensis*	95	277	371
LbrM08_V2.0280	*L. braziliensis*	43	399	441
LbrM08_V2.0280	*L. braziliensis*	73	497	569
LbrM08_V2.0280	*L. braziliensis*	144	629	772
LbrM08_V2.0320	*L. braziliensis*	41	332	372
LbrM08_V2.0350	*L. braziliensis*	51	1	51
LbrM08_V2.0350	*L. braziliensis*	53	246	298
LbrM08_V2.0350	*L. braziliensis*	53	246	298
LbrM08_V2.0380	*L. braziliensis*	88	1	88
LbrM08_V2.0380	*L. braziliensis*	100	155	254
LbrM08_V2.0380	*L. braziliensis*	66	533	598
LbrM08_V2.0380	*L. braziliensis*	41	789	829
LbrM08_V2.0380	*L. braziliensis*	47	966	1012
LbrM08_V2.0380	*L. braziliensis*	70	1348	1417
LbrM08_V2.0380	*L. braziliensis*	126	1519	1644
LbrM08_V2.0380	*L. braziliensis*	53	1680	1732
LbrM08_V2.0380	*L. braziliensis*	57	1858	1914
LbrM08_V2.0380	*L. braziliensis*	45	2429	2473
LbrM08_V2.0380	*L. braziliensis*	65	2849	2913
LbrM08_V2.0380	*L. braziliensis*	189	3171	3359
LbrM08_V2.0380	*L. braziliensis*	105	3392	3496

LbrM08_V2.0380	*L. braziliensis*	52	3677	3728
LbrM08_V2.0390	*L. braziliensis*	53	66	118
LbrM08_V2.0390	*L. braziliensis*	51	198	248
LbrM08_V2.0390	*L. braziliensis*	152	305	456
LbrM08_V2.0390	*L. braziliensis*	254	474	727
LbrM08_V2.0390	*L. braziliensis*	83	896	978
LbrM08_V2.0390	*L. braziliensis*	111	1055	1165
LbrM08_V2.0390	*L. braziliensis*	51	1204	1254
LbrM08_V2.0390	*L. braziliensis*	285	1339	1623
LbrM08_V2.0390	*L. braziliensis*	1182	1632	2813
LbrM08_V2.0400	*L. braziliensis*	45	175	219
LbrM08_V2.0400	*L. braziliensis*	79	268	346
LbrM08_V2.0400	*L. braziliensis*	63	434	496
LbrM08_V2.0400	*L. braziliensis*	53	578	630
LbrM08_V2.0400	*L. braziliensis*	41	839	879
LbrM08_V2.0400	*L. braziliensis*	56	881	936
LbrM08_V2.0410	*L. braziliensis*	379	1	379
LbrM08_V2.0410	*L. braziliensis*	47	397	443
LbrM08_V2.0410	*L. braziliensis*	79	461	539
LbrM08_V2.0410	*L. braziliensis*	391	570	960
LbrM08_V2.0410	*L. braziliensis*	72	1062	1133
LbrM08_V2.0420	*L. braziliensis*	74	71	144
LbrM08_V2.0420	*L. braziliensis*	121	251	371
LbrM08_V2.0420	*L. braziliensis*	80	404	483
LbrM08_V2.0430	*L. braziliensis*	42	67	108
LbrM08_V2.0440	*L. braziliensis*	186	1	186
LbrM08_V2.0440	*L. braziliensis*	225	195	419
LbrM08_V2.0440	*L. braziliensis*	97	500	596
LbrM08_V2.0440	*L. braziliensis*	93	679	771
LbrM08_V2.0440	*L. braziliensis*	51	785	835
LbrM08_V2.0440	*L. braziliensis*	82	887	968
LbrM08_V2.0440	*L. braziliensis*	276	1144	1419
LbrM08_V2.0440	*L. braziliensis*	166	1703	1868
LbrM08_V2.0450	*L. braziliensis*	62	1	62
LbrM08_V2.0450	*L. braziliensis*	581	83	663
LbrM08_V2.0450	*L. braziliensis*	86	735	820
LbrM08_V2.0450	*L. braziliensis*	140	838	977
LbrM08_V2.0450	*L. braziliensis*	143	1000	1142
LbrM08_V2.0450	*L. braziliensis*	111	1197	1307
LbrM08_V2.0450	*L. braziliensis*	155	1453	1607
LbrM08_V2.0450	*L. braziliensis*	51	1658	1708
LbrM08_V2.0450	*L. braziliensis*	159	1721	1879
LbrM08_V2.0470	*L. braziliensis*	57	166	222
LbrM08_V2.0470	*L. braziliensis*	40	973	1012
LbrM08_V2.0470	*L. braziliensis*	40	973	1012
LbrM08_V2.0480	*L. braziliensis*	186	1	186
LbrM08_V2.0480	*L. braziliensis*	111	438	548

LbrM08_V2.0480	*L. braziliensis*	337	566	902
LbrM08_V2.0480	*L. braziliensis*	62	912	973
LbrM08_V2.0480	*L. braziliensis*	53	1088	1140
LbrM08_V2.0480	*L. braziliensis*	84	1498	1581
LbrM08_V2.0480	*L. braziliensis*	102	1596	1697
LbrM08_V2.0480	*L. braziliensis*	110	1882	1991
LbrM08_V2.0490	*L. braziliensis*	45	104	148
LbrM08_V2.0490	*L. braziliensis*	145	178	322
LbrM08_V2.0510	*L. braziliensis*	89	23	111
LbrM08_V2.0510	*L. braziliensis*	114	163	276
LbrM08_V2.0520	*L. braziliensis*	334	1	334
LbrM08_V2.0520	*L. braziliensis*	99	374	472
LbrM08_V2.0530	*L. braziliensis*	104	1	104
LbrM08_V2.0530	*L. braziliensis*	48	112	159
LbrM08_V2.0530	*L. braziliensis*	69	189	257
LbrM08_V2.0530	*L. braziliensis*	80	263	342
LbrM08_V2.0550	*L. braziliensis*	41	1	41
LbrM08_V2.0550	*L. braziliensis*	85	348	432
LbrM08_V2.0550	*L. braziliensis*	112	527	638
LbrM08_V2.0550	*L. braziliensis*	65	917	981
LbrM08_V2.0550	*L. braziliensis*	72	1046	1117
LbrM08_V2.0550	*L. braziliensis*	106	1161	1266
LbrM08_V2.0550	*L. braziliensis*	43	1435	1477
LbrM08_V2.0550	*L. braziliensis*	146	1532	1677
LbrM08_V2.0550	*L. braziliensis*	76	1803	1878
LbrM08_V2.0560	*L. braziliensis*	155	1	155
LbrM08_V2.0560	*L. braziliensis*	45	189	233
LbrM08_V2.0560	*L. braziliensis*	46	363	408
LbrM08_V2.0570	*L. braziliensis*	54	1	54
LbrM08_V2.0570	*L. braziliensis*	78	141	218
LbrM08_V2.0570	*L. braziliensis*	150	220	369
LbrM08_V2.0570	*L. braziliensis*	215	457	671
LbrM08_V2.0570	*L. braziliensis*	168	702	869
LbrM08_V2.0570	*L. braziliensis*	165	899	1063
LbrM08_V2.0570	*L. braziliensis*	72	1091	1162
LbrM08_V2.0570	*L. braziliensis*	178	1170	1347
LbrM08_V2.0570	*L. braziliensis*	154	1354	1507
LbrM08_V2.0570	*L. braziliensis*	100	1613	1712
LbrM08_V2.0570	*L. braziliensis*	61	1818	1878
LbrM08_V2.0580	*L. braziliensis*	64	50	113
LbrM08_V2.0580	*L. braziliensis*	47	142	188
LbrM08_V2.0580	*L. braziliensis*	82	271	352
LbrM08_V2.0590	*L. braziliensis*	95	1	95
LbrM08_V2.0600	*L. braziliensis*	106	1	106
LbrM08_V2.0600	*L. braziliensis*	64	121	184
LbrM08_V2.0610	*L. braziliensis*	49	39	87
LbrM08_V2.0620	*L. braziliensis*	68	150	217

LbrM08_V2.0630	*L. braziliensis*	64	159	222
LbrM08_V2.0640	*L. braziliensis*	41	281	321
LbrM08_V2.0640	*L. braziliensis*	180	509	688
LbrM08_V2.0640	*L. braziliensis*	58	805	862
LbrM08_V2.0640	*L. braziliensis*	100	959	1058
LbrM08_V2.0640	*L. braziliensis*	70	1470	1539
LbrM08_V2.0640	*L. braziliensis*	40	1708	1747
LbrM08_V2.0640	*L. braziliensis*	53	1799	1851
LbrM08_V2.0640	*L. braziliensis*	52	2015	2066
LbrM08_V2.0640	*L. braziliensis*	59	2068	2126
LbrM08_V2.0640	*L. braziliensis*	63	2253	2315
LbrM08_V2.0640	*L. braziliensis*	47	2409	2455
LbrM08_V2.0660	*L. braziliensis*	66	14	79
LbrM08_V2.0660	*L. braziliensis*	47	195	241
LbrM08_V2.0660	*L. braziliensis*	47	195	241
LbrM08_V2.0700	*L. braziliensis*	64	1	64
LbrM08_V2.0700	*L. braziliensis*	40	107	146
LbrM08_V2.0700	*L. braziliensis*	67	182	248
LbrM08_V2.0700	*L. braziliensis*	60	288	347
LbrM08_V2.0700	*L. braziliensis*	90	364	453
LbrM08_V2.0700	*L. braziliensis*	78	465	542
LbrM08_V2.0700	*L. braziliensis*	191	872	1062
LbrM08_V2.0700	*L. braziliensis*	66	1085	1150
LbrM08_V2.0700	*L. braziliensis*	141	1182	1322
LbrM08_V2.0700	*L. braziliensis*	77	1707	1783
LbrM08_V2.0710	*L. braziliensis*	58	39	96
LbrM08_V2.0710	*L. braziliensis*	44	333	376
LbrM08_V2.0710	*L. braziliensis*	44	333	376
LbrM08_V2.0720	*L. braziliensis*	83	1	83
LbrM08_V2.0720	*L. braziliensis*	519	93	611
LbrM08_V2.0720	*L. braziliensis*	270	640	909
LbrM08_V2.0720	*L. braziliensis*	46	957	1002
LbrM08_V2.0720	*L. braziliensis*	82	1343	1424
LbrM08_V2.0730	*L. braziliensis*	132	30	161
LbrM08_V2.0730	*L. braziliensis*	48	197	244
LbrM08_V2.0730	*L. braziliensis*	56	292	347
LbrM08_V2.0730	*L. braziliensis*	73	445	517
LbrM08_V2.0730	*L. braziliensis*	41	640	680
LbrM08_V2.0730	*L. braziliensis*	49	696	744
LbrM08_V2.0730	*L. braziliensis*	107	787	893
LbrM08_V2.0730	*L. braziliensis*	54	941	994
LbrM08_V2.0730	*L. braziliensis*	185	1022	1206
LbrM08_V2.0730	*L. braziliensis*	85	1252	1336
LbrM08_V2.0730	*L. braziliensis*	70	1359	1428
LbrM08_V2.0730	*L. braziliensis*	80	1531	1610
LbrM08_V2.0730	*L. braziliensis*	62	1714	1775
LbrM08_V2.0730	*L. braziliensis*	104	1780	1883

LbrM08_V2.0730	*L. braziliensis*	99	1930	2028
LbrM08_V2.0740	*L. braziliensis*	50	156	205
LbrM08_V2.0750	*L. braziliensis*	520	311	830
LbrM08_V2.0750	*L. braziliensis*	122	931	1052
LbrM08_V2.0760	*L. braziliensis*	67	25	91
LbrM08_V2.0760	*L. braziliensis*	106	152	257
LbrM08_V2.0760	*L. braziliensis*	106	152	257
LbrM08_V2.0770	*L. braziliensis*	146	1	146
LbrM08_V2.0780	*L. braziliensis*	94	80	173
LbrM08_V2.0780	*L. braziliensis*	49	316	364
LbrM08_V2.0780	*L. braziliensis*	48	381	428
LbrM08_V2.0780	*L. braziliensis*	98	472	569
LbrM08_V2.0790	*L. braziliensis*	218	23	240
LbrM08_V2.0790	*L. braziliensis*	138	253	390
LbrM08_V2.0790	*L. braziliensis*	211	440	650
LbrM08_V2.0790	*L. braziliensis*	67	773	839
LbrM08_V2.0800	*L. braziliensis*	40	239	278
LbrM08_V2.0800	*L. braziliensis*	68	291	358
LbrM08_V2.0800	*L. braziliensis*	74	445	518
LbrM08_V2.0800	*L. braziliensis*	49	762	810
LbrM08_V2.0800	*L. braziliensis*	55	936	990
LbrM08_V2.0800	*L. braziliensis*	64	1355	1418
LbrM08_V2.0800	*L. braziliensis*	133	1744	1876
LbrM08_V2.0800	*L. braziliensis*	57	2015	2071
LbrM08_V2.0800	*L. braziliensis*	63	2075	2137
LbrM08_V2.0810	*L. braziliensis*	67	336	402
LbrM08_V2.0820	*L. braziliensis*	106	336	441
LbrM08_V2.0830	*L. braziliensis*	106	336	441
LbrM08_V2.0840	*L. braziliensis*	49	1	49
LbrM08_V2.0840	*L. braziliensis*	80	526	605
LbrM08_V2.0840	*L. braziliensis*	80	526	605
LbrM08_V2.0850	*L. braziliensis*	51	92	142
LbrM08_V2.0860	*L. braziliensis*	51	118	168
LbrM08_V2.0870	*L. braziliensis*	46	30	75
LbrM08_V2.0870	*L. braziliensis*	48	120	167
LbrM08_V2.0870	*L. braziliensis*	68	275	342
LbrM08_V2.0880	*L. braziliensis*	73	182	254
LbrM08_V2.0880	*L. braziliensis*	41	336	376
LbrM08_V2.0880	*L. braziliensis*	47	469	515
LbrM08_V2.0890	*L. braziliensis*	107	1	107
LbrM08_V2.0890	*L. braziliensis*	48	427	474
LbrM08_V2.0890	*L. braziliensis*	43	699	741
LbrM08_V2.0910	*L. braziliensis*	291	377	667
LbrM08_V2.0910	*L. braziliensis*	59	732	790
LbrM08_V2.0920	*L. braziliensis*	89	204	292
LbrM08_V2.0920	*L. braziliensis*	84	469	552
LbrM08_V2.0920	*L. braziliensis*	53	630	682

LbrM08_V2.0920	*L. braziliensis*	81	698	778
LbrM08_V2.0950	*L. braziliensis*	47	159	205
LbrM08_V2.0950	*L. braziliensis*	106	265	370
LbrM08_V2.0960	*L. braziliensis*	73	1060	1132
LbrM08_V2.0980	*L. braziliensis*	209	77	285
LbrM08_V2.0980	*L. braziliensis*	56	336	391
LbrM08_V2.1000	*L. braziliensis*	125	155	279
LbrM08_V2.1000	*L. braziliensis*	83	363	445
LbrM08_V2.1000	*L. braziliensis*	51	466	516
LbrM08_V2.1000	*L. braziliensis*	436	673	1108
LbrM08_V2.1000	*L. braziliensis*	248	1544	1791
LbrM08_V2.1000	*L. braziliensis*	207	1925	2131
LbrM08_V2.1020	*L. braziliensis*	45	115	159
LbrM08_V2.1020	*L. braziliensis*	41	245	285
LbrM08_V2.1020	*L. braziliensis*	41	245	285
LbrM08_V2.1040	*L. braziliensis*	139	60	198
LbrM08_V2.1070	*L. braziliensis*	87	1	87
LbrM08_V2.1070	*L. braziliensis*	80	116	195
LbrM08_V2.1070	*L. braziliensis*	131	268	398
LbrM08_V2.1070	*L. braziliensis*	128	414	541
LbrM08_V2.1070	*L. braziliensis*	643	564	1206
LbrM08_V2.1070	*L. braziliensis*	143	1215	1357
LbrM09_V2.0010	*L. braziliensis*	75	13	87
LbrM09_V2.0040	*L. braziliensis*	80	525	604
LbrM09_V2.0050	*L. braziliensis*	45	260	304
LbrM09_V2.0090	*L. braziliensis*	42	118	159
LbrM09_V2.0090	*L. braziliensis*	59	257	315
LbrM09_V2.0090	*L. braziliensis*	62	319	380
LbrM09_V2.0090	*L. braziliensis*	45	573	617
LbrM09_V2.0100	*L. braziliensis*	59	167	225
LbrM09_V2.0110	*L. braziliensis*	77	24	100
LbrM09_V2.0110	*L. braziliensis*	65	179	243
LbrM09_V2.0110	*L. braziliensis*	65	179	243
LbrM09_V2.0120	*L. braziliensis*	87	92	178
LbrM09_V2.0120	*L. braziliensis*	322	261	582
LbrM09_V2.0140	*L. braziliensis*	228	25	252
LbrM09_V2.0140	*L. braziliensis*	207	261	467
LbrM09_V2.0140	*L. braziliensis*	202	499	700
LbrM09_V2.0150	*L. braziliensis*	66	185	250
LbrM09_V2.0150	*L. braziliensis*	47	471	517
LbrM09_V2.0150	*L. braziliensis*	55	557	611
LbrM09_V2.0150	*L. braziliensis*	283	635	917
LbrM09_V2.0150	*L. braziliensis*	127	961	1087
LbrM09_V2.0160	*L. braziliensis*	65	245	309
LbrM09_V2.0190	*L. braziliensis*	40	82	121
LbrM09_V2.0210	*L. braziliensis*	79	54	132
LbrM09_V2.0210	*L. braziliensis*	108	172	279

LbrM09_V2.0210	*L. braziliensis*	108	172	279
LbrM09_V2.0220	*L. braziliensis*	53	573	625
LbrM09_V2.0230	*L. braziliensis*	69	170	238
LbrM09_V2.0240	*L. braziliensis*	89	57	145
LbrM09_V2.0240	*L. braziliensis*	114	254	367
LbrM09_V2.0240	*L. braziliensis*	62	724	785
LbrM09_V2.0250	*L. braziliensis*	40	47	86
LbrM09_V2.0250	*L. braziliensis*	68	629	696
LbrM09_V2.0250	*L. braziliensis*	41	764	804
LbrM09_V2.0260	*L. braziliensis*	122	1	122
LbrM09_V2.0270	*L. braziliensis*	77	120	196
LbrM09_V2.0270	*L. braziliensis*	57	247	303
LbrM09_V2.0270	*L. braziliensis*	112	305	416
LbrM09_V2.0270	*L. braziliensis*	106	581	686
LbrM09_V2.0280	*L. braziliensis*	87	1	87
LbrM09_V2.0280	*L. braziliensis*	49	244	292
LbrM09_V2.0280	*L. braziliensis*	84	297	380
LbrM09_V2.0280	*L. braziliensis*	106	438	543
LbrM09_V2.0280	*L. braziliensis*	71	574	644
LbrM09_V2.0280	*L. braziliensis*	40	659	698
LbrM09_V2.0280	*L. braziliensis*	90	709	798
LbrM09_V2.0280	*L. braziliensis*	475	822	1296
LbrM09_V2.0280	*L. braziliensis*	45	1304	1348
LbrM09_V2.0290	*L. braziliensis*	56	54	109
LbrM09_V2.0290	*L. braziliensis*	65	144	208
LbrM09_V2.0290	*L. braziliensis*	65	144	208
LbrM09_V2.0300	*L. braziliensis*	450	1	450
LbrM09_V2.0300	*L. braziliensis*	43	699	741
LbrM09_V2.0300	*L. braziliensis*	40	792	831
LbrM09_V2.0310	*L. braziliensis*	112	531	642
LbrM09_V2.0320	*L. braziliensis*	62	636	697
LbrM09_V2.0330	*L. braziliensis*	274	631	904
LbrM09_V2.0330	*L. braziliensis*	44	987	1030
LbrM09_V2.0340	*L. braziliensis*	267	1	267
LbrM09_V2.0340	*L. braziliensis*	203	288	490
LbrM09_V2.0340	*L. braziliensis*	86	497	582
LbrM09_V2.0340	*L. braziliensis*	84	614	697
LbrM09_V2.0340	*L. braziliensis*	56	755	810
LbrM09_V2.0350	*L. braziliensis*	182	25	206
LbrM09_V2.0350	*L. braziliensis*	40	920	959
LbrM09_V2.0360	*L. braziliensis*	88	1	88
LbrM09_V2.0390	*L. braziliensis*	67	95	161
LbrM09_V2.0390	*L. braziliensis*	164	260	423
LbrM09_V2.0400	*L. braziliensis*	48	1	48
LbrM09_V2.0400	*L. braziliensis*	280	84	363
LbrM09_V2.0400	*L. braziliensis*	246	501	746
LbrM09_V2.0400	*L. braziliensis*	63	944	1006

LbrM09_V2.0400	*L. braziliensis*	112	1055	1166
LbrM09_V2.0400	*L. braziliensis*	166	1188	1353
LbrM09_V2.0400	*L. braziliensis*	78	1489	1566
LbrM09_V2.0400	*L. braziliensis*	173	1610	1782
LbrM09_V2.0400	*L. braziliensis*	85	1860	1944
LbrM09_V2.0410	*L. braziliensis*	109	1	109
LbrM09_V2.0420	*L. braziliensis*	97	19	115
LbrM09_V2.0430	*L. braziliensis*	60	165	224
LbrM09_V2.0430	*L. braziliensis*	85	417	501
LbrM09_V2.0430	*L. braziliensis*	80	582	661
LbrM09_V2.0430	*L. braziliensis*	92	726	817
LbrM09_V2.0430	*L. braziliensis*	64	839	902
LbrM09_V2.0430	*L. braziliensis*	158	922	1079
LbrM09_V2.0440	*L. braziliensis*	241	123	363
LbrM09_V2.0450	*L. braziliensis*	82	110	191
LbrM09_V2.0450	*L. braziliensis*	63	246	308
LbrM09_V2.0460	*L. braziliensis*	118	1	118
LbrM09_V2.0460	*L. braziliensis*	100	207	306
LbrM09_V2.0460	*L. braziliensis*	243	390	632
LbrM09_V2.0460	*L. braziliensis*	118	668	785
LbrM09_V2.0460	*L. braziliensis*	64	831	894
LbrM09_V2.0460	*L. braziliensis*	101	922	1022
LbrM09_V2.0470	*L. braziliensis*	50	1	50
LbrM09_V2.0480	*L. braziliensis*	62	1	62
LbrM09_V2.0480	*L. braziliensis*	152	198	349
LbrM09_V2.0480	*L. braziliensis*	47	771	817
LbrM09_V2.0480	*L. braziliensis*	66	843	908
LbrM09_V2.0480	*L. braziliensis*	68	961	1028
LbrM09_V2.0480	*L. braziliensis*	67	1422	1488
LbrM09_V2.0480	*L. braziliensis*	71	1594	1664
LbrM09_V2.0480	*L. braziliensis*	103	1873	1975
LbrM09_V2.0480	*L. braziliensis*	59	1985	2043
LbrM09_V2.0490	*L. braziliensis*	106	1	106
LbrM09_V2.0490	*L. braziliensis*	114	134	247
LbrM09_V2.0490	*L. braziliensis*	114	134	247
LbrM09_V2.0500	*L. braziliensis*	64	32	95
LbrM09_V2.0500	*L. braziliensis*	206	307	512
LbrM09_V2.0510	*L. braziliensis*	40	364	403
LbrM09_V2.0520	*L. braziliensis*	292	4	295
LbrM09_V2.0520	*L. braziliensis*	44	333	376
LbrM09_V2.0520	*L. braziliensis*	45	641	685
LbrM09_V2.0520	*L. braziliensis*	157	726	882
LbrM09_V2.0520	*L. braziliensis*	79	1248	1326
LbrM09_V2.0520	*L. braziliensis*	356	1359	1714
LbrM09_V2.0520	*L. braziliensis*	60	1742	1801
LbrM09_V2.0530	*L. braziliensis*	147	162	308
LbrM09_V2.0540	*L. braziliensis*	64	59	122

LbrM09_V2.0540	*L. braziliensis*	63	164	226
LbrM09_V2.0540	*L. braziliensis*	170	600	769
LbrM09_V2.0550	*L. braziliensis*	60	1	60
LbrM09_V2.0550	*L. braziliensis*	87	77	163
LbrM09_V2.0550	*L. braziliensis*	226	763	988
LbrM09_V2.0560	*L. braziliensis*	723	1	723
LbrM09_V2.0560	*L. braziliensis*	69	778	846
LbrM09_V2.0560	*L. braziliensis*	76	869	944
LbrM09_V2.0560	*L. braziliensis*	52	957	1008
LbrM09_V2.0560	*L. braziliensis*	70	1046	1115
LbrM09_V2.0570	*L. braziliensis*	71	41	111
LbrM09_V2.0570	*L. braziliensis*	68	172	239
LbrM09_V2.0570	*L. braziliensis*	74	260	333
LbrM09_V2.0590	*L. braziliensis*	67	94	160
LbrM09_V2.0590	*L. braziliensis*	134	213	346
LbrM09_V2.0590	*L. braziliensis*	51	447	497
LbrM09_V2.0610	*L. braziliensis*	58	35	92
LbrM09_V2.0610	*L. braziliensis*	73	101	173
LbrM09_V2.0610	*L. braziliensis*	57	191	247
LbrM09_V2.0610	*L. braziliensis*	98	261	358
LbrM09_V2.0610	*L. braziliensis*	56	367	422
LbrM09_V2.0620	*L. braziliensis*	494	1	494
LbrM09_V2.0630	*L. braziliensis*	67	221	287
LbrM09_V2.0630	*L. braziliensis*	48	485	532
LbrM09_V2.0630	*L. braziliensis*	68	571	638
LbrM09_V2.0630	*L. braziliensis*	98	648	745
LbrM09_V2.0630	*L. braziliensis*	119	995	1113
LbrM09_V2.0630	*L. braziliensis*	67	1130	1196
LbrM09_V2.0630	*L. braziliensis*	81	1232	1312
LbrM09_V2.0630	*L. braziliensis*	117	1336	1452
LbrM09_V2.0630	*L. braziliensis*	154	1544	1697
LbrM09_V2.0630	*L. braziliensis*	107	1877	1983
LbrM09_V2.0630	*L. braziliensis*	61	2381	2441
LbrM09_V2.0640	*L. braziliensis*	55	1	55
LbrM09_V2.0670	*L. braziliensis*	105	1	105
LbrM09_V2.0670	*L. braziliensis*	71	122	192
LbrM09_V2.0670	*L. braziliensis*	117	772	888
LbrM09_V2.0670	*L. braziliensis*	80	898	977
LbrM09_V2.0700	*L. braziliensis*	206	1	206
LbrM09_V2.0700	*L. braziliensis*	113	244	356
LbrM09_V2.0700	*L. braziliensis*	72	504	575
LbrM09_V2.0700	*L. braziliensis*	44	649	692
LbrM09_V2.0710	*L. braziliensis*	112	50	161
LbrM09_V2.0710	*L. braziliensis*	44	209	252
LbrM09_V2.0710	*L. braziliensis*	51	359	409
LbrM09_V2.0710	*L. braziliensis*	194	636	829
LbrM09_V2.0710	*L. braziliensis*	413	836	1248

LbrM09_V2.0720	*L. braziliensis*	48	141	188
LbrM09_V2.0720	*L. braziliensis*	49	409	457
LbrM09_V2.0720	*L. braziliensis*	49	409	457
LbrM09_V2.0730	*L. braziliensis*	70	476	545
LbrM09_V2.0730	*L. braziliensis*	48	577	624
LbrM09_V2.0740	*L. braziliensis*	46	1	46
LbrM09_V2.0740	*L. braziliensis*	55	158	212
LbrM09_V2.0750	*L. braziliensis*	59	84	142
LbrM09_V2.0750	*L. braziliensis*	42	189	230
LbrM09_V2.0750	*L. braziliensis*	42	189	230
LbrM09_V2.0760	*L. braziliensis*	58	66	123
LbrM09_V2.0760	*L. braziliensis*	56	179	234
LbrM09_V2.0760	*L. braziliensis*	86	260	345
LbrM09_V2.0780	*L. braziliensis*	406	48	453
LbrM09_V2.0790	*L. braziliensis*	104	1	104
LbrM09_V2.0790	*L. braziliensis*	103	233	335
LbrM09_V2.0790	*L. braziliensis*	51	364	414
LbrM09_V2.0790	*L. braziliensis*	51	442	492
LbrM09_V2.0800	*L. braziliensis*	147	208	354
LbrM09_V2.0810	*L. braziliensis*	84	1	84
LbrM09_V2.0810	*L. braziliensis*	96	445	540
LbrM09_V2.0810	*L. braziliensis*	96	445	540
LbrM09_V2.0820	*L. braziliensis*	47	198	244
LbrM09_V2.0820	*L. braziliensis*	45	279	323
LbrM09_V2.0820	*L. braziliensis*	65	806	870
LbrM09_V2.0820	*L. braziliensis*	57	915	971
LbrM09_V2.0820	*L. braziliensis*	44	1059	1102
LbrM09_V2.0820	*L. braziliensis*	46	1259	1304
LbrM09_V2.0820	*L. braziliensis*	64	1343	1406
LbrM09_V2.0820	*L. braziliensis*	97	1561	1657
LbrM09_V2.0830	*L. braziliensis*	43	37	79
LbrM09_V2.0830	*L. braziliensis*	84	139	222
LbrM09_V2.0830	*L. braziliensis*	77	634	710
LbrM09_V2.0830	*L. braziliensis*	72	1001	1072
LbrM09_V2.0840	*L. braziliensis*	74	22	95
LbrM09_V2.0840	*L. braziliensis*	102	116	217
LbrM09_V2.0840	*L. braziliensis*	75	274	348
LbrM09_V2.0840	*L. braziliensis*	57	531	587
LbrM09_V2.0840	*L. braziliensis*	83	707	789
LbrM09_V2.0840	*L. braziliensis*	101	821	921
LbrM09_V2.0840	*L. braziliensis*	66	952	1017
LbrM09_V2.0840	*L. braziliensis*	49	1217	1265
LbrM09_V2.0840	*L. braziliensis*	146	1319	1464
LbrM09_V2.0840	*L. braziliensis*	42	1495	1536
LbrM09_V2.0840	*L. braziliensis*	57	1628	1684
LbrM09_V2.0840	*L. braziliensis*	41	1792	1832
LbrM09_V2.0840	*L. braziliensis*	67	1975	2041

LbrM09_V2.0840	*L. braziliensis*	140	2153	2292
LbrM09_V2.0840	*L. braziliensis*	74	2330	2403
LbrM09_V2.0840	*L. braziliensis*	57	2491	2547
LbrM09_V2.0840	*L. braziliensis*	84	2593	2676
LbrM09_V2.0840	*L. braziliensis*	43	2775	2817
LbrM09_V2.0840	*L. braziliensis*	195	2867	3061
LbrM09_V2.0840	*L. braziliensis*	46	3073	3118
LbrM09_V2.0840	*L. braziliensis*	86	3178	3263
LbrM09_V2.0840	*L. braziliensis*	48	3559	3606
LbrM09_V2.0840	*L. braziliensis*	76	3691	3766
LbrM09_V2.0840	*L. braziliensis*	62	3801	3862
LbrM09_V2.0840	*L. braziliensis*	52	3918	3969
LbrM09_V2.0840	*L. braziliensis*	49	4140	4188
LbrM09_V2.0840	*L. braziliensis*	72	4216	4287
LbrM09_V2.0840	*L. braziliensis*	46	4328	4373
LbrM09_V2.0840	*L. braziliensis*	87	4529	4615
LbrM09_V2.0840	*L. braziliensis*	67	4674	4740
LbrM09_V2.0840	*L. braziliensis*	70	4854	4923
LbrM09_V2.0840	*L. braziliensis*	72	4962	5033
LbrM09_V2.0840	*L. braziliensis*	284	5066	5349
LbrM09_V2.0840	*L. braziliensis*	40	5385	5424
LbrM09_V2.0850	*L. braziliensis*	46	1	46
LbrM09_V2.0870	*L. braziliensis*	63	1	63
LbrM09_V2.0870	*L. braziliensis*	286	127	412
LbrM09_V2.0870	*L. braziliensis*	50	445	494
LbrM09_V2.0870	*L. braziliensis*	64	523	586
LbrM09_V2.0870	*L. braziliensis*	67	1128	1194
LbrM09_V2.0880	*L. braziliensis*	63	40	102
LbrM09_V2.0880	*L. braziliensis*	212	126	337
LbrM09_V2.0880	*L. braziliensis*	230	450	679
LbrM09_V2.0880	*L. braziliensis*	58	724	781
LbrM09_V2.0880	*L. braziliensis*	498	844	1341
LbrM09_V2.0890	*L. braziliensis*	44	82	125
LbrM09_V2.0900	*L. braziliensis*	88	286	373
LbrM09_V2.0900	*L. braziliensis*	51	509	559
LbrM09_V2.0910	*L. braziliensis*	49	1	49
LbrM09_V2.0910	*L. braziliensis*	48	74	121
LbrM09_V2.0910	*L. braziliensis*	54	410	463
LbrM09_V2.0930	*L. braziliensis*	56	204	259
LbrM09_V2.0950	*L. braziliensis*	66	106	171
LbrM09_V2.0960	*L. braziliensis*	89	1	89
LbrM09_V2.0970	*L. braziliensis*	89	1	89
LbrM09_V2.0980	*L. braziliensis*	89	1	89
LbrM09_V2.0990	*L. braziliensis*	46	532	577
LbrM09_V2.0990	*L. braziliensis*	317	745	1061
LbrM09_V2.0990	*L. braziliensis*	66	1109	1174
LbrM09_V2.1000	*L. braziliensis*	70	1	70

LbrM09_V2.1000	*L. braziliensis*	51	214	264
LbrM09_V2.1000	*L. braziliensis*	72	507	578
LbrM09_V2.1010	*L. braziliensis*	59	255	313
LbrM09_V2.1010	*L. braziliensis*	90	563	652
LbrM09_V2.1010	*L. braziliensis*	90	563	652
LbrM09_V2.1020	*L. braziliensis*	61	202	262
LbrM09_V2.1030	*L. braziliensis*	132	1	132
LbrM09_V2.1030	*L. braziliensis*	231	173	403
LbrM09_V2.1030	*L. braziliensis*	123	421	543
LbrM09_V2.1030	*L. braziliensis*	94	550	643
LbrM09_V2.1030	*L. braziliensis*	355	686	1040
LbrM09_V2.1040	*L. braziliensis*	49	1025	1073
LbrM09_V2.1040	*L. braziliensis*	56	1152	1207
LbrM09_V2.1040	*L. braziliensis*	106	1664	1769
LbrM09_V2.1040	*L. braziliensis*	65	1832	1896
LbrM09_V2.1040	*L. braziliensis*	100	1912	2011
LbrM09_V2.1040	*L. braziliensis*	76	2074	2149
LbrM09_V2.1040	*L. braziliensis*	87	2244	2330
LbrM09_V2.1040	*L. braziliensis*	75	2544	2618
LbrM09_V2.1040	*L. braziliensis*	176	2758	2933
LbrM09_V2.1040	*L. braziliensis*	169	3065	3233
LbrM09_V2.1040	*L. braziliensis*	62	3404	3465
LbrM09_V2.1040	*L. braziliensis*	200	3486	3685
LbrM09_V2.1040	*L. braziliensis*	45	4154	4198
LbrM09_V2.1040	*L. braziliensis*	57	4545	4601
LbrM09_V2.1040	*L. braziliensis*	52	4668	4719
LbrM09_V2.1040	*L. braziliensis*	233	4809	5041
LbrM09_V2.1040	*L. braziliensis*	46	5045	5090
LbrM09_V2.1040	*L. braziliensis*	63	5093	5155
LbrM09_V2.1040	*L. braziliensis*	58	5271	5328
LbrM09_V2.1050	*L. braziliensis*	53	129	181
LbrM09_V2.1060	*L. braziliensis*	94	29	122
LbrM09_V2.1080	*L. braziliensis*	109	1	109
LbrM09_V2.1080	*L. braziliensis*	44	245	288
LbrM09_V2.1090	*L. braziliensis*	99	1	99
LbrM09_V2.1090	*L. braziliensis*	69	117	185
LbrM09_V2.1090	*L. braziliensis*	172	212	383
LbrM09_V2.1100	*L. braziliensis*	152	1	152
LbrM09_V2.1110	*L. braziliensis*	72	138	209
LbrM09_V2.1140	*L. braziliensis*	60	394	453
LbrM09_V2.1150	*L. braziliensis*	104	1	104
LbrM09_V2.1150	*L. braziliensis*	51	110	160
LbrM09_V2.1160	*L. braziliensis*	64	1	64
LbrM09_V2.1160	*L. braziliensis*	338	71	408
LbrM09_V2.1160	*L. braziliensis*	120	410	529
LbrM09_V2.1190	*L. braziliensis*	44	27	70
LbrM09_V2.1200	*L. braziliensis*	58	1	58

LbrM09_V2.1200	*L. braziliensis*	58	123	180
LbrM09_V2.1200	*L. braziliensis*	65	218	282
LbrM09_V2.1210	*L. braziliensis*	90	1	90
LbrM09_V2.1210	*L. braziliensis*	42	248	289
LbrM09_V2.1210	*L. braziliensis*	84	442	525
LbrM09_V2.1210	*L. braziliensis*	50	536	585
LbrM09_V2.1230	*L. braziliensis*	63	1	63
LbrM09_V2.1230	*L. braziliensis*	410	117	526
LbrM09_V2.1240	*L. braziliensis*	92	1	92
LbrM09_V2.1240	*L. braziliensis*	44	235	278
LbrM09_V2.1240	*L. braziliensis*	50	450	499
LbrM09_V2.1250	*L. braziliensis*	88	185	272
LbrM09_V2.1250	*L. braziliensis*	72	623	694
LbrM09_V2.1250	*L. braziliensis*	54	739	792
LbrM09_V2.1250	*L. braziliensis*	53	877	929
LbrM09_V2.1250	*L. braziliensis*	75	1041	1115
LbrM09_V2.1250	*L. braziliensis*	44	1129	1172
LbrM09_V2.1250	*L. braziliensis*	59	1210	1268
LbrM09_V2.1250	*L. braziliensis*	74	1403	1476
LbrM09_V2.1260	*L. braziliensis*	166	230	395
LbrM09_V2.1270	*L. braziliensis*	245	189	433
LbrM09_V2.1270	*L. braziliensis*	40	454	493
LbrM09_V2.1280	*L. braziliensis*	63	1	63
LbrM09_V2.1280	*L. braziliensis*	54	360	413
LbrM09_V2.1280	*L. braziliensis*	54	360	413
LbrM09_V2.1290	*L. braziliensis*	78	1	78
LbrM09_V2.1290	*L. braziliensis*	68	201	268
LbrM09_V2.1290	*L. braziliensis*	41	564	604
LbrM09_V2.1290	*L. braziliensis*	48	655	702
LbrM09_V2.1290	*L. braziliensis*	46	1156	1201
LbrM09_V2.1290	*L. braziliensis*	268	1262	1529
LbrM09_V2.1290	*L. braziliensis*	44	1642	1685
LbrM09_V2.1290	*L. braziliensis*	51	1701	1751
LbrM09_V2.1290	*L. braziliensis*	46	2111	2156
LbrM09_V2.1290	*L. braziliensis*	46	2286	2331
LbrM09_V2.1290	*L. braziliensis*	57	2489	2545
LbrM09_V2.1310	*L. braziliensis*	52	122	173
LbrM09_V2.1310	*L. braziliensis*	172	291	462
LbrM09_V2.1330	*L. braziliensis*	91	60	150
LbrM09_V2.1330	*L. braziliensis*	65	244	308
LbrM09_V2.1330	*L. braziliensis*	65	244	308
LbrM09_V2.1370	*L. braziliensis*	91	247	337
LbrM09_V2.1380	*L. braziliensis*	108	248	355
LbrM09_V2.1380	*L. braziliensis*	66	540	605
LbrM09_V2.1380	*L. braziliensis*	66	540	605
LbrM09_V2.1390	*L. braziliensis*	75	116	190
LbrM09_V2.1400	*L. braziliensis*	44	1	44

LbrM09_V2.1420	*L. braziliensis*	72	1	72
LbrM09_V2.1420	*L. braziliensis*	116	153	268
LbrM09_V2.1430	*L. braziliensis*	187	1	187
LbrM09_V2.1430	*L. braziliensis*	57	475	531
LbrM09_V2.1430	*L. braziliensis*	57	475	531
LbrM09_V2.1470	*L. braziliensis*	78	159	236
LbrM09_V2.1470	*L. braziliensis*	76	408	483
LbrM09_V2.1470	*L. braziliensis*	43	531	573
LbrM09_V2.1480	*L. braziliensis*	98	21	118
LbrM09_V2.1500	*L. braziliensis*	96	57	152
LbrM09_V2.1540	*L. braziliensis*	50	269	318
LbrM09_V2.1540	*L. braziliensis*	41	526	566
LbrM09_V2.1540	*L. braziliensis*	41	526	566
LbrM09_V2.1570	*L. braziliensis*	47	1	47
LbrM09_V2.1580	*L. braziliensis*	57	143	199
LbrM09_V2.1580	*L. braziliensis*	64	229	292
LbrM09_V2.1580	*L. braziliensis*	86	1170	1255
LbrM09_V2.1590	*L. braziliensis*	42	297	338
LbrM09_V2.1600	*L. braziliensis*	75	57	131
LbrM09_V2.1610	*L. braziliensis*	592	72	663
LbrM09_V2.1620	*L. braziliensis*	72	1	72
LbrM10_V2.0030	*L. braziliensis*	60	254	313
LbrM10_V2.0100	*L. braziliensis*	181	54	234
LbrM10_V2.0120	*L. braziliensis*	52	22	73
LbrM10_V2.0120	*L. braziliensis*	50	105	154
LbrM10_V2.0120	*L. braziliensis*	67	210	276
LbrM10_V2.0130	*L. braziliensis*	46	168	213
LbrM10_V2.0130	*L. braziliensis*	476	284	759
LbrM10_V2.0140	*L. braziliensis*	129	71	199
LbrM10_V2.0150	*L. braziliensis*	116	1	116
LbrM10_V2.0150	*L. braziliensis*	233	135	367
LbrM10_V2.0160	*L. braziliensis*	242	1	242
LbrM10_V2.0160	*L. braziliensis*	200	257	456
LbrM10_V2.0170	*L. braziliensis*	50	62	111
LbrM10_V2.0170	*L. braziliensis*	60	431	490
LbrM10_V2.0190	*L. braziliensis*	133	91	223
LbrM10_V2.0190	*L. braziliensis*	281	289	569
LbrM10_V2.0200	*L. braziliensis*	77	331	407
LbrM10_V2.0210	*L. braziliensis*	52	420	471
LbrM10_V2.0220	*L. braziliensis*	43	638	680
LbrM10_V2.0230	*L. braziliensis*	49	224	272
LbrM10_V2.0240	*L. braziliensis*	135	1	135
LbrM10_V2.0250	*L. braziliensis*	46	1	46
LbrM10_V2.0260	*L. braziliensis*	232	86	317
LbrM10_V2.0260	*L. braziliensis*	179	319	497
LbrM10_V2.0260	*L. braziliensis*	55	540	594
LbrM10_V2.0270	*L. braziliensis*	51	1	51

LbrM10_V2.0270	*L. braziliensis*	45	86	130
LbrM10_V2.0270	*L. braziliensis*	46	236	281
LbrM10_V2.0290	*L. braziliensis*	137	1	137
LbrM10_V2.0290	*L. braziliensis*	57	197	253
LbrM10_V2.0290	*L. braziliensis*	89	306	394
LbrM10_V2.0290	*L. braziliensis*	62	418	479
LbrM10_V2.0290	*L. braziliensis*	79	682	760
LbrM10_V2.0290	*L. braziliensis*	42	775	816
LbrM10_V2.0290	*L. braziliensis*	54	875	928
LbrM10_V2.0300	*L. braziliensis*	58	1	58
LbrM10_V2.0300	*L. braziliensis*	112	151	262
LbrM10_V2.0300	*L. braziliensis*	75	408	482
LbrM10_V2.0300	*L. braziliensis*	101	531	631
LbrM10_V2.0300	*L. braziliensis*	345	640	984
LbrM10_V2.0300	*L. braziliensis*	59	1211	1269
LbrM10_V2.0300	*L. braziliensis*	79	1295	1373
LbrM10_V2.0300	*L. braziliensis*	245	1555	1799
LbrM10_V2.0300	*L. braziliensis*	112	1832	1943
LbrM10_V2.0300	*L. braziliensis*	78	2038	2115
LbrM10_V2.0300	*L. braziliensis*	112	2177	2288
LbrM10_V2.0320	*L. braziliensis*	265	1	265
LbrM10_V2.0320	*L. braziliensis*	334	312	645
LbrM10_V2.0330	*L. braziliensis*	236	1	236
LbrM10_V2.0330	*L. braziliensis*	48	447	494
LbrM10_V2.0330	*L. braziliensis*	48	447	494
LbrM10_V2.0340	*L. braziliensis*	83	35	117
LbrM10_V2.0340	*L. braziliensis*	169	243	411
LbrM10_V2.0340	*L. braziliensis*	89	454	542
LbrM10_V2.0340	*L. braziliensis*	104	698	801
LbrM10_V2.0340	*L. braziliensis*	133	838	970
LbrM10_V2.0340	*L. braziliensis*	57	974	1030
LbrM10_V2.0340	*L. braziliensis*	51	1047	1097
LbrM10_V2.0340	*L. braziliensis*	78	1104	1181
LbrM10_V2.0340	*L. braziliensis*	171	1312	1482
LbrM10_V2.0340	*L. braziliensis*	313	1501	1813
LbrM10_V2.0340	*L. braziliensis*	134	1913	2046
LbrM10_V2.0360	*L. braziliensis*	61	24	84
LbrM10_V2.0360	*L. braziliensis*	52	285	336
LbrM10_V2.0360	*L. braziliensis*	130	345	474
LbrM10_V2.0370	*L. braziliensis*	63	270	332
LbrM10_V2.0370	*L. braziliensis*	44	637	680
LbrM10_V2.0370	*L. braziliensis*	44	637	680
LbrM10_V2.0380	*L. braziliensis*	64	274	337
LbrM10_V2.0380	*L. braziliensis*	51	647	697
LbrM10_V2.0390	*L. braziliensis*	63	272	334
LbrM10_V2.0400	*L. braziliensis*	64	274	337
LbrM10_V2.0400	*L. braziliensis*	45	653	697

LbrM10_V2.0410	*L. braziliensis*	49	1	49
LbrM10_V2.0410	*L. braziliensis*	49	320	368
LbrM10_V2.0430	*L. braziliensis*	142	1	142
LbrM10_V2.0430	*L. braziliensis*	261	148	408
LbrM10_V2.0430	*L. braziliensis*	211	483	693
LbrM10_V2.0440	*L. braziliensis*	43	50	92
LbrM10_V2.0450	*L. braziliensis*	53	76	128
LbrM10_V2.0450	*L. braziliensis*	53	176	228
LbrM10_V2.0450	*L. braziliensis*	53	176	228
LbrM10_V2.0480	*L. braziliensis*	44	57	100
LbrM10_V2.0520	*L. braziliensis*	46	456	501
LbrM10_V2.0630	*L. braziliensis*	89	409	497
LbrM10_V2.0630	*L. braziliensis*	48	753	800
LbrM10_V2.0630	*L. braziliensis*	46	1077	1122
LbrM10_V2.0630	*L. braziliensis*	55	1733	1787
LbrM10_V2.0630	*L. braziliensis*	60	2328	2387
LbrM10_V2.0630	*L. braziliensis*	45	4841	4885
LbrM10_V2.0630	*L. braziliensis*	59	6157	6215
LbrM10_V2.0630	*L. braziliensis*	311	6423	6733
LbrM10_V2.0650	*L. braziliensis*	71	24	94
LbrM10_V2.0650	*L. braziliensis*	139	352	490
LbrM10_V2.0650	*L. braziliensis*	144	764	907
LbrM10_V2.0660	*L. braziliensis*	221	1	221
LbrM10_V2.0660	*L. braziliensis*	114	263	376
LbrM10_V2.0660	*L. braziliensis*	59	406	464
LbrM10_V2.0660	*L. braziliensis*	98	479	576
LbrM10_V2.0660	*L. braziliensis*	123	597	719
LbrM10_V2.0660	*L. braziliensis*	66	730	795
LbrM10_V2.0660	*L. braziliensis*	363	810	1172
LbrM10_V2.0670	*L. braziliensis*	56	245	300
LbrM10_V2.0670	*L. braziliensis*	176	529	704
LbrM10_V2.0670	*L. braziliensis*	284	761	1044
LbrM10_V2.0680	*L. braziliensis*	61	1	61
LbrM10_V2.0700	*L. braziliensis*	50	430	479
LbrM10_V2.0710	*L. braziliensis*	122	1	122
LbrM10_V2.0710	*L. braziliensis*	57	161	217
LbrM10_V2.0710	*L. braziliensis*	59	317	375
LbrM10_V2.0710	*L. braziliensis*	46	439	484
LbrM10_V2.0710	*L. braziliensis*	163	535	697
LbrM10_V2.0750	*L. braziliensis*	85	141	225
LbrM10_V2.0750	*L. braziliensis*	42	255	296
LbrM10_V2.0750	*L. braziliensis*	76	429	504
LbrM10_V2.0780	*L. braziliensis*	71	105	175
LbrM10_V2.0790	*L. braziliensis*	77	119	195
LbrM10_V2.0790	*L. braziliensis*	55	230	284
LbrM10_V2.0800	*L. braziliensis*	293	103	395
LbrM10_V2.0800	*L. braziliensis*	94	397	490

LbrM10_V2.0800	*L. braziliensis*	332	495	826
LbrM10_V2.0800	*L. braziliensis*	110	862	971
LbrM10_V2.0800	*L. braziliensis*	215	1000	1214
LbrM10_V2.0800	*L. braziliensis*	135	1241	1375
LbrM10_V2.0800	*L. braziliensis*	416	1385	1800
LbrM10_V2.0810	*L. braziliensis*	112	65	176
LbrM10_V2.0820	*L. braziliensis*	61	290	350
LbrM10_V2.0820	*L. braziliensis*	41	583	623
LbrM10_V2.0820	*L. braziliensis*	69	898	966
LbrM10_V2.0820	*L. braziliensis*	65	1274	1338
LbrM10_V2.0820	*L. braziliensis*	40	1381	1420
LbrM10_V2.0820	*L. braziliensis*	41	1603	1643
LbrM10_V2.0830	*L. braziliensis*	253	113	365
LbrM10_V2.0830	*L. braziliensis*	64	441	504
LbrM10_V2.0830	*L. braziliensis*	107	798	904
LbrM10_V2.0840	*L. braziliensis*	42	34	75
LbrM10_V2.0850	*L. braziliensis*	68	78	145
LbrM10_V2.0860	*L. braziliensis*	112	1	112
LbrM10_V2.0860	*L. braziliensis*	99	140	238
LbrM10_V2.0860	*L. braziliensis*	60	299	358
LbrM10_V2.0860	*L. braziliensis*	48	455	502
LbrM10_V2.0870	*L. braziliensis*	91	103	193
LbrM10_V2.0870	*L. braziliensis*	71	227	297
LbrM10_V2.0870	*L. braziliensis*	71	227	297
LbrM10_V2.0890	*L. braziliensis*	221	1	221
LbrM10_V2.0900	*L. braziliensis*	76	64	139
LbrM10_V2.0900	*L. braziliensis*	253	320	572
LbrM10_V2.0900	*L. braziliensis*	364	899	1262
LbrM10_V2.0910	*L. braziliensis*	60	197	256
LbrM10_V2.0910	*L. braziliensis*	82	688	769
LbrM10_V2.0910	*L. braziliensis*	61	999	1059
LbrM10_V2.0910	*L. braziliensis*	467	1376	1842
LbrM10_V2.0920	*L. braziliensis*	151	1	151
LbrM10_V2.0920	*L. braziliensis*	44	357	400
LbrM10_V2.0930	*L. braziliensis*	1160	1	1160
LbrM10_V2.0930	*L. braziliensis*	42	1167	1208
LbrM10_V2.0930	*L. braziliensis*	70	1341	1410
LbrM10_V2.0940	*L. braziliensis*	52	64	115
LbrM10_V2.0960	*L. braziliensis*	55	67	121
LbrM10_V2.1000	*L. braziliensis*	44	168	211
LbrM10_V2.1000	*L. braziliensis*	48	253	300
LbrM10_V2.1000	*L. braziliensis*	470	553	1022
LbrM10_V2.1000	*L. braziliensis*	114	1164	1277
LbrM10_V2.1000	*L. braziliensis*	40	1360	1399
LbrM10_V2.1000	*L. braziliensis*	56	1428	1483
LbrM10_V2.1030	*L. braziliensis*	200	624	823
LbrM10_V2.1030	*L. braziliensis*	44	865	908

LbrM10_V2.1030	*L. braziliensis*	112	914	1025
LbrM10_V2.1030	*L. braziliensis*	147	1090	1236
LbrM10_V2.1030	*L. braziliensis*	169	1311	1479
LbrM10_V2.1030	*L. braziliensis*	218	1520	1737
LbrM10_V2.1040	*L. braziliensis*	99	226	324
LbrM10_V2.1040	*L. braziliensis*	400	332	731
LbrM10_V2.1040	*L. braziliensis*	169	939	1107
LbrM10_V2.1050	*L. braziliensis*	343	68	410
LbrM10_V2.1050	*L. braziliensis*	174	469	642
LbrM10_V2.1050	*L. braziliensis*	231	721	951
LbrM10_V2.1050	*L. braziliensis*	114	958	1071
LbrM10_V2.1050	*L. braziliensis*	68	1089	1156
LbrM10_V2.1050	*L. braziliensis*	89	1197	1285
LbrM10_V2.1050	*L. braziliensis*	40	1366	1405
LbrM10_V2.1050	*L. braziliensis*	111	1454	1564
LbrM10_V2.1050	*L. braziliensis*	150	1589	1738
LbrM10_V2.1050	*L. braziliensis*	66	1794	1859
LbrM10_V2.1060	*L. braziliensis*	87	1	87
LbrM10_V2.1060	*L. braziliensis*	114	252	365
LbrM10_V2.1060	*L. braziliensis*	215	582	796
LbrM10_V2.1100	*L. braziliensis*	74	51	124
LbrM10_V2.1120	*L. braziliensis*	77	1	77
LbrM10_V2.1130	*L. braziliensis*	179	1	179
LbrM10_V2.1130	*L. braziliensis*	74	317	390
LbrM10_V2.1140	*L. braziliensis*	142	175	316
LbrM10_V2.1140	*L. braziliensis*	86	586	671
LbrM10_V2.1140	*L. braziliensis*	48	749	796
LbrM10_V2.1140	*L. braziliensis*	42	993	1034
LbrM10_V2.1150	*L. braziliensis*	59	68	126
LbrM10_V2.1150	*L. braziliensis*	74	143	216
LbrM10_V2.1150	*L. braziliensis*	74	143	216
LbrM10_V2.1160	*L. braziliensis*	63	234	296
LbrM10_V2.1170	*L. braziliensis*	117	1	117
LbrM10_V2.1170	*L. braziliensis*	307	214	520
LbrM10_V2.1180	*L. braziliensis*	114	1	114
LbrM10_V2.1180	*L. braziliensis*	56	118	173
LbrM10_V2.1180	*L. braziliensis*	71	397	467
LbrM10_V2.1220	*L. braziliensis*	143	161	303
LbrM10_V2.1220	*L. braziliensis*	185	335	519
LbrM10_V2.1240	*L. braziliensis*	104	655	758
LbrM10_V2.1240	*L. braziliensis*	48	1071	1118
LbrM10_V2.1250	*L. braziliensis*	56	202	257
LbrM10_V2.1260	*L. braziliensis*	423	58	480
LbrM10_V2.1270	*L. braziliensis*	40	178	217
LbrM10_V2.1300	*L. braziliensis*	345	1	345
LbrM10_V2.1310	*L. braziliensis*	126	65	190
LbrM10_V2.1330	*L. braziliensis*	68	1	68

LbrM10_V2.1330	*L. braziliensis*	223	292	514
LbrM10_V2.1340	*L. braziliensis*	49	258	306
LbrM10_V2.1450	*L. braziliensis*	60	254	313
LbrM10_V2.1480	*L. braziliensis*	47	77	123
LbrM10_V2.1480	*L. braziliensis*	60	268	327
LbrM10_V2.1480	*L. braziliensis*	55	390	444
LbrM10_V2.1490	*L. braziliensis*	55	75	129
LbrM10_V2.1490	*L. braziliensis*	54	174	227
LbrM10_V2.1490	*L. braziliensis*	79	311	389
LbrM10_V2.1500	*L. braziliensis*	207	1	207
LbrM10_V2.1500	*L. braziliensis*	97	221	317
LbrM10_V2.1530	*L. braziliensis*	61	1	61
LbrM10_V2.1530	*L. braziliensis*	55	68	122
LbrM10_V2.1530	*L. braziliensis*	55	68	122
LbrM10_V2.1550	*L. braziliensis*	173	1	173
LbrM10_V2.1570	*L. braziliensis*	44	57	100
LbrM10_V2.1590	*L. braziliensis*	143	1	143
LbrM10_V2.1610	*L. braziliensis*	44	57	100
LbrM10_V2.1640	*L. braziliensis*	150	1	150
LbrM10_V2.1640	*L. braziliensis*	42	590	631
LbrM10_V2.1650	*L. braziliensis*	44	57	100
LbrM11_V2.0010	*L. braziliensis*	93	90	182
LbrM11_V2.0010	*L. braziliensis*	89	511	599
LbrM11_V2.0010	*L. braziliensis*	77	1004	1080
LbrM11_V2.0020	*L. braziliensis*	156	1	156
LbrM11_V2.0020	*L. braziliensis*	48	245	292
LbrM11_V2.0020	*L. braziliensis*	57	495	551
LbrM11_V2.0020	*L. braziliensis*	81	866	946
LbrM11_V2.0020	*L. braziliensis*	99	977	1075
LbrM11_V2.0030	*L. braziliensis*	78	74	151
LbrM11_V2.0030	*L. braziliensis*	90	347	436
LbrM11_V2.0040	*L. braziliensis*	46	208	253
LbrM11_V2.0050	*L. braziliensis*	101	58	158
LbrM11_V2.0050	*L. braziliensis*	70	317	386
LbrM11_V2.0060	*L. braziliensis*	137	1	137
LbrM11_V2.0060	*L. braziliensis*	383	160	542
LbrM11_V2.0070	*L. braziliensis*	169	1	169
LbrM11_V2.0070	*L. braziliensis*	207	442	648
LbrM11_V2.0070	*L. braziliensis*	62	839	900
LbrM11_V2.0070	*L. braziliensis*	46	983	1028
LbrM11_V2.0080	*L. braziliensis*	159	1	159
LbrM11_V2.0080	*L. braziliensis*	85	554	638
LbrM11_V2.0080	*L. braziliensis*	421	661	1081
LbrM11_V2.0090	*L. braziliensis*	118	183	300
LbrM11_V2.0090	*L. braziliensis*	42	475	516
LbrM11_V2.0090	*L. braziliensis*	42	475	516
LbrM11_V2.0100	*L. braziliensis*	42	391	432

LbrM11_V2.0100	*L. braziliensis*	42	490	531
LbrM11_V2.0100	*L. braziliensis*	42	490	531
LbrM11_V2.0110	*L. braziliensis*	74	37	110
LbrM11_V2.0110	*L. braziliensis*	63	400	462
LbrM11_V2.0110	*L. braziliensis*	41	603	643
LbrM11_V2.0120	*L. braziliensis*	423	183	605
LbrM11_V2.0130	*L. braziliensis*	126	1	126
LbrM11_V2.0130	*L. braziliensis*	49	169	217
LbrM11_V2.0130	*L. braziliensis*	54	462	515
LbrM11_V2.0130	*L. braziliensis*	64	587	650
LbrM11_V2.0130	*L. braziliensis*	44	691	734
LbrM11_V2.0140	*L. braziliensis*	55	86	140
LbrM11_V2.0150	*L. braziliensis*	57	1	57
LbrM11_V2.0150	*L. braziliensis*	105	82	186
LbrM11_V2.0150	*L. braziliensis*	40	295	334
LbrM11_V2.0160	*L. braziliensis*	53	1	53
LbrM11_V2.0190	*L. braziliensis*	57	1	57
LbrM11_V2.0190	*L. braziliensis*	46	267	312
LbrM11_V2.0190	*L. braziliensis*	46	267	312
LbrM11_V2.0210	*L. braziliensis*	151	101	251
LbrM11_V2.0220	*L. braziliensis*	184	93	276
LbrM11_V2.0220	*L. braziliensis*	127	394	520
LbrM11_V2.0220	*L. braziliensis*	101	559	659
LbrM11_V2.0230	*L. braziliensis*	59	1	59
LbrM11_V2.0230	*L. braziliensis*	217	464	680
LbrM11_V2.0240	*L. braziliensis*	149	1	149
LbrM11_V2.0240	*L. braziliensis*	171	194	364
LbrM11_V2.0240	*L. braziliensis*	170	422	591
LbrM11_V2.0240	*L. braziliensis*	73	1139	1211
LbrM11_V2.0250	*L. braziliensis*	99	34	132
LbrM11_V2.0250	*L. braziliensis*	41	442	482
LbrM11_V2.0260	*L. braziliensis*	75	1	75
LbrM11_V2.0260	*L. braziliensis*	80	194	273
LbrM11_V2.0260	*L. braziliensis*	40	496	535
LbrM11_V2.0270	*L. braziliensis*	311	97	407
LbrM11_V2.0280	*L. braziliensis*	55	117	171
LbrM11_V2.0280	*L. braziliensis*	40	473	512
LbrM11_V2.0280	*L. braziliensis*	40	473	512
LbrM11_V2.0300	*L. braziliensis*	55	265	319
LbrM11_V2.0310	*L. braziliensis*	42	1	42
LbrM11_V2.0310	*L. braziliensis*	56	344	399
LbrM11_V2.0310	*L. braziliensis*	56	344	399
LbrM11_V2.0320	*L. braziliensis*	129	242	370
LbrM11_V2.0340	*L. braziliensis*	84	1	84
LbrM11_V2.0340	*L. braziliensis*	159	126	284
LbrM11_V2.0340	*L. braziliensis*	135	293	427
LbrM11_V2.0340	*L. braziliensis*	47	445	491

LbrM11_V2.0340	*L. braziliensis*	89	530	618
LbrM11_V2.0350	*L. braziliensis*	99	1	99
LbrM11_V2.0350	*L. braziliensis*	80	134	213
LbrM11_V2.0350	*L. braziliensis*	75	708	782
LbrM11_V2.0350	*L. braziliensis*	40	863	902
LbrM11_V2.0350	*L. braziliensis*	136	937	1072
LbrM11_V2.0360	*L. braziliensis*	69	1	69
LbrM11_V2.0360	*L. braziliensis*	40	259	298
LbrM11_V2.0360	*L. braziliensis*	40	259	298
LbrM11_V2.0380	*L. braziliensis*	192	58	249
LbrM11_V2.0380	*L. braziliensis*	41	256	296
LbrM11_V2.0380	*L. braziliensis*	204	407	610
LbrM11_V2.0390	*L. braziliensis*	211	24	234
LbrM11_V2.0390	*L. braziliensis*	100	261	360
LbrM11_V2.0390	*L. braziliensis*	245	379	623
LbrM11_V2.0390	*L. braziliensis*	128	627	754
LbrM11_V2.0390	*L. braziliensis*	63	792	854
LbrM11_V2.0390	*L. braziliensis*	93	1015	1107
LbrM11_V2.0420	*L. braziliensis*	69	1	69
LbrM11_V2.0420	*L. braziliensis*	88	72	159
LbrM11_V2.0420	*L. braziliensis*	56	230	285
LbrM11_V2.0420	*L. braziliensis*	218	291	508
LbrM11_V2.0420	*L. braziliensis*	60	573	632
LbrM11_V2.0420	*L. braziliensis*	59	661	719
LbrM11_V2.0420	*L. braziliensis*	109	826	934
LbrM11_V2.0420	*L. braziliensis*	63	964	1026
LbrM11_V2.0420	*L. braziliensis*	80	1168	1247
LbrM11_V2.0420	*L. braziliensis*	59	1285	1343
LbrM11_V2.0420	*L. braziliensis*	239	1355	1593
LbrM11_V2.0420	*L. braziliensis*	42	1621	1662
LbrM11_V2.0430	*L. braziliensis*	59	310	368
LbrM11_V2.0440	*L. braziliensis*	44	313	356
LbrM11_V2.0440	*L. braziliensis*	41	613	653
LbrM11_V2.0470	*L. braziliensis*	44	532	575
LbrM11_V2.0490	*L. braziliensis*	90	43	132
LbrM11_V2.0490	*L. braziliensis*	54	212	265
LbrM11_V2.0490	*L. braziliensis*	54	212	265
LbrM11_V2.0500	*L. braziliensis*	76	1	76
LbrM11_V2.0500	*L. braziliensis*	179	116	294
LbrM11_V2.0500	*L. braziliensis*	77	336	412
LbrM11_V2.0500	*L. braziliensis*	53	414	466
LbrM11_V2.0500	*L. braziliensis*	84	472	555
LbrM11_V2.0510	*L. braziliensis*	207	240	446
LbrM11_V2.0510	*L. braziliensis*	106	533	638
LbrM11_V2.0520	*L. braziliensis*	158	44	201
LbrM11_V2.0520	*L. braziliensis*	96	318	413
LbrM11_V2.0520	*L. braziliensis*	79	534	612

LbrM11_V2.0520	*L. braziliensis*	288	723	1010
LbrM11_V2.0520	*L. braziliensis*	250	1040	1289
LbrM11_V2.0530	*L. braziliensis*	57	1	57
LbrM11_V2.0530	*L. braziliensis*	40	189	228
LbrM11_V2.0530	*L. braziliensis*	87	358	444
LbrM11_V2.0540	*L. braziliensis*	112	88	199
LbrM11_V2.0550	*L. braziliensis*	93	73	165
LbrM11_V2.0560	*L. braziliensis*	93	73	165
LbrM11_V2.0570	*L. braziliensis*	93	79	171
LbrM11_V2.0600	*L. braziliensis*	164	1	164
LbrM11_V2.0600	*L. braziliensis*	257	206	462
LbrM11_V2.0610	*L. braziliensis*	65	24	88
LbrM11_V2.0610	*L. braziliensis*	84	131	214
LbrM11_V2.0610	*L. braziliensis*	49	310	358
LbrM11_V2.0610	*L. braziliensis*	82	441	522
LbrM11_V2.0620	*L. braziliensis*	236	1	236
LbrM11_V2.0620	*L. braziliensis*	337	244	580
LbrM11_V2.0630	*L. braziliensis*	97	1	97
LbrM11_V2.0630	*L. braziliensis*	92	150	241
LbrM11_V2.0630	*L. braziliensis*	41	244	284
LbrM11_V2.0630	*L. braziliensis*	43	292	334
LbrM11_V2.0630	*L. braziliensis*	100	360	459
LbrM11_V2.0630	*L. braziliensis*	72	791	862
LbrM11_V2.0630	*L. braziliensis*	129	867	995
LbrM11_V2.0630	*L. braziliensis*	398	1022	1419
LbrM11_V2.0650	*L. braziliensis*	68	128	195
LbrM11_V2.0660	*L. braziliensis*	61	318	378
LbrM11_V2.0660	*L. braziliensis*	105	642	746
LbrM11_V2.0660	*L. braziliensis*	70	983	1052
LbrM11_V2.0660	*L. braziliensis*	92	1158	1249
LbrM11_V2.0660	*L. braziliensis*	136	1534	1669
LbrM11_V2.0670	*L. braziliensis*	57	192	248
LbrM11_V2.0670	*L. braziliensis*	76	308	383
LbrM11_V2.0670	*L. braziliensis*	222	477	698
LbrM11_V2.0670	*L. braziliensis*	61	736	796
LbrM11_V2.0670	*L. braziliensis*	605	886	1490
LbrM11_V2.0690	*L. braziliensis*	55	35	89
LbrM11_V2.0690	*L. braziliensis*	60	202	261
LbrM11_V2.0690	*L. braziliensis*	40	412	451
LbrM11_V2.0690	*L. braziliensis*	42	453	494
LbrM11_V2.0690	*L. braziliensis*	48	961	1008
LbrM11_V2.0700	*L. braziliensis*	105	86	190
LbrM11_V2.0710	*L. braziliensis*	90	1	90
LbrM11_V2.0710	*L. braziliensis*	253	94	346
LbrM11_V2.0710	*L. braziliensis*	63	764	826
LbrM11_V2.0710	*L. braziliensis*	56	914	969
LbrM11_V2.0710	*L. braziliensis*	53	1020	1072

LbrM11_V2.0710	*L. braziliensis*	48	1120	1167
LbrM11_V2.0710	*L. braziliensis*	155	1291	1445
LbrM11_V2.0710	*L. braziliensis*	40	1486	1525
LbrM11_V2.0720	*L. braziliensis*	75	38	112
LbrM11_V2.0720	*L. braziliensis*	59	271	329
LbrM11_V2.0720	*L. braziliensis*	165	425	589
LbrM11_V2.0730	*L. braziliensis*	197	71	267
LbrM11_V2.0730	*L. braziliensis*	106	272	377
LbrM11_V2.0740	*L. braziliensis*	206	198	403
LbrM11_V2.0740	*L. braziliensis*	513	425	937
LbrM11_V2.0740	*L. braziliensis*	65	1056	1120
LbrM11_V2.0740	*L. braziliensis*	63	1158	1220
LbrM11_V2.0740	*L. braziliensis*	110	1279	1388
LbrM11_V2.0740	*L. braziliensis*	50	1404	1453
LbrM11_V2.0750	*L. braziliensis*	76	1	76
LbrM11_V2.0750	*L. braziliensis*	60	85	144
LbrM11_V2.0750	*L. braziliensis*	67	193	259
LbrM11_V2.0750	*L. braziliensis*	81	279	359
LbrM11_V2.0750	*L. braziliensis*	126	391	516
LbrM11_V2.0780	*L. braziliensis*	61	42	102
LbrM11_V2.0780	*L. braziliensis*	126	124	249
LbrM11_V2.0780	*L. braziliensis*	41	400	440
LbrM11_V2.0790	*L. braziliensis*	62	242	303
LbrM11_V2.0790	*L. braziliensis*	64	700	763
LbrM11_V2.0810	*L. braziliensis*	63	1	63
LbrM11_V2.0810	*L. braziliensis*	72	296	367
LbrM11_V2.0820	*L. braziliensis*	161	5	165
LbrM11_V2.0820	*L. braziliensis*	41	195	235
LbrM11_V2.0820	*L. braziliensis*	51	411	461
LbrM11_V2.0820	*L. braziliensis*	297	750	1046
LbrM11_V2.0820	*L. braziliensis*	132	1091	1222
LbrM11_V2.0830	*L. braziliensis*	51	1	51
LbrM11_V2.0840	*L. braziliensis*	136	1	136
LbrM11_V2.0840	*L. braziliensis*	44	216	259
LbrM11_V2.0840	*L. braziliensis*	413	307	719
LbrM11_V2.0860	*L. braziliensis*	117	1	117
LbrM11_V2.0860	*L. braziliensis*	62	235	296
LbrM11_V2.0870	*L. braziliensis*	143	1	143
LbrM11_V2.0890	*L. braziliensis*	65	83	147
LbrM11_V2.0900	*L. braziliensis*	91	193	283
LbrM11_V2.0910	*L. braziliensis*	65	57	121
LbrM11_V2.0920	*L. braziliensis*	56	395	450
LbrM11_V2.0920	*L. braziliensis*	41	459	499
LbrM11_V2.0920	*L. braziliensis*	387	710	1096
LbrM11_V2.0930	*L. braziliensis*	294	1	294
LbrM11_V2.0940	*L. braziliensis*	41	358	398
LbrM11_V2.0940	*L. braziliensis*	59	514	572

LbrM11_V2.0940	*L. braziliensis*	59	514	572
LbrM11_V2.0960	*L. braziliensis*	52	1721	1772
LbrM11_V2.0970	*L. braziliensis*	211	1	211
LbrM11_V2.0980	*L. braziliensis*	72	310	381
LbrM11_V2.1000	*L. braziliensis*	93	116	208
LbrM11_V2.1010	*L. braziliensis*	144	1	144
LbrM11_V2.1040	*L. braziliensis*	67	1	67
LbrM11_V2.1040	*L. braziliensis*	75	1772	1846
LbrM11_V2.1040	*L. braziliensis*	75	1772	1846
LbrM11_V2.1050	*L. braziliensis*	67	1	67
LbrM11_V2.1080	*L. braziliensis*	114	78	191
LbrM11_V2.1080	*L. braziliensis*	74	220	293
LbrM11_V2.1080	*L. braziliensis*	51	391	441
LbrM11_V2.1080	*L. braziliensis*	122	633	754
LbrM11_V2.1080	*L. braziliensis*	51	894	944
LbrM11_V2.1080	*L. braziliensis*	52	1398	1449
LbrM11_V2.1080	*L. braziliensis*	67	1483	1549
LbrM11_V2.1080	*L. braziliensis*	69	1570	1638
LbrM11_V2.1090	*L. braziliensis*	79	303	381
LbrM11_V2.1110	*L. braziliensis*	51	1	51
LbrM11_V2.1110	*L. braziliensis*	93	89	181
LbrM11_V2.1110	*L. braziliensis*	93	89	181
LbrM11_V2.1120	*L. braziliensis*	50	1	50
LbrM11_V2.1140	*L. braziliensis*	46	1	46
LbrM11_V2.1140	*L. braziliensis*	40	541	580
LbrM11_V2.1140	*L. braziliensis*	40	541	580
LbrM11_V2.1150	*L. braziliensis*	69	109	177
LbrM11_V2.1150	*L. braziliensis*	43	326	368
LbrM11_V2.1150	*L. braziliensis*	43	326	368
LbrM11_V2.1160	*L. braziliensis*	173	204	376
LbrM11_V2.1160	*L. braziliensis*	216	555	770
LbrM11_V2.1160	*L. braziliensis*	175	784	958
LbrM12_V2.0010	*L. braziliensis*	169	1	169
LbrM12_V2.0010	*L. braziliensis*	57	249	305
LbrM12_V2.0090	*L. braziliensis*	432	149	580
LbrM12_V2.0130	*L. braziliensis*	70	134	203
LbrM12_V2.0140	*L. braziliensis*	186	1	186
LbrM12_V2.0140	*L. braziliensis*	462	201	662
LbrM12_V2.0140	*L. braziliensis*	103	712	814
LbrM12_V2.0150	*L. braziliensis*	223	42	264
LbrM12_V2.0150	*L. braziliensis*	85	326	410
LbrM12_V2.0150	*L. braziliensis*	154	420	573
LbrM12_V2.0150	*L. braziliensis*	685	613	1297
LbrM12_V2.0160	*L. braziliensis*	42	182	223
LbrM12_V2.0160	*L. braziliensis*	85	269	353
LbrM12_V2.0160	*L. braziliensis*	72	465	536
LbrM12_V2.0170	*L. braziliensis*	78	280	357

LbrM12_V2.0180	*L. braziliensis*	326	1	326
LbrM12_V2.0180	*L. braziliensis*	113	499	611
LbrM12_V2.0180	*L. braziliensis*	58	774	831
LbrM12_V2.0180	*L. braziliensis*	44	890	933
LbrM12_V2.0180	*L. braziliensis*	137	1107	1243
LbrM12_V2.0190	*L. braziliensis*	82	194	275
LbrM12_V2.0200	*L. braziliensis*	98	118	215
LbrM12_V2.0200	*L. braziliensis*	151	364	514
LbrM12_V2.0200	*L. braziliensis*	151	364	514
LbrM12_V2.0210	*L. braziliensis*	43	1	43
LbrM12_V2.0210	*L. braziliensis*	52	107	158
LbrM12_V2.0210	*L. braziliensis*	55	223	277
LbrM12_V2.0210	*L. braziliensis*	87	380	466
LbrM12_V2.0210	*L. braziliensis*	44	656	699
LbrM12_V2.0210	*L. braziliensis*	68	911	978
LbrM12_V2.0210	*L. braziliensis*	49	1158	1206
LbrM12_V2.0210	*L. braziliensis*	52	1297	1348
LbrM12_V2.0210	*L. braziliensis*	132	1486	1617
LbrM12_V2.0220	*L. braziliensis*	191	1	191
LbrM12_V2.0220	*L. braziliensis*	312	223	534
LbrM12_V2.0220	*L. braziliensis*	557	543	1099
LbrM12_V2.0250	*L. braziliensis*	69	1	69
LbrM12_V2.0250	*L. braziliensis*	69	127	195
LbrM12_V2.0250	*L. braziliensis*	235	238	472
LbrM12_V2.0250	*L. braziliensis*	54	527	580
LbrM12_V2.0250	*L. braziliensis*	45	769	813
LbrM12_V2.0250	*L. braziliensis*	85	899	983
LbrM12_V2.0250	*L. braziliensis*	64	1014	1077
LbrM12_V2.0250	*L. braziliensis*	61	1107	1167
LbrM12_V2.0250	*L. braziliensis*	41	1253	1293
LbrM12_V2.0250	*L. braziliensis*	137	1329	1465
LbrM12_V2.0260	*L. braziliensis*	54	196	249
LbrM12_V2.0260	*L. braziliensis*	130	567	696
LbrM12_V2.0270	*L. braziliensis*	61	142	202
LbrM12_V2.0270	*L. braziliensis*	54	302	355
LbrM12_V2.0270	*L. braziliensis*	139	642	780
LbrM12_V2.0280	*L. braziliensis*	43	328	370
LbrM12_V2.0280	*L. braziliensis*	71	428	498
LbrM12_V2.0280	*L. braziliensis*	58	538	595
LbrM12_V2.0280	*L. braziliensis*	58	723	780
LbrM12_V2.0280	*L. braziliensis*	49	1094	1142
LbrM12_V2.0280	*L. braziliensis*	53	1387	1439
LbrM12_V2.0280	*L. braziliensis*	103	1459	1561
LbrM12_V2.0280	*L. braziliensis*	137	1593	1729
LbrM12_V2.0280	*L. braziliensis*	84	1734	1817
LbrM12_V2.0280	*L. braziliensis*	71	1859	1929
LbrM12_V2.0280	*L. braziliensis*	54	2020	2073

LbrM12_V2.0280	*L. braziliensis*	60	2077	2136
LbrM12_V2.0280	*L. braziliensis*	68	2141	2208
LbrM12_V2.0280	*L. braziliensis*	51	2443	2493
LbrM12_V2.0280	*L. braziliensis*	76	2525	2600
LbrM12_V2.0290	*L. braziliensis*	74	1	74
LbrM12_V2.0290	*L. braziliensis*	64	134	197
LbrM12_V2.0290	*L. braziliensis*	55	301	355
LbrM12_V2.0300	*L. braziliensis*	46	32	77
LbrM12_V2.0310	*L. braziliensis*	154	1	154
LbrM12_V2.0310	*L. braziliensis*	657	172	828
LbrM12_V2.0310	*L. braziliensis*	134	853	986
LbrM12_V2.0310	*L. braziliensis*	81	1034	1114
LbrM12_V2.0310	*L. braziliensis*	63	1256	1318
LbrM12_V2.0310	*L. braziliensis*	116	1334	1449
LbrM12_V2.0330	*L. braziliensis*	92	197	288
LbrM12_V2.0330	*L. braziliensis*	68	355	422
LbrM12_V2.0330	*L. braziliensis*	404	450	853
LbrM12_V2.0330	*L. braziliensis*	68	901	968
LbrM12_V2.0330	*L. braziliensis*	62	991	1052
LbrM12_V2.0330	*L. braziliensis*	65	1239	1303
LbrM12_V2.0340	*L. braziliensis*	95	117	211
LbrM12_V2.0340	*L. braziliensis*	46	339	384
LbrM12_V2.0340	*L. braziliensis*	74	560	633
LbrM12_V2.0350	*L. braziliensis*	128	112	239
LbrM12_V2.0350	*L. braziliensis*	122	302	423
LbrM12_V2.0350	*L. braziliensis*	52	452	503
LbrM12_V2.0350	*L. braziliensis*	41	518	558
LbrM12_V2.0350	*L. braziliensis*	348	717	1064
LbrM12_V2.0350	*L. braziliensis*	69	1084	1152
LbrM12_V2.0350	*L. braziliensis*	52	1197	1248
LbrM12_V2.0350	*L. braziliensis*	65	1276	1340
LbrM12_V2.0350	*L. braziliensis*	68	1382	1449
LbrM12_V2.0350	*L. braziliensis*	99	1477	1575
LbrM12_V2.0350	*L. braziliensis*	45	1604	1648
LbrM12_V2.0350	*L. braziliensis*	40	1828	1867
LbrM12_V2.0350	*L. braziliensis*	99	1974	2072
LbrM12_V2.0350	*L. braziliensis*	47	2157	2203
LbrM12_V2.0350	*L. braziliensis*	124	2276	2399
LbrM12_V2.0350	*L. braziliensis*	48	2404	2451
LbrM12_V2.0350	*L. braziliensis*	223	2535	2757
LbrM12_V2.0350	*L. braziliensis*	61	2777	2837
LbrM12_V2.0350	*L. braziliensis*	133	2908	3040
LbrM12_V2.0360	*L. braziliensis*	253	94	346
LbrM12_V2.0360	*L. braziliensis*	116	396	511
LbrM12_V2.0360	*L. braziliensis*	120	685	804
LbrM12_V2.0360	*L. braziliensis*	66	851	916
LbrM12_V2.0360	*L. braziliensis*	108	1096	1203

LbrM12_V2.0370	*L. braziliensis*	212	1	212
LbrM12_V2.0370	*L. braziliensis*	100	237	336
LbrM12_V2.0370	*L. braziliensis*	168	370	537
LbrM12_V2.0370	*L. braziliensis*	56	547	602
LbrM12_V2.0370	*L. braziliensis*	42	607	648
LbrM12_V2.0370	*L. braziliensis*	594	687	1280
LbrM12_V2.0370	*L. braziliensis*	87	1356	1442
LbrM12_V2.0370	*L. braziliensis*	116	1446	1561
LbrM12_V2.0370	*L. braziliensis*	262	1650	1911
LbrM12_V2.0370	*L. braziliensis*	120	1948	2067
LbrM12_V2.0380	*L. braziliensis*	78	223	300
LbrM12_V2.0390	*L. braziliensis*	52	151	202
LbrM12_V2.0390	*L. braziliensis*	81	222	302
LbrM12_V2.0400	*L. braziliensis*	249	1	249
LbrM12_V2.0400	*L. braziliensis*	122	275	396
LbrM12_V2.0400	*L. braziliensis*	162	404	565
LbrM12_V2.0400	*L. braziliensis*	336	591	926
LbrM12_V2.0400	*L. braziliensis*	66	961	1026
LbrM12_V2.0410	*L. braziliensis*	41	327	367
LbrM12_V2.0430	*L. braziliensis*	68	1	68
LbrM12_V2.0430	*L. braziliensis*	59	105	163
LbrM12_V2.0430	*L. braziliensis*	133	174	306
LbrM12_V2.0430	*L. braziliensis*	43	323	365
LbrM12_V2.0450	*L. braziliensis*	62	59	120
LbrM12_V2.0450	*L. braziliensis*	193	207	399
LbrM12_V2.0450	*L. braziliensis*	106	509	614
LbrM12_V2.0460	*L. braziliensis*	53	58	110
LbrM12_V2.0460	*L. braziliensis*	55	142	196
LbrM12_V2.0460	*L. braziliensis*	116	206	321
LbrM12_V2.0460	*L. braziliensis*	63	381	443
LbrM12_V2.0460	*L. braziliensis*	85	463	547
LbrM12_V2.0460	*L. braziliensis*	150	574	723
LbrM12_V2.0470	*L. braziliensis*	55	1	55
LbrM12_V2.0470	*L. braziliensis*	70	64	133
LbrM12_V2.0470	*L. braziliensis*	79	153	231
LbrM12_V2.0470	*L. braziliensis*	77	278	354
LbrM12_V2.0470	*L. braziliensis*	60	385	444
LbrM12_V2.0470	*L. braziliensis*	101	558	658
LbrM12_V2.0470	*L. braziliensis*	297	679	975
LbrM12_V2.0500	*L. braziliensis*	61	50	110
LbrM12_V2.0510	*L. braziliensis*	98	37	134
LbrM12_V2.0510	*L. braziliensis*	188	143	330
LbrM12_V2.0510	*L. braziliensis*	55	379	433
LbrM12_V2.0510	*L. braziliensis*	142	498	639
LbrM12_V2.0510	*L. braziliensis*	93	648	740
LbrM12_V2.0510	*L. braziliensis*	76	754	829
LbrM12_V2.0520	*L. braziliensis*	62	1	62

LbrM12_V2.0520	*L. braziliensis*	114	224	337
LbrM12_V2.0520	*L. braziliensis*	401	624	1024
LbrM12_V2.0520	*L. braziliensis*	45	1324	1368
LbrM12_V2.0530	*L. braziliensis*	332	62	393
LbrM12_V2.0530	*L. braziliensis*	355	420	774
LbrM12_V2.0530	*L. braziliensis*	77	895	971
LbrM12_V2.0540	*L. braziliensis*	111	53	163
LbrM12_V2.0540	*L. braziliensis*	201	298	498
LbrM12_V2.0540	*L. braziliensis*	93	539	631
LbrM12_V2.0540	*L. braziliensis*	49	667	715
LbrM12_V2.0540	*L. braziliensis*	132	830	961
LbrM12_V2.0540	*L. braziliensis*	90	974	1063
LbrM12_V2.0540	*L. braziliensis*	74	1103	1176
LbrM12_V2.0540	*L. braziliensis*	52	1334	1385
LbrM12_V2.0540	*L. braziliensis*	96	1393	1488
LbrM12_V2.0550	*L. braziliensis*	110	1	110
LbrM12_V2.0550	*L. braziliensis*	104	151	254
LbrM12_V2.0560	*L. braziliensis*	329	1	329
LbrM12_V2.0560	*L. braziliensis*	207	391	597
LbrM12_V2.0570	*L. braziliensis*	78	1	78
LbrM12_V2.0570	*L. braziliensis*	288	99	386
LbrM12_V2.0570	*L. braziliensis*	144	496	639
LbrM12_V2.0570	*L. braziliensis*	63	696	758
LbrM12_V2.0580	*L. braziliensis*	283	47	329
LbrM12_V2.0580	*L. braziliensis*	134	1091	1224
LbrM12_V2.0580	*L. braziliensis*	57	1246	1302
LbrM12_V2.0580	*L. braziliensis*	489	1306	1794
LbrM12_V2.0590	*L. braziliensis*	129	31	159
LbrM12_V2.0590	*L. braziliensis*	47	782	828
LbrM12_V2.0590	*L. braziliensis*	62	1005	1066
LbrM12_V2.0590	*L. braziliensis*	49	1147	1195
LbrM12_V2.0590	*L. braziliensis*	263	1225	1487
LbrM12_V2.0590	*L. braziliensis*	119	1492	1610
LbrM12_V2.0590	*L. braziliensis*	71	1658	1728
LbrM12_V2.0600	*L. braziliensis*	51	1	51
LbrM12_V2.0600	*L. braziliensis*	50	263	312
LbrM12_V2.0600	*L. braziliensis*	50	263	312
LbrM12_V2.0610	*L. braziliensis*	165	183	347
LbrM12_V2.0610	*L. braziliensis*	81	452	532
LbrM12_V2.0610	*L. braziliensis*	160	816	975
LbrM12_V2.0610	*L. braziliensis*	59	982	1040
LbrM12_V2.0620	*L. braziliensis*	642	43	684
LbrM12_V2.0620	*L. braziliensis*	70	689	758
LbrM12_V2.0620	*L. braziliensis*	77	844	920
LbrM12_V2.0620	*L. braziliensis*	400	942	1341
LbrM12_V2.0640	*L. braziliensis*	81	216	296
LbrM12_V2.0640	*L. braziliensis*	45	345	389

LbrM12_V2.0650	*L. braziliensis*	317	1	317
LbrM12_V2.0650	*L. braziliensis*	101	330	430
LbrM12_V2.0650	*L. braziliensis*	184	435	618
LbrM12_V2.0680	*L. braziliensis*	47	319	365
LbrM12_V2.0690	*L. braziliensis*	88	79	166
LbrM12_V2.0700	*L. braziliensis*	57	114	170
LbrM12_V2.0700	*L. braziliensis*	49	230	278
LbrM12_V2.0700	*L. braziliensis*	57	390	446
LbrM12_V2.0700	*L. braziliensis*	162	682	843
LbrM12_V2.0730	*L. braziliensis*	116	36	151
LbrM12_V2.0730	*L. braziliensis*	71	160	230
LbrM12_V2.0730	*L. braziliensis*	105	347	451
LbrM12_V2.0740	*L. braziliensis*	92	218	309
LbrM12_V2.0740	*L. braziliensis*	115	325	439
LbrM12_V2.0750	*L. braziliensis*	45	149	193
LbrM12_V2.0750	*L. braziliensis*	81	306	386
LbrM12_V2.0750	*L. braziliensis*	187	414	600
LbrM12_V2.0750	*L. braziliensis*	212	653	864
LbrM12_V2.0760	*L. braziliensis*	107	1	107
LbrM12_V2.0760	*L. braziliensis*	46	233	278
LbrM12_V2.0760	*L. braziliensis*	40	336	375
LbrM12_V2.0770	*L. braziliensis*	50	1	50
LbrM12_V2.0770	*L. braziliensis*	137	79	215
LbrM12_V2.0770	*L. braziliensis*	99	254	352
LbrM12_V2.0770	*L. braziliensis*	167	361	527
LbrM12_V2.0770	*L. braziliensis*	144	560	703
LbrM12_V2.0770	*L. braziliensis*	142	723	864
LbrM12_V2.0770	*L. braziliensis*	231	900	1130
LbrM12_V2.0770	*L. braziliensis*	58	1275	1332
LbrM12_V2.0770	*L. braziliensis*	170	1424	1593
LbrM12_V2.0780	*L. braziliensis*	63	32	94
LbrM12_V2.0780	*L. braziliensis*	417	151	567
LbrM12_V2.0780	*L. braziliensis*	103	627	729
LbrM12_V2.0790	*L. braziliensis*	309	148	456
LbrM12_V2.0790	*L. braziliensis*	149	459	607
LbrM12_V2.0790	*L. braziliensis*	296	734	1029
LbrM12_V2.0790	*L. braziliensis*	85	1033	1117
LbrM12_V2.0820	*L. braziliensis*	47	386	432
LbrM12_V2.0820	*L. braziliensis*	93	784	876
LbrM12_V2.0820	*L. braziliensis*	93	784	876
LbrM12_V2.0830	*L. braziliensis*	195	32	226
LbrM12_V2.0830	*L. braziliensis*	419	277	695
LbrM12_V2.0830	*L. braziliensis*	224	775	998
LbrM12_V2.0840	*L. braziliensis*	260	262	521
LbrM12_V2.0850	*L. braziliensis*	65	178	242
LbrM12_V2.0850	*L. braziliensis*	207	510	716
LbrM12_V2.0860	*L. braziliensis*	192	32	223

LbrM12_V2.0860	*L. braziliensis*	131	257	387
LbrM12_V2.0860	*L. braziliensis*	93	472	564
LbrM12_V2.0860	*L. braziliensis*	55	653	707
LbrM12_V2.0860	*L. braziliensis*	49	815	863
LbrM12_V2.0870	*L. braziliensis*	50	1	50
LbrM12_V2.0870	*L. braziliensis*	62	165	226
LbrM12_V2.0870	*L. braziliensis*	51	300	350
LbrM12_V2.0870	*L. braziliensis*	47	414	460
LbrM12_V2.0870	*L. braziliensis*	50	532	581
LbrM12_V2.0870	*L. braziliensis*	106	1153	1258
LbrM12_V2.0870	*L. braziliensis*	89	1346	1434
LbrM12_V2.0880	*L. braziliensis*	51	1	51
LbrM12_V2.0880	*L. braziliensis*	86	370	455
LbrM12_V2.0880	*L. braziliensis*	86	370	455
LbrM12_V2.0890	*L. braziliensis*	131	1	131
LbrM12_V2.0890	*L. braziliensis*	350	164	513
LbrM12_V2.0890	*L. braziliensis*	189	577	765
LbrM12_V2.0890	*L. braziliensis*	107	784	890
LbrM12_V2.0900	*L. braziliensis*	226	350	575
LbrM12_V2.0900	*L. braziliensis*	352	990	1341
LbrM12_V2.0900	*L. braziliensis*	45	1361	1405
LbrM12_V2.0920	*L. braziliensis*	91	1	91
LbrM12_V2.0920	*L. braziliensis*	40	215	254
LbrM12_V2.0920	*L. braziliensis*	85	328	412
LbrM12_V2.0920	*L. braziliensis*	75	551	625
LbrM12_V2.0930	*L. braziliensis*	57	666	722
LbrM12_V2.0930	*L. braziliensis*	178	901	1078
LbrM12_V2.0930	*L. braziliensis*	45	1259	1303
LbrM12_V2.0940	*L. braziliensis*	108	75	182
LbrM12_V2.0950	*L. braziliensis*	61	1	61
LbrM12_V2.0950	*L. braziliensis*	215	108	322
LbrM12_V2.0950	*L. braziliensis*	164	357	520
LbrM12_V2.0950	*L. braziliensis*	145	638	782
LbrM12_V2.0960	*L. braziliensis*	191	40	230
LbrM12_V2.0960	*L. braziliensis*	108	347	454
LbrM12_V2.0960	*L. braziliensis*	78	595	672
LbrM13_V2.0010	*L. braziliensis*	57	62	118
LbrM13_V2.0030	*L. braziliensis*	48	1	48
LbrM13_V2.0030	*L. braziliensis*	41	159	199
LbrM13_V2.0030	*L. braziliensis*	41	159	199
LbrM13_V2.0040	*L. braziliensis*	195	1	195
LbrM13_V2.0060	*L. braziliensis*	149	1	149
LbrM13_V2.0060	*L. braziliensis*	115	152	266
LbrM13_V2.0060	*L. braziliensis*	134	276	409
LbrM13_V2.0090	*L. braziliensis*	112	199	310
LbrM13_V2.0100	*L. braziliensis*	45	32	76
LbrM13_V2.0110	*L. braziliensis*	101	1	101

LbrM13_V2.0120	*L. braziliensis*	78	324	401
LbrM13_V2.0120	*L. braziliensis*	142	443	584
LbrM13_V2.0120	*L. braziliensis*	142	443	584
LbrM13_V2.0130	*L. braziliensis*	65	61	125
LbrM13_V2.0140	*L. braziliensis*	157	63	219
LbrM13_V2.0160	*L. braziliensis*	70	503	572
LbrM13_V2.0170	*L. braziliensis*	59	19	77
LbrM13_V2.0170	*L. braziliensis*	56	380	435
LbrM13_V2.0170	*L. braziliensis*	56	380	435
LbrM13_V2.0180	*L. braziliensis*	50	67	116
LbrM13_V2.0180	*L. braziliensis*	62	239	300
LbrM13_V2.0210	*L. braziliensis*	56	93	148
LbrM13_V2.0210	*L. braziliensis*	53	181	233
LbrM13_V2.0210	*L. braziliensis*	53	181	233
LbrM13_V2.0230	*L. braziliensis*	42	37	78
LbrM13_V2.0230	*L. braziliensis*	57	865	921
LbrM13_V2.0250	*L. braziliensis*	68	292	359
LbrM13_V2.0250	*L. braziliensis*	122	366	487
LbrM13_V2.0250	*L. braziliensis*	59	534	592
LbrM13_V2.0270	*L. braziliensis*	42	80	121
LbrM13_V2.0300	*L. braziliensis*	384	19	402
LbrM13_V2.0300	*L. braziliensis*	152	405	556
LbrM13_V2.0300	*L. braziliensis*	138	577	714
LbrM13_V2.0310	*L. braziliensis*	54	225	278
LbrM13_V2.0310	*L. braziliensis*	53	392	444
LbrM13_V2.0310	*L. braziliensis*	53	392	444
LbrM13_V2.0330	*L. braziliensis*	45	51	95
LbrM13_V2.0340	*L. braziliensis*	169	1	169
LbrM13_V2.0340	*L. braziliensis*	40	239	278
LbrM13_V2.0350	*L. braziliensis*	98	1	98
LbrM13_V2.0350	*L. braziliensis*	40	133	172
LbrM13_V2.0350	*L. braziliensis*	132	263	394
LbrM13_V2.0350	*L. braziliensis*	72	396	467
LbrM13_V2.0350	*L. braziliensis*	61	518	578
LbrM13_V2.0350	*L. braziliensis*	227	589	815
LbrM13_V2.0350	*L. braziliensis*	145	822	966
LbrM13_V2.0370	*L. braziliensis*	57	77	133
LbrM13_V2.0370	*L. braziliensis*	155	182	336
LbrM13_V2.0370	*L. braziliensis*	92	505	596
LbrM13_V2.0380	*L. braziliensis*	53	146	198
LbrM13_V2.0400	*L. braziliensis*	94	73	166
LbrM13_V2.0400	*L. braziliensis*	67	413	479
LbrM13_V2.0400	*L. braziliensis*	48	510	557
LbrM13_V2.0400	*L. braziliensis*	159	624	782
LbrM13_V2.0400	*L. braziliensis*	70	784	853
LbrM13_V2.0400	*L. braziliensis*	87	875	961
LbrM13_V2.0400	*L. braziliensis*	68	1121	1188

LbrM13_V2.0400	*L. braziliensis*	43	1227	1269
LbrM13_V2.0400	*L. braziliensis*	61	1316	1376
LbrM13_V2.0400	*L. braziliensis*	53	1403	1455
LbrM13_V2.0420	*L. braziliensis*	116	1	116
LbrM13_V2.0420	*L. braziliensis*	41	147	187
LbrM13_V2.0420	*L. braziliensis*	41	147	187
LbrM13_V2.0430	*L. braziliensis*	41	120	160
LbrM13_V2.0440	*L. braziliensis*	187	1	187
LbrM13_V2.0440	*L. braziliensis*	172	193	364
LbrM13_V2.0450	*L. braziliensis*	131	60	190
LbrM13_V2.0450	*L. braziliensis*	45	233	277
LbrM13_V2.0460	*L. braziliensis*	41	773	813
LbrM13_V2.0460	*L. braziliensis*	142	1115	1256
LbrM13_V2.0470	*L. braziliensis*	160	93	252
LbrM13_V2.0470	*L. braziliensis*	106	506	611
LbrM13_V2.0480	*L. braziliensis*	61	569	629
LbrM13_V2.0490	*L. braziliensis*	158	288	445
LbrM13_V2.0500	*L. braziliensis*	119	1	119
LbrM13_V2.0500	*L. braziliensis*	68	467	534
LbrM13_V2.0510	*L. braziliensis*	99	1	99
LbrM13_V2.0510	*L. braziliensis*	169	116	284
LbrM13_V2.0510	*L. braziliensis*	193	321	513
LbrM13_V2.0510	*L. braziliensis*	347	518	864
LbrM13_V2.0520	*L. braziliensis*	248	1	248
LbrM13_V2.0520	*L. braziliensis*	43	330	372
LbrM13_V2.0520	*L. braziliensis*	49	528	576
LbrM13_V2.0520	*L. braziliensis*	107	621	727
LbrM13_V2.0520	*L. braziliensis*	55	776	830
LbrM13_V2.0530	*L. braziliensis*	40	1	40
LbrM13_V2.0530	*L. braziliensis*	293	112	404
LbrM13_V2.0550	*L. braziliensis*	384	59	442
LbrM13_V2.0550	*L. braziliensis*	230	636	865
LbrM13_V2.0560	*L. braziliensis*	73	1	73
LbrM13_V2.0570	*L. braziliensis*	168	1	168
LbrM13_V2.0580	*L. braziliensis*	214	144	357
LbrM13_V2.0580	*L. braziliensis*	295	520	814
LbrM13_V2.0580	*L. braziliensis*	298	1088	1385
LbrM13_V2.0580	*L. braziliensis*	65	1460	1524
LbrM13_V2.0580	*L. braziliensis*	128	1604	1731
LbrM13_V2.0580	*L. braziliensis*	99	1798	1896
LbrM13_V2.0590	*L. braziliensis*	99	65	163
LbrM13_V2.0590	*L. braziliensis*	47	212	258
LbrM13_V2.0590	*L. braziliensis*	90	376	465
LbrM13_V2.0600	*L. braziliensis*	102	204	305
LbrM13_V2.0610	*L. braziliensis*	98	78	175
LbrM13_V2.0610	*L. braziliensis*	74	193	266
LbrM13_V2.0610	*L. braziliensis*	78	280	357

LbrM13_V2.0610	*L. braziliensis*	41	489	529
LbrM13_V2.0610	*L. braziliensis*	81	584	664
LbrM13_V2.0620	*L. braziliensis*	138	96	233
LbrM13_V2.0630	*L. braziliensis*	41	64	104
LbrM13_V2.0640	*L. braziliensis*	76	41	116
LbrM13_V2.0640	*L. braziliensis*	125	143	267
LbrM13_V2.0640	*L. braziliensis*	150	415	564
LbrM13_V2.0640	*L. braziliensis*	196	596	791
LbrM13_V2.0640	*L. braziliensis*	59	843	901
LbrM13_V2.0650	*L. braziliensis*	108	1	108
LbrM13_V2.0650	*L. braziliensis*	44	139	182
LbrM13_V2.0650	*L. braziliensis*	171	199	369
LbrM13_V2.0650	*L. braziliensis*	106	414	519
LbrM13_V2.0650	*L. braziliensis*	308	551	858
LbrM13_V2.0660	*L. braziliensis*	278	1	278
LbrM13_V2.0660	*L. braziliensis*	84	369	452
LbrM13_V2.0660	*L. braziliensis*	122	490	611
LbrM13_V2.0660	*L. braziliensis*	156	638	793
LbrM13_V2.0670	*L. braziliensis*	141	135	275
LbrM13_V2.0680	*L. braziliensis*	47	135	181
LbrM13_V2.0680	*L. braziliensis*	46	293	338
LbrM13_V2.0680	*L. braziliensis*	46	293	338
LbrM13_V2.0690	*L. braziliensis*	261	79	339
LbrM13_V2.0690	*L. braziliensis*	236	454	689
LbrM13_V2.0700	*L. braziliensis*	46	1	46
LbrM13_V2.0700	*L. braziliensis*	81	90	170
LbrM13_V2.0700	*L. braziliensis*	71	217	287
LbrM13_V2.0710	*L. braziliensis*	120	76	195
LbrM13_V2.0710	*L. braziliensis*	118	302	419
LbrM13_V2.0710	*L. braziliensis*	136	466	601
LbrM13_V2.0720	*L. braziliensis*	57	49	105
LbrM13_V2.0720	*L. braziliensis*	348	185	532
LbrM13_V2.0720	*L. braziliensis*	138	762	899
LbrM13_V2.0730	*L. braziliensis*	54	1	54
LbrM13_V2.0730	*L. braziliensis*	101	73	173
LbrM13_V2.0730	*L. braziliensis*	50	180	229
LbrM13_V2.0730	*L. braziliensis*	43	259	301
LbrM13_V2.0730	*L. braziliensis*	109	414	522
LbrM13_V2.0730	*L. braziliensis*	81	542	622
LbrM13_V2.0730	*L. braziliensis*	148	787	934
LbrM13_V2.0730	*L. braziliensis*	40	1003	1042
LbrM13_V2.0730	*L. braziliensis*	63	1065	1127
LbrM13_V2.0730	*L. braziliensis*	344	1230	1573
LbrM13_V2.0750	*L. braziliensis*	50	18	67
LbrM13_V2.0750	*L. braziliensis*	114	377	490
LbrM13_V2.0760	*L. braziliensis*	91	222	312
LbrM13_V2.0770	*L. braziliensis*	154	108	261

LbrM13_V2.0780	*L. braziliensis*	143	1	143
LbrM13_V2.0780	*L. braziliensis*	50	217	266
LbrM13_V2.0780	*L. braziliensis*	105	406	510
LbrM13_V2.0790	*L. braziliensis*	44	119	162
LbrM13_V2.0790	*L. braziliensis*	76	512	587
LbrM13_V2.0790	*L. braziliensis*	83	818	900
LbrM13_V2.0790	*L. braziliensis*	60	986	1045
LbrM13_V2.0790	*L. braziliensis*	65	1067	1131
LbrM13_V2.0790	*L. braziliensis*	47	1169	1215
LbrM13_V2.0790	*L. braziliensis*	42	1608	1649
LbrM13_V2.0800	*L. braziliensis*	365	1	365
LbrM13_V2.0800	*L. braziliensis*	60	381	440
LbrM13_V2.0810	*L. braziliensis*	69	1	69
LbrM13_V2.0810	*L. braziliensis*	68	133	200
LbrM13_V2.0810	*L. braziliensis*	125	228	352
LbrM13_V2.0830	*L. braziliensis*	52	131	182
LbrM13_V2.0830	*L. braziliensis*	46	463	508
LbrM13_V2.0830	*L. braziliensis*	45	593	637
LbrM13_V2.0860	*L. braziliensis*	48	297	344
LbrM13_V2.0860	*L. braziliensis*	62	546	607
LbrM13_V2.0860	*L. braziliensis*	176	713	888
LbrM13_V2.0860	*L. braziliensis*	46	1122	1167
LbrM13_V2.0860	*L. braziliensis*	43	1356	1398
LbrM13_V2.0860	*L. braziliensis*	43	1409	1451
LbrM13_V2.0890	*L. braziliensis*	40	1	40
LbrM13_V2.0900	*L. braziliensis*	51	102	152
LbrM13_V2.0910	*L. braziliensis*	292	45	336
LbrM13_V2.0910	*L. braziliensis*	152	342	493
LbrM13_V2.0930	*L. braziliensis*	54	89	142
LbrM13_V2.0930	*L. braziliensis*	93	241	333
LbrM13_V2.0930	*L. braziliensis*	93	241	333
LbrM13_V2.0940	*L. braziliensis*	48	172	219
LbrM13_V2.0970	*L. braziliensis*	157	1	157
LbrM13_V2.0980	*L. braziliensis*	75	1	75
LbrM13_V2.0990	*L. braziliensis*	101	230	330
LbrM13_V2.1010	*L. braziliensis*	64	153	216
LbrM13_V2.1010	*L. braziliensis*	77	273	349
LbrM13_V2.1010	*L. braziliensis*	47	497	543
LbrM13_V2.1020	*L. braziliensis*	76	70	145
LbrM13_V2.1030	*L. braziliensis*	73	127	199
LbrM13_V2.1040	*L. braziliensis*	63	41	103
LbrM13_V2.1040	*L. braziliensis*	42	105	146
LbrM13_V2.1040	*L. braziliensis*	44	263	306
LbrM13_V2.1040	*L. braziliensis*	95	485	579
LbrM13_V2.1040	*L. braziliensis*	52	669	720
LbrM13_V2.1040	*L. braziliensis*	99	751	849
LbrM13_V2.1060	*L. braziliensis*	232	1	232

LbrM13_V2.1060	*L. braziliensis*	234	278	511
LbrM13_V2.1060	*L. braziliensis*	56	527	582
LbrM13_V2.1060	*L. braziliensis*	46	613	658
LbrM13_V2.1060	*L. braziliensis*	81	854	934
LbrM13_V2.1060	*L. braziliensis*	40	961	1000
LbrM13_V2.1080	*L. braziliensis*	109	313	421
LbrM13_V2.1090	*L. braziliensis*	55	316	370
LbrM13_V2.1090	*L. braziliensis*	69	405	473
LbrM13_V2.1100	*L. braziliensis*	55	351	405
LbrM13_V2.1110	*L. braziliensis*	127	1	127
LbrM13_V2.1110	*L. braziliensis*	147	435	581
LbrM13_V2.1110	*L. braziliensis*	127	631	757
LbrM13_V2.1110	*L. braziliensis*	109	783	891
LbrM13_V2.1110	*L. braziliensis*	121	912	1032
LbrM13_V2.1110	*L. braziliensis*	259	1039	1297
LbrM13_V2.1120	*L. braziliensis*	92	47	138
LbrM13_V2.1120	*L. braziliensis*	119	200	318
LbrM13_V2.1120	*L. braziliensis*	104	591	694
LbrM13_V2.1120	*L. braziliensis*	65	750	814
LbrM13_V2.1120	*L. braziliensis*	50	859	908
LbrM13_V2.1120	*L. braziliensis*	41	992	1032
LbrM13_V2.1120	*L. braziliensis*	55	1064	1118
LbrM13_V2.1130	*L. braziliensis*	84	353	436
LbrM13_V2.1130	*L. braziliensis*	67	451	517
LbrM13_V2.1130	*L. braziliensis*	69	615	683
LbrM13_V2.1140	*L. braziliensis*	61	57	117
LbrM13_V2.1140	*L. braziliensis*	105	171	275
LbrM13_V2.1140	*L. braziliensis*	45	404	448
LbrM13_V2.1140	*L. braziliensis*	58	478	535
LbrM13_V2.1170	*L. braziliensis*	42	400	441
LbrM13_V2.1180	*L. braziliensis*	126	1	126
LbrM13_V2.1180	*L. braziliensis*	442	133	574
LbrM13_V2.1180	*L. braziliensis*	569	586	1154
LbrM13_V2.1180	*L. braziliensis*	148	1166	1313
LbrM13_V2.1190	*L. braziliensis*	71	11	81
LbrM13_V2.1190	*L. braziliensis*	61	137	197
LbrM13_V2.1190	*L. braziliensis*	61	137	197
LbrM13_V2.1200	*L. braziliensis*	68	16	83
LbrM13_V2.1200	*L. braziliensis*	173	290	462
LbrM13_V2.1200	*L. braziliensis*	130	499	628
LbrM13_V2.1210	*L. braziliensis*	228	41	268
LbrM13_V2.1210	*L. braziliensis*	68	309	376
LbrM13_V2.1210	*L. braziliensis*	53	771	823
LbrM13_V2.1210	*L. braziliensis*	67	825	891
LbrM13_V2.1210	*L. braziliensis*	66	1019	1084
LbrM13_V2.1210	*L. braziliensis*	48	1187	1234
LbrM13_V2.1220	*L. braziliensis*	61	1	61

LbrM13_V2.1230	*L. braziliensis*	62	1	62
LbrM13_V2.1230	*L. braziliensis*	57	270	326
LbrM13_V2.1230	*L. braziliensis*	49	731	779
LbrM13_V2.1230	*L. braziliensis*	87	1183	1269
LbrM13_V2.1230	*L. braziliensis*	72	1390	1461
LbrM13_V2.1230	*L. braziliensis*	44	1510	1553
LbrM13_V2.1230	*L. braziliensis*	57	1655	1711
LbrM13_V2.1230	*L. braziliensis*	89	1915	2003
LbrM13_V2.1230	*L. braziliensis*	78	2048	2125
LbrM13_V2.1230	*L. braziliensis*	52	2149	2200
LbrM13_V2.1230	*L. braziliensis*	45	2370	2414
LbrM13_V2.1230	*L. braziliensis*	57	2432	2488
LbrM13_V2.1230	*L. braziliensis*	49	2874	2922
LbrM13_V2.1230	*L. braziliensis*	214	3073	3286
LbrM13_V2.1230	*L. braziliensis*	51	3706	3756
LbrM13_V2.1230	*L. braziliensis*	116	4063	4178
LbrM13_V2.1230	*L. braziliensis*	45	4203	4247
LbrM13_V2.1230	*L. braziliensis*	46	4591	4636
LbrM13_V2.1230	*L. braziliensis*	44	4693	4736
LbrM13_V2.1230	*L. braziliensis*	44	5309	5352
LbrM13_V2.1250	*L. braziliensis*	43	605	647
LbrM13_V2.1250	*L. braziliensis*	126	684	809
LbrM13_V2.1260	*L. braziliensis*	373	1	373
LbrM13_V2.1260	*L. braziliensis*	260	390	649
LbrM13_V2.1260	*L. braziliensis*	194	651	844
LbrM13_V2.1260	*L. braziliensis*	41	886	926
LbrM13_V2.1270	*L. braziliensis*	177	1	177
LbrM13_V2.1290	*L. braziliensis*	275	1	275
LbrM13_V2.1290	*L. braziliensis*	41	586	626
LbrM13_V2.1290	*L. braziliensis*	41	586	626
LbrM13_V2.1300	*L. braziliensis*	40	14	53
LbrM13_V2.1310	*L. braziliensis*	535	1	535
LbrM13_V2.1310	*L. braziliensis*	123	572	694
LbrM13_V2.1320	*L. braziliensis*	176	1	176
LbrM13_V2.1320	*L. braziliensis*	324	203	526
LbrM13_V2.1320	*L. braziliensis*	213	593	805
LbrM13_V2.1320	*L. braziliensis*	91	821	911
LbrM13_V2.1320	*L. braziliensis*	113	925	1037
LbrM13_V2.1320	*L. braziliensis*	320	1072	1391
LbrM13_V2.1320	*L. braziliensis*	49	1417	1465
LbrM13_V2.1320	*L. braziliensis*	244	1471	1714
LbrM13_V2.1340	*L. braziliensis*	65	294	358
LbrM13_V2.1350	*L. braziliensis*	89	18	106
LbrM13_V2.1350	*L. braziliensis*	161	296	456
LbrM13_V2.1360	*L. braziliensis*	91	1	91
LbrM13_V2.1360	*L. braziliensis*	159	600	758
LbrM13_V2.1370	*L. braziliensis*	40	63	102

LbrM13_V2.1390	*L. braziliensis*	55	46	100
LbrM13_V2.1390	*L. braziliensis*	74	335	408
LbrM13_V2.1390	*L. braziliensis*	74	335	408
LbrM13_V2.1410	*L. braziliensis*	383	703	1085
LbrM13_V2.1430	*L. braziliensis*	65	1	65
LbrM13_V2.1430	*L. braziliensis*	57	90	146
LbrM13_V2.1430	*L. braziliensis*	62	178	239
LbrM13_V2.1450	*L. braziliensis*	642	1	642
LbrM13_V2.1460	*L. braziliensis*	60	334	393
LbrM13_V2.1460	*L. braziliensis*	62	428	489
LbrM13_V2.1460	*L. braziliensis*	72	507	578
LbrM13_V2.1460	*L. braziliensis*	61	609	669
LbrM13_V2.1460	*L. braziliensis*	361	713	1073
LbrM13_V2.1460	*L. braziliensis*	48	1099	1146
LbrM13_V2.1460	*L. braziliensis*	44	1184	1227
LbrM13_V2.1460	*L. braziliensis*	46	1265	1310
LbrM13_V2.1470	*L. braziliensis*	141	66	206
LbrM13_V2.1470	*L. braziliensis*	181	513	693
LbrM13_V2.1510	*L. braziliensis*	46	1	46
LbrM13_V2.1510	*L. braziliensis*	59	1179	1237
LbrM13_V2.1510	*L. braziliensis*	87	3147	3233
LbrM13_V2.1510	*L. braziliensis*	72	4075	4146
LbrM13_V2.1510	*L. braziliensis*	60	4379	4438
LbrM13_V2.1530	*L. braziliensis*	78	1	78
LbrM13_V2.1570	*L. braziliensis*	62	1	62
LbrM13_V2.1570	*L. braziliensis*	45	89	133
LbrM13_V2.1570	*L. braziliensis*	46	243	288
LbrM13_V2.1570	*L. braziliensis*	65	314	378
LbrM13_V2.1590	*L. braziliensis*	188	1	188
LbrM13_V2.1590	*L. braziliensis*	396	210	605
LbrM13_V2.1590	*L. braziliensis*	175	642	816
LbrM13_V2.1590	*L. braziliensis*	302	861	1162
LbrM13_V2.1590	*L. braziliensis*	125	1245	1369
LbrM13_V2.1590	*L. braziliensis*	379	1392	1770
LbrM13_V2.1590	*L. braziliensis*	110	1831	1940
LbrM13_V2.1590	*L. braziliensis*	40	1993	2032
LbrM13_V2.1590	*L. braziliensis*	41	2071	2111
LbrM13_V2.1590	*L. braziliensis*	48	2323	2370
LbrM13_V2.1590	*L. braziliensis*	74	2405	2478
LbrM13_V2.1590	*L. braziliensis*	58	2480	2537
LbrM14_V2.0030	*L. braziliensis*	144	115	258
LbrM14_V2.0040	*L. braziliensis*	87	168	254
LbrM14_V2.0050	*L. braziliensis*	54	219	272
LbrM14_V2.0080	*L. braziliensis*	59	1	59
LbrM14_V2.0090	*L. braziliensis*	66	83	148
LbrM14_V2.0090	*L. braziliensis*	141	208	348
LbrM14_V2.0100	*L. braziliensis*	83	1	83

LbrM14_V2.0100	*L. braziliensis*	44	325	368
LbrM14_V2.0100	*L. braziliensis*	59	389	447
LbrM14_V2.0110	*L. braziliensis*	59	380	438
LbrM14_V2.0110	*L. braziliensis*	66	460	525
LbrM14_V2.0120	*L. braziliensis*	132	1	132
LbrM14_V2.0120	*L. braziliensis*	145	194	338
LbrM14_V2.0140	*L. braziliensis*	70	57	126
LbrM14_V2.0140	*L. braziliensis*	53	187	239
LbrM14_V2.0150	*L. braziliensis*	50	75	124
LbrM14_V2.0160	*L. braziliensis*	58	55	112
LbrM14_V2.0170	*L. braziliensis*	59	1	59
LbrM14_V2.0170	*L. braziliensis*	51	64	114
LbrM14_V2.0170	*L. braziliensis*	54	415	468
LbrM14_V2.0200	*L. braziliensis*	173	20	192
LbrM14_V2.0210	*L. braziliensis*	43	54	96
LbrM14_V2.0210	*L. braziliensis*	62	158	219
LbrM14_V2.0210	*L. braziliensis*	71	224	294
LbrM14_V2.0210	*L. braziliensis*	49	345	393
LbrM14_V2.0220	*L. braziliensis*	71	157	227
LbrM14_V2.0230	*L. braziliensis*	103	151	253
LbrM14_V2.0230	*L. braziliensis*	46	318	363
LbrM14_V2.0230	*L. braziliensis*	96	432	527
LbrM14_V2.0230	*L. braziliensis*	128	560	687
LbrM14_V2.0230	*L. braziliensis*	83	822	904
LbrM14_V2.0230	*L. braziliensis*	64	1144	1207
LbrM14_V2.0230	*L. braziliensis*	288	1537	1824
LbrM14_V2.0260	*L. braziliensis*	46	472	517
LbrM14_V2.0260	*L. braziliensis*	56	661	716
LbrM14_V2.0260	*L. braziliensis*	56	661	716
LbrM14_V2.0280	*L. braziliensis*	137	827	963
LbrM14_V2.0290	*L. braziliensis*	56	53	108
LbrM14_V2.0300	*L. braziliensis*	94	141	234
LbrM14_V2.0310	*L. braziliensis*	63	223	285
LbrM14_V2.0320	*L. braziliensis*	62	1	62
LbrM14_V2.0320	*L. braziliensis*	99	654	752
LbrM14_V2.0320	*L. braziliensis*	99	654	752
LbrM14_V2.0330	*L. braziliensis*	208	1	208
LbrM14_V2.0340	*L. braziliensis*	348	1	348
LbrM14_V2.0340	*L. braziliensis*	66	361	426
LbrM14_V2.0340	*L. braziliensis*	58	439	496
LbrM14_V2.0340	*L. braziliensis*	72	510	581
LbrM14_V2.0340	*L. braziliensis*	41	606	646
LbrM14_V2.0340	*L. braziliensis*	77	683	759
LbrM14_V2.0360	*L. braziliensis*	245	16	260
LbrM14_V2.0360	*L. braziliensis*	57	403	459
LbrM14_V2.0360	*L. braziliensis*	42	594	635
LbrM14_V2.0360	*L. braziliensis*	62	676	737

LbrM14_V2.0360	*L. braziliensis*	253	775	1027
LbrM14_V2.0370	*L. braziliensis*	263	82	344
LbrM14_V2.0370	*L. braziliensis*	286	378	663
LbrM14_V2.0370	*L. braziliensis*	158	675	832
LbrM14_V2.0370	*L. braziliensis*	920	841	1760
LbrM14_V2.0370	*L. braziliensis*	631	1777	2407
LbrM14_V2.0370	*L. braziliensis*	52	2464	2515
LbrM14_V2.0380	*L. braziliensis*	81	22	102
LbrM14_V2.0410	*L. braziliensis*	162	1	162
LbrM14_V2.0420	*L. braziliensis*	343	1	343
LbrM14_V2.0420	*L. braziliensis*	100	565	664
LbrM14_V2.0430	*L. braziliensis*	170	173	342
LbrM14_V2.0430	*L. braziliensis*	493	351	843
LbrM14_V2.0450	*L. braziliensis*	63	231	293
LbrM14_V2.0460	*L. braziliensis*	123	38	160
LbrM14_V2.0460	*L. braziliensis*	64	264	327
LbrM14_V2.0470	*L. braziliensis*	42	1	42
LbrM14_V2.0510	*L. braziliensis*	63	1	63
LbrM14_V2.0520	*L. braziliensis*	147	358	504
LbrM14_V2.0530	*L. braziliensis*	59	1	59
LbrM14_V2.0540	*L. braziliensis*	178	312	489
LbrM14_V2.0550	*L. braziliensis*	98	389	486
LbrM14_V2.0550	*L. braziliensis*	59	917	975
LbrM14_V2.0570	*L. braziliensis*	41	368	408
LbrM14_V2.0570	*L. braziliensis*	72	607	678
LbrM14_V2.0570	*L. braziliensis*	120	680	799
LbrM14_V2.0570	*L. braziliensis*	115	802	916
LbrM14_V2.0570	*L. braziliensis*	210	966	1175
LbrM14_V2.0570	*L. braziliensis*	120	1180	1299
LbrM14_V2.0580	*L. braziliensis*	71	34	104
LbrM14_V2.0580	*L. braziliensis*	61	142	202
LbrM14_V2.0590	*L. braziliensis*	46	106	151
LbrM14_V2.0590	*L. braziliensis*	49	178	226
LbrM14_V2.0590	*L. braziliensis*	45	276	320
LbrM14_V2.0590	*L. braziliensis*	101	367	467
LbrM14_V2.0600	*L. braziliensis*	224	1	224
LbrM14_V2.0600	*L. braziliensis*	197	273	469
LbrM14_V2.0610	*L. braziliensis*	119	93	211
LbrM14_V2.0730	*L. braziliensis*	58	19	76
LbrM14_V2.0730	*L. braziliensis*	95	99	193
LbrM14_V2.0730	*L. braziliensis*	307	884	1190
LbrM14_V2.0730	*L. braziliensis*	71	1193	1263
LbrM14_V2.0730	*L. braziliensis*	463	1313	1775
LbrM14_V2.0750	*L. braziliensis*	267	98	364
LbrM14_V2.0770	*L. braziliensis*	120	1	120
LbrM14_V2.0770	*L. braziliensis*	208	144	351
LbrM14_V2.0770	*L. braziliensis*	57	386	442

LbrM14_V2.0770	*L. braziliensis*	60	576	635
LbrM14_V2.0770	*L. braziliensis*	66	637	702
LbrM14_V2.0770	*L. braziliensis*	84	722	805
LbrM14_V2.0770	*L. braziliensis*	94	930	1023
LbrM14_V2.0770	*L. braziliensis*	47	1044	1090
LbrM14_V2.0770	*L. braziliensis*	148	1158	1305
LbrM14_V2.0770	*L. braziliensis*	42	1400	1441
LbrM14_V2.0770	*L. braziliensis*	72	1497	1568
LbrM14_V2.0770	*L. braziliensis*	66	1688	1753
LbrM14_V2.0770	*L. braziliensis*	176	1783	1958
LbrM14_V2.0770	*L. braziliensis*	71	1963	2033
LbrM14_V2.0770	*L. braziliensis*	73	2044	2116
LbrM14_V2.0770	*L. braziliensis*	75	2179	2253
LbrM14_V2.0770	*L. braziliensis*	84	2317	2400
LbrM14_V2.0770	*L. braziliensis*	449	2403	2851
LbrM14_V2.0770	*L. braziliensis*	209	2861	3069
LbrM14_V2.0770	*L. braziliensis*	849	3086	3934
LbrM14_V2.0770	*L. braziliensis*	82	3969	4050
LbrM14_V2.0770	*L. braziliensis*	127	4063	4189
LbrM14_V2.0780	*L. braziliensis*	425	1	425
LbrM14_V2.0790	*L. braziliensis*	460	114	573
LbrM14_V2.0800	*L. braziliensis*	52	1	52
LbrM14_V2.0800	*L. braziliensis*	102	256	357
LbrM14_V2.0800	*L. braziliensis*	75	451	525
LbrM14_V2.0810	*L. braziliensis*	195	11	205
LbrM14_V2.0810	*L. braziliensis*	93	270	362
LbrM14_V2.0810	*L. braziliensis*	56	404	459
LbrM14_V2.0810	*L. braziliensis*	42	816	857
LbrM14_V2.0810	*L. braziliensis*	114	1051	1164
LbrM14_V2.0810	*L. braziliensis*	85	1174	1258
LbrM14_V2.0820	*L. braziliensis*	53	63	115
LbrM14_V2.0830	*L. braziliensis*	53	63	115
LbrM14_V2.0850	*L. braziliensis*	50	70	119
LbrM14_V2.0850	*L. braziliensis*	79	148	226
LbrM14_V2.0850	*L. braziliensis*	243	296	538
LbrM14_V2.0850	*L. braziliensis*	64	568	631
LbrM14_V2.0860	*L. braziliensis*	70	150	219
LbrM14_V2.0860	*L. braziliensis*	265	257	521
LbrM14_V2.0860	*L. braziliensis*	88	544	631
LbrM14_V2.0870	*L. braziliensis*	45	1	45
LbrM14_V2.0870	*L. braziliensis*	440	62	501
LbrM14_V2.0870	*L. braziliensis*	58	884	941
LbrM14_V2.0880	*L. braziliensis*	41	212	252
LbrM14_V2.0890	*L. braziliensis*	127	1	127
LbrM14_V2.0890	*L. braziliensis*	90	495	584
LbrM14_V2.0890	*L. braziliensis*	43	1032	1074
LbrM14_V2.0890	*L. braziliensis*	45	1198	1242

LbrM14_V2.0900	*L. braziliensis*	44	165	208
LbrM14_V2.0900	*L. braziliensis*	85	371	455
LbrM14_V2.0900	*L. braziliensis*	85	371	455
LbrM14_V2.0910	*L. braziliensis*	132	1	132
LbrM14_V2.0910	*L. braziliensis*	446	135	580
LbrM14_V2.0910	*L. braziliensis*	45	647	691
LbrM14_V2.0910	*L. braziliensis*	40	855	894
LbrM14_V2.0930	*L. braziliensis*	212	1	212
LbrM14_V2.0930	*L. braziliensis*	58	398	455
LbrM14_V2.0930	*L. braziliensis*	56	710	765
LbrM14_V2.0930	*L. braziliensis*	40	818	857
LbrM14_V2.0930	*L. braziliensis*	50	945	994
LbrM14_V2.0930	*L. braziliensis*	69	1039	1107
LbrM14_V2.0940	*L. braziliensis*	266	110	375
LbrM14_V2.0950	*L. braziliensis*	40	211	250
LbrM14_V2.0960	*L. braziliensis*	96	1	96
LbrM14_V2.0960	*L. braziliensis*	50	110	159
LbrM14_V2.0960	*L. braziliensis*	60	726	785
LbrM14_V2.0980	*L. braziliensis*	59	1	59
LbrM14_V2.0990	*L. braziliensis*	166	31	196
LbrM14_V2.0990	*L. braziliensis*	98	450	547
LbrM14_V2.0990	*L. braziliensis*	47	838	884
LbrM14_V2.1000	*L. braziliensis*	95	62	156
LbrM14_V2.1000	*L. braziliensis*	44	186	229
LbrM14_V2.1000	*L. braziliensis*	44	186	229
LbrM14_V2.1050	*L. braziliensis*	96	1531	1626
LbrM14_V2.1050	*L. braziliensis*	51	1848	1898
LbrM14_V2.1050	*L. braziliensis*	111	2624	2734
LbrM14_V2.1050	*L. braziliensis*	49	2844	2892
LbrM14_V2.1050	*L. braziliensis*	65	3282	3346
LbrM14_V2.1050	*L. braziliensis*	75	3827	3901
LbrM14_V2.1050	*L. braziliensis*	92	3910	4001
LbrM14_V2.1060	*L. braziliensis*	348	1	348
LbrM14_V2.1060	*L. braziliensis*	64	369	432
LbrM14_V2.1060	*L. braziliensis*	49	499	547
LbrM14_V2.1060	*L. braziliensis*	77	609	685
LbrM14_V2.1060	*L. braziliensis*	61	923	983
LbrM14_V2.1070	*L. braziliensis*	66	64	129
LbrM14_V2.1070	*L. braziliensis*	44	169	212
LbrM14_V2.1070	*L. braziliensis*	40	277	316
LbrM14_V2.1100	*L. braziliensis*	71	1	71
LbrM14_V2.1100	*L. braziliensis*	63	258	320
LbrM14_V2.1100	*L. braziliensis*	228	409	636
LbrM14_V2.1100	*L. braziliensis*	46	688	733
LbrM14_V2.1100	*L. braziliensis*	1318	742	2059
LbrM14_V2.1120	*L. braziliensis*	63	113	175
LbrM14_V2.1120	*L. braziliensis*	165	264	428

LbrM14_V2.1120	*L. braziliensis*	114	482	595
LbrM14_V2.1120	*L. braziliensis*	51	597	647
LbrM14_V2.1120	*L. braziliensis*	1113	739	1851
LbrM14_V2.1120	*L. braziliensis*	89	1878	1966
LbrM14_V2.1130	*L. braziliensis*	139	1	139
LbrM14_V2.1130	*L. braziliensis*	77	255	331
LbrM14_V2.1130	*L. braziliensis*	86	584	669
LbrM14_V2.1140	*L. braziliensis*	57	158	214
LbrM14_V2.1140	*L. braziliensis*	42	296	337
LbrM14_V2.1140	*L. braziliensis*	48	437	484
LbrM14_V2.1150	*L. braziliensis*	60	21	80
LbrM14_V2.1150	*L. braziliensis*	77	240	316
LbrM14_V2.1160	*L. braziliensis*	133	119	251
LbrM14_V2.1170	*L. braziliensis*	53	1	53
LbrM14_V2.1170	*L. braziliensis*	270	597	866
LbrM14_V2.1170	*L. braziliensis*	145	934	1078
LbrM14_V2.1180	*L. braziliensis*	115	29	143
LbrM14_V2.1190	*L. braziliensis*	153	1	153
LbrM14_V2.1200	*L. braziliensis*	88	1	88
LbrM14_V2.1200	*L. braziliensis*	330	188	517
LbrM14_V2.1210	*L. braziliensis*	67	1	67
LbrM14_V2.1210	*L. braziliensis*	47	134	180
LbrM14_V2.1210	*L. braziliensis*	74	194	267
LbrM14_V2.1210	*L. braziliensis*	56	635	690
LbrM14_V2.1230	*L. braziliensis*	348	19	366
LbrM14_V2.1230	*L. braziliensis*	55	437	491
LbrM14_V2.1230	*L. braziliensis*	230	510	739
LbrM14_V2.1230	*L. braziliensis*	163	781	943
LbrM14_V2.1230	*L. braziliensis*	62	960	1021
LbrM14_V2.1240	*L. braziliensis*	59	1	59
LbrM14_V2.1240	*L. braziliensis*	58	325	382
LbrM14_V2.1240	*L. braziliensis*	58	325	382
LbrM14_V2.1250	*L. braziliensis*	69	29	97
LbrM14_V2.1260	*L. braziliensis*	186	1	186
LbrM14_V2.1260	*L. braziliensis*	76	215	290
LbrM14_V2.1260	*L. braziliensis*	113	476	588
LbrM14_V2.1260	*L. braziliensis*	55	623	677
LbrM14_V2.1270	*L. braziliensis*	133	203	335
LbrM14_V2.1270	*L. braziliensis*	100	363	462
LbrM14_V2.1270	*L. braziliensis*	117	609	725
LbrM14_V2.1270	*L. braziliensis*	60	764	823
LbrM14_V2.1270	*L. braziliensis*	40	891	930
LbrM14_V2.1290	*L. braziliensis*	42	170	211
LbrM14_V2.1320	*L. braziliensis*	65	66	130
LbrM14_V2.1340	*L. braziliensis*	69	40	108
LbrM14_V2.1340	*L. braziliensis*	145	174	318
LbrM14_V2.1340	*L. braziliensis*	48	370	417

LbrM14_V2.1340	*L. braziliensis*	161	591	751
LbrM14_V2.1340	*L. braziliensis*	58	789	846
LbrM14_V2.1350	*L. braziliensis*	60	432	491
LbrM14_V2.1350	*L. braziliensis*	146	564	709
LbrM14_V2.1380	*L. braziliensis*	77	80	156
LbrM14_V2.1390	*L. braziliensis*	62	41	102
LbrM14_V2.1390	*L. braziliensis*	68	139	206
LbrM14_V2.1390	*L. braziliensis*	137	234	370
LbrM14_V2.1390	*L. braziliensis*	86	455	540
LbrM14_V2.1390	*L. braziliensis*	134	597	730
LbrM14_V2.1390	*L. braziliensis*	95	848	942
LbrM14_V2.1400	*L. braziliensis*	52	303	354
LbrM14_V2.1400	*L. braziliensis*	57	402	458
LbrM14_V2.1410	*L. braziliensis*	66	122	187
LbrM14_V2.1410	*L. braziliensis*	42	245	286
LbrM14_V2.1410	*L. braziliensis*	42	245	286
LbrM14_V2.1430	*L. braziliensis*	47	1	47
LbrM14_V2.1440	*L. braziliensis*	57	66	122
LbrM14_V2.1450	*L. braziliensis*	41	68	108
LbrM14_V2.1450	*L. braziliensis*	58	282	339
LbrM14_V2.1450	*L. braziliensis*	51	351	401
LbrM14_V2.1450	*L. braziliensis*	44	450	493
LbrM14_V2.1460	*L. braziliensis*	93	1	93
LbrM14_V2.1460	*L. braziliensis*	50	170	219
LbrM14_V2.1460	*L. braziliensis*	80	253	332
LbrM14_V2.1460	*L. braziliensis*	65	352	416
LbrM14_V2.1460	*L. braziliensis*	371	436	806
LbrM14_V2.1470	*L. braziliensis*	57	1	57
LbrM14_V2.1470	*L. braziliensis*	50	105	154
LbrM14_V2.1470	*L. braziliensis*	329	385	713
LbrM14_V2.1480	*L. braziliensis*	225	19	243
LbrM14_V2.1480	*L. braziliensis*	42	268	309
LbrM14_V2.1480	*L. braziliensis*	42	268	309
LbrM14_V2.1500	*L. braziliensis*	171	345	515
LbrM14_V2.1520	*L. braziliensis*	47	1	47
LbrM14_V2.1520	*L. braziliensis*	101	93	193
LbrM14_V2.1520	*L. braziliensis*	77	243	319
LbrM14_V2.1520	*L. braziliensis*	46	463	508
LbrM14_V2.1570	*L. braziliensis*	69	29	97
LbrM14_V2.1570	*L. braziliensis*	61	356	416
LbrM14_V2.1570	*L. braziliensis*	61	356	416
LbrM14_V2.1600	*L. braziliensis*	256	294	549
LbrM14_V2.1620	*L. braziliensis*	125	777	901
LbrM14_V2.1620	*L. braziliensis*	106	942	1047
LbrM14_V2.1620	*L. braziliensis*	155	1150	1304
LbrM14_V2.1620	*L. braziliensis*	114	1408	1521
LbrM14_V2.1630	*L. braziliensis*	125	1	125

LbrM14_V2.1630	*L. braziliensis*	361	558	918
LbrM14_V2.1660	*L. braziliensis*	154	33	186
LbrM14_V2.1660	*L. braziliensis*	68	237	304
LbrM14_V2.1660	*L. braziliensis*	58	343	400
LbrM14_V2.1680	*L. braziliensis*	45	728	772
LbrM14_V2.1690	*L. braziliensis*	1423	1	1423
LbrM15_V2.0010	*L. braziliensis*	42	1	42
LbrM15_V2.0050	*L. braziliensis*	42	234	275
LbrM15_V2.0060	*L. braziliensis*	108	1	108
LbrM15_V2.0060	*L. braziliensis*	69	262	330
LbrM15_V2.0060	*L. braziliensis*	168	430	597
LbrM15_V2.0070	*L. braziliensis*	142	117	258
LbrM15_V2.0070	*L. braziliensis*	135	333	467
LbrM15_V2.0080	*L. braziliensis*	40	64	103
LbrM15_V2.0080	*L. braziliensis*	57	205	261
LbrM15_V2.0080	*L. braziliensis*	45	277	321
LbrM15_V2.0090	*L. braziliensis*	43	1	43
LbrM15_V2.0090	*L. braziliensis*	44	139	182
LbrM15_V2.0090	*L. braziliensis*	45	188	232
LbrM15_V2.0110	*L. braziliensis*	40	75	114
LbrM15_V2.0110	*L. braziliensis*	58	201	258
LbrM15_V2.0110	*L. braziliensis*	61	322	382
LbrM15_V2.0110	*L. braziliensis*	55	656	710
LbrM15_V2.0130	*L. braziliensis*	65	273	337
LbrM15_V2.0130	*L. braziliensis*	171	513	683
LbrM15_V2.0140	*L. braziliensis*	65	68	132
LbrM15_V2.0150	*L. braziliensis*	222	1	222
LbrM15_V2.0150	*L. braziliensis*	89	250	338
LbrM15_V2.0150	*L. braziliensis*	167	352	518
LbrM15_V2.0150	*L. braziliensis*	53	559	611
LbrM15_V2.0150	*L. braziliensis*	52	638	689
LbrM15_V2.0160	*L. braziliensis*	102	1	102
LbrM15_V2.0170	*L. braziliensis*	331	25	355
LbrM15_V2.0170	*L. braziliensis*	71	726	796
LbrM15_V2.0180	*L. braziliensis*	317	1	317
LbrM15_V2.0180	*L. braziliensis*	119	348	466
LbrM15_V2.0180	*L. braziliensis*	176	493	668
LbrM15_V2.0200	*L. braziliensis*	159	1	159
LbrM15_V2.0210	*L. braziliensis*	46	177	222
LbrM15_V2.0220	*L. braziliensis*	78	203	280
LbrM15_V2.0220	*L. braziliensis*	148	317	464
LbrM15_V2.0240	*L. braziliensis*	52	218	269
LbrM15_V2.0280	*L. braziliensis*	185	1	185
LbrM15_V2.0280	*L. braziliensis*	114	248	361
LbrM15_V2.0280	*L. braziliensis*	75	405	479
LbrM15_V2.0280	*L. braziliensis*	85	493	577
LbrM15_V2.0280	*L. braziliensis*	76	658	733

LbrM15_V2.0280	*L. braziliensis*	54	812	865
LbrM15_V2.0280	*L. braziliensis*	75	900	974
LbrM15_V2.0280	*L. braziliensis*	49	1326	1374
LbrM15_V2.0280	*L. braziliensis*	49	1539	1587
LbrM15_V2.0280	*L. braziliensis*	70	1643	1712
LbrM15_V2.0290	*L. braziliensis*	181	42	222
LbrM15_V2.0290	*L. braziliensis*	41	269	309
LbrM15_V2.0290	*L. braziliensis*	80	391	470
LbrM15_V2.0310	*L. braziliensis*	42	146	187
LbrM15_V2.0320	*L. braziliensis*	42	146	187
LbrM15_V2.0330	*L. braziliensis*	53	64	116
LbrM15_V2.0330	*L. braziliensis*	216	579	794
LbrM15_V2.0350	*L. braziliensis*	67	1	67
LbrM15_V2.0350	*L. braziliensis*	137	132	268
LbrM15_V2.0350	*L. braziliensis*	232	351	582
LbrM15_V2.0360	*L. braziliensis*	52	101	152
LbrM15_V2.0360	*L. braziliensis*	90	167	256
LbrM15_V2.0360	*L. braziliensis*	47	270	316
LbrM15_V2.0360	*L. braziliensis*	71	620	690
LbrM15_V2.0370	*L. braziliensis*	328	90	417
LbrM15_V2.0370	*L. braziliensis*	87	451	537
LbrM15_V2.0370	*L. braziliensis*	93	781	873
LbrM15_V2.0370	*L. braziliensis*	52	893	944
LbrM15_V2.0390	*L. braziliensis*	42	1	42
LbrM15_V2.0390	*L. braziliensis*	61	269	329
LbrM15_V2.0400	*L. braziliensis*	51	1	51
LbrM15_V2.0400	*L. braziliensis*	56	206	261
LbrM15_V2.0400	*L. braziliensis*	45	276	320
LbrM15_V2.0400	*L. braziliensis*	73	507	579
LbrM15_V2.0400	*L. braziliensis*	40	615	654
LbrM15_V2.0400	*L. braziliensis*	92	755	846
LbrM15_V2.0400	*L. braziliensis*	59	905	963
LbrM15_V2.0420	*L. braziliensis*	55	125	179
LbrM15_V2.0420	*L. braziliensis*	81	225	305
LbrM15_V2.0420	*L. braziliensis*	81	225	305
LbrM15_V2.0430	*L. braziliensis*	58	406	463
LbrM15_V2.0450	*L. braziliensis*	104	163	266
LbrM15_V2.0460	*L. braziliensis*	147	1	147
LbrM15_V2.0460	*L. braziliensis*	63	169	231
LbrM15_V2.0460	*L. braziliensis*	328	281	608
LbrM15_V2.0460	*L. braziliensis*	70	621	690
LbrM15_V2.0460	*L. braziliensis*	1428	705	2132
LbrM15_V2.0470	*L. braziliensis*	82	1	82
LbrM15_V2.0470	*L. braziliensis*	198	185	382
LbrM15_V2.0470	*L. braziliensis*	200	413	612
LbrM15_V2.0470	*L. braziliensis*	99	642	740
LbrM15_V2.0470	*L. braziliensis*	52	843	894

LbrM15_V2.0480	*L. braziliensis*	56	34	89
LbrM15_V2.0480	*L. braziliensis*	85	329	413
LbrM15_V2.0480	*L. braziliensis*	69	529	597
LbrM15_V2.0480	*L. braziliensis*	110	922	1031
LbrM15_V2.0480	*L. braziliensis*	74	1053	1126
LbrM15_V2.0480	*L. braziliensis*	384	1130	1513
LbrM15_V2.0480	*L. braziliensis*	61	1573	1633
LbrM15_V2.0480	*L. braziliensis*	48	1735	1782
LbrM15_V2.0480	*L. braziliensis*	151	1931	2081
LbrM15_V2.0490	*L. braziliensis*	216	1	216
LbrM15_V2.0490	*L. braziliensis*	143	232	374
LbrM15_V2.0490	*L. braziliensis*	58	446	503
LbrM15_V2.0490	*L. braziliensis*	42	528	569
LbrM15_V2.0500	*L. braziliensis*	360	166	525
LbrM15_V2.0500	*L. braziliensis*	101	544	644
LbrM15_V2.0500	*L. braziliensis*	80	821	900
LbrM15_V2.0510	*L. braziliensis*	109	32	140
LbrM15_V2.0510	*L. braziliensis*	84	144	227
LbrM15_V2.0510	*L. braziliensis*	95	264	358
LbrM15_V2.0510	*L. braziliensis*	101	769	869
LbrM15_V2.0510	*L. braziliensis*	92	916	1007
LbrM15_V2.0510	*L. braziliensis*	84	1073	1156
LbrM15_V2.0510	*L. braziliensis*	185	1158	1342
LbrM15_V2.0530	*L. braziliensis*	112	1	112
LbrM15_V2.0530	*L. braziliensis*	54	119	172
LbrM15_V2.0530	*L. braziliensis*	56	212	267
LbrM15_V2.0530	*L. braziliensis*	181	280	460
LbrM15_V2.0550	*L. braziliensis*	177	133	309
LbrM15_V2.0560	*L. braziliensis*	589	1	589
LbrM15_V2.0560	*L. braziliensis*	66	671	736
LbrM15_V2.0560	*L. braziliensis*	241	785	1025
LbrM15_V2.0560	*L. braziliensis*	311	1083	1393
LbrM15_V2.0560	*L. braziliensis*	41	1736	1776
LbrM15_V2.0560	*L. braziliensis*	50	1810	1859
LbrM15_V2.0570	*L. braziliensis*	54	1	54
LbrM15_V2.0570	*L. braziliensis*	114	189	302
LbrM15_V2.0570	*L. braziliensis*	137	321	457
LbrM15_V2.0570	*L. braziliensis*	81	549	629
LbrM15_V2.0570	*L. braziliensis*	47	632	678
LbrM15_V2.0580	*L. braziliensis*	80	1	80
LbrM15_V2.0580	*L. braziliensis*	63	122	184
LbrM15_V2.0580	*L. braziliensis*	80	479	558
LbrM15_V2.0580	*L. braziliensis*	41	561	601
LbrM15_V2.0580	*L. braziliensis*	223	619	841
LbrM15_V2.0580	*L. braziliensis*	95	888	982
LbrM15_V2.0580	*L. braziliensis*	58	1004	1061
LbrM15_V2.0580	*L. braziliensis*	127	1337	1463

LbrM15_V2.0580	*L. braziliensis*	43	1619	1661
LbrM15_V2.0590	*L. braziliensis*	55	1	55
LbrM15_V2.0590	*L. braziliensis*	71	89	159
LbrM15_V2.0590	*L. braziliensis*	66	179	244
LbrM15_V2.0590	*L. braziliensis*	105	290	394
LbrM15_V2.0590	*L. braziliensis*	128	400	527
LbrM15_V2.0600	*L. braziliensis*	76	103	178
LbrM15_V2.0610	*L. braziliensis*	54	64	117
LbrM15_V2.0610	*L. braziliensis*	69	186	254
LbrM15_V2.0610	*L. braziliensis*	69	186	254
LbrM15_V2.0620	*L. braziliensis*	177	47	223
LbrM15_V2.0630	*L. braziliensis*	425	1	425
LbrM15_V2.0630	*L. braziliensis*	55	430	484
LbrM15_V2.0630	*L. braziliensis*	135	604	738
LbrM15_V2.0630	*L. braziliensis*	323	978	1300
LbrM15_V2.0630	*L. braziliensis*	93	1302	1394
LbrM15_V2.0630	*L. braziliensis*	160	1460	1619
LbrM15_V2.0640	*L. braziliensis*	110	15	124
LbrM15_V2.0640	*L. braziliensis*	343	152	494
LbrM15_V2.0640	*L. braziliensis*	460	659	1118
LbrM15_V2.0650	*L. braziliensis*	45	373	417
LbrM15_V2.0660	*L. braziliensis*	49	1	49
LbrM15_V2.0660	*L. braziliensis*	93	259	351
LbrM15_V2.0670	*L. braziliensis*	224	1	224
LbrM15_V2.0670	*L. braziliensis*	253	239	491
LbrM15_V2.0670	*L. braziliensis*	85	528	612
LbrM15_V2.0670	*L. braziliensis*	79	641	719
LbrM15_V2.0670	*L. braziliensis*	145	787	931
LbrM15_V2.0680	*L. braziliensis*	110	1	110
LbrM15_V2.0680	*L. braziliensis*	71	121	191
LbrM15_V2.0680	*L. braziliensis*	43	226	268
LbrM15_V2.0680	*L. braziliensis*	43	342	384
LbrM15_V2.0680	*L. braziliensis*	82	784	865
LbrM15_V2.0690	*L. braziliensis*	53	1	53
LbrM15_V2.0690	*L. braziliensis*	43	159	201
LbrM15_V2.0690	*L. braziliensis*	43	159	201
LbrM15_V2.0710	*L. braziliensis*	72	266	337
LbrM15_V2.0710	*L. braziliensis*	107	381	487
LbrM15_V2.0710	*L. braziliensis*	82	600	681
LbrM15_V2.0710	*L. braziliensis*	65	760	824
LbrM15_V2.0710	*L. braziliensis*	75	952	1026
LbrM15_V2.0720	*L. braziliensis*	132	15	146
LbrM15_V2.0720	*L. braziliensis*	119	240	358
LbrM15_V2.0720	*L. braziliensis*	52	418	469
LbrM15_V2.0720	*L. braziliensis*	59	542	600
LbrM15_V2.0720	*L. braziliensis*	77	952	1028
LbrM15_V2.0720	*L. braziliensis*	57	1081	1137

LbrM15_V2.0720	*L. braziliensis*	45	1207	1251
LbrM15_V2.0720	*L. braziliensis*	71	1462	1532
LbrM15_V2.0720	*L. braziliensis*	45	1564	1608
LbrM15_V2.0720	*L. braziliensis*	92	1687	1778
LbrM15_V2.0720	*L. braziliensis*	46	1926	1971
LbrM15_V2.0720	*L. braziliensis*	52	2098	2149
LbrM15_V2.0720	*L. braziliensis*	126	2278	2403
LbrM15_V2.0720	*L. braziliensis*	80	2421	2500
LbrM15_V2.0720	*L. braziliensis*	61	2546	2606
LbrM15_V2.0730	*L. braziliensis*	91	17	107
LbrM15_V2.0740	*L. braziliensis*	46	335	380
LbrM15_V2.0750	*L. braziliensis*	274	1	274
LbrM15_V2.0750	*L. braziliensis*	130	297	426
LbrM15_V2.0750	*L. braziliensis*	136	466	601
LbrM15_V2.0750	*L. braziliensis*	197	645	841
LbrM15_V2.0750	*L. braziliensis*	326	848	1173
LbrM15_V2.0750	*L. braziliensis*	63	1215	1277
LbrM15_V2.0750	*L. braziliensis*	49	1286	1334
LbrM15_V2.0750	*L. braziliensis*	270	1362	1631
LbrM15_V2.0750	*L. braziliensis*	97	1675	1771
LbrM15_V2.0750	*L. braziliensis*	118	1846	1963
LbrM15_V2.0750	*L. braziliensis*	73	1979	2051
LbrM15_V2.0760	*L. braziliensis*	45	350	394
LbrM15_V2.0760	*L. braziliensis*	42	622	663
LbrM15_V2.0760	*L. braziliensis*	93	1555	1647
LbrM15_V2.0760	*L. braziliensis*	48	1731	1778
LbrM15_V2.0760	*L. braziliensis*	228	1859	2086
LbrM15_V2.0770	*L. braziliensis*	81	575	655
LbrM15_V2.0780	*L. braziliensis*	70	16	85
LbrM15_V2.0780	*L. braziliensis*	267	94	360
LbrM15_V2.0790	*L. braziliensis*	242	433	674
LbrM15_V2.0810	*L. braziliensis*	135	1	135
LbrM15_V2.0810	*L. braziliensis*	156	163	318
LbrM15_V2.0810	*L. braziliensis*	136	345	480
LbrM15_V2.0810	*L. braziliensis*	75	571	645
LbrM15_V2.0820	*L. braziliensis*	163	1009	1171
LbrM15_V2.0820	*L. braziliensis*	42	1699	1740
LbrM15_V2.0820	*L. braziliensis*	60	1762	1821
LbrM15_V2.0820	*L. braziliensis*	114	1835	1948
LbrM15_V2.0820	*L. braziliensis*	66	2187	2252
LbrM15_V2.0820	*L. braziliensis*	42	2298	2339
LbrM15_V2.0820	*L. braziliensis*	126	2381	2506
LbrM15_V2.0830	*L. braziliensis*	59	1	59
LbrM15_V2.0850	*L. braziliensis*	44	727	770
LbrM15_V2.0850	*L. braziliensis*	40	933	972
LbrM15_V2.0850	*L. braziliensis*	40	933	972
LbrM15_V2.0860	*L. braziliensis*	127	1	127

LbrM15_V2.0860	*L. braziliensis*	89	156	244
LbrM15_V2.0860	*L. braziliensis*	49	308	356
LbrM15_V2.0870	*L. braziliensis*	120	1	120
LbrM15_V2.0870	*L. braziliensis*	196	195	390
LbrM15_V2.0870	*L. braziliensis*	390	449	838
LbrM15_V2.0880	*L. braziliensis*	157	1	157
LbrM15_V2.0880	*L. braziliensis*	56	221	276
LbrM15_V2.0880	*L. braziliensis*	44	441	484
LbrM15_V2.0880	*L. braziliensis*	60	494	553
LbrM15_V2.0880	*L. braziliensis*	42	712	753
LbrM15_V2.0890	*L. braziliensis*	244	54	297
LbrM15_V2.0900	*L. braziliensis*	59	1	59
LbrM15_V2.0900	*L. braziliensis*	59	343	401
LbrM15_V2.0900	*L. braziliensis*	59	343	401
LbrM15_V2.0910	*L. braziliensis*	84	141	224
LbrM15_V2.0920	*L. braziliensis*	88	154	241
LbrM15_V2.0930	*L. braziliensis*	55	1	55
LbrM15_V2.0940	*L. braziliensis*	60	123	182
LbrM15_V2.0950	*L. braziliensis*	127	60	186
LbrM15_V2.0950	*L. braziliensis*	48	224	271
LbrM15_V2.0950	*L. braziliensis*	159	335	493
LbrM15_V2.0950	*L. braziliensis*	147	531	677
LbrM15_V2.0950	*L. braziliensis*	41	714	754
LbrM15_V2.0950	*L. braziliensis*	116	849	964
LbrM15_V2.0950	*L. braziliensis*	56	1113	1168
LbrM15_V2.0950	*L. braziliensis*	429	1268	1696
LbrM15_V2.0950	*L. braziliensis*	85	1804	1888
LbrM15_V2.0950	*L. braziliensis*	57	2019	2075
LbrM15_V2.0950	*L. braziliensis*	50	2101	2150
LbrM15_V2.0950	*L. braziliensis*	94	2196	2289
LbrM15_V2.0950	*L. braziliensis*	41	2372	2412
LbrM15_V2.0950	*L. braziliensis*	74	2481	2554
LbrM15_V2.0960	*L. braziliensis*	179	1	179
LbrM15_V2.0960	*L. braziliensis*	83	580	662
LbrM15_V2.0980	*L. braziliensis*	55	1	55
LbrM15_V2.0980	*L. braziliensis*	56	147	202
LbrM15_V2.0980	*L. braziliensis*	88	219	306
LbrM15_V2.0980	*L. braziliensis*	200	338	537
LbrM15_V2.0980	*L. braziliensis*	73	600	672
LbrM15_V2.0980	*L. braziliensis*	64	679	742
LbrM15_V2.0980	*L. braziliensis*	55	827	881
LbrM15_V2.0980	*L. braziliensis*	40	925	964
LbrM15_V2.0980	*L. braziliensis*	232	997	1228
LbrM15_V2.1000	*L. braziliensis*	175	120	294
LbrM15_V2.1010	*L. braziliensis*	115	278	392
LbrM15_V2.1020	*L. braziliensis*	135	215	349
LbrM15_V2.1020	*L. braziliensis*	195	445	639

LbrM15_V2.1040	*L. braziliensis*	74	87	160
LbrM15_V2.1050	*L. braziliensis*	73	660	732
LbrM15_V2.1070	*L. braziliensis*	117	18	134
LbrM15_V2.1070	*L. braziliensis*	195	157	351
LbrM15_V2.1070	*L. braziliensis*	58	364	421
LbrM15_V2.1090	*L. braziliensis*	54	63	116
LbrM15_V2.1110	*L. braziliensis*	41	50	90
LbrM15_V2.1110	*L. braziliensis*	42	294	335
LbrM15_V2.1110	*L. braziliensis*	57	372	428
LbrM15_V2.1120	*L. braziliensis*	40	213	252
LbrM15_V2.1120	*L. braziliensis*	52	449	500
LbrM15_V2.1120	*L. braziliensis*	174	534	707
LbrM15_V2.1120	*L. braziliensis*	49	818	866
LbrM15_V2.1120	*L. braziliensis*	46	1088	1133
LbrM15_V2.1120	*L. braziliensis*	47	1731	1777
LbrM15_V2.1120	*L. braziliensis*	83	1836	1918
LbrM15_V2.1120	*L. braziliensis*	68	2288	2355
LbrM15_V2.1120	*L. braziliensis*	44	2430	2473
LbrM15_V2.1120	*L. braziliensis*	192	2509	2700
LbrM15_V2.1120	*L. braziliensis*	407	2707	3113
LbrM15_V2.1120	*L. braziliensis*	88	3148	3235
LbrM15_V2.1130	*L. braziliensis*	76	29	104
LbrM15_V2.1130	*L. braziliensis*	73	770	842
LbrM15_V2.1140	*L. braziliensis*	140	1	140
LbrM15_V2.1140	*L. braziliensis*	206	176	381
LbrM15_V2.1140	*L. braziliensis*	71	1025	1095
LbrM15_V2.1140	*L. braziliensis*	99	1105	1203
LbrM15_V2.1140	*L. braziliensis*	356	1602	1957
LbrM15_V2.1140	*L. braziliensis*	65	1961	2025
LbrM15_V2.1140	*L. braziliensis*	271	2072	2342
LbrM15_V2.1140	*L. braziliensis*	397	2358	2754
LbrM15_V2.1150	*L. braziliensis*	66	47	112
LbrM15_V2.1160	*L. braziliensis*	59	367	425
LbrM15_V2.1170	*L. braziliensis*	45	47	91
LbrM15_V2.1170	*L. braziliensis*	71	149	219
LbrM15_V2.1170	*L. braziliensis*	53	232	284
LbrM15_V2.1200	*L. braziliensis*	55	337	391
LbrM15_V2.1210	*L. braziliensis*	89	222	310
LbrM15_V2.1210	*L. braziliensis*	60	427	486
LbrM15_V2.1220	*L. braziliensis*	90	74	163
LbrM15_V2.1220	*L. braziliensis*	157	215	371
LbrM15_V2.1220	*L. braziliensis*	67	374	440
LbrM15_V2.1220	*L. braziliensis*	321	467	787
LbrM15_V2.1220	*L. braziliensis*	214	790	1003
LbrM15_V2.1220	*L. braziliensis*	106	1178	1283
LbrM15_V2.1220	*L. braziliensis*	143	1423	1565
LbrM15_V2.1220	*L. braziliensis*	46	1722	1767

LbrM15_V2.1230	*L. braziliensis*	111	1118	1228
LbrM15_V2.1240	*L. braziliensis*	125	800	924
LbrM15_V2.1260	*L. braziliensis*	85	1	85
LbrM15_V2.1260	*L. braziliensis*	123	91	213
LbrM15_V2.1260	*L. braziliensis*	126	225	350
LbrM15_V2.1260	*L. braziliensis*	67	411	477
LbrM15_V2.1270	*L. braziliensis*	43	1	43
LbrM15_V2.1270	*L. braziliensis*	276	53	328
LbrM15_V2.1270	*L. braziliensis*	82	367	448
LbrM15_V2.1270	*L. braziliensis*	43	582	624
LbrM15_V2.1270	*L. braziliensis*	129	735	863
LbrM15_V2.1270	*L. braziliensis*	46	1077	1122
LbrM15_V2.1270	*L. braziliensis*	287	1202	1488
LbrM15_V2.1290	*L. braziliensis*	96	123	218
LbrM15_V2.1310	*L. braziliensis*	49	1	49
LbrM15_V2.1310	*L. braziliensis*	251	81	331
LbrM15_V2.1320	*L. braziliensis*	48	1	48
LbrM15_V2.1320	*L. braziliensis*	98	77	174
LbrM15_V2.1320	*L. braziliensis*	98	77	174
LbrM15_V2.1330	*L. braziliensis*	108	10	117
LbrM15_V2.1330	*L. braziliensis*	73	401	473
LbrM15_V2.1340	*L. braziliensis*	59	1	59
LbrM15_V2.1350	*L. braziliensis*	41	180	220
LbrM15_V2.1380	*L. braziliensis*	90	355	444
LbrM15_V2.1400	*L. braziliensis*	67	212	278
LbrM15_V2.1410	*L. braziliensis*	154	338	491
LbrM15_V2.1420	*L. braziliensis*	74	133	206
LbrM15_V2.1420	*L. braziliensis*	49	957	1005
LbrM15_V2.1420	*L. braziliensis*	49	957	1005
LbrM15_V2.1430	*L. braziliensis*	43	190	232
LbrM15_V2.1440	*L. braziliensis*	42	231	272
LbrM15_V2.1460	*L. braziliensis*	44	82	125
LbrM15_V2.1490	*L. braziliensis*	44	1	44
LbrM15_V2.1490	*L. braziliensis*	79	104	182
LbrM15_V2.1490	*L. braziliensis*	244	375	618
LbrM15_V2.1540	*L. braziliensis*	55	362	416
LbrM15_V2.1550	*L. braziliensis*	102	197	298
LbrM15_V2.1570	*L. braziliensis*	53	503	555
LbrM15_V2.1570	*L. braziliensis*	49	1209	1257
LbrM15_V2.1570	*L. braziliensis*	49	1209	1257
LbrM15_V2.1580	*L. braziliensis*	236	308	543
LbrM16_V2.0060	*L. braziliensis*	51	183	233
LbrM16_V2.0060	*L. braziliensis*	40	270	309
LbrM16_V2.0060	*L. braziliensis*	48	535	582
LbrM16_V2.0070	*L. braziliensis*	78	882	959
LbrM16_V2.0090	*L. braziliensis*	76	1	76
LbrM16_V2.0090	*L. braziliensis*	65	379	443

LbrM16_V2.0090	*L. braziliensis*	82	508	589
LbrM16_V2.0090	*L. braziliensis*	81	651	731
LbrM16_V2.0100	*L. braziliensis*	44	367	410
LbrM16_V2.0120	*L. braziliensis*	51	579	629
LbrM16_V2.0130	*L. braziliensis*	60	198	257
LbrM16_V2.0130	*L. braziliensis*	51	497	547
LbrM16_V2.0130	*L. braziliensis*	51	497	547
LbrM16_V2.0140	*L. braziliensis*	57	1	57
LbrM16_V2.0140	*L. braziliensis*	95	74	168
LbrM16_V2.0140	*L. braziliensis*	60	171	230
LbrM16_V2.0150	*L. braziliensis*	40	1	40
LbrM16_V2.0150	*L. braziliensis*	53	116	168
LbrM16_V2.0150	*L. braziliensis*	53	116	168
LbrM16_V2.0160	*L. braziliensis*	59	87	145
LbrM16_V2.0160	*L. braziliensis*	141	184	324
LbrM16_V2.0170	*L. braziliensis*	57	249	305
LbrM16_V2.0180	*L. braziliensis*	115	142	256
LbrM16_V2.0180	*L. braziliensis*	89	301	389
LbrM16_V2.0180	*L. braziliensis*	231	1123	1353
LbrM16_V2.0180	*L. braziliensis*	284	1511	1794
LbrM16_V2.0180	*L. braziliensis*	47	1942	1988
LbrM16_V2.0180	*L. braziliensis*	51	2002	2052
LbrM16_V2.0180	*L. braziliensis*	55	2305	2359
LbrM16_V2.0180	*L. braziliensis*	63	2396	2458
LbrM16_V2.0180	*L. braziliensis*	40	2567	2606
LbrM16_V2.0180	*L. braziliensis*	105	2735	2839
LbrM16_V2.0180	*L. braziliensis*	42	2918	2959
LbrM16_V2.0180	*L. braziliensis*	46	3062	3107
LbrM16_V2.0180	*L. braziliensis*	71	3340	3410
LbrM16_V2.0180	*L. braziliensis*	83	3447	3529
LbrM16_V2.0180	*L. braziliensis*	45	3630	3674
LbrM16_V2.0180	*L. braziliensis*	74	4264	4337
LbrM16_V2.0180	*L. braziliensis*	55	4379	4433
LbrM16_V2.0190	*L. braziliensis*	77	1	77
LbrM16_V2.0190	*L. braziliensis*	95	90	184
LbrM16_V2.0200	*L. braziliensis*	70	1	70
LbrM16_V2.0200	*L. braziliensis*	114	244	357
LbrM16_V2.0220	*L. braziliensis*	70	104	173
LbrM16_V2.0230	*L. braziliensis*	51	82	132
LbrM16_V2.0250	*L. braziliensis*	52	110	161
LbrM16_V2.0270	*L. braziliensis*	42	91	132
LbrM16_V2.0280	*L. braziliensis*	48	53	100
LbrM16_V2.0280	*L. braziliensis*	67	140	206
LbrM16_V2.0280	*L. braziliensis*	67	140	206
LbrM16_V2.0290	*L. braziliensis*	50	1	50
LbrM16_V2.0290	*L. braziliensis*	66	88	153
LbrM16_V2.0290	*L. braziliensis*	66	88	153

LbrM16_V2.0300	*L. braziliensis*	121	277	397
LbrM16_V2.0300	*L. braziliensis*	52	426	477
LbrM16_V2.0300	*L. braziliensis*	48	484	531
LbrM16_V2.0320	*L. braziliensis*	66	70	135
LbrM16_V2.0320	*L. braziliensis*	72	179	250
LbrM16_V2.0320	*L. braziliensis*	72	179	250
LbrM16_V2.0330	*L. braziliensis*	492	384	875
LbrM16_V2.0330	*L. braziliensis*	223	937	1159
LbrM16_V2.0340	*L. braziliensis*	42	23	64
LbrM16_V2.0340	*L. braziliensis*	55	133	187
LbrM16_V2.0340	*L. braziliensis*	55	133	187
LbrM16_V2.0360	*L. braziliensis*	70	1	70
LbrM16_V2.0370	*L. braziliensis*	60	17	76
LbrM16_V2.0370	*L. braziliensis*	134	278	411
LbrM16_V2.0370	*L. braziliensis*	156	429	584
LbrM16_V2.0380	*L. braziliensis*	50	73	122
LbrM16_V2.0390	*L. braziliensis*	174	1	174
LbrM16_V2.0390	*L. braziliensis*	58	193	250
LbrM16_V2.0390	*L. braziliensis*	80	437	516
LbrM16_V2.0400	*L. braziliensis*	57	44	100
LbrM16_V2.0400	*L. braziliensis*	52	254	305
LbrM16_V2.0400	*L. braziliensis*	204	657	860
LbrM16_V2.0400	*L. braziliensis*	130	864	993
LbrM16_V2.0410	*L. braziliensis*	48	54	101
LbrM16_V2.0420	*L. braziliensis*	48	77	124
LbrM16_V2.0420	*L. braziliensis*	41	164	204
LbrM16_V2.0420	*L. braziliensis*	143	404	546
LbrM16_V2.0430	*L. braziliensis*	54	1	54
LbrM16_V2.0430	*L. braziliensis*	42	433	474
LbrM16_V2.0430	*L. braziliensis*	42	524	565
LbrM16_V2.0440	*L. braziliensis*	93	1	93
LbrM16_V2.0440	*L. braziliensis*	72	112	183
LbrM16_V2.0450	*L. braziliensis*	43	1	43
LbrM16_V2.0460	*L. braziliensis*	64	1	64
LbrM16_V2.0460	*L. braziliensis*	85	173	257
LbrM16_V2.0460	*L. braziliensis*	92	264	355
LbrM16_V2.0460	*L. braziliensis*	87	362	448
LbrM16_V2.0460	*L. braziliensis*	191	774	964
LbrM16_V2.0460	*L. braziliensis*	66	987	1052
LbrM16_V2.0460	*L. braziliensis*	141	1084	1224
LbrM16_V2.0470	*L. braziliensis*	193	38	230
LbrM16_V2.0470	*L. braziliensis*	66	253	318
LbrM16_V2.0470	*L. braziliensis*	141	350	490
LbrM16_V2.0500	*L. braziliensis*	41	709	749
LbrM16_V2.0530	*L. braziliensis*	49	28	76
LbrM16_V2.0530	*L. braziliensis*	67	141	207
LbrM16_V2.0530	*L. braziliensis*	43	225	267

LbrM16_V2.0530	*L. braziliensis*	91	308	398
LbrM16_V2.0530	*L. braziliensis*	356	495	850
LbrM16_V2.0580	*L. braziliensis*	81	155	235
LbrM16_V2.0580	*L. braziliensis*	65	407	471
LbrM16_V2.0610	*L. braziliensis*	215	58	272
LbrM16_V2.0610	*L. braziliensis*	184	427	610
LbrM16_V2.0610	*L. braziliensis*	99	880	978
LbrM16_V2.0610	*L. braziliensis*	52	1049	1100
LbrM16_V2.0620	*L. braziliensis*	58	70	127
LbrM16_V2.0620	*L. braziliensis*	54	240	293
LbrM16_V2.0620	*L. braziliensis*	80	417	496
LbrM16_V2.0630	*L. braziliensis*	113	116	228
LbrM16_V2.0630	*L. braziliensis*	77	438	514
LbrM16_V2.0630	*L. braziliensis*	77	438	514
LbrM16_V2.0650	*L. braziliensis*	495	71	565
LbrM16_V2.0660	*L. braziliensis*	57	30	86
LbrM16_V2.0660	*L. braziliensis*	137	91	227
LbrM16_V2.0660	*L. braziliensis*	51	405	455
LbrM16_V2.0660	*L. braziliensis*	47	532	578
LbrM16_V2.0670	*L. braziliensis*	89	1	89
LbrM16_V2.0680	*L. braziliensis*	65	487	551
LbrM16_V2.0690	*L. braziliensis*	81	1	81
LbrM16_V2.0690	*L. braziliensis*	654	106	759
LbrM16_V2.0690	*L. braziliensis*	71	788	858
LbrM16_V2.0700	*L. braziliensis*	133	1	133
LbrM16_V2.0700	*L. braziliensis*	348	143	490
LbrM16_V2.0700	*L. braziliensis*	94	526	619
LbrM16_V2.0700	*L. braziliensis*	274	640	913
LbrM16_V2.0700	*L. braziliensis*	98	929	1026
LbrM16_V2.0710	*L. braziliensis*	58	55	112
LbrM16_V2.0710	*L. braziliensis*	45	170	214
LbrM16_V2.0710	*L. braziliensis*	41	276	316
LbrM16_V2.0710	*L. braziliensis*	45	468	512
LbrM16_V2.0720	*L. braziliensis*	53	78	130
LbrM16_V2.0720	*L. braziliensis*	100	413	512
LbrM16_V2.0720	*L. braziliensis*	44	613	656
LbrM16_V2.0720	*L. braziliensis*	130	747	876
LbrM16_V2.0720	*L. braziliensis*	44	916	959
LbrM16_V2.0720	*L. braziliensis*	56	1039	1094
LbrM16_V2.0720	*L. braziliensis*	112	1115	1226
LbrM16_V2.0720	*L. braziliensis*	89	1371	1459
LbrM16_V2.0720	*L. braziliensis*	40	1592	1631
LbrM16_V2.0730	*L. braziliensis*	68	51	118
LbrM16_V2.0730	*L. braziliensis*	108	136	243
LbrM16_V2.0730	*L. braziliensis*	60	284	343
LbrM16_V2.0730	*L. braziliensis*	52	346	397
LbrM16_V2.0730	*L. braziliensis*	146	466	611

LbrM16_V2.0730	*L. braziliensis*	74	675	748
LbrM16_V2.0740	*L. braziliensis*	64	149	212
LbrM16_V2.0740	*L. braziliensis*	48	361	408
LbrM16_V2.0740	*L. braziliensis*	70	464	533
LbrM16_V2.0740	*L. braziliensis*	69	539	607
LbrM16_V2.0760	*L. braziliensis*	313	53	365
LbrM16_V2.0760	*L. braziliensis*	47	510	556
LbrM16_V2.0770	*L. braziliensis*	41	48	88
LbrM16_V2.0770	*L. braziliensis*	72	124	195
LbrM16_V2.0770	*L. braziliensis*	98	473	570
LbrM16_V2.0770	*L. braziliensis*	44	704	747
LbrM16_V2.0770	*L. braziliensis*	56	869	924
LbrM16_V2.0770	*L. braziliensis*	119	1012	1130
LbrM16_V2.0770	*L. braziliensis*	58	1252	1309
LbrM16_V2.0770	*L. braziliensis*	62	1405	1466
LbrM16_V2.0770	*L. braziliensis*	89	1614	1702
LbrM16_V2.0770	*L. braziliensis*	70	1749	1818
LbrM16_V2.0770	*L. braziliensis*	59	1893	1951
LbrM16_V2.0770	*L. braziliensis*	40	1997	2036
LbrM16_V2.0770	*L. braziliensis*	68	2090	2157
LbrM16_V2.0770	*L. braziliensis*	48	2363	2410
LbrM16_V2.0770	*L. braziliensis*	68	2455	2522
LbrM16_V2.0770	*L. braziliensis*	52	2560	2611
LbrM16_V2.0770	*L. braziliensis*	58	2688	2745
LbrM16_V2.0770	*L. braziliensis*	49	2838	2886
LbrM16_V2.0770	*L. braziliensis*	92	3004	3095
LbrM16_V2.0770	*L. braziliensis*	61	3376	3436
LbrM16_V2.0770	*L. braziliensis*	67	3731	3797
LbrM16_V2.0770	*L. braziliensis*	48	4369	4416
LbrM16_V2.0770	*L. braziliensis*	284	5218	5501
LbrM16_V2.0780	*L. braziliensis*	64	1	64
LbrM16_V2.0780	*L. braziliensis*	40	107	146
LbrM16_V2.0780	*L. braziliensis*	70	180	249
LbrM16_V2.0780	*L. braziliensis*	71	271	341
LbrM16_V2.0780	*L. braziliensis*	80	366	445
LbrM16_V2.0780	*L. braziliensis*	82	462	543
LbrM16_V2.0780	*L. braziliensis*	77	564	640
LbrM16_V2.0790	*L. braziliensis*	120	1	120
LbrM16_V2.0790	*L. braziliensis*	63	143	205
LbrM16_V2.0790	*L. braziliensis*	136	245	380
LbrM16_V2.0800	*L. braziliensis*	41	290	330
LbrM16_V2.0810	*L. braziliensis*	139	269	407
LbrM16_V2.0810	*L. braziliensis*	43	593	635
LbrM16_V2.0810	*L. braziliensis*	43	593	635
LbrM16_V2.0820	*L. braziliensis*	68	179	246
LbrM16_V2.0830	*L. braziliensis*	63	132	194
LbrM16_V2.0830	*L. braziliensis*	169	196	364

LbrM16_V2.0830	*L. braziliensis*	97	536	632
LbrM16_V2.0840	*L. braziliensis*	162	1	162
LbrM16_V2.0850	*L. braziliensis*	46	318	363
LbrM16_V2.0850	*L. braziliensis*	47	410	456
LbrM16_V2.0850	*L. braziliensis*	47	410	456
LbrM16_V2.0860	*L. braziliensis*	145	329	473
LbrM16_V2.0870	*L. braziliensis*	48	1	48
LbrM16_V2.0870	*L. braziliensis*	109	91	199
LbrM16_V2.0870	*L. braziliensis*	109	91	199
LbrM16_V2.0880	*L. braziliensis*	142	1	142
LbrM16_V2.0890	*L. braziliensis*	84	96	179
LbrM16_V2.0890	*L. braziliensis*	57	293	349
LbrM16_V2.0890	*L. braziliensis*	51	456	506
LbrM16_V2.0890	*L. braziliensis*	132	531	662
LbrM16_V2.0900	*L. braziliensis*	58	58	115
LbrM16_V2.0900	*L. braziliensis*	152	147	298
LbrM16_V2.0910	*L. braziliensis*	99	171	269
LbrM16_V2.0910	*L. braziliensis*	62	1149	1210
LbrM16_V2.0910	*L. braziliensis*	62	1149	1210
LbrM16_V2.0930	*L. braziliensis*	44	1	44
LbrM16_V2.0940	*L. braziliensis*	66	1	66
LbrM16_V2.0940	*L. braziliensis*	91	133	223
LbrM16_V2.0970	*L. braziliensis*	160	188	347
LbrM16_V2.0980	*L. braziliensis*	86	163	248
LbrM16_V2.0990	*L. braziliensis*	830	1	830
LbrM16_V2.1000	*L. braziliensis*	42	150	191
LbrM16_V2.1000	*L. braziliensis*	48	307	354
LbrM16_V2.1000	*L. braziliensis*	48	307	354
LbrM16_V2.1020	*L. braziliensis*	89	98	186
LbrM16_V2.1040	*L. braziliensis*	124	68	191
LbrM16_V2.1060	*L. braziliensis*	63	1	63
LbrM16_V2.1070	*L. braziliensis*	45	271	315
LbrM16_V2.1080	*L. braziliensis*	53	1	53
LbrM16_V2.1080	*L. braziliensis*	41	248	288
LbrM16_V2.1080	*L. braziliensis*	41	248	288
LbrM16_V2.1100	*L. braziliensis*	57	16	72
LbrM16_V2.1100	*L. braziliensis*	59	155	213
LbrM16_V2.1100	*L. braziliensis*	47	439	485
LbrM16_V2.1110	*L. braziliensis*	369	78	446
LbrM16_V2.1110	*L. braziliensis*	41	454	494
LbrM16_V2.1110	*L. braziliensis*	86	520	605
LbrM16_V2.1120	*L. braziliensis*	59	1	59
LbrM16_V2.1120	*L. braziliensis*	248	93	340
LbrM16_V2.1120	*L. braziliensis*	62	406	467
LbrM16_V2.1120	*L. braziliensis*	287	496	782
LbrM16_V2.1120	*L. braziliensis*	438	816	1253
LbrM16_V2.1120	*L. braziliensis*	90	1286	1375

LbrM16_V2.1120	*L. braziliensis*	235	1393	1627
LbrM16_V2.1120	*L. braziliensis*	117	1658	1774
LbrM16_V2.1120	*L. braziliensis*	532	1811	2342
LbrM16_V2.1130	*L. braziliensis*	69	1	69
LbrM16_V2.1130	*L. braziliensis*	154	79	232
LbrM16_V2.1130	*L. braziliensis*	58	282	339
LbrM16_V2.1130	*L. braziliensis*	84	376	459
LbrM16_V2.1130	*L. braziliensis*	134	487	620
LbrM16_V2.1130	*L. braziliensis*	105	648	752
LbrM16_V2.1130	*L. braziliensis*	123	790	912
LbrM16_V2.1140	*L. braziliensis*	226	147	372
LbrM16_V2.1170	*L. braziliensis*	177	214	390
LbrM16_V2.1170	*L. braziliensis*	117	409	525
LbrM16_V2.1190	*L. braziliensis*	91	1	91
LbrM16_V2.1200	*L. braziliensis*	86	126	211
LbrM16_V2.1200	*L. braziliensis*	66	268	333
LbrM16_V2.1200	*L. braziliensis*	40	418	457
LbrM16_V2.1220	*L. braziliensis*	146	143	288
LbrM16_V2.1230	*L. braziliensis*	106	142	247
LbrM16_V2.1230	*L. braziliensis*	53	426	478
LbrM16_V2.1230	*L. braziliensis*	80	534	613
LbrM16_V2.1230	*L. braziliensis*	97	1038	1134
LbrM16_V2.1230	*L. braziliensis*	143	1215	1357
LbrM16_V2.1240	*L. braziliensis*	107	243	349
LbrM16_V2.1250	*L. braziliensis*	42	1	42
LbrM16_V2.1250	*L. braziliensis*	135	91	225
LbrM16_V2.1250	*L. braziliensis*	142	331	472
LbrM16_V2.1250	*L. braziliensis*	43	528	570
LbrM16_V2.1250	*L. braziliensis*	62	853	914
LbrM16_V2.1250	*L. braziliensis*	81	976	1056
LbrM16_V2.1250	*L. braziliensis*	43	1078	1120
LbrM16_V2.1250	*L. braziliensis*	75	1201	1275
LbrM16_V2.1260	*L. braziliensis*	174	1	174
LbrM16_V2.1260	*L. braziliensis*	386	264	649
LbrM16_V2.1260	*L. braziliensis*	239	758	996
LbrM16_V2.1260	*L. braziliensis*	87	1173	1259
LbrM16_V2.1260	*L. braziliensis*	105	1262	1366
LbrM16_V2.1260	*L. braziliensis*	49	1486	1534
LbrM16_V2.1260	*L. braziliensis*	44	1617	1660
LbrM16_V2.1260	*L. braziliensis*	51	1876	1926
LbrM16_V2.1270	*L. braziliensis*	61	620	680
LbrM16_V2.1270	*L. braziliensis*	167	953	1119
LbrM16_V2.1280	*L. braziliensis*	51	21	71
LbrM16_V2.1290	*L. braziliensis*	176	63	238
LbrM16_V2.1290	*L. braziliensis*	117	263	379
LbrM16_V2.1290	*L. braziliensis*	274	424	697
LbrM16_V2.1290	*L. braziliensis*	50	805	854

LbrM16_V2.1290	*L. braziliensis*	114	910	1023
LbrM16_V2.1290	*L. braziliensis*	43	1131	1173
LbrM16_V2.1290	*L. braziliensis*	167	1238	1404
LbrM16_V2.1290	*L. braziliensis*	184	1412	1595
LbrM16_V2.1290	*L. braziliensis*	117	2010	2126
LbrM16_V2.1310	*L. braziliensis*	242	44	285
LbrM16_V2.1310	*L. braziliensis*	86	318	403
LbrM16_V2.1310	*L. braziliensis*	129	738	866
LbrM16_V2.1310	*L. braziliensis*	94	898	991
LbrM16_V2.1310	*L. braziliensis*	52	1083	1134
LbrM16_V2.1310	*L. braziliensis*	103	1386	1488
LbrM16_V2.1310	*L. braziliensis*	72	1538	1609
LbrM16_V2.1310	*L. braziliensis*	65	1685	1749
LbrM16_V2.1320	*L. braziliensis*	239	1	239
LbrM16_V2.1320	*L. braziliensis*	196	275	470
LbrM16_V2.1320	*L. braziliensis*	166	553	718
LbrM16_V2.1320	*L. braziliensis*	61	826	886
LbrM16_V2.1320	*L. braziliensis*	610	1020	1629
LbrM16_V2.1320	*L. braziliensis*	69	1738	1806
LbrM16_V2.1320	*L. braziliensis*	50	1852	1901
LbrM16_V2.1320	*L. braziliensis*	82	1914	1995
LbrM16_V2.1320	*L. braziliensis*	170	2080	2249
LbrM16_V2.1340	*L. braziliensis*	54	1	54
LbrM16_V2.1340	*L. braziliensis*	47	273	319
LbrM16_V2.1340	*L. braziliensis*	47	273	319
LbrM16_V2.1350	*L. braziliensis*	89	51	139
LbrM16_V2.1350	*L. braziliensis*	85	163	247
LbrM16_V2.1350	*L. braziliensis*	92	287	378
LbrM16_V2.1350	*L. braziliensis*	147	487	633
LbrM16_V2.1350	*L. braziliensis*	61	897	957
LbrM16_V2.1380	*L. braziliensis*	567	1	567
LbrM16_V2.1380	*L. braziliensis*	70	1176	1245
LbrM16_V2.1390	*L. braziliensis*	48	30	77
LbrM16_V2.1390	*L. braziliensis*	188	421	608
LbrM16_V2.1390	*L. braziliensis*	57	1266	1322
LbrM16_V2.1390	*L. braziliensis*	54	1453	1506
LbrM16_V2.1390	*L. braziliensis*	75	1696	1770
LbrM16_V2.1390	*L. braziliensis*	71	1813	1883
LbrM16_V2.1390	*L. braziliensis*	41	1933	1973
LbrM16_V2.1390	*L. braziliensis*	61	2196	2256
LbrM16_V2.1390	*L. braziliensis*	41	2503	2543
LbrM16_V2.1390	*L. braziliensis*	85	2653	2737
LbrM16_V2.1390	*L. braziliensis*	55	3006	3060
LbrM16_V2.1390	*L. braziliensis*	51	3113	3163
LbrM16_V2.1390	*L. braziliensis*	88	3195	3282
LbrM16_V2.1390	*L. braziliensis*	61	3289	3349
LbrM16_V2.1400	*L. braziliensis*	71	191	261

LbrM16_V2.1400	*L. braziliensis*	46	931	976
LbrM16_V2.1400	*L. braziliensis*	56	1107	1162
LbrM16_V2.1400	*L. braziliensis*	178	1376	1553
LbrM16_V2.1440	*L. braziliensis*	136	1	136
LbrM16_V2.1440	*L. braziliensis*	212	147	358
LbrM16_V2.1440	*L. braziliensis*	224	498	721
LbrM16_V2.1440	*L. braziliensis*	167	759	925
LbrM16_V2.1450	*L. braziliensis*	44	184	227
LbrM16_V2.1470	*L. braziliensis*	75	75	149
LbrM16_V2.1480	*L. braziliensis*	46	130	175
LbrM16_V2.1480	*L. braziliensis*	120	259	378
LbrM16_V2.1480	*L. braziliensis*	45	485	529
LbrM16_V2.1480	*L. braziliensis*	57	543	599
LbrM16_V2.1490	*L. braziliensis*	124	1	124
LbrM16_V2.1490	*L. braziliensis*	45	231	275
LbrM16_V2.1490	*L. braziliensis*	57	289	345
LbrM16_V2.1500	*L. braziliensis*	49	465	513
LbrM16_V2.1500	*L. braziliensis*	94	684	777
LbrM16_V2.1500	*L. braziliensis*	46	963	1008
LbrM16_V2.1500	*L. braziliensis*	120	1123	1242
LbrM16_V2.1510	*L. braziliensis*	566	1	566
LbrM16_V2.1510	*L. braziliensis*	57	664	720
LbrM16_V2.1530	*L. braziliensis*	133	1	133
LbrM16_V2.1530	*L. braziliensis*	307	585	891
LbrM16_V2.1530	*L. braziliensis*	49	989	1037
LbrM16_V2.1540	*L. braziliensis*	40	1	40
LbrM16_V2.1540	*L. braziliensis*	60	52	111
LbrM16_V2.1540	*L. braziliensis*	106	220	325
LbrM16_V2.1550	*L. braziliensis*	97	268	364
LbrM16_V2.1560	*L. braziliensis*	77	344	420
LbrM16_V2.1580	*L. braziliensis*	61	26	86
LbrM16_V2.1580	*L. braziliensis*	80	242	321
LbrM16_V2.1580	*L. braziliensis*	145	692	836
LbrM16_V2.1580	*L. braziliensis*	157	987	1143
LbrM16_V2.1600	*L. braziliensis*	227	1	227
LbrM16_V2.1610	*L. braziliensis*	339	559	897
LbrM16_V2.1610	*L. braziliensis*	58	947	1004
LbrM16_V2.1620	*L. braziliensis*	59	284	342
LbrM16_V2.1630	*L. braziliensis*	134	141	274
LbrM16_V2.1640	*L. braziliensis*	112	332	443
LbrM16_V2.1640	*L. braziliensis*	54	490	543
LbrM16_V2.1640	*L. braziliensis*	80	786	865
LbrM16_V2.1650	*L. braziliensis*	99	1	99
LbrM16_V2.1650	*L. braziliensis*	73	209	281
LbrM16_V2.1650	*L. braziliensis*	73	209	281
LbrM16_V2.1660	*L. braziliensis*	114	189	302
LbrM16_V2.1680	*L. braziliensis*	46	314	359

LbrM16_V2.1690	*L. braziliensis*	51	71	121
LbrM16_V2.1690	*L. braziliensis*	88	152	239
LbrM16_V2.1690	*L. braziliensis*	88	152	239
LbrM16_V2.1720	*L. braziliensis*	41	751	791
LbrM16_V2.1720	*L. braziliensis*	75	1555	1629
LbrM16_V2.1720	*L. braziliensis*	63	3834	3896
LbrM16_V2.1730	*L. braziliensis*	47	15	61
LbrM16_V2.1730	*L. braziliensis*	66	95	160
LbrM16_V2.1730	*L. braziliensis*	53	204	256
LbrM16_V2.1730	*L. braziliensis*	71	284	354
LbrM16_V2.1730	*L. braziliensis*	77	382	458
LbrM16_V2.1730	*L. braziliensis*	71	480	550
LbrM16_V2.1730	*L. braziliensis*	89	574	662
LbrM16_V2.1730	*L. braziliensis*	73	675	747
LbrM16_V2.1730	*L. braziliensis*	86	769	854
LbrM16_V2.1740	*L. braziliensis*	60	1	60
LbrM16_V2.1740	*L. braziliensis*	80	85	164
LbrM16_V2.1740	*L. braziliensis*	81	182	262
LbrM16_V2.1740	*L. braziliensis*	90	279	368
LbrM16_V2.1740	*L. braziliensis*	77	381	457
LbrM16_V2.1740	*L. braziliensis*	191	787	977
LbrM16_V2.1740	*L. braziliensis*	66	1000	1065
LbrM16_V2.1740	*L. braziliensis*	141	1097	1237
LbrM16_V2.1740	*L. braziliensis*	129	1485	1613
LbrM17_V2.0010	*L. braziliensis*	65	1244	1308
LbrM17_V2.0020	*L. braziliensis*	48	79	126
LbrM17_V2.0020	*L. braziliensis*	113	514	626
LbrM17_V2.0020	*L. braziliensis*	89	686	774
LbrM17_V2.0040	*L. braziliensis*	64	107	170
LbrM17_V2.0050	*L. braziliensis*	49	214	262
LbrM17_V2.0060	*L. braziliensis*	63	146	208
LbrM17_V2.0070	*L. braziliensis*	69	431	499
LbrM17_V2.0100	*L. braziliensis*	91	939	1029
LbrM17_V2.0130	*L. braziliensis*	155	1251	1405
LbrM17_V2.0140	*L. braziliensis*	50	24	73
LbrM17_V2.0140	*L. braziliensis*	68	398	465
LbrM17_V2.0140	*L. braziliensis*	115	507	621
LbrM17_V2.0150	*L. braziliensis*	114	1	114
LbrM17_V2.0150	*L. braziliensis*	294	139	432
LbrM17_V2.0170	*L. braziliensis*	186	661	846
LbrM17_V2.0190	*L. braziliensis*	41	446	486
LbrM17_V2.0190	*L. braziliensis*	60	518	577
LbrM17_V2.0190	*L. braziliensis*	46	610	655
LbrM17_V2.0190	*L. braziliensis*	50	771	820
LbrM17_V2.0200	*L. braziliensis*	106	45	150
LbrM17_V2.0200	*L. braziliensis*	128	217	344
LbrM17_V2.0200	*L. braziliensis*	140	378	517

LbrM17_V2.0200	*L. braziliensis*	110	527	636
LbrM17_V2.0210	*L. braziliensis*	248	1	248
LbrM17_V2.0210	*L. braziliensis*	55	350	404
LbrM17_V2.0220	*L. braziliensis*	72	1	72
LbrM17_V2.0220	*L. braziliensis*	152	75	226
LbrM17_V2.0220	*L. braziliensis*	213	275	487
LbrM17_V2.0220	*L. braziliensis*	62	965	1026
LbrM17_V2.0220	*L. braziliensis*	148	1102	1249
LbrM17_V2.0240	*L. braziliensis*	177	49	225
LbrM17_V2.0240	*L. braziliensis*	148	261	408
LbrM17_V2.0240	*L. braziliensis*	318	438	755
LbrM17_V2.0240	*L. braziliensis*	61	758	818
LbrM17_V2.0250	*L. braziliensis*	54	221	274
LbrM17_V2.0260	*L. braziliensis*	81	1	81
LbrM17_V2.0270	*L. braziliensis*	216	82	297
LbrM17_V2.0270	*L. braziliensis*	45	403	447
LbrM17_V2.0270	*L. braziliensis*	47	582	628
LbrM17_V2.0280	*L. braziliensis*	40	156	195
LbrM17_V2.0290	*L. braziliensis*	53	1	53
LbrM17_V2.0290	*L. braziliensis*	84	57	140
LbrM17_V2.0290	*L. braziliensis*	73	209	281
LbrM17_V2.0290	*L. braziliensis*	350	381	730
LbrM17_V2.0290	*L. braziliensis*	44	733	776
LbrM17_V2.0290	*L. braziliensis*	391	783	1173
LbrM17_V2.0290	*L. braziliensis*	158	1185	1342
LbrM17_V2.0300	*L. braziliensis*	72	149	220
LbrM17_V2.0300	*L. braziliensis*	68	231	298
LbrM17_V2.0300	*L. braziliensis*	68	231	298
LbrM17_V2.0310	*L. braziliensis*	61	151	211
LbrM17_V2.0310	*L. braziliensis*	48	320	367
LbrM17_V2.0310	*L. braziliensis*	51	760	810
LbrM17_V2.0310	*L. braziliensis*	50	848	897
LbrM17_V2.0310	*L. braziliensis*	42	1268	1309
LbrM17_V2.0310	*L. braziliensis*	41	1644	1684
LbrM17_V2.0330	*L. braziliensis*	229	1	229
LbrM17_V2.0350	*L. braziliensis*	91	1	91
LbrM17_V2.0350	*L. braziliensis*	61	181	241
LbrM17_V2.0350	*L. braziliensis*	267	374	640
LbrM17_V2.0360	*L. braziliensis*	59	1	59
LbrM17_V2.0360	*L. braziliensis*	99	107	205
LbrM17_V2.0360	*L. braziliensis*	89	232	320
LbrM17_V2.0370	*L. braziliensis*	73	1	73
LbrM17_V2.0370	*L. braziliensis*	66	82	147
LbrM17_V2.0370	*L. braziliensis*	73	260	332
LbrM17_V2.0370	*L. braziliensis*	64	594	657
LbrM17_V2.0370	*L. braziliensis*	113	723	835
LbrM17_V2.0370	*L. braziliensis*	52	1089	1140

LbrM17_V2.0380	*L. braziliensis*	216	39	254
LbrM17_V2.0380	*L. braziliensis*	86	297	382
LbrM17_V2.0380	*L. braziliensis*	90	482	571
LbrM17_V2.0390	*L. braziliensis*	95	56	150
LbrM17_V2.0390	*L. braziliensis*	97	490	586
LbrM17_V2.0390	*L. braziliensis*	297	701	997
LbrM17_V2.0400	*L. braziliensis*	50	224	273
LbrM17_V2.0400	*L. braziliensis*	142	347	488
LbrM17_V2.0400	*L. braziliensis*	142	347	488
LbrM17_V2.0410	*L. braziliensis*	365	1	365
LbrM17_V2.0410	*L. braziliensis*	136	398	533
LbrM17_V2.0410	*L. braziliensis*	409	539	947
LbrM17_V2.0410	*L. braziliensis*	143	999	1141
LbrM17_V2.0410	*L. braziliensis*	48	1199	1246
LbrM17_V2.0420	*L. braziliensis*	77	17	93
LbrM17_V2.0420	*L. braziliensis*	71	124	194
LbrM17_V2.0420	*L. braziliensis*	331	326	656
LbrM17_V2.0420	*L. braziliensis*	203	669	871
LbrM17_V2.0420	*L. braziliensis*	554	876	1429
LbrM17_V2.0420	*L. braziliensis*	70	1578	1647
LbrM17_V2.0430	*L. braziliensis*	171	91	261
LbrM17_V2.0430	*L. braziliensis*	68	352	419
LbrM17_V2.0430	*L. braziliensis*	62	479	540
LbrM17_V2.0430	*L. braziliensis*	67	595	661
LbrM17_V2.0430	*L. braziliensis*	124	779	902
LbrM17_V2.0430	*L. braziliensis*	43	1153	1195
LbrM17_V2.0430	*L. braziliensis*	61	1313	1373
LbrM17_V2.0440	*L. braziliensis*	120	18	137
LbrM17_V2.0450	*L. braziliensis*	41	32	72
LbrM17_V2.0450	*L. braziliensis*	61	90	150
LbrM17_V2.0450	*L. braziliensis*	42	282	323
LbrM17_V2.0450	*L. braziliensis*	145	433	577
LbrM17_V2.0450	*L. braziliensis*	87	614	700
LbrM17_V2.0450	*L. braziliensis*	82	976	1057
LbrM17_V2.0450	*L. braziliensis*	95	1359	1453
LbrM17_V2.0450	*L. braziliensis*	64	1644	1707
LbrM17_V2.0450	*L. braziliensis*	64	2117	2180
LbrM17_V2.0450	*L. braziliensis*	62	2298	2359
LbrM17_V2.0450	*L. braziliensis*	65	2573	2637
LbrM17_V2.0450	*L. braziliensis*	49	2674	2722
LbrM17_V2.0450	*L. braziliensis*	76	2989	3064
LbrM17_V2.0450	*L. braziliensis*	63	3199	3261
LbrM17_V2.0460	*L. braziliensis*	40	48	87
LbrM17_V2.0460	*L. braziliensis*	99	118	216
LbrM17_V2.0460	*L. braziliensis*	99	118	216
LbrM17_V2.0470	*L. braziliensis*	244	1	244
LbrM17_V2.0470	*L. braziliensis*	89	274	362

LbrM17_V2.0470	*L. braziliensis*	178	367	544
LbrM17_V2.0470	*L. braziliensis*	73	613	685
LbrM17_V2.0470	*L. braziliensis*	112	735	846
LbrM17_V2.0480	*L. braziliensis*	168	1	168
LbrM17_V2.0480	*L. braziliensis*	71	215	285
LbrM17_V2.0480	*L. braziliensis*	113	298	410
LbrM17_V2.0490	*L. braziliensis*	130	1	130
LbrM17_V2.0490	*L. braziliensis*	260	138	397
LbrM17_V2.0490	*L. braziliensis*	164	433	596
LbrM17_V2.0490	*L. braziliensis*	75	603	677
LbrM17_V2.0490	*L. braziliensis*	85	716	800
LbrM17_V2.0490	*L. braziliensis*	84	847	930
LbrM17_V2.0500	*L. braziliensis*	57	50	106
LbrM17_V2.0500	*L. braziliensis*	208	110	317
LbrM17_V2.0500	*L. braziliensis*	408	334	741
LbrM17_V2.0500	*L. braziliensis*	49	760	808
LbrM17_V2.0500	*L. braziliensis*	60	816	875
LbrM17_V2.0500	*L. braziliensis*	139	885	1023
LbrM17_V2.0510	*L. braziliensis*	235	1	235
LbrM17_V2.0510	*L. braziliensis*	83	253	335
LbrM17_V2.0510	*L. braziliensis*	210	365	574
LbrM17_V2.0510	*L. braziliensis*	255	654	908
LbrM17_V2.0520	*L. braziliensis*	46	36	81
LbrM17_V2.0520	*L. braziliensis*	418	108	525
LbrM17_V2.0520	*L. braziliensis*	253	554	806
LbrM17_V2.0520	*L. braziliensis*	54	846	899
LbrM17_V2.0530	*L. braziliensis*	160	1	160
LbrM17_V2.0530	*L. braziliensis*	162	177	338
LbrM17_V2.0530	*L. braziliensis*	62	477	538
LbrM17_V2.0530	*L. braziliensis*	41	977	1017
LbrM17_V2.0530	*L. braziliensis*	274	1032	1305
LbrM17_V2.0540	*L. braziliensis*	172	55	226
LbrM17_V2.0560	*L. braziliensis*	219	10	228
LbrM17_V2.0560	*L. braziliensis*	167	247	413
LbrM17_V2.0560	*L. braziliensis*	405	426	830
LbrM17_V2.0560	*L. braziliensis*	55	852	906
LbrM17_V2.0560	*L. braziliensis*	120	1015	1134
LbrM17_V2.0570	*L. braziliensis*	69	174	242
LbrM17_V2.0580	*L. braziliensis*	67	1	67
LbrM17_V2.0580	*L. braziliensis*	72	392	463
LbrM17_V2.0580	*L. braziliensis*	90	571	660
LbrM17_V2.0580	*L. braziliensis*	182	706	887
LbrM17_V2.0580	*L. braziliensis*	43	893	935
LbrM17_V2.0580	*L. braziliensis*	59	1081	1139
LbrM17_V2.0580	*L. braziliensis*	144	1160	1303
LbrM17_V2.0580	*L. braziliensis*	118	1355	1472
LbrM17_V2.0580	*L. braziliensis*	51	1562	1612

LbrM17_V2.0590	*L. braziliensis*	43	625	667
LbrM17_V2.0600	*L. braziliensis*	191	301	491
LbrM17_V2.0640	*L. braziliensis*	40	305	344
LbrM17_V2.0650	*L. braziliensis*	49	1	49
LbrM17_V2.0650	*L. braziliensis*	64	53	116
LbrM17_V2.0650	*L. braziliensis*	119	173	291
LbrM17_V2.0650	*L. braziliensis*	405	345	749
LbrM17_V2.0660	*L. braziliensis*	41	214	254
LbrM17_V2.0670	*L. braziliensis*	137	134	270
LbrM17_V2.0680	*L. braziliensis*	171	21	191
LbrM17_V2.0690	*L. braziliensis*	44	1	44
LbrM17_V2.0690	*L. braziliensis*	210	273	482
LbrM17_V2.0710	*L. braziliensis*	98	1	98
LbrM17_V2.0710	*L. braziliensis*	132	206	337
LbrM17_V2.0710	*L. braziliensis*	45	575	619
LbrM17_V2.0710	*L. braziliensis*	60	647	706
LbrM17_V2.0710	*L. braziliensis*	76	809	884
LbrM17_V2.0710	*L. braziliensis*	80	1049	1128
LbrM17_V2.0720	*L. braziliensis*	44	194	237
LbrM17_V2.0720	*L. braziliensis*	43	278	320
LbrM17_V2.0720	*L. braziliensis*	74	453	526
LbrM17_V2.0720	*L. braziliensis*	40	698	737
LbrM17_V2.0720	*L. braziliensis*	169	768	936
LbrM17_V2.0720	*L. braziliensis*	50	1047	1096
LbrM17_V2.0720	*L. braziliensis*	40	1207	1246
LbrM17_V2.0730	*L. braziliensis*	202	1	202
LbrM17_V2.0730	*L. braziliensis*	465	224	688
LbrM17_V2.0730	*L. braziliensis*	40	845	884
LbrM17_V2.0730	*L. braziliensis*	176	1000	1175
LbrM17_V2.0730	*L. braziliensis*	105	1191	1295
LbrM17_V2.0730	*L. braziliensis*	52	1466	1517
LbrM17_V2.0730	*L. braziliensis*	43	1587	1629
LbrM17_V2.0730	*L. braziliensis*	63	1646	1708
LbrM17_V2.0730	*L. braziliensis*	86	1725	1810
LbrM17_V2.0740	*L. braziliensis*	73	371	443
LbrM17_V2.0750	*L. braziliensis*	314	211	524
LbrM17_V2.0760	*L. braziliensis*	79	161	239
LbrM17_V2.0760	*L. braziliensis*	79	396	474
LbrM17_V2.0770	*L. braziliensis*	532	112	643
LbrM17_V2.0770	*L. braziliensis*	47	761	807
LbrM17_V2.0780	*L. braziliensis*	58	68	125
LbrM17_V2.0780	*L. braziliensis*	116	165	280
LbrM17_V2.0780	*L. braziliensis*	165	539	703
LbrM17_V2.0780	*L. braziliensis*	63	764	826
LbrM17_V2.0810	*L. braziliensis*	67	198	264
LbrM17_V2.0810	*L. braziliensis*	43	315	357
LbrM17_V2.0810	*L. braziliensis*	73	360	432

LbrM17_V2.0810	*L. braziliensis*	106	476	581
LbrM17_V2.0810	*L. braziliensis*	176	586	761
LbrM17_V2.0810	*L. braziliensis*	147	782	928
LbrM17_V2.0820	*L. braziliensis*	174	104	277
LbrM17_V2.0820	*L. braziliensis*	62	296	357
LbrM17_V2.0820	*L. braziliensis*	53	405	457
LbrM17_V2.0820	*L. braziliensis*	171	493	663
LbrM17_V2.0830	*L. braziliensis*	57	1	57
LbrM17_V2.0830	*L. braziliensis*	44	155	198
LbrM17_V2.0830	*L. braziliensis*	91	209	299
LbrM17_V2.0830	*L. braziliensis*	185	324	508
LbrM17_V2.0840	*L. braziliensis*	95	103	197
LbrM17_V2.0840	*L. braziliensis*	43	217	259
LbrM17_V2.0840	*L. braziliensis*	122	356	477
LbrM17_V2.0840	*L. braziliensis*	49	1009	1057
LbrM17_V2.0840	*L. braziliensis*	56	1210	1265
LbrM17_V2.0840	*L. braziliensis*	69	1317	1385
LbrM17_V2.0850	*L. braziliensis*	106	1	106
LbrM17_V2.0850	*L. braziliensis*	218	140	357
LbrM17_V2.0850	*L. braziliensis*	157	387	543
LbrM17_V2.0850	*L. braziliensis*	243	559	801
LbrM17_V2.0860	*L. braziliensis*	54	260	313
LbrM17_V2.0860	*L. braziliensis*	46	459	504
LbrM17_V2.0860	*L. braziliensis*	48	572	619
LbrM17_V2.0870	*L. braziliensis*	130	1	130
LbrM17_V2.0870	*L. braziliensis*	45	415	459
LbrM17_V2.0870	*L. braziliensis*	66	1073	1138
LbrM17_V2.0870	*L. braziliensis*	43	1190	1232
LbrM17_V2.0870	*L. braziliensis*	48	1575	1622
LbrM17_V2.0870	*L. braziliensis*	123	2020	2142
LbrM17_V2.0870	*L. braziliensis*	130	2327	2456
LbrM17_V2.0870	*L. braziliensis*	48	2480	2527
LbrM17_V2.0870	*L. braziliensis*	92	2530	2621
LbrM17_V2.0870	*L. braziliensis*	60	2826	2885
LbrM17_V2.0870	*L. braziliensis*	54	3270	3323
LbrM17_V2.0870	*L. braziliensis*	55	3462	3516
LbrM17_V2.0870	*L. braziliensis*	101	3568	3668
LbrM17_V2.0870	*L. braziliensis*	59	4087	4145
LbrM17_V2.0870	*L. braziliensis*	41	4874	4914
LbrM17_V2.0870	*L. braziliensis*	57	4975	5031
LbrM17_V2.0870	*L. braziliensis*	76	5079	5154
LbrM17_V2.0880	*L. braziliensis*	48	147	194
LbrM17_V2.0890	*L. braziliensis*	87	196	282
LbrM17_V2.0890	*L. braziliensis*	262	326	587
LbrM17_V2.0900	*L. braziliensis*	63	257	319
LbrM17_V2.0900	*L. braziliensis*	40	375	414
LbrM17_V2.0900	*L. braziliensis*	44	575	618

LbrM17_V2.0910	*L. braziliensis*	246	69	314
LbrM17_V2.0920	*L. braziliensis*	63	20	82
LbrM17_V2.0920	*L. braziliensis*	53	164	216
LbrM17_V2.0920	*L. braziliensis*	148	457	604
LbrM17_V2.0940	*L. braziliensis*	93	120	212
LbrM17_V2.0940	*L. braziliensis*	48	1866	1913
LbrM17_V2.0950	*L. braziliensis*	127	1	127
LbrM17_V2.0950	*L. braziliensis*	69	222	290
LbrM17_V2.0950	*L. braziliensis*	255	375	629
LbrM17_V2.0950	*L. braziliensis*	234	631	864
LbrM17_V2.0950	*L. braziliensis*	274	1177	1450
LbrM17_V2.0960	*L. braziliensis*	90	35	124
LbrM17_V2.0960	*L. braziliensis*	48	157	204
LbrM17_V2.0960	*L. braziliensis*	69	243	311
LbrM17_V2.0960	*L. braziliensis*	83	434	516
LbrM17_V2.0960	*L. braziliensis*	181	550	730
LbrM17_V2.0960	*L. braziliensis*	47	851	897
LbrM17_V2.0960	*L. braziliensis*	57	941	997
LbrM17_V2.1000	*L. braziliensis*	105	16	120
LbrM17_V2.1000	*L. braziliensis*	52	247	298
LbrM17_V2.1000	*L. braziliensis*	52	247	298
LbrM17_V2.1010	*L. braziliensis*	59	1	59
LbrM17_V2.1010	*L. braziliensis*	142	70	211
LbrM17_V2.1020	*L. braziliensis*	94	106	199
LbrM17_V2.1020	*L. braziliensis*	53	208	260
LbrM17_V2.1020	*L. braziliensis*	104	294	397
LbrM17_V2.1020	*L. braziliensis*	189	406	594
LbrM17_V2.1030	*L. braziliensis*	46	1	46
LbrM17_V2.1030	*L. braziliensis*	57	447	503
LbrM17_V2.1030	*L. braziliensis*	120	511	630
LbrM17_V2.1040	*L. braziliensis*	147	29	175
LbrM17_V2.1040	*L. braziliensis*	61	235	295
LbrM17_V2.1040	*L. braziliensis*	59	307	365
LbrM17_V2.1040	*L. braziliensis*	41	419	459
LbrM17_V2.1050	*L. braziliensis*	71	1	71
LbrM17_V2.1060	*L. braziliensis*	108	25	132
LbrM17_V2.1080	*L. braziliensis*	64	1	64
LbrM17_V2.1080	*L. braziliensis*	410	78	487
LbrM17_V2.1080	*L. braziliensis*	268	501	768
LbrM17_V2.1080	*L. braziliensis*	205	771	975
LbrM17_V2.1080	*L. braziliensis*	145	1344	1488
LbrM17_V2.1090	*L. braziliensis*	76	158	233
LbrM17_V2.1090	*L. braziliensis*	61	632	692
LbrM17_V2.1090	*L. braziliensis*	61	632	692
LbrM17_V2.1100	*L. braziliensis*	133	161	293
LbrM17_V2.1100	*L. braziliensis*	72	344	415
LbrM17_V2.1100	*L. braziliensis*	104	431	534

LbrM17_V2.1100	*L. braziliensis*	110	777	886
LbrM17_V2.1100	*L. braziliensis*	171	898	1068
LbrM17_V2.1100	*L. braziliensis*	40	1143	1182
LbrM17_V2.1100	*L. braziliensis*	287	1223	1509
LbrM17_V2.1100	*L. braziliensis*	90	1630	1719
LbrM17_V2.1100	*L. braziliensis*	115	1914	2028
LbrM17_V2.1100	*L. braziliensis*	50	2127	2176
LbrM17_V2.1100	*L. braziliensis*	48	2535	2582
LbrM17_V2.1100	*L. braziliensis*	82	2802	2883
LbrM17_V2.1100	*L. braziliensis*	52	2935	2986
LbrM17_V2.1100	*L. braziliensis*	75	3070	3144
LbrM17_V2.1100	*L. braziliensis*	113	3282	3394
LbrM17_V2.1100	*L. braziliensis*	261	3401	3661
LbrM17_V2.1100	*L. braziliensis*	236	3697	3932
LbrM17_V2.1100	*L. braziliensis*	44	4149	4192
LbrM17_V2.1100	*L. braziliensis*	40	4221	4260
LbrM17_V2.1100	*L. braziliensis*	42	4292	4333
LbrM17_V2.1100	*L. braziliensis*	72	4372	4443
LbrM17_V2.1100	*L. braziliensis*	94	4823	4916
LbrM17_V2.1100	*L. braziliensis*	135	5050	5184
LbrM17_V2.1100	*L. braziliensis*	193	5256	5448
LbrM17_V2.1100	*L. braziliensis*	67	5470	5536
LbrM17_V2.1100	*L. braziliensis*	108	5570	5677
LbrM17_V2.1100	*L. braziliensis*	87	5985	6071
LbrM17_V2.1100	*L. braziliensis*	54	6073	6126
LbrM17_V2.1100	*L. braziliensis*	159	6301	6459
LbrM17_V2.1100	*L. braziliensis*	61	6493	6553
LbrM17_V2.1110	*L. braziliensis*	67	1	67
LbrM17_V2.1110	*L. braziliensis*	133	236	368
LbrM17_V2.1110	*L. braziliensis*	53	501	553
LbrM17_V2.1140	*L. braziliensis*	485	1	485
LbrM17_V2.1150	*L. braziliensis*	86	174	259
LbrM17_V2.1160	*L. braziliensis*	46	276	321
LbrM17_V2.1170	*L. braziliensis*	57	306	362
LbrM17_V2.1170	*L. braziliensis*	74	435	508
LbrM17_V2.1170	*L. braziliensis*	122	527	648
LbrM17_V2.1170	*L. braziliensis*	53	671	723
LbrM17_V2.1180	*L. braziliensis*	42	45	86
LbrM17_V2.1180	*L. braziliensis*	43	259	301
LbrM17_V2.1180	*L. braziliensis*	43	259	301
LbrM17_V2.1190	*L. braziliensis*	55	1	55
LbrM17_V2.1190	*L. braziliensis*	48	161	208
LbrM17_V2.1190	*L. braziliensis*	43	354	396
LbrM17_V2.1190	*L. braziliensis*	81	519	599
LbrM17_V2.1190	*L. braziliensis*	41	674	714
LbrM17_V2.1200	*L. braziliensis*	525	1	525
LbrM17_V2.1200	*L. braziliensis*	44	617	660

LbrM17_V2.1200	*L. braziliensis*	75	818	892
LbrM17_V2.1200	*L. braziliensis*	62	933	994
LbrM17_V2.1200	*L. braziliensis*	93	1092	1184
LbrM17_V2.1200	*L. braziliensis*	61	1220	1280
LbrM17_V2.1210	*L. braziliensis*	43	361	403
LbrM17_V2.1220	*L. braziliensis*	41	228	268
LbrM17_V2.1220	*L. braziliensis*	60	369	428
LbrM17_V2.1220	*L. braziliensis*	237	1171	1407
LbrM17_V2.1240	*L. braziliensis*	126	132	257
LbrM17_V2.1240	*L. braziliensis*	97	423	519
LbrM17_V2.1240	*L. braziliensis*	43	862	904
LbrM17_V2.1240	*L. braziliensis*	99	1075	1173
LbrM17_V2.1250	*L. braziliensis*	218	96	313
LbrM17_V2.1250	*L. braziliensis*	627	317	943
LbrM17_V2.1250	*L. braziliensis*	251	981	1231
LbrM17_V2.1250	*L. braziliensis*	58	1238	1295
LbrM17_V2.1260	*L. braziliensis*	84	135	218
LbrM17_V2.1260	*L. braziliensis*	56	463	518
LbrM17_V2.1260	*L. braziliensis*	79	806	884
LbrM17_V2.1280	*L. braziliensis*	95	136	230
LbrM17_V2.1280	*L. braziliensis*	151	317	467
LbrM17_V2.1280	*L. braziliensis*	60	550	609
LbrM17_V2.1290	*L. braziliensis*	251	174	424
LbrM17_V2.1290	*L. braziliensis*	64	442	505
LbrM17_V2.1290	*L. braziliensis*	405	518	922
LbrM17_V2.1300	*L. braziliensis*	103	60	162
LbrM17_V2.1300	*L. braziliensis*	66	225	290
LbrM17_V2.1300	*L. braziliensis*	191	329	519
LbrM17_V2.1300	*L. braziliensis*	62	579	640
LbrM17_V2.1310	*L. braziliensis*	286	1	286
LbrM17_V2.1310	*L. braziliensis*	112	294	405
LbrM17_V2.1310	*L. braziliensis*	422	427	848
LbrM17_V2.1320	*L. braziliensis*	114	1	114
LbrM17_V2.1330	*L. braziliensis*	43	1	43
LbrM17_V2.1340	*L. braziliensis*	281	77	357
LbrM17_V2.1340	*L. braziliensis*	167	359	525
LbrM17_V2.1350	*L. braziliensis*	50	1	50
LbrM17_V2.1350	*L. braziliensis*	51	193	243
LbrM17_V2.1350	*L. braziliensis*	57	294	350
LbrM17_V2.1360	*L. braziliensis*	42	187	228
LbrM17_V2.1360	*L. braziliensis*	92	385	476
LbrM17_V2.1360	*L. braziliensis*	175	653	827
LbrM17_V2.1360	*L. braziliensis*	41	1345	1385
LbrM17_V2.1370	*L. braziliensis*	51	220	270
LbrM17_V2.1380	*L. braziliensis*	87	44	130
LbrM17_V2.1390	*L. braziliensis*	52	1	52
LbrM17_V2.1400	*L. braziliensis*	67	273	339

LbrM17_V2.1400	*L. braziliensis*	43	857	899
LbrM17_V2.1410	*L. braziliensis*	43	440	482
LbrM17_V2.1410	*L. braziliensis*	60	959	1018
LbrM17_V2.1410	*L. braziliensis*	40	1041	1080
LbrM17_V2.1420	*L. braziliensis*	65	260	324
LbrM17_V2.1450	*L. braziliensis*	76	596	671
LbrM17_V2.1480	*L. braziliensis*	51	80	130
LbrM17_V2.1490	*L. braziliensis*	48	235	282
LbrM17_V2.1500	*L. braziliensis*	70	229	298
LbrM17_V2.1500	*L. braziliensis*	373	493	865
LbrM17_V2.1520	*L. braziliensis*	71	116	186
LbrM17_V2.1520	*L. braziliensis*	42	244	285
LbrM17_V2.1520	*L. braziliensis*	42	244	285
LbrM17_V2.1540	*L. braziliensis*	44	1	44
LbrM17_V2.1550	*L. braziliensis*	40	1	40
LbrM17_V2.1550	*L. braziliensis*	59	425	483
LbrM17_V2.1570	*L. braziliensis*	210	48	257
LbrM17_V2.1580	*L. braziliensis*	58	1	58
LbrM17_V2.1600	*L. braziliensis*	70	72	141
LbrM17_V2.1620	*L. braziliensis*	184	59	242
LbrM18_V2.0010	*L. braziliensis*	58	447	504
LbrM18_V2.0020	*L. braziliensis*	59	681	739
LbrM18_V2.0030	*L. braziliensis*	41	203	243
LbrM18_V2.0040	*L. braziliensis*	56	82	137
LbrM18_V2.0040	*L. braziliensis*	42	179	220
LbrM18_V2.0080	*L. braziliensis*	43	115	157
LbrM18_V2.0090	*L. braziliensis*	56	110	165
LbrM18_V2.0090	*L. braziliensis*	151	188	338
LbrM18_V2.0090	*L. braziliensis*	287	364	650
LbrM18_V2.0100	*L. braziliensis*	41	402	442
LbrM18_V2.0120	*L. braziliensis*	72	123	194
LbrM18_V2.0130	*L. braziliensis*	50	312	361
LbrM18_V2.0150	*L. braziliensis*	55	292	346
LbrM18_V2.0150	*L. braziliensis*	53	589	641
LbrM18_V2.0150	*L. braziliensis*	53	589	641
LbrM18_V2.0160	*L. braziliensis*	44	542	585
LbrM18_V2.0160	*L. braziliensis*	47	700	746
LbrM18_V2.0160	*L. braziliensis*	47	700	746
LbrM18_V2.0180	*L. braziliensis*	44	655	698
LbrM18_V2.0190	*L. braziliensis*	120	209	328
LbrM18_V2.0200	*L. braziliensis*	49	209	257
LbrM18_V2.0210	*L. braziliensis*	48	199	246
LbrM18_V2.0230	*L. braziliensis*	58	173	230
LbrM18_V2.0240	*L. braziliensis*	323	82	404
LbrM18_V2.0250	*L. braziliensis*	124	1	124
LbrM18_V2.0260	*L. braziliensis*	76	250	325
LbrM18_V2.0270	*L. braziliensis*	51	77	127

LbrM18_V2.0290	*L. braziliensis*	43	1	43
LbrM18_V2.0300	*L. braziliensis*	88	438	525
LbrM18_V2.0310	*L. braziliensis*	52	14	65
LbrM18_V2.0310	*L. braziliensis*	41	187	227
LbrM18_V2.0310	*L. braziliensis*	41	187	227
LbrM18_V2.0320	*L. braziliensis*	112	137	248
LbrM18_V2.0320	*L. braziliensis*	55	309	363
LbrM18_V2.0320	*L. braziliensis*	55	309	363
LbrM18_V2.0350	*L. braziliensis*	74	102	175
LbrM18_V2.0360	*L. braziliensis*	71	1	71
LbrM18_V2.0360	*L. braziliensis*	41	364	404
LbrM18_V2.0370	*L. braziliensis*	121	1	121
LbrM18_V2.0390	*L. braziliensis*	52	155	206
LbrM18_V2.0400	*L. braziliensis*	72	1	72
LbrM18_V2.0400	*L. braziliensis*	68	428	495
LbrM18_V2.0400	*L. braziliensis*	44	967	1010
LbrM18_V2.0410	*L. braziliensis*	787	1	787
LbrM18_V2.0410	*L. braziliensis*	174	832	1005
LbrM18_V2.0410	*L. braziliensis*	190	1167	1356
LbrM18_V2.0430	*L. braziliensis*	57	98	154
LbrM18_V2.0440	*L. braziliensis*	61	38	98
LbrM18_V2.0450	*L. braziliensis*	101	111	211
LbrM18_V2.0480	*L. braziliensis*	101	111	211
LbrM18_V2.0500	*L. braziliensis*	81	27	107
LbrM18_V2.0500	*L. braziliensis*	52	218	269
LbrM18_V2.0500	*L. braziliensis*	52	218	269
LbrM18_V2.0520	*L. braziliensis*	43	113	155
LbrM18_V2.0530	*L. braziliensis*	60	1	60
LbrM18_V2.0530	*L. braziliensis*	218	129	346
LbrM18_V2.0540	*L. braziliensis*	133	1	133
LbrM18_V2.0550	*L. braziliensis*	100	143	242
LbrM18_V2.0560	*L. braziliensis*	120	1	120
LbrM18_V2.0560	*L. braziliensis*	70	312	381
LbrM18_V2.0560	*L. braziliensis*	70	312	381
LbrM18_V2.0580	*L. braziliensis*	77	1	77
LbrM18_V2.0580	*L. braziliensis*	150	86	235
LbrM18_V2.0580	*L. braziliensis*	50	251	300
LbrM18_V2.0580	*L. braziliensis*	183	324	506
LbrM18_V2.0580	*L. braziliensis*	105	508	612
LbrM18_V2.0590	*L. braziliensis*	41	132	172
LbrM18_V2.0590	*L. braziliensis*	141	382	522
LbrM18_V2.0600	*L. braziliensis*	60	419	478
LbrM18_V2.0610	*L. braziliensis*	106	215	320
LbrM18_V2.0630	*L. braziliensis*	43	566	608
LbrM18_V2.0650	*L. braziliensis*	162	1	162
LbrM18_V2.0650	*L. braziliensis*	212	216	427
LbrM18_V2.0660	*L. braziliensis*	564	1	564

LbrM18_V2.0680	*L. braziliensis*	48	27	74
LbrM18_V2.0680	*L. braziliensis*	77	120	196
LbrM18_V2.0700	*L. braziliensis*	49	1	49
LbrM18_V2.0710	*L. braziliensis*	323	1	323
LbrM18_V2.0710	*L. braziliensis*	150	343	492
LbrM18_V2.0730	*L. braziliensis*	104	59	162
LbrM18_V2.0730	*L. braziliensis*	59	251	309
LbrM18_V2.0730	*L. braziliensis*	64	352	415
LbrM18_V2.0730	*L. braziliensis*	57	462	518
LbrM18_V2.0730	*L. braziliensis*	96	603	698
LbrM18_V2.0730	*L. braziliensis*	83	804	886
LbrM18_V2.0730	*L. braziliensis*	53	982	1034
LbrM18_V2.0730	*L. braziliensis*	45	1196	1240
LbrM18_V2.0740	*L. braziliensis*	114	596	709
LbrM18_V2.0750	*L. braziliensis*	70	1	70
LbrM18_V2.0750	*L. braziliensis*	128	89	216
LbrM18_V2.0750	*L. braziliensis*	120	517	636
LbrM18_V2.0750	*L. braziliensis*	103	650	752
LbrM18_V2.0760	*L. braziliensis*	52	1	52
LbrM18_V2.0780	*L. braziliensis*	82	197	278
LbrM18_V2.0780	*L. braziliensis*	63	331	393
LbrM18_V2.0780	*L. braziliensis*	141	624	764
LbrM18_V2.0780	*L. braziliensis*	93	771	863
LbrM18_V2.0780	*L. braziliensis*	76	934	1009
LbrM18_V2.0780	*L. braziliensis*	52	1117	1168
LbrM18_V2.0780	*L. braziliensis*	222	1239	1460
LbrM18_V2.0780	*L. braziliensis*	41	1499	1539
LbrM18_V2.0780	*L. braziliensis*	70	1570	1639
LbrM18_V2.0810	*L. braziliensis*	83	165	247
LbrM18_V2.0810	*L. braziliensis*	153	367	519
LbrM18_V2.0810	*L. braziliensis*	42	682	723
LbrM18_V2.0820	*L. braziliensis*	119	1	119
LbrM18_V2.0820	*L. braziliensis*	55	121	175
LbrM18_V2.0830	*L. braziliensis*	41	361	401
LbrM18_V2.0840	*L. braziliensis*	105	43	147
LbrM18_V2.0840	*L. braziliensis*	105	334	438
LbrM18_V2.0840	*L. braziliensis*	49	476	524
LbrM18_V2.0850	*L. braziliensis*	78	1	78
LbrM18_V2.0860	*L. braziliensis*	51	88	138
LbrM18_V2.0860	*L. braziliensis*	389	189	577
LbrM18_V2.0860	*L. braziliensis*	114	682	795
LbrM18_V2.0860	*L. braziliensis*	178	927	1104
LbrM18_V2.0860	*L. braziliensis*	253	1150	1402
LbrM18_V2.0860	*L. braziliensis*	61	1409	1469
LbrM18_V2.0860	*L. braziliensis*	222	1500	1721
LbrM18_V2.0870	*L. braziliensis*	41	147	187
LbrM18_V2.0880	*L. braziliensis*	47	67	113

LbrM18_V2.0880	*L. braziliensis*	73	133	205
LbrM18_V2.0880	*L. braziliensis*	73	133	205
LbrM18_V2.0890	*L. braziliensis*	52	75	126
LbrM18_V2.0890	*L. braziliensis*	53	133	185
LbrM18_V2.0890	*L. braziliensis*	53	133	185
LbrM18_V2.0910	*L. braziliensis*	41	1827	1867
LbrM18_V2.0920	*L. braziliensis*	44	244	287
LbrM18_V2.0920	*L. braziliensis*	42	714	755
LbrM18_V2.0920	*L. braziliensis*	42	714	755
LbrM18_V2.0930	*L. braziliensis*	55	97	151
LbrM18_V2.0930	*L. braziliensis*	126	342	467
LbrM18_V2.0940	*L. braziliensis*	40	225	264
LbrM18_V2.0940	*L. braziliensis*	54	552	605
LbrM18_V2.0940	*L. braziliensis*	54	737	790
LbrM18_V2.0940	*L. braziliensis*	64	806	869
LbrM18_V2.0950	*L. braziliensis*	40	138	177
LbrM18_V2.0960	*L. braziliensis*	85	49	133
LbrM18_V2.0960	*L. braziliensis*	41	801	841
LbrM18_V2.0970	*L. braziliensis*	96	64	159
LbrM18_V2.0970	*L. braziliensis*	41	188	228
LbrM18_V2.0970	*L. braziliensis*	49	334	382
LbrM18_V2.0970	*L. braziliensis*	52	629	680
LbrM18_V2.0970	*L. braziliensis*	110	926	1035
LbrM18_V2.0970	*L. braziliensis*	84	1053	1136
LbrM18_V2.0980	*L. braziliensis*	113	430	542
LbrM18_V2.0990	*L. braziliensis*	101	85	185
LbrM18_V2.0990	*L. braziliensis*	204	234	437
LbrM18_V2.0990	*L. braziliensis*	144	444	587
LbrM18_V2.0990	*L. braziliensis*	72	626	697
LbrM18_V2.0990	*L. braziliensis*	194	700	893
LbrM18_V2.1010	*L. braziliensis*	47	319	365
LbrM18_V2.1020	*L. braziliensis*	59	143	201
LbrM18_V2.1020	*L. braziliensis*	40	1007	1046
LbrM18_V2.1020	*L. braziliensis*	128	1141	1268
LbrM18_V2.1030	*L. braziliensis*	70	40	109
LbrM18_V2.1030	*L. braziliensis*	170	139	308
LbrM18_V2.1030	*L. braziliensis*	64	409	472
LbrM18_V2.1040	*L. braziliensis*	191	1	191
LbrM18_V2.1060	*L. braziliensis*	41	1	41
LbrM18_V2.1080	*L. braziliensis*	254	1	254
LbrM18_V2.1080	*L. braziliensis*	47	277	323
LbrM18_V2.1080	*L. braziliensis*	190	335	524
LbrM18_V2.1080	*L. braziliensis*	179	541	719
LbrM18_V2.1080	*L. braziliensis*	44	721	764
LbrM18_V2.1080	*L. braziliensis*	166	784	949
LbrM18_V2.1080	*L. braziliensis*	335	1054	1388
LbrM18_V2.1080	*L. braziliensis*	91	1425	1515

LbrM18_V2.1100	*L. braziliensis*	44	666	709
LbrM18_V2.1110	*L. braziliensis*	92	1	92
LbrM18_V2.1120	*L. braziliensis*	48	14	61
LbrM18_V2.1120	*L. braziliensis*	151	105	255
LbrM18_V2.1120	*L. braziliensis*	185	421	605
LbrM18_V2.1130	*L. braziliensis*	376	20	395
LbrM18_V2.1130	*L. braziliensis*	69	543	611
LbrM18_V2.1130	*L. braziliensis*	55	629	683
LbrM18_V2.1150	*L. braziliensis*	61	207	267
LbrM18_V2.1150	*L. braziliensis*	47	915	961
LbrM18_V2.1190	*L. braziliensis*	63	189	251
LbrM18_V2.1200	*L. braziliensis*	49	131	179
LbrM18_V2.1210	*L. braziliensis*	41	154	194
LbrM18_V2.1220	*L. braziliensis*	105	84	188
LbrM18_V2.1240	*L. braziliensis*	146	1	146
LbrM18_V2.1240	*L. braziliensis*	687	155	841
LbrM18_V2.1240	*L. braziliensis*	146	871	1016
LbrM18_V2.1250	*L. braziliensis*	79	1	79
LbrM18_V2.1280	*L. braziliensis*	72	1	72
LbrM18_V2.1280	*L. braziliensis*	295	120	414
LbrM18_V2.1280	*L. braziliensis*	59	441	499
LbrM18_V2.1280	*L. braziliensis*	195	537	731
LbrM18_V2.1280	*L. braziliensis*	87	768	854
LbrM18_V2.1280	*L. braziliensis*	378	876	1253
LbrM18_V2.1290	*L. braziliensis*	43	1	43
LbrM18_V2.1290	*L. braziliensis*	49	161	209
LbrM18_V2.1290	*L. braziliensis*	50	534	583
LbrM18_V2.1300	*L. braziliensis*	184	154	337
LbrM18_V2.1300	*L. braziliensis*	46	343	388
LbrM18_V2.1310	*L. braziliensis*	43	654	696
LbrM18_V2.1320	*L. braziliensis*	45	36	80
LbrM18_V2.1320	*L. braziliensis*	164	90	253
LbrM18_V2.1320	*L. braziliensis*	40	337	376
LbrM18_V2.1330	*L. braziliensis*	99	1	99
LbrM18_V2.1330	*L. braziliensis*	105	115	219
LbrM18_V2.1330	*L. braziliensis*	93	273	365
LbrM18_V2.1330	*L. braziliensis*	299	467	765
LbrM18_V2.1340	*L. braziliensis*	53	1	53
LbrM18_V2.1340	*L. braziliensis*	175	133	307
LbrM18_V2.1340	*L. braziliensis*	64	323	386
LbrM18_V2.1340	*L. braziliensis*	90	420	509
LbrM18_V2.1340	*L. braziliensis*	181	528	708
LbrM18_V2.1340	*L. braziliensis*	55	757	811
LbrM18_V2.1370	*L. braziliensis*	41	1	41
LbrM18_V2.1370	*L. braziliensis*	83	104	186
LbrM18_V2.1370	*L. braziliensis*	65	275	339
LbrM18_V2.1370	*L. braziliensis*	107	456	562

LbrM18_V2.1390	*L. braziliensis*	303	1	303
LbrM18_V2.1390	*L. braziliensis*	312	351	662
LbrM18_V2.1390	*L. braziliensis*	268	695	962
LbrM18_V2.1400	*L. braziliensis*	84	513	596
LbrM18_V2.1400	*L. braziliensis*	84	738	821
LbrM18_V2.1410	*L. braziliensis*	50	1	50
LbrM18_V2.1410	*L. braziliensis*	76	282	357
LbrM18_V2.1410	*L. braziliensis*	76	282	357
LbrM18_V2.1420	*L. braziliensis*	47	1	47
LbrM18_V2.1440	*L. braziliensis*	180	72	251
LbrM18_V2.1440	*L. braziliensis*	137	255	391
LbrM18_V2.1440	*L. braziliensis*	227	407	633
LbrM18_V2.1440	*L. braziliensis*	83	671	753
LbrM18_V2.1440	*L. braziliensis*	44	797	840
LbrM18_V2.1440	*L. braziliensis*	64	847	910
LbrM18_V2.1440	*L. braziliensis*	92	943	1034
LbrM18_V2.1450	*L. braziliensis*	66	87	152
LbrM18_V2.1450	*L. braziliensis*	320	530	849
LbrM18_V2.1460	*L. braziliensis*	500	113	612
LbrM18_V2.1460	*L. braziliensis*	116	794	909
LbrM18_V2.1460	*L. braziliensis*	66	921	986
LbrM18_V2.1460	*L. braziliensis*	109	994	1102
LbrM18_V2.1470	*L. braziliensis*	47	62	108
LbrM18_V2.1480	*L. braziliensis*	170	1	170
LbrM18_V2.1480	*L. braziliensis*	689	175	863
LbrM18_V2.1480	*L. braziliensis*	60	953	1012
LbrM18_V2.1480	*L. braziliensis*	119	1150	1268
LbrM18_V2.1480	*L. braziliensis*	63	1364	1426
LbrM18_V2.1480	*L. braziliensis*	108	1487	1594
LbrM18_V2.1480	*L. braziliensis*	345	1672	2016
LbrM18_V2.1490	*L. braziliensis*	231	1	231
LbrM18_V2.1490	*L. braziliensis*	101	269	369
LbrM18_V2.1490	*L. braziliensis*	63	602	664
LbrM18_V2.1500	*L. braziliensis*	41	156	196
LbrM18_V2.1510	*L. braziliensis*	163	1	163
LbrM18_V2.1540	*L. braziliensis*	65	1	65
LbrM18_V2.1540	*L. braziliensis*	96	151	246
LbrM18_V2.1540	*L. braziliensis*	125	407	531
LbrM18_V2.1550	*L. braziliensis*	73	1	73
LbrM18_V2.1560	*L. braziliensis*	62	1	62
LbrM18_V2.1560	*L. braziliensis*	60	161	220
LbrM18_V2.1560	*L. braziliensis*	74	319	392
LbrM18_V2.1560	*L. braziliensis*	138	408	545
LbrM18_V2.1560	*L. braziliensis*	179	758	936
LbrM18_V2.1560	*L. braziliensis*	137	957	1093
LbrM18_V2.1580	*L. braziliensis*	59	208	266
LbrM18_V2.1630	*L. braziliensis*	85	323	407

LbrM18_V2.1630	*L. braziliensis*	220	572	791
LbrM18_V2.1640	*L. braziliensis*	150	1	150
LbrM18_V2.1640	*L. braziliensis*	44	611	654
LbrM18_V2.1670	*L. braziliensis*	74	50	123
LbrM18_V2.1700	*L. braziliensis*	40	229	268
LbrM18_V2.1710	*L. braziliensis*	57	152	208
LbrM18_V2.1710	*L. braziliensis*	221	217	437
LbrM18_V2.1710	*L. braziliensis*	54	452	505
LbrM18_V2.1730	*L. braziliensis*	55	272	326
LbrM18_V2.1740	*L. braziliensis*	82	1	82
LbrM18_V2.1750	*L. braziliensis*	213	125	337
LbrM18_V2.1750	*L. braziliensis*	118	358	475
LbrM18_V2.1750	*L. braziliensis*	67	556	622
LbrM18_V2.1750	*L. braziliensis*	67	648	714
LbrM18_V2.1750	*L. braziliensis*	85	738	822
LbrM18_V2.1750	*L. braziliensis*	114	879	992
LbrM18_V2.1750	*L. braziliensis*	77	1043	1119
LbrM18_V2.1750	*L. braziliensis*	45	1175	1219
LbrM18_V2.1750	*L. braziliensis*	45	1225	1269
LbrM18_V2.1750	*L. braziliensis*	130	1384	1513
LbrM18_V2.1750	*L. braziliensis*	43	1521	1563
LbrM18_V2.1750	*L. braziliensis*	112	1618	1729
LbrM18_V2.1750	*L. braziliensis*	70	1738	1807
LbrM18_V2.1750	*L. braziliensis*	92	1895	1986
LbrM18_V2.1760	*L. braziliensis*	56	1	56
LbrM18_V2.1760	*L. braziliensis*	134	73	206
LbrM18_V2.1760	*L. braziliensis*	60	303	362
LbrM18_V2.1760	*L. braziliensis*	58	402	459
LbrM18_V2.1760	*L. braziliensis*	92	677	768
LbrM18_V2.1760	*L. braziliensis*	55	880	934
LbrM18_V2.1760	*L. braziliensis*	230	938	1167
LbrM18_V2.1760	*L. braziliensis*	136	1506	1641
LbrM18_V2.1760	*L. braziliensis*	144	1668	1811
LbrM18_V2.1760	*L. braziliensis*	90	1859	1948
LbrM18_V2.1760	*L. braziliensis*	82	2114	2195
LbrM18_V2.1760	*L. braziliensis*	152	2223	2374
LbrM18_V2.1760	*L. braziliensis*	143	2415	2557
LbrM18_V2.1760	*L. braziliensis*	152	2642	2793
LbrM18_V2.1770	*L. braziliensis*	79	62	140
LbrM18_V2.1770	*L. braziliensis*	50	190	239
LbrM18_V2.1770	*L. braziliensis*	82	584	665
LbrM18_V2.1770	*L. braziliensis*	46	738	783
LbrM18_V2.1780	*L. braziliensis*	187	1	187
LbrM18_V2.1780	*L. braziliensis*	445	207	651
LbrM18_V2.1780	*L. braziliensis*	191	680	870
LbrM18_V2.1780	*L. braziliensis*	122	1071	1192
LbrM18_V2.1780	*L. braziliensis*	336	1333	1668

LbrM19_V2.0010	*L. braziliensis*	152	1	152
LbrM19_V2.0010	*L. braziliensis*	51	245	295
LbrM19_V2.0010	*L. braziliensis*	87	303	389
LbrM19_V2.0010	*L. braziliensis*	259	574	832
LbrM19_V2.0010	*L. braziliensis*	73	850	922
LbrM19_V2.0010	*L. braziliensis*	88	936	1023
LbrM19_V2.0010	*L. braziliensis*	57	1174	1230
LbrM19_V2.0010	*L. braziliensis*	112	1550	1661
LbrM19_V2.0010	*L. braziliensis*	49	1864	1912
LbrM19_V2.0010	*L. braziliensis*	60	1964	2023
LbrM19_V2.0020	*L. braziliensis*	160	107	266
LbrM19_V2.0030	*L. braziliensis*	403	1	403
LbrM19_V2.0030	*L. braziliensis*	69	429	497
LbrM19_V2.0040	*L. braziliensis*	40	1	40
LbrM19_V2.0040	*L. braziliensis*	236	90	325
LbrM19_V2.0040	*L. braziliensis*	49	550	598
LbrM19_V2.0040	*L. braziliensis*	41	706	746
LbrM19_V2.0050	*L. braziliensis*	134	87	220
LbrM19_V2.0060	*L. braziliensis*	44	442	485
LbrM19_V2.0070	*L. braziliensis*	251	1	251
LbrM19_V2.0070	*L. braziliensis*	243	271	513
LbrM19_V2.0070	*L. braziliensis*	78	574	651
LbrM19_V2.0070	*L. braziliensis*	49	781	829
LbrM19_V2.0070	*L. braziliensis*	173	864	1036
LbrM19_V2.0090	*L. braziliensis*	51	37	87
LbrM19_V2.0090	*L. braziliensis*	60	375	434
LbrM19_V2.0090	*L. braziliensis*	60	375	434
LbrM19_V2.0120	*L. braziliensis*	179	1	179
LbrM19_V2.0130	*L. braziliensis*	74	438	511
LbrM19_V2.0130	*L. braziliensis*	56	597	652
LbrM19_V2.0150	*L. braziliensis*	181	95	275
LbrM19_V2.0150	*L. braziliensis*	59	297	355
LbrM19_V2.0150	*L. braziliensis*	314	390	703
LbrM19_V2.0160	*L. braziliensis*	72	158	229
LbrM19_V2.0160	*L. braziliensis*	54	990	1043
LbrM19_V2.0170	*L. braziliensis*	203	1	203
LbrM19_V2.0170	*L. braziliensis*	356	281	636
LbrM19_V2.0170	*L. braziliensis*	153	660	812
LbrM19_V2.0170	*L. braziliensis*	42	842	883
LbrM19_V2.0180	*L. braziliensis*	96	138	233
LbrM19_V2.0220	*L. braziliensis*	61	51	111
LbrM19_V2.0230	*L. braziliensis*	49	302	350
LbrM19_V2.0230	*L. braziliensis*	47	578	624
LbrM19_V2.0230	*L. braziliensis*	47	578	624
LbrM19_V2.0240	*L. braziliensis*	85	1	85
LbrM19_V2.0240	*L. braziliensis*	55	161	215
LbrM19_V2.0240	*L. braziliensis*	55	161	215

LbrM19_V2.0250	*L. braziliensis*	77	1	77
LbrM19_V2.0250	*L. braziliensis*	512	125	636
LbrM19_V2.0250	*L. braziliensis*	104	740	843
LbrM19_V2.0260	*L. braziliensis*	46	132	177
LbrM19_V2.0260	*L. braziliensis*	96	352	447
LbrM19_V2.0270	*L. braziliensis*	61	378	438
LbrM19_V2.0270	*L. braziliensis*	42	586	627
LbrM19_V2.0270	*L. braziliensis*	42	586	627
LbrM19_V2.0280	*L. braziliensis*	63	239	301
LbrM19_V2.0290	*L. braziliensis*	54	254	307
LbrM19_V2.0310	*L. braziliensis*	120	99	218
LbrM19_V2.0320	*L. braziliensis*	143	1	143
LbrM19_V2.0320	*L. braziliensis*	95	149	243
LbrM19_V2.0330	*L. braziliensis*	85	1	85
LbrM19_V2.0330	*L. braziliensis*	153	101	253
LbrM19_V2.0340	*L. braziliensis*	57	62	118
LbrM19_V2.0350	*L. braziliensis*	57	62	118
LbrM19_V2.0360	*L. braziliensis*	47	1	47
LbrM19_V2.0370	*L. braziliensis*	53	1	53
LbrM19_V2.0380	*L. braziliensis*	131	1	131
LbrM19_V2.0380	*L. braziliensis*	62	262	323
LbrM19_V2.0390	*L. braziliensis*	376	57	432
LbrM19_V2.0400	*L. braziliensis*	49	1	49
LbrM19_V2.0400	*L. braziliensis*	58	149	206
LbrM19_V2.0400	*L. braziliensis*	53	243	295
LbrM19_V2.0410	*L. braziliensis*	112	89	200
LbrM19_V2.0420	*L. braziliensis*	122	1	122
LbrM19_V2.0440	*L. braziliensis*	43	211	253
LbrM19_V2.0450	*L. braziliensis*	191	371	561
LbrM19_V2.0480	*L. braziliensis*	83	116	198
LbrM19_V2.0480	*L. braziliensis*	50	454	503
LbrM19_V2.0480	*L. braziliensis*	97	1389	1485
LbrM19_V2.0490	*L. braziliensis*	103	305	407
LbrM19_V2.0500	*L. braziliensis*	87	1	87
LbrM19_V2.0500	*L. braziliensis*	43	121	163
LbrM19_V2.0550	*L. braziliensis*	107	1	107
LbrM19_V2.0550	*L. braziliensis*	83	437	519
LbrM19_V2.0550	*L. braziliensis*	59	614	672
LbrM19_V2.0560	*L. braziliensis*	52	190	241
LbrM19_V2.0560	*L. braziliensis*	94	243	336
LbrM19_V2.0560	*L. braziliensis*	73	670	742
LbrM19_V2.0570	*L. braziliensis*	42	1	42
LbrM19_V2.0570	*L. braziliensis*	157	264	420
LbrM19_V2.0570	*L. braziliensis*	42	517	558
LbrM19_V2.0580	*L. braziliensis*	387	1	387
LbrM19_V2.0580	*L. braziliensis*	343	461	803
LbrM19_V2.0590	*L. braziliensis*	196	431	626

LbrM19_V2.0610	*L. braziliensis*	61	1	61
LbrM19_V2.0610	*L. braziliensis*	94	135	228
LbrM19_V2.0620	*L. braziliensis*	75	171	245
LbrM19_V2.0620	*L. braziliensis*	98	295	392
LbrM19_V2.0630	*L. braziliensis*	40	100	139
LbrM19_V2.0640	*L. braziliensis*	63	186	248
LbrM19_V2.0640	*L. braziliensis*	51	399	449
LbrM19_V2.0640	*L. braziliensis*	51	399	449
LbrM19_V2.0660	*L. braziliensis*	59	86	144
LbrM19_V2.0670	*L. braziliensis*	56	46	101
LbrM19_V2.0680	*L. braziliensis*	65	1	65
LbrM19_V2.0680	*L. braziliensis*	121	90	210
LbrM19_V2.0680	*L. braziliensis*	203	226	428
LbrM19_V2.0680	*L. braziliensis*	43	442	484
LbrM19_V2.0680	*L. braziliensis*	174	498	671
LbrM19_V2.0680	*L. braziliensis*	50	701	750
LbrM19_V2.0680	*L. braziliensis*	212	786	997
LbrM19_V2.0680	*L. braziliensis*	143	1006	1148
LbrM19_V2.0680	*L. braziliensis*	40	1307	1346
LbrM19_V2.0680	*L. braziliensis*	108	1438	1545
LbrM19_V2.0680	*L. braziliensis*	68	1575	1642
LbrM19_V2.0680	*L. braziliensis*	66	1802	1867
LbrM19_V2.0690	*L. braziliensis*	168	1	168
LbrM19_V2.0690	*L. braziliensis*	275	181	455
LbrM19_V2.0690	*L. braziliensis*	44	486	529
LbrM19_V2.0690	*L. braziliensis*	135	546	680
LbrM19_V2.0690	*L. braziliensis*	149	723	871
LbrM19_V2.0690	*L. braziliensis*	87	890	976
LbrM19_V2.0700	*L. braziliensis*	336	187	522
LbrM19_V2.0700	*L. braziliensis*	52	708	759
LbrM19_V2.0700	*L. braziliensis*	46	1040	1085
LbrM19_V2.0700	*L. braziliensis*	71	1106	1176
LbrM19_V2.0700	*L. braziliensis*	93	1214	1306
LbrM19_V2.0700	*L. braziliensis*	153	1491	1643
LbrM19_V2.0710	*L. braziliensis*	54	1	54
LbrM19_V2.0720	*L. braziliensis*	44	156	199
LbrM19_V2.0730	*L. braziliensis*	40	1	40
LbrM19_V2.0740	*L. braziliensis*	63	63	125
LbrM19_V2.0750	*L. braziliensis*	60	37	96
LbrM19_V2.0750	*L. braziliensis*	125	159	283
LbrM19_V2.0750	*L. braziliensis*	61	285	345
LbrM19_V2.0750	*L. braziliensis*	45	477	521
LbrM19_V2.0750	*L. braziliensis*	58	822	879
LbrM19_V2.0750	*L. braziliensis*	50	1086	1135
LbrM19_V2.0760	*L. braziliensis*	53	1	53
LbrM19_V2.0760	*L. braziliensis*	54	230	283
LbrM19_V2.0760	*L. braziliensis*	69	286	354

LbrM19_V2.0770	*L. braziliensis*	152	58	209
LbrM19_V2.0770	*L. braziliensis*	452	211	662
LbrM19_V2.0770	*L. braziliensis*	42	713	754
LbrM19_V2.0770	*L. braziliensis*	58	819	876
LbrM19_V2.0780	*L. braziliensis*	185	51	235
LbrM19_V2.0790	*L. braziliensis*	63	117	179
LbrM19_V2.0790	*L. braziliensis*	92	266	357
LbrM19_V2.0800	*L. braziliensis*	93	1	93
LbrM19_V2.0810	*L. braziliensis*	84	1	84
LbrM19_V2.0820	*L. braziliensis*	106	27	132
LbrM19_V2.0820	*L. braziliensis*	161	139	299
LbrM19_V2.0830	*L. braziliensis*	132	1	132
LbrM19_V2.0830	*L. braziliensis*	118	155	272
LbrM19_V2.0830	*L. braziliensis*	170	341	510
LbrM19_V2.0830	*L. braziliensis*	149	553	701
LbrM19_V2.0830	*L. braziliensis*	90	925	1014
LbrM19_V2.0840	*L. braziliensis*	50	38	87
LbrM19_V2.0840	*L. braziliensis*	45	154	198
LbrM19_V2.0840	*L. braziliensis*	51	276	326
LbrM19_V2.0840	*L. braziliensis*	67	335	401
LbrM19_V2.0850	*L. braziliensis*	106	34	139
LbrM19_V2.0850	*L. braziliensis*	214	500	713
LbrM19_V2.0860	*L. braziliensis*	65	64	128
LbrM19_V2.0870	*L. braziliensis*	999	1	999
LbrM19_V2.0870	*L. braziliensis*	60	1040	1099
LbrM19_V2.0870	*L. braziliensis*	40	1444	1483
LbrM19_V2.0870	*L. braziliensis*	56	1833	1888
LbrM19_V2.0870	*L. braziliensis*	235	2495	2729
LbrM19_V2.0870	*L. braziliensis*	57	2731	2787
LbrM19_V2.0870	*L. braziliensis*	238	2817	3054
LbrM19_V2.0880	*L. braziliensis*	75	34	108
LbrM19_V2.0880	*L. braziliensis*	66	194	259
LbrM19_V2.0880	*L. braziliensis*	186	340	525
LbrM19_V2.0880	*L. braziliensis*	41	549	589
LbrM19_V2.0880	*L. braziliensis*	186	650	835
LbrM19_V2.0880	*L. braziliensis*	268	1037	1304
LbrM19_V2.0880	*L. braziliensis*	141	1318	1458
LbrM19_V2.0880	*L. braziliensis*	497	1466	1962
LbrM19_V2.0880	*L. braziliensis*	68	2015	2082
LbrM19_V2.0880	*L. braziliensis*	84	2118	2201
LbrM19_V2.0880	*L. braziliensis*	47	2340	2386
LbrM19_V2.0880	*L. braziliensis*	63	2545	2607
LbrM19_V2.0880	*L. braziliensis*	301	2695	2995
LbrM19_V2.0880	*L. braziliensis*	66	3056	3121
LbrM19_V2.0890	*L. braziliensis*	199	245	443
LbrM19_V2.0890	*L. braziliensis*	85	607	691
LbrM19_V2.0890	*L. braziliensis*	70	1088	1157

LbrM19_V2.0890	*L. braziliensis*	55	1196	1250
LbrM19_V2.0910	*L. braziliensis*	145	1	145
LbrM19_V2.0910	*L. braziliensis*	75	148	222
LbrM19_V2.0910	*L. braziliensis*	40	224	263
LbrM19_V2.0910	*L. braziliensis*	281	285	565
LbrM19_V2.0910	*L. braziliensis*	121	591	711
LbrM19_V2.0910	*L. braziliensis*	178	928	1105
LbrM19_V2.0910	*L. braziliensis*	70	1206	1275
LbrM19_V2.0910	*L. braziliensis*	301	1300	1600
LbrM19_V2.0920	*L. braziliensis*	520	1	520
LbrM19_V2.0920	*L. braziliensis*	110	544	653
LbrM19_V2.0920	*L. braziliensis*	69	687	755
LbrM19_V2.0940	*L. braziliensis*	53	63	115
LbrM19_V2.0940	*L. braziliensis*	61	244	304
LbrM19_V2.0940	*L. braziliensis*	46	612	657
LbrM19_V2.0950	*L. braziliensis*	78	1	78
LbrM19_V2.0970	*L. braziliensis*	48	1	48
LbrM19_V2.0970	*L. braziliensis*	46	826	871
LbrM19_V2.0970	*L. braziliensis*	43	1131	1173
LbrM19_V2.0970	*L. braziliensis*	49	1205	1253
LbrM19_V2.0970	*L. braziliensis*	58	1340	1397
LbrM19_V2.0970	*L. braziliensis*	244	1432	1675
LbrM19_V2.0970	*L. braziliensis*	130	1737	1866
LbrM19_V2.0980	*L. braziliensis*	113	1	113
LbrM19_V2.0990	*L. braziliensis*	152	1	152
LbrM19_V2.0990	*L. braziliensis*	53	186	238
LbrM19_V2.0990	*L. braziliensis*	98	263	360
LbrM19_V2.1000	*L. braziliensis*	86	1	86
LbrM19_V2.1000	*L. braziliensis*	56	160	215
LbrM19_V2.1000	*L. braziliensis*	46	268	313
LbrM19_V2.1000	*L. braziliensis*	69	626	694
LbrM19_V2.1000	*L. braziliensis*	231	740	970
LbrM19_V2.1000	*L. braziliensis*	91	1007	1097
LbrM19_V2.1010	*L. braziliensis*	76	1	76
LbrM19_V2.1010	*L. braziliensis*	41	171	211
LbrM19_V2.1010	*L. braziliensis*	72	222	293
LbrM19_V2.1010	*L. braziliensis*	53	333	385
LbrM19_V2.1010	*L. braziliensis*	344	565	908
LbrM19_V2.1010	*L. braziliensis*	1372	916	2287
LbrM19_V2.1030	*L. braziliensis*	173	85	257
LbrM19_V2.1040	*L. braziliensis*	75	311	385
LbrM19_V2.1050	*L. braziliensis*	126	126	251
LbrM19_V2.1050	*L. braziliensis*	47	368	414
LbrM19_V2.1060	*L. braziliensis*	102	1	102
LbrM19_V2.1060	*L. braziliensis*	56	116	171
LbrM19_V2.1060	*L. braziliensis*	43	201	243
LbrM19_V2.1060	*L. braziliensis*	49	539	587

LbrM19_V2.1070	*L. braziliensis*	117	166	282
LbrM19_V2.1080	*L. braziliensis*	105	1	105
LbrM19_V2.1080	*L. braziliensis*	75	115	189
LbrM19_V2.1100	*L. braziliensis*	80	372	451
LbrM19_V2.1100	*L. braziliensis*	114	632	745
LbrM19_V2.1110	*L. braziliensis*	63	1	63
LbrM19_V2.1110	*L. braziliensis*	54	311	364
LbrM19_V2.1120	*L. braziliensis*	42	52	93
LbrM19_V2.1120	*L. braziliensis*	153	729	881
LbrM19_V2.1120	*L. braziliensis*	153	729	881
LbrM19_V2.1160	*L. braziliensis*	79	1	79
LbrM19_V2.1160	*L. braziliensis*	57	162	218
LbrM19_V2.1190	*L. braziliensis*	62	59	120
LbrM19_V2.1190	*L. braziliensis*	88	248	335
LbrM19_V2.1190	*L. braziliensis*	70	379	448
LbrM19_V2.1190	*L. braziliensis*	232	503	734
LbrM19_V2.1200	*L. braziliensis*	457	106	562
LbrM19_V2.1210	*L. braziliensis*	56	118	173
LbrM19_V2.1210	*L. braziliensis*	186	191	376
LbrM19_V2.1210	*L. braziliensis*	57	479	535
LbrM19_V2.1210	*L. braziliensis*	81	551	631
LbrM19_V2.1210	*L. braziliensis*	45	642	686
LbrM19_V2.1240	*L. braziliensis*	46	62	107
LbrM19_V2.1240	*L. braziliensis*	52	215	266
LbrM19_V2.1240	*L. braziliensis*	119	278	396
LbrM19_V2.1240	*L. braziliensis*	84	678	761
LbrM19_V2.1240	*L. braziliensis*	69	805	873
LbrM19_V2.1240	*L. braziliensis*	79	920	998
LbrM19_V2.1240	*L. braziliensis*	74	1081	1154
LbrM19_V2.1240	*L. braziliensis*	50	1234	1283
LbrM19_V2.1240	*L. braziliensis*	55	1386	1440
LbrM19_V2.1240	*L. braziliensis*	41	1516	1556
LbrM19_V2.1240	*L. braziliensis*	96	1648	1743
LbrM19_V2.1240	*L. braziliensis*	64	1752	1815
LbrM19_V2.1240	*L. braziliensis*	47	1944	1990
LbrM19_V2.1240	*L. braziliensis*	46	2088	2133
LbrM19_V2.1240	*L. braziliensis*	89	2274	2362
LbrM19_V2.1240	*L. braziliensis*	43	2544	2586
LbrM19_V2.1240	*L. braziliensis*	57	2632	2688
LbrM19_V2.1240	*L. braziliensis*	118	2787	2904
LbrM19_V2.1240	*L. braziliensis*	72	2939	3010
LbrM19_V2.1240	*L. braziliensis*	47	3055	3101
LbrM19_V2.1240	*L. braziliensis*	50	3156	3205
LbrM19_V2.1240	*L. braziliensis*	94	3324	3417
LbrM19_V2.1240	*L. braziliensis*	44	3435	3478
LbrM19_V2.1240	*L. braziliensis*	60	3647	3706
LbrM19_V2.1240	*L. braziliensis*	43	3804	3846

LbrM19_V2.1240	*L. braziliensis*	112	3995	4106
LbrM19_V2.1240	*L. braziliensis*	140	4240	4379
LbrM19_V2.1240	*L. braziliensis*	130	4429	4558
LbrM19_V2.1240	*L. braziliensis*	61	4697	4757
LbrM19_V2.1240	*L. braziliensis*	44	4814	4857
LbrM19_V2.1240	*L. braziliensis*	121	4878	4998
LbrM19_V2.1240	*L. braziliensis*	149	5110	5258
LbrM19_V2.1240	*L. braziliensis*	74	5284	5357
LbrM19_V2.1240	*L. braziliensis*	86	5364	5449
LbrM19_V2.1240	*L. braziliensis*	73	5531	5603
LbrM19_V2.1240	*L. braziliensis*	42	5606	5647
LbrM19_V2.1250	*L. braziliensis*	137	41	177
LbrM19_V2.1250	*L. braziliensis*	107	200	306
LbrM19_V2.1250	*L. braziliensis*	66	351	416
LbrM19_V2.1250	*L. braziliensis*	49	449	497
LbrM19_V2.1260	*L. braziliensis*	165	1	165
LbrM19_V2.1260	*L. braziliensis*	390	232	621
LbrM19_V2.1260	*L. braziliensis*	65	677	741
LbrM19_V2.1260	*L. braziliensis*	80	883	962
LbrM19_V2.1280	*L. braziliensis*	46	503	548
LbrM19_V2.1290	*L. braziliensis*	72	66	137
LbrM19_V2.1290	*L. braziliensis*	56	232	287
LbrM19_V2.1290	*L. braziliensis*	72	335	406
LbrM19_V2.1290	*L. braziliensis*	111	410	520
LbrM19_V2.1290	*L. braziliensis*	148	575	722
LbrM19_V2.1300	*L. braziliensis*	76	1	76
LbrM19_V2.1300	*L. braziliensis*	51	322	372
LbrM19_V2.1300	*L. braziliensis*	51	322	372
LbrM19_V2.1310	*L. braziliensis*	76	1	76
LbrM19_V2.1310	*L. braziliensis*	40	85	124
LbrM19_V2.1310	*L. braziliensis*	138	148	285
LbrM19_V2.1330	*L. braziliensis*	47	1	47
LbrM19_V2.1330	*L. braziliensis*	468	101	568
LbrM19_V2.1330	*L. braziliensis*	189	693	881
LbrM19_V2.1330	*L. braziliensis*	83	888	970
LbrM19_V2.1330	*L. braziliensis*	210	1017	1226
LbrM19_V2.1340	*L. braziliensis*	98	27	124
LbrM19_V2.1340	*L. braziliensis*	351	231	581
LbrM19_V2.1340	*L. braziliensis*	65	638	702
LbrM19_V2.1340	*L. braziliensis*	108	816	923
LbrM19_V2.1340	*L. braziliensis*	158	959	1116
LbrM19_V2.1340	*L. braziliensis*	218	1166	1383
LbrM19_V2.1350	*L. braziliensis*	110	184	293
LbrM19_V2.1360	*L. braziliensis*	50	1	50
LbrM19_V2.1390	*L. braziliensis*	65	35	99
LbrM19_V2.1390	*L. braziliensis*	532	132	663
LbrM19_V2.1390	*L. braziliensis*	123	721	843

LbrM19_V2.1390	*L. braziliensis*	239	960	1198
LbrM19_V2.1390	*L. braziliensis*	128	1205	1332
LbrM19_V2.1390	*L. braziliensis*	325	1380	1704
LbrM19_V2.1400	*L. braziliensis*	60	1	60
LbrM19_V2.1400	*L. braziliensis*	502	69	570
LbrM19_V2.1400	*L. braziliensis*	122	586	707
LbrM19_V2.1400	*L. braziliensis*	100	721	820
LbrM19_V2.1400	*L. braziliensis*	81	854	934
LbrM19_V2.1410	*L. braziliensis*	127	53	179
LbrM19_V2.1420	*L. braziliensis*	154	1	154
LbrM19_V2.1420	*L. braziliensis*	156	168	323
LbrM19_V2.1420	*L. braziliensis*	448	337	784
LbrM19_V2.1420	*L. braziliensis*	71	806	876
LbrM19_V2.1420	*L. braziliensis*	85	912	996
LbrM19_V2.1420	*L. braziliensis*	151	1059	1209
LbrM19_V2.1420	*L. braziliensis*	101	1218	1318
LbrM19_V2.1450	*L. braziliensis*	53	102	154
LbrM19_V2.1450	*L. braziliensis*	156	202	357
LbrM19_V2.1460	*L. braziliensis*	40	39	78
LbrM19_V2.1460	*L. braziliensis*	78	169	246
LbrM19_V2.1460	*L. braziliensis*	44	356	399
LbrM19_V2.1460	*L. braziliensis*	47	636	682
LbrM19_V2.1460	*L. braziliensis*	45	758	802
LbrM19_V2.1460	*L. braziliensis*	90	855	944
LbrM19_V2.1460	*L. braziliensis*	205	1328	1532
LbrM19_V2.1460	*L. braziliensis*	94	1658	1751
LbrM19_V2.1470	*L. braziliensis*	46	153	198
LbrM19_V2.1470	*L. braziliensis*	40	368	407
LbrM19_V2.1470	*L. braziliensis*	40	368	407
LbrM19_V2.1480	*L. braziliensis*	54	51	104
LbrM19_V2.1490	*L. braziliensis*	141	110	250
LbrM19_V2.1510	*L. braziliensis*	55	117	171
LbrM19_V2.1530	*L. braziliensis*	66	1	66
LbrM19_V2.1530	*L. braziliensis*	42	176	217
LbrM19_V2.1530	*L. braziliensis*	42	176	217
LbrM19_V2.1540	*L. braziliensis*	214	31	244
LbrM19_V2.1540	*L. braziliensis*	140	282	421
LbrM19_V2.1540	*L. braziliensis*	74	457	530
LbrM19_V2.1540	*L. braziliensis*	181	564	744
LbrM19_V2.1540	*L. braziliensis*	50	793	842
LbrM19_V2.1540	*L. braziliensis*	81	855	935
LbrM19_V2.1560	*L. braziliensis*	129	73	201
LbrM19_V2.1560	*L. braziliensis*	54	317	370
LbrM19_V2.1560	*L. braziliensis*	54	317	370
LbrM19_V2.1600	*L. braziliensis*	89	1	89
LbrM19_V2.1600	*L. braziliensis*	54	205	258
LbrM19_V2.1600	*L. braziliensis*	54	205	258

LbrM19_V2.1610	*L. braziliensis*	53	323	375
LbrM19_V2.1640	*L. braziliensis*	288	104	391
LbrM19_V2.1640	*L. braziliensis*	91	618	708
LbrM19_V2.1650	*L. braziliensis*	42	1	42
LbrM19_V2.1650	*L. braziliensis*	45	173	217
LbrM19_V2.1650	*L. braziliensis*	52	467	518
LbrM19_V2.1650	*L. braziliensis*	62	554	615
LbrM19_V2.1680	*L. braziliensis*	73	155	227
LbrM19_V2.1680	*L. braziliensis*	58	704	761
LbrM19_V2.1680	*L. braziliensis*	50	885	934
LbrM19_V2.1690	*L. braziliensis*	96	338	433
LbrM19_V2.1700	*L. braziliensis*	78	169	246
LbrM19_V2.1720	*L. braziliensis*	48	213	260
LbrM19_V2.1740	*L. braziliensis*	91	250	340
LbrM19_V2.1750	*L. braziliensis*	44	195	238
LbrM19_V2.1780	*L. braziliensis*	178	1	178
LbrM19_V2.1780	*L. braziliensis*	51	187	237
LbrM19_V2.1780	*L. braziliensis*	82	271	352
LbrM19_V2.1780	*L. braziliensis*	43	465	507
LbrM19_V2.1780	*L. braziliensis*	46	665	710
LbrM19_V2.1780	*L. braziliensis*	46	773	818
LbrM19_V2.1810	*L. braziliensis*	47	89	135
LbrM19_V2.1830	*L. braziliensis*	60	1	60
LbrM19_V2.1840	*L. braziliensis*	40	197	236
LbrM19_V2.1850	*L. braziliensis*	40	74	113
LbrM19_V2.1850	*L. braziliensis*	68	477	544
LbrM19_V2.1850	*L. braziliensis*	49	550	598
LbrM19_V2.1880	*L. braziliensis*	44	300	343
LbrM19_V2.1910	*L. braziliensis*	635	86	720
LbrM19_V2.1910	*L. braziliensis*	69	778	846
LbrM19_V2.1910	*L. braziliensis*	1013	876	1888
LbrM19_V2.1910	*L. braziliensis*	426	1904	2329
LbrM19_V2.1910	*L. braziliensis*	277	2354	2630
LbrM19_V2.1920	*L. braziliensis*	60	1	60
LbrM19_V2.1920	*L. braziliensis*	64	83	146
LbrM19_V2.1930	*L. braziliensis*	56	1	56
LbrM19_V2.1930	*L. braziliensis*	67	100	166
LbrM19_V2.1930	*L. braziliensis*	67	100	166
LbrM20_V2.0060	*L. braziliensis*	40	124	163
LbrM20_V2.0070	*L. braziliensis*	41	1	41
LbrM20_V2.0070	*L. braziliensis*	52	327	378
LbrM20_V2.0070	*L. braziliensis*	52	327	378
LbrM20_V2.0080	*L. braziliensis*	42	241	282
LbrM20_V2.0110	*L. braziliensis*	46	295	340
LbrM20_V2.0140	*L. braziliensis*	50	238	287
LbrM20_V2.0140	*L. braziliensis*	60	515	574
LbrM20_V2.0140	*L. braziliensis*	43	653	695

LbrM20_V2.0140	*L. braziliensis*	41	742	782
LbrM20_V2.0140	*L. braziliensis*	146	905	1050
LbrM20_V2.0170	*L. braziliensis*	179	1	179
LbrM20_V2.0180	*L. braziliensis*	146	1	146
LbrM20_V2.0180	*L. braziliensis*	118	169	286
LbrM20_V2.0180	*L. braziliensis*	268	354	621
LbrM20_V2.0190	*L. braziliensis*	48	1	48
LbrM20_V2.0190	*L. braziliensis*	79	339	417
LbrM20_V2.0190	*L. braziliensis*	46	483	528
LbrM20_V2.0190	*L. braziliensis*	65	725	789
LbrM20_V2.0210	*L. braziliensis*	47	359	405
LbrM20_V2.0220	*L. braziliensis*	89	119	207
LbrM20_V2.0230	*L. braziliensis*	58	228	285
LbrM20_V2.0240	*L. braziliensis*	222	223	444
LbrM20_V2.0240	*L. braziliensis*	42	547	588
LbrM20_V2.0240	*L. braziliensis*	54	605	658
LbrM20_V2.0250	*L. braziliensis*	734	90	823
LbrM20_V2.0250	*L. braziliensis*	331	992	1322
LbrM20_V2.0260	*L. braziliensis*	295	1	295
LbrM20_V2.0260	*L. braziliensis*	82	302	383
LbrM20_V2.0280	*L. braziliensis*	70	301	370
LbrM20_V2.0280	*L. braziliensis*	40	547	586
LbrM20_V2.0280	*L. braziliensis*	40	547	586
LbrM20_V2.0290	*L. braziliensis*	159	1	159
LbrM20_V2.0290	*L. braziliensis*	55	231	285
LbrM20_V2.0290	*L. braziliensis*	55	324	378
LbrM20_V2.0290	*L. braziliensis*	46	1030	1075
LbrM20_V2.0290	*L. braziliensis*	47	1487	1533
LbrM20_V2.0300	*L. braziliensis*	40	333	372
LbrM20_V2.0310	*L. braziliensis*	95	225	319
LbrM20_V2.0310	*L. braziliensis*	67	385	451
LbrM20_V2.0310	*L. braziliensis*	67	582	648
LbrM20_V2.0320	*L. braziliensis*	71	137	207
LbrM20_V2.0330	*L. braziliensis*	59	731	789
LbrM20_V2.0330	*L. braziliensis*	92	1011	1102
LbrM20_V2.0330	*L. braziliensis*	82	1488	1569
LbrM20_V2.0340	*L. braziliensis*	116	1	116
LbrM20_V2.0340	*L. braziliensis*	49	145	193
LbrM20_V2.0340	*L. braziliensis*	40	420	459
LbrM20_V2.0340	*L. braziliensis*	68	565	632
LbrM20_V2.0350	*L. braziliensis*	78	169	246
LbrM20_V2.0360	*L. braziliensis*	52	311	362
LbrM20_V2.0380	*L. braziliensis*	116	271	386
LbrM20_V2.0390	*L. braziliensis*	127	1	127
LbrM20_V2.0390	*L. braziliensis*	79	317	395
LbrM20_V2.0390	*L. braziliensis*	41	640	680
LbrM20_V2.0400	*L. braziliensis*	48	55	102

LbrM20_V2.0410	*L. braziliensis*	40	1	40
LbrM20_V2.0420	*L. braziliensis*	191	1	191
LbrM20_V2.0420	*L. braziliensis*	371	196	566
LbrM20_V2.0420	*L. braziliensis*	380	568	947
LbrM20_V2.0430	*L. braziliensis*	85	1803	1887
LbrM20_V2.0430	*L. braziliensis*	76	1907	1982
LbrM20_V2.0430	*L. braziliensis*	103	2029	2131
LbrM20_V2.0440	*L. braziliensis*	90	496	585
LbrM20_V2.0450	*L. braziliensis*	136	418	553
LbrM20_V2.0450	*L. braziliensis*	118	572	689
LbrM20_V2.0450	*L. braziliensis*	118	572	689
LbrM20_V2.0460	*L. braziliensis*	227	1	227
LbrM20_V2.0460	*L. braziliensis*	122	687	808
LbrM20_V2.0460	*L. braziliensis*	117	843	959
LbrM20_V2.0460	*L. braziliensis*	49	1024	1072
LbrM20_V2.0460	*L. braziliensis*	106	1455	1560
LbrM20_V2.0460	*L. braziliensis*	169	2596	2764
LbrM20_V2.0480	*L. braziliensis*	55	221	275
LbrM20_V2.0490	*L. braziliensis*	41	34	74
LbrM20_V2.0490	*L. braziliensis*	81	77	157
LbrM20_V2.0490	*L. braziliensis*	43	259	301
LbrM20_V2.0490	*L. braziliensis*	60	780	839
LbrM20_V2.0490	*L. braziliensis*	116	1031	1146
LbrM20_V2.0490	*L. braziliensis*	67	1679	1745
LbrM20_V2.0490	*L. braziliensis*	43	1747	1789
LbrM20_V2.0500	*L. braziliensis*	46	786	831
LbrM20_V2.0510	*L. braziliensis*	88	35	122
LbrM20_V2.0520	*L. braziliensis*	63	174	236
LbrM20_V2.0520	*L. braziliensis*	40	424	463
LbrM20_V2.0520	*L. braziliensis*	48	646	693
LbrM20_V2.0520	*L. braziliensis*	47	834	880
LbrM20_V2.0520	*L. braziliensis*	45	906	950
LbrM20_V2.0520	*L. braziliensis*	41	1049	1089
LbrM20_V2.0530	*L. braziliensis*	113	1	113
LbrM20_V2.0530	*L. braziliensis*	70	180	249
LbrM20_V2.0530	*L. braziliensis*	247	287	533
LbrM20_V2.0530	*L. braziliensis*	324	535	858
LbrM20_V2.0550	*L. braziliensis*	827	1	827
LbrM20_V2.0550	*L. braziliensis*	256	969	1224
LbrM20_V2.0550	*L. braziliensis*	315	1274	1588
LbrM20_V2.0550	*L. braziliensis*	775	1603	2377
LbrM20_V2.0560	*L. braziliensis*	173	48	220
LbrM20_V2.0560	*L. braziliensis*	70	253	322
LbrM20_V2.0560	*L. braziliensis*	105	444	548
LbrM20_V2.0560	*L. braziliensis*	59	563	621
LbrM20_V2.0560	*L. braziliensis*	62	656	717
LbrM20_V2.0570	*L. braziliensis*	46	141	186

LbrM20_V2.0570	*L. braziliensis*	60	212	271
LbrM20_V2.0590	*L. braziliensis*	45	1	45
LbrM20_V2.0590	*L. braziliensis*	65	55	119
LbrM20_V2.0590	*L. braziliensis*	77	223	299
LbrM20_V2.0590	*L. braziliensis*	86	307	392
LbrM20_V2.0590	*L. braziliensis*	45	529	573
LbrM20_V2.0590	*L. braziliensis*	357	661	1017
LbrM20_V2.0600	*L. braziliensis*	60	349	408
LbrM20_V2.0600	*L. braziliensis*	68	641	708
LbrM20_V2.0600	*L. braziliensis*	59	778	836
LbrM20_V2.0600	*L. braziliensis*	47	975	1021
LbrM20_V2.0600	*L. braziliensis*	86	1253	1338
LbrM20_V2.0600	*L. braziliensis*	40	1718	1757
LbrM20_V2.0600	*L. braziliensis*	59	1907	1965
LbrM20_V2.0610	*L. braziliensis*	51	70	120
LbrM20_V2.0610	*L. braziliensis*	79	377	455
LbrM20_V2.0610	*L. braziliensis*	106	470	575
LbrM20_V2.0610	*L. braziliensis*	117	663	779
LbrM20_V2.0630	*L. braziliensis*	49	91	139
LbrM20_V2.0630	*L. braziliensis*	60	150	209
LbrM20_V2.0640	*L. braziliensis*	49	761	809
LbrM20_V2.0650	*L. braziliensis*	40	213	252
LbrM20_V2.0650	*L. braziliensis*	134	285	418
LbrM20_V2.0670	*L. braziliensis*	60	1	60
LbrM20_V2.0670	*L. braziliensis*	47	341	387
LbrM20_V2.0670	*L. braziliensis*	47	341	387
LbrM20_V2.0690	*L. braziliensis*	63	138	200
LbrM20_V2.0700	*L. braziliensis*	98	69	166
LbrM20_V2.0750	*L. braziliensis*	98	25	122
LbrM20_V2.0770	*L. braziliensis*	101	1	101
LbrM20_V2.0770	*L. braziliensis*	84	142	225
LbrM20_V2.0810	*L. braziliensis*	97	60	156
LbrM20_V2.0840	*L. braziliensis*	66	1	66
LbrM20_V2.0840	*L. braziliensis*	82	164	245
LbrM20_V2.0840	*L. braziliensis*	94	284	377
LbrM20_V2.0840	*L. braziliensis*	144	403	546
LbrM20_V2.0850	*L. braziliensis*	53	1	53
LbrM20_V2.0850	*L. braziliensis*	79	441	519
LbrM20_V2.0850	*L. braziliensis*	58	800	857
LbrM20_V2.0860	*L. braziliensis*	67	1	67
LbrM20_V2.0870	*L. braziliensis*	101	164	264
LbrM20_V2.0890	*L. braziliensis*	99	166	264
LbrM20_V2.0900	*L. braziliensis*	99	166	264
LbrM20_V2.0920	*L. braziliensis*	264	1	264
LbrM20_V2.0920	*L. braziliensis*	72	445	516
LbrM20_V2.0940	*L. braziliensis*	436	1	436
LbrM20_V2.0940	*L. braziliensis*	67	695	761

LbrM20_V2.0940	*L. braziliensis*	60	836	895
LbrM20_V2.0940	*L. braziliensis*	61	925	985
LbrM20_V2.0970	*L. braziliensis*	97	232	328
LbrM20_V2.0970	*L. braziliensis*	153	1213	1365
LbrM20_V2.0980	*L. braziliensis*	117	130	246
LbrM20_V2.0980	*L. braziliensis*	53	277	329
LbrM20_V2.0980	*L. braziliensis*	104	369	472
LbrM20_V2.0990	*L. braziliensis*	56	1	56
LbrM20_V2.1000	*L. braziliensis*	64	25	88
LbrM20_V2.1020	*L. braziliensis*	59	175	233
LbrM20_V2.1030	*L. braziliensis*	127	22	148
LbrM20_V2.1030	*L. braziliensis*	71	323	393
LbrM20_V2.1040	*L. braziliensis*	87	274	360
LbrM20_V2.1050	*L. braziliensis*	196	1	196
LbrM20_V2.1100	*L. braziliensis*	117	78	194
LbrM20_V2.1100	*L. braziliensis*	70	706	775
LbrM20_V2.1100	*L. braziliensis*	70	706	775
LbrM20_V2.1110	*L. braziliensis*	40	1	40
LbrM20_V2.1120	*L. braziliensis*	44	60	103
LbrM20_V2.1120	*L. braziliensis*	55	463	517
LbrM20_V2.1120	*L. braziliensis*	52	586	637
LbrM20_V2.1120	*L. braziliensis*	80	923	1002
LbrM20_V2.1130	*L. braziliensis*	145	26	170
LbrM20_V2.1140	*L. braziliensis*	259	272	530
LbrM20_V2.1150	*L. braziliensis*	88	125	212
LbrM20_V2.1160	*L. braziliensis*	1150	1	1150
LbrM20_V2.1160	*L. braziliensis*	150	1293	1442
LbrM20_V2.1160	*L. braziliensis*	489	1560	2048
LbrM20_V2.1160	*L. braziliensis*	222	2063	2284
LbrM20_V2.1160	*L. braziliensis*	81	2287	2367
LbrM20_V2.1170	*L. braziliensis*	41	58	98
LbrM20_V2.1180	*L. braziliensis*	58	1	58
LbrM20_V2.1180	*L. braziliensis*	133	108	240
LbrM20_V2.1180	*L. braziliensis*	54	280	333
LbrM20_V2.1190	*L. braziliensis*	81	88	168
LbrM20_V2.1200	*L. braziliensis*	102	349	450
LbrM20_V2.1210	*L. braziliensis*	471	28	498
LbrM20_V2.1220	*L. braziliensis*	71	177	247
LbrM20_V2.1230	*L. braziliensis*	79	52	130
LbrM20_V2.1230	*L. braziliensis*	48	160	207
LbrM20_V2.1230	*L. braziliensis*	97	250	346
LbrM20_V2.1240	*L. braziliensis*	264	172	435
LbrM20_V2.1240	*L. braziliensis*	46	482	527
LbrM20_V2.1240	*L. braziliensis*	53	643	695
LbrM20_V2.1240	*L. braziliensis*	71	808	878
LbrM20_V2.1240	*L. braziliensis*	79	1266	1344
LbrM20_V2.1240	*L. braziliensis*	54	1547	1600

LbrM20_V2.1240	*L. braziliensis*	66	1663	1728
LbrM20_V2.1240	*L. braziliensis*	60	1885	1944
LbrM20_V2.1260	*L. braziliensis*	47	119	165
LbrM20_V2.1260	*L. braziliensis*	70	486	555
LbrM20_V2.1260	*L. braziliensis*	54	773	826
LbrM20_V2.1260	*L. braziliensis*	62	1153	1214
LbrM20_V2.1260	*L. braziliensis*	88	1291	1378
LbrM20_V2.1260	*L. braziliensis*	127	1411	1537
LbrM20_V2.1290	*L. braziliensis*	409	1	409
LbrM20_V2.1290	*L. braziliensis*	40	652	691
LbrM20_V2.1290	*L. braziliensis*	99	861	959
LbrM20_V2.1300	*L. braziliensis*	42	53	94
LbrM20_V2.1300	*L. braziliensis*	65	97	161
LbrM20_V2.1300	*L. braziliensis*	80	516	595
LbrM20_V2.1300	*L. braziliensis*	67	708	774
LbrM20_V2.1300	*L. braziliensis*	105	787	891
LbrM20_V2.1300	*L. braziliensis*	58	924	981
LbrM20_V2.1310	*L. braziliensis*	65	216	280
LbrM20_V2.1320	*L. braziliensis*	202	515	716
LbrM20_V2.1330	*L. braziliensis*	72	47	118
LbrM20_V2.1330	*L. braziliensis*	169	199	367
LbrM20_V2.1340	*L. braziliensis*	99	1	99
LbrM20_V2.1340	*L. braziliensis*	118	271	388
LbrM20_V2.1340	*L. braziliensis*	67	692	758
LbrM20_V2.1340	*L. braziliensis*	114	817	930
LbrM20_V2.1350	*L. braziliensis*	243	62	304
LbrM20_V2.1350	*L. braziliensis*	104	417	520
LbrM20_V2.1350	*L. braziliensis*	70	560	629
LbrM20_V2.1350	*L. braziliensis*	67	697	763
LbrM20_V2.1360	*L. braziliensis*	49	45	93
LbrM20_V2.1370	*L. braziliensis*	45	73	117
LbrM20_V2.1370	*L. braziliensis*	79	202	280
LbrM20_V2.1370	*L. braziliensis*	234	340	573
LbrM20_V2.1370	*L. braziliensis*	129	983	1111
LbrM20_V2.1370	*L. braziliensis*	148	1150	1297
LbrM20_V2.1380	*L. braziliensis*	52	1	52
LbrM20_V2.1380	*L. braziliensis*	60	90	149
LbrM20_V2.1380	*L. braziliensis*	143	215	357
LbrM20_V2.1390	*L. braziliensis*	103	1	103
LbrM20_V2.1390	*L. braziliensis*	48	165	212
LbrM20_V2.1390	*L. braziliensis*	47	412	458
LbrM20_V2.1390	*L. braziliensis*	44	935	978
LbrM20_V2.1390	*L. braziliensis*	151	1686	1836
LbrM20_V2.1390	*L. braziliensis*	49	1903	1951
LbrM20_V2.1390	*L. braziliensis*	77	2189	2265
LbrM20_V2.1390	*L. braziliensis*	174	2438	2611
LbrM20_V2.1400	*L. braziliensis*	67	124	190

LbrM20_V2.1410	*L. braziliensis*	129	1	129
LbrM20_V2.1410	*L. braziliensis*	98	157	254
LbrM20_V2.1410	*L. braziliensis*	106	422	527
LbrM20_V2.1420	*L. braziliensis*	57	100	156
LbrM20_V2.1420	*L. braziliensis*	45	202	246
LbrM20_V2.1420	*L. braziliensis*	80	474	553
LbrM20_V2.1420	*L. braziliensis*	67	704	770
LbrM20_V2.1420	*L. braziliensis*	48	1684	1731
LbrM20_V2.1420	*L. braziliensis*	58	1990	2047
LbrM20_V2.1420	*L. braziliensis*	69	2288	2356
LbrM20_V2.1420	*L. braziliensis*	50	2427	2476
LbrM20_V2.1420	*L. braziliensis*	51	2597	2647
LbrM20_V2.1420	*L. braziliensis*	48	3344	3391
LbrM20_V2.1420	*L. braziliensis*	46	4246	4291
LbrM20_V2.1420	*L. braziliensis*	83	4521	4603
LbrM20_V2.1420	*L. braziliensis*	51	4679	4729
LbrM20_V2.1440	*L. braziliensis*	52	1	52
LbrM20_V2.1440	*L. braziliensis*	44	470	513
LbrM20_V2.1450	*L. braziliensis*	120	76	195
LbrM20_V2.1450	*L. braziliensis*	76	498	573
LbrM20_V2.1450	*L. braziliensis*	70	703	772
LbrM20_V2.1460	*L. braziliensis*	40	1	40
LbrM20_V2.1470	*L. braziliensis*	140	1	140
LbrM20_V2.1490	*L. braziliensis*	122	195	316
LbrM20_V2.1490	*L. braziliensis*	207	437	643
LbrM20_V2.1490	*L. braziliensis*	46	656	701
LbrM20_V2.1510	*L. braziliensis*	93	16	108
LbrM20_V2.1520	*L. braziliensis*	47	54	100
LbrM20_V2.1520	*L. braziliensis*	147	479	625
LbrM20_V2.1530	*L. braziliensis*	65	185	249
LbrM20_V2.1530	*L. braziliensis*	50	283	332
LbrM20_V2.1530	*L. braziliensis*	210	364	573
LbrM20_V2.1530	*L. braziliensis*	61	855	915
LbrM20_V2.1530	*L. braziliensis*	47	1074	1120
LbrM20_V2.1540	*L. braziliensis*	165	443	607
LbrM20_V2.1540	*L. braziliensis*	96	708	803
LbrM20_V2.1550	*L. braziliensis*	90	88	177
LbrM20_V2.1550	*L. braziliensis*	52	179	230
LbrM20_V2.1550	*L. braziliensis*	52	179	230
LbrM20_V2.1560	*L. braziliensis*	64	1	64
LbrM20_V2.1570	*L. braziliensis*	89	1	89
LbrM20_V2.1570	*L. braziliensis*	114	217	330
LbrM20_V2.1570	*L. braziliensis*	95	398	492
LbrM20_V2.1570	*L. braziliensis*	49	729	777
LbrM20_V2.1570	*L. braziliensis*	50	812	861
LbrM20_V2.1570	*L. braziliensis*	50	885	934
LbrM20_V2.1570	*L. braziliensis*	135	1048	1182

LbrM20_V2.1570	*L. braziliensis*	44	1248	1291
LbrM20_V2.1570	*L. braziliensis*	46	1617	1662
LbrM20_V2.1570	*L. braziliensis*	59	1686	1744
LbrM20_V2.1570	*L. braziliensis*	54	1759	1812
LbrM20_V2.1570	*L. braziliensis*	117	1819	1935
LbrM20_V2.1590	*L. braziliensis*	51	95	145
LbrM20_V2.1590	*L. braziliensis*	79	334	412
LbrM20_V2.1590	*L. braziliensis*	233	479	711
LbrM20_V2.1600	*L. braziliensis*	51	24	74
LbrM20_V2.1600	*L. braziliensis*	69	299	367
LbrM20_V2.1600	*L. braziliensis*	41	373	413
LbrM20_V2.1600	*L. braziliensis*	86	415	500
LbrM20_V2.1610	*L. braziliensis*	48	259	306
LbrM20_V2.1650	*L. braziliensis*	42	220	261
LbrM20_V2.1650	*L. braziliensis*	94	296	389
LbrM20_V2.1650	*L. braziliensis*	102	665	766
LbrM20_V2.1650	*L. braziliensis*	124	774	897
LbrM20_V2.1650	*L. braziliensis*	45	1104	1148
LbrM20_V2.1650	*L. braziliensis*	99	1205	1303
LbrM20_V2.1650	*L. braziliensis*	57	1355	1411
LbrM20_V2.1660	*L. braziliensis*	121	58	178
LbrM20_V2.1670	*L. braziliensis*	44	277	320
LbrM20_V2.1670	*L. braziliensis*	42	494	535
LbrM20_V2.1670	*L. braziliensis*	42	494	535
LbrM20_V2.1680	*L. braziliensis*	63	778	840
LbrM20_V2.1690	*L. braziliensis*	58	343	400
LbrM20_V2.1690	*L. braziliensis*	57	694	750
LbrM20_V2.1690	*L. braziliensis*	48	941	988
LbrM20_V2.1700	*L. braziliensis*	54	139	192
LbrM20_V2.1710	*L. braziliensis*	72	1	72
LbrM20_V2.1710	*L. braziliensis*	114	80	193
LbrM20_V2.1710	*L. braziliensis*	58	224	281
LbrM20_V2.1710	*L. braziliensis*	213	323	535
LbrM20_V2.1710	*L. braziliensis*	55	561	615
LbrM20_V2.1710	*L. braziliensis*	204	659	862
LbrM20_V2.1710	*L. braziliensis*	80	888	967
LbrM20_V2.1710	*L. braziliensis*	91	984	1074
LbrM20_V2.1720	*L. braziliensis*	125	1	125
LbrM20_V2.1720	*L. braziliensis*	123	149	271
LbrM20_V2.1720	*L. braziliensis*	50	702	751
LbrM20_V2.1740	*L. braziliensis*	47	175	221
LbrM20_V2.1740	*L. braziliensis*	59	289	347
LbrM20_V2.1740	*L. braziliensis*	59	289	347
LbrM20_V2.1750	*L. braziliensis*	190	1	190
LbrM20_V2.1750	*L. braziliensis*	64	608	671
LbrM20_V2.1750	*L. braziliensis*	65	712	776
LbrM20_V2.1750	*L. braziliensis*	68	779	846

LbrM20_V2.1750	*L. braziliensis*	95	922	1016
LbrM20_V2.1750	*L. braziliensis*	127	1053	1179
LbrM20_V2.1750	*L. braziliensis*	198	1182	1379
LbrM20_V2.1750	*L. braziliensis*	147	1437	1583
LbrM20_V2.1750	*L. braziliensis*	54	1693	1746
LbrM20_V2.1750	*L. braziliensis*	108	1825	1932
LbrM20_V2.1760	*L. braziliensis*	55	1	55
LbrM20_V2.1770	*L. braziliensis*	155	390	544
LbrM20_V2.1780	*L. braziliensis*	184	555	738
LbrM20_V2.1790	*L. braziliensis*	64	268	331
LbrM20_V2.1840	*L. braziliensis*	93	75	167
LbrM20_V2.1850	*L. braziliensis*	78	1	78
LbrM20_V2.1860	*L. braziliensis*	428	1	428
LbrM20_V2.1870	*L. braziliensis*	171	13	183
LbrM20_V2.1870	*L. braziliensis*	100	266	365
LbrM20_V2.1880	*L. braziliensis*	160	1	160
LbrM20_V2.1880	*L. braziliensis*	189	175	363
LbrM20_V2.1890	*L. braziliensis*	50	1	50
LbrM20_V2.1890	*L. braziliensis*	85	82	166
LbrM20_V2.1890	*L. braziliensis*	85	82	166
LbrM20_V2.1930	*L. braziliensis*	173	1	173
LbrM20_V2.1930	*L. braziliensis*	127	225	351
LbrM20_V2.1930	*L. braziliensis*	51	413	463
LbrM20_V2.1930	*L. braziliensis*	51	754	804
LbrM20_V2.1940	*L. braziliensis*	83	106	188
LbrM20_V2.1950	*L. braziliensis*	53	429	481
LbrM20_V2.1950	*L. braziliensis*	101	544	644
LbrM20_V2.1950	*L. braziliensis*	91	1133	1223
LbrM20_V2.1950	*L. braziliensis*	55	1660	1714
LbrM20_V2.1960	*L. braziliensis*	134	28	161
LbrM20_V2.1960	*L. braziliensis*	92	355	446
LbrM20_V2.1980	*L. braziliensis*	48	91	138
LbrM20_V2.1990	*L. braziliensis*	89	148	236
LbrM20_V2.2000	*L. braziliensis*	107	221	327
LbrM20_V2.2010	*L. braziliensis*	187	132	318
LbrM20_V2.2010	*L. braziliensis*	215	322	536
LbrM20_V2.2010	*L. braziliensis*	113	550	662
LbrM20_V2.2010	*L. braziliensis*	58	740	797
LbrM20_V2.2010	*L. braziliensis*	261	854	1114
LbrM20_V2.2020	*L. braziliensis*	91	1	91
LbrM20_V2.2020	*L. braziliensis*	42	188	229
LbrM20_V2.2020	*L. braziliensis*	42	188	229
LbrM20_V2.2030	*L. braziliensis*	84	209	292
LbrM20_V2.2040	*L. braziliensis*	69	1	69
LbrM20_V2.2040	*L. braziliensis*	60	75	134
LbrM20_V2.2040	*L. braziliensis*	60	75	134
LbrM20_V2.2050	*L. braziliensis*	148	1	148

LbrM20_V2.2060	*L. braziliensis*	138	1	138
LbrM20_V2.2060	*L. braziliensis*	46	208	253
LbrM20_V2.2060	*L. braziliensis*	110	377	486
LbrM20_V2.2060	*L. braziliensis*	58	511	568
LbrM20_V2.2060	*L. braziliensis*	42	775	816
LbrM20_V2.2060	*L. braziliensis*	91	898	988
LbrM20_V2.2060	*L. braziliensis*	40	1458	1497
LbrM20_V2.2070	*L. braziliensis*	307	56	362
LbrM20_V2.2070	*L. braziliensis*	80	404	483
LbrM20_V2.2070	*L. braziliensis*	117	524	640
LbrM20_V2.2070	*L. braziliensis*	391	668	1058
LbrM20_V2.2080	*L. braziliensis*	44	1	44
LbrM20_V2.2080	*L. braziliensis*	682	66	747
LbrM20_V2.2080	*L. braziliensis*	91	749	839
LbrM20_V2.2080	*L. braziliensis*	399	937	1335
LbrM20_V2.2080	*L. braziliensis*	83	1337	1419
LbrM20_V2.2090	*L. braziliensis*	370	35	404
LbrM20_V2.2090	*L. braziliensis*	91	406	496
LbrM20_V2.2090	*L. braziliensis*	458	594	1051
LbrM20_V2.2090	*L. braziliensis*	94	1078	1171
LbrM20_V2.2090	*L. braziliensis*	98	1173	1270
LbrM20_V2.2100	*L. braziliensis*	70	53	122
LbrM20_V2.2100	*L. braziliensis*	41	165	205
LbrM20_V2.2100	*L. braziliensis*	42	336	377
LbrM20_V2.2100	*L. braziliensis*	89	410	498
LbrM20_V2.2100	*L. braziliensis*	49	527	575
LbrM20_V2.2100	*L. braziliensis*	270	597	866
LbrM20_V2.2100	*L. braziliensis*	199	964	1162
LbrM20_V2.2100	*L. braziliensis*	357	1169	1525
LbrM20_V2.2110	*L. braziliensis*	282	37	318
LbrM20_V2.2110	*L. braziliensis*	47	374	420
LbrM20_V2.2110	*L. braziliensis*	182	511	692
LbrM20_V2.2110	*L. braziliensis*	53	804	856
LbrM20_V2.2120	*L. braziliensis*	61	157	217
LbrM20_V2.2120	*L. braziliensis*	55	225	279
LbrM20_V2.2120	*L. braziliensis*	352	322	673
LbrM20_V2.2120	*L. braziliensis*	47	708	754
LbrM20_V2.2130	*L. braziliensis*	271	133	403
LbrM20_V2.2130	*L. braziliensis*	48	512	559
LbrM20_V2.2140	*L. braziliensis*	80	119	198
LbrM20_V2.2150	*L. braziliensis*	45	373	417
LbrM20_V2.2170	*L. braziliensis*	51	1	51
LbrM20_V2.2170	*L. braziliensis*	52	129	180
LbrM20_V2.2170	*L. braziliensis*	52	129	180
LbrM20_V2.2180	*L. braziliensis*	40	360	399
LbrM20_V2.2190	*L. braziliensis*	51	189	239
LbrM20_V2.2190	*L. braziliensis*	42	284	325

LbrM20_V2.2190	*L. braziliensis*	94	329	422
LbrM20_V2.2190	*L. braziliensis*	41	1470	1510
LbrM20_V2.2220	*L. braziliensis*	42	25	66
LbrM20_V2.2220	*L. braziliensis*	68	289	356
LbrM20_V2.2220	*L. braziliensis*	56	648	703
LbrM20_V2.2220	*L. braziliensis*	48	755	802
LbrM20_V2.2240	*L. braziliensis*	105	275	379
LbrM20_V2.2240	*L. braziliensis*	54	445	498
LbrM20_V2.2250	*L. braziliensis*	209	24	232
LbrM20_V2.2250	*L. braziliensis*	130	308	437
LbrM20_V2.2250	*L. braziliensis*	97	459	555
LbrM20_V2.2260	*L. braziliensis*	48	1	48
LbrM20_V2.2260	*L. braziliensis*	84	107	190
LbrM20_V2.2270	*L. braziliensis*	262	1	262
LbrM20_V2.2280	*L. braziliensis*	56	370	425
LbrM20_V2.2290	*L. braziliensis*	102	181	282
LbrM20_V2.2300	*L. braziliensis*	50	137	186
LbrM20_V2.2300	*L. braziliensis*	124	302	425
LbrM20_V2.2310	*L. braziliensis*	47	65	111
LbrM20_V2.2310	*L. braziliensis*	43	259	301
LbrM20_V2.2320	*L. braziliensis*	119	35	153
LbrM20_V2.2320	*L. braziliensis*	121	195	315
LbrM20_V2.2330	*L. braziliensis*	64	112	175
LbrM20_V2.2330	*L. braziliensis*	55	269	323
LbrM20_V2.2330	*L. braziliensis*	46	372	417
LbrM20_V2.2330	*L. braziliensis*	40	566	605
LbrM20_V2.2350	*L. braziliensis*	46	356	401
LbrM20_V2.2350	*L. braziliensis*	43	560	602
LbrM20_V2.2350	*L. braziliensis*	43	560	602
LbrM20_V2.2380	*L. braziliensis*	42	316	357
LbrM20_V2.2430	*L. braziliensis*	78	1	78
LbrM20_V2.2430	*L. braziliensis*	76	201	276
LbrM20_V2.2430	*L. braziliensis*	62	328	389
LbrM20_V2.2430	*L. braziliensis*	81	806	886
LbrM20_V2.2440	*L. braziliensis*	92	50	141
LbrM20_V2.2440	*L. braziliensis*	190	162	351
LbrM20_V2.2440	*L. braziliensis*	63	582	644
LbrM20_V2.2460	*L. braziliensis*	46	603	648
LbrM20_V2.2480	*L. braziliensis*	42	1	42
LbrM20_V2.2480	*L. braziliensis*	61	166	226
LbrM20_V2.2480	*L. braziliensis*	61	166	226
LbrM20_V2.2510	*L. braziliensis*	52	58	109
LbrM20_V2.2540	*L. braziliensis*	51	164	214
LbrM20_V2.2550	*L. braziliensis*	101	91	191
LbrM20_V2.2570	*L. braziliensis*	92	110	201
LbrM20_V2.2580	*L. braziliensis*	69	1	69
LbrM20_V2.2580	*L. braziliensis*	218	133	350

LbrM20_V2.2580	*L. braziliensis*	95	389	483
LbrM20_V2.2580	*L. braziliensis*	59	516	574
LbrM20_V2.2590	*L. braziliensis*	42	208	249
LbrM20_V2.2600	*L. braziliensis*	125	13	137
LbrM20_V2.2600	*L. braziliensis*	132	145	276
LbrM20_V2.2600	*L. braziliensis*	70	615	684
LbrM20_V2.2600	*L. braziliensis*	45	698	742
LbrM20_V2.2600	*L. braziliensis*	156	752	907
LbrM20_V2.2610	*L. braziliensis*	79	1	79
LbrM20_V2.2610	*L. braziliensis*	60	86	145
LbrM20_V2.2620	*L. braziliensis*	81	194	274
LbrM20_V2.2620	*L. braziliensis*	43	383	425
LbrM20_V2.2640	*L. braziliensis*	67	58	124
LbrM20_V2.2650	*L. braziliensis*	87	22	108
LbrM20_V2.2650	*L. braziliensis*	113	182	294
LbrM20_V2.2660	*L. braziliensis*	118	618	735
LbrM20_V2.2670	*L. braziliensis*	44	15	58
LbrM20_V2.2670	*L. braziliensis*	142	78	219
LbrM20_V2.2690	*L. braziliensis*	45	47	91
LbrM20_V2.2720	*L. braziliensis*	50	1	50
LbrM20_V2.2720	*L. braziliensis*	71	182	252
LbrM20_V2.2730	*L. braziliensis*	84	1	84
LbrM20_V2.2740	*L. braziliensis*	70	1	70
LbrM20_V2.2740	*L. braziliensis*	150	118	267
LbrM20_V2.2740	*L. braziliensis*	43	545	587
LbrM20_V2.2750	*L. braziliensis*	134	83	216
LbrM20_V2.2750	*L. braziliensis*	165	270	434
LbrM20_V2.2760	*L. braziliensis*	181	40	220
LbrM20_V2.2760	*L. braziliensis*	145	253	397
LbrM20_V2.2770	*L. braziliensis*	77	33	109
LbrM20_V2.2770	*L. braziliensis*	51	438	488
LbrM20_V2.2770	*L. braziliensis*	51	438	488
LbrM20_V2.2780	*L. braziliensis*	52	169	220
LbrM20_V2.2780	*L. braziliensis*	50	814	863
LbrM20_V2.2780	*L. braziliensis*	150	876	1025
LbrM20_V2.2810	*L. braziliensis*	41	48	88
LbrM20_V2.2810	*L. braziliensis*	121	396	516
LbrM20_V2.2810	*L. braziliensis*	91	665	755
LbrM20_V2.2810	*L. braziliensis*	73	1291	1363
LbrM20_V2.2810	*L. braziliensis*	57	1748	1804
LbrM20_V2.2840	*L. braziliensis*	89	241	329
LbrM20_V2.2840	*L. braziliensis*	48	332	379
LbrM20_V2.2840	*L. braziliensis*	40	495	534
LbrM20_V2.2840	*L. braziliensis*	104	537	640
LbrM20_V2.2850	*L. braziliensis*	59	125	183
LbrM20_V2.2860	*L. braziliensis*	51	95	145
LbrM20_V2.2860	*L. braziliensis*	101	238	338

LbrM20_V2.2860	*L. braziliensis*	113	367	479
LbrM20_V2.2880	*L. braziliensis*	42	391	432
LbrM20_V2.2890	*L. braziliensis*	40	173	212
LbrM20_V2.2930	*L. braziliensis*	46	9	54
LbrM20_V2.2930	*L. braziliensis*	129	310	438
LbrM20_V2.2930	*L. braziliensis*	85	464	548
LbrM20_V2.2940	*L. braziliensis*	121	377	497
LbrM20_V2.2980	*L. braziliensis*	50	117	166
LbrM20_V2.2980	*L. braziliensis*	88	249	336
LbrM20_V2.2990	*L. braziliensis*	68	235	302
LbrM20_V2.3010	*L. braziliensis*	41	547	587
LbrM20_V2.3020	*L. braziliensis*	42	137	178
LbrM20_V2.3020	*L. braziliensis*	170	468	637
LbrM20_V2.3030	*L. braziliensis*	71	46	116
LbrM20_V2.3030	*L. braziliensis*	64	296	359
LbrM20_V2.3030	*L. braziliensis*	62	376	437
LbrM20_V2.3030	*L. braziliensis*	141	518	658
LbrM20_V2.3030	*L. braziliensis*	285	817	1101
LbrM20_V2.3030	*L. braziliensis*	56	1126	1181
LbrM20_V2.3070	*L. braziliensis*	63	32	94
LbrM20_V2.3110	*L. braziliensis*	45	1359	1403
LbrM20_V2.3110	*L. braziliensis*	45	1887	1931
LbrM20_V2.3110	*L. braziliensis*	45	1887	1931
LbrM20_V2.3130	*L. braziliensis*	132	101	232
LbrM20_V2.3130	*L. braziliensis*	84	242	325
LbrM20_V2.3140	*L. braziliensis*	130	195	324
LbrM20_V2.3150	*L. braziliensis*	658	112	769
LbrM20_V2.3150	*L. braziliensis*	226	824	1049
LbrM20_V2.3160	*L. braziliensis*	65	67	131
LbrM20_V2.3170	*L. braziliensis*	42	123	164
LbrM20_V2.3170	*L. braziliensis*	62	259	320
LbrM20_V2.3170	*L. braziliensis*	62	259	320
LbrM20_V2.3220	*L. braziliensis*	84	150	233
LbrM20_V2.3280	*L. braziliensis*	67	45	111
LbrM20_V2.3280	*L. braziliensis*	41	593	633
LbrM20_V2.3280	*L. braziliensis*	46	858	903
LbrM20_V2.3280	*L. braziliensis*	68	1309	1376
LbrM20_V2.3290	*L. braziliensis*	54	180	233
LbrM20_V2.3300	*L. braziliensis*	120	51	170
LbrM20_V2.3320	*L. braziliensis*	44	1	44
LbrM20_V2.3340	*L. braziliensis*	43	101	143
LbrM20_V2.3340	*L. braziliensis*	142	188	329
LbrM20_V2.3340	*L. braziliensis*	166	335	500
LbrM20_V2.3350	*L. braziliensis*	60	22	81
LbrM20_V2.3350	*L. braziliensis*	73	92	164
LbrM20_V2.3350	*L. braziliensis*	189	323	511
LbrM20_V2.3370	*L. braziliensis*	51	1	51

LbrM20_V2.3370	*L. braziliensis*	52	148	199
LbrM20_V2.3370	*L. braziliensis*	52	148	199
LbrM20_V2.3380	*L. braziliensis*	42	17	58
LbrM20_V2.3390	*L. braziliensis*	72	642	713
LbrM20_V2.3390	*L. braziliensis*	58	942	999
LbrM20_V2.3390	*L. braziliensis*	42	1059	1100
LbrM20_V2.3410	*L. braziliensis*	58	140	197
LbrM20_V2.3430	*L. braziliensis*	72	1	72
LbrM20_V2.3430	*L. braziliensis*	51	106	156
LbrM20_V2.3440	*L. braziliensis*	113	1	113
LbrM20_V2.3440	*L. braziliensis*	60	122	181
LbrM20_V2.3440	*L. braziliensis*	60	122	181
LbrM20_V2.3480	*L. braziliensis*	144	1	144
LbrM20_V2.3480	*L. braziliensis*	64	547	610
LbrM20_V2.3480	*L. braziliensis*	64	547	610
LbrM20_V2.3490	*L. braziliensis*	50	2589	2638
LbrM20_V2.3490	*L. braziliensis*	85	3192	3276
LbrM20_V2.3490	*L. braziliensis*	69	3399	3467
LbrM20_V2.3490	*L. braziliensis*	85	3716	3800
LbrM20_V2.3510	*L. braziliensis*	67	115	181
LbrM20_V2.3530	*L. braziliensis*	42	1	42
LbrM20_V2.3530	*L. braziliensis*	42	83	124
LbrM20_V2.3530	*L. braziliensis*	92	126	217
LbrM20_V2.3550	*L. braziliensis*	50	1	50
LbrM20_V2.3550	*L. braziliensis*	49	747	795
LbrM20_V2.3550	*L. braziliensis*	139	2433	2571
LbrM20_V2.3560	*L. braziliensis*	178	1	178
LbrM20_V2.3590	*L. braziliensis*	52	227	278
LbrM20_V2.3590	*L. braziliensis*	45	347	391
LbrM20_V2.3590	*L. braziliensis*	59	603	661
LbrM20_V2.3600	*L. braziliensis*	87	634	720
LbrM20_V2.3630	*L. braziliensis*	46	126	171
LbrM20_V2.3650	*L. braziliensis*	80	1	80
LbrM20_V2.3670	*L. braziliensis*	49	42	90
LbrM20_V2.3680	*L. braziliensis*	41	1	41
LbrM20_V2.3680	*L. braziliensis*	50	69	118
LbrM20_V2.3680	*L. braziliensis*	51	127	177
LbrM20_V2.3680	*L. braziliensis*	75	600	674
LbrM20_V2.3700	*L. braziliensis*	56	1	56
LbrM20_V2.3710	*L. braziliensis*	44	60	103
LbrM20_V2.3720	*L. braziliensis*	205	56	260
LbrM20_V2.3730	*L. braziliensis*	48	20	67
LbrM20_V2.3740	*L. braziliensis*	55	124	178
LbrM20_V2.3740	*L. braziliensis*	52	432	483
LbrM20_V2.3740	*L. braziliensis*	67	730	796
LbrM20_V2.3740	*L. braziliensis*	43	1503	1545
LbrM20_V2.3770	*L. braziliensis*	108	1	108

LbrM20_V2.3770	*L. braziliensis*	192	131	322
LbrM20_V2.3770	*L. braziliensis*	101	369	469
LbrM20_V2.3780	*L. braziliensis*	47	420	466
LbrM20_V2.3790	*L. braziliensis*	42	1054	1095
LbrM20_V2.3790	*L. braziliensis*	70	2837	2906
LbrM20_V2.3790	*L. braziliensis*	73	3046	3118
LbrM20_V2.3790	*L. braziliensis*	67	3349	3415
LbrM20_V2.3800	*L. braziliensis*	61	1	61
LbrM20_V2.3800	*L. braziliensis*	75	75	149
LbrM20_V2.3810	*L. braziliensis*	127	1	127
LbrM20_V2.3840	*L. braziliensis*	288	84	371
LbrM20_V2.3840	*L. braziliensis*	83	416	498
LbrM20_V2.3870	*L. braziliensis*	47	107	153
LbrM20_V2.3870	*L. braziliensis*	91	167	257
LbrM20_V2.3880	*L. braziliensis*	89	17	105
LbrM20_V2.3890	*L. braziliensis*	89	21	109
LbrM20_V2.3890	*L. braziliensis*	187	453	639
LbrM20_V2.3890	*L. braziliensis*	136	661	796
LbrM20_V2.3900	*L. braziliensis*	51	1	51
LbrM20_V2.3910	*L. braziliensis*	71	1	71
LbrM20_V2.3910	*L. braziliensis*	104	122	225
LbrM20_V2.3930	*L. braziliensis*	52	814	865
LbrM20_V2.3930	*L. braziliensis*	46	1085	1130
LbrM20_V2.3990	*L. braziliensis*	50	441	490
LbrM20_V2.3990	*L. braziliensis*	75	1349	1423
LbrM20_V2.3990	*L. braziliensis*	44	2319	2362
LbrM20_V2.4000	*L. braziliensis*	108	1	108
LbrM20_V2.4010	*L. braziliensis*	79	1	79
LbrM20_V2.4020	*L. braziliensis*	137	191	327
LbrM20_V2.4060	*L. braziliensis*	117	1	117
LbrM20_V2.4100	*L. braziliensis*	59	352	410
LbrM20_V2.4120	*L. braziliensis*	74	265	338
LbrM20_V2.4130	*L. braziliensis*	85	1	85
LbrM20_V2.4150	*L. braziliensis*	120	27	146
LbrM20_V2.4150	*L. braziliensis*	175	161	335
LbrM20_V2.4150	*L. braziliensis*	51	584	634
LbrM20_V2.4150	*L. braziliensis*	76	863	938
LbrM20_V2.4170	*L. braziliensis*	80	256	335
LbrM20_V2.4180	*L. braziliensis*	45	38	82
LbrM20_V2.4180	*L. braziliensis*	43	658	700
LbrM20_V2.4180	*L. braziliensis*	43	658	700
LbrM20_V2.4190	*L. braziliensis*	87	360	446
LbrM20_V2.4210	*L. braziliensis*	55	390	444
LbrM20_V2.4220	*L. braziliensis*	150	184	333
LbrM20_V2.4220	*L. braziliensis*	189	411	599
LbrM20_V2.4220	*L. braziliensis*	42	689	730
LbrM20_V2.4220	*L. braziliensis*	41	767	807

LbrM20_V2.4220	*L. braziliensis*	92	1098	1189
LbrM20_V2.4230	*L. braziliensis*	48	706	753
LbrM20_V2.4240	*L. braziliensis*	138	542	679
LbrM20_V2.4260	*L. braziliensis*	217	55	271
LbrM20_V2.4270	*L. braziliensis*	50	50	99
LbrM20_V2.4270	*L. braziliensis*	59	304	362
LbrM20_V2.4270	*L. braziliensis*	47	398	444
LbrM20_V2.4270	*L. braziliensis*	48	501	548
LbrM20_V2.4280	*L. braziliensis*	95	58	152
LbrM20_V2.4280	*L. braziliensis*	771	188	958
LbrM20_V2.4290	*L. braziliensis*	228	25	252
LbrM20_V2.4330	*L. braziliensis*	48	350	397
LbrM20_V2.4370	*L. braziliensis*	65	42	106
LbrM20_V2.4370	*L. braziliensis*	63	156	218
LbrM20_V2.4370	*L. braziliensis*	171	272	442
LbrM20_V2.4370	*L. braziliensis*	42	702	743
LbrM20_V2.4380	*L. braziliensis*	177	1	177
LbrM20_V2.4380	*L. braziliensis*	44	208	251
LbrM20_V2.4390	*L. braziliensis*	64	118	181
LbrM20_V2.4390	*L. braziliensis*	43	367	409
LbrM20_V2.4390	*L. braziliensis*	43	367	409
LbrM20_V2.4430	*L. braziliensis*	67	1	67
LbrM20_V2.4450	*L. braziliensis*	72	1	72
LbrM20_V2.4460	*L. braziliensis*	41	872	912
LbrM20_V2.4460	*L. braziliensis*	41	1330	1370
LbrM20_V2.4460	*L. braziliensis*	72	1491	1562
LbrM20_V2.4460	*L. braziliensis*	49	2270	2318
LbrM20_V2.4460	*L. braziliensis*	48	2383	2430
LbrM20_V2.4460	*L. braziliensis*	53	2469	2521
LbrM20_V2.4460	*L. braziliensis*	63	2842	2904
LbrM20_V2.4460	*L. braziliensis*	94	3213	3306
LbrM20_V2.4460	*L. braziliensis*	550	3413	3962
LbrM20_V2.4460	*L. braziliensis*	40	4095	4134
LbrM20_V2.4460	*L. braziliensis*	47	4178	4224
LbrM20_V2.4480	*L. braziliensis*	55	101	155
LbrM20_V2.4480	*L. braziliensis*	40	222	261
LbrM20_V2.4480	*L. braziliensis*	40	222	261
LbrM20_V2.4490	*L. braziliensis*	220	92	311
LbrM20_V2.4500	*L. braziliensis*	53	1	53
LbrM20_V2.4510	*L. braziliensis*	60	396	455
LbrM20_V2.4510	*L. braziliensis*	50	706	755
LbrM20_V2.4510	*L. braziliensis*	50	706	755
LbrM20_V2.4540	*L. braziliensis*	40	134	173
LbrM20_V2.4540	*L. braziliensis*	79	213	291
LbrM20_V2.4540	*L. braziliensis*	169	447	615
LbrM20_V2.4550	*L. braziliensis*	200	1	200
LbrM20_V2.4560	*L. braziliensis*	73	170	242

LbrM20_V2.4560	*L. braziliensis*	86	305	390
LbrM20_V2.4560	*L. braziliensis*	86	305	390
LbrM20_V2.4570	*L. braziliensis*	43	73	115
LbrM20_V2.4570	*L. braziliensis*	41	192	232
LbrM20_V2.4570	*L. braziliensis*	41	192	232
LbrM20_V2.4580	*L. braziliensis*	115	1	115
LbrM20_V2.4580	*L. braziliensis*	114	392	505
LbrM20_V2.4590	*L. braziliensis*	51	68	118
LbrM20_V2.4590	*L. braziliensis*	122	152	273
LbrM20_V2.4590	*L. braziliensis*	285	299	583
LbrM20_V2.4600	*L. braziliensis*	40	264	303
LbrM20_V2.4610	*L. braziliensis*	50	299	348
LbrM20_V2.4610	*L. braziliensis*	63	414	476
LbrM20_V2.4610	*L. braziliensis*	63	414	476
LbrM20_V2.4620	*L. braziliensis*	55	473	527
LbrM20_V2.4620	*L. braziliensis*	156	597	752
LbrM20_V2.4620	*L. braziliensis*	56	1360	1415
LbrM20_V2.4630	*L. braziliensis*	44	218	261
LbrM20_V2.4640	*L. braziliensis*	78	212	289
LbrM20_V2.4650	*L. braziliensis*	62	179	240
LbrM20_V2.4650	*L. braziliensis*	60	313	372
LbrM20_V2.4650	*L. braziliensis*	60	442	501
LbrM20_V2.4650	*L. braziliensis*	68	506	573
LbrM20_V2.4660	*L. braziliensis*	427	1	427
LbrM20_V2.4660	*L. braziliensis*	231	430	660
LbrM20_V2.4660	*L. braziliensis*	671	672	1342
LbrM20_V2.4670	*L. braziliensis*	137	1	137
LbrM20_V2.4680	*L. braziliensis*	43	24	66
LbrM20_V2.4680	*L. braziliensis*	89	325	413
LbrM20_V2.4680	*L. braziliensis*	89	325	413
LbrM20_V2.4740	*L. braziliensis*	46	232	277
LbrM20_V2.4750	*L. braziliensis*	58	329	386
LbrM20_V2.4760	*L. braziliensis*	365	103	467
LbrM20_V2.4760	*L. braziliensis*	108	470	577
LbrM20_V2.4760	*L. braziliensis*	56	685	740
LbrM20_V2.4780	*L. braziliensis*	45	1	45
LbrM20_V2.4780	*L. braziliensis*	106	127	232
LbrM20_V2.4780	*L. braziliensis*	106	127	232
LbrM20_V2.4790	*L. braziliensis*	41	37	77
LbrM20_V2.4790	*L. braziliensis*	67	132	198
LbrM20_V2.4790	*L. braziliensis*	49	240	288
LbrM20_V2.4790	*L. braziliensis*	71	441	511
LbrM20_V2.4810	*L. braziliensis*	53	122	174
LbrM20_V2.4810	*L. braziliensis*	126	408	533
LbrM20_V2.4810	*L. braziliensis*	126	408	533
LbrM20_V2.4820	*L. braziliensis*	149	1	149
LbrM20_V2.4820	*L. braziliensis*	69	367	435

LbrM20_V2.4830	*L. braziliensis*	67	62	128
LbrM20_V2.4830	*L. braziliensis*	211	175	385
LbrM20_V2.4830	*L. braziliensis*	109	498	606
LbrM20_V2.4850	*L. braziliensis*	88	1	88
LbrM20_V2.4850	*L. braziliensis*	87	189	275
LbrM20_V2.4850	*L. braziliensis*	75	338	412
LbrM20_V2.4850	*L. braziliensis*	182	439	620
LbrM20_V2.4850	*L. braziliensis*	131	670	800
LbrM20_V2.4850	*L. braziliensis*	53	821	873
LbrM20_V2.4850	*L. braziliensis*	234	878	1111
LbrM20_V2.4850	*L. braziliensis*	147	1122	1268
LbrM20_V2.4850	*L. braziliensis*	48	1434	1481
LbrM20_V2.4850	*L. braziliensis*	53	1490	1542
LbrM20_V2.4860	*L. braziliensis*	195	19	213
LbrM20_V2.4860	*L. braziliensis*	63	225	287
LbrM20_V2.4860	*L. braziliensis*	141	311	451
LbrM20_V2.4860	*L. braziliensis*	280	589	868
LbrM20_V2.4860	*L. braziliensis*	81	886	966
LbrM20_V2.4860	*L. braziliensis*	56	1002	1057
LbrM20_V2.4860	*L. braziliensis*	62	1115	1176
LbrM20_V2.4870	*L. braziliensis*	161	1	161
LbrM20_V2.4870	*L. braziliensis*	61	184	244
LbrM20_V2.4870	*L. braziliensis*	81	522	602
LbrM20_V2.4870	*L. braziliensis*	315	745	1059
LbrM20_V2.4880	*L. braziliensis*	67	145	211
LbrM20_V2.4880	*L. braziliensis*	104	356	459
LbrM20_V2.4890	*L. braziliensis*	41	116	156
LbrM20_V2.4920	*L. braziliensis*	53	71	123
LbrM20_V2.4920	*L. braziliensis*	135	159	293
LbrM20_V2.4920	*L. braziliensis*	172	433	604
LbrM20_V2.4920	*L. braziliensis*	184	651	834
LbrM20_V2.4920	*L. braziliensis*	445	836	1280
LbrM20_V2.4930	*L. braziliensis*	155	119	273
LbrM20_V2.4930	*L. braziliensis*	377	621	997
LbrM20_V2.4940	*L. braziliensis*	176	34	209
LbrM20_V2.4940	*L. braziliensis*	74	454	527
LbrM20_V2.4940	*L. braziliensis*	45	544	588
LbrM20_V2.4940	*L. braziliensis*	55	651	705
LbrM20_V2.4940	*L. braziliensis*	64	838	901
LbrM20_V2.4950	*L. braziliensis*	248	1	248
LbrM20_V2.4950	*L. braziliensis*	422	292	713
LbrM20_V2.4950	*L. braziliensis*	78	739	816
LbrM20_V2.4950	*L. braziliensis*	146	840	985
LbrM20_V2.4950	*L. braziliensis*	81	1012	1092
LbrM20_V2.4950	*L. braziliensis*	89	1117	1205
LbrM20_V2.4950	*L. braziliensis*	577	1207	1783
LbrM20_V2.4950	*L. braziliensis*	204	1804	2007

LbrM20_V2.4950	*L. braziliensis*	169	2188	2356
LbrM20_V2.4950	*L. braziliensis*	173	2451	2623
LbrM20_V2.4950	*L. braziliensis*	85	2640	2724
LbrM20_V2.4950	*L. braziliensis*	285	3019	3303
LbrM20_V2.4950	*L. braziliensis*	121	3818	3938
LbrM20_V2.4960	*L. braziliensis*	98	82	179
LbrM20_V2.4960	*L. braziliensis*	70	458	527
LbrM20_V2.4970	*L. braziliensis*	55	32	86
LbrM20_V2.4970	*L. braziliensis*	67	93	159
LbrM20_V2.4970	*L. braziliensis*	41	337	377
LbrM20_V2.4970	*L. braziliensis*	53	696	748
LbrM20_V2.4980	*L. braziliensis*	114	1	114
LbrM20_V2.4980	*L. braziliensis*	116	161	276
LbrM20_V2.4980	*L. braziliensis*	55	287	341
LbrM20_V2.5030	*L. braziliensis*	48	163	210
LbrM20_V2.5030	*L. braziliensis*	92	608	699
LbrM20_V2.5030	*L. braziliensis*	64	755	818
LbrM20_V2.5030	*L. braziliensis*	66	975	1040
LbrM20_V2.5030	*L. braziliensis*	51	1170	1220
LbrM20_V2.5030	*L. braziliensis*	118	1354	1471
LbrM20_V2.5030	*L. braziliensis*	86	1496	1581
LbrM20_V2.5040	*L. braziliensis*	67	179	245
LbrM20_V2.5050	*L. braziliensis*	49	146	194
LbrM20_V2.5050	*L. braziliensis*	56	262	317
LbrM20_V2.5050	*L. braziliensis*	90	601	690
LbrM20_V2.5050	*L. braziliensis*	84	710	793
LbrM20_V2.5060	*L. braziliensis*	60	97	156
LbrM20_V2.5070	*L. braziliensis*	54	1	54
LbrM20_V2.5070	*L. braziliensis*	45	135	179
LbrM20_V2.5070	*L. braziliensis*	73	203	275
LbrM20_V2.5080	*L. braziliensis*	44	34	77
LbrM20_V2.5080	*L. braziliensis*	78	89	166
LbrM20_V2.5080	*L. braziliensis*	154	454	607
LbrM20_V2.5080	*L. braziliensis*	47	778	824
LbrM20_V2.5090	*L. braziliensis*	175	97	271
LbrM20_V2.5100	*L. braziliensis*	91	37	127
LbrM20_V2.5100	*L. braziliensis*	80	140	219
LbrM20_V2.5100	*L. braziliensis*	83	337	419
LbrM20_V2.5100	*L. braziliensis*	41	529	569
LbrM20_V2.5110	*L. braziliensis*	78	166	243
LbrM20_V2.5110	*L. braziliensis*	45	476	520
LbrM20_V2.5110	*L. braziliensis*	53	669	721
LbrM20_V2.5110	*L. braziliensis*	61	749	809
LbrM20_V2.5110	*L. braziliensis*	50	1787	1836
LbrM20_V2.5110	*L. braziliensis*	41	1921	1961
LbrM20_V2.5110	*L. braziliensis*	126	1990	2115
LbrM20_V2.5120	*L. braziliensis*	155	197	351

LbrM20_V2.5120	*L. braziliensis*	77	356	432
LbrM20_V2.5130	*L. braziliensis*	68	1	68
LbrM20_V2.5130	*L. braziliensis*	49	119	167
LbrM20_V2.5130	*L. braziliensis*	56	310	365
LbrM20_V2.5130	*L. braziliensis*	44	383	426
LbrM20_V2.5130	*L. braziliensis*	274	463	736
LbrM20_V2.5140	*L. braziliensis*	195	1	195
LbrM20_V2.5140	*L. braziliensis*	69	269	337
LbrM20_V2.5140	*L. braziliensis*	130	365	494
LbrM20_V2.5140	*L. braziliensis*	74	580	653
LbrM20_V2.5140	*L. braziliensis*	147	692	838
LbrM20_V2.5140	*L. braziliensis*	141	864	1004
LbrM20_V2.5140	*L. braziliensis*	292	1043	1334
LbrM20_V2.5140	*L. braziliensis*	251	1432	1682
LbrM20_V2.5140	*L. braziliensis*	148	1722	1869
LbrM20_V2.5140	*L. braziliensis*	350	1960	2309
LbrM20_V2.5140	*L. braziliensis*	208	2324	2531
LbrM20_V2.5140	*L. braziliensis*	58	2549	2606
LbrM20_V2.5140	*L. braziliensis*	101	2619	2719
LbrM20_V2.5140	*L. braziliensis*	76	3037	3112
LbrM20_V2.5140	*L. braziliensis*	83	3270	3352
LbrM20_V2.5140	*L. braziliensis*	88	3425	3512
LbrM20_V2.5160	*L. braziliensis*	91	333	423
LbrM20_V2.5160	*L. braziliensis*	97	449	545
LbrM20_V2.5160	*L. braziliensis*	97	449	545
LbrM20_V2.5170	*L. braziliensis*	609	121	729
LbrM20_V2.5190	*L. braziliensis*	128	1	128
LbrM20_V2.5190	*L. braziliensis*	273	136	408
LbrM20_V2.5190	*L. braziliensis*	107	445	551
LbrM20_V2.5190	*L. braziliensis*	170	617	786
LbrM20_V2.5190	*L. braziliensis*	296	816	1111
LbrM20_V2.5190	*L. braziliensis*	202	1117	1318
LbrM20_V2.5200	*L. braziliensis*	151	1	151
LbrM20_V2.5200	*L. braziliensis*	53	165	217
LbrM20_V2.5210	*L. braziliensis*	204	1	204
LbrM20_V2.5210	*L. braziliensis*	132	285	416
LbrM20_V2.5210	*L. braziliensis*	77	421	497
LbrM20_V2.5210	*L. braziliensis*	147	502	648
LbrM20_V2.5210	*L. braziliensis*	128	700	827
LbrM20_V2.5210	*L. braziliensis*	92	911	1002
LbrM20_V2.5220	*L. braziliensis*	112	1	112
LbrM20_V2.5220	*L. braziliensis*	372	129	500
LbrM20_V2.5220	*L. braziliensis*	291	618	908
LbrM20_V2.5220	*L. braziliensis*	373	926	1298
LbrM20_V2.5230	*L. braziliensis*	267	1	267
LbrM20_V2.5240	*L. braziliensis*	243	1	243
LbrM20_V2.5240	*L. braziliensis*	55	285	339

LbrM20_V2.5240	*L. braziliensis*	63	427	489
LbrM20_V2.5240	*L. braziliensis*	73	657	729
LbrM20_V2.5240	*L. braziliensis*	204	1004	1207
LbrM20_V2.5240	*L. braziliensis*	110	1239	1348
LbrM20_V2.5240	*L. braziliensis*	54	1353	1406
LbrM20_V2.5240	*L. braziliensis*	66	1445	1510
LbrM20_V2.5240	*L. braziliensis*	209	1579	1787
LbrM20_V2.5240	*L. braziliensis*	126	1872	1997
LbrM20_V2.5240	*L. braziliensis*	145	1999	2143
LbrM20_V2.5240	*L. braziliensis*	262	2298	2559
LbrM20_V2.5250	*L. braziliensis*	275	1	275
LbrM20_V2.5250	*L. braziliensis*	416	350	765
LbrM20_V2.5250	*L. braziliensis*	52	793	844
LbrM20_V2.5250	*L. braziliensis*	120	891	1010
LbrM20_V2.5250	*L. braziliensis*	183	1186	1368
LbrM20_V2.5250	*L. braziliensis*	136	1416	1551
LbrM20_V2.5250	*L. braziliensis*	99	1665	1763
LbrM20_V2.5250	*L. braziliensis*	53	1923	1975
LbrM20_V2.5250	*L. braziliensis*	131	2057	2187
LbrM20_V2.5250	*L. braziliensis*	40	2257	2296
LbrM20_V2.5260	*L. braziliensis*	145	48	192
LbrM20_V2.5260	*L. braziliensis*	82	233	314
LbrM20_V2.5260	*L. braziliensis*	40	348	387
LbrM20_V2.5260	*L. braziliensis*	58	480	537
LbrM20_V2.5260	*L. braziliensis*	42	811	852
LbrM20_V2.5260	*L. braziliensis*	45	963	1007
LbrM20_V2.5260	*L. braziliensis*	91	1020	1110
LbrM20_V2.5260	*L. braziliensis*	52	1591	1642
LbrM20_V2.5260	*L. braziliensis*	200	1700	1899
LbrM20_V2.5270	*L. braziliensis*	107	1	107
LbrM20_V2.5270	*L. braziliensis*	1024	115	1138
LbrM20_V2.5280	*L. braziliensis*	71	1	71
LbrM20_V2.5280	*L. braziliensis*	77	89	165
LbrM20_V2.5280	*L. braziliensis*	41	188	228
LbrM20_V2.5280	*L. braziliensis*	138	610	747
LbrM20_V2.5280	*L. braziliensis*	211	797	1007
LbrM20_V2.5280	*L. braziliensis*	136	1613	1748
LbrM20_V2.5280	*L. braziliensis*	206	1859	2064
LbrM20_V2.5290	*L. braziliensis*	62	67	128
LbrM20_V2.5290	*L. braziliensis*	107	281	387
LbrM20_V2.5290	*L. braziliensis*	47	485	531
LbrM20_V2.5290	*L. braziliensis*	88	566	653
LbrM20_V2.5300	*L. braziliensis*	50	103	152
LbrM20_V2.5310	*L. braziliensis*	52	150	201
LbrM20_V2.5340	*L. braziliensis*	220	31	250
LbrM20_V2.5340	*L. braziliensis*	58	284	341
LbrM20_V2.5350	*L. braziliensis*	65	21	85

LbrM20_V2.5350	*L. braziliensis*	46	117	162
LbrM20_V2.5350	*L. braziliensis*	46	117	162
LbrM20_V2.5370	*L. braziliensis*	71	1	71
LbrM20_V2.5370	*L. braziliensis*	68	160	227
LbrM20_V2.5370	*L. braziliensis*	68	160	227
LbrM20_V2.5380	*L. braziliensis*	178	1	178
LbrM20_V2.5410	*L. braziliensis*	56	546	601
LbrM20_V2.5420	*L. braziliensis*	135	1	135
LbrM20_V2.5420	*L. braziliensis*	42	193	234
LbrM20_V2.5460	*L. braziliensis*	224	1	224
LbrM20_V2.5460	*L. braziliensis*	72	291	362
LbrM20_V2.5460	*L. braziliensis*	134	467	600
LbrM20_V2.5460	*L. braziliensis*	204	872	1075
LbrM20_V2.5460	*L. braziliensis*	74	1311	1384
LbrM20_V2.5460	*L. braziliensis*	51	1416	1466
LbrM20_V2.5460	*L. braziliensis*	81	1605	1685
LbrM20_V2.5460	*L. braziliensis*	74	1757	1830
LbrM20_V2.5460	*L. braziliensis*	95	1914	2008
LbrM20_V2.5460	*L. braziliensis*	46	2603	2648
LbrM20_V2.5470	*L. braziliensis*	164	1	164
LbrM20_V2.5480	*L. braziliensis*	55	63	117
LbrM20_V2.5480	*L. braziliensis*	225	137	361
LbrM20_V2.5480	*L. braziliensis*	101	405	505
LbrM20_V2.5480	*L. braziliensis*	138	581	718
LbrM20_V2.5490	*L. braziliensis*	47	66	112
LbrM20_V2.5510	*L. braziliensis*	157	125	281
LbrM20_V2.5540	*L. braziliensis*	160	1	160
LbrM20_V2.5560	*L. braziliensis*	72	495	566
LbrM20_V2.5570	*L. braziliensis*	48	158	205
LbrM20_V2.5580	*L. braziliensis*	124	295	418
LbrM20_V2.5590	*L. braziliensis*	105	1	105
LbrM20_V2.5610	*L. braziliensis*	57	119	175
LbrM20_V2.5620	*L. braziliensis*	136	1	136
LbrM20_V2.5620	*L. braziliensis*	90	337	426
LbrM20_V2.5620	*L. braziliensis*	51	554	604
LbrM20_V2.5620	*L. braziliensis*	616	646	1261
LbrM20_V2.5630	*L. braziliensis*	199	54	252
LbrM20_V2.5640	*L. braziliensis*	101	52	152
LbrM20_V2.5650	*L. braziliensis*	54	17	70
LbrM20_V2.5650	*L. braziliensis*	69	402	470
LbrM20_V2.5650	*L. braziliensis*	183	640	822
LbrM20_V2.5660	*L. braziliensis*	118	104	221
LbrM20_V2.5660	*L. braziliensis*	48	301	348
LbrM20_V2.5660	*L. braziliensis*	72	460	531
LbrM20_V2.5660	*L. braziliensis*	85	578	662
LbrM20_V2.5660	*L. braziliensis*	381	715	1095
LbrM20_V2.5680	*L. braziliensis*	144	1	144

LbrM20_V2.5690	*L. braziliensis*	156	192	347
LbrM20_V2.5700	*L. braziliensis*	102	142	243
LbrM20_V2.5710	*L. braziliensis*	58	45	102
LbrM20_V2.5710	*L. braziliensis*	43	366	408
LbrM20_V2.5710	*L. braziliensis*	43	366	408
LbrM20_V2.5720	*L. braziliensis*	43	64	106
LbrM20_V2.5720	*L. braziliensis*	106	163	268
LbrM20_V2.5720	*L. braziliensis*	106	163	268
LbrM20_V2.5730	*L. braziliensis*	71	86	156
LbrM20_V2.5730	*L. braziliensis*	148	391	538
LbrM20_V2.5730	*L. braziliensis*	42	689	730
LbrM20_V2.5740	*L. braziliensis*	143	793	935
LbrM20_V2.5750	*L. braziliensis*	93	334	426
LbrM20_V2.5750	*L. braziliensis*	46	495	540
LbrM20_V2.5750	*L. braziliensis*	62	701	762
LbrM20_V2.5750	*L. braziliensis*	195	781	975
LbrM20_V2.5760	*L. braziliensis*	62	1347	1408
LbrM20_V2.5760	*L. braziliensis*	62	1729	1790
LbrM20_V2.5760	*L. braziliensis*	41	1864	1904
LbrM20_V2.5770	*L. braziliensis*	119	1	119
LbrM20_V2.5770	*L. braziliensis*	50	409	458
LbrM20_V2.5770	*L. braziliensis*	48	641	688
LbrM20_V2.5770	*L. braziliensis*	53	958	1010
LbrM20_V2.5770	*L. braziliensis*	48	1181	1228
LbrM20_V2.5770	*L. braziliensis*	44	1347	1390
LbrM20_V2.5770	*L. braziliensis*	72	1440	1511
LbrM20_V2.5770	*L. braziliensis*	148	1588	1735
LbrM20_V2.5780	*L. braziliensis*	45	211	255
LbrM20_V2.5780	*L. braziliensis*	50	262	311
LbrM20_V2.5780	*L. braziliensis*	51	449	499
LbrM20_V2.5780	*L. braziliensis*	44	678	721
LbrM20_V2.5780	*L. braziliensis*	81	743	823
LbrM20_V2.5790	*L. braziliensis*	148	37	184
LbrM20_V2.5800	*L. braziliensis*	65	90	154
LbrM20_V2.5800	*L. braziliensis*	49	165	213
LbrM20_V2.5800	*L. braziliensis*	49	165	213
LbrM20_V2.5810	*L. braziliensis*	45	1	45
LbrM20_V2.5820	*L. braziliensis*	136	265	400
LbrM20_V2.5830	*L. braziliensis*	101	1	101
LbrM20_V2.5830	*L. braziliensis*	51	266	316
LbrM20_V2.5840	*L. braziliensis*	47	31	77
LbrM20_V2.5840	*L. braziliensis*	45	102	146
LbrM20_V2.5840	*L. braziliensis*	41	761	801
LbrM20_V2.5860	*L. braziliensis*	77	1	77
LbrM20_V2.5860	*L. braziliensis*	91	101	191
LbrM20_V2.5860	*L. braziliensis*	44	235	278
LbrM20_V2.5870	*L. braziliensis*	51	103	153

LbrM20_V2.5870	*L. braziliensis*	44	561	604
LbrM20_V2.5870	*L. braziliensis*	44	561	604
LbrM20_V2.5880	*L. braziliensis*	55	183	237
LbrM20_V2.5890	*L. braziliensis*	51	276	326
LbrM20_V2.5900	*L. braziliensis*	48	72	119
LbrM20_V2.5920	*L. braziliensis*	44	1	44
LbrM20_V2.5940	*L. braziliensis*	92	80	171
LbrM20_V2.5940	*L. braziliensis*	54	617	670
LbrM20_V2.5940	*L. braziliensis*	78	941	1018
LbrM20_V2.5970	*L. braziliensis*	40	114	153
LbrM20_V2.5980	*L. braziliensis*	62	170	231
LbrM20_V2.5990	*L. braziliensis*	268	103	370
LbrM20_V2.5990	*L. braziliensis*	52	388	439
LbrM20_V2.6000	*L. braziliensis*	49	496	544
LbrM20_V2.6030	*L. braziliensis*	91	218	308
LbrM20_V2.6040	*L. braziliensis*	78	1	78
LbrM20_V2.6050	*L. braziliensis*	358	1	358
LbrM20_V2.6050	*L. braziliensis*	263	360	622
LbrM20_V2.6070	*L. braziliensis*	172	215	386
LbrM20_V2.6070	*L. braziliensis*	111	566	676
LbrM20_V2.6070	*L. braziliensis*	40	893	932
LbrM20_V2.6070	*L. braziliensis*	58	1467	1524
LbrM20_V2.6080	*L. braziliensis*	177	195	371
LbrM20_V2.6080	*L. braziliensis*	111	551	661
LbrM20_V2.6080	*L. braziliensis*	43	875	917
LbrM20_V2.6090	*L. braziliensis*	60	67	126
LbrM20_V2.6090	*L. braziliensis*	44	301	344
LbrM20_V2.6090	*L. braziliensis*	89	389	477
LbrM20_V2.6090	*L. braziliensis*	176	689	864
LbrM20_V2.6090	*L. braziliensis*	111	1044	1154
LbrM20_V2.6090	*L. braziliensis*	41	1371	1411
LbrM20_V2.6100	*L. braziliensis*	674	1	674
LbrM20_V2.6100	*L. braziliensis*	99	688	786
LbrM20_V2.6100	*L. braziliensis*	81	800	880
LbrM20_V2.6110	*L. braziliensis*	90	1	90
LbrM20_V2.6110	*L. braziliensis*	171	295	465
LbrM20_V2.6110	*L. braziliensis*	120	630	749
LbrM20_V2.6110	*L. braziliensis*	58	1540	1597
LbrM20_V2.6120	*L. braziliensis*	176	167	342
LbrM20_V2.6120	*L. braziliensis*	110	521	630
LbrM20_V2.6120	*L. braziliensis*	42	845	886
LbrM20_V2.6120	*L. braziliensis*	58	1422	1479
LbrM20_V2.6130	*L. braziliensis*	111	1	111
LbrM20_V2.6130	*L. braziliensis*	201	113	313
LbrM20_V2.6130	*L. braziliensis*	148	323	470
LbrM20_V2.6130	*L. braziliensis*	92	509	600
LbrM20_V2.6130	*L. braziliensis*	60	623	682

LbrM20_V2.6130	*L. braziliensis*	44	684	727
LbrM20_V2.6140	*L. braziliensis*	172	52	223
LbrM20_V2.6140	*L. braziliensis*	112	402	513
LbrM20_V2.6140	*L. braziliensis*	41	730	770
LbrM20_V2.6140	*L. braziliensis*	62	847	908
LbrM20_V2.6140	*L. braziliensis*	58	1304	1361
LbrM21_V2.0010	*L. braziliensis*	60	1	60
LbrM21_V2.0060	*L. braziliensis*	80	120	199
LbrM21_V2.0070	*L. braziliensis*	46	1	46
LbrM21_V2.0070	*L. braziliensis*	68	121	188
LbrM21_V2.0070	*L. braziliensis*	68	121	188
LbrM21_V2.0080	*L. braziliensis*	41	111	151
LbrM21_V2.0100	*L. braziliensis*	56	1	56
LbrM21_V2.0110	*L. braziliensis*	52	246	297
LbrM21_V2.0140	*L. braziliensis*	67	85	151
LbrM21_V2.0160	*L. braziliensis*	101	488	588
LbrM21_V2.0160	*L. braziliensis*	53	696	748
LbrM21_V2.0160	*L. braziliensis*	53	696	748
LbrM21_V2.0170	*L. braziliensis*	48	379	426
LbrM21_V2.0170	*L. braziliensis*	42	538	579
LbrM21_V2.0170	*L. braziliensis*	55	745	799
LbrM21_V2.0180	*L. braziliensis*	82	168	249
LbrM21_V2.0180	*L. braziliensis*	43	783	825
LbrM21_V2.0190	*L. braziliensis*	54	87	140
LbrM21_V2.0190	*L. braziliensis*	202	482	683
LbrM21_V2.0200	*L. braziliensis*	43	508	550
LbrM21_V2.0210	*L. braziliensis*	44	1	44
LbrM21_V2.0220	*L. braziliensis*	96	430	525
LbrM21_V2.0230	*L. braziliensis*	62	1	62
LbrM21_V2.0230	*L. braziliensis*	43	154	196
LbrM21_V2.0230	*L. braziliensis*	43	154	196
LbrM21_V2.0240	*L. braziliensis*	129	180	308
LbrM21_V2.0240	*L. braziliensis*	150	314	463
LbrM21_V2.0240	*L. braziliensis*	40	1221	1260
LbrM21_V2.0250	*L. braziliensis*	85	59	143
LbrM21_V2.0250	*L. braziliensis*	92	514	605
LbrM21_V2.0250	*L. braziliensis*	92	514	605
LbrM21_V2.0260	*L. braziliensis*	47	113	159
LbrM21_V2.0300	*L. braziliensis*	49	299	347
LbrM21_V2.0300	*L. braziliensis*	59	463	521
LbrM21_V2.0300	*L. braziliensis*	58	538	595
LbrM21_V2.0330	*L. braziliensis*	51	159	209
LbrM21_V2.0330	*L. braziliensis*	96	272	367
LbrM21_V2.0330	*L. braziliensis*	57	600	656
LbrM21_V2.0330	*L. braziliensis*	90	717	806
LbrM21_V2.0330	*L. braziliensis*	64	970	1033
LbrM21_V2.0340	*L. braziliensis*	88	185	272

LbrM21_V2.0340	*L. braziliensis*	46	414	459
LbrM21_V2.0350	*L. braziliensis*	69	39	107
LbrM21_V2.0350	*L. braziliensis*	68	153	220
LbrM21_V2.0350	*L. braziliensis*	52	277	328
LbrM21_V2.0350	*L. braziliensis*	53	371	423
LbrM21_V2.0350	*L. braziliensis*	59	558	616
LbrM21_V2.0350	*L. braziliensis*	53	648	700
LbrM21_V2.0350	*L. braziliensis*	73	779	851
LbrM21_V2.0350	*L. braziliensis*	41	1037	1077
LbrM21_V2.0360	*L. braziliensis*	48	1	48
LbrM21_V2.0360	*L. braziliensis*	61	182	242
LbrM21_V2.0380	*L. braziliensis*	73	1	73
LbrM21_V2.0380	*L. braziliensis*	111	127	237
LbrM21_V2.0390	*L. braziliensis*	76	1	76
LbrM21_V2.0390	*L. braziliensis*	77	641	717
LbrM21_V2.0390	*L. braziliensis*	183	856	1038
LbrM21_V2.0410	*L. braziliensis*	92	1	92
LbrM21_V2.0420	*L. braziliensis*	92	1	92
LbrM21_V2.0420	*L. braziliensis*	164	112	275
LbrM21_V2.0430	*L. braziliensis*	136	96	231
LbrM21_V2.0450	*L. braziliensis*	40	87	126
LbrM21_V2.0450	*L. braziliensis*	45	249	293
LbrM21_V2.0450	*L. braziliensis*	45	249	293
LbrM21_V2.0460	*L. braziliensis*	97	1	97
LbrM21_V2.0460	*L. braziliensis*	146	129	274
LbrM21_V2.0460	*L. braziliensis*	49	418	466
LbrM21_V2.0460	*L. braziliensis*	59	603	661
LbrM21_V2.0470	*L. braziliensis*	66	52	117
LbrM21_V2.0470	*L. braziliensis*	40	204	243
LbrM21_V2.0470	*L. braziliensis*	44	387	430
LbrM21_V2.0470	*L. braziliensis*	48	632	679
LbrM21_V2.0480	*L. braziliensis*	49	29	77
LbrM21_V2.0480	*L. braziliensis*	58	334	391
LbrM21_V2.0480	*L. braziliensis*	51	517	567
LbrM21_V2.0490	*L. braziliensis*	192	169	360
LbrM21_V2.0520	*L. braziliensis*	43	206	248
LbrM21_V2.0520	*L. braziliensis*	98	386	483
LbrM21_V2.0520	*L. braziliensis*	270	500	769
LbrM21_V2.0520	*L. braziliensis*	41	814	854
LbrM21_V2.0530	*L. braziliensis*	65	229	293
LbrM21_V2.0530	*L. braziliensis*	51	712	762
LbrM21_V2.0530	*L. braziliensis*	51	712	762
LbrM21_V2.0540	*L. braziliensis*	204	1	204
LbrM21_V2.0540	*L. braziliensis*	75	440	514
LbrM21_V2.0550	*L. braziliensis*	89	369	457
LbrM21_V2.0570	*L. braziliensis*	122	434	555
LbrM21_V2.0580	*L. braziliensis*	48	96	143

LbrM21_V2.0580	*L. braziliensis*	46	366	411
LbrM21_V2.0580	*L. braziliensis*	46	366	411
LbrM21_V2.0590	*L. braziliensis*	48	89	136
LbrM21_V2.0590	*L. braziliensis*	71	156	226
LbrM21_V2.0590	*L. braziliensis*	68	251	318
LbrM21_V2.0590	*L. braziliensis*	51	371	421
LbrM21_V2.0590	*L. braziliensis*	40	441	480
LbrM21_V2.0590	*L. braziliensis*	64	741	804
LbrM21_V2.0600	*L. braziliensis*	49	175	223
LbrM21_V2.0600	*L. braziliensis*	67	270	336
LbrM21_V2.0610	*L. braziliensis*	64	91	154
LbrM21_V2.0610	*L. braziliensis*	64	156	219
LbrM21_V2.0610	*L. braziliensis*	64	156	219
LbrM21_V2.0630	*L. braziliensis*	242	180	421
LbrM21_V2.0640	*L. braziliensis*	102	136	237
LbrM21_V2.0640	*L. braziliensis*	63	272	334
LbrM21_V2.0640	*L. braziliensis*	49	373	421
LbrM21_V2.0650	*L. braziliensis*	219	1	219
LbrM21_V2.0650	*L. braziliensis*	119	263	381
LbrM21_V2.0660	*L. braziliensis*	57	369	425
LbrM21_V2.0670	*L. braziliensis*	76	216	291
LbrM21_V2.0670	*L. braziliensis*	60	426	485
LbrM21_V2.0680	*L. braziliensis*	43	331	373
LbrM21_V2.0680	*L. braziliensis*	48	497	544
LbrM21_V2.0680	*L. braziliensis*	209	550	758
LbrM21_V2.0690	*L. braziliensis*	192	566	757
LbrM21_V2.0710	*L. braziliensis*	79	198	276
LbrM21_V2.0740	*L. braziliensis*	40	393	432
LbrM21_V2.0760	*L. braziliensis*	398	224	621
LbrM21_V2.0770	*L. braziliensis*	83	577	659
LbrM21_V2.0780	*L. braziliensis*	68	51	118
LbrM21_V2.0800	*L. braziliensis*	40	66	105
LbrM21_V2.0830	*L. braziliensis*	46	589	634
LbrM21_V2.0850	*L. braziliensis*	48	32	79
LbrM21_V2.0850	*L. braziliensis*	182	308	489
LbrM21_V2.0850	*L. braziliensis*	182	308	489
LbrM21_V2.0860	*L. braziliensis*	242	57	298
LbrM21_V2.0860	*L. braziliensis*	114	408	521
LbrM21_V2.0860	*L. braziliensis*	42	660	701
LbrM21_V2.0860	*L. braziliensis*	187	787	973
LbrM21_V2.0870	*L. braziliensis*	103	184	286
LbrM21_V2.0890	*L. braziliensis*	58	75	132
LbrM21_V2.0890	*L. braziliensis*	57	767	823
LbrM21_V2.0890	*L. braziliensis*	84	1071	1154
LbrM21_V2.0890	*L. braziliensis*	86	1405	1490
LbrM21_V2.0910	*L. braziliensis*	353	1	353
LbrM21_V2.0910	*L. braziliensis*	49	879	927

LbrM21_V2.0920	*L. braziliensis*	58	62	119
LbrM21_V2.0920	*L. braziliensis*	43	136	178
LbrM21_V2.0920	*L. braziliensis*	61	246	306
LbrM21_V2.0920	*L. braziliensis*	136	390	525
LbrM21_V2.0920	*L. braziliensis*	71	706	776
LbrM21_V2.0920	*L. braziliensis*	63	813	875
LbrM21_V2.0930	*L. braziliensis*	47	1	47
LbrM21_V2.0930	*L. braziliensis*	137	81	217
LbrM21_V2.0930	*L. braziliensis*	536	221	756
LbrM21_V2.0930	*L. braziliensis*	136	825	960
LbrM21_V2.0930	*L. braziliensis*	474	987	1460
LbrM21_V2.0930	*L. braziliensis*	406	1496	1901
LbrM21_V2.0930	*L. braziliensis*	83	1980	2062
LbrM21_V2.0930	*L. braziliensis*	755	2088	2842
LbrM21_V2.0930	*L. braziliensis*	64	2889	2952
LbrM21_V2.0930	*L. braziliensis*	87	2987	3073
LbrM21_V2.0930	*L. braziliensis*	84	3098	3181
LbrM21_V2.0950	*L. braziliensis*	163	1	163
LbrM21_V2.0960	*L. braziliensis*	59	60	118
LbrM21_V2.0980	*L. braziliensis*	109	127	235
LbrM21_V2.0980	*L. braziliensis*	73	353	425
LbrM21_V2.1010	*L. braziliensis*	163	156	318
LbrM21_V2.1010	*L. braziliensis*	231	495	725
LbrM21_V2.1010	*L. braziliensis*	60	788	847
LbrM21_V2.1010	*L. braziliensis*	77	910	986
LbrM21_V2.1010	*L. braziliensis*	240	1005	1244
LbrM21_V2.1010	*L. braziliensis*	280	1288	1567
LbrM21_V2.1010	*L. braziliensis*	198	1585	1782
LbrM21_V2.1010	*L. braziliensis*	200	1874	2073
LbrM21_V2.1010	*L. braziliensis*	40	2122	2161
LbrM21_V2.1010	*L. braziliensis*	70	2219	2288
LbrM21_V2.1020	*L. braziliensis*	41	1	41
LbrM21_V2.1020	*L. braziliensis*	55	91	145
LbrM21_V2.1020	*L. braziliensis*	46	181	226
LbrM21_V2.1030	*L. braziliensis*	231	1	231
LbrM21_V2.1030	*L. braziliensis*	364	283	646
LbrM21_V2.1030	*L. braziliensis*	327	680	1006
LbrM21_V2.1030	*L. braziliensis*	173	1008	1180
LbrM21_V2.1030	*L. braziliensis*	209	1201	1409
LbrM21_V2.1030	*L. braziliensis*	129	1470	1598
LbrM21_V2.1030	*L. braziliensis*	132	1701	1832
LbrM21_V2.1030	*L. braziliensis*	259	1951	2209
LbrM21_V2.1030	*L. braziliensis*	62	2217	2278
LbrM21_V2.1040	*L. braziliensis*	77	64	140
LbrM21_V2.1040	*L. braziliensis*	50	176	225
LbrM21_V2.1040	*L. braziliensis*	58	411	468
LbrM21_V2.1040	*L. braziliensis*	40	524	563

LbrM21_V2.1050	*L. braziliensis*	129	1	129
LbrM21_V2.1050	*L. braziliensis*	166	131	296
LbrM21_V2.1050	*L. braziliensis*	60	350	409
LbrM21_V2.1050	*L. braziliensis*	409	504	912
LbrM21_V2.1060	*L. braziliensis*	302	548	849
LbrM21_V2.1070	*L. braziliensis*	136	1	136
LbrM21_V2.1070	*L. braziliensis*	180	156	335
LbrM21_V2.1070	*L. braziliensis*	85	636	720
LbrM21_V2.1080	*L. braziliensis*	111	1	111
LbrM21_V2.1080	*L. braziliensis*	90	316	405
LbrM21_V2.1080	*L. braziliensis*	54	530	583
LbrM21_V2.1080	*L. braziliensis*	41	863	903
LbrM21_V2.1110	*L. braziliensis*	47	349	395
LbrM21_V2.1110	*L. braziliensis*	47	449	495
LbrM21_V2.1110	*L. braziliensis*	97	585	681
LbrM21_V2.1110	*L. braziliensis*	164	750	913
LbrM21_V2.1120	*L. braziliensis*	76	31	106
LbrM21_V2.1130	*L. braziliensis*	136	22	157
LbrM21_V2.1130	*L. braziliensis*	48	387	434
LbrM21_V2.1140	*L. braziliensis*	43	90	132
LbrM21_V2.1150	*L. braziliensis*	42	91	132
LbrM21_V2.1160	*L. braziliensis*	43	90	132
LbrM21_V2.1180	*L. braziliensis*	51	79	129
LbrM21_V2.1180	*L. braziliensis*	52	137	188
LbrM21_V2.1180	*L. braziliensis*	55	349	403
LbrM21_V2.1190	*L. braziliensis*	41	122	162
LbrM21_V2.1190	*L. braziliensis*	151	229	379
LbrM21_V2.1190	*L. braziliensis*	293	416	708
LbrM21_V2.1190	*L. braziliensis*	177	738	914
LbrM21_V2.1200	*L. braziliensis*	120	223	342
LbrM21_V2.1200	*L. braziliensis*	65	512	576
LbrM21_V2.1230	*L. braziliensis*	49	1	49
LbrM21_V2.1230	*L. braziliensis*	52	131	182
LbrM21_V2.1230	*L. braziliensis*	59	225	283
LbrM21_V2.1230	*L. braziliensis*	953	381	1333
LbrM21_V2.1230	*L. braziliensis*	45	1355	1399
LbrM21_V2.1230	*L. braziliensis*	238	1439	1676
LbrM21_V2.1230	*L. braziliensis*	375	1711	2085
LbrM21_V2.1250	*L. braziliensis*	80	1	80
LbrM21_V2.1250	*L. braziliensis*	75	232	306
LbrM21_V2.1260	*L. braziliensis*	145	1	145
LbrM21_V2.1260	*L. braziliensis*	668	170	837
LbrM21_V2.1270	*L. braziliensis*	57	87	143
LbrM21_V2.1270	*L. braziliensis*	56	513	568
LbrM21_V2.1270	*L. braziliensis*	50	635	684
LbrM21_V2.1270	*L. braziliensis*	342	855	1196
LbrM21_V2.1270	*L. braziliensis*	108	1323	1430

LbrM21_V2.1270	*L. braziliensis*	47	1624	1670
LbrM21_V2.1270	*L. braziliensis*	155	1680	1834
LbrM21_V2.1270	*L. braziliensis*	249	1871	2119
LbrM21_V2.1320	*L. braziliensis*	44	1	44
LbrM21_V2.1330	*L. braziliensis*	311	453	763
LbrM21_V2.1330	*L. braziliensis*	42	823	864
LbrM21_V2.1330	*L. braziliensis*	53	1240	1292
LbrM21_V2.1330	*L. braziliensis*	50	1295	1344
LbrM21_V2.1330	*L. braziliensis*	49	1486	1534
LbrM21_V2.1330	*L. braziliensis*	40	1691	1730
LbrM21_V2.1340	*L. braziliensis*	52	1	52
LbrM21_V2.1340	*L. braziliensis*	44	205	248
LbrM21_V2.1340	*L. braziliensis*	74	535	608
LbrM21_V2.1350	*L. braziliensis*	441	12	452
LbrM21_V2.1350	*L. braziliensis*	69	476	544
LbrM21_V2.1360	*L. braziliensis*	345	1	345
LbrM21_V2.1360	*L. braziliensis*	274	372	645
LbrM21_V2.1360	*L. braziliensis*	178	720	897
LbrM21_V2.1360	*L. braziliensis*	60	941	1000
LbrM21_V2.1390	*L. braziliensis*	129	125	253
LbrM21_V2.1390	*L. braziliensis*	51	738	788
LbrM21_V2.1400	*L. braziliensis*	136	1	136
LbrM21_V2.1410	*L. braziliensis*	104	180	283
LbrM21_V2.1420	*L. braziliensis*	234	53	286
LbrM21_V2.1420	*L. braziliensis*	144	395	538
LbrM21_V2.1420	*L. braziliensis*	103	613	715
LbrM21_V2.1420	*L. braziliensis*	255	722	976
LbrM21_V2.1420	*L. braziliensis*	363	1002	1364
LbrM21_V2.1440	*L. braziliensis*	71	1	71
LbrM21_V2.1440	*L. braziliensis*	146	418	563
LbrM21_V2.1440	*L. braziliensis*	64	565	628
LbrM21_V2.1440	*L. braziliensis*	160	748	907
LbrM21_V2.1440	*L. braziliensis*	95	941	1035
LbrM21_V2.1440	*L. braziliensis*	198	1042	1239
LbrM21_V2.1460	*L. braziliensis*	137	196	332
LbrM21_V2.1460	*L. braziliensis*	136	346	481
LbrM21_V2.1470	*L. braziliensis*	63	325	387
LbrM21_V2.1470	*L. braziliensis*	96	457	552
LbrM21_V2.1470	*L. braziliensis*	63	734	796
LbrM21_V2.1480	*L. braziliensis*	294	149	442
LbrM21_V2.1480	*L. braziliensis*	45	663	707
LbrM21_V2.1480	*L. braziliensis*	50	717	766
LbrM21_V2.1510	*L. braziliensis*	72	82	153
LbrM21_V2.1510	*L. braziliensis*	80	156	235
LbrM21_V2.1520	*L. braziliensis*	201	19	219
LbrM21_V2.1520	*L. braziliensis*	193	225	417
LbrM21_V2.1560	*L. braziliensis*	68	1	68

LbrM21_V2.1560	*L. braziliensis*	76	132	207
LbrM21_V2.1570	*L. braziliensis*	172	1	172
LbrM21_V2.1570	*L. braziliensis*	47	367	413
LbrM21_V2.1570	*L. braziliensis*	247	424	670
LbrM21_V2.1570	*L. braziliensis*	443	810	1252
LbrM21_V2.1570	*L. braziliensis*	114	1475	1588
LbrM21_V2.1590	*L. braziliensis*	67	1	67
LbrM21_V2.1590	*L. braziliensis*	281	77	357
LbrM21_V2.1590	*L. braziliensis*	67	359	425
LbrM21_V2.1590	*L. braziliensis*	85	912	996
LbrM21_V2.1590	*L. braziliensis*	456	1131	1586
LbrM21_V2.1600	*L. braziliensis*	44	123	166
LbrM21_V2.1610	*L. braziliensis*	181	330	510
LbrM21_V2.1610	*L. braziliensis*	100	517	616
LbrM21_V2.1610	*L. braziliensis*	49	740	788
LbrM21_V2.1610	*L. braziliensis*	84	816	899
LbrM21_V2.1610	*L. braziliensis*	112	938	1049
LbrM21_V2.1620	*L. braziliensis*	181	1	181
LbrM21_V2.1620	*L. braziliensis*	47	284	330
LbrM21_V2.1620	*L. braziliensis*	54	379	432
LbrM21_V2.1620	*L. braziliensis*	298	444	741
LbrM21_V2.1630	*L. braziliensis*	153	1	153
LbrM21_V2.1630	*L. braziliensis*	133	238	370
LbrM21_V2.1630	*L. braziliensis*	229	444	672
LbrM21_V2.1640	*L. braziliensis*	56	470	525
LbrM21_V2.1650	*L. braziliensis*	61	189	249
LbrM21_V2.1650	*L. braziliensis*	45	256	300
LbrM21_V2.1650	*L. braziliensis*	130	324	453
LbrM21_V2.1650	*L. braziliensis*	60	606	665
LbrM21_V2.1650	*L. braziliensis*	67	699	765
LbrM21_V2.1650	*L. braziliensis*	105	816	920
LbrM21_V2.1650	*L. braziliensis*	41	1177	1217
LbrM21_V2.1650	*L. braziliensis*	67	1273	1339
LbrM21_V2.1660	*L. braziliensis*	47	22	68
LbrM21_V2.1660	*L. braziliensis*	137	163	299
LbrM21_V2.1660	*L. braziliensis*	137	163	299
LbrM21_V2.1670	*L. braziliensis*	53	282	334
LbrM21_V2.1670	*L. braziliensis*	42	576	617
LbrM21_V2.1670	*L. braziliensis*	94	730	823
LbrM21_V2.1670	*L. braziliensis*	331	1413	1743
LbrM21_V2.1680	*L. braziliensis*	45	1	45
LbrM21_V2.1690	*L. braziliensis*	187	247	433
LbrM21_V2.1690	*L. braziliensis*	69	448	516
LbrM21_V2.1700	*L. braziliensis*	46	368	413
LbrM21_V2.1720	*L. braziliensis*	40	262	301
LbrM21_V2.1730	*L. braziliensis*	71	72	142
LbrM21_V2.1730	*L. braziliensis*	165	174	338

LbrM21_V2.1750	*L. braziliensis*	443	267	709
LbrM21_V2.1760	*L. braziliensis*	302	86	387
LbrM21_V2.1760	*L. braziliensis*	62	406	467
LbrM21_V2.1770	*L. braziliensis*	79	1	79
LbrM21_V2.1780	*L. braziliensis*	46	25	70
LbrM21_V2.1790	*L. braziliensis*	55	130	184
LbrM21_V2.1790	*L. braziliensis*	53	391	443
LbrM21_V2.1790	*L. braziliensis*	53	391	443
LbrM21_V2.1830	*L. braziliensis*	44	86	129
LbrM21_V2.1830	*L. braziliensis*	105	382	486
LbrM21_V2.1840	*L. braziliensis*	177	1	177
LbrM21_V2.1840	*L. braziliensis*	303	220	522
LbrM21_V2.1840	*L. braziliensis*	178	532	709
LbrM21_V2.1860	*L. braziliensis*	76	322	397
LbrM21_V2.1880	*L. braziliensis*	48	1	48
LbrM21_V2.1880	*L. braziliensis*	66	115	180
LbrM21_V2.1880	*L. braziliensis*	66	115	180
LbrM21_V2.1890	*L. braziliensis*	40	97	136
LbrM21_V2.1900	*L. braziliensis*	66	96	161
LbrM21_V2.1930	*L. braziliensis*	50	1	50
LbrM21_V2.1940	*L. braziliensis*	50	31	80
LbrM21_V2.1970	*L. braziliensis*	61	40	100
LbrM21_V2.1990	*L. braziliensis*	146	28	173
LbrM21_V2.1990	*L. braziliensis*	72	502	573
LbrM21_V2.1990	*L. braziliensis*	72	502	573
LbrM21_V2.2030	*L. braziliensis*	65	1	65
LbrM21_V2.2040	*L. braziliensis*	170	1	170
LbrM21_V2.2080	*L. braziliensis*	47	155	201
LbrM21_V2.2090	*L. braziliensis*	45	1	45
LbrM21_V2.2090	*L. braziliensis*	48	184	231
LbrM21_V2.2090	*L. braziliensis*	63	268	330
LbrM21_V2.2090	*L. braziliensis*	123	419	541
LbrM21_V2.2100	*L. braziliensis*	157	39	195
LbrM21_V2.2100	*L. braziliensis*	95	204	298
LbrM21_V2.2100	*L. braziliensis*	104	408	511
LbrM21_V2.2120	*L. braziliensis*	43	287	329
LbrM21_V2.2130	*L. braziliensis*	83	150	232
LbrM21_V2.2140	*L. braziliensis*	94	192	285
LbrM21_V2.2140	*L. braziliensis*	49	491	539
LbrM21_V2.2150	*L. braziliensis*	40	404	443
LbrM21_V2.2160	*L. braziliensis*	368	1	368
LbrM21_V2.2160	*L. braziliensis*	108	378	485
LbrM21_V2.2160	*L. braziliensis*	144	548	691
LbrM21_V2.2160	*L. braziliensis*	318	704	1021
LbrM21_V2.2160	*L. braziliensis*	594	1031	1624
LbrM21_V2.2160	*L. braziliensis*	83	1638	1720
LbrM21_V2.2170	*L. braziliensis*	43	153	195

LbrM21_V2.2170	*L. braziliensis*	85	229	313
LbrM21_V2.2170	*L. braziliensis*	85	229	313
LbrM21_V2.2180	*L. braziliensis*	101	112	212
LbrM21_V2.2190	*L. braziliensis*	63	105	167
LbrM21_V2.2190	*L. braziliensis*	111	235	345
LbrM22_V2.0010	*L. braziliensis*	40	1	40
LbrM22_V2.0010	*L. braziliensis*	62	503	564
LbrM22_V2.0010	*L. braziliensis*	62	503	564
LbrM22_V2.0020	*L. braziliensis*	48	98	145
LbrM22_V2.0030	*L. braziliensis*	50	139	188
LbrM22_V2.0050	*L. braziliensis*	159	1	159
LbrM22_V2.0050	*L. braziliensis*	243	228	470
LbrM22_V2.0060	*L. braziliensis*	283	86	368
LbrM22_V2.0060	*L. braziliensis*	64	391	454
LbrM22_V2.0060	*L. braziliensis*	85	474	558
LbrM22_V2.0070	*L. braziliensis*	70	220	289
LbrM22_V2.0080	*L. braziliensis*	53	1	53
LbrM22_V2.0080	*L. braziliensis*	54	195	248
LbrM22_V2.0080	*L. braziliensis*	54	195	248
LbrM22_V2.0090	*L. braziliensis*	48	154	201
LbrM22_V2.0120	*L. braziliensis*	42	1	42
LbrM22_V2.0120	*L. braziliensis*	170	44	213
LbrM22_V2.0120	*L. braziliensis*	170	44	213
LbrM22_V2.0130	*L. braziliensis*	184	295	478
LbrM22_V2.0140	*L. braziliensis*	221	79	299
LbrM22_V2.0160	*L. braziliensis*	43	99	141
LbrM22_V2.0160	*L. braziliensis*	135	207	341
LbrM22_V2.0170	*L. braziliensis*	358	1	358
LbrM22_V2.0180	*L. braziliensis*	74	11	84
LbrM22_V2.0190	*L. braziliensis*	97	1	97
LbrM22_V2.0200	*L. braziliensis*	121	1	121
LbrM22_V2.0200	*L. braziliensis*	78	203	280
LbrM22_V2.0200	*L. braziliensis*	43	379	421
LbrM22_V2.0200	*L. braziliensis*	130	925	1054
LbrM22_V2.0200	*L. braziliensis*	127	1399	1525
LbrM22_V2.0210	*L. braziliensis*	51	701	751
LbrM22_V2.0220	*L. braziliensis*	71	1	71
LbrM22_V2.0230	*L. braziliensis*	93	1	93
LbrM22_V2.0230	*L. braziliensis*	78	111	188
LbrM22_V2.0230	*L. braziliensis*	82	190	271
LbrM22_V2.0230	*L. braziliensis*	120	328	447
LbrM22_V2.0250	*L. braziliensis*	62	215	276
LbrM22_V2.0270	*L. braziliensis*	51	501	551
LbrM22_V2.0270	*L. braziliensis*	53	725	777
LbrM22_V2.0280	*L. braziliensis*	47	219	265
LbrM22_V2.0280	*L. braziliensis*	64	336	399
LbrM22_V2.0280	*L. braziliensis*	64	336	399

LbrM22_V2.0290	*L. braziliensis*	55	1	55
LbrM22_V2.0290	*L. braziliensis*	49	416	464
LbrM22_V2.0290	*L. braziliensis*	67	495	561
LbrM22_V2.0300	*L. braziliensis*	61	509	569
LbrM22_V2.0310	*L. braziliensis*	42	1	42
LbrM22_V2.0310	*L. braziliensis*	101	116	216
LbrM22_V2.0310	*L. braziliensis*	49	304	352
LbrM22_V2.0320	*L. braziliensis*	479	1	479
LbrM22_V2.0320	*L. braziliensis*	122	504	625
LbrM22_V2.0320	*L. braziliensis*	65	697	761
LbrM22_V2.0320	*L. braziliensis*	142	763	904
LbrM22_V2.0320	*L. braziliensis*	61	1004	1064
LbrM22_V2.0330	*L. braziliensis*	124	1	124
LbrM22_V2.0340	*L. braziliensis*	119	1	119
LbrM22_V2.0350	*L. braziliensis*	59	64	122
LbrM22_V2.0350	*L. braziliensis*	53	153	205
LbrM22_V2.0360	*L. braziliensis*	82	284	365
LbrM22_V2.0360	*L. braziliensis*	97	582	678
LbrM22_V2.0360	*L. braziliensis*	91	728	818
LbrM22_V2.0370	*L. braziliensis*	42	49	90
LbrM22_V2.0380	*L. braziliensis*	134	1	134
LbrM22_V2.0380	*L. braziliensis*	57	259	315
LbrM22_V2.0380	*L. braziliensis*	55	439	493
LbrM22_V2.0390	*L. braziliensis*	43	306	348
LbrM22_V2.0390	*L. braziliensis*	51	762	812
LbrM22_V2.0390	*L. braziliensis*	63	1108	1170
LbrM22_V2.0400	*L. braziliensis*	45	39	83
LbrM22_V2.0410	*L. braziliensis*	65	66	130
LbrM22_V2.0410	*L. braziliensis*	57	179	235
LbrM22_V2.0410	*L. braziliensis*	58	712	769
LbrM22_V2.0410	*L. braziliensis*	107	959	1065
LbrM22_V2.0420	*L. braziliensis*	82	183	264
LbrM22_V2.0430	*L. braziliensis*	59	260	318
LbrM22_V2.0440	*L. braziliensis*	46	1	46
LbrM22_V2.0440	*L. braziliensis*	43	100	142
LbrM22_V2.0440	*L. braziliensis*	62	252	313
LbrM22_V2.0440	*L. braziliensis*	101	457	557
LbrM22_V2.0450	*L. braziliensis*	263	16	278
LbrM22_V2.0450	*L. braziliensis*	56	419	474
LbrM22_V2.0460	*L. braziliensis*	40	47	86
LbrM22_V2.0460	*L. braziliensis*	56	165	220
LbrM22_V2.0460	*L. braziliensis*	87	261	347
LbrM22_V2.0460	*L. braziliensis*	114	407	520
LbrM22_V2.0460	*L. braziliensis*	56	601	656
LbrM22_V2.0470	*L. braziliensis*	40	50	89
LbrM22_V2.0470	*L. braziliensis*	78	127	204
LbrM22_V2.0470	*L. braziliensis*	55	228	282

LbrM22_V2.0470	*L. braziliensis*	47	324	370
LbrM22_V2.0470	*L. braziliensis*	76	489	564
LbrM22_V2.0470	*L. braziliensis*	112	587	698
LbrM22_V2.0480	*L. braziliensis*	41	1	41
LbrM22_V2.0480	*L. braziliensis*	120	168	287
LbrM22_V2.0480	*L. braziliensis*	120	168	287
LbrM22_V2.0490	*L. braziliensis*	50	94	143
LbrM22_V2.0490	*L. braziliensis*	151	236	386
LbrM22_V2.0490	*L. braziliensis*	132	702	833
LbrM22_V2.0490	*L. braziliensis*	50	852	901
LbrM22_V2.0500	*L. braziliensis*	42	155	196
LbrM22_V2.0500	*L. braziliensis*	93	249	341
LbrM22_V2.0500	*L. braziliensis*	221	500	720
LbrM22_V2.0510	*L. braziliensis*	52	307	358
LbrM22_V2.0530	*L. braziliensis*	74	94	167
LbrM22_V2.0530	*L. braziliensis*	53	309	361
LbrM22_V2.0540	*L. braziliensis*	44	86	129
LbrM22_V2.0540	*L. braziliensis*	58	233	290
LbrM22_V2.0540	*L. braziliensis*	66	369	434
LbrM22_V2.0550	*L. braziliensis*	107	1	107
LbrM22_V2.0550	*L. braziliensis*	56	205	260
LbrM22_V2.0560	*L. braziliensis*	90	1	90
LbrM22_V2.0570	*L. braziliensis*	165	107	271
LbrM22_V2.0570	*L. braziliensis*	47	593	639
LbrM22_V2.0570	*L. braziliensis*	48	659	706
LbrM22_V2.0570	*L. braziliensis*	50	924	973
LbrM22_V2.0570	*L. braziliensis*	71	1325	1395
LbrM22_V2.0570	*L. braziliensis*	93	1577	1669
LbrM22_V2.0570	*L. braziliensis*	66	1718	1783
LbrM22_V2.0580	*L. braziliensis*	65	1	65
LbrM22_V2.0590	*L. braziliensis*	382	1	382
LbrM22_V2.0590	*L. braziliensis*	252	405	656
LbrM22_V2.0590	*L. braziliensis*	80	697	776
LbrM22_V2.0590	*L. braziliensis*	48	785	832
LbrM22_V2.0590	*L. braziliensis*	57	913	969
LbrM22_V2.0590	*L. braziliensis*	64	976	1039
LbrM22_V2.0590	*L. braziliensis*	49	1086	1134
LbrM22_V2.0600	*L. braziliensis*	45	113	157
LbrM22_V2.0630	*L. braziliensis*	1121	1	1121
LbrM22_V2.0630	*L. braziliensis*	277	1160	1436
LbrM22_V2.0630	*L. braziliensis*	56	1462	1517
LbrM22_V2.0650	*L. braziliensis*	82	1	82
LbrM22_V2.0650	*L. braziliensis*	72	158	229
LbrM22_V2.0650	*L. braziliensis*	130	499	628
LbrM22_V2.0650	*L. braziliensis*	78	661	738
LbrM22_V2.0650	*L. braziliensis*	155	756	910
LbrM22_V2.0660	*L. braziliensis*	64	1	64

LbrM22_V2.0660	*L. braziliensis*	172	167	338
LbrM22_V2.0670	*L. braziliensis*	69	123	191
LbrM22_V2.0680	*L. braziliensis*	42	98	139
LbrM22_V2.0680	*L. braziliensis*	67	241	307
LbrM22_V2.0680	*L. braziliensis*	102	319	420
LbrM22_V2.0680	*L. braziliensis*	141	434	574
LbrM22_V2.0690	*L. braziliensis*	57	1	57
LbrM22_V2.0690	*L. braziliensis*	138	71	208
LbrM22_V2.0690	*L. braziliensis*	161	268	428
LbrM22_V2.0690	*L. braziliensis*	82	433	514
LbrM22_V2.0690	*L. braziliensis*	276	854	1129
LbrM22_V2.0710	*L. braziliensis*	60	230	289
LbrM22_V2.0740	*L. braziliensis*	78	1	78
LbrM22_V2.0750	*L. braziliensis*	87	340	426
LbrM22_V2.0770	*L. braziliensis*	65	68	132
LbrM22_V2.0770	*L. braziliensis*	94	158	251
LbrM22_V2.0770	*L. braziliensis*	64	275	338
LbrM22_V2.0770	*L. braziliensis*	73	394	466
LbrM22_V2.0770	*L. braziliensis*	101	543	643
LbrM22_V2.0780	*L. braziliensis*	90	1	90
LbrM22_V2.0790	*L. braziliensis*	71	1	71
LbrM22_V2.0790	*L. braziliensis*	189	77	265
LbrM22_V2.0790	*L. braziliensis*	128	597	724
LbrM22_V2.0790	*L. braziliensis*	55	757	811
LbrM22_V2.0800	*L. braziliensis*	171	1	171
LbrM22_V2.0800	*L. braziliensis*	50	199	248
LbrM22_V2.0820	*L. braziliensis*	44	25	68
LbrM22_V2.0820	*L. braziliensis*	73	107	179
LbrM22_V2.0820	*L. braziliensis*	42	231	272
LbrM22_V2.0820	*L. braziliensis*	95	368	462
LbrM22_V2.0820	*L. braziliensis*	45	705	749
LbrM22_V2.0840	*L. braziliensis*	80	426	505
LbrM22_V2.0840	*L. braziliensis*	76	596	671
LbrM22_V2.0840	*L. braziliensis*	76	596	671
LbrM22_V2.0860	*L. braziliensis*	60	43	102
LbrM22_V2.0860	*L. braziliensis*	69	219	287
LbrM22_V2.0860	*L. braziliensis*	68	563	630
LbrM22_V2.0860	*L. braziliensis*	202	665	866
LbrM22_V2.0860	*L. braziliensis*	55	908	962
LbrM22_V2.0860	*L. braziliensis*	82	1007	1088
LbrM22_V2.0860	*L. braziliensis*	232	1095	1326
LbrM22_V2.0860	*L. braziliensis*	83	1350	1432
LbrM22_V2.0870	*L. braziliensis*	69	55	123
LbrM22_V2.0870	*L. braziliensis*	134	154	287
LbrM22_V2.0870	*L. braziliensis*	87	319	405
LbrM22_V2.0870	*L. braziliensis*	251	504	754
LbrM22_V2.0870	*L. braziliensis*	94	775	868

LbrM22_V2.0870	*L. braziliensis*	98	909	1006
LbrM22_V2.0870	*L. braziliensis*	64	1069	1132
LbrM22_V2.0870	*L. braziliensis*	65	1155	1219
LbrM22_V2.0870	*L. braziliensis*	163	1294	1456
LbrM22_V2.0870	*L. braziliensis*	206	1460	1665
LbrM22_V2.0870	*L. braziliensis*	67	1701	1767
LbrM22_V2.0880	*L. braziliensis*	43	1	43
LbrM22_V2.0880	*L. braziliensis*	50	384	433
LbrM22_V2.0880	*L. braziliensis*	50	384	433
LbrM22_V2.0890	*L. braziliensis*	96	675	770
LbrM22_V2.0890	*L. braziliensis*	63	1155	1217
LbrM22_V2.0890	*L. braziliensis*	63	1155	1217
LbrM22_V2.0900	*L. braziliensis*	65	21	85
LbrM22_V2.0900	*L. braziliensis*	41	149	189
LbrM22_V2.0900	*L. braziliensis*	47	287	333
LbrM22_V2.0910	*L. braziliensis*	42	216	257
LbrM22_V2.0910	*L. braziliensis*	78	461	538
LbrM22_V2.0910	*L. braziliensis*	78	461	538
LbrM22_V2.0920	*L. braziliensis*	63	132	194
LbrM22_V2.0920	*L. braziliensis*	48	235	282
LbrM22_V2.0920	*L. braziliensis*	48	235	282
LbrM22_V2.0930	*L. braziliensis*	199	20	218
LbrM22_V2.0930	*L. braziliensis*	88	367	454
LbrM22_V2.0930	*L. braziliensis*	93	721	813
LbrM22_V2.0940	*L. braziliensis*	66	122	187
LbrM22_V2.0940	*L. braziliensis*	217	239	455
LbrM22_V2.0940	*L. braziliensis*	84	575	658
LbrM22_V2.0950	*L. braziliensis*	70	208	277
LbrM22_V2.0950	*L. braziliensis*	63	503	565
LbrM22_V2.0950	*L. braziliensis*	157	571	727
LbrM22_V2.0950	*L. braziliensis*	96	759	854
LbrM22_V2.0970	*L. braziliensis*	112	1	112
LbrM22_V2.0970	*L. braziliensis*	343	163	505
LbrM22_V2.0970	*L. braziliensis*	184	512	695
LbrM22_V2.0970	*L. braziliensis*	102	713	814
LbrM22_V2.0980	*L. braziliensis*	57	103	159
LbrM22_V2.0990	*L. braziliensis*	50	170	219
LbrM22_V2.0990	*L. braziliensis*	109	349	457
LbrM22_V2.0990	*L. braziliensis*	128	490	617
LbrM22_V2.1000	*L. braziliensis*	130	77	206
LbrM22_V2.1000	*L. braziliensis*	119	226	344
LbrM22_V2.1000	*L. braziliensis*	127	389	515
LbrM22_V2.1000	*L. braziliensis*	77	524	600
LbrM22_V2.1000	*L. braziliensis*	398	663	1060
LbrM22_V2.1000	*L. braziliensis*	291	1097	1387
LbrM22_V2.1000	*L. braziliensis*	107	1434	1540
LbrM22_V2.1000	*L. braziliensis*	156	1642	1797

LbrM22_V2.1000	*L. braziliensis*	62	1826	1887
LbrM22_V2.1000	*L. braziliensis*	112	1914	2025
LbrM22_V2.1000	*L. braziliensis*	134	2032	2165
LbrM22_V2.1000	*L. braziliensis*	64	2172	2235
LbrM22_V2.1000	*L. braziliensis*	111	2287	2397
LbrM22_V2.1010	*L. braziliensis*	82	1	82
LbrM22_V2.1010	*L. braziliensis*	88	736	823
LbrM22_V2.1010	*L. braziliensis*	43	855	897
LbrM22_V2.1010	*L. braziliensis*	70	1150	1219
LbrM22_V2.1010	*L. braziliensis*	64	2533	2596
LbrM22_V2.1010	*L. braziliensis*	63	2734	2796
LbrM22_V2.1010	*L. braziliensis*	50	3456	3505
LbrM22_V2.1010	*L. braziliensis*	107	3675	3781
LbrM22_V2.1010	*L. braziliensis*	51	4418	4468
LbrM22_V2.1020	*L. braziliensis*	57	72	128
LbrM22_V2.1050	*L. braziliensis*	47	1	47
LbrM22_V2.1050	*L. braziliensis*	44	174	217
LbrM22_V2.1050	*L. braziliensis*	255	749	1003
LbrM22_V2.1060	*L. braziliensis*	77	83	159
LbrM22_V2.1060	*L. braziliensis*	53	398	450
LbrM22_V2.1070	*L. braziliensis*	132	119	250
LbrM22_V2.1070	*L. braziliensis*	54	431	484
LbrM22_V2.1070	*L. braziliensis*	88	510	597
LbrM22_V2.1070	*L. braziliensis*	153	626	778
LbrM22_V2.1070	*L. braziliensis*	116	787	902
LbrM22_V2.1080	*L. braziliensis*	48	129	176
LbrM22_V2.1090	*L. braziliensis*	166	1	166
LbrM22_V2.1090	*L. braziliensis*	229	177	405
LbrM22_V2.1090	*L. braziliensis*	232	408	639
LbrM22_V2.1090	*L. braziliensis*	57	714	770
LbrM22_V2.1110	*L. braziliensis*	101	92	192
LbrM22_V2.1110	*L. braziliensis*	44	685	728
LbrM22_V2.1120	*L. braziliensis*	45	1	45
LbrM22_V2.1120	*L. braziliensis*	44	93	136
LbrM22_V2.1120	*L. braziliensis*	62	712	773
LbrM22_V2.1130	*L. braziliensis*	72	255	326
LbrM22_V2.1140	*L. braziliensis*	421	143	563
LbrM22_V2.1140	*L. braziliensis*	277	606	882
LbrM22_V2.1140	*L. braziliensis*	102	916	1017
LbrM22_V2.1150	*L. braziliensis*	78	45	122
LbrM22_V2.1150	*L. braziliensis*	175	208	382
LbrM22_V2.1160	*L. braziliensis*	46	121	166
LbrM22_V2.1160	*L. braziliensis*	70	187	256
LbrM22_V2.1160	*L. braziliensis*	70	187	256
LbrM22_V2.1170	*L. braziliensis*	53	1	53
LbrM22_V2.1180	*L. braziliensis*	70	49	118
LbrM22_V2.1190	*L. braziliensis*	147	1	147

LbrM22_V2.1190	*L. braziliensis*	197	170	366
LbrM22_V2.1190	*L. braziliensis*	56	375	430
LbrM22_V2.1190	*L. braziliensis*	70	467	536
LbrM22_V2.1190	*L. braziliensis*	109	601	709
LbrM22_V2.1190	*L. braziliensis*	104	728	831
LbrM22_V2.1190	*L. braziliensis*	114	913	1026
LbrM22_V2.1190	*L. braziliensis*	78	1035	1112
LbrM22_V2.1210	*L. braziliensis*	44	1	44
LbrM22_V2.1210	*L. braziliensis*	146	280	425
LbrM22_V2.1210	*L. braziliensis*	50	523	572
LbrM22_V2.1210	*L. braziliensis*	92	609	700
LbrM22_V2.1230	*L. braziliensis*	148	254	401
LbrM22_V2.1250	*L. braziliensis*	99	34	132
LbrM22_V2.1250	*L. braziliensis*	225	185	409
LbrM22_V2.1250	*L. braziliensis*	225	185	409
LbrM22_V2.1260	*L. braziliensis*	149	120	268
LbrM22_V2.1270	*L. braziliensis*	79	1	79
LbrM22_V2.1280	*L. braziliensis*	61	1	61
LbrM22_V2.1290	*L. braziliensis*	55	95	149
LbrM22_V2.1320	*L. braziliensis*	53	1	53
LbrM22_V2.1320	*L. braziliensis*	59	86	144
LbrM22_V2.1340	*L. braziliensis*	71	142	212
LbrM22_V2.1350	*L. braziliensis*	48	199	246
LbrM22_V2.1360	*L. braziliensis*	58	1	58
LbrM22_V2.1400	*L. braziliensis*	45	960	1004
LbrM22_V2.1400	*L. braziliensis*	46	1038	1083
LbrM22_V2.1400	*L. braziliensis*	46	1038	1083
LbrM22_V2.1420	*L. braziliensis*	40	72	111
LbrM22_V2.1420	*L. braziliensis*	49	127	175
LbrM22_V2.1420	*L. braziliensis*	49	127	175
LbrM22_V2.1440	*L. braziliensis*	44	919	962
LbrM22_V2.1460	*L. braziliensis*	40	72	111
LbrM22_V2.1460	*L. braziliensis*	49	127	175
LbrM22_V2.1460	*L. braziliensis*	49	127	175
LbrM22_V2.1470	*L. braziliensis*	129	54	182
LbrM22_V2.1480	*L. braziliensis*	141	22	162
LbrM22_V2.1480	*L. braziliensis*	87	185	271
LbrM22_V2.1480	*L. braziliensis*	94	282	375
LbrM22_V2.1490	*L. braziliensis*	40	211	250
LbrM22_V2.1490	*L. braziliensis*	67	297	363
LbrM22_V2.1490	*L. braziliensis*	67	297	363
LbrM22_V2.1510	*L. braziliensis*	213	1	213
LbrM22_V2.1510	*L. braziliensis*	68	238	305
LbrM22_V2.1510	*L. braziliensis*	51	560	610
LbrM22_V2.1510	*L. braziliensis*	68	614	681
LbrM22_V2.1520	*L. braziliensis*	75	456	530
LbrM22_V2.1530	*L. braziliensis*	56	1	56

LbrM22_V2.1530	*L. braziliensis*	40	492	531
LbrM22_V2.1530	*L. braziliensis*	42	975	1016
LbrM22_V2.1530	*L. braziliensis*	50	1088	1137
LbrM22_V2.1530	*L. braziliensis*	69	1423	1491
LbrM22_V2.1530	*L. braziliensis*	43	2372	2414
LbrM22_V2.1540	*L. braziliensis*	55	284	338
LbrM22_V2.1570	*L. braziliensis*	116	36	151
LbrM22_V2.1570	*L. braziliensis*	57	494	550
LbrM22_V2.1570	*L. braziliensis*	57	494	550
LbrM22_V2.1600	*L. braziliensis*	1384	1	1384
LbrM22_V2.1610	*L. braziliensis*	80	34	113
LbrM22_V2.1610	*L. braziliensis*	76	204	279
LbrM22_V2.1610	*L. braziliensis*	76	204	279
LbrM22_V2.1620	*L. braziliensis*	298	1	298
LbrM22_V2.1620	*L. braziliensis*	1692	316	2007
LbrM23_V2.0060	*L. braziliensis*	126	171	296
LbrM23_V2.0070	*L. braziliensis*	151	23	173
LbrM23_V2.0090	*L. braziliensis*	183	1	183
LbrM23_V2.0090	*L. braziliensis*	60	248	307
LbrM23_V2.0090	*L. braziliensis*	58	309	366
LbrM23_V2.0100	*L. braziliensis*	98	210	307
LbrM23_V2.0130	*L. braziliensis*	49	1588	1636
LbrM23_V2.0130	*L. braziliensis*	63	1705	1767
LbrM23_V2.0130	*L. braziliensis*	63	1705	1767
LbrM23_V2.0190	*L. braziliensis*	88	72	159
LbrM23_V2.0210	*L. braziliensis*	93	33	125
LbrM23_V2.0230	*L. braziliensis*	47	1	47
LbrM23_V2.0230	*L. braziliensis*	67	600	666
LbrM23_V2.0230	*L. braziliensis*	82	862	943
LbrM23_V2.0240	*L. braziliensis*	81	19	99
LbrM23_V2.0250	*L. braziliensis*	132	1	132
LbrM23_V2.0260	*L. braziliensis*	62	124	185
LbrM23_V2.0280	*L. braziliensis*	41	1	41
LbrM23_V2.0280	*L. braziliensis*	49	619	667
LbrM23_V2.0280	*L. braziliensis*	71	877	947
LbrM23_V2.0280	*L. braziliensis*	51	1258	1308
LbrM23_V2.0310	*L. braziliensis*	237	1	237
LbrM23_V2.0310	*L. braziliensis*	45	275	319
LbrM23_V2.0310	*L. braziliensis*	209	340	548
LbrM23_V2.0310	*L. braziliensis*	199	581	779
LbrM23_V2.0310	*L. braziliensis*	55	1102	1156
LbrM23_V2.0330	*L. braziliensis*	73	98	170
LbrM23_V2.0340	*L. braziliensis*	70	113	182
LbrM23_V2.0340	*L. braziliensis*	77	267	343
LbrM23_V2.0340	*L. braziliensis*	47	429	475
LbrM23_V2.0340	*L. braziliensis*	73	668	740
LbrM23_V2.0340	*L. braziliensis*	87	858	944

LbrM23_V2.0340	*L. braziliensis*	67	1101	1167
LbrM23_V2.0350	*L. braziliensis*	61	151	211
LbrM23_V2.0370	*L. braziliensis*	97	1	97
LbrM23_V2.0380	*L. braziliensis*	58	1	58
LbrM23_V2.0380	*L. braziliensis*	111	190	300
LbrM23_V2.0390	*L. braziliensis*	48	546	593
LbrM23_V2.0390	*L. braziliensis*	89	692	780
LbrM23_V2.0390	*L. braziliensis*	45	889	933
LbrM23_V2.0390	*L. braziliensis*	50	1637	1686
LbrM23_V2.0390	*L. braziliensis*	64	1702	1765
LbrM23_V2.0420	*L. braziliensis*	97	465	561
LbrM23_V2.0420	*L. braziliensis*	60	864	923
LbrM23_V2.0420	*L. braziliensis*	60	864	923
LbrM23_V2.0430	*L. braziliensis*	144	39	182
LbrM23_V2.0430	*L. braziliensis*	68	208	275
LbrM23_V2.0430	*L. braziliensis*	86	560	645
LbrM23_V2.0430	*L. braziliensis*	142	691	832
LbrM23_V2.0430	*L. braziliensis*	43	890	932
LbrM23_V2.0430	*L. braziliensis*	53	1000	1052
LbrM23_V2.0430	*L. braziliensis*	42	1057	1098
LbrM23_V2.0430	*L. braziliensis*	58	1170	1227
LbrM23_V2.0430	*L. braziliensis*	186	1270	1455
LbrM23_V2.0430	*L. braziliensis*	90	1696	1785
LbrM23_V2.0430	*L. braziliensis*	112	1827	1938
LbrM23_V2.0440	*L. braziliensis*	296	1	296
LbrM23_V2.0440	*L. braziliensis*	109	459	567
LbrM23_V2.0440	*L. braziliensis*	45	795	839
LbrM23_V2.0450	*L. braziliensis*	76	1	76
LbrM23_V2.0470	*L. braziliensis*	48	142	189
LbrM23_V2.0490	*L. braziliensis*	53	94	146
LbrM23_V2.0500	*L. braziliensis*	61	1	61
LbrM23_V2.0500	*L. braziliensis*	240	105	344
LbrM23_V2.0510	*L. braziliensis*	147	1	147
LbrM23_V2.0510	*L. braziliensis*	61	167	227
LbrM23_V2.0510	*L. braziliensis*	185	260	444
LbrM23_V2.0510	*L. braziliensis*	197	451	647
LbrM23_V2.0510	*L. braziliensis*	229	667	895
LbrM23_V2.0510	*L. braziliensis*	287	947	1233
LbrM23_V2.0510	*L. braziliensis*	101	1304	1404
LbrM23_V2.0520	*L. braziliensis*	47	93	139
LbrM23_V2.0550	*L. braziliensis*	105	66	170
LbrM23_V2.0570	*L. braziliensis*	53	233	285
LbrM23_V2.0580	*L. braziliensis*	59	1	59
LbrM23_V2.0590	*L. braziliensis*	97	80	176
LbrM23_V2.0600	*L. braziliensis*	87	263	349
LbrM23_V2.0600	*L. braziliensis*	52	586	637
LbrM23_V2.0600	*L. braziliensis*	92	759	850

LbrM23_V2.0600	*L. braziliensis*	120	946	1065
LbrM23_V2.0610	*L. braziliensis*	44	148	191
LbrM23_V2.0610	*L. braziliensis*	423	429	851
LbrM23_V2.0620	*L. braziliensis*	56	230	285
LbrM23_V2.0630	*L. braziliensis*	47	1	47
LbrM23_V2.0630	*L. braziliensis*	196	126	321
LbrM23_V2.0640	*L. braziliensis*	70	153	222
LbrM23_V2.0650	*L. braziliensis*	59	204	262
LbrM23_V2.0650	*L. braziliensis*	60	533	592
LbrM23_V2.0650	*L. braziliensis*	60	533	592
LbrM23_V2.0660	*L. braziliensis*	394	177	570
LbrM23_V2.0660	*L. braziliensis*	124	609	732
LbrM23_V2.0660	*L. braziliensis*	68	909	976
LbrM23_V2.0660	*L. braziliensis*	109	1060	1168
LbrM23_V2.0660	*L. braziliensis*	47	1635	1681
LbrM23_V2.0660	*L. braziliensis*	60	1689	1748
LbrM23_V2.0660	*L. braziliensis*	82	2193	2274
LbrM23_V2.0660	*L. braziliensis*	47	2301	2347
LbrM23_V2.0660	*L. braziliensis*	68	2353	2420
LbrM23_V2.0660	*L. braziliensis*	160	2438	2597
LbrM23_V2.0660	*L. braziliensis*	835	2606	3440
LbrM23_V2.0710	*L. braziliensis*	46	471	516
LbrM23_V2.0710	*L. braziliensis*	171	550	720
LbrM23_V2.0710	*L. braziliensis*	171	550	720
LbrM23_V2.0720	*L. braziliensis*	102	1	102
LbrM23_V2.0720	*L. braziliensis*	65	174	238
LbrM23_V2.0720	*L. braziliensis*	195	273	467
LbrM23_V2.0720	*L. braziliensis*	59	508	566
LbrM23_V2.0720	*L. braziliensis*	56	737	792
LbrM23_V2.0720	*L. braziliensis*	128	836	963
LbrM23_V2.0730	*L. braziliensis*	57	1	57
LbrM23_V2.0730	*L. braziliensis*	76	83	158
LbrM23_V2.0730	*L. braziliensis*	75	239	313
LbrM23_V2.0740	*L. braziliensis*	47	1	47
LbrM23_V2.0740	*L. braziliensis*	67	600	666
LbrM23_V2.0740	*L. braziliensis*	58	876	933
LbrM23_V2.0750	*L. braziliensis*	53	34	86
LbrM23_V2.0760	*L. braziliensis*	43	39	81
LbrM23_V2.0760	*L. braziliensis*	132	220	351
LbrM23_V2.0760	*L. braziliensis*	99	512	610
LbrM23_V2.0760	*L. braziliensis*	66	626	691
LbrM23_V2.0780	*L. braziliensis*	109	1	109
LbrM23_V2.0780	*L. braziliensis*	65	470	534
LbrM23_V2.0790	*L. braziliensis*	50	58	107
LbrM23_V2.0790	*L. braziliensis*	75	113	187
LbrM23_V2.0790	*L. braziliensis*	42	234	275
LbrM23_V2.0790	*L. braziliensis*	47	516	562

LbrM23_V2.0790	*L. braziliensis*	62	642	703
LbrM23_V2.0800	*L. braziliensis*	50	1	50
LbrM23_V2.0810	*L. braziliensis*	72	1	72
LbrM23_V2.0810	*L. braziliensis*	64	152	215
LbrM23_V2.0810	*L. braziliensis*	240	299	538
LbrM23_V2.0810	*L. braziliensis*	43	543	585
LbrM23_V2.0820	*L. braziliensis*	140	1	140
LbrM23_V2.0820	*L. braziliensis*	277	205	481
LbrM23_V2.0820	*L. braziliensis*	86	496	581
LbrM23_V2.0830	*L. braziliensis*	89	13	101
LbrM23_V2.0830	*L. braziliensis*	56	219	274
LbrM23_V2.0830	*L. braziliensis*	55	354	408
LbrM23_V2.0860	*L. braziliensis*	189	28	216
LbrM23_V2.0860	*L. braziliensis*	58	236	293
LbrM23_V2.0860	*L. braziliensis*	137	320	456
LbrM23_V2.0860	*L. braziliensis*	200	567	766
LbrM23_V2.0860	*L. braziliensis*	200	847	1046
LbrM23_V2.0860	*L. braziliensis*	49	1096	1144
LbrM23_V2.0860	*L. braziliensis*	53	1198	1250
LbrM23_V2.0870	*L. braziliensis*	45	1	45
LbrM23_V2.0870	*L. braziliensis*	154	96	249
LbrM23_V2.0870	*L. braziliensis*	146	527	672
LbrM23_V2.0870	*L. braziliensis*	73	682	754
LbrM23_V2.0890	*L. braziliensis*	81	1	81
LbrM23_V2.0890	*L. braziliensis*	67	146	212
LbrM23_V2.0910	*L. braziliensis*	128	57	184
LbrM23_V2.0920	*L. braziliensis*	139	559	697
LbrM23_V2.0920	*L. braziliensis*	86	1099	1184
LbrM23_V2.0930	*L. braziliensis*	57	42	98
LbrM23_V2.0930	*L. braziliensis*	55	362	416
LbrM23_V2.0930	*L. braziliensis*	55	362	416
LbrM23_V2.0950	*L. braziliensis*	163	1	163
LbrM23_V2.0950	*L. braziliensis*	107	202	308
LbrM23_V2.0950	*L. braziliensis*	143	314	456
LbrM23_V2.0950	*L. braziliensis*	42	500	541
LbrM23_V2.0950	*L. braziliensis*	91	583	673
LbrM23_V2.0950	*L. braziliensis*	210	710	919
LbrM23_V2.0960	*L. braziliensis*	71	90	160
LbrM23_V2.0960	*L. braziliensis*	64	258	321
LbrM23_V2.0960	*L. braziliensis*	115	468	582
LbrM23_V2.0960	*L. braziliensis*	100	686	785
LbrM23_V2.1010	*L. braziliensis*	63	1	63
LbrM23_V2.1010	*L. braziliensis*	162	129	290
LbrM23_V2.1010	*L. braziliensis*	45	361	405
LbrM23_V2.1010	*L. braziliensis*	111	408	518
LbrM23_V2.1010	*L. braziliensis*	43	558	600
LbrM23_V2.1010	*L. braziliensis*	202	649	850

LbrM23_V2.1010	*L. braziliensis*	132	852	983
LbrM23_V2.1010	*L. braziliensis*	160	996	1155
LbrM23_V2.1010	*L. braziliensis*	73	1184	1256
LbrM23_V2.1010	*L. braziliensis*	57	1266	1322
LbrM23_V2.1010	*L. braziliensis*	100	1348	1447
LbrM23_V2.1010	*L. braziliensis*	77	1470	1546
LbrM23_V2.1010	*L. braziliensis*	42	1570	1611
LbrM23_V2.1010	*L. braziliensis*	72	1687	1758
LbrM23_V2.1010	*L. braziliensis*	92	1943	2034
LbrM23_V2.1010	*L. braziliensis*	72	2067	2138
LbrM23_V2.1010	*L. braziliensis*	57	2221	2277
LbrM23_V2.1010	*L. braziliensis*	50	2380	2429
LbrM23_V2.1010	*L. braziliensis*	261	2489	2749
LbrM23_V2.1030	*L. braziliensis*	47	74	120
LbrM23_V2.1040	*L. braziliensis*	101	218	318
LbrM23_V2.1050	*L. braziliensis*	114	101	214
LbrM23_V2.1050	*L. braziliensis*	88	298	385
LbrM23_V2.1050	*L. braziliensis*	88	298	385
LbrM23_V2.1060	*L. braziliensis*	60	152	211
LbrM23_V2.1070	*L. braziliensis*	68	128	195
LbrM23_V2.1070	*L. braziliensis*	159	251	409
LbrM23_V2.1070	*L. braziliensis*	119	446	564
LbrM23_V2.1070	*L. braziliensis*	155	595	749
LbrM23_V2.1080	*L. braziliensis*	61	50	110
LbrM23_V2.1080	*L. braziliensis*	76	116	191
LbrM23_V2.1080	*L. braziliensis*	76	116	191
LbrM23_V2.1090	*L. braziliensis*	47	79	125
LbrM23_V2.1090	*L. braziliensis*	59	214	272
LbrM23_V2.1090	*L. braziliensis*	61	510	570
LbrM23_V2.1090	*L. braziliensis*	44	622	665
LbrM23_V2.1090	*L. braziliensis*	302	799	1100
LbrM23_V2.1100	*L. braziliensis*	100	1	100
LbrM23_V2.1120	*L. braziliensis*	169	1	169
LbrM23_V2.1130	*L. braziliensis*	81	180	260
LbrM23_V2.1130	*L. braziliensis*	118	325	442
LbrM23_V2.1130	*L. braziliensis*	101	457	557
LbrM23_V2.1140	*L. braziliensis*	118	20	137
LbrM23_V2.1140	*L. braziliensis*	48	170	217
LbrM23_V2.1140	*L. braziliensis*	126	234	359
LbrM23_V2.1140	*L. braziliensis*	190	378	567
LbrM23_V2.1140	*L. braziliensis*	52	641	692
LbrM23_V2.1160	*L. braziliensis*	48	4	51
LbrM23_V2.1160	*L. braziliensis*	73	81	153
LbrM23_V2.1160	*L. braziliensis*	54	298	351
LbrM23_V2.1170	*L. braziliensis*	62	243	304
LbrM23_V2.1180	*L. braziliensis*	100	1	100
LbrM23_V2.1180	*L. braziliensis*	61	140	200

LbrM23_V2.1180	*L. braziliensis*	40	223	262
LbrM23_V2.1200	*L. braziliensis*	65	494	558
LbrM23_V2.1210	*L. braziliensis*	125	184	308
LbrM23_V2.1210	*L. braziliensis*	59	356	414
LbrM23_V2.1220	*L. braziliensis*	156	22	177
LbrM23_V2.1220	*L. braziliensis*	362	222	583
LbrM23_V2.1220	*L. braziliensis*	83	773	855
LbrM23_V2.1220	*L. braziliensis*	447	904	1350
LbrM23_V2.1230	*L. braziliensis*	87	1	87
LbrM23_V2.1230	*L. braziliensis*	230	121	350
LbrM23_V2.1230	*L. braziliensis*	53	388	440
LbrM23_V2.1230	*L. braziliensis*	49	457	505
LbrM23_V2.1240	*L. braziliensis*	79	210	288
LbrM23_V2.1260	*L. braziliensis*	133	378	510
LbrM23_V2.1270	*L. braziliensis*	61	1	61
LbrM23_V2.1300	*L. braziliensis*	78	51	128
LbrM23_V2.1340	*L. braziliensis*	149	58	206
LbrM23_V2.1340	*L. braziliensis*	88	270	357
LbrM23_V2.1340	*L. braziliensis*	265	451	715
LbrM23_V2.1350	*L. braziliensis*	204	26	229
LbrM23_V2.1350	*L. braziliensis*	51	351	401
LbrM23_V2.1350	*L. braziliensis*	42	438	479
LbrM23_V2.1350	*L. braziliensis*	197	717	913
LbrM23_V2.1350	*L. braziliensis*	63	1070	1132
LbrM23_V2.1360	*L. braziliensis*	45	91	135
LbrM23_V2.1360	*L. braziliensis*	50	1024	1073
LbrM23_V2.1360	*L. braziliensis*	75	1121	1195
LbrM23_V2.1360	*L. braziliensis*	84	1222	1305
LbrM23_V2.1370	*L. braziliensis*	269	1	269
LbrM23_V2.1380	*L. braziliensis*	180	106	285
LbrM23_V2.1390	*L. braziliensis*	51	127	177
LbrM23_V2.1390	*L. braziliensis*	280	509	788
LbrM23_V2.1390	*L. braziliensis*	162	848	1009
LbrM23_V2.1400	*L. braziliensis*	128	1	128
LbrM23_V2.1400	*L. braziliensis*	74	136	209
LbrM23_V2.1400	*L. braziliensis*	45	330	374
LbrM23_V2.1400	*L. braziliensis*	51	431	481
LbrM23_V2.1400	*L. braziliensis*	154	483	636
LbrM23_V2.1400	*L. braziliensis*	43	1052	1094
LbrM23_V2.1410	*L. braziliensis*	135	1	135
LbrM23_V2.1410	*L. braziliensis*	194	151	344
LbrM23_V2.1410	*L. braziliensis*	250	348	597
LbrM23_V2.1410	*L. braziliensis*	49	599	647
LbrM23_V2.1430	*L. braziliensis*	99	25	123
LbrM23_V2.1430	*L. braziliensis*	61	257	317
LbrM23_V2.1430	*L. braziliensis*	107	3516	3622
LbrM23_V2.1430	*L. braziliensis*	58	3729	3786

LbrM23_V2.1430	*L. braziliensis*	76	4025	4100
LbrM23_V2.1450	*L. braziliensis*	57	1	57
LbrM23_V2.1450	*L. braziliensis*	56	228	283
LbrM23_V2.1450	*L. braziliensis*	110	367	476
LbrM23_V2.1450	*L. braziliensis*	41	482	522
LbrM23_V2.1450	*L. braziliensis*	292	562	853
LbrM23_V2.1450	*L. braziliensis*	47	927	973
LbrM23_V2.1450	*L. braziliensis*	57	1006	1062
LbrM23_V2.1450	*L. braziliensis*	218	1125	1342
LbrM23_V2.1450	*L. braziliensis*	127	1369	1495
LbrM23_V2.1450	*L. braziliensis*	84	1564	1647
LbrM23_V2.1450	*L. braziliensis*	140	1820	1959
LbrM23_V2.1450	*L. braziliensis*	42	1975	2016
LbrM23_V2.1450	*L. braziliensis*	150	2615	2764
LbrM23_V2.1460	*L. braziliensis*	67	55	121
LbrM23_V2.1460	*L. braziliensis*	40	240	279
LbrM23_V2.1460	*L. braziliensis*	40	240	279
LbrM23_V2.1480	*L. braziliensis*	74	1	74
LbrM23_V2.1480	*L. braziliensis*	42	115	156
LbrM23_V2.1480	*L. braziliensis*	156	259	414
LbrM23_V2.1500	*L. braziliensis*	321	1	321
LbrM23_V2.1500	*L. braziliensis*	535	336	870
LbrM23_V2.1500	*L. braziliensis*	334	879	1212
LbrM23_V2.1500	*L. braziliensis*	165	1782	1946
LbrM23_V2.1550	*L. braziliensis*	136	86	221
LbrM23_V2.1560	*L. braziliensis*	193	177	369
LbrM23_V2.1570	*L. braziliensis*	209	28	236
LbrM23_V2.1570	*L. braziliensis*	273	258	530
LbrM23_V2.1580	*L. braziliensis*	66	1	66
LbrM23_V2.1610	*L. braziliensis*	59	76	134
LbrM23_V2.1620	*L. braziliensis*	42	102	143
LbrM23_V2.1630	*L. braziliensis*	75	1	75
LbrM23_V2.1630	*L. braziliensis*	140	117	256
LbrM23_V2.1630	*L. braziliensis*	43	313	355
LbrM23_V2.1640	*L. braziliensis*	186	24	209
LbrM23_V2.1650	*L. braziliensis*	53	1	53
LbrM23_V2.1650	*L. braziliensis*	85	98	182
LbrM23_V2.1650	*L. braziliensis*	85	98	182
LbrM23_V2.1660	*L. braziliensis*	47	28	74
LbrM23_V2.1660	*L. braziliensis*	40	120	159
LbrM23_V2.1660	*L. braziliensis*	92	161	252
LbrM23_V2.1660	*L. braziliensis*	59	337	395
LbrM23_V2.1660	*L. braziliensis*	75	425	499
LbrM23_V2.1660	*L. braziliensis*	50	532	581
LbrM23_V2.1680	*L. braziliensis*	59	1	59
LbrM23_V2.1690	*L. braziliensis*	40	13	52
LbrM23_V2.1690	*L. braziliensis*	413	78	490

LbrM23_V2.1710	*L. braziliensis*	81	150	230
LbrM23_V2.1740	*L. braziliensis*	50	421	470
LbrM23_V2.1760	*L. braziliensis*	53	32	84
LbrM23_V2.1770	*L. braziliensis*	50	40	89
LbrM23_V2.1770	*L. braziliensis*	51	299	349
LbrM23_V2.1770	*L. braziliensis*	51	299	349
LbrM23_V2.1800	*L. braziliensis*	51	77	127
LbrM23_V2.1830	*L. braziliensis*	43	21	63
LbrM23_V2.1840	*L. braziliensis*	46	1	46
LbrM23_V2.1840	*L. braziliensis*	42	78	119
LbrM23_V2.1840	*L. braziliensis*	60	340	399
LbrM23_V2.1840	*L. braziliensis*	174	456	629
LbrM23_V2.1840	*L. braziliensis*	142	699	840
LbrM23_V2.1850	*L. braziliensis*	166	16	181
LbrM23_V2.1850	*L. braziliensis*	58	222	279
LbrM23_V2.1850	*L. braziliensis*	97	311	407
LbrM23_V2.1850	*L. braziliensis*	152	446	597
LbrM23_V2.1850	*L. braziliensis*	42	864	905
LbrM23_V2.1850	*L. braziliensis*	99	968	1066
LbrM23_V2.1850	*L. braziliensis*	87	1230	1316
LbrM23_V2.1850	*L. braziliensis*	187	1469	1655
LbrM23_V2.1850	*L. braziliensis*	46	1795	1840
LbrM23_V2.1850	*L. braziliensis*	75	1860	1934
LbrM23_V2.1850	*L. braziliensis*	48	1937	1984
LbrM23_V2.1860	*L. braziliensis*	108	1	108
LbrM23_V2.1860	*L. braziliensis*	50	189	238
LbrM23_V2.1860	*L. braziliensis*	41	546	586
LbrM23_V2.1860	*L. braziliensis*	207	591	797
LbrM23_V2.1860	*L. braziliensis*	289	915	1203
LbrM23_V2.1870	*L. braziliensis*	448	1	448
LbrM23_V2.1870	*L. braziliensis*	147	570	716
LbrM23_V2.1870	*L. braziliensis*	179	735	913
LbrM23_V2.1880	*L. braziliensis*	93	1	93
LbrM23_V2.1880	*L. braziliensis*	92	119	210
LbrM23_V2.1880	*L. braziliensis*	155	331	485
LbrM23_V2.1880	*L. braziliensis*	61	616	676
LbrM23_V2.1880	*L. braziliensis*	201	684	884
LbrM23_V2.1890	*L. braziliensis*	44	137	180
LbrM23_V2.1890	*L. braziliensis*	42	617	658
LbrM23_V2.1890	*L. braziliensis*	41	854	894
LbrM23_V2.1900	*L. braziliensis*	116	78	193
LbrM23_V2.1900	*L. braziliensis*	40	546	585
LbrM23_V2.1900	*L. braziliensis*	130	782	911
LbrM23_V2.1920	*L. braziliensis*	90	52	141
LbrM23_V2.1930	*L. braziliensis*	154	1	154
LbrM23_V2.1930	*L. braziliensis*	50	156	205
LbrM23_V2.1930	*L. braziliensis*	66	229	294

LbrM23_V2.1930	*L. braziliensis*	47	428	474
LbrM23_V2.1940	*L. braziliensis*	64	61	124
LbrM23_V2.1960	*L. braziliensis*	145	1	145
LbrM23_V2.1960	*L. braziliensis*	76	360	435
LbrM23_V2.1960	*L. braziliensis*	64	521	584
LbrM23_V2.1960	*L. braziliensis*	129	593	721
LbrM24_V2.0010	*L. braziliensis*	43	37	79
LbrM24_V2.0020	*L. braziliensis*	189	298	486
LbrM24_V2.0030	*L. braziliensis*	51	1	51
LbrM24_V2.0030	*L. braziliensis*	53	114	166
LbrM24_V2.0060	*L. braziliensis*	61	339	399
LbrM24_V2.0070	*L. braziliensis*	86	209	294
LbrM24_V2.0070	*L. braziliensis*	53	387	439
LbrM24_V2.0100	*L. braziliensis*	67	1	67
LbrM24_V2.0130	*L. braziliensis*	40	1	40
LbrM24_V2.0150	*L. braziliensis*	98	1	98
LbrM24_V2.0150	*L. braziliensis*	69	221	289
LbrM24_V2.0170	*L. braziliensis*	45	296	340
LbrM24_V2.0180	*L. braziliensis*	47	13	59
LbrM24_V2.0180	*L. braziliensis*	138	675	812
LbrM24_V2.0190	*L. braziliensis*	84	50	133
LbrM24_V2.0200	*L. braziliensis*	191	1	191
LbrM24_V2.0200	*L. braziliensis*	88	224	311
LbrM24_V2.0220	*L. braziliensis*	77	1	77
LbrM24_V2.0230	*L. braziliensis*	45	178	222
LbrM24_V2.0240	*L. braziliensis*	49	348	396
LbrM24_V2.0240	*L. braziliensis*	49	499	547
LbrM24_V2.0240	*L. braziliensis*	49	558	606
LbrM24_V2.0260	*L. braziliensis*	65	1	65
LbrM24_V2.0270	*L. braziliensis*	172	126	297
LbrM24_V2.0270	*L. braziliensis*	42	330	371
LbrM24_V2.0270	*L. braziliensis*	95	431	525
LbrM24_V2.0270	*L. braziliensis*	53	833	885
LbrM24_V2.0270	*L. braziliensis*	96	911	1006
LbrM24_V2.0270	*L. braziliensis*	167	1013	1179
LbrM24_V2.0280	*L. braziliensis*	70	101	170
LbrM24_V2.0280	*L. braziliensis*	47	190	236
LbrM24_V2.0280	*L. braziliensis*	47	190	236
LbrM24_V2.0290	*L. braziliensis*	52	1	52
LbrM24_V2.0290	*L. braziliensis*	78	62	139
LbrM24_V2.0290	*L. braziliensis*	78	62	139
LbrM24_V2.0300	*L. braziliensis*	41	485	525
LbrM24_V2.0300	*L. braziliensis*	96	676	771
LbrM24_V2.0310	*L. braziliensis*	147	26	172
LbrM24_V2.0320	*L. braziliensis*	40	354	393
LbrM24_V2.0320	*L. braziliensis*	48	408	455
LbrM24_V2.0320	*L. braziliensis*	48	408	455

LbrM24_V2.0330	*L. braziliensis*	59	83	141
LbrM24_V2.0330	*L. braziliensis*	55	245	299
LbrM24_V2.0330	*L. braziliensis*	89	309	397
LbrM24_V2.0330	*L. braziliensis*	94	574	667
LbrM24_V2.0330	*L. braziliensis*	127	830	956
LbrM24_V2.0340	*L. braziliensis*	41	868	908
LbrM24_V2.0350	*L. braziliensis*	70	220	289
LbrM24_V2.0380	*L. braziliensis*	226	35	260
LbrM24_V2.0380	*L. braziliensis*	127	347	473
LbrM24_V2.0380	*L. braziliensis*	246	479	724
LbrM24_V2.0390	*L. braziliensis*	57	200	256
LbrM24_V2.0390	*L. braziliensis*	42	291	332
LbrM24_V2.0390	*L. braziliensis*	66	447	512
LbrM24_V2.0410	*L. braziliensis*	210	97	306
LbrM24_V2.0430	*L. braziliensis*	153	1	153
LbrM24_V2.0430	*L. braziliensis*	46	744	789
LbrM24_V2.0430	*L. braziliensis*	41	1020	1060
LbrM24_V2.0450	*L. braziliensis*	43	135	177
LbrM24_V2.0460	*L. braziliensis*	92	126	217
LbrM24_V2.0460	*L. braziliensis*	113	324	436
LbrM24_V2.0460	*L. braziliensis*	52	465	516
LbrM24_V2.0460	*L. braziliensis*	103	521	623
LbrM24_V2.0480	*L. braziliensis*	41	132	172
LbrM24_V2.0480	*L. braziliensis*	70	688	757
LbrM24_V2.0480	*L. braziliensis*	42	1154	1195
LbrM24_V2.0490	*L. braziliensis*	73	43	115
LbrM24_V2.0490	*L. braziliensis*	87	175	261
LbrM24_V2.0490	*L. braziliensis*	65	602	666
LbrM24_V2.0490	*L. braziliensis*	225	694	918
LbrM24_V2.0490	*L. braziliensis*	153	934	1086
LbrM24_V2.0500	*L. braziliensis*	63	1	63
LbrM24_V2.0500	*L. braziliensis*	89	345	433
LbrM24_V2.0500	*L. braziliensis*	40	742	781
LbrM24_V2.0500	*L. braziliensis*	50	852	901
LbrM24_V2.0500	*L. braziliensis*	46	1476	1521
LbrM24_V2.0500	*L. braziliensis*	75	1700	1774
LbrM24_V2.0510	*L. braziliensis*	82	1	82
LbrM24_V2.0510	*L. braziliensis*	102	136	237
LbrM24_V2.0520	*L. braziliensis*	59	1	59
LbrM24_V2.0520	*L. braziliensis*	66	67	132
LbrM24_V2.0520	*L. braziliensis*	66	163	228
LbrM24_V2.0520	*L. braziliensis*	43	297	339
LbrM24_V2.0520	*L. braziliensis*	52	424	475
LbrM24_V2.0520	*L. braziliensis*	135	508	642
LbrM24_V2.0530	*L. braziliensis*	48	257	304
LbrM24_V2.0530	*L. braziliensis*	66	536	601
LbrM24_V2.0530	*L. braziliensis*	41	649	689

LbrM24_V2.0540	*L. braziliensis*	48	78	125
LbrM24_V2.0540	*L. braziliensis*	99	133	231
LbrM24_V2.0540	*L. braziliensis*	99	133	231
LbrM24_V2.0550	*L. braziliensis*	45	157	201
LbrM24_V2.0560	*L. braziliensis*	65	60	124
LbrM24_V2.0570	*L. braziliensis*	46	85	130
LbrM24_V2.0600	*L. braziliensis*	197	661	857
LbrM24_V2.0610	*L. braziliensis*	73	815	887
LbrM24_V2.0610	*L. braziliensis*	45	969	1013
LbrM24_V2.0610	*L. braziliensis*	83	1029	1111
LbrM24_V2.0610	*L. braziliensis*	51	1187	1237
LbrM24_V2.0610	*L. braziliensis*	40	1400	1439
LbrM24_V2.0610	*L. braziliensis*	70	1710	1779
LbrM24_V2.0620	*L. braziliensis*	377	615	991
LbrM24_V2.0620	*L. braziliensis*	104	993	1096
LbrM24_V2.0630	*L. braziliensis*	66	34	99
LbrM24_V2.0630	*L. braziliensis*	44	396	439
LbrM24_V2.0630	*L. braziliensis*	309	499	807
LbrM24_V2.0650	*L. braziliensis*	163	319	481
LbrM24_V2.0660	*L. braziliensis*	863	157	1019
LbrM24_V2.0670	*L. braziliensis*	79	1	79
LbrM24_V2.0680	*L. braziliensis*	98	1	98
LbrM24_V2.0680	*L. braziliensis*	261	422	682
LbrM24_V2.0680	*L. braziliensis*	95	730	824
LbrM24_V2.0680	*L. braziliensis*	189	867	1055
LbrM24_V2.0680	*L. braziliensis*	178	1063	1240
LbrM24_V2.0690	*L. braziliensis*	99	447	545
LbrM24_V2.0700	*L. braziliensis*	41	112	152
LbrM24_V2.0720	*L. braziliensis*	60	171	230
LbrM24_V2.0720	*L. braziliensis*	44	289	332
LbrM24_V2.0740	*L. braziliensis*	89	478	566
LbrM24_V2.0750	*L. braziliensis*	231	1	231
LbrM24_V2.0750	*L. braziliensis*	291	280	570
LbrM24_V2.0750	*L. braziliensis*	68	785	852
LbrM24_V2.0750	*L. braziliensis*	102	956	1057
LbrM24_V2.0770	*L. braziliensis*	212	1	212
LbrM24_V2.0770	*L. braziliensis*	49	312	360
LbrM24_V2.0770	*L. braziliensis*	49	743	791
LbrM24_V2.0770	*L. braziliensis*	53	973	1025
LbrM24_V2.0770	*L. braziliensis*	70	1057	1126
LbrM24_V2.0800	*L. braziliensis*	99	1	99
LbrM24_V2.0800	*L. braziliensis*	154	110	263
LbrM24_V2.0810	*L. braziliensis*	207	1	207
LbrM24_V2.0810	*L. braziliensis*	87	230	316
LbrM24_V2.0820	*L. braziliensis*	84	19	102
LbrM24_V2.0820	*L. braziliensis*	115	144	258
LbrM24_V2.0820	*L. braziliensis*	355	262	616

LbrM24_V2.0830	*L. braziliensis*	99	284	382
LbrM24_V2.0840	*L. braziliensis*	55	53	107
LbrM24_V2.0840	*L. braziliensis*	92	207	298
LbrM24_V2.0840	*L. braziliensis*	51	302	352
LbrM24_V2.0840	*L. braziliensis*	79	815	893
LbrM24_V2.0840	*L. braziliensis*	58	975	1032
LbrM24_V2.0840	*L. braziliensis*	103	1081	1183
LbrM24_V2.0840	*L. braziliensis*	47	1506	1552
LbrM24_V2.0840	*L. braziliensis*	104	1728	1831
LbrM24_V2.0840	*L. braziliensis*	137	2253	2389
LbrM24_V2.0840	*L. braziliensis*	104	2604	2707
LbrM24_V2.0860	*L. braziliensis*	46	1	46
LbrM24_V2.0860	*L. braziliensis*	61	319	379
LbrM24_V2.0860	*L. braziliensis*	105	707	811
LbrM24_V2.0900	*L. braziliensis*	97	181	277
LbrM24_V2.0900	*L. braziliensis*	60	283	342
LbrM24_V2.0900	*L. braziliensis*	43	627	669
LbrM24_V2.0900	*L. braziliensis*	57	798	854
LbrM24_V2.0910	*L. braziliensis*	690	1	690
LbrM24_V2.0920	*L. braziliensis*	57	181	237
LbrM24_V2.0920	*L. braziliensis*	50	561	610
LbrM24_V2.0930	*L. braziliensis*	102	1	102
LbrM24_V2.0930	*L. braziliensis*	40	236	275
LbrM24_V2.0940	*L. braziliensis*	42	104	145
LbrM24_V2.0940	*L. braziliensis*	87	518	604
LbrM24_V2.0940	*L. braziliensis*	41	660	700
LbrM24_V2.0940	*L. braziliensis*	66	841	906
LbrM24_V2.0960	*L. braziliensis*	77	160	236
LbrM24_V2.0970	*L. braziliensis*	121	16	136
LbrM24_V2.0970	*L. braziliensis*	813	200	1012
LbrM24_V2.0980	*L. braziliensis*	82	175	256
LbrM24_V2.0990	*L. braziliensis*	143	56	198
LbrM24_V2.0990	*L. braziliensis*	145	212	356
LbrM24_V2.0990	*L. braziliensis*	885	359	1243
LbrM24_V2.0990	*L. braziliensis*	183	1354	1536
LbrM24_V2.1000	*L. braziliensis*	173	492	664
LbrM24_V2.1010	*L. braziliensis*	86	76	161
LbrM24_V2.1010	*L. braziliensis*	165	165	329
LbrM24_V2.1010	*L. braziliensis*	88	397	484
LbrM24_V2.1020	*L. braziliensis*	55	84	138
LbrM24_V2.1020	*L. braziliensis*	76	164	239
LbrM24_V2.1020	*L. braziliensis*	141	289	429
LbrM24_V2.1030	*L. braziliensis*	103	1	103
LbrM24_V2.1030	*L. braziliensis*	65	198	262
LbrM24_V2.1030	*L. braziliensis*	80	416	495
LbrM24_V2.1030	*L. braziliensis*	42	535	576
LbrM24_V2.1030	*L. braziliensis*	201	662	862

LbrM24_V2.1030	*L. braziliensis*	117	943	1059
LbrM24_V2.1050	*L. braziliensis*	51	1	51
LbrM24_V2.1050	*L. braziliensis*	46	81	126
LbrM24_V2.1050	*L. braziliensis*	123	156	278
LbrM24_V2.1050	*L. braziliensis*	157	347	503
LbrM24_V2.1050	*L. braziliensis*	87	649	735
LbrM24_V2.1050	*L. braziliensis*	118	800	917
LbrM24_V2.1060	*L. braziliensis*	276	28	303
LbrM24_V2.1060	*L. braziliensis*	42	413	454
LbrM24_V2.1060	*L. braziliensis*	55	527	581
LbrM24_V2.1060	*L. braziliensis*	140	760	899
LbrM24_V2.1060	*L. braziliensis*	60	1184	1243
LbrM24_V2.1060	*L. braziliensis*	148	1251	1398
LbrM24_V2.1070	*L. braziliensis*	151	80	230
LbrM24_V2.1080	*L. braziliensis*	54	133	186
LbrM24_V2.1080	*L. braziliensis*	62	233	294
LbrM24_V2.1080	*L. braziliensis*	184	496	679
LbrM24_V2.1080	*L. braziliensis*	51	716	766
LbrM24_V2.1080	*L. braziliensis*	44	790	833
LbrM24_V2.1080	*L. braziliensis*	45	1290	1334
LbrM24_V2.1080	*L. braziliensis*	213	1351	1563
LbrM24_V2.1080	*L. braziliensis*	133	1874	2006
LbrM24_V2.1080	*L. braziliensis*	65	2068	2132
LbrM24_V2.1080	*L. braziliensis*	61	2222	2282
LbrM24_V2.1080	*L. braziliensis*	57	2303	2359
LbrM24_V2.1090	*L. braziliensis*	77	298	374
LbrM24_V2.1100	*L. braziliensis*	128	249	376
LbrM24_V2.1100	*L. braziliensis*	42	539	580
LbrM24_V2.1110	*L. braziliensis*	49	74	122
LbrM24_V2.1110	*L. braziliensis*	43	193	235
LbrM24_V2.1110	*L. braziliensis*	141	239	379
LbrM24_V2.1110	*L. braziliensis*	46	696	741
LbrM24_V2.1110	*L. braziliensis*	82	1007	1088
LbrM24_V2.1120	*L. braziliensis*	53	305	357
LbrM24_V2.1120	*L. braziliensis*	296	420	715
LbrM24_V2.1120	*L. braziliensis*	66	940	1005
LbrM24_V2.1120	*L. braziliensis*	60	1116	1175
LbrM24_V2.1130	*L. braziliensis*	160	284	443
LbrM24_V2.1130	*L. braziliensis*	132	523	654
LbrM24_V2.1130	*L. braziliensis*	133	815	947
LbrM24_V2.1140	*L. braziliensis*	266	1	266
LbrM24_V2.1140	*L. braziliensis*	176	293	468
LbrM24_V2.1150	*L. braziliensis*	145	64	208
LbrM24_V2.1150	*L. braziliensis*	90	211	300
LbrM24_V2.1150	*L. braziliensis*	59	309	367
LbrM24_V2.1150	*L. braziliensis*	232	403	634
LbrM24_V2.1150	*L. braziliensis*	253	652	904

LbrM24_V2.1150	*L. braziliensis*	66	942	1007
LbrM24_V2.1160	*L. braziliensis*	115	1	115
LbrM24_V2.1160	*L. braziliensis*	40	246	285
LbrM24_V2.1170	*L. braziliensis*	130	1	130
LbrM24_V2.1180	*L. braziliensis*	56	17	72
LbrM24_V2.1180	*L. braziliensis*	58	152	209
LbrM24_V2.1180	*L. braziliensis*	85	232	316
LbrM24_V2.1180	*L. braziliensis*	106	318	423
LbrM24_V2.1180	*L. braziliensis*	332	437	768
LbrM24_V2.1190	*L. braziliensis*	44	914	957
LbrM24_V2.1190	*L. braziliensis*	86	978	1063
LbrM24_V2.1190	*L. braziliensis*	65	1112	1176
LbrM24_V2.1200	*L. braziliensis*	69	1	69
LbrM24_V2.1200	*L. braziliensis*	175	86	260
LbrM24_V2.1210	*L. braziliensis*	55	1	55
LbrM24_V2.1220	*L. braziliensis*	277	1	277
LbrM24_V2.1240	*L. braziliensis*	111	24	134
LbrM24_V2.1240	*L. braziliensis*	99	177	275
LbrM24_V2.1240	*L. braziliensis*	57	380	436
LbrM24_V2.1240	*L. braziliensis*	74	469	542
LbrM24_V2.1250	*L. braziliensis*	155	1	155
LbrM24_V2.1250	*L. braziliensis*	164	220	383
LbrM24_V2.1250	*L. braziliensis*	53	617	669
LbrM24_V2.1280	*L. braziliensis*	52	18	69
LbrM24_V2.1300	*L. braziliensis*	42	323	364
LbrM24_V2.1300	*L. braziliensis*	67	429	495
LbrM24_V2.1300	*L. braziliensis*	67	429	495
LbrM24_V2.1310	*L. braziliensis*	75	83	157
LbrM24_V2.1310	*L. braziliensis*	131	190	320
LbrM24_V2.1310	*L. braziliensis*	57	328	384
LbrM24_V2.1310	*L. braziliensis*	56	488	543
LbrM24_V2.1310	*L. braziliensis*	74	726	799
LbrM24_V2.1330	*L. braziliensis*	106	1	106
LbrM24_V2.1330	*L. braziliensis*	313	137	449
LbrM24_V2.1330	*L. braziliensis*	177	1153	1329
LbrM24_V2.1330	*L. braziliensis*	161	1438	1598
LbrM24_V2.1330	*L. braziliensis*	275	1609	1883
LbrM24_V2.1330	*L. braziliensis*	130	1902	2031
LbrM24_V2.1340	*L. braziliensis*	73	68	140
LbrM24_V2.1340	*L. braziliensis*	138	143	280
LbrM24_V2.1340	*L. braziliensis*	102	311	412
LbrM24_V2.1340	*L. braziliensis*	64	414	477
LbrM24_V2.1340	*L. braziliensis*	183	564	746
LbrM24_V2.1340	*L. braziliensis*	54	890	943
LbrM24_V2.1360	*L. braziliensis*	86	24	109
LbrM24_V2.1370	*L. braziliensis*	100	34	133
LbrM24_V2.1370	*L. braziliensis*	92	262	353

LbrM24_V2.1370	*L. braziliensis*	83	551	633
LbrM24_V2.1380	*L. braziliensis*	150	112	261
LbrM24_V2.1380	*L. braziliensis*	56	304	359
LbrM24_V2.1380	*L. braziliensis*	72	419	490
LbrM24_V2.1380	*L. braziliensis*	100	500	599
LbrM24_V2.1380	*L. braziliensis*	70	756	825
LbrM24_V2.1380	*L. braziliensis*	88	1303	1390
LbrM24_V2.1380	*L. braziliensis*	64	1401	1464
LbrM24_V2.1380	*L. braziliensis*	55	1526	1580
LbrM24_V2.1380	*L. braziliensis*	62	1611	1672
LbrM24_V2.1380	*L. braziliensis*	93	1836	1928
LbrM24_V2.1380	*L. braziliensis*	61	1968	2028
LbrM24_V2.1380	*L. braziliensis*	194	2134	2327
LbrM24_V2.1380	*L. braziliensis*	230	2376	2605
LbrM24_V2.1380	*L. braziliensis*	251	2615	2865
LbrM24_V2.1390	*L. braziliensis*	189	1	189
LbrM24_V2.1390	*L. braziliensis*	53	392	444
LbrM24_V2.1390	*L. braziliensis*	176	506	681
LbrM24_V2.1390	*L. braziliensis*	43	764	806
LbrM24_V2.1410	*L. braziliensis*	254	1	254
LbrM24_V2.1420	*L. braziliensis*	57	25	81
LbrM24_V2.1420	*L. braziliensis*	74	459	532
LbrM24_V2.1440	*L. braziliensis*	661	1	661
LbrM24_V2.1440	*L. braziliensis*	175	741	915
LbrM24_V2.1450	*L. braziliensis*	186	1	186
LbrM24_V2.1450	*L. braziliensis*	418	194	611
LbrM24_V2.1450	*L. braziliensis*	91	669	759
LbrM24_V2.1450	*L. braziliensis*	305	777	1081
LbrM24_V2.1450	*L. braziliensis*	170	1127	1296
LbrM24_V2.1450	*L. braziliensis*	41	1302	1342
LbrM24_V2.1450	*L. braziliensis*	49	1666	1714
LbrM24_V2.1450	*L. braziliensis*	177	1732	1908
LbrM24_V2.1450	*L. braziliensis*	235	1992	2226
LbrM24_V2.1450	*L. braziliensis*	80	2255	2334
LbrM24_V2.1450	*L. braziliensis*	47	2378	2424
LbrM24_V2.1450	*L. braziliensis*	150	2448	2597
LbrM24_V2.1450	*L. braziliensis*	76	2651	2726
LbrM24_V2.1450	*L. braziliensis*	118	2739	2856
LbrM24_V2.1450	*L. braziliensis*	56	2882	2937
LbrM24_V2.1450	*L. braziliensis*	53	2971	3023
LbrM24_V2.1450	*L. braziliensis*	51	3090	3140
LbrM24_V2.1450	*L. braziliensis*	59	3191	3249
LbrM24_V2.1460	*L. braziliensis*	57	215	271
LbrM24_V2.1460	*L. braziliensis*	81	297	377
LbrM24_V2.1460	*L. braziliensis*	80	495	574
LbrM24_V2.1460	*L. braziliensis*	175	580	754
LbrM24_V2.1460	*L. braziliensis*	167	760	926

LbrM24_V2.1460	*L. braziliensis*	77	1193	1269
LbrM24_V2.1480	*L. braziliensis*	148	1	148
LbrM24_V2.1490	*L. braziliensis*	157	73	229
LbrM24_V2.1500	*L. braziliensis*	157	73	229
LbrM24_V2.1510	*L. braziliensis*	52	382	433
LbrM24_V2.1520	*L. braziliensis*	42	1	42
LbrM24_V2.1520	*L. braziliensis*	102	367	468
LbrM24_V2.1530	*L. braziliensis*	82	235	316
LbrM24_V2.1540	*L. braziliensis*	59	440	498
LbrM24_V2.1540	*L. braziliensis*	52	623	674
LbrM24_V2.1540	*L. braziliensis*	60	833	892
LbrM24_V2.1620	*L. braziliensis*	67	134	200
LbrM24_V2.1620	*L. braziliensis*	81	1105	1185
LbrM24_V2.1620	*L. braziliensis*	53	1329	1381
LbrM24_V2.1630	*L. braziliensis*	81	76	156
LbrM24_V2.1640	*L. braziliensis*	60	303	362
LbrM24_V2.1650	*L. braziliensis*	64	266	329
LbrM24_V2.1650	*L. braziliensis*	191	334	524
LbrM24_V2.1700	*L. braziliensis*	67	326	392
LbrM24_V2.1710	*L. braziliensis*	100	1	100
LbrM24_V2.1710	*L. braziliensis*	74	165	238
LbrM24_V2.1710	*L. braziliensis*	140	261	400
LbrM24_V2.1710	*L. braziliensis*	65	437	501
LbrM24_V2.1720	*L. braziliensis*	101	1	101
LbrM24_V2.1720	*L. braziliensis*	46	226	271
LbrM24_V2.1730	*L. braziliensis*	102	173	274
LbrM24_V2.1740	*L. braziliensis*	45	213	257
LbrM24_V2.1760	*L. braziliensis*	88	149	236
LbrM24_V2.1770	*L. braziliensis*	42	1	42
LbrM24_V2.1780	*L. braziliensis*	251	1	251
LbrM24_V2.1780	*L. braziliensis*	80	278	357
LbrM24_V2.1780	*L. braziliensis*	43	536	578
LbrM24_V2.1780	*L. braziliensis*	82	756	837
LbrM24_V2.1790	*L. braziliensis*	265	1	265
LbrM24_V2.1790	*L. braziliensis*	52	287	338
LbrM24_V2.1790	*L. braziliensis*	78	495	572
LbrM24_V2.1790	*L. braziliensis*	71	900	970
LbrM24_V2.1790	*L. braziliensis*	59	1104	1162
LbrM24_V2.1800	*L. braziliensis*	98	114	211
LbrM24_V2.1810	*L. braziliensis*	52	1	52
LbrM24_V2.1810	*L. braziliensis*	93	60	152
LbrM24_V2.1810	*L. braziliensis*	93	60	152
LbrM24_V2.1820	*L. braziliensis*	58	60	117
LbrM24_V2.1830	*L. braziliensis*	58	60	117
LbrM24_V2.1850	*L. braziliensis*	44	469	512
LbrM24_V2.1850	*L. braziliensis*	52	588	639
LbrM24_V2.1850	*L. braziliensis*	50	1095	1144

LbrM24_V2.1850	*L. braziliensis*	78	1180	1257
LbrM24_V2.1860	*L. braziliensis*	126	1	126
LbrM24_V2.1880	*L. braziliensis*	43	1	43
LbrM24_V2.1880	*L. braziliensis*	59	323	381
LbrM24_V2.1890	*L. braziliensis*	40	97	136
LbrM24_V2.1900	*L. braziliensis*	136	1	136
LbrM24_V2.1920	*L. braziliensis*	171	86	256
LbrM24_V2.1930	*L. braziliensis*	188	1	188
LbrM24_V2.1930	*L. braziliensis*	297	202	498
LbrM24_V2.1940	*L. braziliensis*	80	40	119
LbrM24_V2.1940	*L. braziliensis*	86	334	419
LbrM24_V2.1940	*L. braziliensis*	68	462	529
LbrM24_V2.1940	*L. braziliensis*	84	656	739
LbrM24_V2.1940	*L. braziliensis*	104	798	901
LbrM24_V2.1940	*L. braziliensis*	79	1020	1098
LbrM24_V2.1940	*L. braziliensis*	99	1137	1235
LbrM24_V2.1950	*L. braziliensis*	104	88	191
LbrM24_V2.1950	*L. braziliensis*	199	738	936
LbrM24_V2.1960	*L. braziliensis*	173	1	173
LbrM24_V2.1960	*L. braziliensis*	41	206	246
LbrM24_V2.1960	*L. braziliensis*	151	373	523
LbrM24_V2.1960	*L. braziliensis*	149	560	708
LbrM24_V2.1970	*L. braziliensis*	44	493	536
LbrM24_V2.1980	*L. braziliensis*	41	326	366
LbrM24_V2.2000	*L. braziliensis*	114	1	114
LbrM24_V2.2000	*L. braziliensis*	70	228	297
LbrM24_V2.2000	*L. braziliensis*	107	371	477
LbrM24_V2.2000	*L. braziliensis*	47	481	527
LbrM24_V2.2010	*L. braziliensis*	119	1	119
LbrM24_V2.2010	*L. braziliensis*	48	261	308
LbrM24_V2.2010	*L. braziliensis*	49	433	481
LbrM24_V2.2010	*L. braziliensis*	57	549	605
LbrM24_V2.2020	*L. braziliensis*	115	37	151
LbrM24_V2.2030	*L. braziliensis*	85	607	691
LbrM24_V2.2040	*L. braziliensis*	160	18	177
LbrM24_V2.2040	*L. braziliensis*	126	237	362
LbrM24_V2.2040	*L. braziliensis*	129	407	535
LbrM24_V2.2040	*L. braziliensis*	282	538	819
LbrM24_V2.2050	*L. braziliensis*	54	32	85
LbrM24_V2.2050	*L. braziliensis*	161	191	351
LbrM24_V2.2060	*L. braziliensis*	185	47	231
LbrM24_V2.2070	*L. braziliensis*	120	24	143
LbrM24_V2.2070	*L. braziliensis*	78	178	255
LbrM24_V2.2070	*L. braziliensis*	77	272	348
LbrM24_V2.2080	*L. braziliensis*	41	194	234
LbrM24_V2.2090	*L. braziliensis*	170	543	712
LbrM24_V2.2090	*L. braziliensis*	62	763	824

LbrM24_V2.2100	*L. braziliensis*	69	1	69
LbrM24_V2.2100	*L. braziliensis*	42	149	190
LbrM24_V2.2100	*L. braziliensis*	57	1388	1444
LbrM24_V2.2130	*L. braziliensis*	40	1	40
LbrM24_V2.2130	*L. braziliensis*	40	104	143
LbrM24_V2.2130	*L. braziliensis*	40	104	143
LbrM24_V2.2140	*L. braziliensis*	66	246	311
LbrM24_V2.2150	*L. braziliensis*	46	1	46
LbrM24_V2.2150	*L. braziliensis*	81	122	202
LbrM24_V2.2150	*L. braziliensis*	81	122	202
LbrM24_V2.2160	*L. braziliensis*	46	1	46
LbrM24_V2.2160	*L. braziliensis*	81	122	202
LbrM24_V2.2160	*L. braziliensis*	81	122	202
LbrM24_V2.2180	*L. braziliensis*	108	196	303
LbrM24_V2.2200	*L. braziliensis*	46	19	64
LbrM24_V2.2200	*L. braziliensis*	245	79	323
LbrM24_V2.2200	*L. braziliensis*	42	403	444
LbrM24_V2.2220	*L. braziliensis*	98	1	98
LbrM24_V2.2230	*L. braziliensis*	83	1	83
LbrM24_V2.2230	*L. braziliensis*	56	89	144
LbrM24_V2.2250	*L. braziliensis*	53	65	117
LbrM24_V2.2250	*L. braziliensis*	50	593	642
LbrM24_V2.2250	*L. braziliensis*	50	593	642
LbrM24_V2.2260	*L. braziliensis*	621	21	641
LbrM24_V2.2260	*L. braziliensis*	351	661	1011
LbrM24_V2.2260	*L. braziliensis*	228	1049	1276
LbrM24_V2.2260	*L. braziliensis*	161	1278	1438
LbrM24_V2.2270	*L. braziliensis*	56	1	56
LbrM24_V2.2280	*L. braziliensis*	40	206	245
LbrM24_V2.2290	*L. braziliensis*	41	84	124
LbrM24_V2.2320	*L. braziliensis*	77	122	198
LbrM24_V2.2320	*L. braziliensis*	40	209	248
LbrM24_V2.2340	*L. braziliensis*	69	1	69
LbrM24_V2.2360	*L. braziliensis*	76	738	813
LbrM24_V2.2380	*L. braziliensis*	57	78	134
LbrM24_V2.2390	*L. braziliensis*	59	105	163
LbrM24_V2.2400	*L. braziliensis*	44	29	72
LbrM24_V2.2410	*L. braziliensis*	41	63	103
LbrM24_V2.2410	*L. braziliensis*	83	176	258
LbrM24_V2.2420	*L. braziliensis*	40	184	223
LbrM24_V2.2430	*L. braziliensis*	178	199	376
LbrM24_V2.2430	*L. braziliensis*	111	556	666
LbrM24_V2.2430	*L. braziliensis*	92	855	946
LbrM24_V2.2430	*L. braziliensis*	56	1460	1515
LbrM24_V2.2440	*L. braziliensis*	146	351	496
LbrM24_V2.2450	*L. braziliensis*	154	1	154
LbrM24_V2.2450	*L. braziliensis*	67	162	228

LbrM24_V2.2450	*L. braziliensis*	293	389	681
LbrM25_V2.0010	*L. braziliensis*	42	191	232
LbrM25_V2.0030	*L. braziliensis*	100	366	465
LbrM25_V2.0040	*L. braziliensis*	69	1	69
LbrM25_V2.0040	*L. braziliensis*	46	166	211
LbrM25_V2.0040	*L. braziliensis*	72	213	284
LbrM25_V2.0040	*L. braziliensis*	124	376	499
LbrM25_V2.0040	*L. braziliensis*	142	556	697
LbrM25_V2.0040	*L. braziliensis*	48	994	1041
LbrM25_V2.0040	*L. braziliensis*	41	1189	1229
LbrM25_V2.0050	*L. braziliensis*	57	115	171
LbrM25_V2.0070	*L. braziliensis*	57	1	57
LbrM25_V2.0080	*L. braziliensis*	147	345	491
LbrM25_V2.0090	*L. braziliensis*	66	1	66
LbrM25_V2.0110	*L. braziliensis*	48	253	300
LbrM25_V2.0150	*L. braziliensis*	41	151	191
LbrM25_V2.0150	*L. braziliensis*	59	332	390
LbrM25_V2.0150	*L. braziliensis*	59	332	390
LbrM25_V2.0160	*L. braziliensis*	70	14	83
LbrM25_V2.0160	*L. braziliensis*	44	129	172
LbrM25_V2.0160	*L. braziliensis*	44	129	172
LbrM25_V2.0170	*L. braziliensis*	119	393	511
LbrM25_V2.0170	*L. braziliensis*	84	675	758
LbrM25_V2.0200	*L. braziliensis*	67	767	833
LbrM25_V2.0220	*L. braziliensis*	154	124	277
LbrM25_V2.0220	*L. braziliensis*	156	558	713
LbrM25_V2.0230	*L. braziliensis*	79	1	79
LbrM25_V2.0230	*L. braziliensis*	57	271	327
LbrM25_V2.0230	*L. braziliensis*	57	271	327
LbrM25_V2.0240	*L. braziliensis*	110	1	110
LbrM25_V2.0250	*L. braziliensis*	258	341	598
LbrM25_V2.0250	*L. braziliensis*	264	615	878
LbrM25_V2.0270	*L. braziliensis*	40	1	40
LbrM25_V2.0270	*L. braziliensis*	49	267	315
LbrM25_V2.0270	*L. braziliensis*	58	323	380
LbrM25_V2.0270	*L. braziliensis*	138	415	552
LbrM25_V2.0270	*L. braziliensis*	61	580	640
LbrM25_V2.0270	*L. braziliensis*	151	745	895
LbrM25_V2.0270	*L. braziliensis*	141	936	1076
LbrM25_V2.0270	*L. braziliensis*	107	1078	1184
LbrM25_V2.0280	*L. braziliensis*	72	99	170
LbrM25_V2.0280	*L. braziliensis*	130	223	352
LbrM25_V2.0280	*L. braziliensis*	46	401	446
LbrM25_V2.0280	*L. braziliensis*	84	547	630
LbrM25_V2.0290	*L. braziliensis*	133	171	303
LbrM25_V2.0290	*L. braziliensis*	165	430	594
LbrM25_V2.0310	*L. braziliensis*	138	241	378

LbrM25_V2.0310	*L. braziliensis*	43	509	551
LbrM25_V2.0310	*L. braziliensis*	83	596	678
LbrM25_V2.0310	*L. braziliensis*	120	689	808
LbrM25_V2.0310	*L. braziliensis*	173	911	1083
LbrM25_V2.0330	*L. braziliensis*	490	1	490
LbrM25_V2.0360	*L. braziliensis*	126	1	126
LbrM25_V2.0370	*L. braziliensis*	236	15	250
LbrM25_V2.0370	*L. braziliensis*	115	528	642
LbrM25_V2.0370	*L. braziliensis*	342	646	987
LbrM25_V2.0370	*L. braziliensis*	58	1005	1062
LbrM25_V2.0370	*L. braziliensis*	588	1183	1770
LbrM25_V2.0380	*L. braziliensis*	156	32	187
LbrM25_V2.0390	*L. braziliensis*	43	45	87
LbrM25_V2.0390	*L. braziliensis*	59	268	326
LbrM25_V2.0390	*L. braziliensis*	91	376	466
LbrM25_V2.0390	*L. braziliensis*	61	499	559
LbrM25_V2.0390	*L. braziliensis*	53	613	665
LbrM25_V2.0400	*L. braziliensis*	89	1	89
LbrM25_V2.0410	*L. braziliensis*	61	1	61
LbrM25_V2.0420	*L. braziliensis*	48	981	1028
LbrM25_V2.0430	*L. braziliensis*	40	359	398
LbrM25_V2.0430	*L. braziliensis*	55	436	490
LbrM25_V2.0430	*L. braziliensis*	44	697	740
LbrM25_V2.0430	*L. braziliensis*	46	823	868
LbrM25_V2.0430	*L. braziliensis*	56	1012	1067
LbrM25_V2.0430	*L. braziliensis*	83	1139	1221
LbrM25_V2.0430	*L. braziliensis*	90	1256	1345
LbrM25_V2.0430	*L. braziliensis*	113	1359	1471
LbrM25_V2.0440	*L. braziliensis*	64	105	168
LbrM25_V2.0450	*L. braziliensis*	64	106	169
LbrM25_V2.0460	*L. braziliensis*	54	115	168
LbrM25_V2.0470	*L. braziliensis*	111	129	239
LbrM25_V2.0480	*L. braziliensis*	59	1	59
LbrM25_V2.0490	*L. braziliensis*	57	63	119
LbrM25_V2.0490	*L. braziliensis*	90	190	279
LbrM25_V2.0490	*L. braziliensis*	68	509	576
LbrM25_V2.0490	*L. braziliensis*	81	597	677
LbrM25_V2.0490	*L. braziliensis*	109	717	825
LbrM25_V2.0490	*L. braziliensis*	45	886	930
LbrM25_V2.0490	*L. braziliensis*	44	1001	1044
LbrM25_V2.0490	*L. braziliensis*	57	1129	1185
LbrM25_V2.0500	*L. braziliensis*	422	142	563
LbrM25_V2.0510	*L. braziliensis*	318	1	318
LbrM25_V2.0510	*L. braziliensis*	81	323	403
LbrM25_V2.0510	*L. braziliensis*	360	429	788
LbrM25_V2.0510	*L. braziliensis*	92	839	930
LbrM25_V2.0510	*L. braziliensis*	169	951	1119

LbrM25_V2.0510	*L. braziliensis*	128	1347	1474
LbrM25_V2.0510	*L. braziliensis*	215	1509	1723
LbrM25_V2.0520	*L. braziliensis*	55	448	502
LbrM25_V2.0520	*L. braziliensis*	106	522	627
LbrM25_V2.0530	*L. braziliensis*	111	109	219
LbrM25_V2.0530	*L. braziliensis*	85	345	429
LbrM25_V2.0540	*L. braziliensis*	66	50	115
LbrM25_V2.0540	*L. braziliensis*	84	305	388
LbrM25_V2.0560	*L. braziliensis*	80	201	280
LbrM25_V2.0570	*L. braziliensis*	95	24	118
LbrM25_V2.0580	*L. braziliensis*	43	100	142
LbrM25_V2.0590	*L. braziliensis*	43	100	142
LbrM25_V2.0600	*L. braziliensis*	43	100	142
LbrM25_V2.0610	*L. braziliensis*	43	100	142
LbrM25_V2.0620	*L. braziliensis*	75	82	156
LbrM25_V2.0620	*L. braziliensis*	139	293	431
LbrM25_V2.0620	*L. braziliensis*	69	443	511
LbrM25_V2.0620	*L. braziliensis*	83	848	930
LbrM25_V2.0630	*L. braziliensis*	77	289	365
LbrM25_V2.0640	*L. braziliensis*	49	20	68
LbrM25_V2.0650	*L. braziliensis*	76	1	76
LbrM25_V2.0650	*L. braziliensis*	56	146	201
LbrM25_V2.0650	*L. braziliensis*	77	309	385
LbrM25_V2.0660	*L. braziliensis*	59	83	141
LbrM25_V2.0660	*L. braziliensis*	132	212	343
LbrM25_V2.0670	*L. braziliensis*	52	12	63
LbrM25_V2.0670	*L. braziliensis*	97	247	343
LbrM25_V2.0670	*L. braziliensis*	43	666	708
LbrM25_V2.0670	*L. braziliensis*	86	749	834
LbrM25_V2.0680	*L. braziliensis*	111	16	126
LbrM25_V2.0690	*L. braziliensis*	75	184	258
LbrM25_V2.0690	*L. braziliensis*	127	296	422
LbrM25_V2.0690	*L. braziliensis*	45	645	689
LbrM25_V2.0690	*L. braziliensis*	42	720	761
LbrM25_V2.0690	*L. braziliensis*	50	788	837
LbrM25_V2.0690	*L. braziliensis*	101	887	987
LbrM25_V2.0710	*L. braziliensis*	112	47	158
LbrM25_V2.0710	*L. braziliensis*	122	288	409
LbrM25_V2.0710	*L. braziliensis*	71	547	617
LbrM25_V2.0710	*L. braziliensis*	190	792	981
LbrM25_V2.0720	*L. braziliensis*	43	108	150
LbrM25_V2.0720	*L. braziliensis*	154	267	420
LbrM25_V2.0730	*L. braziliensis*	72	49	120
LbrM25_V2.0730	*L. braziliensis*	42	182	223
LbrM25_V2.0730	*L. braziliensis*	40	286	325
LbrM25_V2.0730	*L. braziliensis*	442	378	819
LbrM25_V2.0730	*L. braziliensis*	106	837	942

LbrM25_V2.0730	*L. braziliensis*	223	948	1170
LbrM25_V2.0740	*L. braziliensis*	328	1	328
LbrM25_V2.0740	*L. braziliensis*	55	505	559
LbrM25_V2.0740	*L. braziliensis*	211	637	847
LbrM25_V2.0740	*L. braziliensis*	229	891	1119
LbrM25_V2.0760	*L. braziliensis*	88	128	215
LbrM25_V2.0760	*L. braziliensis*	358	247	604
LbrM25_V2.0760	*L. braziliensis*	68	663	730
LbrM25_V2.0760	*L. braziliensis*	103	732	834
LbrM25_V2.0760	*L. braziliensis*	165	857	1021
LbrM25_V2.0760	*L. braziliensis*	231	1064	1294
LbrM25_V2.0770	*L. braziliensis*	47	1	47
LbrM25_V2.0780	*L. braziliensis*	73	118	190
LbrM25_V2.0780	*L. braziliensis*	59	270	328
LbrM25_V2.0790	*L. braziliensis*	95	88	182
LbrM25_V2.0800	*L. braziliensis*	181	1	181
LbrM25_V2.0800	*L. braziliensis*	196	346	541
LbrM25_V2.0800	*L. braziliensis*	82	550	631
LbrM25_V2.0800	*L. braziliensis*	149	688	836
LbrM25_V2.0820	*L. braziliensis*	207	1	207
LbrM25_V2.0820	*L. braziliensis*	54	232	285
LbrM25_V2.0820	*L. braziliensis*	189	316	504
LbrM25_V2.0830	*L. braziliensis*	115	213	327
LbrM25_V2.0830	*L. braziliensis*	43	335	377
LbrM25_V2.0830	*L. braziliensis*	43	335	377
LbrM25_V2.0850	*L. braziliensis*	378	87	464
LbrM25_V2.0850	*L. braziliensis*	80	506	585
LbrM25_V2.0850	*L. braziliensis*	337	624	960
LbrM25_V2.0850	*L. braziliensis*	107	1005	1111
LbrM25_V2.0850	*L. braziliensis*	40	1174	1213
LbrM25_V2.0860	*L. braziliensis*	44	2920	2963
LbrM25_V2.0860	*L. braziliensis*	94	3239	3332
LbrM25_V2.0860	*L. braziliensis*	68	3487	3554
LbrM25_V2.0860	*L. braziliensis*	42	3666	3707
LbrM25_V2.0860	*L. braziliensis*	73	3778	3850
LbrM25_V2.0880	*L. braziliensis*	244	1	244
LbrM25_V2.0880	*L. braziliensis*	44	272	315
LbrM25_V2.0880	*L. braziliensis*	42	387	428
LbrM25_V2.0890	*L. braziliensis*	54	272	325
LbrM25_V2.0910	*L. braziliensis*	61	236	296
LbrM25_V2.0910	*L. braziliensis*	100	329	428
LbrM25_V2.0920	*L. braziliensis*	74	1	74
LbrM25_V2.0920	*L. braziliensis*	49	223	271
LbrM25_V2.0920	*L. braziliensis*	41	402	442
LbrM25_V2.0920	*L. braziliensis*	67	565	631
LbrM25_V2.0920	*L. braziliensis*	53	743	795
LbrM25_V2.0930	*L. braziliensis*	268	1	268

LbrM25_V2.0930	*L. braziliensis*	396	455	850
LbrM25_V2.0950	*L. braziliensis*	373	1	373
LbrM25_V2.0950	*L. braziliensis*	276	469	744
LbrM25_V2.0960	*L. braziliensis*	459	1	459
LbrM25_V2.0960	*L. braziliensis*	87	537	623
LbrM25_V2.0960	*L. braziliensis*	180	708	887
LbrM25_V2.0970	*L. braziliensis*	41	42	82
LbrM25_V2.0970	*L. braziliensis*	279	163	441
LbrM25_V2.1000	*L. braziliensis*	647	1	647
LbrM25_V2.1000	*L. braziliensis*	194	683	876
LbrM25_V2.1000	*L. braziliensis*	113	897	1009
LbrM25_V2.1000	*L. braziliensis*	178	1076	1253
LbrM25_V2.1000	*L. braziliensis*	74	1270	1343
LbrM25_V2.1000	*L. braziliensis*	57	1550	1606
LbrM25_V2.1000	*L. braziliensis*	104	1640	1743
LbrM25_V2.1000	*L. braziliensis*	61	1782	1842
LbrM25_V2.1000	*L. braziliensis*	46	2383	2428
LbrM25_V2.1000	*L. braziliensis*	91	2964	3054
LbrM25_V2.1000	*L. braziliensis*	59	3102	3160
LbrM25_V2.1000	*L. braziliensis*	54	3204	3257
LbrM25_V2.1000	*L. braziliensis*	55	3425	3479
LbrM25_V2.1000	*L. braziliensis*	79	3570	3648
LbrM25_V2.1000	*L. braziliensis*	113	3718	3830
LbrM25_V2.1000	*L. braziliensis*	65	3892	3956
LbrM25_V2.1000	*L. braziliensis*	66	4124	4189
LbrM25_V2.1000	*L. braziliensis*	72	4566	4637
LbrM25_V2.1000	*L. braziliensis*	65	4694	4758
LbrM25_V2.1000	*L. braziliensis*	124	4802	4925
LbrM25_V2.1000	*L. braziliensis*	45	4951	4995
LbrM25_V2.1000	*L. braziliensis*	47	5111	5157
LbrM25_V2.1000	*L. braziliensis*	53	5186	5238
LbrM25_V2.1000	*L. braziliensis*	61	5425	5485
LbrM25_V2.1000	*L. braziliensis*	85	5516	5600
LbrM25_V2.1000	*L. braziliensis*	69	5779	5847
LbrM25_V2.1000	*L. braziliensis*	77	5972	6048
LbrM25_V2.1000	*L. braziliensis*	40	6057	6096
LbrM25_V2.1000	*L. braziliensis*	94	6136	6229
LbrM25_V2.1020	*L. braziliensis*	89	678	766
LbrM25_V2.1020	*L. braziliensis*	46	1023	1068
LbrM25_V2.1030	*L. braziliensis*	73	146	218
LbrM25_V2.1030	*L. braziliensis*	116	285	400
LbrM25_V2.1030	*L. braziliensis*	119	562	680
LbrM25_V2.1040	*L. braziliensis*	44	1	44
LbrM25_V2.1050	*L. braziliensis*	249	27	275
LbrM25_V2.1050	*L. braziliensis*	102	388	489
LbrM25_V2.1050	*L. braziliensis*	41	715	755
LbrM25_V2.1050	*L. braziliensis*	74	1178	1251

LbrM25_V2.1050	*L. braziliensis*	69	1646	1714
LbrM25_V2.1050	*L. braziliensis*	71	1923	1993
LbrM25_V2.1050	*L. braziliensis*	43	2035	2077
LbrM25_V2.1050	*L. braziliensis*	73	2110	2182
LbrM25_V2.1060	*L. braziliensis*	50	57	106
LbrM25_V2.1060	*L. braziliensis*	89	150	238
LbrM25_V2.1060	*L. braziliensis*	44	473	516
LbrM25_V2.1060	*L. braziliensis*	66	555	620
LbrM25_V2.1060	*L. braziliensis*	79	624	702
LbrM25_V2.1060	*L. braziliensis*	44	746	789
LbrM25_V2.1060	*L. braziliensis*	83	837	919
LbrM25_V2.1070	*L. braziliensis*	58	101	158
LbrM25_V2.1070	*L. braziliensis*	71	212	282
LbrM25_V2.1070	*L. braziliensis*	84	388	471
LbrM25_V2.1080	*L. braziliensis*	63	39	101
LbrM25_V2.1080	*L. braziliensis*	99	131	229
LbrM25_V2.1120	*L. braziliensis*	127	77	203
LbrM25_V2.1120	*L. braziliensis*	145	226	370
LbrM25_V2.1120	*L. braziliensis*	55	411	465
LbrM25_V2.1120	*L. braziliensis*	59	512	570
LbrM25_V2.1130	*L. braziliensis*	56	1	56
LbrM25_V2.1160	*L. braziliensis*	40	1	40
LbrM25_V2.1170	*L. braziliensis*	91	64	154
LbrM25_V2.1170	*L. braziliensis*	56	267	322
LbrM25_V2.1170	*L. braziliensis*	49	382	430
LbrM25_V2.1170	*L. braziliensis*	66	617	682
LbrM25_V2.1170	*L. braziliensis*	63	716	778
LbrM25_V2.1180	*L. braziliensis*	254	1	254
LbrM25_V2.1180	*L. braziliensis*	52	872	923
LbrM25_V2.1190	*L. braziliensis*	49	85	133
LbrM25_V2.1190	*L. braziliensis*	46	365	410
LbrM25_V2.1190	*L. braziliensis*	46	365	410
LbrM25_V2.1200	*L. braziliensis*	95	1	95
LbrM25_V2.1200	*L. braziliensis*	94	142	235
LbrM25_V2.1200	*L. braziliensis*	129	433	561
LbrM25_V2.1200	*L. braziliensis*	43	647	689
LbrM25_V2.1200	*L. braziliensis*	55	832	886
LbrM25_V2.1200	*L. braziliensis*	89	1010	1098
LbrM25_V2.1200	*L. braziliensis*	85	1372	1456
LbrM25_V2.1200	*L. braziliensis*	85	1612	1696
LbrM25_V2.1200	*L. braziliensis*	43	1788	1830
LbrM25_V2.1210	*L. braziliensis*	48	40	87
LbrM25_V2.1210	*L. braziliensis*	106	157	262
LbrM25_V2.1210	*L. braziliensis*	138	423	560
LbrM25_V2.1210	*L. braziliensis*	51	565	615
LbrM25_V2.1210	*L. braziliensis*	66	736	801
LbrM25_V2.1210	*L. braziliensis*	81	909	989

LbrM25_V2.1210	*L. braziliensis*	138	1432	1569
LbrM25_V2.1210	*L. braziliensis*	43	1643	1685
LbrM25_V2.1210	*L. braziliensis*	74	1700	1773
LbrM25_V2.1220	*L. braziliensis*	66	97	162
LbrM25_V2.1220	*L. braziliensis*	97	192	288
LbrM25_V2.1220	*L. braziliensis*	120	322	441
LbrM25_V2.1220	*L. braziliensis*	40	505	544
LbrM25_V2.1220	*L. braziliensis*	122	715	836
LbrM25_V2.1220	*L. braziliensis*	60	874	933
LbrM25_V2.1220	*L. braziliensis*	279	1020	1298
LbrM25_V2.1220	*L. braziliensis*	67	1364	1430
LbrM25_V2.1220	*L. braziliensis*	85	1457	1541
LbrM25_V2.1220	*L. braziliensis*	40	1711	1750
LbrM25_V2.1220	*L. braziliensis*	89	1821	1909
LbrM25_V2.1220	*L. braziliensis*	198	2013	2210
LbrM25_V2.1230	*L. braziliensis*	57	91	147
LbrM25_V2.1230	*L. braziliensis*	62	174	235
LbrM25_V2.1230	*L. braziliensis*	58	245	302
LbrM25_V2.1230	*L. braziliensis*	158	328	485
LbrM25_V2.1240	*L. braziliensis*	60	1	60
LbrM25_V2.1240	*L. braziliensis*	59	250	308
LbrM25_V2.1240	*L. braziliensis*	78	545	622
LbrM25_V2.1240	*L. braziliensis*	40	713	752
LbrM25_V2.1240	*L. braziliensis*	71	892	962
LbrM25_V2.1260	*L. braziliensis*	60	15	74
LbrM25_V2.1260	*L. braziliensis*	73	135	207
LbrM25_V2.1260	*L. braziliensis*	47	457	503
LbrM25_V2.1260	*L. braziliensis*	92	543	634
LbrM25_V2.1270	*L. braziliensis*	87	1	87
LbrM25_V2.1270	*L. braziliensis*	70	117	186
LbrM25_V2.1320	*L. braziliensis*	40	211	250
LbrM25_V2.1320	*L. braziliensis*	62	643	704
LbrM25_V2.1320	*L. braziliensis*	62	643	704
LbrM25_V2.1350	*L. braziliensis*	81	123	203
LbrM25_V2.1360	*L. braziliensis*	125	224	348
LbrM25_V2.1360	*L. braziliensis*	119	412	530
LbrM25_V2.1360	*L. braziliensis*	290	563	852
LbrM25_V2.1370	*L. braziliensis*	57	1	57
LbrM25_V2.1380	*L. braziliensis*	91	1	91
LbrM25_V2.1380	*L. braziliensis*	746	101	846
LbrM25_V2.1390	*L. braziliensis*	49	1	49
LbrM25_V2.1390	*L. braziliensis*	181	69	249
LbrM25_V2.1390	*L. braziliensis*	94	260	353
LbrM25_V2.1390	*L. braziliensis*	71	379	449
LbrM25_V2.1390	*L. braziliensis*	150	604	753
LbrM25_V2.1390	*L. braziliensis*	188	763	950
LbrM25_V2.1400	*L. braziliensis*	154	11	164

LbrM25_V2.1400	*L. braziliensis*	324	184	507
LbrM25_V2.1410	*L. braziliensis*	101	40	140
LbrM25_V2.1410	*L. braziliensis*	71	155	225
LbrM25_V2.1430	*L. braziliensis*	93	121	213
LbrM25_V2.1450	*L. braziliensis*	120	1	120
LbrM25_V2.1470	*L. braziliensis*	41	166	206
LbrM25_V2.1470	*L. braziliensis*	65	246	310
LbrM25_V2.1470	*L. braziliensis*	65	246	310
LbrM25_V2.1480	*L. braziliensis*	107	1	107
LbrM25_V2.1490	*L. braziliensis*	45	71	115
LbrM25_V2.1500	*L. braziliensis*	45	516	560
LbrM25_V2.1510	*L. braziliensis*	79	317	395
LbrM25_V2.1510	*L. braziliensis*	240	473	712
LbrM25_V2.1510	*L. braziliensis*	57	749	805
LbrM25_V2.1510	*L. braziliensis*	176	870	1045
LbrM25_V2.1520	*L. braziliensis*	40	33	72
LbrM25_V2.1530	*L. braziliensis*	78	1	78
LbrM25_V2.1530	*L. braziliensis*	133	457	589
LbrM25_V2.1530	*L. braziliensis*	126	612	737
LbrM25_V2.1530	*L. braziliensis*	215	780	994
LbrM25_V2.1540	*L. braziliensis*	40	128	167
LbrM25_V2.1540	*L. braziliensis*	332	741	1072
LbrM25_V2.1540	*L. braziliensis*	93	1076	1168
LbrM25_V2.1540	*L. braziliensis*	272	1174	1445
LbrM25_V2.1550	*L. braziliensis*	40	232	271
LbrM25_V2.1550	*L. braziliensis*	50	378	427
LbrM25_V2.1550	*L. braziliensis*	102	448	549
LbrM25_V2.1560	*L. braziliensis*	55	257	311
LbrM25_V2.1560	*L. braziliensis*	95	403	497
LbrM25_V2.1560	*L. braziliensis*	87	809	895
LbrM25_V2.1590	*L. braziliensis*	174	1	174
LbrM25_V2.1610	*L. braziliensis*	131	1	131
LbrM25_V2.1610	*L. braziliensis*	116	654	769
LbrM25_V2.1610	*L. braziliensis*	82	893	974
LbrM25_V2.1620	*L. braziliensis*	81	1	81
LbrM25_V2.1630	*L. braziliensis*	102	131	232
LbrM25_V2.1660	*L. braziliensis*	88	1	88
LbrM25_V2.1670	*L. braziliensis*	76	285	360
LbrM25_V2.1690	*L. braziliensis*	86	1	86
LbrM25_V2.1690	*L. braziliensis*	63	149	211
LbrM25_V2.1700	*L. braziliensis*	80	221	300
LbrM25_V2.1710	*L. braziliensis*	80	221	300
LbrM25_V2.1720	*L. braziliensis*	44	383	426
LbrM25_V2.1730	*L. braziliensis*	101	24	124
LbrM25_V2.1740	*L. braziliensis*	59	243	301
LbrM25_V2.1770	*L. braziliensis*	136	174	309
LbrM25_V2.1770	*L. braziliensis*	48	330	377

LbrM25_V2.1780	*L. braziliensis*	111	111	221
LbrM25_V2.1800	*L. braziliensis*	71	379	449
LbrM25_V2.1800	*L. braziliensis*	58	489	546
LbrM25_V2.1800	*L. braziliensis*	48	585	632
LbrM25_V2.1820	*L. braziliensis*	52	22	73
LbrM25_V2.1830	*L. braziliensis*	42	378	419
LbrM25_V2.1840	*L. braziliensis*	42	1	42
LbrM25_V2.1850	*L. braziliensis*	42	123	164
LbrM25_V2.1890	*L. braziliensis*	59	62	120
LbrM25_V2.1890	*L. braziliensis*	123	261	383
LbrM25_V2.1900	*L. braziliensis*	47	362	408
LbrM25_V2.1900	*L. braziliensis*	41	508	548
LbrM25_V2.1900	*L. braziliensis*	41	508	548
LbrM25_V2.1930	*L. braziliensis*	47	1	47
LbrM25_V2.1930	*L. braziliensis*	50	340	389
LbrM25_V2.1940	*L. braziliensis*	90	48	137
LbrM25_V2.1950	*L. braziliensis*	57	1	57
LbrM25_V2.1950	*L. braziliensis*	90	488	577
LbrM25_V2.2000	*L. braziliensis*	301	395	695
LbrM25_V2.2030	*L. braziliensis*	200	300	499
LbrM25_V2.2040	*L. braziliensis*	65	184	248
LbrM25_V2.2040	*L. braziliensis*	211	270	480
LbrM25_V2.2060	*L. braziliensis*	68	77	144
LbrM25_V2.2060	*L. braziliensis*	40	475	514
LbrM25_V2.2060	*L. braziliensis*	40	475	514
LbrM25_V2.2070	*L. braziliensis*	71	72	142
LbrM25_V2.2070	*L. braziliensis*	104	291	394
LbrM25_V2.2080	*L. braziliensis*	262	1	262
LbrM25_V2.2100	*L. braziliensis*	54	58	111
LbrM25_V2.2100	*L. braziliensis*	79	392	470
LbrM25_V2.2100	*L. braziliensis*	79	392	470
LbrM25_V2.2140	*L. braziliensis*	98	1	98
LbrM25_V2.2140	*L. braziliensis*	82	596	677
LbrM25_V2.2140	*L. braziliensis*	52	1023	1074
LbrM25_V2.2150	*L. braziliensis*	54	51	104
LbrM25_V2.2170	*L. braziliensis*	43	148	190
LbrM25_V2.2180	*L. braziliensis*	105	84	188
LbrM25_V2.2200	*L. braziliensis*	71	49	119
LbrM25_V2.2210	*L. braziliensis*	220	75	294
LbrM25_V2.2220	*L. braziliensis*	47	34	80
LbrM25_V2.2230	*L. braziliensis*	130	43	172
LbrM25_V2.2230	*L. braziliensis*	77	266	342
LbrM25_V2.2230	*L. braziliensis*	62	363	424
LbrM25_V2.2250	*L. braziliensis*	80	28	107
LbrM25_V2.2250	*L. braziliensis*	132	180	311
LbrM25_V2.2260	*L. braziliensis*	66	21	86
LbrM25_V2.2260	*L. braziliensis*	58	149	206

LbrM25_V2.2270	*L. braziliensis*	81	188	268
LbrM25_V2.2270	*L. braziliensis*	61	1175	1235
LbrM25_V2.2270	*L. braziliensis*	96	1464	1559
LbrM25_V2.2300	*L. braziliensis*	47	1	47
LbrM25_V2.2300	*L. braziliensis*	102	228	329
LbrM25_V2.2300	*L. braziliensis*	262	354	615
LbrM25_V2.2300	*L. braziliensis*	98	707	804
LbrM25_V2.2300	*L. braziliensis*	61	821	881
LbrM25_V2.2300	*L. braziliensis*	103	928	1030
LbrM25_V2.2310	*L. braziliensis*	44	54	97
LbrM25_V2.2310	*L. braziliensis*	47	125	171
LbrM25_V2.2310	*L. braziliensis*	47	125	171
LbrM25_V2.2320	*L. braziliensis*	69	78	146
LbrM25_V2.2320	*L. braziliensis*	283	482	764
LbrM25_V2.2320	*L. braziliensis*	44	776	819
LbrM25_V2.2320	*L. braziliensis*	50	825	874
LbrM25_V2.2320	*L. braziliensis*	75	885	959
LbrM25_V2.2320	*L. braziliensis*	42	1012	1053
LbrM25_V2.2340	*L. braziliensis*	109	476	584
LbrM25_V2.2370	*L. braziliensis*	58	193	250
LbrM25_V2.2370	*L. braziliensis*	69	365	433
LbrM25_V2.2380	*L. braziliensis*	45	1	45
LbrM25_V2.2380	*L. braziliensis*	55	68	122
LbrM25_V2.2380	*L. braziliensis*	77	172	248
LbrM25_V2.2400	*L. braziliensis*	41	109	149
LbrM25_V2.2410	*L. braziliensis*	42	119	160
LbrM25_V2.2410	*L. braziliensis*	47	564	610
LbrM25_V2.2410	*L. braziliensis*	52	731	782
LbrM25_V2.2410	*L. braziliensis*	43	1123	1165
LbrM25_V2.2420	*L. braziliensis*	50	23	72
LbrM25_V2.2420	*L. braziliensis*	521	131	651
LbrM25_V2.2420	*L. braziliensis*	67	823	889
LbrM25_V2.2420	*L. braziliensis*	78	1095	1172
LbrM25_V2.2430	*L. braziliensis*	64	355	418
LbrM25_V2.2430	*L. braziliensis*	66	451	516
LbrM25_V2.2430	*L. braziliensis*	66	451	516
LbrM25_V2.2440	*L. braziliensis*	44	112	155
LbrM25_V2.2440	*L. braziliensis*	121	747	867
LbrM25_V2.2440	*L. braziliensis*	61	1121	1181
LbrM25_V2.2440	*L. braziliensis*	50	1227	1276
LbrM25_V2.2450	*L. braziliensis*	874	1	874
LbrM25_V2.2460	*L. braziliensis*	47	1	47
LbrM25_V2.2470	*L. braziliensis*	162	46	207
LbrM25_V2.2470	*L. braziliensis*	91	244	334
LbrM25_V2.2470	*L. braziliensis*	113	432	544
LbrM25_V2.2470	*L. braziliensis*	54	582	635
LbrM25_V2.2470	*L. braziliensis*	53	927	979

LbrM25_V2.2490	*L. braziliensis*	231	30	260
LbrM25_V2.2490	*L. braziliensis*	92	723	814
LbrM25_V2.2520	*L. braziliensis*	94	39	132
LbrM25_V2.2520	*L. braziliensis*	61	391	451
LbrM25_V2.2520	*L. braziliensis*	59	497	555
LbrM25_V2.2520	*L. braziliensis*	43	606	648
LbrM25_V2.2520	*L. braziliensis*	44	688	731
LbrM25_V2.2520	*L. braziliensis*	52	889	940
LbrM25_V2.2520	*L. braziliensis*	124	995	1118
LbrM25_V2.2520	*L. braziliensis*	56	1148	1203
LbrM25_V2.2520	*L. braziliensis*	138	1233	1370
LbrM25_V2.2520	*L. braziliensis*	119	1410	1528
LbrM25_V2.2530	*L. braziliensis*	220	1	220
LbrM25_V2.2530	*L. braziliensis*	70	382	451
LbrM25_V2.2530	*L. braziliensis*	91	527	617
LbrM25_V2.2530	*L. braziliensis*	46	773	818
LbrM25_V2.2550	*L. braziliensis*	83	1	83
LbrM25_V2.2560	*L. braziliensis*	40	512	551
LbrM25_V2.2570	*L. braziliensis*	69	1	69
LbrM25_V2.2570	*L. braziliensis*	95	85	179
LbrM25_V2.2570	*L. braziliensis*	83	209	291
LbrM25_V2.2570	*L. braziliensis*	48	334	381
LbrM25_V2.2570	*L. braziliensis*	50	675	724
LbrM25_V2.2570	*L. braziliensis*	208	733	940
LbrM25_V2.2580	*L. braziliensis*	57	93	149
LbrM25_V2.2580	*L. braziliensis*	187	245	431
LbrM25_V2.2580	*L. braziliensis*	68	436	503
LbrM25_V2.2590	*L. braziliensis*	82	59	140
LbrM25_V2.2590	*L. braziliensis*	44	278	321
LbrM25_V2.2590	*L. braziliensis*	49	445	493
LbrM25_V2.2590	*L. braziliensis*	53	548	600
LbrM25_V2.2600	*L. braziliensis*	131	1	131
LbrM25_V2.2600	*L. braziliensis*	47	218	264
LbrM25_V2.2600	*L. braziliensis*	105	275	379
LbrM25_V2.2600	*L. braziliensis*	158	597	754
LbrM25_V2.2600	*L. braziliensis*	125	919	1043
LbrM25_V2.2600	*L. braziliensis*	71	1073	1143
LbrM25_V2.2600	*L. braziliensis*	172	1156	1327
LbrM25_V2.2610	*L. braziliensis*	121	22	142
LbrM25_V2.2610	*L. braziliensis*	56	935	990
LbrM25_V2.2620	*L. braziliensis*	58	816	873
LbrM26_V2.0030	*L. braziliensis*	58	305	362
LbrM26_V2.0070	*L. braziliensis*	157	1	157
LbrM26_V2.0070	*L. braziliensis*	98	169	266
LbrM26_V2.0080	*L. braziliensis*	67	236	302
LbrM26_V2.0140	*L. braziliensis*	66	130	195
LbrM26_V2.0150	*L. braziliensis*	92	1	92

LbrM26_V2.0160	*L. braziliensis*	60	1	60
LbrM26_V2.0170	*L. braziliensis*	76	1	76
LbrM26_V2.0190	*L. braziliensis*	55	1	55
LbrM26_V2.0210a	*L. braziliensis*	69	158	226
LbrM26_V2.0260	*L. braziliensis*	54	80	133
LbrM26_V2.0260	*L. braziliensis*	70	309	378
LbrM26_V2.0260	*L. braziliensis*	70	309	378
LbrM26_V2.0280	*L. braziliensis*	48	597	644
LbrM26_V2.0320	*L. braziliensis*	64	94	157
LbrM26_V2.0340	*L. braziliensis*	75	1	75
LbrM26_V2.0370	*L. braziliensis*	89	588	676
LbrM26_V2.0380	*L. braziliensis*	74	1	74
LbrM26_V2.0380	*L. braziliensis*	48	775	822
LbrM26_V2.0380	*L. braziliensis*	109	912	1020
LbrM26_V2.0380	*L. braziliensis*	47	1188	1234
LbrM26_V2.0380	*L. braziliensis*	67	1487	1553
LbrM26_V2.0390	*L. braziliensis*	62	464	525
LbrM26_V2.0400	*L. braziliensis*	43	212	254
LbrM26_V2.0410	*L. braziliensis*	43	16	58
LbrM26_V2.0420	*L. braziliensis*	63	1	63
LbrM26_V2.0440	*L. braziliensis*	43	205	247
LbrM26_V2.0440	*L. braziliensis*	80	557	636
LbrM26_V2.0460	*L. braziliensis*	187	19	205
LbrM26_V2.0490	*L. braziliensis*	74	823	896
LbrM26_V2.0500	*L. braziliensis*	40	237	276
LbrM26_V2.0510	*L. braziliensis*	138	1	138
LbrM26_V2.0530	*L. braziliensis*	50	677	726
LbrM26_V2.0540	*L. braziliensis*	131	1	131
LbrM26_V2.0540	*L. braziliensis*	42	319	360
LbrM26_V2.0540	*L. braziliensis*	56	383	438
LbrM26_V2.0550	*L. braziliensis*	70	414	483
LbrM26_V2.0550	*L. braziliensis*	61	853	913
LbrM26_V2.0550	*L. braziliensis*	61	853	913
LbrM26_V2.0560	*L. braziliensis*	84	138	221
LbrM26_V2.0570	*L. braziliensis*	124	1	124
LbrM26_V2.0570	*L. braziliensis*	49	178	226
LbrM26_V2.0570	*L. braziliensis*	230	313	542
LbrM26_V2.0590	*L. braziliensis*	90	70	159
LbrM26_V2.0590	*L. braziliensis*	103	248	350
LbrM26_V2.0590	*L. braziliensis*	54	364	417
LbrM26_V2.0590	*L. braziliensis*	64	533	596
LbrM26_V2.0600	*L. braziliensis*	77	1	77
LbrM26_V2.0600	*L. braziliensis*	69	122	190
LbrM26_V2.0600	*L. braziliensis*	110	392	501
LbrM26_V2.0600	*L. braziliensis*	86	590	675
LbrM26_V2.0610	*L. braziliensis*	126	66	191
LbrM26_V2.0610	*L. braziliensis*	114	220	333

LbrM26_V2.0620	*L. braziliensis*	85	32	116
LbrM26_V2.0660	*L. braziliensis*	71	479	549
LbrM26_V2.0660	*L. braziliensis*	45	692	736
LbrM26_V2.0660	*L. braziliensis*	66	1132	1197
LbrM26_V2.0700	*L. braziliensis*	135	69	203
LbrM26_V2.0710	*L. braziliensis*	108	39	146
LbrM26_V2.0710	*L. braziliensis*	102	230	331
LbrM26_V2.0710	*L. braziliensis*	50	449	498
LbrM26_V2.0720	*L. braziliensis*	55	462	516
LbrM26_V2.0720	*L. braziliensis*	74	603	676
LbrM26_V2.0720	*L. braziliensis*	74	603	676
LbrM26_V2.0730	*L. braziliensis*	62	1	62
LbrM26_V2.0730	*L. braziliensis*	95	118	212
LbrM26_V2.0730	*L. braziliensis*	165	254	418
LbrM26_V2.0730	*L. braziliensis*	130	542	671
LbrM26_V2.0730	*L. braziliensis*	74	920	993
LbrM26_V2.0730	*L. braziliensis*	44	1082	1125
LbrM26_V2.0740	*L. braziliensis*	99	58	156
LbrM26_V2.0740	*L. braziliensis*	177	188	364
LbrM26_V2.0740	*L. braziliensis*	94	400	493
LbrM26_V2.0740	*L. braziliensis*	58	584	641
LbrM26_V2.0740	*L. braziliensis*	197	895	1091
LbrM26_V2.0740	*L. braziliensis*	138	1198	1335
LbrM26_V2.0740	*L. braziliensis*	49	1353	1401
LbrM26_V2.0760	*L. braziliensis*	146	154	299
LbrM26_V2.0760	*L. braziliensis*	144	432	575
LbrM26_V2.0760	*L. braziliensis*	228	1298	1525
LbrM26_V2.0760	*L. braziliensis*	143	1528	1670
LbrM26_V2.0780	*L. braziliensis*	457	87	543
LbrM26_V2.0810	*L. braziliensis*	132	208	339
LbrM26_V2.0850	*L. braziliensis*	282	115	396
LbrM26_V2.0850	*L. braziliensis*	116	593	708
LbrM26_V2.0850	*L. braziliensis*	52	834	885
LbrM26_V2.0860	*L. braziliensis*	74	248	321
LbrM26_V2.0870	*L. braziliensis*	56	461	516
LbrM26_V2.0870	*L. braziliensis*	44	556	599
LbrM26_V2.0870	*L. braziliensis*	65	639	703
LbrM26_V2.0880	*L. braziliensis*	352	161	512
LbrM26_V2.0880	*L. braziliensis*	103	577	679
LbrM26_V2.0880	*L. braziliensis*	75	700	774
LbrM26_V2.0910	*L. braziliensis*	87	1	87
LbrM26_V2.0910	*L. braziliensis*	50	272	321
LbrM26_V2.0910	*L. braziliensis*	127	707	833
LbrM26_V2.0910	*L. braziliensis*	44	895	938
LbrM26_V2.0920	*L. braziliensis*	45	140	184
LbrM26_V2.0930	*L. braziliensis*	42	130	171
LbrM26_V2.0940	*L. braziliensis*	63	240	302

LbrM26_V2.0940	*L. braziliensis*	74	304	377
LbrM26_V2.0940	*L. braziliensis*	74	304	377
LbrM26_V2.0950	*L. braziliensis*	137	148	284
LbrM26_V2.0950	*L. braziliensis*	49	314	362
LbrM26_V2.0960	*L. braziliensis*	58	1	58
LbrM26_V2.0960	*L. braziliensis*	241	70	310
LbrM26_V2.0970	*L. braziliensis*	48	1	48
LbrM26_V2.0970	*L. braziliensis*	45	193	237
LbrM26_V2.0970	*L. braziliensis*	43	308	350
LbrM26_V2.0970	*L. braziliensis*	52	891	942
LbrM26_V2.0970	*L. braziliensis*	70	1176	1245
LbrM26_V2.0970	*L. braziliensis*	40	1285	1324
LbrM26_V2.0980	*L. braziliensis*	126	438	563
LbrM26_V2.0990	*L. braziliensis*	217	1	217
LbrM26_V2.0990	*L. braziliensis*	652	229	880
LbrM26_V2.1010	*L. braziliensis*	96	133	228
LbrM26_V2.1020	*L. braziliensis*	62	276	337
LbrM26_V2.1020	*L. braziliensis*	41	370	410
LbrM26_V2.1020	*L. braziliensis*	41	370	410
LbrM26_V2.1040	*L. braziliensis*	50	1	50
LbrM26_V2.1040	*L. braziliensis*	55	583	637
LbrM26_V2.1040	*L. braziliensis*	66	2635	2700
LbrM26_V2.1040	*L. braziliensis*	40	2834	2873
LbrM26_V2.1040	*L. braziliensis*	63	3145	3207
LbrM26_V2.1050	*L. braziliensis*	53	1	53
LbrM26_V2.1050	*L. braziliensis*	62	77	138
LbrM26_V2.1050	*L. braziliensis*	82	696	777
LbrM26_V2.1080	*L. braziliensis*	230	136	365
LbrM26_V2.1090	*L. braziliensis*	97	24	120
LbrM26_V2.1090	*L. braziliensis*	83	153	235
LbrM26_V2.1090	*L. braziliensis*	45	463	507
LbrM26_V2.1100	*L. braziliensis*	75	1	75
LbrM26_V2.1100	*L. braziliensis*	96	98	193
LbrM26_V2.1100	*L. braziliensis*	296	249	544
LbrM26_V2.1100	*L. braziliensis*	74	559	632
LbrM26_V2.1100	*L. braziliensis*	188	642	829
LbrM26_V2.1100	*L. braziliensis*	40	859	898
LbrM26_V2.1100	*L. braziliensis*	75	925	999
LbrM26_V2.1100	*L. braziliensis*	108	1075	1182
LbrM26_V2.1100	*L. braziliensis*	93	1229	1321
LbrM26_V2.1100	*L. braziliensis*	58	1416	1473
LbrM26_V2.1100	*L. braziliensis*	63	1528	1590
LbrM26_V2.1110	*L. braziliensis*	173	22	194
LbrM26_V2.1140	*L. braziliensis*	108	73	180
LbrM26_V2.1140	*L. braziliensis*	89	203	291
LbrM26_V2.1140	*L. braziliensis*	71	404	474
LbrM26_V2.1150	*L. braziliensis*	75	26	100

LbrM26_V2.1150	*L. braziliensis*	317	111	427
LbrM26_V2.1150	*L. braziliensis*	81	545	625
LbrM26_V2.1150	*L. braziliensis*	82	642	723
LbrM26_V2.1150	*L. braziliensis*	155	734	888
LbrM26_V2.1150	*L. braziliensis*	157	894	1050
LbrM26_V2.1150	*L. braziliensis*	46	1074	1119
LbrM26_V2.1150	*L. braziliensis*	53	1154	1206
LbrM26_V2.1150	*L. braziliensis*	42	1218	1259
LbrM26_V2.1150	*L. braziliensis*	114	1314	1427
LbrM26_V2.1150	*L. braziliensis*	171	1575	1745
LbrM26_V2.1160	*L. braziliensis*	113	1	113
LbrM26_V2.1160	*L. braziliensis*	42	118	159
LbrM26_V2.1170	*L. braziliensis*	133	1	133
LbrM26_V2.1170	*L. braziliensis*	93	291	383
LbrM26_V2.1170	*L. braziliensis*	42	560	601
LbrM26_V2.1170	*L. braziliensis*	88	620	707
LbrM26_V2.1180	*L. braziliensis*	70	122	191
LbrM26_V2.1180	*L. braziliensis*	64	303	366
LbrM26_V2.1180	*L. braziliensis*	41	424	464
LbrM26_V2.1180	*L. braziliensis*	52	484	535
LbrM26_V2.1180	*L. braziliensis*	53	580	632
LbrM26_V2.1180	*L. braziliensis*	62	669	730
LbrM26_V2.1180	*L. braziliensis*	84	749	832
LbrM26_V2.1190	*L. braziliensis*	70	1	70
LbrM26_V2.1190	*L. braziliensis*	45	96	140
LbrM26_V2.1190	*L. braziliensis*	68	212	279
LbrM26_V2.1190	*L. braziliensis*	40	288	327
LbrM26_V2.1190	*L. braziliensis*	127	343	469
LbrM26_V2.1200	*L. braziliensis*	53	1	53
LbrM26_V2.1200	*L. braziliensis*	50	89	138
LbrM26_V2.1200	*L. braziliensis*	46	162	207
LbrM26_V2.1200	*L. braziliensis*	54	245	298
LbrM26_V2.1200	*L. braziliensis*	55	401	455
LbrM26_V2.1210	*L. braziliensis*	184	17	200
LbrM26_V2.1210	*L. braziliensis*	82	209	290
LbrM26_V2.1210	*L. braziliensis*	80	313	392
LbrM26_V2.1210	*L. braziliensis*	71	402	472
LbrM26_V2.1210	*L. braziliensis*	76	480	555
LbrM26_V2.1210	*L. braziliensis*	78	577	654
LbrM26_V2.1210	*L. braziliensis*	418	862	1279
LbrM26_V2.1210	*L. braziliensis*	113	1460	1572
LbrM26_V2.1210	*L. braziliensis*	76	1577	1652
LbrM26_V2.1220	*L. braziliensis*	62	51	112
LbrM26_V2.1220	*L. braziliensis*	42	253	294
LbrM26_V2.1220	*L. braziliensis*	52	326	377
LbrM26_V2.1230	*L. braziliensis*	68	175	242
LbrM26_V2.1240	*L. braziliensis*	59	120	178

LbrM26_V2.1240	*L. braziliensis*	130	240	369
LbrM26_V2.1240	*L. braziliensis*	49	464	512
LbrM26_V2.1240	*L. braziliensis*	149	518	666
LbrM26_V2.1250	*L. braziliensis*	79	1	79
LbrM26_V2.1250	*L. braziliensis*	143	417	559
LbrM26_V2.1250	*L. braziliensis*	123	570	692
LbrM26_V2.1250	*L. braziliensis*	49	804	852
LbrM26_V2.1250	*L. braziliensis*	57	893	949
LbrM26_V2.1250	*L. braziliensis*	41	951	991
LbrM26_V2.1250	*L. braziliensis*	104	1022	1125
LbrM26_V2.1250	*L. braziliensis*	59	1400	1458
LbrM26_V2.1250	*L. braziliensis*	126	1664	1789
LbrM26_V2.1250	*L. braziliensis*	266	1795	2060
LbrM26_V2.1250	*L. braziliensis*	151	2080	2230
LbrM26_V2.1250	*L. braziliensis*	187	2237	2423
LbrM26_V2.1250	*L. braziliensis*	72	2613	2684
LbrM26_V2.1250	*L. braziliensis*	132	2843	2974
LbrM26_V2.1260	*L. braziliensis*	57	494	550
LbrM26_V2.1270	*L. braziliensis*	71	462	532
LbrM26_V2.1270	*L. braziliensis*	165	535	699
LbrM26_V2.1280	*L. braziliensis*	261	78	338
LbrM26_V2.1280	*L. braziliensis*	312	454	765
LbrM26_V2.1280	*L. braziliensis*	44	884	927
LbrM26_V2.1280	*L. braziliensis*	57	1074	1130
LbrM26_V2.1280	*L. braziliensis*	98	1159	1256
LbrM26_V2.1280	*L. braziliensis*	204	1308	1511
LbrM26_V2.1290	*L. braziliensis*	67	1	67
LbrM26_V2.1290	*L. braziliensis*	72	327	398
LbrM26_V2.1290	*L. braziliensis*	44	428	471
LbrM26_V2.1290	*L. braziliensis*	131	562	692
LbrM26_V2.1300	*L. braziliensis*	108	113	220
LbrM26_V2.1300	*L. braziliensis*	314	262	575
LbrM26_V2.1300	*L. braziliensis*	60	680	739
LbrM26_V2.1300	*L. braziliensis*	83	973	1055
LbrM26_V2.1300	*L. braziliensis*	142	1151	1292
LbrM26_V2.1310	*L. braziliensis*	53	49	101
LbrM26_V2.1310	*L. braziliensis*	60	151	210
LbrM26_V2.1310	*L. braziliensis*	54	453	506
LbrM26_V2.1320	*L. braziliensis*	60	440	499
LbrM26_V2.1320	*L. braziliensis*	50	598	647
LbrM26_V2.1320	*L. braziliensis*	51	728	778
LbrM26_V2.1320	*L. braziliensis*	320	870	1189
LbrM26_V2.1330	*L. braziliensis*	60	106	165
LbrM26_V2.1340	*L. braziliensis*	46	14	59
LbrM26_V2.1340	*L. braziliensis*	48	230	277
LbrM26_V2.1340	*L. braziliensis*	48	230	277
LbrM26_V2.1350	*L. braziliensis*	74	268	341

LbrM26_V2.1350	*L. braziliensis*	56	359	414
LbrM26_V2.1350	*L. braziliensis*	56	359	414
LbrM26_V2.1360	*L. braziliensis*	56	1	56
LbrM26_V2.1360	*L. braziliensis*	83	110	192
LbrM26_V2.1360	*L. braziliensis*	45	485	529
LbrM26_V2.1390	*L. braziliensis*	127	1	127
LbrM26_V2.1390	*L. braziliensis*	65	178	242
LbrM26_V2.1390	*L. braziliensis*	97	261	357
LbrM26_V2.1390	*L. braziliensis*	63	493	555
LbrM26_V2.1390	*L. braziliensis*	82	625	706
LbrM26_V2.1410	*L. braziliensis*	107	1	107
LbrM26_V2.1410	*L. braziliensis*	115	160	274
LbrM26_V2.1410	*L. braziliensis*	95	349	443
LbrM26_V2.1410	*L. braziliensis*	55	544	598
LbrM26_V2.1410	*L. braziliensis*	134	637	770
LbrM26_V2.1420	*L. braziliensis*	64	1	64
LbrM26_V2.1420	*L. braziliensis*	61	79	139
LbrM26_V2.1420	*L. braziliensis*	95	164	258
LbrM26_V2.1430	*L. braziliensis*	70	131	200
LbrM26_V2.1430	*L. braziliensis*	74	327	400
LbrM26_V2.1430	*L. braziliensis*	74	327	400
LbrM26_V2.1440	*L. braziliensis*	56	408	463
LbrM26_V2.1440	*L. braziliensis*	49	682	730
LbrM26_V2.1440	*L. braziliensis*	50	813	862
LbrM26_V2.1440	*L. braziliensis*	66	949	1014
LbrM26_V2.1440	*L. braziliensis*	69	1111	1179
LbrM26_V2.1440	*L. braziliensis*	199	1294	1492
LbrM26_V2.1440	*L. braziliensis*	66	1868	1933
LbrM26_V2.1440	*L. braziliensis*	250	1993	2242
LbrM26_V2.1440	*L. braziliensis*	128	2263	2390
LbrM26_V2.1450	*L. braziliensis*	222	1	222
LbrM26_V2.1450	*L. braziliensis*	81	237	317
LbrM26_V2.1450	*L. braziliensis*	44	360	403
LbrM26_V2.1460	*L. braziliensis*	136	74	209
LbrM26_V2.1460	*L. braziliensis*	47	236	282
LbrM26_V2.1460	*L. braziliensis*	121	304	424
LbrM26_V2.1460	*L. braziliensis*	73	629	701
LbrM26_V2.1470	*L. braziliensis*	242	11	252
LbrM26_V2.1470	*L. braziliensis*	78	388	465
LbrM26_V2.1470	*L. braziliensis*	320	494	813
LbrM26_V2.1470	*L. braziliensis*	44	1009	1052
LbrM26_V2.1480	*L. braziliensis*	51	91	141
LbrM26_V2.1480	*L. braziliensis*	57	451	507
LbrM26_V2.1490	*L. braziliensis*	53	35	87
LbrM26_V2.1490	*L. braziliensis*	53	177	229
LbrM26_V2.1500	*L. braziliensis*	49	1	49
LbrM26_V2.1500	*L. braziliensis*	70	86	155

LbrM26_V2.1500	*L. braziliensis*	44	232	275
LbrM26_V2.1500	*L. braziliensis*	68	422	489
LbrM26_V2.1500	*L. braziliensis*	78	786	863
LbrM26_V2.1510	*L. braziliensis*	53	389	441
LbrM26_V2.1520	*L. braziliensis*	136	1	136
LbrM26_V2.1530	*L. braziliensis*	77	1	77
LbrM26_V2.1530	*L. braziliensis*	40	129	168
LbrM26_V2.1530	*L. braziliensis*	129	185	313
LbrM26_V2.1530	*L. braziliensis*	327	369	695
LbrM26_V2.1530	*L. braziliensis*	215	705	919
LbrM26_V2.1530	*L. braziliensis*	50	1043	1092
LbrM26_V2.1530	*L. braziliensis*	109	1158	1266
LbrM26_V2.1530	*L. braziliensis*	48	1322	1369
LbrM26_V2.1530	*L. braziliensis*	72	1408	1479
LbrM26_V2.1550	*L. braziliensis*	103	1	103
LbrM26_V2.1570	*L. braziliensis*	42	1	42
LbrM26_V2.1580	*L. braziliensis*	167	1	167
LbrM26_V2.1580	*L. braziliensis*	41	196	236
LbrM26_V2.1580	*L. braziliensis*	55	1007	1061
LbrM26_V2.1580	*L. braziliensis*	44	1621	1664
LbrM26_V2.1600	*L. braziliensis*	54	1	54
LbrM26_V2.1610	*L. braziliensis*	50	46	95
LbrM26_V2.1610	*L. braziliensis*	51	175	225
LbrM26_V2.1610	*L. braziliensis*	51	175	225
LbrM26_V2.1620	*L. braziliensis*	163	438	600
LbrM26_V2.1640	*L. braziliensis*	41	18	58
LbrM26_V2.1710	*L. braziliensis*	49	152	200
LbrM26_V2.1710	*L. braziliensis*	98	230	327
LbrM26_V2.1720	*L. braziliensis*	40	1	40
LbrM26_V2.1720	*L. braziliensis*	75	740	814
LbrM26_V2.1720	*L. braziliensis*	51	1111	1161
LbrM26_V2.1720	*L. braziliensis*	42	1221	1262
LbrM26_V2.1720	*L. braziliensis*	45	1480	1524
LbrM26_V2.1720	*L. braziliensis*	42	1687	1728
LbrM26_V2.1720	*L. braziliensis*	60	1889	1948
LbrM26_V2.1730	*L. braziliensis*	50	1	50
LbrM26_V2.1740	*L. braziliensis*	43	97	139
LbrM26_V2.1740	*L. braziliensis*	55	177	231
LbrM26_V2.1740	*L. braziliensis*	242	252	493
LbrM26_V2.1740	*L. braziliensis*	42	548	589
LbrM26_V2.1750	*L. braziliensis*	525	1	525
LbrM26_V2.1750	*L. braziliensis*	55	544	598
LbrM26_V2.1750	*L. braziliensis*	440	627	1066
LbrM26_V2.1750	*L. braziliensis*	291	1089	1379
LbrM26_V2.1750	*L. braziliensis*	150	1416	1565
LbrM26_V2.1750	*L. braziliensis*	80	1632	1711
LbrM26_V2.1750	*L. braziliensis*	58	1782	1839

LbrM26_V2.1750	*L. braziliensis*	51	1984	2034
LbrM26_V2.1750	*L. braziliensis*	52	2104	2155
LbrM26_V2.1750	*L. braziliensis*	92	2187	2278
LbrM26_V2.1750	*L. braziliensis*	45	2310	2354
LbrM26_V2.1760	*L. braziliensis*	77	1	77
LbrM26_V2.1760	*L. braziliensis*	322	117	438
LbrM26_V2.1760	*L. braziliensis*	185	685	869
LbrM26_V2.1770	*L. braziliensis*	74	34	107
LbrM26_V2.1770	*L. braziliensis*	72	124	195
LbrM26_V2.1770	*L. braziliensis*	48	223	270
LbrM26_V2.1770	*L. braziliensis*	52	348	399
LbrM26_V2.1770	*L. braziliensis*	69	437	505
LbrM26_V2.1770	*L. braziliensis*	84	570	653
LbrM26_V2.1790	*L. braziliensis*	43	1	43
LbrM26_V2.1790	*L. braziliensis*	58	281	338
LbrM26_V2.1790	*L. braziliensis*	58	281	338
LbrM26_V2.1800	*L. braziliensis*	281	1	281
LbrM26_V2.1800	*L. braziliensis*	98	283	380
LbrM26_V2.1800	*L. braziliensis*	54	473	526
LbrM26_V2.1800	*L. braziliensis*	60	599	658
LbrM26_V2.1800	*L. braziliensis*	56	699	754
LbrM26_V2.1800	*L. braziliensis*	164	975	1138
LbrM26_V2.1800	*L. braziliensis*	121	1150	1270
LbrM26_V2.1800	*L. braziliensis*	133	1284	1416
LbrM26_V2.1800	*L. braziliensis*	81	1454	1534
LbrM26_V2.1800	*L. braziliensis*	62	1702	1763
LbrM26_V2.1800	*L. braziliensis*	237	1779	2015
LbrM26_V2.1800	*L. braziliensis*	40	2058	2097
LbrM26_V2.1800	*L. braziliensis*	338	2274	2611
LbrM26_V2.1800	*L. braziliensis*	118	2700	2817
LbrM26_V2.1810	*L. braziliensis*	76	63	138
LbrM26_V2.1810	*L. braziliensis*	55	178	232
LbrM26_V2.1810	*L. braziliensis*	112	522	633
LbrM26_V2.1820	*L. braziliensis*	72	67	138
LbrM26_V2.1820	*L. braziliensis*	87	206	292
LbrM26_V2.1820	*L. braziliensis*	87	206	292
LbrM26_V2.1830	*L. braziliensis*	61	151	211
LbrM26_V2.1830	*L. braziliensis*	50	500	549
LbrM26_V2.1830	*L. braziliensis*	67	1055	1121
LbrM26_V2.1840	*L. braziliensis*	74	115	188
LbrM26_V2.1870	*L. braziliensis*	72	60	131
LbrM26_V2.1870	*L. braziliensis*	49	241	289
LbrM26_V2.1870	*L. braziliensis*	75	373	447
LbrM26_V2.1870	*L. braziliensis*	46	1692	1737
LbrM26_V2.1870	*L. braziliensis*	41	2223	2263
LbrM26_V2.1870	*L. braziliensis*	73	2452	2524
LbrM26_V2.1880	*L. braziliensis*	111	1	111

LbrM26_V2.1880	*L. braziliensis*	70	171	240
LbrM26_V2.1880	*L. braziliensis*	49	314	362
LbrM26_V2.1880	*L. braziliensis*	64	503	566
LbrM26_V2.1880	*L. braziliensis*	107	772	878
LbrM26_V2.1890	*L. braziliensis*	67	220	286
LbrM26_V2.1900	*L. braziliensis*	173	1	173
LbrM26_V2.1910	*L. braziliensis*	89	129	217
LbrM26_V2.1920	*L. braziliensis*	45	60	104
LbrM26_V2.1930	*L. braziliensis*	100	173	272
LbrM26_V2.1930	*L. braziliensis*	382	342	723
LbrM26_V2.1950	*L. braziliensis*	48	76	123
LbrM26_V2.1960	*L. braziliensis*	85	94	178
LbrM26_V2.1970	*L. braziliensis*	105	58	162
LbrM26_V2.1970	*L. braziliensis*	131	252	382
LbrM26_V2.1970	*L. braziliensis*	109	424	532
LbrM26_V2.1970	*L. braziliensis*	113	785	897
LbrM26_V2.1980	*L. braziliensis*	81	1	81
LbrM26_V2.1980	*L. braziliensis*	63	197	259
LbrM26_V2.1980	*L. braziliensis*	40	355	394
LbrM26_V2.1980	*L. braziliensis*	217	497	713
LbrM26_V2.1980	*L. braziliensis*	50	818	867
LbrM26_V2.1990	*L. braziliensis*	490	40	529
LbrM26_V2.1990	*L. braziliensis*	300	545	844
LbrM26_V2.1990	*L. braziliensis*	79	1233	1311
LbrM26_V2.2000	*L. braziliensis*	78	1	78
LbrM26_V2.2000	*L. braziliensis*	118	115	232
LbrM26_V2.2020	*L. braziliensis*	77	74	150
LbrM26_V2.2020	*L. braziliensis*	72	178	249
LbrM26_V2.2020	*L. braziliensis*	110	252	361
LbrM26_V2.2020	*L. braziliensis*	96	585	680
LbrM26_V2.2020	*L. braziliensis*	113	751	863
LbrM26_V2.2030	*L. braziliensis*	47	1	47
LbrM26_V2.2030	*L. braziliensis*	147	100	246
LbrM26_V2.2030	*L. braziliensis*	71	270	340
LbrM26_V2.2030	*L. braziliensis*	51	428	478
LbrM26_V2.2030	*L. braziliensis*	104	506	609
LbrM26_V2.2030	*L. braziliensis*	141	665	805
LbrM26_V2.2030	*L. braziliensis*	347	969	1315
LbrM26_V2.2030	*L. braziliensis*	113	1412	1524
LbrM26_V2.2030	*L. braziliensis*	207	1538	1744
LbrM26_V2.2040	*L. braziliensis*	118	32	149
LbrM26_V2.2040	*L. braziliensis*	62	154	215
LbrM26_V2.2040	*L. braziliensis*	122	266	387
LbrM26_V2.2040	*L. braziliensis*	56	396	451
LbrM26_V2.2040	*L. braziliensis*	135	460	594
LbrM26_V2.2040	*L. braziliensis*	245	635	879
LbrM26_V2.2040	*L. braziliensis*	623	1061	1683

LbrM26_V2.2040	*L. braziliensis*	48	1700	1747
LbrM26_V2.2040	*L. braziliensis*	167	1777	1943
LbrM26_V2.2040	*L. braziliensis*	283	1973	2255
LbrM26_V2.2040	*L. braziliensis*	437	2355	2791
LbrM26_V2.2040	*L. braziliensis*	98	2810	2907
LbrM26_V2.2040	*L. braziliensis*	110	2935	3044
LbrM26_V2.2040	*L. braziliensis*	62	3050	3111
LbrM26_V2.2040	*L. braziliensis*	140	3308	3447
LbrM26_V2.2040	*L. braziliensis*	77	3483	3559
LbrM26_V2.2040	*L. braziliensis*	126	3711	3836
LbrM26_V2.2040	*L. braziliensis*	182	3870	4051
LbrM26_V2.2040	*L. braziliensis*	90	4090	4179
LbrM26_V2.2050	*L. braziliensis*	40	1	40
LbrM26_V2.2050	*L. braziliensis*	43	172	214
LbrM26_V2.2050	*L. braziliensis*	43	172	214
LbrM26_V2.2070	*L. braziliensis*	40	295	334
LbrM26_V2.2070	*L. braziliensis*	41	443	483
LbrM26_V2.2070	*L. braziliensis*	41	443	483
LbrM26_V2.2080	*L. braziliensis*	61	1	61
LbrM26_V2.2080	*L. braziliensis*	106	144	249
LbrM26_V2.2080	*L. braziliensis*	61	260	320
LbrM26_V2.2080	*L. braziliensis*	46	347	392
LbrM26_V2.2080	*L. braziliensis*	163	525	687
LbrM26_V2.2080	*L. braziliensis*	227	768	994
LbrM26_V2.2080	*L. braziliensis*	485	996	1480
LbrM26_V2.2080	*L. braziliensis*	352	1491	1842
LbrM26_V2.2080	*L. braziliensis*	64	1858	1921
LbrM26_V2.2080	*L. braziliensis*	46	1933	1978
LbrM26_V2.2080	*L. braziliensis*	184	1995	2178
LbrM26_V2.2090	*L. braziliensis*	77	1	77
LbrM26_V2.2090	*L. braziliensis*	116	153	268
LbrM26_V2.2090	*L. braziliensis*	67	326	392
LbrM26_V2.2090	*L. braziliensis*	101	410	510
LbrM26_V2.2090	*L. braziliensis*	970	530	1499
LbrM26_V2.2090	*L. braziliensis*	120	1805	1924
LbrM26_V2.2100	*L. braziliensis*	356	66	421
LbrM26_V2.2100	*L. braziliensis*	625	497	1121
LbrM26_V2.2100	*L. braziliensis*	45	1166	1210
LbrM26_V2.2100	*L. braziliensis*	92	1263	1354
LbrM26_V2.2100	*L. braziliensis*	164	1376	1539
LbrM26_V2.2110	*L. braziliensis*	65	43	107
LbrM26_V2.2110	*L. braziliensis*	57	308	364
LbrM26_V2.2120	*L. braziliensis*	238	1	238
LbrM26_V2.2130	*L. braziliensis*	63	1	63
LbrM26_V2.2130	*L. braziliensis*	205	133	337
LbrM26_V2.2130	*L. braziliensis*	58	486	543
LbrM26_V2.2130	*L. braziliensis*	119	589	707

LbrM26_V2.2160	*L. braziliensis*	46	1	46
LbrM26_V2.2170	*L. braziliensis*	134	10	143
LbrM26_V2.2170	*L. braziliensis*	142	209	350
LbrM26_V2.2170	*L. braziliensis*	41	375	415
LbrM26_V2.2170	*L. braziliensis*	66	516	581
LbrM26_V2.2170	*L. braziliensis*	88	620	707
LbrM26_V2.2180	*L. braziliensis*	91	444	534
LbrM26_V2.2190	*L. braziliensis*	83	185	267
LbrM26_V2.2210	*L. braziliensis*	123	13	135
LbrM26_V2.2220	*L. braziliensis*	232	193	424
LbrM26_V2.2220	*L. braziliensis*	110	676	785
LbrM26_V2.2220	*L. braziliensis*	80	817	896
LbrM26_V2.2220	*L. braziliensis*	42	971	1012
LbrM26_V2.2220	*L. braziliensis*	75	1053	1127
LbrM26_V2.2230	*L. braziliensis*	43	33	75
LbrM26_V2.2240	*L. braziliensis*	105	22	126
LbrM26_V2.2240	*L. braziliensis*	53	134	186
LbrM26_V2.2240	*L. braziliensis*	64	218	281
LbrM26_V2.2250	*L. braziliensis*	42	219	260
LbrM26_V2.2250	*L. braziliensis*	110	278	387
LbrM26_V2.2250	*L. braziliensis*	176	490	665
LbrM26_V2.2260	*L. braziliensis*	43	1	43
LbrM26_V2.2260	*L. braziliensis*	63	65	127
LbrM26_V2.2260	*L. braziliensis*	63	65	127
LbrM26_V2.2270	*L. braziliensis*	43	1	43
LbrM26_V2.2270	*L. braziliensis*	63	65	127
LbrM26_V2.2270	*L. braziliensis*	63	65	127
LbrM26_V2.2280	*L. braziliensis*	102	1	102
LbrM26_V2.2280	*L. braziliensis*	178	270	447
LbrM26_V2.2280	*L. braziliensis*	88	477	564
LbrM26_V2.2280	*L. braziliensis*	122	583	704
LbrM26_V2.2280	*L. braziliensis*	128	723	850
LbrM26_V2.2290	*L. braziliensis*	49	509	557
LbrM26_V2.2300	*L. braziliensis*	56	110	165
LbrM26_V2.2300	*L. braziliensis*	202	181	382
LbrM26_V2.2300	*L. braziliensis*	76	412	487
LbrM26_V2.2300	*L. braziliensis*	59	535	593
LbrM26_V2.2300	*L. braziliensis*	166	612	777
LbrM26_V2.2300	*L. braziliensis*	71	798	868
LbrM26_V2.2300	*L. braziliensis*	82	945	1026
LbrM26_V2.2300	*L. braziliensis*	49	1206	1254
LbrM26_V2.2300	*L. braziliensis*	65	1377	1441
LbrM26_V2.2300	*L. braziliensis*	60	1779	1838
LbrM26_V2.2300	*L. braziliensis*	51	2234	2284
LbrM26_V2.2300	*L. braziliensis*	44	2332	2375
LbrM26_V2.2300	*L. braziliensis*	42	2381	2422
LbrM26_V2.2300	*L. braziliensis*	164	2452	2615

LbrM26_V2.2300	*L. braziliensis*	136	3416	3551
LbrM26_V2.2310	*L. braziliensis*	41	28	68
LbrM26_V2.2310	*L. braziliensis*	74	103	176
LbrM26_V2.2310	*L. braziliensis*	46	219	264
LbrM26_V2.2310	*L. braziliensis*	55	2006	2060
LbrM26_V2.2310	*L. braziliensis*	49	2321	2369
LbrM26_V2.2310	*L. braziliensis*	105	2590	2694
LbrM26_V2.2310	*L. braziliensis*	75	3567	3641
LbrM26_V2.2310	*L. braziliensis*	44	3834	3877
LbrM26_V2.2310	*L. braziliensis*	60	4175	4234
LbrM26_V2.2310	*L. braziliensis*	42	4460	4501
LbrM26_V2.2310	*L. braziliensis*	58	4523	4580
LbrM26_V2.2310	*L. braziliensis*	66	4595	4660
LbrM26_V2.2310	*L. braziliensis*	46	4837	4882
LbrM26_V2.2310	*L. braziliensis*	177	4964	5140
LbrM26_V2.2320	*L. braziliensis*	215	1	215
LbrM26_V2.2320	*L. braziliensis*	100	374	473
LbrM26_V2.2320	*L. braziliensis*	314	492	805
LbrM26_V2.2340	*L. braziliensis*	80	1	80
LbrM26_V2.2340	*L. braziliensis*	51	169	219
LbrM26_V2.2340	*L. braziliensis*	51	169	219
LbrM26_V2.2350	*L. braziliensis*	93	145	237
LbrM26_V2.2370	*L. braziliensis*	281	1	281
LbrM26_V2.2370	*L. braziliensis*	165	363	527
LbrM26_V2.2370	*L. braziliensis*	76	787	862
LbrM26_V2.2370	*L. braziliensis*	305	898	1202
LbrM26_V2.2370	*L. braziliensis*	76	1206	1281
LbrM26_V2.2380	*L. braziliensis*	68	86	153
LbrM26_V2.2380	*L. braziliensis*	52	170	221
LbrM26_V2.2380	*L. braziliensis*	52	170	221
LbrM26_V2.2390	*L. braziliensis*	66	1	66
LbrM26_V2.2390	*L. braziliensis*	81	117	197
LbrM26_V2.2390	*L. braziliensis*	81	117	197
LbrM26_V2.2420	*L. braziliensis*	114	1	114
LbrM26_V2.2420	*L. braziliensis*	62	118	179
LbrM26_V2.2420	*L. braziliensis*	173	219	391
LbrM26_V2.2420	*L. braziliensis*	61	400	460
LbrM26_V2.2420	*L. braziliensis*	296	474	769
LbrM26_V2.2420	*L. braziliensis*	478	803	1280
LbrM26_V2.2420	*L. braziliensis*	84	1310	1393
LbrM26_V2.2420	*L. braziliensis*	77	1399	1475
LbrM26_V2.2420	*L. braziliensis*	68	1499	1566
LbrM26_V2.2460	*L. braziliensis*	119	74	192
LbrM26_V2.2460	*L. braziliensis*	172	303	474
LbrM26_V2.2470	*L. braziliensis*	65	1	65
LbrM26_V2.2470	*L. braziliensis*	93	424	516
LbrM26_V2.2470	*L. braziliensis*	93	424	516

LbrM26_V2.2480	*L. braziliensis*	106	1	106
LbrM26_V2.2490	*L. braziliensis*	44	473	516
LbrM26_V2.2510	*L. braziliensis*	73	79	151
LbrM26_V2.2520	*L. braziliensis*	57	595	651
LbrM26_V2.2530	*L. braziliensis*	40	38	77
LbrM26_V2.2530	*L. braziliensis*	64	112	175
LbrM26_V2.2530	*L. braziliensis*	40	184	223
LbrM26_V2.2530	*L. braziliensis*	263	437	699
LbrM26_V2.2570	*L. braziliensis*	115	118	232
LbrM26_V2.2570	*L. braziliensis*	77	245	321
LbrM26_V2.2580	*L. braziliensis*	57	114	170
LbrM26_V2.2590	*L. braziliensis*	135	1	135
LbrM26_V2.2590	*L. braziliensis*	527	144	670
LbrM26_V2.2600	*L. braziliensis*	42	632	673
LbrM26_V2.2650	*L. braziliensis*	161	27	187
LbrM26_V2.2650	*L. braziliensis*	111	367	477
LbrM26_V2.2650	*L. braziliensis*	40	694	733
LbrM26_V2.2650	*L. braziliensis*	67	1268	1334
LbrM27_V2.0020	*L. braziliensis*	52	116	167
LbrM27_V2.0060	*L. braziliensis*	64	319	382
LbrM27_V2.0060	*L. braziliensis*	71	620	690
LbrM27_V2.0060	*L. braziliensis*	71	620	690
LbrM27_V2.0090	*L. braziliensis*	52	42	93
LbrM27_V2.0090	*L. braziliensis*	58	270	327
LbrM27_V2.0090	*L. braziliensis*	58	270	327
LbrM27_V2.0110	*L. braziliensis*	333	327	659
LbrM27_V2.0130	*L. braziliensis*	57	353	409
LbrM27_V2.0160	*L. braziliensis*	118	101	218
LbrM27_V2.0180	*L. braziliensis*	45	1	45
LbrM27_V2.0190	*L. braziliensis*	47	133	179
LbrM27_V2.0210	*L. braziliensis*	75	256	330
LbrM27_V2.0220	*L. braziliensis*	55	1	55
LbrM27_V2.0250	*L. braziliensis*	831	1	831
LbrM27_V2.0260	*L. braziliensis*	431	1	431
LbrM27_V2.0270	*L. braziliensis*	73	92	164
LbrM27_V2.0270	*L. braziliensis*	43	353	395
LbrM27_V2.0270	*L. braziliensis*	43	353	395
LbrM27_V2.0290	*L. braziliensis*	75	1	75
LbrM27_V2.0290	*L. braziliensis*	81	126	206
LbrM27_V2.0290	*L. braziliensis*	95	238	332
LbrM27_V2.0290	*L. braziliensis*	51	375	425
LbrM27_V2.0290	*L. braziliensis*	454	429	882
LbrM27_V2.0290	*L. braziliensis*	76	920	995
LbrM27_V2.0290	*L. braziliensis*	100	1013	1112
LbrM27_V2.0320	*L. braziliensis*	42	617	658
LbrM27_V2.0330	*L. braziliensis*	60	435	494
LbrM27_V2.0340	*L. braziliensis*	118	1	118

LbrM27_V2.0340	*L. braziliensis*	146	180	325
LbrM27_V2.0350	*L. braziliensis*	57	23	79
LbrM27_V2.0350	*L. braziliensis*	46	384	429
LbrM27_V2.0350	*L. braziliensis*	46	384	429
LbrM27_V2.0360	*L. braziliensis*	151	350	500
LbrM27_V2.0370	*L. braziliensis*	230	1	230
LbrM27_V2.0370	*L. braziliensis*	98	235	332
LbrM27_V2.0370	*L. braziliensis*	68	403	470
LbrM27_V2.0370	*L. braziliensis*	73	472	544
LbrM27_V2.0380	*L. braziliensis*	287	34	320
LbrM27_V2.0380	*L. braziliensis*	95	561	655
LbrM27_V2.0390	*L. braziliensis*	692	1	692
LbrM27_V2.0390	*L. braziliensis*	184	698	881
LbrM27_V2.0390	*L. braziliensis*	134	922	1055
LbrM27_V2.0400	*L. braziliensis*	144	1	144
LbrM27_V2.0400	*L. braziliensis*	111	154	264
LbrM27_V2.0410	*L. braziliensis*	118	1	118
LbrM27_V2.0410	*L. braziliensis*	65	137	201
LbrM27_V2.0430	*L. braziliensis*	46	1	46
LbrM27_V2.0430	*L. braziliensis*	50	506	555
LbrM27_V2.0440	*L. braziliensis*	45	331	375
LbrM27_V2.0470	*L. braziliensis*	148	18	165
LbrM27_V2.0470	*L. braziliensis*	116	258	373
LbrM27_V2.0480	*L. braziliensis*	56	516	571
LbrM27_V2.0480	*L. braziliensis*	74	614	687
LbrM27_V2.0480	*L. braziliensis*	74	614	687
LbrM27_V2.0490	*L. braziliensis*	55	86	140
LbrM27_V2.0500	*L. braziliensis*	89	101	189
LbrM27_V2.0500	*L. braziliensis*	98	206	303
LbrM27_V2.0500	*L. braziliensis*	98	206	303
LbrM27_V2.0520	*L. braziliensis*	610	1	610
LbrM27_V2.0530	*L. braziliensis*	59	437	495
LbrM27_V2.0540	*L. braziliensis*	116	1	116
LbrM27_V2.0550	*L. braziliensis*	52	1	52
LbrM27_V2.0550	*L. braziliensis*	124	218	341
LbrM27_V2.0550	*L. braziliensis*	146	383	528
LbrM27_V2.0550	*L. braziliensis*	87	544	630
LbrM27_V2.0560	*L. braziliensis*	55	86	140
LbrM27_V2.0570	*L. braziliensis*	71	101	171
LbrM27_V2.0590	*L. braziliensis*	76	355	430
LbrM27_V2.0590	*L. braziliensis*	112	488	599
LbrM27_V2.0600	*L. braziliensis*	60	574	633
LbrM27_V2.0600	*L. braziliensis*	142	1219	1360
LbrM27_V2.0600	*L. braziliensis*	74	1414	1487
LbrM27_V2.0600	*L. braziliensis*	132	1489	1620
LbrM27_V2.0600	*L. braziliensis*	82	1720	1801
LbrM27_V2.0600	*L. braziliensis*	48	1893	1940

LbrM27_V2.0600	*L. braziliensis*	1939	2000	3938
LbrM27_V2.0600	*L. braziliensis*	122	4243	4364
LbrM27_V2.0600	*L. braziliensis*	60	4913	4972
LbrM27_V2.0610	*L. braziliensis*	73	1193	1265
LbrM27_V2.0610	*L. braziliensis*	46	1286	1331
LbrM27_V2.0610	*L. braziliensis*	111	1368	1478
LbrM27_V2.0610	*L. braziliensis*	173	1490	1662
LbrM27_V2.0610	*L. braziliensis*	60	1699	1758
LbrM27_V2.0610	*L. braziliensis*	130	1767	1896
LbrM27_V2.0610	*L. braziliensis*	216	1937	2152
LbrM27_V2.0610	*L. braziliensis*	290	2185	2474
LbrM27_V2.0610	*L. braziliensis*	139	2477	2615
LbrM27_V2.0610	*L. braziliensis*	48	2653	2700
LbrM27_V2.0610	*L. braziliensis*	48	2715	2762
LbrM27_V2.0610	*L. braziliensis*	175	2788	2962
LbrM27_V2.0610	*L. braziliensis*	454	2979	3432
LbrM27_V2.0610	*L. braziliensis*	124	3505	3628
LbrM27_V2.0610	*L. braziliensis*	116	3665	3780
LbrM27_V2.0610	*L. braziliensis*	85	3826	3910
LbrM27_V2.0610	*L. braziliensis*	323	3937	4259
LbrM27_V2.0610	*L. braziliensis*	511	4266	4776
LbrM27_V2.0610	*L. braziliensis*	221	4851	5071
LbrM27_V2.0610	*L. braziliensis*	110	5078	5187
LbrM27_V2.0610	*L. braziliensis*	350	5189	5538
LbrM27_V2.0610	*L. braziliensis*	77	6088	6164
LbrM27_V2.0630	*L. braziliensis*	176	1	176
LbrM27_V2.0630	*L. braziliensis*	81	194	274
LbrM27_V2.0640	*L. braziliensis*	124	131	254
LbrM27_V2.0640	*L. braziliensis*	193	339	531
LbrM27_V2.0680	*L. braziliensis*	176	187	362
LbrM27_V2.0680	*L. braziliensis*	75	382	456
LbrM27_V2.0680	*L. braziliensis*	58	487	544
LbrM27_V2.0690	*L. braziliensis*	53	217	269
LbrM27_V2.0700	*L. braziliensis*	106	42	147
LbrM27_V2.0710	*L. braziliensis*	380	1	380
LbrM27_V2.0710	*L. braziliensis*	87	411	497
LbrM27_V2.0710	*L. braziliensis*	44	526	569
LbrM27_V2.0710	*L. braziliensis*	82	654	735
LbrM27_V2.0710	*L. braziliensis*	216	764	979
LbrM27_V2.0720	*L. braziliensis*	362	86	447
LbrM27_V2.0730	*L. braziliensis*	43	1	43
LbrM27_V2.0740	*L. braziliensis*	46	81	126
LbrM27_V2.0740	*L. braziliensis*	65	544	608
LbrM27_V2.0740	*L. braziliensis*	65	544	608
LbrM27_V2.0750	*L. braziliensis*	51	123	173
LbrM27_V2.0770	*L. braziliensis*	98	1	98
LbrM27_V2.0780	*L. braziliensis*	67	1	67

LbrM27_V2.0780	*L. braziliensis*	48	283	330
LbrM27_V2.0780	*L. braziliensis*	47	366	412
LbrM27_V2.0780	*L. braziliensis*	47	479	525
LbrM27_V2.0780	*L. braziliensis*	41	842	882
LbrM27_V2.0790	*L. braziliensis*	67	1	67
LbrM27_V2.0790	*L. braziliensis*	99	85	183
LbrM27_V2.0790	*L. braziliensis*	115	344	458
LbrM27_V2.0800	*L. braziliensis*	308	67	374
LbrM27_V2.0860	*L. braziliensis*	164	818	981
LbrM27_V2.0860	*L. braziliensis*	55	1019	1073
LbrM27_V2.0860	*L. braziliensis*	84	1221	1304
LbrM27_V2.0870	*L. braziliensis*	45	156	200
LbrM27_V2.0890	*L. braziliensis*	62	964	1025
LbrM27_V2.0890	*L. braziliensis*	48	1290	1337
LbrM27_V2.0890	*L. braziliensis*	42	1673	1714
LbrM27_V2.0890	*L. braziliensis*	66	1820	1885
LbrM27_V2.0890	*L. braziliensis*	54	2262	2315
LbrM27_V2.0890	*L. braziliensis*	40	2404	2443
LbrM27_V2.0890	*L. braziliensis*	57	2775	2831
LbrM27_V2.0890	*L. braziliensis*	69	2854	2922
LbrM27_V2.0890	*L. braziliensis*	154	3100	3253
LbrM27_V2.0900	*L. braziliensis*	88	62	149
LbrM27_V2.0900	*L. braziliensis*	50	211	260
LbrM27_V2.0900	*L. braziliensis*	49	275	323
LbrM27_V2.0920	*L. braziliensis*	75	25	99
LbrM27_V2.0920	*L. braziliensis*	47	354	400
LbrM27_V2.0920	*L. braziliensis*	122	417	538
LbrM27_V2.0940	*L. braziliensis*	50	380	429
LbrM27_V2.0940	*L. braziliensis*	44	621	664
LbrM27_V2.0940	*L. braziliensis*	44	621	664
LbrM27_V2.0960	*L. braziliensis*	60	948	1007
LbrM27_V2.0970	*L. braziliensis*	161	76	236
LbrM27_V2.0970	*L. braziliensis*	84	678	761
LbrM27_V2.0970	*L. braziliensis*	135	978	1112
LbrM27_V2.0970	*L. braziliensis*	96	1208	1303
LbrM27_V2.0970	*L. braziliensis*	74	1438	1511
LbrM27_V2.0990	*L. braziliensis*	40	416	455
LbrM27_V2.1020	*L. braziliensis*	80	513	592
LbrM27_V2.1030	*L. braziliensis*	142	191	332
LbrM27_V2.1030	*L. braziliensis*	248	350	597
LbrM27_V2.1050	*L. braziliensis*	55	35	89
LbrM27_V2.1050	*L. braziliensis*	41	144	184
LbrM27_V2.1050	*L. braziliensis*	41	1497	1537
LbrM27_V2.1050	*L. braziliensis*	62	1818	1879
LbrM27_V2.1060	*L. braziliensis*	54	104	157
LbrM27_V2.1070	*L. braziliensis*	44	1	44
LbrM27_V2.1070	*L. braziliensis*	47	81	127

LbrM27_V2.1070	*L. braziliensis*	60	184	243
LbrM27_V2.1090	*L. braziliensis*	44	1	44
LbrM27_V2.1090	*L. braziliensis*	62	858	919
LbrM27_V2.1090	*L. braziliensis*	62	858	919
LbrM27_V2.1110	*L. braziliensis*	183	1	183
LbrM27_V2.1110	*L. braziliensis*	56	479	534
LbrM27_V2.1110	*L. braziliensis*	44	783	826
LbrM27_V2.1120	*L. braziliensis*	126	1	126
LbrM27_V2.1120	*L. braziliensis*	43	189	231
LbrM27_V2.1120	*L. braziliensis*	78	288	365
LbrM27_V2.1130	*L. braziliensis*	45	1	45
LbrM27_V2.1140	*L. braziliensis*	89	1	89
LbrM27_V2.1160	*L. braziliensis*	44	65	108
LbrM27_V2.1160	*L. braziliensis*	72	213	284
LbrM27_V2.1160	*L. braziliensis*	105	561	665
LbrM27_V2.1160	*L. braziliensis*	49	781	829
LbrM27_V2.1170	*L. braziliensis*	104	1	104
LbrM27_V2.1170	*L. braziliensis*	44	608	651
LbrM27_V2.1170	*L. braziliensis*	45	836	880
LbrM27_V2.1190	*L. braziliensis*	78	150	227
LbrM27_V2.1190	*L. braziliensis*	197	278	474
LbrM27_V2.1190	*L. braziliensis*	185	476	660
LbrM27_V2.1200	*L. braziliensis*	103	1	103
LbrM27_V2.1200	*L. braziliensis*	44	192	235
LbrM27_V2.1210	*L. braziliensis*	88	137	224
LbrM27_V2.1220	*L. braziliensis*	63	1	63
LbrM27_V2.1230	*L. braziliensis*	171	1	171
LbrM27_V2.1230	*L. braziliensis*	109	702	810
LbrM27_V2.1240	*L. braziliensis*	57	33	89
LbrM27_V2.1240	*L. braziliensis*	397	158	554
LbrM27_V2.1250	*L. braziliensis*	40	74	113
LbrM27_V2.1260	*L. braziliensis*	77	59	135
LbrM27_V2.1260	*L. braziliensis*	47	699	745
LbrM27_V2.1270	*L. braziliensis*	48	49	96
LbrM27_V2.1270	*L. braziliensis*	93	153	245
LbrM27_V2.1280	*L. braziliensis*	56	141	196
LbrM27_V2.1290	*L. braziliensis*	112	1	112
LbrM27_V2.1300	*L. braziliensis*	174	42	215
LbrM27_V2.1300	*L. braziliensis*	156	266	421
LbrM27_V2.1310	*L. braziliensis*	149	1	149
LbrM27_V2.1310	*L. braziliensis*	61	449	509
LbrM27_V2.1320	*L. braziliensis*	90	1	90
LbrM27_V2.1320	*L. braziliensis*	157	94	250
LbrM27_V2.1320	*L. braziliensis*	157	94	250
LbrM27_V2.1330	*L. braziliensis*	43	1	43
LbrM27_V2.1330	*L. braziliensis*	76	205	280
LbrM27_V2.1330	*L. braziliensis*	187	332	518

LbrM27_V2.1390	*L. braziliensis*	157	75	231
LbrM27_V2.1400	*L. braziliensis*	152	1	152
LbrM27_V2.1400	*L. braziliensis*	74	238	311
LbrM27_V2.1400	*L. braziliensis*	55	321	375
LbrM27_V2.1420	*L. braziliensis*	65	1	65
LbrM27_V2.1440	*L. braziliensis*	145	34	178
LbrM27_V2.1440	*L. braziliensis*	52	478	529
LbrM27_V2.1450	*L. braziliensis*	48	489	536
LbrM27_V2.1460	*L. braziliensis*	66	193	258
LbrM27_V2.1460	*L. braziliensis*	76	519	594
LbrM27_V2.1480	*L. braziliensis*	42	73	114
LbrM27_V2.1480	*L. braziliensis*	69	305	373
LbrM27_V2.1490	*L. braziliensis*	65	105	169
LbrM27_V2.1490	*L. braziliensis*	143	171	313
LbrM27_V2.1500	*L. braziliensis*	85	17	101
LbrM27_V2.1500	*L. braziliensis*	405	271	675
LbrM27_V2.1510	*L. braziliensis*	183	1	183
LbrM27_V2.1510	*L. braziliensis*	200	198	397
LbrM27_V2.1510	*L. braziliensis*	117	404	520
LbrM27_V2.1510	*L. braziliensis*	740	752	1491
LbrM27_V2.1520	*L. braziliensis*	54	270	323
LbrM27_V2.1530	*L. braziliensis*	54	270	323
LbrM27_V2.1550	*L. braziliensis*	56	108	163
LbrM27_V2.1560	*L. braziliensis*	202	55	256
LbrM27_V2.1560	*L. braziliensis*	43	365	407
LbrM27_V2.1570	*L. braziliensis*	46	380	425
LbrM27_V2.1570	*L. braziliensis*	95	465	559
LbrM27_V2.1570	*L. braziliensis*	44	608	651
LbrM27_V2.1570	*L. braziliensis*	54	811	864
LbrM27_V2.1580	*L. braziliensis*	215	1	215
LbrM27_V2.1590	*L. braziliensis*	47	447	493
LbrM27_V2.1590	*L. braziliensis*	212	644	855
LbrM27_V2.1600	*L. braziliensis*	47	288	334
LbrM27_V2.1610	*L. braziliensis*	77	1	77
LbrM27_V2.1610	*L. braziliensis*	99	131	229
LbrM27_V2.1610	*L. braziliensis*	67	265	331
LbrM27_V2.1610	*L. braziliensis*	123	709	831
LbrM27_V2.1610	*L. braziliensis*	54	852	905
LbrM27_V2.1610	*L. braziliensis*	181	969	1149
LbrM27_V2.1610	*L. braziliensis*	48	1189	1236
LbrM27_V2.1610	*L. braziliensis*	50	1295	1344
LbrM27_V2.1620	*L. braziliensis*	268	148	415
LbrM27_V2.1620	*L. braziliensis*	52	434	485
LbrM27_V2.1620	*L. braziliensis*	89	577	665
LbrM27_V2.1640	*L. braziliensis*	58	1	58
LbrM27_V2.1650	*L. braziliensis*	58	1	58
LbrM27_V2.1660	*L. braziliensis*	45	244	288

LbrM27_V2.1670	*L. braziliensis*	170	312	481
LbrM27_V2.1670	*L. braziliensis*	64	575	638
LbrM27_V2.1670	*L. braziliensis*	64	575	638
LbrM27_V2.1690	*L. braziliensis*	50	85	134
LbrM27_V2.1700	*L. braziliensis*	101	1	101
LbrM27_V2.1700	*L. braziliensis*	41	585	625
LbrM27_V2.1700	*L. braziliensis*	51	839	889
LbrM27_V2.1710	*L. braziliensis*	41	100	140
LbrM27_V2.1710	*L. braziliensis*	112	219	330
LbrM27_V2.1730	*L. braziliensis*	43	78	120
LbrM27_V2.1760	*L. braziliensis*	66	76	141
LbrM27_V2.1760	*L. braziliensis*	73	313	385
LbrM27_V2.1760	*L. braziliensis*	74	719	792
LbrM27_V2.1770	*L. braziliensis*	70	436	505
LbrM27_V2.1770	*L. braziliensis*	89	598	686
LbrM27_V2.1770	*L. braziliensis*	144	736	879
LbrM27_V2.1780	*L. braziliensis*	76	100	175
LbrM27_V2.1780	*L. braziliensis*	110	232	341
LbrM27_V2.1780	*L. braziliensis*	248	356	603
LbrM27_V2.1780	*L. braziliensis*	274	637	910
LbrM27_V2.1780	*L. braziliensis*	88	943	1030
LbrM27_V2.1780	*L. braziliensis*	93	1052	1144
LbrM27_V2.1780	*L. braziliensis*	52	1167	1218
LbrM27_V2.1780	*L. braziliensis*	56	1228	1283
LbrM27_V2.1790	*L. braziliensis*	40	200	239
LbrM27_V2.1820	*L. braziliensis*	87	1	87
LbrM27_V2.1820	*L. braziliensis*	67	294	360
LbrM27_V2.1840	*L. braziliensis*	114	1	114
LbrM27_V2.1860	*L. braziliensis*	76	103	178
LbrM27_V2.1860	*L. braziliensis*	121	219	339
LbrM27_V2.1870	*L. braziliensis*	54	71	124
LbrM27_V2.1870	*L. braziliensis*	74	186	259
LbrM27_V2.1870	*L. braziliensis*	44	667	710
LbrM27_V2.1870	*L. braziliensis*	52	2720	2771
LbrM27_V2.1870	*L. braziliensis*	129	3048	3176
LbrM27_V2.1870	*L. braziliensis*	83	3270	3352
LbrM27_V2.1870	*L. braziliensis*	64	3567	3630
LbrM27_V2.1870	*L. braziliensis*	76	4136	4211
LbrM27_V2.1880	*L. braziliensis*	54	462	515
LbrM27_V2.1900	*L. braziliensis*	95	1	95
LbrM27_V2.1900	*L. braziliensis*	55	144	198
LbrM27_V2.1900	*L. braziliensis*	47	513	559
LbrM27_V2.1910	*L. braziliensis*	427	93	519
LbrM27_V2.1910	*L. braziliensis*	103	544	646
LbrM27_V2.1910	*L. braziliensis*	138	703	840
LbrM27_V2.1960	*L. braziliensis*	88	12	99
LbrM27_V2.1960	*L. braziliensis*	62	159	220

LbrM27_V2.1960	*L. braziliensis*	151	234	384
LbrM27_V2.1960	*L. braziliensis*	243	413	655
LbrM27_V2.1960	*L. braziliensis*	303	657	959
LbrM27_V2.1960	*L. braziliensis*	57	984	1040
LbrM27_V2.1960	*L. braziliensis*	174	1042	1215
LbrM27_V2.1960	*L. braziliensis*	57	1240	1296
LbrM27_V2.1960	*L. braziliensis*	238	1298	1535
LbrM27_V2.1960	*L. braziliensis*	73	1621	1693
LbrM27_V2.1960	*L. braziliensis*	146	1729	1874
LbrM27_V2.1960	*L. braziliensis*	223	1903	2125
LbrM27_V2.1960	*L. braziliensis*	127	2179	2305
LbrM27_V2.1980	*L. braziliensis*	93	20	112
LbrM27_V2.1980	*L. braziliensis*	98	173	270
LbrM27_V2.1980	*L. braziliensis*	291	326	616
LbrM27_V2.1980	*L. braziliensis*	73	839	911
LbrM27_V2.1980	*L. braziliensis*	51	918	968
LbrM27_V2.1980	*L. braziliensis*	179	1096	1274
LbrM27_V2.1990	*L. braziliensis*	119	263	381
LbrM27_V2.1990	*L. braziliensis*	43	566	608
LbrM27_V2.1990	*L. braziliensis*	57	639	695
LbrM27_V2.2000	*L. braziliensis*	59	1	59
LbrM27_V2.2000	*L. braziliensis*	202	72	273
LbrM27_V2.2000	*L. braziliensis*	71	335	405
LbrM27_V2.2000	*L. braziliensis*	100	520	619
LbrM27_V2.2000	*L. braziliensis*	282	621	902
LbrM27_V2.2020	*L. braziliensis*	56	122	177
LbrM27_V2.2020	*L. braziliensis*	44	188	231
LbrM27_V2.2020	*L. braziliensis*	74	444	517
LbrM27_V2.2030	*L. braziliensis*	76	517	592
LbrM27_V2.2030	*L. braziliensis*	270	594	863
LbrM27_V2.2040	*L. braziliensis*	65	83	147
LbrM27_V2.2040	*L. braziliensis*	135	216	350
LbrM27_V2.2040	*L. braziliensis*	49	707	755
LbrM27_V2.2050	*L. braziliensis*	57	1	57
LbrM27_V2.2060	*L. braziliensis*	46	68	113
LbrM27_V2.2070	*L. braziliensis*	72	1	72
LbrM27_V2.2070	*L. braziliensis*	142	87	228
LbrM27_V2.2070	*L. braziliensis*	43	259	301
LbrM27_V2.2070	*L. braziliensis*	132	415	546
LbrM27_V2.2070	*L. braziliensis*	58	590	647
LbrM27_V2.2070	*L. braziliensis*	104	995	1098
LbrM27_V2.2070	*L. braziliensis*	52	1195	1246
LbrM27_V2.2080	*L. braziliensis*	50	303	352
LbrM27_V2.2090	*L. braziliensis*	198	30	227
LbrM27_V2.2100	*L. braziliensis*	96	1	96
LbrM27_V2.2100	*L. braziliensis*	109	136	244
LbrM27_V2.2100	*L. braziliensis*	118	368	485

LbrM27_V2.2100	*L. braziliensis*	167	793	959
LbrM27_V2.2110	*L. braziliensis*	69	56	124
LbrM27_V2.2110	*L. braziliensis*	82	200	281
LbrM27_V2.2110	*L. braziliensis*	119	564	682
LbrM27_V2.2110	*L. braziliensis*	95	692	786
LbrM27_V2.2110	*L. braziliensis*	106	816	921
LbrM27_V2.2110	*L. braziliensis*	106	967	1072
LbrM27_V2.2110	*L. braziliensis*	41	1097	1137
LbrM27_V2.2110	*L. braziliensis*	148	1297	1444
LbrM27_V2.2120	*L. braziliensis*	154	1	154
LbrM27_V2.2120	*L. braziliensis*	179	260	438
LbrM27_V2.2140	*L. braziliensis*	76	905	980
LbrM27_V2.2140	*L. braziliensis*	54	1057	1110
LbrM27_V2.2140	*L. braziliensis*	87	1162	1248
LbrM27_V2.2140	*L. braziliensis*	96	1268	1363
LbrM27_V2.2140	*L. braziliensis*	123	1398	1520
LbrM27_V2.2140	*L. braziliensis*	153	1537	1689
LbrM27_V2.2140	*L. braziliensis*	124	1738	1861
LbrM27_V2.2140	*L. braziliensis*	153	1877	2029
LbrM27_V2.2140	*L. braziliensis*	124	2078	2201
LbrM27_V2.2140	*L. braziliensis*	153	2217	2369
LbrM27_V2.2140	*L. braziliensis*	40	2418	2457
LbrM27_V2.2140	*L. braziliensis*	107	2755	2861
LbrM27_V2.2140	*L. braziliensis*	40	2903	2942
LbrM27_V2.2140	*L. braziliensis*	76	3083	3158
LbrM27_V2.2140	*L. braziliensis*	202	3518	3719
LbrM27_V2.2140	*L. braziliensis*	40	3914	3953
LbrM27_V2.2140	*L. braziliensis*	92	4195	4286
LbrM27_V2.2140	*L. braziliensis*	121	4396	4516
LbrM27_V2.2140	*L. braziliensis*	82	4530	4611
LbrM27_V2.2140	*L. braziliensis*	64	4817	4880
LbrM27_V2.2140	*L. braziliensis*	101	4945	5045
LbrM27_V2.2140	*L. braziliensis*	128	5200	5327
LbrM27_V2.2140	*L. braziliensis*	182	5332	5513
LbrM27_V2.2150	*L. braziliensis*	112	24	135
LbrM27_V2.2160	*L. braziliensis*	153	318	470
LbrM27_V2.2160	*L. braziliensis*	65	477	541
LbrM27_V2.2160	*L. braziliensis*	214	560	773
LbrM27_V2.2160	*L. braziliensis*	99	829	927
LbrM27_V2.2170	*L. braziliensis*	57	1	57
LbrM27_V2.2170	*L. braziliensis*	184	105	288
LbrM27_V2.2170	*L. braziliensis*	106	306	411
LbrM27_V2.2170	*L. braziliensis*	89	471	559
LbrM27_V2.2170	*L. braziliensis*	111	639	749
LbrM27_V2.2170	*L. braziliensis*	53	923	975
LbrM27_V2.2190	*L. braziliensis*	303	94	396
LbrM27_V2.2190	*L. braziliensis*	52	434	485

LbrM27_V2.2220	*L. braziliensis*	49	180	228
LbrM27_V2.2230	*L. braziliensis*	43	1	43
LbrM27_V2.2240	*L. braziliensis*	50	74	123
LbrM27_V2.2240	*L. braziliensis*	55	204	258
LbrM27_V2.2240	*L. braziliensis*	101	285	385
LbrM27_V2.2250	*L. braziliensis*	282	257	538
LbrM27_V2.2280	*L. braziliensis*	55	89	143
LbrM27_V2.2280	*L. braziliensis*	45	202	246
LbrM27_V2.2280	*L. braziliensis*	100	319	418
LbrM27_V2.2290	*L. braziliensis*	81	1	81
LbrM27_V2.2290	*L. braziliensis*	42	111	152
LbrM27_V2.2290	*L. braziliensis*	72	179	250
LbrM27_V2.2290	*L. braziliensis*	41	288	328
LbrM27_V2.2290	*L. braziliensis*	100	642	741
LbrM27_V2.2290	*L. braziliensis*	87	871	957
LbrM27_V2.2290	*L. braziliensis*	92	964	1055
LbrM27_V2.2300	*L. braziliensis*	118	174	291
LbrM27_V2.2310	*L. braziliensis*	281	124	404
LbrM27_V2.2310	*L. braziliensis*	65	462	526
LbrM27_V2.2310	*L. braziliensis*	56	649	704
LbrM27_V2.2310	*L. braziliensis*	208	1180	1387
LbrM27_V2.2310	*L. braziliensis*	54	1454	1507
LbrM27_V2.2320	*L. braziliensis*	75	1	75
LbrM27_V2.2320	*L. braziliensis*	146	117	262
LbrM27_V2.2320	*L. braziliensis*	75	390	464
LbrM27_V2.2320	*L. braziliensis*	44	597	640
LbrM27_V2.2320	*L. braziliensis*	60	718	777
LbrM27_V2.2350	*L. braziliensis*	48	160	207
LbrM27_V2.2350	*L. braziliensis*	111	393	503
LbrM27_V2.2350	*L. braziliensis*	210	964	1173
LbrM27_V2.2350	*L. braziliensis*	142	1214	1355
LbrM27_V2.2350	*L. braziliensis*	208	1383	1590
LbrM27_V2.2380	*L. braziliensis*	124	1	124
LbrM27_V2.2390	*L. braziliensis*	58	44	101
LbrM27_V2.2400	*L. braziliensis*	66	200	265
LbrM27_V2.2410	*L. braziliensis*	62	54	115
LbrM27_V2.2410	*L. braziliensis*	44	197	240
LbrM27_V2.2410	*L. braziliensis*	73	385	457
LbrM27_V2.2410	*L. braziliensis*	80	632	711
LbrM27_V2.2410	*L. braziliensis*	96	893	988
LbrM27_V2.2410	*L. braziliensis*	56	1011	1066
LbrM27_V2.2410	*L. braziliensis*	46	1862	1907
LbrM27_V2.2410	*L. braziliensis*	92	1970	2061
LbrM27_V2.2410	*L. braziliensis*	42	2164	2205
LbrM27_V2.2420	*L. braziliensis*	121	199	319
LbrM27_V2.2430	*L. braziliensis*	51	196	246
LbrM27_V2.2440	*L. braziliensis*	67	211	277

LbrM27_V2.2440	*L. braziliensis*	51	314	364
LbrM27_V2.2440	*L. braziliensis*	44	453	496
LbrM27_V2.2440	*L. braziliensis*	58	679	736
LbrM27_V2.2440	*L. braziliensis*	62	755	816
LbrM27_V2.2440	*L. braziliensis*	107	903	1009
LbrM27_V2.2440	*L. braziliensis*	51	1016	1066
LbrM27_V2.2440	*L. braziliensis*	47	1078	1124
LbrM27_V2.2450	*L. braziliensis*	459	48	506
LbrM27_V2.2450	*L. braziliensis*	125	545	669
LbrM27_V2.2470	*L. braziliensis*	58	662	719
LbrM27_V2.2480	*L. braziliensis*	163	4	166
LbrM27_V2.2480	*L. braziliensis*	51	643	693
LbrM27_V2.2500	*L. braziliensis*	69	52	120
LbrM27_V2.2530	*L. braziliensis*	106	54	159
LbrM27_V2.2560	*L. braziliensis*	42	104	145
LbrM27_V2.2570	*L. braziliensis*	48	162	209
LbrM27_V2.2570	*L. braziliensis*	71	315	385
LbrM27_V2.2570	*L. braziliensis*	71	315	385
LbrM27_V2.2600	*L. braziliensis*	95	1	95
LbrM27_V2.2600	*L. braziliensis*	202	188	389
LbrM27_V2.2610	*L. braziliensis*	74	33	106
LbrM27_V2.2610	*L. braziliensis*	82	150	231
LbrM27_V2.2610	*L. braziliensis*	57	340	396
LbrM27_V2.2630	*L. braziliensis*	46	1	46
LbrM27_V2.2630	*L. braziliensis*	50	204	253
LbrM27_V2.2630	*L. braziliensis*	50	204	253
LbrM27_V2.2640	*L. braziliensis*	93	52	144
LbrM27_V2.2640	*L. braziliensis*	339	212	550
LbrM27_V2.2660	*L. braziliensis*	74	183	256
LbrM27_V2.2660	*L. braziliensis*	45	386	430
LbrM27_V2.2660	*L. braziliensis*	45	386	430
LbrM27_V2.2680	*L. braziliensis*	86	459	544
LbrM27_V2.2690	*L. braziliensis*	105	124	228
LbrM27_V2.2700	*L. braziliensis*	61	411	471
LbrM27_V2.2710	*L. braziliensis*	100	1	100
LbrM27_V2.2710	*L. braziliensis*	60	470	529
LbrM27_V2.2710	*L. braziliensis*	125	2747	2871
LbrM27_V2.2710	*L. braziliensis*	75	3294	3368
LbrM27_V2.2720	*L. braziliensis*	100	300	399
LbrM27_V2.2720	*L. braziliensis*	53	526	578
LbrM27_V2.2730	*L. braziliensis*	117	1	117
LbrM27_V2.2730	*L. braziliensis*	83	166	248
LbrM27_V2.2730	*L. braziliensis*	84	324	407
LbrM27_V2.2740	*L. braziliensis*	254	69	322
LbrM27_V2.2740	*L. braziliensis*	82	346	427
LbrM27_V2.2740	*L. braziliensis*	67	589	655
LbrM27_V2.2740	*L. braziliensis*	202	727	928

LbrM27_V2.2750	*L. braziliensis*	51	46	96
LbrM27_V2.2750	*L. braziliensis*	74	335	408
LbrM27_V2.2750	*L. braziliensis*	74	335	408
LbrM27_V2.2760	*L. braziliensis*	128	134	261
LbrM27_V2.2780	*L. braziliensis*	46	46	91
LbrM27_V2.2790	*L. braziliensis*	61	1	61
LbrM27_V2.2790	*L. braziliensis*	105	66	170
LbrM27_V2.2790	*L. braziliensis*	93	176	268
LbrM27_V2.2790	*L. braziliensis*	119	300	418
LbrM27_V2.2790	*L. braziliensis*	51	464	514
LbrM27_V2.2810	*L. braziliensis*	349	39	387
LbrM27_V2.2830	*L. braziliensis*	599	39	637
LbrM27_V2.2860	*L. braziliensis*	515	324	838
LbrM27_V2.2860	*L. braziliensis*	795	859	1653
LbrM27_V2.2860	*L. braziliensis*	110	1668	1777
LbrM28_V2.0010	*L. braziliensis*	49	1	49
LbrM28_V2.0010	*L. braziliensis*	53	192	244
LbrM28_V2.0010	*L. braziliensis*	95	264	358
LbrM28_V2.0010	*L. braziliensis*	173	571	743
LbrM28_V2.0010	*L. braziliensis*	112	922	1033
LbrM28_V2.0010	*L. braziliensis*	58	1824	1881
LbrM28_V2.0020	*L. braziliensis*	40	509	548
LbrM28_V2.0050	*L. braziliensis*	166	25	190
LbrM28_V2.0050	*L. braziliensis*	66	223	288
LbrM28_V2.0050	*L. braziliensis*	66	223	288
LbrM28_V2.0060	*L. braziliensis*	57	1	57
LbrM28_V2.0060	*L. braziliensis*	40	91	130
LbrM28_V2.0060	*L. braziliensis*	255	149	403
LbrM28_V2.0070	*L. braziliensis*	59	10	68
LbrM28_V2.0090	*L. braziliensis*	72	220	291
LbrM28_V2.0150	*L. braziliensis*	42	1327	1368
LbrM28_V2.0170	*L. braziliensis*	82	1	82
LbrM28_V2.0180	*L. braziliensis*	103	1	103
LbrM28_V2.0200	*L. braziliensis*	50	1	50
LbrM28_V2.0200	*L. braziliensis*	133	176	308
LbrM28_V2.0200	*L. braziliensis*	73	346	418
LbrM28_V2.0200	*L. braziliensis*	95	422	516
LbrM28_V2.0210	*L. braziliensis*	48	84	131
LbrM28_V2.0220	*L. braziliensis*	45	1	45
LbrM28_V2.0230	*L. braziliensis*	51	307	357
LbrM28_V2.0240	*L. braziliensis*	175	79	253
LbrM28_V2.0270	*L. braziliensis*	41	122	162
LbrM28_V2.0280	*L. braziliensis*	43	173	215
LbrM28_V2.0290	*L. braziliensis*	68	79	146
LbrM28_V2.0300	*L. braziliensis*	43	141	183
LbrM28_V2.0310	*L. braziliensis*	158	1	158
LbrM28_V2.0310	*L. braziliensis*	120	326	445

LbrM28_V2.0310	*L. braziliensis*	52	512	563
LbrM28_V2.0310	*L. braziliensis*	67	596	662
LbrM28_V2.0320	*L. braziliensis*	49	1	49
LbrM28_V2.0340	*L. braziliensis*	58	240	297
LbrM28_V2.0340	*L. braziliensis*	57	864	920
LbrM28_V2.0340	*L. braziliensis*	57	864	920
LbrM28_V2.0390	*L. braziliensis*	48	117	164
LbrM28_V2.0400	*L. braziliensis*	83	1	83
LbrM28_V2.0400	*L. braziliensis*	50	93	142
LbrM28_V2.0400	*L. braziliensis*	53	237	289
LbrM28_V2.0400	*L. braziliensis*	74	308	381
LbrM28_V2.0400	*L. braziliensis*	99	427	525
LbrM28_V2.0400	*L. braziliensis*	81	588	668
LbrM28_V2.0400	*L. braziliensis*	59	723	781
LbrM28_V2.0400	*L. braziliensis*	103	828	930
LbrM28_V2.0410	*L. braziliensis*	139	81	219
LbrM28_V2.0420	*L. braziliensis*	40	35	74
LbrM28_V2.0420	*L. braziliensis*	74	284	357
LbrM28_V2.0420	*L. braziliensis*	74	284	357
LbrM28_V2.0450	*L. braziliensis*	44	246	289
LbrM28_V2.0460	*L. braziliensis*	50	809	858
LbrM28_V2.0470	*L. braziliensis*	96	1	96
LbrM28_V2.0470	*L. braziliensis*	74	238	311
LbrM28_V2.0470	*L. braziliensis*	74	238	311
LbrM28_V2.0480	*L. braziliensis*	55	708	762
LbrM28_V2.0490	*L. braziliensis*	44	165	208
LbrM28_V2.0490	*L. braziliensis*	219	297	515
LbrM28_V2.0500	*L. braziliensis*	60	48	107
LbrM28_V2.0500	*L. braziliensis*	81	140	220
LbrM28_V2.0500	*L. braziliensis*	81	140	220
LbrM28_V2.0520	*L. braziliensis*	80	177	256
LbrM28_V2.0550	*L. braziliensis*	151	198	348
LbrM28_V2.0550	*L. braziliensis*	75	477	551
LbrM28_V2.0550	*L. braziliensis*	92	583	674
LbrM28_V2.0550	*L. braziliensis*	291	817	1107
LbrM28_V2.0570	*L. braziliensis*	51	1	51
LbrM28_V2.0580	*L. braziliensis*	64	239	302
LbrM28_V2.0590	*L. braziliensis*	54	164	217
LbrM28_V2.0600	*L. braziliensis*	67	146	212
LbrM28_V2.0600	*L. braziliensis*	40	295	334
LbrM28_V2.0600	*L. braziliensis*	177	397	573
LbrM28_V2.0600	*L. braziliensis*	428	575	1002
LbrM28_V2.0600	*L. braziliensis*	226	1020	1245
LbrM28_V2.0600	*L. braziliensis*	210	1257	1466
LbrM28_V2.0600	*L. braziliensis*	69	1476	1544
LbrM28_V2.0610	*L. braziliensis*	261	89	349
LbrM28_V2.0610	*L. braziliensis*	142	382	523

LbrM28_V2.0630	*L. braziliensis*	95	32	126
LbrM28_V2.0630	*L. braziliensis*	40	340	379
LbrM28_V2.0630	*L. braziliensis*	106	2794	2899
LbrM28_V2.0630	*L. braziliensis*	82	3331	3412
LbrM28_V2.0640	*L. braziliensis*	155	298	452
LbrM28_V2.0700	*L. braziliensis*	88	146	233
LbrM28_V2.0730	*L. braziliensis*	50	173	222
LbrM28_V2.0730	*L. braziliensis*	56	258	313
LbrM28_V2.0730	*L. braziliensis*	56	258	313
LbrM28_V2.0750	*L. braziliensis*	107	1	107
LbrM28_V2.0750	*L. braziliensis*	287	265	551
LbrM28_V2.0770	*L. braziliensis*	54	65	118
LbrM28_V2.0770	*L. braziliensis*	127	172	298
LbrM28_V2.0770	*L. braziliensis*	43	348	390
LbrM28_V2.0790	*L. braziliensis*	68	669	736
LbrM28_V2.0810	*L. braziliensis*	44	53	96
LbrM28_V2.0820	*L. braziliensis*	65	374	438
LbrM28_V2.0860	*L. braziliensis*	77	39	115
LbrM28_V2.0860	*L. braziliensis*	119	200	318
LbrM28_V2.0860	*L. braziliensis*	88	336	423
LbrM28_V2.0860	*L. braziliensis*	75	429	503
LbrM28_V2.0880	*L. braziliensis*	77	67	143
LbrM28_V2.0890	*L. braziliensis*	80	27	106
LbrM28_V2.0900	*L. braziliensis*	75	49	123
LbrM28_V2.0920	*L. braziliensis*	187	1	187
LbrM28_V2.0920	*L. braziliensis*	71	709	779
LbrM28_V2.0930	*L. braziliensis*	314	55	368
LbrM28_V2.0940	*L. braziliensis*	111	98	208
LbrM28_V2.0970	*L. braziliensis*	216	57	272
LbrM28_V2.0970	*L. braziliensis*	44	279	322
LbrM28_V2.0990	*L. braziliensis*	43	147	189
LbrM28_V2.1000	*L. braziliensis*	77	335	411
LbrM28_V2.1010	*L. braziliensis*	116	91	206
LbrM28_V2.1010	*L. braziliensis*	69	225	293
LbrM28_V2.1030	*L. braziliensis*	49	96	144
LbrM28_V2.1040	*L. braziliensis*	47	1	47
LbrM28_V2.1070	*L. braziliensis*	97	207	303
LbrM28_V2.1070	*L. braziliensis*	51	746	796
LbrM28_V2.1070	*L. braziliensis*	46	989	1034
LbrM28_V2.1090	*L. braziliensis*	85	161	245
LbrM28_V2.1090	*L. braziliensis*	40	337	376
LbrM28_V2.1110	*L. braziliensis*	176	29	204
LbrM28_V2.1110	*L. braziliensis*	92	263	354
LbrM28_V2.1110	*L. braziliensis*	48	410	457
LbrM28_V2.1110	*L. braziliensis*	113	473	585
LbrM28_V2.1110	*L. braziliensis*	63	623	685
LbrM28_V2.1110	*L. braziliensis*	190	694	883

LbrM28_V2.1120	*L. braziliensis*	71	1	71
LbrM28_V2.1120	*L. braziliensis*	45	107	151
LbrM28_V2.1130	*L. braziliensis*	407	1	407
LbrM28_V2.1140	*L. braziliensis*	56	38	93
LbrM28_V2.1140	*L. braziliensis*	217	127	343
LbrM28_V2.1140	*L. braziliensis*	126	352	477
LbrM28_V2.1140	*L. braziliensis*	48	629	676
LbrM28_V2.1140	*L. braziliensis*	60	866	925
LbrM28_V2.1140	*L. braziliensis*	41	950	990
LbrM28_V2.1140	*L. braziliensis*	84	1235	1318
LbrM28_V2.1140	*L. braziliensis*	161	1394	1554
LbrM28_V2.1150	*L. braziliensis*	77	1	77
LbrM28_V2.1150	*L. braziliensis*	54	96	149
LbrM28_V2.1150	*L. braziliensis*	56	553	608
LbrM28_V2.1150	*L. braziliensis*	91	641	731
LbrM28_V2.1160	*L. braziliensis*	57	431	487
LbrM28_V2.1160	*L. braziliensis*	61	610	670
LbrM28_V2.1160	*L. braziliensis*	50	687	736
LbrM28_V2.1180	*L. braziliensis*	186	200	385
LbrM28_V2.1180	*L. braziliensis*	52	492	543
LbrM28_V2.1180	*L. braziliensis*	51	717	767
LbrM28_V2.1180	*L. braziliensis*	56	796	851
LbrM28_V2.1200	*L. braziliensis*	49	1	49
LbrM28_V2.1210	*L. braziliensis*	81	1	81
LbrM28_V2.1230	*L. braziliensis*	54	248	301
LbrM28_V2.1230	*L. braziliensis*	46	520	565
LbrM28_V2.1230	*L. braziliensis*	46	520	565
LbrM28_V2.1240	*L. braziliensis*	48	55	102
LbrM28_V2.1240	*L. braziliensis*	854	461	1314
LbrM28_V2.1240	*L. braziliensis*	166	1323	1488
LbrM28_V2.1260	*L. braziliensis*	78	808	885
LbrM28_V2.1270	*L. braziliensis*	68	33	100
LbrM28_V2.1280	*L. braziliensis*	84	46	129
LbrM28_V2.1300	*L. braziliensis*	43	278	320
LbrM28_V2.1300	*L. braziliensis*	115	515	629
LbrM28_V2.1310	*L. braziliensis*	49	1	49
LbrM28_V2.1310	*L. braziliensis*	89	65	153
LbrM28_V2.1310	*L. braziliensis*	89	180	268
LbrM28_V2.1310	*L. braziliensis*	356	299	654
LbrM28_V2.1320	*L. braziliensis*	162	162	323
LbrM28_V2.1320	*L. braziliensis*	65	406	470
LbrM28_V2.1340	*L. braziliensis*	42	29	70
LbrM28_V2.1350	*L. braziliensis*	107	119	225
LbrM28_V2.1350	*L. braziliensis*	126	314	439
LbrM28_V2.1360	*L. braziliensis*	110	1	110
LbrM28_V2.1360	*L. braziliensis*	92	170	261
LbrM28_V2.1360	*L. braziliensis*	41	276	316

LbrM28_V2.1360	*L. braziliensis*	53	463	515
LbrM28_V2.1360	*L. braziliensis*	62	750	811
LbrM28_V2.1360	*L. braziliensis*	143	975	1117
LbrM28_V2.1370	*L. braziliensis*	92	103	194
LbrM28_V2.1370	*L. braziliensis*	48	301	348
LbrM28_V2.1380	*L. braziliensis*	55	1	55
LbrM28_V2.1390	*L. braziliensis*	55	251	305
LbrM28_V2.1390	*L. braziliensis*	50	340	389
LbrM28_V2.1400	*L. braziliensis*	112	1	112
LbrM28_V2.1410	*L. braziliensis*	76	1	76
LbrM28_V2.1410	*L. braziliensis*	360	538	897
LbrM28_V2.1430	*L. braziliensis*	57	1	57
LbrM28_V2.1430	*L. braziliensis*	102	71	172
LbrM28_V2.1440	*L. braziliensis*	64	23	86
LbrM28_V2.1450	*L. braziliensis*	109	47	155
LbrM28_V2.1450	*L. braziliensis*	89	193	281
LbrM28_V2.1450	*L. braziliensis*	179	351	529
LbrM28_V2.1450	*L. braziliensis*	59	898	956
LbrM28_V2.1460	*L. braziliensis*	47	345	391
LbrM28_V2.1460	*L. braziliensis*	43	734	776
LbrM28_V2.1460	*L. braziliensis*	43	734	776
LbrM28_V2.1470	*L. braziliensis*	120	1	120
LbrM28_V2.1470	*L. braziliensis*	250	152	401
LbrM28_V2.1470	*L. braziliensis*	291	415	705
LbrM28_V2.1470	*L. braziliensis*	70	973	1042
LbrM28_V2.1540	*L. braziliensis*	106	1	106
LbrM28_V2.1550	*L. braziliensis*	200	82	281
LbrM28_V2.1560	*L. braziliensis*	332	66	397
LbrM28_V2.1570	*L. braziliensis*	128	1	128
LbrM28_V2.1570	*L. braziliensis*	134	621	754
LbrM28_V2.1580	*L. braziliensis*	126	1	126
LbrM28_V2.1580	*L. braziliensis*	64	131	194
LbrM28_V2.1590	*L. braziliensis*	78	61	138
LbrM28_V2.1600	*L. braziliensis*	54	1	54
LbrM28_V2.1600	*L. braziliensis*	73	287	359
LbrM28_V2.1600	*L. braziliensis*	73	287	359
LbrM28_V2.1610	*L. braziliensis*	90	17	106
LbrM28_V2.1610	*L. braziliensis*	43	147	189
LbrM28_V2.1610	*L. braziliensis*	50	248	297
LbrM28_V2.1610	*L. braziliensis*	44	436	479
LbrM28_V2.1610	*L. braziliensis*	60	511	570
LbrM28_V2.1620	*L. braziliensis*	47	338	384
LbrM28_V2.1630	*L. braziliensis*	62	1	62
LbrM28_V2.1640	*L. braziliensis*	62	1	62
LbrM28_V2.1680	*L. braziliensis*	166	1	166
LbrM28_V2.1690	*L. braziliensis*	67	192	258
LbrM28_V2.1690	*L. braziliensis*	344	299	642

LbrM28_V2.1710	*L. braziliensis*	53	1	53
LbrM28_V2.1710	*L. braziliensis*	199	61	259
LbrM28_V2.1710	*L. braziliensis*	43	299	341
LbrM28_V2.1710	*L. braziliensis*	103	560	662
LbrM28_V2.1710	*L. braziliensis*	56	796	851
LbrM28_V2.1720	*L. braziliensis*	49	152	200
LbrM28_V2.1730	*L. braziliensis*	126	85	210
LbrM28_V2.1730	*L. braziliensis*	101	246	346
LbrM28_V2.1730	*L. braziliensis*	137	423	559
LbrM28_V2.1770	*L. braziliensis*	46	1	46
LbrM28_V2.1770	*L. braziliensis*	76	85	160
LbrM28_V2.1770	*L. braziliensis*	48	463	510
LbrM28_V2.1770	*L. braziliensis*	102	958	1059
LbrM28_V2.1780	*L. braziliensis*	118	1	118
LbrM28_V2.1780	*L. braziliensis*	164	166	329
LbrM28_V2.1790	*L. braziliensis*	199	47	245
LbrM28_V2.1790	*L. braziliensis*	58	792	849
LbrM28_V2.1810	*L. braziliensis*	86	247	332
LbrM28_V2.1810	*L. braziliensis*	42	436	477
LbrM28_V2.1810	*L. braziliensis*	74	541	614
LbrM28_V2.1820	*L. braziliensis*	52	161	212
LbrM28_V2.1820	*L. braziliensis*	74	330	403
LbrM28_V2.1820	*L. braziliensis*	80	446	525
LbrM28_V2.1820	*L. braziliensis*	48	751	798
LbrM28_V2.1820	*L. braziliensis*	183	805	987
LbrM28_V2.1820	*L. braziliensis*	285	1017	1301
LbrM28_V2.1830	*L. braziliensis*	520	225	744
LbrM28_V2.1840	*L. braziliensis*	173	31	203
LbrM28_V2.1840	*L. braziliensis*	41	266	306
LbrM28_V2.1850	*L. braziliensis*	50	14	63
LbrM28_V2.1850	*L. braziliensis*	44	65	108
LbrM28_V2.1850	*L. braziliensis*	152	264	415
LbrM28_V2.1850	*L. braziliensis*	138	492	629
LbrM28_V2.1850	*L. braziliensis*	54	980	1033
LbrM28_V2.1850	*L. braziliensis*	134	1180	1313
LbrM28_V2.1870	*L. braziliensis*	107	1	107
LbrM28_V2.1880	*L. braziliensis*	78	49	126
LbrM28_V2.1880	*L. braziliensis*	116	160	275
LbrM28_V2.1880	*L. braziliensis*	45	287	331
LbrM28_V2.1880	*L. braziliensis*	42	341	382
LbrM28_V2.1880	*L. braziliensis*	138	403	540
LbrM28_V2.1880	*L. braziliensis*	133	561	693
LbrM28_V2.1880	*L. braziliensis*	41	787	827
LbrM28_V2.1880	*L. braziliensis*	203	879	1081
LbrM28_V2.1880	*L. braziliensis*	375	1138	1512
LbrM28_V2.1880	*L. braziliensis*	86	1605	1690
LbrM28_V2.1880	*L. braziliensis*	118	1692	1809

LbrM28_V2.1880	*L. braziliensis*	90	2019	2108
LbrM28_V2.1890	*L. braziliensis*	260	32	291
LbrM28_V2.1890	*L. braziliensis*	81	323	403
LbrM28_V2.1890	*L. braziliensis*	49	416	464
LbrM28_V2.1890	*L. braziliensis*	52	555	606
LbrM28_V2.1900	*L. braziliensis*	54	288	341
LbrM28_V2.1900	*L. braziliensis*	105	823	927
LbrM28_V2.1910	*L. braziliensis*	115	116	230
LbrM28_V2.1910	*L. braziliensis*	81	402	482
LbrM28_V2.1920	*L. braziliensis*	84	55	138
LbrM28_V2.1920	*L. braziliensis*	117	190	306
LbrM28_V2.1920	*L. braziliensis*	57	388	444
LbrM28_V2.1920	*L. braziliensis*	111	520	630
LbrM28_V2.1920	*L. braziliensis*	56	652	707
LbrM28_V2.1920	*L. braziliensis*	85	791	875
LbrM28_V2.1930	*L. braziliensis*	70	84	153
LbrM28_V2.1930	*L. braziliensis*	93	190	282
LbrM28_V2.1930	*L. braziliensis*	143	899	1041
LbrM28_V2.1930	*L. braziliensis*	207	1341	1547
LbrM28_V2.1930	*L. braziliensis*	43	1968	2010
LbrM28_V2.1940	*L. braziliensis*	59	85	143
LbrM28_V2.1940	*L. braziliensis*	95	169	263
LbrM28_V2.1940	*L. braziliensis*	95	169	263
LbrM28_V2.1950	*L. braziliensis*	44	685	728
LbrM28_V2.1950	*L. braziliensis*	42	825	866
LbrM28_V2.1950	*L. braziliensis*	42	825	866
LbrM28_V2.1960	*L. braziliensis*	91	396	486
LbrM28_V2.1970	*L. braziliensis*	47	1	47
LbrM28_V2.1970	*L. braziliensis*	287	208	494
LbrM28_V2.1970	*L. braziliensis*	113	793	905
LbrM28_V2.1970	*L. braziliensis*	66	1048	1113
LbrM28_V2.1970	*L. braziliensis*	41	1355	1395
LbrM28_V2.1970	*L. braziliensis*	43	1556	1598
LbrM28_V2.1970	*L. braziliensis*	40	1663	1702
LbrM28_V2.1970	*L. braziliensis*	103	1745	1847
LbrM28_V2.1970	*L. braziliensis*	65	1905	1969
LbrM28_V2.1970	*L. braziliensis*	58	2000	2057
LbrM28_V2.1970	*L. braziliensis*	58	2083	2140
LbrM28_V2.1970	*L. braziliensis*	49	2249	2297
LbrM28_V2.1970	*L. braziliensis*	79	2341	2419
LbrM28_V2.1970	*L. braziliensis*	104	2497	2600
LbrM28_V2.1970	*L. braziliensis*	52	2852	2903
LbrM28_V2.1980	*L. braziliensis*	72	33	104
LbrM28_V2.1990	*L. braziliensis*	53	395	447
LbrM28_V2.2000	*L. braziliensis*	392	1	392
LbrM28_V2.2000	*L. braziliensis*	47	501	547
LbrM28_V2.2000	*L. braziliensis*	53	904	956

LbrM28_V2.2000	*L. braziliensis*	48	1241	1288
LbrM28_V2.2020	*L. braziliensis*	263	1	263
LbrM28_V2.2020	*L. braziliensis*	126	277	402
LbrM28_V2.2020	*L. braziliensis*	182	418	599
LbrM28_V2.2030	*L. braziliensis*	83	54	136
LbrM28_V2.2030	*L. braziliensis*	44	231	274
LbrM28_V2.2030	*L. braziliensis*	44	231	274
LbrM28_V2.2040	*L. braziliensis*	139	140	278
LbrM28_V2.2040	*L. braziliensis*	54	328	381
LbrM28_V2.2040	*L. braziliensis*	82	625	706
LbrM28_V2.2050	*L. braziliensis*	56	1	56
LbrM28_V2.2050	*L. braziliensis*	126	386	511
LbrM28_V2.2050	*L. braziliensis*	283	538	820
LbrM28_V2.2050	*L. braziliensis*	85	1172	1256
LbrM28_V2.2060	*L. braziliensis*	41	77	117
LbrM28_V2.2070	*L. braziliensis*	49	1	49
LbrM28_V2.2070	*L. braziliensis*	300	146	445
LbrM28_V2.2070	*L. braziliensis*	98	512	609
LbrM28_V2.2080	*L. braziliensis*	80	1	80
LbrM28_V2.2080	*L. braziliensis*	239	93	331
LbrM28_V2.2080	*L. braziliensis*	55	541	595
LbrM28_V2.2090	*L. braziliensis*	113	1	113
LbrM28_V2.2100	*L. braziliensis*	155	17	171
LbrM28_V2.2110	*L. braziliensis*	64	111	174
LbrM28_V2.2120	*L. braziliensis*	56	1	56
LbrM28_V2.2120	*L. braziliensis*	62	100	161
LbrM28_V2.2120	*L. braziliensis*	42	195	236
LbrM28_V2.2120	*L. braziliensis*	113	342	454
LbrM28_V2.2140	*L. braziliensis*	185	360	544
LbrM28_V2.2140	*L. braziliensis*	232	548	779
LbrM28_V2.2160	*L. braziliensis*	65	213	277
LbrM28_V2.2160	*L. braziliensis*	46	547	592
LbrM28_V2.2160	*L. braziliensis*	46	547	592
LbrM28_V2.2180	*L. braziliensis*	76	284	359
LbrM28_V2.2180	*L. braziliensis*	106	384	489
LbrM28_V2.2180	*L. braziliensis*	41	922	962
LbrM28_V2.2180	*L. braziliensis*	98	1070	1167
LbrM28_V2.2180	*L. braziliensis*	112	1242	1353
LbrM28_V2.2190	*L. braziliensis*	214	292	505
LbrM28_V2.2190	*L. braziliensis*	54	576	629
LbrM28_V2.2190	*L. braziliensis*	206	660	865
LbrM28_V2.2200	*L. braziliensis*	61	59	119
LbrM28_V2.2200	*L. braziliensis*	50	398	447
LbrM28_V2.2200	*L. braziliensis*	50	398	447
LbrM28_V2.2210	*L. braziliensis*	208	27	234
LbrM28_V2.2210	*L. braziliensis*	41	243	283
LbrM28_V2.2210	*L. braziliensis*	197	335	531

LbrM28_V2.2240	*L. braziliensis*	54	149	202
LbrM28_V2.2270	*L. braziliensis*	68	477	544
LbrM28_V2.2310	*L. braziliensis*	55	305	359
LbrM28_V2.2320	*L. braziliensis*	49	647	695
LbrM28_V2.2340	*L. braziliensis*	43	449	491
LbrM28_V2.2350	*L. braziliensis*	116	1	116
LbrM28_V2.2350	*L. braziliensis*	66	516	581
LbrM28_V2.2370	*L. braziliensis*	138	128	265
LbrM28_V2.2370	*L. braziliensis*	121	473	593
LbrM28_V2.2380	*L. braziliensis*	78	1	78
LbrM28_V2.2390	*L. braziliensis*	106	1	106
LbrM28_V2.2390	*L. braziliensis*	58	108	165
LbrM28_V2.2390	*L. braziliensis*	45	174	218
LbrM28_V2.2390	*L. braziliensis*	58	235	292
LbrM28_V2.2390	*L. braziliensis*	94	323	416
LbrM28_V2.2390	*L. braziliensis*	465	419	883
LbrM28_V2.2400	*L. braziliensis*	41	1	41
LbrM28_V2.2400	*L. braziliensis*	59	582	640
LbrM28_V2.2400	*L. braziliensis*	59	582	640
LbrM28_V2.2410	*L. braziliensis*	403	1	403
LbrM28_V2.2430	*L. braziliensis*	75	1	75
LbrM28_V2.2430	*L. braziliensis*	346	95	440
LbrM28_V2.2430	*L. braziliensis*	60	464	523
LbrM28_V2.2430	*L. braziliensis*	109	539	647
LbrM28_V2.2440	*L. braziliensis*	51	87	137
LbrM28_V2.2470	*L. braziliensis*	142	341	482
LbrM28_V2.2480	*L. braziliensis*	44	378	421
LbrM28_V2.2480	*L. braziliensis*	73	1027	1099
LbrM28_V2.2480	*L. braziliensis*	327	1175	1501
LbrM28_V2.2490	*L. braziliensis*	70	540	609
LbrM28_V2.2490	*L. braziliensis*	44	751	794
LbrM28_V2.2490	*L. braziliensis*	44	751	794
LbrM28_V2.2500	*L. braziliensis*	69	1	69
LbrM28_V2.2500	*L. braziliensis*	104	75	178
LbrM28_V2.2500	*L. braziliensis*	116	213	328
LbrM28_V2.2500	*L. braziliensis*	178	363	540
LbrM28_V2.2500	*L. braziliensis*	43	566	608
LbrM28_V2.2510	*L. braziliensis*	64	45	108
LbrM28_V2.2520	*L. braziliensis*	46	1	46
LbrM28_V2.2520	*L. braziliensis*	196	71	266
LbrM28_V2.2520	*L. braziliensis*	284	306	589
LbrM28_V2.2520	*L. braziliensis*	153	608	760
LbrM28_V2.2520	*L. braziliensis*	151	781	931
LbrM28_V2.2530	*L. braziliensis*	95	53	147
LbrM28_V2.2530	*L. braziliensis*	59	251	309
LbrM28_V2.2530	*L. braziliensis*	84	362	445
LbrM28_V2.2530	*L. braziliensis*	42	463	504

LbrM28_V2.2530	*L. braziliensis*	45	621	665
LbrM28_V2.2540	*L. braziliensis*	72	215	286
LbrM28_V2.2570	*L. braziliensis*	64	734	797
LbrM28_V2.2580	*L. braziliensis*	92	695	786
LbrM28_V2.2590	*L. braziliensis*	439	114	552
LbrM28_V2.2590	*L. braziliensis*	45	561	605
LbrM28_V2.2590	*L. braziliensis*	118	608	725
LbrM28_V2.2590	*L. braziliensis*	599	741	1339
LbrM28_V2.2610	*L. braziliensis*	46	73	118
LbrM28_V2.2620	*L. braziliensis*	91	90	180
LbrM28_V2.2640	*L. braziliensis*	51	281	331
LbrM28_V2.2640	*L. braziliensis*	58	507	564
LbrM28_V2.2640	*L. braziliensis*	58	507	564
LbrM28_V2.2650	*L. braziliensis*	41	359	399
LbrM28_V2.2680	*L. braziliensis*	54	49	102
LbrM28_V2.2680	*L. braziliensis*	81	130	210
LbrM28_V2.2680	*L. braziliensis*	81	130	210
LbrM28_V2.2690	*L. braziliensis*	84	14	97
LbrM28_V2.2690	*L. braziliensis*	273	165	437
LbrM28_V2.2700	*L. braziliensis*	112	1	112
LbrM28_V2.2720	*L. braziliensis*	93	1	93
LbrM28_V2.2720	*L. braziliensis*	98	221	318
LbrM28_V2.2720	*L. braziliensis*	91	364	454
LbrM28_V2.2730	*L. braziliensis*	73	67	139
LbrM28_V2.2730	*L. braziliensis*	50	450	499
LbrM28_V2.2730	*L. braziliensis*	64	514	577
LbrM28_V2.2740	*L. braziliensis*	70	22	91
LbrM28_V2.2740	*L. braziliensis*	213	93	305
LbrM28_V2.2740	*L. braziliensis*	307	326	632
LbrM28_V2.2740	*L. braziliensis*	47	893	939
LbrM28_V2.2740	*L. braziliensis*	44	1127	1170
LbrM28_V2.2750	*L. braziliensis*	55	272	326
LbrM28_V2.2750	*L. braziliensis*	69	375	443
LbrM28_V2.2750	*L. braziliensis*	96	883	978
LbrM28_V2.2760	*L. braziliensis*	75	69	143
LbrM28_V2.2770	*L. braziliensis*	75	69	143
LbrM28_V2.2780	*L. braziliensis*	177	1	177
LbrM28_V2.2790	*L. braziliensis*	121	1	121
LbrM28_V2.2800	*L. braziliensis*	66	128	193
LbrM28_V2.2800	*L. braziliensis*	171	335	505
LbrM28_V2.2800	*L. braziliensis*	61	572	632
LbrM28_V2.2800	*L. braziliensis*	137	667	803
LbrM28_V2.2800	*L. braziliensis*	66	805	870
LbrM28_V2.2810	*L. braziliensis*	75	51	125
LbrM28_V2.2810	*L. braziliensis*	49	277	325
LbrM28_V2.2810	*L. braziliensis*	49	277	325
LbrM28_V2.2820	*L. braziliensis*	192	36	227

LbrM28_V2.2820	*L. braziliensis*	67	333	399
LbrM28_V2.2820	*L. braziliensis*	48	544	591
LbrM28_V2.2820	*L. braziliensis*	59	748	806
LbrM28_V2.2820	*L. braziliensis*	63	837	899
LbrM28_V2.2820	*L. braziliensis*	61	1206	1266
LbrM28_V2.2820	*L. braziliensis*	217	1493	1709
LbrM28_V2.2840	*L. braziliensis*	288	1	288
LbrM28_V2.2840	*L. braziliensis*	64	294	357
LbrM28_V2.2850	*L. braziliensis*	43	68	110
LbrM28_V2.2850	*L. braziliensis*	582	171	752
LbrM28_V2.2900	*L. braziliensis*	47	92	138
LbrM28_V2.2900	*L. braziliensis*	48	220	267
LbrM28_V2.2900	*L. braziliensis*	42	353	394
LbrM28_V2.2910	*L. braziliensis*	256	117	372
LbrM28_V2.2940	*L. braziliensis*	47	125	171
LbrM28_V2.2940	*L. braziliensis*	50	277	326
LbrM28_V2.2940	*L. braziliensis*	103	500	602
LbrM28_V2.2990	*L. braziliensis*	161	494	654
LbrM28_V2.3000	*L. braziliensis*	86	30	115
LbrM28_V2.3010	*L. braziliensis*	46	1	46
LbrM28_V2.3010	*L. braziliensis*	44	455	498
LbrM28_V2.3010	*L. braziliensis*	44	455	498
LbrM28_V2.3020	*L. braziliensis*	94	1	94
LbrM28_V2.3030	*L. braziliensis*	63	524	586
LbrM28_V2.3030	*L. braziliensis*	62	600	661
LbrM28_V2.3040	*L. braziliensis*	42	45	86
LbrM28_V2.3040	*L. braziliensis*	125	514	638
LbrM28_V2.3040	*L. braziliensis*	78	701	778
LbrM28_V2.3050	*L. braziliensis*	108	123	230
LbrM28_V2.3050	*L. braziliensis*	115	315	429
LbrM28_V2.3060	*L. braziliensis*	57	1	57
LbrM28_V2.3060	*L. braziliensis*	53	322	374
LbrM28_V2.3060	*L. braziliensis*	53	322	374
LbrM28_V2.3090	*L. braziliensis*	129	73	201
LbrM28_V2.3090	*L. braziliensis*	67	773	839
LbrM28_V2.3090	*L. braziliensis*	122	2737	2858
LbrM28_V2.3090	*L. braziliensis*	73	2970	3042
LbrM28_V2.3090	*L. braziliensis*	83	3286	3368
LbrM28_V2.3090	*L. braziliensis*	49	3933	3981
LbrM28_V2.3100	*L. braziliensis*	58	356	413
LbrM28_V2.3110	*L. braziliensis*	54	478	531
LbrM28_V2.3140	*L. braziliensis*	67	63	129
LbrM28_V2.3160	*L. braziliensis*	110	223	332
LbrM28_V2.3190	*L. braziliensis*	82	1	82
LbrM28_V2.3210	*L. braziliensis*	211	25	235
LbrM28_V2.3250	*L. braziliensis*	119	251	369
LbrM28_V2.3260	*L. braziliensis*	226	1	226

LbrM28_V2.3260	*L. braziliensis*	74	357	430
LbrM28_V2.3260	*L. braziliensis*	517	480	996
LbrM29_V2.0010	*L. braziliensis*	45	40	84
LbrM29_V2.0010	*L. braziliensis*	48	101	148
LbrM29_V2.0010	*L. braziliensis*	119	256	374
LbrM29_V2.0010	*L. braziliensis*	172	614	785
LbrM29_V2.0030	*L. braziliensis*	70	436	505
LbrM29_V2.0030	*L. braziliensis*	41	624	664
LbrM29_V2.0030	*L. braziliensis*	51	738	788
LbrM29_V2.0030	*L. braziliensis*	143	906	1048
LbrM29_V2.0040	*L. braziliensis*	76	392	467
LbrM29_V2.0050	*L. braziliensis*	51	37	87
LbrM29_V2.0060	*L. braziliensis*	40	595	634
LbrM29_V2.0070	*L. braziliensis*	46	338	383
LbrM29_V2.0080	*L. braziliensis*	44	18	61
LbrM29_V2.0080	*L. braziliensis*	72	103	174
LbrM29_V2.0080	*L. braziliensis*	72	103	174
LbrM29_V2.0110	*L. braziliensis*	71	1	71
LbrM29_V2.0110	*L. braziliensis*	46	373	418
LbrM29_V2.0110	*L. braziliensis*	46	373	418
LbrM29_V2.0120	*L. braziliensis*	903	1	903
LbrM29_V2.0120	*L. braziliensis*	41	1707	1747
LbrM29_V2.0120	*L. braziliensis*	74	2195	2268
LbrM29_V2.0130	*L. braziliensis*	51	1	51
LbrM29_V2.0130	*L. braziliensis*	45	73	117
LbrM29_V2.0130	*L. braziliensis*	40	603	642
LbrM29_V2.0140	*L. braziliensis*	47	1	47
LbrM29_V2.0140	*L. braziliensis*	59	50	108
LbrM29_V2.0160	*L. braziliensis*	42	1	42
LbrM29_V2.0160	*L. braziliensis*	40	294	333
LbrM29_V2.0160	*L. braziliensis*	40	294	333
LbrM29_V2.0170	*L. braziliensis*	40	474	513
LbrM29_V2.0180	*L. braziliensis*	49	1	49
LbrM29_V2.0180	*L. braziliensis*	75	63	137
LbrM29_V2.0190	*L. braziliensis*	140	1	140
LbrM29_V2.0190	*L. braziliensis*	60	237	296
LbrM29_V2.0190	*L. braziliensis*	153	328	480
LbrM29_V2.0200	*L. braziliensis*	404	1	404
LbrM29_V2.0200	*L. braziliensis*	45	418	462
LbrM29_V2.0200	*L. braziliensis*	114	493	606
LbrM29_V2.0200	*L. braziliensis*	68	837	904
LbrM29_V2.0200	*L. braziliensis*	74	954	1027
LbrM29_V2.0210	*L. braziliensis*	44	438	481
LbrM29_V2.0210	*L. braziliensis*	50	1042	1091
LbrM29_V2.0210	*L. braziliensis*	190	1322	1511
LbrM29_V2.0230	*L. braziliensis*	219	1	219
LbrM29_V2.0240	*L. braziliensis*	42	1	42

LbrM29_V2.0240	*L. braziliensis*	46	170	215
LbrM29_V2.0240	*L. braziliensis*	46	170	215
LbrM29_V2.0250	*L. braziliensis*	50	94	143
LbrM29_V2.0250	*L. braziliensis*	96	443	538
LbrM29_V2.0260	*L. braziliensis*	62	50	111
LbrM29_V2.0260	*L. braziliensis*	176	210	385
LbrM29_V2.0270	*L. braziliensis*	56	1	56
LbrM29_V2.0270	*L. braziliensis*	60	224	283
LbrM29_V2.0270	*L. braziliensis*	64	313	376
LbrM29_V2.0280	*L. braziliensis*	65	42	106
LbrM29_V2.0280	*L. braziliensis*	96	239	334
LbrM29_V2.0290	*L. braziliensis*	110	28	137
LbrM29_V2.0290	*L. braziliensis*	47	139	185
LbrM29_V2.0290	*L. braziliensis*	72	292	363
LbrM29_V2.0290	*L. braziliensis*	116	775	890
LbrM29_V2.0290	*L. braziliensis*	45	995	1039
LbrM29_V2.0290	*L. braziliensis*	67	1236	1302
LbrM29_V2.0310	*L. braziliensis*	150	30	179
LbrM29_V2.0310	*L. braziliensis*	93	249	341
LbrM29_V2.0310	*L. braziliensis*	81	385	465
LbrM29_V2.0310	*L. braziliensis*	106	501	606
LbrM29_V2.0310	*L. braziliensis*	367	895	1261
LbrM29_V2.0310	*L. braziliensis*	66	1277	1342
LbrM29_V2.0310	*L. braziliensis*	72	1364	1435
LbrM29_V2.0320	*L. braziliensis*	250	58	307
LbrM29_V2.0320	*L. braziliensis*	123	335	457
LbrM29_V2.0320	*L. braziliensis*	109	505	613
LbrM29_V2.0330	*L. braziliensis*	62	199	260
LbrM29_V2.0350	*L. braziliensis*	54	27	80
LbrM29_V2.0350	*L. braziliensis*	119	115	233
LbrM29_V2.0350	*L. braziliensis*	302	239	540
LbrM29_V2.0360	*L. braziliensis*	57	96	152
LbrM29_V2.0360	*L. braziliensis*	115	178	292
LbrM29_V2.0370	*L. braziliensis*	54	412	465
LbrM29_V2.0370	*L. braziliensis*	44	521	564
LbrM29_V2.0370	*L. braziliensis*	44	521	564
LbrM29_V2.0390	*L. braziliensis*	197	1	197
LbrM29_V2.0390	*L. braziliensis*	41	553	593
LbrM29_V2.0390	*L. braziliensis*	41	553	593
LbrM29_V2.0400	*L. braziliensis*	53	1	53
LbrM29_V2.0400	*L. braziliensis*	149	80	228
LbrM29_V2.0400	*L. braziliensis*	117	268	384
LbrM29_V2.0400	*L. braziliensis*	61	416	476
LbrM29_V2.0400	*L. braziliensis*	176	478	653
LbrM29_V2.0400	*L. braziliensis*	50	659	708
LbrM29_V2.0410	*L. braziliensis*	203	208	410
LbrM29_V2.0420	*L. braziliensis*	128	1	128

LbrM29_V2.0420	*L. braziliensis*	53	132	184
LbrM29_V2.0420	*L. braziliensis*	53	132	184
LbrM29_V2.0440	*L. braziliensis*	307	149	455
LbrM29_V2.0460	*L. braziliensis*	54	41	94
LbrM29_V2.0460	*L. braziliensis*	66	157	222
LbrM29_V2.0460	*L. braziliensis*	66	157	222
LbrM29_V2.0470	*L. braziliensis*	174	1	174
LbrM29_V2.0470	*L. braziliensis*	224	192	415
LbrM29_V2.0470	*L. braziliensis*	74	473	546
LbrM29_V2.0470	*L. braziliensis*	81	573	653
LbrM29_V2.0470	*L. braziliensis*	430	669	1098
LbrM29_V2.0470	*L. braziliensis*	47	1121	1167
LbrM29_V2.0470	*L. braziliensis*	131	1235	1365
LbrM29_V2.0470	*L. braziliensis*	100	1371	1470
LbrM29_V2.0470	*L. braziliensis*	327	1500	1826
LbrM29_V2.0470	*L. braziliensis*	610	1859	2468
LbrM29_V2.0470	*L. braziliensis*	53	2479	2531
LbrM29_V2.0470	*L. braziliensis*	108	2666	2773
LbrM29_V2.0470	*L. braziliensis*	69	2790	2858
LbrM29_V2.0480	*L. braziliensis*	213	1	213
LbrM29_V2.0490	*L. braziliensis*	159	1	159
LbrM29_V2.0490	*L. braziliensis*	77	185	261
LbrM29_V2.0490	*L. braziliensis*	337	283	619
LbrM29_V2.0490	*L. braziliensis*	46	836	881
LbrM29_V2.0500	*L. braziliensis*	43	235	277
LbrM29_V2.0530	*L. braziliensis*	203	46	248
LbrM29_V2.0540	*L. braziliensis*	67	176	242
LbrM29_V2.0550	*L. braziliensis*	48	61	108
LbrM29_V2.0550	*L. braziliensis*	56	141	196
LbrM29_V2.0550	*L. braziliensis*	116	269	384
LbrM29_V2.0570	*L. braziliensis*	66	1	66
LbrM29_V2.0580	*L. braziliensis*	53	525	577
LbrM29_V2.0590	*L. braziliensis*	53	1	53
LbrM29_V2.0590	*L. braziliensis*	64	71	134
LbrM29_V2.0590	*L. braziliensis*	46	179	224
LbrM29_V2.0590	*L. braziliensis*	80	251	330
LbrM29_V2.0590	*L. braziliensis*	129	352	480
LbrM29_V2.0600	*L. braziliensis*	143	144	286
LbrM29_V2.0600	*L. braziliensis*	54	699	752
LbrM29_V2.0600	*L. braziliensis*	41	1003	1043
LbrM29_V2.0600	*L. braziliensis*	40	1049	1088
LbrM29_V2.0610	*L. braziliensis*	53	104	156
LbrM29_V2.0610	*L. braziliensis*	188	158	345
LbrM29_V2.0610	*L. braziliensis*	219	360	578
LbrM29_V2.0620	*L. braziliensis*	63	53	115
LbrM29_V2.0620	*L. braziliensis*	175	221	395
LbrM29_V2.0620	*L. braziliensis*	169	439	607

LbrM29_V2.0630	*L. braziliensis*	49	591	639
LbrM29_V2.0640	*L. braziliensis*	85	1	85
LbrM29_V2.0650	*L. braziliensis*	85	1	85
LbrM29_V2.0660	*L. braziliensis*	187	291	477
LbrM29_V2.0670	*L. braziliensis*	130	1	130
LbrM29_V2.0670	*L. braziliensis*	61	194	254
LbrM29_V2.0670	*L. braziliensis*	52	333	384
LbrM29_V2.0670	*L. braziliensis*	51	413	463
LbrM29_V2.0690	*L. braziliensis*	195	1	195
LbrM29_V2.0690	*L. braziliensis*	79	382	460
LbrM29_V2.0690	*L. braziliensis*	173	567	739
LbrM29_V2.0710	*L. braziliensis*	108	69	176
LbrM29_V2.0710	*L. braziliensis*	56	207	262
LbrM29_V2.0710	*L. braziliensis*	49	363	411
LbrM29_V2.0710	*L. braziliensis*	43	539	581
LbrM29_V2.0710	*L. braziliensis*	92	615	706
LbrM29_V2.0720	*L. braziliensis*	49	353	401
LbrM29_V2.0720	*L. braziliensis*	44	460	503
LbrM29_V2.0720	*L. braziliensis*	59	677	735
LbrM29_V2.0720	*L. braziliensis*	43	816	858
LbrM29_V2.0750	*L. braziliensis*	175	1	175
LbrM29_V2.0760	*L. braziliensis*	49	325	373
LbrM29_V2.0770	*L. braziliensis*	62	1	62
LbrM29_V2.0770	*L. braziliensis*	329	118	446
LbrM29_V2.0770	*L. braziliensis*	50	508	557
LbrM29_V2.0770	*L. braziliensis*	53	630	682
LbrM29_V2.0780	*L. braziliensis*	69	80	148
LbrM29_V2.0780	*L. braziliensis*	40	248	287
LbrM29_V2.0780	*L. braziliensis*	65	723	787
LbrM29_V2.0790	*L. braziliensis*	104	1	104
LbrM29_V2.0790	*L. braziliensis*	40	146	185
LbrM29_V2.0790	*L. braziliensis*	40	146	185
LbrM29_V2.0800	*L. braziliensis*	46	473	518
LbrM29_V2.0810	*L. braziliensis*	125	36	160
LbrM29_V2.0810	*L. braziliensis*	89	433	521
LbrM29_V2.0810	*L. braziliensis*	122	707	828
LbrM29_V2.0810	*L. braziliensis*	74	883	956
LbrM29_V2.0810	*L. braziliensis*	59	1421	1479
LbrM29_V2.0810	*L. braziliensis*	66	1505	1570
LbrM29_V2.0820	*L. braziliensis*	155	1	155
LbrM29_V2.0820	*L. braziliensis*	47	207	253
LbrM29_V2.0820	*L. braziliensis*	219	434	652
LbrM29_V2.0830	*L. braziliensis*	408	44	451
LbrM29_V2.0830	*L. braziliensis*	56	506	561
LbrM29_V2.0840	*L. braziliensis*	70	88	157
LbrM29_V2.0840	*L. braziliensis*	340	277	616
LbrM29_V2.0850	*L. braziliensis*	45	198	242

LbrM29_V2.0860	*L. braziliensis*	151	1	151
LbrM29_V2.0860	*L. braziliensis*	103	325	427
LbrM29_V2.0880	*L. braziliensis*	89	57	145
LbrM29_V2.0880	*L. braziliensis*	64	147	210
LbrM29_V2.0880	*L. braziliensis*	87	213	299
LbrM29_V2.0890	*L. braziliensis*	76	1	76
LbrM29_V2.0890	*L. braziliensis*	69	174	242
LbrM29_V2.0890	*L. braziliensis*	72	289	360
LbrM29_V2.0890	*L. braziliensis*	92	423	514
LbrM29_V2.0920	*L. braziliensis*	67	93	159
LbrM29_V2.0960	*L. braziliensis*	61	391	451
LbrM29_V2.0970	*L. braziliensis*	87	129	215
LbrM29_V2.0980	*L. braziliensis*	50	257	306
LbrM29_V2.0990	*L. braziliensis*	56	337	392
LbrM29_V2.0990	*L. braziliensis*	40	465	504
LbrM29_V2.0990	*L. braziliensis*	40	465	504
LbrM29_V2.1000	*L. braziliensis*	41	1	41
LbrM29_V2.1000	*L. braziliensis*	74	319	392
LbrM29_V2.1000	*L. braziliensis*	74	319	392
LbrM29_V2.1010	*L. braziliensis*	158	1	158
LbrM29_V2.1030	*L. braziliensis*	95	40	134
LbrM29_V2.1040	*L. braziliensis*	59	1	59
LbrM29_V2.1040	*L. braziliensis*	132	122	253
LbrM29_V2.1040	*L. braziliensis*	93	267	359
LbrM29_V2.1040	*L. braziliensis*	50	367	416
LbrM29_V2.1050	*L. braziliensis*	604	340	943
LbrM29_V2.1050	*L. braziliensis*	290	1006	1295
LbrM29_V2.1050	*L. braziliensis*	221	1313	1533
LbrM29_V2.1060	*L. braziliensis*	252	1	252
LbrM29_V2.1060	*L. braziliensis*	68	291	358
LbrM29_V2.1060	*L. braziliensis*	141	369	509
LbrM29_V2.1070	*L. braziliensis*	126	195	320
LbrM29_V2.1070	*L. braziliensis*	92	326	417
LbrM29_V2.1070	*L. braziliensis*	238	445	682
LbrM29_V2.1070	*L. braziliensis*	50	749	798
LbrM29_V2.1070	*L. braziliensis*	91	883	973
LbrM29_V2.1080	*L. braziliensis*	107	53	159
LbrM29_V2.1080	*L. braziliensis*	136	172	307
LbrM29_V2.1080	*L. braziliensis*	264	354	617
LbrM29_V2.1100	*L. braziliensis*	91	14	104
LbrM29_V2.1100	*L. braziliensis*	199	206	404
LbrM29_V2.1100	*L. braziliensis*	43	629	671
LbrM29_V2.1110	*L. braziliensis*	147	1	147
LbrM29_V2.1120	*L. braziliensis*	51	1	51
LbrM29_V2.1120	*L. braziliensis*	53	259	311
LbrM29_V2.1120	*L. braziliensis*	49	478	526
LbrM29_V2.1120	*L. braziliensis*	147	838	984

LbrM29_V2.1120	*L. braziliensis*	50	1139	1188
LbrM29_V2.1120	*L. braziliensis*	93	1195	1287
LbrM29_V2.1120	*L. braziliensis*	142	1320	1461
LbrM29_V2.1120	*L. braziliensis*	65	1544	1608
LbrM29_V2.1120	*L. braziliensis*	46	1635	1680
LbrM29_V2.1120	*L. braziliensis*	63	1843	1905
LbrM29_V2.1130	*L. braziliensis*	61	58	118
LbrM29_V2.1130	*L. braziliensis*	56	177	232
LbrM29_V2.1130	*L. braziliensis*	145	301	445
LbrM29_V2.1130	*L. braziliensis*	55	536	590
LbrM29_V2.1140	*L. braziliensis*	51	1	51
LbrM29_V2.1140	*L. braziliensis*	85	60	144
LbrM29_V2.1140	*L. braziliensis*	62	195	256
LbrM29_V2.1140	*L. braziliensis*	77	367	443
LbrM29_V2.1140	*L. braziliensis*	100	464	563
LbrM29_V2.1140	*L. braziliensis*	193	571	763
LbrM29_V2.1140	*L. braziliensis*	138	801	938
LbrM29_V2.1150	*L. braziliensis*	88	286	373
LbrM29_V2.1160	*L. braziliensis*	53	308	360
LbrM29_V2.1170	*L. braziliensis*	64	113	176
LbrM29_V2.1170	*L. braziliensis*	47	579	625
LbrM29_V2.1180	*L. braziliensis*	88	286	373
LbrM29_V2.1200	*L. braziliensis*	103	82	184
LbrM29_V2.1200	*L. braziliensis*	57	245	301
LbrM29_V2.1210	*L. braziliensis*	96	79	174
LbrM29_V2.1250	*L. braziliensis*	182	104	285
LbrM29_V2.1260	*L. braziliensis*	41	48	88
LbrM29_V2.1260	*L. braziliensis*	120	95	214
LbrM29_V2.1260	*L. braziliensis*	128	251	378
LbrM29_V2.1260	*L. braziliensis*	152	455	606
LbrM29_V2.1260	*L. braziliensis*	299	717	1015
LbrM29_V2.1260	*L. braziliensis*	44	1048	1091
LbrM29_V2.1260	*L. braziliensis*	202	1104	1305
LbrM29_V2.1260	*L. braziliensis*	96	1350	1445
LbrM29_V2.1260	*L. braziliensis*	41	1504	1544
LbrM29_V2.1260	*L. braziliensis*	71	1561	1631
LbrM29_V2.1260	*L. braziliensis*	52	1689	1740
LbrM29_V2.1260	*L. braziliensis*	91	1784	1874
LbrM29_V2.1260	*L. braziliensis*	239	1986	2224
LbrM29_V2.1260	*L. braziliensis*	178	2274	2451
LbrM29_V2.1270	*L. braziliensis*	69	37	105
LbrM29_V2.1270	*L. braziliensis*	73	251	323
LbrM29_V2.1270	*L. braziliensis*	73	251	323
LbrM29_V2.1280	*L. braziliensis*	41	104	144
LbrM29_V2.1280	*L. braziliensis*	109	372	480
LbrM29_V2.1300	*L. braziliensis*	339	135	473
LbrM29_V2.1310	*L. braziliensis*	600	1	600

LbrM29_V2.1310	*L. braziliensis*	468	613	1080
LbrM29_V2.1310	*L. braziliensis*	165	1096	1260
LbrM29_V2.1310	*L. braziliensis*	55	1296	1350
LbrM29_V2.1310	*L. braziliensis*	48	1460	1507
LbrM29_V2.1310	*L. braziliensis*	51	1604	1654
LbrM29_V2.1310	*L. braziliensis*	374	1847	2220
LbrM29_V2.1320	*L. braziliensis*	42	379	420
LbrM29_V2.1320	*L. braziliensis*	60	528	587
LbrM29_V2.1320	*L. braziliensis*	48	673	720
LbrM29_V2.1330	*L. braziliensis*	253	42	294
LbrM29_V2.1330	*L. braziliensis*	223	300	522
LbrM29_V2.1330	*L. braziliensis*	303	535	837
LbrM29_V2.1330	*L. braziliensis*	350	850	1199
LbrM29_V2.1330	*L. braziliensis*	262	1233	1494
LbrM29_V2.1330	*L. braziliensis*	355	1530	1884
LbrM29_V2.1330	*L. braziliensis*	48	1901	1948
LbrM29_V2.1350	*L. braziliensis*	45	53	97
LbrM29_V2.1350	*L. braziliensis*	94	407	500
LbrM29_V2.1350	*L. braziliensis*	94	407	500
LbrM29_V2.1360	*L. braziliensis*	103	1	103
LbrM29_V2.1360	*L. braziliensis*	105	134	238
LbrM29_V2.1360	*L. braziliensis*	140	791	930
LbrM29_V2.1360	*L. braziliensis*	71	970	1040
LbrM29_V2.1360	*L. braziliensis*	262	1049	1310
LbrM29_V2.1360	*L. braziliensis*	143	1406	1548
LbrM29_V2.1370	*L. braziliensis*	44	52	95
LbrM29_V2.1400	*L. braziliensis*	71	192	262
LbrM29_V2.1400	*L. braziliensis*	92	316	407
LbrM29_V2.1400	*L. braziliensis*	92	316	407
LbrM29_V2.1410	*L. braziliensis*	138	1	138
LbrM29_V2.1410	*L. braziliensis*	126	140	265
LbrM29_V2.1410	*L. braziliensis*	43	415	457
LbrM29_V2.1410	*L. braziliensis*	42	512	553
LbrM29_V2.1420	*L. braziliensis*	140	1	140
LbrM29_V2.1420	*L. braziliensis*	44	175	218
LbrM29_V2.1420	*L. braziliensis*	44	175	218
LbrM29_V2.1440	*L. braziliensis*	65	1	65
LbrM29_V2.1440	*L. braziliensis*	113	252	364
LbrM29_V2.1440	*L. braziliensis*	86	438	523
LbrM29_V2.1440	*L. braziliensis*	63	544	606
LbrM29_V2.1450	*L. braziliensis*	250	15	264
LbrM29_V2.1450	*L. braziliensis*	67	363	429
LbrM29_V2.1450	*L. braziliensis*	166	456	621
LbrM29_V2.1460	*L. braziliensis*	60	1	60
LbrM29_V2.1460	*L. braziliensis*	270	314	583
LbrM29_V2.1460	*L. braziliensis*	71	714	784
LbrM29_V2.1470	*L. braziliensis*	192	50	241

LbrM29_V2.1480	*L. braziliensis*	206	1	206
LbrM29_V2.1490	*L. braziliensis*	197	1	197
LbrM29_V2.1490	*L. braziliensis*	64	219	282
LbrM29_V2.1490	*L. braziliensis*	165	287	451
LbrM29_V2.1490	*L. braziliensis*	247	487	733
LbrM29_V2.1490	*L. braziliensis*	73	751	823
LbrM29_V2.1490	*L. braziliensis*	286	871	1156
LbrM29_V2.1500	*L. braziliensis*	142	1	142
LbrM29_V2.1500	*L. braziliensis*	124	327	450
LbrM29_V2.1500	*L. braziliensis*	47	589	635
LbrM29_V2.1510	*L. braziliensis*	165	1	165
LbrM29_V2.1510	*L. braziliensis*	46	212	257
LbrM29_V2.1510	*L. braziliensis*	52	282	333
LbrM29_V2.1510	*L. braziliensis*	47	375	421
LbrM29_V2.1510	*L. braziliensis*	246	453	698
LbrM29_V2.1510	*L. braziliensis*	156	708	863
LbrM29_V2.1510	*L. braziliensis*	165	883	1047
LbrM29_V2.1510	*L. braziliensis*	80	1079	1158
LbrM29_V2.1510	*L. braziliensis*	54	1188	1241
LbrM29_V2.1510	*L. braziliensis*	101	1266	1366
LbrM29_V2.1520	*L. braziliensis*	85	1	85
LbrM29_V2.1520	*L. braziliensis*	82	280	361
LbrM29_V2.1520	*L. braziliensis*	82	280	361
LbrM29_V2.1540	*L. braziliensis*	72	32	103
LbrM29_V2.1540	*L. braziliensis*	70	254	323
LbrM29_V2.1540	*L. braziliensis*	59	563	621
LbrM29_V2.1540	*L. braziliensis*	67	644	710
LbrM29_V2.1540	*L. braziliensis*	44	827	870
LbrM29_V2.1550	*L. braziliensis*	49	79	127
LbrM29_V2.1550	*L. braziliensis*	67	183	249
LbrM29_V2.1550	*L. braziliensis*	98	291	388
LbrM29_V2.1550	*L. braziliensis*	93	438	530
LbrM29_V2.1550	*L. braziliensis*	104	930	1033
LbrM29_V2.1560	*L. braziliensis*	134	1	134
LbrM29_V2.1560	*L. braziliensis*	83	264	346
LbrM29_V2.1570	*L. braziliensis*	71	173	243
LbrM29_V2.1580	*L. braziliensis*	72	15	86
LbrM29_V2.1580	*L. braziliensis*	104	374	477
LbrM29_V2.1580	*L. braziliensis*	108	805	912
LbrM29_V2.1590	*L. braziliensis*	114	308	421
LbrM29_V2.1600	*L. braziliensis*	97	292	388
LbrM29_V2.1610	*L. braziliensis*	64	292	355
LbrM29_V2.1620	*L. braziliensis*	47	73	119
LbrM29_V2.1620	*L. braziliensis*	54	175	228
LbrM29_V2.1620	*L. braziliensis*	49	348	396
LbrM29_V2.1620	*L. braziliensis*	61	605	665
LbrM29_V2.1640	*L. braziliensis*	93	235	327

LbrM29_V2.1650	*L. braziliensis*	107	1	107
LbrM29_V2.1650	*L. braziliensis*	128	128	255
LbrM29_V2.1650	*L. braziliensis*	51	536	586
LbrM29_V2.1670	*L. braziliensis*	67	49	115
LbrM29_V2.1670	*L. braziliensis*	85	200	284
LbrM29_V2.1680	*L. braziliensis*	73	122	194
LbrM29_V2.1680	*L. braziliensis*	128	236	363
LbrM29_V2.1700	*L. braziliensis*	63	1	63
LbrM29_V2.1700	*L. braziliensis*	565	144	708
LbrM29_V2.1730	*L. braziliensis*	93	66	158
LbrM29_V2.1730	*L. braziliensis*	72	268	339
LbrM29_V2.1730	*L. braziliensis*	41	540	580
LbrM29_V2.1730	*L. braziliensis*	79	738	816
LbrM29_V2.1730	*L. braziliensis*	58	922	979
LbrM29_V2.1730	*L. braziliensis*	77	1018	1094
LbrM29_V2.1730	*L. braziliensis*	43	1214	1256
LbrM29_V2.1730	*L. braziliensis*	60	1293	1352
LbrM29_V2.1730	*L. braziliensis*	63	1650	1712
LbrM29_V2.1730	*L. braziliensis*	40	1862	1901
LbrM29_V2.1730	*L. braziliensis*	66	2017	2082
LbrM29_V2.1730	*L. braziliensis*	53	2154	2206
LbrM29_V2.1740	*L. braziliensis*	76	1	76
LbrM29_V2.1740	*L. braziliensis*	117	301	417
LbrM29_V2.1740	*L. braziliensis*	61	1159	1219
LbrM29_V2.1750	*L. braziliensis*	44	1	44
LbrM29_V2.1750	*L. braziliensis*	41	195	235
LbrM29_V2.1750	*L. braziliensis*	85	353	437
LbrM29_V2.1760	*L. braziliensis*	123	93	215
LbrM29_V2.1760	*L. braziliensis*	65	266	330
LbrM29_V2.1760	*L. braziliensis*	50	405	454
LbrM29_V2.1770	*L. braziliensis*	41	1	41
LbrM29_V2.1770	*L. braziliensis*	40	134	173
LbrM29_V2.1770	*L. braziliensis*	40	134	173
LbrM29_V2.1790	*L. braziliensis*	52	209	260
LbrM29_V2.1790	*L. braziliensis*	101	594	694
LbrM29_V2.1790	*L. braziliensis*	319	848	1166
LbrM29_V2.1790	*L. braziliensis*	43	1180	1222
LbrM29_V2.1800	*L. braziliensis*	47	1	47
LbrM29_V2.1800	*L. braziliensis*	42	412	453
LbrM29_V2.1800	*L. braziliensis*	52	536	587
LbrM29_V2.1800	*L. braziliensis*	50	932	981
LbrM29_V2.1810	*L. braziliensis*	40	92	131
LbrM29_V2.1820	*L. braziliensis*	40	92	131
LbrM29_V2.1840	*L. braziliensis*	42	1	42
LbrM29_V2.1840	*L. braziliensis*	50	59	108
LbrM29_V2.1840	*L. braziliensis*	50	59	108
LbrM29_V2.1850	*L. braziliensis*	73	40	112

LbrM29_V2.1850	*L. braziliensis*	53	138	190
LbrM29_V2.1850	*L. braziliensis*	44	351	394
LbrM29_V2.1850	*L. braziliensis*	48	602	649
LbrM29_V2.1850	*L. braziliensis*	82	741	822
LbrM29_V2.1860	*L. braziliensis*	101	69	169
LbrM29_V2.1860	*L. braziliensis*	129	283	411
LbrM29_V2.1870	*L. braziliensis*	40	85	124
LbrM29_V2.1870	*L. braziliensis*	42	226	267
LbrM29_V2.1870	*L. braziliensis*	77	293	369
LbrM29_V2.1880	*L. braziliensis*	47	103	149
LbrM29_V2.1880	*L. braziliensis*	64	302	365
LbrM29_V2.1890	*L. braziliensis*	63	193	255
LbrM29_V2.1900	*L. braziliensis*	66	1	66
LbrM29_V2.1900	*L. braziliensis*	115	159	273
LbrM29_V2.1920	*L. braziliensis*	45	58	102
LbrM29_V2.1940	*L. braziliensis*	152	180	331
LbrM29_V2.1940	*L. braziliensis*	126	336	461
LbrM29_V2.1940	*L. braziliensis*	84	603	686
LbrM29_V2.1940	*L. braziliensis*	80	700	779
LbrM29_V2.1940	*L. braziliensis*	152	786	937
LbrM29_V2.1940	*L. braziliensis*	77	1183	1259
LbrM29_V2.1940	*L. braziliensis*	44	1298	1341
LbrM29_V2.1940	*L. braziliensis*	133	1358	1490
LbrM29_V2.1940	*L. braziliensis*	66	1587	1652
LbrM29_V2.1940	*L. braziliensis*	89	1772	1860
LbrM29_V2.1940	*L. braziliensis*	43	1919	1961
LbrM29_V2.1950	*L. braziliensis*	58	26	83
LbrM29_V2.1950	*L. braziliensis*	70	91	160
LbrM29_V2.1950	*L. braziliensis*	43	402	444
LbrM29_V2.1960	*L. braziliensis*	76	41	116
LbrM29_V2.1970	*L. braziliensis*	47	361	407
LbrM29_V2.1970	*L. braziliensis*	58	505	562
LbrM29_V2.1980	*L. braziliensis*	42	494	535
LbrM29_V2.1990	*L. braziliensis*	98	434	531
LbrM29_V2.1990	*L. braziliensis*	90	603	692
LbrM29_V2.1990	*L. braziliensis*	50	709	758
LbrM29_V2.2000	*L. braziliensis*	184	201	384
LbrM29_V2.2010	*L. braziliensis*	201	1	201
LbrM29_V2.2010	*L. braziliensis*	97	215	311
LbrM29_V2.2010	*L. braziliensis*	51	552	602
LbrM29_V2.2010	*L. braziliensis*	59	750	808
LbrM29_V2.2010	*L. braziliensis*	97	864	960
LbrM29_V2.2010	*L. braziliensis*	252	978	1229
LbrM29_V2.2020	*L. braziliensis*	280	16	295
LbrM29_V2.2020	*L. braziliensis*	77	314	390
LbrM29_V2.2020	*L. braziliensis*	58	423	480
LbrM29_V2.2020	*L. braziliensis*	176	555	730

LbrM29_V2.2030	*L. braziliensis*	44	16	59
LbrM29_V2.2030	*L. braziliensis*	52	971	1022
LbrM29_V2.2030	*L. braziliensis*	52	971	1022
LbrM29_V2.2040	*L. braziliensis*	114	1	114
LbrM29_V2.2040	*L. braziliensis*	154	246	399
LbrM29_V2.2070	*L. braziliensis*	88	102	189
LbrM29_V2.2070	*L. braziliensis*	65	353	417
LbrM29_V2.2070	*L. braziliensis*	76	477	552
LbrM29_V2.2070	*L. braziliensis*	93	1047	1139
LbrM29_V2.2070	*L. braziliensis*	59	1494	1552
LbrM29_V2.2080	*L. braziliensis*	65	193	257
LbrM29_V2.2080	*L. braziliensis*	54	358	411
LbrM29_V2.2080	*L. braziliensis*	54	358	411
LbrM29_V2.2090	*L. braziliensis*	40	353	392
LbrM29_V2.2130	*L. braziliensis*	65	51	115
LbrM29_V2.2150	*L. braziliensis*	44	49	92
LbrM29_V2.2150	*L. braziliensis*	66	303	368
LbrM29_V2.2150	*L. braziliensis*	66	546	611
LbrM29_V2.2160	*L. braziliensis*	266	1	266
LbrM29_V2.2170	*L. braziliensis*	51	126	176
LbrM29_V2.2170	*L. braziliensis*	50	998	1047
LbrM29_V2.2170	*L. braziliensis*	50	998	1047
LbrM29_V2.2180	*L. braziliensis*	84	495	578
LbrM29_V2.2180	*L. braziliensis*	52	641	692
LbrM29_V2.2190	*L. braziliensis*	121	165	285
LbrM29_V2.2200	*L. braziliensis*	55	36	90
LbrM29_V2.2200	*L. braziliensis*	67	121	187
LbrM29_V2.2210	*L. braziliensis*	40	37	76
LbrM29_V2.2210	*L. braziliensis*	107	177	283
LbrM29_V2.2230	*L. braziliensis*	198	1	198
LbrM29_V2.2230	*L. braziliensis*	69	548	616
LbrM29_V2.2240	*L. braziliensis*	73	1	73
LbrM29_V2.2240	*L. braziliensis*	74	187	260
LbrM29_V2.2240	*L. braziliensis*	74	187	260
LbrM29_V2.2260	*L. braziliensis*	300	1	300
LbrM29_V2.2270	*L. braziliensis*	48	132	179
LbrM29_V2.2300	*L. braziliensis*	43	168	210
LbrM29_V2.2300	*L. braziliensis*	94	289	382
LbrM29_V2.2350	*L. braziliensis*	70	82	151
LbrM29_V2.2350	*L. braziliensis*	44	174	217
LbrM29_V2.2350	*L. braziliensis*	54	295	348
LbrM29_V2.2360	*L. braziliensis*	70	152	221
LbrM29_V2.2360	*L. braziliensis*	44	244	287
LbrM29_V2.2360	*L. braziliensis*	49	341	389
LbrM29_V2.2370	*L. braziliensis*	50	1	50
LbrM29_V2.2390	*L. braziliensis*	145	42	186
LbrM29_V2.2390	*L. braziliensis*	71	198	268

LbrM29_V2.2390	*L. braziliensis*	345	353	697
LbrM29_V2.2400	*L. braziliensis*	638	55	692
LbrM29_V2.2410	*L. braziliensis*	82	1	82
LbrM29_V2.2430	*L. braziliensis*	83	1	83
LbrM29_V2.2430	*L. braziliensis*	90	131	220
LbrM29_V2.2440	*L. braziliensis*	83	1	83
LbrM29_V2.2440	*L. braziliensis*	90	131	220
LbrM29_V2.2450	*L. braziliensis*	971	1	971
LbrM29_V2.2470	*L. braziliensis*	55	63	117
LbrM29_V2.2470	*L. braziliensis*	65	367	431
LbrM29_V2.2470	*L. braziliensis*	65	367	431
LbrM29_V2.2490	*L. braziliensis*	91	300	390
LbrM29_V2.2520	*L. braziliensis*	61	773	833
LbrM29_V2.2530	*L. braziliensis*	56	1	56
LbrM29_V2.2530	*L. braziliensis*	103	69	171
LbrM29_V2.2530	*L. braziliensis*	40	182	221
LbrM29_V2.2530	*L. braziliensis*	40	293	332
LbrM29_V2.2540	*L. braziliensis*	149	280	428
LbrM29_V2.2550	*L. braziliensis*	48	1	48
LbrM29_V2.2550	*L. braziliensis*	101	78	178
LbrM29_V2.2550	*L. braziliensis*	470	200	669
LbrM29_V2.2560	*L. braziliensis*	57	1	57
LbrM29_V2.2560	*L. braziliensis*	282	145	426
LbrM29_V2.2560	*L. braziliensis*	71	524	594
LbrM29_V2.2570	*L. braziliensis*	43	315	357
LbrM29_V2.2580	*L. braziliensis*	108	1	108
LbrM29_V2.2580	*L. braziliensis*	48	161	208
LbrM29_V2.2580	*L. braziliensis*	72	641	712
LbrM29_V2.2600	*L. braziliensis*	61	113	173
LbrM29_V2.2600	*L. braziliensis*	47	210	256
LbrM29_V2.2600	*L. braziliensis*	90	259	348
LbrM29_V2.2600	*L. braziliensis*	65	378	442
LbrM29_V2.2600	*L. braziliensis*	70	502	571
LbrM29_V2.2600	*L. braziliensis*	66	656	721
LbrM29_V2.2600	*L. braziliensis*	139	781	919
LbrM29_V2.2600	*L. braziliensis*	48	974	1021
LbrM29_V2.2600	*L. braziliensis*	91	1037	1127
LbrM29_V2.2600	*L. braziliensis*	78	1136	1213
LbrM29_V2.2620	*L. braziliensis*	44	1	44
LbrM29_V2.2620	*L. braziliensis*	80	462	541
LbrM29_V2.2620	*L. braziliensis*	65	824	888
LbrM29_V2.2620	*L. braziliensis*	40	947	986
LbrM29_V2.2630	*L. braziliensis*	113	753	865
LbrM29_V2.2640	*L. braziliensis*	71	252	322
LbrM29_V2.2640	*L. braziliensis*	96	348	443
LbrM29_V2.2650	*L. braziliensis*	43	54	96
LbrM29_V2.2650	*L. braziliensis*	50	110	159

LbrM29_V2.2650	*L. braziliensis*	80	194	273
LbrM29_V2.2660	*L. braziliensis*	339	166	504
LbrM29_V2.2680	*L. braziliensis*	90	535	624
LbrM29_V2.2680	*L. braziliensis*	73	651	723
LbrM29_V2.2680	*L. braziliensis*	73	651	723
LbrM29_V2.2690	*L. braziliensis*	80	1	80
LbrM29_V2.2690	*L. braziliensis*	41	82	122
LbrM29_V2.2690	*L. braziliensis*	68	254	321
LbrM29_V2.2690	*L. braziliensis*	82	606	687
LbrM29_V2.2710	*L. braziliensis*	48	74	121
LbrM29_V2.2710	*L. braziliensis*	43	276	318
LbrM29_V2.2710	*L. braziliensis*	168	439	606
LbrM29_V2.2710	*L. braziliensis*	41	664	704
LbrM29_V2.2710	*L. braziliensis*	61	804	864
LbrM29_V2.2710	*L. braziliensis*	99	1104	1202
LbrM29_V2.2710	*L. braziliensis*	49	1248	1296
LbrM29_V2.2710	*L. braziliensis*	86	1308	1393
LbrM29_V2.2710	*L. braziliensis*	164	1503	1666
LbrM29_V2.2710	*L. braziliensis*	61	1802	1862
LbrM29_V2.2720	*L. braziliensis*	84	181	264
LbrM29_V2.2730	*L. braziliensis*	41	66	106
LbrM29_V2.2740	*L. braziliensis*	357	92	448
LbrM29_V2.2740	*L. braziliensis*	158	856	1013
LbrM29_V2.2750	*L. braziliensis*	194	53	246
LbrM29_V2.2780	*L. braziliensis*	43	1	43
LbrM29_V2.2780	*L. braziliensis*	87	238	324
LbrM29_V2.2780	*L. braziliensis*	87	238	324
LbrM29_V2.2790	*L. braziliensis*	45	251	295
LbrM29_V2.2800	*L. braziliensis*	63	143	205
LbrM29_V2.2820	*L. braziliensis*	40	1	40
LbrM29_V2.2870	*L. braziliensis*	179	557	735
LbrM29_V2.2890	*L. braziliensis*	108	1	108
LbrM29_V2.2900	*L. braziliensis*	42	1	42
LbrM29_V2.2910	*L. braziliensis*	110	168	277
LbrM29_V2.2920	*L. braziliensis*	91	1	91
LbrM29_V2.2920	*L. braziliensis*	60	106	165
LbrM29_V2.2920	*L. braziliensis*	51	167	217
LbrM29_V2.2920	*L. braziliensis*	525	398	922
LbrM29_V2.2930	*L. braziliensis*	87	203	289
LbrM29_V2.2930	*L. braziliensis*	76	323	398
LbrM29_V2.2930	*L. braziliensis*	76	323	398
LbrM30_V2.0040	*L. braziliensis*	58	182	239
LbrM30_V2.0060	*L. braziliensis*	170	92	261
LbrM30_V2.0060	*L. braziliensis*	219	543	761
LbrM30_V2.0070	*L. braziliensis*	42	142	183
LbrM30_V2.0090	*L. braziliensis*	55	1	55
LbrM30_V2.0100	*L. braziliensis*	203	1	203

LbrM30_V2.0130	*L. braziliensis*	49	573	621
LbrM30_V2.0140	*L. braziliensis*	43	473	515
LbrM30_V2.0150	*L. braziliensis*	40	1	40
LbrM30_V2.0200	*L. braziliensis*	98	237	334
LbrM30_V2.0200	*L. braziliensis*	48	379	426
LbrM30_V2.0200	*L. braziliensis*	48	379	426
LbrM30_V2.0220	*L. braziliensis*	53	13	65
LbrM30_V2.0230	*L. braziliensis*	120	184	303
LbrM30_V2.0230	*L. braziliensis*	108	325	432
LbrM30_V2.0250	*L. braziliensis*	61	341	401
LbrM30_V2.0250	*L. braziliensis*	60	504	563
LbrM30_V2.0250	*L. braziliensis*	60	504	563
LbrM30_V2.0260	*L. braziliensis*	59	406	464
LbrM30_V2.0260	*L. braziliensis*	67	1062	1128
LbrM30_V2.0260	*L. braziliensis*	67	1062	1128
LbrM30_V2.0270	*L. braziliensis*	69	142	210
LbrM30_V2.0270	*L. braziliensis*	53	313	365
LbrM30_V2.0270	*L. braziliensis*	81	389	469
LbrM30_V2.0270	*L. braziliensis*	60	582	641
LbrM30_V2.0280	*L. braziliensis*	62	334	395
LbrM30_V2.0290	*L. braziliensis*	41	33	73
LbrM30_V2.0300	*L. braziliensis*	45	633	677
LbrM30_V2.0300	*L. braziliensis*	72	1041	1112
LbrM30_V2.0300	*L. braziliensis*	84	1147	1230
LbrM30_V2.0310	*L. braziliensis*	68	15	82
LbrM30_V2.0320	*L. braziliensis*	60	251	310
LbrM30_V2.0320	*L. braziliensis*	320	359	678
LbrM30_V2.0320	*L. braziliensis*	122	880	1001
LbrM30_V2.0320	*L. braziliensis*	353	1011	1363
LbrM30_V2.0330	*L. braziliensis*	57	216	272
LbrM30_V2.0330	*L. braziliensis*	44	301	344
LbrM30_V2.0330	*L. braziliensis*	102	378	479
LbrM30_V2.0340	*L. braziliensis*	119	299	417
LbrM30_V2.0340	*L. braziliensis*	80	436	515
LbrM30_V2.0350	*L. braziliensis*	52	1	52
LbrM30_V2.0350	*L. braziliensis*	163	189	351
LbrM30_V2.0350	*L. braziliensis*	163	189	351
LbrM30_V2.0360	*L. braziliensis*	131	76	206
LbrM30_V2.0360	*L. braziliensis*	259	291	549
LbrM30_V2.0360	*L. braziliensis*	130	696	825
LbrM30_V2.0360	*L. braziliensis*	87	859	945
LbrM30_V2.0360	*L. braziliensis*	53	1261	1313
LbrM30_V2.0370	*L. braziliensis*	108	1	108
LbrM30_V2.0370	*L. braziliensis*	118	612	729
LbrM30_V2.0370	*L. braziliensis*	86	749	834
LbrM30_V2.0380	*L. braziliensis*	44	269	312
LbrM30_V2.0380	*L. braziliensis*	101	545	645

LbrM30_V2.0380	*L. braziliensis*	101	545	645
LbrM30_V2.0390	*L. braziliensis*	60	1	60
LbrM30_V2.0390	*L. braziliensis*	48	379	426
LbrM30_V2.0390	*L. braziliensis*	122	486	607
LbrM30_V2.0420	*L. braziliensis*	40	345	384
LbrM30_V2.0430	*L. braziliensis*	45	27	71
LbrM30_V2.0430	*L. braziliensis*	99	129	227
LbrM30_V2.0430	*L. braziliensis*	99	129	227
LbrM30_V2.0440	*L. braziliensis*	48	1	48
LbrM30_V2.0440	*L. braziliensis*	75	123	197
LbrM30_V2.0440	*L. braziliensis*	53	245	297
LbrM30_V2.0440	*L. braziliensis*	131	300	430
LbrM30_V2.0450	*L. braziliensis*	40	92	131
LbrM30_V2.0460	*L. braziliensis*	68	45	112
LbrM30_V2.0460	*L. braziliensis*	42	290	331
LbrM30_V2.0460	*L. braziliensis*	82	378	459
LbrM30_V2.0460	*L. braziliensis*	177	670	846
LbrM30_V2.0460	*L. braziliensis*	111	1026	1136
LbrM30_V2.0470	*L. braziliensis*	62	322	383
LbrM30_V2.0490	*L. braziliensis*	66	1	66
LbrM30_V2.0500	*L. braziliensis*	58	310	367
LbrM30_V2.0530	*L. braziliensis*	92	1	92
LbrM30_V2.0540	*L. braziliensis*	46	1	46
LbrM30_V2.0550	*L. braziliensis*	67	291	357
LbrM30_V2.0550	*L. braziliensis*	49	498	546
LbrM30_V2.0560	*L. braziliensis*	55	1	55
LbrM30_V2.0560	*L. braziliensis*	144	68	211
LbrM30_V2.0570	*L. braziliensis*	462	1	462
LbrM30_V2.0570	*L. braziliensis*	77	485	561
LbrM30_V2.0570	*L. braziliensis*	136	574	709
LbrM30_V2.0580	*L. braziliensis*	423	1	423
LbrM30_V2.0590	*L. braziliensis*	76	130	205
LbrM30_V2.0590	*L. braziliensis*	96	464	559
LbrM30_V2.0590	*L. braziliensis*	112	572	683
LbrM30_V2.0590	*L. braziliensis*	57	711	767
LbrM30_V2.0590	*L. braziliensis*	72	831	902
LbrM30_V2.0590	*L. braziliensis*	130	940	1069
LbrM30_V2.0590	*L. braziliensis*	41	1102	1142
LbrM30_V2.0600	*L. braziliensis*	48	694	741
LbrM30_V2.0630	*L. braziliensis*	75	535	609
LbrM30_V2.0640	*L. braziliensis*	273	54	326
LbrM30_V2.0650	*L. braziliensis*	136	1	136
LbrM30_V2.0650	*L. braziliensis*	129	350	478
LbrM30_V2.0680	*L. braziliensis*	209	48	256
LbrM30_V2.0680	*L. braziliensis*	94	262	355
LbrM30_V2.0680	*L. braziliensis*	291	627	917
LbrM30_V2.0690	*L. braziliensis*	80	1	80

LbrM30_V2.0700	*L. braziliensis*	126	91	216
LbrM30_V2.0710	*L. braziliensis*	66	1	66
LbrM30_V2.0730	*L. braziliensis*	49	11	59
LbrM30_V2.0740	*L. braziliensis*	73	1	73
LbrM30_V2.0770	*L. braziliensis*	56	45	100
LbrM30_V2.0800	*L. braziliensis*	56	491	546
LbrM30_V2.0820	*L. braziliensis*	98	1	98
LbrM30_V2.0850	*L. braziliensis*	107	629	735
LbrM30_V2.0850	*L. braziliensis*	47	868	914
LbrM30_V2.0850	*L. braziliensis*	91	1063	1153
LbrM30_V2.0850	*L. braziliensis*	200	1191	1390
LbrM30_V2.0850	*L. braziliensis*	93	1721	1813
LbrM30_V2.0850	*L. braziliensis*	132	1818	1949
LbrM30_V2.0860	*L. braziliensis*	138	23	160
LbrM30_V2.0880	*L. braziliensis*	262	881	1142
LbrM30_V2.0900	*L. braziliensis*	128	82	209
LbrM30_V2.0900	*L. braziliensis*	43	321	363
LbrM30_V2.0900	*L. braziliensis*	51	518	568
LbrM30_V2.0910	*L. braziliensis*	178	1	178
LbrM30_V2.0920	*L. braziliensis*	114	397	510
LbrM30_V2.0930	*L. braziliensis*	59	124	182
LbrM30_V2.0950	*L. braziliensis*	129	74	202
LbrM30_V2.0950	*L. braziliensis*	44	218	261
LbrM30_V2.0950	*L. braziliensis*	78	448	525
LbrM30_V2.0950	*L. braziliensis*	163	591	753
LbrM30_V2.0960	*L. braziliensis*	51	1	51
LbrM30_V2.0960	*L. braziliensis*	59	88	146
LbrM30_V2.0960	*L. braziliensis*	59	88	146
LbrM30_V2.0970	*L. braziliensis*	157	1	157
LbrM30_V2.0970	*L. braziliensis*	75	860	934
LbrM30_V2.1020	*L. braziliensis*	217	1	217
LbrM30_V2.1020	*L. braziliensis*	69	352	420
LbrM30_V2.1020	*L. braziliensis*	149	457	605
LbrM30_V2.1030	*L. braziliensis*	49	1	49
LbrM30_V2.1030	*L. braziliensis*	49	125	173
LbrM30_V2.1030	*L. braziliensis*	110	565	674
LbrM30_V2.1030	*L. braziliensis*	44	741	784
LbrM30_V2.1030	*L. braziliensis*	41	927	967
LbrM30_V2.1040	*L. braziliensis*	68	28	95
LbrM30_V2.1040	*L. braziliensis*	57	197	253
LbrM30_V2.1040	*L. braziliensis*	56	343	398
LbrM30_V2.1040	*L. braziliensis*	56	467	522
LbrM30_V2.1040	*L. braziliensis*	57	544	600
LbrM30_V2.1060	*L. braziliensis*	43	15	57
LbrM30_V2.1060	*L. braziliensis*	52	374	425
LbrM30_V2.1060	*L. braziliensis*	53	729	781
LbrM30_V2.1070	*L. braziliensis*	221	1	221

LbrM30_V2.1080	*L. braziliensis*	75	1	75
LbrM30_V2.1080	*L. braziliensis*	163	89	251
LbrM30_V2.1080	*L. braziliensis*	59	316	374
LbrM30_V2.1090	*L. braziliensis*	55	1	55
LbrM30_V2.1100	*L. braziliensis*	66	312	377
LbrM30_V2.1110	*L. braziliensis*	119	1	119
LbrM30_V2.1110	*L. braziliensis*	141	181	321
LbrM30_V2.1110	*L. braziliensis*	50	376	425
LbrM30_V2.1120	*L. braziliensis*	55	126	180
LbrM30_V2.1120	*L. braziliensis*	62	241	302
LbrM30_V2.1120	*L. braziliensis*	50	440	489
LbrM30_V2.1120	*L. braziliensis*	54	529	582
LbrM30_V2.1130	*L. braziliensis*	79	711	789
LbrM30_V2.1140	*L. braziliensis*	68	1	68
LbrM30_V2.1140	*L. braziliensis*	148	230	377
LbrM30_V2.1150	*L. braziliensis*	97	12	108
LbrM30_V2.1150	*L. braziliensis*	80	113	192
LbrM30_V2.1150	*L. braziliensis*	70	200	269
LbrM30_V2.1180	*L. braziliensis*	66	1	66
LbrM30_V2.1180	*L. braziliensis*	40	164	203
LbrM30_V2.1180	*L. braziliensis*	40	164	203
LbrM30_V2.1190	*L. braziliensis*	77	64	140
LbrM30_V2.1210	*L. braziliensis*	91	154	244
LbrM30_V2.1210	*L. braziliensis*	53	282	334
LbrM30_V2.1210	*L. braziliensis*	111	503	613
LbrM30_V2.1210	*L. braziliensis*	185	625	809
LbrM30_V2.1210	*L. braziliensis*	178	817	994
LbrM30_V2.1230	*L. braziliensis*	79	77	155
LbrM30_V2.1230	*L. braziliensis*	44	571	614
LbrM30_V2.1240	*L. braziliensis*	53	1	53
LbrM30_V2.1270	*L. braziliensis*	226	1	226
LbrM30_V2.1270	*L. braziliensis*	118	357	474
LbrM30_V2.1270	*L. braziliensis*	86	526	611
LbrM30_V2.1270	*L. braziliensis*	70	812	881
LbrM30_V2.1280	*L. braziliensis*	314	1	314
LbrM30_V2.1280	*L. braziliensis*	127	444	570
LbrM30_V2.1280	*L. braziliensis*	55	675	729
LbrM30_V2.1280	*L. braziliensis*	42	748	789
LbrM30_V2.1280	*L. braziliensis*	43	920	962
LbrM30_V2.1280	*L. braziliensis*	156	1121	1276
LbrM30_V2.1280	*L. braziliensis*	55	1467	1521
LbrM30_V2.1280	*L. braziliensis*	115	1548	1662
LbrM30_V2.1290	*L. braziliensis*	196	1	196
LbrM30_V2.1290	*L. braziliensis*	52	209	260
LbrM30_V2.1290	*L. braziliensis*	42	714	755
LbrM30_V2.1320	*L. braziliensis*	97	44	140
LbrM30_V2.1320	*L. braziliensis*	94	249	342

LbrM30_V2.1320	*L. braziliensis*	65	410	474
LbrM30_V2.1320	*L. braziliensis*	40	675	714
LbrM30_V2.1320	*L. braziliensis*	123	820	942
LbrM30_V2.1340	*L. braziliensis*	142	1	142
LbrM30_V2.1340	*L. braziliensis*	96	180	275
LbrM30_V2.1340	*L. braziliensis*	72	559	630
LbrM30_V2.1340	*L. braziliensis*	42	676	717
LbrM30_V2.1340	*L. braziliensis*	184	763	946
LbrM30_V2.1350	*L. braziliensis*	62	400	461
LbrM30_V2.1350	*L. braziliensis*	162	522	683
LbrM30_V2.1380	*L. braziliensis*	47	108	154
LbrM30_V2.1380	*L. braziliensis*	45	271	315
LbrM30_V2.1390	*L. braziliensis*	149	275	423
LbrM30_V2.1390	*L. braziliensis*	51	1372	1422
LbrM30_V2.1390	*L. braziliensis*	83	1537	1619
LbrM30_V2.1400	*L. braziliensis*	78	184	261
LbrM30_V2.1400	*L. braziliensis*	183	302	484
LbrM30_V2.1400	*L. braziliensis*	111	508	618
LbrM30_V2.1400	*L. braziliensis*	47	646	692
LbrM30_V2.1410	*L. braziliensis*	218	1	218
LbrM30_V2.1410	*L. braziliensis*	72	468	539
LbrM30_V2.1410	*L. braziliensis*	105	844	948
LbrM30_V2.1410	*L. braziliensis*	48	1013	1060
LbrM30_V2.1410	*L. braziliensis*	46	1258	1303
LbrM30_V2.1420	*L. braziliensis*	82	1	82
LbrM30_V2.1430	*L. braziliensis*	41	186	226
LbrM30_V2.1430	*L. braziliensis*	104	530	633
LbrM30_V2.1430	*L. braziliensis*	84	895	978
LbrM30_V2.1430	*L. braziliensis*	120	1008	1127
LbrM30_V2.1430	*L. braziliensis*	87	1149	1235
LbrM30_V2.1440	*L. braziliensis*	461	1	461
LbrM30_V2.1440	*L. braziliensis*	148	542	689
LbrM30_V2.1440	*L. braziliensis*	116	691	806
LbrM30_V2.1440	*L. braziliensis*	92	867	958
LbrM30_V2.1440	*L. braziliensis*	100	969	1068
LbrM30_V2.1440	*L. braziliensis*	61	1102	1162
LbrM30_V2.1440	*L. braziliensis*	52	1312	1363
LbrM30_V2.1440	*L. braziliensis*	183	1424	1606
LbrM30_V2.1440	*L. braziliensis*	71	1617	1687
LbrM30_V2.1440	*L. braziliensis*	236	1789	2024
LbrM30_V2.1440	*L. braziliensis*	84	2044	2127
LbrM30_V2.1450	*L. braziliensis*	46	148	193
LbrM30_V2.1460	*L. braziliensis*	198	121	318
LbrM30_V2.1470	*L. braziliensis*	163	15	177
LbrM30_V2.1470	*L. braziliensis*	200	268	467
LbrM30_V2.1480	*L. braziliensis*	177	310	486
LbrM30_V2.1490	*L. braziliensis*	169	12	180

LbrM30_V2.1490	*L. braziliensis*	45	275	319
LbrM30_V2.1490	*L. braziliensis*	182	327	508
LbrM30_V2.1490	*L. braziliensis*	155	511	665
LbrM30_V2.1510	*L. braziliensis*	150	1	150
LbrM30_V2.1520	*L. braziliensis*	46	1	46
LbrM30_V2.1530	*L. braziliensis*	51	1	51
LbrM30_V2.1540	*L. braziliensis*	117	42	158
LbrM30_V2.1550	*L. braziliensis*	198	411	608
LbrM30_V2.1550	*L. braziliensis*	274	667	940
LbrM30_V2.1570	*L. braziliensis*	74	155	228
LbrM30_V2.1570	*L. braziliensis*	58	395	452
LbrM30_V2.1570	*L. braziliensis*	58	395	452
LbrM30_V2.1580	*L. braziliensis*	51	130	180
LbrM30_V2.1580	*L. braziliensis*	54	320	373
LbrM30_V2.1580	*L. braziliensis*	147	461	607
LbrM30_V2.1580	*L. braziliensis*	44	819	862
LbrM30_V2.1580	*L. braziliensis*	100	919	1018
LbrM30_V2.1590	*L. braziliensis*	42	196	237
LbrM30_V2.1590	*L. braziliensis*	83	293	375
LbrM30_V2.1610	*L. braziliensis*	104	396	499
LbrM30_V2.1620	*L. braziliensis*	118	56	173
LbrM30_V2.1620	*L. braziliensis*	93	182	274
LbrM30_V2.1620	*L. braziliensis*	44	337	380
LbrM30_V2.1620	*L. braziliensis*	98	438	535
LbrM30_V2.1620	*L. braziliensis*	128	560	687
LbrM30_V2.1620	*L. braziliensis*	70	870	939
LbrM30_V2.1620	*L. braziliensis*	243	1036	1278
LbrM30_V2.1620	*L. braziliensis*	113	1359	1471
LbrM30_V2.1620	*L. braziliensis*	52	1496	1547
LbrM30_V2.1630	*L. braziliensis*	43	64	106
LbrM30_V2.1640	*L. braziliensis*	56	158	213
LbrM30_V2.1650	*L. braziliensis*	47	104	150
LbrM30_V2.1660	*L. braziliensis*	41	41	81
LbrM30_V2.1660	*L. braziliensis*	56	263	318
LbrM30_V2.1660	*L. braziliensis*	109	374	482
LbrM30_V2.1670	*L. braziliensis*	55	190	244
LbrM30_V2.1670	*L. braziliensis*	154	895	1048
LbrM30_V2.1680	*L. braziliensis*	134	18	151
LbrM30_V2.1680	*L. braziliensis*	49	510	558
LbrM30_V2.1680	*L. braziliensis*	87	610	696
LbrM30_V2.1710	*L. braziliensis*	59	33	91
LbrM30_V2.1710	*L. braziliensis*	55	115	169
LbrM30_V2.1710	*L. braziliensis*	55	115	169
LbrM30_V2.1730	*L. braziliensis*	72	86	157
LbrM30_V2.1730	*L. braziliensis*	62	190	251
LbrM30_V2.1730	*L. braziliensis*	59	278	336
LbrM30_V2.1740	*L. braziliensis*	79	46	124

LbrM30_V2.1740	*L. braziliensis*	95	193	287
LbrM30_V2.1740	*L. braziliensis*	40	600	639
LbrM30_V2.1740	*L. braziliensis*	79	657	735
LbrM30_V2.1770	*L. braziliensis*	66	1	66
LbrM30_V2.1770	*L. braziliensis*	140	106	245
LbrM30_V2.1770	*L. braziliensis*	130	262	391
LbrM30_V2.1800	*L. braziliensis*	174	388	561
LbrM30_V2.1800	*L. braziliensis*	135	775	909
LbrM30_V2.1800	*L. braziliensis*	188	1121	1308
LbrM30_V2.1800	*L. braziliensis*	193	1561	1753
LbrM30_V2.1820	*L. braziliensis*	53	69	121
LbrM30_V2.1820	*L. braziliensis*	141	157	297
LbrM30_V2.1820	*L. braziliensis*	502	309	810
LbrM30_V2.1820	*L. braziliensis*	144	830	973
LbrM30_V2.1850	*L. braziliensis*	83	356	438
LbrM30_V2.1850	*L. braziliensis*	107	513	619
LbrM30_V2.1850	*L. braziliensis*	79	683	761
LbrM30_V2.1860	*L. braziliensis*	57	1	57
LbrM30_V2.1860	*L. braziliensis*	50	133	182
LbrM30_V2.1860	*L. braziliensis*	86	263	348
LbrM30_V2.1860	*L. braziliensis*	61	537	597
LbrM30_V2.1880	*L. braziliensis*	46	35	80
LbrM30_V2.1880	*L. braziliensis*	114	136	249
LbrM30_V2.1880	*L. braziliensis*	111	298	408
LbrM30_V2.1880	*L. braziliensis*	104	653	756
LbrM30_V2.1880	*L. braziliensis*	383	789	1171
LbrM30_V2.1890	*L. braziliensis*	83	132	214
LbrM30_V2.1890	*L. braziliensis*	44	319	362
LbrM30_V2.1890	*L. braziliensis*	44	319	362
LbrM30_V2.1900	*L. braziliensis*	52	100	151
LbrM30_V2.1900	*L. braziliensis*	120	262	381
LbrM30_V2.1900	*L. braziliensis*	120	262	381
LbrM30_V2.1910	*L. braziliensis*	88	112	199
LbrM30_V2.1910	*L. braziliensis*	185	226	410
LbrM30_V2.1910	*L. braziliensis*	185	226	410
LbrM30_V2.1920	*L. braziliensis*	49	138	186
LbrM30_V2.1940	*L. braziliensis*	56	54	109
LbrM30_V2.1940	*L. braziliensis*	79	277	355
LbrM30_V2.1940	*L. braziliensis*	42	547	588
LbrM30_V2.1940	*L. braziliensis*	44	627	670
LbrM30_V2.1940	*L. braziliensis*	59	899	957
LbrM30_V2.1950	*L. braziliensis*	56	74	129
LbrM30_V2.1980	*L. braziliensis*	63	151	213
LbrM30_V2.1980	*L. braziliensis*	68	717	784
LbrM30_V2.1980	*L. braziliensis*	47	1050	1096
LbrM30_V2.2000	*L. braziliensis*	50	124	173
LbrM30_V2.2000	*L. braziliensis*	55	235	289

LbrM30_V2.2000	*L. braziliensis*	91	536	626
LbrM30_V2.2000	*L. braziliensis*	249	844	1092
LbrM30_V2.2010	*L. braziliensis*	240	1	240
LbrM30_V2.2020	*L. braziliensis*	89	120	208
LbrM30_V2.2030	*L. braziliensis*	54	1	54
LbrM30_V2.2030	*L. braziliensis*	113	439	551
LbrM30_V2.2030	*L. braziliensis*	113	439	551
LbrM30_V2.2050	*L. braziliensis*	87	235	321
LbrM30_V2.2050	*L. braziliensis*	59	536	594
LbrM30_V2.2050	*L. braziliensis*	59	670	728
LbrM30_V2.2060	*L. braziliensis*	46	152	197
LbrM30_V2.2080	*L. braziliensis*	70	80	149
LbrM30_V2.2080	*L. braziliensis*	120	418	537
LbrM30_V2.2080	*L. braziliensis*	102	636	737
LbrM30_V2.2090	*L. braziliensis*	70	116	185
LbrM30_V2.2090	*L. braziliensis*	53	208	260
LbrM30_V2.2090	*L. braziliensis*	108	290	397
LbrM30_V2.2100	*L. braziliensis*	73	394	466
LbrM30_V2.2110	*L. braziliensis*	57	420	476
LbrM30_V2.2110	*L. braziliensis*	114	505	618
LbrM30_V2.2120	*L. braziliensis*	48	1	48
LbrM30_V2.2130	*L. braziliensis*	320	173	492
LbrM30_V2.2140	*L. braziliensis*	209	172	380
LbrM30_V2.2150	*L. braziliensis*	87	62	148
LbrM30_V2.2160	*L. braziliensis*	40	749	788
LbrM30_V2.2160	*L. braziliensis*	53	1839	1891
LbrM30_V2.2160	*L. braziliensis*	44	2401	2444
LbrM30_V2.2170	*L. braziliensis*	108	1	108
LbrM30_V2.2170	*L. braziliensis*	78	138	215
LbrM30_V2.2170	*L. braziliensis*	60	271	330
LbrM30_V2.2180	*L. braziliensis*	70	74	143
LbrM30_V2.2200	*L. braziliensis*	74	81	154
LbrM30_V2.2200	*L. braziliensis*	76	252	327
LbrM30_V2.2200	*L. braziliensis*	76	252	327
LbrM30_V2.2220	*L. braziliensis*	103	220	322
LbrM30_V2.2220	*L. braziliensis*	53	512	564
LbrM30_V2.2220	*L. braziliensis*	158	582	739
LbrM30_V2.2220	*L. braziliensis*	44	804	847
LbrM30_V2.2220	*L. braziliensis*	60	955	1014
LbrM30_V2.2220	*L. braziliensis*	68	1579	1646
LbrM30_V2.2220	*L. braziliensis*	43	1649	1691
LbrM30_V2.2230	*L. braziliensis*	64	19	82
LbrM30_V2.2230	*L. braziliensis*	49	341	389
LbrM30_V2.2230	*L. braziliensis*	110	576	685
LbrM30_V2.2240	*L. braziliensis*	109	1	109
LbrM30_V2.2240	*L. braziliensis*	67	457	523
LbrM30_V2.2240	*L. braziliensis*	46	714	759

LbrM30_V2.2250	*L. braziliensis*	41	116	156
LbrM30_V2.2250	*L. braziliensis*	72	161	232
LbrM30_V2.2250	*L. braziliensis*	52	386	437
LbrM30_V2.2250	*L. braziliensis*	77	517	593
LbrM30_V2.2250	*L. braziliensis*	135	860	994
LbrM30_V2.2250	*L. braziliensis*	112	1036	1147
LbrM30_V2.2250	*L. braziliensis*	62	1248	1309
LbrM30_V2.2260	*L. braziliensis*	43	1	43
LbrM30_V2.2280	*L. braziliensis*	49	245	293
LbrM30_V2.2290	*L. braziliensis*	93	77	169
LbrM30_V2.2290	*L. braziliensis*	56	180	235
LbrM30_V2.2290	*L. braziliensis*	141	280	420
LbrM30_V2.2290	*L. braziliensis*	131	442	572
LbrM30_V2.2290	*L. braziliensis*	133	612	744
LbrM30_V2.2290	*L. braziliensis*	268	764	1031
LbrM30_V2.2290	*L. braziliensis*	111	1067	1177
LbrM30_V2.2330	*L. braziliensis*	154	12	165
LbrM30_V2.2330	*L. braziliensis*	44	202	245
LbrM30_V2.2330	*L. braziliensis*	44	202	245
LbrM30_V2.2340	*L. braziliensis*	50	266	315
LbrM30_V2.2340	*L. braziliensis*	162	843	1004
LbrM30_V2.2340	*L. braziliensis*	50	1032	1081
LbrM30_V2.2340	*L. braziliensis*	64	1250	1313
LbrM30_V2.2340	*L. braziliensis*	87	1447	1533
LbrM30_V2.2340	*L. braziliensis*	90	2159	2248
LbrM30_V2.2340	*L. braziliensis*	40	2510	2549
LbrM30_V2.2340	*L. braziliensis*	41	2602	2642
LbrM30_V2.2340	*L. braziliensis*	51	2674	2724
LbrM30_V2.2340	*L. braziliensis*	92	2908	2999
LbrM30_V2.2380	*L. braziliensis*	60	269	328
LbrM30_V2.2380	*L. braziliensis*	891	334	1224
LbrM30_V2.2390	*L. braziliensis*	41	131	171
LbrM30_V2.2390	*L. braziliensis*	90	386	475
LbrM30_V2.2390	*L. braziliensis*	57	515	571
LbrM30_V2.2390	*L. braziliensis*	71	668	738
LbrM30_V2.2400	*L. braziliensis*	93	70	162
LbrM30_V2.2410	*L. braziliensis*	90	210	299
LbrM30_V2.2420	*L. braziliensis*	138	514	651
LbrM30_V2.2430	*L. braziliensis*	126	231	356
LbrM30_V2.2440	*L. braziliensis*	145	514	658
LbrM30_V2.2490	*L. braziliensis*	186	1	186
LbrM30_V2.2490	*L. braziliensis*	46	436	481
LbrM30_V2.2510	*L. braziliensis*	193	20	212
LbrM30_V2.2510	*L. braziliensis*	81	324	404
LbrM30_V2.2510	*L. braziliensis*	44	504	547
LbrM30_V2.2510	*L. braziliensis*	72	635	706
LbrM30_V2.2520	*L. braziliensis*	85	104	188

LbrM30_V2.2520	*L. braziliensis*	49	338	386
LbrM30_V2.2520	*L. braziliensis*	49	338	386
LbrM30_V2.2560	*L. braziliensis*	75	1	75
LbrM30_V2.2560	*L. braziliensis*	64	151	214
LbrM30_V2.2570	*L. braziliensis*	370	1	370
LbrM30_V2.2570	*L. braziliensis*	112	601	712
LbrM30_V2.2600	*L. braziliensis*	84	171	254
LbrM30_V2.2600	*L. braziliensis*	343	486	828
LbrM30_V2.2630	*L. braziliensis*	53	57	109
LbrM30_V2.2650	*L. braziliensis*	113	58	170
LbrM30_V2.2650	*L. braziliensis*	104	191	294
LbrM30_V2.2670	*L. braziliensis*	548	17	564
LbrM30_V2.2670	*L. braziliensis*	61	596	656
LbrM30_V2.2670	*L. braziliensis*	192	707	898
LbrM30_V2.2680	*L. braziliensis*	159	139	297
LbrM30_V2.2680	*L. braziliensis*	55	501	555
LbrM30_V2.2690	*L. braziliensis*	163	224	386
LbrM30_V2.2700	*L. braziliensis*	102	43	144
LbrM30_V2.2700	*L. braziliensis*	117	228	344
LbrM30_V2.2700	*L. braziliensis*	117	228	344
LbrM30_V2.2710	*L. braziliensis*	56	179	234
LbrM30_V2.2750	*L. braziliensis*	97	99	195
LbrM30_V2.2750	*L. braziliensis*	59	226	284
LbrM30_V2.2750	*L. braziliensis*	44	322	365
LbrM30_V2.2750	*L. braziliensis*	77	392	468
LbrM30_V2.2760	*L. braziliensis*	75	1	75
LbrM30_V2.2770	*L. braziliensis*	95	17	111
LbrM30_V2.2800	*L. braziliensis*	564	1	564
LbrM30_V2.2800	*L. braziliensis*	196	575	770
LbrM30_V2.2840	*L. braziliensis*	61	72	132
LbrM30_V2.2850	*L. braziliensis*	44	103	146
LbrM30_V2.2850	*L. braziliensis*	58	218	275
LbrM30_V2.2850	*L. braziliensis*	104	383	486
LbrM30_V2.2860	*L. braziliensis*	83	37	119
LbrM30_V2.2870	*L. braziliensis*	51	408	458
LbrM30_V2.2900	*L. braziliensis*	359	283	641
LbrM30_V2.2940	*L. braziliensis*	50	135	184
LbrM30_V2.2980	*L. braziliensis*	253	155	407
LbrM30_V2.3000	*L. braziliensis*	122	201	322
LbrM30_V2.3020	*L. braziliensis*	77	185	261
LbrM30_V2.3020	*L. braziliensis*	61	391	451
LbrM30_V2.3030	*L. braziliensis*	87	51	137
LbrM30_V2.3040	*L. braziliensis*	44	1512	1555
LbrM30_V2.3040	*L. braziliensis*	65	1815	1879
LbrM30_V2.3040	*L. braziliensis*	65	1815	1879
LbrM30_V2.3050	*L. braziliensis*	79	1	79
LbrM30_V2.3050	*L. braziliensis*	133	431	563

LbrM30_V2.3050	*L. braziliensis*	255	638	892
LbrM30_V2.3050	*L. braziliensis*	42	929	970
LbrM30_V2.3050	*L. braziliensis*	43	994	1036
LbrM30_V2.3050	*L. braziliensis*	125	1067	1191
LbrM30_V2.3060	*L. braziliensis*	119	1	119
LbrM30_V2.3060	*L. braziliensis*	65	318	382
LbrM30_V2.3080	*L. braziliensis*	146	132	277
LbrM30_V2.3090	*L. braziliensis*	57	268	324
LbrM30_V2.3130	*L. braziliensis*	108	1	108
LbrM30_V2.3160	*L. braziliensis*	384	1	384
LbrM30_V2.3190	*L. braziliensis*	63	44	106
LbrM30_V2.3190	*L. braziliensis*	44	281	324
LbrM30_V2.3190	*L. braziliensis*	89	369	457
LbrM30_V2.3190	*L. braziliensis*	177	668	844
LbrM30_V2.3190	*L. braziliensis*	111	1024	1134
LbrM30_V2.3190	*L. braziliensis*	40	1351	1390
LbrM30_V2.3190	*L. braziliensis*	58	1925	1982
LbrM30_V2.3200	*L. braziliensis*	171	201	371
LbrM30_V2.3200	*L. braziliensis*	111	551	661
LbrM30_V2.3200	*L. braziliensis*	56	1454	1509
LbrM30_V2.3210	*L. braziliensis*	203	1	203
LbrM30_V2.3210	*L. braziliensis*	111	383	493
LbrM30_V2.3210	*L. braziliensis*	40	710	749
LbrM30_V2.3210	*L. braziliensis*	58	1285	1342
LbrM30_V2.3220	*L. braziliensis*	84	1	84
LbrM30_V2.3220	*L. braziliensis*	162	98	259
LbrM30_V2.3220	*L. braziliensis*	58	771	828
LbrM30_V2.3240	*L. braziliensis*	107	303	409
LbrM30_V2.3240	*L. braziliensis*	192	442	633
LbrM30_V2.3240	*L. braziliensis*	153	729	881
LbrM30_V2.3290	*L. braziliensis*	151	1	151
LbrM30_V2.3290	*L. braziliensis*	124	446	569
LbrM30_V2.3300	*L. braziliensis*	60	353	412
LbrM30_V2.3330	*L. braziliensis*	44	128	171
LbrM30_V2.3330	*L. braziliensis*	64	240	303
LbrM30_V2.3330	*L. braziliensis*	97	466	562
LbrM30_V2.3330	*L. braziliensis*	160	878	1037
LbrM30_V2.3340	*L. braziliensis*	98	69	166
LbrM30_V2.3340	*L. braziliensis*	52	218	269
LbrM30_V2.3340	*L. braziliensis*	40	319	358
LbrM30_V2.3360	*L. braziliensis*	58	1	58
LbrM30_V2.3360	*L. braziliensis*	148	228	375
LbrM30_V2.3370	*L. braziliensis*	186	60	245
LbrM30_V2.3370	*L. braziliensis*	88	253	340
LbrM30_V2.3400	*L. braziliensis*	132	509	640
LbrM30_V2.3410	*L. braziliensis*	483	37	519
LbrM30_V2.3420	*L. braziliensis*	58	174	231

LbrM30_V2.3450	*L. braziliensis*	40	74	113
LbrM30_V2.3450	*L. braziliensis*	101	231	331
LbrM30_V2.3450	*L. braziliensis*	101	231	331
LbrM30_V2.3460	*L. braziliensis*	63	1	63
LbrM30_V2.3460	*L. braziliensis*	67	226	292
LbrM30_V2.3460	*L. braziliensis*	125	316	440
LbrM30_V2.3470	*L. braziliensis*	54	746	799
LbrM30_V2.3480	*L. braziliensis*	65	10	74
LbrM30_V2.3480	*L. braziliensis*	46	217	262
LbrM30_V2.3490	*L. braziliensis*	51	1	51
LbrM30_V2.3520	*L. braziliensis*	70	322	391
LbrM30_V2.3530	*L. braziliensis*	103	63	165
LbrM30_V2.3540	*L. braziliensis*	207	29	235
LbrM30_V2.3570	*L. braziliensis*	64	78	141
LbrM30_V2.3570	*L. braziliensis*	48	179	226
LbrM30_V2.3570	*L. braziliensis*	48	179	226
LbrM30_V2.3580	*L. braziliensis*	84	277	360
LbrM30_V2.3600	*L. braziliensis*	46	1	46
LbrM30_V2.3600	*L. braziliensis*	51	88	138
LbrM30_V2.3600	*L. braziliensis*	55	160	214
LbrM30_V2.3600	*L. braziliensis*	50	218	267
LbrM30_V2.3610	*L. braziliensis*	141	1	141
LbrM30_V2.3610	*L. braziliensis*	85	296	380
LbrM30_V2.3610	*L. braziliensis*	64	553	616
LbrM30_V2.3610	*L. braziliensis*	85	658	742
LbrM30_V2.3620	*L. braziliensis*	49	96	144
LbrM30_V2.3650	*L. braziliensis*	196	1	196
LbrM30_V2.3650	*L. braziliensis*	114	202	315
LbrM30_V2.3680	*L. braziliensis*	62	39	100
LbrM30_V2.3680	*L. braziliensis*	47	158	204
LbrM30_V2.3680	*L. braziliensis*	47	158	204
LbrM30_V2.3710	*L. braziliensis*	47	1	47
LbrM30_V2.3710	*L. braziliensis*	77	366	442
LbrM30_V2.3710	*L. braziliensis*	77	366	442
LbrM30_V2.3720	*L. braziliensis*	76	68	143
LbrM30_V2.3720	*L. braziliensis*	51	534	584
LbrM30_V2.3720	*L. braziliensis*	51	534	584
LbrM30_V2.3730	*L. braziliensis*	40	45	84
LbrM30_V2.3740	*L. braziliensis*	74	1	74
LbrM30_V2.3760	*L. braziliensis*	59	47	105
LbrM30_V2.3770	*L. braziliensis*	92	131	222
LbrM30_V2.3780	*L. braziliensis*	58	447	504
LbrM30_V2.3790	*L. braziliensis*	58	1	58
LbrM30_V2.3790	*L. braziliensis*	69	94	162
LbrM30_V2.3790	*L. braziliensis*	161	258	418
LbrM30_V2.3790	*L. braziliensis*	172	628	799
LbrM30_V2.3790	*L. braziliensis*	110	981	1090

LbrM30_V2.3790	*L. braziliensis*	43	1303	1345
LbrM30_V2.3790	*L. braziliensis*	58	1880	1937
LbrM30_V2.3800	*L. braziliensis*	91	1	91
LbrM30_V2.3800	*L. braziliensis*	121	243	363
LbrM30_V2.3800	*L. braziliensis*	108	535	642
LbrM30_V2.3800	*L. braziliensis*	58	1154	1211
LbrM30_V2.3810	*L. braziliensis*	79	70	148
LbrM30_V2.3810	*L. braziliensis*	40	175	214
LbrM30_V2.3810	*L. braziliensis*	47	254	300
LbrM30_V2.3810	*L. braziliensis*	90	339	428
LbrM30_V2.3810	*L. braziliensis*	109	585	693
LbrM31_V2.0030	*L. braziliensis*	64	952	1015
LbrM31_V2.0040	*L. braziliensis*	61	12	72
LbrM31_V2.0070	*L. braziliensis*	82	869	950
LbrM31_V2.0080	*L. braziliensis*	126	1	126
LbrM31_V2.0080	*L. braziliensis*	70	161	230
LbrM31_V2.0090	*L. braziliensis*	47	305	351
LbrM31_V2.0110	*L. braziliensis*	104	176	279
LbrM31_V2.0120	*L. braziliensis*	48	1	48
LbrM31_V2.0130	*L. braziliensis*	810	1	810
LbrM31_V2.0140	*L. braziliensis*	59	66	124
LbrM31_V2.0140	*L. braziliensis*	49	369	417
LbrM31_V2.0140	*L. braziliensis*	49	369	417
LbrM31_V2.0160	*L. braziliensis*	81	136	216
LbrM31_V2.0160	*L. braziliensis*	125	237	361
LbrM31_V2.0160	*L. braziliensis*	123	473	595
LbrM31_V2.0180	*L. braziliensis*	148	198	345
LbrM31_V2.0180	*L. braziliensis*	69	460	528
LbrM31_V2.0180	*L. braziliensis*	195	569	763
LbrM31_V2.0180	*L. braziliensis*	64	784	847
LbrM31_V2.0180	*L. braziliensis*	49	1074	1122
LbrM31_V2.0200	*L. braziliensis*	386	45	430
LbrM31_V2.0210	*L. braziliensis*	79	161	239
LbrM31_V2.0210	*L. braziliensis*	57	412	468
LbrM31_V2.0210	*L. braziliensis*	92	494	585
LbrM31_V2.0240	*L. braziliensis*	51	12	62
LbrM31_V2.0240	*L. braziliensis*	77	66	142
LbrM31_V2.0240	*L. braziliensis*	62	151	212
LbrM31_V2.0240	*L. braziliensis*	54	238	291
LbrM31_V2.0250	*L. braziliensis*	163	1	163
LbrM31_V2.0260	*L. braziliensis*	114	238	351
LbrM31_V2.0280	*L. braziliensis*	44	141	184
LbrM31_V2.0280	*L. braziliensis*	44	467	510
LbrM31_V2.0280	*L. braziliensis*	44	467	510
LbrM31_V2.0300	*L. braziliensis*	212	1	212
LbrM31_V2.0300	*L. braziliensis*	102	524	625
LbrM31_V2.0330	*L. braziliensis*	118	1	118

LbrM31_V2.0340	*L. braziliensis*	185	14	198
LbrM31_V2.0340	*L. braziliensis*	178	259	436
LbrM31_V2.0340	*L. braziliensis*	186	456	641
LbrM31_V2.0340	*L. braziliensis*	109	666	774
LbrM31_V2.0350	*L. braziliensis*	59	75	133
LbrM31_V2.0360	*L. braziliensis*	325	1	325
LbrM31_V2.0400	*L. braziliensis*	156	93	248
LbrM31_V2.0400	*L. braziliensis*	212	560	771
LbrM31_V2.0400	*L. braziliensis*	69	813	881
LbrM31_V2.0420	*L. braziliensis*	85	279	363
LbrM31_V2.0430	*L. braziliensis*	114	1	114
LbrM31_V2.0440	*L. braziliensis*	79	1	79
LbrM31_V2.0450	*L. braziliensis*	60	217	276
LbrM31_V2.0450	*L. braziliensis*	159	325	483
LbrM31_V2.0460	*L. braziliensis*	86	1	86
LbrM31_V2.0470	*L. braziliensis*	88	1	88
LbrM31_V2.0480	*L. braziliensis*	69	255	323
LbrM31_V2.0480	*L. braziliensis*	49	914	962
LbrM31_V2.0480	*L. braziliensis*	49	914	962
LbrM31_V2.0490	*L. braziliensis*	47	1	47
LbrM31_V2.0490	*L. braziliensis*	48	76	123
LbrM31_V2.0490	*L. braziliensis*	46	147	192
LbrM31_V2.0490	*L. braziliensis*	42	326	367
LbrM31_V2.0490	*L. braziliensis*	44	635	678
LbrM31_V2.0500	*L. braziliensis*	66	1	66
LbrM31_V2.0500	*L. braziliensis*	42	121	162
LbrM31_V2.0500	*L. braziliensis*	56	302	357
LbrM31_V2.0500	*L. braziliensis*	41	391	431
LbrM31_V2.0500	*L. braziliensis*	68	604	671
LbrM31_V2.0500	*L. braziliensis*	330	738	1067
LbrM31_V2.0500	*L. braziliensis*	53	1109	1161
LbrM31_V2.0500	*L. braziliensis*	63	1339	1401
LbrM31_V2.0510	*L. braziliensis*	118	73	190
LbrM31_V2.0520	*L. braziliensis*	123	1	123
LbrM31_V2.0520	*L. braziliensis*	452	139	590
LbrM31_V2.0520	*L. braziliensis*	86	656	741
LbrM31_V2.0530	*L. braziliensis*	44	1	44
LbrM31_V2.0530	*L. braziliensis*	53	131	183
LbrM31_V2.0540	*L. braziliensis*	54	50	103
LbrM31_V2.0570	*L. braziliensis*	75	1	75
LbrM31_V2.0580	*L. braziliensis*	132	1	132
LbrM31_V2.0580	*L. braziliensis*	106	785	890
LbrM31_V2.0580	*L. braziliensis*	106	785	890
LbrM31_V2.0590	*L. braziliensis*	103	21	123
LbrM31_V2.0590	*L. braziliensis*	44	174	217
LbrM31_V2.0590	*L. braziliensis*	74	766	839
LbrM31_V2.0640	*L. braziliensis*	91	147	237

LbrM31_V2.0650	*L. braziliensis*	108	34	141
LbrM31_V2.0650	*L. braziliensis*	75	453	527
LbrM31_V2.0660	*L. braziliensis*	137	1	137
LbrM31_V2.0660	*L. braziliensis*	85	141	225
LbrM31_V2.0660	*L. braziliensis*	58	231	288
LbrM31_V2.0660	*L. braziliensis*	102	361	462
LbrM31_V2.0660	*L. braziliensis*	42	476	517
LbrM31_V2.0660	*L. braziliensis*	61	557	617
LbrM31_V2.0670	*L. braziliensis*	70	32	101
LbrM31_V2.0670	*L. braziliensis*	66	212	277
LbrM31_V2.0670	*L. braziliensis*	65	531	595
LbrM31_V2.0680	*L. braziliensis*	155	73	227
LbrM31_V2.0690	*L. braziliensis*	165	1	165
LbrM31_V2.0690	*L. braziliensis*	154	213	366
LbrM31_V2.0690	*L. braziliensis*	164	372	535
LbrM31_V2.0690	*L. braziliensis*	86	589	674
LbrM31_V2.0700	*L. braziliensis*	84	42	125
LbrM31_V2.0700	*L. braziliensis*	84	753	836
LbrM31_V2.0710	*L. braziliensis*	143	1	143
LbrM31_V2.0710	*L. braziliensis*	74	203	276
LbrM31_V2.0710	*L. braziliensis*	90	331	420
LbrM31_V2.0710	*L. braziliensis*	82	430	511
LbrM31_V2.0710	*L. braziliensis*	178	576	753
LbrM31_V2.0710	*L. braziliensis*	59	1068	1126
LbrM31_V2.0730	*L. braziliensis*	62	1	62
LbrM31_V2.0740	*L. braziliensis*	52	48	99
LbrM31_V2.0750	*L. braziliensis*	116	1	116
LbrM31_V2.0770	*L. braziliensis*	103	30	132
LbrM31_V2.0770	*L. braziliensis*	96	197	292
LbrM31_V2.0770	*L. braziliensis*	71	375	445
LbrM31_V2.0780	*L. braziliensis*	96	1	96
LbrM31_V2.0780	*L. braziliensis*	134	126	259
LbrM31_V2.0780	*L. braziliensis*	40	424	463
LbrM31_V2.0790	*L. braziliensis*	86	85	170
LbrM31_V2.0790	*L. braziliensis*	79	248	326
LbrM31_V2.0790	*L. braziliensis*	61	365	425
LbrM31_V2.0790	*L. braziliensis*	56	632	687
LbrM31_V2.0800	*L. braziliensis*	93	48	140
LbrM31_V2.0800	*L. braziliensis*	69	254	322
LbrM31_V2.0800	*L. braziliensis*	53	457	509
LbrM31_V2.0810	*L. braziliensis*	46	41	86
LbrM31_V2.0810	*L. braziliensis*	47	342	388
LbrM31_V2.0810	*L. braziliensis*	45	435	479
LbrM31_V2.0810	*L. braziliensis*	63	679	741
LbrM31_V2.0830	*L. braziliensis*	45	1	45
LbrM31_V2.0830	*L. braziliensis*	130	72	201
LbrM31_V2.0830	*L. braziliensis*	76	255	330

LbrM31_V2.0830	*L. braziliensis*	80	362	441
LbrM31_V2.0830	*L. braziliensis*	60	448	507
LbrM31_V2.0830	*L. braziliensis*	181	553	733
LbrM31_V2.0830	*L. braziliensis*	70	863	932
LbrM31_V2.0830	*L. braziliensis*	339	935	1273
LbrM31_V2.0830	*L. braziliensis*	160	1640	1799
LbrM31_V2.0840	*L. braziliensis*	489	1	489
LbrM31_V2.0840	*L. braziliensis*	148	572	719
LbrM31_V2.0850	*L. braziliensis*	143	125	267
LbrM31_V2.0860	*L. braziliensis*	124	112	235
LbrM31_V2.0860	*L. braziliensis*	41	320	360
LbrM31_V2.0860	*L. braziliensis*	97	388	484
LbrM31_V2.0870	*L. braziliensis*	110	83	192
LbrM31_V2.0870	*L. braziliensis*	54	228	281
LbrM31_V2.0870	*L. braziliensis*	51	353	403
LbrM31_V2.0870	*L. braziliensis*	106	667	772
LbrM31_V2.0880	*L. braziliensis*	97	62	158
LbrM31_V2.0910	*L. braziliensis*	134	1	134
LbrM31_V2.0910	*L. braziliensis*	248	432	679
LbrM31_V2.0930	*L. braziliensis*	113	60	172
LbrM31_V2.0940	*L. braziliensis*	77	92	168
LbrM31_V2.0950	*L. braziliensis*	71	102	172
LbrM31_V2.0950	*L. braziliensis*	127	223	349
LbrM31_V2.0950	*L. braziliensis*	77	360	436
LbrM31_V2.0960	*L. braziliensis*	90	149	238
LbrM31_V2.0970	*L. braziliensis*	135	138	272
LbrM31_V2.0980	*L. braziliensis*	84	1	84
LbrM31_V2.0980	*L. braziliensis*	108	189	296
LbrM31_V2.0990	*L. braziliensis*	60	1	60
LbrM31_V2.0990	*L. braziliensis*	70	140	209
LbrM31_V2.1020	*L. braziliensis*	47	187	233
LbrM31_V2.1020	*L. braziliensis*	66	306	371
LbrM31_V2.1020	*L. braziliensis*	221	389	609
LbrM31_V2.1030	*L. braziliensis*	62	1	62
LbrM31_V2.1040	*L. braziliensis*	44	1	44
LbrM31_V2.1040	*L. braziliensis*	65	94	158
LbrM31_V2.1060	*L. braziliensis*	52	402	453
LbrM31_V2.1060	*L. braziliensis*	54	559	612
LbrM31_V2.1060	*L. braziliensis*	75	734	808
LbrM31_V2.1060	*L. braziliensis*	405	861	1265
LbrM31_V2.1060	*L. braziliensis*	122	1372	1493
LbrM31_V2.1060	*L. braziliensis*	152	1538	1689
LbrM31_V2.1070	*L. braziliensis*	203	29	231
LbrM31_V2.1070	*L. braziliensis*	166	234	399
LbrM31_V2.1070	*L. braziliensis*	358	409	766
LbrM31_V2.1080	*L. braziliensis*	496	1	496
LbrM31_V2.1080	*L. braziliensis*	73	508	580

LbrM31_V2.1080	*L. braziliensis*	225	582	806
LbrM31_V2.1080	*L. braziliensis*	404	810	1213
LbrM31_V2.1080	*L. braziliensis*	179	1240	1418
LbrM31_V2.1080	*L. braziliensis*	73	1435	1507
LbrM31_V2.1080	*L. braziliensis*	132	1622	1753
LbrM31_V2.1080	*L. braziliensis*	103	1809	1911
LbrM31_V2.1080	*L. braziliensis*	134	1955	2088
LbrM31_V2.1080	*L. braziliensis*	90	2090	2179
LbrM31_V2.1080	*L. braziliensis*	73	2420	2492
LbrM31_V2.1090	*L. braziliensis*	158	41	198
LbrM31_V2.1100	*L. braziliensis*	105	49	153
LbrM31_V2.1100	*L. braziliensis*	77	479	555
LbrM31_V2.1110	*L. braziliensis*	98	56	153
LbrM31_V2.1110	*L. braziliensis*	64	491	554
LbrM31_V2.1130	*L. braziliensis*	105	49	153
LbrM31_V2.1150	*L. braziliensis*	52	148	199
LbrM31_V2.1160	*L. braziliensis*	60	125	184
LbrM31_V2.1170	*L. braziliensis*	65	108	172
LbrM31_V2.1180	*L. braziliensis*	180	476	655
LbrM31_V2.1180	*L. braziliensis*	42	681	722
LbrM31_V2.1190	*L. braziliensis*	104	133	236
LbrM31_V2.1190	*L. braziliensis*	161	250	410
LbrM31_V2.1200	*L. braziliensis*	505	523	1027
LbrM31_V2.1210	*L. braziliensis*	42	89	130
LbrM31_V2.1210	*L. braziliensis*	45	203	247
LbrM31_V2.1210	*L. braziliensis*	65	526	590
LbrM31_V2.1220	*L. braziliensis*	73	11	83
LbrM31_V2.1220	*L. braziliensis*	317	110	426
LbrM31_V2.1220	*L. braziliensis*	42	435	476
LbrM31_V2.1220	*L. braziliensis*	56	694	749
LbrM31_V2.1220	*L. braziliensis*	74	785	858
LbrM31_V2.1220	*L. braziliensis*	48	868	915
LbrM31_V2.1230	*L. braziliensis*	151	43	193
LbrM31_V2.1230	*L. braziliensis*	160	321	480
LbrM31_V2.1230	*L. braziliensis*	41	502	542
LbrM31_V2.1230	*L. braziliensis*	43	730	772
LbrM31_V2.1240	*L. braziliensis*	69	42	110
LbrM31_V2.1240	*L. braziliensis*	45	271	315
LbrM31_V2.1240	*L. braziliensis*	45	271	315
LbrM31_V2.1250	*L. braziliensis*	289	38	326
LbrM31_V2.1260	*L. braziliensis*	179	54	232
LbrM31_V2.1270	*L. braziliensis*	179	54	232
LbrM31_V2.1290	*L. braziliensis*	921	43	963
LbrM31_V2.1300	*L. braziliensis*	40	93	132
LbrM31_V2.1350	*L. braziliensis*	144	26	169
LbrM31_V2.1350	*L. braziliensis*	208	552	759
LbrM31_V2.1350	*L. braziliensis*	194	829	1022

LbrM31_V2.1350	*L. braziliensis*	48	1029	1076
LbrM31_V2.1360	*L. braziliensis*	45	1	45
LbrM31_V2.1360	*L. braziliensis*	45	137	181
LbrM31_V2.1360	*L. braziliensis*	75	564	638
LbrM31_V2.1390	*L. braziliensis*	104	44	147
LbrM31_V2.1440	*L. braziliensis*	43	60	102
LbrM31_V2.1440	*L. braziliensis*	60	351	410
LbrM31_V2.1440	*L. braziliensis*	44	469	512
LbrM31_V2.1440	*L. braziliensis*	47	1104	1150
LbrM31_V2.1440	*L. braziliensis*	52	1553	1604
LbrM31_V2.1440	*L. braziliensis*	63	1689	1751
LbrM31_V2.1440	*L. braziliensis*	94	2132	2225
LbrM31_V2.1440	*L. braziliensis*	90	2917	3006
LbrM31_V2.1440	*L. braziliensis*	46	3052	3097
LbrM31_V2.1440	*L. braziliensis*	62	3817	3878
LbrM31_V2.1440	*L. braziliensis*	97	4295	4391
LbrM31_V2.1440	*L. braziliensis*	59	4653	4711
LbrM31_V2.1440	*L. braziliensis*	125	4749	4873
LbrM31_V2.1460	*L. braziliensis*	46	406	451
LbrM31_V2.1470	*L. braziliensis*	797	1	797
LbrM31_V2.1470	*L. braziliensis*	257	820	1076
LbrM31_V2.1470	*L. braziliensis*	71	1166	1236
LbrM31_V2.1470	*L. braziliensis*	81	1282	1362
LbrM31_V2.1480	*L. braziliensis*	85	123	207
LbrM31_V2.1480	*L. braziliensis*	74	316	389
LbrM31_V2.1480	*L. braziliensis*	203	452	654
LbrM31_V2.1480	*L. braziliensis*	66	684	749
LbrM31_V2.1480	*L. braziliensis*	313	757	1069
LbrM31_V2.1490	*L. braziliensis*	82	87	168
LbrM31_V2.1490	*L. braziliensis*	96	283	378
LbrM31_V2.1490	*L. braziliensis*	141	431	571
LbrM31_V2.1500	*L. braziliensis*	46	1	46
LbrM31_V2.1500	*L. braziliensis*	47	175	221
LbrM31_V2.1500	*L. braziliensis*	86	672	757
LbrM31_V2.1500	*L. braziliensis*	68	973	1040
LbrM31_V2.1500	*L. braziliensis*	42	1371	1412
LbrM31_V2.1510	*L. braziliensis*	100	124	223
LbrM31_V2.1510	*L. braziliensis*	40	1499	1538
LbrM31_V2.1510	*L. braziliensis*	40	1499	1538
LbrM31_V2.1520	*L. braziliensis*	49	123	171
LbrM31_V2.1530	*L. braziliensis*	204	1	204
LbrM31_V2.1530	*L. braziliensis*	662	243	904
LbrM31_V2.1540	*L. braziliensis*	63	1	63
LbrM31_V2.1540	*L. braziliensis*	106	69	174
LbrM31_V2.1540	*L. braziliensis*	193	248	440
LbrM31_V2.1550	*L. braziliensis*	54	73	126
LbrM31_V2.1560	*L. braziliensis*	60	98	157

LbrM31_V2.1560	*L. braziliensis*	95	203	297
LbrM31_V2.1560	*L. braziliensis*	41	408	448
LbrM31_V2.1560	*L. braziliensis*	60	543	602
LbrM31_V2.1560	*L. braziliensis*	101	636	736
LbrM31_V2.1560	*L. braziliensis*	75	845	919
LbrM31_V2.1560	*L. braziliensis*	56	948	1003
LbrM31_V2.1560	*L. braziliensis*	106	1099	1204
LbrM31_V2.1560	*L. braziliensis*	62	1208	1269
LbrM31_V2.1570	*L. braziliensis*	145	79	223
LbrM31_V2.1570	*L. braziliensis*	82	238	319
LbrM31_V2.1570	*L. braziliensis*	44	356	399
LbrM31_V2.1570	*L. braziliensis*	62	451	512
LbrM31_V2.1580	*L. braziliensis*	49	1	49
LbrM31_V2.1580	*L. braziliensis*	179	77	255
LbrM31_V2.1580	*L. braziliensis*	179	77	255
LbrM31_V2.1590	*L. braziliensis*	95	1	95
LbrM31_V2.1590	*L. braziliensis*	169	141	309
LbrM31_V2.1600	*L. braziliensis*	713	1	713
LbrM31_V2.1600	*L. braziliensis*	41	728	768
LbrM31_V2.1600	*L. braziliensis*	218	816	1033
LbrM31_V2.1600	*L. braziliensis*	50	1153	1202
LbrM31_V2.1610	*L. braziliensis*	370	1	370
LbrM31_V2.1610	*L. braziliensis*	181	374	554
LbrM31_V2.1610	*L. braziliensis*	84	567	650
LbrM31_V2.1610	*L. braziliensis*	48	653	700
LbrM31_V2.1610	*L. braziliensis*	52	736	787
LbrM31_V2.1610	*L. braziliensis*	186	805	990
LbrM31_V2.1610	*L. braziliensis*	116	994	1109
LbrM31_V2.1620	*L. braziliensis*	192	1	192
LbrM31_V2.1620	*L. braziliensis*	257	225	481
LbrM31_V2.1620	*L. braziliensis*	73	521	593
LbrM31_V2.1620	*L. braziliensis*	44	624	667
LbrM31_V2.1630	*L. braziliensis*	48	84	131
LbrM31_V2.1630	*L. braziliensis*	43	220	262
LbrM31_V2.1630	*L. braziliensis*	65	359	423
LbrM31_V2.1630	*L. braziliensis*	46	554	599
LbrM31_V2.1630	*L. braziliensis*	117	651	767
LbrM31_V2.1630	*L. braziliensis*	78	827	904
LbrM31_V2.1630	*L. braziliensis*	68	957	1024
LbrM31_V2.1630	*L. braziliensis*	59	1130	1188
LbrM31_V2.1630	*L. braziliensis*	291	1306	1596
LbrM31_V2.1630	*L. braziliensis*	357	1602	1958
LbrM31_V2.1630	*L. braziliensis*	236	2004	2239
LbrM31_V2.1630	*L. braziliensis*	95	2278	2372
LbrM31_V2.1640	*L. braziliensis*	43	44	86
LbrM31_V2.1640	*L. braziliensis*	63	165	227
LbrM31_V2.1640	*L. braziliensis*	85	626	710

LbrM31_V2.1640	*L. braziliensis*	102	741	842
LbrM31_V2.1640	*L. braziliensis*	79	849	927
LbrM31_V2.1640	*L. braziliensis*	80	938	1017
LbrM31_V2.1640	*L. braziliensis*	62	1053	1114
LbrM31_V2.1650	*L. braziliensis*	46	666	711
LbrM31_V2.1650	*L. braziliensis*	59	791	849
LbrM31_V2.1650	*L. braziliensis*	154	969	1122
LbrM31_V2.1650	*L. braziliensis*	49	1464	1512
LbrM31_V2.1650	*L. braziliensis*	50	1774	1823
LbrM31_V2.1660	*L. braziliensis*	367	141	507
LbrM31_V2.1670	*L. braziliensis*	350	275	624
LbrM31_V2.1690	*L. braziliensis*	45	109	153
LbrM31_V2.1690	*L. braziliensis*	53	230	282
LbrM31_V2.1690	*L. braziliensis*	62	408	469
LbrM31_V2.1690	*L. braziliensis*	49	504	552
LbrM31_V2.1690	*L. braziliensis*	149	579	727
LbrM31_V2.1690	*L. braziliensis*	100	773	872
LbrM31_V2.1690	*L. braziliensis*	65	926	990
LbrM31_V2.1700	*L. braziliensis*	78	367	444
LbrM31_V2.1700	*L. braziliensis*	71	511	581
LbrM31_V2.1700	*L. braziliensis*	120	747	866
LbrM31_V2.1700	*L. braziliensis*	41	1145	1185
LbrM31_V2.1700	*L. braziliensis*	66	1395	1460
LbrM31_V2.1700	*L. braziliensis*	43	1540	1582
LbrM31_V2.1710	*L. braziliensis*	88	55	142
LbrM31_V2.1710	*L. braziliensis*	88	302	389
LbrM31_V2.1710	*L. braziliensis*	48	415	462
LbrM31_V2.1710	*L. braziliensis*	41	1274	1314
LbrM31_V2.1720	*L. braziliensis*	80	83	162
LbrM31_V2.1720	*L. braziliensis*	47	223	269
LbrM31_V2.1720	*L. braziliensis*	69	303	371
LbrM31_V2.1720	*L. braziliensis*	76	434	509
LbrM31_V2.1720	*L. braziliensis*	53	549	601
LbrM31_V2.1720	*L. braziliensis*	152	810	961
LbrM31_V2.1720	*L. braziliensis*	150	989	1138
LbrM31_V2.1730	*L. braziliensis*	46	500	545
LbrM31_V2.1730	*L. braziliensis*	245	629	873
LbrM31_V2.1740	*L. braziliensis*	422	1	422
LbrM31_V2.1740	*L. braziliensis*	89	472	560
LbrM31_V2.1740	*L. braziliensis*	261	595	855
LbrM31_V2.1740	*L. braziliensis*	285	942	1226
LbrM31_V2.1740	*L. braziliensis*	143	1332	1474
LbrM31_V2.1740	*L. braziliensis*	54	1587	1640
LbrM31_V2.1740	*L. braziliensis*	196	1835	2030
LbrM31_V2.1740	*L. braziliensis*	150	2122	2271
LbrM31_V2.1750	*L. braziliensis*	109	33	141
LbrM31_V2.1750	*L. braziliensis*	160	161	320

LbrM31_V2.1750	*L. braziliensis*	61	329	389
LbrM31_V2.1760	*L. braziliensis*	62	201	262
LbrM31_V2.1800	*L. braziliensis*	42	129	170
LbrM31_V2.1840	*L. braziliensis*	67	227	293
LbrM31_V2.1860	*L. braziliensis*	177	200	376
LbrM31_V2.1860	*L. braziliensis*	110	556	665
LbrM31_V2.1860	*L. braziliensis*	58	1457	1514
LbrM31_V2.1870	*L. braziliensis*	55	192	246
LbrM31_V2.1880	*L. braziliensis*	55	421	475
LbrM31_V2.1910	*L. braziliensis*	156	63	218
LbrM31_V2.1920	*L. braziliensis*	52	1	52
LbrM31_V2.1920	*L. braziliensis*	44	327	370
LbrM31_V2.1920	*L. braziliensis*	77	541	617
LbrM31_V2.1920	*L. braziliensis*	53	817	869
LbrM31_V2.1920	*L. braziliensis*	47	1109	1155
LbrM31_V2.1930	*L. braziliensis*	185	29	213
LbrM31_V2.1930	*L. braziliensis*	55	532	586
LbrM31_V2.1940	*L. braziliensis*	200	1	200
LbrM31_V2.1940	*L. braziliensis*	87	239	325
LbrM31_V2.1940	*L. braziliensis*	65	501	565
LbrM31_V2.1950	*L. braziliensis*	53	21	73
LbrM31_V2.1950	*L. braziliensis*	41	471	511
LbrM31_V2.1950	*L. braziliensis*	75	726	800
LbrM31_V2.1950	*L. braziliensis*	41	921	961
LbrM31_V2.1960	*L. braziliensis*	266	36	301
LbrM31_V2.1960	*L. braziliensis*	57	328	384
LbrM31_V2.1970	*L. braziliensis*	53	60	112
LbrM31_V2.1970	*L. braziliensis*	68	218	285
LbrM31_V2.1970	*L. braziliensis*	90	321	410
LbrM31_V2.1980	*L. braziliensis*	52	86	137
LbrM31_V2.1980	*L. braziliensis*	74	232	305
LbrM31_V2.1980	*L. braziliensis*	83	325	407
LbrM31_V2.1990	*L. braziliensis*	73	1	73
LbrM31_V2.2000	*L. braziliensis*	44	1	44
LbrM31_V2.2010	*L. braziliensis*	75	385	459
LbrM31_V2.2020	*L. braziliensis*	44	1	44
LbrM31_V2.2020	*L. braziliensis*	40	447	486
LbrM31_V2.2030	*L. braziliensis*	106	500	605
LbrM31_V2.2030	*L. braziliensis*	61	644	704
LbrM31_V2.2030	*L. braziliensis*	56	933	988
LbrM31_V2.2040	*L. braziliensis*	73	1	73
LbrM31_V2.2040	*L. braziliensis*	40	476	515
LbrM31_V2.2040	*L. braziliensis*	40	476	515
LbrM31_V2.2050	*L. braziliensis*	56	52	107
LbrM31_V2.2050	*L. braziliensis*	42	434	475
LbrM31_V2.2060	*L. braziliensis*	73	1	73
LbrM31_V2.2060	*L. braziliensis*	40	476	515

LbrM31_V2.2070	*L. braziliensis*	104	512	615
LbrM31_V2.2070	*L. braziliensis*	61	652	712
LbrM31_V2.2080	*L. braziliensis*	106	502	607
LbrM31_V2.2080	*L. braziliensis*	61	646	706
LbrM31_V2.2080	*L. braziliensis*	97	874	970
LbrM31_V2.2140	*L. braziliensis*	701	34	734
LbrM31_V2.2140	*L. braziliensis*	45	741	785
LbrM31_V2.2150	*L. braziliensis*	110	1	110
LbrM31_V2.2150	*L. braziliensis*	40	224	263
LbrM31_V2.2150	*L. braziliensis*	319	424	742
LbrM31_V2.2150	*L. braziliensis*	100	992	1091
LbrM31_V2.2160	*L. braziliensis*	41	65	105
LbrM31_V2.2160	*L. braziliensis*	41	163	203
LbrM31_V2.2160	*L. braziliensis*	69	584	652
LbrM31_V2.2170	*L. braziliensis*	281	42	322
LbrM31_V2.2170	*L. braziliensis*	112	348	459
LbrM31_V2.2180	*L. braziliensis*	322	48	369
LbrM31_V2.2180	*L. braziliensis*	186	385	570
LbrM31_V2.2240	*L. braziliensis*	60	84	143
LbrM31_V2.2260	*L. braziliensis*	60	435	494
LbrM31_V2.2270	*L. braziliensis*	97	1	97
LbrM31_V2.2270	*L. braziliensis*	108	104	211
LbrM31_V2.2270	*L. braziliensis*	338	234	571
LbrM31_V2.2300	*L. braziliensis*	74	11	84
LbrM31_V2.2300	*L. braziliensis*	62	200	261
LbrM31_V2.2300	*L. braziliensis*	70	285	354
LbrM31_V2.2300	*L. braziliensis*	62	394	455
LbrM31_V2.2330	*L. braziliensis*	46	1	46
LbrM31_V2.2340	*L. braziliensis*	138	1	138
LbrM31_V2.2340	*L. braziliensis*	66	237	302
LbrM31_V2.2340	*L. braziliensis*	84	319	402
LbrM31_V2.2350	*L. braziliensis*	266	1	266
LbrM31_V2.2350	*L. braziliensis*	470	275	744
LbrM31_V2.2350	*L. braziliensis*	58	774	831
LbrM31_V2.2350	*L. braziliensis*	88	870	957
LbrM31_V2.2350	*L. braziliensis*	61	987	1047
LbrM31_V2.2360	*L. braziliensis*	389	1	389
LbrM31_V2.2370	*L. braziliensis*	141	1	141
LbrM31_V2.2370	*L. braziliensis*	153	176	328
LbrM31_V2.2380	*L. braziliensis*	104	1	104
LbrM31_V2.2380	*L. braziliensis*	75	127	201
LbrM31_V2.2380	*L. braziliensis*	63	234	296
LbrM31_V2.2380	*L. braziliensis*	113	324	436
LbrM31_V2.2380	*L. braziliensis*	132	447	578
LbrM31_V2.2380	*L. braziliensis*	118	611	728
LbrM31_V2.2390	*L. braziliensis*	63	1	63
LbrM31_V2.2400	*L. braziliensis*	666	1	666

LbrM31_V2.2400	*L. braziliensis*	99	681	779
LbrM31_V2.2400	*L. braziliensis*	82	811	892
LbrM31_V2.2400	*L. braziliensis*	92	910	1001
LbrM31_V2.2420	*L. braziliensis*	311	1	311
LbrM31_V2.2420	*L. braziliensis*	45	352	396
LbrM31_V2.2420	*L. braziliensis*	367	425	791
LbrM31_V2.2420	*L. braziliensis*	53	837	889
LbrM31_V2.2430	*L. braziliensis*	45	961	1005
LbrM31_V2.2440	*L. braziliensis*	592	1	592
LbrM31_V2.2440	*L. braziliensis*	56	594	649
LbrM31_V2.2440	*L. braziliensis*	68	705	772
LbrM31_V2.2450	*L. braziliensis*	530	1	530
LbrM31_V2.2450	*L. braziliensis*	174	543	716
LbrM31_V2.2460	*L. braziliensis*	50	1	50
LbrM31_V2.2460	*L. braziliensis*	57	71	127
LbrM31_V2.2460	*L. braziliensis*	86	154	239
LbrM31_V2.2460	*L. braziliensis*	51	247	297
LbrM31_V2.2460	*L. braziliensis*	103	323	425
LbrM31_V2.2470	*L. braziliensis*	85	1	85
LbrM31_V2.2470	*L. braziliensis*	64	280	343
LbrM31_V2.2480	*L. braziliensis*	131	48	178
LbrM31_V2.2480	*L. braziliensis*	65	214	278
LbrM31_V2.2480	*L. braziliensis*	153	296	448
LbrM31_V2.2490	*L. braziliensis*	41	118	158
LbrM31_V2.2500	*L. braziliensis*	228	1	228
LbrM31_V2.2500	*L. braziliensis*	92	328	419
LbrM31_V2.2500	*L. braziliensis*	88	451	538
LbrM31_V2.2500	*L. braziliensis*	90	585	674
LbrM31_V2.2500	*L. braziliensis*	326	709	1034
LbrM31_V2.2500	*L. braziliensis*	665	1053	1717
LbrM31_V2.2510	*L. braziliensis*	131	48	178
LbrM31_V2.2510	*L. braziliensis*	64	215	278
LbrM31_V2.2510	*L. braziliensis*	153	296	448
LbrM31_V2.2520	*L. braziliensis*	84	1	84
LbrM31_V2.2520	*L. braziliensis*	80	264	343
LbrM31_V2.2530	*L. braziliensis*	54	131	184
LbrM31_V2.2530	*L. braziliensis*	45	502	546
LbrM31_V2.2530	*L. braziliensis*	45	502	546
LbrM31_V2.2540	*L. braziliensis*	108	17	124
LbrM31_V2.2540	*L. braziliensis*	41	156	196
LbrM31_V2.2590	*L. braziliensis*	54	71	124
LbrM31_V2.2590	*L. braziliensis*	73	154	226
LbrM31_V2.2590	*L. braziliensis*	40	323	362
LbrM31_V2.2600	*L. braziliensis*	84	1	84
LbrM31_V2.2600	*L. braziliensis*	64	280	343
LbrM31_V2.2610	*L. braziliensis*	45	1	45
LbrM31_V2.2640	*L. braziliensis*	52	1298	1349

LbrM31_V2.2650	*L. braziliensis*	148	1	148
LbrM31_V2.2650	*L. braziliensis*	195	152	346
LbrM31_V2.2650	*L. braziliensis*	206	377	582
LbrM31_V2.2660	*L. braziliensis*	63	1	63
LbrM31_V2.2670	*L. braziliensis*	103	1	103
LbrM31_V2.2670	*L. braziliensis*	173	140	312
LbrM31_V2.2670	*L. braziliensis*	337	356	692
LbrM31_V2.2670	*L. braziliensis*	133	698	830
LbrM31_V2.2680	*L. braziliensis*	61	205	265
LbrM31_V2.2690	*L. braziliensis*	98	58	155
LbrM31_V2.2690	*L. braziliensis*	118	184	301
LbrM31_V2.2690	*L. braziliensis*	88	396	483
LbrM31_V2.2690	*L. braziliensis*	114	522	635
LbrM31_V2.2690	*L. braziliensis*	116	696	811
LbrM31_V2.2690	*L. braziliensis*	43	895	937
LbrM31_V2.2690	*L. braziliensis*	42	1056	1097
LbrM31_V2.2690	*L. braziliensis*	68	1157	1224
LbrM31_V2.2700	*L. braziliensis*	137	1	137
LbrM31_V2.2710	*L. braziliensis*	46	119	164
LbrM31_V2.2730	*L. braziliensis*	51	104	154
LbrM31_V2.2770	*L. braziliensis*	66	1	66
LbrM31_V2.2790	*L. braziliensis*	46	15	60
LbrM31_V2.2820	*L. braziliensis*	66	1	66
LbrM31_V2.2840	*L. braziliensis*	40	1	40
LbrM31_V2.2850	*L. braziliensis*	76	10	85
LbrM31_V2.2870	*L. braziliensis*	81	215	295
LbrM31_V2.2870	*L. braziliensis*	55	413	467
LbrM31_V2.2870	*L. braziliensis*	55	413	467
LbrM31_V2.2880	*L. braziliensis*	81	552	632
LbrM31_V2.2890	*L. braziliensis*	52	51	102
LbrM31_V2.2890	*L. braziliensis*	163	541	703
LbrM31_V2.2900	*L. braziliensis*	68	46	113
LbrM31_V2.2900	*L. braziliensis*	256	513	768
LbrM31_V2.2900	*L. braziliensis*	74	816	889
LbrM31_V2.2910	*L. braziliensis*	183	116	298
LbrM31_V2.2910	*L. braziliensis*	54	554	607
LbrM31_V2.2910	*L. braziliensis*	172	642	813
LbrM31_V2.2910	*L. braziliensis*	212	837	1048
LbrM31_V2.2910	*L. braziliensis*	40	1067	1106
LbrM31_V2.2930	*L. braziliensis*	41	88	128
LbrM31_V2.2930	*L. braziliensis*	62	621	682
LbrM31_V2.2930	*L. braziliensis*	62	621	682
LbrM31_V2.2940	*L. braziliensis*	104	206	309
LbrM31_V2.2940	*L. braziliensis*	69	354	422
LbrM31_V2.2950	*L. braziliensis*	42	675	716
LbrM31_V2.2950	*L. braziliensis*	48	1463	1510
LbrM31_V2.2950	*L. braziliensis*	53	1541	1593

LbrM31_V2.2950	*L. braziliensis*	61	1602	1662
LbrM31_V2.2960	*L. braziliensis*	44	1	44
LbrM31_V2.2970	*L. braziliensis*	68	20	87
LbrM31_V2.2990	*L. braziliensis*	41	1	41
LbrM31_V2.2990	*L. braziliensis*	91	92	182
LbrM31_V2.2990	*L. braziliensis*	91	92	182
LbrM31_V2.3000	*L. braziliensis*	342	40	381
LbrM31_V2.3000	*L. braziliensis*	179	412	590
LbrM31_V2.3020	*L. braziliensis*	147	76	222
LbrM31_V2.3020	*L. braziliensis*	41	257	297
LbrM31_V2.3020	*L. braziliensis*	86	477	562
LbrM31_V2.3020	*L. braziliensis*	71	602	672
LbrM31_V2.3030	*L. braziliensis*	86	154	239
LbrM31_V2.3030	*L. braziliensis*	84	308	391
LbrM31_V2.3030	*L. braziliensis*	40	509	548
LbrM31_V2.3030	*L. braziliensis*	76	655	730
LbrM31_V2.3030	*L. braziliensis*	52	1032	1083
LbrM31_V2.3030	*L. braziliensis*	42	1217	1258
LbrM31_V2.3050	*L. braziliensis*	49	1	49
LbrM31_V2.3050	*L. braziliensis*	83	262	344
LbrM31_V2.3050	*L. braziliensis*	369	352	720
LbrM31_V2.3070	*L. braziliensis*	42	100	141
LbrM31_V2.3100	*L. braziliensis*	204	1	204
LbrM31_V2.3100	*L. braziliensis*	111	238	348
LbrM31_V2.3100	*L. braziliensis*	40	355	394
LbrM31_V2.3110	*L. braziliensis*	1165	1	1165
LbrM31_V2.3110	*L. braziliensis*	41	1177	1217
LbrM31_V2.3120	*L. braziliensis*	97	117	213
LbrM31_V2.3120	*L. braziliensis*	84	229	312
LbrM31_V2.3130	*L. braziliensis*	235	1	235
LbrM31_V2.3160	*L. braziliensis*	172	1	172
LbrM31_V2.3170	*L. braziliensis*	87	44	130
LbrM31_V2.3180	*L. braziliensis*	51	220	270
LbrM31_V2.3190	*L. braziliensis*	250	10	259
LbrM31_V2.3190	*L. braziliensis*	252	372	623
LbrM31_V2.3200	*L. braziliensis*	74	30	103
LbrM31_V2.3210	*L. braziliensis*	90	70	159
LbrM31_V2.3220	*L. braziliensis*	125	1	125
LbrM31_V2.3220	*L. braziliensis*	68	145	212
LbrM31_V2.3220	*L. braziliensis*	83	252	334
LbrM31_V2.3250	*L. braziliensis*	52	182	233
LbrM31_V2.3250	*L. braziliensis*	42	375	416
LbrM31_V2.3250	*L. braziliensis*	42	860	901
LbrM31_V2.3260	*L. braziliensis*	176	1	176
LbrM31_V2.3260	*L. braziliensis*	68	244	311
LbrM31_V2.3260	*L. braziliensis*	69	330	398
LbrM31_V2.3280	*L. braziliensis*	97	79	175

LbrM31_V2.3280	*L. braziliensis*	42	205	246
LbrM31_V2.3340	*L. braziliensis*	79	1331	1409
LbrM31_V2.3350	*L. braziliensis*	42	35	76
LbrM31_V2.3350	*L. braziliensis*	49	765	813
LbrM31_V2.3350	*L. braziliensis*	171	818	988
LbrM31_V2.3360	*L. braziliensis*	43	135	177
LbrM31_V2.3360	*L. braziliensis*	137	228	364
LbrM31_V2.3360	*L. braziliensis*	105	527	631
LbrM31_V2.3360	*L. braziliensis*	82	742	823
LbrM31_V2.3360	*L. braziliensis*	83	913	995
LbrM31_V2.3360	*L. braziliensis*	106	1030	1135
LbrM31_V2.3360	*L. braziliensis*	79	1211	1289
LbrM31_V2.3360	*L. braziliensis*	84	1361	1444
LbrM31_V2.3360	*L. braziliensis*	199	1507	1705
LbrM31_V2.3360	*L. braziliensis*	153	1780	1932
LbrM31_V2.3370	*L. braziliensis*	68	44	111
LbrM31_V2.3380	*L. braziliensis*	125	119	243
LbrM31_V2.3380	*L. braziliensis*	109	348	456
LbrM31_V2.3380	*L. braziliensis*	109	348	456
LbrM31_V2.3390	*L. braziliensis*	198	1	198
LbrM31_V2.3390	*L. braziliensis*	170	258	427
LbrM31_V2.3410	*L. braziliensis*	99	30	128
LbrM31_V2.3410	*L. braziliensis*	40	132	171
LbrM31_V2.3410	*L. braziliensis*	182	196	377
LbrM31_V2.3410	*L. braziliensis*	308	410	717
LbrM31_V2.3430	*L. braziliensis*	76	1	76
LbrM31_V2.3430	*L. braziliensis*	96	153	248
LbrM31_V2.3430	*L. braziliensis*	96	153	248
LbrM31_V2.3450	*L. braziliensis*	92	1	92
LbrM31_V2.3460	*L. braziliensis*	46	1	46
LbrM31_V2.3470	*L. braziliensis*	62	126	187
LbrM31_V2.3480	*L. braziliensis*	53	164	216
LbrM31_V2.3490	*L. braziliensis*	55	164	218
LbrM31_V2.3500	*L. braziliensis*	53	139	191
LbrM31_V2.3510	*L. braziliensis*	46	1	46
LbrM31_V2.3520	*L. braziliensis*	62	441	502
LbrM31_V2.3530	*L. braziliensis*	50	47	96
LbrM31_V2.3530	*L. braziliensis*	73	145	217
LbrM31_V2.3530	*L. braziliensis*	73	145	217
LbrM31_V2.3550	*L. braziliensis*	555	1	555
LbrM31_V2.3550	*L. braziliensis*	266	563	828
LbrM31_V2.3560	*L. braziliensis*	47	536	582
LbrM31_V2.3600	*L. braziliensis*	72	759	830
LbrM31_V2.3600	*L. braziliensis*	84	970	1053
LbrM31_V2.3600	*L. braziliensis*	52	1330	1381
LbrM31_V2.3600	*L. braziliensis*	41	1429	1469
LbrM31_V2.3620	*L. braziliensis*	40	324	363

LbrM31_V2.3630	*L. braziliensis*	42	1	42
LbrM31_V2.3650	*L. braziliensis*	256	1	256
LbrM31_V2.3650	*L. braziliensis*	443	294	736
LbrM31_V2.3650	*L. braziliensis*	43	766	808
LbrM31_V2.3650	*L. braziliensis*	183	816	998
LbrM31_V2.3650	*L. braziliensis*	60	1106	1165
LbrM31_V2.3650	*L. braziliensis*	234	1195	1428
LbrM31_V2.3660	*L. braziliensis*	64	313	376
LbrM31_V2.3670	*L. braziliensis*	349	1	349
LbrM31_V2.3670	*L. braziliensis*	45	351	395
LbrM31_V2.3670	*L. braziliensis*	273	488	760
LbrM31_V2.3680	*L. braziliensis*	295	1	295
LbrM31_V2.3680	*L. braziliensis*	691	305	995
LbrM31_V2.3680	*L. braziliensis*	172	1006	1177
LbrM31_V2.3680	*L. braziliensis*	139	1185	1323
LbrM31_V2.3680	*L. braziliensis*	193	1363	1555
LbrM31_V2.3700	*L. braziliensis*	101	282	382
LbrM31_V2.3710	*L. braziliensis*	83	49	131
LbrM31_V2.3710	*L. braziliensis*	76	340	415
LbrM31_V2.3720	*L. braziliensis*	281	42	322
LbrM31_V2.3720	*L. braziliensis*	63	348	410
LbrM31_V2.3720	*L. braziliensis*	45	415	459
LbrM31_V2.3730	*L. braziliensis*	72	1	72
LbrM31_V2.3730	*L. braziliensis*	179	100	278
LbrM31_V2.3730	*L. braziliensis*	62	486	547
LbrM31_V2.3750	*L. braziliensis*	455	1	455
LbrM32_V2.0010	*L. braziliensis*	161	200	360
LbrM32_V2.0010	*L. braziliensis*	120	525	644
LbrM32_V2.0010	*L. braziliensis*	58	1435	1492
LbrM32_V2.0030	*L. braziliensis*	115	1	115
LbrM32_V2.0030	*L. braziliensis*	54	139	192
LbrM32_V2.0030	*L. braziliensis*	148	202	349
LbrM32_V2.0030	*L. braziliensis*	54	354	407
LbrM32_V2.0030	*L. braziliensis*	42	443	484
LbrM32_V2.0040	*L. braziliensis*	50	1	50
LbrM32_V2.0040	*L. braziliensis*	58	585	642
LbrM32_V2.0040	*L. braziliensis*	58	585	642
LbrM32_V2.0060	*L. braziliensis*	115	1	115
LbrM32_V2.0060	*L. braziliensis*	54	139	192
LbrM32_V2.0060	*L. braziliensis*	148	202	349
LbrM32_V2.0060	*L. braziliensis*	55	354	408
LbrM32_V2.0060	*L. braziliensis*	54	431	484
LbrM32_V2.0070	*L. braziliensis*	93	246	338
LbrM32_V2.0080	*L. braziliensis*	56	37	92
LbrM32_V2.0080	*L. braziliensis*	54	231	284
LbrM32_V2.0080	*L. braziliensis*	80	303	382
LbrM32_V2.0080	*L. braziliensis*	50	388	437

LbrM32_V2.0090	*L. braziliensis*	51	179	229
LbrM32_V2.0090	*L. braziliensis*	91	252	342
LbrM32_V2.0110	*L. braziliensis*	75	1	75
LbrM32_V2.0110	*L. braziliensis*	96	258	353
LbrM32_V2.0110	*L. braziliensis*	96	258	353
LbrM32_V2.0120	*L. braziliensis*	156	1	156
LbrM32_V2.0130	*L. braziliensis*	49	1	49
LbrM32_V2.0140	*L. braziliensis*	93	122	214
LbrM32_V2.0180	*L. braziliensis*	157	1	157
LbrM32_V2.0180	*L. braziliensis*	87	537	623
LbrM32_V2.0180	*L. braziliensis*	47	633	679
LbrM32_V2.0180	*L. braziliensis*	137	774	910
LbrM32_V2.0180	*L. braziliensis*	116	962	1077
LbrM32_V2.0200	*L. braziliensis*	67	36	102
LbrM32_V2.0200	*L. braziliensis*	90	114	203
LbrM32_V2.0210	*L. braziliensis*	224	1	224
LbrM32_V2.0210	*L. braziliensis*	47	226	272
LbrM32_V2.0210	*L. braziliensis*	47	321	367
LbrM32_V2.0210	*L. braziliensis*	157	406	562
LbrM32_V2.0210	*L. braziliensis*	248	570	817
LbrM32_V2.0210	*L. braziliensis*	51	830	880
LbrM32_V2.0210	*L. braziliensis*	71	923	993
LbrM32_V2.0220	*L. braziliensis*	95	14	108
LbrM32_V2.0220	*L. braziliensis*	83	176	258
LbrM32_V2.0220	*L. braziliensis*	54	325	378
LbrM32_V2.0240	*L. braziliensis*	135	174	308
LbrM32_V2.0260	*L. braziliensis*	76	528	603
LbrM32_V2.0270	*L. braziliensis*	93	91	183
LbrM32_V2.0270	*L. braziliensis*	78	192	269
LbrM32_V2.0270	*L. braziliensis*	68	272	339
LbrM32_V2.0280	*L. braziliensis*	111	423	533
LbrM32_V2.0290	*L. braziliensis*	105	97	201
LbrM32_V2.0290	*L. braziliensis*	164	271	434
LbrM32_V2.0290	*L. braziliensis*	44	520	563
LbrM32_V2.0290	*L. braziliensis*	115	734	848
LbrM32_V2.0310	*L. braziliensis*	51	417	467
LbrM32_V2.0310	*L. braziliensis*	71	513	583
LbrM32_V2.0310	*L. braziliensis*	387	605	991
LbrM32_V2.0310	*L. braziliensis*	61	1022	1082
LbrM32_V2.0320	*L. braziliensis*	466	1	466
LbrM32_V2.0320	*L. braziliensis*	57	580	636
LbrM32_V2.0320	*L. braziliensis*	74	923	996
LbrM32_V2.0320	*L. braziliensis*	48	1584	1631
LbrM32_V2.0320	*L. braziliensis*	355	1660	2014
LbrM32_V2.0330	*L. braziliensis*	220	258	477
LbrM32_V2.0330	*L. braziliensis*	902	484	1385
LbrM32_V2.0340	*L. braziliensis*	50	128	177

LbrM32_V2.0340	*L. braziliensis*	60	748	807
LbrM32_V2.0340	*L. braziliensis*	40	910	949
LbrM32_V2.0340	*L. braziliensis*	121	1091	1211
LbrM32_V2.0360	*L. braziliensis*	104	115	218
LbrM32_V2.0370	*L. braziliensis*	50	1	50
LbrM32_V2.0370	*L. braziliensis*	85	106	190
LbrM32_V2.0370	*L. braziliensis*	50	242	291
LbrM32_V2.0370	*L. braziliensis*	96	603	698
LbrM32_V2.0370	*L. braziliensis*	176	709	884
LbrM32_V2.0380	*L. braziliensis*	46	595	640
LbrM32_V2.0380	*L. braziliensis*	59	721	779
LbrM32_V2.0390	*L. braziliensis*	53	20	72
LbrM32_V2.0400	*L. braziliensis*	156	1	156
LbrM32_V2.0400	*L. braziliensis*	44	407	450
LbrM32_V2.0400	*L. braziliensis*	498	541	1038
LbrM32_V2.0400	*L. braziliensis*	46	1286	1331
LbrM32_V2.0400	*L. braziliensis*	70	1377	1446
LbrM32_V2.0400	*L. braziliensis*	92	1478	1569
LbrM32_V2.0410	*L. braziliensis*	189	29	217
LbrM32_V2.0410	*L. braziliensis*	69	240	308
LbrM32_V2.0410	*L. braziliensis*	263	631	893
LbrM32_V2.0420	*L. braziliensis*	231	1	231
LbrM32_V2.0420	*L. braziliensis*	49	312	360
LbrM32_V2.0420	*L. braziliensis*	102	364	465
LbrM32_V2.0430	*L. braziliensis*	287	1	287
LbrM32_V2.0430	*L. braziliensis*	58	300	357
LbrM32_V2.0440	*L. braziliensis*	52	451	502
LbrM32_V2.0440	*L. braziliensis*	60	832	891
LbrM32_V2.0440	*L. braziliensis*	60	832	891
LbrM32_V2.0450	*L. braziliensis*	89	1	89
LbrM32_V2.0450	*L. braziliensis*	194	105	298
LbrM32_V2.0450	*L. braziliensis*	131	333	463
LbrM32_V2.0470	*L. braziliensis*	102	1	102
LbrM32_V2.0470	*L. braziliensis*	98	507	604
LbrM32_V2.0480	*L. braziliensis*	55	1	55
LbrM32_V2.0480	*L. braziliensis*	43	273	315
LbrM32_V2.0480	*L. braziliensis*	46	659	704
LbrM32_V2.0490	*L. braziliensis*	69	375	443
LbrM32_V2.0520	*L. braziliensis*	52	1	52
LbrM32_V2.0540	*L. braziliensis*	196	126	321
LbrM32_V2.0540	*L. braziliensis*	125	334	458
LbrM32_V2.0560	*L. braziliensis*	49	33	81
LbrM32_V2.0590	*L. braziliensis*	48	1	48
LbrM32_V2.0610	*L. braziliensis*	52	19	70
LbrM32_V2.0610	*L. braziliensis*	44	116	159
LbrM32_V2.0610	*L. braziliensis*	86	298	383
LbrM32_V2.0640	*L. braziliensis*	47	80	126

LbrM32_V2.0650	*L. braziliensis*	111	1	111
LbrM32_V2.0650	*L. braziliensis*	74	511	584
LbrM32_V2.0660	*L. braziliensis*	157	108	264
LbrM32_V2.0660	*L. braziliensis*	177	315	491
LbrM32_V2.0670	*L. braziliensis*	76	98	173
LbrM32_V2.0670	*L. braziliensis*	42	226	267
LbrM32_V2.0680	*L. braziliensis*	81	375	455
LbrM32_V2.0680	*L. braziliensis*	40	520	559
LbrM32_V2.0680	*L. braziliensis*	40	520	559
LbrM32_V2.0690	*L. braziliensis*	55	160	214
LbrM32_V2.0700	*L. braziliensis*	154	1	154
LbrM32_V2.0700	*L. braziliensis*	40	245	284
LbrM32_V2.0720	*L. braziliensis*	61	1	61
LbrM32_V2.0720	*L. braziliensis*	105	500	604
LbrM32_V2.0730	*L. braziliensis*	50	28	77
LbrM32_V2.0750	*L. braziliensis*	69	99	167
LbrM32_V2.0750	*L. braziliensis*	46	198	243
LbrM32_V2.0750	*L. braziliensis*	46	198	243
LbrM32_V2.0760	*L. braziliensis*	154	334	487
LbrM32_V2.0760	*L. braziliensis*	164	515	678
LbrM32_V2.0760	*L. braziliensis*	317	802	1118
LbrM32_V2.0780	*L. braziliensis*	87	147	233
LbrM32_V2.0810	*L. braziliensis*	110	1	110
LbrM32_V2.0820	*L. braziliensis*	43	151	193
LbrM32_V2.0820	*L. braziliensis*	198	280	477
LbrM32_V2.0820	*L. braziliensis*	108	486	593
LbrM32_V2.0840	*L. braziliensis*	59	1	59
LbrM32_V2.0860	*L. braziliensis*	159	207	365
LbrM32_V2.0870	*L. braziliensis*	93	93	185
LbrM32_V2.0870	*L. braziliensis*	49	264	312
LbrM32_V2.0870	*L. braziliensis*	48	525	572
LbrM32_V2.0870	*L. braziliensis*	154	599	752
LbrM32_V2.0890	*L. braziliensis*	79	127	205
LbrM32_V2.0890	*L. braziliensis*	48	490	537
LbrM32_V2.0890	*L. braziliensis*	43	790	832
LbrM32_V2.0900	*L. braziliensis*	128	927	1054
LbrM32_V2.0920	*L. braziliensis*	118	194	311
LbrM32_V2.0920	*L. braziliensis*	60	328	387
LbrM32_V2.0920	*L. braziliensis*	47	420	466
LbrM32_V2.0930	*L. braziliensis*	50	1	50
LbrM32_V2.0930	*L. braziliensis*	246	52	297
LbrM32_V2.0940	*L. braziliensis*	140	57	196
LbrM32_V2.0940	*L. braziliensis*	182	271	452
LbrM32_V2.0940	*L. braziliensis*	109	643	751
LbrM32_V2.0950	*L. braziliensis*	51	476	526
LbrM32_V2.0960	*L. braziliensis*	40	284	323
LbrM32_V2.0990	*L. braziliensis*	56	52	107

LbrM32_V2.1010	*L. braziliensis*	63	700	762
LbrM32_V2.1020	*L. braziliensis*	42	559	600
LbrM32_V2.1030	*L. braziliensis*	139	250	388
LbrM32_V2.1030	*L. braziliensis*	143	667	809
LbrM32_V2.1030	*L. braziliensis*	143	667	809
LbrM32_V2.1040	*L. braziliensis*	48	305	352
LbrM32_V2.1050	*L. braziliensis*	88	148	235
LbrM32_V2.1060	*L. braziliensis*	54	43	96
LbrM32_V2.1060	*L. braziliensis*	88	455	542
LbrM32_V2.1060	*L. braziliensis*	74	715	788
LbrM32_V2.1060	*L. braziliensis*	60	985	1044
LbrM32_V2.1060	*L. braziliensis*	67	1115	1181
LbrM32_V2.1060	*L. braziliensis*	102	1374	1475
LbrM32_V2.1060	*L. braziliensis*	91	1494	1584
LbrM32_V2.1060	*L. braziliensis*	76	1607	1682
LbrM32_V2.1060	*L. braziliensis*	106	1711	1816
LbrM32_V2.1070	*L. braziliensis*	154	1	154
LbrM32_V2.1070	*L. braziliensis*	75	273	347
LbrM32_V2.1070	*L. braziliensis*	50	428	477
LbrM32_V2.1080	*L. braziliensis*	45	335	379
LbrM32_V2.1090	*L. braziliensis*	66	162	227
LbrM32_V2.1100	*L. braziliensis*	51	230	280
LbrM32_V2.1110	*L. braziliensis*	152	190	341
LbrM32_V2.1120	*L. braziliensis*	61	47	107
LbrM32_V2.1120	*L. braziliensis*	206	178	383
LbrM32_V2.1120	*L. braziliensis*	51	494	544
LbrM32_V2.1120	*L. braziliensis*	47	753	799
LbrM32_V2.1140	*L. braziliensis*	58	15	72
LbrM32_V2.1140	*L. braziliensis*	66	113	178
LbrM32_V2.1140	*L. braziliensis*	102	207	308
LbrM32_V2.1140	*L. braziliensis*	120	347	466
LbrM32_V2.1160	*L. braziliensis*	85	1	85
LbrM32_V2.1160	*L. braziliensis*	93	509	601
LbrM32_V2.1190	*L. braziliensis*	43	297	339
LbrM32_V2.1190	*L. braziliensis*	74	370	443
LbrM32_V2.1190	*L. braziliensis*	107	831	937
LbrM32_V2.1190	*L. braziliensis*	57	957	1013
LbrM32_V2.1190	*L. braziliensis*	45	1033	1077
LbrM32_V2.1210	*L. braziliensis*	60	1	60
LbrM32_V2.1230	*L. braziliensis*	196	1	196
LbrM32_V2.1230	*L. braziliensis*	848	216	1063
LbrM32_V2.1240	*L. braziliensis*	45	146	190
LbrM32_V2.1250	*L. braziliensis*	144	1	144
LbrM32_V2.1260	*L. braziliensis*	43	90	132
LbrM32_V2.1270	*L. braziliensis*	43	90	132
LbrM32_V2.1280	*L. braziliensis*	151	1	151
LbrM32_V2.1280	*L. braziliensis*	166	164	329

LbrM32_V2.1280	*L. braziliensis*	204	361	564
LbrM32_V2.1290	*L. braziliensis*	46	19	64
LbrM32_V2.1290	*L. braziliensis*	106	79	184
LbrM32_V2.1290	*L. braziliensis*	49	222	270
LbrM32_V2.1290	*L. braziliensis*	44	396	439
LbrM32_V2.1290	*L. braziliensis*	59	495	553
LbrM32_V2.1300	*L. braziliensis*	40	184	223
LbrM32_V2.1330	*L. braziliensis*	110	146	255
LbrM32_V2.1330	*L. braziliensis*	80	418	497
LbrM32_V2.1330	*L. braziliensis*	135	532	666
LbrM32_V2.1340	*L. braziliensis*	50	92	141
LbrM32_V2.1340	*L. braziliensis*	193	186	378
LbrM32_V2.1340	*L. braziliensis*	123	405	527
LbrM32_V2.1360	*L. braziliensis*	53	365	417
LbrM32_V2.1370	*L. braziliensis*	40	219	258
LbrM32_V2.1370	*L. braziliensis*	133	399	531
LbrM32_V2.1370	*L. braziliensis*	107	584	690
LbrM32_V2.1370	*L. braziliensis*	93	705	797
LbrM32_V2.1380	*L. braziliensis*	73	120	192
LbrM32_V2.1380	*L. braziliensis*	170	239	408
LbrM32_V2.1390	*L. braziliensis*	55	109	163
LbrM32_V2.1390	*L. braziliensis*	77	459	535
LbrM32_V2.1390	*L. braziliensis*	41	779	819
LbrM32_V2.1400	*L. braziliensis*	103	67	169
LbrM32_V2.1410	*L. braziliensis*	41	397	437
LbrM32_V2.1420	*L. braziliensis*	40	316	355
LbrM32_V2.1420	*L. braziliensis*	47	396	442
LbrM32_V2.1420	*L. braziliensis*	47	396	442
LbrM32_V2.1430	*L. braziliensis*	44	26	69
LbrM32_V2.1430	*L. braziliensis*	174	169	342
LbrM32_V2.1430	*L. braziliensis*	41	450	490
LbrM32_V2.1430	*L. braziliensis*	44	594	637
LbrM32_V2.1450	*L. braziliensis*	69	86	154
LbrM32_V2.1460	*L. braziliensis*	63	1	63
LbrM32_V2.1470	*L. braziliensis*	111	112	222
LbrM32_V2.1470	*L. braziliensis*	156	226	381
LbrM32_V2.1480	*L. braziliensis*	256	98	353
LbrM32_V2.1480	*L. braziliensis*	98	387	484
LbrM32_V2.1490	*L. braziliensis*	102	1	102
LbrM32_V2.1500	*L. braziliensis*	108	167	274
LbrM32_V2.1500	*L. braziliensis*	47	581	627
LbrM32_V2.1500	*L. braziliensis*	62	629	690
LbrM32_V2.1500	*L. braziliensis*	47	1118	1164
LbrM32_V2.1510	*L. braziliensis*	46	33	78
LbrM32_V2.1520	*L. braziliensis*	115	1	115
LbrM32_V2.1530	*L. braziliensis*	243	1	243
LbrM32_V2.1530	*L. braziliensis*	44	276	319

LbrM32_V2.1540	*L. braziliensis*	154	88	241
LbrM32_V2.1540	*L. braziliensis*	65	288	352
LbrM32_V2.1550	*L. braziliensis*	81	99	179
LbrM32_V2.1560	*L. braziliensis*	49	324	372
LbrM32_V2.1560	*L. braziliensis*	93	628	720
LbrM32_V2.1560	*L. braziliensis*	44	928	971
LbrM32_V2.1560	*L. braziliensis*	44	995	1038
LbrM32_V2.1560	*L. braziliensis*	299	1131	1429
LbrM32_V2.1560	*L. braziliensis*	170	1509	1678
LbrM32_V2.1570	*L. braziliensis*	49	253	301
LbrM32_V2.1580	*L. braziliensis*	41	458	498
LbrM32_V2.1590	*L. braziliensis*	461	1	461
LbrM32_V2.1590	*L. braziliensis*	168	790	957
LbrM32_V2.1600	*L. braziliensis*	44	104	147
LbrM32_V2.1610	*L. braziliensis*	49	1	49
LbrM32_V2.1620	*L. braziliensis*	52	1	52
LbrM32_V2.1620	*L. braziliensis*	53	202	254
LbrM32_V2.1620	*L. braziliensis*	94	860	953
LbrM32_V2.1620	*L. braziliensis*	74	1375	1448
LbrM32_V2.1630	*L. braziliensis*	80	123	202
LbrM32_V2.1630	*L. braziliensis*	48	232	279
LbrM32_V2.1630	*L. braziliensis*	48	232	279
LbrM32_V2.1640	*L. braziliensis*	62	189	250
LbrM32_V2.1640	*L. braziliensis*	51	423	473
LbrM32_V2.1640	*L. braziliensis*	51	423	473
LbrM32_V2.1650	*L. braziliensis*	190	1	190
LbrM32_V2.1660	*L. braziliensis*	131	1	131
LbrM32_V2.1660	*L. braziliensis*	104	162	265
LbrM32_V2.1670	*L. braziliensis*	106	1	106
LbrM32_V2.1670	*L. braziliensis*	48	154	201
LbrM32_V2.1670	*L. braziliensis*	53	407	459
LbrM32_V2.1690	*L. braziliensis*	128	108	235
LbrM32_V2.1690	*L. braziliensis*	106	514	619
LbrM32_V2.1690	*L. braziliensis*	106	514	619
LbrM32_V2.1700	*L. braziliensis*	86	1	86
LbrM32_V2.1700	*L. braziliensis*	49	304	352
LbrM32_V2.1700	*L. braziliensis*	49	304	352
LbrM32_V2.1710	*L. braziliensis*	65	60	124
LbrM32_V2.1710	*L. braziliensis*	50	183	232
LbrM32_V2.1710	*L. braziliensis*	50	183	232
LbrM32_V2.1720	*L. braziliensis*	93	106	198
LbrM32_V2.1730	*L. braziliensis*	90	1	90
LbrM32_V2.1730	*L. braziliensis*	85	779	863
LbrM32_V2.1740	*L. braziliensis*	69	135	203
LbrM32_V2.1740	*L. braziliensis*	353	242	594
LbrM32_V2.1740	*L. braziliensis*	113	624	736
LbrM32_V2.1760	*L. braziliensis*	67	47	113

LbrM32_V2.1760	*L. braziliensis*	43	206	248
LbrM32_V2.1760	*L. braziliensis*	59	362	420
LbrM32_V2.1760	*L. braziliensis*	40	647	686
LbrM32_V2.1770	*L. braziliensis*	560	1	560
LbrM32_V2.1770	*L. braziliensis*	59	608	666
LbrM32_V2.1790	*L. braziliensis*	62	300	361
LbrM32_V2.1790	*L. braziliensis*	47	722	768
LbrM32_V2.1800	*L. braziliensis*	109	147	255
LbrM32_V2.1800	*L. braziliensis*	122	268	389
LbrM32_V2.1810	*L. braziliensis*	83	133	215
LbrM32_V2.1820	*L. braziliensis*	207	1	207
LbrM32_V2.1820	*L. braziliensis*	159	219	377
LbrM32_V2.1820	*L. braziliensis*	70	430	499
LbrM32_V2.1830	*L. braziliensis*	266	1	266
LbrM32_V2.1830	*L. braziliensis*	510	270	779
LbrM32_V2.1830	*L. braziliensis*	110	849	958
LbrM32_V2.1830	*L. braziliensis*	141	981	1121
LbrM32_V2.1830	*L. braziliensis*	53	1137	1189
LbrM32_V2.1860	*L. braziliensis*	44	1	44
LbrM32_V2.1860	*L. braziliensis*	170	397	566
LbrM32_V2.1870	*L. braziliensis*	56	209	264
LbrM32_V2.1870	*L. braziliensis*	51	294	344
LbrM32_V2.1870	*L. braziliensis*	103	398	500
LbrM32_V2.1880	*L. braziliensis*	81	292	372
LbrM32_V2.1890	*L. braziliensis*	83	541	623
LbrM32_V2.1910	*L. braziliensis*	75	53	127
LbrM32_V2.1920	*L. braziliensis*	66	368	433
LbrM32_V2.1920	*L. braziliensis*	63	708	770
LbrM32_V2.1930	*L. braziliensis*	47	108	154
LbrM32_V2.1930	*L. braziliensis*	128	195	322
LbrM32_V2.1930	*L. braziliensis*	124	352	475
LbrM32_V2.1940	*L. braziliensis*	43	25	67
LbrM32_V2.1950	*L. braziliensis*	68	1	68
LbrM32_V2.1960	*L. braziliensis*	53	249	301
LbrM32_V2.1970	*L. braziliensis*	244	1	244
LbrM32_V2.1980	*L. braziliensis*	79	43	121
LbrM32_V2.1990	*L. braziliensis*	68	58	125
LbrM32_V2.2030	*L. braziliensis*	54	541	594
LbrM32_V2.2040	*L. braziliensis*	98	106	203
LbrM32_V2.2050	*L. braziliensis*	41	176	216
LbrM32_V2.2050	*L. braziliensis*	250	360	609
LbrM32_V2.2060	*L. braziliensis*	78	1	78
LbrM32_V2.2080	*L. braziliensis*	42	517	558
LbrM32_V2.2080	*L. braziliensis*	46	591	636
LbrM32_V2.2080	*L. braziliensis*	50	1073	1122
LbrM32_V2.2090	*L. braziliensis*	99	129	227
LbrM32_V2.2090	*L. braziliensis*	150	284	433

LbrM32_V2.2090	*L. braziliensis*	93	478	570
LbrM32_V2.2100	*L. braziliensis*	109	336	444
LbrM32_V2.2120	*L. braziliensis*	303	1	303
LbrM32_V2.2120	*L. braziliensis*	212	386	597
LbrM32_V2.2120	*L. braziliensis*	47	707	753
LbrM32_V2.2140	*L. braziliensis*	77	1	77
LbrM32_V2.2140	*L. braziliensis*	66	303	368
LbrM32_V2.2140	*L. braziliensis*	81	370	450
LbrM32_V2.2160	*L. braziliensis*	102	1	102
LbrM32_V2.2160	*L. braziliensis*	95	206	300
LbrM32_V2.2160	*L. braziliensis*	55	367	421
LbrM32_V2.2170	*L. braziliensis*	42	1	42
LbrM32_V2.2170	*L. braziliensis*	73	221	293
LbrM32_V2.2180	*L. braziliensis*	148	1	148
LbrM32_V2.2190	*L. braziliensis*	144	37	180
LbrM32_V2.2190	*L. braziliensis*	105	227	331
LbrM32_V2.2190	*L. braziliensis*	115	380	494
LbrM32_V2.2190	*L. braziliensis*	109	497	605
LbrM32_V2.2190	*L. braziliensis*	99	628	726
LbrM32_V2.2200	*L. braziliensis*	110	1	110
LbrM32_V2.2210	*L. braziliensis*	101	270	370
LbrM32_V2.2230	*L. braziliensis*	52	71	122
LbrM32_V2.2250	*L. braziliensis*	218	1	218
LbrM32_V2.2250	*L. braziliensis*	87	231	317
LbrM32_V2.2250	*L. braziliensis*	284	549	832
LbrM32_V2.2270	*L. braziliensis*	41	585	625
LbrM32_V2.2290	*L. braziliensis*	88	41	128
LbrM32_V2.2290	*L. braziliensis*	62	187	248
LbrM32_V2.2290	*L. braziliensis*	45	291	335
LbrM32_V2.2290	*L. braziliensis*	52	407	458
LbrM32_V2.2310	*L. braziliensis*	48	80	127
LbrM32_V2.2310	*L. braziliensis*	231	312	542
LbrM32_V2.2310	*L. braziliensis*	115	560	674
LbrM32_V2.2310	*L. braziliensis*	178	723	900
LbrM32_V2.2320	*L. braziliensis*	112	45	156
LbrM32_V2.2320	*L. braziliensis*	148	208	355
LbrM32_V2.2320	*L. braziliensis*	193	463	655
LbrM32_V2.2340	*L. braziliensis*	114	64	177
LbrM32_V2.2340	*L. braziliensis*	192	212	403
LbrM32_V2.2370	*L. braziliensis*	131	17	147
LbrM32_V2.2370	*L. braziliensis*	80	358	437
LbrM32_V2.2380	*L. braziliensis*	175	1130	1304
LbrM32_V2.2390	*L. braziliensis*	132	68	199
LbrM32_V2.2410	*L. braziliensis*	349	1	349
LbrM32_V2.2410	*L. braziliensis*	91	475	565
LbrM32_V2.2420	*L. braziliensis*	126	1	126
LbrM32_V2.2430	*L. braziliensis*	62	145	206

LbrM32_V2.2440	*L. braziliensis*	114	82	195
LbrM32_V2.2440	*L. braziliensis*	893	198	1090
LbrM32_V2.2440	*L. braziliensis*	273	1099	1371
LbrM32_V2.2440	*L. braziliensis*	155	1491	1645
LbrM32_V2.2450	*L. braziliensis*	195	1	195
LbrM32_V2.2450	*L. braziliensis*	59	426	484
LbrM32_V2.2460	*L. braziliensis*	52	1	52
LbrM32_V2.2460	*L. braziliensis*	80	104	183
LbrM32_V2.2460	*L. braziliensis*	48	314	361
LbrM32_V2.2460	*L. braziliensis*	96	436	531
LbrM32_V2.2460	*L. braziliensis*	128	791	918
LbrM32_V2.2480	*L. braziliensis*	48	1	48
LbrM32_V2.2480	*L. braziliensis*	65	138	202
LbrM32_V2.2480	*L. braziliensis*	65	138	202
LbrM32_V2.2490	*L. braziliensis*	60	1	60
LbrM32_V2.2490	*L. braziliensis*	71	117	187
LbrM32_V2.2490	*L. braziliensis*	71	117	187
LbrM32_V2.2500	*L. braziliensis*	1023	227	1249
LbrM32_V2.2500	*L. braziliensis*	207	1265	1471
LbrM32_V2.2520	*L. braziliensis*	149	1	149
LbrM32_V2.2530	*L. braziliensis*	87	1	87
LbrM32_V2.2530	*L. braziliensis*	226	190	415
LbrM32_V2.2540	*L. braziliensis*	103	173	275
LbrM32_V2.2540	*L. braziliensis*	40	586	625
LbrM32_V2.2540	*L. braziliensis*	149	654	802
LbrM32_V2.2540	*L. braziliensis*	96	958	1053
LbrM32_V2.2540	*L. braziliensis*	53	1070	1122
LbrM32_V2.2540	*L. braziliensis*	40	1153	1192
LbrM32_V2.2580	*L. braziliensis*	397	1	397
LbrM32_V2.2590	*L. braziliensis*	72	49	120
LbrM32_V2.2590	*L. braziliensis*	74	389	462
LbrM32_V2.2590	*L. braziliensis*	74	389	462
LbrM32_V2.2610	*L. braziliensis*	77	328	404
LbrM32_V2.2620	*L. braziliensis*	43	1	43
LbrM32_V2.2620	*L. braziliensis*	97	83	179
LbrM32_V2.2620	*L. braziliensis*	276	196	471
LbrM32_V2.2650	*L. braziliensis*	45	1	45
LbrM32_V2.2650	*L. braziliensis*	68	314	381
LbrM32_V2.2650	*L. braziliensis*	101	436	536
LbrM32_V2.2650	*L. braziliensis*	287	543	829
LbrM32_V2.2650	*L. braziliensis*	190	864	1053
LbrM32_V2.2650	*L. braziliensis*	94	1064	1157
LbrM32_V2.2650	*L. braziliensis*	311	1177	1487
LbrM32_V2.2650	*L. braziliensis*	184	1520	1703
LbrM32_V2.2650	*L. braziliensis*	58	1768	1825
LbrM32_V2.2650	*L. braziliensis*	63	1889	1951
LbrM32_V2.2650	*L. braziliensis*	167	1960	2126

LbrM32_V2.2660	*L. braziliensis*	110	33	142
LbrM32_V2.2660	*L. braziliensis*	68	230	297
LbrM32_V2.2660	*L. braziliensis*	59	479	537
LbrM32_V2.2660	*L. braziliensis*	75	792	866
LbrM32_V2.2670	*L. braziliensis*	56	77	132
LbrM32_V2.2680	*L. braziliensis*	52	1	52
LbrM32_V2.2680	*L. braziliensis*	56	361	416
LbrM32_V2.2700	*L. braziliensis*	140	121	260
LbrM32_V2.2700	*L. braziliensis*	623	308	930
LbrM32_V2.2710	*L. braziliensis*	52	1	52
LbrM32_V2.2720	*L. braziliensis*	144	39	182
LbrM32_V2.2720	*L. braziliensis*	98	615	712
LbrM32_V2.2720	*L. braziliensis*	41	1142	1182
LbrM32_V2.2740	*L. braziliensis*	66	60	125
LbrM32_V2.2740	*L. braziliensis*	46	458	503
LbrM32_V2.2740	*L. braziliensis*	46	458	503
LbrM32_V2.2750	*L. braziliensis*	70	1	70
LbrM32_V2.2750	*L. braziliensis*	831	82	912
LbrM32_V2.2760	*L. braziliensis*	74	149	222
LbrM32_V2.2760	*L. braziliensis*	75	448	522
LbrM32_V2.2770	*L. braziliensis*	81	531	611
LbrM32_V2.2770	*L. braziliensis*	55	619	673
LbrM32_V2.2780	*L. braziliensis*	54	123	176
LbrM32_V2.2780	*L. braziliensis*	55	1390	1444
LbrM32_V2.2780	*L. braziliensis*	44	1488	1531
LbrM32_V2.2790	*L. braziliensis*	48	133	180
LbrM32_V2.2800	*L. braziliensis*	335	1	335
LbrM32_V2.2800	*L. braziliensis*	85	342	426
LbrM32_V2.2800	*L. braziliensis*	76	439	514
LbrM32_V2.2800	*L. braziliensis*	49	520	568
LbrM32_V2.2800	*L. braziliensis*	138	594	731
LbrM32_V2.2810	*L. braziliensis*	51	316	366
LbrM32_V2.2820	*L. braziliensis*	50	1	50
LbrM32_V2.2830	*L. braziliensis*	51	1	51
LbrM32_V2.2830	*L. braziliensis*	124	321	444
LbrM32_V2.2830	*L. braziliensis*	169	547	715
LbrM32_V2.2840	*L. braziliensis*	467	1	467
LbrM32_V2.2840	*L. braziliensis*	367	482	848
LbrM32_V2.2890	*L. braziliensis*	67	222	288
LbrM32_V2.2900	*L. braziliensis*	59	1	59
LbrM32_V2.2910	*L. braziliensis*	40	24	63
LbrM32_V2.2910	*L. braziliensis*	132	120	251
LbrM32_V2.2910	*L. braziliensis*	66	363	428
LbrM32_V2.2910	*L. braziliensis*	77	440	516
LbrM32_V2.2910	*L. braziliensis*	94	543	636
LbrM32_V2.2940	*L. braziliensis*	57	226	282
LbrM32_V2.2940	*L. braziliensis*	51	590	640

LbrM32_V2.2970	*L. braziliensis*	43	302	344
LbrM32_V2.2970	*L. braziliensis*	53	405	457
LbrM32_V2.2970	*L. braziliensis*	53	405	457
LbrM32_V2.2990	*L. braziliensis*	149	1	149
LbrM32_V2.2990	*L. braziliensis*	160	437	596
LbrM32_V2.2990	*L. braziliensis*	71	832	902
LbrM32_V2.2990	*L. braziliensis*	56	1168	1223
LbrM32_V2.3000	*L. braziliensis*	262	1	262
LbrM32_V2.3000	*L. braziliensis*	40	390	429
LbrM32_V2.3000	*L. braziliensis*	203	490	692
LbrM32_V2.3000	*L. braziliensis*	152	702	853
LbrM32_V2.3010	*L. braziliensis*	50	1	50
LbrM32_V2.3010	*L. braziliensis*	791	161	951
LbrM32_V2.3020	*L. braziliensis*	48	984	1031
LbrM32_V2.3020	*L. braziliensis*	56	1037	1092
LbrM32_V2.3020	*L. braziliensis*	56	1037	1092
LbrM32_V2.3030	*L. braziliensis*	48	1	48
LbrM32_V2.3040	*L. braziliensis*	178	1	178
LbrM32_V2.3040	*L. braziliensis*	68	413	480
LbrM32_V2.3050	*L. braziliensis*	127	1	127
LbrM32_V2.3050	*L. braziliensis*	43	215	257
LbrM32_V2.3060	*L. braziliensis*	71	1	71
LbrM32_V2.3070	*L. braziliensis*	283	33	315
LbrM32_V2.3070	*L. braziliensis*	62	400	461
LbrM32_V2.3070	*L. braziliensis*	51	463	513
LbrM32_V2.3070	*L. braziliensis*	528	593	1120
LbrM32_V2.3080	*L. braziliensis*	141	110	250
LbrM32_V2.3080	*L. braziliensis*	90	262	351
LbrM32_V2.3080	*L. braziliensis*	80	376	455
LbrM32_V2.3090	*L. braziliensis*	184	96	279
LbrM32_V2.3090	*L. braziliensis*	54	393	446
LbrM32_V2.3090	*L. braziliensis*	50	499	548
LbrM32_V2.3100	*L. braziliensis*	130	168	297
LbrM32_V2.3100	*L. braziliensis*	153	312	464
LbrM32_V2.3120	*L. braziliensis*	317	1	317
LbrM32_V2.3120	*L. braziliensis*	116	384	499
LbrM32_V2.3120	*L. braziliensis*	84	528	611
LbrM32_V2.3120	*L. braziliensis*	60	659	718
LbrM32_V2.3130	*L. braziliensis*	46	134	179
LbrM32_V2.3130	*L. braziliensis*	71	183	253
LbrM32_V2.3130	*L. braziliensis*	43	343	385
LbrM32_V2.3140	*L. braziliensis*	92	1	92
LbrM32_V2.3150	*L. braziliensis*	63	1	63
LbrM32_V2.3150	*L. braziliensis*	147	161	307
LbrM32_V2.3150	*L. braziliensis*	49	354	402
LbrM32_V2.3150	*L. braziliensis*	77	472	548
LbrM32_V2.3150	*L. braziliensis*	62	557	618

LbrM32_V2.3160	*L. braziliensis*	101	15	115
LbrM32_V2.3160	*L. braziliensis*	47	883	929
LbrM32_V2.3160	*L. braziliensis*	44	1063	1106
LbrM32_V2.3180	*L. braziliensis*	54	344	397
LbrM32_V2.3220	*L. braziliensis*	41	625	665
LbrM32_V2.3230	*L. braziliensis*	80	1	80
LbrM32_V2.3240	*L. braziliensis*	53	1	53
LbrM32_V2.3260	*L. braziliensis*	61	681	741
LbrM32_V2.3300	*L. braziliensis*	80	806	885
LbrM32_V2.3310	*L. braziliensis*	129	352	480
LbrM32_V2.3310	*L. braziliensis*	74	488	561
LbrM32_V2.3310	*L. braziliensis*	41	652	692
LbrM32_V2.3310	*L. braziliensis*	41	722	762
LbrM32_V2.3320	*L. braziliensis*	192	80	271
LbrM32_V2.3340	*L. braziliensis*	41	1	41
LbrM32_V2.3350	*L. braziliensis*	89	39	127
LbrM32_V2.3350	*L. braziliensis*	58	185	242
LbrM32_V2.3350	*L. braziliensis*	140	422	561
LbrM32_V2.3350	*L. braziliensis*	56	628	683
LbrM32_V2.3350	*L. braziliensis*	242	851	1092
LbrM32_V2.3370	*L. braziliensis*	232	1	232
LbrM32_V2.3370	*L. braziliensis*	133	313	445
LbrM32_V2.3370	*L. braziliensis*	49	615	663
LbrM32_V2.3380	*L. braziliensis*	64	1	64
LbrM32_V2.3390	*L. braziliensis*	301	1	301
LbrM32_V2.3390	*L. braziliensis*	173	305	477
LbrM32_V2.3390	*L. braziliensis*	184	497	680
LbrM32_V2.3390	*L. braziliensis*	49	738	786
LbrM32_V2.3390	*L. braziliensis*	51	814	864
LbrM32_V2.3390	*L. braziliensis*	63	925	987
LbrM32_V2.3390	*L. braziliensis*	46	998	1043
LbrM32_V2.3390	*L. braziliensis*	177	1256	1432
LbrM32_V2.3390	*L. braziliensis*	75	1434	1508
LbrM32_V2.3400	*L. braziliensis*	46	214	259
LbrM32_V2.3400	*L. braziliensis*	51	420	470
LbrM32_V2.3400	*L. braziliensis*	51	420	470
LbrM32_V2.3410	*L. braziliensis*	42	1	42
LbrM32_V2.3420	*L. braziliensis*	87	68	154
LbrM32_V2.3430	*L. braziliensis*	56	1	56
LbrM32_V2.3440	*L. braziliensis*	98	1	98
LbrM32_V2.3440	*L. braziliensis*	365	280	644
LbrM32_V2.3440	*L. braziliensis*	74	677	750
LbrM32_V2.3440	*L. braziliensis*	54	760	813
LbrM32_V2.3440	*L. braziliensis*	55	862	916
LbrM32_V2.3440	*L. braziliensis*	89	988	1076
LbrM32_V2.3440	*L. braziliensis*	352	1086	1437
LbrM32_V2.3440	*L. braziliensis*	79	1448	1526

LbrM32_V2.3440	*L. braziliensis*	325	1595	1919
LbrM32_V2.3440	*L. braziliensis*	70	2060	2129
LbrM32_V2.3440	*L. braziliensis*	101	2151	2251
LbrM32_V2.3440	*L. braziliensis*	65	2348	2412
LbrM32_V2.3450	*L. braziliensis*	88	39	126
LbrM32_V2.3450	*L. braziliensis*	85	248	332
LbrM32_V2.3450	*L. braziliensis*	114	537	650
LbrM32_V2.3450	*L. braziliensis*	70	701	770
LbrM32_V2.3470	*L. braziliensis*	46	251	296
LbrM32_V2.3470	*L. braziliensis*	45	631	675
LbrM32_V2.3470	*L. braziliensis*	45	631	675
LbrM32_V2.3480	*L. braziliensis*	44	177	220
LbrM32_V2.3480	*L. braziliensis*	51	452	502
LbrM32_V2.3480	*L. braziliensis*	42	737	778
LbrM32_V2.3480	*L. braziliensis*	54	937	990
LbrM32_V2.3490	*L. braziliensis*	63	12	74
LbrM32_V2.3490	*L. braziliensis*	95	142	236
LbrM32_V2.3490	*L. braziliensis*	62	301	362
LbrM32_V2.3490	*L. braziliensis*	143	391	533
LbrM32_V2.3490	*L. braziliensis*	53	822	874
LbrM32_V2.3490	*L. braziliensis*	44	1081	1124
LbrM32_V2.3490	*L. braziliensis*	40	1160	1199
LbrM32_V2.3510	*L. braziliensis*	286	1	286
LbrM32_V2.3510	*L. braziliensis*	72	403	474
LbrM32_V2.3520	*L. braziliensis*	65	1	65
LbrM32_V2.3520	*L. braziliensis*	98	331	428
LbrM32_V2.3530	*L. braziliensis*	59	203	261
LbrM32_V2.3530	*L. braziliensis*	80	511	590
LbrM32_V2.3530	*L. braziliensis*	48	1124	1171
LbrM32_V2.3540	*L. braziliensis*	635	287	921
LbrM32_V2.3540	*L. braziliensis*	91	1018	1108
LbrM32_V2.3570	*L. braziliensis*	49	20	68
LbrM32_V2.3570	*L. braziliensis*	51	194	244
LbrM32_V2.3570	*L. braziliensis*	50	367	416
LbrM32_V2.3570	*L. braziliensis*	159	462	620
LbrM32_V2.3570	*L. braziliensis*	122	695	816
LbrM32_V2.3580	*L. braziliensis*	81	160	240
LbrM32_V2.3590	*L. braziliensis*	81	86	166
LbrM32_V2.3590	*L. braziliensis*	103	212	314
LbrM32_V2.3590	*L. braziliensis*	103	212	314
LbrM32_V2.3600	*L. braziliensis*	48	224	271
LbrM32_V2.3620	*L. braziliensis*	49	66	114
LbrM32_V2.3630	*L. braziliensis*	43	38	80
LbrM32_V2.3630	*L. braziliensis*	47	137	183
LbrM32_V2.3630	*L. braziliensis*	88	810	897
LbrM32_V2.3650	*L. braziliensis*	55	230	284
LbrM32_V2.3650	*L. braziliensis*	257	389	645

LbrM32_V2.3650	*L. braziliensis*	110	664	773
LbrM32_V2.3650	*L. braziliensis*	84	1028	1111
LbrM32_V2.3650	*L. braziliensis*	49	1181	1229
LbrM32_V2.3660	*L. braziliensis*	172	1	172
LbrM32_V2.3660	*L. braziliensis*	56	229	284
LbrM32_V2.3680	*L. braziliensis*	103	62	164
LbrM32_V2.3680	*L. braziliensis*	78	291	368
LbrM32_V2.3680	*L. braziliensis*	60	387	446
LbrM32_V2.3690	*L. braziliensis*	47	18	64
LbrM32_V2.3690	*L. braziliensis*	47	93	139
LbrM32_V2.3690	*L. braziliensis*	48	553	600
LbrM32_V2.3700	*L. braziliensis*	44	385	428
LbrM32_V2.3720	*L. braziliensis*	76	1	76
LbrM32_V2.3720	*L. braziliensis*	61	131	191
LbrM32_V2.3720	*L. braziliensis*	175	251	425
LbrM32_V2.3720	*L. braziliensis*	99	437	535
LbrM32_V2.3720	*L. braziliensis*	80	564	643
LbrM32_V2.3720	*L. braziliensis*	151	652	802
LbrM32_V2.3730	*L. braziliensis*	74	180	253
LbrM32_V2.3740	*L. braziliensis*	69	1	69
LbrM32_V2.3740	*L. braziliensis*	54	109	162
LbrM32_V2.3740	*L. braziliensis*	296	199	494
LbrM32_V2.3740	*L. braziliensis*	71	538	608
LbrM32_V2.3760	*L. braziliensis*	90	106	195
LbrM32_V2.3760	*L. braziliensis*	97	358	454
LbrM32_V2.3770	*L. braziliensis*	151	1	151
LbrM32_V2.3770	*L. braziliensis*	50	342	391
LbrM32_V2.3780	*L. braziliensis*	63	1	63
LbrM32_V2.3780	*L. braziliensis*	75	515	589
LbrM32_V2.3800	*L. braziliensis*	65	76	140
LbrM32_V2.3800	*L. braziliensis*	66	173	238
LbrM32_V2.3800	*L. braziliensis*	73	453	525
LbrM32_V2.3810	*L. braziliensis*	63	64	126
LbrM32_V2.3810	*L. braziliensis*	48	234	281
LbrM32_V2.3810	*L. braziliensis*	136	314	449
LbrM32_V2.3830	*L. braziliensis*	51	51	101
LbrM32_V2.3830	*L. braziliensis*	83	117	199
LbrM32_V2.3830	*L. braziliensis*	106	430	535
LbrM32_V2.3840	*L. braziliensis*	156	1	156
LbrM32_V2.3840	*L. braziliensis*	79	236	314
LbrM32_V2.3840	*L. braziliensis*	61	325	385
LbrM32_V2.3840	*L. braziliensis*	95	528	622
LbrM32_V2.3840	*L. braziliensis*	60	1016	1075
LbrM32_V2.3850	*L. braziliensis*	73	1	73
LbrM32_V2.3850	*L. braziliensis*	41	106	146
LbrM32_V2.3850	*L. braziliensis*	41	106	146
LbrM32_V2.3870	*L. braziliensis*	118	1	118

LbrM32_V2.3880	*L. braziliensis*	235	1	235
LbrM32_V2.3880	*L. braziliensis*	66	255	320
LbrM32_V2.3890	*L. braziliensis*	73	353	425
LbrM32_V2.3940	*L. braziliensis*	72	286	357
LbrM32_V2.3940	*L. braziliensis*	92	546	637
LbrM32_V2.3940	*L. braziliensis*	66	663	728
LbrM32_V2.3950	*L. braziliensis*	49	1	49
LbrM32_V2.3950	*L. braziliensis*	44	92	135
LbrM32_V2.3950	*L. braziliensis*	44	92	135
LbrM32_V2.3990	*L. braziliensis*	54	54	107
LbrM32_V2.4010	*L. braziliensis*	70	63	132
LbrM32_V2.4020	*L. braziliensis*	63	1	63
LbrM32_V2.4020	*L. braziliensis*	48	96	143
LbrM32_V2.4020	*L. braziliensis*	48	96	143
LbrM32_V2.4040	*L. braziliensis*	46	31	76
LbrM32_V2.4050	*L. braziliensis*	44	112	155
LbrM32_V2.4050	*L. braziliensis*	56	321	376
LbrM32_V2.4090	*L. braziliensis*	178	1	178
LbrM32_V2.4090	*L. braziliensis*	67	222	288
LbrM32_V2.4090	*L. braziliensis*	67	222	288
LbrM32_V2.4100	*L. braziliensis*	41	357	397
LbrM32_V2.4110	*L. braziliensis*	43	589	631
LbrM32_V2.4130	*L. braziliensis*	60	339	398
LbrM32_V2.4140	*L. braziliensis*	63	198	260
LbrM32_V2.4160	*L. braziliensis*	71	696	766
LbrM32_V2.4170	*L. braziliensis*	54	99	152
LbrM32_V2.4170	*L. braziliensis*	52	1262	1313
LbrM32_V2.4170	*L. braziliensis*	52	1262	1313
LbrM32_V2.4190	*L. braziliensis*	108	807	914
LbrM32_V2.4220	*L. braziliensis*	58	123	180
LbrM32_V2.4230	*L. braziliensis*	78	56	133
LbrM33_V2.0010	*L. braziliensis*	54	769	822
LbrM33_V2.0030	*L. braziliensis*	44	424	467
LbrM33_V2.0040	*L. braziliensis*	80	246	325
LbrM33_V2.0050	*L. braziliensis*	76	144	219
LbrM33_V2.0060	*L. braziliensis*	102	1	102
LbrM33_V2.0070	*L. braziliensis*	55	172	226
LbrM33_V2.0070	*L. braziliensis*	74	334	407
LbrM33_V2.0070	*L. braziliensis*	74	334	407
LbrM33_V2.0080	*L. braziliensis*	74	101	174
LbrM33_V2.0110	*L. braziliensis*	91	1	91
LbrM33_V2.0120	*L. braziliensis*	102	89	190
LbrM33_V2.0120	*L. braziliensis*	83	221	303
LbrM33_V2.0130	*L. braziliensis*	107	223	329
LbrM33_V2.0150	*L. braziliensis*	45	364	408
LbrM33_V2.0160	*L. braziliensis*	86	194	279
LbrM33_V2.0210	*L. braziliensis*	65	1	65

LbrM33_V2.0210	*L. braziliensis*	74	480	553
LbrM33_V2.0210	*L. braziliensis*	80	807	886
LbrM33_V2.0210	*L. braziliensis*	40	1111	1150
LbrM33_V2.0210	*L. braziliensis*	61	1283	1343
LbrM33_V2.0210	*L. braziliensis*	112	1374	1485
LbrM33_V2.0220	*L. braziliensis*	146	115	260
LbrM33_V2.0220	*L. braziliensis*	78	516	593
LbrM33_V2.0220	*L. braziliensis*	78	516	593
LbrM33_V2.0230	*L. braziliensis*	96	1	96
LbrM33_V2.0240	*L. braziliensis*	74	156	229
LbrM33_V2.0260	*L. braziliensis*	44	1	44
LbrM33_V2.0260	*L. braziliensis*	53	131	183
LbrM33_V2.0280	*L. braziliensis*	42	164	205
LbrM33_V2.0280	*L. braziliensis*	42	299	340
LbrM33_V2.0280	*L. braziliensis*	42	299	340
LbrM33_V2.0290	*L. braziliensis*	74	14	87
LbrM33_V2.0320	*L. braziliensis*	60	1	60
LbrM33_V2.0320	*L. braziliensis*	44	131	174
LbrM33_V2.0320	*L. braziliensis*	74	592	665
LbrM33_V2.0330	*L. braziliensis*	58	163	220
LbrM33_V2.0340	*L. braziliensis*	104	176	279
LbrM33_V2.0340	*L. braziliensis*	58	508	565
LbrM33_V2.0350	*L. braziliensis*	58	183	240
LbrM33_V2.0360	*L. braziliensis*	188	1	188
LbrM33_V2.0360	*L. braziliensis*	107	233	339
LbrM33_V2.0380	*L. braziliensis*	190	66	255
LbrM33_V2.0390	*L. braziliensis*	41	1	41
LbrM33_V2.0400	*L. braziliensis*	51	875	925
LbrM33_V2.0420	*L. braziliensis*	51	216	266
LbrM33_V2.0420	*L. braziliensis*	93	561	653
LbrM33_V2.0420	*L. braziliensis*	68	674	741
LbrM33_V2.0430	*L. braziliensis*	43	109	151
LbrM33_V2.0430	*L. braziliensis*	45	176	220
LbrM33_V2.0430	*L. braziliensis*	45	176	220
LbrM33_V2.0440	*L. braziliensis*	60	88	147
LbrM33_V2.0450	*L. braziliensis*	45	33	77
LbrM33_V2.0450	*L. braziliensis*	113	104	216
LbrM33_V2.0460	*L. braziliensis*	53	489	541
LbrM33_V2.0470	*L. braziliensis*	228	18	245
LbrM33_V2.0470	*L. braziliensis*	65	257	321
LbrM33_V2.0480	*L. braziliensis*	95	34	128
LbrM33_V2.0480	*L. braziliensis*	96	152	247
LbrM33_V2.0480	*L. braziliensis*	55	297	351
LbrM33_V2.0480	*L. braziliensis*	40	420	459
LbrM33_V2.0480	*L. braziliensis*	93	582	674
LbrM33_V2.0480	*L. braziliensis*	61	800	860
LbrM33_V2.0500	*L. braziliensis*	43	16	58

LbrM33_V2.0500	*L. braziliensis*	52	294	345
LbrM33_V2.0500	*L. braziliensis*	56	640	695
LbrM33_V2.0530	*L. braziliensis*	90	75	164
LbrM33_V2.0530	*L. braziliensis*	73	201	273
LbrM33_V2.0530	*L. braziliensis*	85	491	575
LbrM33_V2.0530	*L. braziliensis*	55	680	734
LbrM33_V2.0550	*L. braziliensis*	76	135	210
LbrM33_V2.0550	*L. braziliensis*	93	339	431
LbrM33_V2.0560	*L. braziliensis*	77	49	125
LbrM33_V2.0560	*L. braziliensis*	41	653	693
LbrM33_V2.0560	*L. braziliensis*	46	765	810
LbrM33_V2.0560	*L. braziliensis*	46	977	1022
LbrM33_V2.0560	*L. braziliensis*	45	1106	1150
LbrM33_V2.0560	*L. braziliensis*	48	1310	1357
LbrM33_V2.0560	*L. braziliensis*	51	1420	1470
LbrM33_V2.0560	*L. braziliensis*	89	1862	1950
LbrM33_V2.0590	*L. braziliensis*	50	86	135
LbrM33_V2.0590	*L. braziliensis*	78	495	572
LbrM33_V2.0600	*L. braziliensis*	310	9	318
LbrM33_V2.0600	*L. braziliensis*	59	320	378
LbrM33_V2.0610	*L. braziliensis*	115	172	286
LbrM33_V2.0610	*L. braziliensis*	312	304	615
LbrM33_V2.0620	*L. braziliensis*	68	1	68
LbrM33_V2.0620	*L. braziliensis*	191	76	266
LbrM33_V2.0620	*L. braziliensis*	181	310	490
LbrM33_V2.0620	*L. braziliensis*	91	552	642
LbrM33_V2.0620	*L. braziliensis*	188	658	845
LbrM33_V2.0630	*L. braziliensis*	45	146	190
LbrM33_V2.0660	*L. braziliensis*	63	57	119
LbrM33_V2.0660	*L. braziliensis*	86	152	237
LbrM33_V2.0660	*L. braziliensis*	86	152	237
LbrM33_V2.0670	*L. braziliensis*	55	276	330
LbrM33_V2.0690	*L. braziliensis*	219	32	250
LbrM33_V2.0690	*L. braziliensis*	77	384	460
LbrM33_V2.0690	*L. braziliensis*	555	473	1027
LbrM33_V2.0690	*L. braziliensis*	364	1043	1406
LbrM33_V2.0710	*L. braziliensis*	280	72	351
LbrM33_V2.0710	*L. braziliensis*	53	390	442
LbrM33_V2.0710	*L. braziliensis*	44	788	831
LbrM33_V2.0720	*L. braziliensis*	51	1	51
LbrM33_V2.0740	*L. braziliensis*	46	196	241
LbrM33_V2.0750	*L. braziliensis*	46	180	225
LbrM33_V2.0760	*L. braziliensis*	74	86	159
LbrM33_V2.0770	*L. braziliensis*	135	1	135
LbrM33_V2.0780	*L. braziliensis*	92	177	268
LbrM33_V2.0780	*L. braziliensis*	49	274	322
LbrM33_V2.0790	*L. braziliensis*	164	145	308

LbrM33_V2.0790	*L. braziliensis*	52	487	538
LbrM33_V2.0790	*L. braziliensis*	59	685	743
LbrM33_V2.0800	*L. braziliensis*	70	1	70
LbrM33_V2.0800	*L. braziliensis*	40	120	159
LbrM33_V2.0800	*L. braziliensis*	59	183	241
LbrM33_V2.0800	*L. braziliensis*	94	255	348
LbrM33_V2.0810	*L. braziliensis*	51	9	59
LbrM33_V2.0810	*L. braziliensis*	185	68	252
LbrM33_V2.0810	*L. braziliensis*	148	255	402
LbrM33_V2.0820	*L. braziliensis*	59	115	173
LbrM33_V2.0820	*L. braziliensis*	237	316	552
LbrM33_V2.0820	*L. braziliensis*	242	640	881
LbrM33_V2.0840	*L. braziliensis*	120	130	249
LbrM33_V2.0900	*L. braziliensis*	54	1	54
LbrM33_V2.0900	*L. braziliensis*	46	81	126
LbrM33_V2.0900	*L. braziliensis*	283	179	461
LbrM33_V2.0910	*L. braziliensis*	220	33	252
LbrM33_V2.0910	*L. braziliensis*	54	274	327
LbrM33_V2.0910	*L. braziliensis*	332	600	931
LbrM33_V2.0920	*L. braziliensis*	40	404	443
LbrM33_V2.0930	*L. braziliensis*	40	404	443
LbrM33_V2.0940	*L. braziliensis*	156	309	464
LbrM33_V2.0950	*L. braziliensis*	40	184	223
LbrM33_V2.0960	*L. braziliensis*	40	404	443
LbrM33_V2.0970	*L. braziliensis*	55	1	55
LbrM33_V2.0970	*L. braziliensis*	238	63	300
LbrM33_V2.0970	*L. braziliensis*	343	341	683
LbrM33_V2.0970	*L. braziliensis*	70	762	831
LbrM33_V2.0970	*L. braziliensis*	93	943	1035
LbrM33_V2.0970	*L. braziliensis*	149	1121	1269
LbrM33_V2.0980	*L. braziliensis*	92	224	315
LbrM33_V2.0980	*L. braziliensis*	44	354	397
LbrM33_V2.0980	*L. braziliensis*	51	707	757
LbrM33_V2.0980	*L. braziliensis*	71	1049	1119
LbrM33_V2.0980	*L. braziliensis*	107	1519	1625
LbrM33_V2.1010	*L. braziliensis*	40	138	177
LbrM33_V2.1020	*L. braziliensis*	51	559	609
LbrM33_V2.1030	*L. braziliensis*	205	1	205
LbrM33_V2.1030	*L. braziliensis*	74	237	310
LbrM33_V2.1030	*L. braziliensis*	198	513	710
LbrM33_V2.1040	*L. braziliensis*	299	329	627
LbrM33_V2.1040	*L. braziliensis*	62	750	811
LbrM33_V2.1040	*L. braziliensis*	47	818	864
LbrM33_V2.1050	*L. braziliensis*	53	80	132
LbrM33_V2.1050	*L. braziliensis*	53	365	417
LbrM33_V2.1050	*L. braziliensis*	53	365	417
LbrM33_V2.1060	*L. braziliensis*	197	1	197

LbrM33_V2.1060	*L. braziliensis*	89	266	354
LbrM33_V2.1070	*L. braziliensis*	52	50	101
LbrM33_V2.1070	*L. braziliensis*	46	120	165
LbrM33_V2.1070	*L. braziliensis*	45	254	298
LbrM33_V2.1090	*L. braziliensis*	91	316	406
LbrM33_V2.1090	*L. braziliensis*	88	493	580
LbrM33_V2.1120	*L. braziliensis*	55	1	55
LbrM33_V2.1120	*L. braziliensis*	135	102	236
LbrM33_V2.1120	*L. braziliensis*	65	277	341
LbrM33_V2.1120	*L. braziliensis*	73	518	590
LbrM33_V2.1120	*L. braziliensis*	61	730	790
LbrM33_V2.1130	*L. braziliensis*	91	1	91
LbrM33_V2.1130	*L. braziliensis*	381	170	550
LbrM33_V2.1140	*L. braziliensis*	280	36	315
LbrM33_V2.1140	*L. braziliensis*	51	430	480
LbrM33_V2.1140	*L. braziliensis*	102	642	743
LbrM33_V2.1140	*L. braziliensis*	150	805	954
LbrM33_V2.1140	*L. braziliensis*	60	1021	1080
LbrM33_V2.1150	*L. braziliensis*	95	90	184
LbrM33_V2.1150	*L. braziliensis*	140	365	504
LbrM33_V2.1150	*L. braziliensis*	51	859	909
LbrM33_V2.1150	*L. braziliensis*	80	1032	1111
LbrM33_V2.1150	*L. braziliensis*	44	1286	1329
LbrM33_V2.1150	*L. braziliensis*	74	1423	1496
LbrM33_V2.1150	*L. braziliensis*	154	1575	1728
LbrM33_V2.1150	*L. braziliensis*	107	1742	1848
LbrM33_V2.1170	*L. braziliensis*	71	120	190
LbrM33_V2.1170	*L. braziliensis*	154	375	528
LbrM33_V2.1180	*L. braziliensis*	64	1	64
LbrM33_V2.1200	*L. braziliensis*	44	439	482
LbrM33_V2.1200	*L. braziliensis*	50	728	777
LbrM33_V2.1200	*L. braziliensis*	50	728	777
LbrM33_V2.1210	*L. braziliensis*	328	1	328
LbrM33_V2.1210	*L. braziliensis*	147	340	486
LbrM33_V2.1210	*L. braziliensis*	107	509	615
LbrM33_V2.1210	*L. braziliensis*	55	795	849
LbrM33_V2.1220	*L. braziliensis*	52	398	449
LbrM33_V2.1230	*L. braziliensis*	160	1	160
LbrM33_V2.1240	*L. braziliensis*	70	1	70
LbrM33_V2.1240	*L. braziliensis*	109	113	221
LbrM33_V2.1240	*L. braziliensis*	54	402	455
LbrM33_V2.1250	*L. braziliensis*	48	89	136
LbrM33_V2.1260	*L. braziliensis*	41	408	448
LbrM33_V2.1260	*L. braziliensis*	57	855	911
LbrM33_V2.1260	*L. braziliensis*	57	855	911
LbrM33_V2.1280	*L. braziliensis*	45	130	174
LbrM33_V2.1280	*L. braziliensis*	53	196	248

LbrM33_V2.1280	*L. braziliensis*	53	196	248
LbrM33_V2.1300	*L. braziliensis*	81	45	125
LbrM33_V2.1300	*L. braziliensis*	112	278	389
LbrM33_V2.1300	*L. braziliensis*	272	424	695
LbrM33_V2.1310	*L. braziliensis*	67	98	164
LbrM33_V2.1310	*L. braziliensis*	54	184	237
LbrM33_V2.1310	*L. braziliensis*	141	313	453
LbrM33_V2.1310	*L. braziliensis*	119	500	618
LbrM33_V2.1310	*L. braziliensis*	122	632	753
LbrM33_V2.1320	*L. braziliensis*	217	1	217
LbrM33_V2.1320	*L. braziliensis*	68	243	310
LbrM33_V2.1330	*L. braziliensis*	109	546	654
LbrM33_V2.1350	*L. braziliensis*	95	151	245
LbrM33_V2.1360	*L. braziliensis*	75	24	98
LbrM33_V2.1360	*L. braziliensis*	54	107	160
LbrM33_V2.1360	*L. braziliensis*	63	205	267
LbrM33_V2.1360	*L. braziliensis*	109	274	382
LbrM33_V2.1380	*L. braziliensis*	91	1	91
LbrM33_V2.1380	*L. braziliensis*	59	94	152
LbrM33_V2.1380	*L. braziliensis*	55	184	238
LbrM33_V2.1380	*L. braziliensis*	106	301	406
LbrM33_V2.1380	*L. braziliensis*	42	454	495
LbrM33_V2.1380	*L. braziliensis*	63	740	802
LbrM33_V2.1380	*L. braziliensis*	40	831	870
LbrM33_V2.1380	*L. braziliensis*	80	1114	1193
LbrM33_V2.1390	*L. braziliensis*	152	37	188
LbrM33_V2.1390	*L. braziliensis*	42	216	257
LbrM33_V2.1390	*L. braziliensis*	71	355	425
LbrM33_V2.1400	*L. braziliensis*	59	481	539
LbrM33_V2.1400	*L. braziliensis*	103	735	837
LbrM33_V2.1400	*L. braziliensis*	103	735	837
LbrM33_V2.1410	*L. braziliensis*	82	409	490
LbrM33_V2.1410	*L. braziliensis*	45	568	612
LbrM33_V2.1420	*L. braziliensis*	88	196	283
LbrM33_V2.1430	*L. braziliensis*	108	159	266
LbrM33_V2.1430	*L. braziliensis*	118	270	387
LbrM33_V2.1430	*L. braziliensis*	77	427	503
LbrM33_V2.1430	*L. braziliensis*	58	550	607
LbrM33_V2.1440	*L. braziliensis*	213	422	634
LbrM33_V2.1450	*L. braziliensis*	180	513	692
LbrM33_V2.1450	*L. braziliensis*	77	700	776
LbrM33_V2.1460	*L. braziliensis*	217	1	217
LbrM33_V2.1480	*L. braziliensis*	138	239	376
LbrM33_V2.1480	*L. braziliensis*	361	403	763
LbrM33_V2.1490	*L. braziliensis*	55	245	299
LbrM33_V2.1500	*L. braziliensis*	46	17	62
LbrM33_V2.1510	*L. braziliensis*	113	1	113

LbrM33_V2.1510	*L. braziliensis*	67	523	589
LbrM33_V2.1520	*L. braziliensis*	42	316	357
LbrM33_V2.1530	*L. braziliensis*	164	1	164
LbrM33_V2.1560	*L. braziliensis*	54	454	507
LbrM33_V2.1570	*L. braziliensis*	122	37	158
LbrM33_V2.1590	*L. braziliensis*	122	37	158
LbrM33_V2.1640	*L. braziliensis*	44	189	232
LbrM33_V2.1640	*L. braziliensis*	76	281	356
LbrM33_V2.1640	*L. braziliensis*	76	281	356
LbrM33_V2.1650	*L. braziliensis*	64	345	408
LbrM33_V2.1660	*L. braziliensis*	114	48	161
LbrM33_V2.1670	*L. braziliensis*	72	93	164
LbrM33_V2.1670	*L. braziliensis*	89	257	345
LbrM33_V2.1670	*L. braziliensis*	259	378	636
LbrM33_V2.1670	*L. braziliensis*	172	699	870
LbrM33_V2.1670	*L. braziliensis*	100	980	1079
LbrM33_V2.1670	*L. braziliensis*	89	1140	1228
LbrM33_V2.1670	*L. braziliensis*	335	1253	1587
LbrM33_V2.1670	*L. braziliensis*	140	1601	1740
LbrM33_V2.1670	*L. braziliensis*	202	1766	1967
LbrM33_V2.1680	*L. braziliensis*	113	56	168
LbrM33_V2.1680	*L. braziliensis*	60	220	279
LbrM33_V2.1680	*L. braziliensis*	60	220	279
LbrM33_V2.1700	*L. braziliensis*	41	51	91
LbrM33_V2.1700	*L. braziliensis*	70	112	181
LbrM33_V2.1700	*L. braziliensis*	50	279	328
LbrM33_V2.1700	*L. braziliensis*	79	338	416
LbrM33_V2.1710	*L. braziliensis*	78	57	134
LbrM33_V2.1710	*L. braziliensis*	184	143	326
LbrM33_V2.1710	*L. braziliensis*	134	392	525
LbrM33_V2.1750	*L. braziliensis*	101	7	107
LbrM33_V2.1750	*L. braziliensis*	115	171	285
LbrM33_V2.1750	*L. braziliensis*	85	328	412
LbrM33_V2.1750	*L. braziliensis*	89	485	573
LbrM33_V2.1750	*L. braziliensis*	53	607	659
LbrM33_V2.1750	*L. braziliensis*	287	676	962
LbrM33_V2.1750	*L. braziliensis*	70	1216	1285
LbrM33_V2.1750	*L. braziliensis*	206	1493	1698
LbrM33_V2.1750	*L. braziliensis*	48	1753	1800
LbrM33_V2.1750	*L. braziliensis*	68	1928	1995
LbrM33_V2.1750	*L. braziliensis*	362	2146	2507
LbrM33_V2.1750	*L. braziliensis*	76	2594	2669
LbrM33_V2.1750	*L. braziliensis*	135	2769	2903
LbrM33_V2.1750	*L. braziliensis*	48	2963	3010
LbrM33_V2.1760	*L. braziliensis*	56	380	435
LbrM33_V2.1760	*L. braziliensis*	47	720	766
LbrM33_V2.1760	*L. braziliensis*	47	720	766

LbrM33_V2.1770	*L. braziliensis*	41	154	194
LbrM33_V2.1770	*L. braziliensis*	50	244	293
LbrM33_V2.1770	*L. braziliensis*	52	329	380
LbrM33_V2.1770	*L. braziliensis*	40	454	493
LbrM33_V2.1770	*L. braziliensis*	45	543	587
LbrM33_V2.1810	*L. braziliensis*	51	132	182
LbrM33_V2.1810	*L. braziliensis*	42	213	254
LbrM33_V2.1810	*L. braziliensis*	42	213	254
LbrM33_V2.1820	*L. braziliensis*	159	515	673
LbrM33_V2.1840	*L. braziliensis*	42	109	150
LbrM33_V2.1840	*L. braziliensis*	60	270	329
LbrM33_V2.1840	*L. braziliensis*	81	431	511
LbrM33_V2.1850	*L. braziliensis*	50	133	182
LbrM33_V2.1870	*L. braziliensis*	55	34	88
LbrM33_V2.1870	*L. braziliensis*	58	226	283
LbrM33_V2.1870	*L. braziliensis*	50	308	357
LbrM33_V2.1870	*L. braziliensis*	53	713	765
LbrM33_V2.1870	*L. braziliensis*	61	806	866
LbrM33_V2.1970	*L. braziliensis*	41	111	151
LbrM33_V2.1970	*L. braziliensis*	49	868	916
LbrM33_V2.1970	*L. braziliensis*	49	868	916
LbrM33_V2.1980	*L. braziliensis*	49	303	351
LbrM33_V2.2000	*L. braziliensis*	53	846	898
LbrM33_V2.2040	*L. braziliensis*	92	1	92
LbrM33_V2.2040	*L. braziliensis*	110	291	400
LbrM33_V2.2040	*L. braziliensis*	53	1099	1151
LbrM33_V2.2040	*L. braziliensis*	98	1529	1626
LbrM33_V2.2090	*L. braziliensis*	53	303	355
LbrM33_V2.2090	*L. braziliensis*	307	389	695
LbrM33_V2.2090	*L. braziliensis*	165	698	862
LbrM33_V2.2090	*L. braziliensis*	71	881	951
LbrM33_V2.2120	*L. braziliensis*	59	62	120
LbrM33_V2.2140	*L. braziliensis*	56	365	420
LbrM33_V2.2140	*L. braziliensis*	149	504	652
LbrM33_V2.2160	*L. braziliensis*	68	278	345
LbrM33_V2.2160	*L. braziliensis*	93	455	547
LbrM33_V2.2180	*L. braziliensis*	132	1	132
LbrM33_V2.2180	*L. braziliensis*	69	136	204
LbrM33_V2.2180	*L. braziliensis*	69	136	204
LbrM33_V2.2190	*L. braziliensis*	124	1	124
LbrM33_V2.2190	*L. braziliensis*	41	216	256
LbrM33_V2.2230	*L. braziliensis*	54	10	63
LbrM33_V2.2230	*L. braziliensis*	57	232	288
LbrM33_V2.2230	*L. braziliensis*	133	555	687
LbrM33_V2.2240	*L. braziliensis*	342	1	342
LbrM33_V2.2240	*L. braziliensis*	41	389	429
LbrM33_V2.2250	*L. braziliensis*	70	194	263

LbrM33_V2.2250	*L. braziliensis*	106	335	440
LbrM33_V2.2250	*L. braziliensis*	106	335	440
LbrM33_V2.2270	*L. braziliensis*	126	142	267
LbrM33_V2.2270	*L. braziliensis*	61	300	360
LbrM33_V2.2270	*L. braziliensis*	89	451	539
LbrM33_V2.2290	*L. braziliensis*	152	77	228
LbrM33_V2.2300	*L. braziliensis*	198	94	291
LbrM33_V2.2300	*L. braziliensis*	235	309	543
LbrM33_V2.2300	*L. braziliensis*	93	757	849
LbrM33_V2.2310	*L. braziliensis*	309	1	309
LbrM33_V2.2310	*L. braziliensis*	132	536	667
LbrM33_V2.2310	*L. braziliensis*	41	1044	1084
LbrM33_V2.2330	*L. braziliensis*	40	192	231
LbrM33_V2.2330	*L. braziliensis*	55	334	388
LbrM33_V2.2330	*L. braziliensis*	55	334	388
LbrM33_V2.2350	*L. braziliensis*	441	407	847
LbrM33_V2.2350	*L. braziliensis*	122	864	985
LbrM33_V2.2360	*L. braziliensis*	52	106	157
LbrM33_V2.2360	*L. braziliensis*	43	285	327
LbrM33_V2.2360	*L. braziliensis*	112	407	518
LbrM33_V2.2360	*L. braziliensis*	67	557	623
LbrM33_V2.2360	*L. braziliensis*	93	656	748
LbrM33_V2.2370	*L. braziliensis*	130	61	190
LbrM33_V2.2370	*L. braziliensis*	51	835	885
LbrM33_V2.2370	*L. braziliensis*	51	835	885
LbrM33_V2.2380	*L. braziliensis*	107	185	291
LbrM33_V2.2380	*L. braziliensis*	63	364	426
LbrM33_V2.2380	*L. braziliensis*	63	364	426
LbrM33_V2.2390	*L. braziliensis*	179	1	179
LbrM33_V2.2390	*L. braziliensis*	59	275	333
LbrM33_V2.2400	*L. braziliensis*	145	1	145
LbrM33_V2.2410	*L. braziliensis*	54	1	54
LbrM33_V2.2410	*L. braziliensis*	54	105	158
LbrM33_V2.2410	*L. braziliensis*	87	189	275
LbrM33_V2.2410	*L. braziliensis*	196	388	583
LbrM33_V2.2410	*L. braziliensis*	225	710	934
LbrM33_V2.2420	*L. braziliensis*	46	401	446
LbrM33_V2.2420	*L. braziliensis*	424	541	964
LbrM33_V2.2430	*L. braziliensis*	191	125	315
LbrM33_V2.2430	*L. braziliensis*	61	466	526
LbrM33_V2.2430	*L. braziliensis*	61	466	526
LbrM33_V2.2450	*L. braziliensis*	136	168	303
LbrM33_V2.2450	*L. braziliensis*	107	493	599
LbrM33_V2.2450	*L. braziliensis*	181	649	829
LbrM33_V2.2450	*L. braziliensis*	268	1201	1468
LbrM33_V2.2460	*L. braziliensis*	278	73	350
LbrM33_V2.2460	*L. braziliensis*	98	374	471

LbrM33_V2.2460	*L. braziliensis*	55	480	534
LbrM33_V2.2460	*L. braziliensis*	85	552	636
LbrM33_V2.2460	*L. braziliensis*	82	852	933
LbrM33_V2.2460	*L. braziliensis*	51	973	1023
LbrM33_V2.2460	*L. braziliensis*	65	1050	1114
LbrM33_V2.2460	*L. braziliensis*	107	1192	1298
LbrM33_V2.2460	*L. braziliensis*	45	1334	1378
LbrM33_V2.2470	*L. braziliensis*	187	13	199
LbrM33_V2.2470	*L. braziliensis*	240	251	490
LbrM33_V2.2470	*L. braziliensis*	73	528	600
LbrM33_V2.2470	*L. braziliensis*	104	662	765
LbrM33_V2.2470	*L. braziliensis*	56	793	848
LbrM33_V2.2470	*L. braziliensis*	191	885	1075
LbrM33_V2.2480	*L. braziliensis*	302	1	302
LbrM33_V2.2480	*L. braziliensis*	72	326	397
LbrM33_V2.2500	*L. braziliensis*	70	1	70
LbrM33_V2.2500	*L. braziliensis*	83	72	154
LbrM33_V2.2500	*L. braziliensis*	83	72	154
LbrM33_V2.2510	*L. braziliensis*	182	1	182
LbrM33_V2.2510	*L. braziliensis*	205	243	447
LbrM33_V2.2510	*L. braziliensis*	144	452	595
LbrM33_V2.2510	*L. braziliensis*	50	600	649
LbrM33_V2.2520	*L. braziliensis*	52	1	52
LbrM33_V2.2520	*L. braziliensis*	79	74	152
LbrM33_V2.2540	*L. braziliensis*	85	114	198
LbrM33_V2.2540	*L. braziliensis*	172	268	439
LbrM33_V2.2550	*L. braziliensis*	77	155	231
LbrM33_V2.2550	*L. braziliensis*	69	334	402
LbrM33_V2.2550	*L. braziliensis*	42	617	658
LbrM33_V2.2560	*L. braziliensis*	63	1	63
LbrM33_V2.2560	*L. braziliensis*	689	78	766
LbrM33_V2.2560	*L. braziliensis*	74	808	881
LbrM33_V2.2570	*L. braziliensis*	41	603	643
LbrM33_V2.2570	*L. braziliensis*	60	932	991
LbrM33_V2.2570	*L. braziliensis*	60	932	991
LbrM33_V2.2580	*L. braziliensis*	217	1	217
LbrM33_V2.2580	*L. braziliensis*	62	286	347
LbrM33_V2.2580	*L. braziliensis*	88	861	948
LbrM33_V2.2580	*L. braziliensis*	43	1241	1283
LbrM33_V2.2580	*L. braziliensis*	91	1289	1379
LbrM33_V2.2580	*L. braziliensis*	137	1703	1839
LbrM33_V2.2580	*L. braziliensis*	51	1871	1921
LbrM33_V2.2600	*L. braziliensis*	47	75	121
LbrM33_V2.2600	*L. braziliensis*	77	130	206
LbrM33_V2.2600	*L. braziliensis*	146	213	358
LbrM33_V2.2610	*L. braziliensis*	124	146	269
LbrM33_V2.2610	*L. braziliensis*	73	616	688

LbrM33_V2.2620	*L. braziliensis*	61	1	61
LbrM33_V2.2620	*L. braziliensis*	108	63	170
LbrM33_V2.2620	*L. braziliensis*	180	176	355
LbrM33_V2.2620	*L. braziliensis*	94	417	510
LbrM33_V2.2620	*L. braziliensis*	81	536	616
LbrM33_V2.2620	*L. braziliensis*	41	745	785
LbrM33_V2.2620	*L. braziliensis*	160	842	1001
LbrM33_V2.2620	*L. braziliensis*	81	1083	1163
LbrM33_V2.2640	*L. braziliensis*	108	1	108
LbrM33_V2.2640	*L. braziliensis*	119	611	729
LbrM33_V2.2640	*L. braziliensis*	81	749	829
LbrM33_V2.2650	*L. braziliensis*	45	1	45
LbrM33_V2.2660	*L. braziliensis*	264	279	542
LbrM33_V2.2680	*L. braziliensis*	184	1	184
LbrM33_V2.2690	*L. braziliensis*	49	136	184
LbrM33_V2.2700	*L. braziliensis*	57	525	581
LbrM33_V2.2710	*L. braziliensis*	97	1	97
LbrM33_V2.2710	*L. braziliensis*	133	190	322
LbrM33_V2.2710	*L. braziliensis*	167	345	511
LbrM33_V2.2720	*L. braziliensis*	66	341	406
LbrM33_V2.2720	*L. braziliensis*	188	543	730
LbrM33_V2.2720	*L. braziliensis*	81	760	840
LbrM33_V2.2720	*L. braziliensis*	52	887	938
LbrM33_V2.2720	*L. braziliensis*	101	1001	1101
LbrM33_V2.2720	*L. braziliensis*	508	1138	1645
LbrM33_V2.2730	*L. braziliensis*	74	262	335
LbrM33_V2.2730	*L. braziliensis*	96	431	526
LbrM33_V2.2730	*L. braziliensis*	59	986	1044
LbrM33_V2.2760	*L. braziliensis*	65	40	104
LbrM33_V2.2760	*L. braziliensis*	74	128	201
LbrM33_V2.2760	*L. braziliensis*	59	307	365
LbrM33_V2.2760	*L. braziliensis*	46	397	442
LbrM33_V2.2760	*L. braziliensis*	46	446	491
LbrM33_V2.2780	*L. braziliensis*	55	49	103
LbrM33_V2.2780	*L. braziliensis*	67	250	316
LbrM33_V2.2780	*L. braziliensis*	58	358	415
LbrM33_V2.2800	*L. braziliensis*	71	1	71
LbrM33_V2.2800	*L. braziliensis*	43	565	607
LbrM33_V2.2800	*L. braziliensis*	61	1004	1064
LbrM33_V2.2800	*L. braziliensis*	158	1094	1251
LbrM33_V2.2800	*L. braziliensis*	42	1258	1299
LbrM33_V2.2830	*L. braziliensis*	43	1	43
LbrM33_V2.2830	*L. braziliensis*	48	210	257
LbrM33_V2.2830	*L. braziliensis*	81	357	437
LbrM33_V2.2830	*L. braziliensis*	176	500	675
LbrM33_V2.2830	*L. braziliensis*	58	717	774
LbrM33_V2.2830	*L. braziliensis*	260	870	1129

LbrM33_V2.2830	*L. braziliensis*	80	1156	1235
LbrM33_V2.2830	*L. braziliensis*	43	1652	1694
LbrM33_V2.2830	*L. braziliensis*	70	1923	1992
LbrM33_V2.2860	*L. braziliensis*	74	1	74
LbrM33_V2.2860	*L. braziliensis*	40	101	140
LbrM33_V2.2860	*L. braziliensis*	43	255	297
LbrM33_V2.2860	*L. braziliensis*	60	457	516
LbrM33_V2.2870	*L. braziliensis*	88	251	338
LbrM33_V2.2870	*L. braziliensis*	98	369	466
LbrM33_V2.2870	*L. braziliensis*	129	520	648
LbrM33_V2.2870	*L. braziliensis*	69	746	814
LbrM33_V2.2870	*L. braziliensis*	141	910	1050
LbrM33_V2.2880	*L. braziliensis*	46	751	796
LbrM33_V2.2900	*L. braziliensis*	74	378	451
LbrM33_V2.2910	*L. braziliensis*	117	172	288
LbrM33_V2.2910	*L. braziliensis*	161	294	454
LbrM33_V2.2910	*L. braziliensis*	143	525	667
LbrM33_V2.2910	*L. braziliensis*	110	721	830
LbrM33_V2.2910	*L. braziliensis*	55	1037	1091
LbrM33_V2.2920	*L. braziliensis*	118	1	118
LbrM33_V2.2930	*L. braziliensis*	46	178	223
LbrM33_V2.2930	*L. braziliensis*	44	374	417
LbrM33_V2.2930	*L. braziliensis*	61	667	727
LbrM33_V2.2940	*L. braziliensis*	58	44	101
LbrM33_V2.2940	*L. braziliensis*	43	299	341
LbrM33_V2.2940	*L. braziliensis*	60	346	405
LbrM33_V2.2940	*L. braziliensis*	126	603	728
LbrM33_V2.2940	*L. braziliensis*	58	751	808
LbrM33_V2.2940	*L. braziliensis*	51	857	907
LbrM33_V2.2940	*L. braziliensis*	92	1059	1150
LbrM33_V2.2950	*L. braziliensis*	65	17	81
LbrM33_V2.2950	*L. braziliensis*	178	106	283
LbrM33_V2.2950	*L. braziliensis*	369	347	715
LbrM33_V2.2950	*L. braziliensis*	40	737	776
LbrM33_V2.2950	*L. braziliensis*	183	838	1020
LbrM33_V2.2950	*L. braziliensis*	120	1074	1193
LbrM33_V2.2960	*L. braziliensis*	123	18	140
LbrM33_V2.2960	*L. braziliensis*	167	173	339
LbrM33_V2.2960	*L. braziliensis*	245	347	591
LbrM33_V2.2960	*L. braziliensis*	153	689	841
LbrM33_V2.2970	*L. braziliensis*	41	1	41
LbrM33_V2.2970	*L. braziliensis*	81	135	215
LbrM33_V2.2970	*L. braziliensis*	81	135	215
LbrM33_V2.2980	*L. braziliensis*	65	542	606
LbrM33_V2.2980	*L. braziliensis*	41	691	731
LbrM33_V2.2980	*L. braziliensis*	43	746	788
LbrM33_V2.2990	*L. braziliensis*	48	70	117

LbrM33_V2.3020	*L. braziliensis*	224	1	224
LbrM33_V2.3030	*L. braziliensis*	51	46	96
LbrM33_V2.3030	*L. braziliensis*	47	195	241
LbrM33_V2.3030	*L. braziliensis*	42	301	342
LbrM33_V2.3030	*L. braziliensis*	89	379	467
LbrM33_V2.3030	*L. braziliensis*	49	582	630
LbrM33_V2.3030	*L. braziliensis*	52	756	807
LbrM33_V2.3030	*L. braziliensis*	61	917	977
LbrM33_V2.3030	*L. braziliensis*	50	1014	1063
LbrM33_V2.3030	*L. braziliensis*	40	1068	1107
LbrM33_V2.3040	*L. braziliensis*	124	43	166
LbrM33_V2.3040	*L. braziliensis*	181	254	434
LbrM33_V2.3040	*L. braziliensis*	86	521	606
LbrM33_V2.3060	*L. braziliensis*	101	300	400
LbrM33_V2.3060	*L. braziliensis*	88	504	591
LbrM33_V2.3060	*L. braziliensis*	189	704	892
LbrM33_V2.3060	*L. braziliensis*	51	1001	1051
LbrM33_V2.3060	*L. braziliensis*	139	1145	1283
LbrM33_V2.3060	*L. braziliensis*	78	1795	1872
LbrM33_V2.3060	*L. braziliensis*	148	2365	2512
LbrM33_V2.3060	*L. braziliensis*	77	2567	2643
LbrM33_V2.3060	*L. braziliensis*	194	2746	2939
LbrM33_V2.3060	*L. braziliensis*	160	2965	3124
LbrM33_V2.3060	*L. braziliensis*	174	3220	3393
LbrM33_V2.3060	*L. braziliensis*	72	3744	3815
LbrM33_V2.3060	*L. braziliensis*	63	3923	3985
LbrM33_V2.3060	*L. braziliensis*	54	4381	4434
LbrM33_V2.3060	*L. braziliensis*	56	4844	4899
LbrM33_V2.3060	*L. braziliensis*	40	4941	4980
LbrM33_V2.3070	*L. braziliensis*	72	187	258
LbrM33_V2.3070	*L. braziliensis*	83	270	352
LbrM33_V2.3080	*L. braziliensis*	40	23	62
LbrM33_V2.3090	*L. braziliensis*	101	73	173
LbrM33_V2.3090	*L. braziliensis*	68	248	315
LbrM33_V2.3090	*L. braziliensis*	68	248	315
LbrM33_V2.3100	*L. braziliensis*	221	77	297
LbrM33_V2.3100	*L. braziliensis*	56	493	548
LbrM33_V2.3120	*L. braziliensis*	160	38	197
LbrM33_V2.3120	*L. braziliensis*	162	233	394
LbrM33_V2.3140	*L. braziliensis*	58	1	58
LbrM33_V2.3150	*L. braziliensis*	81	1	81
LbrM33_V2.3170	*L. braziliensis*	50	173	222
LbrM33_V2.3170	*L. braziliensis*	71	258	328
LbrM33_V2.3170	*L. braziliensis*	59	350	408
LbrM33_V2.3180	*L. braziliensis*	144	109	252
LbrM33_V2.3190	*L. braziliensis*	71	81	151
LbrM33_V2.3190	*L. braziliensis*	101	185	285

LbrM33_V2.3190	*L. braziliensis*	76	319	394
LbrM33_V2.3190	*L. braziliensis*	108	426	533
LbrM33_V2.3190	*L. braziliensis*	126	543	668
LbrM33_V2.3190	*L. braziliensis*	454	723	1176
LbrM33_V2.3190	*L. braziliensis*	143	1527	1669
LbrM33_V2.3190	*L. braziliensis*	289	1685	1973
LbrM33_V2.3190	*L. braziliensis*	87	2285	2371
LbrM33_V2.3200	*L. braziliensis*	106	94	199
LbrM33_V2.3200	*L. braziliensis*	50	306	355
LbrM33_V2.3200	*L. braziliensis*	53	467	519
LbrM33_V2.3200	*L. braziliensis*	45	542	586
LbrM33_V2.3200	*L. braziliensis*	140	846	985
LbrM33_V2.3200	*L. braziliensis*	131	1264	1394
LbrM33_V2.3200	*L. braziliensis*	52	1657	1708
LbrM33_V2.3210	*L. braziliensis*	314	1	314
LbrM33_V2.3220	*L. braziliensis*	308	14	321
LbrM33_V2.3220	*L. braziliensis*	154	549	702
LbrM33_V2.3220	*L. braziliensis*	82	988	1069
LbrM33_V2.3230	*L. braziliensis*	289	1	289
LbrM33_V2.3230	*L. braziliensis*	87	544	630
LbrM33_V2.3240	*L. braziliensis*	329	1	329
LbrM33_V2.3240	*L. braziliensis*	74	578	651
LbrM33_V2.3250	*L. braziliensis*	78	30	107
LbrM33_V2.3250	*L. braziliensis*	44	125	168
LbrM33_V2.3250	*L. braziliensis*	70	204	273
LbrM33_V2.3250	*L. braziliensis*	59	300	358
LbrM33_V2.3250	*L. braziliensis*	167	384	550
LbrM33_V2.3250	*L. braziliensis*	197	568	764
LbrM33_V2.3250	*L. braziliensis*	88	822	909
LbrM33_V2.3250	*L. braziliensis*	67	994	1060
LbrM33_V2.3250	*L. braziliensis*	40	1097	1136
LbrM33_V2.3260	*L. braziliensis*	131	1	131
LbrM33_V2.3260	*L. braziliensis*	244	150	393
LbrM33_V2.3260	*L. braziliensis*	55	714	768
LbrM33_V2.3260	*L. braziliensis*	40	785	824
LbrM33_V2.3260	*L. braziliensis*	181	1058	1238
LbrM33_V2.3270	*L. braziliensis*	140	1	140
LbrM33_V2.3270	*L. braziliensis*	109	222	330
LbrM33_V2.3280	*L. braziliensis*	482	1	482
LbrM33_V2.3280	*L. braziliensis*	50	754	803
LbrM33_V2.3280	*L. braziliensis*	62	903	964
LbrM33_V2.3290	*L. braziliensis*	90	1	90
LbrM33_V2.3290	*L. braziliensis*	625	128	752
LbrM33_V2.3300	*L. braziliensis*	127	49	175
LbrM33_V2.3300	*L. braziliensis*	54	208	261
LbrM33_V2.3300	*L. braziliensis*	198	276	473
LbrM33_V2.3300	*L. braziliensis*	113	499	611

LbrM33_V2.3300	*L. braziliensis*	202	627	828
LbrM33_V2.3300	*L. braziliensis*	84	834	917
LbrM33_V2.3310	*L. braziliensis*	75	1	75
LbrM33_V2.3310	*L. braziliensis*	253	125	377
LbrM33_V2.3310	*L. braziliensis*	72	427	498
LbrM33_V2.3310	*L. braziliensis*	175	549	723
LbrM33_V2.3310	*L. braziliensis*	234	754	987
LbrM33_V2.3320	*L. braziliensis*	47	660	706
LbrM33_V2.3330	*L. braziliensis*	44	421	464
LbrM33_V2.3330	*L. braziliensis*	48	633	680
LbrM33_V2.3330	*L. braziliensis*	48	633	680
LbrM33_V2.3350	*L. braziliensis*	72	114	185
LbrM33_V2.3350	*L. braziliensis*	72	272	343
LbrM33_V2.3350	*L. braziliensis*	68	430	497
LbrM33_V2.3350	*L. braziliensis*	67	589	655
LbrM33_V2.3350	*L. braziliensis*	64	747	810
LbrM33_V2.3350	*L. braziliensis*	63	904	966
LbrM33_V2.3350	*L. braziliensis*	45	1078	1122
LbrM33_V2.3350	*L. braziliensis*	63	1216	1278
LbrM33_V2.3350	*L. braziliensis*	63	1372	1434
LbrM33_V2.3350	*L. braziliensis*	63	1528	1590
LbrM33_V2.3350	*L. braziliensis*	69	1681	1749
LbrM33_V2.3350	*L. braziliensis*	63	1842	1904
LbrM33_V2.3350	*L. braziliensis*	73	1995	2067
LbrM33_V2.3350	*L. braziliensis*	69	2153	2221
LbrM33_V2.3350	*L. braziliensis*	64	2313	2376
LbrM33_V2.3350	*L. braziliensis*	67	2469	2535
LbrM33_V2.3350	*L. braziliensis*	72	2626	2697
LbrM33_V2.3350	*L. braziliensis*	72	2783	2854
LbrM33_V2.3350	*L. braziliensis*	72	2941	3012
LbrM33_V2.3350	*L. braziliensis*	68	3100	3167
LbrM33_V2.3350	*L. braziliensis*	68	3258	3325
LbrM33_V2.3350	*L. braziliensis*	64	3417	3480
LbrM33_V2.3350	*L. braziliensis*	73	3571	3643
LbrM33_V2.3350	*L. braziliensis*	72	3729	3800
LbrM33_V2.3350	*L. braziliensis*	64	3889	3952
LbrM33_V2.3350	*L. braziliensis*	64	4045	4108
LbrM33_V2.3350	*L. braziliensis*	69	4199	4267
LbrM33_V2.3360	*L. braziliensis*	69	113	181
LbrM33_V2.3360	*L. braziliensis*	72	272	343
LbrM33_V2.3360	*L. braziliensis*	64	431	494
LbrM33_V2.3360	*L. braziliensis*	45	606	650
LbrM33_V2.3360	*L. braziliensis*	73	741	813
LbrM33_V2.3360	*L. braziliensis*	73	899	971
LbrM33_V2.3360	*L. braziliensis*	73	1057	1129
LbrM33_V2.3360	*L. braziliensis*	73	1215	1287
LbrM33_V2.3360	*L. braziliensis*	73	1373	1445

LbrM33_V2.3360	*L. braziliensis*	58	1534	1591
LbrM33_V2.3360	*L. braziliensis*	72	1746	1817
LbrM33_V2.3360	*L. braziliensis*	71	2086	2156
LbrM33_V2.3360	*L. braziliensis*	169	2159	2327
LbrM33_V2.3370	*L. braziliensis*	48	85	132
LbrM33_V2.3370	*L. braziliensis*	60	261	320
LbrM33_V2.3370	*L. braziliensis*	64	436	499
LbrM33_V2.3370	*L. braziliensis*	56	613	668
LbrM33_V2.3370	*L. braziliensis*	322	708	1029
LbrM33_V2.3370	*L. braziliensis*	72	1088	1159
LbrM33_V2.3370	*L. braziliensis*	45	1184	1228
LbrM33_V2.3370	*L. braziliensis*	50	1240	1289
LbrM33_V2.3370	*L. braziliensis*	42	1378	1419
LbrM33_V2.3370	*L. braziliensis*	233	1661	1893
LbrM33_V2.3370	*L. braziliensis*	162	1905	2066
LbrM33_V2.3370	*L. braziliensis*	168	2087	2254
LbrM33_V2.3380	*L. braziliensis*	131	64	194
LbrM33_V2.3380	*L. braziliensis*	44	1198	1241
LbrM33_V2.3380	*L. braziliensis*	44	1198	1241
LbrM33_V2.3390	*L. braziliensis*	113	234	346
LbrM33_V2.3420	*L. braziliensis*	54	1	54
LbrM33_V2.3430	*L. braziliensis*	71	1	71
LbrM33_V2.3430	*L. braziliensis*	63	273	335
LbrM33_V2.3430	*L. braziliensis*	63	273	335
LbrM33_V2.3440	*L. braziliensis*	57	121	177
LbrM33_V2.3440	*L. braziliensis*	117	238	354
LbrM33_V2.3440	*L. braziliensis*	119	374	492
LbrM33_V2.3450	*L. braziliensis*	46	346	391
LbrM33_V2.3450	*L. braziliensis*	189	417	605
LbrM33_V2.3480	*L. braziliensis*	111	794	904
LbrM33_V2.3490	*L. braziliensis*	69	137	205
LbrM33_V2.3490	*L. braziliensis*	63	237	299
LbrM33_V2.3490	*L. braziliensis*	109	310	418
LbrM33_V2.3510	*L. braziliensis*	50	29	78
LbrM33_V2.3520	*L. braziliensis*	98	40	137
LbrM33_V2.3530	*L. braziliensis*	127	1	127
LbrM33_V2.3530	*L. braziliensis*	195	135	329
LbrM33_V2.3540	*L. braziliensis*	58	417	474
LbrM34_V2.0030	*L. braziliensis*	42	1	42
LbrM34_V2.0050	*L. braziliensis*	42	1	42
LbrM34_V2.0100	*L. braziliensis*	472	177	648
LbrM34_V2.0110	*L. braziliensis*	41	194	234
LbrM34_V2.0120	*L. braziliensis*	43	1	43
LbrM34_V2.0130	*L. braziliensis*	127	1	127
LbrM34_V2.0210	*L. braziliensis*	56	76	131
LbrM34_V2.0210	*L. braziliensis*	68	206	273
LbrM34_V2.0210	*L. braziliensis*	68	206	273

LbrM34_V2.0220	*L. braziliensis*	139	1	139
LbrM34_V2.0220	*L. braziliensis*	251	180	430
LbrM34_V2.0220	*L. braziliensis*	58	462	519
LbrM34_V2.0220	*L. braziliensis*	53	537	589
LbrM34_V2.0220	*L. braziliensis*	145	621	765
LbrM34_V2.0220	*L. braziliensis*	82	781	862
LbrM34_V2.0240	*L. braziliensis*	362	69	430
LbrM34_V2.0240	*L. braziliensis*	51	455	505
LbrM34_V2.0240	*L. braziliensis*	61	598	658
LbrM34_V2.0240	*L. braziliensis*	146	718	863
LbrM34_V2.0240	*L. braziliensis*	97	909	1005
LbrM34_V2.0240	*L. braziliensis*	95	1016	1110
LbrM34_V2.0240	*L. braziliensis*	57	1235	1291
LbrM34_V2.0240	*L. braziliensis*	171	1328	1498
LbrM34_V2.0240	*L. braziliensis*	220	1510	1729
LbrM34_V2.0240	*L. braziliensis*	332	1761	2092
LbrM34_V2.0240	*L. braziliensis*	184	2262	2445
LbrM34_V2.0260	*L. braziliensis*	106	158	263
LbrM34_V2.0270	*L. braziliensis*	45	142	186
LbrM34_V2.0270	*L. braziliensis*	54	294	347
LbrM34_V2.0270	*L. braziliensis*	54	294	347
LbrM34_V2.0290	*L. braziliensis*	69	108	176
LbrM34_V2.0290	*L. braziliensis*	116	326	441
LbrM34_V2.0290	*L. braziliensis*	44	554	597
LbrM34_V2.0290	*L. braziliensis*	48	671	718
LbrM34_V2.0300	*L. braziliensis*	139	171	309
LbrM34_V2.0300	*L. braziliensis*	86	406	491
LbrM34_V2.0330	*L. braziliensis*	711	36	746
LbrM34_V2.0330	*L. braziliensis*	51	774	824
LbrM34_V2.0330	*L. braziliensis*	48	1420	1467
LbrM34_V2.0340	*L. braziliensis*	100	15	114
LbrM34_V2.0340	*L. braziliensis*	76	149	224
LbrM34_V2.0340	*L. braziliensis*	43	258	300
LbrM34_V2.0350	*L. braziliensis*	67	302	368
LbrM34_V2.0360	*L. braziliensis*	62	1	62
LbrM34_V2.0380	*L. braziliensis*	54	148	201
LbrM34_V2.0380	*L. braziliensis*	41	329	369
LbrM34_V2.0380	*L. braziliensis*	41	329	369
LbrM34_V2.0390	*L. braziliensis*	74	71	144
LbrM34_V2.0390	*L. braziliensis*	54	146	199
LbrM34_V2.0390	*L. braziliensis*	86	439	524
LbrM34_V2.0400	*L. braziliensis*	49	248	296
LbrM34_V2.0410	*L. braziliensis*	154	10	163
LbrM34_V2.0410	*L. braziliensis*	116	229	344
LbrM34_V2.0410	*L. braziliensis*	59	555	613
LbrM34_V2.0420	*L. braziliensis*	53	29	81
LbrM34_V2.0420	*L. braziliensis*	66	158	223

LbrM34_V2.0420	*L. braziliensis*	41	328	368
LbrM34_V2.0420	*L. braziliensis*	47	464	510
LbrM34_V2.0450	*L. braziliensis*	129	12	140
LbrM34_V2.0450	*L. braziliensis*	56	177	232
LbrM34_V2.0450	*L. braziliensis*	200	253	452
LbrM34_V2.0460	*L. braziliensis*	58	253	310
LbrM34_V2.0460	*L. braziliensis*	45	348	392
LbrM34_V2.0480	*L. braziliensis*	211	695	905
LbrM34_V2.0490	*L. braziliensis*	68	28	95
LbrM34_V2.0490	*L. braziliensis*	62	140	201
LbrM34_V2.0490	*L. braziliensis*	41	219	259
LbrM34_V2.0510	*L. braziliensis*	397	277	673
LbrM34_V2.0510	*L. braziliensis*	136	784	919
LbrM34_V2.0510	*L. braziliensis*	96	1084	1179
LbrM34_V2.0510	*L. braziliensis*	372	1267	1638
LbrM34_V2.0520	*L. braziliensis*	47	341	387
LbrM34_V2.0520	*L. braziliensis*	219	652	870
LbrM34_V2.0530	*L. braziliensis*	58	294	351
LbrM34_V2.0530	*L. braziliensis*	3709	616	4324
LbrM34_V2.0540	*L. braziliensis*	2500	176	2675
LbrM34_V2.0540	*L. braziliensis*	41	3805	3845
LbrM34_V2.0540	*L. braziliensis*	620	4558	5177
LbrM34_V2.0550	*L. braziliensis*	55	299	353
LbrM34_V2.0550	*L. braziliensis*	124	617	740
LbrM34_V2.0560	*L. braziliensis*	82	1	82
LbrM34_V2.0560	*L. braziliensis*	284	90	373
LbrM34_V2.0560	*L. braziliensis*	272	404	675
LbrM34_V2.0560	*L. braziliensis*	46	701	746
LbrM34_V2.0570	*L. braziliensis*	49	156	204
LbrM34_V2.0570	*L. braziliensis*	43	425	467
LbrM34_V2.0570	*L. braziliensis*	64	516	579
LbrM34_V2.0570	*L. braziliensis*	64	924	987
LbrM34_V2.0570	*L. braziliensis*	301	1341	1641
LbrM34_V2.0570	*L. braziliensis*	94	1702	1795
LbrM34_V2.0570	*L. braziliensis*	88	1866	1953
LbrM34_V2.0570	*L. braziliensis*	119	1999	2117
LbrM34_V2.0580	*L. braziliensis*	223	1	223
LbrM34_V2.0590	*L. braziliensis*	73	266	338
LbrM34_V2.0590	*L. braziliensis*	50	357	406
LbrM34_V2.0590	*L. braziliensis*	48	605	652
LbrM34_V2.0610	*L. braziliensis*	67	1	67
LbrM34_V2.0610	*L. braziliensis*	106	111	216
LbrM34_V2.0610	*L. braziliensis*	87	412	498
LbrM34_V2.0610	*L. braziliensis*	54	865	918
LbrM34_V2.0610	*L. braziliensis*	55	974	1028
LbrM34_V2.0610	*L. braziliensis*	108	1062	1169
LbrM34_V2.0610	*L. braziliensis*	107	1692	1798

LbrM34_V2.0610	*L. braziliensis*	46	1912	1957
LbrM34_V2.0610	*L. braziliensis*	60	2041	2100
LbrM34_V2.0610	*L. braziliensis*	46	2293	2338
LbrM34_V2.0610	*L. braziliensis*	84	2409	2492
LbrM34_V2.0630	*L. braziliensis*	75	1	75
LbrM34_V2.0630	*L. braziliensis*	159	148	306
LbrM34_V2.0630	*L. braziliensis*	47	406	452
LbrM34_V2.0640	*L. braziliensis*	45	431	475
LbrM34_V2.0650	*L. braziliensis*	44	1	44
LbrM34_V2.0650	*L. braziliensis*	44	49	92
LbrM34_V2.0650	*L. braziliensis*	67	177	243
LbrM34_V2.0650	*L. braziliensis*	273	270	542
LbrM34_V2.0650	*L. braziliensis*	75	626	700
LbrM34_V2.0650	*L. braziliensis*	58	856	913
LbrM34_V2.0650	*L. braziliensis*	45	945	989
LbrM34_V2.0650	*L. braziliensis*	56	1182	1237
LbrM34_V2.0660	*L. braziliensis*	53	269	321
LbrM34_V2.0660	*L. braziliensis*	46	495	540
LbrM34_V2.0660	*L. braziliensis*	65	720	784
LbrM34_V2.0660	*L. braziliensis*	46	852	897
LbrM34_V2.0660	*L. braziliensis*	46	945	990
LbrM34_V2.0660	*L. braziliensis*	45	1318	1362
LbrM34_V2.0660	*L. braziliensis*	103	2130	2232
LbrM34_V2.0660	*L. braziliensis*	53	2647	2699
LbrM34_V2.0660	*L. braziliensis*	94	2854	2947
LbrM34_V2.0660	*L. braziliensis*	40	3100	3139
LbrM34_V2.0660	*L. braziliensis*	110	3241	3350
LbrM34_V2.0660	*L. braziliensis*	43	4060	4102
LbrM34_V2.0660	*L. braziliensis*	68	4114	4181
LbrM34_V2.0670	*L. braziliensis*	203	1	203
LbrM34_V2.0670	*L. braziliensis*	223	237	459
LbrM34_V2.0670	*L. braziliensis*	143	519	661
LbrM34_V2.0670	*L. braziliensis*	182	692	873
LbrM34_V2.0670	*L. braziliensis*	73	969	1041
LbrM34_V2.0680	*L. braziliensis*	41	582	622
LbrM34_V2.0690	*L. braziliensis*	45	200	244
LbrM34_V2.0700	*L. braziliensis*	40	117	156
LbrM34_V2.0710	*L. braziliensis*	46	396	441
LbrM34_V2.0720	*L. braziliensis*	252	1	252
LbrM34_V2.0720	*L. braziliensis*	42	260	301
LbrM34_V2.0720	*L. braziliensis*	50	477	526
LbrM34_V2.0720	*L. braziliensis*	74	647	720
LbrM34_V2.0730	*L. braziliensis*	66	368	433
LbrM34_V2.0730	*L. braziliensis*	176	470	645
LbrM34_V2.0730	*L. braziliensis*	193	699	891
LbrM34_V2.0750	*L. braziliensis*	72	229	300
LbrM34_V2.0760	*L. braziliensis*	95	151	245

LbrM34_V2.0780	*L. braziliensis*	58	37	94
LbrM34_V2.0780	*L. braziliensis*	67	303	369
LbrM34_V2.0780	*L. braziliensis*	67	303	369
LbrM34_V2.0790	*L. braziliensis*	165	76	240
LbrM34_V2.0790	*L. braziliensis*	75	270	344
LbrM34_V2.0790	*L. braziliensis*	49	424	472
LbrM34_V2.0790	*L. braziliensis*	54	515	568
LbrM34_V2.0790	*L. braziliensis*	128	610	737
LbrM34_V2.0790	*L. braziliensis*	210	764	973
LbrM34_V2.0800	*L. braziliensis*	126	1	126
LbrM34_V2.0800	*L. braziliensis*	178	138	315
LbrM34_V2.0800	*L. braziliensis*	456	318	773
LbrM34_V2.0800	*L. braziliensis*	115	807	921
LbrM34_V2.0800	*L. braziliensis*	248	924	1171
LbrM34_V2.0800	*L. braziliensis*	70	1189	1258
LbrM34_V2.0800	*L. braziliensis*	69	1284	1352
LbrM34_V2.0820	*L. braziliensis*	46	261	306
LbrM34_V2.0830	*L. braziliensis*	111	50	160
LbrM34_V2.0850	*L. braziliensis*	97	65	161
LbrM34_V2.0870	*L. braziliensis*	69	1	69
LbrM34_V2.0870	*L. braziliensis*	42	305	346
LbrM34_V2.0870	*L. braziliensis*	49	419	467
LbrM34_V2.0880	*L. braziliensis*	109	1	109
LbrM34_V2.0890	*L. braziliensis*	236	1	236
LbrM34_V2.0900	*L. braziliensis*	321	1	321
LbrM34_V2.0900	*L. braziliensis*	426	452	877
LbrM34_V2.0900	*L. braziliensis*	94	884	977
LbrM34_V2.0900	*L. braziliensis*	369	982	1350
LbrM34_V2.0900	*L. braziliensis*	67	1359	1425
LbrM34_V2.0900	*L. braziliensis*	168	1435	1602
LbrM34_V2.0900	*L. braziliensis*	45	1659	1703
LbrM34_V2.0900	*L. braziliensis*	51	1725	1775
LbrM34_V2.0900	*L. braziliensis*	50	1868	1917
LbrM34_V2.0910	*L. braziliensis*	198	109	306
LbrM34_V2.0920	*L. braziliensis*	55	1	55
LbrM34_V2.0920	*L. braziliensis*	47	83	129
LbrM34_V2.0920	*L. braziliensis*	67	144	210
LbrM34_V2.0930	*L. braziliensis*	100	23	122
LbrM34_V2.0930	*L. braziliensis*	51	143	193
LbrM34_V2.0930	*L. braziliensis*	51	143	193
LbrM34_V2.0940	*L. braziliensis*	41	1	41
LbrM34_V2.0940	*L. braziliensis*	137	75	211
LbrM34_V2.0950	*L. braziliensis*	111	104	214
LbrM34_V2.0950	*L. braziliensis*	79	235	313
LbrM34_V2.0950	*L. braziliensis*	54	438	491
LbrM34_V2.0950	*L. braziliensis*	45	503	547
LbrM34_V2.0950	*L. braziliensis*	58	588	645

LbrM34_V2.0950	*L. braziliensis*	42	708	749
LbrM34_V2.0980	*L. braziliensis*	88	37	124
LbrM34_V2.0980	*L. braziliensis*	65	261	325
LbrM34_V2.0980	*L. braziliensis*	92	452	543
LbrM34_V2.0980	*L. braziliensis*	272	579	850
LbrM34_V2.1000	*L. braziliensis*	52	303	354
LbrM34_V2.1010	*L. braziliensis*	133	1	133
LbrM34_V2.1010	*L. braziliensis*	136	203	338
LbrM34_V2.1020	*L. braziliensis*	49	1	49
LbrM34_V2.1030	*L. braziliensis*	103	70	172
LbrM34_V2.1030	*L. braziliensis*	92	213	304
LbrM34_V2.1040	*L. braziliensis*	64	78	141
LbrM34_V2.1080	*L. braziliensis*	111	70	180
LbrM34_V2.1080	*L. braziliensis*	100	502	601
LbrM34_V2.1110	*L. braziliensis*	85	1	85
LbrM34_V2.1120	*L. braziliensis*	44	436	479
LbrM34_V2.1160	*L. braziliensis*	137	1	137
LbrM34_V2.1160	*L. braziliensis*	195	196	390
LbrM34_V2.1160	*L. braziliensis*	123	423	545
LbrM34_V2.1160	*L. braziliensis*	93	603	695
LbrM34_V2.1160	*L. braziliensis*	48	701	748
LbrM34_V2.1160	*L. braziliensis*	61	786	846
LbrM34_V2.1190	*L. braziliensis*	56	42	97
LbrM34_V2.1190	*L. braziliensis*	95	201	295
LbrM34_V2.1210	*L. braziliensis*	100	53	152
LbrM34_V2.1210	*L. braziliensis*	58	191	248
LbrM34_V2.1210	*L. braziliensis*	58	191	248
LbrM34_V2.1230	*L. braziliensis*	43	1	43
LbrM34_V2.1240	*L. braziliensis*	134	72	205
LbrM34_V2.1240	*L. braziliensis*	116	226	341
LbrM34_V2.1240	*L. braziliensis*	127	473	599
LbrM34_V2.1240	*L. braziliensis*	124	862	985
LbrM34_V2.1240	*L. braziliensis*	212	993	1204
LbrM34_V2.1240	*L. braziliensis*	43	1237	1279
LbrM34_V2.1240	*L. braziliensis*	172	1296	1467
LbrM34_V2.1240	*L. braziliensis*	53	1547	1599
LbrM34_V2.1240	*L. braziliensis*	75	1627	1701
LbrM34_V2.1260	*L. braziliensis*	61	160	220
LbrM34_V2.1270	*L. braziliensis*	200	40	239
LbrM34_V2.1280	*L. braziliensis*	139	72	210
LbrM34_V2.1280	*L. braziliensis*	83	248	330
LbrM34_V2.1280	*L. braziliensis*	86	341	426
LbrM34_V2.1310	*L. braziliensis*	54	1	54
LbrM34_V2.1310	*L. braziliensis*	55	118	172
LbrM34_V2.1310	*L. braziliensis*	74	311	384
LbrM34_V2.1310	*L. braziliensis*	139	571	709
LbrM34_V2.1320	*L. braziliensis*	113	120	232

LbrM34_V2.1330	*L. braziliensis*	45	497	541
LbrM34_V2.1330	*L. braziliensis*	45	596	640
LbrM34_V2.1330	*L. braziliensis*	45	596	640
LbrM34_V2.1340	*L. braziliensis*	187	1	187
LbrM34_V2.1340	*L. braziliensis*	112	216	327
LbrM34_V2.1340	*L. braziliensis*	112	216	327
LbrM34_V2.1350	*L. braziliensis*	63	198	260
LbrM34_V2.1360	*L. braziliensis*	63	198	260
LbrM34_V2.1370	*L. braziliensis*	100	173	272
LbrM34_V2.1370	*L. braziliensis*	71	388	458
LbrM34_V2.1370	*L. braziliensis*	161	467	627
LbrM34_V2.1370	*L. braziliensis*	58	698	755
LbrM34_V2.1370	*L. braziliensis*	44	837	880
LbrM34_V2.1370	*L. braziliensis*	76	1058	1133
LbrM34_V2.1370	*L. braziliensis*	40	1408	1447
LbrM34_V2.1370	*L. braziliensis*	247	1653	1899
LbrM34_V2.1370	*L. braziliensis*	128	1981	2108
LbrM34_V2.1370	*L. braziliensis*	87	2124	2210
LbrM34_V2.1370	*L. braziliensis*	155	2280	2434
LbrM34_V2.1370	*L. braziliensis*	179	2480	2658
LbrM34_V2.1370	*L. braziliensis*	281	2718	2998
LbrM34_V2.1370	*L. braziliensis*	79	3102	3180
LbrM34_V2.1390	*L. braziliensis*	60	1	60
LbrM34_V2.1420	*L. braziliensis*	347	297	643
LbrM34_V2.1430	*L. braziliensis*	64	236	299
LbrM34_V2.1430	*L. braziliensis*	67	386	452
LbrM34_V2.1430	*L. braziliensis*	85	482	566
LbrM34_V2.1440	*L. braziliensis*	88	44	131
LbrM34_V2.1440	*L. braziliensis*	88	167	254
LbrM34_V2.1440	*L. braziliensis*	88	167	254
LbrM34_V2.1460	*L. braziliensis*	130	39	168
LbrM34_V2.1460	*L. braziliensis*	61	340	400
LbrM34_V2.1460	*L. braziliensis*	61	340	400
LbrM34_V2.1470	*L. braziliensis*	64	136	199
LbrM34_V2.1470	*L. braziliensis*	66	842	907
LbrM34_V2.1470	*L. braziliensis*	67	989	1055
LbrM34_V2.1480	*L. braziliensis*	77	25	101
LbrM34_V2.1490	*L. braziliensis*	113	323	435
LbrM34_V2.1500	*L. braziliensis*	69	67	135
LbrM34_V2.1520	*L. braziliensis*	50	475	524
LbrM34_V2.1520	*L. braziliensis*	56	880	935
LbrM34_V2.1520	*L. braziliensis*	56	880	935
LbrM34_V2.1530	*L. braziliensis*	92	246	337
LbrM34_V2.1530	*L. braziliensis*	64	429	492
LbrM34_V2.1530	*L. braziliensis*	50	603	652
LbrM34_V2.1530	*L. braziliensis*	52	847	898
LbrM34_V2.1530	*L. braziliensis*	48	1039	1086

LbrM34_V2.1530	*L. braziliensis*	60	1275	1334
LbrM34_V2.1530	*L. braziliensis*	58	1889	1946
LbrM34_V2.1540	*L. braziliensis*	255	159	413
LbrM34_V2.1550	*L. braziliensis*	59	84	142
LbrM34_V2.1560	*L. braziliensis*	60	27	86
LbrM34_V2.1560	*L. braziliensis*	419	115	533
LbrM34_V2.1560	*L. braziliensis*	96	544	639
LbrM34_V2.1570	*L. braziliensis*	69	72	140
LbrM34_V2.1570	*L. braziliensis*	170	417	586
LbrM34_V2.1580	*L. braziliensis*	40	1	40
LbrM34_V2.1580	*L. braziliensis*	40	104	143
LbrM34_V2.1580	*L. braziliensis*	40	104	143
LbrM34_V2.1590	*L. braziliensis*	75	212	286
LbrM34_V2.1600	*L. braziliensis*	41	18	58
LbrM34_V2.1600	*L. braziliensis*	187	232	418
LbrM34_V2.1620	*L. braziliensis*	68	52	119
LbrM34_V2.1640	*L. braziliensis*	49	688	736
LbrM34_V2.1680	*L. braziliensis*	60	117	176
LbrM34_V2.1690	*L. braziliensis*	49	97	145
LbrM34_V2.1690	*L. braziliensis*	54	157	210
LbrM34_V2.1690	*L. braziliensis*	54	157	210
LbrM34_V2.1710	*L. braziliensis*	49	208	256
LbrM34_V2.1720	*L. braziliensis*	252	57	308
LbrM34_V2.1720	*L. braziliensis*	42	367	408
LbrM34_V2.1720	*L. braziliensis*	42	367	408
LbrM34_V2.1730	*L. braziliensis*	124	1	124
LbrM34_V2.1730	*L. braziliensis*	112	162	273
LbrM34_V2.1770	*L. braziliensis*	98	1	98
LbrM34_V2.1770	*L. braziliensis*	72	125	196
LbrM34_V2.1780	*L. braziliensis*	42	145	186
LbrM34_V2.1780	*L. braziliensis*	115	198	312
LbrM34_V2.1780	*L. braziliensis*	42	407	448
LbrM34_V2.1780	*L. braziliensis*	50	533	582
LbrM34_V2.1780	*L. braziliensis*	42	695	736
LbrM34_V2.1790	*L. braziliensis*	93	213	305
LbrM34_V2.1800	*L. braziliensis*	93	213	305
LbrM34_V2.1810	*L. braziliensis*	62	35	96
LbrM34_V2.1810	*L. braziliensis*	47	154	200
LbrM34_V2.1810	*L. braziliensis*	47	154	200
LbrM34_V2.1830	*L. braziliensis*	44	37	80
LbrM34_V2.1840	*L. braziliensis*	73	63	135
LbrM34_V2.1840	*L. braziliensis*	69	199	267
LbrM34_V2.1850	*L. braziliensis*	394	229	622
LbrM34_V2.1880	*L. braziliensis*	116	138	253
LbrM34_V2.1890	*L. braziliensis*	54	358	411
LbrM34_V2.1890	*L. braziliensis*	164	428	591
LbrM34_V2.1900	*L. braziliensis*	54	200	253

LbrM34_V2.1900	*L. braziliensis*	60	391	450
LbrM34_V2.1900	*L. braziliensis*	45	543	587
LbrM34_V2.1900	*L. braziliensis*	78	656	733
LbrM34_V2.1900	*L. braziliensis*	49	771	819
LbrM34_V2.1910	*L. braziliensis*	64	1	64
LbrM34_V2.1910	*L. braziliensis*	47	409	455
LbrM34_V2.1910	*L. braziliensis*	51	800	850
LbrM34_V2.1920	*L. braziliensis*	120	130	249
LbrM34_V2.1940	*L. braziliensis*	82	131	212
LbrM34_V2.1940	*L. braziliensis*	78	304	381
LbrM34_V2.1940	*L. braziliensis*	88	438	525
LbrM34_V2.1940	*L. braziliensis*	45	1050	1094
LbrM34_V2.1940	*L. braziliensis*	46	1152	1197
LbrM34_V2.1940	*L. braziliensis*	79	1256	1334
LbrM34_V2.1950	*L. braziliensis*	91	117	207
LbrM34_V2.1950	*L. braziliensis*	41	638	678
LbrM34_V2.1950	*L. braziliensis*	42	982	1023
LbrM34_V2.1950	*L. braziliensis*	66	1814	1879
LbrM34_V2.1950	*L. braziliensis*	43	2063	2105
LbrM34_V2.1950	*L. braziliensis*	44	2428	2471
LbrM34_V2.1950	*L. braziliensis*	43	2658	2700
LbrM34_V2.1950	*L. braziliensis*	51	3076	3126
LbrM34_V2.1950	*L. braziliensis*	91	3646	3736
LbrM34_V2.1960	*L. braziliensis*	65	1	65
LbrM34_V2.1970	*L. braziliensis*	54	1	54
LbrM34_V2.1970	*L. braziliensis*	63	207	269
LbrM34_V2.1970	*L. braziliensis*	94	396	489
LbrM34_V2.1980	*L. braziliensis*	288	47	334
LbrM34_V2.1990	*L. braziliensis*	75	1030	1104
LbrM34_V2.2000	*L. braziliensis*	81	214	294
LbrM34_V2.2000	*L. braziliensis*	89	481	569
LbrM34_V2.2000	*L. braziliensis*	89	481	569
LbrM34_V2.2010	*L. braziliensis*	45	85	129
LbrM34_V2.2010	*L. braziliensis*	56	178	233
LbrM34_V2.2010	*L. braziliensis*	88	552	639
LbrM34_V2.2010	*L. braziliensis*	44	888	931
LbrM34_V2.2020	*L. braziliensis*	160	87	246
LbrM34_V2.2020	*L. braziliensis*	47	386	432
LbrM34_V2.2020	*L. braziliensis*	64	625	688
LbrM34_V2.2030	*L. braziliensis*	264	98	361
LbrM34_V2.2030	*L. braziliensis*	58	370	427
LbrM34_V2.2050	*L. braziliensis*	224	1	224
LbrM34_V2.2060	*L. braziliensis*	72	69	140
LbrM34_V2.2060	*L. braziliensis*	43	238	280
LbrM34_V2.2060	*L. braziliensis*	43	238	280
LbrM34_V2.2070	*L. braziliensis*	84	1	84
LbrM34_V2.2070	*L. braziliensis*	87	387	473

LbrM34_V2.2070	*L. braziliensis*	87	387	473
LbrM34_V2.2110	*L. braziliensis*	125	1	125
LbrM34_V2.2110	*L. braziliensis*	52	297	348
LbrM34_V2.2110	*L. braziliensis*	92	394	485
LbrM34_V2.2110	*L. braziliensis*	88	627	714
LbrM34_V2.2120	*L. braziliensis*	41	84	124
LbrM34_V2.2130	*L. braziliensis*	93	103	195
LbrM34_V2.2180	*L. braziliensis*	81	1	81
LbrM34_V2.2180	*L. braziliensis*	62	148	209
LbrM34_V2.2180	*L. braziliensis*	62	148	209
LbrM34_V2.2190	*L. braziliensis*	97	56	152
LbrM34_V2.2190	*L. braziliensis*	78	182	259
LbrM34_V2.2190	*L. braziliensis*	46	273	318
LbrM34_V2.2190	*L. braziliensis*	40	358	397
LbrM34_V2.2190	*L. braziliensis*	63	455	517
LbrM34_V2.2190	*L. braziliensis*	43	553	595
LbrM34_V2.2190	*L. braziliensis*	49	771	819
LbrM34_V2.2190	*L. braziliensis*	147	887	1033
LbrM34_V2.2190	*L. braziliensis*	125	1206	1330
LbrM34_V2.2190	*L. braziliensis*	42	1445	1486
LbrM34_V2.2190	*L. braziliensis*	41	1522	1562
LbrM34_V2.2190	*L. braziliensis*	48	1743	1790
LbrM34_V2.2190	*L. braziliensis*	71	1845	1915
LbrM34_V2.2190	*L. braziliensis*	119	2123	2241
LbrM34_V2.2190	*L. braziliensis*	141	2388	2528
LbrM34_V2.2200	*L. braziliensis*	177	200	376
LbrM34_V2.2200	*L. braziliensis*	111	556	666
LbrM34_V2.2200	*L. braziliensis*	73	880	952
LbrM34_V2.2200	*L. braziliensis*	58	1457	1514
LbrM34_V2.2210	*L. braziliensis*	53	121	173
LbrM34_V2.2220	*L. braziliensis*	113	1	113
LbrM34_V2.2220	*L. braziliensis*	42	614	655
LbrM34_V2.2220	*L. braziliensis*	63	1078	1140
LbrM34_V2.2230	*L. braziliensis*	61	1	61
LbrM34_V2.2240	*L. braziliensis*	86	229	314
LbrM34_V2.2240	*L. braziliensis*	56	538	593
LbrM34_V2.2240	*L. braziliensis*	136	829	964
LbrM34_V2.2240	*L. braziliensis*	82	1021	1102
LbrM34_V2.2240	*L. braziliensis*	113	1222	1334
LbrM34_V2.2250	*L. braziliensis*	110	1	110
LbrM34_V2.2250	*L. braziliensis*	47	359	405
LbrM34_V2.2260	*L. braziliensis*	45	12	56
LbrM34_V2.2270	*L. braziliensis*	49	1	49
LbrM34_V2.2270	*L. braziliensis*	41	76	116
LbrM34_V2.2270	*L. braziliensis*	55	454	508
LbrM34_V2.2270	*L. braziliensis*	118	607	724
LbrM34_V2.2270	*L. braziliensis*	118	749	866

LbrM34_V2.2270	*L. braziliensis*	213	868	1080
LbrM34_V2.2280	*L. braziliensis*	40	343	382
LbrM34_V2.2310	*L. braziliensis*	43	1	43
LbrM34_V2.2310	*L. braziliensis*	71	51	121
LbrM34_V2.2310	*L. braziliensis*	45	144	188
LbrM34_V2.2320	*L. braziliensis*	93	178	270
LbrM34_V2.2320	*L. braziliensis*	52	320	371
LbrM34_V2.2320	*L. braziliensis*	147	414	560
LbrM34_V2.2330	*L. braziliensis*	43	1	43
LbrM34_V2.2330	*L. braziliensis*	59	97	155
LbrM34_V2.2330	*L. braziliensis*	62	414	475
LbrM34_V2.2330	*L. braziliensis*	42	762	803
LbrM34_V2.2330	*L. braziliensis*	80	1010	1089
LbrM34_V2.2330	*L. braziliensis*	194	1106	1299
LbrM34_V2.2340	*L. braziliensis*	55	98	152
LbrM34_V2.2350	*L. braziliensis*	43	424	466
LbrM34_V2.2350	*L. braziliensis*	61	780	840
LbrM34_V2.2350	*L. braziliensis*	43	1180	1222
LbrM34_V2.2360	*L. braziliensis*	492	1	492
LbrM34_V2.2360	*L. braziliensis*	67	542	608
LbrM34_V2.2360	*L. braziliensis*	69	859	927
LbrM34_V2.2370	*L. braziliensis*	60	145	204
LbrM34_V2.2370	*L. braziliensis*	47	316	362
LbrM34_V2.2370	*L. braziliensis*	41	378	418
LbrM34_V2.2380	*L. braziliensis*	51	27	77
LbrM34_V2.2380	*L. braziliensis*	54	126	179
LbrM34_V2.2380	*L. braziliensis*	92	236	327
LbrM34_V2.2380	*L. braziliensis*	63	361	423
LbrM34_V2.2380	*L. braziliensis*	190	463	652
LbrM34_V2.2380	*L. braziliensis*	78	665	742
LbrM34_V2.2400	*L. braziliensis*	56	184	239
LbrM34_V2.2400	*L. braziliensis*	52	545	596
LbrM34_V2.2400	*L. braziliensis*	41	794	834
LbrM34_V2.2400	*L. braziliensis*	62	1022	1083
LbrM34_V2.2400	*L. braziliensis*	69	1169	1237
LbrM34_V2.2400	*L. braziliensis*	66	1378	1443
LbrM34_V2.2400	*L. braziliensis*	107	1683	1789
LbrM34_V2.2400	*L. braziliensis*	88	2165	2252
LbrM34_V2.2410	*L. braziliensis*	209	1	209
LbrM34_V2.2410	*L. braziliensis*	93	302	394
LbrM34_V2.2420	*L. braziliensis*	257	1	257
LbrM34_V2.2420	*L. braziliensis*	50	291	340
LbrM34_V2.2430	*L. braziliensis*	95	16	110
LbrM34_V2.2430	*L. braziliensis*	43	537	579
LbrM34_V2.2430	*L. braziliensis*	296	822	1117
LbrM34_V2.2430	*L. braziliensis*	40	1119	1158
LbrM34_V2.2430	*L. braziliensis*	200	1201	1400

LbrM34_V2.2430	*L. braziliensis*	48	1435	1482
LbrM34_V2.2430	*L. braziliensis*	53	1486	1538
LbrM34_V2.2430	*L. braziliensis*	68	1544	1611
LbrM34_V2.2440	*L. braziliensis*	60	183	242
LbrM34_V2.2460	*L. braziliensis*	52	1	52
LbrM34_V2.2460	*L. braziliensis*	97	56	152
LbrM34_V2.2460	*L. braziliensis*	43	298	340
LbrM34_V2.2480	*L. braziliensis*	153	1	153
LbrM34_V2.2480	*L. braziliensis*	128	331	458
LbrM34_V2.2490	*L. braziliensis*	53	25	77
LbrM34_V2.2490	*L. braziliensis*	125	291	415
LbrM34_V2.2500	*L. braziliensis*	151	68	218
LbrM34_V2.2510	*L. braziliensis*	80	1	80
LbrM34_V2.2510	*L. braziliensis*	66	124	189
LbrM34_V2.2530	*L. braziliensis*	186	25	210
LbrM34_V2.2540	*L. braziliensis*	54	1	54
LbrM34_V2.2540	*L. braziliensis*	103	167	269
LbrM34_V2.2540	*L. braziliensis*	117	289	405
LbrM34_V2.2550	*L. braziliensis*	93	110	202
LbrM34_V2.2550	*L. braziliensis*	151	395	545
LbrM34_V2.2570	*L. braziliensis*	93	1	93
LbrM34_V2.2570	*L. braziliensis*	312	113	424
LbrM34_V2.2570	*L. braziliensis*	149	438	586
LbrM34_V2.2570	*L. braziliensis*	74	595	668
LbrM34_V2.2580	*L. braziliensis*	92	1	92
LbrM34_V2.2590	*L. braziliensis*	57	332	388
LbrM34_V2.2590	*L. braziliensis*	46	1059	1104
LbrM34_V2.2590	*L. braziliensis*	72	1147	1218
LbrM34_V2.2600	*L. braziliensis*	56	42	97
LbrM34_V2.2600	*L. braziliensis*	95	165	259
LbrM34_V2.2610	*L. braziliensis*	60	52	111
LbrM34_V2.2610	*L. braziliensis*	60	149	208
LbrM34_V2.2610	*L. braziliensis*	60	149	208
LbrM34_V2.2620	*L. braziliensis*	184	89	272
LbrM34_V2.2620	*L. braziliensis*	46	460	505
LbrM34_V2.2620	*L. braziliensis*	46	697	742
LbrM34_V2.2630	*L. braziliensis*	68	1	68
LbrM34_V2.2640	*L. braziliensis*	55	284	338
LbrM34_V2.2650	*L. braziliensis*	301	106	406
LbrM34_V2.2650	*L. braziliensis*	95	411	505
LbrM34_V2.2660	*L. braziliensis*	60	11	70
LbrM34_V2.2660	*L. braziliensis*	128	300	427
LbrM34_V2.2660	*L. braziliensis*	67	587	653
LbrM34_V2.2670	*L. braziliensis*	40	61	100
LbrM34_V2.2670	*L. braziliensis*	58	359	416
LbrM34_V2.2670	*L. braziliensis*	74	941	1014
LbrM34_V2.2680	*L. braziliensis*	47	273	319

LbrM34_V2.2680	*L. braziliensis*	45	527	571
LbrM34_V2.2680	*L. braziliensis*	83	580	662
LbrM34_V2.2690	*L. braziliensis*	46	318	363
LbrM34_V2.2710	*L. braziliensis*	41	265	305
LbrM34_V2.2710	*L. braziliensis*	83	356	438
LbrM34_V2.2730	*L. braziliensis*	194	231	424
LbrM34_V2.2740	*L. braziliensis*	41	282	322
LbrM34_V2.2760	*L. braziliensis*	194	286	479
LbrM34_V2.2770	*L. braziliensis*	153	1	153
LbrM34_V2.2780	*L. braziliensis*	45	139	183
LbrM34_V2.2780	*L. braziliensis*	90	379	468
LbrM34_V2.2780	*L. braziliensis*	45	510	554
LbrM34_V2.2780	*L. braziliensis*	51	565	615
LbrM34_V2.2800	*L. braziliensis*	58	207	264
LbrM34_V2.2800	*L. braziliensis*	40	270	309
LbrM34_V2.2800	*L. braziliensis*	105	361	465
LbrM34_V2.2810	*L. braziliensis*	46	289	334
LbrM34_V2.2820	*L. braziliensis*	53	1	53
LbrM34_V2.2830	*L. braziliensis*	58	30	87
LbrM34_V2.2830	*L. braziliensis*	62	90	151
LbrM34_V2.2830	*L. braziliensis*	59	225	283
LbrM34_V2.2830	*L. braziliensis*	54	318	371
LbrM34_V2.2830	*L. braziliensis*	148	546	693
LbrM34_V2.2850	*L. braziliensis*	76	1	76
LbrM34_V2.2860	*L. braziliensis*	70	921	990
LbrM34_V2.2870	*L. braziliensis*	60	510	569
LbrM34_V2.2880	*L. braziliensis*	86	145	230
LbrM34_V2.2880	*L. braziliensis*	49	332	380
LbrM34_V2.2890	*L. braziliensis*	268	1	268
LbrM34_V2.2890	*L. braziliensis*	86	393	478
LbrM34_V2.2900	*L. braziliensis*	55	20	74
LbrM34_V2.2900	*L. braziliensis*	44	369	412
LbrM34_V2.2950	*L. braziliensis*	46	11	56
LbrM34_V2.2960	*L. braziliensis*	109	86	194
LbrM34_V2.2960	*L. braziliensis*	60	273	332
LbrM34_V2.2960	*L. braziliensis*	56	357	412
LbrM34_V2.2970	*L. braziliensis*	43	355	397
LbrM34_V2.2970	*L. braziliensis*	50	864	913
LbrM34_V2.2970	*L. braziliensis*	50	864	913
LbrM34_V2.2980	*L. braziliensis*	53	1	53
LbrM34_V2.2990	*L. braziliensis*	43	470	512
LbrM34_V2.3000	*L. braziliensis*	75	67	141
LbrM34_V2.3000	*L. braziliensis*	59	186	244
LbrM34_V2.3000	*L. braziliensis*	50	379	428
LbrM34_V2.3010	*L. braziliensis*	368	1	368
LbrM34_V2.3010	*L. braziliensis*	71	790	860
LbrM34_V2.3020	*L. braziliensis*	41	160	200

LbrM34_V2.3020	*L. braziliensis*	49	340	388
LbrM34_V2.3020	*L. braziliensis*	49	340	388
LbrM34_V2.3030	*L. braziliensis*	62	69	130
LbrM34_V2.3030	*L. braziliensis*	41	187	227
LbrM34_V2.3030	*L. braziliensis*	49	894	942
LbrM34_V2.3030	*L. braziliensis*	64	966	1029
LbrM34_V2.3030	*L. braziliensis*	67	1274	1340
LbrM34_V2.3030	*L. braziliensis*	99	1353	1451
LbrM34_V2.3030	*L. braziliensis*	45	1461	1505
LbrM34_V2.3030	*L. braziliensis*	45	1533	1577
LbrM34_V2.3030	*L. braziliensis*	91	1617	1707
LbrM34_V2.3030	*L. braziliensis*	49	1712	1760
LbrM34_V2.3040	*L. braziliensis*	55	1	55
LbrM34_V2.3060	*L. braziliensis*	73	1	73
LbrM34_V2.3060	*L. braziliensis*	149	208	356
LbrM34_V2.3080	*L. braziliensis*	396	1	396
LbrM34_V2.3080	*L. braziliensis*	154	430	583
LbrM34_V2.3080	*L. braziliensis*	108	789	896
LbrM34_V2.3080	*L. braziliensis*	246	1129	1374
LbrM34_V2.3080	*L. braziliensis*	189	1398	1586
LbrM34_V2.3080	*L. braziliensis*	43	1720	1762
LbrM34_V2.3090	*L. braziliensis*	44	1	44
LbrM34_V2.3090	*L. braziliensis*	41	55	95
LbrM34_V2.3090	*L. braziliensis*	72	124	195
LbrM34_V2.3100	*L. braziliensis*	40	1	40
LbrM34_V2.3100	*L. braziliensis*	54	164	217
LbrM34_V2.3100	*L. braziliensis*	239	301	539
LbrM34_V2.3100	*L. braziliensis*	48	549	596
LbrM34_V2.3100	*L. braziliensis*	69	740	808
LbrM34_V2.3110	*L. braziliensis*	124	383	506
LbrM34_V2.3110	*L. braziliensis*	73	517	589
LbrM34_V2.3110	*L. braziliensis*	102	595	696
LbrM34_V2.3120	*L. braziliensis*	170	52	221
LbrM34_V2.3150	*L. braziliensis*	108	1	108
LbrM34_V2.3150	*L. braziliensis*	249	210	458
LbrM34_V2.3150	*L. braziliensis*	167	492	658
LbrM34_V2.3150	*L. braziliensis*	112	689	800
LbrM34_V2.3170	*L. braziliensis*	205	1	205
LbrM34_V2.3170	*L. braziliensis*	85	228	312
LbrM34_V2.3180	*L. braziliensis*	170	19	188
LbrM34_V2.3190	*L. braziliensis*	103	1	103
LbrM34_V2.3200	*L. braziliensis*	103	1	103
LbrM34_V2.3210	*L. braziliensis*	82	302	383
LbrM34_V2.3210	*L. braziliensis*	55	453	507
LbrM34_V2.3210	*L. braziliensis*	47	552	598
LbrM34_V2.3220	*L. braziliensis*	159	116	274
LbrM34_V2.3240	*L. braziliensis*	42	321	362

LbrM34_V2.3250	*L. braziliensis*	40	129	168
LbrM34_V2.3260	*L. braziliensis*	105	358	462
LbrM34_V2.3270	*L. braziliensis*	55	584	638
LbrM34_V2.3270	*L. braziliensis*	54	982	1035
LbrM34_V2.3270	*L. braziliensis*	45	1701	1745
LbrM34_V2.3270	*L. braziliensis*	56	2021	2076
LbrM34_V2.3280	*L. braziliensis*	129	1	129
LbrM34_V2.3290	*L. braziliensis*	94	438	531
LbrM34_V2.3300	*L. braziliensis*	42	172	213
LbrM34_V2.3310	*L. braziliensis*	46	251	296
LbrM34_V2.3320	*L. braziliensis*	42	1	42
LbrM34_V2.3320	*L. braziliensis*	48	93	140
LbrM34_V2.3320	*L. braziliensis*	48	551	598
LbrM34_V2.3330	*L. braziliensis*	125	1	125
LbrM34_V2.3340	*L. braziliensis*	60	160	219
LbrM34_V2.3340	*L. braziliensis*	61	481	541
LbrM34_V2.3340	*L. braziliensis*	61	481	541
LbrM34_V2.3360	*L. braziliensis*	60	45	104
LbrM34_V2.3360	*L. braziliensis*	95	376	470
LbrM34_V2.3370	*L. braziliensis*	83	48	130
LbrM34_V2.3380	*L. braziliensis*	52	286	337
LbrM34_V2.3390	*L. braziliensis*	120	222	341
LbrM34_V2.3410	*L. braziliensis*	105	140	244
LbrM34_V2.3410	*L. braziliensis*	308	431	738
LbrM34_V2.3420	*L. braziliensis*	241	186	426
LbrM34_V2.3430	*L. braziliensis*	111	73	183
LbrM34_V2.3440	*L. braziliensis*	72	72	143
LbrM34_V2.3440	*L. braziliensis*	360	192	551
LbrM34_V2.3440	*L. braziliensis*	70	885	954
LbrM34_V2.3440	*L. braziliensis*	83	986	1068
LbrM34_V2.3440	*L. braziliensis*	75	1087	1161
LbrM34_V2.3450	*L. braziliensis*	187	1	187
LbrM34_V2.3450	*L. braziliensis*	53	413	465
LbrM34_V2.3450	*L. braziliensis*	53	413	465
LbrM34_V2.3460	*L. braziliensis*	103	28	130
LbrM34_V2.3480	*L. braziliensis*	42	1	42
LbrM34_V2.3480	*L. braziliensis*	221	120	340
LbrM34_V2.3480	*L. braziliensis*	226	374	599
LbrM34_V2.3480	*L. braziliensis*	79	670	748
LbrM34_V2.3490	*L. braziliensis*	74	295	368
LbrM34_V2.3500	*L. braziliensis*	42	1	42
LbrM34_V2.3500	*L. braziliensis*	221	120	340
LbrM34_V2.3500	*L. braziliensis*	226	374	599
LbrM34_V2.3530	*L. braziliensis*	168	1	168
LbrM34_V2.3530	*L. braziliensis*	55	781	835
LbrM34_V2.3530	*L. braziliensis*	86	986	1071
LbrM34_V2.3530	*L. braziliensis*	62	1097	1158

LbrM34_V2.3550	*L. braziliensis*	105	231	335
LbrM34_V2.3550	*L. braziliensis*	326	403	728
LbrM34_V2.3550	*L. braziliensis*	60	970	1029
LbrM34_V2.3560	*L. braziliensis*	60	1	60
LbrM34_V2.3590	*L. braziliensis*	63	558	620
LbrM34_V2.3610	*L. braziliensis*	57	51	107
LbrM34_V2.3610	*L. braziliensis*	65	138	202
LbrM34_V2.3620	*L. braziliensis*	220	1	220
LbrM34_V2.3620	*L. braziliensis*	48	251	298
LbrM34_V2.3620	*L. braziliensis*	48	251	298
LbrM34_V2.3630	*L. braziliensis*	53	724	776
LbrM34_V2.3630	*L. braziliensis*	42	810	851
LbrM34_V2.3630	*L. braziliensis*	48	1012	1059
LbrM34_V2.3630	*L. braziliensis*	83	1260	1342
LbrM34_V2.3690	*L. braziliensis*	96	1	96
LbrM34_V2.3700	*L. braziliensis*	114	1	114
LbrM34_V2.3700	*L. braziliensis*	144	173	316
LbrM34_V2.3700	*L. braziliensis*	56	385	440
LbrM34_V2.3710	*L. braziliensis*	96	10	105
LbrM34_V2.3710	*L. braziliensis*	79	141	219
LbrM34_V2.3710	*L. braziliensis*	67	252	318
LbrM34_V2.3710	*L. braziliensis*	47	435	481
LbrM34_V2.3720	*L. braziliensis*	57	85	141
LbrM34_V2.3730	*L. braziliensis*	48	197	244
LbrM34_V2.3730	*L. braziliensis*	62	307	368
LbrM34_V2.3730	*L. braziliensis*	53	495	547
LbrM34_V2.3730	*L. braziliensis*	126	566	691
LbrM34_V2.3740	*L. braziliensis*	40	1	40
LbrM34_V2.3750	*L. braziliensis*	72	225	296
LbrM34_V2.3760	*L. braziliensis*	40	1	40
LbrM34_V2.3800	*L. braziliensis*	55	1	55
LbrM34_V2.3800	*L. braziliensis*	85	66	150
LbrM34_V2.3800	*L. braziliensis*	47	159	205
LbrM34_V2.3800	*L. braziliensis*	40	310	349
LbrM34_V2.3830	*L. braziliensis*	87	226	312
LbrM34_V2.3840	*L. braziliensis*	50	522	571
LbrM34_V2.3860	*L. braziliensis*	67	378	444
LbrM34_V2.3870	*L. braziliensis*	45	25	69
LbrM34_V2.3910	*L. braziliensis*	392	35	426
LbrM34_V2.3920	*L. braziliensis*	42	19	60
LbrM34_V2.3930	*L. braziliensis*	78	1	78
LbrM34_V2.3940	*L. braziliensis*	50	1	50
LbrM34_V2.3940	*L. braziliensis*	46	500	545
LbrM34_V2.3940	*L. braziliensis*	45	829	873
LbrM34_V2.3940	*L. braziliensis*	40	2081	2120
LbrM34_V2.3950	*L. braziliensis*	233	79	311
LbrM34_V2.3950	*L. braziliensis*	42	442	483

LbrM34_V2.3950	*L. braziliensis*	92	598	689
LbrM34_V2.3960	*L. braziliensis*	743	30	772
LbrM34_V2.3980	*L. braziliensis*	135	1	135
LbrM34_V2.3980	*L. braziliensis*	65	228	292
LbrM34_V2.3980	*L. braziliensis*	54	332	385
LbrM34_V2.3980	*L. braziliensis*	111	403	513
LbrM34_V2.3980	*L. braziliensis*	84	570	653
LbrM34_V2.3990	*L. braziliensis*	52	1	52
LbrM34_V2.4000	*L. braziliensis*	63	87	149
LbrM34_V2.4010	*L. braziliensis*	93	404	496
LbrM34_V2.4010	*L. braziliensis*	122	570	691
LbrM34_V2.4010	*L. braziliensis*	122	570	691
LbrM34_V2.4020	*L. braziliensis*	48	35	82
LbrM34_V2.4020	*L. braziliensis*	200	114	313
LbrM34_V2.4020	*L. braziliensis*	40	335	374
LbrM34_V2.4040	*L. braziliensis*	52	237	288
LbrM34_V2.4040	*L. braziliensis*	76	375	450
LbrM34_V2.4040	*L. braziliensis*	287	453	739
LbrM34_V2.4040	*L. braziliensis*	160	908	1067
LbrM34_V2.4060	*L. braziliensis*	43	141	183
LbrM34_V2.4060	*L. braziliensis*	52	287	338
LbrM34_V2.4060	*L. braziliensis*	52	287	338
LbrM34_V2.4070	*L. braziliensis*	106	1	106
LbrM34_V2.4070	*L. braziliensis*	435	134	568
LbrM34_V2.4070	*L. braziliensis*	119	706	824
LbrM34_V2.4070	*L. braziliensis*	44	845	888
LbrM34_V2.4070	*L. braziliensis*	102	906	1007
LbrM34_V2.4070	*L. braziliensis*	45	1020	1064
LbrM34_V2.4090	*L. braziliensis*	55	1	55
LbrM34_V2.4100	*L. braziliensis*	84	54	137
LbrM34_V2.4110	*L. braziliensis*	90	80	169
LbrM34_V2.4110	*L. braziliensis*	60	214	273
LbrM34_V2.4110	*L. braziliensis*	117	409	525
LbrM34_V2.4110	*L. braziliensis*	168	579	746
LbrM34_V2.4110	*L. braziliensis*	62	766	827
LbrM34_V2.4120	*L. braziliensis*	583	1	583
LbrM34_V2.4130	*L. braziliensis*	70	236	305
LbrM34_V2.4130	*L. braziliensis*	152	368	519
LbrM34_V2.4140	*L. braziliensis*	54	241	294
LbrM34_V2.4150	*L. braziliensis*	93	1	93
LbrM34_V2.4150	*L. braziliensis*	101	131	231
LbrM34_V2.4170	*L. braziliensis*	196	41	236
LbrM34_V2.4190	*L. braziliensis*	84	1	84
LbrM34_V2.4200	*L. braziliensis*	389	17	405
LbrM34_V2.4200	*L. braziliensis*	121	481	601
LbrM34_V2.4210	*L. braziliensis*	74	145	218
LbrM34_V2.4210	*L. braziliensis*	63	304	366

LbrM34_V2.4210	*L. braziliensis*	70	612	681
LbrM34_V2.4240	*L. braziliensis*	177	74	250
LbrM34_V2.4260	*L. braziliensis*	152	1	152
LbrM34_V2.4260	*L. braziliensis*	119	298	416
LbrM34_V2.4260	*L. braziliensis*	55	455	509
LbrM34_V2.4260	*L. braziliensis*	82	514	595
LbrM34_V2.4260	*L. braziliensis*	116	682	797
LbrM34_V2.4270	*L. braziliensis*	64	72	135
LbrM34_V2.4270	*L. braziliensis*	127	149	275
LbrM34_V2.4270	*L. braziliensis*	127	149	275
LbrM34_V2.4280	*L. braziliensis*	41	33	73
LbrM34_V2.4280	*L. braziliensis*	40	187	226
LbrM34_V2.4280	*L. braziliensis*	40	187	226
LbrM34_V2.4300	*L. braziliensis*	50	42	91
LbrM34_V2.4310	*L. braziliensis*	74	1	74
LbrM34_V2.4310	*L. braziliensis*	81	214	294
LbrM34_V2.4310	*L. braziliensis*	208	403	610
LbrM34_V2.4330	*L. braziliensis*	53	238	290
LbrM34_V2.4340	*L. braziliensis*	91	675	765
LbrM34_V2.4340	*L. braziliensis*	141	1215	1355
LbrM34_V2.4340	*L. braziliensis*	40	2087	2126
LbrM34_V2.4350	*L. braziliensis*	46	69	114
LbrM34_V2.4360	*L. braziliensis*	480	46	525
LbrM34_V2.4380	*L. braziliensis*	60	10	69
LbrM34_V2.4380	*L. braziliensis*	70	82	151
LbrM34_V2.4380	*L. braziliensis*	44	162	205
LbrM34_V2.4430	*L. braziliensis*	46	1	46
LbrM34_V2.4430	*L. braziliensis*	83	101	183
LbrM34_V2.4430	*L. braziliensis*	43	222	264
LbrM34_V2.4450	*L. braziliensis*	91	111	201
LbrM34_V2.4490	*L. braziliensis*	69	1	69
LbrM34_V2.4510	*L. braziliensis*	57	258	314
LbrM34_V2.4510	*L. braziliensis*	72	515	586
LbrM34_V2.4510	*L. braziliensis*	72	515	586
LbrM34_V2.4520	*L. braziliensis*	54	438	491
LbrM34_V2.4520	*L. braziliensis*	41	919	959
LbrM34_V2.4520	*L. braziliensis*	41	919	959
LbrM34_V2.4540	*L. braziliensis*	62	118	179
LbrM34_V2.4560	*L. braziliensis*	54	331	384
LbrM34_V2.4560	*L. braziliensis*	158	403	560
LbrM34_V2.4580	*L. braziliensis*	436	87	522
LbrM34_V2.4600	*L. braziliensis*	52	231	282
LbrM34_V2.4620	*L. braziliensis*	114	1	114
LbrM34_V2.4630	*L. braziliensis*	137	28	164
LbrM34_V2.4640	*L. braziliensis*	81	116	196
LbrM34_V2.4650	*L. braziliensis*	135	295	429
LbrM34_V2.4670	*L. braziliensis*	46	211	256

LbrM34_V2.4730	*L. braziliensis*	54	301	354
LbrM34_V2.4750	*L. braziliensis*	41	1	41
LbrM34_V2.4760	*L. braziliensis*	63	1	63
LbrM34_V2.4760	*L. braziliensis*	148	1319	1466
LbrM34_V2.4790	*L. braziliensis*	49	329	377
LbrM34_V2.4800	*L. braziliensis*	41	1	41
LbrM34_V2.4820	*L. braziliensis*	64	1	64
LbrM34_V2.4830	*L. braziliensis*	874	39	912
LbrM34_V2.4860	*L. braziliensis*	81	1	81
LbrM34_V2.4870	*L. braziliensis*	166	751	916
LbrM34_V2.4880	*L. braziliensis*	57	1	57
LbrM34_V2.4880	*L. braziliensis*	72	93	164
LbrM34_V2.4880	*L. braziliensis*	72	93	164
LbrM34_V2.4890	*L. braziliensis*	158	29	186
LbrM34_V2.4900	*L. braziliensis*	60	1	60
LbrM34_V2.4900	*L. braziliensis*	577	175	751
LbrM34_V2.4900	*L. braziliensis*	188	805	992
LbrM34_V2.4910	*L. braziliensis*	65	1	65
LbrM34_V2.4910	*L. braziliensis*	44	105	148
LbrM34_V2.4910	*L. braziliensis*	44	105	148
LbrM34_V2.4930	*L. braziliensis*	61	325	385
LbrM34_V2.4940	*L. braziliensis*	81	1	81
LbrM34_V2.4950	*L. braziliensis*	122	270	391
LbrM34_V2.4960	*L. braziliensis*	43	96	138
LbrM34_V2.4960	*L. braziliensis*	57	437	493
LbrM34_V2.4960	*L. braziliensis*	57	437	493
LbrM34_V2.4970	*L. braziliensis*	128	34	161
LbrM34_V2.4970	*L. braziliensis*	86	174	259
LbrM34_V2.4980	*L. braziliensis*	118	381	498
LbrM34_V2.4990	*L. braziliensis*	279	62	340
LbrM34_V2.4990	*L. braziliensis*	271	375	645
LbrM34_V2.5000	*L. braziliensis*	65	87	151
LbrM34_V2.5000	*L. braziliensis*	63	270	332
LbrM34_V2.5030	*L. braziliensis*	56	1	56
LbrM34_V2.5050	*L. braziliensis*	43	61	103
LbrM34_V2.5050	*L. braziliensis*	60	224	283
LbrM34_V2.5050	*L. braziliensis*	60	224	283
LbrM34_V2.5070	*L. braziliensis*	161	34	194
LbrM34_V2.5070	*L. braziliensis*	41	262	302
LbrM34_V2.5070	*L. braziliensis*	43	326	368
LbrM34_V2.5090	*L. braziliensis*	41	261	301
LbrM34_V2.5120	*L. braziliensis*	85	1	85
LbrM34_V2.5150	*L. braziliensis*	41	162	202
LbrM34_V2.5150	*L. braziliensis*	50	208	257
LbrM34_V2.5150	*L. braziliensis*	50	208	257
LbrM34_V2.5160	*L. braziliensis*	62	403	464
LbrM34_V2.5170	*L. braziliensis*	53	1	53

LbrM34_V2.5170	*L. braziliensis*	72	98	169
LbrM34_V2.5170	*L. braziliensis*	48	215	262
LbrM34_V2.5210	*L. braziliensis*	41	108	148
LbrM34_V2.5230	*L. braziliensis*	50	64	113
LbrM34_V2.5240	*L. braziliensis*	44	169	212
LbrM34_V2.5250	*L. braziliensis*	63	1	63
LbrM34_V2.5260	*L. braziliensis*	49	1	49
LbrM34_V2.5260	*L. braziliensis*	66	86	151
LbrM34_V2.5260	*L. braziliensis*	66	86	151
LbrM34_V2.5280	*L. braziliensis*	48	154	201
LbrM34_V2.5330	*L. braziliensis*	114	1	114
LbrM34_V2.5330	*L. braziliensis*	66	1458	1523
LbrM34_V2.5330	*L. braziliensis*	66	2455	2520
LbrM34_V2.5330	*L. braziliensis*	62	4326	4387
LbrM34_V2.5330	*L. braziliensis*	59	5081	5139
LbrM34_V2.5340	*L. braziliensis*	55	90	144
LbrM34_V2.5340	*L. braziliensis*	78	147	224
LbrM34_V2.5340	*L. braziliensis*	172	436	607
LbrM34_V2.5340	*L. braziliensis*	112	786	897
LbrM34_V2.5340	*L. braziliensis*	43	1111	1153
LbrM34_V2.5340	*L. braziliensis*	58	1688	1745
LbrM34_V2.5350	*L. braziliensis*	927	36	962
LbrM35_V2.0020	*L. braziliensis*	110	19	128
LbrM35_V2.0030	*L. braziliensis*	177	200	376
LbrM35_V2.0030	*L. braziliensis*	111	556	666
LbrM35_V2.0030	*L. braziliensis*	58	1457	1514
LbrM35_V2.0040	*L. braziliensis*	60	1	60
LbrM35_V2.0060	*L. braziliensis*	60	1	60
LbrM35_V2.0070	*L. braziliensis*	78	1	78
LbrM35_V2.0090	*L. braziliensis*	55	1	55
LbrM35_V2.0090	*L. braziliensis*	124	428	551
LbrM35_V2.0090	*L. braziliensis*	124	428	551
LbrM35_V2.0110	*L. braziliensis*	61	1	61
LbrM35_V2.0110	*L. braziliensis*	182	98	279
LbrM35_V2.0120	*L. braziliensis*	54	88	141
LbrM35_V2.0130	*L. braziliensis*	68	71	138
LbrM35_V2.0180	*L. braziliensis*	41	1	41
LbrM35_V2.0180	*L. braziliensis*	78	148	225
LbrM35_V2.0180	*L. braziliensis*	78	148	225
LbrM35_V2.0200	*L. braziliensis*	138	101	238
LbrM35_V2.0210	*L. braziliensis*	57	585	641
LbrM35_V2.0230	*L. braziliensis*	47	239	285
LbrM35_V2.0240	*L. braziliensis*	41	115	155
LbrM35_V2.0320	*L. braziliensis*	80	1	80
LbrM35_V2.0320	*L. braziliensis*	55	118	172
LbrM35_V2.0320	*L. braziliensis*	55	118	172
LbrM35_V2.0330	*L. braziliensis*	48	311	358

LbrM35_V2.0340	*L. braziliensis*	56	1	56
LbrM35_V2.0350	*L. braziliensis*	114	80	193
LbrM35_V2.0350	*L. braziliensis*	88	589	676
LbrM35_V2.0350	*L. braziliensis*	88	589	676
LbrM35_V2.0360	*L. braziliensis*	56	146	201
LbrM35_V2.0370	*L. braziliensis*	44	192	235
LbrM35_V2.0420	*L. braziliensis*	52	77	128
LbrM35_V2.0440	*L. braziliensis*	198	1	198
LbrM35_V2.0450	*L. braziliensis*	42	72	113
LbrM35_V2.0460	*L. braziliensis*	62	1	62
LbrM35_V2.0460	*L. braziliensis*	56	326	381
LbrM35_V2.0460	*L. braziliensis*	56	326	381
LbrM35_V2.0470	*L. braziliensis*	146	1	146
LbrM35_V2.0480	*L. braziliensis*	128	1	128
LbrM35_V2.0480	*L. braziliensis*	92	300	391
LbrM35_V2.0490	*L. braziliensis*	61	46	106
LbrM35_V2.0490	*L. braziliensis*	66	147	212
LbrM35_V2.0490	*L. braziliensis*	119	250	368
LbrM35_V2.0500	*L. braziliensis*	46	1	46
LbrM35_V2.0510	*L. braziliensis*	51	1	51
LbrM35_V2.0520	*L. braziliensis*	57	52	108
LbrM35_V2.0540	*L. braziliensis*	78	1	78
LbrM35_V2.0540	*L. braziliensis*	414	125	538
LbrM35_V2.0550	*L. braziliensis*	40	174	213
LbrM35_V2.0550	*L. braziliensis*	42	576	617
LbrM35_V2.0550	*L. braziliensis*	42	576	617
LbrM35_V2.0560	*L. braziliensis*	68	171	238
LbrM35_V2.0570	*L. braziliensis*	46	14	59
LbrM35_V2.0580	*L. braziliensis*	159	40	198
LbrM35_V2.0580	*L. braziliensis*	139	203	341
LbrM35_V2.0590	*L. braziliensis*	43	338	380
LbrM35_V2.0590	*L. braziliensis*	100	411	510
LbrM35_V2.0590	*L. braziliensis*	100	411	510
LbrM35_V2.0600	*L. braziliensis*	48	1	48
LbrM35_V2.0610	*L. braziliensis*	80	27	106
LbrM35_V2.0610	*L. braziliensis*	49	293	341
LbrM35_V2.0610	*L. braziliensis*	49	293	341
LbrM35_V2.0630	*L. braziliensis*	49	93	141
LbrM35_V2.0650	*L. braziliensis*	203	73	275
LbrM35_V2.0670	*L. braziliensis*	83	1	83
LbrM35_V2.0670	*L. braziliensis*	135	121	255
LbrM35_V2.0680	*L. braziliensis*	51	413	463
LbrM35_V2.0680	*L. braziliensis*	42	705	746
LbrM35_V2.0680	*L. braziliensis*	42	705	746
LbrM35_V2.0720	*L. braziliensis*	56	67	122
LbrM35_V2.0730	*L. braziliensis*	65	1	65
LbrM35_V2.0730	*L. braziliensis*	50	111	160

LbrM35_V2.0730	*L. braziliensis*	50	111	160
LbrM35_V2.0780	*L. braziliensis*	54	44	97
LbrM35_V2.0800	*L. braziliensis*	55	1	55
LbrM35_V2.0800	*L. braziliensis*	63	102	164
LbrM35_V2.0800	*L. braziliensis*	417	196	612
LbrM35_V2.0810	*L. braziliensis*	76	11	86
LbrM35_V2.0820	*L. braziliensis*	56	17	72
LbrM35_V2.0820	*L. braziliensis*	52	178	229
LbrM35_V2.0830	*L. braziliensis*	74	127	200
LbrM35_V2.0840	*L. braziliensis*	141	320	460
LbrM35_V2.0850	*L. braziliensis*	77	1	77
LbrM35_V2.0850	*L. braziliensis*	202	295	496
LbrM35_V2.0860	*L. braziliensis*	195	1	195
LbrM35_V2.0860	*L. braziliensis*	59	209	267
LbrM35_V2.0860	*L. braziliensis*	43	372	414
LbrM35_V2.0860	*L. braziliensis*	44	485	528
LbrM35_V2.0870	*L. braziliensis*	102	116	217
LbrM35_V2.0870	*L. braziliensis*	263	297	559
LbrM35_V2.0870	*L. braziliensis*	41	940	980
LbrM35_V2.0870	*L. braziliensis*	59	1122	1180
LbrM35_V2.0870	*L. braziliensis*	47	1247	1293
LbrM35_V2.0880	*L. braziliensis*	174	1	174
LbrM35_V2.0880	*L. braziliensis*	44	342	385
LbrM35_V2.0880	*L. braziliensis*	81	703	783
LbrM35_V2.0890	*L. braziliensis*	125	1	125
LbrM35_V2.0890	*L. braziliensis*	193	154	346
LbrM35_V2.0900	*L. braziliensis*	173	1	173
LbrM35_V2.0900	*L. braziliensis*	205	221	425
LbrM35_V2.0900	*L. braziliensis*	41	726	766
LbrM35_V2.0910	*L. braziliensis*	124	8	131
LbrM35_V2.0910	*L. braziliensis*	193	190	382
LbrM35_V2.0910	*L. braziliensis*	167	393	559
LbrM35_V2.0920	*L. braziliensis*	45	430	474
LbrM35_V2.0940	*L. braziliensis*	40	46	85
LbrM35_V2.0950	*L. braziliensis*	45	410	454
LbrM35_V2.0950	*L. braziliensis*	158	519	676
LbrM35_V2.0950	*L. braziliensis*	110	802	911
LbrM35_V2.0950	*L. braziliensis*	57	1355	1411
LbrM35_V2.0950	*L. braziliensis*	55	1750	1804
LbrM35_V2.0960	*L. braziliensis*	57	1	57
LbrM35_V2.0960	*L. braziliensis*	45	68	112
LbrM35_V2.0960	*L. braziliensis*	101	391	491
LbrM35_V2.0960	*L. braziliensis*	61	510	570
LbrM35_V2.0960	*L. braziliensis*	132	658	789
LbrM35_V2.0970	*L. braziliensis*	276	27	302
LbrM35_V2.0970	*L. braziliensis*	216	542	757
LbrM35_V2.0970	*L. braziliensis*	41	820	860

LbrM35_V2.0980	*L. braziliensis*	80	321	400
LbrM35_V2.0980	*L. braziliensis*	107	420	526
LbrM35_V2.0990	*L. braziliensis*	121	74	194
LbrM35_V2.0990	*L. braziliensis*	41	238	278
LbrM35_V2.0990	*L. braziliensis*	45	834	878
LbrM35_V2.1020	*L. braziliensis*	105	408	512
LbrM35_V2.1020	*L. braziliensis*	95	557	651
LbrM35_V2.1030	*L. braziliensis*	143	54	196
LbrM35_V2.1030	*L. braziliensis*	124	1101	1224
LbrM35_V2.1030	*L. braziliensis*	53	1226	1278
LbrM35_V2.1040	*L. braziliensis*	47	85	131
LbrM35_V2.1040	*L. braziliensis*	42	427	468
LbrM35_V2.1040	*L. braziliensis*	42	508	549
LbrM35_V2.1050	*L. braziliensis*	65	89	153
LbrM35_V2.1060	*L. braziliensis*	65	89	153
LbrM35_V2.1070	*L. braziliensis*	54	515	568
LbrM35_V2.1070	*L. braziliensis*	59	832	890
LbrM35_V2.1070	*L. braziliensis*	117	2712	2828
LbrM35_V2.1070	*L. braziliensis*	64	2951	3014
LbrM35_V2.1080	*L. braziliensis*	67	351	417
LbrM35_V2.1080	*L. braziliensis*	67	521	587
LbrM35_V2.1080	*L. braziliensis*	51	665	715
LbrM35_V2.1090	*L. braziliensis*	76	1	76
LbrM35_V2.1100	*L. braziliensis*	77	94	170
LbrM35_V2.1110	*L. braziliensis*	77	94	170
LbrM35_V2.1120	*L. braziliensis*	40	635	674
LbrM35_V2.1120	*L. braziliensis*	40	1102	1141
LbrM35_V2.1120	*L. braziliensis*	40	1102	1141
LbrM35_V2.1130	*L. braziliensis*	50	296	345
LbrM35_V2.1130	*L. braziliensis*	44	368	411
LbrM35_V2.1130	*L. braziliensis*	50	571	620
LbrM35_V2.1130	*L. braziliensis*	47	762	808
LbrM35_V2.1130	*L. braziliensis*	51	1145	1195
LbrM35_V2.1160	*L. braziliensis*	215	1	215
LbrM35_V2.1160	*L. braziliensis*	107	303	409
LbrM35_V2.1160	*L. braziliensis*	41	529	569
LbrM35_V2.1160	*L. braziliensis*	52	808	859
LbrM35_V2.1160	*L. braziliensis*	55	1110	1164
LbrM35_V2.1170	*L. braziliensis*	106	181	286
LbrM35_V2.1180	*L. braziliensis*	49	22	70
LbrM35_V2.1180	*L. braziliensis*	44	87	130
LbrM35_V2.1180	*L. braziliensis*	44	87	130
LbrM35_V2.1190	*L. braziliensis*	121	139	259
LbrM35_V2.1190	*L. braziliensis*	72	394	465
LbrM35_V2.1190	*L. braziliensis*	96	568	663
LbrM35_V2.1190	*L. braziliensis*	122	709	830
LbrM35_V2.1190	*L. braziliensis*	51	886	936

LbrM35_V2.1200	*L. braziliensis*	42	83	124
LbrM35_V2.1230	*L. braziliensis*	42	83	124
LbrM35_V2.1240	*L. braziliensis*	102	250	351
LbrM35_V2.1250	*L. braziliensis*	63	62	124
LbrM35_V2.1280	*L. braziliensis*	247	1	247
LbrM35_V2.1280	*L. braziliensis*	58	281	338
LbrM35_V2.1280	*L. braziliensis*	58	494	551
LbrM35_V2.1280	*L. braziliensis*	97	577	673
LbrM35_V2.1280	*L. braziliensis*	46	716	761
LbrM35_V2.1300	*L. braziliensis*	47	66	112
LbrM35_V2.1340	*L. braziliensis*	74	1	74
LbrM35_V2.1350	*L. braziliensis*	67	1	67
LbrM35_V2.1350	*L. braziliensis*	54	107	160
LbrM35_V2.1350	*L. braziliensis*	131	186	316
LbrM35_V2.1370	*L. braziliensis*	72	174	245
LbrM35_V2.1370	*L. braziliensis*	42	394	435
LbrM35_V2.1400	*L. braziliensis*	54	1	54
LbrM35_V2.1400	*L. braziliensis*	53	84	136
LbrM35_V2.1400	*L. braziliensis*	53	84	136
LbrM35_V2.1410	*L. braziliensis*	70	40	109
LbrM35_V2.1410	*L. braziliensis*	84	332	415
LbrM35_V2.1410	*L. braziliensis*	84	332	415
LbrM35_V2.1420	*L. braziliensis*	156	102	257
LbrM35_V2.1430	*L. braziliensis*	182	1	182
LbrM35_V2.1430	*L. braziliensis*	101	280	380
LbrM35_V2.1440	*L. braziliensis*	65	94	158
LbrM35_V2.1460	*L. braziliensis*	40	17	56
LbrM35_V2.1460	*L. braziliensis*	77	132	208
LbrM35_V2.1460	*L. braziliensis*	40	228	267
LbrM35_V2.1490	*L. braziliensis*	48	265	312
LbrM35_V2.1490	*L. braziliensis*	40	746	785
LbrM35_V2.1490	*L. braziliensis*	40	746	785
LbrM35_V2.1500	*L. braziliensis*	59	1	59
LbrM35_V2.1520	*L. braziliensis*	68	93	160
LbrM35_V2.1520	*L. braziliensis*	83	266	348
LbrM35_V2.1520	*L. braziliensis*	52	367	418
LbrM35_V2.1520	*L. braziliensis*	83	455	537
LbrM35_V2.1520	*L. braziliensis*	176	560	735
LbrM35_V2.1520	*L. braziliensis*	252	923	1174
LbrM35_V2.1540	*L. braziliensis*	97	145	241
LbrM35_V2.1540	*L. braziliensis*	191	260	450
LbrM35_V2.1550	*L. braziliensis*	56	77	132
LbrM35_V2.1550	*L. braziliensis*	47	141	187
LbrM35_V2.1550	*L. braziliensis*	99	389	487
LbrM35_V2.1560	*L. braziliensis*	74	189	262
LbrM35_V2.1560	*L. braziliensis*	70	360	429
LbrM35_V2.1560	*L. braziliensis*	70	360	429

LbrM35_V2.1570	*L. braziliensis*	75	51	125
LbrM35_V2.1580	*L. braziliensis*	52	143	194
LbrM35_V2.1580	*L. braziliensis*	49	852	900
LbrM35_V2.1580	*L. braziliensis*	63	936	998
LbrM35_V2.1580	*L. braziliensis*	51	1826	1876
LbrM35_V2.1580	*L. braziliensis*	54	1899	1952
LbrM35_V2.1580	*L. braziliensis*	72	2054	2125
LbrM35_V2.1580	*L. braziliensis*	41	2238	2278
LbrM35_V2.1580	*L. braziliensis*	90	2337	2426
LbrM35_V2.1580	*L. braziliensis*	57	3255	3311
LbrM35_V2.1580	*L. braziliensis*	59	3769	3827
LbrM35_V2.1580	*L. braziliensis*	61	4058	4118
LbrM35_V2.1580	*L. braziliensis*	58	4126	4183
LbrM35_V2.1580	*L. braziliensis*	51	4533	4583
LbrM35_V2.1580	*L. braziliensis*	41	4950	4990
LbrM35_V2.1580	*L. braziliensis*	84	5209	5292
LbrM35_V2.1600	*L. braziliensis*	320	1	320
LbrM35_V2.1600	*L. braziliensis*	217	344	560
LbrM35_V2.1600	*L. braziliensis*	58	569	626
LbrM35_V2.1610	*L. braziliensis*	79	27	105
LbrM35_V2.1610	*L. braziliensis*	108	443	550
LbrM35_V2.1610	*L. braziliensis*	116	625	740
LbrM35_V2.1610	*L. braziliensis*	84	806	889
LbrM35_V2.1610	*L. braziliensis*	112	892	1003
LbrM35_V2.1620	*L. braziliensis*	94	1	94
LbrM35_V2.1620	*L. braziliensis*	68	106	173
LbrM35_V2.1620	*L. braziliensis*	71	297	367
LbrM35_V2.1630	*L. braziliensis*	456	1	456
LbrM35_V2.1630	*L. braziliensis*	636	534	1169
LbrM35_V2.1630	*L. braziliensis*	93	1210	1302
LbrM35_V2.1640	*L. braziliensis*	125	223	347
LbrM35_V2.1640	*L. braziliensis*	41	541	581
LbrM35_V2.1640	*L. braziliensis*	62	737	798
LbrM35_V2.1640	*L. braziliensis*	100	803	902
LbrM35_V2.1650	*L. braziliensis*	46	59	104
LbrM35_V2.1670	*L. braziliensis*	51	324	374
LbrM35_V2.1680	*L. braziliensis*	167	60	226
LbrM35_V2.1680	*L. braziliensis*	125	253	377
LbrM35_V2.1680	*L. braziliensis*	81	382	462
LbrM35_V2.1680	*L. braziliensis*	44	610	653
LbrM35_V2.1690	*L. braziliensis*	60	1	60
LbrM35_V2.1690	*L. braziliensis*	65	114	178
LbrM35_V2.1690	*L. braziliensis*	82	194	275
LbrM35_V2.1690	*L. braziliensis*	50	513	562
LbrM35_V2.1690	*L. braziliensis*	84	607	690
LbrM35_V2.1690	*L. braziliensis*	121	729	849
LbrM35_V2.1700	*L. braziliensis*	105	275	379

LbrM35_V2.1710	*L. braziliensis*	330	40	369
LbrM35_V2.1710	*L. braziliensis*	41	434	474
LbrM35_V2.1720	*L. braziliensis*	130	1	130
LbrM35_V2.1720	*L. braziliensis*	72	579	650
LbrM35_V2.1720	*L. braziliensis*	275	695	969
LbrM35_V2.1720	*L. braziliensis*	41	1283	1323
LbrM35_V2.1720	*L. braziliensis*	68	1473	1540
LbrM35_V2.1730	*L. braziliensis*	94	1	94
LbrM35_V2.1730	*L. braziliensis*	146	1065	1210
LbrM35_V2.1740	*L. braziliensis*	123	1	123
LbrM35_V2.1760	*L. braziliensis*	115	1	115
LbrM35_V2.1770	*L. braziliensis*	115	1	115
LbrM35_V2.1780	*L. braziliensis*	71	1624	1694
LbrM35_V2.1790	*L. braziliensis*	135	1	135
LbrM35_V2.1810	*L. braziliensis*	184	1	184
LbrM35_V2.1810	*L. braziliensis*	284	209	492
LbrM35_V2.1820	*L. braziliensis*	94	1	94
LbrM35_V2.1820	*L. braziliensis*	69	106	174
LbrM35_V2.1820	*L. braziliensis*	74	294	367
LbrM35_V2.1830	*L. braziliensis*	276	1	276
LbrM35_V2.1850	*L. braziliensis*	311	18	328
LbrM35_V2.1850	*L. braziliensis*	68	594	661
LbrM35_V2.1860	*L. braziliensis*	40	145	184
LbrM35_V2.1860	*L. braziliensis*	48	363	410
LbrM35_V2.1860	*L. braziliensis*	48	363	410
LbrM35_V2.1870	*L. braziliensis*	67	244	310
LbrM35_V2.1870	*L. braziliensis*	53	342	394
LbrM35_V2.1870	*L. braziliensis*	79	521	599
LbrM35_V2.1870	*L. braziliensis*	94	642	735
LbrM35_V2.1880	*L. braziliensis*	57	132	188
LbrM35_V2.1890	*L. braziliensis*	510	13	522
LbrM35_V2.1900	*L. braziliensis*	74	1	74
LbrM35_V2.1910	*L. braziliensis*	42	12	53
LbrM35_V2.1910	*L. braziliensis*	52	73	124
LbrM35_V2.1910	*L. braziliensis*	41	152	192
LbrM35_V2.1910	*L. braziliensis*	45	418	462
LbrM35_V2.1910	*L. braziliensis*	76	546	621
LbrM35_V2.1910	*L. braziliensis*	106	884	989
LbrM35_V2.1910	*L. braziliensis*	59	1065	1123
LbrM35_V2.1910	*L. braziliensis*	53	1453	1505
LbrM35_V2.1910	*L. braziliensis*	77	2282	2358
LbrM35_V2.1910	*L. braziliensis*	50	2587	2636
LbrM35_V2.1910	*L. braziliensis*	74	2860	2933
LbrM35_V2.1910	*L. braziliensis*	51	3060	3110
LbrM35_V2.1910	*L. braziliensis*	50	3219	3268
LbrM35_V2.1910	*L. braziliensis*	145	3320	3464
LbrM35_V2.1910	*L. braziliensis*	108	3965	4072

LbrM35_V2.1920	*L. braziliensis*	50	54	103
LbrM35_V2.1920	*L. braziliensis*	80	246	325
LbrM35_V2.1920	*L. braziliensis*	161	820	980
LbrM35_V2.1930	*L. braziliensis*	48	1	48
LbrM35_V2.1930	*L. braziliensis*	55	76	130
LbrM35_V2.1930	*L. braziliensis*	87	290	376
LbrM35_V2.1930	*L. braziliensis*	65	630	694
LbrM35_V2.1930	*L. braziliensis*	44	1196	1239
LbrM35_V2.1970	*L. braziliensis*	121	108	228
LbrM35_V2.1970	*L. braziliensis*	46	354	399
LbrM35_V2.1970	*L. braziliensis*	117	639	755
LbrM35_V2.1980	*L. braziliensis*	196	704	899
LbrM35_V2.1990	*L. braziliensis*	63	1	63
LbrM35_V2.1990	*L. braziliensis*	174	133	306
LbrM35_V2.1990	*L. braziliensis*	174	133	306
LbrM35_V2.2000	*L. braziliensis*	43	1	43
LbrM35_V2.2000	*L. braziliensis*	60	335	394
LbrM35_V2.2010	*L. braziliensis*	124	186	309
LbrM35_V2.2010	*L. braziliensis*	215	366	580
LbrM35_V2.2010	*L. braziliensis*	296	599	894
LbrM35_V2.2020	*L. braziliensis*	325	1	325
LbrM35_V2.2020	*L. braziliensis*	186	329	514
LbrM35_V2.2020	*L. braziliensis*	78	530	607
LbrM35_V2.2030	*L. braziliensis*	152	1	152
LbrM35_V2.2030	*L. braziliensis*	50	605	654
LbrM35_V2.2040	*L. braziliensis*	52	1	52
LbrM35_V2.2040	*L. braziliensis*	105	423	527
LbrM35_V2.2050	*L. braziliensis*	48	358	405
LbrM35_V2.2060	*L. braziliensis*	134	666	799
LbrM35_V2.2070	*L. braziliensis*	40	199	238
LbrM35_V2.2070	*L. braziliensis*	54	378	431
LbrM35_V2.2090	*L. braziliensis*	71	1	71
LbrM35_V2.2090	*L. braziliensis*	47	921	967
LbrM35_V2.2090	*L. braziliensis*	320	993	1312
LbrM35_V2.2090	*L. braziliensis*	217	1359	1575
LbrM35_V2.2090	*L. braziliensis*	65	1597	1661
LbrM35_V2.2100	*L. braziliensis*	258	1	258
LbrM35_V2.2100	*L. braziliensis*	78	300	377
LbrM35_V2.2100	*L. braziliensis*	43	397	439
LbrM35_V2.2110	*L. braziliensis*	150	66	215
LbrM35_V2.2110	*L. braziliensis*	170	250	419
LbrM35_V2.2120	*L. braziliensis*	71	1	71
LbrM35_V2.2120	*L. braziliensis*	41	74	114
LbrM35_V2.2120	*L. braziliensis*	42	453	494
LbrM35_V2.2140	*L. braziliensis*	115	64	178
LbrM35_V2.2140	*L. braziliensis*	50	228	277
LbrM35_V2.2150	*L. braziliensis*	79	252	330

LbrM35_V2.2160	*L. braziliensis*	45	547	591
LbrM35_V2.2220	*L. braziliensis*	53	600	652
LbrM35_V2.2220	*L. braziliensis*	78	1033	1110
LbrM35_V2.2220	*L. braziliensis*	73	1149	1221
LbrM35_V2.2230	*L. braziliensis*	64	1	64
LbrM35_V2.2230	*L. braziliensis*	112	98	209
LbrM35_V2.2230	*L. braziliensis*	112	98	209
LbrM35_V2.2260	*L. braziliensis*	107	1	107
LbrM35_V2.2260	*L. braziliensis*	58	152	209
LbrM35_V2.2260	*L. braziliensis*	57	236	292
LbrM35_V2.2260	*L. braziliensis*	49	304	352
LbrM35_V2.2260	*L. braziliensis*	117	463	579
LbrM35_V2.2260	*L. braziliensis*	62	591	652
LbrM35_V2.2280	*L. braziliensis*	131	1	131
LbrM35_V2.2280	*L. braziliensis*	41	245	285
LbrM35_V2.2280	*L. braziliensis*	48	376	423
LbrM35_V2.2280	*L. braziliensis*	64	493	556
LbrM35_V2.2280	*L. braziliensis*	116	582	697
LbrM35_V2.2290	*L. braziliensis*	47	1	47
LbrM35_V2.2290	*L. braziliensis*	55	238	292
LbrM35_V2.2290	*L. braziliensis*	108	398	505
LbrM35_V2.2300	*L. braziliensis*	105	160	264
LbrM35_V2.2310	*L. braziliensis*	102	1	102
LbrM35_V2.2310	*L. braziliensis*	267	169	435
LbrM35_V2.2310	*L. braziliensis*	196	470	665
LbrM35_V2.2310	*L. braziliensis*	463	737	1199
LbrM35_V2.2320	*L. braziliensis*	47	213	259
LbrM35_V2.2320	*L. braziliensis*	213	384	596
LbrM35_V2.2320	*L. braziliensis*	81	603	683
LbrM35_V2.2320	*L. braziliensis*	45	732	776
LbrM35_V2.2330	*L. braziliensis*	70	69	138
LbrM35_V2.2340	*L. braziliensis*	54	69	122
LbrM35_V2.2340	*L. braziliensis*	48	225	272
LbrM35_V2.2340	*L. braziliensis*	48	225	272
LbrM35_V2.2350	*L. braziliensis*	101	38	138
LbrM35_V2.2350	*L. braziliensis*	253	140	392
LbrM35_V2.2350	*L. braziliensis*	367	429	795
LbrM35_V2.2360	*L. braziliensis*	265	1	265
LbrM35_V2.2360	*L. braziliensis*	307	316	622
LbrM35_V2.2360	*L. braziliensis*	317	643	959
LbrM35_V2.2360	*L. braziliensis*	87	987	1073
LbrM35_V2.2360	*L. braziliensis*	136	1115	1250
LbrM35_V2.2360	*L. braziliensis*	70	1334	1403
LbrM35_V2.2360	*L. braziliensis*	64	1406	1469
LbrM35_V2.2370	*L. braziliensis*	52	1	52
LbrM35_V2.2370	*L. braziliensis*	84	488	571
LbrM35_V2.2410	*L. braziliensis*	80	478	557

LbrM35_V2.2420	*L. braziliensis*	104	1	104
LbrM35_V2.2420	*L. braziliensis*	103	123	225
LbrM35_V2.2420	*L. braziliensis*	103	123	225
LbrM35_V2.2430	*L. braziliensis*	129	1	129
LbrM35_V2.2430	*L. braziliensis*	95	138	232
LbrM35_V2.2430	*L. braziliensis*	59	276	334
LbrM35_V2.2430	*L. braziliensis*	237	355	591
LbrM35_V2.2440	*L. braziliensis*	62	38	99
LbrM35_V2.2440	*L. braziliensis*	172	450	621
LbrM35_V2.2460	*L. braziliensis*	59	161	219
LbrM35_V2.2460	*L. braziliensis*	42	248	289
LbrM35_V2.2460	*L. braziliensis*	42	248	289
LbrM35_V2.2480	*L. braziliensis*	59	1	59
LbrM35_V2.2480	*L. braziliensis*	63	228	290
LbrM35_V2.2480	*L. braziliensis*	104	1106	1209
LbrM35_V2.2480	*L. braziliensis*	123	1217	1339
LbrM35_V2.2480	*L. braziliensis*	123	1431	1553
LbrM35_V2.2480	*L. braziliensis*	47	1592	1638
LbrM35_V2.2480	*L. braziliensis*	135	1666	1800
LbrM35_V2.2480	*L. braziliensis*	68	1992	2059
LbrM35_V2.2480	*L. braziliensis*	308	2233	2540
LbrM35_V2.2500	*L. braziliensis*	62	177	238
LbrM35_V2.2510	*L. braziliensis*	54	200	253
LbrM35_V2.2510	*L. braziliensis*	41	345	385
LbrM35_V2.2510	*L. braziliensis*	41	345	385
LbrM35_V2.2520	*L. braziliensis*	43	1	43
LbrM35_V2.2520	*L. braziliensis*	68	425	492
LbrM35_V2.2520	*L. braziliensis*	68	425	492
LbrM35_V2.2530	*L. braziliensis*	169	315	483
LbrM35_V2.2540	*L. braziliensis*	93	233	325
LbrM35_V2.2560	*L. braziliensis*	45	1	45
LbrM35_V2.2560	*L. braziliensis*	42	98	139
LbrM35_V2.2560	*L. braziliensis*	42	98	139
LbrM35_V2.2570	*L. braziliensis*	61	285	345
LbrM35_V2.2580	*L. braziliensis*	91	1	91
LbrM35_V2.2590	*L. braziliensis*	83	1	83
LbrM35_V2.2590	*L. braziliensis*	57	147	203
LbrM35_V2.2590	*L. braziliensis*	42	222	263
LbrM35_V2.2590	*L. braziliensis*	80	340	419
LbrM35_V2.2590	*L. braziliensis*	72	445	516
LbrM35_V2.2590	*L. braziliensis*	85	722	806
LbrM35_V2.2600	*L. braziliensis*	47	1	47
LbrM35_V2.2600	*L. braziliensis*	52	314	365
LbrM35_V2.2600	*L. braziliensis*	52	314	365
LbrM35_V2.2620	*L. braziliensis*	62	119	180
LbrM35_V2.2620	*L. braziliensis*	103	278	380
LbrM35_V2.2620	*L. braziliensis*	117	416	532

LbrM35_V2.2620	*L. braziliensis*	270	734	1003
LbrM35_V2.2620	*L. braziliensis*	46	1135	1180
LbrM35_V2.2620	*L. braziliensis*	43	1350	1392
LbrM35_V2.2620	*L. braziliensis*	62	1469	1530
LbrM35_V2.2620	*L. braziliensis*	100	1532	1631
LbrM35_V2.2620	*L. braziliensis*	73	1759	1831
LbrM35_V2.2640	*L. braziliensis*	90	30	119
LbrM35_V2.2650	*L. braziliensis*	201	17	217
LbrM35_V2.2660	*L. braziliensis*	66	216	281
LbrM35_V2.2670	*L. braziliensis*	61	267	327
LbrM35_V2.2690	*L. braziliensis*	50	88	137
LbrM35_V2.2690	*L. braziliensis*	119	156	274
LbrM35_V2.2700	*L. braziliensis*	209	1	209
LbrM35_V2.2710	*L. braziliensis*	55	307	361
LbrM35_V2.2710	*L. braziliensis*	59	419	477
LbrM35_V2.2710	*L. braziliensis*	66	574	639
LbrM35_V2.2720	*L. braziliensis*	102	1	102
LbrM35_V2.2730	*L. braziliensis*	142	107	248
LbrM35_V2.2730	*L. braziliensis*	41	310	350
LbrM35_V2.2740	*L. braziliensis*	139	1	139
LbrM35_V2.2740	*L. braziliensis*	43	441	483
LbrM35_V2.2740	*L. braziliensis*	41	677	717
LbrM35_V2.2770	*L. braziliensis*	54	528	581
LbrM35_V2.2780	*L. braziliensis*	65	270	334
LbrM35_V2.2790	*L. braziliensis*	44	253	296
LbrM35_V2.2790	*L. braziliensis*	57	302	358
LbrM35_V2.2790	*L. braziliensis*	73	367	439
LbrM35_V2.2790	*L. braziliensis*	48	728	775
LbrM35_V2.2810	*L. braziliensis*	70	132	201
LbrM35_V2.2810	*L. braziliensis*	81	345	425
LbrM35_V2.2810	*L. braziliensis*	67	536	602
LbrM35_V2.2820	*L. braziliensis*	50	1	50
LbrM35_V2.2830	*L. braziliensis*	203	1	203
LbrM35_V2.2830	*L. braziliensis*	61	580	640
LbrM35_V2.2830	*L. braziliensis*	58	715	772
LbrM35_V2.2830	*L. braziliensis*	50	777	826
LbrM35_V2.2830	*L. braziliensis*	52	1134	1185
LbrM35_V2.2830	*L. braziliensis*	85	1467	1551
LbrM35_V2.2840	*L. braziliensis*	90	1	90
LbrM35_V2.2840	*L. braziliensis*	165	106	270
LbrM35_V2.2840	*L. braziliensis*	104	303	406
LbrM35_V2.2840	*L. braziliensis*	88	463	550
LbrM35_V2.2840	*L. braziliensis*	53	628	680
LbrM35_V2.2840	*L. braziliensis*	49	724	772
LbrM35_V2.2840	*L. braziliensis*	47	780	826
LbrM35_V2.2840	*L. braziliensis*	111	843	953
LbrM35_V2.2840	*L. braziliensis*	122	999	1120

LbrM35_V2.2840	*L. braziliensis*	75	1165	1239
LbrM35_V2.2840	*L. braziliensis*	48	1474	1521
LbrM35_V2.2840	*L. braziliensis*	46	1697	1742
LbrM35_V2.2840	*L. braziliensis*	49	1981	2029
LbrM35_V2.2840	*L. braziliensis*	184	2129	2312
LbrM35_V2.2840	*L. braziliensis*	176	2341	2516
LbrM35_V2.2840	*L. braziliensis*	137	2526	2662
LbrM35_V2.2840	*L. braziliensis*	43	2698	2740
LbrM35_V2.2840	*L. braziliensis*	58	2796	2853
LbrM35_V2.2840	*L. braziliensis*	48	2906	2953
LbrM35_V2.2840	*L. braziliensis*	49	3221	3269
LbrM35_V2.2860	*L. braziliensis*	52	147	198
LbrM35_V2.2870	*L. braziliensis*	133	100	232
LbrM35_V2.2880	*L. braziliensis*	42	22	63
LbrM35_V2.2900	*L. braziliensis*	51	483	533
LbrM35_V2.2910	*L. braziliensis*	139	1	139
LbrM35_V2.2910	*L. braziliensis*	270	384	653
LbrM35_V2.2910	*L. braziliensis*	68	1375	1442
LbrM35_V2.2920	*L. braziliensis*	56	44	99
LbrM35_V2.2920	*L. braziliensis*	47	152	198
LbrM35_V2.2920	*L. braziliensis*	55	204	258
LbrM35_V2.2920	*L. braziliensis*	54	286	339
LbrM35_V2.2930	*L. braziliensis*	85	27	111
LbrM35_V2.2960	*L. braziliensis*	44	977	1020
LbrM35_V2.2980	*L. braziliensis*	56	311	366
LbrM35_V2.2990	*L. braziliensis*	613	1	613
LbrM35_V2.3010	*L. braziliensis*	52	489	540
LbrM35_V2.3010	*L. braziliensis*	56	1335	1390
LbrM35_V2.3010	*L. braziliensis*	44	2077	2120
LbrM35_V2.3010	*L. braziliensis*	60	2531	2590
LbrM35_V2.3020	*L. braziliensis*	52	26	77
LbrM35_V2.3020	*L. braziliensis*	66	108	173
LbrM35_V2.3020	*L. braziliensis*	57	195	251
LbrM35_V2.3020	*L. braziliensis*	157	256	412
LbrM35_V2.3020	*L. braziliensis*	120	496	615
LbrM35_V2.3030	*L. braziliensis*	66	93	158
LbrM35_V2.3030	*L. braziliensis*	89	337	425
LbrM35_V2.3030	*L. braziliensis*	64	522	585
LbrM35_V2.3040	*L. braziliensis*	89	309	397
LbrM35_V2.3040	*L. braziliensis*	162	437	598
LbrM35_V2.3050	*L. braziliensis*	45	57	101
LbrM35_V2.3050	*L. braziliensis*	252	148	399
LbrM35_V2.3060	*L. braziliensis*	72	42	113
LbrM35_V2.3060	*L. braziliensis*	49	123	171
LbrM35_V2.3090	*L. braziliensis*	45	93	137
LbrM35_V2.3100	*L. braziliensis*	56	1511	1566
LbrM35_V2.3110	*L. braziliensis*	88	307	394

LbrM35_V2.3120	*L. braziliensis*	50	386	435
LbrM35_V2.3120	*L. braziliensis*	70	499	568
LbrM35_V2.3120	*L. braziliensis*	43	616	658
LbrM35_V2.3120	*L. braziliensis*	127	719	845
LbrM35_V2.3130	*L. braziliensis*	77	1	77
LbrM35_V2.3130	*L. braziliensis*	49	299	347
LbrM35_V2.3150	*L. braziliensis*	191	362	552
LbrM35_V2.3160	*L. braziliensis*	60	1568	1627
LbrM35_V2.3160	*L. braziliensis*	44	2240	2283
LbrM35_V2.3160	*L. braziliensis*	48	2607	2654
LbrM35_V2.3160	*L. braziliensis*	110	2689	2798
LbrM35_V2.3160	*L. braziliensis*	42	3761	3802
LbrM35_V2.3200	*L. braziliensis*	113	124	236
LbrM35_V2.3220	*L. braziliensis*	55	228	282
LbrM35_V2.3220	*L. braziliensis*	109	650	758
LbrM35_V2.3220	*L. braziliensis*	109	650	758
LbrM35_V2.3260	*L. braziliensis*	41	120	160
LbrM35_V2.3310	*L. braziliensis*	274	245	518
LbrM35_V2.3310	*L. braziliensis*	42	770	811
LbrM35_V2.3320	*L. braziliensis*	72	1	72
LbrM35_V2.3330	*L. braziliensis*	47	198	244
LbrM35_V2.3360	*L. braziliensis*	51	93	143
LbrM35_V2.3370	*L. braziliensis*	293	130	422
LbrM35_V2.3380	*L. braziliensis*	64	1	64
LbrM35_V2.3380	*L. braziliensis*	67	81	147
LbrM35_V2.3380	*L. braziliensis*	67	81	147
LbrM35_V2.3400	*L. braziliensis*	45	82	126
LbrM35_V2.3400	*L. braziliensis*	43	450	492
LbrM35_V2.3400	*L. braziliensis*	50	1184	1233
LbrM35_V2.3410	*L. braziliensis*	48	1	48
LbrM35_V2.3410	*L. braziliensis*	56	50	105
LbrM35_V2.3410	*L. braziliensis*	71	146	216
LbrM35_V2.3410	*L. braziliensis*	73	253	325
LbrM35_V2.3420	*L. braziliensis*	67	852	918
LbrM35_V2.3440	*L. braziliensis*	469	21	489
LbrM35_V2.3460	*L. braziliensis*	40	268	307
LbrM35_V2.3470	*L. braziliensis*	106	486	591
LbrM35_V2.3480	*L. braziliensis*	78	132	209
LbrM35_V2.3490	*L. braziliensis*	64	95	158
LbrM35_V2.3510	*L. braziliensis*	40	1	40
LbrM35_V2.3510	*L. braziliensis*	62	114	175
LbrM35_V2.3510	*L. braziliensis*	49	200	248
LbrM35_V2.3510	*L. braziliensis*	65	1105	1169
LbrM35_V2.3530	*L. braziliensis*	59	1	59
LbrM35_V2.3530	*L. braziliensis*	235	61	295
LbrM35_V2.3530	*L. braziliensis*	293	440	732
LbrM35_V2.3560	*L. braziliensis*	69	187	255

LbrM35_V2.3560	*L. braziliensis*	59	263	321
LbrM35_V2.3560	*L. braziliensis*	59	263	321
LbrM35_V2.3570	*L. braziliensis*	51	318	368
LbrM35_V2.3570	*L. braziliensis*	41	507	547
LbrM35_V2.3570	*L. braziliensis*	59	1206	1264
LbrM35_V2.3580	*L. braziliensis*	41	47	87
LbrM35_V2.3660	*L. braziliensis*	48	1	48
LbrM35_V2.3660	*L. braziliensis*	74	295	368
LbrM35_V2.3660	*L. braziliensis*	63	408	470
LbrM35_V2.3660	*L. braziliensis*	64	633	696
LbrM35_V2.3670	*L. braziliensis*	209	9	217
LbrM35_V2.3680	*L. braziliensis*	41	138	178
LbrM35_V2.3710	*L. braziliensis*	162	49	210
LbrM35_V2.3720	*L. braziliensis*	116	223	338
LbrM35_V2.3730	*L. braziliensis*	145	1	145
LbrM35_V2.3730	*L. braziliensis*	210	180	389
LbrM35_V2.3730	*L. braziliensis*	47	448	494
LbrM35_V2.3740	*L. braziliensis*	42	68	109
LbrM35_V2.3740	*L. braziliensis*	54	130	183
LbrM35_V2.3740	*L. braziliensis*	54	130	183
LbrM35_V2.3750	*L. braziliensis*	66	1	66
LbrM35_V2.3770	*L. braziliensis*	58	45	102
LbrM35_V2.3770	*L. braziliensis*	46	186	231
LbrM35_V2.3780	*L. braziliensis*	330	221	550
LbrM35_V2.3780	*L. braziliensis*	433	570	1002
LbrM35_V2.3790	*L. braziliensis*	40	1	40
LbrM35_V2.3800	*L. braziliensis*	167	102	268
LbrM35_V2.3830	*L. braziliensis*	106	23	128
LbrM35_V2.3830	*L. braziliensis*	119	287	405
LbrM35_V2.3860	*L. braziliensis*	117	117	233
LbrM35_V2.3870	*L. braziliensis*	43	57	99
LbrM35_V2.3900	*L. braziliensis*	64	219	282
LbrM35_V2.3920	*L. braziliensis*	40	652	691
LbrM35_V2.3960	*L. braziliensis*	58	20	77
LbrM35_V2.3960	*L. braziliensis*	123	122	244
LbrM35_V2.3960	*L. braziliensis*	54	532	585
LbrM35_V2.3980	*L. braziliensis*	51	1	51
LbrM35_V2.3980	*L. braziliensis*	70	100	169
LbrM35_V2.4000	*L. braziliensis*	49	1	49
LbrM35_V2.4010	*L. braziliensis*	41	1	41
LbrM35_V2.4030	*L. braziliensis*	166	145	310
LbrM35_V2.4030	*L. braziliensis*	88	333	420
LbrM35_V2.4030	*L. braziliensis*	165	488	652
LbrM35_V2.4030	*L. braziliensis*	268	689	956
LbrM35_V2.4050	*L. braziliensis*	55	76	130
LbrM35_V2.4060	*L. braziliensis*	73	185	257
LbrM35_V2.4080	*L. braziliensis*	225	1	225

LbrM35_V2.4100	*L. braziliensis*	99	61	159
LbrM35_V2.4100	*L. braziliensis*	40	172	211
LbrM35_V2.4100	*L. braziliensis*	75	415	489
LbrM35_V2.4100	*L. braziliensis*	85	530	614
LbrM35_V2.4120	*L. braziliensis*	46	1	46
LbrM35_V2.4120	*L. braziliensis*	83	91	173
LbrM35_V2.4130	*L. braziliensis*	42	176	217
LbrM35_V2.4170	*L. braziliensis*	42	115	156
LbrM35_V2.4170	*L. braziliensis*	83	229	311
LbrM35_V2.4170	*L. braziliensis*	100	756	855
LbrM35_V2.4170	*L. braziliensis*	63	857	919
LbrM35_V2.4170	*L. braziliensis*	122	1331	1452
LbrM35_V2.4180	*L. braziliensis*	75	73	147
LbrM35_V2.4180	*L. braziliensis*	93	340	432
LbrM35_V2.4180	*L. braziliensis*	55	543	597
LbrM35_V2.4180	*L. braziliensis*	108	677	784
LbrM35_V2.4190	*L. braziliensis*	214	1	214
LbrM35_V2.4190	*L. braziliensis*	108	231	338
LbrM35_V2.4200	*L. braziliensis*	113	1	113
LbrM35_V2.4200	*L. braziliensis*	59	146	204
LbrM35_V2.4200	*L. braziliensis*	292	313	604
LbrM35_V2.4200	*L. braziliensis*	105	660	764
LbrM35_V2.4200	*L. braziliensis*	116	901	1016
LbrM35_V2.4210	*L. braziliensis*	140	1	140
LbrM35_V2.4210	*L. braziliensis*	58	250	307
LbrM35_V2.4210	*L. braziliensis*	45	423	467
LbrM35_V2.4210	*L. braziliensis*	78	789	866
LbrM35_V2.4210	*L. braziliensis*	79	927	1005
LbrM35_V2.4210	*L. braziliensis*	419	1093	1511
LbrM35_V2.4220	*L. braziliensis*	41	1	41
LbrM35_V2.4220	*L. braziliensis*	74	96	169
LbrM35_V2.4250	*L. braziliensis*	84	168	251
LbrM35_V2.4250	*L. braziliensis*	107	317	423
LbrM35_V2.4260	*L. braziliensis*	137	49	185
LbrM35_V2.4270	*L. braziliensis*	593	1	593
LbrM35_V2.4270	*L. braziliensis*	92	633	724
LbrM35_V2.4270	*L. braziliensis*	85	1042	1126
LbrM35_V2.4270	*L. braziliensis*	104	1348	1451
LbrM35_V2.4270	*L. braziliensis*	47	1489	1535
LbrM35_V2.4280	*L. braziliensis*	100	1	100
LbrM35_V2.4280	*L. braziliensis*	130	304	433
LbrM35_V2.4300	*L. braziliensis*	53	8	60
LbrM35_V2.4310	*L. braziliensis*	47	142	188
LbrM35_V2.4320	*L. braziliensis*	111	154	264
LbrM35_V2.4320	*L. braziliensis*	182	268	449
LbrM35_V2.4320	*L. braziliensis*	48	482	529
LbrM35_V2.4330	*L. braziliensis*	66	189	254

LbrM35_V2.4330	*L. braziliensis*	87	304	390
LbrM35_V2.4340	*L. braziliensis*	52	49	100
LbrM35_V2.4340	*L. braziliensis*	97	230	326
LbrM35_V2.4340	*L. braziliensis*	47	868	914
LbrM35_V2.4340	*L. braziliensis*	99	1251	1349
LbrM35_V2.4340	*L. braziliensis*	45	1358	1402
LbrM35_V2.4350	*L. braziliensis*	54	1	54
LbrM35_V2.4350	*L. braziliensis*	56	77	132
LbrM35_V2.4350	*L. braziliensis*	60	689	748
LbrM35_V2.4350	*L. braziliensis*	50	866	915
LbrM35_V2.4360	*L. braziliensis*	44	1	44
LbrM35_V2.4400	*L. braziliensis*	77	1	77
LbrM35_V2.4400	*L. braziliensis*	47	120	166
LbrM35_V2.4400	*L. braziliensis*	78	203	280
LbrM35_V2.4400	*L. braziliensis*	121	302	422
LbrM35_V2.4400	*L. braziliensis*	105	432	536
LbrM35_V2.4400	*L. braziliensis*	122	543	664
LbrM35_V2.4410	*L. braziliensis*	59	1	59
LbrM35_V2.4410	*L. braziliensis*	96	66	161
LbrM35_V2.4410	*L. braziliensis*	166	165	330
LbrM35_V2.4440	*L. braziliensis*	121	230	350
LbrM35_V2.4450	*L. braziliensis*	139	240	378
LbrM35_V2.4450	*L. braziliensis*	132	393	524
LbrM35_V2.4450	*L. braziliensis*	46	718	763
LbrM35_V2.4450	*L. braziliensis*	47	786	832
LbrM35_V2.4460	*L. braziliensis*	59	698	756
LbrM35_V2.4470	*L. braziliensis*	62	1	62
LbrM35_V2.4470	*L. braziliensis*	283	181	463
LbrM35_V2.4490	*L. braziliensis*	137	75	211
LbrM35_V2.4490	*L. braziliensis*	94	305	398
LbrM35_V2.4490	*L. braziliensis*	54	614	667
LbrM35_V2.4500	*L. braziliensis*	181	194	374
LbrM35_V2.4500	*L. braziliensis*	63	494	556
LbrM35_V2.4500	*L. braziliensis*	122	590	711
LbrM35_V2.4500	*L. braziliensis*	71	743	813
LbrM35_V2.4510	*L. braziliensis*	78	1	78
LbrM35_V2.4510	*L. braziliensis*	82	176	257
LbrM35_V2.4510	*L. braziliensis*	83	263	345
LbrM35_V2.4520	*L. braziliensis*	58	288	345
LbrM35_V2.4520	*L. braziliensis*	76	1231	1306
LbrM35_V2.4520	*L. braziliensis*	45	1510	1554
LbrM35_V2.4530	*L. braziliensis*	92	354	445
LbrM35_V2.4530	*L. braziliensis*	45	488	532
LbrM35_V2.4540	*L. braziliensis*	93	900	992
LbrM35_V2.4540	*L. braziliensis*	65	1170	1234
LbrM35_V2.4560	*L. braziliensis*	64	179	242
LbrM35_V2.4560	*L. braziliensis*	138	276	413

LbrM35_V2.4560	*L. braziliensis*	201	936	1136
LbrM35_V2.4560	*L. braziliensis*	71	1663	1733
LbrM35_V2.4570	*L. braziliensis*	186	1	186
LbrM35_V2.4570	*L. braziliensis*	48	971	1018
LbrM35_V2.4570	*L. braziliensis*	42	1268	1309
LbrM35_V2.4570	*L. braziliensis*	105	1528	1632
LbrM35_V2.4580	*L. braziliensis*	548	1	548
LbrM35_V2.4590	*L. braziliensis*	54	1	54
LbrM35_V2.4590	*L. braziliensis*	80	67	146
LbrM35_V2.4590	*L. braziliensis*	143	379	521
LbrM35_V2.4590	*L. braziliensis*	100	535	634
LbrM35_V2.4590	*L. braziliensis*	256	636	891
LbrM35_V2.4600	*L. braziliensis*	42	35	76
LbrM35_V2.4610	*L. braziliensis*	49	1	49
LbrM35_V2.4610	*L. braziliensis*	87	441	527
LbrM35_V2.4610	*L. braziliensis*	180	678	857
LbrM35_V2.4610	*L. braziliensis*	52	985	1036
LbrM35_V2.4610	*L. braziliensis*	175	1129	1303
LbrM35_V2.4610	*L. braziliensis*	49	1412	1460
LbrM35_V2.4610	*L. braziliensis*	81	1742	1822
LbrM35_V2.4610	*L. braziliensis*	43	1911	1953
LbrM35_V2.4620	*L. braziliensis*	50	18	67
LbrM35_V2.4620	*L. braziliensis*	77	162	238
LbrM35_V2.4620	*L. braziliensis*	75	350	424
LbrM35_V2.4630	*L. braziliensis*	54	60	113
LbrM35_V2.4630	*L. braziliensis*	61	358	418
LbrM35_V2.4630	*L. braziliensis*	44	612	655
LbrM35_V2.4640	*L. braziliensis*	111	56	166
LbrM35_V2.4640	*L. braziliensis*	53	216	268
LbrM35_V2.4640	*L. braziliensis*	165	453	617
LbrM35_V2.4640	*L. braziliensis*	45	930	974
LbrM35_V2.4650	*L. braziliensis*	185	16	200
LbrM35_V2.4710	*L. braziliensis*	119	157	275
LbrM35_V2.4720	*L. braziliensis*	117	132	248
LbrM35_V2.4720	*L. braziliensis*	137	511	647
LbrM35_V2.4730	*L. braziliensis*	47	325	371
LbrM35_V2.4750	*L. braziliensis*	53	146	198
LbrM35_V2.4760	*L. braziliensis*	76	550	625
LbrM35_V2.4770	*L. braziliensis*	99	1	99
LbrM35_V2.4790	*L. braziliensis*	66	1	66
LbrM35_V2.4800	*L. braziliensis*	58	230	287
LbrM35_V2.4810	*L. braziliensis*	83	1	83
LbrM35_V2.4810	*L. braziliensis*	240	103	342
LbrM35_V2.4810	*L. braziliensis*	240	103	342
LbrM35_V2.4820	*L. braziliensis*	80	1	80
LbrM35_V2.4830	*L. braziliensis*	99	13	111
LbrM35_V2.4830	*L. braziliensis*	72	317	388

LbrM35_V2.4830	*L. braziliensis*	72	317	388
LbrM35_V2.4840	*L. braziliensis*	83	56	138
LbrM35_V2.4840	*L. braziliensis*	57	203	259
LbrM35_V2.4840	*L. braziliensis*	73	284	356
LbrM35_V2.4840	*L. braziliensis*	54	542	595
LbrM35_V2.4840	*L. braziliensis*	66	632	697
LbrM35_V2.4860	*L. braziliensis*	46	14	59
LbrM35_V2.4860	*L. braziliensis*	62	97	158
LbrM35_V2.4860	*L. braziliensis*	40	231	270
LbrM35_V2.4860	*L. braziliensis*	92	324	415
LbrM35_V2.4870	*L. braziliensis*	44	48	91
LbrM35_V2.4870	*L. braziliensis*	56	721	776
LbrM35_V2.4870	*L. braziliensis*	56	721	776
LbrM35_V2.4890	*L. braziliensis*	187	365	551
LbrM35_V2.4910	*L. braziliensis*	40	68	107
LbrM35_V2.4910	*L. braziliensis*	120	258	377
LbrM35_V2.4910	*L. braziliensis*	84	701	784
LbrM35_V2.4910	*L. braziliensis*	86	813	898
LbrM35_V2.4920	*L. braziliensis*	95	16	110
LbrM35_V2.4940	*L. braziliensis*	44	195	238
LbrM35_V2.4940	*L. braziliensis*	777	654	1430
LbrM35_V2.4940	*L. braziliensis*	116	1459	1574
LbrM35_V2.4940	*L. braziliensis*	186	1601	1786
LbrM35_V2.4950	*L. braziliensis*	66	59	124
LbrM35_V2.4950	*L. braziliensis*	40	174	213
LbrM35_V2.4950	*L. braziliensis*	40	275	314
LbrM35_V2.4950	*L. braziliensis*	95	319	413
LbrM35_V2.4950	*L. braziliensis*	91	559	649
LbrM35_V2.4950	*L. braziliensis*	53	780	832
LbrM35_V2.4960	*L. braziliensis*	46	148	193
LbrM35_V2.4960	*L. braziliensis*	280	447	726
LbrM35_V2.4970	*L. braziliensis*	113	627	739
LbrM35_V2.4970	*L. braziliensis*	135	1307	1441
LbrM35_V2.4970	*L. braziliensis*	135	1307	1441
LbrM35_V2.4980	*L. braziliensis*	51	169	219
LbrM35_V2.4980	*L. braziliensis*	82	453	534
LbrM35_V2.4980	*L. braziliensis*	376	908	1283
LbrM35_V2.4980	*L. braziliensis*	142	1335	1476
LbrM35_V2.4990	*L. braziliensis*	48	109	156
LbrM35_V2.4990	*L. braziliensis*	65	483	547
LbrM35_V2.4990	*L. braziliensis*	102	592	693
LbrM35_V2.4990	*L. braziliensis*	137	769	905
LbrM35_V2.4990	*L. braziliensis*	302	927	1228
LbrM35_V2.4990	*L. braziliensis*	106	1293	1398
LbrM35_V2.5000	*L. braziliensis*	41	42	82
LbrM35_V2.5010	*L. braziliensis*	46	75	120
LbrM35_V2.5010	*L. braziliensis*	54	143	196

LbrM35_V2.5010	*L. braziliensis*	54	143	196
LbrM35_V2.5040	*L. braziliensis*	113	50	162
LbrM35_V2.5040	*L. braziliensis*	185	196	380
LbrM35_V2.5040	*L. braziliensis*	287	460	746
LbrM35_V2.5040	*L. braziliensis*	76	845	920
LbrM35_V2.5050	*L. braziliensis*	83	219	301
LbrM35_V2.5070	*L. braziliensis*	54	1	54
LbrM35_V2.5070	*L. braziliensis*	106	69	174
LbrM35_V2.5070	*L. braziliensis*	106	69	174
LbrM35_V2.5080	*L. braziliensis*	65	122	186
LbrM35_V2.5090	*L. braziliensis*	42	138	179
LbrM35_V2.5090	*L. braziliensis*	70	359	428
LbrM35_V2.5090	*L. braziliensis*	58	777	834
LbrM35_V2.5090	*L. braziliensis*	113	891	1003
LbrM35_V2.5100	*L. braziliensis*	96	25	120
LbrM35_V2.5100	*L. braziliensis*	84	219	302
LbrM35_V2.5100	*L. braziliensis*	119	489	607
LbrM35_V2.5130	*L. braziliensis*	103	216	318
LbrM35_V2.5150	*L. braziliensis*	122	1	122
LbrM35_V2.5150	*L. braziliensis*	171	127	297
LbrM35_V2.5150	*L. braziliensis*	40	1158	1197
LbrM35_V2.5150	*L. braziliensis*	80	1363	1442
LbrM35_V2.5150	*L. braziliensis*	54	1599	1652
LbrM35_V2.5150	*L. braziliensis*	76	1742	1817
LbrM35_V2.5150	*L. braziliensis*	47	1981	2027
LbrM35_V2.5150	*L. braziliensis*	57	2075	2131
LbrM35_V2.5160	*L. braziliensis*	360	1	360
LbrM35_V2.5170	*L. braziliensis*	94	103	196
LbrM35_V2.5170	*L. braziliensis*	222	342	563
LbrM35_V2.5170	*L. braziliensis*	40	592	631
LbrM35_V2.5200	*L. braziliensis*	117	199	315
LbrM35_V2.5200	*L. braziliensis*	69	586	654
LbrM35_V2.5210	*L. braziliensis*	168	260	427
LbrM35_V2.5220	*L. braziliensis*	63	85	147
LbrM35_V2.5220	*L. braziliensis*	79	212	290
LbrM35_V2.5220	*L. braziliensis*	55	299	353
LbrM35_V2.5230	*L. braziliensis*	108	49	156
LbrM35_V2.5250	*L. braziliensis*	50	294	343
LbrM35_V2.5270	*L. braziliensis*	242	68	309
LbrM35_V2.5270	*L. braziliensis*	51	312	362
LbrM35_V2.5270	*L. braziliensis*	228	384	611
LbrM35_V2.5270	*L. braziliensis*	173	673	845
LbrM35_V2.5280	*L. braziliensis*	317	1	317
LbrM35_V2.5310	*L. braziliensis*	43	10	52
LbrM35_V2.5310	*L. braziliensis*	98	156	253
LbrM35_V2.5320	*L. braziliensis*	44	107	150
LbrM35_V2.5330	*L. braziliensis*	115	1	115

LbrM35_V2.5330	*L. braziliensis*	62	212	273
LbrM35_V2.5330	*L. braziliensis*	67	354	420
LbrM35_V2.5340	*L. braziliensis*	64	213	276
LbrM35_V2.5350	*L. braziliensis*	255	1	255
LbrM35_V2.5350	*L. braziliensis*	154	375	528
LbrM35_V2.5350	*L. braziliensis*	65	612	676
LbrM35_V2.5360	*L. braziliensis*	42	203	244
LbrM35_V2.5390	*L. braziliensis*	217	294	510
LbrM35_V2.5400	*L. braziliensis*	260	366	625
LbrM35_V2.5410	*L. braziliensis*	70	199	268
LbrM35_V2.5410	*L. braziliensis*	76	300	375
LbrM35_V2.5440	*L. braziliensis*	227	1	227
LbrM35_V2.5440	*L. braziliensis*	73	669	741
LbrM35_V2.5440	*L. braziliensis*	87	1055	1141
LbrM35_V2.5460	*L. braziliensis*	55	1137	1191
LbrM35_V2.5490	*L. braziliensis*	79	53	131
LbrM35_V2.5490	*L. braziliensis*	75	294	368
LbrM35_V2.5500.A	*L. braziliensis*	43	53	95
LbrM35_V2.5500.A	*L. braziliensis*	41	160	200
LbrM35_V2.5500.A	*L. braziliensis*	57	267	323
LbrM35_V2.5500.A	*L. braziliensis*	158	358	515
LbrM35_V2.5500.A	*L. braziliensis*	87	555	641
LbrM35_V2.5500.A	*L. braziliensis*	724	907	1630
LbrM35_V2.5510	*L. braziliensis*	60	265	324
LbrM35_V2.5510	*L. braziliensis*	158	689	846
LbrM35_V2.5520	*L. braziliensis*	62	50	111
LbrM35_V2.5520	*L. braziliensis*	41	280	320
LbrM35_V2.5520	*L. braziliensis*	83	663	745
LbrM35_V2.5530	*L. braziliensis*	64	308	371
LbrM35_V2.5530	*L. braziliensis*	92	435	526
LbrM35_V2.5530	*L. braziliensis*	42	683	724
LbrM35_V2.5540	*L. braziliensis*	74	11	84
LbrM35_V2.5540	*L. braziliensis*	41	117	157
LbrM35_V2.5540	*L. braziliensis*	41	117	157
LbrM35_V2.5550	*L. braziliensis*	49	1	49
LbrM35_V2.5550	*L. braziliensis*	101	105	205
LbrM35_V2.5550	*L. braziliensis*	203	498	700
LbrM35_V2.5560	*L. braziliensis*	48	1	48
LbrM35_V2.5560	*L. braziliensis*	80	93	172
LbrM35_V2.5560	*L. braziliensis*	54	318	371
LbrM35_V2.5570	*L. braziliensis*	84	426	509
LbrM35_V2.5580	*L. braziliensis*	151	143	293
LbrM35_V2.5580	*L. braziliensis*	56	693	748
LbrM35_V2.5580	*L. braziliensis*	55	887	941
LbrM35_V2.5580	*L. braziliensis*	85	946	1030
LbrM35_V2.5590	*L. braziliensis*	646	1	646
LbrM35_V2.5590	*L. braziliensis*	40	684	723

LbrM35_V2.5590	*L. braziliensis*	45	752	796
LbrM35_V2.5600	*L. braziliensis*	42	1	42
LbrM35_V2.5600	*L. braziliensis*	71	339	409
LbrM35_V2.5600	*L. braziliensis*	57	452	508
LbrM35_V2.5600	*L. braziliensis*	85	614	698
LbrM35_V2.5600	*L. braziliensis*	308	713	1020
LbrM35_V2.5620	*L. braziliensis*	44	1	44
LbrM35_V2.5630	*L. braziliensis*	175	314	488
LbrM35_V2.5640	*L. braziliensis*	77	451	527
LbrM35_V2.5660	*L. braziliensis*	72	1	72
LbrM35_V2.5660	*L. braziliensis*	97	343	439
LbrM35_V2.5680	*L. braziliensis*	99	186	284
LbrM35_V2.5690	*L. braziliensis*	46	197	242
LbrM35_V2.5700	*L. braziliensis*	288	1	288
LbrM35_V2.5700	*L. braziliensis*	49	390	438
LbrM35_V2.5710	*L. braziliensis*	46	141	186
LbrM35_V2.5730	*L. braziliensis*	80	24	103
LbrM35_V2.5730	*L. braziliensis*	103	189	291
LbrM35_V2.5730	*L. braziliensis*	67	357	423
LbrM35_V2.5730	*L. braziliensis*	44	668	711
LbrM35_V2.5740	*L. braziliensis*	63	1	63
LbrM35_V2.5750	*L. braziliensis*	80	1	80
LbrM35_V2.5750	*L. braziliensis*	65	154	218
LbrM35_V2.5760	*L. braziliensis*	137	28	164
LbrM35_V2.5760	*L. braziliensis*	121	404	524
LbrM35_V2.5760	*L. braziliensis*	64	561	624
LbrM35_V2.5770	*L. braziliensis*	97	1	97
LbrM35_V2.5770	*L. braziliensis*	64	156	219
LbrM35_V2.5770	*L. braziliensis*	45	251	295
LbrM35_V2.5780	*L. braziliensis*	63	1	63
LbrM35_V2.5790	*L. braziliensis*	59	1	59
LbrM35_V2.5820	*L. braziliensis*	73	11	83
LbrM35_V2.5820	*L. braziliensis*	85	480	564
LbrM35_V2.5820	*L. braziliensis*	55	1248	1302
LbrM35_V2.5830	*L. braziliensis*	57	324	380
LbrM35_V2.5850	*L. braziliensis*	70	89	158
LbrM35_V2.5850	*L. braziliensis*	63	302	364
LbrM35_V2.5850	*L. braziliensis*	63	302	364
LbrM35_V2.5860	*L. braziliensis*	58	42	99
LbrM35_V2.5880	*L. braziliensis*	173	29	201
LbrM35_V2.5880	*L. braziliensis*	60	341	400
LbrM35_V2.5910	*L. braziliensis*	69	86	154
LbrM35_V2.5920	*L. braziliensis*	107	384	490
LbrM35_V2.5930	*L. braziliensis*	79	201	279
LbrM35_V2.5940	*L. braziliensis*	108	291	398
LbrM35_V2.5940	*L. braziliensis*	44	524	567
LbrM35_V2.5940	*L. braziliensis*	67	650	716

LbrM35_V2.5950	*L. braziliensis*	55	548	602
LbrM35_V2.5970	*L. braziliensis*	48	229	276
LbrM35_V2.5980	*L. braziliensis*	51	412	462
LbrM35_V2.5980	*L. braziliensis*	61	505	565
LbrM35_V2.5980	*L. braziliensis*	157	634	790
LbrM35_V2.6010	*L. braziliensis*	62	332	393
LbrM35_V2.6020	*L. braziliensis*	40	26	65
LbrM35_V2.6020	*L. braziliensis*	285	75	359
LbrM35_V2.6020	*L. braziliensis*	68	405	472
LbrM35_V2.6020	*L. braziliensis*	211	480	690
LbrM35_V2.6030	*L. braziliensis*	53	77	129
LbrM35_V2.6040	*L. braziliensis*	40	200	239
LbrM35_V2.6040	*L. braziliensis*	53	243	295
LbrM35_V2.6040	*L. braziliensis*	56	334	389
LbrM35_V2.6040	*L. braziliensis*	43	864	906
LbrM35_V2.6040	*L. braziliensis*	59	1073	1131
LbrM35_V2.6040	*L. braziliensis*	53	1449	1501
LbrM35_V2.6040	*L. braziliensis*	73	1625	1697
LbrM35_V2.6060	*L. braziliensis*	64	153	216
LbrM35_V2.6070	*L. braziliensis*	54	215	268
LbrM35_V2.6080	*L. braziliensis*	50	26	75
LbrM35_V2.6080	*L. braziliensis*	47	297	343
LbrM35_V2.6080	*L. braziliensis*	77	787	863
LbrM35_V2.6080	*L. braziliensis*	48	1090	1137
LbrM35_V2.6090	*L. braziliensis*	90	204	293
LbrM35_V2.6090	*L. braziliensis*	67	354	420
LbrM35_V2.6100	*L. braziliensis*	267	69	335
LbrM35_V2.6100	*L. braziliensis*	56	380	435
LbrM35_V2.6110	*L. braziliensis*	61	1	61
LbrM35_V2.6120	*L. braziliensis*	43	30	72
LbrM35_V2.6120	*L. braziliensis*	255	91	345
LbrM35_V2.6130	*L. braziliensis*	77	34	110
LbrM35_V2.6150	*L. braziliensis*	159	1	159
LbrM35_V2.6150	*L. braziliensis*	69	201	269
LbrM35_V2.6150	*L. braziliensis*	228	278	505
LbrM35_V2.6160	*L. braziliensis*	77	47	123
LbrM35_V2.6170	*L. braziliensis*	60	220	279
LbrM35_V2.6170	*L. braziliensis*	67	593	659
LbrM35_V2.6170	*L. braziliensis*	67	593	659
LbrM35_V2.6190	*L. braziliensis*	64	62	125
LbrM35_V2.6190	*L. braziliensis*	107	421	527
LbrM35_V2.6190	*L. braziliensis*	59	1120	1178
LbrM35_V2.6190	*L. braziliensis*	57	1258	1314
LbrM35_V2.6190	*L. braziliensis*	49	1843	1891
LbrM35_V2.6210	*L. braziliensis*	49	32	80
LbrM35_V2.6210	*L. braziliensis*	50	117	166
LbrM35_V2.6220	*L. braziliensis*	56	92	147

LbrM35_V2.6230	*L. braziliensis*	292	1	292
LbrM35_V2.6240	*L. braziliensis*	75	61	135
LbrM35_V2.6260	*L. braziliensis*	55	1	55
LbrM35_V2.6260	*L. braziliensis*	58	92	149
LbrM35_V2.6260	*L. braziliensis*	107	223	329
LbrM35_V2.6270	*L. braziliensis*	50	1	50
LbrM35_V2.6280	*L. braziliensis*	46	375	420
LbrM35_V2.6280	*L. braziliensis*	213	424	636
LbrM35_V2.6290	*L. braziliensis*	156	1	156
LbrM35_V2.6290	*L. braziliensis*	51	491	541
LbrM35_V2.6300	*L. braziliensis*	95	157	251
LbrM35_V2.6300	*L. braziliensis*	292	385	676
LbrM35_V2.6310	*L. braziliensis*	59	1	59
LbrM35_V2.6310	*L. braziliensis*	89	78	166
LbrM35_V2.6310	*L. braziliensis*	50	197	246
LbrM35_V2.6310	*L. braziliensis*	234	263	496
LbrM35_V2.6310	*L. braziliensis*	84	658	741
LbrM35_V2.6310	*L. braziliensis*	61	764	824
LbrM35_V2.6320	*L. braziliensis*	185	43	227
LbrM35_V2.6320	*L. braziliensis*	97	257	353
LbrM35_V2.6320	*L. braziliensis*	151	388	538
LbrM35_V2.6340	*L. braziliensis*	84	560	643
LbrM35_V2.6340	*L. braziliensis*	116	860	975
LbrM35_V2.6340	*L. braziliensis*	57	1014	1070
LbrM35_V2.6340	*L. braziliensis*	56	1107	1162
LbrM35_V2.6340	*L. braziliensis*	84	1299	1382
LbrM35_V2.6340	*L. braziliensis*	119	1444	1562
LbrM35_V2.6340	*L. braziliensis*	64	1663	1726
LbrM35_V2.6350	*L. braziliensis*	69	1	69
LbrM35_V2.6350	*L. braziliensis*	45	91	135
LbrM35_V2.6350	*L. braziliensis*	40	275	314
LbrM35_V2.6350	*L. braziliensis*	50	370	419
LbrM35_V2.6350	*L. braziliensis*	59	865	923
LbrM35_V2.6370	*L. braziliensis*	40	156	195
LbrM35_V2.6370	*L. braziliensis*	183	335	517
LbrM35_V2.6380	*L. braziliensis*	168	1	168
LbrM35_V2.6390	*L. braziliensis*	69	38	106
LbrM35_V2.6430	*L. braziliensis*	53	270	322
LbrM35_V2.6440	*L. braziliensis*	361	12	372
LbrM35_V2.6440	*L. braziliensis*	52	411	462
LbrM35_V2.6440	*L. braziliensis*	91	475	565
LbrM35_V2.6440	*L. braziliensis*	92	576	667
LbrM35_V2.6450	*L. braziliensis*	81	118	198
LbrM35_V2.6450	*L. braziliensis*	507	273	779
LbrM35_V2.6460	*L. braziliensis*	183	110	292
LbrM35_V2.6470	*L. braziliensis*	212	1	212
LbrM35_V2.6520	*L. braziliensis*	43	1	43

LbrM35_V2.6530	*L. braziliensis*	40	831	870
LbrM35_V2.6530	*L. braziliensis*	40	1125	1164
LbrM35_V2.6530	*L. braziliensis*	40	1125	1164
LbrM35_V2.6540	*L. braziliensis*	51	50	100
LbrM35_V2.6540	*L. braziliensis*	119	137	255
LbrM35_V2.6540	*L. braziliensis*	49	264	312
LbrM35_V2.6540	*L. braziliensis*	65	322	386
LbrM35_V2.6540	*L. braziliensis*	445	459	903
LbrM35_V2.6540	*L. braziliensis*	174	918	1091
LbrM35_V2.6540	*L. braziliensis*	333	1168	1500
LbrM35_V2.6560	*L. braziliensis*	55	1	55
LbrM35_V2.6560	*L. braziliensis*	334	301	634
LbrM35_V2.6560	*L. braziliensis*	85	650	734
LbrM35_V2.6570	*L. braziliensis*	133	167	299
LbrM35_V2.6590	*L. braziliensis*	83	30	112
LbrM35_V2.6590	*L. braziliensis*	44	123	166
LbrM35_V2.6590	*L. braziliensis*	44	123	166
LbrM35_V2.6620	*L. braziliensis*	40	1	40
LbrM35_V2.6620	*L. braziliensis*	112	2410	2521
LbrM35_V2.6620	*L. braziliensis*	112	2410	2521
LbrM35_V2.6640	*L. braziliensis*	45	1299	1343
LbrM35_V2.6640	*L. braziliensis*	46	1882	1927
LbrM35_V2.6640	*L. braziliensis*	44	2235	2278
LbrM35_V2.6640	*L. braziliensis*	87	2282	2368
LbrM35_V2.6640	*L. braziliensis*	41	2622	2662
LbrM35_V2.6640	*L. braziliensis*	60	2999	3058
LbrM35_V2.6640	*L. braziliensis*	45	3132	3176
LbrM35_V2.6640	*L. braziliensis*	63	3216	3278
LbrM35_V2.6670	*L. braziliensis*	95	44	138
LbrM35_V2.6690	*L. braziliensis*	46	1	46
LbrM35_V2.6690	*L. braziliensis*	62	205	266
LbrM35_V2.6690	*L. braziliensis*	62	205	266
LbrM35_V2.6700	*L. braziliensis*	205	74	278
LbrM35_V2.6710	*L. braziliensis*	61	357	417
LbrM35_V2.6730	*L. braziliensis*	168	1	168
LbrM35_V2.6740	*L. braziliensis*	322	192	513
LbrM35_V2.6750	*L. braziliensis*	57	149	205
LbrM35_V2.6760	*L. braziliensis*	102	15	116
LbrM35_V2.6770	*L. braziliensis*	70	242	311
LbrM35_V2.6800	*L. braziliensis*	388	53	440
LbrM35_V2.6820	*L. braziliensis*	62	429	490
LbrM35_V2.6830	*L. braziliensis*	139	187	325
LbrM35_V2.6840	*L. braziliensis*	157	114	270
LbrM35_V2.6840	*L. braziliensis*	57	524	580
LbrM35_V2.6850	*L. braziliensis*	49	19	67
LbrM35_V2.6860	*L. braziliensis*	132	1	132
LbrM35_V2.6860	*L. braziliensis*	58	210	267

LbrM35_V2.6860	*L. braziliensis*	335	366	700
LbrM35_V2.6880	*L. braziliensis*	143	11	153
LbrM35_V2.6880	*L. braziliensis*	110	454	563
LbrM35_V2.6890	*L. braziliensis*	131	47	177
LbrM35_V2.6890	*L. braziliensis*	176	219	394
LbrM35_V2.6920	*L. braziliensis*	91	93	183
LbrM35_V2.6920	*L. braziliensis*	79	218	296
LbrM35_V2.6930	*L. braziliensis*	299	64	362
LbrM35_V2.6950	*L. braziliensis*	111	1	111
LbrM35_V2.6950	*L. braziliensis*	43	144	186
LbrM35_V2.6950	*L. braziliensis*	88	195	282
LbrM35_V2.6950	*L. braziliensis*	71	297	367
LbrM35_V2.6950	*L. braziliensis*	41	406	446
LbrM35_V2.6950	*L. braziliensis*	41	526	566
LbrM35_V2.6960	*L. braziliensis*	46	691	736
LbrM35_V2.6970	*L. braziliensis*	45	1	45
LbrM35_V2.6990	*L. braziliensis*	45	83	127
LbrM35_V2.7020	*L. braziliensis*	51	348	398
LbrM35_V2.7040	*L. braziliensis*	102	37	138
LbrM35_V2.7080	*L. braziliensis*	76	391	466
LbrM35_V2.7080	*L. braziliensis*	68	828	895
LbrM35_V2.7120	*L. braziliensis*	143	604	746
LbrM35_V2.7130	*L. braziliensis*	46	247	292
LbrM35_V2.7150	*L. braziliensis*	50	432	481
LbrM35_V2.7160	*L. braziliensis*	56	266	321
LbrM35_V2.7200	*L. braziliensis*	41	1	41
LbrM35_V2.7200	*L. braziliensis*	70	81	150
LbrM35_V2.7200	*L. braziliensis*	70	81	150
LbrM35_V2.7220	*L. braziliensis*	65	569	633
LbrM35_V2.7220	*L. braziliensis*	54	639	692
LbrM35_V2.7220	*L. braziliensis*	54	639	692
LbrM35_V2.7230	*L. braziliensis*	110	1	110
LbrM35_V2.7250	*L. braziliensis*	116	171	286
LbrM35_V2.7270	*L. braziliensis*	163	375	537
LbrM35_V2.7270	*L. braziliensis*	171	556	726
LbrM35_V2.7300	*L. braziliensis*	73	81	153
LbrM35_V2.7300	*L. braziliensis*	275	235	509
LbrM35_V2.7320	*L. braziliensis*	53	121	173
LbrM35_V2.7340	*L. braziliensis*	209	1	209
LbrM35_V2.7340	*L. braziliensis*	45	280	324
LbrM35_V2.7360	*L. braziliensis*	55	1	55
LbrM35_V2.7360	*L. braziliensis*	60	127	186
LbrM35_V2.7360	*L. braziliensis*	43	689	731
LmjF01.0010	*L. major*	44	289	332
LmjF01.0020	*L. major*	68	23	90
LmjF01.0020	*L. major*	57	413	469
LmjF01.0030	*L. major*	65	70	134

LmjF01.0030	*L. major*	43	314	356
LmjF01.0030	*L. major*	131	449	579
LmjF01.0040	*L. major*	68	1	68
LmjF01.0060	*L. major*	112	138	249
LmjF01.0070	*L. major*	72	75	146
LmjF01.0070	*L. major*	60	239	298
LmjF01.0070	*L. major*	98	501	598
LmjF01.0110	*L. major*	66	27	92
LmjF01.0120	*L. major*	337	96	432
LmjF01.0130	*L. major*	52	1	52
LmjF01.0130	*L. major*	187	178	364
LmjF01.0150	*L. major*	41	63	103
LmjF01.0170	*L. major*	223	1	223
LmjF01.0170	*L. major*	40	304	343
LmjF01.0170	*L. major*	110	349	458
LmjF01.0180	*L. major*	46	727	772
LmjF01.0190	*L. major*	48	31	78
LmjF01.0190	*L. major*	83	114	196
LmjF01.0200	*L. major*	92	94	185
LmjF01.0210	*L. major*	105	1	105
LmjF01.0210	*L. major*	110	238	347
LmjF01.0210	*L. major*	108	373	480
LmjF01.0210	*L. major*	59	670	728
LmjF01.0220	*L. major*	48	57	104
LmjF01.0240	*L. major*	123	1	123
LmjF01.0240	*L. major*	41	389	429
LmjF01.0240	*L. major*	215	711	925
LmjF01.0250	*L. major*	187	1	187
LmjF01.0250	*L. major*	303	212	514
LmjF01.0260	*L. major*	281	1	281
LmjF01.0280	*L. major*	42	13	54
LmjF01.0280	*L. major*	40	126	165
LmjF01.0280	*L. major*	40	126	165
LmjF01.0290	*L. major*	54	1	54
LmjF01.0340	*L. major*	134	1	134
LmjF01.0350	*L. major*	219	374	592
LmjF01.0350	*L. major*	132	603	734
LmjF01.0360	*L. major*	114	1	114
LmjF01.0370	*L. major*	41	148	188
LmjF01.0380	*L. major*	98	661	758
LmjF01.0390	*L. major*	107	1	107
LmjF01.0390	*L. major*	96	208	303
LmjF01.0390	*L. major*	80	490	569
LmjF01.0390	*L. major*	77	746	822
LmjF01.0400	*L. major*	55	83	137
LmjF01.0400	*L. major*	143	225	367
LmjF01.0400	*L. major*	143	225	367

LmjF01.0410	*L. major*	41	1	41
LmjF01.0420	*L. major*	41	1	41
LmjF01.0430	*L. major*	64	61	124
LmjF01.0430	*L. major*	134	237	370
LmjF01.0430	*L. major*	140	492	631
LmjF01.0430	*L. major*	60	684	743
LmjF01.0460	*L. major*	340	1	340
LmjF01.0460	*L. major*	46	774	819
LmjF01.0460	*L. major*	215	924	1138
LmjF01.0460	*L. major*	265	1162	1426
LmjF01.0510	*L. major*	84	63	146
LmjF01.0510	*L. major*	41	154	194
LmjF01.0530	*L. major*	269	29	297
LmjF01.0530	*L. major*	68	399	466
LmjF01.0530	*L. major*	72	503	574
LmjF01.0530	*L. major*	392	654	1045
LmjF01.0530	*L. major*	68	1083	1150
LmjF01.0530	*L. major*	51	1487	1537
LmjF01.0540	*L. major*	126	1	126
LmjF01.0540	*L. major*	60	173	232
LmjF01.0540	*L. major*	120	313	432
LmjF01.0540	*L. major*	182	437	618
LmjF01.0550	*L. major*	83	88	170
LmjF01.0550	*L. major*	90	200	289
LmjF01.0560	*L. major*	235	1	235
LmjF01.0560	*L. major*	73	309	381
LmjF01.0580	*L. major*	136	1	136
LmjF01.0580	*L. major*	78	289	366
LmjF01.0580	*L. major*	40	866	905
LmjF01.0600	*L. major*	45	1	45
LmjF01.0600	*L. major*	145	86	230
LmjF01.0600	*L. major*	41	449	489
LmjF01.0600	*L. major*	166	739	904
LmjF01.0600	*L. major*	48	1493	1540
LmjF01.0600	*L. major*	97	1614	1710
LmjF01.0610	*L. major*	44	165	208
LmjF01.0620	*L. major*	55	1	55
LmjF01.0620	*L. major*	51	134	184
LmjF01.0620	*L. major*	144	246	389
LmjF01.0620	*L. major*	174	418	591
LmjF01.0630	*L. major*	57	1	57
LmjF01.0630	*L. major*	381	186	566
LmjF01.0630	*L. major*	50	576	625
LmjF01.0640	*L. major*	41	1	41
LmjF01.0640	*L. major*	98	119	216
LmjF01.0640	*L. major*	74	324	397
LmjF01.0640	*L. major*	43	528	570

LmjF01.0640	*L. major*	89	886	974
LmjF01.0660	*L. major*	245	1	245
LmjF01.0660	*L. major*	74	321	394
LmjF01.0660	*L. major*	77	453	529
LmjF01.0660	*L. major*	121	653	773
LmjF01.0660	*L. major*	269	828	1096
LmjF01.0660	*L. major*	61	1098	1158
LmjF01.0660	*L. major*	49	1182	1230
LmjF01.0660	*L. major*	54	1318	1371
LmjF01.0670	*L. major*	283	23	305
LmjF01.0680	*L. major*	167	1	167
LmjF01.0680	*L. major*	118	189	306
LmjF01.0680	*L. major*	145	321	465
LmjF01.0680	*L. major*	302	499	800
LmjF01.0700	*L. major*	58	1	58
LmjF01.0700	*L. major*	92	163	254
LmjF01.0710	*L. major*	50	270	319
LmjF01.0720	*L. major*	261	1	261
LmjF01.0720	*L. major*	67	342	408
LmjF01.0720	*L. major*	48	731	778
LmjF01.0720	*L. major*	52	808	859
LmjF01.0720	*L. major*	77	1130	1206
LmjF01.0720	*L. major*	46	1426	1471
LmjF01.0720	*L. major*	66	1512	1577
LmjF01.0730	*L. major*	205	26	230
LmjF01.0730	*L. major*	124	310	433
LmjF01.0730	*L. major*	76	633	708
LmjF01.0730	*L. major*	56	833	888
LmjF01.0730	*L. major*	91	909	999
LmjF01.0730	*L. major*	78	1229	1306
LmjF01.0730	*L. major*	47	1324	1370
LmjF01.0730	*L. major*	100	1699	1798
LmjF01.0730	*L. major*	103	2085	2187
LmjF01.0740	*L. major*	152	112	263
LmjF01.0740	*L. major*	184	381	564
LmjF01.0750	*L. major*	64	1	64
LmjF01.0750	*L. major*	288	103	390
LmjF01.0750	*L. major*	94	488	581
LmjF01.0750	*L. major*	76	673	748
LmjF01.0750	*L. major*	60	755	814
LmjF01.0750	*L. major*	48	894	941
LmjF01.0750	*L. major*	103	967	1069
LmjF01.0750	*L. major*	43	1143	1185
LmjF01.0750	*L. major*	66	1270	1335
LmjF01.0760	*L. major*	54	182	235
LmjF01.0790	*L. major*	161	1	161
LmjF01.0790	*L. major*	210	181	390

LmjF01.0790	*L. major*	94	395	488
LmjF01.0800	*L. major*	178	44	221
LmjF01.0800	*L. major*	109	401	509
LmjF01.0810	*L. major*	176	551	726
LmjF01.0820	*L. major*	46	1	46
LmjF01.0820	*L. major*	138	687	824
LmjF01.0830	*L. major*	54	1	54
LmjF01.0830	*L. major*	45	139	183
LmjF01.0830	*L. major*	40	280	319
LmjF02.0010	*L. major*	79	1	79
LmjF02.0010	*L. major*	45	145	189
LmjF02.0020	*L. major*	49	1	49
LmjF02.0060	*L. major*	41	82	122
LmjF02.0070	*L. major*	63	335	397
LmjF02.0080	*L. major*	53	1	53
LmjF02.0080	*L. major*	44	780	823
LmjF02.0080	*L. major*	60	937	996
LmjF02.0085	*L. major*	140	1	140
LmjF02.0090	*L. major*	153	1	153
LmjF02.0090	*L. major*	52	301	352
LmjF02.0110	*L. major*	121	716	836
LmjF02.0120	*L. major*	144	108	251
LmjF02.0120	*L. major*	138	641	778
LmjF02.0120	*L. major*	46	922	967
LmjF02.0120	*L. major*	71	1415	1485
LmjF02.0120	*L. major*	70	2055	2124
LmjF02.0120	*L. major*	51	2167	2217
LmjF02.0120	*L. major*	77	2616	2692
LmjF02.0120	*L. major*	67	2700	2766
LmjF02.0120	*L. major*	63	2930	2992
LmjF02.0120	*L. major*	67	3076	3142
LmjF02.0120	*L. major*	46	3290	3335
LmjF02.0120	*L. major*	219	3522	3740
LmjF02.0120	*L. major*	55	3875	3929
LmjF02.0120	*L. major*	125	4106	4230
LmjF02.0120	*L. major*	44	4822	4865
LmjF02.0130	*L. major*	65	55	119
LmjF02.0140	*L. major*	188	123	310
LmjF02.0140	*L. major*	52	367	418
LmjF02.0140	*L. major*	70	766	835
LmjF02.0150	*L. major*	310	73	382
LmjF02.0150	*L. major*	115	573	687
LmjF02.0150	*L. major*	116	751	866
LmjF02.0150	*L. major*	42	958	999
LmjF02.0150	*L. major*	41	1052	1092
LmjF02.0150	*L. major*	156	1106	1261
LmjF02.0150	*L. major*	58	1447	1504

LmjF02.0150	*L. major*	166	1751	1916
LmjF02.0160	*L. major*	57	32	88
LmjF02.0160	*L. major*	46	345	390
LmjF02.0160	*L. major*	46	345	390
LmjF02.0170	*L. major*	46	373	418
LmjF02.0180	*L. major*	66	1	66
LmjF02.0190	*L. major*	223	211	433
LmjF02.0190	*L. major*	47	530	576
LmjF02.0190	*L. major*	53	750	802
LmjF02.0190	*L. major*	49	921	969
LmjF02.0210	*L. major*	46	373	418
LmjF02.0210	*L. major*	42	587	628
LmjF02.0210	*L. major*	42	587	628
LmjF02.0220	*L. major*	66	1	66
LmjF02.0230	*L. major*	224	21	244
LmjF02.0230	*L. major*	47	341	387
LmjF02.0230	*L. major*	53	561	613
LmjF02.0230	*L. major*	49	732	780
LmjF02.0240	*L. major*	59	102	160
LmjF02.0240	*L. major*	53	234	286
LmjF02.0240	*L. major*	42	336	377
LmjF02.0250	*L. major*	56	1	56
LmjF02.0250	*L. major*	51	616	666
LmjF02.0250	*L. major*	45	669	713
LmjF02.0250	*L. major*	43	857	899
LmjF02.0250	*L. major*	40	996	1035
LmjF02.0250	*L. major*	46	1062	1107
LmjF02.0260	*L. major*	89	186	274
LmjF02.0280	*L. major*	96	74	169
LmjF02.0290	*L. major*	54	10	63
LmjF02.0290	*L. major*	48	456	503
LmjF02.0290	*L. major*	48	456	503
LmjF02.0300	*L. major*	60	1	60
LmjF02.0300	*L. major*	96	75	170
LmjF02.0300	*L. major*	83	866	948
LmjF02.0300	*L. major*	197	1275	1471
LmjF02.0300	*L. major*	85	1919	2003
LmjF02.0300	*L. major*	44	2347	2390
LmjF02.0310	*L. major*	144	1	144
LmjF02.0310	*L. major*	63	212	274
LmjF02.0310	*L. major*	110	402	511
LmjF02.0320	*L. major*	87	168	254
LmjF02.0330	*L. major*	187	1	187
LmjF02.0330	*L. major*	53	475	527
LmjF02.0330	*L. major*	44	1128	1171
LmjF02.0330	*L. major*	59	1305	1363
LmjF02.0330	*L. major*	82	1393	1474

LmjF02.0340	*L. major*	47	1	47
LmjF02.0340	*L. major*	43	106	148
LmjF02.0340	*L. major*	64	161	224
LmjF02.0340	*L. major*	138	293	430
LmjF02.0340	*L. major*	42	543	584
LmjF02.0340	*L. major*	86	679	764
LmjF02.0340	*L. major*	56	834	889
LmjF02.0350	*L. major*	310	1	310
LmjF02.0350	*L. major*	49	339	387
LmjF02.0350	*L. major*	52	412	463
LmjF02.0350	*L. major*	65	688	752
LmjF02.0350	*L. major*	109	885	993
LmjF02.0370	*L. major*	55	473	527
LmjF02.0380	*L. major*	43	123	165
LmjF02.0380	*L. major*	64	262	325
LmjF02.0380	*L. major*	72	388	459
LmjF02.0410	*L. major*	56	1	56
LmjF02.0410	*L. major*	197	94	290
LmjF02.0410	*L. major*	112	300	411
LmjF02.0410	*L. major*	84	438	521
LmjF02.0410	*L. major*	42	550	591
LmjF02.0410	*L. major*	74	618	691
LmjF02.0410	*L. major*	66	788	853
LmjF02.0420	*L. major*	94	201	294
LmjF02.0420	*L. major*	73	395	467
LmjF02.0420	*L. major*	156	513	668
LmjF02.0420	*L. major*	59	942	1000
LmjF02.0430	*L. major*	41	88	128
LmjF02.0470	*L. major*	165	1	165
LmjF02.0470	*L. major*	396	210	605
LmjF02.0470	*L. major*	71	628	698
LmjF02.0470	*L. major*	630	755	1384
LmjF02.0470	*L. major*	710	1414	2123
LmjF02.0470	*L. major*	183	2135	2317
LmjF02.0470	*L. major*	478	2367	2844
LmjF02.0470	*L. major*	158	2848	3005
LmjF02.0470	*L. major*	123	3033	3155
LmjF02.0480	*L. major*	187	1	187
LmjF02.0490	*L. major*	149	79	227
LmjF02.0490	*L. major*	75	249	323
LmjF02.0490	*L. major*	87	388	474
LmjF02.0490	*L. major*	43	502	544
LmjF02.0490	*L. major*	105	554	658
LmjF02.0500	*L. major*	99	1	99
LmjF02.0500	*L. major*	54	137	190
LmjF02.0500	*L. major*	64	511	574
LmjF02.0500	*L. major*	50	670	719

LmjF02.0510	*L. major*	170	1	170
LmjF02.0520	*L. major*	154	1	154
LmjF02.0520	*L. major*	951	194	1144
LmjF02.0520	*L. major*	598	1153	1750
LmjF02.0520	*L. major*	123	1789	1911
LmjF02.0530	*L. major*	131	61	191
LmjF02.0530	*L. major*	107	223	329
LmjF02.0530	*L. major*	403	365	767
LmjF02.0540	*L. major*	50	210	259
LmjF02.0550	*L. major*	76	30	105
LmjF02.0560	*L. major*	59	1	59
LmjF02.0560	*L. major*	95	323	417
LmjF02.0560	*L. major*	83	440	522
LmjF02.0560	*L. major*	298	552	849
LmjF02.0570	*L. major*	297	1	297
LmjF02.0570	*L. major*	92	329	420
LmjF02.0570	*L. major*	69	642	710
LmjF02.0570	*L. major*	164	714	877
LmjF02.0570	*L. major*	41	1002	1042
LmjF02.0570	*L. major*	53	1070	1122
LmjF02.0570	*L. major*	70	1355	1424
LmjF02.0580	*L. major*	55	826	880
LmjF02.0580	*L. major*	88	926	1013
LmjF02.0580	*L. major*	102	1101	1202
LmjF02.0580	*L. major*	51	1626	1676
LmjF02.0580	*L. major*	99	1779	1877
LmjF02.0580	*L. major*	179	2049	2227
LmjF02.0580	*L. major*	51	2239	2289
LmjF02.0580	*L. major*	56	2434	2489
LmjF02.0580	*L. major*	154	2567	2720
LmjF02.0580	*L. major*	112	2955	3066
LmjF02.0580	*L. major*	59	3154	3212
LmjF02.0580	*L. major*	54	3329	3382
LmjF02.0580	*L. major*	275	3411	3685
LmjF02.0590	*L. major*	138	1	138
LmjF02.0590	*L. major*	89	258	346
LmjF02.0590	*L. major*	138	378	515
LmjF02.0610	*L. major*	69	1	69
LmjF02.0610	*L. major*	191	106	296
LmjF02.0620	*L. major*	140	1	140
LmjF02.0630	*L. major*	201	42	242
LmjF02.0630	*L. major*	50	287	336
LmjF02.0640	*L. major*	64	112	175
LmjF02.0640	*L. major*	150	274	423
LmjF02.0640	*L. major*	121	516	636
LmjF02.0640	*L. major*	42	1100	1141
LmjF02.0640	*L. major*	49	1207	1255

LmjF02.0640	*L. major*	42	1355	1396
LmjF02.0650	*L. major*	49	1	49
LmjF02.0650	*L. major*	86	122	207
LmjF02.0650	*L. major*	41	246	286
LmjF02.0660	*L. major*	251	1	251
LmjF02.0660	*L. major*	278	261	538
LmjF02.0660	*L. major*	144	787	930
LmjF02.0690	*L. major*	134	1	134
LmjF02.0690	*L. major*	101	150	250
LmjF02.0690	*L. major*	77	346	422
LmjF02.0700	*L. major*	54	1	54
LmjF02.0700	*L. major*	162	116	277
LmjF02.0700	*L. major*	62	280	341
LmjF02.0700	*L. major*	75	350	424
LmjF02.0700	*L. major*	120	595	714
LmjF02.0700	*L. major*	157	781	937
LmjF02.0710	*L. major*	102	1	102
LmjF02.0710	*L. major*	88	335	422
LmjF02.0710	*L. major*	88	335	422
LmjF02.0740	*L. major*	53	1	53
LmjF03.0020	*L. major*	41	68	108
LmjF03.0070	*L. major*	43	107	149
LmjF03.0090	*L. major*	49	86	134
LmjF03.0090	*L. major*	40	528	567
LmjF03.0090	*L. major*	40	528	567
LmjF03.0110	*L. major*	57	16	72
LmjF03.0110	*L. major*	347	237	583
LmjF03.0110	*L. major*	92	660	751
LmjF03.0110	*L. major*	74	774	847
LmjF03.0110	*L. major*	62	894	955
LmjF03.0120	*L. major*	487	203	689
LmjF03.0120	*L. major*	47	717	763
LmjF03.0120	*L. major*	115	777	891
LmjF03.0140	*L. major*	95	45	139
LmjF03.0160	*L. major*	89	90	178
LmjF03.0160	*L. major*	42	438	479
LmjF03.0160	*L. major*	42	438	479
LmjF03.0170	*L. major*	57	43	99
LmjF03.0170	*L. major*	74	119	192
LmjF03.0180	*L. major*	246	31	276
LmjF03.0180	*L. major*	46	419	464
LmjF03.0180	*L. major*	46	419	464
LmjF03.0190	*L. major*	50	161	210
LmjF03.0220	*L. major*	45	45	89
LmjF03.0230	*L. major*	67	21	87
LmjF03.0260	*L. major*	57	34	90
LmjF03.0260	*L. major*	61	125	185

LmjF03.0260	*L. major*	61	125	185
LmjF03.0270	*L. major*	2170	82	2251
LmjF03.0270	*L. major*	69	2286	2354
LmjF03.0280	*L. major*	65	45	109
LmjF03.0280	*L. major*	48	227	274
LmjF03.0280	*L. major*	150	291	440
LmjF03.0280	*L. major*	49	497	545
LmjF03.0280	*L. major*	84	623	706
LmjF03.0280	*L. major*	100	778	877
LmjF03.0310	*L. major*	105	1	105
LmjF03.0310	*L. major*	693	143	835
LmjF03.0310	*L. major*	1066	851	1916
LmjF03.0310	*L. major*	151	1937	2087
LmjF03.0320	*L. major*	46	1	46
LmjF03.0320	*L. major*	114	61	174
LmjF03.0320	*L. major*	451	182	632
LmjF03.0320	*L. major*	269	764	1032
LmjF03.0330	*L. major*	41	1	41
LmjF03.0330	*L. major*	83	192	274
LmjF03.0330	*L. major*	90	312	401
LmjF03.0330	*L. major*	45	586	630
LmjF03.0340	*L. major*	232	66	297
LmjF03.0350	*L. major*	355	1	355
LmjF03.0350	*L. major*	63	421	483
LmjF03.0350	*L. major*	278	529	806
LmjF03.0350	*L. major*	51	983	1033
LmjF03.0350	*L. major*	73	1056	1128
LmjF03.0350	*L. major*	90	1159	1248
LmjF03.0350	*L. major*	208	1320	1527
LmjF03.0360	*L. major*	313	1	313
LmjF03.0360	*L. major*	196	315	510
LmjF03.0360	*L. major*	957	519	1475
LmjF03.0360	*L. major*	173	1488	1660
LmjF03.0370	*L. major*	50	1	50
LmjF03.0370	*L. major*	64	59	122
LmjF03.0370	*L. major*	64	59	122
LmjF03.0380	*L. major*	128	1	128
LmjF03.0390	*L. major*	86	1	86
LmjF03.0390	*L. major*	59	185	243
LmjF03.0390	*L. major*	45	393	437
LmjF03.0410	*L. major*	64	1	64
LmjF03.0410	*L. major*	111	283	393
LmjF03.0410	*L. major*	46	638	683
LmjF03.0420	*L. major*	46	1	46
LmjF03.0420	*L. major*	164	59	222
LmjF03.0420	*L. major*	95	229	323
LmjF03.0420	*L. major*	89	339	427

LmjF03.0420	*L. major*	147	435	581
LmjF03.0420	*L. major*	43	612	654
LmjF03.0420	*L. major*	56	752	807
LmjF03.0420	*L. major*	60	932	991
LmjF03.0420	*L. major*	219	1288	1506
LmjF03.0420	*L. major*	90	1627	1716
LmjF03.0430	*L. major*	61	51	111
LmjF03.0440	*L. major*	61	51	111
LmjF03.0450	*L. major*	52	1	52
LmjF03.0450	*L. major*	111	111	221
LmjF03.0450	*L. major*	102	254	355
LmjF03.0450	*L. major*	93	410	502
LmjF03.0450	*L. major*	41	530	570
LmjF03.0450	*L. major*	45	684	728
LmjF03.0450	*L. major*	81	808	888
LmjF03.0450	*L. major*	220	910	1129
LmjF03.0450	*L. major*	45	1196	1240
LmjF03.0450	*L. major*	80	1275	1354
LmjF03.0450	*L. major*	47	1384	1430
LmjF03.0450	*L. major*	49	1508	1556
LmjF03.0460	*L. major*	404	1	404
LmjF03.0460	*L. major*	117	422	538
LmjF03.0460	*L. major*	65	569	633
LmjF03.0460	*L. major*	64	668	731
LmjF03.0460	*L. major*	46	774	819
LmjF03.0460	*L. major*	62	838	899
LmjF03.0460	*L. major*	149	914	1062
LmjF03.0470	*L. major*	53	1	53
LmjF03.0470	*L. major*	103	93	195
LmjF03.0470	*L. major*	46	266	311
LmjF03.0470	*L. major*	47	389	435
LmjF03.0470	*L. major*	48	467	514
LmjF03.0470	*L. major*	52	535	586
LmjF03.0490	*L. major*	68	25	92
LmjF03.0490	*L. major*	267	121	387
LmjF03.0490	*L. major*	181	405	585
LmjF03.0490	*L. major*	93	595	687
LmjF03.0490	*L. major*	65	958	1022
LmjF03.0500	*L. major*	122	252	373
LmjF03.0510	*L. major*	170	1	170
LmjF03.0510	*L. major*	155	196	350
LmjF03.0510	*L. major*	737	364	1100
LmjF03.0510	*L. major*	253	1142	1394
LmjF03.0510	*L. major*	120	1418	1537
LmjF03.0510	*L. major*	118	1581	1698
LmjF03.0510	*L. major*	47	1753	1799
LmjF03.0510	*L. major*	47	1827	1873

LmjF03.0510	*L. major*	200	1961	2160
LmjF03.0510	*L. major*	49	2239	2287
LmjF03.0510	*L. major*	63	2384	2446
LmjF03.0520	*L. major*	353	1	353
LmjF03.0520	*L. major*	48	536	583
LmjF03.0520	*L. major*	46	655	700
LmjF03.0530	*L. major*	41	33	73
LmjF03.0530	*L. major*	106	149	254
LmjF03.0530	*L. major*	95	304	398
LmjF03.0530	*L. major*	69	474	542
LmjF03.0530	*L. major*	40	819	858
LmjF03.0530	*L. major*	90	994	1083
LmjF03.0530	*L. major*	47	1133	1179
LmjF03.0530	*L. major*	56	1190	1245
LmjF03.0530	*L. major*	68	1289	1356
LmjF03.0530	*L. major*	56	1406	1461
LmjF03.0530	*L. major*	118	1480	1597
LmjF03.0530	*L. major*	96	1684	1779
LmjF03.0530	*L. major*	132	1819	1950
LmjF03.0540	*L. major*	49	1	49
LmjF03.0550	*L. major*	72	145	216
LmjF03.0550	*L. major*	74	339	412
LmjF03.0550	*L. major*	54	451	504
LmjF03.0550	*L. major*	340	613	952
LmjF03.0550	*L. major*	86	1153	1238
LmjF03.0560	*L. major*	117	1	117
LmjF03.0560	*L. major*	257	140	396
LmjF03.0560	*L. major*	69	471	539
LmjF03.0560	*L. major*	46	573	618
LmjF03.0580	*L. major*	99	80	178
LmjF03.0590	*L. major*	96	17	112
LmjF03.0590	*L. major*	235	127	361
LmjF03.0590	*L. major*	88	469	556
LmjF03.0590	*L. major*	42	638	679
LmjF03.0590	*L. major*	107	728	834
LmjF03.0590	*L. major*	85	1112	1196
LmjF03.0600	*L. major*	51	1	51
LmjF03.0620	*L. major*	56	136	191
LmjF03.0620	*L. major*	54	549	602
LmjF03.0630	*L. major*	77	67	143
LmjF03.0630	*L. major*	42	182	223
LmjF03.0630	*L. major*	42	182	223
LmjF03.0640	*L. major*	59	52	110
LmjF03.0640	*L. major*	56	214	269
LmjF03.0650	*L. major*	147	1	147
LmjF03.0650	*L. major*	41	160	200
LmjF03.0650	*L. major*	70	222	291

LmjF03.0650	*L. major*	81	575	655
LmjF03.0650	*L. major*	81	813	893
LmjF03.0660	*L. major*	91	7	97
LmjF03.0660	*L. major*	170	402	571
LmjF03.0690	*L. major*	40	32	71
LmjF03.0690	*L. major*	68	284	351
LmjF03.0690	*L. major*	89	685	773
LmjF03.0690	*L. major*	58	1447	1504
LmjF03.0690	*L. major*	66	1552	1617
LmjF03.0700	*L. major*	222	68	289
LmjF03.0700	*L. major*	114	396	509
LmjF03.0700	*L. major*	49	521	569
LmjF03.0700	*L. major*	86	589	674
LmjF03.0700	*L. major*	50	714	763
LmjF03.0700	*L. major*	45	798	842
LmjF03.0700	*L. major*	80	897	976
LmjF03.0700	*L. major*	63	992	1054
LmjF03.0700	*L. major*	51	1134	1184
LmjF03.0700	*L. major*	96	1287	1382
LmjF03.0700	*L. major*	66	1431	1496
LmjF03.0700	*L. major*	74	1709	1782
LmjF03.0700	*L. major*	59	1907	1965
LmjF03.0700	*L. major*	55	2054	2108
LmjF03.0700	*L. major*	99	2121	2219
LmjF03.0700	*L. major*	55	2274	2328
LmjF03.0700	*L. major*	112	2344	2455
LmjF03.0700	*L. major*	51	2549	2599
LmjF03.0710	*L. major*	60	47	106
LmjF03.0720	*L. major*	47	10	56
LmjF03.0720	*L. major*	57	86	142
LmjF03.0720	*L. major*	145	415	559
LmjF03.0720	*L. major*	90	784	873
LmjF03.0730	*L. major*	56	1	56
LmjF03.0730	*L. major*	91	113	203
LmjF03.0730	*L. major*	61	302	362
LmjF03.0750	*L. major*	81	1	81
LmjF03.0750	*L. major*	54	367	420
LmjF03.0750	*L. major*	252	504	755
LmjF03.0760	*L. major*	90	1	90
LmjF03.0760	*L. major*	46	152	197
LmjF03.0770	*L. major*	41	178	218
LmjF03.0780	*L. major*	232	10	241
LmjF03.0780	*L. major*	629	356	984
LmjF03.0780	*L. major*	50	1094	1143
LmjF03.0780	*L. major*	140	1209	1348
LmjF03.0780	*L. major*	118	1360	1477
LmjF03.0780	*L. major*	64	1555	1618

LmjF03.0780	*L. major*	149	1657	1805
LmjF03.0780	*L. major*	901	1821	2721
LmjF03.0780	*L. major*	41	2755	2795
LmjF03.0780	*L. major*	298	2815	3112
LmjF03.0790	*L. major*	115	1	115
LmjF03.0790	*L. major*	134	257	390
LmjF03.0800	*L. major*	631	1	631
LmjF03.0800	*L. major*	146	642	787
LmjF03.0800	*L. major*	65	790	854
LmjF03.0800	*L. major*	150	997	1146
LmjF03.0800	*L. major*	158	1165	1322
LmjF03.0800	*L. major*	45	1328	1372
LmjF03.0800	*L. major*	126	1401	1526
LmjF03.0800	*L. major*	199	1547	1745
LmjF03.0800	*L. major*	96	2044	2139
LmjF03.0800	*L. major*	71	2178	2248
LmjF03.0800	*L. major*	68	2355	2422
LmjF03.0810	*L. major*	317	1	317
LmjF03.0810	*L. major*	50	347	396
LmjF03.0810	*L. major*	124	557	680
LmjF03.0810	*L. major*	61	731	791
LmjF03.0820	*L. major*	651	1	651
LmjF03.0820	*L. major*	131	654	784
LmjF03.0820	*L. major*	880	816	1695
LmjF03.0820	*L. major*	139	1843	1981
LmjF03.0820	*L. major*	187	2024	2210
LmjF03.0820	*L. major*	259	2232	2490
LmjF03.0820	*L. major*	438	2499	2936
LmjF03.0830	*L. major*	58	1	58
LmjF03.0830	*L. major*	65	110	174
LmjF03.0830	*L. major*	65	110	174
LmjF03.0840	*L. major*	87	56	142
LmjF03.0840	*L. major*	71	228	298
LmjF03.0840	*L. major*	76	494	569
LmjF03.0840	*L. major*	42	707	748
LmjF03.0840	*L. major*	112	863	974
LmjF03.0850	*L. major*	99	1	99
LmjF03.0850	*L. major*	537	114	650
LmjF03.0850	*L. major*	263	681	943
LmjF03.0850	*L. major*	85	949	1033
LmjF03.0850	*L. major*	87	1066	1152
LmjF03.0850	*L. major*	80	1212	1291
LmjF03.0850	*L. major*	590	1305	1894
LmjF03.0850	*L. major*	60	1902	1961
LmjF03.0850	*L. major*	79	1992	2070
LmjF03.0850	*L. major*	303	2080	2382
LmjF03.0860	*L. major*	140	54	193

LmjF03.0860	*L. major*	64	226	289
LmjF03.0860	*L. major*	55	393	447
LmjF03.0860	*L. major*	89	727	815
LmjF03.0860	*L. major*	40	874	913
LmjF03.0860	*L. major*	43	1053	1095
LmjF03.0860	*L. major*	60	1105	1164
LmjF03.0870	*L. major*	260	161	420
LmjF03.0870	*L. major*	604	673	1276
LmjF03.0880	*L. major*	125	1	125
LmjF03.0880	*L. major*	84	251	334
LmjF03.0880	*L. major*	42	500	541
LmjF03.0880	*L. major*	83	637	719
LmjF03.0880	*L. major*	111	796	906
LmjF03.0880	*L. major*	64	1332	1395
LmjF03.0880	*L. major*	45	1809	1853
LmjF03.0890	*L. major*	82	61	142
LmjF03.0890	*L. major*	104	180	283
LmjF03.0890	*L. major*	136	415	550
LmjF03.0890	*L. major*	108	603	710
LmjF03.0900	*L. major*	81	294	374
LmjF03.0940	*L. major*	144	1	144
LmjF03.0980	*L. major*	60	373	432
LmjF04.0030	*L. major*	45	66	110
LmjF04.0030	*L. major*	81	477	557
LmjF04.0030	*L. major*	54	672	725
LmjF04.0040	*L. major*	40	1	40
LmjF04.0040	*L. major*	90	44	133
LmjF04.0040	*L. major*	41	399	439
LmjF04.0050	*L. major*	182	1	182
LmjF04.0050	*L. major*	149	214	362
LmjF04.0050	*L. major*	121	396	516
LmjF04.0060	*L. major*	201	1	201
LmjF04.0080	*L. major*	135	24	158
LmjF04.0090	*L. major*	88	1	88
LmjF04.0090	*L. major*	40	1112	1151
LmjF04.0090	*L. major*	55	1336	1390
LmjF04.0100	*L. major*	112	207	318
LmjF04.0100	*L. major*	130	450	579
LmjF04.0110	*L. major*	65	81	145
LmjF04.0110	*L. major*	156	192	347
LmjF04.0123	*L. major*	136	23	158
LmjF04.0127	*L. major*	82	1	82
LmjF04.0127	*L. major*	156	116	271
LmjF04.0127	*L. major*	61	330	390
LmjF04.0130	*L. major*	63	51	113
LmjF04.0130	*L. major*	66	356	421
LmjF04.0130	*L. major*	64	434	497

LmjF04.0140	*L. major*	52	51	102
LmjF04.0140	*L. major*	66	356	421
LmjF04.0140	*L. major*	64	434	497
LmjF04.0150	*L. major*	52	51	102
LmjF04.0150	*L. major*	66	356	421
LmjF04.0150	*L. major*	64	434	497
LmjF04.0160	*L. major*	52	51	102
LmjF04.0160	*L. major*	66	356	421
LmjF04.0160	*L. major*	64	434	497
LmjF04.0170	*L. major*	52	51	102
LmjF04.0170	*L. major*	66	356	421
LmjF04.0170	*L. major*	64	434	497
LmjF04.0180	*L. major*	96	159	254
LmjF04.0180	*L. major*	85	336	420
LmjF04.0180	*L. major*	85	336	420
LmjF04.0190	*L. major*	114	383	496
LmjF04.0190	*L. major*	85	541	625
LmjF04.0190	*L. major*	85	541	625
LmjF04.0200	*L. major*	104	1	104
LmjF04.0210	*L. major*	117	120	236
LmjF04.0220	*L. major*	62	293	354
LmjF04.0230	*L. major*	63	104	166
LmjF04.0230	*L. major*	69	190	258
LmjF04.0240	*L. major*	99	1	99
LmjF04.0250	*L. major*	92	1	92
LmjF04.0250	*L. major*	74	132	205
LmjF04.0250	*L. major*	127	360	486
LmjF04.0250	*L. major*	61	655	715
LmjF04.0250	*L. major*	58	794	851
LmjF04.0250	*L. major*	72	1017	1088
LmjF04.0250	*L. major*	106	1213	1318
LmjF04.0260	*L. major*	42	1	42
LmjF04.0260	*L. major*	62	74	135
LmjF04.0260	*L. major*	62	74	135
LmjF04.0270	*L. major*	81	65	145
LmjF04.0270	*L. major*	51	240	290
LmjF04.0270	*L. major*	51	240	290
LmjF04.0280	*L. major*	122	129	250
LmjF04.0280	*L. major*	73	310	382
LmjF04.0280	*L. major*	44	684	727
LmjF04.0280	*L. major*	60	1059	1118
LmjF04.0280	*L. major*	81	1165	1245
LmjF04.0280	*L. major*	48	1407	1454
LmjF04.0280	*L. major*	231	1863	2093
LmjF04.0320	*L. major*	40	1	40
LmjF04.0330	*L. major*	59	1	59
LmjF04.0330	*L. major*	41	92	132

LmjF04.0330	*L. major*	55	373	427
LmjF04.0330	*L. major*	41	438	478
LmjF04.0350	*L. major*	305	1	305
LmjF04.0350	*L. major*	344	385	728
LmjF04.0350	*L. major*	83	736	818
LmjF04.0350	*L. major*	86	831	916
LmjF04.0350	*L. major*	50	919	968
LmjF04.0350	*L. major*	97	1012	1108
LmjF04.0350	*L. major*	203	1207	1409
LmjF04.0350	*L. major*	60	1489	1548
LmjF04.0350	*L. major*	55	1627	1681
LmjF04.0350	*L. major*	47	1797	1843
LmjF04.0350	*L. major*	57	2050	2106
LmjF04.0370	*L. major*	65	1	65
LmjF04.0370	*L. major*	103	166	268
LmjF04.0390	*L. major*	56	189	244
LmjF04.0400	*L. major*	51	1	51
LmjF04.0400	*L. major*	91	175	265
LmjF04.0410	*L. major*	478	1	478
LmjF04.0410	*L. major*	362	530	891
LmjF04.0410	*L. major*	76	898	973
LmjF04.0410	*L. major*	109	996	1104
LmjF04.0420	*L. major*	69	293	361
LmjF04.0430	*L. major*	63	195	257
LmjF04.0430	*L. major*	69	303	371
LmjF04.0430	*L. major*	124	526	649
LmjF04.0430	*L. major*	43	802	844
LmjF04.0430	*L. major*	66	1077	1142
LmjF04.0430	*L. major*	43	1205	1247
LmjF04.0430	*L. major*	119	1277	1395
LmjF04.0430	*L. major*	41	2871	2911
LmjF04.0430	*L. major*	69	2915	2983
LmjF04.0430	*L. major*	42	3030	3071
LmjF04.0440	*L. major*	260	37	296
LmjF04.0440	*L. major*	573	312	884
LmjF04.0440	*L. major*	49	925	973
LmjF04.0440	*L. major*	59	1005	1063
LmjF04.0440	*L. major*	62	1206	1267
LmjF04.0450	*L. major*	143	1	143
LmjF04.0470	*L. major*	50	139	188
LmjF04.0490	*L. major*	230	89	318
LmjF04.0500	*L. major*	608	1	608
LmjF04.0500	*L. major*	428	610	1037
LmjF04.0500	*L. major*	276	1124	1399
LmjF04.0500	*L. major*	198	1423	1620
LmjF04.0500	*L. major*	70	1747	1816
LmjF04.0500	*L. major*	49	1839	1887

LmjF04.0500	*L. major*	246	1922	2167
LmjF04.0500	*L. major*	405	2287	2691
LmjF04.0500	*L. major*	142	2698	2839
LmjF04.0500	*L. major*	162	2851	3012
LmjF04.0510	*L. major*	173	1	173
LmjF04.0510	*L. major*	296	201	496
LmjF04.0510	*L. major*	68	648	715
LmjF04.0510	*L. major*	81	725	805
LmjF04.0510	*L. major*	118	815	932
LmjF04.0510	*L. major*	78	1116	1193
LmjF04.0510	*L. major*	134	1197	1330
LmjF04.0520	*L. major*	59	96	154
LmjF04.0520	*L. major*	85	279	363
LmjF04.0520	*L. major*	114	888	1001
LmjF04.0530	*L. major*	195	1	195
LmjF04.0530	*L. major*	96	249	344
LmjF04.0530	*L. major*	53	396	448
LmjF04.0540	*L. major*	153	104	256
LmjF04.0540	*L. major*	168	421	588
LmjF04.0550	*L. major*	69	1252	1320
LmjF04.0560	*L. major*	101	200	300
LmjF04.0570	*L. major*	51	31	81
LmjF04.0570	*L. major*	48	488	535
LmjF04.0570	*L. major*	57	581	637
LmjF04.0570	*L. major*	123	675	797
LmjF04.0570	*L. major*	63	1005	1067
LmjF04.0570	*L. major*	42	1205	1246
LmjF04.0570	*L. major*	106	1322	1427
LmjF04.0570	*L. major*	114	1452	1565
LmjF04.0590	*L. major*	41	130	170
LmjF04.0600	*L. major*	43	76	118
LmjF04.0600	*L. major*	240	236	475
LmjF04.0600	*L. major*	66	589	654
LmjF04.0600	*L. major*	150	743	892
LmjF04.0600	*L. major*	191	914	1104
LmjF04.0600	*L. major*	46	1119	1164
LmjF04.0600	*L. major*	141	1277	1417
LmjF04.0600	*L. major*	72	1500	1571
LmjF04.0600	*L. major*	103	1633	1735
LmjF04.0600	*L. major*	123	1782	1904
LmjF04.0600	*L. major*	287	1962	2248
LmjF04.0600	*L. major*	75	2258	2332
LmjF04.0600	*L. major*	174	2387	2560
LmjF04.0600	*L. major*	64	2625	2688
LmjF04.0600	*L. major*	179	2700	2878
LmjF04.0600	*L. major*	123	2890	3012
LmjF04.0610	*L. major*	168	1	168

LmjF04.0620	*L. major*	42	1	42
LmjF04.0620	*L. major*	1136	111	1246
LmjF04.0625	*L. major*	144	195	338
LmjF04.0650	*L. major*	51	117	167
LmjF04.0650	*L. major*	74	197	270
LmjF04.0650	*L. major*	86	480	565
LmjF04.0650	*L. major*	96	582	677
LmjF04.0650	*L. major*	49	728	776
LmjF04.0650	*L. major*	123	1009	1131
LmjF04.0660	*L. major*	49	1	49
LmjF04.0670	*L. major*	54	1	54
LmjF04.0690	*L. major*	82	1	82
LmjF04.0690	*L. major*	124	119	242
LmjF04.0690	*L. major*	45	324	368
LmjF04.0690	*L. major*	76	490	565
LmjF04.0690	*L. major*	55	700	754
LmjF04.0700	*L. major*	284	1	284
LmjF04.0700	*L. major*	100	305	404
LmjF04.0700	*L. major*	192	521	712
LmjF04.0700	*L. major*	85	754	838
LmjF04.0700	*L. major*	65	940	1004
LmjF04.0710	*L. major*	249	86	334
LmjF04.0710	*L. major*	118	348	465
LmjF04.0710	*L. major*	98	596	693
LmjF04.0720	*L. major*	227	1	227
LmjF04.0720	*L. major*	88	246	333
LmjF04.0720	*L. major*	281	363	643
LmjF04.0730	*L. major*	172	3	174
LmjF04.0730	*L. major*	69	183	251
LmjF04.0730	*L. major*	47	383	429
LmjF04.0730	*L. major*	49	562	610
LmjF04.0740	*L. major*	53	1	53
LmjF04.0740	*L. major*	122	128	249
LmjF04.0740	*L. major*	92	281	372
LmjF04.0740	*L. major*	365	452	816
LmjF04.0760	*L. major*	73	48	120
LmjF04.0760	*L. major*	68	162	229
LmjF04.0760	*L. major*	68	162	229
LmjF04.0770	*L. major*	48	1	48
LmjF04.0770	*L. major*	68	90	157
LmjF04.0770	*L. major*	68	90	157
LmjF04.0780	*L. major*	55	224	278
LmjF04.0780	*L. major*	40	377	416
LmjF04.0780	*L. major*	75	499	573
LmjF04.0790	*L. major*	738	340	1077
LmjF04.0800	*L. major*	79	1	79
LmjF04.0800	*L. major*	95	88	182

LmjF04.0800	*L. major*	91	213	303
LmjF04.0810	*L. major*	60	257	316
LmjF04.0810	*L. major*	213	459	671
LmjF04.0820	*L. major*	42	1	42
LmjF04.0820	*L. major*	93	73	165
LmjF04.0820	*L. major*	53	449	501
LmjF04.0830	*L. major*	209	1	209
LmjF04.0830	*L. major*	303	214	516
LmjF04.0830	*L. major*	192	571	762
LmjF04.0830	*L. major*	428	773	1200
LmjF04.0830	*L. major*	104	1209	1312
LmjF04.0840	*L. major*	123	1	123
LmjF04.0840	*L. major*	266	127	392
LmjF04.0840	*L. major*	74	580	653
LmjF04.0840	*L. major*	126	684	809
LmjF04.0840	*L. major*	78	814	891
LmjF04.0850	*L. major*	92	1	92
LmjF04.0850	*L. major*	52	138	189
LmjF04.0860	*L. major*	61	35	95
LmjF04.0860	*L. major*	56	191	246
LmjF04.0860	*L. major*	68	342	409
LmjF04.0870	*L. major*	72	1	72
LmjF04.0870	*L. major*	1155	87	1241
LmjF04.0880	*L. major*	180	1	180
LmjF04.0890	*L. major*	102	1	102
LmjF04.0890	*L. major*	81	242	322
LmjF04.0890	*L. major*	810	470	1279
LmjF04.0900	*L. major*	975	1	975
LmjF04.0900	*L. major*	56	1028	1083
LmjF04.0900	*L. major*	90	1355	1444
LmjF04.0900	*L. major*	86	1642	1727
LmjF04.0900	*L. major*	48	1845	1892
LmjF04.0910	*L. major*	124	65	188
LmjF04.0920	*L. major*	86	1	86
LmjF04.0920	*L. major*	154	140	293
LmjF04.0920	*L. major*	205	309	513
LmjF04.0930	*L. major*	90	1	90
LmjF04.0930	*L. major*	83	159	241
LmjF04.0930	*L. major*	83	159	241
LmjF04.0940	*L. major*	64	24	87
LmjF04.0940	*L. major*	136	95	230
LmjF04.0940	*L. major*	92	264	355
LmjF04.0970	*L. major*	307	18	324
LmjF04.0975	*L. major*	84	31	114
LmjF04.0975	*L. major*	127	192	318
LmjF04.0975	*L. major*	118	327	444
LmjF04.0980	*L. major*	256	1	256

LmjF04.0980	*L. major*	53	292	344
LmjF04.0980	*L. major*	46	387	432
LmjF04.0980	*L. major*	58	446	503
LmjF04.0980	*L. major*	106	745	850
LmjF04.0980	*L. major*	59	994	1052
LmjF04.0990	*L. major*	170	1	170
LmjF04.0990	*L. major*	110	224	333
LmjF04.0990	*L. major*	73	372	444
LmjF04.1000	*L. major*	52	71	122
LmjF04.1000	*L. major*	64	713	776
LmjF04.1000	*L. major*	223	956	1178
LmjF04.1010	*L. major*	222	1	222
LmjF04.1020	*L. major*	44	1	44
LmjF04.1030	*L. major*	54	141	194
LmjF04.1040	*L. major*	68	1	68
LmjF04.1050	*L. major*	50	307	356
LmjF04.1060	*L. major*	50	55	104
LmjF04.1060	*L. major*	306	180	485
LmjF04.1070	*L. major*	59	1	59
LmjF04.1080	*L. major*	243	1	243
LmjF04.1080	*L. major*	65	826	890
LmjF04.1090	*L. major*	932	1	932
LmjF04.1090	*L. major*	107	979	1085
LmjF04.1090	*L. major*	210	1120	1329
LmjF04.1100	*L. major*	55	81	135
LmjF04.1110	*L. major*	45	894	938
LmjF04.1120	*L. major*	53	38	90
LmjF04.1120	*L. major*	43	133	175
LmjF04.1120	*L. major*	141	202	342
LmjF04.1120	*L. major*	74	373	446
LmjF04.1120	*L. major*	338	494	831
LmjF04.1130	*L. major*	48	37	84
LmjF04.1140	*L. major*	300	1	300
LmjF04.1150	*L. major*	59	110	168
LmjF04.1170	*L. major*	90	1	90
LmjF04.1170	*L. major*	46	266	311
LmjF04.1180	*L. major*	56	1084	1139
LmjF04.1180	*L. major*	174	1253	1426
LmjF04.1180	*L. major*	154	1440	1593
LmjF04.1180	*L. major*	234	1612	1845
LmjF04.1180	*L. major*	600	1860	2459
LmjF04.1210	*L. major*	61	308	368
LmjF04.1220	*L. major*	353	107	459
LmjF04.1220	*L. major*	84	951	1034
LmjF05.0010	*L. major*	105	1	105
LmjF05.0010	*L. major*	54	133	186
LmjF05.0010	*L. major*	42	203	244

LmjF05.0010	*L. major*	172	254	425
LmjF05.0010	*L. major*	44	431	474
LmjF05.0010	*L. major*	91	525	615
LmjF05.0010	*L. major*	155	684	838
LmjF05.0020	*L. major*	44	16	59
LmjF05.0040	*L. major*	355	1	355
LmjF05.0040	*L. major*	93	382	474
LmjF05.0050	*L. major*	105	46	150
LmjF05.0050	*L. major*	86	185	270
LmjF05.0050	*L. major*	54	357	410
LmjF05.0050	*L. major*	80	754	833
LmjF05.0060	*L. major*	157	618	774
LmjF05.0080	*L. major*	110	1	110
LmjF05.0080	*L. major*	44	158	201
LmjF05.0080	*L. major*	44	158	201
LmjF05.0100	*L. major*	53	226	278
LmjF05.0120	*L. major*	44	272	315
LmjF05.0130	*L. major*	74	359	432
LmjF05.0140	*L. major*	89	1	89
LmjF05.0140	*L. major*	57	618	674
LmjF05.0150	*L. major*	49	393	441
LmjF05.0160	*L. major*	245	3	247
LmjF05.0160	*L. major*	229	290	518
LmjF05.0170	*L. major*	427	1	427
LmjF05.0170	*L. major*	324	438	761
LmjF05.0180	*L. major*	64	119	182
LmjF05.0180	*L. major*	113	187	299
LmjF05.0190	*L. major*	245	120	364
LmjF05.0200	*L. major*	48	1	48
LmjF05.0200	*L. major*	121	260	380
LmjF05.0200	*L. major*	83	584	666
LmjF05.0200	*L. major*	99	691	789
LmjF05.0210	*L. major*	117	1	117
LmjF05.0210	*L. major*	70	172	241
LmjF05.0210	*L. major*	42	407	448
LmjF05.0210	*L. major*	64	1054	1117
LmjF05.0210	*L. major*	187	1286	1472
LmjF05.0220	*L. major*	42	1	42
LmjF05.0220	*L. major*	458	208	665
LmjF05.0230	*L. major*	50	1	50
LmjF05.0230	*L. major*	40	158	197
LmjF05.0230	*L. major*	57	354	410
LmjF05.0230	*L. major*	67	525	591
LmjF05.0240	*L. major*	70	103	172
LmjF05.0240	*L. major*	860	174	1033
LmjF05.0240	*L. major*	103	1137	1239
LmjF05.0260	*L. major*	50	100	149

LmjF05.0260	*L. major*	111	283	393
LmjF05.0270	*L. major*	115	1	115
LmjF05.0270	*L. major*	108	212	319
LmjF05.0270	*L. major*	108	212	319
LmjF05.0285	*L. major*	89	1	89
LmjF05.0290	*L. major*	41	11	51
LmjF05.0290	*L. major*	43	387	429
LmjF05.0290	*L. major*	43	387	429
LmjF05.0300	*L. major*	95	94	188
LmjF05.0300	*L. major*	86	209	294
LmjF05.0300	*L. major*	91	477	567
LmjF05.0300	*L. major*	107	603	709
LmjF05.0300	*L. major*	40	729	768
LmjF05.0300	*L. major*	64	801	864
LmjF05.0310	*L. major*	79	1	79
LmjF05.0330	*L. major*	48	309	356
LmjF05.0330	*L. major*	111	386	496
LmjF05.0330	*L. major*	41	806	846
LmjF05.0340	*L. major*	42	146	187
LmjF05.0360	*L. major*	57	185	241
LmjF05.0360	*L. major*	148	478	625
LmjF05.0370	*L. major*	53	75	127
LmjF05.0370	*L. major*	169	160	328
LmjF05.0380	*L. major*	1484	4	1487
LmjF05.0380	*L. major*	282	1489	1770
LmjF05.0380	*L. major*	43	1777	1819
LmjF05.0390	*L. major*	97	1	97
LmjF05.0390	*L. major*	113	320	432
LmjF05.0400	*L. major*	319	177	495
LmjF05.0400	*L. major*	217	726	942
LmjF05.0410	*L. major*	138	1	138
LmjF05.0410	*L. major*	69	199	267
LmjF05.0410	*L. major*	43	315	357
LmjF05.0410	*L. major*	100	432	531
LmjF05.0410	*L. major*	60	562	621
LmjF05.0430	*L. major*	59	42	100
LmjF05.0430	*L. major*	59	198	256
LmjF05.0430	*L. major*	59	198	256
LmjF05.0440	*L. major*	69	86	154
LmjF05.0440	*L. major*	45	182	226
LmjF05.0440	*L. major*	40	447	486
LmjF05.0440	*L. major*	43	722	764
LmjF05.0460	*L. major*	69	1	69
LmjF05.0460	*L. major*	86	110	195
LmjF05.0460	*L. major*	154	474	627
LmjF05.0470	*L. major*	158	186	343
LmjF05.0480	*L. major*	122	211	332

LmjF05.0490	*L. major*	43	1	43
LmjF05.0530	*L. major*	261	1	261
LmjF05.0530	*L. major*	386	325	710
LmjF05.0530	*L. major*	209	723	931
LmjF05.0530	*L. major*	115	961	1075
LmjF05.0530	*L. major*	56	1122	1177
LmjF05.0530	*L. major*	150	1247	1396
LmjF05.0530	*L. major*	153	1437	1589
LmjF05.0530	*L. major*	193	1660	1852
LmjF05.0540	*L. major*	46	82	127
LmjF05.0560	*L. major*	122	54	175
LmjF05.0560	*L. major*	73	206	278
LmjF05.0560	*L. major*	59	369	427
LmjF05.0560	*L. major*	127	511	637
LmjF05.0570	*L. major*	58	193	250
LmjF05.0580	*L. major*	132	1	132
LmjF05.0580	*L. major*	49	338	386
LmjF05.0580	*L. major*	232	553	784
LmjF05.0590	*L. major*	241	13	253
LmjF05.0590	*L. major*	45	572	616
LmjF05.0590	*L. major*	57	675	731
LmjF05.0590	*L. major*	142	806	947
LmjF05.0600	*L. major*	41	146	186
LmjF05.0600	*L. major*	42	479	520
LmjF05.0600	*L. major*	46	628	673
LmjF05.0600	*L. major*	40	863	902
LmjF05.0610	*L. major*	145	1	145
LmjF05.0610	*L. major*	156	266	421
LmjF05.0610	*L. major*	42	551	592
LmjF05.0610	*L. major*	92	597	688
LmjF05.0610	*L. major*	122	699	820
LmjF05.0610	*L. major*	43	836	878
LmjF05.0620	*L. major*	398	45	442
LmjF05.0620	*L. major*	133	556	688
LmjF05.0620	*L. major*	160	715	874
LmjF05.0630	*L. major*	117	1	117
LmjF05.0630	*L. major*	50	193	242
LmjF05.0630	*L. major*	43	554	596
LmjF05.0630	*L. major*	54	632	685
LmjF05.0630	*L. major*	89	706	794
LmjF05.0630	*L. major*	304	924	1227
LmjF05.0640	*L. major*	47	1	47
LmjF05.0640	*L. major*	66	85	150
LmjF05.0640	*L. major*	69	175	243
LmjF05.0640	*L. major*	95	363	457
LmjF05.0640	*L. major*	41	720	760
LmjF05.0640	*L. major*	85	822	906

LmjF05.0640	*L. major*	67	1097	1163
LmjF05.0640	*L. major*	55	1183	1237
LmjF05.0640	*L. major*	192	1352	1543
LmjF05.0640	*L. major*	105	1681	1785
LmjF05.0650	*L. major*	110	1	110
LmjF05.0650	*L. major*	47	303	349
LmjF05.0650	*L. major*	213	387	599
LmjF05.0650	*L. major*	145	670	814
LmjF05.0660	*L. major*	90	1	90
LmjF05.0670	*L. major*	1212	1	1212
LmjF05.0670	*L. major*	365	1225	1589
LmjF05.0670	*L. major*	709	1638	2346
LmjF05.0670	*L. major*	168	2377	2544
LmjF05.0680	*L. major*	51	1	51
LmjF05.0690	*L. major*	155	1	155
LmjF05.0690	*L. major*	247	157	403
LmjF05.0690	*L. major*	326	452	777
LmjF05.0690	*L. major*	127	950	1076
LmjF05.0690	*L. major*	130	1157	1286
LmjF05.0690	*L. major*	320	1323	1642
LmjF05.0690	*L. major*	60	1725	1784
LmjF05.0690	*L. major*	690	1800	2489
LmjF05.0690	*L. major*	61	2510	2570
LmjF05.0690	*L. major*	81	2607	2687
LmjF05.0700	*L. major*	1078	1	1078
LmjF05.0700	*L. major*	179	1086	1264
LmjF05.0700	*L. major*	193	1278	1470
LmjF05.0700	*L. major*	90	1521	1610
LmjF05.0700	*L. major*	118	1626	1743
LmjF05.0710	*L. major*	659	1	659
LmjF05.0710	*L. major*	52	825	876
LmjF05.0710	*L. major*	504	920	1423
LmjF05.0720	*L. major*	303	1	303
LmjF05.0720	*L. major*	261	306	566
LmjF05.0720	*L. major*	142	704	845
LmjF05.0720	*L. major*	390	913	1302
LmjF05.0730	*L. major*	579	81	659
LmjF05.0730	*L. major*	64	675	738
LmjF05.0740	*L. major*	179	155	333
LmjF05.0750	*L. major*	70	31	100
LmjF05.0750	*L. major*	136	283	418
LmjF05.0750	*L. major*	136	283	418
LmjF05.0760	*L. major*	74	27	100
LmjF05.0760	*L. major*	151	113	263
LmjF05.0760	*L. major*	194	423	616
LmjF05.0760	*L. major*	43	635	677
LmjF05.0760	*L. major*	79	685	763

LmjF05.0760	*L. major*	150	864	1013
LmjF05.0760	*L. major*	95	1070	1164
LmjF05.0760	*L. major*	83	1172	1254
LmjF05.0770	*L. major*	782	19	800
LmjF05.0770	*L. major*	515	816	1330
LmjF05.0790	*L. major*	276	1	276
LmjF05.0790	*L. major*	45	299	343
LmjF05.0800	*L. major*	41	52	92
LmjF05.0800	*L. major*	77	175	251
LmjF05.0820	*L. major*	44	1	44
LmjF05.0820	*L. major*	53	199	251
LmjF05.0820	*L. major*	45	357	401
LmjF05.0840	*L. major*	634	1	634
LmjF05.0840	*L. major*	754	643	1396
LmjF05.0850	*L. major*	115	1	115
LmjF05.0850	*L. major*	351	160	510
LmjF05.0850	*L. major*	153	512	664
LmjF05.0860	*L. major*	95	61	155
LmjF05.0870	*L. major*	156	203	358
LmjF05.0870	*L. major*	130	441	570
LmjF05.0870	*L. major*	41	603	643
LmjF05.0870	*L. major*	1277	646	1922
LmjF05.0880	*L. major*	59	1	59
LmjF05.0880	*L. major*	46	160	205
LmjF05.0880	*L. major*	46	160	205
LmjF05.0890	*L. major*	65	248	312
LmjF05.0890	*L. major*	41	534	574
LmjF05.0890	*L. major*	188	630	817
LmjF05.0890	*L. major*	58	861	918
LmjF05.0890	*L. major*	59	1075	1133
LmjF05.0890	*L. major*	74	1138	1211
LmjF05.0890	*L. major*	59	1287	1345
LmjF05.0890	*L. major*	41	1367	1407
LmjF05.0890	*L. major*	43	1573	1615
LmjF05.0890	*L. major*	51	1930	1980
LmjF05.0910	*L. major*	56	1	56
LmjF05.0910	*L. major*	71	102	172
LmjF05.0910	*L. major*	65	509	573
LmjF05.0910	*L. major*	51	614	664
LmjF05.0910	*L. major*	96	719	814
LmjF05.0910	*L. major*	58	975	1032
LmjF05.0920	*L. major*	135	1	135
LmjF05.0920	*L. major*	295	147	441
LmjF05.0920	*L. major*	60	545	604
LmjF05.0920	*L. major*	54	645	698
LmjF05.0920	*L. major*	271	844	1114
LmjF05.0930	*L. major*	251	160	410

LmjF05.0940	*L. major*	507	127	633
LmjF05.0940	*L. major*	199	709	907
LmjF05.0940	*L. major*	247	932	1178
LmjF05.0950	*L. major*	66	225	290
LmjF05.0950	*L. major*	41	338	378
LmjF05.0950	*L. major*	51	711	761
LmjF05.1010	*L. major*	41	95	135
LmjF05.1010	*L. major*	141	475	615
LmjF05.1020	*L. major*	66	179	244
LmjF05.1020	*L. major*	61	307	367
LmjF05.1020	*L. major*	46	455	500
LmjF05.1020	*L. major*	42	668	709
LmjF05.1020	*L. major*	48	740	787
LmjF05.1020	*L. major*	55	810	864
LmjF05.1030	*L. major*	50	24	73
LmjF05.1040	*L. major*	44	11	54
LmjF05.1040	*L. major*	61	212	272
LmjF05.1040	*L. major*	61	212	272
LmjF05.1050	*L. major*	68	1	68
LmjF05.1050	*L. major*	41	139	179
LmjF05.1060	*L. major*	43	145	187
LmjF05.1060	*L. major*	47	404	450
LmjF05.1060	*L. major*	47	404	450
LmjF05.1070	*L. major*	98	150	247
LmjF05.1080	*L. major*	62	1	62
LmjF05.1080	*L. major*	73	137	209
LmjF05.1080	*L. major*	54	535	588
LmjF05.1080	*L. major*	66	661	726
LmjF05.1090	*L. major*	195	188	382
LmjF05.1090	*L. major*	128	392	519
LmjF05.1110	*L. major*	120	1	120
LmjF05.1110	*L. major*	101	218	318
LmjF05.1110	*L. major*	105	389	493
LmjF05.1110	*L. major*	224	686	909
LmjF05.1110	*L. major*	71	1065	1135
LmjF05.1130	*L. major*	43	522	564
LmjF05.1160	*L. major*	225	99	323
LmjF05.1160	*L. major*	169	356	524
LmjF05.1160	*L. major*	191	541	731
LmjF05.1160	*L. major*	85	752	836
LmjF05.1180	*L. major*	100	1	100
LmjF05.1180	*L. major*	48	248	295
LmjF05.1180	*L. major*	135	324	458
LmjF05.1190	*L. major*	66	1	66
LmjF05.1190	*L. major*	63	221	283
LmjF05.1190	*L. major*	40	449	488
LmjF05.1190	*L. major*	121	551	671

LmjF05.1190	*L. major*	52	730	781
LmjF05.1190	*L. major*	66	848	913
LmjF05.1190	*L. major*	84	956	1039
LmjF05.1190	*L. major*	97	1206	1302
LmjF05.1190	*L. major*	289	1325	1613
LmjF05.1190	*L. major*	336	1625	1960
LmjF05.1200	*L. major*	55	1	55
LmjF05.1200	*L. major*	41	92	132
LmjF05.1200	*L. major*	41	92	132
LmjF05.1210	*L. major*	64	1	64
LmjF05.1220	*L. major*	42	407	448
LmjF05.1230	*L. major*	112	45	156
LmjF06.0010	*L. major*	46	1	46
LmjF06.0030	*L. major*	67	52	118
LmjF06.0030	*L. major*	57	166	222
LmjF06.0030	*L. major*	41	324	364
LmjF06.0050	*L. major*	141	1	141
LmjF06.0060	*L. major*	103	56	158
LmjF06.0060	*L. major*	94	228	321
LmjF06.0060	*L. major*	94	228	321
LmjF06.0080	*L. major*	45	15	59
LmjF06.0090	*L. major*	60	1	60
LmjF06.0110	*L. major*	94	104	197
LmjF06.0110	*L. major*	100	342	441
LmjF06.0110	*L. major*	100	342	441
LmjF06.0170	*L. major*	43	53	95
LmjF06.0180	*L. major*	52	51	102
LmjF06.0180	*L. major*	53	187	239
LmjF06.0180	*L. major*	122	326	447
LmjF06.0180	*L. major*	167	506	672
LmjF06.0190	*L. major*	79	101	179
LmjF06.0190	*L. major*	60	225	284
LmjF06.0190	*L. major*	174	319	492
LmjF06.0190	*L. major*	54	534	587
LmjF06.0200	*L. major*	331	1	331
LmjF06.0210	*L. major*	72	62	133
LmjF06.0210	*L. major*	78	203	280
LmjF06.0210	*L. major*	91	288	378
LmjF06.0210	*L. major*	57	395	451
LmjF06.0210	*L. major*	54	562	615
LmjF06.0220	*L. major*	76	212	287
LmjF06.0230	*L. major*	111	1	111
LmjF06.0240	*L. major*	52	551	602
LmjF06.0240	*L. major*	47	650	696
LmjF06.0240	*L. major*	96	702	797
LmjF06.0250	*L. major*	144	113	256
LmjF06.0260	*L. major*	50	1061	1110

LmjF06.0260	*L. major*	262	1180	1441
LmjF06.0270	*L. major*	67	103	169
LmjF06.0270	*L. major*	43	189	231
LmjF06.0270	*L. major*	43	189	231
LmjF06.0280	*L. major*	77	78	154
LmjF06.0280	*L. major*	61	217	277
LmjF06.0280	*L. major*	94	525	618
LmjF06.0280	*L. major*	64	662	725
LmjF06.0280	*L. major*	100	741	840
LmjF06.0310	*L. major*	70	286	355
LmjF06.0320	*L. major*	108	12	119
LmjF06.0320	*L. major*	162	132	293
LmjF06.0320	*L. major*	380	302	681
LmjF06.0320	*L. major*	182	691	872
LmjF06.0320	*L. major*	42	899	940
LmjF06.0330	*L. major*	156	311	466
LmjF06.0330	*L. major*	87	790	876
LmjF06.0330	*L. major*	179	885	1063
LmjF06.0340	*L. major*	49	34	82
LmjF06.0340	*L. major*	48	858	905
LmjF06.0340	*L. major*	48	858	905
LmjF06.0380	*L. major*	44	29	72
LmjF06.0380	*L. major*	68	311	378
LmjF06.0380	*L. major*	68	311	378
LmjF06.0390	*L. major*	47	385	431
LmjF06.0390	*L. major*	41	475	515
LmjF06.0390	*L. major*	101	646	746
LmjF06.0390	*L. major*	57	778	834
LmjF06.0390	*L. major*	66	842	907
LmjF06.0410	*L. major*	63	74	136
LmjF06.0410	*L. major*	116	152	267
LmjF06.0412	*L. major*	131	35	165
LmjF06.0415	*L. major*	63	54	116
LmjF06.0415	*L. major*	123	132	254
LmjF06.0420	*L. major*	55	19	73
LmjF06.0420	*L. major*	314	139	452
LmjF06.0420	*L. major*	75	499	573
LmjF06.0430	*L. major*	114	1	114
LmjF06.0430	*L. major*	87	178	264
LmjF06.0430	*L. major*	118	477	594
LmjF06.0430	*L. major*	82	609	690
LmjF06.0440	*L. major*	118	17	134
LmjF06.0440	*L. major*	44	292	335
LmjF06.0440	*L. major*	314	423	736
LmjF06.0440	*L. major*	116	747	862
LmjF06.0440	*L. major*	92	902	993
LmjF06.0450	*L. major*	167	1	167

LmjF06.0450	*L. major*	45	179	223
LmjF06.0450	*L. major*	119	234	352
LmjF06.0460	*L. major*	153	32	184
LmjF06.0460	*L. major*	177	307	483
LmjF06.0460	*L. major*	279	497	775
LmjF06.0460	*L. major*	80	783	862
LmjF06.0460	*L. major*	69	931	999
LmjF06.0470	*L. major*	110	1	110
LmjF06.0470	*L. major*	85	176	260
LmjF06.0470	*L. major*	56	305	360
LmjF06.0470	*L. major*	197	373	569
LmjF06.0470	*L. major*	105	599	703
LmjF06.0470	*L. major*	69	794	862
LmjF06.0470	*L. major*	106	864	969
LmjF06.0470	*L. major*	95	1006	1100
LmjF06.0500	*L. major*	58	47	104
LmjF06.0500	*L. major*	50	124	173
LmjF06.0500	*L. major*	93	307	399
LmjF06.0500	*L. major*	75	429	503
LmjF06.0510	*L. major*	44	80	123
LmjF06.0510	*L. major*	79	293	371
LmjF06.0510	*L. major*	77	507	583
LmjF06.0520	*L. major*	44	1	44
LmjF06.0520	*L. major*	304	106	409
LmjF06.0520	*L. major*	210	446	655
LmjF06.0520	*L. major*	160	682	841
LmjF06.0520	*L. major*	254	872	1125
LmjF06.0520	*L. major*	79	1256	1334
LmjF06.0520	*L. major*	73	1392	1464
LmjF06.0530	*L. major*	52	1	52
LmjF06.0530	*L. major*	100	98	197
LmjF06.0540	*L. major*	133	34	166
LmjF06.0540	*L. major*	297	416	712
LmjF06.0540	*L. major*	41	723	763
LmjF06.0550	*L. major*	132	1	132
LmjF06.0550	*L. major*	77	214	290
LmjF06.0550	*L. major*	58	299	356
LmjF06.0550	*L. major*	61	365	425
LmjF06.0550	*L. major*	61	568	628
LmjF06.0550	*L. major*	60	743	802
LmjF06.0550	*L. major*	91	824	914
LmjF06.0550	*L. major*	116	1133	1248
LmjF06.0550	*L. major*	58	1554	1611
LmjF06.0550	*L. major*	51	1670	1720
LmjF06.0570	*L. major*	56	1	56
LmjF06.0580	*L. major*	56	1	56
LmjF06.0590	*L. major*	74	81	154

LmjF06.0590	*L. major*	68	241	308
LmjF06.0590	*L. major*	107	443	549
LmjF06.0590	*L. major*	282	555	836
LmjF06.0590	*L. major*	650	845	1494
LmjF06.0600	*L. major*	69	175	243
LmjF06.0600	*L. major*	203	272	474
LmjF06.0600	*L. major*	61	487	547
LmjF06.0600	*L. major*	69	600	668
LmjF06.0600	*L. major*	41	817	857
LmjF06.0600	*L. major*	59	875	933
LmjF06.0600	*L. major*	93	960	1052
LmjF06.0600	*L. major*	119	1138	1256
LmjF06.0600	*L. major*	103	1262	1364
LmjF06.0610	*L. major*	51	1	51
LmjF06.0620	*L. major*	44	515	558
LmjF06.0630	*L. major*	102	1	102
LmjF06.0630	*L. major*	42	148	189
LmjF06.0630	*L. major*	40	277	316
LmjF06.0630	*L. major*	220	372	591
LmjF06.0630	*L. major*	229	735	963
LmjF06.0630	*L. major*	138	985	1122
LmjF06.0630	*L. major*	166	1152	1317
LmjF06.0630	*L. major*	118	1460	1577
LmjF06.0630	*L. major*	59	1624	1682
LmjF06.0640	*L. major*	155	1	155
LmjF06.0640	*L. major*	40	159	198
LmjF06.0640	*L. major*	56	499	554
LmjF06.0640	*L. major*	279	612	890
LmjF06.0640	*L. major*	86	957	1042
LmjF06.0640	*L. major*	124	1173	1296
LmjF06.0640	*L. major*	150	1331	1480
LmjF06.0640	*L. major*	287	1504	1790
LmjF06.0640	*L. major*	55	1832	1886
LmjF06.0640	*L. major*	537	1918	2454
LmjF06.0640	*L. major*	48	2491	2538
LmjF06.0640	*L. major*	113	2592	2704
LmjF06.0640	*L. major*	158	2985	3142
LmjF06.0650	*L. major*	44	1	44
LmjF06.0660	*L. major*	372	1	372
LmjF06.0660	*L. major*	70	382	451
LmjF06.0660	*L. major*	129	486	614
LmjF06.0670	*L. major*	273	1	273
LmjF06.0670	*L. major*	87	275	361
LmjF06.0670	*L. major*	736	365	1100
LmjF06.0670	*L. major*	136	1147	1282
LmjF06.0680	*L. major*	99	1	99
LmjF06.0685	*L. major*	164	25	188

LmjF06.0685	*L. major*	91	202	292
LmjF06.0685	*L. major*	79	297	375
LmjF06.0690	*L. major*	61	61	121
LmjF06.0690	*L. major*	49	282	330
LmjF06.0690	*L. major*	53	367	419
LmjF06.0690	*L. major*	45	563	607
LmjF06.0690	*L. major*	60	862	921
LmjF06.0690	*L. major*	54	979	1032
LmjF06.0690	*L. major*	75	1272	1346
LmjF06.0690	*L. major*	47	1620	1666
LmjF06.0690	*L. major*	67	2037	2103
LmjF06.0690	*L. major*	212	2145	2356
LmjF06.0700	*L. major*	468	130	597
LmjF06.0710	*L. major*	52	1	52
LmjF06.0710	*L. major*	90	193	282
LmjF06.0710	*L. major*	157	372	528
LmjF06.0720	*L. major*	62	1	62
LmjF06.0720	*L. major*	542	91	632
LmjF06.0720	*L. major*	46	645	690
LmjF06.0720	*L. major*	76	721	796
LmjF06.0720	*L. major*	84	842	925
LmjF06.0720	*L. major*	52	1005	1056
LmjF06.0720	*L. major*	194	1084	1277
LmjF06.0720	*L. major*	85	1345	1429
LmjF06.0720	*L. major*	78	1609	1686
LmjF06.0740	*L. major*	671	1	671
LmjF06.0740	*L. major*	90	709	798
LmjF06.0740	*L. major*	131	810	940
LmjF06.0740	*L. major*	58	980	1037
LmjF06.0750	*L. major*	129	129	257
LmjF06.0750	*L. major*	60	387	446
LmjF06.0750	*L. major*	71	535	605
LmjF06.0760	*L. major*	164	1	164
LmjF06.0760	*L. major*	108	170	277
LmjF06.0760	*L. major*	66	316	381
LmjF06.0760	*L. major*	393	469	861
LmjF06.0760	*L. major*	107	882	988
LmjF06.0760	*L. major*	162	1017	1178
LmjF06.0770	*L. major*	101	1	101
LmjF06.0770	*L. major*	446	107	552
LmjF06.0770	*L. major*	176	566	741
LmjF06.0770	*L. major*	322	806	1127
LmjF06.0770	*L. major*	75	1205	1279
LmjF06.0770	*L. major*	133	1374	1506
LmjF06.0770	*L. major*	471	1538	2008
LmjF06.0780	*L. major*	578	1	578
LmjF06.0780	*L. major*	859	580	1438

LmjF06.0780	*L. major*	206	1448	1653
LmjF06.0780	*L. major*	193	1729	1921
LmjF06.0780	*L. major*	132	1935	2066
LmjF06.0780	*L. major*	298	2103	2400
LmjF06.0780	*L. major*	367	2417	2783
LmjF06.0790	*L. major*	366	66	431
LmjF06.0790	*L. major*	368	441	808
LmjF06.0790	*L. major*	252	822	1073
LmjF06.0800	*L. major*	495	1	495
LmjF06.0800	*L. major*	172	600	771
LmjF06.0800	*L. major*	203	786	988
LmjF06.0800	*L. major*	114	1016	1129
LmjF06.0800	*L. major*	76	1193	1268
LmjF06.0800	*L. major*	42	1353	1394
LmjF06.0800	*L. major*	117	1402	1518
LmjF06.0810	*L. major*	86	1	86
LmjF06.0810	*L. major*	54	139	192
LmjF06.0810	*L. major*	56	197	252
LmjF06.0810	*L. major*	200	375	574
LmjF06.0810	*L. major*	70	677	746
LmjF06.0810	*L. major*	57	1134	1190
LmjF06.0810	*L. major*	47	1319	1365
LmjF06.0810	*L. major*	57	1493	1549
LmjF06.0810	*L. major*	87	1560	1646
LmjF06.0810	*L. major*	47	2197	2243
LmjF06.0810	*L. major*	64	2370	2433
LmjF06.0810	*L. major*	125	2491	2615
LmjF06.0810	*L. major*	49	2650	2698
LmjF06.0810	*L. major*	43	2751	2793
LmjF06.0810	*L. major*	95	2931	3025
LmjF06.0810	*L. major*	99	3124	3222
LmjF06.0820	*L. major*	187	1	187
LmjF06.0820	*L. major*	73	228	300
LmjF06.0830	*L. major*	42	88	129
LmjF06.0830	*L. major*	304	157	460
LmjF06.0830	*L. major*	126	497	622
LmjF06.0830	*L. major*	234	864	1097
LmjF06.0830	*L. major*	337	1115	1451
LmjF06.0840	*L. major*	161	73	233
LmjF06.0850	*L. major*	49	1	49
LmjF06.0850	*L. major*	456	51	506
LmjF06.0850	*L. major*	278	518	795
LmjF06.0850	*L. major*	51	881	931
LmjF06.0850	*L. major*	85	1001	1085
LmjF06.0850	*L. major*	95	1129	1223
LmjF06.0850	*L. major*	131	1228	1358
LmjF06.0850	*L. major*	84	1386	1469

LmjF06.0850	*L. major*	91	1516	1606
LmjF06.0850	*L. major*	268	1683	1950
LmjF06.0850	*L. major*	383	1963	2345
LmjF06.0850	*L. major*	203	2377	2579
LmjF06.0850	*L. major*	45	2583	2627
LmjF06.0850	*L. major*	140	2696	2835
LmjF06.0850	*L. major*	42	2914	2955
LmjF06.0850	*L. major*	190	3154	3343
LmjF06.0870	*L. major*	72	1	72
LmjF06.0870	*L. major*	40	399	438
LmjF06.0870	*L. major*	40	399	438
LmjF06.0880	*L. major*	40	1	40
LmjF06.0890	*L. major*	120	22	141
LmjF06.0890	*L. major*	102	228	329
LmjF06.0890	*L. major*	62	427	488
LmjF06.0890	*L. major*	208	530	737
LmjF06.0900	*L. major*	180	1	180
LmjF06.0900	*L. major*	498	212	709
LmjF06.0900	*L. major*	428	748	1175
LmjF06.0900	*L. major*	58	1214	1271
LmjF06.0900	*L. major*	73	1322	1394
LmjF06.0900	*L. major*	183	1475	1657
LmjF06.0900	*L. major*	115	1707	1821
LmjF06.0920	*L. major*	147	250	396
LmjF06.0940	*L. major*	64	25	88
LmjF06.0940	*L. major*	51	155	205
LmjF06.0940	*L. major*	45	254	298
LmjF06.0940	*L. major*	68	321	388
LmjF06.0940	*L. major*	67	527	593
LmjF06.0970	*L. major*	96	1	96
LmjF06.0970	*L. major*	54	277	330
LmjF06.0970	*L. major*	222	433	654
LmjF06.0970	*L. major*	85	696	780
LmjF06.0970	*L. major*	114	1218	1331
LmjF06.0970	*L. major*	130	1374	1503
LmjF06.0980	*L. major*	52	1	52
LmjF06.0980	*L. major*	52	296	347
LmjF06.0980	*L. major*	74	798	871
LmjF06.0990	*L. major*	99	1	99
LmjF06.0990	*L. major*	77	173	249
LmjF06.0990	*L. major*	163	874	1036
LmjF06.0995	*L. major*	47	69	115
LmjF06.1000	*L. major*	89	83	171
LmjF06.1010	*L. major*	42	23	64
LmjF06.1010	*L. major*	68	118	185
LmjF06.1010	*L. major*	45	559	603
LmjF06.1020	*L. major*	151	80	230

LmjF06.1030	*L. major*	43	154	196
LmjF06.1030	*L. major*	82	381	462
LmjF06.1030	*L. major*	227	471	697
LmjF06.1030	*L. major*	127	708	834
LmjF06.1030	*L. major*	42	845	886
LmjF06.1040	*L. major*	188	1	188
LmjF06.1040	*L. major*	142	274	415
LmjF06.1040	*L. major*	88	480	567
LmjF06.1040	*L. major*	63	621	683
LmjF06.1060	*L. major*	41	1	41
LmjF06.1060	*L. major*	52	271	322
LmjF06.1060	*L. major*	52	271	322
LmjF06.1090	*L. major*	145	1	145
LmjF06.1090	*L. major*	80	186	265
LmjF06.1090	*L. major*	104	282	385
LmjF06.1090	*L. major*	133	463	595
LmjF06.1100	*L. major*	42	263	304
LmjF06.1110	*L. major*	134	1	134
LmjF06.1110	*L. major*	720	205	924
LmjF06.1110	*L. major*	125	984	1108
LmjF06.1140	*L. major*	101	79	179
LmjF06.1140	*L. major*	52	224	275
LmjF06.1140	*L. major*	169	398	566
LmjF06.1140	*L. major*	44	804	847
LmjF06.1150	*L. major*	95	98	192
LmjF06.1150	*L. major*	101	238	338
LmjF06.1150	*L. major*	65	445	509
LmjF06.1160	*L. major*	59	66	124
LmjF06.1160	*L. major*	70	133	202
LmjF06.1160	*L. major*	43	497	539
LmjF06.1160	*L. major*	40	559	598
LmjF06.1180	*L. major*	54	220	273
LmjF06.1190	*L. major*	45	1	45
LmjF06.1200	*L. major*	83	1	83
LmjF06.1200	*L. major*	58	152	209
LmjF06.1200	*L. major*	146	230	375
LmjF06.1210	*L. major*	55	452	506
LmjF06.1220	*L. major*	76	80	155
LmjF06.1220	*L. major*	74	377	450
LmjF06.1220	*L. major*	210	515	724
LmjF06.1220	*L. major*	62	729	790
LmjF06.1240	*L. major*	45	10	54
LmjF06.1240	*L. major*	43	307	349
LmjF06.1240	*L. major*	43	307	349
LmjF06.1245	*L. major*	42	79	120
LmjF06.1250	*L. major*	52	1	52
LmjF06.1260	*L. major*	49	270	318

LmjF06.1300	*L. major*	58	1	58
LmjF06.1300	*L. major*	72	504	575
LmjF06.1300	*L. major*	72	504	575
LmjF07.0020	*L. major*	48	43	90
LmjF07.0020	*L. major*	57	169	225
LmjF07.0020	*L. major*	57	169	225
LmjF07.0040	*L. major*	71	245	315
LmjF07.0050	*L. major*	48	644	691
LmjF07.0060	*L. major*	49	69	117
LmjF07.0070	*L. major*	61	11	71
LmjF07.0080	*L. major*	45	401	445
LmjF07.0090	*L. major*	40	633	672
LmjF07.0100	*L. major*	81	496	576
LmjF07.0120	*L. major*	134	31	164
LmjF07.0120	*L. major*	45	408	452
LmjF07.0120	*L. major*	71	642	712
LmjF07.0120	*L. major*	62	964	1025
LmjF07.0120	*L. major*	47	1771	1817
LmjF07.0120	*L. major*	52	1981	2032
LmjF07.0120	*L. major*	67	2128	2194
LmjF07.0130	*L. major*	91	276	366
LmjF07.0140	*L. major*	87	265	351
LmjF07.0150	*L. major*	58	20	77
LmjF07.0150	*L. major*	84	503	586
LmjF07.0150	*L. major*	84	503	586
LmjF07.0160	*L. major*	602	246	847
LmjF07.0170	*L. major*	125	195	319
LmjF07.0170	*L. major*	293	457	749
LmjF07.0170	*L. major*	146	829	974
LmjF07.0180	*L. major*	41	1	41
LmjF07.0180	*L. major*	71	73	143
LmjF07.0180	*L. major*	121	245	365
LmjF07.0180	*L. major*	60	408	467
LmjF07.0180	*L. major*	42	495	536
LmjF07.0180	*L. major*	481	736	1216
LmjF07.0180	*L. major*	149	1301	1449
LmjF07.0180	*L. major*	58	1478	1535
LmjF07.0180	*L. major*	40	1641	1680
LmjF07.0180	*L. major*	74	1708	1781
LmjF07.0180	*L. major*	92	1856	1947
LmjF07.0180	*L. major*	41	1984	2024
LmjF07.0200	*L. major*	43	41	83
LmjF07.0200	*L. major*	70	204	273
LmjF07.0200	*L. major*	74	379	452
LmjF07.0210	*L. major*	45	182	226
LmjF07.0220	*L. major*	90	48	137
LmjF07.0220	*L. major*	50	175	224

LmjF07.0220	*L. major*	40	596	635
LmjF07.0230	*L. major*	82	1	82
LmjF07.0240	*L. major*	79	149	227
LmjF07.0240	*L. major*	67	373	439
LmjF07.0250	*L. major*	556	1	556
LmjF07.0250	*L. major*	233	564	796
LmjF07.0250	*L. major*	324	826	1149
LmjF07.0250	*L. major*	57	1160	1216
LmjF07.0250	*L. major*	129	1345	1473
LmjF07.0250	*L. major*	42	1488	1529
LmjF07.0250	*L. major*	144	1597	1740
LmjF07.0260	*L. major*	43	263	305
LmjF07.0280	*L. major*	67	166	232
LmjF07.0280	*L. major*	162	334	495
LmjF07.0280	*L. major*	49	513	561
LmjF07.0280	*L. major*	42	631	672
LmjF07.0280	*L. major*	169	827	995
LmjF07.0280	*L. major*	54	1040	1093
LmjF07.0280	*L. major*	143	1281	1423
LmjF07.0280	*L. major*	62	1479	1540
LmjF07.0280	*L. major*	41	1620	1660
LmjF07.0280	*L. major*	101	1734	1834
LmjF07.0280	*L. major*	224	1951	2174
LmjF07.0280	*L. major*	93	2243	2335
LmjF07.0280	*L. major*	111	2541	2651
LmjF07.0280	*L. major*	44	2779	2822
LmjF07.0280	*L. major*	242	2933	3174
LmjF07.0280	*L. major*	86	3207	3292
LmjF07.0280	*L. major*	96	3439	3534
LmjF07.0280	*L. major*	41	3595	3635
LmjF07.0280	*L. major*	123	3807	3929
LmjF07.0280	*L. major*	83	3981	4063
LmjF07.0280	*L. major*	431	4083	4513
LmjF07.0280	*L. major*	262	4538	4799
LmjF07.0280	*L. major*	94	4884	4977
LmjF07.0280	*L. major*	154	5030	5183
LmjF07.0280	*L. major*	60	5258	5317
LmjF07.0280	*L. major*	47	5549	5595
LmjF07.0280	*L. major*	81	5765	5845
LmjF07.0290	*L. major*	41	36	76
LmjF07.0300	*L. major*	246	58	303
LmjF07.0300	*L. major*	55	434	488
LmjF07.0300	*L. major*	91	547	637
LmjF07.0310	*L. major*	101	1	101
LmjF07.0310	*L. major*	53	243	295
LmjF07.0310	*L. major*	46	301	346
LmjF07.0310	*L. major*	75	575	649

LmjF07.0310	*L. major*	88	651	738
LmjF07.0320	*L. major*	76	59	134
LmjF07.0320	*L. major*	50	510	559
LmjF07.0320	*L. major*	196	570	765
LmjF07.0320	*L. major*	69	787	855
LmjF07.0330	*L. major*	59	141	199
LmjF07.0330	*L. major*	85	263	347
LmjF07.0330	*L. major*	156	438	593
LmjF07.0335	*L. major*	58	61	118
LmjF07.0335	*L. major*	55	304	358
LmjF07.0335	*L. major*	53	428	480
LmjF07.0335	*L. major*	62	583	644
LmjF07.0335	*L. major*	71	646	716
LmjF07.0340	*L. major*	92	1	92
LmjF07.0340	*L. major*	64	529	592
LmjF07.0350	*L. major*	162	242	403
LmjF07.0350	*L. major*	91	422	512
LmjF07.0350	*L. major*	80	577	656
LmjF07.0370	*L. major*	376	1	376
LmjF07.0370	*L. major*	61	448	508
LmjF07.0370	*L. major*	141	639	779
LmjF07.0380	*L. major*	42	68	109
LmjF07.0380	*L. major*	133	162	294
LmjF07.0380	*L. major*	93	393	485
LmjF07.0380	*L. major*	90	619	708
LmjF07.0380	*L. major*	104	828	931
LmjF07.0380	*L. major*	62	969	1030
LmjF07.0380	*L. major*	88	1203	1290
LmjF07.0380	*L. major*	54	1609	1662
LmjF07.0380	*L. major*	85	2107	2191
LmjF07.0380	*L. major*	108	2251	2358
LmjF07.0380	*L. major*	48	2421	2468
LmjF07.0380	*L. major*	125	2485	2609
LmjF07.0380	*L. major*	49	2738	2786
LmjF07.0380	*L. major*	41	2799	2839
LmjF07.0380	*L. major*	52	2864	2915
LmjF07.0380	*L. major*	42	3115	3156
LmjF07.0380	*L. major*	67	3450	3516
LmjF07.0390	*L. major*	43	63	105
LmjF07.0390	*L. major*	94	124	217
LmjF07.0390	*L. major*	91	287	377
LmjF07.0390	*L. major*	77	462	538
LmjF07.0390	*L. major*	46	884	929
LmjF07.0400	*L. major*	248	127	374
LmjF07.0420	*L. major*	51	59	109
LmjF07.0470	*L. major*	68	29	96
LmjF07.0470	*L. major*	51	446	496

LmjF07.0480	*L. major*	51	2303	2353
LmjF07.0480	*L. major*	56	2368	2423
LmjF07.0480	*L. major*	43	2715	2757
LmjF07.0480	*L. major*	99	2811	2909
LmjF07.0480	*L. major*	73	3608	3680
LmjF07.0480	*L. major*	71	3718	3788
LmjF07.0480	*L. major*	43	4005	4047
LmjF07.0480	*L. major*	82	4214	4295
LmjF07.0480	*L. major*	57	4438	4494
LmjF07.0480	*L. major*	55	4727	4781
LmjF07.0490	*L. major*	41	1	41
LmjF07.0490	*L. major*	50	118	167
LmjF07.0490	*L. major*	89	212	300
LmjF07.0490	*L. major*	92	307	398
LmjF07.0500	*L. major*	64	72	135
LmjF07.0510	*L. major*	64	72	135
LmjF07.0520	*L. major*	144	29	172
LmjF07.0530	*L. major*	69	1	69
LmjF07.0540	*L. major*	163	1	163
LmjF07.0540	*L. major*	77	233	309
LmjF07.0540	*L. major*	238	323	560
LmjF07.0550	*L. major*	60	1	60
LmjF07.0550	*L. major*	57	98	154
LmjF07.0550	*L. major*	61	191	251
LmjF07.0550	*L. major*	170	353	522
LmjF07.0550	*L. major*	64	549	612
LmjF07.0550	*L. major*	110	631	740
LmjF07.0550	*L. major*	44	783	826
LmjF07.0550	*L. major*	50	871	920
LmjF07.0550	*L. major*	76	958	1033
LmjF07.0550	*L. major*	58	1071	1128
LmjF07.0550	*L. major*	89	1211	1299
LmjF07.0550	*L. major*	209	1302	1510
LmjF07.0560	*L. major*	57	169	225
LmjF07.0570	*L. major*	134	1	134
LmjF07.0570	*L. major*	241	224	464
LmjF07.0580	*L. major*	74	1	74
LmjF07.0580	*L. major*	301	85	385
LmjF07.0580	*L. major*	91	457	547
LmjF07.0580	*L. major*	67	593	659
LmjF07.0580	*L. major*	112	849	960
LmjF07.0580	*L. major*	88	976	1063
LmjF07.0590	*L. major*	366	1	366
LmjF07.0590	*L. major*	40	475	514
LmjF07.0590	*L. major*	124	754	877
LmjF07.0610	*L. major*	95	102	196
LmjF07.0615	*L. major*	54	122	175

LmjF07.0620	*L. major*	102	23	124
LmjF07.0620	*L. major*	154	130	283
LmjF07.0620	*L. major*	228	303	530
LmjF07.0620	*L. major*	40	579	618
LmjF07.0650	*L. major*	55	1	55
LmjF07.0660	*L. major*	58	25	82
LmjF07.0660	*L. major*	43	84	126
LmjF07.0660	*L. major*	43	84	126
LmjF07.0670	*L. major*	109	1	109
LmjF07.0690	*L. major*	89	1	89
LmjF07.0690	*L. major*	229	98	326
LmjF07.0690	*L. major*	72	329	400
LmjF07.0690	*L. major*	206	410	615
LmjF07.0690	*L. major*	329	670	998
LmjF07.0690	*L. major*	178	1105	1282
LmjF07.0690	*L. major*	172	1462	1633
LmjF07.0700	*L. major*	86	1	86
LmjF07.0700	*L. major*	201	110	310
LmjF07.0700	*L. major*	56	324	379
LmjF07.0700	*L. major*	56	703	758
LmjF07.0700	*L. major*	133	784	916
LmjF07.0710	*L. major*	40	1	40
LmjF07.0715	*L. major*	160	30	189
LmjF07.0715	*L. major*	107	214	320
LmjF07.0715	*L. major*	266	326	591
LmjF07.0720	*L. major*	95	861	955
LmjF07.0730	*L. major*	131	1	131
LmjF07.0730	*L. major*	48	309	356
LmjF07.0740	*L. major*	71	1	71
LmjF07.0740	*L. major*	65	181	245
LmjF07.0740	*L. major*	120	302	421
LmjF07.0740	*L. major*	108	819	926
LmjF07.0740	*L. major*	82	988	1069
LmjF07.0740	*L. major*	56	1099	1154
LmjF07.0740	*L. major*	50	1298	1347
LmjF07.0740	*L. major*	49	1406	1454
LmjF07.0740	*L. major*	156	1568	1723
LmjF07.0740	*L. major*	45	1957	2001
LmjF07.0740	*L. major*	55	2007	2061
LmjF07.0740	*L. major*	42	2109	2150
LmjF07.0740	*L. major*	62	2212	2273
LmjF07.0740	*L. major*	41	2382	2422
LmjF07.0740	*L. major*	69	2490	2558
LmjF07.0740	*L. major*	120	2579	2698
LmjF07.0740	*L. major*	50	2737	2786
LmjF07.0740	*L. major*	117	2897	3013
LmjF07.0740	*L. major*	92	3080	3171

LmjF07.0740	*L. major*	48	3198	3245
LmjF07.0740	*L. major*	45	3288	3332
LmjF07.0740	*L. major*	316	3457	3772
LmjF07.0743	*L. major*	138	1	138
LmjF07.0750	*L. major*	129	49	177
LmjF07.0760	*L. major*	183	1	183
LmjF07.0760	*L. major*	157	264	420
LmjF07.0760	*L. major*	137	476	612
LmjF07.0760	*L. major*	122	648	769
LmjF07.0760	*L. major*	71	878	948
LmjF07.0760	*L. major*	59	1027	1085
LmjF07.0770	*L. major*	143	1	143
LmjF07.0780	*L. major*	48	1	48
LmjF07.0780	*L. major*	187	97	283
LmjF07.0790	*L. major*	239	1	239
LmjF07.0800	*L. major*	55	380	434
LmjF07.0800	*L. major*	46	551	596
LmjF07.0800	*L. major*	46	551	596
LmjF07.0802	*L. major*	132	71	202
LmjF07.0802	*L. major*	123	230	352
LmjF07.0820	*L. major*	59	437	495
LmjF07.0820	*L. major*	74	725	798
LmjF07.0820	*L. major*	52	1427	1478
LmjF07.0820	*L. major*	43	1933	1975
LmjF07.0830	*L. major*	47	425	471
LmjF07.0830	*L. major*	43	630	672
LmjF07.0830	*L. major*	52	1412	1463
LmjF07.0830	*L. major*	46	1869	1914
LmjF07.0830	*L. major*	414	1971	2384
LmjF07.0840	*L. major*	52	1410	1461
LmjF07.0840	*L. major*	513	1831	2343
LmjF07.0850	*L. major*	52	1	52
LmjF07.0860	*L. major*	132	1	132
LmjF07.0860	*L. major*	1003	147	1149
LmjF07.0860	*L. major*	116	1177	1292
LmjF07.0860	*L. major*	102	1322	1423
LmjF07.0870	*L. major*	76	1	76
LmjF07.0870	*L. major*	40	141	180
LmjF07.0870	*L. major*	126	247	372
LmjF07.0880	*L. major*	78	44	121
LmjF07.0880	*L. major*	502	142	643
LmjF07.0880	*L. major*	68	653	720
LmjF07.0880	*L. major*	47	734	780
LmjF07.0880	*L. major*	85	889	973
LmjF07.0880	*L. major*	49	977	1025
LmjF07.0880	*L. major*	41	1176	1216
LmjF07.0890	*L. major*	75	1	75

LmjF07.0890	*L. major*	176	80	255
LmjF07.0890	*L. major*	117	323	439
LmjF07.0890	*L. major*	187	443	629
LmjF07.0910	*L. major*	351	1	351
LmjF07.0910	*L. major*	259	357	615
LmjF07.0910	*L. major*	47	788	834
LmjF07.0910	*L. major*	57	925	981
LmjF07.0910	*L. major*	138	1242	1379
LmjF07.0910	*L. major*	79	1390	1468
LmjF07.0910	*L. major*	154	1691	1844
LmjF07.0920	*L. major*	44	1	44
LmjF07.0920	*L. major*	82	167	248
LmjF07.0920	*L. major*	85	387	471
LmjF07.0920	*L. major*	43	580	622
LmjF07.0920	*L. major*	88	670	757
LmjF07.0920	*L. major*	144	812	955
LmjF07.0920	*L. major*	42	1110	1151
LmjF07.0920	*L. major*	63	1188	1250
LmjF07.0920	*L. major*	86	1315	1400
LmjF07.0920	*L. major*	280	1455	1734
LmjF07.0930	*L. major*	83	103	185
LmjF07.0940	*L. major*	62	1	62
LmjF07.0940	*L. major*	77	382	458
LmjF07.0940	*L. major*	56	505	560
LmjF07.0940	*L. major*	67	611	677
LmjF07.0940	*L. major*	82	1820	1901
LmjF07.0940	*L. major*	40	2322	2361
LmjF07.0950	*L. major*	209	1	209
LmjF07.0950	*L. major*	102	245	346
LmjF07.0950	*L. major*	140	419	558
LmjF07.0950	*L. major*	250	684	933
LmjF07.0950	*L. major*	174	1138	1311
LmjF07.0950	*L. major*	45	1326	1370
LmjF07.0950	*L. major*	195	1493	1687
LmjF07.0950	*L. major*	65	1730	1794
LmjF07.0960	*L. major*	285	78	362
LmjF07.0960	*L. major*	742	393	1134
LmjF07.0970	*L. major*	78	43	120
LmjF07.0970	*L. major*	162	161	322
LmjF07.0970	*L. major*	133	672	804
LmjF07.0990	*L. major*	265	88	352
LmjF07.1000	*L. major*	237	92	328
LmjF07.1010	*L. major*	72	49	120
LmjF07.1020	*L. major*	258	1	258
LmjF07.1020	*L. major*	44	484	527
LmjF07.1020	*L. major*	133	819	951
LmjF07.1020	*L. major*	50	1023	1072

LmjF07.1040	*L. major*	40	1	40
LmjF07.1050	*L. major*	124	871	994
LmjF07.1055	*L. major*	46	1	46
LmjF07.1070	*L. major*	54	1	54
LmjF07.1070	*L. major*	127	141	267
LmjF07.1070	*L. major*	90	284	373
LmjF07.1080	*L. major*	231	307	537
LmjF07.1080	*L. major*	56	668	723
LmjF07.1100	*L. major*	84	1	84
LmjF07.1100	*L. major*	144	163	306
LmjF07.1110	*L. major*	104	215	318
LmjF07.1120	*L. major*	160	187	346
LmjF07.1150	*L. major*	146	41	186
LmjF07.1150	*L. major*	81	1092	1172
LmjF07.1150	*L. major*	54	1272	1325
LmjF07.1150	*L. major*	43	1424	1466
LmjF07.1150	*L. major*	50	1553	1602
LmjF07.1160	*L. major*	83	1	83
LmjF07.1170	*L. major*	92	1	92
LmjF07.1170	*L. major*	55	133	187
LmjF08.0010	*L. major*	51	176	226
LmjF08.0010	*L. major*	89	235	323
LmjF08.0010	*L. major*	177	330	506
LmjF08.0010	*L. major*	165	674	838
LmjF08.0010	*L. major*	83	855	937
LmjF08.0010	*L. major*	59	967	1025
LmjF08.0040	*L. major*	67	61	127
LmjF08.0070	*L. major*	80	70	149
LmjF08.0070	*L. major*	79	201	279
LmjF08.0070	*L. major*	181	294	474
LmjF08.0080	*L. major*	147	418	564
LmjF08.0090	*L. major*	41	545	585
LmjF08.0090	*L. major*	175	649	823
LmjF08.0090	*L. major*	175	649	823
LmjF08.0100	*L. major*	47	1	47
LmjF08.0100	*L. major*	75	252	326
LmjF08.0100	*L. major*	66	386	451
LmjF08.0110	*L. major*	109	51	159
LmjF08.0110	*L. major*	101	171	271
LmjF08.0110	*L. major*	266	378	643
LmjF08.0120	*L. major*	44	198	241
LmjF08.0130	*L. major*	95	1	95
LmjF08.0130	*L. major*	171	146	316
LmjF08.0130	*L. major*	418	347	764
LmjF08.0130	*L. major*	373	774	1146
LmjF08.0140	*L. major*	58	304	361
LmjF08.0140	*L. major*	58	524	581

LmjF08.0140	*L. major*	85	685	769
LmjF08.0140	*L. major*	61	791	851
LmjF08.0140	*L. major*	48	859	906
LmjF08.0140	*L. major*	63	959	1021
LmjF08.0150	*L. major*	61	76	136
LmjF08.0150	*L. major*	55	386	440
LmjF08.0150	*L. major*	55	386	440
LmjF08.0160	*L. major*	89	14	102
LmjF08.0170	*L. major*	51	1	51
LmjF08.0170	*L. major*	109	236	344
LmjF08.0170	*L. major*	109	236	344
LmjF08.0180	*L. major*	60	235	294
LmjF08.0190	*L. major*	48	195	242
LmjF08.0190	*L. major*	53	581	633
LmjF08.0200	*L. major*	56	540	595
LmjF08.0210	*L. major*	400	1	400
LmjF08.0210	*L. major*	40	422	461
LmjF08.0220	*L. major*	98	236	333
LmjF08.0220	*L. major*	49	339	387
LmjF08.0220	*L. major*	74	473	546
LmjF08.0220	*L. major*	97	730	826
LmjF08.0240	*L. major*	50	1	50
LmjF08.0240	*L. major*	51	212	262
LmjF08.0240	*L. major*	51	212	262
LmjF08.0250	*L. major*	40	1	40
LmjF08.0250	*L. major*	50	100	149
LmjF08.0250	*L. major*	50	100	149
LmjF08.0260	*L. major*	384	1	384
LmjF08.0270	*L. major*	134	1	134
LmjF08.0270	*L. major*	97	229	325
LmjF08.0270	*L. major*	196	370	565
LmjF08.0270	*L. major*	138	584	721
LmjF08.0270	*L. major*	45	752	796
LmjF08.0280	*L. major*	43	283	325
LmjF08.0310	*L. major*	66	1	66
LmjF08.0310	*L. major*	52	260	311
LmjF08.0310	*L. major*	52	260	311
LmjF08.0340	*L. major*	153	1	153
LmjF08.0340	*L. major*	50	548	597
LmjF08.0340	*L. major*	129	621	749
LmjF08.0340	*L. major*	56	826	881
LmjF08.0340	*L. major*	48	1134	1181
LmjF08.0340	*L. major*	84	1209	1292
LmjF08.0340	*L. major*	42	1311	1352
LmjF08.0340	*L. major*	91	1404	1494
LmjF08.0340	*L. major*	48	1745	1792
LmjF08.0340	*L. major*	138	1813	1950

LmjF08.0340	*L. major*	75	2303	2377
LmjF08.0340	*L. major*	53	2448	2500
LmjF08.0340	*L. major*	139	2644	2782
LmjF08.0340	*L. major*	119	2995	3113
LmjF08.0340	*L. major*	80	3148	3227
LmjF08.0340	*L. major*	133	3253	3385
LmjF08.0340	*L. major*	42	3434	3475
LmjF08.0340	*L. major*	129	3600	3728
LmjF08.0340	*L. major*	126	4150	4275
LmjF08.0340	*L. major*	54	4367	4420
LmjF08.0340	*L. major*	47	4454	4500
LmjF08.0340	*L. major*	42	4615	4656
LmjF08.0340	*L. major*	75	4677	4751
LmjF08.0350	*L. major*	262	79	340
LmjF08.0350	*L. major*	445	383	827
LmjF08.0350	*L. major*	125	948	1072
LmjF08.0350	*L. major*	50	1219	1268
LmjF08.0350	*L. major*	45	1282	1326
LmjF08.0360	*L. major*	143	195	337
LmjF08.0360	*L. major*	233	399	631
LmjF08.0370	*L. major*	108	1	108
LmjF08.0380	*L. major*	144	1	144
LmjF08.0380	*L. major*	194	195	388
LmjF08.0380	*L. major*	175	413	587
LmjF08.0380	*L. major*	87	688	774
LmjF08.0380	*L. major*	73	884	956
LmjF08.0380	*L. major*	248	1149	1396
LmjF08.0380	*L. major*	324	1490	1813
LmjF08.0390	*L. major*	70	18	87
LmjF08.0390	*L. major*	370	110	479
LmjF08.0390	*L. major*	106	502	607
LmjF08.0390	*L. major*	59	638	696
LmjF08.0390	*L. major*	53	736	788
LmjF08.0390	*L. major*	130	824	953
LmjF08.0390	*L. major*	48	1021	1068
LmjF08.0390	*L. major*	77	1082	1158
LmjF08.0390	*L. major*	42	1199	1240
LmjF08.0390	*L. major*	162	1264	1425
LmjF08.0390	*L. major*	95	1431	1525
LmjF08.0390	*L. major*	258	1546	1803
LmjF08.0400	*L. major*	83	1	83
LmjF08.0400	*L. major*	250	124	373
LmjF08.0400	*L. major*	97	513	609
LmjF08.0400	*L. major*	51	681	731
LmjF08.0400	*L. major*	53	744	796
LmjF08.0400	*L. major*	159	957	1115
LmjF08.0410	*L. major*	45	21	65

LmjF08.0410	*L. major*	78	160	237
LmjF08.0410	*L. major*	56	354	409
LmjF08.0410	*L. major*	41	978	1018
LmjF08.0420	*L. major*	60	1	60
LmjF08.0420	*L. major*	117	312	428
LmjF08.0420	*L. major*	257	450	706
LmjF08.0420	*L. major*	44	731	774
LmjF08.0420	*L. major*	69	785	853
LmjF08.0420	*L. major*	55	966	1020
LmjF08.0420	*L. major*	93	1370	1462
LmjF08.0420	*L. major*	108	1464	1571
LmjF08.0420	*L. major*	106	1759	1864
LmjF08.0430	*L. major*	192	84	275
LmjF08.0430	*L. major*	170	329	498
LmjF08.0430	*L. major*	91	509	599
LmjF08.0430	*L. major*	144	638	781
LmjF08.0440	*L. major*	43	102	144
LmjF08.0440	*L. major*	51	193	243
LmjF08.0440	*L. major*	183	484	666
LmjF08.0440	*L. major*	54	785	838
LmjF08.0440	*L. major*	73	979	1051
LmjF08.0440	*L. major*	67	1460	1526
LmjF08.0440	*L. major*	75	1776	1850
LmjF08.0440	*L. major*	46	1967	2012
LmjF08.0440	*L. major*	65	2230	2294
LmjF08.0440	*L. major*	47	2388	2434
LmjF08.0480	*L. major*	56	61	116
LmjF08.0480	*L. major*	114	169	282
LmjF08.0490	*L. major*	220	1	220
LmjF08.0500	*L. major*	92	1	92
LmjF08.0500	*L. major*	81	105	185
LmjF08.0500	*L. major*	69	202	270
LmjF08.0500	*L. major*	50	282	331
LmjF08.0510	*L. major*	46	86	131
LmjF08.0510	*L. major*	81	560	640
LmjF08.0510	*L. major*	54	654	707
LmjF08.0520	*L. major*	99	352	450
LmjF08.0520	*L. major*	100	532	631
LmjF08.0520	*L. major*	60	660	719
LmjF08.0520	*L. major*	58	917	974
LmjF08.0520	*L. major*	98	1013	1110
LmjF08.0520	*L. major*	109	1148	1256
LmjF08.0520	*L. major*	68	1523	1590
LmjF08.0520	*L. major*	64	1599	1662
LmjF08.0520	*L. major*	144	1772	1915
LmjF08.0520	*L. major*	153	1919	2071
LmjF08.0520	*L. major*	51	2102	2152

LmjF08.0520	*L. major*	56	2272	2327
LmjF08.0530	*L. major*	413	84	496
LmjF08.0530	*L. major*	115	551	665
LmjF08.0530	*L. major*	308	685	992
LmjF08.0530	*L. major*	192	999	1190
LmjF08.0530	*L. major*	169	1199	1367
LmjF08.0540	*L. major*	68	47	114
LmjF08.0540	*L. major*	48	142	189
LmjF08.0540	*L. major*	85	268	352
LmjF08.0550	*L. major*	210	1	210
LmjF08.0560	*L. major*	42	1	42
LmjF08.0560	*L. major*	41	104	144
LmjF08.0560	*L. major*	41	104	144
LmjF08.0570	*L. major*	64	116	179
LmjF08.0580	*L. major*	57	91	147
LmjF08.0590	*L. major*	132	68	199
LmjF08.0590	*L. major*	150	209	358
LmjF08.0590	*L. major*	67	407	473
LmjF08.0600	*L. major*	52	92	143
LmjF08.0620	*L. major*	119	66	184
LmjF08.0620	*L. major*	47	200	246
LmjF08.0620	*L. major*	134	299	432
LmjF08.0620	*L. major*	245	477	721
LmjF08.0620	*L. major*	62	903	964
LmjF08.0620	*L. major*	100	1045	1144
LmjF08.0620	*L. major*	56	1173	1228
LmjF08.0620	*L. major*	1514	1309	2822
LmjF08.0630	*L. major*	119	149	267
LmjF08.0630	*L. major*	151	358	508
LmjF08.0630	*L. major*	420	662	1081
LmjF08.0630	*L. major*	56	1295	1350
LmjF08.0630	*L. major*	338	1520	1857
LmjF08.0630	*L. major*	202	1942	2143
LmjF08.0650	*L. major*	193	73	265
LmjF08.0660	*L. major*	50	505	554
LmjF08.0660	*L. major*	85	831	915
LmjF08.0660	*L. major*	85	831	915
LmjF08.0790	*L. major*	48	1	48
LmjF08.0790	*L. major*	41	226	266
LmjF08.0790	*L. major*	41	226	266
LmjF08.0795	*L. major*	93	302	394
LmjF08.0860	*L. major*	290	1	290
LmjF08.0870	*L. major*	75	73	147
LmjF08.0870	*L. major*	43	185	227
LmjF08.0870	*L. major*	92	308	399
LmjF08.0880	*L. major*	58	83	140
LmjF08.0880	*L. major*	43	291	333

LmjF08.0880	*L. major*	53	400	452
LmjF08.0890	*L. major*	63	34	96
LmjF08.0890	*L. major*	44	333	376
LmjF08.0890	*L. major*	44	333	376
LmjF08.0900	*L. major*	136	1	136
LmjF08.0900	*L. major*	44	189	232
LmjF08.0900	*L. major*	75	573	647
LmjF08.0910	*L. major*	125	29	153
LmjF08.0910	*L. major*	44	190	233
LmjF08.0910	*L. major*	79	261	339
LmjF08.0910	*L. major*	104	403	506
LmjF08.0910	*L. major*	52	559	610
LmjF08.0910	*L. major*	61	619	679
LmjF08.0910	*L. major*	56	689	744
LmjF08.0910	*L. major*	100	808	907
LmjF08.0910	*L. major*	56	956	1011
LmjF08.0910	*L. major*	179	1029	1207
LmjF08.0910	*L. major*	176	1269	1444
LmjF08.0910	*L. major*	92	1538	1629
LmjF08.0910	*L. major*	145	1711	1855
LmjF08.0910	*L. major*	95	1925	2019
LmjF08.0910	*L. major*	72	2052	2123
LmjF08.0920	*L. major*	47	158	204
LmjF08.0930	*L. major*	536	321	856
LmjF08.0930	*L. major*	88	958	1045
LmjF08.0940	*L. major*	56	121	176
LmjF08.0940	*L. major*	41	199	239
LmjF08.0950	*L. major*	149	1	149
LmjF08.0960	*L. major*	153	41	193
LmjF08.0960	*L. major*	47	196	242
LmjF08.0960	*L. major*	44	317	360
LmjF08.0960	*L. major*	54	380	433
LmjF08.0960	*L. major*	54	470	523
LmjF08.0970	*L. major*	147	22	168
LmjF08.0970	*L. major*	207	223	429
LmjF08.0970	*L. major*	61	550	610
LmjF08.0980	*L. major*	93	1	93
LmjF08.0980	*L. major*	925	117	1041
LmjF08.0990	*L. major*	49	86	134
LmjF08.0990	*L. major*	117	210	326
LmjF08.0990	*L. major*	117	210	326
LmjF08.1000	*L. major*	126	293	418
LmjF08.1000	*L. major*	60	505	564
LmjF08.1000	*L. major*	49	994	1042
LmjF08.1000	*L. major*	68	1422	1489
LmjF08.1000	*L. major*	42	1505	1546
LmjF08.1000	*L. major*	49	1809	1857

LmjF08.1000	*L. major*	79	1859	1937
LmjF08.1000	*L. major*	80	1978	2057
LmjF08.1000	*L. major*	62	2067	2128
LmjF08.1000	*L. major*	58	2138	2195
LmjF08.1090	*L. major*	66	441	506
LmjF08.1100	*L. major*	45	54	98
LmjF08.1100	*L. major*	68	298	365
LmjF08.1100	*L. major*	68	298	365
LmjF08.1110	*L. major*	60	184	243
LmjF08.1110	*L. major*	43	334	376
LmjF08.1110	*L. major*	65	469	533
LmjF08.1120	*L. major*	65	43	107
LmjF08.1120	*L. major*	50	428	477
LmjF08.1120	*L. major*	43	702	744
LmjF08.1140	*L. major*	338	380	717
LmjF08.1140	*L. major*	43	749	791
LmjF08.1150	*L. major*	54	221	274
LmjF08.1150	*L. major*	74	469	542
LmjF08.1150	*L. major*	147	632	778
LmjF08.1160	*L. major*	79	127	205
LmjF08.1180	*L. major*	58	95	152
LmjF08.1180	*L. major*	106	265	370
LmjF08.1180	*L. major*	106	265	370
LmjF08.1190	*L. major*	97	1060	1156
LmjF08.1210	*L. major*	59	151	209
LmjF08.1215	*L. major*	150	117	266
LmjF08.1215	*L. major*	151	356	506
LmjF08.1218	*L. major*	160	1	160
LmjF08.1218	*L. major*	99	163	261
LmjF08.1218	*L. major*	119	361	479
LmjF08.1228	*L. major*	89	193	281
LmjF08.1228	*L. major*	43	513	555
LmjF08.1228	*L. major*	43	513	555
LmjF08.1230	*L. major*	40	404	443
LmjF08.1240	*L. major*	150	117	266
LmjF08.1240	*L. major*	151	356	506
LmjF08.1250	*L. major*	160	1	160
LmjF08.1250	*L. major*	99	163	261
LmjF08.1250	*L. major*	119	361	479
LmjF08.1280	*L. major*	89	193	281
LmjF08.1280	*L. major*	43	513	555
LmjF08.1280	*L. major*	43	513	555
LmjF09.0003	*L. major*	104	1	104
LmjF09.0020	*L. major*	42	251	292
LmjF09.0020	*L. major*	86	478	563
LmjF09.0020	*L. major*	86	478	563
LmjF09.0030	*L. major*	57	196	252

LmjF09.0060	*L. major*	46	86	131
LmjF09.0070	*L. major*	88	209	296
LmjF09.0070	*L. major*	44	527	570
LmjF09.0070	*L. major*	44	527	570
LmjF09.0080	*L. major*	82	107	188
LmjF09.0090	*L. major*	63	37	99
LmjF09.0090	*L. major*	90	177	266
LmjF09.0090	*L. major*	90	177	266
LmjF09.0100	*L. major*	87	92	178
LmjF09.0100	*L. major*	323	260	582
LmjF09.0105	*L. major*	64	1	64
LmjF09.0110	*L. major*	235	1	235
LmjF09.0110	*L. major*	201	270	470
LmjF09.0110	*L. major*	188	525	712
LmjF09.0120	*L. major*	59	157	215
LmjF09.0120	*L. major*	151	425	575
LmjF09.0120	*L. major*	101	620	720
LmjF09.0120	*L. major*	144	737	880
LmjF09.0120	*L. major*	138	928	1065
LmjF09.0140	*L. major*	59	246	304
LmjF09.0190	*L. major*	43	78	120
LmjF09.0200	*L. major*	41	66	106
LmjF09.0210	*L. major*	78	52	129
LmjF09.0210	*L. major*	91	187	277
LmjF09.0210	*L. major*	91	187	277
LmjF09.0220	*L. major*	44	570	613
LmjF09.0230	*L. major*	59	169	227
LmjF09.0240	*L. major*	92	75	166
LmjF09.0240	*L. major*	122	279	400
LmjF09.0240	*L. major*	70	752	821
LmjF09.0250	*L. major*	75	606	680
LmjF09.0260	*L. major*	110	1	110
LmjF09.0260	*L. major*	89	181	269
LmjF09.0270	*L. major*	71	274	344
LmjF09.0270	*L. major*	60	562	621
LmjF09.0270	*L. major*	60	562	621
LmjF09.0280	*L. major*	71	132	202
LmjF09.0280	*L. major*	185	254	438
LmjF09.0280	*L. major*	46	471	516
LmjF09.0280	*L. major*	116	602	717
LmjF09.0290	*L. major*	87	247	333
LmjF09.0290	*L. major*	96	403	498
LmjF09.0290	*L. major*	67	508	574
LmjF09.0290	*L. major*	644	621	1264
LmjF09.0300	*L. major*	60	53	112
LmjF09.0300	*L. major*	79	127	205
LmjF09.0300	*L. major*	79	127	205

LmjF09.0310	*L. major*	452	1	452
LmjF09.0310	*L. major*	44	697	740
LmjF09.0320	*L. major*	85	1	85
LmjF09.0320	*L. major*	135	641	775
LmjF09.0330	*L. major*	162	546	707
LmjF09.0340	*L. major*	218	588	805
LmjF09.0340	*L. major*	65	839	903
LmjF09.0340	*L. major*	60	1005	1064
LmjF09.0345	*L. major*	63	105	167
LmjF09.0345	*L. major*	60	191	250
LmjF09.0345	*L. major*	60	191	250
LmjF09.0350	*L. major*	237	1	237
LmjF09.0350	*L. major*	344	245	588
LmjF09.0350	*L. major*	179	633	811
LmjF09.0360	*L. major*	131	1	131
LmjF09.0360	*L. major*	61	164	224
LmjF09.0365	*L. major*	82	1	82
LmjF09.0370	*L. major*	88	1	88
LmjF09.0380	*L. major*	62	1	62
LmjF09.0380	*L. major*	164	157	320
LmjF09.0390	*L. major*	303	65	367
LmjF09.0390	*L. major*	220	499	718
LmjF09.0390	*L. major*	52	751	802
LmjF09.0390	*L. major*	113	1033	1145
LmjF09.0390	*L. major*	160	1164	1323
LmjF09.0390	*L. major*	57	1456	1512
LmjF09.0390	*L. major*	193	1566	1758
LmjF09.0390	*L. major*	83	1830	1912
LmjF09.0400	*L. major*	61	1	61
LmjF09.0410	*L. major*	49	1	49
LmjF09.0420	*L. major*	54	101	154
LmjF09.0420	*L. major*	79	350	428
LmjF09.0420	*L. major*	146	494	639
LmjF09.0420	*L. major*	91	648	738
LmjF09.0420	*L. major*	174	761	934
LmjF09.0420	*L. major*	57	941	997
LmjF09.0430	*L. major*	233	145	377
LmjF09.0440	*L. major*	81	35	115
LmjF09.0440	*L. major*	63	183	245
LmjF09.0450	*L. major*	102	110	211
LmjF09.0450	*L. major*	264	281	544
LmjF09.0450	*L. major*	56	571	626
LmjF09.0450	*L. major*	72	643	714
LmjF09.0450	*L. major*	64	750	813
LmjF09.0450	*L. major*	104	831	934
LmjF09.0460	*L. major*	50	1	50
LmjF09.0460	*L. major*	83	95	177

LmjF09.0460	*L. major*	123	313	435
LmjF09.0460	*L. major*	49	594	642
LmjF09.0460	*L. major*	120	667	786
LmjF09.0460	*L. major*	66	819	884
LmjF09.0460	*L. major*	142	1052	1193
LmjF09.0460	*L. major*	87	1520	1606
LmjF09.0460	*L. major*	59	1688	1746
LmjF09.0460	*L. major*	59	1808	1866
LmjF09.0460	*L. major*	45	1941	1985
LmjF09.0460	*L. major*	40	2226	2265
LmjF09.0460	*L. major*	79	2447	2525
LmjF09.0460	*L. major*	78	2721	2798
LmjF09.0460	*L. major*	72	2838	2909
LmjF09.0470	*L. major*	113	1	113
LmjF09.0470	*L. major*	112	142	253
LmjF09.0480	*L. major*	91	1	91
LmjF09.0480	*L. major*	264	291	554
LmjF09.0490	*L. major*	42	322	363
LmjF09.0500	*L. major*	63	1	63
LmjF09.0500	*L. major*	46	142	187
LmjF09.0500	*L. major*	47	211	257
LmjF09.0500	*L. major*	53	312	364
LmjF09.0500	*L. major*	86	391	476
LmjF09.0500	*L. major*	87	491	577
LmjF09.0500	*L. major*	63	854	916
LmjF09.0500	*L. major*	106	933	1038
LmjF09.0500	*L. major*	65	1178	1242
LmjF09.0500	*L. major*	259	1245	1503
LmjF09.0510	*L. major*	50	166	215
LmjF09.0510	*L. major*	97	227	323
LmjF09.0520	*L. major*	65	164	228
LmjF09.0520	*L. major*	101	602	702
LmjF09.0520	*L. major*	61	711	771
LmjF09.0530	*L. major*	113	1	113
LmjF09.0530	*L. major*	49	129	177
LmjF09.0530	*L. major*	198	800	997
LmjF09.0540	*L. major*	66	1	66
LmjF09.0540	*L. major*	688	79	766
LmjF09.0540	*L. major*	185	817	1001
LmjF09.0550	*L. major*	91	40	130
LmjF09.0550	*L. major*	78	182	259
LmjF09.0550	*L. major*	74	275	348
LmjF09.0550	*L. major*	45	439	483
LmjF09.0560	*L. major*	41	138	178
LmjF09.0570	*L. major*	72	88	159
LmjF09.0570	*L. major*	143	210	352
LmjF09.0590	*L. major*	220	596	815

LmjF09.0610	*L. major*	95	23	117
LmjF09.0610	*L. major*	40	533	572
LmjF09.0610	*L. major*	41	688	728
LmjF09.0620	*L. major*	86	1	86
LmjF09.0620	*L. major*	113	125	237
LmjF09.0620	*L. major*	65	381	445
LmjF09.0620	*L. major*	43	520	562
LmjF09.0630	*L. major*	110	50	159
LmjF09.0630	*L. major*	42	360	401
LmjF09.0630	*L. major*	52	622	673
LmjF09.0630	*L. major*	91	688	778
LmjF09.0630	*L. major*	291	780	1070
LmjF09.0630	*L. major*	78	1084	1161
LmjF09.0640	*L. major*	48	141	188
LmjF09.0640	*L. major*	54	408	461
LmjF09.0640	*L. major*	54	408	461
LmjF09.0650	*L. major*	65	465	529
LmjF09.0660	*L. major*	70	1	70
LmjF09.0660	*L. major*	44	178	221
LmjF09.0670	*L. major*	48	1	48
LmjF09.0670	*L. major*	42	97	138
LmjF09.0670	*L. major*	42	97	138
LmjF09.0680	*L. major*	71	60	130
LmjF09.0680	*L. major*	45	182	226
LmjF09.0680	*L. major*	79	259	337
LmjF09.0700	*L. major*	189	1	189
LmjF09.0700	*L. major*	189	268	456
LmjF09.0710	*L. major*	84	1	84
LmjF09.0710	*L. major*	61	105	165
LmjF09.0710	*L. major*	103	214	316
LmjF09.0710	*L. major*	62	338	399
LmjF09.0710	*L. major*	56	428	483
LmjF09.0720	*L. major*	132	208	339
LmjF09.0730	*L. major*	87	417	503
LmjF09.0740	*L. major*	45	278	322
LmjF09.0740	*L. major*	63	894	956
LmjF09.0740	*L. major*	45	1244	1288
LmjF09.0740	*L. major*	64	1330	1393
LmjF09.0740	*L. major*	79	1546	1624
LmjF09.0750	*L. major*	40	155	194
LmjF09.0750	*L. major*	41	218	258
LmjF09.0750	*L. major*	55	428	482
LmjF09.0750	*L. major*	94	788	881
LmjF09.0760	*L. major*	51	42	92
LmjF09.0760	*L. major*	112	106	217
LmjF09.0760	*L. major*	77	271	347
LmjF09.0760	*L. major*	40	694	733

LmjF09.0760	*L. major*	45	742	786
LmjF09.0760	*L. major*	108	821	928
LmjF09.0760	*L. major*	71	961	1031
LmjF09.0760	*L. major*	53	1241	1293
LmjF09.0760	*L. major*	104	1391	1494
LmjF09.0760	*L. major*	57	1585	1641
LmjF09.0760	*L. major*	45	1769	1813
LmjF09.0760	*L. major*	48	1828	1875
LmjF09.0760	*L. major*	50	2008	2057
LmjF09.0760	*L. major*	64	2195	2258
LmjF09.0760	*L. major*	69	2264	2332
LmjF09.0760	*L. major*	88	2362	2449
LmjF09.0760	*L. major*	55	2538	2592
LmjF09.0760	*L. major*	80	2644	2723
LmjF09.0760	*L. major*	61	2802	2862
LmjF09.0760	*L. major*	184	2990	3173
LmjF09.0760	*L. major*	99	3228	3326
LmjF09.0760	*L. major*	50	3610	3659
LmjF09.0760	*L. major*	75	3745	3819
LmjF09.0760	*L. major*	80	3839	3918
LmjF09.0760	*L. major*	75	4241	4315
LmjF09.0760	*L. major*	150	4504	4653
LmjF09.0760	*L. major*	61	4709	4769
LmjF09.0760	*L. major*	77	4891	4967
LmjF09.0760	*L. major*	90	4995	5084
LmjF09.0760	*L. major*	262	5120	5381
LmjF09.0760	*L. major*	57	5425	5481
LmjF09.0770	*L. major*	60	1	60
LmjF09.0790	*L. major*	91	1	91
LmjF09.0790	*L. major*	241	99	339
LmjF09.0790	*L. major*	83	364	446
LmjF09.0790	*L. major*	81	469	549
LmjF09.0790	*L. major*	45	655	699
LmjF09.0790	*L. major*	77	1075	1151
LmjF09.0800	*L. major*	191	10	200
LmjF09.0800	*L. major*	185	306	490
LmjF09.0800	*L. major*	66	526	591
LmjF09.0800	*L. major*	308	678	985
LmjF09.0800	*L. major*	210	1030	1239
LmjF09.0810	*L. major*	45	84	128
LmjF09.0820	*L. major*	88	257	344
LmjF09.0820	*L. major*	41	490	530
LmjF09.0830	*L. major*	53	1	53
LmjF09.0830	*L. major*	60	410	469
LmjF09.0830	*L. major*	60	410	469
LmjF09.0850	*L. major*	54	1	54
LmjF09.0850	*L. major*	62	206	267

LmjF09.0850	*L. major*	62	206	267
LmjF09.0870	*L. major*	86	1	86
LmjF09.0870	*L. major*	75	106	180
LmjF09.0870	*L. major*	169	194	362
LmjF09.0870	*L. major*	50	370	419
LmjF09.0880	*L. major*	76	1	76
LmjF09.0880	*L. major*	368	117	484
LmjF09.0890	*L. major*	136	163	298
LmjF09.0890	*L. major*	283	435	717
LmjF09.0890	*L. major*	87	998	1084
LmjF09.0890	*L. major*	86	1086	1171
LmjF09.0890	*L. major*	105	1217	1321
LmjF09.0890	*L. major*	63	1346	1408
LmjF09.0890	*L. major*	64	1449	1512
LmjF09.0890	*L. major*	223	1582	1804
LmjF09.0890	*L. major*	89	1971	2059
LmjF09.0890	*L. major*	64	2462	2525
LmjF09.0900	*L. major*	63	105	167
LmjF09.0910	*L. major*	89	1	89
LmjF09.0920	*L. major*	89	1	89
LmjF09.0930	*L. major*	89	1	89
LmjF09.0940	*L. major*	56	168	223
LmjF09.0940	*L. major*	47	529	575
LmjF09.0940	*L. major*	325	746	1070
LmjF09.0940	*L. major*	64	1137	1200
LmjF09.0950	*L. major*	53	158	210
LmjF09.0950	*L. major*	45	248	292
LmjF09.0950	*L. major*	63	447	509
LmjF09.0960	*L. major*	43	33	75
LmjF09.0960	*L. major*	90	457	546
LmjF09.0960	*L. major*	90	457	546
LmjF09.0970	*L. major*	62	203	264
LmjF09.0980	*L. major*	277	12	288
LmjF09.0980	*L. major*	62	307	368
LmjF09.0980	*L. major*	104	439	542
LmjF09.0980	*L. major*	394	560	953
LmjF09.0990	*L. major*	44	211	254
LmjF09.0990	*L. major*	49	1045	1093
LmjF09.0990	*L. major*	118	1165	1282
LmjF09.0990	*L. major*	93	1734	1826
LmjF09.0990	*L. major*	48	1912	1959
LmjF09.0990	*L. major*	141	1979	2119
LmjF09.0990	*L. major*	77	2158	2234
LmjF09.0990	*L. major*	77	2324	2400
LmjF09.0990	*L. major*	76	2611	2686
LmjF09.0990	*L. major*	185	2828	3012
LmjF09.0990	*L. major*	177	3139	3315

LmjF09.0990	*L. major*	201	3532	3732
LmjF09.0990	*L. major*	48	4209	4256
LmjF09.0990	*L. major*	86	4693	4778
LmjF09.0990	*L. major*	41	4867	4907
LmjF09.0990	*L. major*	165	4909	5073
LmjF09.0990	*L. major*	45	5153	5197
LmjF09.0990	*L. major*	75	5307	5381
LmjF09.1000	*L. major*	58	125	182
LmjF09.1010	*L. major*	127	28	154
LmjF09.1010	*L. major*	48	486	533
LmjF09.1020	*L. major*	106	1	106
LmjF09.1030	*L. major*	109	1	109
LmjF09.1030	*L. major*	155	111	265
LmjF09.1040	*L. major*	94	1	94
LmjF09.1050	*L. major*	69	138	206
LmjF09.1060	*L. major*	49	144	192
LmjF09.1090	*L. major*	58	392	449
LmjF09.1100	*L. major*	41	1	41
LmjF09.1100	*L. major*	183	44	226
LmjF09.1100	*L. major*	183	44	226
LmjF09.1110	*L. major*	183	1	183
LmjF09.1110	*L. major*	240	219	458
LmjF09.1120	*L. major*	55	25	79
LmjF09.1140	*L. major*	61	1	61
LmjF09.1140	*L. major*	43	144	186
LmjF09.1150	*L. major*	58	1	58
LmjF09.1150	*L. major*	70	218	287
LmjF09.1160	*L. major*	60	1	60
LmjF09.1160	*L. major*	73	227	299
LmjF09.1160	*L. major*	62	445	506
LmjF09.1160	*L. major*	48	537	584
LmjF09.1180	*L. major*	580	64	643
LmjF09.1180	*L. major*	134	723	856
LmjF09.1190	*L. major*	86	114	199
LmjF09.1190	*L. major*	122	390	511
LmjF09.1200	*L. major*	104	1	104
LmjF09.1200	*L. major*	92	143	234
LmjF09.1200	*L. major*	66	560	625
LmjF09.1200	*L. major*	56	818	873
LmjF09.1200	*L. major*	157	895	1051
LmjF09.1200	*L. major*	55	1149	1203
LmjF09.1200	*L. major*	73	1343	1415
LmjF09.1210	*L. major*	191	234	424
LmjF09.1220	*L. major*	47	80	126
LmjF09.1220	*L. major*	78	334	411
LmjF09.1220	*L. major*	131	432	562
LmjF09.1220	*L. major*	40	583	622

LmjF09.1230	*L. major*	69	1	69
LmjF09.1230	*L. major*	51	358	408
LmjF09.1230	*L. major*	51	358	408
LmjF09.1240	*L. major*	46	245	290
LmjF09.1240	*L. major*	52	335	386
LmjF09.1240	*L. major*	47	841	887
LmjF09.1240	*L. major*	265	954	1218
LmjF09.1240	*L. major*	117	1328	1444
LmjF09.1240	*L. major*	53	1793	1845
LmjF09.1260	*L. major*	44	128	171
LmjF09.1260	*L. major*	173	296	468
LmjF09.1280	*L. major*	55	1	55
LmjF09.1280	*L. major*	61	90	150
LmjF09.1280	*L. major*	94	211	304
LmjF09.1290	*L. major*	56	1	56
LmjF09.1290	*L. major*	54	98	151
LmjF09.1290	*L. major*	187	198	384
LmjF09.1310	*L. major*	51	204	254
LmjF09.1315	*L. major*	57	146	202
LmjF09.1315	*L. major*	77	274	350
LmjF09.1320	*L. major*	109	246	354
LmjF09.1320	*L. major*	65	541	605
LmjF09.1320	*L. major*	65	541	605
LmjF09.1330	*L. major*	45	84	128
LmjF09.1330	*L. major*	53	159	211
LmjF09.1330	*L. major*	53	159	211
LmjF09.1340	*L. major*	40	1	40
LmjF09.1360	*L. major*	140	1	140
LmjF09.1370	*L. major*	69	1	69
LmjF09.1370	*L. major*	133	136	268
LmjF09.1380	*L. major*	214	1	214
LmjF09.1380	*L. major*	55	276	330
LmjF09.1380	*L. major*	56	500	555
LmjF09.1390	*L. major*	74	22	95
LmjF09.1400	*L. major*	123	133	255
LmjF09.1410	*L. major*	66	57	122
LmjF09.1410	*L. major*	126	146	271
LmjF09.1410	*L. major*	44	530	573
LmjF09.1420	*L. major*	108	32	139
LmjF09.1440	*L. major*	128	93	220
LmjF09.1440	*L. major*	83	245	327
LmjF09.1440	*L. major*	85	329	413
LmjF09.1480	*L. major*	42	351	392
LmjF09.1480	*L. major*	42	528	569
LmjF09.1480	*L. major*	42	528	569
LmjF09.1505	*L. major*	59	1	59
LmjF09.1510	*L. major*	43	59	101

LmjF09.1510	*L. major*	71	136	206
LmjF09.1510	*L. major*	96	1075	1170
LmjF09.1530	*L. major*	50	59	108
LmjF09.1540	*L. major*	477	1	477
LmjF09.1540	*L. major*	106	502	607
LmjF09.1550	*L. major*	45	1	45
LmjF10.0020	*L. major*	44	274	317
LmjF10.0030	*L. major*	58	258	315
LmjF10.0060	*L. major*	40	114	153
LmjF10.0080	*L. major*	41	68	108
LmjF10.0100	*L. major*	237	1	237
LmjF10.0120	*L. major*	54	1	54
LmjF10.0120	*L. major*	63	73	135
LmjF10.0120	*L. major*	60	202	261
LmjF10.0130	*L. major*	56	164	219
LmjF10.0130	*L. major*	114	267	380
LmjF10.0130	*L. major*	245	400	644
LmjF10.0130	*L. major*	80	656	735
LmjF10.0140	*L. major*	54	1	54
LmjF10.0140	*L. major*	147	79	225
LmjF10.0150	*L. major*	48	64	111
LmjF10.0150	*L. major*	189	131	319
LmjF10.0150	*L. major*	50	322	371
LmjF10.0160	*L. major*	436	70	505
LmjF10.0170	*L. major*	54	39	92
LmjF10.0170	*L. major*	99	416	514
LmjF10.0170	*L. major*	99	416	514
LmjF10.0185	*L. major*	41	1	41
LmjF10.0185	*L. major*	47	108	154
LmjF10.0185	*L. major*	47	108	154
LmjF10.0190	*L. major*	51	45	95
LmjF10.0190	*L. major*	126	115	240
LmjF10.0190	*L. major*	282	294	575
LmjF10.0195	*L. major*	90	1	90
LmjF10.0200	*L. major*	50	358	407
LmjF10.0210	*L. major*	54	420	473
LmjF10.0218	*L. major*	56	62	117
LmjF10.0219	*L. major*	53	224	276
LmjF10.0220	*L. major*	117	1	117
LmjF10.0230	*L. major*	44	1	44
LmjF10.0240	*L. major*	438	97	534
LmjF10.0240	*L. major*	131	538	668
LmjF10.0250	*L. major*	73	36	108
LmjF10.0250	*L. major*	48	212	259
LmjF10.0250	*L. major*	48	212	259
LmjF10.0270	*L. major*	92	1	92
LmjF10.0270	*L. major*	58	153	210

LmjF10.0270	*L. major*	114	252	365
LmjF10.0270	*L. major*	73	374	446
LmjF10.0270	*L. major*	94	654	747
LmjF10.0270	*L. major*	61	750	810
LmjF10.0270	*L. major*	72	860	931
LmjF10.0280	*L. major*	88	40	127
LmjF10.0280	*L. major*	113	220	332
LmjF10.0280	*L. major*	65	476	540
LmjF10.0280	*L. major*	590	566	1155
LmjF10.0280	*L. major*	88	1341	1428
LmjF10.0280	*L. major*	103	1623	1725
LmjF10.0280	*L. major*	48	1832	1879
LmjF10.0280	*L. major*	119	1905	2023
LmjF10.0280	*L. major*	173	2173	2345
LmjF10.0300	*L. major*	264	1	264
LmjF10.0300	*L. major*	331	275	605
LmjF10.0320	*L. major*	124	66	189
LmjF10.0320	*L. major*	48	220	267
LmjF10.0320	*L. major*	76	322	397
LmjF10.0320	*L. major*	40	446	485
LmjF10.0320	*L. major*	108	579	686
LmjF10.0320	*L. major*	186	729	914
LmjF10.0320	*L. major*	130	925	1054
LmjF10.0320	*L. major*	179	1170	1348
LmjF10.0320	*L. major*	310	1382	1691
LmjF10.0320	*L. major*	140	1780	1919
LmjF10.0330	*L. major*	46	171	216
LmjF10.0330	*L. major*	52	246	297
LmjF10.0330	*L. major*	129	306	434
LmjF10.0360	*L. major*	62	245	306
LmjF10.0360	*L. major*	40	626	665
LmjF10.0360	*L. major*	40	626	665
LmjF10.0370	*L. major*	62	182	243
LmjF10.0380	*L. major*	62	264	325
LmjF10.0380	*L. major*	44	648	691
LmjF10.0380	*L. major*	44	648	691
LmjF10.0385	*L. major*	62	275	336
LmjF10.0385	*L. major*	51	658	708
LmjF10.0385	*L. major*	51	658	708
LmjF10.0390	*L. major*	69	1	69
LmjF10.0390	*L. major*	62	309	370
LmjF10.0390	*L. major*	40	693	732
LmjF10.0400	*L. major*	78	293	370
LmjF10.0440	*L. major*	59	39	97
LmjF10.0483	*L. major*	216	36	251
LmjF10.0483	*L. major*	93	261	353
LmjF10.0483	*L. major*	99	390	488

LmjF10.0485	*L. major*	42	76	117
LmjF10.0485	*L. major*	64	147	210
LmjF10.0485	*L. major*	64	147	210
LmjF10.0500	*L. major*	92	411	502
LmjF10.0500	*L. major*	58	2339	2396
LmjF10.0500	*L. major*	50	4849	4898
LmjF10.0500	*L. major*	45	5560	5604
LmjF10.0500	*L. major*	296	6440	6735
LmjF10.0520	*L. major*	80	21	100
LmjF10.0520	*L. major*	40	135	174
LmjF10.0520	*L. major*	139	354	492
LmjF10.0520	*L. major*	165	743	907
LmjF10.0530	*L. major*	56	1	56
LmjF10.0530	*L. major*	127	66	192
LmjF10.0530	*L. major*	121	222	342
LmjF10.0530	*L. major*	74	364	437
LmjF10.0530	*L. major*	99	446	544
LmjF10.0530	*L. major*	125	565	689
LmjF10.0530	*L. major*	70	695	764
LmjF10.0530	*L. major*	370	775	1144
LmjF10.0540	*L. major*	59	242	300
LmjF10.0540	*L. major*	58	517	574
LmjF10.0540	*L. major*	110	576	685
LmjF10.0540	*L. major*	163	765	927
LmjF10.0540	*L. major*	131	958	1088
LmjF10.0550	*L. major*	60	1	60
LmjF10.0550	*L. major*	85	347	431
LmjF10.0570	*L. major*	44	90	133
LmjF10.0570	*L. major*	50	429	478
LmjF10.0570	*L. major*	50	429	478
LmjF10.0580	*L. major*	190	61	250
LmjF10.0580	*L. major*	60	266	325
LmjF10.0580	*L. major*	78	428	505
LmjF10.0580	*L. major*	49	545	593
LmjF10.0580	*L. major*	167	641	807
LmjF10.0610	*L. major*	54	34	87
LmjF10.0610	*L. major*	99	136	234
LmjF10.0610	*L. major*	46	252	297
LmjF10.0640	*L. major*	107	102	208
LmjF10.0650	*L. major*	135	62	196
LmjF10.0650	*L. major*	49	238	286
LmjF10.0660	*L. major*	222	133	354
LmjF10.0660	*L. major*	280	361	640
LmjF10.0660	*L. major*	60	663	722
LmjF10.0660	*L. major*	177	749	925
LmjF10.0660	*L. major*	387	941	1327
LmjF10.0660	*L. major*	156	1358	1513

LmjF10.0660	*L. major*	423	1520	1942
LmjF10.0670	*L. major*	88	32	119
LmjF10.0680	*L. major*	66	1	66
LmjF10.0680	*L. major*	304	72	375
LmjF10.0690	*L. major*	86	210	295
LmjF10.0690	*L. major*	43	337	379
LmjF10.0690	*L. major*	70	1201	1270
LmjF10.0690	*L. major*	52	1312	1363
LmjF10.0690	*L. major*	46	1417	1462
LmjF10.0690	*L. major*	55	1553	1607
LmjF10.0700	*L. major*	205	114	318
LmjF10.0700	*L. major*	45	320	364
LmjF10.0700	*L. major*	64	441	504
LmjF10.0700	*L. major*	106	783	888
LmjF10.0710	*L. major*	73	1	73
LmjF10.0715	*L. major*	73	1	73
LmjF10.0720	*L. major*	73	1	73
LmjF10.0730	*L. major*	45	103	147
LmjF10.0740	*L. major*	68	57	124
LmjF10.0750	*L. major*	373	20	392
LmjF10.0750	*L. major*	209	421	629
LmjF10.0750	*L. major*	491	664	1154
LmjF10.0750	*L. major*	247	1225	1471
LmjF10.0750	*L. major*	113	1499	1611
LmjF10.0750	*L. major*	154	1627	1780
LmjF10.0750	*L. major*	157	1839	1995
LmjF10.0760	*L. major*	64	1	64
LmjF10.0760	*L. major*	98	136	233
LmjF10.0760	*L. major*	44	337	380
LmjF10.0760	*L. major*	52	464	515
LmjF10.0770	*L. major*	114	168	281
LmjF10.0770	*L. major*	49	457	505
LmjF10.0770	*L. major*	49	457	505
LmjF10.0790	*L. major*	221	1	221
LmjF10.0800	*L. major*	297	101	397
LmjF10.0800	*L. major*	388	698	1085
LmjF10.0810	*L. major*	84	187	270
LmjF10.0810	*L. major*	80	388	467
LmjF10.0810	*L. major*	47	639	685
LmjF10.0810	*L. major*	82	702	783
LmjF10.0810	*L. major*	68	1009	1076
LmjF10.0810	*L. major*	181	1394	1574
LmjF10.0810	*L. major*	245	1581	1825
LmjF10.0820	*L. major*	83	1	83
LmjF10.0820	*L. major*	63	89	151
LmjF10.0830	*L. major*	1058	1	1058
LmjF10.0830	*L. major*	75	1181	1255

LmjF10.0840	*L. major*	64	314	377
LmjF10.0840	*L. major*	41	494	534
LmjF10.0840	*L. major*	41	494	534
LmjF10.0860	*L. major*	55	67	121
LmjF10.0870	*L. major*	49	1	49
LmjF10.0880	*L. major*	193	1	193
LmjF10.0880	*L. major*	346	221	566
LmjF10.0880	*L. major*	76	609	684
LmjF10.0880	*L. major*	300	697	996
LmjF10.0880	*L. major*	103	1016	1118
LmjF10.0890	*L. major*	56	1	56
LmjF10.0900	*L. major*	179	49	227
LmjF10.0900	*L. major*	63	260	322
LmjF10.0900	*L. major*	59	338	396
LmjF10.0900	*L. major*	452	751	1202
LmjF10.0900	*L. major*	53	1253	1305
LmjF10.0900	*L. major*	67	1341	1407
LmjF10.0900	*L. major*	66	1435	1500
LmjF10.0900	*L. major*	87	1600	1686
LmjF10.0940	*L. major*	68	1	68
LmjF10.0940	*L. major*	240	360	599
LmjF10.0940	*L. major*	97	601	697
LmjF10.0940	*L. major*	185	699	883
LmjF10.0940	*L. major*	163	1059	1221
LmjF10.0950	*L. major*	125	128	252
LmjF10.0960	*L. major*	118	1	118
LmjF10.0960	*L. major*	109	247	355
LmjF10.0960	*L. major*	150	529	678
LmjF10.0960	*L. major*	126	680	805
LmjF10.0970	*L. major*	173	1	173
LmjF10.0980	*L. major*	45	119	163
LmjF10.0980	*L. major*	55	229	283
LmjF10.0980	*L. major*	47	393	439
LmjF10.0980	*L. major*	136	466	601
LmjF10.0990	*L. major*	49	1	49
LmjF10.1000	*L. major*	63	33	95
LmjF10.1000	*L. major*	49	224	272
LmjF10.1000	*L. major*	49	224	272
LmjF10.1020	*L. major*	78	1	78
LmjF10.1030	*L. major*	169	1	169
LmjF10.1030	*L. major*	56	324	379
LmjF10.1040	*L. major*	111	167	277
LmjF10.1040	*L. major*	69	579	647
LmjF10.1040	*L. major*	97	718	814
LmjF10.1040	*L. major*	44	955	998
LmjF10.1050	*L. major*	50	99	148
LmjF10.1060	*L. major*	43	52	94

LmjF10.1060	*L. major*	58	238	295
LmjF10.1070	*L. major*	112	1	112
LmjF10.1070	*L. major*	52	134	185
LmjF10.1070	*L. major*	302	187	488
LmjF10.1070	*L. major*	74	499	572
LmjF10.1080	*L. major*	107	1	107
LmjF10.1080	*L. major*	55	119	173
LmjF10.1080	*L. major*	69	397	465
LmjF10.1100	*L. major*	48	378	425
LmjF10.1110	*L. major*	149	161	309
LmjF10.1110	*L. major*	186	338	523
LmjF10.1130	*L. major*	82	1	82
LmjF10.1130	*L. major*	172	86	257
LmjF10.1130	*L. major*	96	898	993
LmjF10.1130	*L. major*	61	1273	1333
LmjF10.1140	*L. major*	62	205	266
LmjF10.1150	*L. major*	45	1	45
LmjF10.1150	*L. major*	429	52	480
LmjF10.1160	*L. major*	42	175	216
LmjF10.1190	*L. major*	378	1	378
LmjF10.1195	*L. major*	128	62	189
LmjF10.1210	*L. major*	73	1	73
LmjF10.1210	*L. major*	79	88	166
LmjF10.1210	*L. major*	205	294	498
LmjF10.1225	*L. major*	40	17	56
LmjF10.1300	*L. major*	58	258	315
LmjF10.1310	*L. major*	44	274	317
LmjF11.0010	*L. major*	130	1	130
LmjF11.0020	*L. major*	40	111	150
LmjF11.0020	*L. major*	47	243	289
LmjF11.0020	*L. major*	47	243	289
LmjF11.0030	*L. major*	54	254	307
LmjF11.0040	*L. major*	55	243	297
LmjF11.0050	*L. major*	40	399	438
LmjF11.0060	*L. major*	64	12	75
LmjF11.0060	*L. major*	106	249	354
LmjF11.0060	*L. major*	50	985	1034
LmjF11.0070	*L. major*	48	1	48
LmjF11.0070	*L. major*	889	129	1017
LmjF11.0070	*L. major*	112	1099	1210
LmjF11.0080	*L. major*	86	1	86
LmjF11.0080	*L. major*	65	161	225
LmjF11.0080	*L. major*	65	161	225
LmjF11.0090	*L. major*	58	151	208
LmjF11.0090	*L. major*	46	441	486
LmjF11.0090	*L. major*	46	441	486
LmjF11.0100	*L. major*	82	49	130

LmjF11.0140	*L. major*	116	32	147
LmjF11.0150	*L. major*	284	1	284
LmjF11.0150	*L. major*	350	290	639
LmjF11.0150	*L. major*	220	666	885
LmjF11.0160	*L. major*	73	142	214
LmjF11.0170	*L. major*	159	121	279
LmjF11.0170	*L. major*	49	301	349
LmjF11.0170	*L. major*	327	391	717
LmjF11.0190	*L. major*	42	56	97
LmjF11.0190	*L. major*	57	553	609
LmjF11.0190	*L. major*	57	553	609
LmjF11.0200	*L. major*	146	1	146
LmjF11.0210	*L. major*	90	1	90
LmjF11.0230	*L. major*	87	56	142
LmjF11.0230	*L. major*	60	329	388
LmjF11.0230	*L. major*	51	395	445
LmjF11.0230	*L. major*	46	454	499
LmjF11.0250	*L. major*	242	1	242
LmjF11.0250	*L. major*	185	306	490
LmjF11.0250	*L. major*	87	546	632
LmjF11.0250	*L. major*	50	756	805
LmjF11.0250	*L. major*	130	861	990
LmjF11.0290	*L. major*	245	1	245
LmjF11.0290	*L. major*	114	261	374
LmjF11.0290	*L. major*	42	401	442
LmjF11.0300	*L. major*	162	17	178
LmjF11.0310	*L. major*	61	1	61
LmjF11.0310	*L. major*	80	102	181
LmjF11.0310	*L. major*	242	376	617
LmjF11.0310	*L. major*	63	646	708
LmjF11.0310	*L. major*	86	726	811
LmjF11.0310	*L. major*	50	965	1014
LmjF11.0310	*L. major*	48	1244	1291
LmjF11.0310	*L. major*	118	1339	1456
LmjF11.0310	*L. major*	41	1499	1539
LmjF11.0310	*L. major*	49	1759	1807
LmjF11.0320	*L. major*	62	1	62
LmjF11.0320	*L. major*	148	173	320
LmjF11.0320	*L. major*	46	422	467
LmjF11.0320	*L. major*	90	655	744
LmjF11.0320	*L. major*	40	819	858
LmjF11.0320	*L. major*	77	1147	1223
LmjF11.0330	*L. major*	66	1	66
LmjF11.0330	*L. major*	45	166	210
LmjF11.0330	*L. major*	54	414	467
LmjF11.0330	*L. major*	91	784	874
LmjF11.0330	*L. major*	102	893	994

LmjF11.0340	*L. major*	44	1	44
LmjF11.0340	*L. major*	89	64	152
LmjF11.0340	*L. major*	109	343	451
LmjF11.0350	*L. major*	49	205	253
LmjF11.0360	*L. major*	112	60	171
LmjF11.0360	*L. major*	123	245	367
LmjF11.0370	*L. major*	137	1	137
LmjF11.0370	*L. major*	418	151	568
LmjF11.0380	*L. major*	172	1	172
LmjF11.0380	*L. major*	44	435	478
LmjF11.0380	*L. major*	62	515	576
LmjF11.0380	*L. major*	60	592	651
LmjF11.0380	*L. major*	62	841	902
LmjF11.0390	*L. major*	94	70	163
LmjF11.0390	*L. major*	44	246	289
LmjF11.0390	*L. major*	79	402	480
LmjF11.0400	*L. major*	109	1	109
LmjF11.0400	*L. major*	74	127	200
LmjF11.0400	*L. major*	66	554	619
LmjF11.0400	*L. major*	426	668	1093
LmjF11.0410	*L. major*	153	1	153
LmjF11.0410	*L. major*	113	249	361
LmjF11.0410	*L. major*	42	541	582
LmjF11.0420	*L. major*	41	528	568
LmjF11.0430	*L. major*	96	25	120
LmjF11.0430	*L. major*	59	398	456
LmjF11.0430	*L. major*	46	599	644
LmjF11.0440	*L. major*	405	182	586
LmjF11.0450	*L. major*	43	149	191
LmjF11.0450	*L. major*	41	230	270
LmjF11.0450	*L. major*	51	324	374
LmjF11.0450	*L. major*	52	434	485
LmjF11.0450	*L. major*	70	556	625
LmjF11.0450	*L. major*	46	662	707
LmjF11.0460	*L. major*	45	1	45
LmjF11.0460	*L. major*	56	79	134
LmjF11.0460	*L. major*	40	165	204
LmjF11.0460	*L. major*	42	314	355
LmjF11.0470	*L. major*	50	1	50
LmjF11.0470	*L. major*	102	86	187
LmjF11.0470	*L. major*	47	294	340
LmjF11.0480	*L. major*	40	1021	1060
LmjF11.0490	*L. major*	57	1	57
LmjF11.0490	*L. major*	91	220	310
LmjF11.0510	*L. major*	114	56	169
LmjF11.0510	*L. major*	43	477	519
LmjF11.0530	*L. major*	99	28	126

LmjF11.0530	*L. major*	46	273	318
LmjF11.0530	*L. major*	43	457	499
LmjF11.0540	*L. major*	51	111	161
LmjF11.0540	*L. major*	70	324	393
LmjF11.0540	*L. major*	70	324	393
LmjF11.0550	*L. major*	67	309	375
LmjF11.0560	*L. major*	113	1	113
LmjF11.0560	*L. major*	41	144	184
LmjF11.0560	*L. major*	45	268	312
LmjF11.0560	*L. major*	74	719	792
LmjF11.0560	*L. major*	52	875	926
LmjF11.0560	*L. major*	145	938	1082
LmjF11.0580	*L. major*	56	60	115
LmjF11.0600	*L. major*	226	61	286
LmjF11.0600	*L. major*	210	390	599
LmjF11.0610	*L. major*	210	1	210
LmjF11.0610	*L. major*	486	242	727
LmjF11.0610	*L. major*	62	755	816
LmjF11.0610	*L. major*	111	966	1076
LmjF11.0640	*L. major*	85	1	85
LmjF11.0640	*L. major*	173	181	353
LmjF11.0640	*L. major*	68	384	451
LmjF11.0640	*L. major*	57	489	545
LmjF11.0640	*L. major*	56	586	641
LmjF11.0640	*L. major*	110	737	846
LmjF11.0640	*L. major*	48	889	936
LmjF11.0640	*L. major*	57	1101	1157
LmjF11.0640	*L. major*	305	1198	1502
LmjF11.0640	*L. major*	41	1536	1576
LmjF11.0650	*L. major*	56	309	364
LmjF11.0660	*L. major*	65	312	376
LmjF11.0660	*L. major*	43	611	653
LmjF11.0680	*L. major*	47	491	537
LmjF11.0700	*L. major*	91	43	133
LmjF11.0700	*L. major*	49	220	268
LmjF11.0700	*L. major*	49	220	268
LmjF11.0710	*L. major*	192	21	212
LmjF11.0710	*L. major*	44	253	296
LmjF11.0710	*L. major*	133	338	470
LmjF11.0720	*L. major*	59	253	311
LmjF11.0720	*L. major*	112	348	459
LmjF11.0720	*L. major*	140	514	653
LmjF11.0730	*L. major*	86	1	86
LmjF11.0730	*L. major*	99	202	300
LmjF11.0730	*L. major*	85	408	492
LmjF11.0730	*L. major*	148	603	750
LmjF11.0730	*L. major*	276	756	1031

LmjF11.0730	*L. major*	55	1067	1121
LmjF11.0740	*L. major*	59	1	59
LmjF11.0740	*L. major*	50	271	320
LmjF11.0740	*L. major*	87	358	444
LmjF11.0750	*L. major*	118	88	205
LmjF11.0760	*L. major*	96	69	164
LmjF11.0780	*L. major*	96	69	164
LmjF11.0790	*L. major*	49	122	170
LmjF11.0810	*L. major*	83	77	159
LmjF11.0810	*L. major*	51	478	528
LmjF11.0810	*L. major*	51	478	528
LmjF11.0820	*L. major*	270	69	338
LmjF11.0830	*L. major*	98	1	98
LmjF11.0830	*L. major*	220	112	331
LmjF11.0830	*L. major*	102	352	453
LmjF11.0830	*L. major*	228	781	1008
LmjF11.0830	*L. major*	393	1017	1409
LmjF11.0860	*L. major*	127	1	127
LmjF11.0860	*L. major*	47	246	292
LmjF11.0860	*L. major*	62	320	381
LmjF11.0860	*L. major*	40	543	582
LmjF11.0860	*L. major*	94	644	737
LmjF11.0860	*L. major*	51	1017	1067
LmjF11.0860	*L. major*	72	1177	1248
LmjF11.0860	*L. major*	135	1532	1666
LmjF11.0870	*L. major*	53	126	178
LmjF11.0870	*L. major*	59	232	290
LmjF11.0870	*L. major*	242	394	635
LmjF11.0870	*L. major*	63	668	730
LmjF11.0870	*L. major*	530	812	1341
LmjF11.0870	*L. major*	61	1357	1417
LmjF11.0880	*L. major*	44	1	44
LmjF11.0890	*L. major*	92	1	92
LmjF11.0890	*L. major*	73	441	513
LmjF11.0900	*L. major*	103	88	190
LmjF11.0910	*L. major*	347	1	347
LmjF11.0910	*L. major*	66	766	831
LmjF11.0910	*L. major*	66	917	982
LmjF11.0910	*L. major*	53	1019	1071
LmjF11.0910	*L. major*	50	1117	1166
LmjF11.0910	*L. major*	164	1302	1465
LmjF11.0920	*L. major*	124	50	173
LmjF11.0920	*L. major*	59	300	358
LmjF11.0920	*L. major*	167	454	620
LmjF11.0930	*L. major*	179	87	265
LmjF11.0930	*L. major*	108	270	377
LmjF11.0940	*L. major*	43	27	69

LmjF11.0940	*L. major*	51	129	179
LmjF11.0940	*L. major*	76	212	287
LmjF11.0940	*L. major*	618	293	910
LmjF11.0940	*L. major*	124	920	1043
LmjF11.0940	*L. major*	139	1045	1183
LmjF11.0940	*L. major*	75	1196	1270
LmjF11.0940	*L. major*	144	1306	1449
LmjF11.0950	*L. major*	46	1	46
LmjF11.0950	*L. major*	47	95	141
LmjF11.0950	*L. major*	62	183	244
LmjF11.0950	*L. major*	149	265	413
LmjF11.0980	*L. major*	68	36	103
LmjF11.0980	*L. major*	149	106	254
LmjF11.0980	*L. major*	71	357	427
LmjF11.0990	*L. major*	82	682	763
LmjF11.1010	*L. major*	44	45	88
LmjF11.1010	*L. major*	47	130	176
LmjF11.1010	*L. major*	69	227	295
LmjF11.1010	*L. major*	72	518	589
LmjF11.1020	*L. major*	213	1	213
LmjF11.1020	*L. major*	53	449	501
LmjF11.1020	*L. major*	187	690	876
LmjF11.1020	*L. major*	132	907	1038
LmjF11.1030	*L. major*	45	1	45
LmjF11.1030	*L. major*	41	109	149
LmjF11.1030	*L. major*	41	109	149
LmjF11.1040	*L. major*	113	1	113
LmjF11.1040	*L. major*	40	121	160
LmjF11.1040	*L. major*	56	240	295
LmjF11.1040	*L. major*	256	313	568
LmjF11.1040	*L. major*	158	576	733
LmjF11.1070	*L. major*	202	1	202
LmjF11.1080	*L. major*	90	1	90
LmjF11.1080	*L. major*	63	126	188
LmjF11.1080	*L. major*	67	227	293
LmjF11.1090	*L. major*	75	1	75
LmjF11.1110	*L. major*	81	67	147
LmjF11.1120	*L. major*	76	69	144
LmjF11.1120	*L. major*	65	193	257
LmjF11.1120	*L. major*	65	193	257
LmjF11.1130	*L. major*	81	67	147
LmjF11.1160	*L. major*	367	717	1083
LmjF11.1170	*L. major*	334	1	334
LmjF11.1200	*L. major*	46	357	402
LmjF11.1200	*L. major*	56	637	692
LmjF11.1220	*L. major*	56	1721	1776
LmjF11.1240	*L. major*	98	1	98

LmjF11.1250	*L. major*	56	1721	1776
LmjF11.1290	*L. major*	126	1734	1859
LmjF11.1300	*L. major*	113	1	113
LmjF11.1310	*L. major*	51	107	157
LmjF11.1330	*L. major*	110	69	178
LmjF11.1330	*L. major*	85	201	285
LmjF11.1330	*L. major*	57	363	419
LmjF11.1330	*L. major*	84	462	545
LmjF11.1330	*L. major*	110	622	731
LmjF11.1330	*L. major*	65	855	919
LmjF11.1330	*L. major*	49	1449	1497
LmjF11.1330	*L. major*	88	1527	1614
LmjF11.1340	*L. major*	103	304	406
LmjF11.1350	*L. major*	40	1	40
LmjF11.1350	*L. major*	92	79	170
LmjF11.1350	*L. major*	92	79	170
LmjF11.1360	*L. major*	71	1	71
LmjF11.1380	*L. major*	91	105	195
LmjF11.1380	*L. major*	46	214	259
LmjF11.1380	*L. major*	85	302	386
LmjF12.0060	*L. major*	42	151	192
LmjF12.0070	*L. major*	54	63	116
LmjF12.0070	*L. major*	430	150	579
LmjF12.0100	*L. major*	64	118	181
LmjF12.0110	*L. major*	156	1	156
LmjF12.0110	*L. major*	446	188	633
LmjF12.0110	*L. major*	67	706	772
LmjF12.0120	*L. major*	243	43	285
LmjF12.0120	*L. major*	262	317	578
LmjF12.0120	*L. major*	691	608	1298
LmjF12.0130	*L. major*	101	255	355
LmjF12.0130	*L. major*	63	469	531
LmjF12.0140	*L. major*	147	214	360
LmjF12.0150	*L. major*	186	188	373
LmjF12.0150	*L. major*	62	401	462
LmjF12.0150	*L. major*	50	546	595
LmjF12.0150	*L. major*	58	651	708
LmjF12.0150	*L. major*	155	871	1025
LmjF12.0160	*L. major*	54	168	221
LmjF12.0160	*L. major*	68	355	422
LmjF12.0160	*L. major*	48	483	530
LmjF12.0160	*L. major*	62	660	721
LmjF12.0170	*L. major*	62	1	62
LmjF12.0170	*L. major*	125	82	206
LmjF12.0170	*L. major*	96	231	326
LmjF12.0180	*L. major*	136	120	255
LmjF12.0180	*L. major*	150	364	513

LmjF12.0180	*L. major*	150	364	513
LmjF12.0190	*L. major*	99	86	184
LmjF12.0190	*L. major*	56	205	260
LmjF12.0190	*L. major*	83	340	422
LmjF12.0190	*L. major*	84	587	670
LmjF12.0190	*L. major*	124	844	967
LmjF12.0190	*L. major*	68	1009	1076
LmjF12.0190	*L. major*	54	1137	1190
LmjF12.0190	*L. major*	43	1290	1332
LmjF12.0190	*L. major*	158	1466	1623
LmjF12.0200	*L. major*	82	1	82
LmjF12.0200	*L. major*	333	98	430
LmjF12.0200	*L. major*	373	481	853
LmjF12.0200	*L. major*	217	861	1077
LmjF12.0210	*L. major*	50	1	50
LmjF12.0230	*L. major*	90	1	90
LmjF12.0230	*L. major*	60	138	197
LmjF12.0230	*L. major*	122	325	446
LmjF12.0230	*L. major*	75	515	589
LmjF12.0230	*L. major*	46	646	691
LmjF12.0230	*L. major*	118	944	1061
LmjF12.0240	*L. major*	126	579	704
LmjF12.0250	*L. major*	49	145	193
LmjF12.0250	*L. major*	57	303	359
LmjF12.0250	*L. major*	141	644	784
LmjF12.0260	*L. major*	42	344	385
LmjF12.0260	*L. major*	57	462	518
LmjF12.0260	*L. major*	63	559	621
LmjF12.0260	*L. major*	56	740	795
LmjF12.0260	*L. major*	70	849	918
LmjF12.0260	*L. major*	59	939	997
LmjF12.0260	*L. major*	45	1125	1169
LmjF12.0260	*L. major*	41	1419	1459
LmjF12.0260	*L. major*	331	1597	1927
LmjF12.0260	*L. major*	96	2091	2186
LmjF12.0260	*L. major*	50	2285	2334
LmjF12.0260	*L. major*	63	2430	2492
LmjF12.0260	*L. major*	82	2520	2601
LmjF12.0270	*L. major*	56	1	56
LmjF12.0270	*L. major*	77	117	193
LmjF12.0270	*L. major*	60	289	348
LmjF12.0280	*L. major*	92	33	124
LmjF12.0290	*L. major*	57	1	57
LmjF12.0290	*L. major*	353	80	432
LmjF12.0290	*L. major*	668	440	1107
LmjF12.0290	*L. major*	55	1143	1197
LmjF12.0290	*L. major*	197	1354	1550

LmjF12.0300	*L. major*	126	531	656
LmjF12.0300	*L. major*	115	679	793
LmjF12.0300	*L. major*	385	795	1179
LmjF12.0300	*L. major*	68	1227	1294
LmjF12.0300	*L. major*	87	1324	1410
LmjF12.0300	*L. major*	77	1564	1640
LmjF12.0310	*L. major*	153	84	236
LmjF12.0310	*L. major*	86	424	509
LmjF12.0310	*L. major*	60	585	644
LmjF12.0320	*L. major*	172	112	283
LmjF12.0320	*L. major*	54	318	371
LmjF12.0320	*L. major*	61	460	520
LmjF12.0320	*L. major*	98	543	640
LmjF12.0320	*L. major*	54	739	792
LmjF12.0320	*L. major*	497	807	1303
LmjF12.0320	*L. major*	59	1342	1400
LmjF12.0320	*L. major*	89	1420	1508
LmjF12.0320	*L. major*	85	1531	1615
LmjF12.0320	*L. major*	88	1654	1741
LmjF12.0320	*L. major*	49	1856	1904
LmjF12.0320	*L. major*	96	2143	2238
LmjF12.0320	*L. major*	42	2325	2366
LmjF12.0320	*L. major*	199	2443	2641
LmjF12.0320	*L. major*	210	2718	2927
LmjF12.0320	*L. major*	74	2938	3011
LmjF12.0320	*L. major*	168	3079	3246
LmjF12.0330	*L. major*	65	223	287
LmjF12.0330	*L. major*	40	328	367
LmjF12.0330	*L. major*	44	438	481
LmjF12.0330	*L. major*	213	514	726
LmjF12.0330	*L. major*	45	754	798
LmjF12.0340	*L. major*	454	123	576
LmjF12.0340	*L. major*	163	607	769
LmjF12.0340	*L. major*	582	793	1374
LmjF12.0340	*L. major*	109	1427	1535
LmjF12.0340	*L. major*	47	1563	1609
LmjF12.0340	*L. major*	48	1692	1739
LmjF12.0340	*L. major*	269	1775	2043
LmjF12.0340	*L. major*	123	2081	2203
LmjF12.0350	*L. major*	342	1	342
LmjF12.0350	*L. major*	60	503	562
LmjF12.0360	*L. major*	100	199	298
LmjF12.0370	*L. major*	43	153	195
LmjF12.0370	*L. major*	79	211	289
LmjF12.0370	*L. major*	79	211	289
LmjF12.0380	*L. major*	116	1	116
LmjF12.0380	*L. major*	107	191	297

LmjF12.0380	*L. major*	101	318	418
LmjF12.0380	*L. major*	160	430	589
LmjF12.0380	*L. major*	384	592	975
LmjF12.0380	*L. major*	54	1011	1064
LmjF12.0390	*L. major*	47	331	377
LmjF12.0405	*L. major*	144	1	144
LmjF12.0405	*L. major*	159	172	330
LmjF12.0410	*L. major*	50	135	184
LmjF12.0430	*L. major*	55	42	96
LmjF12.0430	*L. major*	164	194	357
LmjF12.0430	*L. major*	56	367	422
LmjF12.0430	*L. major*	105	508	612
LmjF12.0440	*L. major*	79	27	105
LmjF12.0440	*L. major*	108	238	345
LmjF12.0440	*L. major*	60	368	427
LmjF12.0440	*L. major*	86	438	523
LmjF12.0440	*L. major*	152	559	710
LmjF12.0450	*L. major*	163	1	163
LmjF12.0450	*L. major*	173	194	366
LmjF12.0450	*L. major*	46	377	422
LmjF12.0450	*L. major*	128	433	560
LmjF12.0450	*L. major*	427	665	1091
LmjF12.0460	*L. major*	511	1	511
LmjF12.0460	*L. major*	116	670	785
LmjF12.0470	*L. major*	411	1	411
LmjF12.0470	*L. major*	79	441	519
LmjF12.0470	*L. major*	61	542	602
LmjF12.0470	*L. major*	45	691	735
LmjF12.0480	*L. major*	48	1	48
LmjF12.0480	*L. major*	174	65	238
LmjF12.0480	*L. major*	79	242	320
LmjF12.0480	*L. major*	41	386	426
LmjF12.0480	*L. major*	153	449	601
LmjF12.0490	*L. major*	149	47	195
LmjF12.0490	*L. major*	88	199	286
LmjF12.0490	*L. major*	88	199	286
LmjF12.0500	*L. major*	55	1	55
LmjF12.0500	*L. major*	181	99	279
LmjF12.0500	*L. major*	56	320	375
LmjF12.0500	*L. major*	113	402	514
LmjF12.0500	*L. major*	90	521	610
LmjF12.0500	*L. major*	255	625	879
LmjF12.0500	*L. major*	68	881	948
LmjF12.0500	*L. major*	63	974	1036
LmjF12.0510	*L. major*	565	62	626
LmjF12.0510	*L. major*	143	633	775
LmjF12.0510	*L. major*	53	778	830

LmjF12.0510	*L. major*	77	894	970
LmjF12.0540	*L. major*	76	1	76
LmjF12.0550	*L. major*	100	48	147
LmjF12.0550	*L. major*	225	182	406
LmjF12.0550	*L. major*	87	413	499
LmjF12.0560	*L. major*	62	1	62
LmjF12.0560	*L. major*	123	219	341
LmjF12.0560	*L. major*	46	496	541
LmjF12.0560	*L. major*	293	624	916
LmjF12.0560	*L. major*	126	921	1046
LmjF12.0570	*L. major*	93	105	197
LmjF12.0570	*L. major*	104	224	327
LmjF12.0570	*L. major*	162	1094	1255
LmjF12.0570	*L. major*	348	1276	1623
LmjF12.0570	*L. major*	189	1628	1816
LmjF12.0590	*L. major*	220	1	220
LmjF12.0590	*L. major*	45	855	899
LmjF12.0590	*L. major*	119	1028	1146
LmjF12.0590	*L. major*	42	1221	1262
LmjF12.0590	*L. major*	358	1297	1654
LmjF12.0590	*L. major*	90	1688	1777
LmjF12.0600	*L. major*	46	1	46
LmjF12.0600	*L. major*	47	181	227
LmjF12.0600	*L. major*	61	252	312
LmjF12.0610	*L. major*	178	179	356
LmjF12.0610	*L. major*	63	450	512
LmjF12.0610	*L. major*	137	812	948
LmjF12.0610	*L. major*	60	966	1025
LmjF12.0620	*L. major*	936	43	978
LmjF12.0620	*L. major*	321	996	1316
LmjF12.0640	*L. major*	86	175	260
LmjF12.0640	*L. major*	53	307	359
LmjF12.0640	*L. major*	53	307	359
LmjF12.0650	*L. major*	65	16	80
LmjF12.0650	*L. major*	455	187	641
LmjF12.0650	*L. major*	138	681	818
LmjF12.0660	*L. major*	63	1	63
LmjF12.0680	*L. major*	41	135	175
LmjF12.0680	*L. major*	62	328	389
LmjF12.0690	*L. major*	73	94	166
LmjF12.0700	*L. major*	47	123	169
LmjF12.0700	*L. major*	44	239	282
LmjF12.0700	*L. major*	67	398	464
LmjF12.0700	*L. major*	171	676	846
LmjF12.0715	*L. major*	91	219	309
LmjF12.0715	*L. major*	145	338	482
LmjF12.0730	*L. major*	66	192	257

LmjF12.0730	*L. major*	63	290	352
LmjF12.0730	*L. major*	76	429	504
LmjF12.0740	*L. major*	162	237	398
LmjF12.0740	*L. major*	63	429	491
LmjF12.0740	*L. major*	195	523	717
LmjF12.0755	*L. major*	125	403	527
LmjF12.0760	*L. major*	113	140	252
LmjF12.0760	*L. major*	119	289	407
LmjF12.0760	*L. major*	119	426	544
LmjF12.0765	*L. major*	200	330	529
LmjF12.0770	*L. major*	91	219	309
LmjF12.0770	*L. major*	145	338	482
LmjF12.0780	*L. major*	86	120	205
LmjF12.0780	*L. major*	97	236	332
LmjF12.0800	*L. major*	91	219	309
LmjF12.0800	*L. major*	142	336	477
LmjF12.0810	*L. major*	87	119	205
LmjF12.0810	*L. major*	107	236	342
LmjF12.0820	*L. major*	91	219	309
LmjF12.0820	*L. major*	147	336	482
LmjF12.0830	*L. major*	52	279	330
LmjF12.0830	*L. major*	40	357	396
LmjF12.0830	*L. major*	178	426	603
LmjF12.0840	*L. major*	96	219	314
LmjF12.0850	*L. major*	60	363	422
LmjF12.0850	*L. major*	129	452	580
LmjF12.0860	*L. major*	472	235	706
LmjF12.0870	*L. major*	175	330	504
LmjF12.0880	*L. major*	147	1	147
LmjF12.0880	*L. major*	71	286	356
LmjF12.0880	*L. major*	71	286	356
LmjF12.0885	*L. major*	147	1	147
LmjF12.0885	*L. major*	71	286	356
LmjF12.0885	*L. major*	71	286	356
LmjF12.0890	*L. major*	63	119	181
LmjF12.0890	*L. major*	45	307	351
LmjF12.0890	*L. major*	45	307	351
LmjF12.0895	*L. major*	90	219	308
LmjF12.0895	*L. major*	147	336	482
LmjF12.0905	*L. major*	213	1	213
LmjF12.0905	*L. major*	41	391	431
LmjF12.0905	*L. major*	41	391	431
LmjF12.0930	*L. major*	91	219	309
LmjF12.0930	*L. major*	147	336	482
LmjF12.0940	*L. major*	86	120	205
LmjF12.0940	*L. major*	107	236	342
LmjF12.0950	*L. major*	91	219	309

LmjF12.0950	*L. major*	147	336	482
LmjF12.0960	*L. major*	51	279	329
LmjF12.0960	*L. major*	41	356	396
LmjF12.0960	*L. major*	198	426	623
LmjF12.0970	*L. major*	91	219	309
LmjF12.0970	*L. major*	106	338	443
LmjF12.0970	*L. major*	87	498	584
LmjF12.0980	*L. major*	86	120	205
LmjF12.0980	*L. major*	107	236	342
LmjF12.0990	*L. major*	84	146	229
LmjF12.0990	*L. major*	164	262	425
LmjF12.0990	*L. major*	60	430	489
LmjF12.0990	*L. major*	43	524	566
LmjF12.0995	*L. major*	265	85	349
LmjF12.0995	*L. major*	71	488	558
LmjF12.0995	*L. major*	71	488	558
LmjF12.1000	*L. major*	86	120	205
LmjF12.1000	*L. major*	107	236	342
LmjF12.1005	*L. major*	260	1	260
LmjF12.1015	*L. major*	213	1	213
LmjF12.1015	*L. major*	71	352	422
LmjF12.1015	*L. major*	71	352	422
LmjF12.1020	*L. major*	232	235	466
LmjF12.1030	*L. major*	91	172	262
LmjF12.1030	*L. major*	147	289	435
LmjF12.1040	*L. major*	256	235	490
LmjF12.1050	*L. major*	90	219	308
LmjF12.1050	*L. major*	107	333	439
LmjF12.1060	*L. major*	108	382	489
LmjF12.1070	*L. major*	62	360	421
LmjF12.1070	*L. major*	210	452	661
LmjF12.1090	*L. major*	148	281	428
LmjF12.1100	*L. major*	142	1	142
LmjF12.1100	*L. major*	62	298	359
LmjF12.1100	*L. major*	282	372	653
LmjF12.1100	*L. major*	64	658	721
LmjF12.1100	*L. major*	51	748	798
LmjF12.1100	*L. major*	222	835	1056
LmjF12.1100	*L. major*	90	1411	1500
LmjF12.1110	*L. major*	73	13	85
LmjF12.1110	*L. major*	485	116	600
LmjF12.1110	*L. major*	92	642	733
LmjF12.1120	*L. major*	440	145	584
LmjF12.1120	*L. major*	265	708	972
LmjF12.1120	*L. major*	50	1015	1064
LmjF12.1140	*L. major*	74	375	448
LmjF12.1150	*L. major*	96	30	125

LmjF12.1150	*L. major*	66	158	223
LmjF12.1150	*L. major*	411	286	696
LmjF12.1150	*L. major*	87	777	863
LmjF12.1150	*L. major*	49	895	943
LmjF12.1150	*L. major*	48	1051	1098
LmjF12.1160	*L. major*	109	200	308
LmjF12.1160	*L. major*	104	329	432
LmjF12.1160	*L. major*	253	695	947
LmjF12.1170	*L. major*	201	524	724
LmjF12.1180	*L. major*	364	27	390
LmjF12.1180	*L. major*	63	466	528
LmjF12.1180	*L. major*	67	650	716
LmjF12.1180	*L. major*	53	813	865
LmjF12.1190	*L. major*	60	1	60
LmjF12.1190	*L. major*	62	159	220
LmjF12.1190	*L. major*	54	262	315
LmjF12.1190	*L. major*	82	507	588
LmjF12.1190	*L. major*	44	663	706
LmjF12.1190	*L. major*	97	1143	1239
LmjF12.1190	*L. major*	59	1272	1330
LmjF12.1200	*L. major*	95	1	95
LmjF12.1200	*L. major*	160	369	528
LmjF12.1210	*L. major*	245	1	245
LmjF12.1210	*L. major*	291	281	571
LmjF12.1210	*L. major*	92	643	734
LmjF12.1210	*L. major*	259	748	1006
LmjF12.1220	*L. major*	42	289	330
LmjF12.1220	*L. major*	223	350	572
LmjF12.1220	*L. major*	371	988	1358
LmjF12.1220	*L. major*	48	1360	1407
LmjF12.1240	*L. major*	79	1	79
LmjF12.1240	*L. major*	56	111	166
LmjF12.1240	*L. major*	86	298	383
LmjF12.1240	*L. major*	106	408	513
LmjF12.1240	*L. major*	136	588	723
LmjF12.1250	*L. major*	55	665	719
LmjF12.1250	*L. major*	95	900	994
LmjF12.1250	*L. major*	83	1006	1088
LmjF12.1250	*L. major*	48	1254	1301
LmjF12.1260	*L. major*	110	74	183
LmjF12.1280	*L. major*	654	1	654
LmjF12.1290	*L. major*	64	150	213
LmjF12.1300	*L. major*	68	220	287
LmjF12.1330	*L. major*	48	493	540
LmjF12.1340	*L. major*	160	103	262
LmjF12.1340	*L. major*	62	278	339
LmjF12.1340	*L. major*	51	388	438

LmjF13.0010	*L. major*	73	73	145
LmjF13.0020	*L. major*	41	285	325
LmjF13.0030	*L. major*	51	1	51
LmjF13.0040	*L. major*	148	1	148
LmjF13.0060	*L. major*	56	1	56
LmjF13.0060	*L. major*	114	86	199
LmjF13.0060	*L. major*	93	260	352
LmjF13.0070	*L. major*	41	35	75
LmjF13.0070	*L. major*	40	88	127
LmjF13.0070	*L. major*	177	131	307
LmjF13.0080	*L. major*	52	1378	1429
LmjF13.0100	*L. major*	47	231	277
LmjF13.0100	*L. major*	119	415	533
LmjF13.0110	*L. major*	44	33	76
LmjF13.0120	*L. major*	56	1	56
LmjF13.0130	*L. major*	70	1	70
LmjF13.0130	*L. major*	73	225	297
LmjF13.0130	*L. major*	83	386	468
LmjF13.0130	*L. major*	131	520	650
LmjF13.0140	*L. major*	42	61	102
LmjF13.0150	*L. major*	148	68	215
LmjF13.0170	*L. major*	47	133	179
LmjF13.0170	*L. major*	78	499	576
LmjF13.0180	*L. major*	57	45	101
LmjF13.0190	*L. major*	75	18	92
LmjF13.0190	*L. major*	59	131	189
LmjF13.0200	*L. major*	45	1	45
LmjF13.0200	*L. major*	49	126	174
LmjF13.0200	*L. major*	77	574	650
LmjF13.0210	*L. major*	191	392	582
LmjF13.0230	*L. major*	328	93	420
LmjF13.0240	*L. major*	58	1	58
LmjF13.0240	*L. major*	55	64	118
LmjF13.0240	*L. major*	76	153	228
LmjF13.0250	*L. major*	63	65	127
LmjF13.0260	*L. major*	99	162	260
LmjF13.0270	*L. major*	73	117	189
LmjF13.0270	*L. major*	176	290	465
LmjF13.0270	*L. major*	67	655	721
LmjF13.0270	*L. major*	93	961	1053
LmjF13.0270	*L. major*	97	1153	1249
LmjF13.0270	*L. major*	44	1376	1419
LmjF13.0270	*L. major*	145	1436	1580
LmjF13.0270	*L. major*	53	1665	1717
LmjF13.0400	*L. major*	50	52	101
LmjF13.0400	*L. major*	157	150	306
LmjF13.0400	*L. major*	102	1456	1557

LmjF13.0400	*L. major*	48	1848	1895
LmjF13.0400	*L. major*	100	1932	2031
LmjF13.0410	*L. major*	41	37	77
LmjF13.0410	*L. major*	75	659	733
LmjF13.0410	*L. major*	63	861	923
LmjF13.0430	*L. major*	68	290	357
LmjF13.0430	*L. major*	122	366	487
LmjF13.0430	*L. major*	61	531	591
LmjF13.0470	*L. major*	96	392	487
LmjF13.0480	*L. major*	155	1	155
LmjF13.0480	*L. major*	145	182	326
LmjF13.0480	*L. major*	125	342	466
LmjF13.0490	*L. major*	67	1	67
LmjF13.0490	*L. major*	48	272	319
LmjF13.0490	*L. major*	48	435	482
LmjF13.0520	*L. major*	188	1	188
LmjF13.0520	*L. major*	42	237	278
LmjF13.0530	*L. major*	123	1	123
LmjF13.0530	*L. major*	128	282	409
LmjF13.0530	*L. major*	77	412	488
LmjF13.0530	*L. major*	138	547	684
LmjF13.0530	*L. major*	301	696	996
LmjF13.0550	*L. major*	52	95	146
LmjF13.0550	*L. major*	129	232	360
LmjF13.0550	*L. major*	93	530	622
LmjF13.0560	*L. major*	51	148	198
LmjF13.0580	*L. major*	43	1	43
LmjF13.0580	*L. major*	98	110	207
LmjF13.0580	*L. major*	101	457	557
LmjF13.0580	*L. major*	68	659	726
LmjF13.0580	*L. major*	44	733	776
LmjF13.0580	*L. major*	71	824	894
LmjF13.0580	*L. major*	73	935	1007
LmjF13.0580	*L. major*	59	1043	1101
LmjF13.0580	*L. major*	77	1161	1237
LmjF13.0580	*L. major*	43	1270	1312
LmjF13.0580	*L. major*	45	1352	1396
LmjF13.0580	*L. major*	44	1436	1479
LmjF13.0590	*L. major*	40	211	250
LmjF13.0600	*L. major*	83	1	83
LmjF13.0600	*L. major*	41	112	152
LmjF13.0600	*L. major*	41	112	152
LmjF13.0610	*L. major*	55	122	176
LmjF13.0620	*L. major*	392	1	392
LmjF13.0630	*L. major*	131	62	192
LmjF13.0630	*L. major*	65	238	302
LmjF13.0640	*L. major*	40	778	817

LmjF13.0640	*L. major*	158	1120	1277
LmjF13.0650	*L. major*	158	93	250
LmjF13.0650	*L. major*	83	525	607
LmjF13.0660	*L. major*	62	569	630
LmjF13.0670	*L. major*	224	252	475
LmjF13.0680	*L. major*	162	1	162
LmjF13.0690	*L. major*	173	20	192
LmjF13.0690	*L. major*	556	216	771
LmjF13.0690	*L. major*	40	823	862
LmjF13.0700	*L. major*	51	1	51
LmjF13.0700	*L. major*	476	97	572
LmjF13.0700	*L. major*	63	647	709
LmjF13.0700	*L. major*	127	790	916
LmjF13.0700	*L. major*	72	986	1057
LmjF13.0700	*L. major*	63	1216	1278
LmjF13.0710	*L. major*	302	121	422
LmjF13.0730	*L. major*	366	73	438
LmjF13.0730	*L. major*	48	489	536
LmjF13.0730	*L. major*	240	606	845
LmjF13.0740	*L. major*	116	1	116
LmjF13.0740	*L. major*	45	735	779
LmjF13.0740	*L. major*	45	735	779
LmjF13.0750	*L. major*	138	1	138
LmjF13.0760	*L. major*	225	145	369
LmjF13.0760	*L. major*	297	515	811
LmjF13.0760	*L. major*	291	1087	1377
LmjF13.0760	*L. major*	81	1451	1531
LmjF13.0760	*L. major*	72	1595	1666
LmjF13.0760	*L. major*	52	1690	1741
LmjF13.0760	*L. major*	109	1789	1897
LmjF13.0780	*L. major*	47	210	256
LmjF13.0790	*L. major*	109	65	173
LmjF13.0790	*L. major*	83	183	265
LmjF13.0790	*L. major*	65	308	372
LmjF13.0790	*L. major*	84	592	675
LmjF13.0800	*L. major*	41	1	41
LmjF13.0800	*L. major*	129	97	225
LmjF13.0810	*L. major*	188	1	188
LmjF13.0810	*L. major*	394	213	606
LmjF13.0810	*L. major*	180	628	807
LmjF13.0810	*L. major*	125	858	982
LmjF13.0810	*L. major*	134	997	1130
LmjF13.0810	*L. major*	105	1228	1332
LmjF13.0810	*L. major*	130	1355	1484
LmjF13.0810	*L. major*	243	1496	1738
LmjF13.0810	*L. major*	119	1800	1918
LmjF13.0810	*L. major*	77	1965	2041

LmjF13.0810	*L. major*	51	2329	2379
LmjF13.0810	*L. major*	150	2412	2561
LmjF13.0820	*L. major*	40	116	155
LmjF13.0830	*L. major*	121	1	121
LmjF13.0830	*L. major*	151	152	302
LmjF13.0830	*L. major*	125	424	548
LmjF13.0830	*L. major*	79	601	679
LmjF13.0830	*L. major*	93	682	774
LmjF13.0830	*L. major*	46	820	865
LmjF13.0830	*L. major*	54	1183	1236
LmjF13.0840	*L. major*	58	1	58
LmjF13.0840	*L. major*	112	97	208
LmjF13.0840	*L. major*	52	243	294
LmjF13.0840	*L. major*	151	324	474
LmjF13.0840	*L. major*	69	497	565
LmjF13.0840	*L. major*	407	604	1010
LmjF13.0850	*L. major*	400	1	400
LmjF13.0850	*L. major*	119	462	580
LmjF13.0850	*L. major*	129	614	742
LmjF13.0850	*L. major*	166	760	925
LmjF13.0860	*L. major*	65	105	169
LmjF13.0860	*L. major*	150	239	388
LmjF13.0880	*L. major*	263	80	342
LmjF13.0880	*L. major*	243	456	698
LmjF13.0890	*L. major*	79	103	181
LmjF13.0890	*L. major*	97	234	330
LmjF13.0890	*L. major*	97	234	330
LmjF13.0900	*L. major*	149	51	199
LmjF13.0900	*L. major*	127	299	425
LmjF13.0900	*L. major*	165	455	619
LmjF13.0910	*L. major*	59	48	106
LmjF13.0910	*L. major*	311	210	520
LmjF13.0910	*L. major*	44	788	831
LmjF13.0920	*L. major*	105	1	105
LmjF13.0920	*L. major*	40	183	222
LmjF13.0920	*L. major*	43	255	297
LmjF13.0920	*L. major*	44	319	362
LmjF13.0920	*L. major*	98	419	516
LmjF13.0920	*L. major*	89	547	635
LmjF13.0920	*L. major*	82	800	881
LmjF13.0920	*L. major*	92	883	974
LmjF13.0920	*L. major*	109	1050	1158
LmjF13.0920	*L. major*	203	1265	1467
LmjF13.0940	*L. major*	43	20	62
LmjF13.0940	*L. major*	124	373	496
LmjF13.0950	*L. major*	67	235	301
LmjF13.0960	*L. major*	156	105	260

LmjF13.0970	*L. major*	110	1	110
LmjF13.0970	*L. major*	49	183	231
LmjF13.0970	*L. major*	164	318	481
LmjF13.0980	*L. major*	46	122	167
LmjF13.0980	*L. major*	55	264	318
LmjF13.0980	*L. major*	68	505	572
LmjF13.0980	*L. major*	72	809	880
LmjF13.0980	*L. major*	150	939	1088
LmjF13.0990	*L. major*	375	1	375
LmjF13.0990	*L. major*	47	381	427
LmjF13.0990	*L. major*	50	441	490
LmjF13.1000	*L. major*	62	1	62
LmjF13.1000	*L. major*	66	135	200
LmjF13.1000	*L. major*	144	228	371
LmjF13.1010	*L. major*	44	89	132
LmjF13.1020	*L. major*	73	119	191
LmjF13.1020	*L. major*	60	222	281
LmjF13.1020	*L. major*	49	469	517
LmjF13.1020	*L. major*	42	606	647
LmjF13.1020	*L. major*	61	720	780
LmjF13.1020	*L. major*	45	1053	1097
LmjF13.1020	*L. major*	135	1169	1303
LmjF13.1030	*L. major*	82	1	82
LmjF13.1030	*L. major*	48	261	308
LmjF13.1030	*L. major*	48	261	308
LmjF13.1040	*L. major*	61	437	497
LmjF13.1040	*L. major*	52	561	612
LmjF13.1040	*L. major*	118	641	758
LmjF13.1040	*L. major*	43	782	824
LmjF13.1040	*L. major*	42	835	876
LmjF13.1040	*L. major*	96	1114	1209
LmjF13.1040	*L. major*	49	1274	1322
LmjF13.1070	*L. major*	205	152	356
LmjF13.1070	*L. major*	47	395	441
LmjF13.1070	*L. major*	89	660	748
LmjF13.1070	*L. major*	49	897	945
LmjF13.1070	*L. major*	54	1142	1195
LmjF13.1070	*L. major*	52	1203	1254
LmjF13.1070	*L. major*	60	1640	1699
LmjF13.1070	*L. major*	40	1754	1793
LmjF13.1070	*L. major*	89	1915	2003
LmjF13.1070	*L. major*	67	2068	2134
LmjF13.1070	*L. major*	84	2144	2227
LmjF13.1080	*L. major*	42	88	129
LmjF13.1080	*L. major*	54	138	191
LmjF13.1080	*L. major*	91	634	724
LmjF13.1080	*L. major*	115	818	932

LmjF13.1080	*L. major*	67	1221	1287
LmjF13.1090	*L. major*	45	1	45
LmjF13.1100	*L. major*	49	102	150
LmjF13.1110	*L. major*	454	43	496
LmjF13.1130	*L. major*	63	91	153
LmjF13.1130	*L. major*	108	226	333
LmjF13.1160	*L. major*	96	39	134
LmjF13.1170	*L. major*	119	20	138
LmjF13.1170	*L. major*	104	174	277
LmjF13.1170	*L. major*	75	396	470
LmjF13.1190	*L. major*	114	1	114
LmjF13.1200	*L. major*	74	1	74
LmjF13.1210	*L. major*	105	229	333
LmjF13.1240	*L. major*	41	1	41
LmjF13.1240	*L. major*	75	308	382
LmjF13.1240	*L. major*	42	537	578
LmjF13.1250	*L. major*	76	1	76
LmjF13.1250	*L. major*	80	138	217
LmjF13.1260	*L. major*	64	129	192
LmjF13.1270	*L. major*	40	110	149
LmjF13.1270	*L. major*	52	255	306
LmjF13.1270	*L. major*	113	482	594
LmjF13.1270	*L. major*	72	654	725
LmjF13.1280	*L. major*	41	216	256
LmjF13.1290	*L. major*	198	1	198
LmjF13.1290	*L. major*	222	227	448
LmjF13.1290	*L. major*	43	564	606
LmjF13.1290	*L. major*	83	711	793
LmjF13.1310	*L. major*	109	312	420
LmjF13.1320	*L. major*	154	311	464
LmjF13.1330	*L. major*	51	92	142
LmjF13.1330	*L. major*	61	361	421
LmjF13.1340	*L. major*	159	1	159
LmjF13.1340	*L. major*	153	432	584
LmjF13.1340	*L. major*	137	628	764
LmjF13.1340	*L. major*	109	790	898
LmjF13.1340	*L. major*	220	921	1140
LmjF13.1340	*L. major*	233	1156	1388
LmjF13.1350	*L. major*	71	45	115
LmjF13.1350	*L. major*	99	198	296
LmjF13.1350	*L. major*	116	564	679
LmjF13.1350	*L. major*	63	728	790
LmjF13.1350	*L. major*	69	821	889
LmjF13.1350	*L. major*	46	969	1014
LmjF13.1350	*L. major*	83	1016	1098
LmjF13.1360	*L. major*	145	383	527
LmjF13.1360	*L. major*	73	611	683

LmjF13.1360	*L. major*	73	611	683
LmjF13.1370	*L. major*	57	55	111
LmjF13.1370	*L. major*	115	160	274
LmjF13.1370	*L. major*	43	407	449
LmjF13.1370	*L. major*	66	471	536
LmjF13.1390	*L. major*	47	1	47
LmjF13.1400	*L. major*	48	541	588
LmjF13.1410	*L. major*	250	1	250
LmjF13.1410	*L. major*	271	260	530
LmjF13.1410	*L. major*	251	585	835
LmjF13.1410	*L. major*	241	848	1088
LmjF13.1410	*L. major*	254	1156	1409
LmjF13.1420	*L. major*	89	1	89
LmjF13.1420	*L. major*	62	133	194
LmjF13.1420	*L. major*	62	133	194
LmjF13.1430	*L. major*	90	18	107
LmjF13.1430	*L. major*	175	295	469
LmjF13.1430	*L. major*	132	502	633
LmjF13.1440	*L. major*	121	379	499
LmjF13.1440	*L. major*	80	596	675
LmjF13.1440	*L. major*	50	780	829
LmjF13.1450	*L. major*	66	1	66
LmjF13.1460	*L. major*	58	11	68
LmjF13.1470	*L. major*	62	1	62
LmjF13.1470	*L. major*	63	258	320
LmjF13.1470	*L. major*	50	747	796
LmjF13.1470	*L. major*	93	1195	1287
LmjF13.1470	*L. major*	72	1405	1476
LmjF13.1470	*L. major*	56	1672	1727
LmjF13.1470	*L. major*	53	1928	1980
LmjF13.1470	*L. major*	83	2053	2135
LmjF13.1470	*L. major*	59	2158	2216
LmjF13.1470	*L. major*	119	2386	2504
LmjF13.1470	*L. major*	40	2614	2653
LmjF13.1470	*L. major*	41	2814	2854
LmjF13.1470	*L. major*	209	3100	3308
LmjF13.1470	*L. major*	194	4104	4297
LmjF13.1470	*L. major*	43	4638	4680
LmjF13.1470	*L. major*	46	4744	4789
LmjF13.1470	*L. major*	42	4922	4963
LmjF13.1470	*L. major*	180	5380	5559
LmjF13.1490	*L. major*	43	312	354
LmjF13.1490	*L. major*	46	366	411
LmjF13.1490	*L. major*	189	577	765
LmjF13.1500	*L. major*	640	1	640
LmjF13.1500	*L. major*	214	664	877
LmjF13.1500	*L. major*	45	1033	1077

LmjF13.1510	*L. major*	82	1	82
LmjF13.1520	*L. major*	62	166	227
LmjF13.1520	*L. major*	52	284	335
LmjF13.1520	*L. major*	84	365	448
LmjF13.1520	*L. major*	79	502	580
LmjF13.1540	*L. major*	83	60	142
LmjF13.1540	*L. major*	66	334	399
LmjF13.1540	*L. major*	66	334	399
LmjF13.1550	*L. major*	44	485	528
LmjF13.1550	*L. major*	177	758	934
LmjF13.1550	*L. major*	200	944	1143
LmjF13.1570	*L. major*	58	89	146
LmjF13.1570	*L. major*	76	169	244
LmjF13.1570	*L. major*	76	169	244
LmjF13.1590	*L. major*	617	1	617
LmjF13.1590	*L. major*	44	707	750
LmjF13.1600	*L. major*	201	100	300
LmjF13.1600	*L. major*	153	337	489
LmjF13.1600	*L. major*	63	518	580
LmjF13.1600	*L. major*	355	618	972
LmjF13.1600	*L. major*	46	1015	1060
LmjF13.1600	*L. major*	44	1091	1134
LmjF13.1610	*L. major*	148	58	205
LmjF13.1610	*L. major*	152	512	663
LmjF13.1640	*L. major*	53	553	605
LmjF13.1650	*L. major*	62	1202	1263
LmjF13.1650	*L. major*	90	3145	3234
LmjF13.1650	*L. major*	62	3359	3420
LmjF13.1650	*L. major*	75	4075	4149
LmjF13.1650	*L. major*	60	4380	4439
LmjF13.1670	*L. major*	49	30	78
LmjF14.0030	*L. major*	42	35	76
LmjF14.0030	*L. major*	110	130	239
LmjF14.0040	*L. major*	86	92	177
LmjF14.0050	*L. major*	105	629	733
LmjF14.0070	*L. major*	42	16	57
LmjF14.0090	*L. major*	113	88	200
LmjF14.0090	*L. major*	129	217	345
LmjF14.0100	*L. major*	54	1	54
LmjF14.0100	*L. major*	52	322	373
LmjF14.0100	*L. major*	48	391	438
LmjF14.0110	*L. major*	62	379	440
LmjF14.0110	*L. major*	49	475	523
LmjF14.0110	*L. major*	49	475	523
LmjF14.0120	*L. major*	155	1	155
LmjF14.0120	*L. major*	113	210	322
LmjF14.0140	*L. major*	76	57	132

LmjF14.0140	*L. major*	54	185	238
LmjF14.0150	*L. major*	48	76	123
LmjF14.0160	*L. major*	65	54	118
LmjF14.0170	*L. major*	40	1	40
LmjF14.0170	*L. major*	126	187	312
LmjF14.0170	*L. major*	56	630	685
LmjF14.0190	*L. major*	43	157	199
LmjF14.0200	*L. major*	152	39	190
LmjF14.0210	*L. major*	46	1	46
LmjF14.0210	*L. major*	43	59	101
LmjF14.0210	*L. major*	52	154	205
LmjF14.0210	*L. major*	53	339	391
LmjF14.0220	*L. major*	73	157	229
LmjF14.0230	*L. major*	112	149	260
LmjF14.0230	*L. major*	44	323	366
LmjF14.0230	*L. major*	77	439	515
LmjF14.0230	*L. major*	50	548	597
LmjF14.0230	*L. major*	71	618	688
LmjF14.0230	*L. major*	78	821	898
LmjF14.0230	*L. major*	75	1143	1217
LmjF14.0230	*L. major*	272	1572	1843
LmjF14.0260	*L. major*	43	362	404
LmjF14.0260	*L. major*	40	593	632
LmjF14.0260	*L. major*	40	593	632
LmjF14.0270	*L. major*	52	1	52
LmjF14.0280	*L. major*	141	824	964
LmjF14.0290	*L. major*	46	1	46
LmjF14.0300	*L. major*	83	74	156
LmjF14.0310	*L. major*	53	233	285
LmjF14.0320	*L. major*	43	589	631
LmjF14.0320	*L. major*	153	651	803
LmjF14.0330	*L. major*	185	1	185
LmjF14.0340	*L. major*	397	1	397
LmjF14.0340	*L. major*	109	427	535
LmjF14.0340	*L. major*	95	550	644
LmjF14.0340	*L. major*	121	717	837
LmjF14.0340	*L. major*	43	927	969
LmjF14.0350	*L. major*	56	621	676
LmjF14.0360	*L. major*	202	70	271
LmjF14.0360	*L. major*	60	408	467
LmjF14.0360	*L. major*	141	598	738
LmjF14.0360	*L. major*	207	772	978
LmjF14.0360	*L. major*	56	984	1039
LmjF14.0370	*L. major*	381	46	426
LmjF14.0370	*L. major*	465	428	892
LmjF14.0370	*L. major*	69	902	970
LmjF14.0370	*L. major*	673	981	1653

LmjF14.0370	*L. major*	183	1658	1840
LmjF14.0370	*L. major*	566	1867	2432
LmjF14.0370	*L. major*	282	2452	2733
LmjF14.0370	*L. major*	70	2745	2814
LmjF14.0370	*L. major*	70	2852	2921
LmjF14.0380	*L. major*	58	45	102
LmjF14.0405	*L. major*	154	1	154
LmjF14.0410	*L. major*	336	1	336
LmjF14.0410	*L. major*	100	563	662
LmjF14.0420	*L. major*	156	171	326
LmjF14.0420	*L. major*	479	339	817
LmjF14.0450	*L. major*	121	40	160
LmjF14.0450	*L. major*	61	268	328
LmjF14.0500	*L. major*	83	1	83
LmjF14.0500	*L. major*	200	93	292
LmjF14.0500	*L. major*	50	305	354
LmjF14.0500	*L. major*	103	398	500
LmjF14.0520	*L. major*	71	1	71
LmjF14.0530	*L. major*	134	340	473
LmjF14.0540	*L. major*	44	413	456
LmjF14.0550	*L. major*	69	48	116
LmjF14.0550	*L. major*	40	517	556
LmjF14.0550	*L. major*	40	517	556
LmjF14.0560	*L. major*	147	315	461
LmjF14.0570	*L. major*	49	27	75
LmjF14.0570	*L. major*	92	406	497
LmjF14.0570	*L. major*	46	727	772
LmjF14.0570	*L. major*	57	939	995
LmjF14.0570	*L. major*	44	1088	1131
LmjF14.0590	*L. major*	101	315	415
LmjF14.0590	*L. major*	44	434	477
LmjF14.0590	*L. major*	162	632	793
LmjF14.0590	*L. major*	97	883	979
LmjF14.0590	*L. major*	212	1037	1248
LmjF14.0590	*L. major*	123	1256	1378
LmjF14.0600	*L. major*	92	1	92
LmjF14.0600	*L. major*	79	131	209
LmjF14.0610	*L. major*	107	56	162
LmjF14.0610	*L. major*	44	185	228
LmjF14.0610	*L. major*	63	268	330
LmjF14.0610	*L. major*	103	368	470
LmjF14.0620	*L. major*	90	31	120
LmjF14.0620	*L. major*	192	194	385
LmjF14.0620	*L. major*	238	431	668
LmjF14.0625	*L. major*	156	71	226
LmjF14.0750	*L. major*	122	22	143
LmjF14.0750	*L. major*	58	211	268

LmjF14.0750	*L. major*	99	289	387
LmjF14.0750	*L. major*	161	1079	1239
LmjF14.0750	*L. major*	104	1243	1346
LmjF14.0750	*L. major*	85	1361	1445
LmjF14.0750	*L. major*	477	1481	1957
LmjF14.0760	*L. major*	82	1	82
LmjF14.0760	*L. major*	111	306	416
LmjF14.0770	*L. major*	108	80	187
LmjF14.0770	*L. major*	123	203	325
LmjF14.0790	*L. major*	73	1	73
LmjF14.0790	*L. major*	190	169	358
LmjF14.0790	*L. major*	54	389	442
LmjF14.0790	*L. major*	81	616	696
LmjF14.0790	*L. major*	83	723	805
LmjF14.0790	*L. major*	176	852	1027
LmjF14.0790	*L. major*	43	1050	1092
LmjF14.0790	*L. major*	152	1161	1312
LmjF14.0790	*L. major*	49	1403	1451
LmjF14.0790	*L. major*	67	1498	1564
LmjF14.0790	*L. major*	130	1670	1799
LmjF14.0790	*L. major*	72	1806	1877
LmjF14.0790	*L. major*	48	1882	1929
LmjF14.0790	*L. major*	139	2013	2151
LmjF14.0790	*L. major*	106	2196	2301
LmjF14.0790	*L. major*	106	2308	2413
LmjF14.0790	*L. major*	1475	2449	3923
LmjF14.0790	*L. major*	73	3952	4024
LmjF14.0790	*L. major*	125	4041	4165
LmjF14.0800	*L. major*	429	1	429
LmjF14.0810	*L. major*	87	76	162
LmjF14.0810	*L. major*	178	603	780
LmjF14.0810	*L. major*	131	829	959
LmjF14.0810	*L. major*	255	973	1227
LmjF14.0810	*L. major*	62	1254	1315
LmjF14.0820	*L. major*	56	82	137
LmjF14.0820	*L. major*	140	144	283
LmjF14.0820	*L. major*	264	314	577
LmjF14.0830	*L. major*	406	1	406
LmjF14.0830	*L. major*	125	608	732
LmjF14.0830	*L. major*	95	819	913
LmjF14.0840	*L. major*	205	1	205
LmjF14.0840	*L. major*	228	219	446
LmjF14.0840	*L. major*	40	743	782
LmjF14.0840	*L. major*	116	1041	1156
LmjF14.0840	*L. major*	92	1161	1252
LmjF14.0850	*L. major*	48	68	115
LmjF14.0880	*L. major*	87	1	87

LmjF14.0880	*L. major*	69	110	178
LmjF14.0880	*L. major*	65	253	317
LmjF14.0880	*L. major*	72	395	466
LmjF14.0880	*L. major*	105	475	579
LmjF14.0880	*L. major*	231	637	867
LmjF14.0880	*L. major*	51	913	963
LmjF14.0890	*L. major*	49	172	220
LmjF14.0890	*L. major*	288	262	549
LmjF14.0890	*L. major*	68	594	661
LmjF14.0900	*L. major*	302	1	302
LmjF14.0900	*L. major*	88	322	409
LmjF14.0900	*L. major*	48	637	684
LmjF14.0900	*L. major*	58	797	854
LmjF14.0920	*L. major*	59	1	59
LmjF14.0920	*L. major*	89	418	506
LmjF14.0920	*L. major*	40	957	996
LmjF14.0920	*L. major*	44	1116	1159
LmjF14.0930	*L. major*	44	164	207
LmjF14.0930	*L. major*	107	368	474
LmjF14.0930	*L. major*	40	529	568
LmjF14.0930	*L. major*	94	638	731
LmjF14.0940	*L. major*	62	1	62
LmjF14.0940	*L. major*	315	102	416
LmjF14.0940	*L. major*	169	418	586
LmjF14.0940	*L. major*	63	630	692
LmjF14.0940	*L. major*	110	765	874
LmjF14.0960	*L. major*	219	1	219
LmjF14.0960	*L. major*	56	397	452
LmjF14.0960	*L. major*	46	717	762
LmjF14.0960	*L. major*	41	816	856
LmjF14.0960	*L. major*	50	940	989
LmjF14.0960	*L. major*	77	1039	1115
LmjF14.0980	*L. major*	99	548	646
LmjF14.1000	*L. major*	47	1	47
LmjF14.1010	*L. major*	179	1	179
LmjF14.1010	*L. major*	41	196	236
LmjF14.1010	*L. major*	40	620	659
LmjF14.1010	*L. major*	53	825	877
LmjF14.1010	*L. major*	51	967	1017
LmjF14.1020	*L. major*	114	66	179
LmjF14.1020	*L. major*	41	186	226
LmjF14.1020	*L. major*	54	261	314
LmjF14.1060	*L. major*	59	1577	1635
LmjF14.1060	*L. major*	46	1851	1896
LmjF14.1060	*L. major*	65	2018	2082
LmjF14.1060	*L. major*	117	2628	2744
LmjF14.1060	*L. major*	46	2852	2897

LmjF14.1060	*L. major*	72	3282	3353
LmjF14.1060	*L. major*	74	3827	3900
LmjF14.1060	*L. major*	84	3915	3998
LmjF14.1070	*L. major*	228	1	228
LmjF14.1070	*L. major*	74	243	316
LmjF14.1070	*L. major*	51	380	430
LmjF14.1070	*L. major*	80	489	568
LmjF14.1070	*L. major*	66	803	868
LmjF14.1080	*L. major*	46	150	195
LmjF14.1095	*L. major*	52	184	235
LmjF14.1095	*L. major*	45	295	339
LmjF14.1095	*L. major*	45	295	339
LmjF14.1100	*L. major*	42	1	42
LmjF14.1100	*L. major*	63	244	306
LmjF14.1100	*L. major*	232	391	622
LmjF14.1100	*L. major*	2109	657	2765
LmjF14.1110	*L. major*	40	1	40
LmjF14.1110	*L. major*	63	214	276
LmjF14.1110	*L. major*	219	361	579
LmjF14.1110	*L. major*	508	646	1153
LmjF14.1120	*L. major*	63	188	250
LmjF14.1120	*L. major*	159	335	493
LmjF14.1120	*L. major*	171	554	724
LmjF14.1120	*L. major*	2158	819	2976
LmjF14.1130	*L. major*	204	1	204
LmjF14.1130	*L. major*	110	304	413
LmjF14.1130	*L. major*	79	663	741
LmjF14.1140	*L. major*	54	152	205
LmjF14.1140	*L. major*	45	432	476
LmjF14.1140	*L. major*	45	432	476
LmjF14.1150	*L. major*	64	191	254
LmjF14.1155	*L. major*	56	1	56
LmjF14.1155	*L. major*	58	117	174
LmjF14.1155	*L. major*	128	178	305
LmjF14.1170	*L. major*	144	83	226
LmjF14.1170	*L. major*	43	281	323
LmjF14.1170	*L. major*	147	502	648
LmjF14.1170	*L. major*	79	688	766
LmjF14.1180	*L. major*	63	432	494
LmjF14.1180	*L. major*	139	562	700
LmjF14.1200	*L. major*	82	1	82
LmjF14.1210	*L. major*	65	85	149
LmjF14.1220	*L. major*	151	55	205
LmjF14.1220	*L. major*	170	221	390
LmjF14.1220	*L. major*	100	420	519
LmjF14.1220	*L. major*	168	576	743
LmjF14.1220	*L. major*	96	840	935

LmjF14.1230	*L. major*	48	299	346
LmjF14.1230	*L. major*	44	411	454
LmjF14.1230	*L. major*	54	488	541
LmjF14.1240	*L. major*	74	121	194
LmjF14.1240	*L. major*	58	245	302
LmjF14.1270	*L. major*	56	66	121
LmjF14.1280	*L. major*	40	64	103
LmjF14.1280	*L. major*	45	200	244
LmjF14.1280	*L. major*	127	287	413
LmjF14.1280	*L. major*	51	448	498
LmjF14.1290	*L. major*	87	14	100
LmjF14.1290	*L. major*	168	252	419
LmjF14.1290	*L. major*	369	433	801
LmjF14.1300	*L. major*	68	1	68
LmjF14.1300	*L. major*	247	381	627
LmjF14.1310	*L. major*	193	17	209
LmjF14.1310	*L. major*	50	280	329
LmjF14.1310	*L. major*	50	280	329
LmjF14.1330	*L. major*	165	400	564
LmjF14.1350	*L. major*	65	1	65
LmjF14.1350	*L. major*	62	109	170
LmjF14.1350	*L. major*	48	331	378
LmjF14.1370	*L. major*	50	215	264
LmjF14.1380	*L. major*	47	271	317
LmjF14.1395	*L. major*	41	1	41
LmjF14.1400	*L. major*	53	1	53
LmjF14.1400	*L. major*	40	56	95
LmjF14.1400	*L. major*	53	222	274
LmjF14.1400	*L. major*	73	546	618
LmjF14.1400	*L. major*	57	901	957
LmjF14.1410	*L. major*	265	291	555
LmjF14.1420	*L. major*	49	484	532
LmjF14.1430	*L. major*	131	761	891
LmjF14.1430	*L. major*	122	938	1059
LmjF14.1430	*L. major*	157	1151	1307
LmjF14.1430	*L. major*	121	1405	1525
LmjF14.1430	*L. major*	41	1556	1596
LmjF14.1440	*L. major*	100	1	100
LmjF14.1440	*L. major*	397	534	930
LmjF14.1450	*L. major*	42	1	42
LmjF14.1470	*L. major*	171	25	195
LmjF14.1470	*L. major*	65	237	301
LmjF14.1470	*L. major*	59	343	401
LmjF14.1470	*L. major*	74	411	484
LmjF14.1480	*L. major*	40	228	267
LmjF14.1490	*L. major*	53	33	85
LmjF14.1490	*L. major*	45	727	771

LmjF15.0010	*L. major*	50	1	50
LmjF15.0050	*L. major*	41	1	41
LmjF15.0050	*L. major*	42	238	279
LmjF15.0050	*L. major*	42	238	279
LmjF15.0060	*L. major*	104	1	104
LmjF15.0060	*L. major*	67	168	234
LmjF15.0060	*L. major*	64	264	327
LmjF15.0060	*L. major*	169	430	598
LmjF15.0070	*L. major*	146	111	256
LmjF15.0070	*L. major*	138	331	468
LmjF15.0080	*L. major*	96	190	285
LmjF15.0090	*L. major*	45	106	150
LmjF15.0090	*L. major*	45	155	199
LmjF15.0090	*L. major*	45	155	199
LmjF15.0110	*L. major*	56	196	251
LmjF15.0110	*L. major*	61	324	384
LmjF15.0110	*L. major*	60	653	712
LmjF15.0130	*L. major*	62	241	302
LmjF15.0130	*L. major*	169	480	648
LmjF15.0140	*L. major*	141	41	181
LmjF15.0160	*L. major*	230	1	230
LmjF15.0160	*L. major*	304	236	539
LmjF15.0160	*L. major*	67	592	658
LmjF15.0160	*L. major*	61	660	720
LmjF15.0170	*L. major*	131	1	131
LmjF15.0180	*L. major*	78	1	78
LmjF15.0180	*L. major*	93	429	521
LmjF15.0185	*L. major*	184	1	184
LmjF15.0185	*L. major*	386	201	586
LmjF15.0195	*L. major*	74	94	167
LmjF15.0195	*L. major*	40	228	267
LmjF15.0195	*L. major*	40	228	267
LmjF15.0200	*L. major*	46	177	222
LmjF15.0210	*L. major*	82	211	292
LmjF15.0210	*L. major*	108	352	459
LmjF15.0223	*L. major*	59	157	215
LmjF15.0230	*L. major*	64	1	64
LmjF15.0250	*L. major*	176	1	176
LmjF15.0250	*L. major*	95	253	347
LmjF15.0250	*L. major*	65	409	473
LmjF15.0250	*L. major*	83	506	588
LmjF15.0250	*L. major*	45	604	648
LmjF15.0250	*L. major*	88	655	742
LmjF15.0250	*L. major*	138	774	911
LmjF15.0250	*L. major*	62	935	996
LmjF15.0250	*L. major*	43	1101	1143
LmjF15.0250	*L. major*	44	1355	1398

LmjF15.0250	*L. major*	58	1551	1608
LmjF15.0260	*L. major*	177	44	220
LmjF15.0260	*L. major*	82	268	349
LmjF15.0260	*L. major*	89	381	469
LmjF15.0290	*L. major*	67	50	116
LmjF15.0290	*L. major*	217	584	800
LmjF15.0310	*L. major*	64	30	93
LmjF15.0310	*L. major*	104	105	208
LmjF15.0310	*L. major*	132	234	365
LmjF15.0310	*L. major*	236	393	628
LmjF15.0320	*L. major*	71	120	190
LmjF15.0320	*L. major*	56	231	286
LmjF15.0320	*L. major*	44	311	354
LmjF15.0320	*L. major*	60	649	708
LmjF15.0320	*L. major*	46	716	761
LmjF15.0330	*L. major*	412	83	494
LmjF15.0330	*L. major*	85	737	821
LmjF15.0330	*L. major*	42	863	904
LmjF15.0330	*L. major*	45	948	992
LmjF15.0340	*L. major*	173	1	173
LmjF15.0340	*L. major*	112	207	318
LmjF15.0340	*L. major*	100	321	420
LmjF15.0350	*L. major*	63	257	319
LmjF15.0360	*L. major*	64	246	309
LmjF15.0360	*L. major*	102	526	627
LmjF15.0360	*L. major*	109	663	771
LmjF15.0360	*L. major*	98	803	900
LmjF15.0360	*L. major*	58	959	1016
LmjF15.0370	*L. major*	51	58	108
LmjF15.0380	*L. major*	51	58	108
LmjF15.0400	*L. major*	52	34	85
LmjF15.0400	*L. major*	46	205	250
LmjF15.0400	*L. major*	59	280	338
LmjF15.0400	*L. major*	90	375	464
LmjF15.0410	*L. major*	77	403	479
LmjF15.0420	*L. major*	50	1	50
LmjF15.0430	*L. major*	70	46	115
LmjF15.0430	*L. major*	105	184	288
LmjF15.0490	*L. major*	395	1	395
LmjF15.0490	*L. major*	52	590	641
LmjF15.0500	*L. major*	111	1	111
LmjF15.0500	*L. major*	441	202	642
LmjF15.0500	*L. major*	94	823	916
LmjF15.0520	*L. major*	245	137	381
LmjF15.0530	*L. major*	562	1	562
LmjF15.0530	*L. major*	68	643	710
LmjF15.0530	*L. major*	212	748	959

LmjF15.0530	*L. major*	243	1019	1261
LmjF15.0530	*L. major*	48	1604	1651
LmjF15.0530	*L. major*	45	1685	1729
LmjF15.0540	*L. major*	58	1	58
LmjF15.0540	*L. major*	123	192	314
LmjF15.0540	*L. major*	136	319	454
LmjF15.0540	*L. major*	123	557	679
LmjF15.0550	*L. major*	42	1	42
LmjF15.0550	*L. major*	79	120	198
LmjF15.0550	*L. major*	48	214	261
LmjF15.0550	*L. major*	60	389	448
LmjF15.0550	*L. major*	220	603	822
LmjF15.0550	*L. major*	107	857	963
LmjF15.0550	*L. major*	103	990	1092
LmjF15.0550	*L. major*	121	1137	1257
LmjF15.0550	*L. major*	114	1344	1457
LmjF15.0550	*L. major*	114	1613	1726
LmjF15.0560	*L. major*	111	1	111
LmjF15.0560	*L. major*	216	125	340
LmjF15.0560	*L. major*	83	366	448
LmjF15.0560	*L. major*	69	474	542
LmjF15.0570	*L. major*	74	97	170
LmjF15.0580	*L. major*	42	76	117
LmjF15.0580	*L. major*	46	187	232
LmjF15.0580	*L. major*	46	187	232
LmjF15.0590	*L. major*	118	40	157
LmjF15.0590	*L. major*	59	167	225
LmjF15.0600	*L. major*	109	17	125
LmjF15.0600	*L. major*	46	144	189
LmjF15.0600	*L. major*	333	221	553
LmjF15.0600	*L. major*	434	723	1156
LmjF15.0610	*L. major*	40	375	414
LmjF15.0620	*L. major*	44	1	44
LmjF15.0620	*L. major*	95	258	352
LmjF15.0630	*L. major*	489	51	539
LmjF15.0630	*L. major*	506	550	1055
LmjF15.0630	*L. major*	173	1098	1270
LmjF15.0640	*L. major*	190	74	263
LmjF15.0640	*L. major*	103	300	402
LmjF15.0640	*L. major*	105	806	910
LmjF15.0640	*L. major*	80	963	1042
LmjF15.0640	*L. major*	75	1108	1182
LmjF15.0640	*L. major*	172	1201	1372
LmjF15.0650	*L. major*	391	1	391
LmjF15.0650	*L. major*	139	501	639
LmjF15.0660	*L. major*	176	1	176
LmjF15.0660	*L. major*	57	216	272

LmjF15.0660	*L. major*	176	286	461
LmjF15.0670	*L. major*	112	1	112
LmjF15.0670	*L. major*	66	121	186
LmjF15.0670	*L. major*	62	225	286
LmjF15.0670	*L. major*	43	410	452
LmjF15.0670	*L. major*	41	686	726
LmjF15.0670	*L. major*	81	795	875
LmjF15.0680	*L. major*	59	1	59
LmjF15.0680	*L. major*	43	159	201
LmjF15.0680	*L. major*	43	159	201
LmjF15.0700	*L. major*	45	1	45
LmjF15.0700	*L. major*	96	162	257
LmjF15.0700	*L. major*	63	281	343
LmjF15.0700	*L. major*	103	386	488
LmjF15.0700	*L. major*	151	545	695
LmjF15.0700	*L. major*	61	771	831
LmjF15.0700	*L. major*	50	955	1004
LmjF15.0710	*L. major*	82	72	153
LmjF15.0710	*L. major*	58	206	263
LmjF15.0710	*L. major*	54	382	435
LmjF15.0710	*L. major*	59	743	801
LmjF15.0710	*L. major*	48	867	914
LmjF15.0710	*L. major*	66	1124	1189
LmjF15.0710	*L. major*	45	1220	1264
LmjF15.0710	*L. major*	230	1915	2144
LmjF15.0710	*L. major*	68	2177	2244
LmjF15.0710	*L. major*	41	2624	2664
LmjF15.0720	*L. major*	96	91	186
LmjF15.0720	*L. major*	49	302	350
LmjF15.0730	*L. major*	61	1	61
LmjF15.0730	*L. major*	72	86	157
LmjF15.0730	*L. major*	72	86	157
LmjF15.0750	*L. major*	341	1	341
LmjF15.0750	*L. major*	119	348	466
LmjF15.0750	*L. major*	91	587	677
LmjF15.0760	*L. major*	133	1	133
LmjF15.0760	*L. major*	66	740	805
LmjF15.0760	*L. major*	265	1147	1411
LmjF15.0760	*L. major*	70	1890	1959
LmjF15.0760	*L. major*	163	1969	2131
LmjF15.0760	*L. major*	67	2368	2434
LmjF15.0760	*L. major*	131	2561	2691
LmjF15.0770	*L. major*	92	363	454
LmjF15.0770	*L. major*	210	457	666
LmjF15.0780	*L. major*	85	571	655
LmjF15.0790	*L. major*	62	75	136
LmjF15.0790	*L. major*	113	353	465

LmjF15.0790	*L. major*	60	852	911
LmjF15.0790	*L. major*	45	1005	1049
LmjF15.0790	*L. major*	65	1134	1198
LmjF15.0790	*L. major*	54	1258	1311
LmjF15.0800	*L. major*	287	1	287
LmjF15.0800	*L. major*	132	306	437
LmjF15.0800	*L. major*	125	517	641
LmjF15.0800	*L. major*	445	661	1105
LmjF15.0800	*L. major*	199	1112	1310
LmjF15.0800	*L. major*	55	1318	1372
LmjF15.0800	*L. major*	294	1384	1677
LmjF15.0800	*L. major*	88	1712	1799
LmjF15.0800	*L. major*	137	1865	2001
LmjF15.0800	*L. major*	77	2007	2083
LmjF15.0810	*L. major*	49	337	385
LmjF15.0810	*L. major*	41	452	492
LmjF15.0810	*L. major*	41	452	492
LmjF15.0820	*L. major*	41	913	953
LmjF15.0825	*L. major*	109	1	109
LmjF15.0825	*L. major*	46	121	166
LmjF15.0825	*L. major*	73	174	246
LmjF15.0825	*L. major*	46	251	296
LmjF15.0830	*L. major*	97	1	97
LmjF15.0830	*L. major*	250	169	418
LmjF15.0830	*L. major*	373	432	804
LmjF15.0850	*L. major*	168	15	182
LmjF15.0850	*L. major*	69	218	286
LmjF15.0850	*L. major*	42	452	493
LmjF15.0850	*L. major*	66	507	572
LmjF15.0860	*L. major*	271	39	309
LmjF15.0870	*L. major*	46	260	305
LmjF15.0870	*L. major*	67	331	397
LmjF15.0870	*L. major*	67	331	397
LmjF15.0880	*L. major*	89	193	281
LmjF15.0890	*L. major*	85	657	741
LmjF15.0900	*L. major*	70	1	70
LmjF15.0900	*L. major*	65	160	224
LmjF15.0900	*L. major*	65	160	224
LmjF15.0910	*L. major*	105	56	160
LmjF15.0910	*L. major*	45	199	243
LmjF15.0910	*L. major*	199	290	488
LmjF15.0910	*L. major*	168	512	679
LmjF15.0910	*L. major*	51	706	756
LmjF15.0910	*L. major*	122	837	958
LmjF15.0910	*L. major*	103	1254	1356
LmjF15.0910	*L. major*	306	1359	1664
LmjF15.0910	*L. major*	41	1706	1746

LmjF15.0910	*L. major*	85	1786	1870
LmjF15.0910	*L. major*	101	1998	2098
LmjF15.0910	*L. major*	119	2152	2270
LmjF15.0910	*L. major*	61	2444	2504
LmjF15.0920	*L. major*	129	1	129
LmjF15.0920	*L. major*	78	533	610
LmjF15.0940	*L. major*	118	119	236
LmjF15.0940	*L. major*	171	332	502
LmjF15.0940	*L. major*	89	530	618
LmjF15.0940	*L. major*	62	646	707
LmjF15.0940	*L. major*	68	785	852
LmjF15.0940	*L. major*	41	889	929
LmjF15.0940	*L. major*	250	948	1197
LmjF15.0960	*L. major*	67	123	189
LmjF15.0960	*L. major*	86	203	288
LmjF15.0970	*L. major*	119	278	396
LmjF15.0980	*L. major*	90	14	103
LmjF15.0980	*L. major*	150	319	468
LmjF15.0980	*L. major*	215	569	783
LmjF15.0990	*L. major*	41	157	197
LmjF15.1000	*L. major*	70	93	162
LmjF15.1010	*L. major*	48	1	48
LmjF15.1010	*L. major*	53	661	713
LmjF15.1010	*L. major*	53	661	713
LmjF15.1020	*L. major*	51	309	359
LmjF15.1030	*L. major*	132	1	132
LmjF15.1030	*L. major*	263	165	427
LmjF15.1050	*L. major*	55	62	116
LmjF15.1070	*L. major*	55	62	116
LmjF15.1090	*L. major*	55	62	116
LmjF15.1110	*L. major*	55	62	116
LmjF15.1130	*L. major*	55	62	116
LmjF15.1150	*L. major*	55	62	116
LmjF15.1170	*L. major*	52	67	118
LmjF15.1170	*L. major*	52	290	341
LmjF15.1170	*L. major*	62	367	428
LmjF15.1180	*L. major*	59	213	271
LmjF15.1180	*L. major*	43	364	406
LmjF15.1180	*L. major*	80	442	521
LmjF15.1180	*L. major*	44	723	766
LmjF15.1180	*L. major*	61	986	1046
LmjF15.1180	*L. major*	63	1078	1140
LmjF15.1180	*L. major*	40	1406	1445
LmjF15.1180	*L. major*	185	1663	1847
LmjF15.1180	*L. major*	71	2211	2281
LmjF15.1180	*L. major*	210	2438	2647
LmjF15.1180	*L. major*	499	2669	3167

LmjF15.1190	*L. major*	269	42	310
LmjF15.1190	*L. major*	56	400	455
LmjF15.1190	*L. major*	108	936	1043
LmjF15.1200	*L. major*	103	1	103
LmjF15.1200	*L. major*	240	137	376
LmjF15.1200	*L. major*	91	610	700
LmjF15.1200	*L. major*	73	991	1063
LmjF15.1200	*L. major*	82	1067	1148
LmjF15.1200	*L. major*	411	1596	2006
LmjF15.1200	*L. major*	252	2050	2301
LmjF15.1200	*L. major*	136	2327	2462
LmjF15.1200	*L. major*	168	2483	2650
LmjF15.1203	*L. major*	53	59	111
LmjF15.1207	*L. major*	53	59	111
LmjF15.1210	*L. major*	98	354	451
LmjF15.1220	*L. major*	111	15	125
LmjF15.1220	*L. major*	65	414	478
LmjF15.1230	*L. major*	57	1	57
LmjF15.1230	*L. major*	51	85	135
LmjF15.1230	*L. major*	69	410	478
LmjF15.1240	*L. major*	69	245	313
LmjF15.1260	*L. major*	47	44	90
LmjF15.1260	*L. major*	71	337	407
LmjF15.1260	*L. major*	71	337	407
LmjF15.1270	*L. major*	50	1	50
LmjF15.1270	*L. major*	89	252	340
LmjF15.1270	*L. major*	76	458	533
LmjF15.1280	*L. major*	87	67	153
LmjF15.1280	*L. major*	102	200	301
LmjF15.1280	*L. major*	251	395	645
LmjF15.1280	*L. major*	365	676	1040
LmjF15.1280	*L. major*	79	1067	1145
LmjF15.1280	*L. major*	69	1189	1257
LmjF15.1280	*L. major*	180	1369	1548
LmjF15.1280	*L. major*	49	1594	1642
LmjF15.1280	*L. major*	131	1659	1789
LmjF15.1290	*L. major*	44	390	433
LmjF15.1290	*L. major*	65	1149	1213
LmjF15.1290	*L. major*	65	1149	1213
LmjF15.1300	*L. major*	110	1	110
LmjF15.1300	*L. major*	148	900	1047
LmjF15.1310	*L. major*	77	1	77
LmjF15.1310	*L. major*	127	93	219
LmjF15.1310	*L. major*	98	251	348
LmjF15.1310	*L. major*	69	413	481
LmjF15.1320	*L. major*	41	1	41
LmjF15.1320	*L. major*	227	63	289

LmjF15.1320	*L. major*	70	340	409
LmjF15.1320	*L. major*	116	705	820
LmjF15.1320	*L. major*	253	1173	1425
LmjF15.1330	*L. major*	51	240	290
LmjF15.1340	*L. major*	87	152	238
LmjF15.1345	*L. major*	67	1	67
LmjF15.1345	*L. major*	221	83	303
LmjF15.1350	*L. major*	51	1	51
LmjF15.1350	*L. major*	86	86	171
LmjF15.1355	*L. major*	47	150	196
LmjF15.1380	*L. major*	83	1	83
LmjF15.1380	*L. major*	74	529	602
LmjF15.1400	*L. major*	83	213	295
LmjF15.1410	*L. major*	140	339	478
LmjF15.1420	*L. major*	68	141	208
LmjF15.1440	*L. major*	49	1	49
LmjF15.1450	*L. major*	59	166	224
LmjF15.1480	*L. major*	54	39	92
LmjF15.1490	*L. major*	48	1	48
LmjF15.1490	*L. major*	69	113	181
LmjF15.1490	*L. major*	264	381	644
LmjF15.1500	*L. major*	41	138	178
LmjF15.1510	*L. major*	41	1	41
LmjF15.1520	*L. major*	53	57	109
LmjF15.1540	*L. major*	49	368	416
LmjF15.1545	*L. major*	54	1	54
LmjF15.1545	*L. major*	74	149	222
LmjF15.1545	*L. major*	74	149	222
LmjF15.1560	*L. major*	40	153	192
LmjF15.1560	*L. major*	49	505	553
LmjF15.1560	*L. major*	56	870	925
LmjF15.1560	*L. major*	47	1210	1256
LmjF15.1570	*L. major*	119	305	423
LmjF15.1570	*L. major*	58	486	543
LmjF16.0005	*L. major*	43	184	226
LmjF16.0050	*L. major*	61	181	241
LmjF16.0050	*L. major*	48	536	583
LmjF16.0060	*L. major*	76	884	959
LmjF16.0070	*L. major*	110	1	110
LmjF16.0080	*L. major*	75	1	75
LmjF16.0080	*L. major*	66	381	446
LmjF16.0080	*L. major*	84	508	591
LmjF16.0080	*L. major*	116	621	736
LmjF16.0100	*L. major*	63	167	229
LmjF16.0120	*L. major*	51	1	51
LmjF16.0120	*L. major*	49	417	465
LmjF16.0130	*L. major*	126	60	185

LmjF16.0140	*L. major*	40	1	40
LmjF16.0140	*L. major*	53	116	168
LmjF16.0140	*L. major*	53	116	168
LmjF16.0150	*L. major*	150	85	234
LmjF16.0160	*L. major*	41	1	41
LmjF16.0160	*L. major*	58	248	305
LmjF16.0160	*L. major*	58	248	305
LmjF16.0170	*L. major*	92	144	235
LmjF16.0170	*L. major*	57	301	357
LmjF16.0170	*L. major*	112	1156	1267
LmjF16.0170	*L. major*	71	1283	1353
LmjF16.0170	*L. major*	303	1513	1815
LmjF16.0170	*L. major*	51	1924	1974
LmjF16.0170	*L. major*	63	2395	2457
LmjF16.0170	*L. major*	43	2566	2608
LmjF16.0170	*L. major*	113	2729	2841
LmjF16.0170	*L. major*	48	2904	2951
LmjF16.0170	*L. major*	62	3041	3102
LmjF16.0170	*L. major*	73	3333	3405
LmjF16.0170	*L. major*	81	3441	3521
LmjF16.0170	*L. major*	68	4267	4334
LmjF16.0170	*L. major*	47	4389	4435
LmjF16.0180	*L. major*	158	1	158
LmjF16.0190	*L. major*	82	1	82
LmjF16.0190	*L. major*	119	247	365
LmjF16.0210	*L. major*	55	110	164
LmjF16.0210	*L. major*	45	339	383
LmjF16.0220	*L. major*	51	81	131
LmjF16.0240	*L. major*	65	1	65
LmjF16.0240	*L. major*	53	115	167
LmjF16.0240	*L. major*	43	662	704
LmjF16.0260	*L. major*	64	68	131
LmjF16.0270	*L. major*	57	47	103
LmjF16.0270	*L. major*	96	149	244
LmjF16.0270	*L. major*	40	302	341
LmjF16.0270	*L. major*	65	399	463
LmjF16.0270	*L. major*	41	962	1002
LmjF16.0280	*L. major*	53	451	503
LmjF16.0280	*L. major*	86	785	870
LmjF16.0280	*L. major*	47	1152	1198
LmjF16.0280	*L. major*	53	2132	2184
LmjF16.0280	*L. major*	63	2777	2839
LmjF16.0290	*L. major*	70	87	156
LmjF16.0300	*L. major*	140	258	397
LmjF16.0300	*L. major*	101	404	504
LmjF16.0300	*L. major*	101	404	504
LmjF16.0310	*L. major*	62	87	148

LmjF16.0310	*L. major*	64	186	249
LmjF16.0320	*L. major*	739	380	1118
LmjF16.0330	*L. major*	41	23	63
LmjF16.0330	*L. major*	50	142	191
LmjF16.0330	*L. major*	50	142	191
LmjF16.0350	*L. major*	50	1	50
LmjF16.0360	*L. major*	128	123	250
LmjF16.0360	*L. major*	128	402	529
LmjF16.0360	*L. major*	119	549	667
LmjF16.0370	*L. major*	137	62	198
LmjF16.0380	*L. major*	191	1	191
LmjF16.0380	*L. major*	55	196	250
LmjF16.0380	*L. major*	60	262	321
LmjF16.0380	*L. major*	89	506	594
LmjF16.0390	*L. major*	59	42	100
LmjF16.0390	*L. major*	51	251	301
LmjF16.0390	*L. major*	333	653	985
LmjF16.0400	*L. major*	44	107	150
LmjF16.0410	*L. major*	237	339	575
LmjF16.0420	*L. major*	65	1	65
LmjF16.0430	*L. major*	187	1	187
LmjF16.0440	*L. major*	50	746	795
LmjF16.0480	*L. major*	50	746	795
LmjF16.0510	*L. major*	40	39	78
LmjF16.0510	*L. major*	87	146	232
LmjF16.0510	*L. major*	96	295	390
LmjF16.0510	*L. major*	362	463	824
LmjF16.0520	*L. major*	43	286	328
LmjF16.0560	*L. major*	107	144	250
LmjF16.0560	*L. major*	90	374	463
LmjF16.0560	*L. major*	97	514	610
LmjF16.0570	*L. major*	49	1	49
LmjF16.0575	*L. major*	49	1	49
LmjF16.0580	*L. major*	55	158	212
LmjF16.0600	*L. major*	49	1	49
LmjF16.0610	*L. major*	49	1	49
LmjF16.0620	*L. major*	225	55	279
LmjF16.0620	*L. major*	76	433	508
LmjF16.0620	*L. major*	144	516	659
LmjF16.0620	*L. major*	115	863	977
LmjF16.0620	*L. major*	50	1053	1102
LmjF16.0630	*L. major*	73	59	131
LmjF16.0630	*L. major*	53	240	292
LmjF16.0630	*L. major*	81	414	494
LmjF16.0630	*L. major*	57	518	574
LmjF16.0640	*L. major*	42	1	42
LmjF16.0640	*L. major*	137	146	282

LmjF16.0640	*L. major*	52	515	566
LmjF16.0660	*L. major*	48	65	112
LmjF16.0660	*L. major*	452	115	566
LmjF16.0670	*L. major*	206	42	247
LmjF16.0670	*L. major*	85	538	622
LmjF16.0680	*L. major*	94	1	94
LmjF16.0680	*L. major*	44	227	270
LmjF16.0690	*L. major*	63	489	551
LmjF16.0700	*L. major*	648	111	758
LmjF16.0700	*L. major*	73	777	849
LmjF16.0710	*L. major*	105	1	105
LmjF16.0710	*L. major*	348	134	481
LmjF16.0710	*L. major*	107	515	621
LmjF16.0710	*L. major*	252	633	884
LmjF16.0710	*L. major*	64	908	971
LmjF16.0720	*L. major*	57	73	129
LmjF16.0720	*L. major*	67	206	272
LmjF16.0730	*L. major*	88	46	133
LmjF16.0730	*L. major*	56	193	248
LmjF16.0730	*L. major*	43	595	637
LmjF16.0730	*L. major*	61	733	793
LmjF16.0730	*L. major*	50	864	913
LmjF16.0730	*L. major*	79	917	995
LmjF16.0730	*L. major*	52	1035	1086
LmjF16.0730	*L. major*	40	1169	1208
LmjF16.0730	*L. major*	108	1248	1355
LmjF16.0730	*L. major*	78	1511	1588
LmjF16.0730	*L. major*	57	1722	1778
LmjF16.0740	*L. major*	120	1	120
LmjF16.0740	*L. major*	111	135	245
LmjF16.0740	*L. major*	118	278	395
LmjF16.0740	*L. major*	90	465	554
LmjF16.0740	*L. major*	43	560	602
LmjF16.0740	*L. major*	67	673	739
LmjF16.0750	*L. major*	80	150	229
LmjF16.0750	*L. major*	143	461	603
LmjF16.0750	*L. major*	143	461	603
LmjF16.0770	*L. major*	63	1	63
LmjF16.0770	*L. major*	345	93	437
LmjF16.0780	*L. major*	40	48	87
LmjF16.0780	*L. major*	72	125	196
LmjF16.0780	*L. major*	73	477	549
LmjF16.0780	*L. major*	44	717	760
LmjF16.0780	*L. major*	52	885	936
LmjF16.0780	*L. major*	122	1025	1146
LmjF16.0780	*L. major*	72	1262	1333
LmjF16.0780	*L. major*	83	1429	1511

LmjF16.0780	*L. major*	76	1655	1730
LmjF16.0780	*L. major*	53	1768	1820
LmjF16.0780	*L. major*	70	1909	1978
LmjF16.0780	*L. major*	72	2121	2192
LmjF16.0780	*L. major*	54	2394	2447
LmjF16.0780	*L. major*	59	2491	2549
LmjF16.0780	*L. major*	46	2592	2637
LmjF16.0780	*L. major*	60	2716	2775
LmjF16.0780	*L. major*	43	2870	2912
LmjF16.0780	*L. major*	108	3026	3133
LmjF16.0780	*L. major*	53	3413	3465
LmjF16.0780	*L. major*	58	3765	3822
LmjF16.0780	*L. major*	48	4415	4462
LmjF16.0780	*L. major*	92	5262	5353
LmjF16.0780	*L. major*	176	5377	5552
LmjF16.0800	*L. major*	96	273	368
LmjF16.0810	*L. major*	40	114	153
LmjF16.0810	*L. major*	74	177	250
LmjF16.0820	*L. major*	239	173	411
LmjF16.0820	*L. major*	51	558	608
LmjF16.0820	*L. major*	57	621	677
LmjF16.0830	*L. major*	147	9	155
LmjF16.0840	*L. major*	48	237	284
LmjF16.0840	*L. major*	44	325	368
LmjF16.0840	*L. major*	44	325	368
LmjF16.0850	*L. major*	56	330	385
LmjF16.0850	*L. major*	53	426	478
LmjF16.0850	*L. major*	53	426	478
LmjF16.0860	*L. major*	69	50	118
LmjF16.0860	*L. major*	87	148	234
LmjF16.0860	*L. major*	87	148	234
LmjF16.0870	*L. major*	142	1	142
LmjF16.0870	*L. major*	40	158	197
LmjF16.0870	*L. major*	48	364	411
LmjF16.0880	*L. major*	64	60	123
LmjF16.0880	*L. major*	60	176	235
LmjF16.0880	*L. major*	60	176	235
LmjF16.0890	*L. major*	83	95	177
LmjF16.0890	*L. major*	55	291	345
LmjF16.0890	*L. major*	46	457	502
LmjF16.0890	*L. major*	129	534	662
LmjF16.0895	*L. major*	91	41	131
LmjF16.0895	*L. major*	65	163	227
LmjF16.0895	*L. major*	65	163	227
LmjF16.0900	*L. major*	41	156	196
LmjF16.0900	*L. major*	83	198	280
LmjF16.0900	*L. major*	46	552	597

LmjF16.0900	*L. major*	45	1092	1136
LmjF16.0900	*L. major*	42	1138	1179
LmjF16.0900	*L. major*	46	1506	1551
LmjF16.0930	*L. major*	42	1	42
LmjF16.0930	*L. major*	91	130	220
LmjF16.0960	*L. major*	57	192	248
LmjF16.0960	*L. major*	81	267	347
LmjF16.0960	*L. major*	81	267	347
LmjF16.0970	*L. major*	97	166	262
LmjF16.0970	*L. major*	48	290	337
LmjF16.0970	*L. major*	105	600	704
LmjF16.0980	*L. major*	56	1	56
LmjF16.0980	*L. major*	642	66	707
LmjF16.0990	*L. major*	59	148	206
LmjF16.0990	*L. major*	63	297	359
LmjF16.1005	*L. major*	127	1	127
LmjF16.1005	*L. major*	88	229	316
LmjF16.1010	*L. major*	119	99	217
LmjF16.1010	*L. major*	44	523	566
LmjF16.1015	*L. major*	125	1	125
LmjF16.1015	*L. major*	64	682	745
LmjF16.1020	*L. major*	125	1	125
LmjF16.1030	*L. major*	125	1	125
LmjF16.1040	*L. major*	44	259	302
LmjF16.1050	*L. major*	58	92	149
LmjF16.1050	*L. major*	49	285	333
LmjF16.1050	*L. major*	51	498	548
LmjF16.1060	*L. major*	42	138	179
LmjF16.1060	*L. major*	56	598	653
LmjF16.1060	*L. major*	56	598	653
LmjF16.1065	*L. major*	77	77	153
LmjF16.1070	*L. major*	69	155	223
LmjF16.1070	*L. major*	49	442	490
LmjF16.1070	*L. major*	49	442	490
LmjF16.1080	*L. major*	44	1	44
LmjF16.1080	*L. major*	462	88	549
LmjF16.1080	*L. major*	72	569	640
LmjF16.1090	*L. major*	163	1	163
LmjF16.1090	*L. major*	71	228	298
LmjF16.1090	*L. major*	747	336	1082
LmjF16.1090	*L. major*	782	1085	1866
LmjF16.1090	*L. major*	221	1927	2147
LmjF16.1100	*L. major*	492	67	558
LmjF16.1110	*L. major*	123	1	123
LmjF16.1120	*L. major*	55	86	140
LmjF16.1140	*L. major*	162	216	377
LmjF16.1140	*L. major*	106	401	506

LmjF16.1140	*L. major*	121	509	629
LmjF16.1160	*L. major*	89	127	215
LmjF16.1160	*L. major*	65	271	335
LmjF16.1160	*L. major*	44	423	466
LmjF16.1180	*L. major*	183	140	322
LmjF16.1190	*L. major*	103	147	249
LmjF16.1190	*L. major*	55	430	484
LmjF16.1190	*L. major*	96	1051	1146
LmjF16.1190	*L. major*	212	1230	1441
LmjF16.1200	*L. major*	122	245	366
LmjF16.1210	*L. major*	160	63	222
LmjF16.1210	*L. major*	147	334	480
LmjF16.1210	*L. major*	51	862	912
LmjF16.1210	*L. major*	79	1157	1235
LmjF16.1220	*L. major*	79	1	79
LmjF16.1220	*L. major*	52	105	156
LmjF16.1220	*L. major*	551	252	802
LmjF16.1220	*L. major*	240	906	1145
LmjF16.1220	*L. major*	259	1303	1561
LmjF16.1220	*L. major*	41	1676	1716
LmjF16.1220	*L. major*	41	1809	1849
LmjF16.1220	*L. major*	51	2063	2113
LmjF16.1230	*L. major*	76	1	76
LmjF16.1230	*L. major*	59	424	482
LmjF16.1230	*L. major*	44	540	583
LmjF16.1230	*L. major*	59	1320	1378
LmjF16.1230	*L. major*	171	1643	1813
LmjF16.1240	*L. major*	120	65	184
LmjF16.1240	*L. major*	59	238	296
LmjF16.1240	*L. major*	274	372	645
LmjF16.1240	*L. major*	48	754	801
LmjF16.1240	*L. major*	115	903	1017
LmjF16.1240	*L. major*	52	1120	1171
LmjF16.1240	*L. major*	382	1203	1584
LmjF16.1240	*L. major*	48	1824	1871
LmjF16.1240	*L. major*	116	1993	2108
LmjF16.1250	*L. major*	47	52	98
LmjF16.1260	*L. major*	274	16	289
LmjF16.1260	*L. major*	63	671	733
LmjF16.1260	*L. major*	105	757	861
LmjF16.1260	*L. major*	53	977	1029
LmjF16.1260	*L. major*	86	1283	1368
LmjF16.1260	*L. major*	51	1418	1468
LmjF16.1260	*L. major*	44	1604	1647
LmjF16.1270	*L. major*	245	1	245
LmjF16.1270	*L. major*	182	282	463
LmjF16.1270	*L. major*	43	549	591

LmjF16.1270	*L. major*	150	599	748
LmjF16.1270	*L. major*	44	916	959
LmjF16.1270	*L. major*	460	1065	1524
LmjF16.1270	*L. major*	85	1535	1619
LmjF16.1270	*L. major*	67	1725	1791
LmjF16.1270	*L. major*	76	1899	1974
LmjF16.1270	*L. major*	74	2075	2148
LmjF16.1290	*L. major*	146	1	146
LmjF16.1290	*L. major*	44	372	415
LmjF16.1290	*L. major*	42	671	712
LmjF16.1300	*L. major*	192	49	240
LmjF16.1300	*L. major*	91	279	369
LmjF16.1300	*L. major*	151	460	610
LmjF16.1300	*L. major*	41	795	835
LmjF16.1300	*L. major*	75	859	933
LmjF16.1330	*L. major*	641	1	641
LmjF16.1330	*L. major*	69	1273	1341
LmjF16.1340	*L. major*	135	25	159
LmjF16.1340	*L. major*	72	175	246
LmjF16.1340	*L. major*	43	390	432
LmjF16.1340	*L. major*	64	625	688
LmjF16.1340	*L. major*	214	747	960
LmjF16.1340	*L. major*	41	1047	1087
LmjF16.1340	*L. major*	53	1441	1493
LmjF16.1340	*L. major*	55	1619	1673
LmjF16.1340	*L. major*	45	1808	1852
LmjF16.1340	*L. major*	48	2230	2277
LmjF16.1340	*L. major*	46	2312	2357
LmjF16.1340	*L. major*	136	2499	2634
LmjF16.1340	*L. major*	93	3037	3129
LmjF16.1340	*L. major*	54	3308	3361
LmjF16.1340	*L. major*	56	3413	3468
LmjF16.1340	*L. major*	65	3511	3575
LmjF16.1340	*L. major*	158	3601	3758
LmjF16.1350	*L. major*	48	218	265
LmjF16.1350	*L. major*	51	1110	1160
LmjF16.1350	*L. major*	165	1385	1549
LmjF16.1390	*L. major*	47	182	228
LmjF16.1410	*L. major*	77	63	139
LmjF16.1410	*L. major*	162	323	484
LmjF16.1410	*L. major*	162	323	484
LmjF16.1420	*L. major*	68	89	156
LmjF16.1425	*L. major*	120	259	378
LmjF16.1425	*L. major*	42	487	528
LmjF16.1425	*L. major*	42	487	528
LmjF16.1427	*L. major*	120	259	378
LmjF16.1427	*L. major*	42	487	528

LmjF16.1427	*L. major*	42	487	528
LmjF16.1430	*L. major*	120	259	378
LmjF16.1430	*L. major*	42	487	528
LmjF16.1430	*L. major*	42	487	528
LmjF16.1440	*L. major*	88	1	88
LmjF16.1440	*L. major*	42	95	136
LmjF16.1440	*L. major*	55	354	408
LmjF16.1440	*L. major*	78	604	681
LmjF16.1440	*L. major*	72	726	797
LmjF16.1440	*L. major*	97	1010	1106
LmjF16.1440	*L. major*	59	1259	1317
LmjF16.1450	*L. major*	84	1	84
LmjF16.1450	*L. major*	62	124	185
LmjF16.1450	*L. major*	55	187	241
LmjF16.1460	*L. major*	1656	448	2103
LmjF16.1460	*L. major*	50	2208	2257
LmjF16.1470	*L. major*	216	448	663
LmjF16.1470	*L. major*	43	765	807
LmjF16.1480	*L. major*	43	1	43
LmjF16.1480	*L. major*	93	271	363
LmjF16.1480	*L. major*	93	271	363
LmjF16.1490	*L. major*	56	268	323
LmjF16.1500	*L. major*	48	151	198
LmjF16.1500	*L. major*	68	344	411
LmjF16.1500	*L. major*	41	528	568
LmjF16.1520	*L. major*	45	40	84
LmjF16.1520	*L. major*	67	253	319
LmjF16.1520	*L. major*	139	699	837
LmjF16.1520	*L. major*	143	995	1137
LmjF16.1540	*L. major*	113	1	113
LmjF16.1540	*L. major*	101	115	215
LmjF16.1550	*L. major*	445	713	1157
LmjF16.1560	*L. major*	53	285	337
LmjF16.1560	*L. major*	77	615	691
LmjF16.1560	*L. major*	77	615	691
LmjF16.1570	*L. major*	136	141	276
LmjF16.1580	*L. major*	75	217	291
LmjF16.1580	*L. major*	138	487	624
LmjF16.1580	*L. major*	104	648	751
LmjF16.1580	*L. major*	42	946	987
LmjF16.1590	*L. major*	133	1	133
LmjF16.1600	*L. major*	58	133	190
LmjF16.1600	*L. major*	113	281	393
LmjF16.1620	*L. major*	68	187	254
LmjF16.1620	*L. major*	71	291	361
LmjF16.1620	*L. major*	71	291	361
LmjF16.1630	*L. major*	50	72	121

LmjF16.1630	*L. major*	87	153	239
LmjF16.1630	*L. major*	87	153	239
LmjF16.1660	*L. major*	42	799	840
LmjF16.1660	*L. major*	193	1580	1772
LmjF16.1660	*L. major*	87	3830	3916
LmjF16.1660	*L. major*	271	3927	4197
LmjF17.0010	*L. major*	43	78	120
LmjF17.0010	*L. major*	110	517	626
LmjF17.0010	*L. major*	100	675	774
LmjF17.0030	*L. major*	51	1	51
LmjF17.0030	*L. major*	67	122	188
LmjF17.0030	*L. major*	67	122	188
LmjF17.0040	*L. major*	49	214	262
LmjF17.0050	*L. major*	51	150	200
LmjF17.0060	*L. major*	72	482	553
LmjF17.0100	*L. major*	57	1	57
LmjF17.0100	*L. major*	49	363	411
LmjF17.0100	*L. major*	48	465	512
LmjF17.0100	*L. major*	81	516	596
LmjF17.0110	*L. major*	131	1	131
LmjF17.0110	*L. major*	367	160	526
LmjF17.0120	*L. major*	40	1	40
LmjF17.0130	*L. major*	183	659	841
LmjF17.0150	*L. major*	43	313	355
LmjF17.0150	*L. major*	54	391	444
LmjF17.0150	*L. major*	54	391	444
LmjF17.0160	*L. major*	42	46	87
LmjF17.0160	*L. major*	120	156	275
LmjF17.0160	*L. major*	106	312	417
LmjF17.0160	*L. major*	128	453	580
LmjF17.0160	*L. major*	279	628	906
LmjF17.0160	*L. major*	52	983	1034
LmjF17.0170	*L. major*	206	1	206
LmjF17.0180	*L. major*	52	1274	1325
LmjF17.0190	*L. major*	122	1293	1414
LmjF17.0200	*L. major*	115	1244	1358
LmjF17.0230	*L. major*	44	776	819
LmjF17.0240	*L. major*	131	1	131
LmjF17.0240	*L. major*	206	190	395
LmjF17.0240	*L. major*	91	876	966
LmjF17.0240	*L. major*	126	1017	1142
LmjF17.0260	*L. major*	178	28	205
LmjF17.0260	*L. major*	546	240	785
LmjF17.0270	*L. major*	42	232	273
LmjF17.0280	*L. major*	75	1	75
LmjF17.0290	*L. major*	217	82	298
LmjF17.0290	*L. major*	55	587	641

LmjF17.0300	*L. major*	41	1	41
LmjF17.0300	*L. major*	63	141	203
LmjF17.0300	*L. major*	63	141	203
LmjF17.0310	*L. major*	145	1	145
LmjF17.0310	*L. major*	71	215	285
LmjF17.0310	*L. major*	341	364	704
LmjF17.0310	*L. major*	615	733	1347
LmjF17.0320	*L. major*	82	1	82
LmjF17.0320	*L. major*	171	159	329
LmjF17.0330	*L. major*	47	237	283
LmjF17.0330	*L. major*	44	666	709
LmjF17.0330	*L. major*	41	1253	1293
LmjF17.0330	*L. major*	47	1337	1383
LmjF17.0350	*L. major*	232	1	232
LmjF17.0370	*L. major*	87	1	87
LmjF17.0370	*L. major*	118	184	301
LmjF17.0370	*L. major*	323	314	636
LmjF17.0380	*L. major*	48	22	69
LmjF17.0380	*L. major*	147	87	233
LmjF17.0390	*L. major*	70	1	70
LmjF17.0390	*L. major*	55	97	151
LmjF17.0390	*L. major*	50	265	314
LmjF17.0390	*L. major*	84	587	670
LmjF17.0390	*L. major*	109	743	851
LmjF17.0390	*L. major*	50	1119	1168
LmjF17.0400	*L. major*	209	40	248
LmjF17.0400	*L. major*	69	307	375
LmjF17.0400	*L. major*	80	481	560
LmjF17.0410	*L. major*	50	54	103
LmjF17.0410	*L. major*	119	421	539
LmjF17.0410	*L. major*	405	658	1062
LmjF17.0420	*L. major*	57	227	283
LmjF17.0420	*L. major*	40	296	335
LmjF17.0420	*L. major*	149	354	502
LmjF17.0430	*L. major*	919	1	919
LmjF17.0430	*L. major*	143	980	1122
LmjF17.0430	*L. major*	48	1241	1288
LmjF17.0440	*L. major*	82	112	193
LmjF17.0440	*L. major*	79	216	294
LmjF17.0440	*L. major*	55	345	399
LmjF17.0440	*L. major*	274	455	728
LmjF17.0440	*L. major*	564	756	1319
LmjF17.0440	*L. major*	223	1373	1595
LmjF17.0440	*L. major*	238	1697	1934
LmjF17.0450	*L. major*	161	93	253
LmjF17.0450	*L. major*	64	354	417
LmjF17.0450	*L. major*	66	461	526

LmjF17.0450	*L. major*	68	586	653
LmjF17.0450	*L. major*	198	727	924
LmjF17.0450	*L. major*	43	1187	1229
LmjF17.0450	*L. major*	65	1312	1376
LmjF17.0470	*L. major*	64	137	200
LmjF17.0470	*L. major*	86	306	391
LmjF17.0470	*L. major*	48	406	453
LmjF17.0470	*L. major*	66	485	550
LmjF17.0470	*L. major*	77	828	904
LmjF17.0470	*L. major*	106	1201	1306
LmjF17.0470	*L. major*	50	1498	1547
LmjF17.0470	*L. major*	44	1845	1888
LmjF17.0470	*L. major*	71	1973	2043
LmjF17.0470	*L. major*	49	2084	2132
LmjF17.0470	*L. major*	49	2152	2200
LmjF17.0470	*L. major*	43	2321	2363
LmjF17.0470	*L. major*	46	2423	2468
LmjF17.0470	*L. major*	63	2509	2571
LmjF17.0470	*L. major*	97	2809	2905
LmjF17.0470	*L. major*	74	3044	3117
LmjF17.0480	*L. major*	42	49	90
LmjF17.0480	*L. major*	112	123	234
LmjF17.0490	*L. major*	259	1	259
LmjF17.0490	*L. major*	100	331	430
LmjF17.0490	*L. major*	61	462	522
LmjF17.0490	*L. major*	52	525	576
LmjF17.0490	*L. major*	41	743	783
LmjF17.0500	*L. major*	362	1	362
LmjF17.0500	*L. major*	116	399	514
LmjF17.0500	*L. major*	91	553	643
LmjF17.0500	*L. major*	64	678	741
LmjF17.0500	*L. major*	112	800	911
LmjF17.0500	*L. major*	221	920	1140
LmjF17.0500	*L. major*	71	1212	1282
LmjF17.0500	*L. major*	126	1300	1425
LmjF17.0510	*L. major*	477	1	477
LmjF17.0510	*L. major*	240	522	761
LmjF17.0510	*L. major*	56	763	818
LmjF17.0510	*L. major*	213	820	1032
LmjF17.0520	*L. major*	70	1	70
LmjF17.0520	*L. major*	354	78	431
LmjF17.0520	*L. major*	121	457	577
LmjF17.0520	*L. major*	240	606	845
LmjF17.0530	*L. major*	149	1	149
LmjF17.0530	*L. major*	46	175	220
LmjF17.0530	*L. major*	432	228	659
LmjF17.0530	*L. major*	263	661	923

LmjF17.0530	*L. major*	88	963	1050
LmjF17.0540	*L. major*	50	1	50
LmjF17.0540	*L. major*	59	59	117
LmjF17.0540	*L. major*	182	142	323
LmjF17.0540	*L. major*	49	610	658
LmjF17.0540	*L. major*	60	910	969
LmjF17.0540	*L. major*	271	1000	1270
LmjF17.0550	*L. major*	163	55	217
LmjF17.0560	*L. major*	60	62	121
LmjF17.0570	*L. major*	184	15	198
LmjF17.0570	*L. major*	711	207	917
LmjF17.0570	*L. major*	47	948	994
LmjF17.0570	*L. major*	126	1022	1147
LmjF17.0580	*L. major*	58	189	246
LmjF17.0590	*L. major*	40	123	162
LmjF17.0590	*L. major*	43	193	235
LmjF17.0590	*L. major*	91	274	364
LmjF17.0590	*L. major*	50	388	437
LmjF17.0590	*L. major*	73	575	647
LmjF17.0590	*L. major*	259	677	935
LmjF17.0590	*L. major*	64	983	1046
LmjF17.0590	*L. major*	71	1080	1150
LmjF17.0590	*L. major*	165	1175	1339
LmjF17.0590	*L. major*	154	1341	1494
LmjF17.0590	*L. major*	67	1572	1638
LmjF17.0600	*L. major*	40	433	472
LmjF17.0600	*L. major*	40	566	605
LmjF17.0600	*L. major*	40	1012	1051
LmjF17.0610	*L. major*	238	261	498
LmjF17.0650	*L. major*	102	1	102
LmjF17.0650	*L. major*	136	211	346
LmjF17.0650	*L. major*	89	550	638
LmjF17.0650	*L. major*	60	659	718
LmjF17.0650	*L. major*	92	822	913
LmjF17.0650	*L. major*	91	1044	1134
LmjF17.0660	*L. major*	42	182	223
LmjF17.0660	*L. major*	143	368	510
LmjF17.0660	*L. major*	62	525	586
LmjF17.0660	*L. major*	245	668	912
LmjF17.0670	*L. major*	89	1	89
LmjF17.0670	*L. major*	201	105	305
LmjF17.0670	*L. major*	250	313	562
LmjF17.0670	*L. major*	42	598	639
LmjF17.0670	*L. major*	269	882	1150
LmjF17.0670	*L. major*	47	1274	1320
LmjF17.0670	*L. major*	51	1341	1391
LmjF17.0670	*L. major*	41	1458	1498

LmjF17.0670	*L. major*	173	1509	1681
LmjF17.0680	*L. major*	93	352	444
LmjF17.0690	*L. major*	66	104	169
LmjF17.0690	*L. major*	324	181	504
LmjF17.0700	*L. major*	66	177	242
LmjF17.0700	*L. major*	74	408	481
LmjF17.0710	*L. major*	451	112	562
LmjF17.0710	*L. major*	105	574	678
LmjF17.0710	*L. major*	45	766	810
LmjF17.0720	*L. major*	104	161	264
LmjF17.0720	*L. major*	59	328	386
LmjF17.0720	*L. major*	41	420	460
LmjF17.0720	*L. major*	144	525	668
LmjF17.0720	*L. major*	57	742	798
LmjF17.0730	*L. major*	126	80	205
LmjF17.0733	*L. major*	187	1	187
LmjF17.0733	*L. major*	40	231	270
LmjF17.0740	*L. major*	41	215	255
LmjF17.0750	*L. major*	179	1	179
LmjF17.0750	*L. major*	366	210	575
LmjF17.0760	*L. major*	43	281	323
LmjF17.0770	*L. major*	98	62	159
LmjF17.0770	*L. major*	82	200	281
LmjF17.0770	*L. major*	74	370	443
LmjF17.0790	*L. major*	44	1	44
LmjF17.0790	*L. major*	213	277	489
LmjF17.0800	*L. major*	68	197	264
LmjF17.0800	*L. major*	95	318	412
LmjF17.0800	*L. major*	75	494	568
LmjF17.0800	*L. major*	337	602	938
LmjF17.0810	*L. major*	65	56	120
LmjF17.0810	*L. major*	48	252	299
LmjF17.0815	*L. major*	207	1	207
LmjF17.0820	*L. major*	50	1	50
LmjF17.0820	*L. major*	102	55	156
LmjF17.0820	*L. major*	56	160	215
LmjF17.0820	*L. major*	87	240	326
LmjF17.0820	*L. major*	192	359	550
LmjF17.0830	*L. major*	46	1	46
LmjF17.0830	*L. major*	184	436	619
LmjF17.0840	*L. major*	118	1	118
LmjF17.0840	*L. major*	65	176	240
LmjF17.0840	*L. major*	47	360	406
LmjF17.0850	*L. major*	77	1	77
LmjF17.0860	*L. major*	129	19	147
LmjF17.0900	*L. major*	50	1	50
LmjF17.0900	*L. major*	151	452	602

LmjF17.0920	*L. major*	116	123	238
LmjF17.0920	*L. major*	48	366	413
LmjF17.0920	*L. major*	46	1872	1917
LmjF17.0930	*L. major*	116	76	191
LmjF17.0930	*L. major*	255	225	479
LmjF17.0930	*L. major*	234	498	731
LmjF17.0930	*L. major*	260	1041	1300
LmjF17.0940	*L. major*	41	34	74
LmjF17.0940	*L. major*	54	150	203
LmjF17.0940	*L. major*	76	247	322
LmjF17.0940	*L. major*	71	430	500
LmjF17.0940	*L. major*	105	510	614
LmjF17.0940	*L. major*	153	636	788
LmjF17.0940	*L. major*	51	943	993
LmjF17.0950	*L. major*	66	1	66
LmjF17.0950	*L. major*	363	89	451
LmjF17.0950	*L. major*	216	492	707
LmjF17.0950	*L. major*	76	712	787
LmjF17.0950	*L. major*	118	838	955
LmjF17.0950	*L. major*	53	1293	1345
LmjF17.0950	*L. major*	86	1369	1454
LmjF17.0950	*L. major*	49	1501	1549
LmjF17.0960	*L. major*	44	1	44
LmjF17.0960	*L. major*	316	57	372
LmjF17.0960	*L. major*	419	378	796
LmjF17.0960	*L. major*	71	807	877
LmjF17.0960	*L. major*	59	931	989
LmjF17.0960	*L. major*	392	1187	1578
LmjF17.0980	*L. major*	47	155	201
LmjF17.0980	*L. major*	56	633	688
LmjF17.0980	*L. major*	56	633	688
LmjF17.0990	*L. major*	65	51	115
LmjF17.0990	*L. major*	98	150	247
LmjF17.0990	*L. major*	57	482	538
LmjF17.0990	*L. major*	340	552	891
LmjF17.0990	*L. major*	270	936	1205
LmjF17.0990	*L. major*	90	1225	1314
LmjF17.0990	*L. major*	103	1410	1512
LmjF17.0990	*L. major*	122	1708	1829
LmjF17.0990	*L. major*	106	1926	2031
LmjF17.0990	*L. major*	57	2330	2386
LmjF17.0990	*L. major*	96	2588	2683
LmjF17.0990	*L. major*	60	2716	2775
LmjF17.0990	*L. major*	50	2881	2930
LmjF17.0990	*L. major*	238	3125	3362
LmjF17.0990	*L. major*	239	3369	3607
LmjF17.0990	*L. major*	150	3625	3774

LmjF17.0990	*L. major*	45	3985	4029
LmjF17.0990	*L. major*	43	4053	4095
LmjF17.0990	*L. major*	41	4129	4169
LmjF17.0990	*L. major*	84	4209	4292
LmjF17.0990	*L. major*	166	4610	4775
LmjF17.0990	*L. major*	138	4910	5047
LmjF17.0990	*L. major*	177	5119	5295
LmjF17.0990	*L. major*	61	5335	5395
LmjF17.0990	*L. major*	112	5432	5543
LmjF17.0990	*L. major*	50	5590	5639
LmjF17.0990	*L. major*	51	5698	5748
LmjF17.0990	*L. major*	73	5861	5933
LmjF17.0990	*L. major*	65	5950	6014
LmjF17.0990	*L. major*	167	6189	6355
LmjF17.0990	*L. major*	94	6358	6451
LmjF17.1000	*L. major*	77	1	77
LmjF17.1000	*L. major*	87	246	332
LmjF17.1000	*L. major*	63	501	563
LmjF17.1030	*L. major*	237	1	237
LmjF17.1030	*L. major*	47	247	293
LmjF17.1030	*L. major*	181	331	511
LmjF17.1030	*L. major*	50	773	822
LmjF17.1040	*L. major*	62	199	260
LmjF17.1050	*L. major*	42	1	42
LmjF17.1060	*L. major*	46	317	362
LmjF17.1060	*L. major*	152	416	567
LmjF17.1060	*L. major*	120	597	716
LmjF17.1070	*L. major*	163	1	163
LmjF17.1070	*L. major*	100	274	373
LmjF17.1080	*L. major*	137	1	137
LmjF17.1080	*L. major*	44	160	203
LmjF17.1080	*L. major*	120	297	416
LmjF17.1080	*L. major*	65	666	730
LmjF17.1090	*L. major*	454	1	454
LmjF17.1090	*L. major*	73	459	531
LmjF17.1090	*L. major*	51	621	671
LmjF17.1090	*L. major*	82	815	896
LmjF17.1090	*L. major*	63	935	997
LmjF17.1090	*L. major*	41	1122	1162
LmjF17.1090	*L. major*	58	1208	1265
LmjF17.1100	*L. major*	44	363	406
LmjF17.1110	*L. major*	41	229	269
LmjF17.1110	*L. major*	65	365	429
LmjF17.1110	*L. major*	208	1170	1377
LmjF17.1130	*L. major*	111	1	111
LmjF17.1130	*L. major*	139	168	306
LmjF17.1130	*L. major*	57	502	558

LmjF17.1130	*L. major*	83	650	732
LmjF17.1130	*L. major*	62	903	964
LmjF17.1130	*L. major*	54	1000	1053
LmjF17.1130	*L. major*	100	1115	1214
LmjF17.1140	*L. major*	1226	105	1330
LmjF17.1140	*L. major*	59	1340	1398
LmjF17.1150	*L. major*	41	182	222
LmjF17.1150	*L. major*	95	356	450
LmjF17.1150	*L. major*	60	506	565
LmjF17.1150	*L. major*	40	747	786
LmjF17.1150	*L. major*	104	831	934
LmjF17.1150	*L. major*	68	949	1016
LmjF17.1170	*L. major*	53	35	87
LmjF17.1170	*L. major*	105	138	242
LmjF17.1170	*L. major*	336	270	605
LmjF17.1180	*L. major*	43	1	43
LmjF17.1180	*L. major*	440	204	643
LmjF17.1180	*L. major*	219	660	878
LmjF17.1180	*L. major*	59	887	945
LmjF17.1190	*L. major*	46	1	46
LmjF17.1190	*L. major*	98	75	172
LmjF17.1190	*L. major*	78	214	291
LmjF17.1190	*L. major*	197	323	519
LmjF17.1190	*L. major*	122	571	692
LmjF17.1200	*L. major*	283	1	283
LmjF17.1200	*L. major*	534	300	833
LmjF17.1210	*L. major*	57	189	245
LmjF17.1210	*L. major*	163	292	454
LmjF17.1220	*L. major*	42	1	42
LmjF17.1230	*L. major*	41	183	223
LmjF17.1230	*L. major*	83	381	463
LmjF17.1230	*L. major*	182	661	842
LmjF17.1240	*L. major*	47	101	147
LmjF17.1240	*L. major*	69	277	345
LmjF17.1240	*L. major*	42	730	771
LmjF17.1250	*L. major*	49	422	470
LmjF17.1250	*L. major*	47	663	709
LmjF17.1250	*L. major*	53	957	1009
LmjF17.1260	*L. major*	115	210	324
LmjF17.1260	*L. major*	83	359	441
LmjF17.1260	*L. major*	83	359	441
LmjF17.1290	*L. major*	70	599	668
LmjF17.1310	*L. major*	92	115	206
LmjF17.1310	*L. major*	120	523	642
LmjF17.1315	*L. major*	40	91	130
LmjF17.1315	*L. major*	54	180	233
LmjF17.1315	*L. major*	72	290	361

LmjF17.1320	*L. major*	397	40	436
LmjF17.1350	*L. major*	46	38	83
LmjF17.1370	*L. major*	75	229	303
LmjF17.1370	*L. major*	373	493	865
LmjF17.1380	*L. major*	85	1	85
LmjF17.1380	*L. major*	80	172	251
LmjF17.1380	*L. major*	75	254	328
LmjF17.1380	*L. major*	40	638	677
LmjF17.1410	*L. major*	43	1	43
LmjF17.1410	*L. major*	73	425	497
LmjF17.1420	*L. major*	210	48	257
LmjF17.1430	*L. major*	58	1	58
LmjF17.1440	*L. major*	49	212	260
LmjF17.1440	*L. major*	60	559	618
LmjF17.1440	*L. major*	60	559	618
LmjF17.1450	*L. major*	69	72	140
LmjF17.1470	*L. major*	191	53	243
LmjF18.0010	*L. major*	60	682	741
LmjF18.0020	*L. major*	49	192	240
LmjF18.0030	*L. major*	54	85	138
LmjF18.0060	*L. major*	43	115	157
LmjF18.0070	*L. major*	52	109	160
LmjF18.0070	*L. major*	154	188	341
LmjF18.0070	*L. major*	49	365	413
LmjF18.0070	*L. major*	46	427	472
LmjF18.0070	*L. major*	89	497	585
LmjF18.0070	*L. major*	132	592	723
LmjF18.0080	*L. major*	41	403	443
LmjF18.0100	*L. major*	89	124	212
LmjF18.0130	*L. major*	45	357	401
LmjF18.0130	*L. major*	54	635	688
LmjF18.0130	*L. major*	54	635	688
LmjF18.0140	*L. major*	42	545	586
LmjF18.0140	*L. major*	51	696	746
LmjF18.0140	*L. major*	51	696	746
LmjF18.0170	*L. major*	141	207	347
LmjF18.0180	*L. major*	92	197	288
LmjF18.0190	*L. major*	52	195	246
LmjF18.0190	*L. major*	43	253	295
LmjF18.0190	*L. major*	43	253	295
LmjF18.0210	*L. major*	63	169	231
LmjF18.0220	*L. major*	329	81	409
LmjF18.0230	*L. major*	112	1	112
LmjF18.0240	*L. major*	48	183	230
LmjF18.0240	*L. major*	76	250	325
LmjF18.0240	*L. major*	76	250	325
LmjF18.0250	*L. major*	68	1	68

LmjF18.0250	*L. major*	46	97	142
LmjF18.0270	*L. major*	43	1	43
LmjF18.0280	*L. major*	45	1	45
LmjF18.0290	*L. major*	40	188	227
LmjF18.0300	*L. major*	124	133	256
LmjF18.0300	*L. major*	54	313	366
LmjF18.0330	*L. major*	100	75	174
LmjF18.0340	*L. major*	46	102	147
LmjF18.0340	*L. major*	64	289	352
LmjF18.0340	*L. major*	64	289	352
LmjF18.0350	*L. major*	41	1	41
LmjF18.0370	*L. major*	44	167	210
LmjF18.0380	*L. major*	71	307	377
LmjF18.0390	*L. major*	172	1	172
LmjF18.0390	*L. major*	71	198	268
LmjF18.0390	*L. major*	897	282	1178
LmjF18.0390	*L. major*	171	1216	1386
LmjF18.0390	*L. major*	176	1565	1740
LmjF18.0400	*L. major*	134	102	235
LmjF18.0410	*L. major*	131	70	200
LmjF18.0420	*L. major*	53	46	98
LmjF18.0430	*L. major*	147	77	223
LmjF18.0440	*L. major*	126	102	227
LmjF18.0460	*L. major*	46	108	153
LmjF18.0470	*L. major*	63	1	63
LmjF18.0470	*L. major*	211	123	333
LmjF18.0490	*L. major*	88	155	242
LmjF18.0500	*L. major*	121	1	121
LmjF18.0500	*L. major*	79	301	379
LmjF18.0520	*L. major*	46	1	46
LmjF18.0520	*L. major*	175	79	253
LmjF18.0520	*L. major*	358	275	632
LmjF18.0530	*L. major*	44	130	173
LmjF18.0530	*L. major*	137	386	522
LmjF18.0540	*L. major*	95	419	513
LmjF18.0550	*L. major*	105	221	325
LmjF18.0570	*L. major*	51	556	606
LmjF18.0570	*L. major*	50	638	687
LmjF18.0570	*L. major*	50	638	687
LmjF18.0590	*L. major*	168	1	168
LmjF18.0590	*L. major*	173	245	417
LmjF18.0600	*L. major*	323	1	323
LmjF18.0600	*L. major*	212	352	563
LmjF18.0630	*L. major*	495	1	495
LmjF18.0650	*L. major*	109	56	164
LmjF18.0650	*L. major*	46	195	240
LmjF18.0650	*L. major*	62	254	315

LmjF18.0650	*L. major*	54	363	416
LmjF18.0650	*L. major*	71	448	518
LmjF18.0650	*L. major*	98	611	708
LmjF18.0650	*L. major*	84	811	894
LmjF18.0650	*L. major*	58	996	1053
LmjF18.0650	*L. major*	77	1194	1270
LmjF18.0680	*L. major*	50	1	50
LmjF18.0690	*L. major*	192	1	192
LmjF18.0690	*L. major*	124	456	579
LmjF18.0690	*L. major*	69	591	659
LmjF18.0700	*L. major*	116	596	711
LmjF18.0710	*L. major*	99	104	202
LmjF18.0710	*L. major*	57	276	332
LmjF18.0710	*L. major*	45	373	417
LmjF18.0710	*L. major*	50	454	503
LmjF18.0710	*L. major*	117	540	656
LmjF18.0710	*L. major*	118	824	941
LmjF18.0710	*L. major*	43	1000	1042
LmjF18.0710	*L. major*	113	1178	1290
LmjF18.0710	*L. major*	266	1299	1564
LmjF18.0720	*L. major*	102	146	247
LmjF18.0720	*L. major*	128	376	503
LmjF18.0720	*L. major*	102	619	720
LmjF18.0730	*L. major*	111	1	111
LmjF18.0740	*L. major*	44	360	403
LmjF18.0750	*L. major*	212	62	273
LmjF18.0750	*L. major*	109	376	484
LmjF18.0750	*L. major*	48	518	565
LmjF18.0770	*L. major*	45	18	62
LmjF18.0770	*L. major*	71	67	137
LmjF18.0770	*L. major*	308	214	521
LmjF18.0770	*L. major*	157	632	788
LmjF18.0770	*L. major*	205	899	1103
LmjF18.0770	*L. major*	246	1141	1386
LmjF18.0770	*L. major*	107	1530	1636
LmjF18.0790	*L. major*	60	65	124
LmjF18.0790	*L. major*	99	128	226
LmjF18.0800	*L. major*	59	1	59
LmjF18.0800	*L. major*	51	71	121
LmjF18.0800	*L. major*	51	71	121
LmjF18.0820	*L. major*	59	29	87
LmjF18.0820	*L. major*	47	156	202
LmjF18.0820	*L. major*	64	445	508
LmjF18.0820	*L. major*	58	1825	1882
LmjF18.0840	*L. major*	229	113	341
LmjF18.0840	*L. major*	123	362	484
LmjF18.0840	*L. major*	68	531	598

LmjF18.0840	*L. major*	81	614	694
LmjF18.0840	*L. major*	84	735	818
LmjF18.0840	*L. major*	122	857	978
LmjF18.0840	*L. major*	238	1035	1272
LmjF18.0840	*L. major*	177	1394	1570
LmjF18.0840	*L. major*	219	1623	1841
LmjF18.0840	*L. major*	96	1930	2025
LmjF18.0850	*L. major*	236	1	236
LmjF18.0850	*L. major*	61	308	368
LmjF18.0850	*L. major*	97	408	504
LmjF18.0850	*L. major*	86	687	772
LmjF18.0850	*L. major*	334	848	1181
LmjF18.0850	*L. major*	41	1278	1318
LmjF18.0850	*L. major*	157	1517	1673
LmjF18.0850	*L. major*	161	1699	1859
LmjF18.0850	*L. major*	87	1904	1990
LmjF18.0850	*L. major*	82	2158	2239
LmjF18.0850	*L. major*	143	2274	2416
LmjF18.0850	*L. major*	116	2471	2586
LmjF18.0850	*L. major*	51	2674	2724
LmjF18.0850	*L. major*	93	2734	2826
LmjF18.0860	*L. major*	49	1	49
LmjF18.0860	*L. major*	84	100	183
LmjF18.0860	*L. major*	43	239	281
LmjF18.0860	*L. major*	60	329	388
LmjF18.0860	*L. major*	82	632	713
LmjF18.0860	*L. major*	123	785	907
LmjF18.0870	*L. major*	59	94	152
LmjF18.0870	*L. major*	41	195	235
LmjF18.0870	*L. major*	152	316	467
LmjF18.0880	*L. major*	44	79	122
LmjF18.0880	*L. major*	71	499	569
LmjF18.0880	*L. major*	177	657	833
LmjF18.0900	*L. major*	94	36	129
LmjF18.0900	*L. major*	41	517	557
LmjF18.0910	*L. major*	80	55	134
LmjF18.0910	*L. major*	57	168	224
LmjF18.0910	*L. major*	54	294	347
LmjF18.0910	*L. major*	211	896	1106
LmjF18.0920	*L. major*	62	1	62
LmjF18.0920	*L. major*	68	432	499
LmjF18.0920	*L. major*	77	528	604
LmjF18.0930	*L. major*	83	90	172
LmjF18.0930	*L. major*	293	246	538
LmjF18.0930	*L. major*	281	560	840
LmjF18.0940	*L. major*	48	144	191
LmjF18.0940	*L. major*	143	1138	1280

LmjF18.0950	*L. major*	201	1	201
LmjF18.0950	*L. major*	46	209	254
LmjF18.0950	*L. major*	62	303	364
LmjF18.0970	*L. major*	915	1	915
LmjF18.0970	*L. major*	115	1160	1274
LmjF18.0970	*L. major*	81	1384	1464
LmjF18.0970	*L. major*	113	1479	1591
LmjF18.0970	*L. major*	103	1601	1703
LmjF18.0970	*L. major*	85	1725	1809
LmjF18.0970	*L. major*	209	1845	2053
LmjF18.0980	*L. major*	191	1	191
LmjF18.1000	*L. major*	41	1	41
LmjF18.1020	*L. major*	51	1	51
LmjF18.1020	*L. major*	233	102	334
LmjF18.1020	*L. major*	69	345	413
LmjF18.1020	*L. major*	63	430	492
LmjF18.1020	*L. major*	54	511	564
LmjF18.1020	*L. major*	176	570	745
LmjF18.1020	*L. major*	378	840	1217
LmjF18.1020	*L. major*	102	1222	1323
LmjF18.1030	*L. major*	73	116	188
LmjF18.1040	*L. major*	43	668	710
LmjF18.1050	*L. major*	86	1	86
LmjF18.1050	*L. major*	54	423	476
LmjF18.1050	*L. major*	52	629	680
LmjF18.1050	*L. major*	49	863	911
LmjF18.1050	*L. major*	54	1132	1185
LmjF18.1050	*L. major*	43	1381	1423
LmjF18.1060	*L. major*	40	512	551
LmjF18.1090	*L. major*	78	174	251
LmjF18.1100	*L. major*	57	42	98
LmjF18.1110	*L. major*	46	160	205
LmjF18.1120	*L. major*	103	85	187
LmjF18.1140	*L. major*	920	26	945
LmjF18.1150	*L. major*	105	1	105
LmjF18.1150	*L. major*	44	157	200
LmjF18.1180	*L. major*	129	1	129
LmjF18.1180	*L. major*	188	165	352
LmjF18.1180	*L. major*	88	385	472
LmjF18.1180	*L. major*	49	499	547
LmjF18.1180	*L. major*	197	584	780
LmjF18.1180	*L. major*	476	817	1292
LmjF18.1190	*L. major*	73	35	107
LmjF18.1190	*L. major*	42	343	384
LmjF18.1190	*L. major*	53	408	460
LmjF18.1190	*L. major*	55	707	761
LmjF18.1190	*L. major*	46	935	980

LmjF18.1190	*L. major*	49	1211	1259
LmjF18.1200	*L. major*	182	42	223
LmjF18.1210	*L. major*	48	175	222
LmjF18.1210	*L. major*	47	664	710
LmjF18.1220	*L. major*	48	175	222
LmjF18.1220	*L. major*	47	664	710
LmjF18.1230	*L. major*	115	79	193
LmjF18.1230	*L. major*	74	479	552
LmjF18.1230	*L. major*	74	479	552
LmjF18.1240	*L. major*	100	1	100
LmjF18.1240	*L. major*	60	155	214
LmjF18.1240	*L. major*	102	269	370
LmjF18.1240	*L. major*	204	464	667
LmjF18.1240	*L. major*	82	678	759
LmjF18.1250	*L. major*	53	1	53
LmjF18.1250	*L. major*	92	124	215
LmjF18.1250	*L. major*	170	217	386
LmjF18.1250	*L. major*	98	419	516
LmjF18.1250	*L. major*	162	531	692
LmjF18.1250	*L. major*	54	743	796
LmjF18.1310	*L. major*	114	58	171
LmjF18.1310	*L. major*	93	235	327
LmjF18.1310	*L. major*	75	413	487
LmjF18.1310	*L. major*	123	601	723
LmjF18.1320	*L. major*	390	1	390
LmjF18.1320	*L. major*	62	550	611
LmjF18.1330	*L. major*	43	115	157
LmjF18.1340	*L. major*	173	1	173
LmjF18.1340	*L. major*	208	202	409
LmjF18.1340	*L. major*	78	561	638
LmjF18.1340	*L. major*	104	665	768
LmjF18.1350	*L. major*	85	1	85
LmjF18.1350	*L. major*	158	124	281
LmjF18.1350	*L. major*	148	440	587
LmjF18.1360	*L. major*	229	1	229
LmjF18.1360	*L. major*	55	240	294
LmjF18.1360	*L. major*	611	350	960
LmjF18.1370	*L. major*	63	512	574
LmjF18.1370	*L. major*	119	705	823
LmjF18.1380	*L. major*	73	282	354
LmjF18.1390	*L. major*	53	1	53
LmjF18.1400	*L. major*	51	1	51
LmjF18.1400	*L. major*	71	98	168
LmjF18.1410	*L. major*	563	68	630
LmjF18.1410	*L. major*	107	644	750
LmjF18.1410	*L. major*	111	801	911
LmjF18.1410	*L. major*	88	949	1036

LmjF18.1420	*L. major*	78	87	164
LmjF18.1420	*L. major*	358	533	890
LmjF18.1430	*L. major*	586	76	661
LmjF18.1430	*L. major*	404	716	1119
LmjF18.1450	*L. major*	772	1	772
LmjF18.1450	*L. major*	65	866	930
LmjF18.1450	*L. major*	119	1063	1181
LmjF18.1450	*L. major*	67	1277	1343
LmjF18.1450	*L. major*	108	1403	1510
LmjF18.1450	*L. major*	399	1566	1964
LmjF18.1460	*L. major*	258	1	258
LmjF18.1460	*L. major*	107	295	401
LmjF18.1500	*L. major*	83	14	96
LmjF18.1500	*L. major*	58	211	268
LmjF18.1500	*L. major*	65	379	443
LmjF18.1500	*L. major*	70	451	520
LmjF18.1500	*L. major*	127	615	741
LmjF18.1510	*L. major*	82	1	82
LmjF18.1510	*L. major*	72	903	974
LmjF18.1520	*L. major*	82	1	82
LmjF18.1520	*L. major*	72	903	974
LmjF18.1530	*L. major*	187	1	187
LmjF18.1530	*L. major*	52	299	350
LmjF18.1530	*L. major*	45	466	510
LmjF18.1530	*L. major*	113	605	717
LmjF18.1530	*L. major*	119	902	1020
LmjF18.1530	*L. major*	213	1030	1242
LmjF18.1550	*L. major*	65	214	278
LmjF18.1600	*L. major*	85	323	407
LmjF18.1600	*L. major*	220	577	796
LmjF18.1610	*L. major*	153	1	153
LmjF18.1610	*L. major*	46	609	654
LmjF18.1630	*L. major*	74	49	122
LmjF18.1630	*L. major*	40	217	256
LmjF18.1630	*L. major*	40	217	256
LmjF18.1650	*L. major*	44	76	119
LmjF18.1650	*L. major*	56	467	522
LmjF18.1650	*L. major*	56	467	522
LmjF18.1660	*L. major*	42	223	264
LmjF18.1670	*L. major*	93	1	93
LmjF18.1670	*L. major*	213	131	343
LmjF18.1670	*L. major*	80	399	478
LmjF18.1670	*L. major*	66	483	548
LmjF19.0010	*L. major*	200	1	200
LmjF19.0020	*L. major*	86	1	86
LmjF19.0020	*L. major*	154	103	256
LmjF19.0060	*L. major*	48	1	48

LmjF19.0070	*L. major*	152	50	201
LmjF19.0070	*L. major*	55	316	370
LmjF19.0080	*L. major*	375	58	432
LmjF19.0090	*L. major*	64	1	64
LmjF19.0090	*L. major*	63	145	207
LmjF19.0090	*L. major*	50	244	293
LmjF19.0100	*L. major*	63	1	63
LmjF19.0110	*L. major*	121	1	121
LmjF19.0120	*L. major*	185	14	198
LmjF19.0130	*L. major*	41	213	253
LmjF19.0140	*L. major*	213	349	561
LmjF19.0170	*L. major*	92	118	209
LmjF19.0170	*L. major*	40	486	525
LmjF19.0170	*L. major*	94	1398	1491
LmjF19.0180	*L. major*	118	290	407
LmjF19.0190	*L. major*	87	1	87
LmjF19.0190	*L. major*	46	118	163
LmjF19.0230	*L. major*	52	1	52
LmjF19.0230	*L. major*	43	204	246
LmjF19.0230	*L. major*	43	204	246
LmjF19.0240	*L. major*	110	1	110
LmjF19.0240	*L. major*	86	435	520
LmjF19.0240	*L. major*	54	618	671
LmjF19.0250	*L. major*	101	244	344
LmjF19.0250	*L. major*	74	657	730
LmjF19.0260	*L. major*	40	1	40
LmjF19.0260	*L. major*	110	262	371
LmjF19.0270	*L. major*	410	1	410
LmjF19.0270	*L. major*	323	496	818
LmjF19.0280	*L. major*	206	428	633
LmjF19.0290	*L. major*	48	39	86
LmjF19.0290	*L. major*	75	140	214
LmjF19.0290	*L. major*	46	224	269
LmjF19.0290	*L. major*	51	325	375
LmjF19.0290	*L. major*	45	477	521
LmjF19.0290	*L. major*	46	586	631
LmjF19.0290	*L. major*	85	664	748
LmjF19.0290	*L. major*	81	943	1023
LmjF19.0290	*L. major*	117	1025	1141
LmjF19.0290	*L. major*	81	1199	1279
LmjF19.0300	*L. major*	96	136	231
LmjF19.0310	*L. major*	40	99	138
LmjF19.0320	*L. major*	41	1	41
LmjF19.0320	*L. major*	70	180	249
LmjF19.0320	*L. major*	43	414	456
LmjF19.0340	*L. major*	109	81	189
LmjF19.0350	*L. major*	41	1	41

LmjF19.0350	*L. major*	110	52	161
LmjF19.0350	*L. major*	110	52	161
LmjF19.0360	*L. major*	143	65	207
LmjF19.0360	*L. major*	170	235	404
LmjF19.0360	*L. major*	553	566	1118
LmjF19.0360	*L. major*	215	1389	1603
LmjF19.0360	*L. major*	47	1709	1755
LmjF19.0360	*L. major*	69	1778	1846
LmjF19.0370	*L. major*	459	1	459
LmjF19.0370	*L. major*	170	513	682
LmjF19.0370	*L. major*	415	740	1154
LmjF19.0380	*L. major*	71	1	71
LmjF19.0380	*L. major*	157	96	252
LmjF19.0380	*L. major*	325	453	777
LmjF19.0380	*L. major*	64	961	1024
LmjF19.0380	*L. major*	66	1170	1235
LmjF19.0380	*L. major*	45	1295	1339
LmjF19.0380	*L. major*	71	1362	1432
LmjF19.0380	*L. major*	105	1470	1574
LmjF19.0380	*L. major*	58	1741	1798
LmjF19.0380	*L. major*	93	1803	1895
LmjF19.0390	*L. major*	54	1	54
LmjF19.0400	*L. major*	45	155	199
LmjF19.0410	*L. major*	42	1	42
LmjF19.0420	*L. major*	55	61	115
LmjF19.0430	*L. major*	220	1	220
LmjF19.0430	*L. major*	43	339	381
LmjF19.0430	*L. major*	44	684	727
LmjF19.0430	*L. major*	50	943	992
LmjF19.0430	*L. major*	40	1027	1066
LmjF19.0440	*L. major*	55	1	55
LmjF19.0440	*L. major*	51	300	350
LmjF19.0450	*L. major*	212	61	272
LmjF19.0450	*L. major*	348	287	634
LmjF19.0450	*L. major*	68	702	769
LmjF19.0450	*L. major*	60	817	876
LmjF19.0460	*L. major*	187	54	240
LmjF19.0470	*L. major*	130	68	197
LmjF19.0470	*L. major*	102	259	360
LmjF19.0490	*L. major*	83	1	83
LmjF19.0500	*L. major*	273	27	299
LmjF19.0510	*L. major*	138	12	149
LmjF19.0510	*L. major*	101	192	292
LmjF19.0510	*L. major*	265	314	578
LmjF19.0510	*L. major*	127	605	731
LmjF19.0510	*L. major*	129	816	944
LmjF19.0510	*L. major*	117	1028	1144

LmjF19.0510	*L. major*	160	1167	1326
LmjF19.0510	*L. major*	109	1495	1603
LmjF19.0520	*L. major*	78	1	78
LmjF19.0520	*L. major*	51	118	168
LmjF19.0520	*L. major*	57	268	324
LmjF19.0530	*L. major*	169	1	169
LmjF19.0530	*L. major*	42	459	500
LmjF19.0530	*L. major*	221	526	746
LmjF19.0550	*L. major*	67	64	130
LmjF19.0560	*L. major*	82	132	213
LmjF19.0560	*L. major*	136	268	403
LmjF19.0560	*L. major*	109	584	692
LmjF19.0560	*L. major*	93	1069	1161
LmjF19.0560	*L. major*	65	1201	1265
LmjF19.0570	*L. major*	42	1	42
LmjF19.0580	*L. major*	671	1	671
LmjF19.0580	*L. major*	57	758	814
LmjF19.0580	*L. major*	43	1050	1092
LmjF19.0580	*L. major*	58	1141	1198
LmjF19.0580	*L. major*	103	1295	1397
LmjF19.0580	*L. major*	67	1808	1874
LmjF19.0580	*L. major*	50	2076	2125
LmjF19.0580	*L. major*	112	2202	2313
LmjF19.0580	*L. major*	165	2320	2484
LmjF19.0580	*L. major*	99	2523	2621
LmjF19.0580	*L. major*	153	2636	2788
LmjF19.0590	*L. major*	99	1	99
LmjF19.0590	*L. major*	127	102	228
LmjF19.0590	*L. major*	197	439	635
LmjF19.0590	*L. major*	177	710	886
LmjF19.0590	*L. major*	172	946	1117
LmjF19.0600	*L. major*	340	1	340
LmjF19.0600	*L. major*	115	352	466
LmjF19.0600	*L. major*	81	511	591
LmjF19.0600	*L. major*	51	713	763
LmjF19.0620	*L. major*	74	58	131
LmjF19.0620	*L. major*	57	243	299
LmjF19.0620	*L. major*	52	428	479
LmjF19.0620	*L. major*	47	612	658
LmjF19.0630	*L. major*	64	1	64
LmjF19.0650	*L. major*	69	69	137
LmjF19.0650	*L. major*	50	899	948
LmjF19.0650	*L. major*	48	977	1024
LmjF19.0650	*L. major*	44	1205	1248
LmjF19.0650	*L. major*	48	1279	1326
LmjF19.0650	*L. major*	64	1410	1473
LmjF19.0650	*L. major*	261	1479	1739

LmjF19.0650	*L. major*	132	1805	1936
LmjF19.0660	*L. major*	124	1	124
LmjF19.0670	*L. major*	152	1	152
LmjF19.0670	*L. major*	178	184	361
LmjF19.0680	*L. major*	55	94	148
LmjF19.0680	*L. major*	48	201	248
LmjF19.0680	*L. major*	81	560	640
LmjF19.0680	*L. major*	212	709	920
LmjF19.0680	*L. major*	102	960	1061
LmjF19.0690	*L. major*	56	97	152
LmjF19.0690	*L. major*	48	211	258
LmjF19.0690	*L. major*	77	576	652
LmjF19.0690	*L. major*	221	691	911
LmjF19.0690	*L. major*	70	965	1034
LmjF19.0700	*L. major*	77	170	246
LmjF19.0700	*L. major*	321	515	835
LmjF19.0700	*L. major*	1332	890	2221
LmjF19.0720	*L. major*	154	94	247
LmjF19.0730	*L. major*	92	294	385
LmjF19.0740	*L. major*	109	125	233
LmjF19.0740	*L. major*	62	343	404
LmjF19.0750	*L. major*	64	36	99
LmjF19.0750	*L. major*	47	120	166
LmjF19.0750	*L. major*	53	466	518
LmjF19.0760	*L. major*	63	166	228
LmjF19.0760	*L. major*	41	331	371
LmjF19.0760	*L. major*	41	331	371
LmjF19.0770	*L. major*	45	142	186
LmjF19.0790	*L. major*	80	369	448
LmjF19.0790	*L. major*	151	590	740
LmjF19.0800	*L. major*	63	1	63
LmjF19.0800	*L. major*	50	310	359
LmjF19.0810	*L. major*	45	48	92
LmjF19.0810	*L. major*	47	462	508
LmjF19.0810	*L. major*	140	730	869
LmjF19.0840	*L. major*	41	1	41
LmjF19.0844	*L. major*	41	1	41
LmjF19.0848	*L. major*	41	1	41
LmjF19.0920	*L. major*	69	281	349
LmjF19.0930	*L. major*	53	1	53
LmjF19.0930	*L. major*	67	72	138
LmjF19.0930	*L. major*	80	382	461
LmjF19.0930	*L. major*	166	518	683
LmjF19.0930	*L. major*	70	693	762
LmjF19.0930	*L. major*	49	862	910
LmjF19.0940	*L. major*	487	96	582
LmjF19.0950	*L. major*	255	118	372

LmjF19.0950	*L. major*	69	376	444
LmjF19.0950	*L. major*	210	481	690
LmjF19.0980	*L. major*	50	1	50
LmjF19.0980	*L. major*	398	93	490
LmjF19.0980	*L. major*	48	560	607
LmjF19.0980	*L. major*	84	755	838
LmjF19.0985	*L. major*	43	571	613
LmjF19.1000	*L. major*	44	72	115
LmjF19.1000	*L. major*	43	534	576
LmjF19.1000	*L. major*	43	534	576
LmjF19.1005	*L. major*	89	515	603
LmjF19.1010	*L. major*	76	67	142
LmjF19.1010	*L. major*	47	247	293
LmjF19.1010	*L. major*	97	341	437
LmjF19.1010	*L. major*	85	447	531
LmjF19.1010	*L. major*	163	582	744
LmjF19.1020	*L. major*	71	1	71
LmjF19.1020	*L. major*	55	321	375
LmjF19.1020	*L. major*	55	321	375
LmjF19.1030	*L. major*	286	1	286
LmjF19.1040	*L. major*	44	304	347
LmjF19.1050	*L. major*	48	1	48
LmjF19.1050	*L. major*	457	99	555
LmjF19.1050	*L. major*	244	674	917
LmjF19.1050	*L. major*	229	988	1216
LmjF19.1060	*L. major*	113	12	124
LmjF19.1060	*L. major*	260	208	467
LmjF19.1060	*L. major*	83	501	583
LmjF19.1060	*L. major*	152	643	794
LmjF19.1060	*L. major*	163	833	995
LmjF19.1060	*L. major*	219	1032	1250
LmjF19.1070	*L. major*	77	75	151
LmjF19.1070	*L. major*	63	188	250
LmjF19.1070	*L. major*	43	350	392
LmjF19.1070	*L. major*	251	475	725
LmjF19.1070	*L. major*	45	902	946
LmjF19.1080	*L. major*	84	36	119
LmjF19.1080	*L. major*	62	178	239
LmjF19.1080	*L. major*	91	295	385
LmjF19.1080	*L. major*	138	400	537
LmjF19.1080	*L. major*	47	560	606
LmjF19.1080	*L. major*	218	665	882
LmjF19.1080	*L. major*	847	1015	1861
LmjF19.1080	*L. major*	269	1867	2135
LmjF19.1080	*L. major*	48	2178	2225
LmjF19.1080	*L. major*	55	2250	2304
LmjF19.1080	*L. major*	310	2608	2917

LmjF19.1080	*L. major*	97	2958	3054
LmjF19.1080	*L. major*	41	3554	3594
LmjF19.1080	*L. major*	102	3616	3717
LmjF19.1100	*L. major*	352	1	352
LmjF19.1100	*L. major*	292	359	650
LmjF19.1100	*L. major*	213	652	864
LmjF19.1100	*L. major*	59	905	963
LmjF19.1100	*L. major*	297	1022	1318
LmjF19.1130	*L. major*	89	1	89
LmjF19.1130	*L. major*	165	278	442
LmjF19.1130	*L. major*	80	682	761
LmjF19.1130	*L. major*	67	801	867
LmjF19.1130	*L. major*	73	916	988
LmjF19.1130	*L. major*	57	1069	1125
LmjF19.1130	*L. major*	74	1231	1304
LmjF19.1130	*L. major*	43	1359	1401
LmjF19.1130	*L. major*	57	1411	1467
LmjF19.1130	*L. major*	325	1532	1856
LmjF19.1130	*L. major*	67	1990	2056
LmjF19.1130	*L. major*	87	2318	2404
LmjF19.1130	*L. major*	55	2678	2732
LmjF19.1130	*L. major*	55	2829	2883
LmjF19.1130	*L. major*	43	2979	3021
LmjF19.1130	*L. major*	42	3088	3129
LmjF19.1130	*L. major*	44	3263	3306
LmjF19.1130	*L. major*	99	3409	3507
LmjF19.1130	*L. major*	59	3566	3624
LmjF19.1130	*L. major*	103	3690	3792
LmjF19.1130	*L. major*	47	3824	3870
LmjF19.1130	*L. major*	131	4017	4147
LmjF19.1130	*L. major*	176	4289	4464
LmjF19.1130	*L. major*	124	4481	4604
LmjF19.1130	*L. major*	71	4727	4797
LmjF19.1130	*L. major*	112	4926	5037
LmjF19.1130	*L. major*	149	5143	5291
LmjF19.1130	*L. major*	162	5321	5482
LmjF19.1130	*L. major*	129	5558	5686
LmjF19.1140	*L. major*	262	44	305
LmjF19.1140	*L. major*	50	334	383
LmjF19.1140	*L. major*	57	409	465
LmjF19.1140	*L. major*	151	502	652
LmjF19.1140	*L. major*	49	698	746
LmjF19.1150	*L. major*	806	83	888
LmjF19.1150	*L. major*	704	907	1610
LmjF19.1150	*L. major*	136	1643	1778
LmjF19.1150	*L. major*	639	1781	2419
LmjF19.1150	*L. major*	102	2421	2522

LmjF19.1150	*L. major*	123	2534	2656
LmjF19.1160	*L. major*	107	56	162
LmjF19.1160	*L. major*	64	226	289
LmjF19.1160	*L. major*	73	297	369
LmjF19.1170	*L. major*	47	1	47
LmjF19.1170	*L. major*	570	61	630
LmjF19.1170	*L. major*	268	661	928
LmjF19.1180	*L. major*	136	1	136
LmjF19.1180	*L. major*	490	162	651
LmjF19.1180	*L. major*	88	723	810
LmjF19.1180	*L. major*	255	924	1178
LmjF19.1180	*L. major*	139	1202	1340
LmjF19.1180	*L. major*	329	1355	1683
LmjF19.1210	*L. major*	48	1	48
LmjF19.1220	*L. major*	99	134	232
LmjF19.1230	*L. major*	48	61	108
LmjF19.1230	*L. major*	168	153	320
LmjF19.1240	*L. major*	42	70	111
LmjF19.1240	*L. major*	40	148	187
LmjF19.1240	*L. major*	136	223	358
LmjF19.1240	*L. major*	58	463	520
LmjF19.1240	*L. major*	52	757	808
LmjF19.1240	*L. major*	75	980	1054
LmjF19.1240	*L. major*	86	1277	1362
LmjF19.1240	*L. major*	184	1437	1620
LmjF19.1240	*L. major*	85	1772	1856
LmjF19.1250	*L. major*	56	87	142
LmjF19.1250	*L. major*	45	155	199
LmjF19.1250	*L. major*	40	368	407
LmjF19.1260	*L. major*	64	47	110
LmjF19.1270	*L. major*	107	121	227
LmjF19.1300	*L. major*	63	118	180
LmjF19.1310	*L. major*	227	31	257
LmjF19.1310	*L. major*	164	293	456
LmjF19.1310	*L. major*	103	466	568
LmjF19.1310	*L. major*	174	597	770
LmjF19.1310	*L. major*	88	869	956
LmjF19.1330	*L. major*	40	135	174
LmjF19.1340	*L. major*	53	1	53
LmjF19.1340	*L. major*	40	169	208
LmjF19.1340	*L. major*	40	169	208
LmjF19.1345	*L. major*	53	1	53
LmjF19.1345	*L. major*	42	172	213
LmjF19.1345	*L. major*	42	172	213
LmjF19.1347	*L. major*	47	146	192
LmjF19.1347	*L. major*	60	444	503
LmjF19.1347	*L. major*	60	444	503

LmjF19.1350	*L. major*	53	320	372
LmjF19.1365	*L. major*	53	24	76
LmjF19.1370	*L. major*	124	111	234
LmjF19.1370	*L. major*	133	250	382
LmjF19.1370	*L. major*	71	622	692
LmjF19.1380	*L. major*	42	1	42
LmjF19.1380	*L. major*	46	172	217
LmjF19.1380	*L. major*	60	460	519
LmjF19.1380	*L. major*	61	553	613
LmjF19.1410	*L. major*	68	164	231
LmjF19.1410	*L. major*	73	705	777
LmjF19.1410	*L. major*	73	705	777
LmjF19.1430	*L. major*	44	150	193
LmjF19.1430	*L. major*	78	210	287
LmjF19.1450	*L. major*	59	205	263
LmjF19.1470	*L. major*	41	1	41
LmjF19.1470	*L. major*	47	344	390
LmjF19.1470	*L. major*	44	509	552
LmjF19.1480	*L. major*	54	190	243
LmjF19.1500	*L. major*	54	190	243
LmjF19.1520	*L. major*	153	1	153
LmjF19.1520	*L. major*	88	180	267
LmjF19.1520	*L. major*	44	385	428
LmjF19.1520	*L. major*	44	578	621
LmjF19.1520	*L. major*	87	679	765
LmjF19.1550	*L. major*	40	89	128
LmjF19.1580	*L. major*	114	197	310
LmjF19.1590	*L. major*	41	73	113
LmjF19.1590	*L. major*	51	477	527
LmjF19.1590	*L. major*	49	550	598
LmjF19.1600	*L. major*	49	1	49
LmjF19.1620	*L. major*	49	260	308
LmjF19.1640	*L. major*	69	1	69
LmjF19.1640	*L. major*	42	508	549
LmjF19.1640	*L. major*	42	508	549
LmjF19.1650	*L. major*	112	45	156
LmjF20.0010	*L. major*	42	867	908
LmjF20.0020	*L. major*	58	353	410
LmjF20.0030	*L. major*	53	1	53
LmjF20.0060	*L. major*	284	12	295
LmjF20.0060	*L. major*	56	570	625
LmjF20.0060	*L. major*	52	855	906
LmjF20.0070	*L. major*	40	1	40
LmjF20.0080	*L. major*	99	352	450
LmjF20.0130	*L. major*	172	273	444
LmjF20.0140	*L. major*	158	183	340
LmjF20.0140	*L. major*	225	403	627

LmjF20.0140	*L. major*	96	1124	1219
LmjF20.0150	*L. major*	42	710	751
LmjF20.0150	*L. major*	41	868	908
LmjF20.0150	*L. major*	41	868	908
LmjF20.0160	*L. major*	170	524	693
LmjF20.0180	*L. major*	80	51	130
LmjF20.0180	*L. major*	53	171	223
LmjF20.0180	*L. major*	132	226	357
LmjF20.0180	*L. major*	86	390	475
LmjF20.0180	*L. major*	53	524	576
LmjF20.0180	*L. major*	40	706	745
LmjF20.0190	*L. major*	106	97	202
LmjF20.0200	*L. major*	70	1	70
LmjF20.0200	*L. major*	66	249	314
LmjF20.0200	*L. major*	43	356	398
LmjF20.0220	*L. major*	44	373	416
LmjF20.0230	*L. major*	67	1	67
LmjF20.0250	*L. major*	79	1	79
LmjF20.0260	*L. major*	41	1119	1159
LmjF20.0260	*L. major*	41	1881	1921
LmjF20.0260	*L. major*	83	2024	2106
LmjF20.0260	*L. major*	51	2916	2966
LmjF20.0260	*L. major*	52	3006	3057
LmjF20.0260	*L. major*	64	3379	3442
LmjF20.0260	*L. major*	90	3753	3842
LmjF20.0260	*L. major*	556	3949	4504
LmjF20.0260	*L. major*	53	4716	4768
LmjF20.0265	*L. major*	59	95	153
LmjF20.0265	*L. major*	40	223	262
LmjF20.0265	*L. major*	40	223	262
LmjF20.0270	*L. major*	67	1	67
LmjF20.0270	*L. major*	97	107	203
LmjF20.0270	*L. major*	85	213	297
LmjF20.0280	*L. major*	52	1	52
LmjF20.0290	*L. major*	53	154	206
LmjF20.0290	*L. major*	41	408	448
LmjF20.0290	*L. major*	56	718	773
LmjF20.0320	*L. major*	96	211	306
LmjF20.0320	*L. major*	119	446	564
LmjF20.0320	*L. major*	55	576	630
LmjF20.0330	*L. major*	164	31	194
LmjF20.0340	*L. major*	96	164	259
LmjF20.0340	*L. major*	84	306	389
LmjF20.0343	*L. major*	77	31	107
LmjF20.0346	*L. major*	51	67	117
LmjF20.0350	*L. major*	129	218	346
LmjF20.0350	*L. major*	129	602	730

LmjF20.0355	*L. major*	153	72	224
LmjF20.0355	*L. major*	41	264	304
LmjF20.0370	*L. major*	83	266	348
LmjF20.0370	*L. major*	50	428	477
LmjF20.0370	*L. major*	68	1146	1213
LmjF20.0375	*L. major*	66	464	529
LmjF20.0375	*L. major*	150	584	733
LmjF20.0375	*L. major*	58	1342	1399
LmjF20.0380	*L. major*	44	221	264
LmjF20.0390	*L. major*	91	221	311
LmjF20.0400	*L. major*	55	167	221
LmjF20.0400	*L. major*	49	298	346
LmjF20.0400	*L. major*	42	415	456
LmjF20.0400	*L. major*	56	496	551
LmjF20.0410	*L. major*	719	26	744
LmjF20.0410	*L. major*	601	746	1346
LmjF20.0420	*L. major*	139	1	139
LmjF20.0430	*L. major*	44	26	69
LmjF20.0430	*L. major*	95	329	423
LmjF20.0430	*L. major*	41	684	724
LmjF20.0440	*L. major*	49	169	217
LmjF20.0470	*L. major*	42	116	157
LmjF20.0470	*L. major*	112	390	501
LmjF20.0480	*L. major*	67	124	190
LmjF20.0480	*L. major*	40	270	309
LmjF20.0490	*L. major*	89	1	89
LmjF20.0500	*L. major*	69	209	277
LmjF20.0510	*L. major*	65	1	65
LmjF20.0510	*L. major*	61	329	389
LmjF20.0510	*L. major*	61	329	389
LmjF20.0520	*L. major*	226	121	346
LmjF20.0520	*L. major*	222	366	587
LmjF20.0520	*L. major*	54	703	756
LmjF20.0540	*L. major*	44	1	44
LmjF20.0540	*L. major*	121	112	232
LmjF20.0540	*L. major*	121	112	232
LmjF20.0550	*L. major*	41	72	112
LmjF20.0550	*L. major*	67	167	233
LmjF20.0550	*L. major*	43	486	528
LmjF20.0570	*L. major*	91	118	208
LmjF20.0570	*L. major*	42	308	349
LmjF20.0570	*L. major*	77	470	546
LmjF20.0580	*L. major*	146	1	146
LmjF20.0580	*L. major*	119	313	431
LmjF20.0590	*L. major*	67	64	130
LmjF20.0590	*L. major*	212	178	389
LmjF20.0590	*L. major*	70	509	578

LmjF20.0620	*L. major*	86	74	159
LmjF20.0620	*L. major*	58	228	285
LmjF20.0620	*L. major*	186	321	506
LmjF20.0620	*L. major*	435	542	976
LmjF20.0620	*L. major*	183	1036	1218
LmjF20.0620	*L. major*	84	1390	1473
LmjF20.0630	*L. major*	298	11	308
LmjF20.0630	*L. major*	152	310	461
LmjF20.0630	*L. major*	180	503	682
LmjF20.0630	*L. major*	397	692	1088
LmjF20.0630	*L. major*	121	1090	1210
LmjF20.0640	*L. major*	154	1	154
LmjF20.0640	*L. major*	58	185	242
LmjF20.0640	*L. major*	82	519	600
LmjF20.0640	*L. major*	252	740	991
LmjF20.0640	*L. major*	45	1017	1061
LmjF20.0650	*L. major*	70	142	211
LmjF20.0650	*L. major*	96	360	455
LmjF20.0650	*L. major*	96	360	455
LmjF20.0660	*L. major*	45	110	154
LmjF20.0660	*L. major*	42	558	599
LmjF20.0660	*L. major*	42	558	599
LmjF20.0670	*L. major*	42	143	184
LmjF20.0690	*L. major*	58	71	128
LmjF20.0690	*L. major*	139	162	300
LmjF20.0690	*L. major*	178	439	616
LmjF20.0690	*L. major*	169	668	836
LmjF20.0690	*L. major*	422	848	1269
LmjF20.0700	*L. major*	185	115	299
LmjF20.0700	*L. major*	440	621	1060
LmjF20.0705	*L. major*	170	42	211
LmjF20.0705	*L. major*	155	442	596
LmjF20.0705	*L. major*	56	660	715
LmjF20.0705	*L. major*	411	846	1256
LmjF20.0705	*L. major*	45	1279	1323
LmjF20.0705	*L. major*	316	1329	1644
LmjF20.0770	*L. major*	242	1	242
LmjF20.0770	*L. major*	403	292	694
LmjF20.0770	*L. major*	124	703	826
LmjF20.0770	*L. major*	136	850	985
LmjF20.0770	*L. major*	103	1023	1125
LmjF20.0770	*L. major*	615	1136	1750
LmjF20.0770	*L. major*	203	1772	1974
LmjF20.0770	*L. major*	277	2155	2431
LmjF20.0770	*L. major*	176	2524	2699
LmjF20.0770	*L. major*	99	2704	2802
LmjF20.0770	*L. major*	278	3096	3373

LmjF20.0770	*L. major*	115	3885	3999
LmjF20.0780	*L. major*	117	85	201
LmjF20.0780	*L. major*	56	452	507
LmjF20.0790	*L. major*	46	74	119
LmjF20.0790	*L. major*	42	303	344
LmjF20.0790	*L. major*	68	519	586
LmjF20.0790	*L. major*	58	668	725
LmjF20.0800	*L. major*	153	1	153
LmjF20.0800	*L. major*	112	183	294
LmjF20.0800	*L. major*	56	303	358
LmjF20.0810	*L. major*	53	686	738
LmjF20.0820	*L. major*	88	1	88
LmjF20.0830	*L. major*	53	8	60
LmjF20.0850	*L. major*	50	163	212
LmjF20.0850	*L. major*	90	605	694
LmjF20.0850	*L. major*	59	754	812
LmjF20.0850	*L. major*	66	969	1034
LmjF20.0850	*L. major*	52	1167	1218
LmjF20.0850	*L. major*	133	1327	1459
LmjF20.0850	*L. major*	51	1492	1542
LmjF20.0860	*L. major*	56	179	234
LmjF20.0870	*L. major*	73	129	201
LmjF20.0870	*L. major*	55	262	316
LmjF20.0870	*L. major*	56	431	486
LmjF20.0870	*L. major*	168	601	768
LmjF20.0880	*L. major*	58	103	160
LmjF20.0890	*L. major*	52	1	52
LmjF20.0890	*L. major*	160	133	292
LmjF20.0900	*L. major*	132	1	132
LmjF20.0900	*L. major*	60	421	480
LmjF20.0900	*L. major*	95	485	579
LmjF20.0900	*L. major*	72	709	780
LmjF20.0900	*L. major*	82	809	890
LmjF20.0910	*L. major*	189	95	283
LmjF20.0920	*L. major*	250	35	284
LmjF20.0920	*L. major*	60	348	407
LmjF20.0920	*L. major*	43	529	571
LmjF20.0930	*L. major*	47	204	250
LmjF20.0930	*L. major*	43	490	532
LmjF20.0930	*L. major*	51	666	716
LmjF20.0930	*L. major*	44	1524	1567
LmjF20.0930	*L. major*	56	1691	1746
LmjF20.0930	*L. major*	53	1766	1818
LmjF20.0930	*L. major*	125	1968	2092
LmjF20.0940	*L. major*	142	242	383
LmjF20.0940	*L. major*	71	392	462
LmjF20.0950	*L. major*	41	107	147

LmjF20.0950	*L. major*	51	297	347
LmjF20.0950	*L. major*	47	362	408
LmjF20.0950	*L. major*	256	448	703
LmjF20.0960	*L. major*	136	1	136
LmjF20.0960	*L. major*	131	142	272
LmjF20.0960	*L. major*	173	292	464
LmjF20.0960	*L. major*	77	509	585
LmjF20.0960	*L. major*	288	617	904
LmjF20.0960	*L. major*	331	918	1248
LmjF20.0960	*L. major*	93	1337	1429
LmjF20.0960	*L. major*	404	1441	1844
LmjF20.0960	*L. major*	630	1925	2554
LmjF20.0960	*L. major*	100	2589	2688
LmjF20.0960	*L. major*	43	2954	2996
LmjF20.0960	*L. major*	75	3172	3246
LmjF20.0960	*L. major*	44	3397	3440
LmjF20.0980	*L. major*	58	6	63
LmjF20.0980	*L. major*	41	73	113
LmjF20.0980	*L. major*	55	462	516
LmjF20.0980	*L. major*	160	533	692
LmjF20.0990	*L. major*	79	47	125
LmjF20.0990	*L. major*	63	375	437
LmjF20.0990	*L. major*	53	452	504
LmjF20.1000	*L. major*	611	112	722
LmjF20.1020	*L. major*	455	31	485
LmjF20.1020	*L. major*	492	529	1020
LmjF20.1030	*L. major*	151	1	151
LmjF20.1030	*L. major*	55	163	217
LmjF20.1040	*L. major*	432	30	461
LmjF20.1040	*L. major*	219	463	681
LmjF20.1040	*L. major*	45	703	747
LmjF20.1040	*L. major*	141	752	892
LmjF20.1050	*L. major*	597	1	597
LmjF20.1050	*L. major*	151	614	764
LmjF20.1050	*L. major*	158	780	937
LmjF20.1060	*L. major*	189	1	189
LmjF20.1060	*L. major*	293	207	499
LmjF20.1060	*L. major*	752	516	1267
LmjF20.1070	*L. major*	291	1	291
LmjF20.1080	*L. major*	53	75	127
LmjF20.1080	*L. major*	64	215	278
LmjF20.1080	*L. major*	49	454	502
LmjF20.1080	*L. major*	49	652	700
LmjF20.1080	*L. major*	87	780	866
LmjF20.1080	*L. major*	152	874	1025
LmjF20.1080	*L. major*	156	1043	1198
LmjF20.1080	*L. major*	132	1204	1335

LmjF20.1080	*L. major*	225	1397	1621
LmjF20.1080	*L. major*	126	1705	1830
LmjF20.1080	*L. major*	138	1848	1985
LmjF20.1080	*L. major*	274	2106	2379
LmjF20.1090	*L. major*	80	60	139
LmjF20.1090	*L. major*	224	150	373
LmjF20.1090	*L. major*	60	403	462
LmjF20.1090	*L. major*	223	496	718
LmjF20.1090	*L. major*	194	802	995
LmjF20.1090	*L. major*	131	1041	1171
LmjF20.1090	*L. major*	114	1272	1385
LmjF20.1090	*L. major*	57	1543	1599
LmjF20.1090	*L. major*	137	1661	1797
LmjF20.1090	*L. major*	53	1856	1908
LmjF20.1100	*L. major*	105	30	134
LmjF20.1100	*L. major*	42	155	196
LmjF20.1100	*L. major*	71	240	310
LmjF20.1100	*L. major*	65	344	408
LmjF20.1100	*L. major*	49	487	535
LmjF20.1100	*L. major*	81	761	841
LmjF20.1100	*L. major*	47	956	1002
LmjF20.1100	*L. major*	91	1010	1100
LmjF20.1100	*L. major*	64	1238	1301
LmjF20.1100	*L. major*	50	1450	1499
LmjF20.1100	*L. major*	120	1513	1632
LmjF20.1100	*L. major*	209	1686	1894
LmjF20.1100	*L. major*	41	1908	1948
LmjF20.1110	*L. major*	99	1	99
LmjF20.1110	*L. major*	1035	110	1144
LmjF20.1120	*L. major*	167	1	167
LmjF20.1120	*L. major*	119	609	727
LmjF20.1120	*L. major*	208	788	995
LmjF20.1120	*L. major*	132	1587	1718
LmjF20.1120	*L. major*	197	1849	2045
LmjF20.1120	*L. major*	47	2064	2110
LmjF20.1130	*L. major*	62	67	128
LmjF20.1130	*L. major*	106	283	388
LmjF20.1130	*L. major*	40	484	523
LmjF20.1130	*L. major*	86	568	653
LmjF20.1140	*L. major*	53	99	151
LmjF20.1150	*L. major*	70	130	199
LmjF20.1150	*L. major*	59	211	269
LmjF20.1150	*L. major*	59	211	269
LmjF20.1180	*L. major*	289	1	289
LmjF20.1185	*L. major*	133	1	133
LmjF20.1200	*L. major*	74	546	619
LmjF20.1240	*L. major*	221	1	221

LmjF20.1240	*L. major*	48	285	332
LmjF20.1240	*L. major*	49	464	512
LmjF20.1240	*L. major*	65	543	607
LmjF20.1240	*L. major*	209	851	1059
LmjF20.1240	*L. major*	42	1113	1154
LmjF20.1240	*L. major*	69	1319	1387
LmjF20.1240	*L. major*	60	1418	1477
LmjF20.1240	*L. major*	96	1593	1688
LmjF20.1240	*L. major*	76	1759	1834
LmjF20.1240	*L. major*	120	1918	2037
LmjF20.1240	*L. major*	44	2162	2205
LmjF20.1240	*L. major*	45	2243	2287
LmjF20.1240	*L. major*	52	2346	2397
LmjF20.1240	*L. major*	64	2656	2719
LmjF20.1250	*L. major*	122	1	122
LmjF20.1260	*L. major*	101	1	101
LmjF20.1260	*L. major*	223	153	375
LmjF20.1260	*L. major*	72	406	477
LmjF20.1260	*L. major*	253	592	844
LmjF20.1270	*L. major*	50	62	111
LmjF20.1290	*L. major*	117	121	237
LmjF20.1320	*L. major*	123	1	123
LmjF20.1340	*L. major*	94	284	377
LmjF20.1350	*L. major*	40	1	40
LmjF20.1350	*L. major*	71	496	566
LmjF20.1350	*L. major*	71	496	566
LmjF20.1360	*L. major*	71	38	108
LmjF20.1360	*L. major*	47	157	203
LmjF20.1360	*L. major*	47	157	203
LmjF20.1365	*L. major*	73	308	380
LmjF20.1365	*L. major*	40	434	473
LmjF20.1365	*L. major*	40	434	473
LmjF20.1370	*L. major*	84	308	391
LmjF20.1380	*L. major*	103	1	103
LmjF20.1380	*L. major*	77	286	362
LmjF20.1380	*L. major*	211	632	842
LmjF20.1380	*L. major*	385	870	1254
LmjF20.1390	*L. major*	373	1	373
LmjF20.1410	*L. major*	41	1	41
LmjF20.1410	*L. major*	152	61	212
LmjF20.1410	*L. major*	85	442	526
LmjF20.1410	*L. major*	53	692	744
LmjF20.1410	*L. major*	48	751	798
LmjF20.1420	*L. major*	110	105	214
LmjF20.1420	*L. major*	77	250	326
LmjF20.1420	*L. major*	99	448	546
LmjF20.1420	*L. major*	569	578	1146

LmjF20.1440	*L. major*	145	1	145
LmjF20.1450	*L. major*	174	194	367
LmjF20.1460	*L. major*	88	260	347
LmjF20.1470	*L. major*	85	1	85
LmjF20.1470	*L. major*	57	164	220
LmjF20.1470	*L. major*	41	374	414
LmjF20.1480	*L. major*	66	93	158
LmjF20.1480	*L. major*	141	390	530
LmjF20.1490	*L. major*	48	768	815
LmjF20.1490	*L. major*	71	859	929
LmjF20.1490	*L. major*	71	859	929
LmjF20.1500	*L. major*	91	334	424
LmjF20.1500	*L. major*	57	482	538
LmjF20.1500	*L. major*	80	700	779
LmjF20.1500	*L. major*	154	800	953
LmjF20.1510	*L. major*	47	1	47
LmjF20.1510	*L. major*	58	347	404
LmjF20.1510	*L. major*	40	790	829
LmjF20.1510	*L. major*	64	1347	1410
LmjF20.1510	*L. major*	45	1729	1773
LmjF20.1520	*L. major*	47	1	47
LmjF20.1520	*L. major*	69	314	382
LmjF20.1520	*L. major*	53	559	611
LmjF20.1520	*L. major*	55	864	918
LmjF20.1520	*L. major*	53	972	1024
LmjF20.1520	*L. major*	49	1084	1132
LmjF20.1520	*L. major*	95	1339	1433
LmjF20.1520	*L. major*	63	1463	1525
LmjF20.1530	*L. major*	91	177	267
LmjF20.1530	*L. major*	49	502	550
LmjF20.1530	*L. major*	43	739	781
LmjF20.1530	*L. major*	93	789	881
LmjF20.1540	*L. major*	62	60	121
LmjF20.1550	*L. major*	40	61	100
LmjF20.1560	*L. major*	40	62	101
LmjF20.1570	*L. major*	40	82	121
LmjF20.1580	*L. major*	65	99	163
LmjF20.1580	*L. major*	56	696	751
LmjF20.1580	*L. major*	43	818	860
LmjF20.1580	*L. major*	128	891	1018
LmjF20.1600	*L. major*	41	687	727
LmjF20.1610	*L. major*	47	208	254
LmjF20.1620	*L. major*	46	185	230
LmjF20.1630	*L. major*	44	1	44
LmjF20.1630	*L. major*	151	91	241
LmjF20.1640	*L. major*	43	113	155
LmjF20.1640	*L. major*	49	165	213

LmjF20.1640	*L. major*	49	165	213
LmjF20.1660	*L. major*	153	251	403
LmjF20.1670	*L. major*	103	1	103
LmjF20.1670	*L. major*	53	265	317
LmjF20.1680	*L. major*	120	686	805
LmjF20.1700	*L. major*	182	1	182
LmjF20.1700	*L. major*	74	206	279
LmjF20.1710	*L. major*	41	103	143
LmjF20.1710	*L. major*	49	554	602
LmjF20.1720	*L. major*	50	119	168
LmjF20.1740	*L. major*	47	72	118
LmjF20.1750	*L. major*	62	60	121
LmjF21.0010	*L. major*	93	1	93
LmjF21.0010	*L. major*	45	143	187
LmjF21.0030	*L. major*	96	104	199
LmjF21.0040	*L. major*	64	1	64
LmjF21.0040	*L. major*	60	125	184
LmjF21.0040	*L. major*	60	125	184
LmjF21.0050	*L. major*	62	90	151
LmjF21.0060	*L. major*	53	1	53
LmjF21.0070	*L. major*	45	182	226
LmjF21.0080	*L. major*	49	39	87
LmjF21.0100	*L. major*	57	95	151
LmjF21.0120	*L. major*	73	544	616
LmjF21.0120	*L. major*	57	662	718
LmjF21.0120	*L. major*	41	1036	1076
LmjF21.0125	*L. major*	47	380	426
LmjF21.0125	*L. major*	61	520	580
LmjF21.0125	*L. major*	41	693	733
LmjF21.0125	*L. major*	71	742	812
LmjF21.0130	*L. major*	65	1	65
LmjF21.0130	*L. major*	103	82	184
LmjF21.0130	*L. major*	100	199	298
LmjF21.0130	*L. major*	169	1227	1395
LmjF21.0140	*L. major*	71	24	94
LmjF21.0140	*L. major*	51	573	623
LmjF21.0140	*L. major*	51	573	623
LmjF21.0150	*L. major*	46	1	46
LmjF21.0160	*L. major*	45	141	185
LmjF21.0160	*L. major*	72	473	544
LmjF21.0160	*L. major*	72	473	544
LmjF21.0170	*L. major*	69	1	69
LmjF21.0170	*L. major*	44	152	195
LmjF21.0170	*L. major*	43	681	723
LmjF21.0180	*L. major*	328	174	501
LmjF21.0180	*L. major*	53	879	931
LmjF21.0180	*L. major*	53	1388	1440

LmjF21.0190	*L. major*	96	64	159
LmjF21.0190	*L. major*	47	195	241
LmjF21.0190	*L. major*	47	195	241
LmjF21.0200	*L. major*	92	454	545
LmjF21.0200	*L. major*	78	758	835
LmjF21.0200	*L. major*	78	758	835
LmjF21.0210	*L. major*	53	430	482
LmjF21.0230	*L. major*	46	299	344
LmjF21.0230	*L. major*	63	457	519
LmjF21.0230	*L. major*	63	457	519
LmjF21.0270	*L. major*	52	159	210
LmjF21.0270	*L. major*	114	274	387
LmjF21.0270	*L. major*	72	593	664
LmjF21.0270	*L. major*	89	720	808
LmjF21.0270	*L. major*	71	972	1042
LmjF21.0280	*L. major*	175	100	274
LmjF21.0280	*L. major*	45	409	453
LmjF21.0290	*L. major*	70	41	110
LmjF21.0290	*L. major*	66	149	214
LmjF21.0290	*L. major*	65	261	325
LmjF21.0290	*L. major*	54	370	423
LmjF21.0290	*L. major*	59	555	613
LmjF21.0290	*L. major*	61	778	838
LmjF21.0290	*L. major*	86	973	1058
LmjF21.0300	*L. major*	53	1	53
LmjF21.0300	*L. major*	57	187	243
LmjF21.0300	*L. major*	57	187	243
LmjF21.0310	*L. major*	44	176	219
LmjF21.0320	*L. major*	72	1	72
LmjF21.0320	*L. major*	103	128	230
LmjF21.0330	*L. major*	78	1	78
LmjF21.0330	*L. major*	90	639	728
LmjF21.0330	*L. major*	202	865	1066
LmjF21.0360	*L. major*	160	111	270
LmjF21.0370	*L. major*	61	1	61
LmjF21.0370	*L. major*	155	137	291
LmjF21.0370	*L. major*	73	341	413
LmjF21.0370	*L. major*	49	433	481
LmjF21.0390	*L. major*	41	252	292
LmjF21.0400	*L. major*	190	1	190
LmjF21.0400	*L. major*	55	338	392
LmjF21.0400	*L. major*	64	523	586
LmjF21.0410	*L. major*	98	49	146
LmjF21.0410	*L. major*	49	201	249
LmjF21.0410	*L. major*	42	399	440
LmjF21.0410	*L. major*	53	534	586
LmjF21.0410	*L. major*	62	657	718

LmjF21.0410	*L. major*	40	1086	1125
LmjF21.0420	*L. major*	63	26	88
LmjF21.0420	*L. major*	53	342	394
LmjF21.0420	*L. major*	51	523	573
LmjF21.0430	*L. major*	168	160	327
LmjF21.0450	*L. major*	51	55	105
LmjF21.0460	*L. major*	48	39	86
LmjF21.0460	*L. major*	41	213	253
LmjF21.0460	*L. major*	193	384	576
LmjF21.0460	*L. major*	146	608	753
LmjF21.0460	*L. major*	42	798	839
LmjF21.0470	*L. major*	64	240	303
LmjF21.0480	*L. major*	94	17	110
LmjF21.0480	*L. major*	86	120	205
LmjF21.0480	*L. major*	49	284	332
LmjF21.0480	*L. major*	56	441	496
LmjF21.0490	*L. major*	57	35	91
LmjF21.0490	*L. major*	83	366	448
LmjF21.0510	*L. major*	43	1	43
LmjF21.0510	*L. major*	46	63	108
LmjF21.0510	*L. major*	109	457	565
LmjF21.0520	*L. major*	50	367	416
LmjF21.0530	*L. major*	43	87	129
LmjF21.0530	*L. major*	144	157	300
LmjF21.0530	*L. major*	56	422	477
LmjF21.0530	*L. major*	58	737	794
LmjF21.0540	*L. major*	59	166	224
LmjF21.0540	*L. major*	72	268	339
LmjF21.0550	*L. major*	62	91	152
LmjF21.0550	*L. major*	133	221	353
LmjF21.0550	*L. major*	133	221	353
LmjF21.0570	*L. major*	235	181	415
LmjF21.0580	*L. major*	108	139	246
LmjF21.0580	*L. major*	86	251	336
LmjF21.0580	*L. major*	45	382	426
LmjF21.0590	*L. major*	160	1	160
LmjF21.0590	*L. major*	40	178	217
LmjF21.0590	*L. major*	131	258	388
LmjF21.0600	*L. major*	53	360	412
LmjF21.0610	*L. major*	76	213	288
LmjF21.0610	*L. major*	71	424	494
LmjF21.0620	*L. major*	51	169	219
LmjF21.0620	*L. major*	45	357	401
LmjF21.0620	*L. major*	65	511	575
LmjF21.0620	*L. major*	195	592	786
LmjF21.0630	*L. major*	54	146	199
LmjF21.0630	*L. major*	40	336	375

LmjF21.0630	*L. major*	242	519	760
LmjF21.0650	*L. major*	49	87	135
LmjF21.0650	*L. major*	94	200	293
LmjF21.0650	*L. major*	48	496	543
LmjF21.0660	*L. major*	47	1	47
LmjF21.0700	*L. major*	379	243	621
LmjF21.0710	*L. major*	83	577	659
LmjF21.0720	*L. major*	64	68	131
LmjF21.0725	*L. major*	45	333	377
LmjF21.0770	*L. major*	73	1	73
LmjF21.0770	*L. major*	196	302	497
LmjF21.0780	*L. major*	107	95	201
LmjF21.0780	*L. major*	130	475	604
LmjF21.0780	*L. major*	50	615	664
LmjF21.0790	*L. major*	151	138	288
LmjF21.0800	*L. major*	59	764	822
LmjF21.0800	*L. major*	74	1075	1148
LmjF21.0800	*L. major*	44	1316	1359
LmjF21.0800	*L. major*	40	1385	1424
LmjF21.0800	*L. major*	50	1448	1497
LmjF21.0820	*L. major*	538	1	538
LmjF21.0820	*L. major*	49	1066	1114
LmjF21.0823	*L. major*	53	64	116
LmjF21.0823	*L. major*	61	242	302
LmjF21.0823	*L. major*	131	393	523
LmjF21.0823	*L. major*	77	700	776
LmjF21.0823	*L. major*	120	806	925
LmjF21.0825	*L. major*	250	1	250
LmjF21.0825	*L. major*	524	276	799
LmjF21.0825	*L. major*	1067	856	1922
LmjF21.0825	*L. major*	429	2002	2430
LmjF21.0825	*L. major*	346	2440	2785
LmjF21.0825	*L. major*	71	2797	2867
LmjF21.0825	*L. major*	92	2875	2966
LmjF21.0825	*L. major*	86	3005	3090
LmjF21.0825	*L. major*	58	3123	3180
LmjF21.0827	*L. major*	44	61	104
LmjF21.0830	*L. major*	66	257	322
LmjF21.0840	*L. major*	163	1	163
LmjF21.0843	*L. major*	136	1	136
LmjF21.0843	*L. major*	66	254	319
LmjF21.0853	*L. major*	157	141	297
LmjF21.0853	*L. major*	272	484	755
LmjF21.0853	*L. major*	76	759	834
LmjF21.0853	*L. major*	338	864	1201
LmjF21.0853	*L. major*	127	1211	1337
LmjF21.0853	*L. major*	352	1386	1737

LmjF21.0853	*L. major*	61	1784	1844
LmjF21.0853	*L. major*	187	1864	2050
LmjF21.0853	*L. major*	93	2153	2245
LmjF21.0855	*L. major*	52	1	52
LmjF21.0855	*L. major*	57	88	144
LmjF21.0855	*L. major*	48	188	235
LmjF21.0861	*L. major*	1598	1	1598
LmjF21.0861	*L. major*	147	1699	1845
LmjF21.0861	*L. major*	213	1868	2080
LmjF21.0861	*L. major*	57	2164	2220
LmjF21.0861	*L. major*	198	2235	2432
LmjF21.0861	*L. major*	122	2450	2571
LmjF21.0865	*L. major*	110	1	110
LmjF21.0865	*L. major*	42	139	180
LmjF21.0865	*L. major*	62	225	286
LmjF21.0865	*L. major*	60	383	442
LmjF21.0865	*L. major*	51	491	541
LmjF21.0865	*L. major*	47	630	676
LmjF21.0870	*L. major*	125	1	125
LmjF21.0870	*L. major*	99	144	242
LmjF21.0870	*L. major*	40	248	287
LmjF21.0870	*L. major*	98	319	416
LmjF21.0870	*L. major*	187	481	667
LmjF21.0870	*L. major*	229	685	913
LmjF21.0873	*L. major*	294	562	855
LmjF21.0875	*L. major*	48	1	48
LmjF21.0875	*L. major*	186	82	267
LmjF21.0875	*L. major*	184	285	468
LmjF21.0875	*L. major*	87	768	854
LmjF21.0880	*L. major*	93	1	93
LmjF21.0880	*L. major*	81	295	375
LmjF21.0880	*L. major*	54	435	488
LmjF21.0880	*L. major*	70	509	578
LmjF21.0885	*L. major*	90	1	90
LmjF21.0885	*L. major*	140	97	236
LmjF21.0885	*L. major*	41	517	557
LmjF21.0885	*L. major*	45	580	624
LmjF21.0885	*L. major*	67	657	723
LmjF21.0885	*L. major*	86	812	897
LmjF21.0885	*L. major*	166	982	1147
LmjF21.0891	*L. major*	48	248	295
LmjF21.0891	*L. major*	116	437	552
LmjF21.0895	*L. major*	155	1	155
LmjF21.0895	*L. major*	63	390	452
LmjF21.0901	*L. major*	85	1	85
LmjF21.0901	*L. major*	97	126	222
LmjF21.0901	*L. major*	97	126	222

LmjF21.0905	*L. major*	51	61	111
LmjF21.0905	*L. major*	47	159	205
LmjF21.0905	*L. major*	47	159	205
LmjF21.0910	*L. major*	85	1	85
LmjF21.0915	*L. major*	48	85	132
LmjF21.0920	*L. major*	48	85	132
LmjF21.0930	*L. major*	48	85	132
LmjF21.0950	*L. major*	109	78	186
LmjF21.0950	*L. major*	52	279	330
LmjF21.0950	*L. major*	66	365	430
LmjF21.0960	*L. major*	234	1	234
LmjF21.0960	*L. major*	278	271	548
LmjF21.0960	*L. major*	100	614	713
LmjF21.0960	*L. major*	155	773	927
LmjF21.0960	*L. major*	474	978	1451
LmjF21.0970	*L. major*	93	1	93
LmjF21.0970	*L. major*	43	116	158
LmjF21.0970	*L. major*	117	225	341
LmjF21.0970	*L. major*	66	513	578
LmjF21.0970	*L. major*	60	654	713
LmjF21.1000	*L. major*	69	1	69
LmjF21.1000	*L. major*	76	108	183
LmjF21.1000	*L. major*	69	222	290
LmjF21.1000	*L. major*	884	379	1262
LmjF21.1000	*L. major*	783	1267	2049
LmjF21.1010	*L. major*	40	202	241
LmjF21.1020	*L. major*	76	212	287
LmjF21.1030	*L. major*	126	32	157
LmjF21.1030	*L. major*	647	168	814
LmjF21.1040	*L. major*	58	93	150
LmjF21.1040	*L. major*	59	630	688
LmjF21.1040	*L. major*	344	847	1190
LmjF21.1040	*L. major*	134	1313	1446
LmjF21.1040	*L. major*	206	1617	1822
LmjF21.1040	*L. major*	266	1856	2121
LmjF21.1090	*L. major*	43	1	43
LmjF21.1100	*L. major*	113	37	149
LmjF21.1100	*L. major*	53	172	224
LmjF21.1100	*L. major*	64	237	300
LmjF21.1100	*L. major*	88	613	700
LmjF21.1110	*L. major*	47	73	119
LmjF21.1110	*L. major*	75	173	247
LmjF21.1110	*L. major*	336	468	803
LmjF21.1110	*L. major*	124	846	969
LmjF21.1110	*L. major*	64	1247	1310
LmjF21.1110	*L. major*	40	1513	1552
LmjF21.1110	*L. major*	71	1591	1661

LmjF21.1110	*L. major*	49	1713	1761
LmjF21.1120	*L. major*	41	209	249
LmjF21.1120	*L. major*	78	537	614
LmjF21.1130	*L. major*	128	15	142
LmjF21.1130	*L. major*	248	207	454
LmjF21.1130	*L. major*	67	477	543
LmjF21.1140	*L. major*	52	64	115
LmjF21.1140	*L. major*	150	154	303
LmjF21.1150	*L. major*	325	1	325
LmjF21.1150	*L. major*	45	334	378
LmjF21.1150	*L. major*	87	380	466
LmjF21.1150	*L. major*	169	533	701
LmjF21.1150	*L. major*	217	743	959
LmjF21.1150	*L. major*	65	987	1051
LmjF21.1150	*L. major*	521	1061	1581
LmjF21.1150	*L. major*	51	1602	1652
LmjF21.1150	*L. major*	79	1654	1732
LmjF21.1160	*L. major*	655	1	655
LmjF21.1160	*L. major*	97	723	819
LmjF21.1160	*L. major*	52	869	920
LmjF21.1160	*L. major*	49	1086	1134
LmjF21.1170	*L. major*	44	147	190
LmjF21.1180	*L. major*	47	141	187
LmjF21.1190	*L. major*	142	115	256
LmjF21.1190	*L. major*	72	715	786
LmjF21.1200	*L. major*	126	56	181
LmjF21.1210	*L. major*	104	181	284
LmjF21.1220	*L. major*	219	66	284
LmjF21.1220	*L. major*	155	380	534
LmjF21.1220	*L. major*	376	611	986
LmjF21.1220	*L. major*	61	989	1049
LmjF21.1220	*L. major*	300	1073	1372
LmjF21.1230	*L. major*	93	1	93
LmjF21.1230	*L. major*	58	423	480
LmjF21.1230	*L. major*	58	524	581
LmjF21.1230	*L. major*	45	594	638
LmjF21.1240	*L. major*	71	1	71
LmjF21.1240	*L. major*	48	419	466
LmjF21.1240	*L. major*	201	476	676
LmjF21.1240	*L. major*	433	762	1194
LmjF21.1240	*L. major*	44	1219	1262
LmjF21.1260	*L. major*	74	196	269
LmjF21.1260	*L. major*	173	328	500
LmjF21.1270	*L. major*	76	316	391
LmjF21.1270	*L. major*	96	442	537
LmjF21.1270	*L. major*	73	718	790
LmjF21.1280	*L. major*	110	1	110

LmjF21.1280	*L. major*	42	335	376
LmjF21.1295	*L. major*	138	98	235
LmjF21.1300	*L. major*	130	1	130
LmjF21.1300	*L. major*	188	137	324
LmjF21.1300	*L. major*	41	389	429
LmjF21.1300	*L. major*	50	488	537
LmjF21.1330	*L. major*	169	1	169
LmjF21.1330	*L. major*	49	368	416
LmjF21.1330	*L. major*	253	419	671
LmjF21.1330	*L. major*	170	814	983
LmjF21.1330	*L. major*	261	990	1250
LmjF21.1330	*L. major*	109	1484	1592
LmjF21.1350	*L. major*	80	1	80
LmjF21.1350	*L. major*	53	103	155
LmjF21.1350	*L. major*	360	186	545
LmjF21.1350	*L. major*	45	557	601
LmjF21.1350	*L. major*	57	807	863
LmjF21.1350	*L. major*	98	1100	1197
LmjF21.1350	*L. major*	463	1311	1773
LmjF21.1360	*L. major*	52	85	136
LmjF21.1370	*L. major*	216	282	497
LmjF21.1370	*L. major*	85	529	613
LmjF21.1370	*L. major*	74	713	786
LmjF21.1370	*L. major*	66	835	900
LmjF21.1370	*L. major*	119	931	1049
LmjF21.1380	*L. major*	41	122	162
LmjF21.1380	*L. major*	122	246	367
LmjF21.1390	*L. major*	107	113	219
LmjF21.1400	*L. major*	107	113	219
LmjF21.1410	*L. major*	51	1	51
LmjF21.1410	*L. major*	74	90	163
LmjF21.1410	*L. major*	92	221	312
LmjF21.1430	*L. major*	40	75	114
LmjF21.1430	*L. major*	40	369	408
LmjF21.1430	*L. major*	40	369	408
LmjF21.1450	*L. major*	59	117	175
LmjF21.1450	*L. major*	112	431	542
LmjF21.1460	*L. major*	274	28	301
LmjF21.1460	*L. major*	180	332	511
LmjF21.1480	*L. major*	182	1	182
LmjF21.1480	*L. major*	78	273	350
LmjF21.1480	*L. major*	83	362	444
LmjF21.1480	*L. major*	300	453	752
LmjF21.1490	*L. major*	349	1	349
LmjF21.1490	*L. major*	230	441	670
LmjF21.1500	*L. major*	56	495	550
LmjF21.1510	*L. major*	80	57	136

LmjF21.1510	*L. major*	269	192	460
LmjF21.1510	*L. major*	53	624	676
LmjF21.1510	*L. major*	80	716	795
LmjF21.1510	*L. major*	41	832	872
LmjF21.1510	*L. major*	131	879	1009
LmjF21.1510	*L. major*	164	1239	1402
LmjF21.1520	*L. major*	95	65	159
LmjF21.1520	*L. major*	185	187	371
LmjF21.1530	*L. major*	57	452	508
LmjF21.1530	*L. major*	62	578	639
LmjF21.1530	*L. major*	62	578	639
LmjF21.1540	*L. major*	158	1	158
LmjF21.1540	*L. major*	47	417	463
LmjF21.1540	*L. major*	41	650	690
LmjF21.1540	*L. major*	335	1552	1886
LmjF21.1552	*L. major*	46	390	435
LmjF21.1558	*L. major*	89	10	98
LmjF21.1558	*L. major*	42	133	174
LmjF21.1558	*L. major*	42	133	174
LmjF21.1560	*L. major*	251	280	530
LmjF21.1561	*L. major*	107	1	107
LmjF21.1561	*L. major*	72	170	241
LmjF21.1561	*L. major*	167	271	437
LmjF21.1565	*L. major*	436	266	701
LmjF21.1566	*L. major*	300	82	381
LmjF21.1566	*L. major*	52	399	450
LmjF21.1568	*L. major*	74	1	74
LmjF21.1569	*L. major*	51	126	176
LmjF21.1569	*L. major*	51	389	439
LmjF21.1569	*L. major*	51	389	439
LmjF21.1580	*L. major*	66	324	389
LmjF21.1600	*L. major*	48	1	48
LmjF21.1600	*L. major*	74	102	175
LmjF21.1600	*L. major*	74	102	175
LmjF21.1605	*L. major*	48	1	48
LmjF21.1610	*L. major*	65	96	160
LmjF21.1650	*L. major*	59	31	89
LmjF21.1675	*L. major*	74	21	94
LmjF21.1680	*L. major*	143	29	171
LmjF21.1680	*L. major*	67	503	569
LmjF21.1720	*L. major*	63	1	63
LmjF21.1730	*L. major*	171	1	171
LmjF21.1750	*L. major*	54	131	184
LmjF21.1780	*L. major*	120	130	249
LmjF21.1840	*L. major*	44	1	44
LmjF21.1840	*L. major*	56	91	146
LmjF21.1840	*L. major*	286	179	464

LmjF21.1850	*L. major*	95	1	95
LmjF21.1850	*L. major*	72	132	203
LmjF21.1850	*L. major*	338	308	645
LmjF21.1860	*L. major*	40	404	443
LmjF21.1870	*L. major*	57	42	98
LmjF21.1870	*L. major*	156	311	466
LmjF21.1880	*L. major*	191	53	243
LmjF22.0010	*L. major*	58	1	58
LmjF22.0010	*L. major*	72	504	575
LmjF22.0010	*L. major*	72	504	575
LmjF22.0020	*L. major*	48	97	144
LmjF22.0030	*L. major*	50	139	188
LmjF22.0050	*L. major*	194	1	194
LmjF22.0050	*L. major*	267	204	470
LmjF22.0060	*L. major*	284	87	370
LmjF22.0060	*L. major*	183	372	554
LmjF22.0060	*L. major*	183	372	554
LmjF22.0070	*L. major*	77	213	289
LmjF22.0080	*L. major*	86	52	137
LmjF22.0080	*L. major*	53	279	331
LmjF22.0080	*L. major*	53	279	331
LmjF22.0090	*L. major*	45	683	727
LmjF22.0100	*L. major*	56	21	76
LmjF22.0120	*L. major*	194	20	213
LmjF22.0140	*L. major*	225	174	398
LmjF22.0150	*L. major*	61	84	144
LmjF22.0160	*L. major*	71	1	71
LmjF22.0160	*L. major*	48	166	213
LmjF22.0160	*L. major*	148	263	410
LmjF22.0170	*L. major*	422	1	422
LmjF22.0180	*L. major*	144	1	144
LmjF22.0210	*L. major*	145	1	145
LmjF22.0210	*L. major*	113	214	326
LmjF22.0210	*L. major*	40	386	425
LmjF22.0210	*L. major*	129	929	1057
LmjF22.0210	*L. major*	144	1385	1528
LmjF22.0220	*L. major*	52	692	743
LmjF22.0220	*L. major*	43	852	894
LmjF22.0230	*L. major*	56	1	56
LmjF22.0240	*L. major*	70	1	70
LmjF22.0240	*L. major*	237	93	329
LmjF22.0260	*L. major*	64	219	282
LmjF22.0280	*L. major*	42	364	405
LmjF22.0280	*L. major*	50	572	621
LmjF22.0280	*L. major*	65	777	841
LmjF22.0290	*L. major*	48	216	263
LmjF22.0290	*L. major*	62	341	402

LmjF22.0290	*L. major*	62	341	402
LmjF22.0300	*L. major*	57	1	57
LmjF22.0300	*L. major*	49	185	233
LmjF22.0300	*L. major*	50	415	464
LmjF22.0300	*L. major*	75	487	561
LmjF22.0310	*L. major*	50	500	549
LmjF22.0320	*L. major*	90	43	132
LmjF22.0320	*L. major*	51	232	282
LmjF22.0330	*L. major*	486	1	486
LmjF22.0330	*L. major*	123	511	633
LmjF22.0330	*L. major*	62	660	721
LmjF22.0330	*L. major*	53	723	775
LmjF22.0330	*L. major*	253	790	1042
LmjF22.0350	*L. major*	136	1	136
LmjF22.0360	*L. major*	58	1	58
LmjF22.0360	*L. major*	60	114	173
LmjF22.0360	*L. major*	45	268	312
LmjF22.0370	*L. major*	41	45	85
LmjF22.0370	*L. major*	43	132	174
LmjF22.0370	*L. major*	43	132	174
LmjF22.0380	*L. major*	46	1	46
LmjF22.0380	*L. major*	105	212	316
LmjF22.0380	*L. major*	63	519	581
LmjF22.0380	*L. major*	80	687	766
LmjF22.0390	*L. major*	48	43	90
LmjF22.0390	*L. major*	41	411	451
LmjF22.0390	*L. major*	41	411	451
LmjF22.0400	*L. major*	104	26	129
LmjF22.0400	*L. major*	119	190	308
LmjF22.0400	*L. major*	67	416	482
LmjF22.0410	*L. major*	51	302	352
LmjF22.0410	*L. major*	41	767	807
LmjF22.0410	*L. major*	76	1110	1185
LmjF22.0420	*L. major*	45	39	83
LmjF22.0440	*L. major*	123	1	123
LmjF22.0440	*L. major*	81	161	241
LmjF22.0440	*L. major*	60	276	335
LmjF22.0440	*L. major*	165	991	1155
LmjF22.0450	*L. major*	86	1	86
LmjF22.0460	*L. major*	45	39	83
LmjF22.0470	*L. major*	90	175	264
LmjF22.0480	*L. major*	61	209	269
LmjF22.0490	*L. major*	42	1	42
LmjF22.0490	*L. major*	44	94	137
LmjF22.0490	*L. major*	62	248	309
LmjF22.0490	*L. major*	122	455	576
LmjF22.0500	*L. major*	229	54	282

LmjF22.0500	*L. major*	53	426	478
LmjF22.0510	*L. major*	42	1	42
LmjF22.0510	*L. major*	63	85	147
LmjF22.0510	*L. major*	97	180	276
LmjF22.0510	*L. major*	117	328	444
LmjF22.0510	*L. major*	46	526	571
LmjF22.0520	*L. major*	154	131	284
LmjF22.0520	*L. major*	50	318	367
LmjF22.0520	*L. major*	83	493	575
LmjF22.0520	*L. major*	136	602	737
LmjF22.0520	*L. major*	63	1059	1121
LmjF22.0530	*L. major*	41	1	41
LmjF22.0530	*L. major*	140	169	308
LmjF22.0540	*L. major*	54	93	146
LmjF22.0540	*L. major*	154	236	389
LmjF22.0540	*L. major*	45	521	565
LmjF22.0540	*L. major*	131	701	831
LmjF22.0560	*L. major*	40	152	191
LmjF22.0560	*L. major*	114	227	340
LmjF22.0560	*L. major*	239	485	723
LmjF22.0560	*L. major*	49	756	804
LmjF22.0570	*L. major*	52	307	358
LmjF22.0590	*L. major*	81	94	174
LmjF22.0590	*L. major*	75	304	378
LmjF22.0590	*L. major*	75	304	378
LmjF22.0600	*L. major*	61	110	170
LmjF22.0600	*L. major*	45	281	325
LmjF22.0600	*L. major*	75	395	469
LmjF22.0610	*L. major*	105	1	105
LmjF22.0610	*L. major*	57	204	260
LmjF22.0620	*L. major*	129	1	129
LmjF22.0630	*L. major*	275	1	275
LmjF22.0630	*L. major*	268	319	586
LmjF22.0630	*L. major*	96	602	697
LmjF22.0630	*L. major*	193	723	915
LmjF22.0630	*L. major*	96	926	1021
LmjF22.0630	*L. major*	71	1071	1141
LmjF22.0640	*L. major*	176	62	237
LmjF22.0640	*L. major*	51	545	595
LmjF22.0640	*L. major*	42	991	1032
LmjF22.0640	*L. major*	67	1271	1337
LmjF22.0640	*L. major*	57	1515	1571
LmjF22.0660	*L. major*	274	1	274
LmjF22.0660	*L. major*	125	308	432
LmjF22.0660	*L. major*	72	458	529
LmjF22.0660	*L. major*	53	803	855
LmjF22.0660	*L. major*	83	971	1053

LmjF22.0670	*L. major*	42	116	157
LmjF22.0690	*L. major*	114	103	216
LmjF22.0690	*L. major*	125	316	440
LmjF22.0690	*L. major*	49	467	515
LmjF22.0690	*L. major*	69	531	599
LmjF22.0700	*L. major*	798	1	798
LmjF22.0700	*L. major*	95	810	904
LmjF22.0700	*L. major*	230	916	1145
LmjF22.0700	*L. major*	337	1189	1525
LmjF22.0720	*L. major*	179	67	245
LmjF22.0720	*L. major*	69	320	388
LmjF22.0720	*L. major*	190	641	830
LmjF22.0720	*L. major*	70	833	902
LmjF22.0720	*L. major*	118	904	1021
LmjF22.0720	*L. major*	56	1025	1080
LmjF22.0730	*L. major*	160	1	160
LmjF22.0730	*L. major*	162	264	425
LmjF22.0740	*L. major*	172	241	412
LmjF22.0740	*L. major*	120	434	553
LmjF22.0750	*L. major*	72	10	81
LmjF22.0750	*L. major*	149	104	252
LmjF22.0750	*L. major*	79	267	345
LmjF22.0750	*L. major*	92	347	438
LmjF22.0750	*L. major*	208	459	666
LmjF22.0750	*L. major*	277	855	1131
LmjF22.0770	*L. major*	42	248	289
LmjF22.0780	*L. major*	51	1	51
LmjF22.0780	*L. major*	42	248	289
LmjF22.0780	*L. major*	42	248	289
LmjF22.0790	*L. major*	51	215	265
LmjF22.0800	*L. major*	83	140	222
LmjF22.0810	*L. major*	88	285	372
LmjF22.0820	*L. major*	51	1	51
LmjF22.0820	*L. major*	69	126	194
LmjF22.0820	*L. major*	384	447	830
LmjF22.0820	*L. major*	56	834	889
LmjF22.0840	*L. major*	798	1	798
LmjF22.0840	*L. major*	95	810	904
LmjF22.0840	*L. major*	128	916	1043
LmjF22.0840	*L. major*	96	1050	1145
LmjF22.0840	*L. major*	337	1189	1525
LmjF22.0850	*L. major*	106	111	216
LmjF22.0850	*L. major*	125	316	440
LmjF22.0850	*L. major*	49	467	515
LmjF22.0850	*L. major*	69	531	599
LmjF22.0860	*L. major*	86	117	202
LmjF22.0860	*L. major*	47	406	452

LmjF22.0860	*L. major*	80	672	751
LmjF22.0860	*L. major*	47	803	849
LmjF22.0880	*L. major*	110	67	176
LmjF22.0880	*L. major*	142	196	337
LmjF22.0880	*L. major*	70	393	462
LmjF22.0880	*L. major*	95	546	640
LmjF22.0890	*L. major*	90	1	90
LmjF22.0900	*L. major*	247	1	247
LmjF22.0900	*L. major*	118	598	715
LmjF22.0900	*L. major*	69	748	816
LmjF22.0910	*L. major*	170	1	170
LmjF22.0910	*L. major*	55	199	253
LmjF22.0920	*L. major*	57	69	125
LmjF22.0920	*L. major*	46	129	174
LmjF22.0920	*L. major*	85	405	489
LmjF22.0920	*L. major*	92	736	827
LmjF22.0930	*L. major*	46	465	510
LmjF22.0930	*L. major*	76	611	686
LmjF22.0930	*L. major*	76	611	686
LmjF22.0940	*L. major*	46	478	523
LmjF22.0940	*L. major*	76	624	699
LmjF22.0940	*L. major*	76	624	699
LmjF22.0960	*L. major*	58	21	78
LmjF22.0960	*L. major*	98	355	452
LmjF22.0960	*L. major*	430	471	900
LmjF22.0960	*L. major*	347	911	1257
LmjF22.0970	*L. major*	441	58	498
LmjF22.0970	*L. major*	388	613	1000
LmjF22.0970	*L. major*	91	1035	1125
LmjF22.0970	*L. major*	177	1207	1383
LmjF22.0970	*L. major*	449	1470	1918
LmjF22.0980	*L. major*	61	1	61
LmjF22.0980	*L. major*	53	398	450
LmjF22.0980	*L. major*	53	398	450
LmjF22.0990	*L. major*	45	139	183
LmjF22.0990	*L. major*	59	715	773
LmjF22.0990	*L. major*	54	872	925
LmjF22.0990	*L. major*	79	1189	1267
LmjF22.1000	*L. major*	85	97	181
LmjF22.1000	*L. major*	106	186	291
LmjF22.1000	*L. major*	60	375	434
LmjF22.1010	*L. major*	43	254	296
LmjF22.1010	*L. major*	84	497	580
LmjF22.1020	*L. major*	173	1	173
LmjF22.1020	*L. major*	47	298	344
LmjF22.1020	*L. major*	42	384	425
LmjF22.1030	*L. major*	236	21	256

LmjF22.1030	*L. major*	40	477	516
LmjF22.1030	*L. major*	82	782	863
LmjF22.1030	*L. major*	49	1187	1235
LmjF22.1040	*L. major*	60	93	152
LmjF22.1040	*L. major*	69	181	249
LmjF22.1040	*L. major*	221	300	520
LmjF22.1040	*L. major*	88	652	739
LmjF22.1050	*L. major*	101	1	101
LmjF22.1050	*L. major*	83	295	377
LmjF22.1050	*L. major*	148	672	819
LmjF22.1050	*L. major*	60	868	927
LmjF22.1070	*L. major*	110	1	110
LmjF22.1070	*L. major*	322	138	459
LmjF22.1070	*L. major*	178	490	667
LmjF22.1070	*L. major*	64	699	762
LmjF22.1080	*L. major*	65	1	65
LmjF22.1080	*L. major*	68	129	196
LmjF22.1090	*L. major*	56	1	56
LmjF22.1090	*L. major*	47	174	220
LmjF22.1090	*L. major*	173	355	527
LmjF22.1090	*L. major*	67	538	604
LmjF22.1090	*L. major*	41	662	702
LmjF22.1100	*L. major*	112	76	187
LmjF22.1100	*L. major*	142	220	361
LmjF22.1100	*L. major*	91	433	523
LmjF22.1100	*L. major*	96	531	626
LmjF22.1100	*L. major*	387	676	1062
LmjF22.1100	*L. major*	52	1097	1148
LmjF22.1100	*L. major*	61	1176	1236
LmjF22.1100	*L. major*	230	1238	1467
LmjF22.1100	*L. major*	69	1473	1541
LmjF22.1100	*L. major*	63	1574	1636
LmjF22.1100	*L. major*	180	1653	1832
LmjF22.1100	*L. major*	59	1857	1915
LmjF22.1100	*L. major*	98	1945	2042
LmjF22.1100	*L. major*	202	2068	2269
LmjF22.1100	*L. major*	109	2321	2429
LmjF22.1110	*L. major*	70	116	185
LmjF22.1110	*L. major*	64	293	356
LmjF22.1110	*L. major*	93	1033	1125
LmjF22.1110	*L. major*	41	1443	1483
LmjF22.1110	*L. major*	91	2994	3084
LmjF22.1110	*L. major*	62	3716	3777
LmjF22.1110	*L. major*	100	3950	4049
LmjF22.1110	*L. major*	50	4688	4737
LmjF22.1110	*L. major*	45	5465	5509
LmjF22.1120	*L. major*	53	71	123

LmjF22.1150	*L. major*	59	1	59
LmjF22.1150	*L. major*	283	738	1020
LmjF22.1160	*L. major*	77	85	161
LmjF22.1170	*L. major*	129	118	246
LmjF22.1170	*L. major*	156	431	586
LmjF22.1170	*L. major*	43	615	657
LmjF22.1170	*L. major*	240	660	899
LmjF22.1180	*L. major*	316	264	579
LmjF22.1200	*L. major*	58	1	58
LmjF22.1200	*L. major*	40	174	213
LmjF22.1210	*L. major*	162	97	258
LmjF22.1210	*L. major*	458	268	725
LmjF22.1210	*L. major*	50	801	850
LmjF22.1210	*L. major*	47	1041	1087
LmjF22.1210	*L. major*	171	1126	1296
LmjF22.1210	*L. major*	48	1412	1459
LmjF22.1210	*L. major*	162	1522	1683
LmjF22.1225	*L. major*	203	1	203
LmjF22.1230	*L. major*	60	1	60
LmjF22.1230	*L. major*	68	227	294
LmjF22.1230	*L. major*	59	812	870
LmjF22.1240	*L. major*	62	1	62
LmjF22.1240	*L. major*	49	96	144
LmjF22.1240	*L. major*	62	714	775
LmjF22.1250	*L. major*	88	244	331
LmjF22.1260	*L. major*	324	146	469
LmjF22.1260	*L. major*	42	477	518
LmjF22.1260	*L. major*	50	529	578
LmjF22.1260	*L. major*	60	609	668
LmjF22.1260	*L. major*	325	713	1037
LmjF22.1270	*L. major*	73	81	153
LmjF22.1270	*L. major*	61	307	367
LmjF22.1280	*L. major*	42	123	164
LmjF22.1280	*L. major*	65	189	253
LmjF22.1280	*L. major*	65	189	253
LmjF22.1290	*L. major*	76	1	76
LmjF22.1300	*L. major*	113	1	113
LmjF22.1310	*L. major*	454	1	454
LmjF22.1310	*L. major*	112	472	583
LmjF22.1310	*L. major*	236	599	834
LmjF22.1310	*L. major*	130	846	975
LmjF22.1310	*L. major*	49	1038	1086
LmjF22.1310	*L. major*	48	1094	1141
LmjF22.1320	*L. major*	68	1	68
LmjF22.1320	*L. major*	62	138	199
LmjF22.1320	*L. major*	96	415	510
LmjF22.1320	*L. major*	277	536	812

LmjF22.1320	*L. major*	53	916	968
LmjF22.1320	*L. major*	686	1073	1758
LmjF22.1320	*L. major*	79	1799	1877
LmjF22.1320	*L. major*	80	1882	1961
LmjF22.1320	*L. major*	67	1980	2046
LmjF22.1330	*L. major*	408	1	408
LmjF22.1330	*L. major*	1062	412	1473
LmjF22.1330	*L. major*	90	1640	1729
LmjF22.1330	*L. major*	67	1731	1797
LmjF22.1330	*L. major*	50	1829	1878
LmjF22.1330	*L. major*	99	1911	2009
LmjF22.1350	*L. major*	142	254	395
LmjF22.1370	*L. major*	108	35	142
LmjF22.1370	*L. major*	249	180	428
LmjF22.1380	*L. major*	148	120	267
LmjF22.1390	*L. major*	65	1	65
LmjF22.1390	*L. major*	42	149	190
LmjF22.1390	*L. major*	42	149	190
LmjF22.1410	*L. major*	56	94	149
LmjF22.1420	*L. major*	51	1	51
LmjF22.1440	*L. major*	52	1	52
LmjF22.1440	*L. major*	65	84	148
LmjF22.1440	*L. major*	72	396	467
LmjF22.1460	*L. major*	57	150	206
LmjF22.1470	*L. major*	40	155	194
LmjF22.1470	*L. major*	83	197	279
LmjF22.1470	*L. major*	83	197	279
LmjF22.1480	*L. major*	41	11	51
LmjF22.1500	*L. major*	52	1036	1087
LmjF22.1510	*L. major*	157	64	220
LmjF22.1520	*L. major*	44	68	111
LmjF22.1520	*L. major*	51	125	175
LmjF22.1520	*L. major*	51	125	175
LmjF22.1540	*L. major*	43	920	962
LmjF22.1550	*L. major*	53	392	444
LmjF22.1560	*L. major*	44	68	111
LmjF22.1560	*L. major*	51	125	175
LmjF22.1560	*L. major*	51	125	175
LmjF22.1570	*L. major*	140	17	156
LmjF22.1570	*L. major*	82	178	259
LmjF22.1570	*L. major*	82	284	365
LmjF22.1580	*L. major*	41	219	259
LmjF22.1590	*L. major*	78	234	311
LmjF22.1600	*L. major*	312	1	312
LmjF22.1600	*L. major*	122	563	684
LmjF22.1610	*L. major*	88	360	447
LmjF22.1620	*L. major*	59	1	59

LmjF22.1620	*L. major*	58	476	533
LmjF22.1620	*L. major*	52	1088	1139
LmjF22.1620	*L. major*	82	1416	1497
LmjF22.1620	*L. major*	53	1771	1823
LmjF22.1630	*L. major*	54	285	338
LmjF22.1660	*L. major*	127	33	159
LmjF22.1660	*L. major*	80	493	572
LmjF22.1660	*L. major*	80	493	572
LmjF22.1680	*L. major*	110	319	428
LmjF22.1690	*L. major*	509	117	625
LmjF22.1690	*L. major*	389	661	1049
LmjF22.1690	*L. major*	794	1197	1990
LmjF22.1690	*L. major*	114	2009	2122
LmjF22.1690	*L. major*	55	2145	2199
LmjF22.1700	*L. major*	114	2739	2852
LmjF23.0020	*L. major*	76	183	258
LmjF23.0050	*L. major*	124	172	295
LmjF23.0060	*L. major*	149	25	173
LmjF23.0080	*L. major*	238	1	238
LmjF23.0080	*L. major*	45	248	292
LmjF23.0080	*L. major*	57	363	419
LmjF23.0090	*L. major*	138	209	346
LmjF23.0120	*L. major*	49	1411	1459
LmjF23.0120	*L. major*	68	1523	1590
LmjF23.0120	*L. major*	68	1523	1590
LmjF23.0160	*L. major*	48	34	81
LmjF23.0170	*L. major*	76	85	160
LmjF23.0190	*L. major*	125	1	125
LmjF23.0210	*L. major*	42	1	42
LmjF23.0210	*L. major*	42	172	213
LmjF23.0210	*L. major*	61	606	666
LmjF23.0210	*L. major*	67	875	941
LmjF23.0220	*L. major*	50	1	50
LmjF23.0220	*L. major*	66	615	680
LmjF23.0220	*L. major*	55	887	941
LmjF23.0223	*L. major*	60	1	60
LmjF23.0225	*L. major*	159	33	191
LmjF23.0230	*L. major*	44	1	44
LmjF23.0230	*L. major*	40	122	161
LmjF23.0230	*L. major*	40	122	161
LmjF23.0250	*L. major*	45	625	669
LmjF23.0250	*L. major*	53	881	933
LmjF23.0250	*L. major*	53	881	933
LmjF23.0280	*L. major*	195	1	195
LmjF23.0280	*L. major*	142	294	435
LmjF23.0280	*L. major*	85	511	595
LmjF23.0280	*L. major*	97	612	708

LmjF23.0280	*L. major*	49	765	813
LmjF23.0280	*L. major*	63	1038	1100
LmjF23.0300	*L. major*	64	1	64
LmjF23.0310	*L. major*	91	11	101
LmjF23.0310	*L. major*	69	198	266
LmjF23.0310	*L. major*	77	346	422
LmjF23.0310	*L. major*	44	508	551
LmjF23.0310	*L. major*	76	750	825
LmjF23.0310	*L. major*	77	946	1022
LmjF23.0320	*L. major*	41	1	41
LmjF23.0320	*L. major*	42	77	118
LmjF23.0320	*L. major*	61	146	206
LmjF23.0340	*L. major*	97	1	97
LmjF23.0350	*L. major*	55	1	55
LmjF23.0350	*L. major*	112	183	294
LmjF23.0355	*L. major*	54	114	167
LmjF23.0380	*L. major*	101	475	575
LmjF23.0380	*L. major*	70	871	940
LmjF23.0390	*L. major*	147	39	185
LmjF23.0390	*L. major*	59	208	266
LmjF23.0390	*L. major*	53	462	514
LmjF23.0390	*L. major*	74	574	647
LmjF23.0390	*L. major*	180	672	851
LmjF23.0390	*L. major*	48	914	961
LmjF23.0390	*L. major*	58	1197	1254
LmjF23.0390	*L. major*	215	1271	1485
LmjF23.0390	*L. major*	94	1736	1829
LmjF23.0390	*L. major*	75	1906	1980
LmjF23.0410	*L. major*	206	1	206
LmjF23.0410	*L. major*	107	343	449
LmjF23.0410	*L. major*	52	657	708
LmjF23.0410	*L. major*	56	936	991
LmjF23.0410	*L. major*	93	1201	1293
LmjF23.0420	*L. major*	68	1	68
LmjF23.0440	*L. major*	63	146	208
LmjF23.0460	*L. major*	71	1	71
LmjF23.0460	*L. major*	46	153	198
LmjF23.0470	*L. major*	187	1	187
LmjF23.0470	*L. major*	56	220	275
LmjF23.0470	*L. major*	70	280	349
LmjF23.0480	*L. major*	637	1	637
LmjF23.0480	*L. major*	139	647	785
LmjF23.0480	*L. major*	66	824	889
LmjF23.0480	*L. major*	207	930	1136
LmjF23.0480	*L. major*	47	1348	1394
LmjF23.0490	*L. major*	41	101	141
LmjF23.0530	*L. major*	68	218	285

LmjF23.0540	*L. major*	60	1	60
LmjF23.0542	*L. major*	87	84	170
LmjF23.0543	*L. major*	93	264	356
LmjF23.0543	*L. major*	50	474	523
LmjF23.0543	*L. major*	103	750	852
LmjF23.0543	*L. major*	135	934	1068
LmjF23.0547	*L. major*	70	281	350
LmjF23.0547	*L. major*	303	436	738
LmjF23.0560	*L. major*	49	402	450
LmjF23.0560	*L. major*	173	484	656
LmjF23.0560	*L. major*	173	484	656
LmjF23.0570	*L. major*	92	1	92
LmjF23.0570	*L. major*	68	174	241
LmjF23.0570	*L. major*	210	265	474
LmjF23.0570	*L. major*	48	507	554
LmjF23.0570	*L. major*	64	737	800
LmjF23.0570	*L. major*	123	834	956
LmjF23.0580	*L. major*	185	26	210
LmjF23.0580	*L. major*	95	310	404
LmjF23.0580	*L. major*	49	788	836
LmjF23.0580	*L. major*	101	863	963
LmjF23.0580	*L. major*	85	1017	1101
LmjF23.0590	*L. major*	90	89	178
LmjF23.0590	*L. major*	220	192	411
LmjF23.0600	*L. major*	134	128	261
LmjF23.0610	*L. major*	66	161	226
LmjF23.0610	*L. major*	80	247	326
LmjF23.0610	*L. major*	227	382	608
LmjF23.0620	*L. major*	89	1	89
LmjF23.0620	*L. major*	217	464	680
LmjF23.0630	*L. major*	151	36	186
LmjF23.0630	*L. major*	156	285	440
LmjF23.0630	*L. major*	68	988	1055
LmjF23.0640	*L. major*	181	33	213
LmjF23.0640	*L. major*	41	515	555
LmjF23.0650	*L. major*	58	37	94
LmjF23.0660	*L. major*	83	1	83
LmjF23.0660	*L. major*	49	182	230
LmjF23.0660	*L. major*	56	245	300
LmjF23.0660	*L. major*	136	380	515
LmjF23.0660	*L. major*	131	667	797
LmjF23.0660	*L. major*	54	803	856
LmjF23.0680	*L. major*	115	1	115
LmjF23.0680	*L. major*	65	468	532
LmjF23.0710	*L. major*	50	1	50
LmjF23.0720	*L. major*	55	1	55
LmjF23.0720	*L. major*	72	135	206

LmjF23.0720	*L. major*	280	286	565
LmjF23.0730	*L. major*	49	1	49
LmjF23.0730	*L. major*	73	56	128
LmjF23.0730	*L. major*	273	205	477
LmjF23.0730	*L. major*	107	493	599
LmjF23.0740	*L. major*	47	1	47
LmjF23.0740	*L. major*	61	124	184
LmjF23.0740	*L. major*	59	255	313
LmjF23.0750	*L. major*	41	88	128
LmjF23.0750	*L. major*	121	285	405
LmjF23.0750	*L. major*	57	453	509
LmjF23.0750	*L. major*	66	866	931
LmjF23.0770	*L. major*	308	13	320
LmjF23.0770	*L. major*	210	325	534
LmjF23.0770	*L. major*	204	573	776
LmjF23.0770	*L. major*	57	786	842
LmjF23.0770	*L. major*	130	973	1102
LmjF23.0770	*L. major*	57	1258	1314
LmjF23.0780	*L. major*	167	92	258
LmjF23.0780	*L. major*	76	534	609
LmjF23.0780	*L. major*	55	613	667
LmjF23.0780	*L. major*	168	705	872
LmjF23.0790	*L. major*	77	51	127
LmjF23.0790	*L. major*	122	165	286
LmjF23.0790	*L. major*	75	397	471
LmjF23.0790	*L. major*	76	495	570
LmjF23.0790	*L. major*	105	752	856
LmjF23.0800	*L. major*	89	1	89
LmjF23.0820	*L. major*	117	116	232
LmjF23.0830	*L. major*	114	560	673
LmjF23.0830	*L. major*	128	1091	1218
LmjF23.0850	*L. major*	67	95	161
LmjF23.0850	*L. major*	82	245	326
LmjF23.0850	*L. major*	110	469	578
LmjF23.0850	*L. major*	100	677	776
LmjF23.0900	*L. major*	64	1	64
LmjF23.0900	*L. major*	161	130	290
LmjF23.0900	*L. major*	109	410	518
LmjF23.0900	*L. major*	468	642	1109
LmjF23.0900	*L. major*	209	1117	1325
LmjF23.0900	*L. major*	111	1362	1472
LmjF23.0900	*L. major*	73	1486	1558
LmjF23.0900	*L. major*	68	1586	1653
LmjF23.0900	*L. major*	69	1703	1771
LmjF23.0900	*L. major*	107	1942	2048
LmjF23.0900	*L. major*	64	2081	2144
LmjF23.0900	*L. major*	71	2218	2288

LmjF23.0900	*L. major*	78	2485	2562
LmjF23.0900	*L. major*	166	2566	2731
LmjF23.0900	*L. major*	105	2772	2876
LmjF23.0910	*L. major*	113	57	169
LmjF23.0910	*L. major*	56	301	356
LmjF23.0910	*L. major*	117	369	485
LmjF23.0910	*L. major*	63	853	915
LmjF23.0910	*L. major*	127	1024	1150
LmjF23.0910	*L. major*	116	1154	1269
LmjF23.0950	*L. major*	52	182	233
LmjF23.0960	*L. major*	118	208	325
LmjF23.0970	*L. major*	122	99	220
LmjF23.0970	*L. major*	91	298	388
LmjF23.0980	*L. major*	68	1	68
LmjF23.0980	*L. major*	57	189	245
LmjF23.0990	*L. major*	60	138	197
LmjF23.0990	*L. major*	143	280	422
LmjF23.0990	*L. major*	225	454	678
LmjF23.0990	*L. major*	70	680	749
LmjF23.1000	*L. major*	50	51	100
LmjF23.1000	*L. major*	68	116	183
LmjF23.1000	*L. major*	68	116	183
LmjF23.1010	*L. major*	43	77	119
LmjF23.1010	*L. major*	59	219	277
LmjF23.1010	*L. major*	50	309	358
LmjF23.1010	*L. major*	44	513	556
LmjF23.1010	*L. major*	46	626	671
LmjF23.1010	*L. major*	41	757	797
LmjF23.1010	*L. major*	309	802	1110
LmjF23.1020	*L. major*	150	1	150
LmjF23.1025	*L. major*	46	217	262
LmjF23.1025	*L. major*	232	326	557
LmjF23.1035	*L. major*	112	9	120
LmjF23.1035	*L. major*	78	133	210
LmjF23.1035	*L. major*	180	233	412
LmjF23.1035	*L. major*	81	421	501
LmjF23.1035	*L. major*	44	534	577
LmjF23.1045	*L. major*	42	262	303
LmjF23.1055	*L. major*	50	1	50
LmjF23.1055	*L. major*	58	94	151
LmjF23.1055	*L. major*	56	296	351
LmjF23.1060	*L. major*	177	1	177
LmjF23.1065	*L. major*	153	270	422
LmjF23.1070	*L. major*	177	1	177
LmjF23.1090	*L. major*	51	1	51
LmjF23.1090	*L. major*	75	135	209
LmjF23.1090	*L. major*	47	231	277

LmjF23.1090	*L. major*	49	312	360
LmjF23.1090	*L. major*	45	488	532
LmjF23.1100	*L. major*	40	251	290
LmjF23.1110	*L. major*	61	518	578
LmjF23.1120	*L. major*	88	250	337
LmjF23.1130	*L. major*	138	177	314
LmjF23.1130	*L. major*	72	338	409
LmjF23.1140	*L. major*	169	22	190
LmjF23.1140	*L. major*	208	204	411
LmjF23.1140	*L. major*	143	413	555
LmjF23.1140	*L. major*	52	644	695
LmjF23.1140	*L. major*	107	755	861
LmjF23.1140	*L. major*	469	881	1349
LmjF23.1150	*L. major*	142	13	154
LmjF23.1150	*L. major*	273	178	450
LmjF23.1150	*L. major*	46	502	547
LmjF23.1150	*L. major*	43	566	608
LmjF23.1155	*L. major*	82	31	112
LmjF23.1155	*L. major*	44	121	164
LmjF23.1155	*L. major*	137	238	374
LmjF23.1155	*L. major*	55	391	445
LmjF23.1165	*L. major*	141	361	501
LmjF23.1200	*L. major*	74	47	120
LmjF23.1200	*L. major*	42	199	240
LmjF23.1240	*L. major*	141	58	198
LmjF23.1240	*L. major*	40	262	301
LmjF23.1240	*L. major*	46	309	354
LmjF23.1240	*L. major*	269	450	718
LmjF23.1250	*L. major*	192	1	192
LmjF23.1250	*L. major*	203	233	435
LmjF23.1250	*L. major*	52	558	609
LmjF23.1250	*L. major*	44	646	689
LmjF23.1250	*L. major*	40	817	856
LmjF23.1250	*L. major*	192	932	1123
LmjF23.1250	*L. major*	82	1271	1352
LmjF23.1260	*L. major*	56	105	160
LmjF23.1260	*L. major*	40	1019	1058
LmjF23.1260	*L. major*	134	1121	1254
LmjF23.1260	*L. major*	41	1256	1296
LmjF23.1263	*L. major*	269	1	269
LmjF23.1267	*L. major*	300	83	382
LmjF23.1270	*L. major*	77	283	359
LmjF23.1270	*L. major*	300	528	827
LmjF23.1270	*L. major*	182	832	1013
LmjF23.1280	*L. major*	103	1	103
LmjF23.1280	*L. major*	218	404	621
LmjF23.1280	*L. major*	87	653	739

LmjF23.1280	*L. major*	43	764	806
LmjF23.1280	*L. major*	54	930	983
LmjF23.1280	*L. major*	43	1028	1070
LmjF23.1290	*L. major*	351	1	351
LmjF23.1290	*L. major*	243	353	595
LmjF23.1290	*L. major*	46	611	656
LmjF23.1310	*L. major*	71	79	149
LmjF23.1310	*L. major*	108	3314	3421
LmjF23.1310	*L. major*	59	3527	3585
LmjF23.1310	*L. major*	75	3824	3898
LmjF23.1330	*L. major*	125	62	186
LmjF23.1330	*L. major*	116	346	461
LmjF23.1330	*L. major*	89	553	641
LmjF23.1330	*L. major*	329	663	991
LmjF23.1330	*L. major*	202	1249	1450
LmjF23.1330	*L. major*	204	1468	1671
LmjF23.1330	*L. major*	64	1706	1769
LmjF23.1330	*L. major*	203	1918	2120
LmjF23.1330	*L. major*	157	2726	2882
LmjF23.1330	*L. major*	53	2933	2985
LmjF23.1340	*L. major*	51	38	88
LmjF23.1340	*L. major*	60	139	198
LmjF23.1350	*L. major*	164	1	164
LmjF23.1360	*L. major*	82	1	82
LmjF23.1360	*L. major*	155	261	415
LmjF23.1370	*L. major*	52	1	52
LmjF23.1380	*L. major*	661	1	661
LmjF23.1380	*L. major*	183	663	845
LmjF23.1380	*L. major*	45	1030	1074
LmjF23.1380	*L. major*	86	1417	1502
LmjF23.1380	*L. major*	88	1522	1609
LmjF23.1380	*L. major*	58	1808	1865
LmjF23.1405	*L. major*	45	286	330
LmjF23.1430	*L. major*	75	93	167
LmjF23.1430	*L. major*	75	376	450
LmjF23.1430	*L. major*	75	376	450
LmjF23.1450	*L. major*	52	1	52
LmjF23.1450	*L. major*	420	71	490
LmjF23.1450	*L. major*	420	71	490
LmjF23.1470	*L. major*	73	148	220
LmjF23.1500	*L. major*	42	259	300
LmjF23.1510	*L. major*	46	61	106
LmjF23.1520	*L. major*	44	77	120
LmjF23.1530	*L. major*	67	107	173
LmjF23.1550	*L. major*	41	1	41
LmjF23.1560	*L. major*	66	63	128
LmjF23.1590	*L. major*	43	231	273

LmjF23.1610	*L. major*	43	1	43
LmjF23.1610	*L. major*	226	126	351
LmjF23.1620	*L. major*	118	94	211
LmjF23.1630	*L. major*	110	159	268
LmjF23.1640	*L. major*	46	111	156
LmjF23.1640	*L. major*	95	177	271
LmjF23.1640	*L. major*	196	286	481
LmjF23.1640	*L. major*	42	545	586
LmjF23.1640	*L. major*	44	601	644
LmjF23.1640	*L. major*	43	691	733
LmjF23.1640	*L. major*	75	812	886
LmjF23.1640	*L. major*	132	946	1077
LmjF23.1640	*L. major*	71	1102	1172
LmjF23.1640	*L. major*	56	1536	1591
LmjF23.1640	*L. major*	48	1593	1640
LmjF23.1640	*L. major*	61	1722	1782
LmjF23.1640	*L. major*	114	2104	2217
LmjF23.1640	*L. major*	137	2227	2363
LmjF23.1640	*L. major*	970	2371	3340
LmjF23.1660	*L. major*	57	210	266
LmjF23.1670	*L. major*	221	148	368
LmjF23.1670	*L. major*	76	561	636
LmjF23.1680	*L. major*	114	1	114
LmjF23.1680	*L. major*	203	133	335
LmjF23.1690	*L. major*	63	1	63
LmjF23.1710	*L. major*	96	69	164
LmjF23.1710	*L. major*	53	201	253
LmjF23.1710	*L. major*	90	382	471
LmjF23.1715	*L. major*	57	74	130
LmjF23.1720	*L. major*	42	97	138
LmjF23.1730	*L. major*	97	1	97
LmjF23.1730	*L. major*	149	108	256
LmjF23.1730	*L. major*	46	315	360
LmjF23.1740	*L. major*	50	27	76
LmjF23.1740	*L. major*	81	128	208
LmjF23.1740	*L. major*	81	128	208
LmjF23.1750	*L. major*	44	1	44
LmjF23.1750	*L. major*	76	128	203
LmjF23.1750	*L. major*	76	128	203
LmjF23.1760	*L. major*	91	1	91
LmjF23.1760	*L. major*	131	134	264
LmjF23.1760	*L. major*	58	346	403
LmjF23.1760	*L. major*	90	409	498
LmjF23.1760	*L. major*	46	544	589
LmjF24.0010	*L. major*	43	69	111
LmjF24.0020	*L. major*	202	282	483
LmjF24.0030	*L. major*	49	118	166

LmjF24.0050	*L. major*	57	55	111
LmjF24.0060	*L. major*	56	359	414
LmjF24.0070	*L. major*	92	204	295
LmjF24.0070	*L. major*	53	387	439
LmjF24.0100	*L. major*	211	12	222
LmjF24.0140	*L. major*	55	236	290
LmjF24.0160	*L. major*	136	1	136
LmjF24.0160	*L. major*	44	143	186
LmjF24.0160	*L. major*	42	237	278
LmjF24.0190	*L. major*	112	1	112
LmjF24.0190	*L. major*	137	677	813
LmjF24.0200	*L. major*	100	1	100
LmjF24.0200	*L. major*	55	123	177
LmjF24.0200	*L. major*	51	255	305
LmjF24.0210	*L. major*	169	1	169
LmjF24.0210	*L. major*	67	250	316
LmjF24.0220	*L. major*	67	117	183
LmjF24.0230	*L. major*	77	1	77
LmjF24.0230	*L. major*	40	335	374
LmjF24.0240	*L. major*	50	175	224
LmjF24.0250	*L. major*	51	347	397
LmjF24.0250	*L. major*	43	501	543
LmjF24.0250	*L. major*	46	560	605
LmjF24.0260	*L. major*	79	38	116
LmjF24.0260	*L. major*	55	240	294
LmjF24.0260	*L. major*	41	786	826
LmjF24.0260	*L. major*	63	1295	1357
LmjF24.0260	*L. major*	45	1594	1638
LmjF24.0270	*L. major*	222	65	286
LmjF24.0270	*L. major*	108	352	459
LmjF24.0270	*L. major*	77	752	828
LmjF24.0270	*L. major*	215	881	1095
LmjF24.0280	*L. major*	73	106	178
LmjF24.0280	*L. major*	53	186	238
LmjF24.0280	*L. major*	53	186	238
LmjF24.0300	*L. major*	54	485	538
LmjF24.0300	*L. major*	98	693	790
LmjF24.0310	*L. major*	108	1	108
LmjF24.0310	*L. major*	62	121	182
LmjF24.0320	*L. major*	49	409	457
LmjF24.0330	*L. major*	89	1	89
LmjF24.0330	*L. major*	57	170	226
LmjF24.0330	*L. major*	60	329	388
LmjF24.0330	*L. major*	90	393	482
LmjF24.0330	*L. major*	97	658	754
LmjF24.0330	*L. major*	126	915	1040
LmjF24.0350	*L. major*	67	197	263

LmjF24.0360	*L. major*	123	29	151
LmjF24.0380	*L. major*	75	1	75
LmjF24.0380	*L. major*	130	302	431
LmjF24.0380	*L. major*	524	484	1007
LmjF24.0390	*L. major*	46	231	276
LmjF24.0390	*L. major*	76	297	372
LmjF24.0390	*L. major*	41	485	525
LmjF24.0410	*L. major*	267	95	361
LmjF24.0430	*L. major*	95	45	139
LmjF24.0430	*L. major*	147	162	308
LmjF24.0430	*L. major*	48	613	660
LmjF24.0430	*L. major*	52	1152	1203
LmjF24.0440	*L. major*	86	134	219
LmjF24.0440	*L. major*	46	423	468
LmjF24.0440	*L. major*	80	689	768
LmjF24.0440	*L. major*	40	1281	1320
LmjF24.0450	*L. major*	86	129	214
LmjF24.0450	*L. major*	46	418	463
LmjF24.0450	*L. major*	83	681	763
LmjF24.0470	*L. major*	232	189	420
LmjF24.0480	*L. major*	103	1	103
LmjF24.0480	*L. major*	75	143	217
LmjF24.0480	*L. major*	136	272	407
LmjF24.0480	*L. major*	233	711	943
LmjF24.0480	*L. major*	233	957	1189
LmjF24.0490	*L. major*	77	1	77
LmjF24.0490	*L. major*	63	141	203
LmjF24.0490	*L. major*	61	888	948
LmjF24.0490	*L. major*	76	970	1045
LmjF24.0490	*L. major*	68	1265	1332
LmjF24.0490	*L. major*	51	1626	1676
LmjF24.0490	*L. major*	67	1872	1938
LmjF24.0500	*L. major*	89	1	89
LmjF24.0500	*L. major*	136	99	234
LmjF24.0510	*L. major*	132	1	132
LmjF24.0510	*L. major*	53	423	475
LmjF24.0510	*L. major*	110	508	617
LmjF24.0520	*L. major*	57	624	680
LmjF24.0530	*L. major*	83	81	163
LmjF24.0530	*L. major*	60	168	227
LmjF24.0530	*L. major*	60	168	227
LmjF24.0540	*L. major*	63	341	403
LmjF24.0550	*L. major*	77	30	106
LmjF24.0552	*L. major*	50	81	130
LmjF24.0600	*L. major*	68	820	887
LmjF24.0600	*L. major*	41	1060	1100
LmjF24.0600	*L. major*	41	1189	1229

LmjF24.0600	*L. major*	85	1705	1789
LmjF24.0610	*L. major*	58	385	442
LmjF24.0610	*L. major*	322	627	948
LmjF24.0610	*L. major*	70	960	1029
LmjF24.0620	*L. major*	56	45	100
LmjF24.0620	*L. major*	316	494	809
LmjF24.0640	*L. major*	114	322	435
LmjF24.0640	*L. major*	41	437	477
LmjF24.0650	*L. major*	758	213	970
LmjF24.0670	*L. major*	47	114	160
LmjF24.0670	*L. major*	409	333	741
LmjF24.0670	*L. major*	156	808	963
LmjF24.0670	*L. major*	160	1004	1163
LmjF24.0680	*L. major*	55	493	547
LmjF24.0690	*L. major*	65	110	174
LmjF24.0700	*L. major*	138	1	138
LmjF24.0700	*L. major*	77	278	354
LmjF24.0700	*L. major*	40	574	613
LmjF24.0710	*L. major*	56	49	104
LmjF24.0710	*L. major*	61	176	236
LmjF24.0710	*L. major*	48	290	337
LmjF24.0730	*L. major*	90	430	519
LmjF24.0740	*L. major*	88	1	88
LmjF24.0740	*L. major*	141	105	245
LmjF24.0740	*L. major*	97	272	368
LmjF24.0740	*L. major*	266	370	635
LmjF24.0740	*L. major*	148	807	954
LmjF24.0740	*L. major*	107	968	1074
LmjF24.0750	*L. major*	59	198	256
LmjF24.0760	*L. major*	52	1	52
LmjF24.0760	*L. major*	51	54	104
LmjF24.0760	*L. major*	93	121	213
LmjF24.0760	*L. major*	64	310	373
LmjF24.0760	*L. major*	52	739	790
LmjF24.0760	*L. major*	51	973	1023
LmjF24.0760	*L. major*	66	1062	1127
LmjF24.0780	*L. major*	93	1	93
LmjF24.0780	*L. major*	197	111	307
LmjF24.0790	*L. major*	223	1	223
LmjF24.0790	*L. major*	81	258	338
LmjF24.0790	*L. major*	44	394	437
LmjF24.0800	*L. major*	110	1	110
LmjF24.0800	*L. major*	132	150	281
LmjF24.0800	*L. major*	284	301	584
LmjF24.0810	*L. major*	52	1	52
LmjF24.0810	*L. major*	50	188	237
LmjF24.0810	*L. major*	103	277	379

LmjF24.0820	*L. major*	54	52	105
LmjF24.0820	*L. major*	65	220	284
LmjF24.0820	*L. major*	45	322	366
LmjF24.0820	*L. major*	130	818	947
LmjF24.0820	*L. major*	68	984	1051
LmjF24.0820	*L. major*	90	1086	1175
LmjF24.0820	*L. major*	104	1429	1532
LmjF24.0820	*L. major*	101	1716	1816
LmjF24.0820	*L. major*	41	2023	2063
LmjF24.0820	*L. major*	50	2203	2252
LmjF24.0820	*L. major*	102	2270	2371
LmjF24.0820	*L. major*	99	2575	2673
LmjF24.0830	*L. major*	62	317	378
LmjF24.0830	*L. major*	106	706	811
LmjF24.0830	*L. major*	106	706	811
LmjF24.0840	*L. major*	60	1	60
LmjF24.0840	*L. major*	58	159	216
LmjF24.0840	*L. major*	58	159	216
LmjF24.0860	*L. major*	40	1	40
LmjF24.0860	*L. major*	48	153	200
LmjF24.0860	*L. major*	54	235	288
LmjF24.0890	*L. major*	190	1	190
LmjF24.0890	*L. major*	151	391	541
LmjF24.0890	*L. major*	48	825	872
LmjF24.0890	*L. major*	81	992	1072
LmjF24.0900	*L. major*	77	26	102
LmjF24.0900	*L. major*	633	137	769
LmjF24.0910	*L. major*	46	181	226
LmjF24.0910	*L. major*	48	556	603
LmjF24.0920	*L. major*	89	1	89
LmjF24.0920	*L. major*	42	221	262
LmjF24.0920	*L. major*	58	398	455
LmjF24.0930	*L. major*	42	105	146
LmjF24.0930	*L. major*	87	515	601
LmjF24.0930	*L. major*	65	638	702
LmjF24.0950	*L. major*	97	71	167
LmjF24.0960	*L. major*	103	1	103
LmjF24.0960	*L. major*	82	200	281
LmjF24.0960	*L. major*	692	291	982
LmjF24.0970	*L. major*	45	54	98
LmjF24.0970	*L. major*	50	275	324
LmjF24.0970	*L. major*	59	583	641
LmjF24.0980	*L. major*	282	45	326
LmjF24.0980	*L. major*	949	332	1280
LmjF24.0980	*L. major*	62	1292	1353
LmjF24.0980	*L. major*	216	1373	1588
LmjF24.0990	*L. major*	170	492	661

LmjF24.1000	*L. major*	318	1	318
LmjF24.1000	*L. major*	167	343	509
LmjF24.1010	*L. major*	65	84	148
LmjF24.1010	*L. major*	81	159	239
LmjF24.1010	*L. major*	135	293	427
LmjF24.1020	*L. major*	73	115	187
LmjF24.1020	*L. major*	82	329	410
LmjF24.1020	*L. major*	55	450	504
LmjF24.1020	*L. major*	175	569	743
LmjF24.1020	*L. major*	73	853	925
LmjF24.1020	*L. major*	53	927	979
LmjF24.1040	*L. major*	69	39	107
LmjF24.1040	*L. major*	124	110	233
LmjF24.1040	*L. major*	128	329	456
LmjF24.1040	*L. major*	114	590	703
LmjF24.1040	*L. major*	110	771	880
LmjF24.1050	*L. major*	179	1	179
LmjF24.1050	*L. major*	67	396	462
LmjF24.1050	*L. major*	139	635	773
LmjF24.1050	*L. major*	63	1043	1105
LmjF24.1050	*L. major*	86	1189	1274
LmjF24.1060	*L. major*	73	1	73
LmjF24.1060	*L. major*	96	222	317
LmjF24.1070	*L. major*	83	1	83
LmjF24.1070	*L. major*	52	256	307
LmjF24.1070	*L. major*	79	349	427
LmjF24.1070	*L. major*	163	625	787
LmjF24.1070	*L. major*	178	1476	1653
LmjF24.1070	*L. major*	95	1682	1776
LmjF24.1070	*L. major*	137	2085	2221
LmjF24.1070	*L. major*	77	2396	2472
LmjF24.1070	*L. major*	53	2475	2527
LmjF24.1080	*L. major*	85	298	382
LmjF24.1090	*L. major*	62	255	316
LmjF24.1090	*L. major*	41	478	518
LmjF24.1090	*L. major*	41	478	518
LmjF24.1100	*L. major*	95	1	95
LmjF24.1100	*L. major*	123	102	224
LmjF24.1100	*L. major*	182	249	430
LmjF24.1100	*L. major*	80	1059	1138
LmjF24.1110	*L. major*	67	304	370
LmjF24.1110	*L. major*	305	419	723
LmjF24.1110	*L. major*	58	966	1023
LmjF24.1110	*L. major*	56	1138	1193
LmjF24.1120	*L. major*	59	1	59
LmjF24.1120	*L. major*	42	184	225
LmjF24.1120	*L. major*	70	251	320

LmjF24.1120	*L. major*	107	322	428
LmjF24.1120	*L. major*	101	513	613
LmjF24.1120	*L. major*	124	789	912
LmjF24.1125	*L. major*	224	1	224
LmjF24.1125	*L. major*	147	248	394
LmjF24.1125	*L. major*	44	607	650
LmjF24.1130	*L. major*	318	33	350
LmjF24.1130	*L. major*	218	391	608
LmjF24.1130	*L. major*	101	617	717
LmjF24.1130	*L. major*	131	744	874
LmjF24.1130	*L. major*	101	886	986
LmjF24.1140	*L. major*	76	70	145
LmjF24.1140	*L. major*	41	153	193
LmjF24.1140	*L. major*	42	266	307
LmjF24.1140	*L. major*	63	487	549
LmjF24.1150	*L. major*	69	1	69
LmjF24.1150	*L. major*	255	97	351
LmjF24.1160	*L. major*	142	1	142
LmjF24.1160	*L. major*	124	157	280
LmjF24.1160	*L. major*	173	342	514
LmjF24.1160	*L. major*	113	522	634
LmjF24.1160	*L. major*	383	665	1047
LmjF24.1170	*L. major*	42	132	173
LmjF24.1170	*L. major*	41	226	266
LmjF24.1170	*L. major*	40	602	641
LmjF24.1170	*L. major*	72	1068	1139
LmjF24.1170	*L. major*	61	1190	1250
LmjF24.1180	*L. major*	69	1	69
LmjF24.1180	*L. major*	168	85	252
LmjF24.1190	*L. major*	104	1	104
LmjF24.1200	*L. major*	276	1	276
LmjF24.1220	*L. major*	109	24	132
LmjF24.1220	*L. major*	105	173	277
LmjF24.1220	*L. major*	50	388	437
LmjF24.1220	*L. major*	74	470	543
LmjF24.1230	*L. major*	232	1	232
LmjF24.1230	*L. major*	78	277	354
LmjF24.1230	*L. major*	77	357	433
LmjF24.1230	*L. major*	85	678	762
LmjF24.1240	*L. major*	52	53	104
LmjF24.1240	*L. major*	46	205	250
LmjF24.1240	*L. major*	46	205	250
LmjF24.1290	*L. major*	86	1	86
LmjF24.1290	*L. major*	89	142	230
LmjF24.1290	*L. major*	95	326	420
LmjF24.1290	*L. major*	106	1119	1224
LmjF24.1290	*L. major*	61	1364	1424

LmjF24.1300	*L. major*	86	68	153
LmjF24.1340	*L. major*	64	436	499
LmjF24.1340	*L. major*	81	621	701
LmjF24.1340	*L. major*	61	832	892
LmjF24.1350	*L. major*	66	304	369
LmjF24.1350	*L. major*	84	805	888
LmjF24.1360	*L. major*	43	1	43
LmjF24.1360	*L. major*	94	377	470
LmjF24.1360	*L. major*	94	377	470
LmjF24.1370	*L. major*	80	1	80
LmjF24.1370	*L. major*	50	487	536
LmjF24.1380	*L. major*	142	81	222
LmjF24.1390	*L. major*	72	77	148
LmjF24.1400	*L. major*	162	1	162
LmjF24.1410	*L. major*	49	98	146
LmjF24.1420	*L. major*	53	218	270
LmjF24.1420	*L. major*	78	296	373
LmjF24.1420	*L. major*	63	503	565
LmjF24.1420	*L. major*	271	589	859
LmjF24.1420	*L. major*	59	872	930
LmjF24.1420	*L. major*	128	1192	1319
LmjF24.1430	*L. major*	327	1	327
LmjF24.1430	*L. major*	235	359	593
LmjF24.1430	*L. major*	413	647	1059
LmjF24.1430	*L. major*	105	1100	1204
LmjF24.1430	*L. major*	61	1207	1267
LmjF24.1430	*L. major*	45	1360	1404
LmjF24.1430	*L. major*	251	1630	1880
LmjF24.1430	*L. major*	237	1965	2201
LmjF24.1430	*L. major*	107	2207	2313
LmjF24.1430	*L. major*	49	2346	2394
LmjF24.1430	*L. major*	117	2473	2589
LmjF24.1430	*L. major*	89	2620	2708
LmjF24.1430	*L. major*	119	2725	2843
LmjF24.1430	*L. major*	48	2862	2909
LmjF24.1430	*L. major*	51	2962	3012
LmjF24.1430	*L. major*	46	3088	3133
LmjF24.1430	*L. major*	65	3170	3234
LmjF24.1435	*L. major*	724	1	724
LmjF24.1435	*L. major*	85	730	814
LmjF24.1435	*L. major*	67	851	917
LmjF24.1440	*L. major*	49	30	78
LmjF24.1440	*L. major*	59	103	161
LmjF24.1440	*L. major*	61	478	538
LmjF24.1450	*L. major*	295	23	317
LmjF24.1460	*L. major*	84	1	84
LmjF24.1460	*L. major*	158	110	267

LmjF24.1460	*L. major*	48	360	407
LmjF24.1460	*L. major*	47	477	523
LmjF24.1460	*L. major*	97	589	685
LmjF24.1470	*L. major*	49	101	149
LmjF24.1470	*L. major*	168	282	449
LmjF24.1470	*L. major*	53	470	522
LmjF24.1470	*L. major*	212	587	798
LmjF24.1470	*L. major*	55	826	880
LmjF24.1470	*L. major*	83	918	1000
LmjF24.1470	*L. major*	43	1244	1286
LmjF24.1470	*L. major*	139	1486	1624
LmjF24.1470	*L. major*	61	1709	1769
LmjF24.1470	*L. major*	64	1790	1853
LmjF24.1470	*L. major*	80	2021	2100
LmjF24.1470	*L. major*	72	2119	2190
LmjF24.1470	*L. major*	155	2300	2454
LmjF24.1470	*L. major*	52	2526	2577
LmjF24.1470	*L. major*	173	2604	2776
LmjF24.1470	*L. major*	184	2867	3050
LmjF24.1480	*L. major*	227	1	227
LmjF24.1480	*L. major*	81	276	356
LmjF24.1480	*L. major*	86	539	624
LmjF24.1490	*L. major*	99	1	99
LmjF24.1520	*L. major*	229	67	295
LmjF24.1520	*L. major*	235	312	546
LmjF24.1520	*L. major*	191	596	786
LmjF24.1520	*L. major*	51	901	951
LmjF24.1530	*L. major*	177	1	177
LmjF24.1530	*L. major*	212	819	1030
LmjF24.1530	*L. major*	573	1119	1691
LmjF24.1550	*L. major*	85	73	157
LmjF24.1550	*L. major*	191	193	383
LmjF24.1550	*L. major*	61	411	471
LmjF24.1550	*L. major*	54	480	533
LmjF24.1550	*L. major*	66	731	796
LmjF24.1560	*L. major*	40	325	364
LmjF24.1560	*L. major*	78	419	496
LmjF24.1560	*L. major*	78	419	496
LmjF24.1570	*L. major*	228	1	228
LmjF24.1570	*L. major*	283	410	692
LmjF24.1580	*L. major*	64	301	364
LmjF24.1590	*L. major*	252	269	520
LmjF24.1640	*L. major*	78	318	395
LmjF24.1650	*L. major*	75	1	75
LmjF24.1650	*L. major*	78	149	226
LmjF24.1650	*L. major*	160	242	401
LmjF24.1650	*L. major*	65	420	484

LmjF24.1660	*L. major*	108	1	108
LmjF24.1660	*L. major*	44	234	277
LmjF24.1670	*L. major*	102	173	274
LmjF24.1680	*L. major*	44	84	127
LmjF24.1680	*L. major*	71	215	285
LmjF24.1680	*L. major*	41	326	366
LmjF24.1700	*L. major*	43	1	43
LmjF24.1700	*L. major*	88	149	236
LmjF24.1720	*L. major*	252	1	252
LmjF24.1720	*L. major*	89	274	362
LmjF24.1720	*L. major*	40	536	575
LmjF24.1720	*L. major*	81	758	838
LmjF24.1730	*L. major*	372	1	372
LmjF24.1730	*L. major*	77	494	570
LmjF24.1730	*L. major*	106	915	1020
LmjF24.1730	*L. major*	47	1132	1178
LmjF24.1740	*L. major*	112	94	205
LmjF24.1750	*L. major*	128	81	208
LmjF24.1750	*L. major*	42	294	335
LmjF24.1750	*L. major*	42	294	335
LmjF24.1760	*L. major*	57	59	115
LmjF24.1780	*L. major*	41	471	511
LmjF24.1780	*L. major*	52	588	639
LmjF24.1780	*L. major*	52	1090	1141
LmjF24.1790	*L. major*	129	1	129
LmjF24.1810	*L. major*	57	278	334
LmjF24.1820	*L. major*	105	16	120
LmjF24.1830	*L. major*	135	34	168
LmjF24.1850	*L. major*	92	84	175
LmjF24.1850	*L. major*	82	185	266
LmjF24.1860	*L. major*	412	1	412
LmjF24.1870	*L. major*	80	42	121
LmjF24.1870	*L. major*	60	381	440
LmjF24.1870	*L. major*	60	471	530
LmjF24.1870	*L. major*	80	668	747
LmjF24.1870	*L. major*	133	769	901
LmjF24.1870	*L. major*	143	992	1134
LmjF24.1870	*L. major*	103	1168	1270
LmjF24.1880	*L. major*	112	83	194
LmjF24.1880	*L. major*	198	735	932
LmjF24.1890	*L. major*	108	1	108
LmjF24.1890	*L. major*	59	137	195
LmjF24.1890	*L. major*	160	324	483
LmjF24.1890	*L. major*	151	507	657
LmjF24.1900	*L. major*	54	492	545
LmjF24.1905	*L. major*	50	377	426
LmjF24.1920	*L. major*	102	1	102

LmjF24.1920	*L. major*	68	225	292
LmjF24.1920	*L. major*	62	411	472
LmjF24.1920	*L. major*	47	476	522
LmjF24.1930	*L. major*	121	1	121
LmjF24.1930	*L. major*	44	156	199
LmjF24.1930	*L. major*	70	427	496
LmjF24.1930	*L. major*	49	579	627
LmjF24.1940	*L. major*	147	1	147
LmjF24.1950	*L. major*	54	290	343
LmjF24.1950	*L. major*	44	531	574
LmjF24.1950	*L. major*	44	531	574
LmjF24.1960	*L. major*	363	1	363
LmjF24.1960	*L. major*	55	410	464
LmjF24.1960	*L. major*	50	486	535
LmjF24.1960	*L. major*	291	542	832
LmjF24.1970	*L. major*	54	40	93
LmjF24.1970	*L. major*	60	194	253
LmjF24.1970	*L. major*	43	282	324
LmjF24.1970	*L. major*	40	533	572
LmjF24.1980	*L. major*	190	42	231
LmjF24.1990	*L. major*	120	29	148
LmjF24.1990	*L. major*	153	180	332
LmjF24.2000	*L. major*	63	79	141
LmjF24.2000	*L. major*	42	193	234
LmjF24.2000	*L. major*	42	193	234
LmjF24.2010	*L. major*	71	517	587
LmjF24.2010	*L. major*	108	607	714
LmjF24.2010	*L. major*	60	766	825
LmjF24.2020	*L. major*	87	1	87
LmjF24.2020	*L. major*	40	147	186
LmjF24.2020	*L. major*	41	477	517
LmjF24.2020	*L. major*	62	1384	1445
LmjF24.2050	*L. major*	40	1	40
LmjF24.2050	*L. major*	41	103	143
LmjF24.2050	*L. major*	41	103	143
LmjF24.2070	*L. major*	46	1	46
LmjF24.2070	*L. major*	79	122	200
LmjF24.2070	*L. major*	79	122	200
LmjF24.2080	*L. major*	46	1	46
LmjF24.2080	*L. major*	79	122	200
LmjF24.2080	*L. major*	79	122	200
LmjF24.2100	*L. major*	91	220	310
LmjF24.2120	*L. major*	54	13	66
LmjF24.2120	*L. major*	219	77	295
LmjF24.2140	*L. major*	68	1	68
LmjF24.2150	*L. major*	75	1	75
LmjF24.2170	*L. major*	41	180	220

LmjF24.2170	*L. major*	58	584	641
LmjF24.2170	*L. major*	58	584	641
LmjF24.2180	*L. major*	149	1	149
LmjF24.2180	*L. major*	464	181	644
LmjF24.2180	*L. major*	375	660	1034
LmjF24.2180	*L. major*	368	1071	1438
LmjF24.2190	*L. major*	57	1	57
LmjF24.2190	*L. major*	40	208	247
LmjF24.2240	*L. major*	54	123	176
LmjF24.2260	*L. major*	67	1	67
LmjF24.2280	*L. major*	57	242	298
LmjF24.2280	*L. major*	69	745	813
LmjF24.2280	*L. major*	69	745	813
LmjF24.2310	*L. major*	66	98	163
LmjF24.2320	*L. major*	87	1	87
LmjF24.2330	*L. major*	85	174	258
LmjF24.2340	*L. major*	41	189	229
LmjF25.0030	*L. major*	84	382	465
LmjF25.0040	*L. major*	118	174	291
LmjF25.0040	*L. major*	130	388	517
LmjF25.0040	*L. major*	170	545	714
LmjF25.0040	*L. major*	42	726	767
LmjF25.0040	*L. major*	76	855	930
LmjF25.0040	*L. major*	59	944	1002
LmjF25.0040	*L. major*	66	1191	1256
LmjF25.0050	*L. major*	59	113	171
LmjF25.0060	*L. major*	66	1	66
LmjF25.0070	*L. major*	44	105	148
LmjF25.0080	*L. major*	142	345	486
LmjF25.0090	*L. major*	66	1	66
LmjF25.0110	*L. major*	48	286	333
LmjF25.0120	*L. major*	42	197	238
LmjF25.0150	*L. major*	41	151	191
LmjF25.0150	*L. major*	50	239	288
LmjF25.0150	*L. major*	69	326	394
LmjF25.0160	*L. major*	44	38	81
LmjF25.0160	*L. major*	92	124	215
LmjF25.0160	*L. major*	92	124	215
LmjF25.0170	*L. major*	124	387	510
LmjF25.0170	*L. major*	41	538	578
LmjF25.0170	*L. major*	105	674	778
LmjF25.0180	*L. major*	55	92	146
LmjF25.0200	*L. major*	60	812	871
LmjF25.0220	*L. major*	168	126	293
LmjF25.0220	*L. major*	157	557	713
LmjF25.0230	*L. major*	85	1	85
LmjF25.0240	*L. major*	100	1	100

LmjF25.0250	*L. major*	266	319	584
LmjF25.0250	*L. major*	264	614	877
LmjF25.0270	*L. major*	43	1	43
LmjF25.0270	*L. major*	53	264	316
LmjF25.0270	*L. major*	111	419	529
LmjF25.0270	*L. major*	56	574	629
LmjF25.0270	*L. major*	41	650	690
LmjF25.0270	*L. major*	106	740	845
LmjF25.0270	*L. major*	222	935	1156
LmjF25.0280	*L. major*	106	99	204
LmjF25.0280	*L. major*	153	227	379
LmjF25.0280	*L. major*	63	434	496
LmjF25.0280	*L. major*	130	562	691
LmjF25.0290	*L. major*	120	173	292
LmjF25.0290	*L. major*	161	424	584
LmjF25.0300	*L. major*	41	453	493
LmjF25.0310	*L. major*	195	241	435
LmjF25.0310	*L. major*	50	501	550
LmjF25.0310	*L. major*	137	586	722
LmjF25.0310	*L. major*	69	742	810
LmjF25.0310	*L. major*	174	909	1082
LmjF25.0330	*L. major*	398	1	398
LmjF25.0330	*L. major*	57	417	473
LmjF25.0340	*L. major*	74	448	521
LmjF25.0350	*L. major*	68	46	113
LmjF25.0350	*L. major*	91	124	214
LmjF25.0350	*L. major*	76	253	328
LmjF25.0370	*L. major*	74	1	74
LmjF25.0370	*L. major*	46	134	179
LmjF25.0370	*L. major*	46	134	179
LmjF25.0380	*L. major*	441	63	503
LmjF25.0380	*L. major*	63	524	586
LmjF25.0380	*L. major*	127	621	747
LmjF25.0380	*L. major*	40	814	853
LmjF25.0390	*L. major*	43	1	43
LmjF25.0410	*L. major*	123	1	123
LmjF25.0420	*L. major*	197	97	293
LmjF25.0420	*L. major*	460	525	984
LmjF25.0420	*L. major*	49	1005	1053
LmjF25.0420	*L. major*	277	1171	1447
LmjF25.0420	*L. major*	265	1471	1735
LmjF25.0430	*L. major*	150	87	236
LmjF25.0440	*L. major*	42	44	85
LmjF25.0440	*L. major*	42	269	310
LmjF25.0440	*L. major*	114	365	478
LmjF25.0440	*L. major*	77	497	573
LmjF25.0440	*L. major*	53	620	672

LmjF25.0450	*L. major*	50	1	50
LmjF25.0450	*L. major*	50	104	153
LmjF25.0450	*L. major*	50	104	153
LmjF25.0460	*L. major*	63	1	63
LmjF25.0470	*L. major*	40	556	595
LmjF25.0470	*L. major*	48	1042	1089
LmjF25.0470	*L. major*	48	1042	1089
LmjF25.0480	*L. major*	53	151	203
LmjF25.0480	*L. major*	53	435	487
LmjF25.0480	*L. major*	41	699	739
LmjF25.0480	*L. major*	43	841	883
LmjF25.0480	*L. major*	61	1012	1072
LmjF25.0480	*L. major*	91	1135	1225
LmjF25.0480	*L. major*	255	1254	1508
LmjF25.0490	*L. major*	44	1	44
LmjF25.0490	*L. major*	62	116	177
LmjF25.0490	*L. major*	62	116	177
LmjF25.0500	*L. major*	62	106	167
LmjF25.0510	*L. major*	48	166	213
LmjF25.0520	*L. major*	69	139	207
LmjF25.0540	*L. major*	222	74	295
LmjF25.0550	*L. major*	82	1	82
LmjF25.0550	*L. major*	67	141	207
LmjF25.0550	*L. major*	48	509	556
LmjF25.0560	*L. major*	49	27	75
LmjF25.0570	*L. major*	98	100	197
LmjF25.0590	*L. major*	49	1	49
LmjF25.0600	*L. major*	58	54	111
LmjF25.0600	*L. major*	74	273	346
LmjF25.0600	*L. major*	74	273	346
LmjF25.0610	*L. major*	66	1	66
LmjF25.0610	*L. major*	82	599	680
LmjF25.0610	*L. major*	50	1026	1075
LmjF25.0620	*L. major*	53	1	53
LmjF25.0620	*L. major*	46	283	328
LmjF25.0640	*L. major*	61	30	90
LmjF25.0650	*L. major*	60	42	101
LmjF25.0650	*L. major*	110	364	473
LmjF25.0670	*L. major*	452	142	593
LmjF25.0680	*L. major*	938	1	938
LmjF25.0680	*L. major*	74	942	1015
LmjF25.0680	*L. major*	99	1020	1118
LmjF25.0680	*L. major*	142	1355	1496
LmjF25.0680	*L. major*	213	1514	1726
LmjF25.0690	*L. major*	55	1	55
LmjF25.0690	*L. major*	43	531	573
LmjF25.0690	*L. major*	52	697	748

LmjF25.0690	*L. major*	91	779	869
LmjF25.0700	*L. major*	54	1	54
LmjF25.0700	*L. major*	61	174	234
LmjF25.0700	*L. major*	65	255	319
LmjF25.0700	*L. major*	145	354	498
LmjF25.0710	*L. major*	154	1	154
LmjF25.0715	*L. major*	62	1	62
LmjF25.0715	*L. major*	73	208	280
LmjF25.0715	*L. major*	73	208	280
LmjF25.0720	*L. major*	46	96	141
LmjF25.0730	*L. major*	46	96	141
LmjF25.0740	*L. major*	53	99	151
LmjF25.0740	*L. major*	146	289	434
LmjF25.0740	*L. major*	67	446	512
LmjF25.0740	*L. major*	67	847	913
LmjF25.0750	*L. major*	117	290	406
LmjF25.0760	*L. major*	68	1	68
LmjF25.0770	*L. major*	52	11	62
LmjF25.0770	*L. major*	74	94	167
LmjF25.0770	*L. major*	107	410	516
LmjF25.0780	*L. major*	41	68	108
LmjF25.0780	*L. major*	109	199	307
LmjF25.0790	*L. major*	50	1	50
LmjF25.0790	*L. major*	101	224	324
LmjF25.0790	*L. major*	58	652	709
LmjF25.0790	*L. major*	47	736	782
LmjF25.0790	*L. major*	40	882	921
LmjF25.0800	*L. major*	144	1	144
LmjF25.0810	*L. major*	42	62	103
LmjF25.0810	*L. major*	51	204	254
LmjF25.0810	*L. major*	123	299	421
LmjF25.0810	*L. major*	47	641	687
LmjF25.0810	*L. major*	46	719	764
LmjF25.0810	*L. major*	85	789	873
LmjF25.0810	*L. major*	91	894	984
LmjF25.0830	*L. major*	100	71	170
LmjF25.0830	*L. major*	91	300	390
LmjF25.0830	*L. major*	80	562	641
LmjF25.0830	*L. major*	159	819	977
LmjF25.0840	*L. major*	146	233	378
LmjF25.0850	*L. major*	138	186	323
LmjF25.0850	*L. major*	447	378	824
LmjF25.0850	*L. major*	197	841	1037
LmjF25.0850	*L. major*	103	1045	1147
LmjF25.0860	*L. major*	315	17	331
LmjF25.0860	*L. major*	331	636	966
LmjF25.0860	*L. major*	109	976	1084

LmjF25.0880	*L. major*	47	125	171
LmjF25.0880	*L. major*	581	251	831
LmjF25.0880	*L. major*	59	872	930
LmjF25.0880	*L. major*	115	938	1052
LmjF25.0880	*L. major*	179	1092	1270
LmjF25.0880	*L. major*	49	1275	1323
LmjF25.0900	*L. major*	58	132	189
LmjF25.0900	*L. major*	65	276	340
LmjF25.0910	*L. major*	43	78	120
LmjF25.0920	*L. major*	177	1	177
LmjF25.0920	*L. major*	270	276	545
LmjF25.0920	*L. major*	44	588	631
LmjF25.0920	*L. major*	150	653	802
LmjF25.0920	*L. major*	60	812	871
LmjF25.0930	*L. major*	43	101	143
LmjF25.0940	*L. major*	229	79	307
LmjF25.0940	*L. major*	47	343	389
LmjF25.0940	*L. major*	86	418	503
LmjF25.0940	*L. major*	91	516	606
LmjF25.0950	*L. major*	166	262	427
LmjF25.0970	*L. major*	390	80	469
LmjF25.0970	*L. major*	50	535	584
LmjF25.0970	*L. major*	305	624	928
LmjF25.0970	*L. major*	98	1002	1099
LmjF25.0970	*L. major*	42	1165	1206
LmjF25.0980	*L. major*	40	2889	2928
LmjF25.0980	*L. major*	97	3202	3298
LmjF25.0980	*L. major*	60	3453	3512
LmjF25.0980	*L. major*	47	3631	3677
LmjF25.0980	*L. major*	72	3745	3816
LmjF25.1000	*L. major*	176	1	176
LmjF25.1000	*L. major*	190	178	367
LmjF25.1000	*L. major*	96	378	473
LmjF25.1000	*L. major*	69	488	556
LmjF25.1000	*L. major*	93	558	650
LmjF25.1010	*L. major*	53	272	324
LmjF25.1020	*L. major*	60	236	295
LmjF25.1020	*L. major*	84	348	431
LmjF25.1030	*L. major*	94	451	544
LmjF25.1030	*L. major*	64	640	703
LmjF25.1030	*L. major*	47	712	758
LmjF25.1040	*L. major*	65	1	65
LmjF25.1040	*L. major*	42	164	205
LmjF25.1040	*L. major*	481	236	716
LmjF25.1050	*L. major*	42	21	62
LmjF25.1060	*L. major*	344	1	344
LmjF25.1060	*L. major*	83	426	508

LmjF25.1060	*L. major*	230	526	755
LmjF25.1070	*L. major*	144	1	144
LmjF25.1070	*L. major*	234	210	443
LmjF25.1070	*L. major*	58	567	624
LmjF25.1070	*L. major*	76	704	779
LmjF25.1070	*L. major*	70	789	858
LmjF25.1080	*L. major*	292	164	455
LmjF25.1110	*L. major*	797	1	797
LmjF25.1110	*L. major*	80	876	955
LmjF25.1110	*L. major*	191	1030	1220
LmjF25.1110	*L. major*	132	1236	1367
LmjF25.1110	*L. major*	61	1510	1570
LmjF25.1110	*L. major*	209	1603	1811
LmjF25.1110	*L. major*	67	2214	2280
LmjF25.1110	*L. major*	45	2334	2378
LmjF25.1110	*L. major*	46	2586	2631
LmjF25.1110	*L. major*	46	2697	2742
LmjF25.1110	*L. major*	40	2783	2822
LmjF25.1110	*L. major*	110	2894	3003
LmjF25.1110	*L. major*	69	3065	3133
LmjF25.1110	*L. major*	104	3171	3274
LmjF25.1110	*L. major*	54	3277	3330
LmjF25.1110	*L. major*	87	3681	3767
LmjF25.1110	*L. major*	63	3769	3831
LmjF25.1110	*L. major*	88	3858	3945
LmjF25.1110	*L. major*	56	4122	4177
LmjF25.1110	*L. major*	63	4184	4246
LmjF25.1110	*L. major*	78	4548	4625
LmjF25.1110	*L. major*	142	4674	4815
LmjF25.1110	*L. major*	81	4853	4933
LmjF25.1110	*L. major*	40	4959	4998
LmjF25.1110	*L. major*	106	5083	5188
LmjF25.1110	*L. major*	89	5440	5528
LmjF25.1110	*L. major*	86	5660	5745
LmjF25.1110	*L. major*	58	5808	5865
LmjF25.1110	*L. major*	63	5894	5956
LmjF25.1110	*L. major*	88	5982	6069
LmjF25.1110	*L. major*	103	6156	6258
LmjF25.1110	*L. major*	49	6437	6485
LmjF25.1140	*L. major*	96	67	162
LmjF25.1140	*L. major*	152	207	358
LmjF25.1140	*L. major*	49	385	433
LmjF25.1140	*L. major*	57	487	543
LmjF25.1190	*L. major*	40	1	40
LmjF25.1200	*L. major*	56	60	115
LmjF25.1200	*L. major*	65	250	314
LmjF25.1200	*L. major*	50	371	420

LmjF25.1200	*L. major*	54	617	670
LmjF25.1200	*L. major*	70	724	793
LmjF25.1210	*L. major*	274	1	274
LmjF25.1210	*L. major*	44	466	509
LmjF25.1210	*L. major*	40	901	940
LmjF25.1220	*L. major*	48	83	130
LmjF25.1220	*L. major*	60	177	236
LmjF25.1220	*L. major*	44	528	571
LmjF25.1250	*L. major*	83	34	116
LmjF25.1250	*L. major*	54	154	207
LmjF25.1250	*L. major*	98	262	359
LmjF25.1250	*L. major*	148	535	682
LmjF25.1250	*L. major*	46	771	816
LmjF25.1250	*L. major*	57	855	911
LmjF25.1250	*L. major*	113	974	1086
LmjF25.1250	*L. major*	166	1532	1697
LmjF25.1250	*L. major*	152	1748	1899
LmjF25.1260	*L. major*	65	175	239
LmjF25.1260	*L. major*	95	266	360
LmjF25.1260	*L. major*	132	393	524
LmjF25.1260	*L. major*	125	793	917
LmjF25.1260	*L. major*	61	954	1014
LmjF25.1260	*L. major*	145	1088	1232
LmjF25.1260	*L. major*	127	1252	1378
LmjF25.1260	*L. major*	80	1454	1533
LmjF25.1260	*L. major*	72	1558	1629
LmjF25.1260	*L. major*	64	1658	1721
LmjF25.1260	*L. major*	44	1805	1848
LmjF25.1260	*L. major*	99	1905	2003
LmjF25.1260	*L. major*	224	2103	2326
LmjF25.1270	*L. major*	48	102	149
LmjF25.1270	*L. major*	136	169	304
LmjF25.1270	*L. major*	180	331	510
LmjF25.1280	*L. major*	60	112	171
LmjF25.1280	*L. major*	69	351	419
LmjF25.1280	*L. major*	102	647	748
LmjF25.1280	*L. major*	82	1000	1081
LmjF25.1290	*L. major*	98	52	149
LmjF25.1300	*L. major*	60	12	71
LmjF25.1300	*L. major*	75	135	209
LmjF25.1300	*L. major*	40	460	499
LmjF25.1300	*L. major*	89	540	628
LmjF25.1310	*L. major*	50	1	50
LmjF25.1310	*L. major*	75	111	185
LmjF25.1330	*L. major*	73	147	219
LmjF25.1330	*L. major*	184	255	438
LmjF25.1330	*L. major*	135	555	689

LmjF25.1340	*L. major*	250	1	250
LmjF25.1350	*L. major*	159	23	181
LmjF25.1350	*L. major*	123	183	305
LmjF25.1350	*L. major*	131	349	479
LmjF25.1350	*L. major*	45	620	664
LmjF25.1350	*L. major*	40	717	756
LmjF25.1350	*L. major*	66	849	914
LmjF25.1350	*L. major*	69	1184	1252
LmjF25.1350	*L. major*	82	1640	1721
LmjF25.1350	*L. major*	62	1936	1997
LmjF25.1350	*L. major*	231	2035	2265
LmjF25.1350	*L. major*	57	2338	2394
LmjF25.1350	*L. major*	44	2506	2549
LmjF25.1360	*L. major*	54	1	54
LmjF25.1360	*L. major*	43	86	128
LmjF25.1360	*L. major*	110	160	269
LmjF25.1360	*L. major*	153	577	729
LmjF25.1360	*L. major*	40	772	811
LmjF25.1360	*L. major*	108	858	965
LmjF25.1370	*L. major*	78	98	175
LmjF25.1370	*L. major*	74	209	282
LmjF25.1370	*L. major*	81	393	473
LmjF25.1380	*L. major*	68	34	101
LmjF25.1380	*L. major*	116	121	236
LmjF25.1400	*L. major*	70	73	142
LmjF25.1400	*L. major*	104	292	395
LmjF25.1410	*L. major*	53	116	168
LmjF25.1410	*L. major*	75	360	434
LmjF25.1410	*L. major*	46	511	556
LmjF25.1430	*L. major*	93	155	247
LmjF25.1430	*L. major*	217	271	487
LmjF25.1440	*L. major*	208	302	509
LmjF25.1443	*L. major*	48	97	144
LmjF25.1443	*L. major*	43	205	247
LmjF25.1443	*L. major*	43	205	247
LmjF25.1450	*L. major*	43	297	339
LmjF25.1450	*L. major*	50	345	394
LmjF25.1450	*L. major*	50	345	394
LmjF25.1460	*L. major*	46	208	253
LmjF25.1460	*L. major*	69	410	478
LmjF25.1460	*L. major*	74	647	720
LmjF25.1480	*L. major*	73	122	194
LmjF25.1490	*L. major*	56	64	119
LmjF25.1490	*L. major*	99	194	292
LmjF25.1490	*L. major*	63	532	594
LmjF25.1490	*L. major*	92	615	706
LmjF25.1490	*L. major*	92	765	856

LmjF25.1490	*L. major*	45	919	963
LmjF25.1490	*L. major*	49	1168	1216
LmjF25.1500	*L. major*	47	298	344
LmjF25.1500	*L. major*	83	397	479
LmjF25.1500	*L. major*	83	397	479
LmjF25.1510	*L. major*	259	250	508
LmjF25.1520	*L. major*	185	1	185
LmjF25.1520	*L. major*	103	208	310
LmjF25.1520	*L. major*	61	319	379
LmjF25.1520	*L. major*	43	410	452
LmjF25.1520	*L. major*	52	686	737
LmjF25.1520	*L. major*	204	741	944
LmjF25.1530	*L. major*	40	149	188
LmjF25.1530	*L. major*	42	505	546
LmjF25.1530	*L. major*	42	505	546
LmjF25.1540	*L. major*	85	1	85
LmjF25.1540	*L. major*	40	656	695
LmjF25.1560	*L. major*	126	14	139
LmjF25.1560	*L. major*	74	333	406
LmjF25.1560	*L. major*	98	472	569
LmjF25.1560	*L. major*	40	723	762
LmjF25.1570	*L. major*	40	113	152
LmjF25.1570	*L. major*	65	295	359
LmjF25.1570	*L. major*	77	827	903
LmjF25.1570	*L. major*	68	1148	1215
LmjF25.1570	*L. major*	61	1253	1313
LmjF25.1570	*L. major*	67	1629	1695
LmjF25.1570	*L. major*	65	1774	1838
LmjF25.1570	*L. major*	46	1891	1936
LmjF25.1570	*L. major*	85	2052	2136
LmjF25.1570	*L. major*	94	2171	2264
LmjF25.1580	*L. major*	44	71	114
LmjF25.1580	*L. major*	76	332	407
LmjF25.1580	*L. major*	68	438	505
LmjF25.1590	*L. major*	496	84	579
LmjF25.1590	*L. major*	58	748	805
LmjF25.1590	*L. major*	49	913	961
LmjF25.1590	*L. major*	130	1017	1146
LmjF25.1600	*L. major*	41	123	163
LmjF25.1600	*L. major*	53	577	629
LmjF25.1600	*L. major*	54	735	788
LmjF25.1600	*L. major*	42	1130	1171
LmjF25.1610	*L. major*	69	201	269
LmjF25.1630	*L. major*	121	1	121
LmjF25.1630	*L. major*	76	176	251
LmjF25.1640	*L. major*	58	216	273
LmjF25.1640	*L. major*	47	321	367

LmjF25.1640	*L. major*	70	407	476
LmjF25.1645	*L. major*	58	1	58
LmjF25.1660	*L. major*	86	485	570
LmjF25.1680	*L. major*	136	1	136
LmjF25.1680	*L. major*	97	175	271
LmjF25.1680	*L. major*	183	549	731
LmjF25.1680	*L. major*	167	747	913
LmjF25.1680	*L. major*	44	1109	1152
LmjF25.1680	*L. major*	44	1407	1450
LmjF25.1690	*L. major*	65	83	147
LmjF25.1690	*L. major*	55	151	205
LmjF25.1690	*L. major*	55	151	205
LmjF25.1700	*L. major*	93	216	308
LmjF25.1700	*L. major*	91	334	424
LmjF25.1700	*L. major*	164	443	606
LmjF25.1700	*L. major*	72	717	788
LmjF25.1700	*L. major*	61	833	893
LmjF25.1700	*L. major*	94	937	1030
LmjF25.1720	*L. major*	43	481	523
LmjF25.1720	*L. major*	42	716	757
LmjF25.1720	*L. major*	53	1207	1259
LmjF25.1720	*L. major*	111	1461	1571
LmjF25.1730	*L. major*	87	36	122
LmjF25.1740	*L. major*	106	1	106
LmjF25.1740	*L. major*	125	183	307
LmjF25.1760	*L. major*	147	15	161
LmjF25.1760	*L. major*	89	251	339
LmjF25.1770	*L. major*	42	38	79
LmjF25.1780	*L. major*	112	1	112
LmjF25.1780	*L. major*	137	132	268
LmjF25.1780	*L. major*	223	335	557
LmjF25.1780	*L. major*	94	1025	1118
LmjF25.1800	*L. major*	94	94	187
LmjF25.1800	*L. major*	86	228	313
LmjF25.1800	*L. major*	122	414	535
LmjF25.1800	*L. major*	44	751	794
LmjF25.1800	*L. major*	73	920	992
LmjF25.1810	*L. major*	47	1	47
LmjF25.1820	*L. major*	66	1	66
LmjF25.1820	*L. major*	732	100	831
LmjF25.1830	*L. major*	44	1	44
LmjF25.1830	*L. major*	128	75	202
LmjF25.1830	*L. major*	173	279	451
LmjF25.1830	*L. major*	414	583	996
LmjF25.1840	*L. major*	151	11	161
LmjF25.1840	*L. major*	324	183	506
LmjF25.1850	*L. major*	139	1	139

LmjF25.1850	*L. major*	68	159	226
LmjF25.1860	*L. major*	61	14	74
LmjF25.1870	*L. major*	63	54	116
LmjF25.1870	*L. major*	170	192	361
LmjF25.1890	*L. major*	61	1	61
LmjF25.1900	*L. major*	54	1	54
LmjF25.1910	*L. major*	47	62	108
LmjF25.1910	*L. major*	85	135	219
LmjF25.1910	*L. major*	44	251	294
LmjF25.1920	*L. major*	121	1	121
LmjF25.1920	*L. major*	45	358	402
LmjF25.1940	*L. major*	59	395	453
LmjF25.1950	*L. major*	79	317	395
LmjF25.1950	*L. major*	249	471	719
LmjF25.1950	*L. major*	72	755	826
LmjF25.1950	*L. major*	191	872	1062
LmjF25.1970	*L. major*	76	1	76
LmjF25.1970	*L. major*	137	464	600
LmjF25.1970	*L. major*	98	623	720
LmjF25.1970	*L. major*	51	843	893
LmjF25.1970	*L. major*	85	920	1004
LmjF25.1980	*L. major*	76	1	76
LmjF25.1980	*L. major*	693	790	1482
LmjF25.1990	*L. major*	44	234	277
LmjF25.1990	*L. major*	197	380	576
LmjF25.2000	*L. major*	54	263	316
LmjF25.2000	*L. major*	78	408	485
LmjF25.2000	*L. major*	120	782	901
LmjF25.2030	*L. major*	174	1	174
LmjF25.2050	*L. major*	212	1	212
LmjF25.2050	*L. major*	83	758	840
LmjF25.2050	*L. major*	75	969	1043
LmjF25.2060	*L. major*	80	1	80
LmjF25.2070	*L. major*	95	133	227
LmjF25.2070	*L. major*	53	432	484
LmjF25.2070	*L. major*	53	432	484
LmjF25.2095	*L. major*	113	1	113
LmjF25.2100	*L. major*	92	262	353
LmjF25.2110	*L. major*	40	326	365
LmjF25.2120	*L. major*	105	1	105
LmjF25.2120	*L. major*	59	153	211
LmjF25.2130	*L. major*	46	243	288
LmjF25.2140	*L. major*	46	243	288
LmjF25.2150	*L. major*	59	346	404
LmjF25.2155	*L. major*	83	50	132
LmjF25.2155	*L. major*	54	164	217
LmjF25.2155	*L. major*	54	164	217

LmjF25.2160	*L. major*	46	191	236
LmjF25.2170	*L. major*	55	1	55
LmjF25.2190	*L. major*	137	174	310
LmjF25.2190	*L. major*	49	329	377
LmjF25.2200	*L. major*	123	1	123
LmjF25.2220	*L. major*	70	377	446
LmjF25.2220	*L. major*	53	481	533
LmjF25.2235	*L. major*	54	166	219
LmjF25.2240	*L. major*	94	377	470
LmjF25.2250	*L. major*	63	77	139
LmjF25.2260	*L. major*	46	78	123
LmjF25.2270	*L. major*	41	109	149
LmjF25.2300	*L. major*	149	261	409
LmjF25.2310	*L. major*	42	368	409
LmjF25.2330	*L. major*	40	798	837
LmjF25.2340	*L. major*	45	1	45
LmjF25.2340	*L. major*	59	330	388
LmjF25.2340	*L. major*	59	330	388
LmjF25.2350	*L. major*	41	45	85
LmjF25.2350	*L. major*	45	150	194
LmjF25.2350	*L. major*	45	150	194
LmjF25.2360	*L. major*	57	1	57
LmjF25.2360	*L. major*	90	484	573
LmjF25.2410	*L. major*	269	397	665
LmjF25.2450	*L. major*	49	1	49
LmjF25.2460	*L. major*	93	1	93
LmjF25.2460	*L. major*	52	136	187
LmjF26.0010	*L. major*	57	306	362
LmjF26.0050	*L. major*	87	1	87
LmjF26.0050	*L. major*	177	91	267
LmjF26.0055	*L. major*	66	1	66
LmjF26.0055	*L. major*	152	151	302
LmjF26.0055	*L. major*	152	151	302
LmjF26.0150	*L. major*	78	120	197
LmjF26.0150	*L. major*	40	503	542
LmjF26.0150	*L. major*	51	548	598
LmjF26.0160	*L. major*	92	1	92
LmjF26.0170	*L. major*	72	1	72
LmjF26.0180	*L. major*	72	1	72
LmjF26.0210	*L. major*	64	254	317
LmjF26.0230	*L. major*	64	69	132
LmjF26.0230	*L. major*	76	301	376
LmjF26.0230	*L. major*	76	301	376
LmjF26.0250	*L. major*	45	340	384
LmjF26.0260	*L. major*	42	111	152
LmjF26.0290	*L. major*	69	92	160
LmjF26.0310	*L. major*	72	1	72

LmjF26.0350	*L. major*	94	588	681
LmjF26.0360	*L. major*	60	14	73
LmjF26.0360	*L. major*	50	184	233
LmjF26.0360	*L. major*	44	648	691
LmjF26.0360	*L. major*	42	782	823
LmjF26.0360	*L. major*	85	917	1001
LmjF26.0360	*L. major*	85	1161	1245
LmjF26.0360	*L. major*	40	1247	1286
LmjF26.0360	*L. major*	67	1490	1556
LmjF26.0370	*L. major*	72	469	540
LmjF26.0390	*L. major*	46	8	53
LmjF26.0400	*L. major*	58	80	137
LmjF26.0400	*L. major*	77	151	227
LmjF26.0400	*L. major*	43	287	329
LmjF26.0420	*L. major*	59	300	358
LmjF26.0420	*L. major*	78	559	636
LmjF26.0440	*L. major*	186	30	215
LmjF26.0460	*L. major*	67	100	166
LmjF26.0470	*L. major*	43	1	43
LmjF26.0470	*L. major*	44	915	958
LmjF26.0470	*L. major*	44	915	958
LmjF26.0480	*L. major*	45	223	267
LmjF26.0490	*L. major*	111	1	111
LmjF26.0490	*L. major*	56	189	244
LmjF26.0500	*L. major*	44	276	319
LmjF26.0500	*L. major*	41	389	429
LmjF26.0500	*L. major*	41	389	429
LmjF26.0540	*L. major*	45	17	61
LmjF26.0540	*L. major*	52	144	195
LmjF26.0540	*L. major*	49	641	689
LmjF26.0540	*L. major*	74	1030	1103
LmjF26.0550	*L. major*	92	1	92
LmjF26.0550	*L. major*	44	272	315
LmjF26.0560	*L. major*	125	1	125
LmjF26.0560	*L. major*	216	320	535
LmjF26.0580	*L. major*	72	85	156
LmjF26.0580	*L. major*	77	246	322
LmjF26.0580	*L. major*	49	346	394
LmjF26.0580	*L. major*	64	516	579
LmjF26.0590	*L. major*	111	1	111
LmjF26.0590	*L. major*	73	149	221
LmjF26.0590	*L. major*	100	434	533
LmjF26.0590	*L. major*	66	638	703
LmjF26.0600	*L. major*	122	64	185
LmjF26.0600	*L. major*	113	220	332
LmjF26.0610	*L. major*	108	12	119
LmjF26.0650	*L. major*	78	471	548

LmjF26.0650	*L. major*	65	1126	1190
LmjF26.0650	*L. major*	65	1126	1190
LmjF26.0660	*L. major*	68	294	361
LmjF26.0690	*L. major*	110	85	194
LmjF26.0700	*L. major*	241	1	241
LmjF26.0700	*L. major*	46	261	306
LmjF26.0700	*L. major*	42	446	487
LmjF26.0710	*L. major*	143	1	143
LmjF26.0710	*L. major*	48	466	513
LmjF26.0710	*L. major*	49	588	636
LmjF26.0710	*L. major*	48	747	794
LmjF26.0720	*L. major*	74	60	133
LmjF26.0720	*L. major*	165	167	331
LmjF26.0720	*L. major*	140	468	607
LmjF26.0720	*L. major*	73	861	933
LmjF26.0720	*L. major*	78	1012	1089
LmjF26.0730	*L. major*	73	1	73
LmjF26.0730	*L. major*	197	93	289
LmjF26.0730	*L. major*	338	294	631
LmjF26.0730	*L. major*	186	661	846
LmjF26.0730	*L. major*	113	867	979
LmjF26.0730	*L. major*	78	1065	1142
LmjF26.0730	*L. major*	199	1370	1568
LmjF26.0730	*L. major*	141	1677	1817
LmjF26.0730	*L. major*	42	1850	1891
LmjF26.0750	*L. major*	57	180	236
LmjF26.0750	*L. major*	136	426	561
LmjF26.0750	*L. major*	401	1306	1706
LmjF26.0770	*L. major*	433	99	531
LmjF26.0840	*L. major*	40	1	40
LmjF26.0840	*L. major*	295	115	409
LmjF26.0840	*L. major*	123	595	717
LmjF26.0840	*L. major*	45	841	885
LmjF26.0850	*L. major*	57	248	304
LmjF26.0860	*L. major*	50	340	389
LmjF26.0860	*L. major*	72	606	677
LmjF26.0860	*L. major*	72	606	677
LmjF26.0870	*L. major*	49	87	135
LmjF26.0870	*L. major*	415	165	579
LmjF26.0870	*L. major*	101	599	699
LmjF26.0870	*L. major*	90	718	807
LmjF26.0900	*L. major*	92	1	92
LmjF26.0900	*L. major*	55	276	330
LmjF26.0900	*L. major*	142	710	851
LmjF26.0900	*L. major*	45	900	944
LmjF26.0910	*L. major*	47	57	103
LmjF26.0910	*L. major*	44	162	205

LmjF26.0910	*L. major*	40	275	314
LmjF26.0930	*L. major*	143	235	377
LmjF26.0940	*L. major*	41	1	41
LmjF26.0940	*L. major*	134	146	279
LmjF26.0940	*L. major*	98	305	402
LmjF26.0950	*L. major*	380	1	380
LmjF26.0960	*L. major*	48	45	92
LmjF26.0960	*L. major*	121	103	223
LmjF26.0960	*L. major*	52	968	1019
LmjF26.0960	*L. major*	58	1064	1121
LmjF26.0960	*L. major*	56	1346	1401
LmjF26.0960	*L. major*	45	1442	1486
LmjF26.0960	*L. major*	50	1611	1660
LmjF26.0970	*L. major*	126	438	563
LmjF26.0980	*L. major*	836	1	836
LmjF26.0990	*L. major*	117	474	590
LmjF26.1000	*L. major*	73	1	73
LmjF26.1000	*L. major*	75	77	151
LmjF26.1000	*L. major*	54	166	219
LmjF26.1010	*L. major*	63	279	341
LmjF26.1010	*L. major*	42	369	410
LmjF26.1010	*L. major*	42	369	410
LmjF26.1020	*L. major*	47	1	47
LmjF26.1020	*L. major*	58	581	638
LmjF26.1020	*L. major*	44	1832	1875
LmjF26.1020	*L. major*	68	2633	2700
LmjF26.1020	*L. major*	40	3167	3206
LmjF26.1030	*L. major*	110	31	140
LmjF26.1030	*L. major*	83	697	779
LmjF26.1040	*L. major*	45	164	208
LmjF26.1060	*L. major*	217	114	330
LmjF26.1070	*L. major*	76	1	76
LmjF26.1070	*L. major*	128	87	214
LmjF26.1070	*L. major*	51	411	461
LmjF26.1080	*L. major*	78	1	78
LmjF26.1080	*L. major*	116	80	195
LmjF26.1080	*L. major*	291	261	551
LmjF26.1080	*L. major*	248	571	818
LmjF26.1080	*L. major*	53	849	901
LmjF26.1080	*L. major*	143	918	1060
LmjF26.1080	*L. major*	47	1124	1170
LmjF26.1080	*L. major*	48	1309	1356
LmjF26.1080	*L. major*	61	1402	1462
LmjF26.1080	*L. major*	98	1514	1611
LmjF26.1090	*L. major*	112	1	112
LmjF26.1110	*L. major*	41	31	71
LmjF26.1110	*L. major*	75	165	239

LmjF26.1110	*L. major*	47	263	309
LmjF26.1110	*L. major*	45	528	572
LmjF26.1110	*L. major*	86	687	772
LmjF26.1110	*L. major*	46	856	901
LmjF26.1110	*L. major*	146	1002	1147
LmjF26.1110	*L. major*	63	1174	1236
LmjF26.1110	*L. major*	93	1452	1544
LmjF26.1110	*L. major*	57	1576	1632
LmjF26.1110	*L. major*	103	1670	1772
LmjF26.1110	*L. major*	45	2331	2375
LmjF26.1110	*L. major*	90	2414	2503
LmjF26.1110	*L. major*	48	2704	2751
LmjF26.1110	*L. major*	63	3074	3136
LmjF26.1110	*L. major*	142	3191	3332
LmjF26.1110	*L. major*	59	3424	3482
LmjF26.1110	*L. major*	44	3541	3584
LmjF26.1110	*L. major*	52	3599	3650
LmjF26.1110	*L. major*	76	3783	3858
LmjF26.1110	*L. major*	68	3882	3949
LmjF26.1110	*L. major*	87	3960	4046
LmjF26.1110	*L. major*	43	4049	4091
LmjF26.1120	*L. major*	83	68	150
LmjF26.1120	*L. major*	95	192	286
LmjF26.1120	*L. major*	71	396	466
LmjF26.1130	*L. major*	256	1	256
LmjF26.1130	*L. major*	133	288	420
LmjF26.1130	*L. major*	230	557	786
LmjF26.1130	*L. major*	441	798	1238
LmjF26.1130	*L. major*	200	1246	1445
LmjF26.1130	*L. major*	138	1523	1660
LmjF26.1140	*L. major*	96	17	112
LmjF26.1140	*L. major*	42	126	167
LmjF26.1140	*L. major*	42	126	167
LmjF26.1150	*L. major*	46	139	184
LmjF26.1150	*L. major*	52	239	290
LmjF26.1150	*L. major*	141	509	649
LmjF26.1160	*L. major*	75	123	197
LmjF26.1160	*L. major*	80	288	367
LmjF26.1160	*L. major*	139	398	536
LmjF26.1160	*L. major*	58	670	727
LmjF26.1160	*L. major*	91	756	846
LmjF26.1160	*L. major*	60	900	959
LmjF26.1170	*L. major*	133	16	148
LmjF26.1170	*L. major*	122	223	344
LmjF26.1170	*L. major*	153	369	521
LmjF26.1180	*L. major*	54	1	54
LmjF26.1180	*L. major*	54	90	143

LmjF26.1180	*L. major*	54	161	214
LmjF26.1180	*L. major*	59	239	297
LmjF26.1180	*L. major*	70	391	460
LmjF26.1180	*L. major*	44	563	606
LmjF26.1190	*L. major*	605	26	630
LmjF26.1190	*L. major*	57	652	708
LmjF26.1190	*L. major*	424	914	1337
LmjF26.1190	*L. major*	53	1371	1423
LmjF26.1190	*L. major*	138	1507	1644
LmjF26.1190	*L. major*	85	1652	1736
LmjF26.1200	*L. major*	71	27	97
LmjF26.1200	*L. major*	41	241	281
LmjF26.1200	*L. major*	49	310	358
LmjF26.1210	*L. major*	54	183	236
LmjF26.1220	*L. major*	63	116	178
LmjF26.1220	*L. major*	139	232	370
LmjF26.1220	*L. major*	42	463	504
LmjF26.1220	*L. major*	151	520	670
LmjF26.1230	*L. major*	94	1	94
LmjF26.1230	*L. major*	50	273	322
LmjF26.1230	*L. major*	175	435	609
LmjF26.1230	*L. major*	104	614	717
LmjF26.1230	*L. major*	46	809	854
LmjF26.1230	*L. major*	91	904	994
LmjF26.1230	*L. major*	89	1032	1120
LmjF26.1230	*L. major*	88	1207	1294
LmjF26.1230	*L. major*	60	1402	1461
LmjF26.1230	*L. major*	399	1666	2064
LmjF26.1230	*L. major*	158	2082	2239
LmjF26.1230	*L. major*	185	2261	2445
LmjF26.1230	*L. major*	64	2466	2529
LmjF26.1230	*L. major*	111	2649	2759
LmjF26.1230	*L. major*	67	2877	2943
LmjF26.1240	*L. major*	105	495	599
LmjF26.1250	*L. major*	208	450	657
LmjF26.1260	*L. major*	202	143	344
LmjF26.1260	*L. major*	131	358	488
LmjF26.1260	*L. major*	74	511	584
LmjF26.1260	*L. major*	45	618	662
LmjF26.1260	*L. major*	97	912	1008
LmjF26.1260	*L. major*	121	1041	1161
LmjF26.1260	*L. major*	64	1204	1267
LmjF26.1270	*L. major*	60	1	60
LmjF26.1270	*L. major*	56	84	139
LmjF26.1270	*L. major*	61	333	393
LmjF26.1270	*L. major*	41	424	464
LmjF26.1270	*L. major*	128	553	680

LmjF26.1280	*L. major*	254	51	304
LmjF26.1280	*L. major*	43	423	465
LmjF26.1280	*L. major*	43	509	551
LmjF26.1280	*L. major*	75	690	764
LmjF26.1280	*L. major*	162	852	1013
LmjF26.1290	*L. major*	59	44	102
LmjF26.1290	*L. major*	69	146	214
LmjF26.1290	*L. major*	51	453	503
LmjF26.1300	*L. major*	60	181	240
LmjF26.1300	*L. major*	85	245	329
LmjF26.1300	*L. major*	90	407	496
LmjF26.1300	*L. major*	64	584	647
LmjF26.1300	*L. major*	50	726	775
LmjF26.1300	*L. major*	324	867	1190
LmjF26.1310	*L. major*	49	106	154
LmjF26.1320	*L. major*	40	14	53
LmjF26.1320	*L. major*	48	231	278
LmjF26.1320	*L. major*	48	231	278
LmjF26.1330	*L. major*	112	219	330
LmjF26.1340	*L. major*	61	1	61
LmjF26.1340	*L. major*	81	108	188
LmjF26.1340	*L. major*	51	477	527
LmjF26.1360	*L. major*	52	29	80
LmjF26.1360	*L. major*	59	137	195
LmjF26.1360	*L. major*	59	137	195
LmjF26.1370	*L. major*	129	1	129
LmjF26.1370	*L. major*	84	270	353
LmjF26.1370	*L. major*	101	469	569
LmjF26.1370	*L. major*	92	617	708
LmjF26.1390	*L. major*	210	1	210
LmjF26.1390	*L. major*	115	275	389
LmjF26.1390	*L. major*	125	412	536
LmjF26.1390	*L. major*	139	576	714
LmjF26.1390	*L. major*	63	725	787
LmjF26.1390	*L. major*	60	895	954
LmjF26.1390	*L. major*	50	1031	1080
LmjF26.1400	*L. major*	65	1	65
LmjF26.1400	*L. major*	43	215	257
LmjF26.1410	*L. major*	64	134	197
LmjF26.1410	*L. major*	73	325	397
LmjF26.1410	*L. major*	73	325	397
LmjF26.1420	*L. major*	62	404	465
LmjF26.1420	*L. major*	56	594	649
LmjF26.1420	*L. major*	63	689	751
LmjF26.1420	*L. major*	52	837	888
LmjF26.1420	*L. major*	90	964	1053
LmjF26.1420	*L. major*	77	1132	1208

LmjF26.1420	*L. major*	188	1317	1504
LmjF26.1420	*L. major*	66	1875	1940
LmjF26.1420	*L. major*	235	2018	2252
LmjF26.1420	*L. major*	115	2269	2383
LmjF26.1430	*L. major*	171	1	171
LmjF26.1430	*L. major*	782	191	972
LmjF26.1430	*L. major*	76	977	1052
LmjF26.1440	*L. major*	138	69	206
LmjF26.1440	*L. major*	117	306	422
LmjF26.1440	*L. major*	72	621	692
LmjF26.1450	*L. major*	256	1	256
LmjF26.1450	*L. major*	68	412	479
LmjF26.1450	*L. major*	313	508	820
LmjF26.1450	*L. major*	63	942	1004
LmjF26.1450	*L. major*	64	1010	1073
LmjF26.1460	*L. major*	57	279	335
LmjF26.1470	*L. major*	56	33	88
LmjF26.1470	*L. major*	74	172	245
LmjF26.1480	*L. major*	41	1	41
LmjF26.1480	*L. major*	67	80	146
LmjF26.1480	*L. major*	92	196	287
LmjF26.1480	*L. major*	71	429	499
LmjF26.1480	*L. major*	80	798	877
LmjF26.1490	*L. major*	49	398	446
LmjF26.1500	*L. major*	136	1	136
LmjF26.1510	*L. major*	254	1	254
LmjF26.1510	*L. major*	188	270	457
LmjF26.1510	*L. major*	393	491	883
LmjF26.1510	*L. major*	232	1003	1234
LmjF26.1510	*L. major*	55	1259	1313
LmjF26.1510	*L. major*	67	1341	1407
LmjF26.1510	*L. major*	59	1457	1515
LmjF26.1520	*L. major*	43	159	201
LmjF26.1530	*L. major*	98	1	98
LmjF26.1560	*L. major*	44	880	923
LmjF26.1560	*L. major*	44	1471	1514
LmjF26.1560	*L. major*	48	1579	1626
LmjF26.1580	*L. major*	42	1	42
LmjF26.1590	*L. major*	112	15	126
LmjF26.1590	*L. major*	86	195	280
LmjF26.1590	*L. major*	86	195	280
LmjF26.1600	*L. major*	159	475	633
LmjF26.1620	*L. major*	59	1	59
LmjF26.1660	*L. major*	78	1	78
LmjF26.1665	*L. major*	61	203	263
LmjF26.1700	*L. major*	47	290	336
LmjF26.1720	*L. major*	99	32	130

LmjF26.1720	*L. major*	117	360	476
LmjF26.1720	*L. major*	186	521	706
LmjF26.1740	*L. major*	103	26	128
LmjF26.1740	*L. major*	45	285	329
LmjF26.1750	*L. major*	64	73	136
LmjF26.1755	*L. major*	173	1	173
LmjF26.1755	*L. major*	45	233	277
LmjF26.1760	*L. major*	64	1	64
LmjF26.1760	*L. major*	107	93	199
LmjF26.1760	*L. major*	142	252	393
LmjF26.1760	*L. major*	58	429	486
LmjF26.1760	*L. major*	46	529	574
LmjF26.1780	*L. major*	62	151	212
LmjF26.1785	*L. major*	45	1	45
LmjF26.1785	*L. major*	55	144	198
LmjF26.1785	*L. major*	105	225	329
LmjF26.1790	*L. major*	40	1	40
LmjF26.1790	*L. major*	69	753	821
LmjF26.1790	*L. major*	47	904	950
LmjF26.1790	*L. major*	45	1122	1166
LmjF26.1790	*L. major*	97	1437	1533
LmjF26.1790	*L. major*	54	1690	1743
LmjF26.1790	*L. major*	76	1884	1959
LmjF26.1800	*L. major*	52	1	52
LmjF26.1810	*L. major*	52	1	52
LmjF26.1810	*L. major*	52	85	136
LmjF26.1810	*L. major*	237	172	408
LmjF26.1820	*L. major*	511	1	511
LmjF26.1820	*L. major*	455	517	971
LmjF26.1820	*L. major*	304	984	1287
LmjF26.1820	*L. major*	168	1317	1484
LmjF26.1820	*L. major*	74	1535	1608
LmjF26.1820	*L. major*	62	1682	1743
LmjF26.1820	*L. major*	54	1885	1938
LmjF26.1820	*L. major*	49	2017	2065
LmjF26.1820	*L. major*	98	2096	2193
LmjF26.1820	*L. major*	51	2220	2270
LmjF26.1830	*L. major*	295	16	310
LmjF26.1830	*L. major*	229	525	753
LmjF26.1840	*L. major*	134	39	172
LmjF26.1840	*L. major*	173	234	406
LmjF26.1840	*L. major*	46	430	475
LmjF26.1840	*L. major*	62	539	600
LmjF26.1840	*L. major*	52	678	729
LmjF26.1840	*L. major*	76	786	861
LmjF26.1860	*L. major*	47	1	47
LmjF26.1860	*L. major*	142	88	229

LmjF26.1860	*L. major*	58	467	524
LmjF26.1870	*L. major*	113	1	113
LmjF26.1870	*L. major*	49	208	256
LmjF26.1870	*L. major*	59	331	389
LmjF26.1870	*L. major*	53	435	487
LmjF26.1870	*L. major*	71	792	862
LmjF26.1870	*L. major*	267	887	1153
LmjF26.1870	*L. major*	84	1180	1263
LmjF26.1870	*L. major*	89	1437	1525
LmjF26.1870	*L. major*	223	1531	1753
LmjF26.1870	*L. major*	47	1797	1843
LmjF26.1870	*L. major*	388	1985	2372
LmjF26.1870	*L. major*	78	2456	2533
LmjF26.1870	*L. major*	41	2589	2629
LmjF26.1880	*L. major*	74	66	139
LmjF26.1880	*L. major*	57	180	236
LmjF26.1880	*L. major*	117	526	642
LmjF26.1890	*L. major*	65	69	133
LmjF26.1890	*L. major*	79	212	290
LmjF26.1900	*L. major*	72	140	211
LmjF26.1900	*L. major*	47	501	547
LmjF26.1900	*L. major*	68	1057	1124
LmjF26.1910	*L. major*	69	115	183
LmjF26.1930	*L. major*	448	1	448
LmjF26.1930	*L. major*	112	460	571
LmjF26.1930	*L. major*	46	823	868
LmjF26.1930	*L. major*	191	883	1073
LmjF26.1930	*L. major*	82	1124	1205
LmjF26.1930	*L. major*	131	1327	1457
LmjF26.1930	*L. major*	73	1586	1658
LmjF26.1930	*L. major*	56	1713	1768
LmjF26.1940	*L. major*	66	291	356
LmjF26.1950	*L. major*	58	81	138
LmjF26.1950	*L. major*	94	241	334
LmjF26.1950	*L. major*	81	383	463
LmjF26.1950	*L. major*	127	526	652
LmjF26.1950	*L. major*	44	1087	1130
LmjF26.1950	*L. major*	49	1786	1834
LmjF26.1950	*L. major*	69	2547	2615
LmjF26.1960	*L. major*	75	82	156
LmjF26.1960	*L. major*	70	413	482
LmjF26.1960	*L. major*	105	703	807
LmjF26.1970	*L. major*	67	220	286
LmjF26.1980	*L. major*	119	1	119
LmjF26.1990	*L. major*	40	1	40
LmjF26.1990	*L. major*	100	113	212
LmjF26.2000	*L. major*	41	56	96

LmjF26.2010	*L. major*	123	169	291
LmjF26.2010	*L. major*	397	331	727
LmjF26.2030	*L. major*	80	117	196
LmjF26.2030	*L. major*	47	486	532
LmjF26.2030	*L. major*	47	486	532
LmjF26.2040	*L. major*	70	110	179
LmjF26.2050	*L. major*	50	108	157
LmjF26.2050	*L. major*	101	242	342
LmjF26.2050	*L. major*	45	347	391
LmjF26.2050	*L. major*	72	449	520
LmjF26.2050	*L. major*	59	633	691
LmjF26.2050	*L. major*	138	751	888
LmjF26.2060	*L. major*	42	1	42
LmjF26.2060	*L. major*	48	99	146
LmjF26.2060	*L. major*	70	182	251
LmjF26.2060	*L. major*	71	327	397
LmjF26.2060	*L. major*	156	487	642
LmjF26.2060	*L. major*	41	662	702
LmjF26.2060	*L. major*	43	724	766
LmjF26.2060	*L. major*	60	807	866
LmjF26.2070	*L. major*	538	1	538
LmjF26.2070	*L. major*	206	564	769
LmjF26.2070	*L. major*	50	1028	1077
LmjF26.2070	*L. major*	47	1226	1272
LmjF26.2080	*L. major*	122	1	122
LmjF26.2080	*L. major*	145	152	296
LmjF26.2100	*L. major*	96	55	150
LmjF26.2100	*L. major*	168	173	340
LmjF26.2100	*L. major*	113	567	679
LmjF26.2100	*L. major*	135	723	857
LmjF26.2100	*L. major*	47	872	918
LmjF26.2110	*L. major*	44	1	44
LmjF26.2110	*L. major*	220	99	318
LmjF26.2110	*L. major*	63	424	486
LmjF26.2110	*L. major*	117	499	615
LmjF26.2110	*L. major*	142	668	809
LmjF26.2110	*L. major*	309	978	1286
LmjF26.2110	*L. major*	343	1417	1759
LmjF26.2120	*L. major*	222	1	222
LmjF26.2120	*L. major*	142	253	394
LmjF26.2120	*L. major*	179	417	595
LmjF26.2120	*L. major*	226	639	864
LmjF26.2120	*L. major*	41	868	908
LmjF26.2120	*L. major*	47	958	1004
LmjF26.2120	*L. major*	573	1094	1666
LmjF26.2120	*L. major*	63	1696	1758
LmjF26.2120	*L. major*	361	1780	2140

LmjF26.2120	*L. major*	91	2154	2244
LmjF26.2120	*L. major*	410	2333	2742
LmjF26.2120	*L. major*	94	2775	2868
LmjF26.2120	*L. major*	179	2902	3080
LmjF26.2120	*L. major*	62	3172	3233
LmjF26.2120	*L. major*	139	3268	3406
LmjF26.2120	*L. major*	141	3440	3580
LmjF26.2120	*L. major*	198	3624	3821
LmjF26.2120	*L. major*	158	3850	4007
LmjF26.2120	*L. major*	69	4069	4137
LmjF26.2130	*L. major*	44	1	44
LmjF26.2130	*L. major*	53	157	209
LmjF26.2150	*L. major*	55	45	99
LmjF26.2150	*L. major*	51	285	335
LmjF26.2150	*L. major*	51	285	335
LmjF26.2160	*L. major*	424	23	446
LmjF26.2160	*L. major*	219	618	836
LmjF26.2160	*L. major*	558	844	1401
LmjF26.2160	*L. major*	282	1419	1700
LmjF26.2160	*L. major*	118	1766	1883
LmjF26.2160	*L. major*	194	1937	2130
LmjF26.2170	*L. major*	93	1	93
LmjF26.2170	*L. major*	95	186	280
LmjF26.2170	*L. major*	63	302	364
LmjF26.2170	*L. major*	93	377	469
LmjF26.2170	*L. major*	1844	509	2352
LmjF26.2170	*L. major*	146	2636	2781
LmjF26.2180	*L. major*	340	65	404
LmjF26.2180	*L. major*	630	480	1109
LmjF26.2180	*L. major*	201	1167	1367
LmjF26.2180	*L. major*	138	1411	1548
LmjF26.2190	*L. major*	67	47	113
LmjF26.2190	*L. major*	65	301	365
LmjF26.2200	*L. major*	311	1	311
LmjF26.2210	*L. major*	63	1	63
LmjF26.2210	*L. major*	205	131	335
LmjF26.2210	*L. major*	118	571	688
LmjF26.2250	*L. major*	60	1	60
LmjF26.2250	*L. major*	78	90	167
LmjF26.2250	*L. major*	140	208	347
LmjF26.2250	*L. major*	98	373	470
LmjF26.2250	*L. major*	40	557	596
LmjF26.2250	*L. major*	89	644	732
LmjF26.2260	*L. major*	58	1	58
LmjF26.2260	*L. major*	92	515	606
LmjF26.2260	*L. major*	92	515	606
LmjF26.2270	*L. major*	84	184	267

LmjF26.2290	*L. major*	45	91	135
LmjF26.2300	*L. major*	99	41	139
LmjF26.2300	*L. major*	215	191	405
LmjF26.2300	*L. major*	49	465	513
LmjF26.2300	*L. major*	72	515	586
LmjF26.2300	*L. major*	137	653	789
LmjF26.2300	*L. major*	232	805	1036
LmjF26.2300	*L. major*	79	1044	1122
LmjF26.2310	*L. major*	91	21	111
LmjF26.2310	*L. major*	50	135	184
LmjF26.2310	*L. major*	62	220	281
LmjF26.2320	*L. major*	160	270	429
LmjF26.2320	*L. major*	165	494	658
LmjF26.2330	*L. major*	43	1	43
LmjF26.2330	*L. major*	52	76	127
LmjF26.2330	*L. major*	52	76	127
LmjF26.2340	*L. major*	43	1	43
LmjF26.2340	*L. major*	52	76	127
LmjF26.2340	*L. major*	52	76	127
LmjF26.2350	*L. major*	105	23	127
LmjF26.2350	*L. major*	257	144	400
LmjF26.2350	*L. major*	65	414	478
LmjF26.2350	*L. major*	261	482	742
LmjF26.2350	*L. major*	74	762	835
LmjF26.2350	*L. major*	50	838	887
LmjF26.2360	*L. major*	40	363	402
LmjF26.2360	*L. major*	62	554	615
LmjF26.2360	*L. major*	62	554	615
LmjF26.2370	*L. major*	44	122	165
LmjF26.2370	*L. major*	133	181	313
LmjF26.2370	*L. major*	42	335	376
LmjF26.2370	*L. major*	70	413	482
LmjF26.2370	*L. major*	61	527	587
LmjF26.2370	*L. major*	162	610	771
LmjF26.2370	*L. major*	66	817	882
LmjF26.2370	*L. major*	94	936	1029
LmjF26.2370	*L. major*	52	1202	1253
LmjF26.2370	*L. major*	49	1381	1429
LmjF26.2370	*L. major*	49	1580	1628
LmjF26.2370	*L. major*	65	1785	1849
LmjF26.2370	*L. major*	55	2238	2292
LmjF26.2370	*L. major*	154	2447	2600
LmjF26.2370	*L. major*	42	3228	3269
LmjF26.2370	*L. major*	140	3427	3566
LmjF26.2380	*L. major*	61	15	75
LmjF26.2380	*L. major*	73	97	169
LmjF26.2380	*L. major*	55	206	260

LmjF26.2380	*L. major*	53	565	617
LmjF26.2380	*L. major*	68	1975	2042
LmjF26.2380	*L. major*	46	2465	2510
LmjF26.2380	*L. major*	85	2577	2661
LmjF26.2380	*L. major*	46	2742	2787
LmjF26.2380	*L. major*	114	3035	3148
LmjF26.2380	*L. major*	69	3532	3600
LmjF26.2380	*L. major*	89	3792	3880
LmjF26.2380	*L. major*	68	4135	4202
LmjF26.2380	*L. major*	113	4428	4540
LmjF26.2380	*L. major*	40	4575	4614
LmjF26.2380	*L. major*	40	4789	4828
LmjF26.2380	*L. major*	126	4949	5074
LmjF26.2390	*L. major*	209	9	217
LmjF26.2390	*L. major*	125	351	475
LmjF26.2390	*L. major*	292	517	808
LmjF26.2410	*L. major*	44	32	75
LmjF26.2410	*L. major*	63	136	198
LmjF26.2410	*L. major*	63	136	198
LmjF26.2420	*L. major*	126	146	271
LmjF26.2440	*L. major*	111	74	184
LmjF26.2440	*L. major*	101	189	289
LmjF26.2440	*L. major*	81	547	627
LmjF26.2440	*L. major*	185	664	848
LmjF26.2440	*L. major*	188	856	1043
LmjF26.2450	*L. major*	68	85	152
LmjF26.2450	*L. major*	53	169	221
LmjF26.2460	*L. major*	81	20	100
LmjF26.2490	*L. major*	95	1	95
LmjF26.2490	*L. major*	220	134	353
LmjF26.2490	*L. major*	316	362	677
LmjF26.2490	*L. major*	134	695	828
LmjF26.2490	*L. major*	537	836	1372
LmjF26.2490	*L. major*	61	1401	1461
LmjF26.2520	*L. major*	131	75	205
LmjF26.2520	*L. major*	192	298	489
LmjF26.2530	*L. major*	126	374	499
LmjF26.2570	*L. major*	45	312	356
LmjF26.2580	*L. major*	151	1	151
LmjF26.2590	*L. major*	55	593	647
LmjF26.2600	*L. major*	230	1	230
LmjF26.2600	*L. major*	51	258	308
LmjF26.2600	*L. major*	259	443	701
LmjF26.2640	*L. major*	109	118	226
LmjF26.2640	*L. major*	77	245	321
LmjF26.2650	*L. major*	69	1	69
LmjF26.2650	*L. major*	68	108	175

LmjF26.2650	*L. major*	120	227	346
LmjF26.2660	*L. major*	599	1	599
LmjF26.2660	*L. major*	64	601	664
LmjF27.0010	*L. major*	97	115	211
LmjF27.0050	*L. major*	128	1	128
LmjF27.0050	*L. major*	59	417	475
LmjF27.0050	*L. major*	76	713	788
LmjF27.0100	*L. major*	337	324	660
LmjF27.0120	*L. major*	54	367	420
LmjF27.0130	*L. major*	44	1	44
LmjF27.0140	*L. major*	87	67	153
LmjF27.0150	*L. major*	115	69	183
LmjF27.0170	*L. major*	46	1	46
LmjF27.0170	*L. major*	49	86	134
LmjF27.0170	*L. major*	49	86	134
LmjF27.0180	*L. major*	44	136	179
LmjF27.0200	*L. major*	79	254	332
LmjF27.0210	*L. major*	52	1	52
LmjF27.0220	*L. major*	54	140	193
LmjF27.0220	*L. major*	56	209	264
LmjF27.0220	*L. major*	56	209	264
LmjF27.0240	*L. major*	729	1	729
LmjF27.0250	*L. major*	59	92	150
LmjF27.0250	*L. major*	40	356	395
LmjF27.0250	*L. major*	40	356	395
LmjF27.0270	*L. major*	148	1	148
LmjF27.0270	*L. major*	82	203	284
LmjF27.0270	*L. major*	58	332	389
LmjF27.0270	*L. major*	350	419	768
LmjF27.0270	*L. major*	161	783	943
LmjF27.0270	*L. major*	97	964	1060
LmjF27.0290	*L. major*	44	1	44
LmjF27.0310	*L. major*	40	636	675
LmjF27.0320	*L. major*	59	436	494
LmjF27.0330	*L. major*	113	13	125
LmjF27.0330	*L. major*	138	234	371
LmjF27.0340	*L. major*	52	1	52
LmjF27.0350	*L. major*	57	321	377
LmjF27.0380	*L. major*	596	1	596
LmjF27.0380	*L. major*	120	628	747
LmjF27.0380	*L. major*	116	862	977
LmjF27.0390	*L. major*	48	660	707
LmjF27.0390	*L. major*	111	767	877
LmjF27.0390	*L. major*	111	767	877
LmjF27.0410	*L. major*	99	30	128
LmjF27.0410	*L. major*	111	148	258
LmjF27.0430	*L. major*	610	1	610

LmjF27.0440	*L. major*	46	443	488
LmjF27.0450	*L. major*	117	1	117
LmjF27.0460	*L. major*	52	1	52
LmjF27.0460	*L. major*	133	212	344
LmjF27.0460	*L. major*	236	390	625
LmjF27.0480	*L. major*	77	355	431
LmjF27.0480	*L. major*	70	488	557
LmjF27.0490	*L. major*	52	586	637
LmjF27.0490	*L. major*	420	1196	1615
LmjF27.0490	*L. major*	112	1677	1788
LmjF27.0490	*L. major*	1569	1955	3523
LmjF27.0490	*L. major*	158	3628	3785
LmjF27.0490	*L. major*	113	3823	3935
LmjF27.0490	*L. major*	62	4492	4553
LmjF27.0500	*L. major*	75	1194	1268
LmjF27.0500	*L. major*	55	1287	1341
LmjF27.0500	*L. major*	115	1362	1476
LmjF27.0500	*L. major*	75	1483	1557
LmjF27.0500	*L. major*	59	1566	1624
LmjF27.0500	*L. major*	132	1687	1818
LmjF27.0500	*L. major*	79	1820	1898
LmjF27.0500	*L. major*	225	1926	2150
LmjF27.0500	*L. major*	497	2187	2683
LmjF27.0500	*L. major*	54	2709	2762
LmjF27.0500	*L. major*	187	2788	2974
LmjF27.0500	*L. major*	445	2976	3420
LmjF27.0500	*L. major*	293	3502	3794
LmjF27.0500	*L. major*	83	3825	3907
LmjF27.0500	*L. major*	48	3931	3978
LmjF27.0500	*L. major*	783	3994	4776
LmjF27.0500	*L. major*	716	4813	5528
LmjF27.0500	*L. major*	74	6091	6164
LmjF27.0510	*L. major*	75	1192	1266
LmjF27.0510	*L. major*	64	1350	1413
LmjF27.0510	*L. major*	191	1428	1618
LmjF27.0510	*L. major*	258	1700	1957
LmjF27.0510	*L. major*	258	2039	2296
LmjF27.0510	*L. major*	258	2378	2635
LmjF27.0510	*L. major*	206	2717	2922
LmjF27.0510	*L. major*	48	3056	3103
LmjF27.0510	*L. major*	89	3107	3195
LmjF27.0510	*L. major*	54	3197	3250
LmjF27.0510	*L. major*	86	3368	3453
LmjF27.0510	*L. major*	61	3465	3525
LmjF27.0510	*L. major*	72	3578	3649
LmjF27.0510	*L. major*	178	3674	3851
LmjF27.0510	*L. major*	56	3916	3971

LmjF27.0510	*L. major*	123	4051	4173
LmjF27.0510	*L. major*	120	4187	4306
LmjF27.0510	*L. major*	58	4317	4374
LmjF27.0510	*L. major*	49	4392	4440
LmjF27.0510	*L. major*	87	4471	4557
LmjF27.0510	*L. major*	101	4610	4710
LmjF27.0510	*L. major*	77	5282	5358
LmjF27.0520	*L. major*	169	1	169
LmjF27.0520	*L. major*	83	194	276
LmjF27.0530	*L. major*	125	153	277
LmjF27.0530	*L. major*	109	306	414
LmjF27.0530	*L. major*	117	426	542
LmjF27.0570	*L. major*	41	1	41
LmjF27.0570	*L. major*	169	190	358
LmjF27.0570	*L. major*	162	388	549
LmjF27.0580	*L. major*	58	217	274
LmjF27.0590	*L. major*	106	39	144
LmjF27.0600	*L. major*	378	1	378
LmjF27.0600	*L. major*	79	414	492
LmjF27.0600	*L. major*	89	647	735
LmjF27.0600	*L. major*	196	793	988
LmjF27.0610	*L. major*	182	74	255
LmjF27.0610	*L. major*	200	263	462
LmjF27.0620	*L. major*	45	1	45
LmjF27.0620	*L. major*	66	149	214
LmjF27.0620	*L. major*	66	149	214
LmjF27.0630	*L. major*	56	524	579
LmjF27.0640	*L. major*	44	118	161
LmjF27.0640	*L. major*	71	192	262
LmjF27.0640	*L. major*	71	192	262
LmjF27.0660	*L. major*	92	206	297
LmjF27.0660	*L. major*	170	336	505
LmjF27.0670	*L. major*	59	16	74
LmjF27.0680	*L. major*	96	1	96
LmjF27.0690	*L. major*	42	1	42
LmjF27.0690	*L. major*	80	340	419
LmjF27.0690	*L. major*	80	340	419
LmjF27.0700	*L. major*	176	1	176
LmjF27.0700	*L. major*	116	344	459
LmjF27.0720	*L. major*	326	66	391
LmjF27.0740	*L. major*	47	1	47
LmjF27.0770	*L. major*	52	731	782
LmjF27.0770	*L. major*	262	788	1049
LmjF27.0770	*L. major*	68	1207	1274
LmjF27.0780	*L. major*	61	1	61
LmjF27.0780	*L. major*	42	154	195
LmjF27.0780	*L. major*	42	154	195

LmjF27.0790	*L. major*	192	1	192
LmjF27.0790	*L. major*	99	239	337
LmjF27.0790	*L. major*	73	819	891
LmjF27.0800	*L. major*	111	16	126
LmjF27.0820	*L. major*	58	194	251
LmjF27.0820	*L. major*	87	347	433
LmjF27.0820	*L. major*	46	967	1012
LmjF27.0820	*L. major*	58	1827	1884
LmjF27.0820	*L. major*	110	2835	2944
LmjF27.0820	*L. major*	158	3119	3276
LmjF27.0830	*L. major*	96	85	180
LmjF27.0830	*L. major*	115	209	323
LmjF27.0850	*L. major*	80	56	135
LmjF27.0850	*L. major*	99	228	326
LmjF27.0850	*L. major*	79	493	571
LmjF27.0860	*L. major*	41	804	844
LmjF27.0870	*L. major*	45	380	424
LmjF27.0870	*L. major*	42	623	664
LmjF27.0870	*L. major*	42	623	664
LmjF27.0880	*L. major*	59	555	613
LmjF27.0880	*L. major*	44	949	992
LmjF27.0880	*L. major*	44	949	992
LmjF27.0890	*L. major*	52	42	93
LmjF27.0890	*L. major*	218	153	370
LmjF27.0890	*L. major*	70	780	849
LmjF27.0890	*L. major*	120	1080	1199
LmjF27.0890	*L. major*	92	1301	1392
LmjF27.0890	*L. major*	42	1517	1558
LmjF27.0890	*L. major*	41	1756	1796
LmjF27.0910	*L. major*	47	336	382
LmjF27.0910	*L. major*	42	414	455
LmjF27.0910	*L. major*	42	414	455
LmjF27.0940	*L. major*	41	404	444
LmjF27.0940	*L. major*	107	479	585
LmjF27.0950	*L. major*	57	1	57
LmjF27.0950	*L. major*	104	195	298
LmjF27.0950	*L. major*	273	346	618
LmjF27.0960	*L. major*	86	676	761
LmjF27.0960	*L. major*	42	836	877
LmjF27.0960	*L. major*	45	1002	1046
LmjF27.0970	*L. major*	144	1	144
LmjF27.0970	*L. major*	60	1785	1844
LmjF27.0970	*L. major*	60	1785	1844
LmjF27.0980	*L. major*	43	1	43
LmjF27.0980	*L. major*	85	88	172
LmjF27.0980	*L. major*	54	1827	1880
LmjF27.0990	*L. major*	43	1	43

LmjF27.0990	*L. major*	46	82	127
LmjF27.0990	*L. major*	47	194	240
LmjF27.1010	*L. major*	45	1	45
LmjF27.1010	*L. major*	60	54	113
LmjF27.1010	*L. major*	103	832	934
LmjF27.1030	*L. major*	156	1	156
LmjF27.1030	*L. major*	98	453	550
LmjF27.1030	*L. major*	58	756	813
LmjF27.1040	*L. major*	121	1	121
LmjF27.1040	*L. major*	99	286	384
LmjF27.1045	*L. major*	45	1	45
LmjF27.1050	*L. major*	89	1	89
LmjF27.1050	*L. major*	72	595	666
LmjF27.1070	*L. major*	65	211	275
LmjF27.1070	*L. major*	97	552	648
LmjF27.1070	*L. major*	97	552	648
LmjF27.1080	*L. major*	104	1	104
LmjF27.1080	*L. major*	40	226	265
LmjF27.1080	*L. major*	65	605	669
LmjF27.1080	*L. major*	45	841	885
LmjF27.1100	*L. major*	72	1	72
LmjF27.1100	*L. major*	81	146	226
LmjF27.1100	*L. major*	382	266	647
LmjF27.1110	*L. major*	103	1	103
LmjF27.1110	*L. major*	54	181	234
LmjF27.1120	*L. major*	82	140	221
LmjF27.1130	*L. major*	169	3	171
LmjF27.1130	*L. major*	107	705	811
LmjF27.1140	*L. major*	57	15	71
LmjF27.1140	*L. major*	40	99	138
LmjF27.1140	*L. major*	420	164	583
LmjF27.1150	*L. major*	77	60	136
LmjF27.1160	*L. major*	60	1	60
LmjF27.1160	*L. major*	60	123	182
LmjF27.1160	*L. major*	54	750	803
LmjF27.1170	*L. major*	51	45	95
LmjF27.1170	*L. major*	116	130	245
LmjF27.1180	*L. major*	48	145	192
LmjF27.1200	*L. major*	214	1	214
LmjF27.1200	*L. major*	111	305	415
LmjF27.1210	*L. major*	146	1	146
LmjF27.1210	*L. major*	58	449	506
LmjF27.1220	*L. major*	128	1	128
LmjF27.1220	*L. major*	58	193	250
LmjF27.1220	*L. major*	54	359	412
LmjF27.1230	*L. major*	51	1	51
LmjF27.1230	*L. major*	122	168	289

LmjF27.1230	*L. major*	187	332	518
LmjF27.1270	*L. major*	141	91	231
LmjF27.1275	*L. major*	160	1	160
LmjF27.1275	*L. major*	201	243	443
LmjF27.1290	*L. major*	62	1	62
LmjF27.1300	*L. major*	163	22	184
LmjF27.1300	*L. major*	52	478	529
LmjF27.1310	*L. major*	46	489	534
LmjF27.1320	*L. major*	40	119	158
LmjF27.1320	*L. major*	77	193	269
LmjF27.1320	*L. major*	74	527	600
LmjF27.1340	*L. major*	49	59	107
LmjF27.1340	*L. major*	114	229	342
LmjF27.1350	*L. major*	264	54	317
LmjF27.1360	*L. major*	67	46	112
LmjF27.1360	*L. major*	42	211	252
LmjF27.1360	*L. major*	424	263	686
LmjF27.1370	*L. major*	545	1	545
LmjF27.1370	*L. major*	574	795	1368
LmjF27.1370	*L. major*	107	1385	1491
LmjF27.1380	*L. major*	57	267	323
LmjF27.1390	*L. major*	57	267	323
LmjF27.1410	*L. major*	65	105	169
LmjF27.1420	*L. major*	96	53	148
LmjF27.1420	*L. major*	106	158	263
LmjF27.1430	*L. major*	57	248	304
LmjF27.1430	*L. major*	71	361	431
LmjF27.1430	*L. major*	116	452	567
LmjF27.1430	*L. major*	57	594	650
LmjF27.1430	*L. major*	56	803	858
LmjF27.1440	*L. major*	215	1	215
LmjF27.1450	*L. major*	205	649	853
LmjF27.1460	*L. major*	47	288	334
LmjF27.1490	*L. major*	262	159	420
LmjF27.1490	*L. major*	50	447	496
LmjF27.1490	*L. major*	96	573	668
LmjF27.1520	*L. major*	46	179	224
LmjF27.1520	*L. major*	98	306	403
LmjF27.1550	*L. major*	48	87	134
LmjF27.1560	*L. major*	54	773	826
LmjF27.1570	*L. major*	49	89	137
LmjF27.1570	*L. major*	115	212	326
LmjF27.1570	*L. major*	115	212	326
LmjF27.1580	*L. major*	50	1	50
LmjF27.1600	*L. major*	41	135	175
LmjF27.1630	*L. major*	50	1	50
LmjF27.1630	*L. major*	67	119	185

LmjF27.1630	*L. major*	76	359	434
LmjF27.1630	*L. major*	78	764	841
LmjF27.1640	*L. major*	43	434	476
LmjF27.1640	*L. major*	111	585	695
LmjF27.1640	*L. major*	180	735	914
LmjF27.1650	*L. major*	102	77	178
LmjF27.1650	*L. major*	121	205	325
LmjF27.1650	*L. major*	246	340	585
LmjF27.1650	*L. major*	98	613	710
LmjF27.1650	*L. major*	193	716	908
LmjF27.1650	*L. major*	168	936	1103
LmjF27.1650	*L. major*	51	1174	1224
LmjF27.1650	*L. major*	49	1242	1290
LmjF27.1660	*L. major*	53	1100	1152
LmjF27.1670	*L. major*	51	272	322
LmjF27.1680	*L. major*	80	1	80
LmjF27.1680	*L. major*	49	307	355
LmjF27.1700	*L. major*	86	1	86
LmjF27.1710	*L. major*	82	1	82
LmjF27.1730	*L. major*	53	123	175
LmjF27.1730	*L. major*	126	215	340
LmjF27.1750	*L. major*	53	73	125
LmjF27.1750	*L. major*	78	189	266
LmjF27.1750	*L. major*	122	3033	3154
LmjF27.1750	*L. major*	79	3261	3339
LmjF27.1750	*L. major*	64	3551	3614
LmjF27.1750	*L. major*	48	4144	4191
LmjF27.1770	*L. major*	65	380	444
LmjF27.1770	*L. major*	59	675	733
LmjF27.1770	*L. major*	59	675	733
LmjF27.1780	*L. major*	97	1	97
LmjF27.1780	*L. major*	44	156	199
LmjF27.1780	*L. major*	50	414	463
LmjF27.1780	*L. major*	49	512	560
LmjF27.1790	*L. major*	499	150	648
LmjF27.1790	*L. major*	132	711	842
LmjF27.1800	*L. major*	634	331	964
LmjF27.1800	*L. major*	351	973	1323
LmjF27.1800	*L. major*	398	1331	1728
LmjF27.1820	*L. major*	133	1	133
LmjF27.1820	*L. major*	65	136	200
LmjF27.1820	*L. major*	129	219	347
LmjF27.1820	*L. major*	242	384	625
LmjF27.1820	*L. major*	879	629	1507
LmjF27.1820	*L. major*	60	1582	1641
LmjF27.1820	*L. major*	403	1674	2076
LmjF27.1820	*L. major*	161	2097	2257

LmjF27.1840	*L. major*	131	71	201
LmjF27.1840	*L. major*	66	237	302
LmjF27.1840	*L. major*	91	306	396
LmjF27.1840	*L. major*	89	458	546
LmjF27.1840	*L. major*	44	550	593
LmjF27.1840	*L. major*	295	628	922
LmjF27.1840	*L. major*	130	1136	1265
LmjF27.1840	*L. major*	169	1396	1564
LmjF27.1850	*L. major*	118	266	383
LmjF27.1850	*L. major*	50	446	495
LmjF27.1850	*L. major*	158	564	721
LmjF27.1860	*L. major*	232	1	232
LmjF27.1860	*L. major*	132	312	443
LmjF27.1860	*L. major*	292	462	753
LmjF27.1860	*L. major*	60	789	848
LmjF27.1880	*L. major*	43	44	86
LmjF27.1880	*L. major*	88	437	524
LmjF27.1880	*L. major*	88	437	524
LmjF27.1890	*L. major*	337	504	840
LmjF27.1895	*L. major*	110	95	204
LmjF27.1895	*L. major*	56	242	297
LmjF27.1895	*L. major*	45	329	373
LmjF27.1895	*L. major*	70	723	792
LmjF27.1900	*L. major*	55	1	55
LmjF27.1910	*L. major*	73	68	140
LmjF27.1920	*L. major*	49	1	49
LmjF27.1920	*L. major*	46	420	465
LmjF27.1920	*L. major*	72	601	672
LmjF27.1930	*L. major*	61	294	354
LmjF27.1940	*L. major*	101	1	101
LmjF27.1940	*L. major*	124	125	248
LmjF27.1940	*L. major*	115	373	487
LmjF27.1940	*L. major*	212	749	960
LmjF27.1945	*L. major*	210	18	227
LmjF27.1950	*L. major*	61	65	125
LmjF27.1950	*L. major*	84	199	282
LmjF27.1950	*L. major*	227	566	792
LmjF27.1950	*L. major*	104	820	923
LmjF27.1950	*L. major*	101	970	1070
LmjF27.1950	*L. major*	162	1285	1446
LmjF27.1960	*L. major*	206	1	206
LmjF27.1960	*L. major*	176	256	431
LmjF27.1980	*L. major*	135	328	462
LmjF27.1980	*L. major*	72	476	547
LmjF27.1980	*L. major*	215	559	773
LmjF27.1980	*L. major*	100	825	924
LmjF27.1990	*L. major*	116	43	158

LmjF27.1990	*L. major*	65	176	240
LmjF27.1990	*L. major*	98	263	360
LmjF27.1990	*L. major*	94	425	518
LmjF27.1990	*L. major*	134	591	724
LmjF27.1990	*L. major*	107	890	996
LmjF27.2010	*L. major*	97	1	97
LmjF27.2010	*L. major*	126	126	251
LmjF27.2040	*L. major*	49	179	227
LmjF27.2050	*L. major*	72	1	72
LmjF27.2060	*L. major*	46	75	120
LmjF27.2060	*L. major*	67	191	257
LmjF27.2060	*L. major*	95	288	382
LmjF27.2070	*L. major*	133	1	133
LmjF27.2070	*L. major*	293	430	722
LmjF27.2070	*L. major*	49	804	852
LmjF27.2100	*L. major*	57	83	139
LmjF27.2100	*L. major*	50	198	247
LmjF27.2100	*L. major*	128	312	439
LmjF27.2110	*L. major*	59	1	59
LmjF27.2110	*L. major*	43	180	222
LmjF27.2110	*L. major*	68	368	435
LmjF27.2110	*L. major*	140	578	717
LmjF27.2120	*L. major*	103	184	286
LmjF27.2130	*L. major*	59	1	59
LmjF27.2130	*L. major*	134	108	241
LmjF27.2130	*L. major*	163	243	405
LmjF27.2130	*L. major*	60	467	526
LmjF27.2130	*L. major*	74	632	705
LmjF27.2130	*L. major*	52	876	927
LmjF27.2130	*L. major*	244	1186	1429
LmjF27.2130	*L. major*	185	1479	1663
LmjF27.2140	*L. major*	348	1	348
LmjF27.2140	*L. major*	60	398	457
LmjF27.2140	*L. major*	64	484	547
LmjF27.2140	*L. major*	51	612	662
LmjF27.2140	*L. major*	65	799	863
LmjF27.2170	*L. major*	48	154	201
LmjF27.2170	*L. major*	111	388	498
LmjF27.2170	*L. major*	262	947	1208
LmjF27.2170	*L. major*	141	1210	1350
LmjF27.2170	*L. major*	175	1383	1557
LmjF27.2200	*L. major*	122	1	122
LmjF27.2210	*L. major*	60	40	99
LmjF27.2220	*L. major*	63	200	262
LmjF27.2230	*L. major*	90	1	90
LmjF27.2230	*L. major*	43	269	311
LmjF27.2230	*L. major*	43	402	444

LmjF27.2230	*L. major*	87	477	563
LmjF27.2230	*L. major*	53	623	675
LmjF27.2230	*L. major*	52	741	792
LmjF27.2230	*L. major*	45	875	919
LmjF27.2230	*L. major*	88	1243	1330
LmjF27.2230	*L. major*	96	1370	1465
LmjF27.2230	*L. major*	82	1691	1772
LmjF27.2230	*L. major*	104	1801	1904
LmjF27.2230	*L. major*	46	1918	1963
LmjF27.2240	*L. major*	125	262	386
LmjF27.2250	*L. major*	51	196	246
LmjF27.2260	*L. major*	68	209	276
LmjF27.2260	*L. major*	58	311	368
LmjF27.2260	*L. major*	55	448	502
LmjF27.2260	*L. major*	83	687	769
LmjF27.2260	*L. major*	52	955	1006
LmjF27.2260	*L. major*	74	1069	1142
LmjF27.2270	*L. major*	56	1	56
LmjF27.2270	*L. major*	379	124	502
LmjF27.2270	*L. major*	120	545	664
LmjF27.2290	*L. major*	59	661	719
LmjF27.2300	*L. major*	172	1	172
LmjF27.2300	*L. major*	51	650	700
LmjF27.2305	*L. major*	53	26	78
LmjF27.2310	*L. major*	53	69	121
LmjF27.2335	*L. major*	57	56	112
LmjF27.2350	*L. major*	48	99	146
LmjF27.2360	*L. major*	85	301	385
LmjF27.2390	*L. major*	94	1	94
LmjF27.2390	*L. major*	207	184	390
LmjF27.2400	*L. major*	54	53	106
LmjF27.2400	*L. major*	50	347	396
LmjF27.2400	*L. major*	50	347	396
LmjF27.2420	*L. major*	47	83	129
LmjF27.2420	*L. major*	48	159	206
LmjF27.2420	*L. major*	48	159	206
LmjF27.2430	*L. major*	149	1	149
LmjF27.2430	*L. major*	348	213	560
LmjF27.2450	*L. major*	77	182	258
LmjF27.2450	*L. major*	151	297	447
LmjF27.2450	*L. major*	151	297	447
LmjF27.2470	*L. major*	78	484	561
LmjF27.2480	*L. major*	103	127	229
LmjF27.2490	*L. major*	57	1	57
LmjF27.2490	*L. major*	71	381	451
LmjF27.2500	*L. major*	137	1	137
LmjF27.2590	*L. major*	47	1	47

LmjF27.2590	*L. major*	88	65	152
LmjF27.2590	*L. major*	57	596	652
LmjF27.2590	*L. major*	110	2874	2983
LmjF27.2590	*L. major*	74	3417	3490
LmjF27.2610	*L. major*	97	1	97
LmjF27.2610	*L. major*	52	125	176
LmjF27.2610	*L. major*	76	194	269
LmjF27.2620	*L. major*	54	1	54
LmjF27.2620	*L. major*	162	116	277
LmjF27.2620	*L. major*	61	280	340
LmjF27.2620	*L. major*	75	350	424
LmjF27.2620	*L. major*	120	595	714
LmjF27.2620	*L. major*	157	781	937
LmjF27.2630	*L. major*	102	1	102
LmjF27.2630	*L. major*	88	335	422
LmjF27.2630	*L. major*	88	335	422
LmjF27.2660	*L. major*	53	1	53
LmjF28.0010	*L. major*	70	481	550
LmjF28.0040	*L. major*	225	1	225
LmjF28.0040	*L. major*	68	227	294
LmjF28.0040	*L. major*	59	436	494
LmjF28.0050	*L. major*	267	110	376
LmjF28.0060	*L. major*	68	1	68
LmjF28.0080	*L. major*	91	39	129
LmjF28.0150	*L. major*	41	488	528
LmjF28.0160	*L. major*	74	1	74
LmjF28.0170	*L. major*	99	1	99
LmjF28.0190	*L. major*	48	1	48
LmjF28.0190	*L. major*	154	165	318
LmjF28.0190	*L. major*	69	392	460
LmjF28.0200	*L. major*	96	67	162
LmjF28.0210	*L. major*	46	1	46
LmjF28.0220	*L. major*	60	9	68
LmjF28.0230	*L. major*	172	1	172
LmjF28.0230	*L. major*	94	178	271
LmjF28.0260	*L. major*	46	118	163
LmjF28.0260	*L. major*	77	845	921
LmjF28.0260	*L. major*	77	845	921
LmjF28.0270	*L. major*	43	173	215
LmjF28.0280	*L. major*	82	73	154
LmjF28.0290	*L. major*	50	138	187
LmjF28.0300	*L. major*	120	1	120
LmjF28.0300	*L. major*	118	295	412
LmjF28.0300	*L. major*	62	481	542
LmjF28.0300	*L. major*	60	572	631
LmjF28.0330	*L. major*	60	861	920
LmjF28.0390	*L. major*	79	1	79

LmjF28.0390	*L. major*	51	100	150
LmjF28.0390	*L. major*	46	177	222
LmjF28.0390	*L. major*	139	227	365
LmjF28.0390	*L. major*	59	466	524
LmjF28.0390	*L. major*	69	723	791
LmjF28.0390	*L. major*	106	824	929
LmjF28.0400	*L. major*	158	77	234
LmjF28.0410	*L. major*	44	34	77
LmjF28.0410	*L. major*	70	284	353
LmjF28.0430	*L. major*	64	1	64
LmjF28.0430	*L. major*	53	230	282
LmjF28.0430	*L. major*	53	230	282
LmjF28.0440	*L. major*	44	233	276
LmjF28.0440	*L. major*	41	469	509
LmjF28.0440	*L. major*	41	469	509
LmjF28.0450	*L. major*	56	81	136
LmjF28.0450	*L. major*	56	814	869
LmjF28.0460	*L. major*	99	1	99
LmjF28.0460	*L. major*	72	240	311
LmjF28.0470	*L. major*	55	707	761
LmjF28.0480	*L. major*	169	367	535
LmjF28.0485	*L. major*	42	49	90
LmjF28.0485	*L. major*	81	147	227
LmjF28.0485	*L. major*	81	147	227
LmjF28.0490	*L. major*	59	1	59
LmjF28.0500	*L. major*	45	1	45
LmjF28.0500	*L. major*	121	135	255
LmjF28.0500	*L. major*	121	135	255
LmjF28.0510	*L. major*	45	341	385
LmjF28.0530	*L. major*	358	198	555
LmjF28.0530	*L. major*	70	571	640
LmjF28.0530	*L. major*	45	701	745
LmjF28.0530	*L. major*	291	819	1109
LmjF28.0550	*L. major*	58	1	58
LmjF28.0560	*L. major*	50	165	214
LmjF28.0560	*L. major*	41	246	286
LmjF28.0560	*L. major*	41	246	286
LmjF28.0580	*L. major*	70	146	215
LmjF28.0580	*L. major*	57	301	357
LmjF28.0580	*L. major*	614	404	1017
LmjF28.0580	*L. major*	452	1037	1488
LmjF28.0580	*L. major*	73	1507	1579
LmjF28.0590	*L. major*	90	1	90
LmjF28.0590	*L. major*	196	127	322
LmjF28.0590	*L. major*	52	353	404
LmjF28.0590	*L. major*	95	407	501
LmjF28.0610	*L. major*	97	1	97

LmjF28.0610	*L. major*	57	817	873
LmjF28.0610	*L. major*	109	2770	2878
LmjF28.0610	*L. major*	78	3313	3390
LmjF28.0620	*L. major*	134	297	430
LmjF28.0620	*L. major*	66	1226	1291
LmjF28.0670	*L. major*	40	80	119
LmjF28.0680	*L. major*	92	142	233
LmjF28.0700	*L. major*	166	251	416
LmjF28.0705	*L. major*	133	117	249
LmjF28.0720	*L. major*	40	1	40
LmjF28.0720	*L. major*	104	43	146
LmjF28.0720	*L. major*	294	267	560
LmjF28.0740	*L. major*	51	69	119
LmjF28.0740	*L. major*	122	181	302
LmjF28.0740	*L. major*	43	352	394
LmjF28.0760	*L. major*	77	660	736
LmjF28.0780	*L. major*	44	41	84
LmjF28.0780	*L. major*	64	377	440
LmjF28.0780	*L. major*	64	377	440
LmjF28.0785	*L. major*	41	150	190
LmjF28.0820	*L. major*	107	8	114
LmjF28.0820	*L. major*	296	195	490
LmjF28.0822	*L. major*	49	119	167
LmjF28.0825	*L. major*	82	61	142
LmjF28.0830	*L. major*	75	29	103
LmjF28.0835	*L. major*	65	48	112
LmjF28.0840	*L. major*	56	1	56
LmjF28.0840	*L. major*	72	173	244
LmjF28.0840	*L. major*	78	781	858
LmjF28.0850	*L. major*	183	1	183
LmjF28.0850	*L. major*	74	706	779
LmjF28.0860	*L. major*	366	71	436
LmjF28.0870	*L. major*	116	99	214
LmjF28.0900	*L. major*	66	45	110
LmjF28.0900	*L. major*	173	118	290
LmjF28.0900	*L. major*	173	118	290
LmjF28.0910	*L. major*	48	1	48
LmjF28.0910	*L. major*	251	59	309
LmjF28.0910	*L. major*	192	400	591
LmjF28.0910	*L. major*	88	727	814
LmjF28.0910	*L. major*	516	847	1362
LmjF28.0920	*L. major*	64	126	189
LmjF28.0930	*L. major*	43	141	183
LmjF28.0930	*L. major*	77	335	411
LmjF28.0930	*L. major*	77	335	411
LmjF28.0940	*L. major*	121	95	215
LmjF28.0940	*L. major*	68	224	291

LmjF28.0960	*L. major*	49	96	144
LmjF28.0970	*L. major*	50	82	131
LmjF28.1000	*L. major*	46	147	192
LmjF28.1000	*L. major*	49	636	684
LmjF28.1000	*L. major*	69	863	931
LmjF28.1020	*L. major*	42	1	42
LmjF28.1020	*L. major*	82	165	246
LmjF28.1020	*L. major*	40	337	376
LmjF28.1040	*L. major*	112	1	112
LmjF28.1040	*L. major*	83	114	196
LmjF28.1040	*L. major*	94	254	347
LmjF28.1040	*L. major*	160	400	559
LmjF28.1040	*L. major*	259	581	839
LmjF28.1050	*L. major*	69	1	69
LmjF28.1050	*L. major*	57	181	237
LmjF28.1050	*L. major*	62	311	372
LmjF28.1060	*L. major*	405	1	405
LmjF28.1070	*L. major*	332	1	332
LmjF28.1070	*L. major*	41	632	672
LmjF28.1070	*L. major*	57	722	778
LmjF28.1070	*L. major*	44	806	849
LmjF28.1070	*L. major*	84	943	1026
LmjF28.1070	*L. major*	72	1100	1171
LmjF28.1070	*L. major*	48	1253	1300
LmjF28.1080	*L. major*	75	417	491
LmjF28.1080	*L. major*	91	523	613
LmjF28.1090	*L. major*	44	431	474
LmjF28.1090	*L. major*	60	671	730
LmjF28.1090	*L. major*	60	671	730
LmjF28.1100	*L. major*	208	198	405
LmjF28.1100	*L. major*	54	490	543
LmjF28.1100	*L. major*	52	711	762
LmjF28.1130	*L. major*	84	1	84
LmjF28.1150	*L. major*	63	252	314
LmjF28.1150	*L. major*	42	516	557
LmjF28.1180	*L. major*	90	82	171
LmjF28.1200	*L. major*	113	515	627
LmjF28.1210	*L. major*	273	9	281
LmjF28.1210	*L. major*	293	307	599
LmjF28.1210	*L. major*	55	610	664
LmjF28.1210	*L. major*	44	700	743
LmjF28.1220	*L. major*	145	165	309
LmjF28.1220	*L. major*	69	404	472
LmjF28.1250	*L. major*	127	100	226
LmjF28.1250	*L. major*	117	322	438
LmjF28.1260	*L. major*	116	1	116
LmjF28.1260	*L. major*	123	145	267

LmjF28.1260	*L. major*	90	280	369
LmjF28.1260	*L. major*	63	392	454
LmjF28.1260	*L. major*	42	458	499
LmjF28.1260	*L. major*	55	759	813
LmjF28.1260	*L. major*	53	853	905
LmjF28.1260	*L. major*	108	1003	1110
LmjF28.1270	*L. major*	88	97	184
LmjF28.1270	*L. major*	53	291	343
LmjF28.1290	*L. major*	62	258	319
LmjF28.1290	*L. major*	58	346	403
LmjF28.1300	*L. major*	146	1	146
LmjF28.1310	*L. major*	74	1	74
LmjF28.1310	*L. major*	82	538	619
LmjF28.1310	*L. major*	264	637	900
LmjF28.1320	*L. major*	59	1	59
LmjF28.1330	*L. major*	72	1	72
LmjF28.1330	*L. major*	110	91	200
LmjF28.1380	*L. major*	48	97	144
LmjF28.1390	*L. major*	112	1	112
LmjF28.1400	*L. major*	292	1	292
LmjF28.1405	*L. major*	339	60	398
LmjF28.1410	*L. major*	143	1	143
LmjF28.1410	*L. major*	47	146	192
LmjF28.1410	*L. major*	151	601	751
LmjF28.1420	*L. major*	64	1	64
LmjF28.1420	*L. major*	52	78	129
LmjF28.1420	*L. major*	109	490	598
LmjF28.1430	*L. major*	64	215	278
LmjF28.1440	*L. major*	58	1	58
LmjF28.1440	*L. major*	68	296	363
LmjF28.1440	*L. major*	68	296	363
LmjF28.1450	*L. major*	143	1	143
LmjF28.1450	*L. major*	98	184	281
LmjF28.1450	*L. major*	52	290	341
LmjF28.1450	*L. major*	44	479	522
LmjF28.1450	*L. major*	61	550	610
LmjF28.1450	*L. major*	75	639	713
LmjF28.1460	*L. major*	46	517	562
LmjF28.1470	*L. major*	62	1	62
LmjF28.1470	*L. major*	50	90	139
LmjF28.1480	*L. major*	62	1	62
LmjF28.1480	*L. major*	50	90	139
LmjF28.1515	*L. major*	160	1	160
LmjF28.1520	*L. major*	122	139	260
LmjF28.1520	*L. major*	353	289	641
LmjF28.1520	*L. major*	353	289	641
LmjF28.1540	*L. major*	69	1	69

LmjF28.1540	*L. major*	62	75	136
LmjF28.1540	*L. major*	102	167	268
LmjF28.1540	*L. major*	92	272	363
LmjF28.1540	*L. major*	90	588	677
LmjF28.1540	*L. major*	46	682	727
LmjF28.1540	*L. major*	94	763	856
LmjF28.1560	*L. major*	186	27	212
LmjF28.1560	*L. major*	117	237	353
LmjF28.1560	*L. major*	154	408	561
LmjF28.1600	*L. major*	48	1	48
LmjF28.1600	*L. major*	76	91	166
LmjF28.1600	*L. major*	48	469	516
LmjF28.1600	*L. major*	59	942	1000
LmjF28.1600	*L. major*	58	1015	1072
LmjF28.1610	*L. major*	110	36	145
LmjF28.1610	*L. major*	165	198	362
LmjF28.1610	*L. major*	165	198	362
LmjF28.1620	*L. major*	190	48	237
LmjF28.1620	*L. major*	46	730	775
LmjF28.1620	*L. major*	67	782	848
LmjF28.1630	*L. major*	42	253	294
LmjF28.1630	*L. major*	61	435	495
LmjF28.1630	*L. major*	94	532	625
LmjF28.1640	*L. major*	51	1	51
LmjF28.1640	*L. major*	50	161	210
LmjF28.1640	*L. major*	59	314	372
LmjF28.1640	*L. major*	102	433	534
LmjF28.1640	*L. major*	490	757	1246
LmjF28.1640	*L. major*	100	1260	1359
LmjF28.1650	*L. major*	260	226	485
LmjF28.1650	*L. major*	243	508	750
LmjF28.1660	*L. major*	53	62	114
LmjF28.1660	*L. major*	159	147	305
LmjF28.1670	*L. major*	86	1	86
LmjF28.1670	*L. major*	195	256	450
LmjF28.1670	*L. major*	100	539	638
LmjF28.1670	*L. major*	62	987	1048
LmjF28.1670	*L. major*	126	1186	1311
LmjF28.1690	*L. major*	106	621	726
LmjF28.1690	*L. major*	65	839	903
LmjF28.1690	*L. major*	96	1051	1146
LmjF28.1690	*L. major*	179	1198	1376
LmjF28.1690	*L. major*	90	1705	1794
LmjF28.1690	*L. major*	131	1821	1951
LmjF28.1700	*L. major*	95	1	95
LmjF28.1700	*L. major*	55	167	221
LmjF28.1700	*L. major*	41	312	352

LmjF28.1700	*L. major*	77	395	471
LmjF28.1710	*L. major*	70	1	70
LmjF28.1710	*L. major*	53	101	153
LmjF28.1710	*L. major*	223	197	419
LmjF28.1710	*L. major*	130	439	568
LmjF28.1710	*L. major*	135	600	734
LmjF28.1710	*L. major*	84	764	847
LmjF28.1710	*L. major*	227	905	1131
LmjF28.1710	*L. major*	420	1169	1588
LmjF28.1710	*L. major*	45	1598	1642
LmjF28.1710	*L. major*	210	1670	1879
LmjF28.1710	*L. major*	92	2082	2173
LmjF28.1720	*L. major*	147	7	153
LmjF28.1730	*L. major*	104	803	906
LmjF28.1740	*L. major*	128	114	241
LmjF28.1740	*L. major*	41	275	315
LmjF28.1740	*L. major*	90	398	487
LmjF28.1750	*L. major*	71	72	142
LmjF28.1750	*L. major*	128	200	327
LmjF28.1750	*L. major*	69	376	444
LmjF28.1750	*L. major*	42	512	553
LmjF28.1750	*L. major*	61	565	625
LmjF28.1750	*L. major*	72	642	713
LmjF28.1750	*L. major*	84	841	924
LmjF28.1760	*L. major*	145	517	661
LmjF28.1760	*L. major*	149	870	1018
LmjF28.1760	*L. major*	119	1031	1149
LmjF28.1770	*L. major*	177	85	261
LmjF28.1780	*L. major*	40	687	726
LmjF28.1780	*L. major*	41	826	866
LmjF28.1780	*L. major*	41	826	866
LmjF28.1790	*L. major*	41	118	158
LmjF28.1800	*L. major*	137	1	137
LmjF28.1800	*L. major*	98	302	399
LmjF28.1800	*L. major*	85	472	556
LmjF28.1800	*L. major*	80	687	766
LmjF28.1800	*L. major*	42	1134	1175
LmjF28.1800	*L. major*	45	1225	1269
LmjF28.1800	*L. major*	53	1315	1367
LmjF28.1800	*L. major*	113	1401	1513
LmjF28.1800	*L. major*	91	1546	1636
LmjF28.1800	*L. major*	115	1666	1780
LmjF28.1800	*L. major*	65	2030	2094
LmjF28.1800	*L. major*	80	2181	2260
LmjF28.1800	*L. major*	43	2391	2433
LmjF28.1810	*L. major*	67	34	100
LmjF28.1820	*L. major*	58	393	450

LmjF28.1830	*L. major*	119	56	174
LmjF28.1830	*L. major*	51	539	589
LmjF28.1830	*L. major*	61	657	717
LmjF28.1830	*L. major*	48	875	922
LmjF28.1850	*L. major*	85	1	85
LmjF28.1850	*L. major*	96	89	184
LmjF28.1850	*L. major*	175	253	427
LmjF28.1860	*L. major*	53	165	217
LmjF28.1870	*L. major*	148	148	295
LmjF28.1870	*L. major*	68	326	393
LmjF28.1880	*L. major*	69	1	69
LmjF28.1880	*L. major*	145	377	521
LmjF28.1880	*L. major*	289	537	825
LmjF28.1880	*L. major*	43	1110	1152
LmjF28.1880	*L. major*	84	1178	1261
LmjF28.1890	*L. major*	46	78	123
LmjF28.1900	*L. major*	451	205	655
LmjF28.1910	*L. major*	340	1	340
LmjF28.1910	*L. major*	72	529	600
LmjF28.1920	*L. major*	113	1	113
LmjF28.1930	*L. major*	43	27	69
LmjF28.1930	*L. major*	55	203	257
LmjF28.1930	*L. major*	55	203	257
LmjF28.1940	*L. major*	58	1	58
LmjF28.1940	*L. major*	68	95	162
LmjF28.1940	*L. major*	44	199	242
LmjF28.1940	*L. major*	43	323	365
LmjF28.1940	*L. major*	55	391	445
LmjF28.1960	*L. major*	115	166	280
LmjF28.1960	*L. major*	54	543	596
LmjF28.1960	*L. major*	54	543	596
LmjF28.1970	*L. major*	40	133	172
LmjF28.1980	*L. major*	219	283	501
LmjF28.1980	*L. major*	53	571	623
LmjF28.1980	*L. major*	45	924	968
LmjF28.1980	*L. major*	71	1108	1178
LmjF28.1980	*L. major*	115	1244	1358
LmjF28.1990	*L. major*	263	360	622
LmjF28.1990	*L. major*	108	640	747
LmjF28.2000	*L. major*	226	301	526
LmjF28.2000	*L. major*	51	588	638
LmjF28.2000	*L. major*	209	669	877
LmjF28.2010	*L. major*	71	58	128
LmjF28.2010	*L. major*	45	398	442
LmjF28.2010	*L. major*	45	398	442
LmjF28.2020	*L. major*	265	27	291
LmjF28.2020	*L. major*	239	327	565

LmjF28.2050	*L. major*	41	148	188
LmjF28.2050	*L. major*	44	925	968
LmjF28.2050	*L. major*	44	925	968
LmjF28.2080	*L. major*	69	476	544
LmjF28.2110	*L. major*	59	305	363
LmjF28.2140	*L. major*	47	455	501
LmjF28.2150	*L. major*	119	1	119
LmjF28.2150	*L. major*	60	523	582
LmjF28.2150	*L. major*	52	637	688
LmjF28.2170	*L. major*	128	115	242
LmjF28.2170	*L. major*	123	473	595
LmjF28.2180	*L. major*	75	1	75
LmjF28.2190	*L. major*	191	1	191
LmjF28.2190	*L. major*	45	249	293
LmjF28.2190	*L. major*	82	348	429
LmjF28.2190	*L. major*	447	466	912
LmjF28.2200	*L. major*	41	1	41
LmjF28.2200	*L. major*	62	581	642
LmjF28.2200	*L. major*	48	670	717
LmjF28.2210	*L. major*	716	1	716
LmjF28.2230	*L. major*	59	35	93
LmjF28.2230	*L. major*	369	113	481
LmjF28.2230	*L. major*	66	490	555
LmjF28.2230	*L. major*	116	565	680
LmjF28.2240	*L. major*	66	78	143
LmjF28.2270	*L. major*	149	374	522
LmjF28.2280	*L. major*	49	374	422
LmjF28.2280	*L. major*	83	1022	1104
LmjF28.2280	*L. major*	48	1111	1158
LmjF28.2280	*L. major*	325	1173	1497
LmjF28.2290	*L. major*	121	511	631
LmjF28.2290	*L. major*	51	770	820
LmjF28.2300	*L. major*	307	1	307
LmjF28.2300	*L. major*	187	339	525
LmjF28.2300	*L. major*	53	536	588
LmjF28.2310	*L. major*	66	43	108
LmjF28.2320	*L. major*	57	1	57
LmjF28.2320	*L. major*	182	86	267
LmjF28.2320	*L. major*	586	308	893
LmjF28.2330	*L. major*	52	50	101
LmjF28.2330	*L. major*	108	153	260
LmjF28.2330	*L. major*	61	360	420
LmjF28.2330	*L. major*	139	473	611
LmjF28.2330	*L. major*	47	727	773
LmjF28.2350	*L. major*	70	216	285
LmjF28.2380	*L. major*	58	545	602
LmjF28.2380	*L. major*	46	738	783

LmjF28.2380	*L. major*	70	1048	1117
LmjF28.2385	*L. major*	40	202	241
LmjF28.2385	*L. major*	88	699	786
LmjF28.2390	*L. major*	1087	102	1188
LmjF28.2390	*L. major*	145	1213	1357
LmjF28.2400	*L. major*	54	294	347
LmjF28.2410	*L. major*	48	74	121
LmjF28.2420	*L. major*	88	91	178
LmjF28.2440	*L. major*	71	117	187
LmjF28.2440	*L. major*	102	228	329
LmjF28.2440	*L. major*	67	498	564
LmjF28.2445	*L. major*	72	1	72
LmjF28.2450	*L. major*	43	197	239
LmjF28.2450	*L. major*	43	344	386
LmjF28.2450	*L. major*	43	344	386
LmjF28.2470	*L. major*	42	251	292
LmjF28.2480	*L. major*	102	131	232
LmjF28.2490	*L. major*	122	1	122
LmjF28.2490	*L. major*	267	162	428
LmjF28.2500	*L. major*	125	1	125
LmjF28.2520	*L. major*	86	1	86
LmjF28.2520	*L. major*	78	245	322
LmjF28.2520	*L. major*	88	367	454
LmjF28.2530	*L. major*	84	1	84
LmjF28.2530	*L. major*	77	147	223
LmjF28.2530	*L. major*	129	529	657
LmjF28.2540	*L. major*	63	16	78
LmjF28.2540	*L. major*	168	91	258
LmjF28.2540	*L. major*	354	271	624
LmjF28.2540	*L. major*	45	724	768
LmjF28.2540	*L. major*	42	889	930
LmjF28.2540	*L. major*	40	1001	1040
LmjF28.2540	*L. major*	52	1113	1164
LmjF28.2540	*L. major*	45	1239	1283
LmjF28.2550	*L. major*	100	222	321
LmjF28.2550	*L. major*	107	349	455
LmjF28.2550	*L. major*	56	582	637
LmjF28.2550	*L. major*	103	878	980
LmjF28.2555	*L. major*	75	69	143
LmjF28.2560	*L. major*	75	69	143
LmjF28.2565	*L. major*	163	57	219
LmjF28.2570	*L. major*	69	1	69
LmjF28.2580	*L. major*	43	134	176
LmjF28.2580	*L. major*	111	394	504
LmjF28.2580	*L. major*	89	568	656
LmjF28.2580	*L. major*	213	673	885
LmjF28.2590	*L. major*	59	64	122

LmjF28.2590	*L. major*	46	284	329
LmjF28.2590	*L. major*	46	284	329
LmjF28.2610	*L. major*	175	37	211
LmjF28.2610	*L. major*	46	228	273
LmjF28.2610	*L. major*	68	328	395
LmjF28.2610	*L. major*	46	534	579
LmjF28.2610	*L. major*	98	700	797
LmjF28.2610	*L. major*	89	810	898
LmjF28.2610	*L. major*	59	1488	1546
LmjF28.2610	*L. major*	115	1582	1696
LmjF28.2620	*L. major*	52	557	608
LmjF28.2630	*L. major*	224	1	224
LmjF28.2630	*L. major*	59	249	307
LmjF28.2630	*L. major*	75	342	416
LmjF28.2640	*L. major*	42	71	112
LmjF28.2640	*L. major*	591	172	762
LmjF28.2690	*L. major*	69	83	151
LmjF28.2690	*L. major*	48	193	240
LmjF28.2690	*L. major*	43	328	370
LmjF28.2700	*L. major*	265	144	408
LmjF28.2730	*L. major*	42	1	42
LmjF28.2730	*L. major*	55	125	179
LmjF28.2730	*L. major*	52	277	328
LmjF28.2730	*L. major*	105	499	603
LmjF28.2770	*L. major*	166	493	658
LmjF28.2780	*L. major*	166	493	658
LmjF28.2800	*L. major*	48	1	48
LmjF28.2800	*L. major*	44	455	498
LmjF28.2800	*L. major*	44	455	498
LmjF28.2810	*L. major*	97	1	97
LmjF28.2820	*L. major*	62	525	586
LmjF28.2820	*L. major*	71	592	662
LmjF28.2830	*L. major*	49	324	372
LmjF28.2830	*L. major*	72	556	627
LmjF28.2830	*L. major*	83	699	781
LmjF28.2840	*L. major*	365	127	491
LmjF28.2850	*L. major*	58	1	58
LmjF28.2850	*L. major*	41	333	373
LmjF28.2850	*L. major*	41	333	373
LmjF28.2880	*L. major*	123	82	204
LmjF28.2880	*L. major*	86	350	435
LmjF28.2880	*L. major*	78	772	849
LmjF28.2880	*L. major*	121	2736	2856
LmjF28.2880	*L. major*	53	2966	3018
LmjF28.2880	*L. major*	87	3284	3370
LmjF28.2890	*L. major*	51	1	51
LmjF28.2890	*L. major*	53	333	385

LmjF28.2890	*L. major*	53	333	385
LmjF28.2900	*L. major*	58	476	533
LmjF28.2930	*L. major*	60	62	121
LmjF28.2950	*L. major*	110	228	337
LmjF28.2970	*L. major*	93	64	156
LmjF28.2990	*L. major*	169	67	235
LmjF28.3010	*L. major*	912	94	1005
LmjF28.3030	*L. major*	45	1	45
LmjF28.3030	*L. major*	207	121	327
LmjF28.3030	*L. major*	72	373	444
LmjF28.3030	*L. major*	209	484	692
LmjF28.3030	*L. major*	71	738	808
LmjF28.3030	*L. major*	187	851	1037
LmjF28.3030	*L. major*	71	1083	1153
LmjF28.3030	*L. major*	207	1196	1402
LmjF28.3030	*L. major*	71	1448	1518
LmjF28.3030	*L. major*	1627	1561	3187
LmjF28.3030	*L. major*	71	3233	3303
LmjF28.3030	*L. major*	372	3346	3717
LmjF28.3030	*L. major*	72	3734	3805
LmjF29.0020	*L. major*	68	438	505
LmjF29.0020	*L. major*	42	623	664
LmjF29.0020	*L. major*	51	738	788
LmjF29.0020	*L. major*	140	906	1045
LmjF29.0030	*L. major*	75	393	467
LmjF29.0040	*L. major*	57	37	93
LmjF29.0040	*L. major*	46	112	157
LmjF29.0040	*L. major*	46	112	157
LmjF29.0060	*L. major*	46	345	390
LmjF29.0070	*L. major*	41	24	64
LmjF29.0070	*L. major*	76	107	182
LmjF29.0070	*L. major*	45	186	230
LmjF29.0100	*L. major*	65	1	65
LmjF29.0100	*L. major*	48	371	418
LmjF29.0100	*L. major*	48	371	418
LmjF29.0110	*L. major*	1039	1	1039
LmjF29.0110	*L. major*	50	1124	1173
LmjF29.0110	*L. major*	45	1857	1901
LmjF29.0110	*L. major*	58	2371	2428
LmjF29.0120	*L. major*	60	1	60
LmjF29.0120	*L. major*	52	300	351
LmjF29.0120	*L. major*	42	602	643
LmjF29.0130	*L. major*	40	61	100
LmjF29.0140	*L. major*	45	293	337
LmjF29.0150	*L. major*	44	1	44
LmjF29.0150	*L. major*	42	293	334
LmjF29.0150	*L. major*	42	293	334

LmjF29.0170	*L. major*	75	63	137
LmjF29.0180	*L. major*	137	1	137
LmjF29.0180	*L. major*	59	237	295
LmjF29.0180	*L. major*	154	327	480
LmjF29.0190	*L. major*	186	1	186
LmjF29.0190	*L. major*	354	202	555
LmjF29.0190	*L. major*	47	558	604
LmjF29.0190	*L. major*	68	832	899
LmjF29.0190	*L. major*	97	924	1020
LmjF29.0200	*L. major*	43	436	478
LmjF29.0200	*L. major*	54	640	693
LmjF29.0200	*L. major*	178	1319	1496
LmjF29.0220	*L. major*	246	1	246
LmjF29.0230	*L. major*	40	176	215
LmjF29.0240	*L. major*	64	22	85
LmjF29.0240	*L. major*	53	91	143
LmjF29.0240	*L. major*	93	446	538
LmjF29.0260	*L. major*	48	157	204
LmjF29.0260	*L. major*	54	207	260
LmjF29.0260	*L. major*	40	295	334
LmjF29.0260	*L. major*	41	376	416
LmjF29.0270	*L. major*	210	142	351
LmjF29.0290	*L. major*	312	54	365
LmjF29.0300	*L. major*	53	88	140
LmjF29.0320	*L. major*	85	1	85
LmjF29.0320	*L. major*	189	160	348
LmjF29.0330	*L. major*	90	1	90
LmjF29.0330	*L. major*	75	217	291
LmjF29.0330	*L. major*	60	328	387
LmjF29.0340	*L. major*	66	43	108
LmjF29.0340	*L. major*	103	240	342
LmjF29.0340	*L. major*	103	240	342
LmjF29.0350	*L. major*	143	47	189
LmjF29.0350	*L. major*	73	294	366
LmjF29.0350	*L. major*	88	783	870
LmjF29.0350	*L. major*	74	989	1062
LmjF29.0350	*L. major*	67	1258	1324
LmjF29.0370	*L. major*	140	1	140
LmjF29.0370	*L. major*	82	211	292
LmjF29.0370	*L. major*	53	334	386
LmjF29.0370	*L. major*	110	421	530
LmjF29.0370	*L. major*	50	673	722
LmjF29.0370	*L. major*	211	848	1058
LmjF29.0370	*L. major*	239	1069	1307
LmjF29.0370	*L. major*	80	1309	1388
LmjF29.0380	*L. major*	47	1	47
LmjF29.0380	*L. major*	252	54	305

LmjF29.0380	*L. major*	143	328	470
LmjF29.0380	*L. major*	135	480	614
LmjF29.0390	*L. major*	64	196	259
LmjF29.0410	*L. major*	52	28	79
LmjF29.0410	*L. major*	453	110	562
LmjF29.0420	*L. major*	125	96	220
LmjF29.0420	*L. major*	86	319	404
LmjF29.0430	*L. major*	45	520	564
LmjF29.0450	*L. major*	269	1	269
LmjF29.0460	*L. major*	125	1	125
LmjF29.0460	*L. major*	153	202	354
LmjF29.0460	*L. major*	355	377	731
LmjF29.0470	*L. major*	231	207	437
LmjF29.0480	*L. major*	150	1	150
LmjF29.0500	*L. major*	229	134	362
LmjF29.0500	*L. major*	80	375	454
LmjF29.0500	*L. major*	40	1019	1058
LmjF29.0500	*L. major*	69	1080	1148
LmjF29.0520	*L. major*	90	38	127
LmjF29.0520	*L. major*	55	170	224
LmjF29.0520	*L. major*	55	170	224
LmjF29.0530	*L. major*	160	1	160
LmjF29.0530	*L. major*	252	206	457
LmjF29.0530	*L. major*	184	480	663
LmjF29.0530	*L. major*	543	684	1226
LmjF29.0530	*L. major*	50	1234	1283
LmjF29.0530	*L. major*	113	1293	1405
LmjF29.0530	*L. major*	360	1407	1766
LmjF29.0530	*L. major*	52	1803	1854
LmjF29.0530	*L. major*	96	1856	1951
LmjF29.0530	*L. major*	639	1986	2624
LmjF29.0530	*L. major*	209	2757	2965
LmjF29.0540	*L. major*	210	1	210
LmjF29.0550	*L. major*	78	1	78
LmjF29.0550	*L. major*	85	80	164
LmjF29.0550	*L. major*	64	191	254
LmjF29.0550	*L. major*	316	307	622
LmjF29.0550	*L. major*	49	832	880
LmjF29.0555	*L. major*	206	85	290
LmjF29.0555	*L. major*	125	300	424
LmjF29.0560	*L. major*	66	1	66
LmjF29.0570	*L. major*	59	1	59
LmjF29.0570	*L. major*	104	158	261
LmjF29.0570	*L. major*	56	534	589
LmjF29.0580	*L. major*	150	70	219
LmjF29.0580	*L. major*	80	254	333
LmjF29.0580	*L. major*	54	371	424

LmjF29.0580	*L. major*	55	429	483
LmjF29.0590	*L. major*	43	88	130
LmjF29.0590	*L. major*	188	144	331
LmjF29.0590	*L. major*	58	701	758
LmjF29.0590	*L. major*	59	1048	1106
LmjF29.0600	*L. major*	76	101	176
LmjF29.0600	*L. major*	145	204	348
LmjF29.0600	*L. major*	88	453	540
LmjF29.0610	*L. major*	83	57	139
LmjF29.0610	*L. major*	170	232	401
LmjF29.0610	*L. major*	193	424	616
LmjF29.0630	*L. major*	87	1	87
LmjF29.0640	*L. major*	102	49	150
LmjF29.0640	*L. major*	52	176	227
LmjF29.0640	*L. major*	52	176	227
LmjF29.0650	*L. major*	218	271	488
LmjF29.0660	*L. major*	91	44	134
LmjF29.0660	*L. major*	47	336	382
LmjF29.0660	*L. major*	56	416	471
LmjF29.0660	*L. major*	53	538	590
LmjF29.0680	*L. major*	269	5	273
LmjF29.0680	*L. major*	93	458	550
LmjF29.0680	*L. major*	176	636	811
LmjF29.0700	*L. major*	111	69	179
LmjF29.0700	*L. major*	67	215	281
LmjF29.0700	*L. major*	46	617	662
LmjF29.0710	*L. major*	62	351	412
LmjF29.0730	*L. major*	187	1	187
LmjF29.0740	*L. major*	54	320	373
LmjF29.0750	*L. major*	56	1	56
LmjF29.0750	*L. major*	315	108	422
LmjF29.0750	*L. major*	77	471	547
LmjF29.0750	*L. major*	74	604	677
LmjF29.0760	*L. major*	70	79	148
LmjF29.0760	*L. major*	51	721	771
LmjF29.0770	*L. major*	102	1	102
LmjF29.0790	*L. major*	126	34	159
LmjF29.0790	*L. major*	44	298	341
LmjF29.0790	*L. major*	44	438	481
LmjF29.0790	*L. major*	125	696	820
LmjF29.0790	*L. major*	75	883	957
LmjF29.0790	*L. major*	63	1421	1483
LmjF29.0790	*L. major*	55	1521	1575
LmjF29.0800	*L. major*	143	1	143
LmjF29.0800	*L. major*	44	209	252
LmjF29.0800	*L. major*	80	298	377
LmjF29.0800	*L. major*	206	445	650

LmjF29.0805	*L. major*	393	43	435
LmjF29.0805	*L. major*	75	484	558
LmjF29.0810	*L. major*	83	90	172
LmjF29.0810	*L. major*	338	280	617
LmjF29.0820	*L. major*	52	194	245
LmjF29.0830	*L. major*	149	1	149
LmjF29.0830	*L. major*	115	328	442
LmjF29.0850	*L. major*	157	55	211
LmjF29.0850	*L. major*	85	215	299
LmjF29.0860	*L. major*	84	1	84
LmjF29.0860	*L. major*	82	160	241
LmjF29.0860	*L. major*	123	292	414
LmjF29.0860	*L. major*	66	455	520
LmjF29.0868	*L. major*	67	102	168
LmjF29.0870	*L. major*	50	331	380
LmjF29.0880	*L. major*	45	1	45
LmjF29.0885	*L. major*	40	310	349
LmjF29.0890	*L. major*	60	393	452
LmjF29.0910	*L. major*	52	258	309
LmjF29.0920	*L. major*	56	336	391
LmjF29.0920	*L. major*	42	460	501
LmjF29.0920	*L. major*	42	460	501
LmjF29.0930	*L. major*	84	313	396
LmjF29.0935	*L. major*	60	1	60
LmjF29.0950	*L. major*	103	1	103
LmjF29.0950	*L. major*	116	108	223
LmjF29.0960	*L. major*	90	1	90
LmjF29.0960	*L. major*	295	137	431
LmjF29.0970	*L. major*	575	380	954
LmjF29.0970	*L. major*	505	1036	1540
LmjF29.0980	*L. major*	248	1	248
LmjF29.0980	*L. major*	263	283	545
LmjF29.0990	*L. major*	52	1	52
LmjF29.0990	*L. major*	122	193	314
LmjF29.0990	*L. major*	252	324	575
LmjF29.0990	*L. major*	152	588	739
LmjF29.0990	*L. major*	83	881	963
LmjF29.1000	*L. major*	77	54	130
LmjF29.1000	*L. major*	393	211	603
LmjF29.1020	*L. major*	67	22	88
LmjF29.1020	*L. major*	100	197	296
LmjF29.1020	*L. major*	109	307	415
LmjF29.1020	*L. major*	48	531	578
LmjF29.1030	*L. major*	147	1	147
LmjF29.1040	*L. major*	56	1	56
LmjF29.1040	*L. major*	47	263	309
LmjF29.1040	*L. major*	93	836	928

LmjF29.1040	*L. major*	77	975	1051
LmjF29.1040	*L. major*	152	1132	1283
LmjF29.1040	*L. major*	98	1315	1412
LmjF29.1040	*L. major*	131	1538	1668
LmjF29.1040	*L. major*	43	1731	1773
LmjF29.1040	*L. major*	82	1817	1898
LmjF29.1050	*L. major*	54	60	113
LmjF29.1050	*L. major*	55	180	234
LmjF29.1050	*L. major*	54	301	354
LmjF29.1050	*L. major*	55	436	490
LmjF29.1050	*L. major*	59	492	550
LmjF29.1050	*L. major*	43	764	806
LmjF29.1060	*L. major*	47	1	47
LmjF29.1060	*L. major*	107	54	160
LmjF29.1060	*L. major*	100	193	292
LmjF29.1060	*L. major*	157	296	452
LmjF29.1060	*L. major*	297	459	755
LmjF29.1060	*L. major*	161	796	956
LmjF29.1070	*L. major*	87	287	373
LmjF29.1080	*L. major*	87	287	373
LmjF29.1090	*L. major*	87	287	373
LmjF29.1100	*L. major*	51	310	360
LmjF29.1110	*L. major*	58	118	175
LmjF29.1110	*L. major*	44	582	625
LmjF29.1110	*L. major*	44	582	625
LmjF29.1120	*L. major*	116	187	302
LmjF29.1120	*L. major*	178	332	509
LmjF29.1120	*L. major*	80	539	618
LmjF29.1120	*L. major*	172	652	823
LmjF29.1130	*L. major*	110	78	187
LmjF29.1130	*L. major*	53	251	303
LmjF29.1140	*L. major*	100	79	178
LmjF29.1170	*L. major*	127	152	278
LmjF29.1180	*L. major*	149	76	224
LmjF29.1180	*L. major*	127	259	385
LmjF29.1180	*L. major*	151	434	584
LmjF29.1180	*L. major*	283	703	985
LmjF29.1180	*L. major*	112	1044	1155
LmjF29.1180	*L. major*	148	1160	1307
LmjF29.1180	*L. major*	101	1325	1425
LmjF29.1180	*L. major*	54	1473	1526
LmjF29.1180	*L. major*	54	1551	1604
LmjF29.1180	*L. major*	77	1772	1848
LmjF29.1180	*L. major*	227	1974	2200
LmjF29.1180	*L. major*	194	2240	2433
LmjF29.1190	*L. major*	68	37	104
LmjF29.1190	*L. major*	74	251	324

LmjF29.1190	*L. major*	41	375	415
LmjF29.1200	*L. major*	102	386	487
LmjF29.1210	*L. major*	43	50	92
LmjF29.1210	*L. major*	47	318	364
LmjF29.1210	*L. major*	47	318	364
LmjF29.1220	*L. major*	343	135	477
LmjF29.1230	*L. major*	1024	1	1024
LmjF29.1230	*L. major*	78	1038	1115
LmjF29.1230	*L. major*	66	1229	1294
LmjF29.1230	*L. major*	75	1348	1422
LmjF29.1230	*L. major*	41	1470	1510
LmjF29.1230	*L. major*	41	1544	1584
LmjF29.1230	*L. major*	349	1620	1968
LmjF29.1240	*L. major*	68	525	592
LmjF29.1240	*L. major*	48	675	722
LmjF29.1240	*L. major*	48	675	722
LmjF29.1250	*L. major*	94	40	133
LmjF29.1250	*L. major*	948	137	1084
LmjF29.1250	*L. major*	711	1108	1818
LmjF29.1260	*L. major*	77	170	246
LmjF29.1260	*L. major*	40	317	356
LmjF29.1260	*L. major*	59	609	667
LmjF29.1260	*L. major*	250	872	1121
LmjF29.1260	*L. major*	139	1221	1359
LmjF29.1270	*L. major*	43	61	103
LmjF29.1270	*L. major*	95	406	500
LmjF29.1270	*L. major*	95	406	500
LmjF29.1280	*L. major*	136	1	136
LmjF29.1280	*L. major*	111	158	268
LmjF29.1280	*L. major*	146	837	982
LmjF29.1280	*L. major*	71	1009	1079
LmjF29.1280	*L. major*	64	1108	1171
LmjF29.1280	*L. major*	185	1174	1358
LmjF29.1280	*L. major*	158	1417	1574
LmjF29.1280	*L. major*	47	1884	1930
LmjF29.1290	*L. major*	47	52	98
LmjF29.1320	*L. major*	53	1	53
LmjF29.1320	*L. major*	68	189	256
LmjF29.1320	*L. major*	104	304	407
LmjF29.1330	*L. major*	139	1	139
LmjF29.1330	*L. major*	129	142	270
LmjF29.1330	*L. major*	45	415	459
LmjF29.1340	*L. major*	65	1	65
LmjF29.1340	*L. major*	95	276	370
LmjF29.1340	*L. major*	205	393	597
LmjF29.1350	*L. major*	79	1	79
LmjF29.1350	*L. major*	124	95	218

LmjF29.1350	*L. major*	518	403	920
LmjF29.1360	*L. major*	142	1	142
LmjF29.1360	*L. major*	94	322	415
LmjF29.1360	*L. major*	136	426	561
LmjF29.1360	*L. major*	56	597	652
LmjF29.1370	*L. major*	162	1	162
LmjF29.1370	*L. major*	45	214	258
LmjF29.1370	*L. major*	52	282	333
LmjF29.1370	*L. major*	179	444	622
LmjF29.1370	*L. major*	315	627	941
LmjF29.1370	*L. major*	91	953	1043
LmjF29.1370	*L. major*	72	1083	1154
LmjF29.1370	*L. major*	55	1190	1244
LmjF29.1370	*L. major*	66	1252	1317
LmjF29.1380	*L. major*	311	14	324
LmjF29.1380	*L. major*	62	411	472
LmjF29.1380	*L. major*	176	483	658
LmjF29.1390	*L. major*	66	1	66
LmjF29.1390	*L. major*	60	224	283
LmjF29.1390	*L. major*	250	344	593
LmjF29.1390	*L. major*	90	688	777
LmjF29.1400	*L. major*	190	51	240
LmjF29.1410	*L. major*	110	1	110
LmjF29.1410	*L. major*	341	136	476
LmjF29.1410	*L. major*	276	540	815
LmjF29.1420	*L. major*	72	290	361
LmjF29.1430	*L. major*	80	1	80
LmjF29.1430	*L. major*	56	291	346
LmjF29.1430	*L. major*	56	291	346
LmjF29.1450	*L. major*	133	28	160
LmjF29.1450	*L. major*	57	284	340
LmjF29.1450	*L. major*	68	588	655
LmjF29.1450	*L. major*	75	673	747
LmjF29.1450	*L. major*	59	860	918
LmjF29.1460	*L. major*	75	172	246
LmjF29.1460	*L. major*	112	290	401
LmjF29.1460	*L. major*	91	443	533
LmjF29.1460	*L. major*	44	996	1039
LmjF29.1470	*L. major*	153	1	153
LmjF29.1470	*L. major*	83	255	337
LmjF29.1480	*L. major*	75	79	153
LmjF29.1490	*L. major*	65	16	80
LmjF29.1490	*L. major*	100	369	468
LmjF29.1490	*L. major*	110	796	905
LmjF29.1490	*L. major*	44	986	1029
LmjF29.1490	*L. major*	49	1129	1177
LmjF29.1500	*L. major*	126	303	428

LmjF29.1510	*L. major*	126	303	428
LmjF29.1520	*L. major*	72	290	361
LmjF29.1530	*L. major*	43	73	115
LmjF29.1530	*L. major*	42	172	213
LmjF29.1530	*L. major*	49	628	676
LmjF29.1550	*L. major*	88	300	387
LmjF29.1560	*L. major*	160	36	195
LmjF29.1560	*L. major*	51	479	529
LmjF29.1575	*L. major*	69	125	193
LmjF29.1575	*L. major*	76	290	365
LmjF29.1575	*L. major*	58	395	452
LmjF29.1580	*L. major*	129	60	188
LmjF29.1580	*L. major*	125	232	356
LmjF29.1590	*L. major*	46	1	46
LmjF29.1590	*L. major*	326	66	391
LmjF29.1590	*L. major*	553	425	977
LmjF29.1610	*L. major*	73	280	352
LmjF29.1620	*L. major*	93	1	93
LmjF29.1620	*L. major*	76	188	263
LmjF29.1620	*L. major*	46	412	457
LmjF29.1620	*L. major*	74	540	613
LmjF29.1620	*L. major*	97	713	809
LmjF29.1620	*L. major*	61	855	915
LmjF29.1620	*L. major*	72	954	1025
LmjF29.1620	*L. major*	47	1151	1197
LmjF29.1620	*L. major*	102	1218	1319
LmjF29.1620	*L. major*	48	1602	1649
LmjF29.1620	*L. major*	48	1735	1782
LmjF29.1620	*L. major*	55	1785	1839
LmjF29.1620	*L. major*	61	1956	2016
LmjF29.1620	*L. major*	56	2088	2143
LmjF29.1630	*L. major*	71	1	71
LmjF29.1630	*L. major*	143	278	420
LmjF29.1630	*L. major*	59	1163	1221
LmjF29.1640	*L. major*	61	1	61
LmjF29.1640	*L. major*	110	355	464
LmjF29.1660	*L. major*	51	1	51
LmjF29.1660	*L. major*	51	134	184
LmjF29.1700	*L. major*	41	1	41
LmjF29.1700	*L. major*	86	207	292
LmjF29.1700	*L. major*	83	580	662
LmjF29.1700	*L. major*	369	840	1208
LmjF29.1710	*L. major*	50	1	50
LmjF29.1710	*L. major*	66	529	594
LmjF29.1710	*L. major*	40	940	979
LmjF29.1710	*L. major*	55	1004	1058
LmjF29.1750	*L. major*	81	136	216

LmjF29.1750	*L. major*	122	239	360
LmjF29.1750	*L. major*	123	473	595
LmjF29.1760	*L. major*	81	136	216
LmjF29.1760	*L. major*	122	239	360
LmjF29.1760	*L. major*	123	473	595
LmjF29.1770	*L. major*	81	136	216
LmjF29.1770	*L. major*	122	239	360
LmjF29.1770	*L. major*	123	473	595
LmjF29.1790	*L. major*	167	183	349
LmjF29.1790	*L. major*	186	566	751
LmjF29.1790	*L. major*	54	798	851
LmjF29.1810	*L. major*	48	161	208
LmjF29.1810	*L. major*	43	423	465
LmjF29.1810	*L. major*	89	499	587
LmjF29.1840	*L. major*	52	1	52
LmjF29.1840	*L. major*	195	62	256
LmjF29.1840	*L. major*	195	62	256
LmjF29.1850	*L. major*	165	1	165
LmjF29.1860	*L. major*	102	238	339
LmjF29.1870	*L. major*	44	65	108
LmjF29.1880	*L. major*	43	52	94
LmjF29.1880	*L. major*	42	150	191
LmjF29.1880	*L. major*	57	268	324
LmjF29.1880	*L. major*	92	665	756
LmjF29.1880	*L. major*	62	878	939
LmjF29.1890	*L. major*	75	95	169
LmjF29.1890	*L. major*	152	244	395
LmjF29.1900	*L. major*	41	85	125
LmjF29.1900	*L. major*	47	219	265
LmjF29.1900	*L. major*	87	282	368
LmjF29.1910	*L. major*	58	89	146
LmjF29.1910	*L. major*	67	289	355
LmjF29.1920	*L. major*	67	44	110
LmjF29.1920	*L. major*	40	207	246
LmjF29.1930	*L. major*	63	1	63
LmjF29.1930	*L. major*	106	160	265
LmjF29.1930	*L. major*	106	160	265
LmjF29.1940	*L. major*	90	1	90
LmjF29.1950	*L. major*	42	1	42
LmjF29.1950	*L. major*	42	670	711
LmjF29.1950	*L. major*	42	670	711
LmjF29.1970	*L. major*	41	102	142
LmjF29.1980	*L. major*	303	170	472
LmjF29.1980	*L. major*	57	476	532
LmjF29.1980	*L. major*	88	596	683
LmjF29.1980	*L. major*	244	709	952
LmjF29.1980	*L. major*	44	1002	1045

LmjF29.1980	*L. major*	89	1160	1248
LmjF29.1980	*L. major*	51	1276	1326
LmjF29.1980	*L. major*	40	1335	1374
LmjF29.1980	*L. major*	86	1384	1469
LmjF29.1980	*L. major*	71	1562	1632
LmjF29.1980	*L. major*	94	1756	1849
LmjF29.1990	*L. major*	43	38	80
LmjF29.1990	*L. major*	89	88	176
LmjF29.1990	*L. major*	47	327	373
LmjF29.1990	*L. major*	49	400	448
LmjF29.1995	*L. major*	186	18	203
LmjF29.2000	*L. major*	93	42	134
LmjF29.2000	*L. major*	48	252	299
LmjF29.2000	*L. major*	40	310	349
LmjF29.2000	*L. major*	108	410	517
LmjF29.2010	*L. major*	103	492	594
LmjF29.2020	*L. major*	98	435	532
LmjF29.2020	*L. major*	163	603	765
LmjF29.2030	*L. major*	52	197	248
LmjF29.2030	*L. major*	120	261	380
LmjF29.2040	*L. major*	223	1	223
LmjF29.2040	*L. major*	94	234	327
LmjF29.2040	*L. major*	51	574	624
LmjF29.2040	*L. major*	56	672	727
LmjF29.2040	*L. major*	53	768	820
LmjF29.2040	*L. major*	97	875	971
LmjF29.2040	*L. major*	300	981	1280
LmjF29.2050	*L. major*	285	1	285
LmjF29.2050	*L. major*	59	335	393
LmjF29.2050	*L. major*	42	425	466
LmjF29.2050	*L. major*	130	553	682
LmjF29.2060	*L. major*	56	968	1023
LmjF29.2070	*L. major*	103	24	126
LmjF29.2070	*L. major*	133	272	404
LmjF29.2080	*L. major*	42	205	246
LmjF29.2090	*L. major*	89	96	184
LmjF29.2090	*L. major*	70	353	422
LmjF29.2090	*L. major*	73	481	553
LmjF29.2090	*L. major*	83	1074	1156
LmjF29.2090	*L. major*	86	1517	1602
LmjF29.2100	*L. major*	50	108	157
LmjF29.2100	*L. major*	56	201	256
LmjF29.2100	*L. major*	56	201	256
LmjF29.2110	*L. major*	61	119	179
LmjF29.2110	*L. major*	40	326	365
LmjF29.2110	*L. major*	40	326	365
LmjF29.2150	*L. major*	68	51	118

LmjF29.2170	*L. major*	85	48	132
LmjF29.2170	*L. major*	79	284	362
LmjF29.2170	*L. major*	44	475	518
LmjF29.2170	*L. major*	69	545	613
LmjF29.2180	*L. major*	243	1	243
LmjF29.2190	*L. major*	49	124	172
LmjF29.2190	*L. major*	41	1032	1072
LmjF29.2190	*L. major*	40	1141	1180
LmjF29.2200	*L. major*	90	495	584
LmjF29.2200	*L. major*	53	644	696
LmjF29.2210	*L. major*	111	165	275
LmjF29.2210	*L. major*	162	327	488
LmjF29.2220	*L. major*	132	204	335
LmjF29.2230	*L. major*	50	45	94
LmjF29.2230	*L. major*	87	204	290
LmjF29.2230	*L. major*	87	204	290
LmjF29.2250	*L. major*	193	1	193
LmjF29.2250	*L. major*	62	550	611
LmjF29.2260	*L. major*	55	20	74
LmjF29.2260	*L. major*	47	175	221
LmjF29.2260	*L. major*	47	175	221
LmjF29.2270	*L. major*	41	278	318
LmjF29.2290	*L. major*	412	1	412
LmjF29.2300	*L. major*	51	460	510
LmjF29.2330	*L. major*	49	365	413
LmjF29.2340	*L. major*	46	1	46
LmjF29.2380	*L. major*	47	1	47
LmjF29.2380	*L. major*	88	151	238
LmjF29.2380	*L. major*	63	246	308
LmjF29.2390	*L. major*	46	1	46
LmjF29.2390	*L. major*	88	149	236
LmjF29.2390	*L. major*	63	244	306
LmjF29.2390	*L. major*	61	349	409
LmjF29.2420	*L. major*	160	1	160
LmjF29.2420	*L. major*	101	163	263
LmjF29.2420	*L. major*	249	294	542
LmjF29.2420	*L. major*	78	552	629
LmjF29.2430	*L. major*	718	1	718
LmjF29.2440	*L. major*	87	1	87
LmjF29.2460	*L. major*	84	1	84
LmjF29.2460	*L. major*	53	129	181
LmjF29.2470	*L. major*	84	1	84
LmjF29.2470	*L. major*	53	129	181
LmjF29.2480	*L. major*	844	1	844
LmjF29.2500	*L. major*	56	66	121
LmjF29.2500	*L. major*	66	367	432
LmjF29.2500	*L. major*	66	367	432

LmjF29.2520	*L. major*	90	303	392
LmjF29.2550	*L. major*	65	771	835
LmjF29.2560	*L. major*	58	69	126
LmjF29.2560	*L. major*	147	140	286
LmjF29.2560	*L. major*	53	361	413
LmjF29.2570	*L. major*	164	274	437
LmjF29.2580	*L. major*	51	1	51
LmjF29.2580	*L. major*	106	71	176
LmjF29.2580	*L. major*	75	206	280
LmjF29.2580	*L. major*	384	294	677
LmjF29.2590	*L. major*	55	1	55
LmjF29.2590	*L. major*	166	141	306
LmjF29.2590	*L. major*	102	321	422
LmjF29.2590	*L. major*	75	516	590
LmjF29.2610	*L. major*	232	1	232
LmjF29.2610	*L. major*	46	245	290
LmjF29.2610	*L. major*	50	366	415
LmjF29.2610	*L. major*	70	649	718
LmjF29.2630	*L. major*	63	112	174
LmjF29.2630	*L. major*	78	289	366
LmjF29.2630	*L. major*	83	503	585
LmjF29.2630	*L. major*	131	775	905
LmjF29.2630	*L. major*	233	1000	1232
LmjF29.2650	*L. major*	58	464	521
LmjF29.2650	*L. major*	73	819	891
LmjF29.2650	*L. major*	41	945	985
LmjF29.2660	*L. major*	60	128	187
LmjF29.2660	*L. major*	118	744	861
LmjF29.2660	*L. major*	118	744	861
LmjF29.2670	*L. major*	75	249	323
LmjF29.2670	*L. major*	79	348	426
LmjF29.2680	*L. major*	89	53	141
LmjF29.2680	*L. major*	316	146	461
LmjF29.2680	*L. major*	181	859	1039
LmjF29.2690	*L. major*	259	50	308
LmjF29.2700	*L. major*	43	747	789
LmjF29.2720	*L. major*	43	1	43
LmjF29.2720	*L. major*	44	233	276
LmjF29.2720	*L. major*	44	233	276
LmjF29.2730	*L. major*	61	244	304
LmjF29.2740	*L. major*	45	178	222
LmjF29.2760	*L. major*	41	1	41
LmjF29.2810	*L. major*	63	1	63
LmjF29.2810	*L. major*	178	561	738
LmjF29.2810	*L. major*	178	561	738
LmjF29.2830	*L. major*	109	1	109
LmjF29.2880	*L. major*	40	740	779

LmjF30.0030	*L. major*	58	157	214
LmjF30.0050	*L. major*	144	94	237
LmjF30.0050	*L. major*	217	546	762
LmjF30.0060	*L. major*	43	142	184
LmjF30.0080	*L. major*	56	1	56
LmjF30.0090	*L. major*	228	1	228
LmjF30.0120	*L. major*	47	575	621
LmjF30.0140	*L. major*	108	1	108
LmjF30.0200	*L. major*	63	136	198
LmjF30.0200	*L. major*	45	239	283
LmjF30.0200	*L. major*	42	293	334
LmjF30.0200	*L. major*	46	384	429
LmjF30.0210	*L. major*	65	1	65
LmjF30.0220	*L. major*	156	213	368
LmjF30.0240	*L. major*	51	352	402
LmjF30.0240	*L. major*	45	407	451
LmjF30.0240	*L. major*	40	504	543
LmjF30.0250	*L. major*	59	406	464
LmjF30.0250	*L. major*	77	1055	1131
LmjF30.0260	*L. major*	56	152	207
LmjF30.0260	*L. major*	116	311	426
LmjF30.0260	*L. major*	59	579	637
LmjF30.0270	*L. major*	91	304	394
LmjF30.0280	*L. major*	87	1	87
LmjF30.0290	*L. major*	61	384	444
LmjF30.0290	*L. major*	145	476	620
LmjF30.0290	*L. major*	90	820	909
LmjF30.0290	*L. major*	74	1102	1175
LmjF30.0300	*L. major*	61	20	80
LmjF30.0310	*L. major*	47	1	47
LmjF30.0310	*L. major*	67	94	160
LmjF30.0310	*L. major*	305	222	526
LmjF30.0310	*L. major*	508	732	1239
LmjF30.0320	*L. major*	125	224	348
LmjF30.0320	*L. major*	61	394	454
LmjF30.0320	*L. major*	61	460	520
LmjF30.0330	*L. major*	247	525	771
LmjF30.0340	*L. major*	91	551	641
LmjF30.0350	*L. major*	50	377	426
LmjF30.0350	*L. major*	122	486	607
LmjF30.0350	*L. major*	122	486	607
LmjF30.0370	*L. major*	48	259	306
LmjF30.0390	*L. major*	54	31	84
LmjF30.0390	*L. major*	125	126	250
LmjF30.0400	*L. major*	106	319	424
LmjF30.0410	*L. major*	63	1	63
LmjF30.0420	*L. major*	54	313	366

LmjF30.0440	*L. major*	42	1	42
LmjF30.0450	*L. major*	96	1	96
LmjF30.0460	*L. major*	47	1	47
LmjF30.0460	*L. major*	46	146	191
LmjF30.0460	*L. major*	46	146	191
LmjF30.0470	*L. major*	71	284	354
LmjF30.0470	*L. major*	60	490	549
LmjF30.0480	*L. major*	51	1	51
LmjF30.0480	*L. major*	153	58	210
LmjF30.0490	*L. major*	345	1	345
LmjF30.0490	*L. major*	109	360	468
LmjF30.0490	*L. major*	217	500	716
LmjF30.0495	*L. major*	62	32	93
LmjF30.0500	*L. major*	454	6	459
LmjF30.0510	*L. major*	46	13	58
LmjF30.0510	*L. major*	103	118	220
LmjF30.0510	*L. major*	88	473	560
LmjF30.0510	*L. major*	192	598	789
LmjF30.0510	*L. major*	70	826	895
LmjF30.0510	*L. major*	132	945	1076
LmjF30.0510	*L. major*	59	1090	1148
LmjF30.0520	*L. major*	45	283	327
LmjF30.0520	*L. major*	53	691	743
LmjF30.0520	*L. major*	53	691	743
LmjF30.0550	*L. major*	78	532	609
LmjF30.0560	*L. major*	272	54	325
LmjF30.0570	*L. major*	134	1	134
LmjF30.0570	*L. major*	127	346	472
LmjF30.0590	*L. major*	53	226	278
LmjF30.0600	*L. major*	156	51	206
LmjF30.0600	*L. major*	143	212	354
LmjF30.0600	*L. major*	310	619	928
LmjF30.0610	*L. major*	91	1	91
LmjF30.0620	*L. major*	68	73	140
LmjF30.0620	*L. major*	103	143	245
LmjF30.0630	*L. major*	66	1	66
LmjF30.0640	*L. major*	63	103	165
LmjF30.0650	*L. major*	59	1	59
LmjF30.0660	*L. major*	72	1	72
LmjF30.0710	*L. major*	55	492	546
LmjF30.0730	*L. major*	93	1	93
LmjF30.0735	*L. major*	53	87	139
LmjF30.0745	*L. major*	116	44	159
LmjF30.0760	*L. major*	201	802	1002
LmjF30.0760	*L. major*	50	1008	1057
LmjF30.0780	*L. major*	155	29	183
LmjF30.0780	*L. major*	45	265	309

LmjF30.0790	*L. major*	179	168	346
LmjF30.0800	*L. major*	104	407	510
LmjF30.0805	*L. major*	62	88	149
LmjF30.0820	*L. major*	129	75	203
LmjF30.0820	*L. major*	55	273	327
LmjF30.0820	*L. major*	83	441	523
LmjF30.0820	*L. major*	178	589	766
LmjF30.0830	*L. major*	49	1	49
LmjF30.0830	*L. major*	60	87	146
LmjF30.0830	*L. major*	60	87	146
LmjF30.0840	*L. major*	139	1	139
LmjF30.0840	*L. major*	73	846	918
LmjF30.0840	*L. major*	73	846	918
LmjF30.0890	*L. major*	75	8	82
LmjF30.0900	*L. major*	221	1	221
LmjF30.0900	*L. major*	284	348	631
LmjF30.0910	*L. major*	107	1	107
LmjF30.0910	*L. major*	58	627	684
LmjF30.0910	*L. major*	52	747	798
LmjF30.0910	*L. major*	42	936	977
LmjF30.0920	*L. major*	61	29	89
LmjF30.0920	*L. major*	57	211	267
LmjF30.0920	*L. major*	58	343	400
LmjF30.0920	*L. major*	55	473	527
LmjF30.0920	*L. major*	53	549	601
LmjF30.0930	*L. major*	40	438	477
LmjF30.0940	*L. major*	67	1	67
LmjF30.0940	*L. major*	52	370	421
LmjF30.0940	*L. major*	52	732	783
LmjF30.0950	*L. major*	211	1	211
LmjF30.0960	*L. major*	249	1	249
LmjF30.0960	*L. major*	82	309	390
LmjF30.0970	*L. major*	55	1	55
LmjF30.0980	*L. major*	41	167	207
LmjF30.0980	*L. major*	91	312	402
LmjF30.0980	*L. major*	91	312	402
LmjF30.0990	*L. major*	58	64	121
LmjF30.0990	*L. major*	80	177	256
LmjF30.0990	*L. major*	45	286	330
LmjF30.0990	*L. major*	65	380	444
LmjF30.1000	*L. major*	53	129	181
LmjF30.1000	*L. major*	66	242	307
LmjF30.1000	*L. major*	60	444	503
LmjF30.1000	*L. major*	82	531	612
LmjF30.1010	*L. major*	80	711	790
LmjF30.1020	*L. major*	72	1	72
LmjF30.1020	*L. major*	142	237	378

LmjF30.1030	*L. major*	216	103	318
LmjF30.1070	*L. major*	142	1	142
LmjF30.1080	*L. major*	131	1	131
LmjF30.1080	*L. major*	49	268	316
LmjF30.1080	*L. major*	917	420	1336
LmjF30.1080	*L. major*	272	1411	1682
LmjF30.1080	*L. major*	167	1690	1856
LmjF30.1080	*L. major*	82	1887	1968
LmjF30.1080	*L. major*	317	2007	2323
LmjF30.1080	*L. major*	43	2337	2379
LmjF30.1090	*L. major*	79	157	235
LmjF30.1090	*L. major*	58	279	336
LmjF30.1090	*L. major*	103	504	606
LmjF30.1090	*L. major*	57	641	697
LmjF30.1090	*L. major*	122	742	863
LmjF30.1090	*L. major*	57	892	948
LmjF30.1110	*L. major*	76	81	156
LmjF30.1110	*L. major*	86	356	441
LmjF30.1110	*L. major*	44	576	619
LmjF30.1120	*L. major*	41	1	41
LmjF30.1150	*L. major*	183	1	183
LmjF30.1150	*L. major*	85	316	400
LmjF30.1150	*L. major*	84	484	567
LmjF30.1150	*L. major*	66	773	838
LmjF30.1160	*L. major*	286	1	286
LmjF30.1160	*L. major*	67	443	509
LmjF30.1160	*L. major*	71	526	596
LmjF30.1160	*L. major*	48	680	727
LmjF30.1160	*L. major*	44	742	785
LmjF30.1160	*L. major*	57	904	960
LmjF30.1160	*L. major*	160	1116	1275
LmjF30.1160	*L. major*	66	1475	1540
LmjF30.1160	*L. major*	108	1561	1668
LmjF30.1170	*L. major*	184	1	184
LmjF30.1170	*L. major*	42	641	682
LmjF30.1190	*L. major*	46	131	176
LmjF30.1200	*L. major*	95	45	139
LmjF30.1200	*L. major*	94	249	342
LmjF30.1200	*L. major*	64	408	471
LmjF30.1200	*L. major*	43	592	634
LmjF30.1200	*L. major*	102	822	923
LmjF30.1220	*L. major*	114	20	133
LmjF30.1220	*L. major*	111	172	282
LmjF30.1220	*L. major*	91	529	619
LmjF30.1220	*L. major*	184	742	925
LmjF30.1230	*L. major*	65	394	458
LmjF30.1230	*L. major*	201	523	723

LmjF30.1240	*L. major*	43	1	43
LmjF30.1240	*L. major*	40	95	134
LmjF30.1240	*L. major*	40	95	134
LmjF30.1255	*L. major*	43	270	312
LmjF30.1260	*L. major*	162	263	424
LmjF30.1260	*L. major*	93	533	625
LmjF30.1260	*L. major*	57	1346	1402
LmjF30.1260	*L. major*	46	1421	1466
LmjF30.1260	*L. major*	116	1522	1637
LmjF30.1260	*L. major*	44	1836	1879
LmjF30.1270	*L. major*	42	100	141
LmjF30.1270	*L. major*	78	190	267
LmjF30.1270	*L. major*	208	285	492
LmjF30.1270	*L. major*	119	502	620
LmjF30.1270	*L. major*	65	641	705
LmjF30.1280	*L. major*	122	1	122
LmjF30.1280	*L. major*	70	374	443
LmjF30.1280	*L. major*	95	758	852
LmjF30.1280	*L. major*	42	925	966
LmjF30.1280	*L. major*	59	1165	1223
LmjF30.1290	*L. major*	81	1	81
LmjF30.1300	*L. major*	41	83	123
LmjF30.1300	*L. major*	40	191	230
LmjF30.1300	*L. major*	57	542	598
LmjF30.1300	*L. major*	205	920	1124
LmjF30.1300	*L. major*	59	1157	1215
LmjF30.1310	*L. major*	241	1	241
LmjF30.1310	*L. major*	251	341	591
LmjF30.1310	*L. major*	193	673	865
LmjF30.1310	*L. major*	79	894	972
LmjF30.1310	*L. major*	67	1108	1174
LmjF30.1310	*L. major*	231	1212	1442
LmjF30.1310	*L. major*	56	1444	1499
LmjF30.1310	*L. major*	101	1615	1715
LmjF30.1330	*L. major*	45	151	195
LmjF30.1340	*L. major*	56	58	113
LmjF30.1340	*L. major*	168	151	318
LmjF30.1350	*L. major*	71	1	71
LmjF30.1350	*L. major*	187	167	353
LmjF30.1360	*L. major*	180	307	486
LmjF30.1370	*L. major*	189	1	189
LmjF30.1370	*L. major*	299	214	512
LmjF30.1370	*L. major*	162	517	678
LmjF30.1400	*L. major*	227	1	227
LmjF30.1410	*L. major*	44	1	44
LmjF30.1420	*L. major*	47	1	47
LmjF30.1430	*L. major*	62	1	62

LmjF30.1440	*L. major*	70	21	90
LmjF30.1440	*L. major*	41	136	176
LmjF30.1440	*L. major*	72	370	441
LmjF30.1440	*L. major*	42	670	711
LmjF30.1440	*L. major*	47	818	864
LmjF30.1440	*L. major*	127	1033	1159
LmjF30.1440	*L. major*	97	1201	1297
LmjF30.1440	*L. major*	84	1334	1417
LmjF30.1450	*L. major*	159	408	566
LmjF30.1450	*L. major*	282	670	951
LmjF30.1520	*L. major*	69	105	173
LmjF30.1530	*L. major*	43	129	171
LmjF30.1530	*L. major*	56	309	364
LmjF30.1530	*L. major*	146	470	615
LmjF30.1530	*L. major*	84	767	850
LmjF30.1530	*L. major*	156	896	1051
LmjF30.1540	*L. major*	64	185	248
LmjF30.1550	*L. major*	49	1	49
LmjF30.1550	*L. major*	84	415	498
LmjF30.1550	*L. major*	84	415	498
LmjF30.1560	*L. major*	49	53	101
LmjF30.1560	*L. major*	127	175	301
LmjF30.1560	*L. major*	73	351	423
LmjF30.1560	*L. major*	78	457	534
LmjF30.1560	*L. major*	236	641	876
LmjF30.1560	*L. major*	104	951	1054
LmjF30.1560	*L. major*	56	1078	1133
LmjF30.1570	*L. major*	42	64	105
LmjF30.1570	*L. major*	57	214	270
LmjF30.1570	*L. major*	57	214	270
LmjF30.1580	*L. major*	100	107	206
LmjF30.1580	*L. major*	74	646	719
LmjF30.1590	*L. major*	47	25	71
LmjF30.1590	*L. major*	67	89	155
LmjF30.1590	*L. major*	67	89	155
LmjF30.1600	*L. major*	52	267	318
LmjF30.1600	*L. major*	115	374	488
LmjF30.1610	*L. major*	44	196	239
LmjF30.1610	*L. major*	164	861	1024
LmjF30.1620	*L. major*	133	18	150
LmjF30.1620	*L. major*	91	601	691
LmjF30.1690	*L. major*	82	115	196
LmjF30.1690	*L. major*	63	213	275
LmjF30.1690	*L. major*	65	290	354
LmjF30.1700	*L. major*	112	32	143
LmjF30.1700	*L. major*	93	201	293
LmjF30.1700	*L. major*	160	598	757

LmjF30.1710	*L. major*	42	194	235
LmjF30.1720	*L. major*	59	23	81
LmjF30.1730	*L. major*	367	24	390
LmjF30.1740	*L. major*	43	1	43
LmjF30.1760	*L. major*	47	197	243
LmjF30.1760	*L. major*	168	479	646
LmjF30.1760	*L. major*	148	861	1008
LmjF30.1760	*L. major*	208	1204	1411
LmjF30.1760	*L. major*	199	1656	1854
LmjF30.1770	*L. major*	154	1	154
LmjF30.1780	*L. major*	53	42	94
LmjF30.1780	*L. major*	47	98	144
LmjF30.1780	*L. major*	70	256	325
LmjF30.1790	*L. major*	94	188	281
LmjF30.1790	*L. major*	96	361	456
LmjF30.1800	*L. major*	56	143	198
LmjF30.1810	*L. major*	133	1	133
LmjF30.1810	*L. major*	80	415	494
LmjF30.1810	*L. major*	51	1190	1240
LmjF30.1810	*L. major*	50	1310	1359
LmjF30.1810	*L. major*	65	1570	1634
LmjF30.1810	*L. major*	92	2046	2137
LmjF30.1810	*L. major*	127	2334	2460
LmjF30.1810	*L. major*	128	2502	2629
LmjF30.1810	*L. major*	56	2660	2715
LmjF30.1810	*L. major*	81	2824	2904
LmjF30.1810	*L. major*	80	3466	3545
LmjF30.1810	*L. major*	88	3604	3691
LmjF30.1810	*L. major*	67	4088	4154
LmjF30.1810	*L. major*	50	4731	4780
LmjF30.1810	*L. major*	45	4871	4915
LmjF30.1810	*L. major*	71	5092	5162
LmjF30.1810	*L. major*	42	5240	5281
LmjF30.1830	*L. major*	52	265	316
LmjF30.1830	*L. major*	102	407	508
LmjF30.1830	*L. major*	48	575	622
LmjF30.1840	*L. major*	107	1	107
LmjF30.1840	*L. major*	193	137	329
LmjF30.1840	*L. major*	200	363	562
LmjF30.1840	*L. major*	136	564	699
LmjF30.1850	*L. major*	87	104	190
LmjF30.1850	*L. major*	72	200	271
LmjF30.1850	*L. major*	83	358	440
LmjF30.1850	*L. major*	69	978	1046
LmjF30.1850	*L. major*	56	1201	1256
LmjF30.1850	*L. major*	69	1306	1374
LmjF30.1860	*L. major*	124	101	224

LmjF30.1860	*L. major*	120	255	374
LmjF30.1860	*L. major*	41	376	416
LmjF30.1870	*L. major*	189	98	286
LmjF30.1870	*L. major*	179	291	469
LmjF30.1870	*L. major*	134	500	633
LmjF30.1870	*L. major*	459	648	1106
LmjF30.1870	*L. major*	87	1118	1204
LmjF30.1870	*L. major*	58	1215	1272
LmjF30.1890	*L. major*	53	118	170
LmjF30.1900	*L. major*	91	164	254
LmjF30.1900	*L. major*	126	312	437
LmjF30.1900	*L. major*	106	532	637
LmjF30.1900	*L. major*	69	725	793
LmjF30.1900	*L. major*	43	944	986
LmjF30.1920	*L. major*	61	88	148
LmjF30.1920	*L. major*	59	237	295
LmjF30.1920	*L. major*	98	508	605
LmjF30.1950	*L. major*	78	1	78
LmjF30.1950	*L. major*	161	290	450
LmjF30.1950	*L. major*	225	625	849
LmjF30.1950	*L. major*	49	856	904
LmjF30.1950	*L. major*	248	912	1159
LmjF30.1960	*L. major*	94	125	218
LmjF30.1960	*L. major*	51	220	270
LmjF30.1970	*L. major*	79	105	183
LmjF30.1970	*L. major*	74	302	375
LmjF30.1970	*L. major*	74	302	375
LmjF30.1975	*L. major*	89	110	198
LmjF30.1980	*L. major*	57	127	183
LmjF30.1980	*L. major*	40	302	341
LmjF30.1980	*L. major*	40	302	341
LmjF30.1985	*L. major*	148	18	165
LmjF30.1990	*L. major*	54	54	107
LmjF30.1990	*L. major*	130	223	352
LmjF30.1990	*L. major*	43	545	587
LmjF30.1990	*L. major*	45	628	672
LmjF30.1990	*L. major*	77	755	831
LmjF30.1990	*L. major*	60	910	969
LmjF30.2000	*L. major*	55	75	129
LmjF30.2010	*L. major*	132	52	183
LmjF30.2010	*L. major*	276	198	473
LmjF30.2010	*L. major*	76	505	580
LmjF30.2010	*L. major*	103	603	705
LmjF30.2010	*L. major*	50	720	769
LmjF30.2010	*L. major*	132	800	931
LmjF30.2010	*L. major*	375	1124	1498
LmjF30.2020	*L. major*	155	1	155

LmjF30.2020	*L. major*	58	189	246
LmjF30.2020	*L. major*	153	280	432
LmjF30.2040	*L. major*	47	320	366
LmjF30.2040	*L. major*	68	709	776
LmjF30.2040	*L. major*	55	886	940
LmjF30.2040	*L. major*	48	1021	1068
LmjF30.2060	*L. major*	119	271	389
LmjF30.2060	*L. major*	149	694	842
LmjF30.2065	*L. major*	237	1	237
LmjF30.2070	*L. major*	89	120	208
LmjF30.2080	*L. major*	57	1	57
LmjF30.2080	*L. major*	105	447	551
LmjF30.2100	*L. major*	84	55	138
LmjF30.2100	*L. major*	64	347	410
LmjF30.2100	*L. major*	46	486	531
LmjF30.2110	*L. major*	54	152	205
LmjF30.2110	*L. major*	144	245	388
LmjF30.2110	*L. major*	85	420	504
LmjF30.2130	*L. major*	76	83	158
LmjF30.2130	*L. major*	134	403	536
LmjF30.2130	*L. major*	73	667	739
LmjF30.2140	*L. major*	71	158	228
LmjF30.2140	*L. major*	172	257	428
LmjF30.2140	*L. major*	172	257	428
LmjF30.2150	*L. major*	59	408	466
LmjF30.2160	*L. major*	53	129	181
LmjF30.2160	*L. major*	41	228	268
LmjF30.2160	*L. major*	178	404	581
LmjF30.2170	*L. major*	60	1	60
LmjF30.2180	*L. major*	275	171	445
LmjF30.2190	*L. major*	184	172	355
LmjF30.2200	*L. major*	85	62	146
LmjF30.2210	*L. major*	72	757	828
LmjF30.2210	*L. major*	54	1372	1425
LmjF30.2210	*L. major*	58	1842	1899
LmjF30.2210	*L. major*	49	2410	2458
LmjF30.2220	*L. major*	74	44	117
LmjF30.2220	*L. major*	57	181	237
LmjF30.2230	*L. major*	76	72	147
LmjF30.2250	*L. major*	71	86	156
LmjF30.2250	*L. major*	121	251	371
LmjF30.2250	*L. major*	121	251	371
LmjF30.2260	*L. major*	41	534	574
LmjF30.2260	*L. major*	110	763	872
LmjF30.2260	*L. major*	110	763	872
LmjF30.2270	*L. major*	124	53	176
LmjF30.2270	*L. major*	153	214	366

LmjF30.2270	*L. major*	190	373	562
LmjF30.2270	*L. major*	128	632	759
LmjF30.2270	*L. major*	44	892	935
LmjF30.2270	*L. major*	109	949	1057
LmjF30.2270	*L. major*	42	1244	1285
LmjF30.2270	*L. major*	121	1576	1696
LmjF30.2280	*L. major*	82	1	82
LmjF30.2280	*L. major*	69	338	406
LmjF30.2280	*L. major*	69	338	406
LmjF30.2290	*L. major*	84	27	110
LmjF30.2290	*L. major*	86	445	530
LmjF30.2290	*L. major*	81	687	767
LmjF30.2300	*L. major*	46	110	155
LmjF30.2300	*L. major*	62	177	238
LmjF30.2300	*L. major*	86	510	595
LmjF30.2300	*L. major*	157	850	1006
LmjF30.2300	*L. major*	105	1036	1140
LmjF30.2300	*L. major*	55	1251	1305
LmjF30.2340	*L. major*	76	85	160
LmjF30.2340	*L. major*	42	192	233
LmjF30.2340	*L. major*	317	267	583
LmjF30.2340	*L. major*	547	651	1197
LmjF30.2360	*L. major*	40	337	376
LmjF30.2380	*L. major*	178	1	178
LmjF30.2380	*L. major*	57	185	241
LmjF30.2380	*L. major*	57	185	241
LmjF30.2390	*L. major*	128	872	999
LmjF30.2390	*L. major*	85	1247	1331
LmjF30.2390	*L. major*	40	1382	1421
LmjF30.2390	*L. major*	73	1456	1528
LmjF30.2390	*L. major*	45	1565	1609
LmjF30.2390	*L. major*	61	1721	1781
LmjF30.2390	*L. major*	61	2499	2559
LmjF30.2390	*L. major*	54	2681	2734
LmjF30.2390	*L. major*	62	2748	2809
LmjF30.2390	*L. major*	56	2951	3006
LmjF30.2390	*L. major*	40	3317	3356
LmjF30.2400	*L. major*	40	234	273
LmjF30.2430	*L. major*	495	272	766
LmjF30.2430	*L. major*	434	785	1218
LmjF30.2440	*L. major*	57	399	455
LmjF30.2440	*L. major*	55	513	567
LmjF30.2440	*L. major*	69	671	739
LmjF30.2450	*L. major*	161	67	227
LmjF30.2460	*L. major*	111	525	635
LmjF30.2470	*L. major*	149	514	662
LmjF30.2480	*L. major*	149	514	662

LmjF30.2490	*L. major*	147	514	660
LmjF30.2540	*L. major*	46	1	46
LmjF30.2540	*L. major*	61	214	274
LmjF30.2540	*L. major*	46	481	526
LmjF30.2550	*L. major*	139	514	652
LmjF30.2560	*L. major*	52	18	69
LmjF30.2560	*L. major*	135	89	223
LmjF30.2560	*L. major*	79	323	401
LmjF30.2560	*L. major*	48	574	621
LmjF30.2560	*L. major*	82	634	715
LmjF30.2560	*L. major*	41	775	815
LmjF30.2560	*L. major*	56	964	1019
LmjF30.2570	*L. major*	91	101	191
LmjF30.2570	*L. major*	51	332	382
LmjF30.2570	*L. major*	51	332	382
LmjF30.2610	*L. major*	76	1	76
LmjF30.2610	*L. major*	60	157	216
LmjF30.2620	*L. major*	209	1	209
LmjF30.2620	*L. major*	100	226	325
LmjF30.2620	*L. major*	117	582	698
LmjF30.2640	*L. major*	83	171	253
LmjF30.2640	*L. major*	327	505	831
LmjF30.2660	*L. major*	43	9	51
LmjF30.2660	*L. major*	64	127	190
LmjF30.2660	*L. major*	64	127	190
LmjF30.2690	*L. major*	110	56	165
LmjF30.2690	*L. major*	88	207	294
LmjF30.2700	*L. major*	46	271	316
LmjF30.2710	*L. major*	265	14	278
LmjF30.2710	*L. major*	135	280	414
LmjF30.2710	*L. major*	163	425	587
LmjF30.2710	*L. major*	82	596	677
LmjF30.2710	*L. major*	201	732	932
LmjF30.2720	*L. major*	62	17	78
LmjF30.2720	*L. major*	172	144	315
LmjF30.2720	*L. major*	47	516	562
LmjF30.2730	*L. major*	50	129	178
LmjF30.2730	*L. major*	171	217	387
LmjF30.2740	*L. major*	76	68	143
LmjF30.2740	*L. major*	116	228	343
LmjF30.2740	*L. major*	116	228	343
LmjF30.2750	*L. major*	58	177	234
LmjF30.2780	*L. major*	98	101	198
LmjF30.2780	*L. major*	66	225	290
LmjF30.2790	*L. major*	75	1	75
LmjF30.2800	*L. major*	84	31	114
LmjF30.2820	*L. major*	40	338	377

LmjF30.2830	*L. major*	764	1	764
LmjF30.2860	*L. major*	63	70	132
LmjF30.2870	*L. major*	40	224	263
LmjF30.2870	*L. major*	100	384	483
LmjF30.2880	*L. major*	87	35	121
LmjF30.2880	*L. major*	42	132	173
LmjF30.2890	*L. major*	47	411	457
LmjF30.2920	*L. major*	56	15	70
LmjF30.2920	*L. major*	378	79	456
LmjF30.2950	*L. major*	67	1	67
LmjF30.2960	*L. major*	91	135	225
LmjF30.3000	*L. major*	87	1	87
LmjF30.3010	*L. major*	239	160	398
LmjF30.3020	*L. major*	121	201	321
LmjF30.3030	*L. major*	77	179	255
LmjF30.3040	*L. major*	93	47	139
LmjF30.3040	*L. major*	59	274	332
LmjF30.3040	*L. major*	42	490	531
LmjF30.3050	*L. major*	44	1513	1556
LmjF30.3050	*L. major*	94	1806	1899
LmjF30.3050	*L. major*	94	1806	1899
LmjF30.3060	*L. major*	78	1	78
LmjF30.3060	*L. major*	136	430	565
LmjF30.3060	*L. major*	201	624	824
LmjF30.3060	*L. major*	52	848	899
LmjF30.3060	*L. major*	104	933	1036
LmjF30.3060	*L. major*	127	1065	1191
LmjF30.3070	*L. major*	119	1	119
LmjF30.3070	*L. major*	57	331	387
LmjF30.3090	*L. major*	139	128	266
LmjF30.3100	*L. major*	48	279	326
LmjF30.3100	*L. major*	40	587	626
LmjF30.3100	*L. major*	40	587	626
LmjF30.3130	*L. major*	61	907	967
LmjF30.3140	*L. major*	109	1	109
LmjF30.3170	*L. major*	381	1	381
LmjF30.3180	*L. major*	48	100	147
LmjF30.3210	*L. major*	49	64	112
LmjF30.3210	*L. major*	109	217	325
LmjF30.3210	*L. major*	212	358	569
LmjF30.3210	*L. major*	155	639	793
LmjF30.3240	*L. major*	42	142	183
LmjF30.3240	*L. major*	48	547	594
LmjF30.3240	*L. major*	48	547	594
LmjF30.3250	*L. major*	179	1	179
LmjF30.3250	*L. major*	125	447	571
LmjF30.3250	*L. major*	45	870	914

LmjF30.3260	*L. major*	122	290	411
LmjF30.3290	*L. major*	43	126	168
LmjF30.3290	*L. major*	43	238	280
LmjF30.3290	*L. major*	43	345	387
LmjF30.3290	*L. major*	101	467	567
LmjF30.3290	*L. major*	41	742	782
LmjF30.3290	*L. major*	161	880	1040
LmjF30.3300	*L. major*	107	57	163
LmjF30.3300	*L. major*	58	208	265
LmjF30.3300	*L. major*	54	306	359
LmjF30.3320	*L. major*	47	1	47
LmjF30.3320	*L. major*	130	229	358
LmjF30.3330	*L. major*	275	64	338
LmjF30.3360	*L. major*	108	515	622
LmjF30.3370	*L. major*	532	39	570
LmjF30.3390	*L. major*	52	883	934
LmjF30.3410	*L. major*	43	73	115
LmjF30.3410	*L. major*	100	231	330
LmjF30.3410	*L. major*	100	231	330
LmjF30.3420	*L. major*	64	1	64
LmjF30.3420	*L. major*	48	228	275
LmjF30.3420	*L. major*	132	332	463
LmjF30.3430	*L. major*	54	743	796
LmjF30.3440	*L. major*	51	1	51
LmjF30.3440	*L. major*	45	132	176
LmjF30.3440	*L. major*	45	132	176
LmjF30.3445	*L. major*	116	1	116
LmjF30.3450	*L. major*	42	32	73
LmjF30.3470	*L. major*	50	308	357
LmjF30.3480	*L. major*	89	77	165
LmjF30.3490	*L. major*	50	1	50
LmjF30.3510	*L. major*	50	1	50
LmjF30.3540	*L. major*	89	48	136
LmjF30.3540	*L. major*	51	171	221
LmjF30.3540	*L. major*	51	171	221
LmjF30.3570	*L. major*	46	1	46
LmjF30.3570	*L. major*	52	88	139
LmjF30.3570	*L. major*	112	155	266
LmjF30.3580	*L. major*	144	1	144
LmjF30.3580	*L. major*	86	299	384
LmjF30.3580	*L. major*	71	553	623
LmjF30.3580	*L. major*	102	646	747
LmjF30.3590	*L. major*	72	92	163
LmjF30.3610	*L. major*	43	171	213
LmjF30.3620	*L. major*	145	49	193
LmjF30.3620	*L. major*	159	212	370
LmjF30.3620	*L. major*	159	212	370

LmjF30.3650	*L. major*	51	39	89
LmjF30.3650	*L. major*	47	158	204
LmjF30.3650	*L. major*	47	158	204
LmjF30.3680	*L. major*	45	1	45
LmjF30.3680	*L. major*	43	87	129
LmjF30.3680	*L. major*	49	235	283
LmjF30.3690	*L. major*	73	69	141
LmjF30.3690	*L. major*	50	532	581
LmjF30.3690	*L. major*	50	532	581
LmjF30.3700	*L. major*	44	17	60
LmjF30.3710	*L. major*	48	269	316
LmjF30.3720	*L. major*	65	42	106
LmjF30.3730	*L. major*	65	61	125
LmjF30.3740	*L. major*	72	45	116
LmjF30.3740	*L. major*	91	132	222
LmjF31.0020	*L. major*	49	1	49
LmjF31.0030	*L. major*	46	66	111
LmjF31.0030	*L. major*	91	1159	1249
LmjF31.0040	*L. major*	88	10	97
LmjF31.0040	*L. major*	47	566	612
LmjF31.0040	*L. major*	47	566	612
LmjF31.0070	*L. major*	93	119	211
LmjF31.0070	*L. major*	46	875	920
LmjF31.0080	*L. major*	127	1	127
LmjF31.0080	*L. major*	80	152	231
LmjF31.0090	*L. major*	49	304	352
LmjF31.0110	*L. major*	96	179	274
LmjF31.0120	*L. major*	47	1	47
LmjF31.0130	*L. major*	729	1	729
LmjF31.0140	*L. major*	48	327	374
LmjF31.0160	*L. major*	45	884	928
LmjF31.0170	*L. major*	65	444	508
LmjF31.0190	*L. major*	223	1	223
LmjF31.0190	*L. major*	96	534	629
LmjF31.0200	*L. major*	45	202	246
LmjF31.0210	*L. major*	40	246	285
LmjF31.0220	*L. major*	115	1	115
LmjF31.0230	*L. major*	204	15	218
LmjF31.0230	*L. major*	178	257	434
LmjF31.0230	*L. major*	311	450	760
LmjF31.0240	*L. major*	41	1	41
LmjF31.0240	*L. major*	72	91	162
LmjF31.0250	*L. major*	51	1	51
LmjF31.0260	*L. major*	99	15	113
LmjF31.0260	*L. major*	57	238	294
LmjF31.0260	*L. major*	57	238	294
LmjF31.0290	*L. major*	222	1	222

LmjF31.0290	*L. major*	181	525	705
LmjF31.0290	*L. major*	48	793	840
LmjF31.0310	*L. major*	90	1	90
LmjF31.0310	*L. major*	104	365	468
LmjF31.0310	*L. major*	104	365	468
LmjF31.0320	*L. major*	99	1	99
LmjF31.0330	*L. major*	58	16	73
LmjF31.0340	*L. major*	40	1	40
LmjF31.0350	*L. major*	90	1	90
LmjF31.0360	*L. major*	125	286	410
LmjF31.0360	*L. major*	78	914	991
LmjF31.0360	*L. major*	78	914	991
LmjF31.0370	*L. major*	71	67	137
LmjF31.0370	*L. major*	71	142	212
LmjF31.0370	*L. major*	50	379	428
LmjF31.0380	*L. major*	89	1	89
LmjF31.0380	*L. major*	51	291	341
LmjF31.0380	*L. major*	94	570	663
LmjF31.0380	*L. major*	56	733	788
LmjF31.0380	*L. major*	70	807	876
LmjF31.0380	*L. major*	114	878	991
LmjF31.0380	*L. major*	44	1088	1131
LmjF31.0380	*L. major*	49	1332	1380
LmjF31.0390	*L. major*	153	30	182
LmjF31.0400	*L. major*	95	88	182
LmjF31.0430	*L. major*	68	151	218
LmjF31.0430	*L. major*	46	773	818
LmjF31.0430	*L. major*	46	773	818
LmjF31.0440	*L. major*	42	1	42
LmjF31.0480	*L. major*	91	233	323
LmjF31.0490	*L. major*	92	1	92
LmjF31.0490	*L. major*	85	416	500
LmjF31.0500	*L. major*	101	1	101
LmjF31.0500	*L. major*	75	144	218
LmjF31.0500	*L. major*	64	222	285
LmjF31.0500	*L. major*	146	356	501
LmjF31.0500	*L. major*	59	548	606
LmjF31.0510	*L. major*	63	33	95
LmjF31.0510	*L. major*	58	225	282
LmjF31.0510	*L. major*	62	536	597
LmjF31.0520	*L. major*	110	1	110
LmjF31.0520	*L. major*	46	251	296
LmjF31.0520	*L. major*	48	361	408
LmjF31.0530	*L. major*	542	1	542
LmjF31.0530	*L. major*	135	544	678
LmjF31.0540	*L. major*	71	42	112
LmjF31.0540	*L. major*	94	746	839

LmjF31.0550	*L. major*	144	1	144
LmjF31.0550	*L. major*	73	205	277
LmjF31.0550	*L. major*	170	316	485
LmjF31.0550	*L. major*	225	522	746
LmjF31.0550	*L. major*	64	911	974
LmjF31.0550	*L. major*	58	1067	1124
LmjF31.0570	*L. major*	42	1	42
LmjF31.0580	*L. major*	60	1	60
LmjF31.0580	*L. major*	43	77	119
LmjF31.0600	*L. major*	98	35	132
LmjF31.0600	*L. major*	103	203	305
LmjF31.0600	*L. major*	82	377	458
LmjF31.0610	*L. major*	91	1	91
LmjF31.0610	*L. major*	56	114	169
LmjF31.0610	*L. major*	82	179	260
LmjF31.0610	*L. major*	55	415	469
LmjF31.0620	*L. major*	85	88	172
LmjF31.0620	*L. major*	173	250	422
LmjF31.0620	*L. major*	54	638	691
LmjF31.0630	*L. major*	113	113	225
LmjF31.0630	*L. major*	67	311	377
LmjF31.0630	*L. major*	59	518	576
LmjF31.0640	*L. major*	44	177	220
LmjF31.0640	*L. major*	44	481	524
LmjF31.0640	*L. major*	44	481	524
LmjF31.0660	*L. major*	183	1	183
LmjF31.0660	*L. major*	44	190	233
LmjF31.0660	*L. major*	111	257	367
LmjF31.0660	*L. major*	101	386	486
LmjF31.0660	*L. major*	241	512	752
LmjF31.0660	*L. major*	47	817	863
LmjF31.0660	*L. major*	187	885	1071
LmjF31.0660	*L. major*	111	1166	1276
LmjF31.0660	*L. major*	112	1644	1755
LmjF31.0660	*L. major*	68	1782	1849
LmjF31.0670	*L. major*	301	1	301
LmjF31.0670	*L. major*	363	327	689
LmjF31.0670	*L. major*	168	697	864
LmjF31.0670	*L. major*	95	929	1023
LmjF31.0680	*L. major*	129	139	267
LmjF31.0690	*L. major*	115	156	270
LmjF31.0690	*L. major*	102	429	530
LmjF31.0690	*L. major*	102	429	530
LmjF31.0700	*L. major*	49	86	134
LmjF31.0700	*L. major*	55	207	261
LmjF31.0700	*L. major*	109	520	628
LmjF31.0710	*L. major*	74	56	129

LmjF31.0720	*L. major*	57	1	57
LmjF31.0720	*L. major*	51	83	133
LmjF31.0720	*L. major*	245	442	686
LmjF31.0740	*L. major*	114	75	188
LmjF31.0750	*L. major*	72	108	179
LmjF31.0750	*L. major*	83	215	297
LmjF31.0750	*L. major*	83	215	297
LmjF31.0760	*L. major*	72	117	188
LmjF31.0760	*L. major*	40	261	300
LmjF31.0760	*L. major*	40	261	300
LmjF31.0770	*L. major*	64	98	161
LmjF31.0780	*L. major*	123	152	274
LmjF31.0790	*L. major*	162	127	288
LmjF31.0800	*L. major*	81	94	174
LmjF31.0800	*L. major*	42	178	219
LmjF31.0800	*L. major*	42	178	219
LmjF31.0810	*L. major*	76	1	76
LmjF31.0810	*L. major*	128	150	277
LmjF31.0820	*L. major*	47	1	47
LmjF31.0820	*L. major*	42	51	92
LmjF31.0820	*L. major*	42	51	92
LmjF31.0850	*L. major*	64	98	161
LmjF31.0850	*L. major*	193	244	436
LmjF31.0860	*L. major*	51	192	242
LmjF31.0860	*L. major*	323	288	610
LmjF31.0870	*L. major*	63	1	63
LmjF31.0880	*L. major*	63	1	63
LmjF31.0890	*L. major*	115	44	158
LmjF31.0910	*L. major*	51	52	102
LmjF31.0920	*L. major*	76	64	139
LmjF31.0920	*L. major*	68	485	552
LmjF31.0930	*L. major*	76	64	139
LmjF31.0930	*L. major*	65	487	551
LmjF31.0940	*L. major*	61	13	73
LmjF31.0940	*L. major*	54	169	222
LmjF31.0940	*L. major*	70	234	303
LmjF31.0950	*L. major*	76	64	139
LmjF31.0950	*L. major*	65	489	553
LmjF31.0960	*L. major*	76	64	139
LmjF31.0960	*L. major*	65	487	551
LmjF31.0970	*L. major*	41	28	68
LmjF31.0970	*L. major*	84	105	188
LmjF31.0970	*L. major*	104	341	444
LmjF31.0970	*L. major*	227	557	783
LmjF31.0980	*L. major*	56	1	56
LmjF31.0980	*L. major*	249	475	723
LmjF31.0990	*L. major*	275	131	405

LmjF31.1000	*L. major*	46	1	46
LmjF31.1000	*L. major*	50	211	260
LmjF31.1000	*L. major*	459	548	1006
LmjF31.1010	*L. major*	175	1	175
LmjF31.1010	*L. major*	54	194	247
LmjF31.1010	*L. major*	62	525	586
LmjF31.1020	*L. major*	73	1	73
LmjF31.1020	*L. major*	95	113	207
LmjF31.1020	*L. major*	257	238	494
LmjF31.1020	*L. major*	52	712	763
LmjF31.1020	*L. major*	50	804	853
LmjF31.1020	*L. major*	72	858	929
LmjF31.1030	*L. major*	60	1	60
LmjF31.1030	*L. major*	86	105	190
LmjF31.1030	*L. major*	222	318	539
LmjF31.1030	*L. major*	40	727	766
LmjF31.1030	*L. major*	42	818	859
LmjF31.1040	*L. major*	71	1	71
LmjF31.1040	*L. major*	45	198	242
LmjF31.1040	*L. major*	69	269	337
LmjF31.1040	*L. major*	40	371	410
LmjF31.1040	*L. major*	43	505	547
LmjF31.1040	*L. major*	64	572	635
LmjF31.1050	*L. major*	388	1	388
LmjF31.1060	*L. major*	45	233	277
LmjF31.1060	*L. major*	56	403	458
LmjF31.1080	*L. major*	157	25	181
LmjF31.1080	*L. major*	777	198	974
LmjF31.1090	*L. major*	50	1	50
LmjF31.1090	*L. major*	489	130	618
LmjF31.1150	*L. major*	167	1	167
LmjF31.1150	*L. major*	73	251	323
LmjF31.1150	*L. major*	176	573	748
LmjF31.1150	*L. major*	155	784	938
LmjF31.1150	*L. major*	119	946	1064
LmjF31.1160	*L. major*	80	398	477
LmjF31.1160	*L. major*	43	541	583
LmjF31.1160	*L. major*	43	541	583
LmjF31.1180	*L. major*	68	68	135
LmjF31.1210	*L. major*	45	59	103
LmjF31.1210	*L. major*	58	352	409
LmjF31.1210	*L. major*	51	470	520
LmjF31.1210	*L. major*	42	733	774
LmjF31.1210	*L. major*	43	1084	1126
LmjF31.1210	*L. major*	41	1301	1341
LmjF31.1210	*L. major*	52	1555	1606
LmjF31.1210	*L. major*	64	1688	1751

LmjF31.1210	*L. major*	89	2131	2219
LmjF31.1210	*L. major*	94	2908	3001
LmjF31.1210	*L. major*	51	3044	3094
LmjF31.1210	*L. major*	40	3230	3269
LmjF31.1210	*L. major*	43	3560	3602
LmjF31.1210	*L. major*	45	3810	3854
LmjF31.1210	*L. major*	108	4263	4370
LmjF31.1210	*L. major*	57	4634	4690
LmjF31.1210	*L. major*	138	4759	4896
LmjF31.1230	*L. major*	82	350	431
LmjF31.1230	*L. major*	40	500	539
LmjF31.1230	*L. major*	131	691	821
LmjF31.1240	*L. major*	401	1	401
LmjF31.1240	*L. major*	320	407	726
LmjF31.1240	*L. major*	257	747	1003
LmjF31.1240	*L. major*	98	1087	1184
LmjF31.1240	*L. major*	80	1214	1293
LmjF31.1250	*L. major*	143	67	209
LmjF31.1250	*L. major*	69	320	388
LmjF31.1250	*L. major*	451	476	926
LmjF31.1250	*L. major*	164	928	1091
LmjF31.1260	*L. major*	84	85	168
LmjF31.1260	*L. major*	101	281	381
LmjF31.1260	*L. major*	130	434	563
LmjF31.1270	*L. major*	50	1	50
LmjF31.1270	*L. major*	41	182	222
LmjF31.1270	*L. major*	78	682	759
LmjF31.1270	*L. major*	68	977	1044
LmjF31.1280	*L. major*	40	131	170
LmjF31.1290	*L. major*	57	1	57
LmjF31.1290	*L. major*	88	166	253
LmjF31.1290	*L. major*	66	872	937
LmjF31.1300	*L. major*	203	1	203
LmjF31.1300	*L. major*	53	245	297
LmjF31.1300	*L. major*	616	308	923
LmjF31.1310	*L. major*	958	1	958
LmjF31.1310	*L. major*	159	965	1123
LmjF31.1310	*L. major*	149	1149	1297
LmjF31.1310	*L. major*	224	1305	1528
LmjF31.1320	*L. major*	578	1	578
LmjF31.1320	*L. major*	650	591	1240
LmjF31.1320	*L. major*	168	1246	1413
LmjF31.1320	*L. major*	73	1457	1529
LmjF31.1320	*L. major*	84	1689	1772
LmjF31.1320	*L. major*	115	1829	1943
LmjF31.1320	*L. major*	102	1998	2099
LmjF31.1320	*L. major*	47	2131	2177

LmjF31.1320	*L. major*	58	2444	2501
LmjF31.1330	*L. major*	595	1	595
LmjF31.1330	*L. major*	132	637	768
LmjF31.1330	*L. major*	119	933	1051
LmjF31.1330	*L. major*	285	1060	1344
LmjF31.1330	*L. major*	129	1394	1522
LmjF31.1330	*L. major*	250	1553	1802
LmjF31.1330	*L. major*	67	1839	1905
LmjF31.1340	*L. major*	46	1	46
LmjF31.1340	*L. major*	156	71	226
LmjF31.1340	*L. major*	98	228	325
LmjF31.1340	*L. major*	43	359	401
LmjF31.1350	*L. major*	91	1	91
LmjF31.1350	*L. major*	169	104	272
LmjF31.1350	*L. major*	44	307	350
LmjF31.1360	*L. major*	99	86	184
LmjF31.1360	*L. major*	152	225	376
LmjF31.1370	*L. major*	89	1	89
LmjF31.1370	*L. major*	748	91	838
LmjF31.1370	*L. major*	209	886	1094
LmjF31.1380	*L. major*	383	1	383
LmjF31.1380	*L. major*	342	385	726
LmjF31.1380	*L. major*	54	753	806
LmjF31.1380	*L. major*	136	813	948
LmjF31.1380	*L. major*	113	956	1068
LmjF31.1390	*L. major*	317	1	317
LmjF31.1390	*L. major*	246	347	592
LmjF31.1390	*L. major*	52	609	660
LmjF31.1395	*L. major*	53	74	126
LmjF31.1400	*L. major*	94	82	175
LmjF31.1400	*L. major*	139	197	335
LmjF31.1400	*L. major*	45	407	451
LmjF31.1400	*L. major*	78	532	609
LmjF31.1400	*L. major*	113	622	734
LmjF31.1400	*L. major*	72	836	907
LmjF31.1400	*L. major*	49	939	987
LmjF31.1400	*L. major*	173	1083	1255
LmjF31.1410	*L. major*	43	85	127
LmjF31.1410	*L. major*	70	357	426
LmjF31.1410	*L. major*	46	554	599
LmjF31.1410	*L. major*	104	644	747
LmjF31.1410	*L. major*	58	828	885
LmjF31.1410	*L. major*	57	951	1007
LmjF31.1410	*L. major*	57	1130	1186
LmjF31.1410	*L. major*	274	1325	1598
LmjF31.1410	*L. major*	544	1629	2172
LmjF31.1410	*L. major*	72	2194	2265

LmjF31.1410	*L. major*	107	2279	2385
LmjF31.1420	*L. major*	72	21	92
LmjF31.1420	*L. major*	81	152	232
LmjF31.1420	*L. major*	70	435	504
LmjF31.1420	*L. major*	91	631	721
LmjF31.1420	*L. major*	291	744	1034
LmjF31.1420	*L. major*	79	1059	1137
LmjF31.1430	*L. major*	47	663	709
LmjF31.1430	*L. major*	41	795	835
LmjF31.1430	*L. major*	144	966	1109
LmjF31.1430	*L. major*	62	1746	1807
LmjF31.1440	*L. major*	206	286	491
LmjF31.1445	*L. major*	140	273	412
LmjF31.1470	*L. major*	48	108	155
LmjF31.1470	*L. major*	118	211	328
LmjF31.1470	*L. major*	70	406	475
LmjF31.1470	*L. major*	53	503	555
LmjF31.1470	*L. major*	126	583	708
LmjF31.1470	*L. major*	84	783	866
LmjF31.1480	*L. major*	137	336	472
LmjF31.1480	*L. major*	51	511	561
LmjF31.1480	*L. major*	41	681	721
LmjF31.1480	*L. major*	106	769	874
LmjF31.1480	*L. major*	73	1149	1221
LmjF31.1480	*L. major*	48	1395	1442
LmjF31.1490	*L. major*	57	179	235
LmjF31.1490	*L. major*	91	424	514
LmjF31.1490	*L. major*	65	1154	1218
LmjF31.1490	*L. major*	67	1296	1362
LmjF31.1490	*L. major*	63	1393	1455
LmjF31.1500	*L. major*	89	80	168
LmjF31.1500	*L. major*	83	296	378
LmjF31.1500	*L. major*	73	437	509
LmjF31.1500	*L. major*	50	550	599
LmjF31.1500	*L. major*	152	804	955
LmjF31.1500	*L. major*	63	975	1037
LmjF31.1500	*L. major*	91	1049	1139
LmjF31.1510	*L. major*	40	330	369
LmjF31.1510	*L. major*	110	509	618
LmjF31.1510	*L. major*	41	745	785
LmjF31.1510	*L. major*	392	850	1241
LmjF31.1510	*L. major*	250	1288	1537
LmjF31.1510	*L. major*	160	1539	1698
LmjF31.1520	*L. major*	95	463	557
LmjF31.1520	*L. major*	247	624	870
LmjF31.1530	*L. major*	102	1	102
LmjF31.1530	*L. major*	94	128	221

LmjF31.1530	*L. major*	217	228	444
LmjF31.1530	*L. major*	45	483	527
LmjF31.1530	*L. major*	72	621	692
LmjF31.1530	*L. major*	154	708	861
LmjF31.1530	*L. major*	199	910	1108
LmjF31.1530	*L. major*	93	1116	1208
LmjF31.1530	*L. major*	156	1312	1467
LmjF31.1530	*L. major*	54	1570	1623
LmjF31.1530	*L. major*	40	1750	1789
LmjF31.1530	*L. major*	260	1839	2098
LmjF31.1530	*L. major*	111	2112	2222
LmjF31.1530	*L. major*	53	2227	2279
LmjF31.1540	*L. major*	183	34	216
LmjF31.1540	*L. major*	92	218	309
LmjF31.1540	*L. major*	77	333	409
LmjF31.1560	*L. major*	42	34	75
LmjF31.1560	*L. major*	55	268	322
LmjF31.1560	*L. major*	55	268	322
LmjF31.1600	*L. major*	45	20	64
LmjF31.1630	*L. major*	61	138	198
LmjF31.1640	*L. major*	61	220	280
LmjF31.1670	*L. major*	40	1	40
LmjF31.1680	*L. major*	162	57	218
LmjF31.1690	*L. major*	73	1	73
LmjF31.1690	*L. major*	47	314	360
LmjF31.1690	*L. major*	71	526	596
LmjF31.1690	*L. major*	47	806	852
LmjF31.1700	*L. major*	195	26	220
LmjF31.1700	*L. major*	59	304	362
LmjF31.1710	*L. major*	160	1	160
LmjF31.1710	*L. major*	96	199	294
LmjF31.1710	*L. major*	40	322	361
LmjF31.1710	*L. major*	40	567	606
LmjF31.1720	*L. major*	50	1	50
LmjF31.1720	*L. major*	66	728	793
LmjF31.1730	*L. major*	280	34	313
LmjF31.1730	*L. major*	56	326	381
LmjF31.1740	*L. major*	67	90	156
LmjF31.1740	*L. major*	56	161	216
LmjF31.1740	*L. major*	114	283	396
LmjF31.1740	*L. major*	90	433	522
LmjF31.1750	*L. major*	56	86	141
LmjF31.1750	*L. major*	72	236	307
LmjF31.1750	*L. major*	81	328	408
LmjF31.1760	*L. major*	79	1	79
LmjF31.1760	*L. major*	49	119	167
LmjF31.1760	*L. major*	52	290	341

LmjF31.1770	*L. major*	97	47	143
LmjF31.1770	*L. major*	65	320	384
LmjF31.1770	*L. major*	402	615	1016
LmjF31.1780	*L. major*	76	386	461
LmjF31.1790	*L. major*	63	1	63
LmjF31.1790	*L. major*	56	447	502
LmjF31.1800	*L. major*	45	1	45
LmjF31.1800	*L. major*	56	429	484
LmjF31.1820	*L. major*	63	1	63
LmjF31.1820	*L. major*	56	447	502
LmjF31.1830	*L. major*	79	537	615
LmjF31.1830	*L. major*	90	653	742
LmjF31.1840	*L. major*	90	652	741
LmjF31.1840	*L. major*	43	958	1000
LmjF31.1855	*L. major*	41	34	74
LmjF31.1860	*L. major*	40	15	54
LmjF31.1860	*L. major*	61	111	171
LmjF31.1860	*L. major*	61	111	171
LmjF31.1870	*L. major*	56	1	56
LmjF31.1870	*L. major*	66	901	966
LmjF31.1870	*L. major*	66	901	966
LmjF31.1880	*L. major*	66	781	846
LmjF31.1890	*L. major*	101	1	101
LmjF31.1910	*L. major*	275	17	291
LmjF31.1910	*L. major*	79	336	414
LmjF31.1910	*L. major*	101	423	523
LmjF31.1910	*L. major*	48	548	595
LmjF31.1910	*L. major*	200	618	817
LmjF31.1920	*L. major*	174	143	316
LmjF31.1920	*L. major*	217	618	834
LmjF31.1920	*L. major*	99	843	941
LmjF31.1920	*L. major*	114	1197	1310
LmjF31.1930	*L. major*	40	72	111
LmjF31.1930	*L. major*	84	533	616
LmjF31.1930	*L. major*	84	533	616
LmjF31.1940	*L. major*	274	53	326
LmjF31.1940	*L. major*	82	345	426
LmjF31.1940	*L. major*	55	431	485
LmjF31.1950	*L. major*	327	45	371
LmjF31.1950	*L. major*	161	412	572
LmjF31.1980	*L. major*	41	1	41
LmjF31.1980	*L. major*	71	150	220
LmjF31.1980	*L. major*	71	150	220
LmjF31.1990	*L. major*	337	49	385
LmjF31.1990	*L. major*	139	496	634
LmjF31.1990	*L. major*	40	670	709
LmjF31.2010	*L. major*	222	1	222

LmjF31.2010	*L. major*	331	237	567
LmjF31.2020	*L. major*	100	1	100
LmjF31.2040	*L. major*	69	15	83
LmjF31.2040	*L. major*	234	125	358
LmjF31.2040	*L. major*	234	125	358
LmjF31.2060	*L. major*	41	1	41
LmjF31.2060	*L. major*	40	341	380
LmjF31.2060	*L. major*	40	341	380
LmjF31.2070	*L. major*	56	131	186
LmjF31.2080	*L. major*	152	1	152
LmjF31.2080	*L. major*	51	230	280
LmjF31.2080	*L. major*	41	403	443
LmjF31.2080	*L. major*	57	655	711
LmjF31.2090	*L. major*	59	1	59
LmjF31.2090	*L. major*	177	106	282
LmjF31.2090	*L. major*	46	338	383
LmjF31.2100	*L. major*	56	1	56
LmjF31.2100	*L. major*	300	78	377
LmjF31.2110	*L. major*	495	1	495
LmjF31.2110	*L. major*	276	518	793
LmjF31.2110	*L. major*	67	826	892
LmjF31.2110	*L. major*	66	943	1008
LmjF31.2115	*L. major*	360	1	360
LmjF31.2120	*L. major*	74	29	102
LmjF31.2120	*L. major*	79	142	220
LmjF31.2120	*L. major*	152	233	384
LmjF31.2120	*L. major*	70	448	517
LmjF31.2120	*L. major*	225	539	763
LmjF31.2130	*L. major*	69	1	69
LmjF31.2130	*L. major*	43	345	387
LmjF31.2130	*L. major*	43	688	730
LmjF31.2140	*L. major*	1024	1	1024
LmjF31.2160	*L. major*	327	1	327
LmjF31.2160	*L. major*	62	348	409
LmjF31.2160	*L. major*	273	421	693
LmjF31.2180	*L. major*	291	1	291
LmjF31.2180	*L. major*	541	320	860
LmjF31.2190	*L. major*	358	1	358
LmjF31.2190	*L. major*	312	406	717
LmjF31.2200	*L. major*	84	1	84
LmjF31.2200	*L. major*	76	155	230
LmjF31.2200	*L. major*	94	233	326
LmjF31.2200	*L. major*	90	338	427
LmjF31.2210	*L. major*	42	116	157
LmjF31.2210	*L. major*	185	177	361
LmjF31.2210	*L. major*	80	384	463
LmjF31.2210	*L. major*	79	466	544

LmjF31.2210	*L. major*	75	570	644
LmjF31.2210	*L. major*	200	754	953
LmjF31.2220	*L. major*	96	1	96
LmjF31.2220	*L. major*	142	116	257
LmjF31.2220	*L. major*	97	339	435
LmjF31.2220	*L. major*	77	465	541
LmjF31.2220	*L. major*	86	656	741
LmjF31.2220	*L. major*	289	757	1045
LmjF31.2220	*L. major*	411	1083	1493
LmjF31.2220	*L. major*	185	1521	1705
LmjF31.2220	*L. major*	51	1748	1798
LmjF31.2220	*L. major*	210	1841	2050
LmjF31.2220	*L. major*	81	2053	2133
LmjF31.2230	*L. major*	280	1	280
LmjF31.2230	*L. major*	108	342	449
LmjF31.2240	*L. major*	84	69	152
LmjF31.2250	*L. major*	57	60	116
LmjF31.2250	*L. major*	65	316	380
LmjF31.2260	*L. major*	52	164	215
LmjF31.2260	*L. major*	45	534	578
LmjF31.2270	*L. major*	130	1	130
LmjF31.2320	*L. major*	49	46	94
LmjF31.2320	*L. major*	48	347	394
LmjF31.2320	*L. major*	123	564	686
LmjF31.2320	*L. major*	44	733	776
LmjF31.2320	*L. major*	57	880	936
LmjF31.2330	*L. major*	48	38	85
LmjF31.2340	*L. major*	86	1	86
LmjF31.2350	*L. major*	61	1	61
LmjF31.2350	*L. major*	57	217	273
LmjF31.2350	*L. major*	51	618	668
LmjF31.2350	*L. major*	44	1045	1088
LmjF31.2350	*L. major*	56	1350	1405
LmjF31.2360	*L. major*	118	52	169
LmjF31.2360	*L. major*	75	184	258
LmjF31.2360	*L. major*	150	280	429
LmjF31.2360	*L. major*	53	432	484
LmjF31.2370	*L. major*	82	43	124
LmjF31.2380	*L. major*	98	1	98
LmjF31.2380	*L. major*	81	143	223
LmjF31.2380	*L. major*	88	253	340
LmjF31.2380	*L. major*	310	366	675
LmjF31.2380	*L. major*	52	709	760
LmjF31.2380	*L. major*	47	770	816
LmjF31.2390	*L. major*	74	205	278
LmjF31.2400	*L. major*	101	53	153
LmjF31.2400	*L. major*	185	166	350

LmjF31.2400	*L. major*	59	417	475
LmjF31.2400	*L. major*	126	525	650
LmjF31.2400	*L. major*	102	704	805
LmjF31.2400	*L. major*	50	899	948
LmjF31.2400	*L. major*	68	1058	1125
LmjF31.2400	*L. major*	68	1158	1225
LmjF31.2410	*L. major*	77	19	95
LmjF31.2410	*L. major*	167	131	297
LmjF31.2420	*L. major*	64	1	64
LmjF31.2430	*L. major*	144	1	144
LmjF31.2440	*L. major*	43	105	147
LmjF31.2470	*L. major*	50	96	145
LmjF31.2480	*L. major*	68	1	68
LmjF31.2510	*L. major*	78	1	78
LmjF31.2520	*L. major*	96	567	662
LmjF31.2520	*L. major*	69	779	847
LmjF31.2520	*L. major*	69	779	847
LmjF31.2530	*L. major*	96	567	662
LmjF31.2530	*L. major*	49	777	825
LmjF31.2530	*L. major*	49	777	825
LmjF31.2540	*L. major*	96	587	682
LmjF31.2540	*L. major*	49	797	845
LmjF31.2540	*L. major*	49	797	845
LmjF31.2550	*L. major*	50	46	95
LmjF31.2550	*L. major*	157	543	699
LmjF31.2550	*L. major*	52	738	789
LmjF31.2560	*L. major*	53	1	53
LmjF31.2560	*L. major*	68	72	139
LmjF31.2560	*L. major*	65	197	261
LmjF31.2560	*L. major*	40	356	395
LmjF31.2560	*L. major*	229	662	890
LmjF31.2560	*L. major*	70	945	1014
LmjF31.2570	*L. major*	185	114	298
LmjF31.2570	*L. major*	274	551	824
LmjF31.2570	*L. major*	213	837	1049
LmjF31.2570	*L. major*	41	1071	1111
LmjF31.2590	*L. major*	47	523	569
LmjF31.2590	*L. major*	41	663	703
LmjF31.2590	*L. major*	41	663	703
LmjF31.2600	*L. major*	91	194	284
LmjF31.2600	*L. major*	50	351	400
LmjF31.2610	*L. major*	42	675	716
LmjF31.2610	*L. major*	74	870	943
LmjF31.2610	*L. major*	52	1463	1514
LmjF31.2610	*L. major*	125	1538	1662
LmjF31.2620	*L. major*	68	16	83
LmjF31.2630	*L. major*	70	23	92

LmjF31.2640	*L. major*	107	1	107
LmjF31.2655	*L. major*	577	1	577
LmjF31.2670	*L. major*	109	50	158
LmjF31.2670	*L. major*	162	228	389
LmjF31.2670	*L. major*	97	426	522
LmjF31.2670	*L. major*	52	571	622
LmjF31.2670	*L. major*	198	644	841
LmjF31.2690	*L. major*	83	309	391
LmjF31.2690	*L. major*	47	439	485
LmjF31.2690	*L. major*	41	506	546
LmjF31.2690	*L. major*	78	656	733
LmjF31.2690	*L. major*	50	751	800
LmjF31.2690	*L. major*	46	983	1028
LmjF31.2690	*L. major*	53	1036	1088
LmjF31.2690	*L. major*	51	1208	1258
LmjF31.2700	*L. major*	53	632	684
LmjF31.2700	*L. major*	51	781	831
LmjF31.2700	*L. major*	51	781	831
LmjF31.2710	*L. major*	453	265	717
LmjF31.2720	*L. major*	54	18	71
LmjF31.2720	*L. major*	42	105	146
LmjF31.2720	*L. major*	42	105	146
LmjF31.2750	*L. major*	204	1	204
LmjF31.2750	*L. major*	158	241	398
LmjF31.2760	*L. major*	800	1	800
LmjF31.2760	*L. major*	119	809	927
LmjF31.2760	*L. major*	250	942	1191
LmjF31.2760	*L. major*	48	1240	1287
LmjF31.2770	*L. major*	95	117	211
LmjF31.2770	*L. major*	85	228	312
LmjF31.2780	*L. major*	228	10	237
LmjF31.2800	*L. major*	183	1	183
LmjF31.2810	*L. major*	112	68	179
LmjF31.2810	*L. major*	49	231	279
LmjF31.2820	*L. major*	51	220	270
LmjF31.2830	*L. major*	215	14	228
LmjF31.2830	*L. major*	251	375	625
LmjF31.2840	*L. major*	106	1	106
LmjF31.2850	*L. major*	76	71	146
LmjF31.2860	*L. major*	129	1	129
LmjF31.2860	*L. major*	73	139	211
LmjF31.2860	*L. major*	55	253	307
LmjF31.2880	*L. major*	48	1	48
LmjF31.2880	*L. major*	43	244	286
LmjF31.2880	*L. major*	43	244	286
LmjF31.2890	*L. major*	102	125	226
LmjF31.2890	*L. major*	42	295	336

LmjF31.2890	*L. major*	56	811	866
LmjF31.2890	*L. major*	49	1134	1182
LmjF31.2890	*L. major*	49	1192	1240
LmjF31.2900	*L. major*	190	1	190
LmjF31.2900	*L. major*	73	337	409
LmjF31.2900	*L. major*	73	337	409
LmjF31.2910	*L. major*	166	82	247
LmjF31.2950	*L. major*	40	1	40
LmjF31.2970	*L. major*	83	1325	1407
LmjF31.2970	*L. major*	43	2126	2168
LmjF31.2970	*L. major*	43	2126	2168
LmjF31.2980	*L. major*	49	702	750
LmjF31.2980	*L. major*	56	756	811
LmjF31.2980	*L. major*	169	819	987
LmjF31.2990	*L. major*	52	1	52
LmjF31.2990	*L. major*	52	136	187
LmjF31.2990	*L. major*	152	223	374
LmjF31.2990	*L. major*	58	437	494
LmjF31.2990	*L. major*	113	517	629
LmjF31.2990	*L. major*	96	743	838
LmjF31.2990	*L. major*	42	919	960
LmjF31.2990	*L. major*	104	1031	1134
LmjF31.2990	*L. major*	79	1211	1289
LmjF31.2990	*L. major*	114	1337	1450
LmjF31.2990	*L. major*	226	1465	1690
LmjF31.2990	*L. major*	170	1766	1935
LmjF31.3000	*L. major*	58	155	212
LmjF31.3000	*L. major*	317	301	617
LmjF31.3000	*L. major*	177	687	863
LmjF31.3000	*L. major*	40	889	928
LmjF31.3010	*L. major*	41	56	96
LmjF31.3010	*L. major*	107	120	226
LmjF31.3010	*L. major*	82	260	341
LmjF31.3010	*L. major*	148	374	521
LmjF31.3020	*L. major*	79	206	284
LmjF31.3020	*L. major*	82	287	368
LmjF31.3020	*L. major*	152	443	594
LmjF31.3030	*L. major*	116	60	175
LmjF31.3030	*L. major*	645	213	857
LmjF31.3030	*L. major*	63	893	955
LmjF31.3030	*L. major*	191	1030	1220
LmjF31.3030	*L. major*	73	1222	1294
LmjF31.3030	*L. major*	88	1311	1398
LmjF31.3030	*L. major*	246	1400	1645
LmjF31.3030	*L. major*	108	1667	1774
LmjF31.3040	*L. major*	87	1	87
LmjF31.3040	*L. major*	96	153	248

LmjF31.3040	*L. major*	96	153	248
LmjF31.3060	*L. major*	95	162	256
LmjF31.3070	*L. major*	95	162	256
LmjF31.3090	*L. major*	65	491	555
LmjF31.3090	*L. major*	54	1053	1106
LmjF31.3110	*L. major*	846	1	846
LmjF31.3120	*L. major*	48	535	582
LmjF31.3150	*L. major*	47	657	703
LmjF31.3150	*L. major*	89	968	1056
LmjF31.3150	*L. major*	72	1072	1143
LmjF31.3150	*L. major*	58	1412	1469
LmjF31.3170	*L. major*	40	323	362
LmjF31.3180	*L. major*	49	1	49
LmjF31.3190	*L. major*	90	1	90
LmjF31.3190	*L. major*	51	136	186
LmjF32.0020	*L. major*	58	57	114
LmjF32.0020	*L. major*	209	203	411
LmjF32.0030	*L. major*	96	243	338
LmjF32.0040	*L. major*	98	245	342
LmjF32.0060	*L. major*	75	1	75
LmjF32.0060	*L. major*	50	262	311
LmjF32.0060	*L. major*	50	262	311
LmjF32.0070	*L. major*	152	1	152
LmjF32.0080	*L. major*	48	1	48
LmjF32.0090	*L. major*	43	155	197
LmjF32.0120	*L. major*	156	1	156
LmjF32.0120	*L. major*	45	166	210
LmjF32.0120	*L. major*	135	545	679
LmjF32.0120	*L. major*	183	732	914
LmjF32.0120	*L. major*	113	964	1076
LmjF32.0140	*L. major*	53	53	105
LmjF32.0140	*L. major*	84	117	200
LmjF32.0140	*L. major*	43	819	861
LmjF32.0150	*L. major*	277	1	277
LmjF32.0150	*L. major*	55	327	381
LmjF32.0150	*L. major*	502	385	886
LmjF32.0150	*L. major*	71	929	999
LmjF32.0160	*L. major*	146	1	146
LmjF32.0160	*L. major*	74	195	268
LmjF32.0160	*L. major*	106	281	386
LmjF32.0170	*L. major*	134	175	308
LmjF32.0190	*L. major*	55	551	605
LmjF32.0200	*L. major*	120	63	182
LmjF32.0200	*L. major*	149	195	343
LmjF32.0210	*L. major*	122	423	544
LmjF32.0220	*L. major*	116	89	204
LmjF32.0220	*L. major*	151	271	421

LmjF32.0220	*L. major*	43	580	622
LmjF32.0220	*L. major*	121	732	852
LmjF32.0240	*L. major*	46	172	217
LmjF32.0240	*L. major*	154	417	570
LmjF32.0240	*L. major*	482	604	1085
LmjF32.0250	*L. major*	239	1	239
LmjF32.0250	*L. major*	51	350	400
LmjF32.0250	*L. major*	73	697	769
LmjF32.0250	*L. major*	102	1311	1412
LmjF32.0250	*L. major*	117	1417	1533
LmjF32.0250	*L. major*	250	1537	1786
LmjF32.0260	*L. major*	1089	261	1349
LmjF32.0270	*L. major*	61	838	898
LmjF32.0270	*L. major*	121	1182	1302
LmjF32.0290	*L. major*	123	99	221
LmjF32.0300	*L. major*	94	1	94
LmjF32.0300	*L. major*	55	110	164
LmjF32.0300	*L. major*	63	455	517
LmjF32.0300	*L. major*	171	582	752
LmjF32.0310	*L. major*	55	596	650
LmjF32.0310	*L. major*	55	727	781
LmjF32.0310	*L. major*	48	1505	1552
LmjF32.0330	*L. major*	161	1	161
LmjF32.0330	*L. major*	44	412	455
LmjF32.0330	*L. major*	478	601	1078
LmjF32.0330	*L. major*	49	1188	1236
LmjF32.0330	*L. major*	56	1272	1327
LmjF32.0330	*L. major*	182	1372	1553
LmjF32.0340	*L. major*	44	74	117
LmjF32.0340	*L. major*	264	361	624
LmjF32.0350	*L. major*	195	33	227
LmjF32.0350	*L. major*	100	371	470
LmjF32.0360	*L. major*	203	1	203
LmjF32.0360	*L. major*	86	209	294
LmjF32.0360	*L. major*	60	307	366
LmjF32.0370	*L. major*	47	297	343
LmjF32.0370	*L. major*	58	678	735
LmjF32.0370	*L. major*	58	678	735
LmjF32.0380	*L. major*	228	1	228
LmjF32.0380	*L. major*	118	257	374
LmjF32.0400	*L. major*	110	1	110
LmjF32.0400	*L. major*	102	513	614
LmjF32.0410	*L. major*	59	1	59
LmjF32.0410	*L. major*	51	262	312
LmjF32.0410	*L. major*	43	357	399
LmjF32.0440	*L. major*	57	22	78
LmjF32.0450	*L. major*	47	1	47

LmjF32.0470	*L. major*	112	146	257
LmjF32.0470	*L. major*	125	341	465
LmjF32.0473	*L. major*	44	1	44
LmjF32.0480	*L. major*	69	1	69
LmjF32.0480	*L. major*	47	114	160
LmjF32.0480	*L. major*	86	298	383
LmjF32.0510	*L. major*	41	205	245
LmjF32.0530	*L. major*	123	23	145
LmjF32.0540	*L. major*	80	218	297
LmjF32.0560	*L. major*	47	80	126
LmjF32.0570	*L. major*	107	1	107
LmjF32.0570	*L. major*	69	512	580
LmjF32.0570	*L. major*	69	512	580
LmjF32.0580	*L. major*	148	113	260
LmjF32.0580	*L. major*	207	316	522
LmjF32.0590	*L. major*	80	93	172
LmjF32.0590	*L. major*	48	225	272
LmjF32.0600	*L. major*	71	382	452
LmjF32.0600	*L. major*	43	518	560
LmjF32.0600	*L. major*	43	518	560
LmjF32.0610	*L. major*	47	161	207
LmjF32.0620	*L. major*	239	1	239
LmjF32.0620	*L. major*	40	326	365
LmjF32.0640	*L. major*	60	1	60
LmjF32.0640	*L. major*	108	498	605
LmjF32.0650	*L. major*	50	32	81
LmjF32.0660	*L. major*	45	1	45
LmjF32.0660	*L. major*	45	119	163
LmjF32.0660	*L. major*	45	119	163
LmjF32.0670	*L. major*	47	198	244
LmjF32.0680	*L. major*	130	335	464
LmjF32.0680	*L. major*	153	515	667
LmjF32.0680	*L. major*	310	808	1117
LmjF32.0690	*L. major*	62	1	62
LmjF32.0700	*L. major*	86	149	234
LmjF32.0720	*L. major*	113	1	113
LmjF32.0730	*L. major*	331	267	597
LmjF32.0740	*L. major*	41	82	122
LmjF32.0750	*L. major*	61	1	61
LmjF32.0770	*L. major*	166	204	369
LmjF32.0770	*L. major*	43	806	848
LmjF32.0770	*L. major*	103	964	1066
LmjF32.0770	*L. major*	70	1298	1367
LmjF32.0780	*L. major*	98	91	188
LmjF32.0780	*L. major*	258	515	772
LmjF32.0800	*L. major*	62	108	169
LmjF32.0800	*L. major*	96	280	375

LmjF32.0800	*L. major*	47	475	521
LmjF32.0810	*L. major*	95	1	95
LmjF32.0810	*L. major*	189	891	1079
LmjF32.0830	*L. major*	258	197	454
LmjF32.0840	*L. major*	306	1	306
LmjF32.0850	*L. major*	175	34	208
LmjF32.0850	*L. major*	187	282	468
LmjF32.0850	*L. major*	108	656	763
LmjF32.0860	*L. major*	45	326	370
LmjF32.0860	*L. major*	48	426	473
LmjF32.0860	*L. major*	52	528	579
LmjF32.0870	*L. major*	40	134	173
LmjF32.0900	*L. major*	77	57	133
LmjF32.0920	*L. major*	47	75	121
LmjF32.0920	*L. major*	76	692	767
LmjF32.0920	*L. major*	76	692	767
LmjF32.0930	*L. major*	73	632	704
LmjF32.0940	*L. major*	150	251	400
LmjF32.0940	*L. major*	168	656	823
LmjF32.0940	*L. major*	168	656	823
LmjF32.0950	*L. major*	40	127	166
LmjF32.0950	*L. major*	48	305	352
LmjF32.0960	*L. major*	87	148	234
LmjF32.0970	*L. major*	45	48	92
LmjF32.0970	*L. major*	44	159	202
LmjF32.0970	*L. major*	98	451	548
LmjF32.0970	*L. major*	60	609	668
LmjF32.0970	*L. major*	78	710	787
LmjF32.0970	*L. major*	81	973	1053
LmjF32.0970	*L. major*	55	1116	1170
LmjF32.0970	*L. major*	47	1245	1291
LmjF32.0970	*L. major*	127	1357	1483
LmjF32.0970	*L. major*	281	1528	1808
LmjF32.0980	*L. major*	93	1	93
LmjF32.0980	*L. major*	60	96	155
LmjF32.0980	*L. major*	87	283	369
LmjF32.0980	*L. major*	43	439	481
LmjF32.0985	*L. major*	49	334	382
LmjF32.0990	*L. major*	521	1	521
LmjF32.0990	*L. major*	113	527	639
LmjF32.0990	*L. major*	84	1113	1196
LmjF32.0990	*L. major*	49	1309	1357
LmjF32.0990	*L. major*	42	1394	1435
LmjF32.1000	*L. major*	50	230	279
LmjF32.1010	*L. major*	149	196	344
LmjF32.1020	*L. major*	46	50	95
LmjF32.1020	*L. major*	195	169	363

LmjF32.1020	*L. major*	43	744	786
LmjF32.1040	*L. major*	44	49	92
LmjF32.1040	*L. major*	78	145	222
LmjF32.1040	*L. major*	100	254	353
LmjF32.1040	*L. major*	88	397	484
LmjF32.1060	*L. major*	43	34	76
LmjF32.1060	*L. major*	93	509	601
LmjF32.1060	*L. major*	93	509	601
LmjF32.1080	*L. major*	44	145	188
LmjF32.1090	*L. major*	52	297	348
LmjF32.1090	*L. major*	76	365	440
LmjF32.1090	*L. major*	106	831	936
LmjF32.1090	*L. major*	63	957	1019
LmjF32.1130	*L. major*	828	1	828
LmjF32.1130	*L. major*	367	833	1199
LmjF32.1130	*L. major*	186	1201	1386
LmjF32.1130	*L. major*	186	1422	1607
LmjF32.1140	*L. major*	111	1	111
LmjF32.1140	*L. major*	79	281	359
LmjF32.1140	*L. major*	49	428	476
LmjF32.1160	*L. major*	67	315	381
LmjF32.1170	*L. major*	83	279	361
LmjF32.1180	*L. major*	120	149	268
LmjF32.1180	*L. major*	248	413	660
LmjF32.1190	*L. major*	198	1	198
LmjF32.1190	*L. major*	135	252	386
LmjF32.1190	*L. major*	96	440	535
LmjF32.1210	*L. major*	52	370	421
LmjF32.1220	*L. major*	150	400	549
LmjF32.1220	*L. major*	118	571	688
LmjF32.1220	*L. major*	84	714	797
LmjF32.1230	*L. major*	157	246	402
LmjF32.1240	*L. major*	54	112	165
LmjF32.1240	*L. major*	86	460	545
LmjF32.1240	*L. major*	86	460	545
LmjF32.1250	*L. major*	126	72	197
LmjF32.1270	*L. major*	70	1	70
LmjF32.1270	*L. major*	184	171	354
LmjF32.1270	*L. major*	41	455	495
LmjF32.1270	*L. major*	60	590	649
LmjF32.1290	*L. major*	114	55	168
LmjF32.1290	*L. major*	43	193	235
LmjF32.1290	*L. major*	41	295	335
LmjF32.1300	*L. major*	57	14	70
LmjF32.1310	*L. major*	272	111	382
LmjF32.1320	*L. major*	256	90	345
LmjF32.1320	*L. major*	59	390	448

LmjF32.1330	*L. major*	141	1	141
LmjF32.1340	*L. major*	110	174	283
LmjF32.1340	*L. major*	45	662	706
LmjF32.1350	*L. major*	87	1	87
LmjF32.1360	*L. major*	136	1	136
LmjF32.1370	*L. major*	255	1	255
LmjF32.1370	*L. major*	45	285	329
LmjF32.1380	*L. major*	226	83	308
LmjF32.1390	*L. major*	50	98	147
LmjF32.1390	*L. major*	107	646	752
LmjF32.1400	*L. major*	49	629	677
LmjF32.1400	*L. major*	69	894	962
LmjF32.1400	*L. major*	77	971	1047
LmjF32.1400	*L. major*	407	1050	1456
LmjF32.1400	*L. major*	217	1475	1691
LmjF32.1430	*L. major*	486	1	486
LmjF32.1430	*L. major*	186	808	993
LmjF32.1460	*L. major*	52	206	257
LmjF32.1460	*L. major*	47	719	765
LmjF32.1460	*L. major*	85	866	950
LmjF32.1460	*L. major*	58	1397	1454
LmjF32.1460	*L. major*	45	2389	2433
LmjF32.1470	*L. major*	104	119	222
LmjF32.1480	*L. major*	62	189	250
LmjF32.1480	*L. major*	51	422	472
LmjF32.1490	*L. major*	190	1	190
LmjF32.1495	*L. major*	59	60	118
LmjF32.1495	*L. major*	106	144	249
LmjF32.1495	*L. major*	106	144	249
LmjF32.1500	*L. major*	116	1	116
LmjF32.1500	*L. major*	57	164	220
LmjF32.1500	*L. major*	50	415	464
LmjF32.1510	*L. major*	127	104	230
LmjF32.1510	*L. major*	162	540	701
LmjF32.1520	*L. major*	55	228	282
LmjF32.1550	*L. major*	93	107	199
LmjF32.1560	*L. major*	78	1	78
LmjF32.1560	*L. major*	96	786	881
LmjF32.1570	*L. major*	91	128	218
LmjF32.1570	*L. major*	493	239	731
LmjF32.1600	*L. major*	129	1	129
LmjF32.1600	*L. major*	411	156	566
LmjF32.1600	*L. major*	41	627	667
LmjF32.1620	*L. major*	46	290	335
LmjF32.1620	*L. major*	49	719	767
LmjF32.1635	*L. major*	74	16	89
LmjF32.1635	*L. major*	120	131	250

LmjF32.1640	*L. major*	83	140	222
LmjF32.1650	*L. major*	212	1	212
LmjF32.1650	*L. major*	144	234	377
LmjF32.1650	*L. major*	42	458	499
LmjF32.1650	*L. major*	49	664	712
LmjF32.1660	*L. major*	436	1	436
LmjF32.1660	*L. major*	120	445	564
LmjF32.1660	*L. major*	333	590	922
LmjF32.1660	*L. major*	51	945	995
LmjF32.1660	*L. major*	76	1004	1079
LmjF32.1660	*L. major*	41	1109	1149
LmjF32.1690	*L. major*	42	1	42
LmjF32.1690	*L. major*	170	394	563
LmjF32.1700	*L. major*	52	213	264
LmjF32.1700	*L. major*	65	289	353
LmjF32.1700	*L. major*	127	383	509
LmjF32.1710	*L. major*	78	92	169
LmjF32.1710	*L. major*	80	298	377
LmjF32.1710	*L. major*	48	381	428
LmjF32.1710	*L. major*	43	629	671
LmjF32.1720	*L. major*	61	189	249
LmjF32.1720	*L. major*	134	537	670
LmjF32.1740	*L. major*	63	67	129
LmjF32.1750	*L. major*	63	371	433
LmjF32.1750	*L. major*	57	573	629
LmjF32.1750	*L. major*	63	718	780
LmjF32.1760	*L. major*	46	101	146
LmjF32.1760	*L. major*	76	199	274
LmjF32.1760	*L. major*	121	337	457
LmjF32.1770	*L. major*	43	30	72
LmjF32.1770	*L. major*	43	349	391
LmjF32.1770	*L. major*	45	452	496
LmjF32.1790	*L. major*	58	278	335
LmjF32.1790	*L. major*	50	605	654
LmjF32.1790	*L. major*	50	605	654
LmjF32.1800	*L. major*	243	1	243
LmjF32.1802	*L. major*	67	41	107
LmjF32.1810	*L. major*	95	28	122
LmjF32.1850	*L. major*	54	541	594
LmjF32.1860	*L. major*	42	200	241
LmjF32.1860	*L. major*	60	256	315
LmjF32.1870	*L. major*	48	175	222
LmjF32.1870	*L. major*	249	377	625
LmjF32.1875	*L. major*	77	1	77
LmjF32.1880	*L. major*	97	1	97
LmjF32.1880	*L. major*	224	126	349
LmjF32.1880	*L. major*	105	377	481

LmjF32.1890	*L. major*	49	30	78
LmjF32.1890	*L. major*	44	507	550
LmjF32.1890	*L. major*	45	584	628
LmjF32.1900	*L. major*	100	124	223
LmjF32.1900	*L. major*	85	289	373
LmjF32.1900	*L. major*	56	382	437
LmjF32.1900	*L. major*	50	529	578
LmjF32.1910	*L. major*	124	327	450
LmjF32.1930	*L. major*	344	1	344
LmjF32.1930	*L. major*	395	387	781
LmjF32.1950	*L. major*	73	25	97
LmjF32.1950	*L. major*	122	303	424
LmjF32.1960	*L. major*	42	263	304
LmjF32.1965	*L. major*	76	1	76
LmjF32.1965	*L. major*	65	115	179
LmjF32.1965	*L. major*	91	231	321
LmjF32.1965	*L. major*	126	329	454
LmjF32.1970	*L. major*	41	1	41
LmjF32.1970	*L. major*	89	209	297
LmjF32.1975	*L. major*	150	1	150
LmjF32.1980	*L. major*	72	116	187
LmjF32.1980	*L. major*	89	244	332
LmjF32.1980	*L. major*	110	398	507
LmjF32.1980	*L. major*	112	515	626
LmjF32.1980	*L. major*	96	649	744
LmjF32.1990	*L. major*	111	1	111
LmjF32.2000	*L. major*	45	214	258
LmjF32.2000	*L. major*	100	270	369
LmjF32.2020	*L. major*	44	78	121
LmjF32.2040	*L. major*	149	1	149
LmjF32.2040	*L. major*	105	153	257
LmjF32.2040	*L. major*	127	470	596
LmjF32.2040	*L. major*	68	605	672
LmjF32.2040	*L. major*	107	692	798
LmjF32.2070	*L. major*	43	110	152
LmjF32.2080	*L. major*	87	39	125
LmjF32.2080	*L. major*	64	185	248
LmjF32.2080	*L. major*	41	298	338
LmjF32.2080	*L. major*	66	402	467
LmjF32.2100	*L. major*	50	101	150
LmjF32.2100	*L. major*	334	338	671
LmjF32.2100	*L. major*	257	679	935
LmjF32.2110	*L. major*	100	50	149
LmjF32.2110	*L. major*	68	218	285
LmjF32.2110	*L. major*	93	473	565
LmjF32.2110	*L. major*	42	574	615
LmjF32.2130	*L. major*	115	64	178

LmjF32.2130	*L. major*	166	239	404
LmjF32.2155	*L. major*	123	16	138
LmjF32.2155	*L. major*	57	181	237
LmjF32.2155	*L. major*	75	339	413
LmjF32.2155	*L. major*	46	989	1034
LmjF32.2160	*L. major*	173	1133	1305
LmjF32.2170	*L. major*	180	27	206
LmjF32.2190	*L. major*	353	1	353
LmjF32.2190	*L. major*	103	483	585
LmjF32.2200	*L. major*	98	1	98
LmjF32.2210	*L. major*	65	142	206
LmjF32.2220	*L. major*	732	86	817
LmjF32.2220	*L. major*	184	849	1032
LmjF32.2220	*L. major*	58	1045	1102
LmjF32.2220	*L. major*	55	1119	1173
LmjF32.2220	*L. major*	122	1241	1362
LmjF32.2230	*L. major*	202	1	202
LmjF32.2230	*L. major*	62	430	491
LmjF32.2240	*L. major*	44	7	50
LmjF32.2240	*L. major*	75	109	183
LmjF32.2240	*L. major*	113	313	425
LmjF32.2240	*L. major*	48	491	538
LmjF32.2240	*L. major*	166	737	902
LmjF32.2250	*L. major*	71	1	71
LmjF32.2250	*L. major*	80	157	236
LmjF32.2250	*L. major*	43	271	313
LmjF32.2260	*L. major*	74	114	187
LmjF32.2270	*L. major*	45	1	45
LmjF32.2270	*L. major*	509	213	721
LmjF32.2270	*L. major*	164	724	887
LmjF32.2280	*L. major*	55	230	284
LmjF32.2290	*L. major*	140	1	140
LmjF32.2290	*L. major*	42	527	568
LmjF32.2300	*L. major*	143	17	159
LmjF32.2300	*L. major*	81	195	275
LmjF32.2300	*L. major*	175	279	453
LmjF32.2310	*L. major*	116	150	265
LmjF32.2310	*L. major*	41	586	626
LmjF32.2310	*L. major*	63	693	755
LmjF32.2310	*L. major*	46	867	912
LmjF32.2310	*L. major*	167	950	1116
LmjF32.2310	*L. major*	47	1149	1195
LmjF32.2350	*L. major*	457	1	457
LmjF32.2350	*L. major*	115	491	605
LmjF32.2360	*L. major*	76	48	123
LmjF32.2380	*L. major*	163	1	163
LmjF32.2380	*L. major*	116	478	593

LmjF32.2390	*L. major*	93	77	169
LmjF32.2390	*L. major*	294	195	488
LmjF32.2410	*L. major*	57	1	57
LmjF32.2410	*L. major*	62	167	228
LmjF32.2410	*L. major*	82	311	392
LmjF32.2410	*L. major*	81	467	547
LmjF32.2410	*L. major*	296	553	848
LmjF32.2410	*L. major*	82	870	951
LmjF32.2410	*L. major*	999	995	1993
LmjF32.2420	*L. major*	61	114	174
LmjF32.2420	*L. major*	43	215	257
LmjF32.2420	*L. major*	79	352	430
LmjF32.2420	*L. major*	40	536	575
LmjF32.2420	*L. major*	94	652	745
LmjF32.2430	*L. major*	47	77	123
LmjF32.2440	*L. major*	42	1	42
LmjF32.2440	*L. major*	71	346	416
LmjF32.2460	*L. major*	145	125	269
LmjF32.2460	*L. major*	594	310	903
LmjF32.2480	*L. major*	55	1	55
LmjF32.2490	*L. major*	144	39	182
LmjF32.2490	*L. major*	40	404	443
LmjF32.2490	*L. major*	41	570	610
LmjF32.2490	*L. major*	84	636	719
LmjF32.2510	*L. major*	104	60	163
LmjF32.2510	*L. major*	54	454	507
LmjF32.2520	*L. major*	872	55	926
LmjF32.2530	*L. major*	131	179	309
LmjF32.2530	*L. major*	84	586	669
LmjF32.2530	*L. major*	75	884	958
LmjF32.2540	*L. major*	145	529	673
LmjF32.2550	*L. major*	50	25	74
LmjF32.2550	*L. major*	50	128	177
LmjF32.2550	*L. major*	69	1392	1460
LmjF32.2550	*L. major*	46	1493	1538
LmjF32.2550	*L. major*	53	1558	1610
LmjF32.2570	*L. major*	289	1	289
LmjF32.2570	*L. major*	67	311	377
LmjF32.2570	*L. major*	234	391	624
LmjF32.2570	*L. major*	50	626	675
LmjF32.2590	*L. major*	45	1	45
LmjF32.2600	*L. major*	129	249	377
LmjF32.2600	*L. major*	203	438	640
LmjF32.2610	*L. major*	462	1	462
LmjF32.2610	*L. major*	373	480	852
LmjF32.2650	*L. major*	71	228	298
LmjF32.2650	*L. major*	40	1819	1858

LmjF32.2660	*L. major*	71	1	71
LmjF32.2670	*L. major*	131	1	131
LmjF32.2670	*L. major*	112	188	299
LmjF32.2670	*L. major*	59	419	477
LmjF32.2670	*L. major*	64	517	580
LmjF32.2670	*L. major*	70	600	669
LmjF32.2670	*L. major*	50	741	790
LmjF32.2700	*L. major*	72	213	284
LmjF32.2700	*L. major*	67	587	653
LmjF32.2700	*L. major*	72	1057	1128
LmjF32.2730	*L. major*	119	269	387
LmjF32.2730	*L. major*	44	448	491
LmjF32.2730	*L. major*	53	612	664
LmjF32.2750	*L. major*	200	1	200
LmjF32.2750	*L. major*	165	471	635
LmjF32.2750	*L. major*	77	891	967
LmjF32.2750	*L. major*	147	1220	1366
LmjF32.2760	*L. major*	313	1	313
LmjF32.2760	*L. major*	83	381	463
LmjF32.2760	*L. major*	235	481	715
LmjF32.2760	*L. major*	127	719	845
LmjF32.2770	*L. major*	52	1	52
LmjF32.2770	*L. major*	808	153	960
LmjF32.2780	*L. major*	40	314	353
LmjF32.2780	*L. major*	91	465	555
LmjF32.2780	*L. major*	117	978	1094
LmjF32.2800	*L. major*	225	1	225
LmjF32.2800	*L. major*	74	409	482
LmjF32.2810	*L. major*	126	1	126
LmjF32.2820	*L. major*	73	1	73
LmjF32.2830	*L. major*	337	1	337
LmjF32.2830	*L. major*	260	378	637
LmjF32.2830	*L. major*	240	699	938
LmjF32.2830	*L. major*	113	981	1093
LmjF32.2840	*L. major*	389	110	498
LmjF32.2850	*L. major*	184	95	278
LmjF32.2850	*L. major*	59	500	558
LmjF32.2860	*L. major*	96	118	213
LmjF32.2860	*L. major*	87	218	304
LmjF32.2860	*L. major*	156	317	472
LmjF32.2870	*L. major*	46	185	230
LmjF32.2880	*L. major*	135	1	135
LmjF32.2880	*L. major*	228	202	429
LmjF32.2880	*L. major*	63	472	534
LmjF32.2885	*L. major*	121	132	252
LmjF32.2890	*L. major*	57	39	95
LmjF32.2900	*L. major*	155	161	315

LmjF32.2900	*L. major*	47	355	401
LmjF32.2900	*L. major*	150	469	618
LmjF32.2910	*L. major*	72	1	72
LmjF32.2910	*L. major*	44	221	264
LmjF32.2910	*L. major*	50	864	913
LmjF32.2910	*L. major*	74	1061	1134
LmjF32.2920	*L. major*	41	549	589
LmjF32.2930	*L. major*	42	68	109
LmjF32.2930	*L. major*	52	329	380
LmjF32.2930	*L. major*	52	329	380
LmjF32.2960	*L. major*	43	622	664
LmjF32.2970	*L. major*	109	1	109
LmjF32.2980	*L. major*	55	1	55
LmjF32.2990	*L. major*	58	681	738
LmjF32.3010	*L. major*	73	803	875
LmjF32.3020	*L. major*	67	366	432
LmjF32.3020	*L. major*	99	502	600
LmjF32.3020	*L. major*	47	671	717
LmjF32.3020	*L. major*	45	743	787
LmjF32.3030	*L. major*	198	72	269
LmjF32.3060	*L. major*	100	30	129
LmjF32.3060	*L. major*	55	189	243
LmjF32.3060	*L. major*	156	406	561
LmjF32.3060	*L. major*	56	629	684
LmjF32.3060	*L. major*	52	733	784
LmjF32.3060	*L. major*	239	860	1098
LmjF32.3070	*L. major*	94	160	253
LmjF32.3080	*L. major*	72	1	72
LmjF32.3090	*L. major*	273	1	273
LmjF32.3090	*L. major*	415	281	695
LmjF32.3090	*L. major*	56	728	783
LmjF32.3090	*L. major*	45	813	857
LmjF32.3090	*L. major*	132	911	1042
LmjF32.3090	*L. major*	257	1256	1512
LmjF32.3100	*L. major*	110	1	110
LmjF32.3100	*L. major*	105	150	254
LmjF32.3110	*L. major*	71	395	465
LmjF32.3110	*L. major*	44	907	950
LmjF32.3110	*L. major*	44	907	950
LmjF32.3130	*L. major*	42	1	42
LmjF32.3130	*L. major*	87	333	419
LmjF32.3130	*L. major*	87	333	419
LmjF32.3140	*L. major*	76	1	76
LmjF32.3150	*L. major*	334	310	643
LmjF32.3150	*L. major*	48	700	747
LmjF32.3150	*L. major*	55	996	1050
LmjF32.3150	*L. major*	428	1063	1490

LmjF32.3150	*L. major*	53	1530	1582
LmjF32.3150	*L. major*	339	1645	1983
LmjF32.3150	*L. major*	43	2054	2096
LmjF32.3150	*L. major*	79	2106	2184
LmjF32.3150	*L. major*	101	2199	2299
LmjF32.3150	*L. major*	63	2375	2437
LmjF32.3160	*L. major*	71	1	71
LmjF32.3160	*L. major*	63	204	266
LmjF32.3160	*L. major*	101	498	598
LmjF32.3160	*L. major*	124	648	771
LmjF32.3180	*L. major*	41	255	295
LmjF32.3180	*L. major*	51	623	673
LmjF32.3180	*L. major*	51	623	673
LmjF32.3190	*L. major*	48	69	116
LmjF32.3190	*L. major*	49	242	290
LmjF32.3190	*L. major*	47	452	498
LmjF32.3190	*L. major*	43	736	778
LmjF32.3190	*L. major*	52	955	1006
LmjF32.3200	*L. major*	74	1	74
LmjF32.3200	*L. major*	80	136	215
LmjF32.3200	*L. major*	108	247	354
LmjF32.3200	*L. major*	142	446	587
LmjF32.3200	*L. major*	40	705	744
LmjF32.3200	*L. major*	49	830	878
LmjF32.3200	*L. major*	74	1057	1130
LmjF32.3200	*L. major*	53	1265	1317
LmjF32.3220	*L. major*	220	1	220
LmjF32.3220	*L. major*	82	248	329
LmjF32.3220	*L. major*	80	407	486
LmjF32.3220	*L. major*	45	527	571
LmjF32.3230	*L. major*	66	1	66
LmjF32.3230	*L. major*	43	333	375
LmjF32.3230	*L. major*	40	386	425
LmjF32.3240	*L. major*	72	197	268
LmjF32.3240	*L. major*	64	425	488
LmjF32.3240	*L. major*	99	492	590
LmjF32.3250	*L. major*	137	289	425
LmjF32.3250	*L. major*	674	433	1106
LmjF32.3280	*L. major*	61	30	90
LmjF32.3280	*L. major*	120	193	312
LmjF32.3280	*L. major*	103	368	470
LmjF32.3280	*L. major*	247	505	751
LmjF32.3280	*L. major*	112	753	864
LmjF32.3290	*L. major*	80	161	240
LmjF32.3300	*L. major*	104	62	165
LmjF32.3300	*L. major*	45	250	294
LmjF32.3300	*L. major*	45	250	294

LmjF32.3330	*L. major*	51	64	114
LmjF32.3340	*L. major*	45	140	184
LmjF32.3340	*L. major*	51	857	907
LmjF32.3340	*L. major*	45	1068	1112
LmjF32.3350	*L. major*	66	235	300
LmjF32.3350	*L. major*	51	315	365
LmjF32.3350	*L. major*	365	397	761
LmjF32.3350	*L. major*	63	825	887
LmjF32.3350	*L. major*	95	1000	1094
LmjF32.3350	*L. major*	49	1150	1198
LmjF32.3360	*L. major*	47	369	415
LmjF32.3380	*L. major*	75	75	149
LmjF32.3380	*L. major*	51	173	223
LmjF32.3380	*L. major*	68	528	595
LmjF32.3390	*L. major*	67	1	67
LmjF32.3390	*L. major*	50	551	600
LmjF32.3390	*L. major*	50	551	600
LmjF32.3410	*L. major*	136	1	136
LmjF32.3410	*L. major*	60	1296	1355
LmjF32.3410	*L. major*	117	1414	1530
LmjF32.3410	*L. major*	72	1627	1698
LmjF32.3410	*L. major*	43	2072	2114
LmjF32.3430	*L. major*	79	1	79
LmjF32.3430	*L. major*	76	114	189
LmjF32.3430	*L. major*	386	254	639
LmjF32.3430	*L. major*	141	667	807
LmjF32.3440	*L. major*	83	181	263
LmjF32.3450	*L. major*	314	143	456
LmjF32.3450	*L. major*	78	481	558
LmjF32.3470	*L. major*	79	119	197
LmjF32.3470	*L. major*	102	351	452
LmjF32.3480	*L. major*	43	1	43
LmjF32.3480	*L. major*	96	79	174
LmjF32.3480	*L. major*	48	321	368
LmjF32.3490	*L. major*	74	1	74
LmjF32.3490	*L. major*	100	490	589
LmjF32.3490	*L. major*	100	490	589
LmjF32.3500	*L. major*	84	39	122
LmjF32.3500	*L. major*	81	424	504
LmjF32.3500	*L. major*	81	424	504
LmjF32.3502	*L. major*	84	39	122
LmjF32.3502	*L. major*	81	424	504
LmjF32.3502	*L. major*	81	424	504
LmjF32.3504	*L. major*	84	39	122
LmjF32.3504	*L. major*	81	424	504
LmjF32.3504	*L. major*	81	424	504
LmjF32.3506	*L. major*	84	39	122

LmjF32.3506	*L. major*	81	424	504
LmjF32.3506	*L. major*	81	424	504
LmjF32.3508	*L. major*	84	39	122
LmjF32.3508	*L. major*	81	424	504
LmjF32.3508	*L. major*	81	424	504
LmjF32.3510	*L. major*	84	39	122
LmjF32.3510	*L. major*	81	424	504
LmjF32.3510	*L. major*	81	424	504
LmjF32.3512	*L. major*	84	39	122
LmjF32.3512	*L. major*	81	424	504
LmjF32.3512	*L. major*	81	424	504
LmjF32.3515	*L. major*	84	39	122
LmjF32.3515	*L. major*	81	424	504
LmjF32.3515	*L. major*	81	424	504
LmjF32.3520	*L. major*	84	39	122
LmjF32.3520	*L. major*	81	424	504
LmjF32.3520	*L. major*	81	424	504
LmjF32.3530	*L. major*	84	39	122
LmjF32.3530	*L. major*	81	424	504
LmjF32.3530	*L. major*	81	424	504
LmjF32.3535	*L. major*	84	39	122
LmjF32.3535	*L. major*	81	424	504
LmjF32.3535	*L. major*	81	424	504
LmjF32.3538	*L. major*	84	39	122
LmjF32.3538	*L. major*	81	424	504
LmjF32.3538	*L. major*	81	424	504
LmjF32.3540	*L. major*	84	39	122
LmjF32.3540	*L. major*	81	424	504
LmjF32.3540	*L. major*	81	424	504
LmjF32.3542	*L. major*	84	39	122
LmjF32.3542	*L. major*	81	424	504
LmjF32.3542	*L. major*	81	424	504
LmjF32.3544	*L. major*	84	39	122
LmjF32.3544	*L. major*	81	424	504
LmjF32.3544	*L. major*	81	424	504
LmjF32.3546	*L. major*	84	39	122
LmjF32.3546	*L. major*	81	424	504
LmjF32.3546	*L. major*	81	424	504
LmjF32.3548	*L. major*	81	373	453
LmjF32.3550	*L. major*	70	63	132
LmjF32.3550	*L. major*	60	234	293
LmjF32.3550	*L. major*	131	314	444
LmjF32.3555	*L. major*	41	178	218
LmjF32.3560	*L. major*	60	42	101
LmjF32.3560	*L. major*	93	117	209
LmjF32.3560	*L. major*	104	429	532
LmjF32.3570	*L. major*	151	1	151

LmjF32.3570	*L. major*	123	262	384
LmjF32.3570	*L. major*	67	691	757
LmjF32.3570	*L. major*	65	1133	1197
LmjF32.3600	*L. major*	76	1	76
LmjF32.3600	*L. major*	48	142	189
LmjF32.3600	*L. major*	48	142	189
LmjF32.3620	*L. major*	106	1	106
LmjF32.3630	*L. major*	202	1	202
LmjF32.3630	*L. major*	53	265	317
LmjF32.3640	*L. major*	74	364	437
LmjF32.3690	*L. major*	843	1	843
LmjF32.3690	*L. major*	87	848	934
LmjF32.3700	*L. major*	52	35	86
LmjF32.3700	*L. major*	65	288	352
LmjF32.3700	*L. major*	88	550	637
LmjF32.3700	*L. major*	67	661	727
LmjF32.3710	*L. major*	48	1	48
LmjF32.3710	*L. major*	44	92	135
LmjF32.3750	*L. major*	56	53	108
LmjF32.3760	*L. major*	44	273	316
LmjF32.3760	*L. major*	55	554	608
LmjF32.3760	*L. major*	55	554	608
LmjF32.3770	*L. major*	94	39	132
LmjF32.3780	*L. major*	42	23	64
LmjF32.3780	*L. major*	44	93	136
LmjF32.3780	*L. major*	44	93	136
LmjF32.3800	*L. major*	57	29	85
LmjF32.3810	*L. major*	43	111	153
LmjF32.3810	*L. major*	81	306	386
LmjF32.3810	*L. major*	81	306	386
LmjF32.3850	*L. major*	108	1	108
LmjF32.3850	*L. major*	52	125	176
LmjF32.3850	*L. major*	49	223	271
LmjF32.3860	*L. major*	41	577	617
LmjF32.3860	*L. major*	43	716	758
LmjF32.3860	*L. major*	71	792	862
LmjF32.3870	*L. major*	43	593	635
LmjF32.3870	*L. major*	47	762	808
LmjF32.3870	*L. major*	47	762	808
LmjF32.3890	*L. major*	55	334	388
LmjF32.3900	*L. major*	63	198	260
LmjF32.3920	*L. major*	87	680	766
LmjF32.3930	*L. major*	63	1	63
LmjF32.3930	*L. major*	51	99	149
LmjF32.3930	*L. major*	50	1263	1312
LmjF32.3950	*L. major*	94	815	908
LmjF32.3960	*L. major*	48	138	185

LmjF32.3970	*L. major*	48	91	138
LmjF32.3980	*L. major*	56	124	179
LmjF32.3990	*L. major*	112	45	156
LmjF33.0010	*L. major*	41	144	184
LmjF33.0010	*L. major*	64	487	550
LmjF33.0010	*L. major*	43	690	732
LmjF33.0010	*L. major*	55	768	822
LmjF33.0040	*L. major*	131	247	377
LmjF33.0050	*L. major*	73	143	215
LmjF33.0060	*L. major*	94	1	94
LmjF33.0070	*L. major*	83	176	258
LmjF33.0070	*L. major*	81	342	422
LmjF33.0080	*L. major*	82	102	183
LmjF33.0090	*L. major*	42	515	556
LmjF33.0110	*L. major*	65	1	65
LmjF33.0110	*L. major*	52	113	164
LmjF33.0110	*L. major*	55	298	352
LmjF33.0115	*L. major*	118	1	118
LmjF33.0115	*L. major*	75	209	283
LmjF33.0120	*L. major*	97	219	315
LmjF33.0140	*L. major*	44	365	408
LmjF33.0150	*L. major*	44	16	59
LmjF33.0150	*L. major*	78	202	279
LmjF33.0160	*L. major*	44	167	210
LmjF33.0160	*L. major*	40	299	338
LmjF33.0180	*L. major*	43	120	162
LmjF33.0190	*L. major*	46	442	487
LmjF33.0200	*L. major*	51	1	51
LmjF33.0200	*L. major*	72	482	553
LmjF33.0200	*L. major*	82	807	888
LmjF33.0200	*L. major*	63	1109	1171
LmjF33.0200	*L. major*	94	1263	1356
LmjF33.0200	*L. major*	122	1363	1484
LmjF33.0210	*L. major*	206	79	284
LmjF33.0210	*L. major*	117	465	581
LmjF33.0210	*L. major*	40	786	825
LmjF33.0210	*L. major*	44	870	913
LmjF33.0220	*L. major*	118	1	118
LmjF33.0230	*L. major*	76	143	218
LmjF33.0230	*L. major*	63	496	558
LmjF33.0230	*L. major*	63	496	558
LmjF33.0250	*L. major*	73	30	102
LmjF33.0260	*L. major*	198	19	216
LmjF33.0260	*L. major*	54	298	351
LmjF33.0270	*L. major*	43	69	111
LmjF33.0280	*L. major*	42	165	206
LmjF33.0280	*L. major*	54	299	352

LmjF33.0280	*L. major*	54	299	352
LmjF33.0290	*L. major*	67	1	67
LmjF33.0295	*L. major*	63	77	139
LmjF33.0310	*L. major*	59	1	59
LmjF33.0310	*L. major*	60	131	190
LmjF33.0310	*L. major*	74	594	667
LmjF33.0312	*L. major*	102	176	277
LmjF33.0312	*L. major*	53	505	557
LmjF33.0314	*L. major*	102	176	277
LmjF33.0314	*L. major*	53	505	557
LmjF33.0316	*L. major*	102	176	277
LmjF33.0316	*L. major*	53	505	557
LmjF33.0318	*L. major*	102	176	277
LmjF33.0318	*L. major*	53	505	557
LmjF33.0320	*L. major*	102	176	277
LmjF33.0320	*L. major*	53	505	557
LmjF33.0323	*L. major*	102	176	277
LmjF33.0323	*L. major*	53	505	557
LmjF33.0326	*L. major*	102	176	277
LmjF33.0326	*L. major*	53	505	557
LmjF33.0330	*L. major*	102	176	277
LmjF33.0330	*L. major*	53	505	557
LmjF33.0333	*L. major*	102	176	277
LmjF33.0333	*L. major*	53	505	557
LmjF33.0336	*L. major*	102	176	277
LmjF33.0336	*L. major*	53	505	557
LmjF33.0340	*L. major*	102	176	277
LmjF33.0340	*L. major*	53	505	557
LmjF33.0343	*L. major*	102	176	277
LmjF33.0343	*L. major*	53	505	557
LmjF33.0346	*L. major*	102	176	277
LmjF33.0346	*L. major*	53	505	557
LmjF33.0350	*L. major*	102	176	277
LmjF33.0350	*L. major*	53	505	557
LmjF33.0355	*L. major*	102	176	277
LmjF33.0355	*L. major*	53	505	557
LmjF33.0360	*L. major*	102	176	277
LmjF33.0360	*L. major*	53	505	557
LmjF33.0365	*L. major*	102	176	277
LmjF33.0365	*L. major*	53	505	557
LmjF33.0370	*L. major*	191	1	191
LmjF33.0370	*L. major*	153	203	355
LmjF33.0390	*L. major*	186	67	252
LmjF33.0400	*L. major*	44	1	44
LmjF33.0410	*L. major*	46	529	574
LmjF33.0430	*L. major*	57	32	88
LmjF33.0430	*L. major*	58	196	253

LmjF33.0430	*L. major*	48	414	461
LmjF33.0430	*L. major*	69	561	629
LmjF33.0430	*L. major*	68	676	743
LmjF33.0440	*L. major*	43	108	150
LmjF33.0450	*L. major*	69	87	155
LmjF33.0460	*L. major*	125	96	220
LmjF33.0480	*L. major*	218	30	247
LmjF33.0480	*L. major*	136	275	410
LmjF33.0480	*L. major*	47	480	526
LmjF33.0480	*L. major*	72	565	636
LmjF33.0490	*L. major*	119	42	160
LmjF33.0490	*L. major*	109	165	273
LmjF33.0490	*L. major*	129	580	708
LmjF33.0490	*L. major*	57	716	772
LmjF33.0490	*L. major*	61	808	868
LmjF33.0510	*L. major*	72	286	357
LmjF33.0510	*L. major*	52	395	446
LmjF33.0510	*L. major*	77	615	691
LmjF33.0530	*L. major*	163	1	163
LmjF33.0530	*L. major*	69	219	287
LmjF33.0530	*L. major*	134	451	584
LmjF33.0530	*L. major*	83	650	732
LmjF33.0540	*L. major*	75	134	208
LmjF33.0540	*L. major*	93	334	426
LmjF33.0540	*L. major*	93	334	426
LmjF33.0550	*L. major*	77	58	134
LmjF33.0550	*L. major*	43	403	445
LmjF33.0550	*L. major*	52	1312	1363
LmjF33.0550	*L. major*	40	1405	1444
LmjF33.0550	*L. major*	40	1455	1494
LmjF33.0550	*L. major*	85	1876	1960
LmjF33.0565	*L. major*	66	79	144
LmjF33.0570	*L. major*	52	88	139
LmjF33.0570	*L. major*	58	525	582
LmjF33.0580	*L. major*	291	30	320
LmjF33.0580	*L. major*	59	322	380
LmjF33.0585	*L. major*	78	25	102
LmjF33.0585	*L. major*	40	123	162
LmjF33.0585	*L. major*	101	191	291
LmjF33.0585	*L. major*	314	304	617
LmjF33.0590	*L. major*	67	1	67
LmjF33.0590	*L. major*	189	76	264
LmjF33.0590	*L. major*	162	313	474
LmjF33.0590	*L. major*	94	552	645
LmjF33.0590	*L. major*	188	657	844
LmjF33.0620	*L. major*	63	57	119
LmjF33.0620	*L. major*	86	153	238

LmjF33.0620	*L. major*	86	153	238
LmjF33.0630	*L. major*	93	31	123
LmjF33.0650	*L. major*	243	1	243
LmjF33.0650	*L. major*	77	379	455
LmjF33.0650	*L. major*	569	460	1028
LmjF33.0650	*L. major*	80	1044	1123
LmjF33.0650	*L. major*	277	1125	1401
LmjF33.0670	*L. major*	223	104	326
LmjF33.0670	*L. major*	48	336	383
LmjF33.0670	*L. major*	47	418	464
LmjF33.0670	*L. major*	48	812	859
LmjF33.0700	*L. major*	73	189	261
LmjF33.0710	*L. major*	46	162	207
LmjF33.0710	*L. major*	41	362	402
LmjF33.0710	*L. major*	41	362	402
LmjF33.0720	*L. major*	70	93	162
LmjF33.0730	*L. major*	128	1	128
LmjF33.0740	*L. major*	75	180	254
LmjF33.0740	*L. major*	53	272	324
LmjF33.0740	*L. major*	53	272	324
LmjF33.0750	*L. major*	44	28	71
LmjF33.0750	*L. major*	165	147	311
LmjF33.0750	*L. major*	51	491	541
LmjF33.0750	*L. major*	91	657	747
LmjF33.0760	*L. major*	141	1	141
LmjF33.0760	*L. major*	80	152	231
LmjF33.0760	*L. major*	76	252	327
LmjF33.0765	*L. major*	420	1	420
LmjF33.0770	*L. major*	52	76	127
LmjF33.0770	*L. major*	46	136	181
LmjF33.0770	*L. major*	299	322	620
LmjF33.0770	*L. major*	254	629	882
LmjF33.0780	*L. major*	677	1	677
LmjF33.0780	*L. major*	86	731	816
LmjF33.0780	*L. major*	135	932	1066
LmjF33.0780	*L. major*	173	1098	1270
LmjF33.0790	*L. major*	99	216	314
LmjF33.0790	*L. major*	45	465	509
LmjF33.0790	*L. major*	71	682	752
LmjF33.0790	*L. major*	125	1049	1173
LmjF33.0790	*L. major*	45	1191	1235
LmjF33.0790	*L. major*	62	1520	1581
LmjF33.0792	*L. major*	40	404	443
LmjF33.0794	*L. major*	40	404	443
LmjF33.0796	*L. major*	40	404	443
LmjF33.0798	*L. major*	40	404	443
LmjF33.0800	*L. major*	40	404	443

LmjF33.0802	*L. major*	40	404	443
LmjF33.0804	*L. major*	40	404	443
LmjF33.0806	*L. major*	40	404	443
LmjF33.0808	*L. major*	40	404	443
LmjF33.0810	*L. major*	40	404	443
LmjF33.0812	*L. major*	40	404	443
LmjF33.0814	*L. major*	40	404	443
LmjF33.0816	*L. major*	40	404	443
LmjF33.0818	*L. major*	40	404	443
LmjF33.0819	*L. major*	40	404	443
LmjF33.0820	*L. major*	40	404	443
LmjF33.0840	*L. major*	205	1	205
LmjF33.0840	*L. major*	70	239	308
LmjF33.0840	*L. major*	197	513	709
LmjF33.0850	*L. major*	59	69	127
LmjF33.0850	*L. major*	60	314	373
LmjF33.0850	*L. major*	156	419	574
LmjF33.0850	*L. major*	88	713	800
LmjF33.0850	*L. major*	48	829	876
LmjF33.0860	*L. major*	56	115	170
LmjF33.0860	*L. major*	54	398	451
LmjF33.0860	*L. major*	54	398	451
LmjF33.0870	*L. major*	197	1	197
LmjF33.0870	*L. major*	72	273	344
LmjF33.0880	*L. major*	47	50	96
LmjF33.0880	*L. major*	60	115	174
LmjF33.0880	*L. major*	60	115	174
LmjF33.0900	*L. major*	93	316	408
LmjF33.0900	*L. major*	86	495	580
LmjF33.0930	*L. major*	57	1	57
LmjF33.0930	*L. major*	50	122	171
LmjF33.0930	*L. major*	64	194	257
LmjF33.0930	*L. major*	240	290	529
LmjF33.0930	*L. major*	81	701	781
LmjF33.0930	*L. major*	81	921	1001
LmjF33.0935	*L. major*	108	1	108
LmjF33.0935	*L. major*	47	165	211
LmjF33.0935	*L. major*	336	213	548
LmjF33.0940	*L. major*	304	36	339
LmjF33.0940	*L. major*	42	448	489
LmjF33.0940	*L. major*	101	666	766
LmjF33.0940	*L. major*	163	827	989
LmjF33.0940	*L. major*	84	1020	1103
LmjF33.0950	*L. major*	117	1	117
LmjF33.0950	*L. major*	124	296	419
LmjF33.0950	*L. major*	53	787	839
LmjF33.0950	*L. major*	54	984	1037

LmjF33.0950	*L. major*	41	1227	1267
LmjF33.0950	*L. major*	201	1365	1565
LmjF33.0970	*L. major*	75	120	194
LmjF33.0970	*L. major*	183	371	553
LmjF33.1000	*L. major*	167	187	353
LmjF33.1000	*L. major*	66	397	462
LmjF33.1000	*L. major*	50	487	536
LmjF33.1000	*L. major*	51	582	632
LmjF33.1000	*L. major*	244	684	927
LmjF33.1010	*L. major*	50	441	490
LmjF33.1010	*L. major*	43	531	573
LmjF33.1010	*L. major*	49	727	775
LmjF33.1020	*L. major*	464	1	464
LmjF33.1020	*L. major*	128	473	600
LmjF33.1020	*L. major*	72	745	816
LmjF33.1030	*L. major*	46	1	46
LmjF33.1035	*L. major*	123	1	123
LmjF33.1040	*L. major*	41	17	57
LmjF33.1040	*L. major*	130	86	215
LmjF33.1040	*L. major*	57	394	450
LmjF33.1050	*L. major*	49	90	138
LmjF33.1060	*L. major*	46	404	449
LmjF33.1060	*L. major*	83	875	957
LmjF33.1080	*L. major*	58	24	81
LmjF33.1080	*L. major*	64	92	155
LmjF33.1080	*L. major*	41	324	364
LmjF33.1080	*L. major*	48	398	445
LmjF33.1100	*L. major*	46	1	46
LmjF33.1100	*L. major*	105	111	215
LmjF33.1100	*L. major*	148	256	403
LmjF33.1100	*L. major*	89	506	594
LmjF33.1100	*L. major*	264	641	904
LmjF33.1110	*L. major*	62	96	157
LmjF33.1110	*L. major*	150	304	453
LmjF33.1110	*L. major*	272	487	758
LmjF33.1120	*L. major*	235	1	235
LmjF33.1120	*L. major*	69	265	333
LmjF33.1130	*L. major*	53	562	614
LmjF33.1150	*L. major*	73	1	73
LmjF33.1150	*L. major*	92	78	169
LmjF33.1150	*L. major*	78	185	262
LmjF33.1150	*L. major*	111	278	388
LmjF33.1150	*L. major*	56	795	850
LmjF33.1160	*L. major*	49	1	49
LmjF33.1170	*L. major*	186	39	224
LmjF33.1170	*L. major*	60	239	298
LmjF33.1170	*L. major*	121	339	459

LmjF33.1170	*L. major*	51	498	548
LmjF33.1170	*L. major*	62	800	861
LmjF33.1170	*L. major*	89	1170	1258
LmjF33.1180	*L. major*	48	23	70
LmjF33.1180	*L. major*	105	143	247
LmjF33.1180	*L. major*	105	143	247
LmjF33.1190	*L. major*	62	466	527
LmjF33.1190	*L. major*	41	537	577
LmjF33.1200	*L. major*	42	143	184
LmjF33.1200	*L. major*	90	418	507
LmjF33.1210	*L. major*	94	193	286
LmjF33.1220	*L. major*	58	1	58
LmjF33.1220	*L. major*	209	253	461
LmjF33.1220	*L. major*	94	484	577
LmjF33.1220	*L. major*	68	624	691
LmjF33.1230	*L. major*	221	401	621
LmjF33.1240	*L. major*	50	65	114
LmjF33.1240	*L. major*	100	385	484
LmjF33.1240	*L. major*	225	532	756
LmjF33.1240	*L. major*	75	782	856
LmjF33.1245	*L. major*	266	1	266
LmjF33.1245	*L. major*	59	306	364
LmjF33.1260	*L. major*	56	78	133
LmjF33.1260	*L. major*	156	214	369
LmjF33.1260	*L. major*	175	377	551
LmjF33.1260	*L. major*	409	570	978
LmjF33.1270	*L. major*	55	250	304
LmjF33.1280	*L. major*	48	15	62
LmjF33.1290	*L. major*	79	513	591
LmjF33.1305	*L. major*	163	1	163
LmjF33.1320	*L. major*	93	1	93
LmjF33.1340	*L. major*	67	1	67
LmjF33.1340	*L. major*	76	139	214
LmjF33.1340	*L. major*	76	139	214
LmjF33.1355	*L. major*	59	104	162
LmjF33.1360	*L. major*	44	79	122
LmjF33.1360	*L. major*	79	171	249
LmjF33.1360	*L. major*	52	387	438
LmjF33.1370	*L. major*	342	84	425
LmjF33.1380	*L. major*	40	244	283
LmjF33.1380	*L. major*	62	347	408
LmjF33.1390	*L. major*	113	48	160
LmjF33.1400	*L. major*	101	91	191
LmjF33.1400	*L. major*	101	230	330
LmjF33.1400	*L. major*	233	359	591
LmjF33.1400	*L. major*	91	715	805
LmjF33.1400	*L. major*	119	911	1029

LmjF33.1400	*L. major*	81	1092	1172
LmjF33.1400	*L. major*	201	1190	1390
LmjF33.1400	*L. major*	306	1406	1711
LmjF33.1400	*L. major*	228	1727	1954
LmjF33.1410	*L. major*	137	55	191
LmjF33.1410	*L. major*	82	199	280
LmjF33.1430	*L. major*	40	52	91
LmjF33.1430	*L. major*	70	114	183
LmjF33.1430	*L. major*	47	209	255
LmjF33.1430	*L. major*	81	337	417
LmjF33.1440	*L. major*	49	1	49
LmjF33.1440	*L. major*	214	52	265
LmjF33.1440	*L. major*	54	283	336
LmjF33.1440	*L. major*	73	375	447
LmjF33.1440	*L. major*	82	455	536
LmjF33.1460	*L. major*	117	93	209
LmjF33.1460	*L. major*	104	224	327
LmjF33.1460	*L. major*	104	224	327
LmjF33.1490	*L. major*	109	1	109
LmjF33.1490	*L. major*	103	137	239
LmjF33.1490	*L. major*	102	241	342
LmjF33.1490	*L. major*	111	406	516
LmjF33.1490	*L. major*	67	583	649
LmjF33.1490	*L. major*	115	718	832
LmjF33.1490	*L. major*	45	885	929
LmjF33.1490	*L. major*	197	959	1155
LmjF33.1490	*L. major*	88	1168	1255
LmjF33.1490	*L. major*	69	1499	1567
LmjF33.1490	*L. major*	232	1781	2012
LmjF33.1490	*L. major*	48	2066	2113
LmjF33.1490	*L. major*	73	2242	2314
LmjF33.1490	*L. major*	329	2492	2820
LmjF33.1490	*L. major*	66	2919	2984
LmjF33.1490	*L. major*	127	3092	3218
LmjF33.1490	*L. major*	64	3277	3340
LmjF33.1500	*L. major*	43	47	89
LmjF33.1500	*L. major*	46	97	142
LmjF33.1500	*L. major*	51	607	657
LmjF33.1500	*L. major*	50	706	755
LmjF33.1510	*L. major*	44	22	65
LmjF33.1510	*L. major*	130	160	289
LmjF33.1510	*L. major*	46	554	599
LmjF33.1550	*L. major*	51	129	179
LmjF33.1550	*L. major*	67	219	285
LmjF33.1550	*L. major*	67	219	285
LmjF33.1560	*L. major*	48	272	319
LmjF33.1560	*L. major*	209	462	670

LmjF33.1575	*L. major*	65	1	65
LmjF33.1575	*L. major*	43	273	315
LmjF33.1575	*L. major*	97	415	511
LmjF33.1600	*L. major*	78	16	93
LmjF33.1600	*L. major*	54	98	151
LmjF33.1600	*L. major*	56	222	277
LmjF33.1600	*L. major*	58	307	364
LmjF33.1600	*L. major*	45	720	764
LmjF33.1660	*L. major*	68	137	204
LmjF33.1700	*L. major*	42	110	151
LmjF33.1700	*L. major*	40	802	841
LmjF33.1700	*L. major*	51	866	916
LmjF33.1710	*L. major*	47	301	347
LmjF33.1730	*L. major*	67	827	893
LmjF33.1780	*L. major*	101	1	101
LmjF33.1780	*L. major*	117	290	406
LmjF33.1780	*L. major*	53	1099	1151
LmjF33.1780	*L. major*	104	1528	1631
LmjF33.1820	*L. major*	56	375	430
LmjF33.1830	*L. major*	58	298	355
LmjF33.1830	*L. major*	379	388	766
LmjF33.1830	*L. major*	175	783	957
LmjF33.1850	*L. major*	68	62	129
LmjF33.1870	*L. major*	59	365	423
LmjF33.1870	*L. major*	152	501	652
LmjF33.1890	*L. major*	72	192	263
LmjF33.1890	*L. major*	44	313	356
LmjF33.1890	*L. major*	124	358	481
LmjF33.1900	*L. major*	65	1	65
LmjF33.1900	*L. major*	73	77	149
LmjF33.1900	*L. major*	44	161	204
LmjF33.1910	*L. major*	122	1	122
LmjF33.1910	*L. major*	40	216	255
LmjF33.1920	*L. major*	57	133	189
LmjF33.1940	*L. major*	59	1	59
LmjF33.1940	*L. major*	49	64	112
LmjF33.1940	*L. major*	49	64	112
LmjF33.1950	*L. major*	54	10	63
LmjF33.1950	*L. major*	70	228	297
LmjF33.1950	*L. major*	125	552	676
LmjF33.1960	*L. major*	111	20	130
LmjF33.1960	*L. major*	219	166	384
LmjF33.1960	*L. major*	49	416	464
LmjF33.1970	*L. major*	74	199	272
LmjF33.1970	*L. major*	155	341	495
LmjF33.1990	*L. major*	108	152	259
LmjF33.1990	*L. major*	65	291	355

LmjF33.1990	*L. major*	126	412	537
LmjF33.2010	*L. major*	172	62	233
LmjF33.2010	*L. major*	46	1071	1116
LmjF33.2020	*L. major*	139	100	238
LmjF33.2020	*L. major*	337	253	589
LmjF33.2020	*L. major*	92	786	877
LmjF33.2030	*L. major*	106	241	346
LmjF33.2030	*L. major*	49	746	794
LmjF33.2030	*L. major*	49	746	794
LmjF33.2070	*L. major*	363	414	776
LmjF33.2070	*L. major*	70	780	849
LmjF33.2070	*L. major*	107	866	972
LmjF33.2080	*L. major*	70	93	162
LmjF33.2080	*L. major*	106	292	397
LmjF33.2080	*L. major*	98	414	511
LmjF33.2080	*L. major*	76	553	628
LmjF33.2080	*L. major*	100	669	768
LmjF33.2080	*L. major*	46	1000	1045
LmjF33.2080	*L. major*	66	1069	1134
LmjF33.2090	*L. major*	167	80	246
LmjF33.2090	*L. major*	65	865	929
LmjF33.2090	*L. major*	65	865	929
LmjF33.2100	*L. major*	77	204	280
LmjF33.2100	*L. major*	109	384	492
LmjF33.2110	*L. major*	177	1	177
LmjF33.2110	*L. major*	62	273	334
LmjF33.2120	*L. major*	146	1	146
LmjF33.2130	*L. major*	91	1	91
LmjF33.2130	*L. major*	178	95	272
LmjF33.2130	*L. major*	200	382	581
LmjF33.2130	*L. major*	160	692	851
LmjF33.2130	*L. major*	66	855	920
LmjF33.2140	*L. major*	49	400	448
LmjF33.2140	*L. major*	422	535	956
LmjF33.2150	*L. major*	207	105	311
LmjF33.2150	*L. major*	58	472	529
LmjF33.2170	*L. major*	144	168	311
LmjF33.2170	*L. major*	47	354	400
LmjF33.2170	*L. major*	97	499	595
LmjF33.2170	*L. major*	222	643	864
LmjF33.2170	*L. major*	313	1164	1476
LmjF33.2180	*L. major*	184	69	252
LmjF33.2180	*L. major*	89	265	353
LmjF33.2180	*L. major*	76	376	451
LmjF33.2180	*L. major*	179	455	633
LmjF33.2180	*L. major*	53	760	812
LmjF33.2180	*L. major*	84	849	932

LmjF33.2180	*L. major*	43	968	1010
LmjF33.2180	*L. major*	68	1175	1242
LmjF33.2190	*L. major*	45	1	45
LmjF33.2190	*L. major*	173	70	242
LmjF33.2190	*L. major*	235	251	485
LmjF33.2190	*L. major*	95	509	603
LmjF33.2190	*L. major*	96	635	730
LmjF33.2200	*L. major*	209	1	209
LmjF33.2200	*L. major*	50	232	281
LmjF33.2200	*L. major*	79	317	395
LmjF33.2205	*L. major*	175	1	175
LmjF33.2210	*L. major*	72	1	72
LmjF33.2220	*L. major*	188	1	188
LmjF33.2220	*L. major*	182	214	395
LmjF33.2220	*L. major*	116	467	582
LmjF33.2220	*L. major*	56	605	660
LmjF33.2240	*L. major*	51	175	225
LmjF33.2250	*L. major*	87	117	203
LmjF33.2250	*L. major*	175	278	452
LmjF33.2260	*L. major*	52	158	209
LmjF33.2260	*L. major*	77	330	406
LmjF33.2260	*L. major*	40	1278	1317
LmjF33.2270	*L. major*	61	1	61
LmjF33.2270	*L. major*	347	104	450
LmjF33.2270	*L. major*	71	499	569
LmjF33.2280	*L. major*	80	935	1014
LmjF33.2290	*L. major*	366	1	366
LmjF33.2290	*L. major*	56	906	961
LmjF33.2290	*L. major*	64	1246	1309
LmjF33.2290	*L. major*	155	1311	1465
LmjF33.2290	*L. major*	224	1691	1914
LmjF33.2310	*L. major*	42	53	94
LmjF33.2310	*L. major*	236	130	365
LmjF33.2320	*L. major*	98	146	243
LmjF33.2320	*L. major*	123	544	666
LmjF33.2330	*L. major*	272	1	272
LmjF33.2330	*L. major*	63	348	410
LmjF33.2330	*L. major*	74	447	520
LmjF33.2330	*L. major*	194	734	927
LmjF33.2330	*L. major*	79	989	1067
LmjF33.2350	*L. major*	104	1	104
LmjF33.2350	*L. major*	40	406	445
LmjF33.2350	*L. major*	247	604	850
LmjF33.2360	*L. major*	100	1	100
LmjF33.2360	*L. major*	72	298	369
LmjF33.2360	*L. major*	103	389	491
LmjF33.2360	*L. major*	83	786	868

LmjF33.2370	*L. major*	56	1	56
LmjF33.2370	*L. major*	160	192	351
LmjF33.2370	*L. major*	160	192	351
LmjF33.2380	*L. major*	253	289	541
LmjF33.2400	*L. major*	169	1	169
LmjF33.2410	*L. major*	45	42	86
LmjF33.2410	*L. major*	59	556	614
LmjF33.2410	*L. major*	59	556	614
LmjF33.2420	*L. major*	151	1	151
LmjF33.2420	*L. major*	136	270	405
LmjF33.2420	*L. major*	90	421	510
LmjF33.2430	*L. major*	58	525	582
LmjF33.2440	*L. major*	140	1	140
LmjF33.2440	*L. major*	87	171	257
LmjF33.2440	*L. major*	58	264	321
LmjF33.2440	*L. major*	164	343	506
LmjF33.2450	*L. major*	47	222	268
LmjF33.2450	*L. major*	48	362	409
LmjF33.2450	*L. major*	287	434	720
LmjF33.2450	*L. major*	72	757	828
LmjF33.2450	*L. major*	68	873	940
LmjF33.2450	*L. major*	85	986	1070
LmjF33.2450	*L. major*	248	1126	1373
LmjF33.2450	*L. major*	240	1403	1642
LmjF33.2460	*L. major*	99	1	99
LmjF33.2460	*L. major*	46	270	315
LmjF33.2460	*L. major*	93	447	539
LmjF33.2460	*L. major*	63	989	1051
LmjF33.2480	*L. major*	68	69	136
LmjF33.2480	*L. major*	40	367	406
LmjF33.2480	*L. major*	40	367	406
LmjF33.2490	*L. major*	185	34	218
LmjF33.2490	*L. major*	51	306	356
LmjF33.2490	*L. major*	94	398	491
LmjF33.2490	*L. major*	86	602	687
LmjF33.2510	*L. major*	55	48	102
LmjF33.2510	*L. major*	69	250	318
LmjF33.2510	*L. major*	57	359	415
LmjF33.2520	*L. major*	51	162	212
LmjF33.2530	*L. major*	73	1	73
LmjF33.2530	*L. major*	41	566	606
LmjF33.2530	*L. major*	62	996	1057
LmjF33.2530	*L. major*	141	1106	1246
LmjF33.2530	*L. major*	42	1251	1292
LmjF33.2560	*L. major*	43	1	43
LmjF33.2560	*L. major*	49	286	334
LmjF33.2560	*L. major*	43	396	438

LmjF33.2560	*L. major*	208	472	679
LmjF33.2560	*L. major*	54	726	779
LmjF33.2560	*L. major*	319	857	1175
LmjF33.2560	*L. major*	44	1657	1700
LmjF33.2585	*L. major*	81	1	81
LmjF33.2585	*L. major*	49	91	139
LmjF33.2585	*L. major*	75	221	295
LmjF33.2585	*L. major*	77	369	445
LmjF33.2590	*L. major*	212	262	473
LmjF33.2590	*L. major*	128	523	650
LmjF33.2590	*L. major*	68	748	815
LmjF33.2590	*L. major*	128	914	1041
LmjF33.2600	*L. major*	51	754	804
LmjF33.2620	*L. major*	56	401	456
LmjF33.2630	*L. major*	217	1	217
LmjF33.2630	*L. major*	161	274	434
LmjF33.2630	*L. major*	100	478	577
LmjF33.2630	*L. major*	66	779	844
LmjF33.2640	*L. major*	55	1	55
LmjF33.2640	*L. major*	64	323	386
LmjF33.2640	*L. major*	64	323	386
LmjF33.2650	*L. major*	51	167	217
LmjF33.2650	*L. major*	68	364	431
LmjF33.2650	*L. major*	46	657	702
LmjF33.2660	*L. major*	85	14	98
LmjF33.2660	*L. major*	42	185	226
LmjF33.2660	*L. major*	44	299	342
LmjF33.2660	*L. major*	79	464	542
LmjF33.2660	*L. major*	41	602	642
LmjF33.2660	*L. major*	58	653	710
LmjF33.2660	*L. major*	46	766	811
LmjF33.2660	*L. major*	82	865	946
LmjF33.2660	*L. major*	54	987	1040
LmjF33.2660	*L. major*	92	1075	1166
LmjF33.2670	*L. major*	84	1	84
LmjF33.2670	*L. major*	186	109	294
LmjF33.2670	*L. major*	507	316	822
LmjF33.2670	*L. major*	193	883	1075
LmjF33.2670	*L. major*	113	1120	1232
LmjF33.2680	*L. major*	313	17	329
LmjF33.2680	*L. major*	243	412	654
LmjF33.2680	*L. major*	134	686	819
LmjF33.2690	*L. major*	77	135	211
LmjF33.2700	*L. major*	61	542	602
LmjF33.2700	*L. major*	125	671	795
LmjF33.2710	*L. major*	51	70	120
LmjF33.2720	*L. major*	43	58	100

LmjF33.2730	*L. major*	50	1	50
LmjF33.2730	*L. major*	66	96	161
LmjF33.2730	*L. major*	66	96	161
LmjF33.2740	*L. major*	222	1	222
LmjF33.2750	*L. major*	55	43	97
LmjF33.2750	*L. major*	66	205	270
LmjF33.2750	*L. major*	95	375	469
LmjF33.2750	*L. major*	71	600	670
LmjF33.2750	*L. major*	59	791	849
LmjF33.2750	*L. major*	141	952	1092
LmjF33.2760	*L. major*	212	1	212
LmjF33.2760	*L. major*	41	222	262
LmjF33.2760	*L. major*	71	340	410
LmjF33.2760	*L. major*	42	582	623
LmjF33.2770	*L. major*	83	161	243
LmjF33.2780	*L. major*	54	35	88
LmjF33.2780	*L. major*	113	295	407
LmjF33.2780	*L. major*	89	527	615
LmjF33.2780	*L. major*	60	659	718
LmjF33.2780	*L. major*	213	773	985
LmjF33.2780	*L. major*	49	1075	1123
LmjF33.2780	*L. major*	95	1243	1337
LmjF33.2780	*L. major*	66	1918	1983
LmjF33.2780	*L. major*	128	2487	2614
LmjF33.2780	*L. major*	81	2627	2707
LmjF33.2780	*L. major*	352	2879	3230
LmjF33.2780	*L. major*	174	3344	3517
LmjF33.2780	*L. major*	66	3872	3937
LmjF33.2780	*L. major*	77	4050	4126
LmjF33.2780	*L. major*	47	4502	4548
LmjF33.2780	*L. major*	51	4970	5020
LmjF33.2790	*L. major*	51	189	239
LmjF33.2790	*L. major*	66	287	352
LmjF33.2790	*L. major*	55	424	478
LmjF33.2800	*L. major*	44	1	44
LmjF33.2810	*L. major*	84	92	175
LmjF33.2810	*L. major*	74	245	318
LmjF33.2810	*L. major*	41	383	423
LmjF33.2820	*L. major*	241	58	298
LmjF33.2820	*L. major*	74	498	571
LmjF33.2835	*L. major*	158	46	203
LmjF33.2835	*L. major*	199	240	438
LmjF33.2850	*L. major*	56	44	99
LmjF33.2860	*L. major*	81	1	81
LmjF33.2860	*L. major*	57	326	382
LmjF33.2860	*L. major*	57	326	382
LmjF33.2880	*L. major*	68	175	242

LmjF33.2880	*L. major*	50	256	305
LmjF33.2880	*L. major*	66	349	414
LmjF33.2890	*L. major*	76	82	157
LmjF33.2890	*L. major*	97	193	289
LmjF33.2890	*L. major*	54	325	378
LmjF33.2890	*L. major*	457	431	887
LmjF33.2890	*L. major*	246	904	1149
LmjF33.2890	*L. major*	122	1167	1288
LmjF33.2890	*L. major*	433	1532	1964
LmjF33.2890	*L. major*	95	2290	2384
LmjF33.2900	*L. major*	67	1	67
LmjF33.2900	*L. major*	334	79	412
LmjF33.2900	*L. major*	200	446	645
LmjF33.2900	*L. major*	81	662	742
LmjF33.2900	*L. major*	351	758	1108
LmjF33.2900	*L. major*	46	1230	1275
LmjF33.2900	*L. major*	64	1640	1703
LmjF33.2900	*L. major*	83	2101	2183
LmjF33.2900	*L. major*	43	2235	2277
LmjF33.2900	*L. major*	45	2602	2646
LmjF33.2910	*L. major*	99	1	99
LmjF33.2910	*L. major*	98	155	252
LmjF33.2910	*L. major*	57	573	629
LmjF33.2910	*L. major*	125	905	1029
LmjF33.2910	*L. major*	48	1287	1334
LmjF33.2910	*L. major*	82	1356	1437
LmjF33.2920	*L. major*	288	24	311
LmjF33.2930	*L. major*	263	51	313
LmjF33.2930	*L. major*	100	593	692
LmjF33.2930	*L. major*	66	1015	1080
LmjF33.2940	*L. major*	201	1	201
LmjF33.2940	*L. major*	100	214	313
LmjF33.2940	*L. major*	84	575	658
LmjF33.2960	*L. major*	203	1	203
LmjF33.2960	*L. major*	164	214	377
LmjF33.2960	*L. major*	77	594	670
LmjF33.2970	*L. major*	50	1	50
LmjF33.2970	*L. major*	54	62	115
LmjF33.2970	*L. major*	162	134	295
LmjF33.2970	*L. major*	241	317	557
LmjF33.2970	*L. major*	358	587	944
LmjF33.2970	*L. major*	89	997	1085
LmjF33.2970	*L. major*	67	1171	1237
LmjF33.2970	*L. major*	90	1251	1340
LmjF33.2980	*L. major*	360	1	360
LmjF33.2980	*L. major*	51	678	728
LmjF33.2980	*L. major*	159	1023	1181

LmjF33.2990	*L. major*	102	1	102
LmjF33.2990	*L. major*	110	223	332
LmjF33.3000	*L. major*	528	1	528
LmjF33.3000	*L. major*	62	898	959
LmjF33.3010	*L. major*	646	42	687
LmjF33.3020	*L. major*	381	1	381
LmjF33.3020	*L. major*	109	395	503
LmjF33.3020	*L. major*	123	511	633
LmjF33.3020	*L. major*	334	642	975
LmjF33.3030	*L. major*	98	1	98
LmjF33.3030	*L. major*	75	101	175
LmjF33.3030	*L. major*	246	196	441
LmjF33.3030	*L. major*	62	460	521
LmjF33.3030	*L. major*	154	632	785
LmjF33.3030	*L. major*	274	801	1074
LmjF33.3040	*L. major*	46	279	324
LmjF33.3040	*L. major*	50	561	610
LmjF33.3040	*L. major*	45	1053	1097
LmjF33.3050	*L. major*	72	81	152
LmjF33.3050	*L. major*	49	237	285
LmjF33.3050	*L. major*	40	569	608
LmjF33.3070	*L. major*	115	1	115
LmjF33.3070	*L. major*	74	199	272
LmjF33.3070	*L. major*	74	359	432
LmjF33.3070	*L. major*	133	519	651
LmjF33.3070	*L. major*	73	739	811
LmjF33.3070	*L. major*	64	898	961
LmjF33.3070	*L. major*	64	1045	1108
LmjF33.3070	*L. major*	64	1192	1255
LmjF33.3070	*L. major*	64	1339	1402
LmjF33.3070	*L. major*	133	1486	1618
LmjF33.3070	*L. major*	73	1706	1778
LmjF33.3070	*L. major*	74	1865	1938
LmjF33.3070	*L. major*	73	2026	2098
LmjF33.3070	*L. major*	74	2185	2258
LmjF33.3070	*L. major*	63	2345	2407
LmjF33.3070	*L. major*	74	2492	2565
LmjF33.3070	*L. major*	64	2652	2715
LmjF33.3070	*L. major*	64	2799	2862
LmjF33.3070	*L. major*	73	2947	3019
LmjF33.3070	*L. major*	137	3106	3242
LmjF33.3070	*L. major*	75	3325	3399
LmjF33.3070	*L. major*	74	3485	3558
LmjF33.3070	*L. major*	136	3645	3780
LmjF33.3070	*L. major*	74	3864	3937
LmjF33.3070	*L. major*	74	4024	4097
LmjF33.3070	*L. major*	137	4184	4320

LmjF33.3070	*L. major*	75	4403	4477
LmjF33.3070	*L. major*	74	4563	4636
LmjF33.3070	*L. major*	137	4723	4859
LmjF33.3070	*L. major*	74	4942	5015
LmjF33.3070	*L. major*	137	5102	5238
LmjF33.3070	*L. major*	1532	5321	6852
LmjF33.3070	*L. major*	133	6971	7103
LmjF33.3070	*L. major*	243	7309	7551
LmjF33.3080	*L. major*	102	35	136
LmjF33.3080	*L. major*	55	164	218
LmjF33.3080	*L. major*	47	259	305
LmjF33.3080	*L. major*	45	343	387
LmjF33.3080	*L. major*	70	414	483
LmjF33.3080	*L. major*	51	604	654
LmjF33.3080	*L. major*	53	690	742
LmjF33.3080	*L. major*	116	753	868
LmjF33.3080	*L. major*	137	879	1015
LmjF33.3080	*L. major*	44	1371	1414
LmjF33.3080	*L. major*	302	1711	2012
LmjF33.3080	*L. major*	97	2054	2150
LmjF33.3090	*L. major*	142	1	142
LmjF33.3090	*L. major*	43	1148	1190
LmjF33.3100	*L. major*	55	233	287
LmjF33.3100	*L. major*	50	358	407
LmjF33.3100	*L. major*	50	358	407
LmjF33.3150	*L. major*	54	1	54
LmjF33.3160	*L. major*	65	1	65
LmjF33.3160	*L. major*	73	269	341
LmjF33.3160	*L. major*	44	367	410
LmjF33.3170	*L. major*	307	183	489
LmjF33.3180	*L. major*	40	343	382
LmjF33.3180	*L. major*	214	399	612
LmjF33.3210	*L. major*	92	793	884
LmjF33.3230	*L. major*	49	30	78
LmjF33.3240	*L. major*	143	1	143
LmjF33.3250	*L. major*	337	1	337
LmjF34.0030	*L. major*	44	352	395
LmjF34.0040	*L. major*	48	398	445
LmjF34.0040	*L. major*	64	508	571
LmjF34.0040	*L. major*	64	508	571
LmjF34.0060	*L. major*	43	237	279
LmjF34.0060	*L. major*	63	509	571
LmjF34.0060	*L. major*	56	614	669
LmjF34.0060	*L. major*	135	882	1016
LmjF34.0090	*L. major*	58	42	99
LmjF34.0100	*L. major*	108	421	528
LmjF34.0110	*L. major*	41	125	165

LmjF34.0170	*L. major*	332	1	332
LmjF34.0175	*L. major*	153	1	153
LmjF34.0175	*L. major*	77	178	254
LmjF34.0175	*L. major*	234	260	493
LmjF34.0175	*L. major*	126	509	634
LmjF34.0180	*L. major*	101	263	363
LmjF34.0180	*L. major*	46	427	472
LmjF34.0200	*L. major*	54	350	403
LmjF34.0210	*L. major*	90	120	209
LmjF34.0230	*L. major*	243	202	444
LmjF34.0230	*L. major*	113	547	659
LmjF34.0240	*L. major*	450	73	522
LmjF34.0240	*L. major*	312	538	849
LmjF34.0240	*L. major*	51	883	933
LmjF34.0240	*L. major*	306	1032	1337
LmjF34.0250	*L. major*	172	1	172
LmjF34.0250	*L. major*	96	189	284
LmjF34.0250	*L. major*	88	296	383
LmjF34.0260	*L. major*	77	1	77
LmjF34.0270	*L. major*	74	300	373
LmjF34.0280	*L. major*	198	1	198
LmjF34.0280	*L. major*	59	231	289
LmjF34.0280	*L. major*	72	322	393
LmjF34.0280	*L. major*	41	1348	1388
LmjF34.0300	*L. major*	82	1	82
LmjF34.0300	*L. major*	150	608	757
LmjF34.0310	*L. major*	146	251	396
LmjF34.0320	*L. major*	62	297	358
LmjF34.0330	*L. major*	62	207	268
LmjF34.0330	*L. major*	47	411	457
LmjF34.0340	*L. major*	43	163	205
LmjF34.0340	*L. major*	105	217	321
LmjF34.0340	*L. major*	66	386	451
LmjF34.0340	*L. major*	86	579	664
LmjF34.0350	*L. major*	75	134	208
LmjF34.0360	*L. major*	42	748	789
LmjF34.0360	*L. major*	103	1004	1106
LmjF34.0360	*L. major*	62	1483	1544
LmjF34.0370	*L. major*	103	1	103
LmjF34.0370	*L. major*	50	133	182
LmjF34.0370	*L. major*	42	269	310
LmjF34.0370	*L. major*	40	486	525
LmjF34.0370	*L. major*	45	780	824
LmjF34.0380	*L. major*	64	162	225
LmjF34.0380	*L. major*	50	376	425
LmjF34.0380	*L. major*	50	376	425
LmjF34.0390	*L. major*	42	323	364

LmjF34.0390	*L. major*	45	368	412
LmjF34.0390	*L. major*	45	368	412
LmjF34.0410	*L. major*	48	332	379
LmjF34.0420	*L. major*	83	43	125
LmjF34.0420	*L. major*	58	254	311
LmjF34.0430	*L. major*	74	29	102
LmjF34.0440	*L. major*	40	1	40
LmjF34.0450	*L. major*	936	1	936
LmjF34.0460	*L. major*	71	1745	1815
LmjF34.0460	*L. major*	68	1855	1922
LmjF34.0460	*L. major*	42	1956	1997
LmjF34.0460	*L. major*	168	2180	2347
LmjF34.0470	*L. major*	333	384	716
LmjF34.0480	*L. major*	133	461	593
LmjF34.0480	*L. major*	119	643	761
LmjF34.0480	*L. major*	66	810	875
LmjF34.0480	*L. major*	81	1268	1348
LmjF34.0480	*L. major*	45	1367	1411
LmjF34.0480	*L. major*	171	2385	2555
LmjF34.0495	*L. major*	217	41	257
LmjF34.0510	*L. major*	50	1	50
LmjF34.0510	*L. major*	51	491	541
LmjF34.0510	*L. major*	51	491	541
LmjF34.0520	*L. major*	291	1	291
LmjF34.0520	*L. major*	442	332	773
LmjF34.0520	*L. major*	52	807	858
LmjF34.0520	*L. major*	555	889	1443
LmjF34.0530	*L. major*	299	1	299
LmjF34.0530	*L. major*	155	337	491
LmjF34.0540	*L. major*	45	67	111
LmjF34.0540	*L. major*	48	342	389
LmjF34.0550	*L. major*	46	1	46
LmjF34.0550	*L. major*	94	59	152
LmjF34.0550	*L. major*	106	163	268
LmjF34.0560	*L. major*	122	33	154
LmjF34.0560	*L. major*	41	426	466
LmjF34.0560	*L. major*	53	752	804
LmjF34.0560	*L. major*	122	989	1110
LmjF34.0560	*L. major*	54	1506	1559
LmjF34.0560	*L. major*	158	1591	1748
LmjF34.0570	*L. major*	51	1	51
LmjF34.0570	*L. major*	105	286	390
LmjF34.0570	*L. major*	42	470	511
LmjF34.0570	*L. major*	46	789	834
LmjF34.0580	*L. major*	90	33	122
LmjF34.0590	*L. major*	88	148	235
LmjF34.0590	*L. major*	41	340	380

LmjF34.0590	*L. major*	49	904	952
LmjF34.0590	*L. major*	44	983	1026
LmjF34.0590	*L. major*	41	1052	1092
LmjF34.0600	*L. major*	115	1	115
LmjF34.0600	*L. major*	97	153	249
LmjF34.0600	*L. major*	259	283	541
LmjF34.0600	*L. major*	311	543	853
LmjF34.0610	*L. major*	71	200	270
LmjF34.0620	*L. major*	772	1	772
LmjF34.0620	*L. major*	134	789	922
LmjF34.0620	*L. major*	59	1022	1080
LmjF34.0620	*L. major*	165	1111	1275
LmjF34.0620	*L. major*	1082	1317	2398
LmjF34.0630	*L. major*	168	49	216
LmjF34.0630	*L. major*	70	256	325
LmjF34.0630	*L. major*	95	443	537
LmjF34.0630	*L. major*	49	568	616
LmjF34.0630	*L. major*	85	648	732
LmjF34.0660	*L. major*	119	1	119
LmjF34.0660	*L. major*	228	162	389
LmjF34.0660	*L. major*	54	507	560
LmjF34.0660	*L. major*	348	651	998
LmjF34.0660	*L. major*	40	1030	1069
LmjF34.0670	*L. major*	68	350	417
LmjF34.0670	*L. major*	67	646	712
LmjF34.0670	*L. major*	60	776	835
LmjF34.0670	*L. major*	46	980	1025
LmjF34.0670	*L. major*	90	1257	1346
LmjF34.0670	*L. major*	78	1682	1759
LmjF34.0670	*L. major*	97	1875	1971
LmjF34.0680	*L. major*	91	372	462
LmjF34.0680	*L. major*	106	479	584
LmjF34.0680	*L. major*	83	602	684
LmjF34.0680	*L. major*	106	701	806
LmjF34.0680	*L. major*	83	824	906
LmjF34.0680	*L. major*	102	923	1024
LmjF34.0680	*L. major*	97	1100	1196
LmjF34.0690	*L. major*	71	598	668
LmjF34.0690	*L. major*	127	670	796
LmjF34.0690	*L. major*	2186	808	2993
LmjF34.0690	*L. major*	43	3027	3069
LmjF34.0700	*L. major*	120	110	229
LmjF34.0710	*L. major*	41	130	170
LmjF34.0710	*L. major*	55	239	293
LmjF34.0710	*L. major*	55	715	769
LmjF34.0720	*L. major*	132	287	418
LmjF34.0740	*L. major*	43	341	383

LmjF34.0760	*L. major*	61	139	199
LmjF34.0770	*L. major*	144	1	144
LmjF34.0820	*L. major*	87	68	154
LmjF34.0840	*L. major*	87	68	154
LmjF34.0860	*L. major*	46	177	222
LmjF34.0870	*L. major*	66	15	80
LmjF34.0870	*L. major*	81	180	260
LmjF34.0870	*L. major*	61	299	359
LmjF34.0870	*L. major*	132	431	562
LmjF34.0880	*L. major*	40	1	40
LmjF34.0880	*L. major*	72	453	524
LmjF34.0880	*L. major*	72	453	524
LmjF34.0890	*L. major*	67	1	67
LmjF34.0900	*L. major*	100	164	263
LmjF34.0920	*L. major*	317	81	397
LmjF34.0920	*L. major*	56	597	652
LmjF34.0940	*L. major*	484	1	484
LmjF34.0940	*L. major*	41	571	611
LmjF34.0940	*L. major*	49	704	752
LmjF34.0940	*L. major*	73	835	907
LmjF34.0940	*L. major*	63	939	1001
LmjF34.0950	*L. major*	46	549	594
LmjF34.1000	*L. major*	50	274	323
LmjF34.1000	*L. major*	40	1054	1093
LmjF34.1000	*L. major*	213	1161	1373
LmjF34.1010	*L. major*	91	125	215
LmjF34.1010	*L. major*	55	246	300
LmjF34.1010	*L. major*	143	345	487
LmjF34.1020	*L. major*	55	1	55
LmjF34.1030	*L. major*	92	21	112
LmjF34.1050	*L. major*	46	1	46
LmjF34.1050	*L. major*	57	180	236
LmjF34.1050	*L. major*	57	180	236
LmjF34.1070	*L. major*	80	275	354
LmjF34.1090	*L. major*	50	50	99
LmjF34.1090	*L. major*	55	596	650
LmjF34.1090	*L. major*	87	933	1019
LmjF34.1100	*L. major*	114	38	151
LmjF34.1100	*L. major*	58	237	294
LmjF34.1110	*L. major*	268	268	535
LmjF34.1120	*L. major*	110	119	228
LmjF34.1130	*L. major*	592	1	592
LmjF34.1130	*L. major*	541	594	1134
LmjF34.1130	*L. major*	114	1184	1297
LmjF34.1130	*L. major*	587	1325	1911
LmjF34.1130	*L. major*	486	1920	2405
LmjF34.1140	*L. major*	42	347	388

LmjF34.1150	*L. major*	67	1	67
LmjF34.1150	*L. major*	95	149	243
LmjF34.1150	*L. major*	49	288	336
LmjF34.1155	*L. major*	87	98	184
LmjF34.1160	*L. major*	65	347	411
LmjF34.1170	*L. major*	617	49	665
LmjF34.1175	*L. major*	79	180	258
LmjF34.1180	*L. major*	55	103	157
LmjF34.1180	*L. major*	82	218	299
LmjF34.1190	*L. major*	91	78	168
LmjF34.1190	*L. major*	64	233	296
LmjF34.1190	*L. major*	76	392	467
LmjF34.1190	*L. major*	64	1139	1202
LmjF34.1190	*L. major*	79	1242	1320
LmjF34.1190	*L. major*	50	1482	1531
LmjF34.1210	*L. major*	42	92	133
LmjF34.1210	*L. major*	76	220	295
LmjF34.1210	*L. major*	65	411	475
LmjF34.1220	*L. major*	48	73	120
LmjF34.1220	*L. major*	49	193	241
LmjF34.1220	*L. major*	160	545	704
LmjF34.1230	*L. major*	83	1	83
LmjF34.1230	*L. major*	43	118	160
LmjF34.1230	*L. major*	77	785	861
LmjF34.1230	*L. major*	43	1089	1131
LmjF34.1230	*L. major*	64	1148	1211
LmjF34.1230	*L. major*	91	1284	1374
LmjF34.1230	*L. major*	136	1408	1543
LmjF34.1240	*L. major*	48	174	221
LmjF34.1260	*L. major*	87	1	87
LmjF34.1260	*L. major*	98	525	622
LmjF34.1270	*L. major*	103	71	173
LmjF34.1270	*L. major*	40	596	635
LmjF34.1270	*L. major*	157	732	888
LmjF34.1270	*L. major*	66	915	980
LmjF34.1270	*L. major*	40	1669	1708
LmjF34.1280	*L. major*	80	225	304
LmjF34.1290	*L. major*	143	517	659
LmjF34.1290	*L. major*	49	667	715
LmjF34.1300	*L. major*	67	48	114
LmjF34.1300	*L. major*	161	196	356
LmjF34.1310	*L. major*	58	1	58
LmjF34.1310	*L. major*	120	197	316
LmjF34.1310	*L. major*	73	643	715
LmjF34.1310	*L. major*	115	743	857
LmjF34.1320	*L. major*	219	63	281
LmjF34.1320	*L. major*	40	344	383

LmjF34.1320	*L. major*	81	420	500
LmjF34.1320	*L. major*	70	562	631
LmjF34.1320	*L. major*	83	689	771
LmjF34.1340	*L. major*	55	70	124
LmjF34.1340	*L. major*	291	326	616
LmjF34.1340	*L. major*	295	1007	1301
LmjF34.1350	*L. major*	66	1	66
LmjF34.1350	*L. major*	76	75	150
LmjF34.1350	*L. major*	154	206	359
LmjF34.1360	*L. major*	101	50	150
LmjF34.1360	*L. major*	50	315	364
LmjF34.1360	*L. major*	40	701	740
LmjF34.1360	*L. major*	43	850	892
LmjF34.1360	*L. major*	139	1610	1748
LmjF34.1360	*L. major*	51	1820	1870
LmjF34.1360	*L. major*	60	2123	2182
LmjF34.1360	*L. major*	156	2354	2509
LmjF34.1370	*L. major*	60	1	60
LmjF34.1370	*L. major*	65	94	158
LmjF34.1370	*L. major*	93	232	324
LmjF34.1380	*L. major*	140	1	140
LmjF34.1380	*L. major*	124	162	285
LmjF34.1380	*L. major*	47	319	365
LmjF34.1380	*L. major*	134	419	552
LmjF34.1380	*L. major*	41	556	596
LmjF34.1380	*L. major*	49	600	648
LmjF34.1380	*L. major*	61	1122	1182
LmjF34.1380	*L. major*	101	1188	1288
LmjF34.1390	*L. major*	40	119	158
LmjF34.1390	*L. major*	55	191	245
LmjF34.1390	*L. major*	187	469	655
LmjF34.1390	*L. major*	64	700	763
LmjF34.1390	*L. major*	40	946	985
LmjF34.1390	*L. major*	40	1380	1419
LmjF34.1390	*L. major*	44	1468	1511
LmjF34.1390	*L. major*	47	1833	1879
LmjF34.1390	*L. major*	79	1971	2049
LmjF34.1390	*L. major*	74	2155	2228
LmjF34.1390	*L. major*	68	2282	2349
LmjF34.1390	*L. major*	49	2587	2635
LmjF34.1390	*L. major*	40	3347	3386
LmjF34.1390	*L. major*	45	4259	4303
LmjF34.1390	*L. major*	86	4535	4620
LmjF34.1390	*L. major*	62	4680	4741
LmjF34.1420	*L. major*	102	117	218
LmjF34.1440	*L. major*	242	207	448
LmjF34.1440	*L. major*	125	608	732

LmjF34.1440	*L. major*	43	739	781
LmjF34.1450	*L. major*	88	50	137
LmjF34.1450	*L. major*	64	185	248
LmjF34.1450	*L. major*	127	317	443
LmjF34.1450	*L. major*	55	466	520
LmjF34.1460	*L. major*	61	1	61
LmjF34.1460	*L. major*	51	93	143
LmjF34.1460	*L. major*	44	477	520
LmjF34.1460	*L. major*	46	660	705
LmjF34.1470	*L. major*	59	1	59
LmjF34.1470	*L. major*	63	226	288
LmjF34.1470	*L. major*	57	320	376
LmjF34.1480	*L. major*	347	1	347
LmjF34.1480	*L. major*	367	421	787
LmjF34.1480	*L. major*	100	818	917
LmjF34.1480	*L. major*	59	948	1006
LmjF34.1500	*L. major*	55	1	55
LmjF34.1500	*L. major*	51	218	268
LmjF34.1500	*L. major*	80	632	711
LmjF34.1510	*L. major*	107	61	167
LmjF34.1510	*L. major*	313	175	487
LmjF34.1520	*L. major*	153	1	153
LmjF34.1530	*L. major*	117	1	117
LmjF34.1540	*L. major*	238	477	714
LmjF34.1540	*L. major*	142	802	943
LmjF34.1550	*L. major*	75	100	174
LmjF34.1550	*L. major*	53	679	731
LmjF34.1550	*L. major*	43	735	777
LmjF34.1570	*L. major*	59	25	83
LmjF34.1570	*L. major*	56	117	172
LmjF34.1570	*L. major*	44	350	393
LmjF34.1590	*L. major*	59	25	83
LmjF34.1590	*L. major*	56	117	172
LmjF34.1590	*L. major*	44	350	393
LmjF34.1610	*L. major*	59	25	83
LmjF34.1610	*L. major*	56	117	172
LmjF34.1610	*L. major*	48	350	397
LmjF34.1630	*L. major*	59	25	83
LmjF34.1630	*L. major*	56	117	172
LmjF34.1630	*L. major*	44	350	393
LmjF34.1650	*L. major*	59	25	83
LmjF34.1650	*L. major*	56	117	172
LmjF34.1650	*L. major*	44	350	393
LmjF34.1670	*L. major*	59	25	83
LmjF34.1670	*L. major*	56	117	172
LmjF34.1670	*L. major*	44	350	393
LmjF34.1690	*L. major*	59	25	83

LmjF34.1690	*L. major*	56	117	172
LmjF34.1690	*L. major*	44	350	393
LmjF34.1710	*L. major*	59	25	83
LmjF34.1710	*L. major*	56	117	172
LmjF34.1710	*L. major*	44	350	393
LmjF34.1730	*L. major*	59	25	83
LmjF34.1730	*L. major*	56	117	172
LmjF34.1730	*L. major*	44	350	393
LmjF34.1750	*L. major*	59	25	83
LmjF34.1750	*L. major*	56	117	172
LmjF34.1750	*L. major*	44	350	393
LmjF34.1770	*L. major*	59	25	83
LmjF34.1770	*L. major*	56	117	172
LmjF34.1770	*L. major*	44	350	393
LmjF34.1790	*L. major*	59	25	83
LmjF34.1790	*L. major*	56	117	172
LmjF34.1790	*L. major*	44	350	393
LmjF34.1810	*L. major*	59	25	83
LmjF34.1810	*L. major*	56	117	172
LmjF34.1810	*L. major*	44	350	393
LmjF34.1830	*L. major*	59	25	83
LmjF34.1830	*L. major*	56	117	172
LmjF34.1830	*L. major*	44	350	393
LmjF34.1850	*L. major*	59	25	83
LmjF34.1850	*L. major*	56	117	172
LmjF34.1850	*L. major*	44	350	393
LmjF34.1870	*L. major*	59	25	83
LmjF34.1870	*L. major*	56	117	172
LmjF34.1870	*L. major*	44	350	393
LmjF34.1890	*L. major*	59	25	83
LmjF34.1890	*L. major*	56	117	172
LmjF34.1890	*L. major*	45	350	394
LmjF34.1910	*L. major*	59	25	83
LmjF34.1910	*L. major*	56	117	172
LmjF34.1910	*L. major*	44	350	393
LmjF34.1930	*L. major*	59	25	83
LmjF34.1930	*L. major*	56	117	172
LmjF34.1930	*L. major*	44	350	393
LmjF34.1950	*L. major*	59	25	83
LmjF34.1950	*L. major*	56	117	172
LmjF34.1950	*L. major*	44	350	393
LmjF34.1970	*L. major*	59	25	83
LmjF34.1970	*L. major*	56	117	172
LmjF34.1970	*L. major*	44	350	393
LmjF34.1990	*L. major*	44	75	118
LmjF34.1990	*L. major*	140	199	338
LmjF34.1990	*L. major*	239	436	674

LmjF34.2010	*L. major*	99	1	99
LmjF34.2020	*L. major*	50	51	100
LmjF34.2020	*L. major*	155	475	629
LmjF34.2030	*L. major*	43	190	232
LmjF34.2030	*L. major*	131	253	383
LmjF34.2030	*L. major*	177	412	588
LmjF34.2030	*L. major*	47	609	655
LmjF34.2030	*L. major*	58	855	912
LmjF34.2030	*L. major*	51	1076	1126
LmjF34.2040	*L. major*	163	71	233
LmjF34.2050	*L. major*	177	433	609
LmjF34.2050	*L. major*	109	695	803
LmjF34.2060	*L. major*	41	215	255
LmjF34.2080	*L. major*	143	136	278
LmjF34.2080	*L. major*	87	321	407
LmjF34.2080	*L. major*	51	655	705
LmjF34.2080	*L. major*	74	728	801
LmjF34.2080	*L. major*	50	811	860
LmjF34.2080	*L. major*	149	980	1128
LmjF34.2080	*L. major*	46	1167	1212
LmjF34.2080	*L. major*	41	1546	1586
LmjF34.2080	*L. major*	48	1619	1666
LmjF34.2080	*L. major*	59	1684	1742
LmjF34.2080	*L. major*	90	1748	1837
LmjF34.2090	*L. major*	47	1	47
LmjF34.2100	*L. major*	84	151	234
LmjF34.2100	*L. major*	145	394	538
LmjF34.2110	*L. major*	48	49	96
LmjF34.2110	*L. major*	98	323	420
LmjF34.2110	*L. major*	108	431	538
LmjF34.2120	*L. major*	46	261	306
LmjF34.2150	*L. major*	44	220	263
LmjF34.2150	*L. major*	49	303	351
LmjF34.2150	*L. major*	41	404	444
LmjF34.2150	*L. major*	68	470	537
LmjF34.2150	*L. major*	238	667	904
LmjF34.2150	*L. major*	46	1110	1155
LmjF34.2150	*L. major*	205	1211	1415
LmjF34.2160	*L. major*	129	41	169
LmjF34.2170	*L. major*	41	64	104
LmjF34.2170	*L. major*	50	270	319
LmjF34.2170	*L. major*	72	460	531
LmjF34.2180	*L. major*	75	780	854
LmjF34.2180	*L. major*	72	1474	1545
LmjF34.2180	*L. major*	72	1474	1545
LmjF34.2190	*L. major*	51	687	737
LmjF34.2190	*L. major*	89	905	993

LmjF34.2190	*L. major*	89	905	993
LmjF34.2200	*L. major*	58	137	194
LmjF34.2200	*L. major*	43	262	304
LmjF34.2200	*L. major*	43	262	304
LmjF34.2210	*L. major*	131	1	131
LmjF34.2210	*L. major*	63	133	195
LmjF34.2210	*L. major*	151	242	392
LmjF34.2210	*L. major*	193	451	643
LmjF34.2220	*L. major*	90	28	117
LmjF34.2220	*L. major*	57	558	614
LmjF34.2240	*L. major*	53	287	339
LmjF34.2240	*L. major*	51	1124	1174
LmjF34.2240	*L. major*	51	1124	1174
LmjF34.2250	*L. major*	142	56	197
LmjF34.2250	*L. major*	73	611	683
LmjF34.2250	*L. major*	70	695	764
LmjF34.2250	*L. major*	57	795	851
LmjF34.2250	*L. major*	62	957	1018
LmjF34.2250	*L. major*	340	1039	1378
LmjF34.2250	*L. major*	137	1443	1579
LmjF34.2250	*L. major*	72	1667	1738
LmjF34.2250	*L. major*	118	1814	1931
LmjF34.2260	*L. major*	61	44	104
LmjF34.2270	*L. major*	148	394	541
LmjF34.2280	*L. major*	43	49	91
LmjF34.2280	*L. major*	62	268	329
LmjF34.2280	*L. major*	59	547	605
LmjF34.2280	*L. major*	98	636	733
LmjF34.2290	*L. major*	47	110	156
LmjF34.2290	*L. major*	50	282	331
LmjF34.2290	*L. major*	50	282	331
LmjF34.2300	*L. major*	75	15	89
LmjF34.2340	*L. major*	184	56	239
LmjF34.2360	*L. major*	151	1	151
LmjF34.2360	*L. major*	78	167	244
LmjF34.2360	*L. major*	182	251	432
LmjF34.2370	*L. major*	183	1	183
LmjF34.2370	*L. major*	99	267	365
LmjF34.2380	*L. major*	154	1	154
LmjF34.2380	*L. major*	186	176	361
LmjF34.2390	*L. major*	50	1	50
LmjF34.2390	*L. major*	85	82	166
LmjF34.2390	*L. major*	85	82	166
LmjF34.2410	*L. major*	54	13	66
LmjF34.2430	*L. major*	67	1	67
LmjF34.2430	*L. major*	69	96	164
LmjF34.2430	*L. major*	125	229	353

LmjF34.2430	*L. major*	52	416	467
LmjF34.2430	*L. major*	48	761	808
LmjF34.2450	*L. major*	63	43	105
LmjF34.2450	*L. major*	48	430	477
LmjF34.2450	*L. major*	130	534	663
LmjF34.2450	*L. major*	43	1256	1298
LmjF34.2450	*L. major*	56	1653	1708
LmjF34.2460	*L. major*	219	1	219
LmjF34.2460	*L. major*	57	321	377
LmjF34.2460	*L. major*	88	414	501
LmjF34.2476	*L. major*	171	1	171
LmjF34.2476	*L. major*	84	294	377
LmjF34.2479	*L. major*	88	195	282
LmjF34.2480	*L. major*	504	111	614
LmjF34.2480	*L. major*	62	738	799
LmjF34.2480	*L. major*	213	857	1069
LmjF34.2485	*L. major*	87	1	87
LmjF34.2490	*L. major*	83	223	305
LmjF34.2500	*L. major*	119	1	119
LmjF34.2510	*L. major*	151	1	151
LmjF34.2515	*L. major*	55	58	112
LmjF34.2520	*L. major*	138	1	138
LmjF34.2520	*L. major*	47	204	250
LmjF34.2520	*L. major*	128	357	484
LmjF34.2520	*L. major*	53	514	566
LmjF34.2520	*L. major*	49	765	813
LmjF34.2520	*L. major*	96	887	982
LmjF34.2520	*L. major*	42	1141	1182
LmjF34.2520	*L. major*	58	1442	1499
LmjF34.2520	*L. major*	45	2151	2195
LmjF34.2530	*L. major*	70	52	121
LmjF34.2530	*L. major*	67	183	249
LmjF34.2530	*L. major*	742	412	1153
LmjF34.2530	*L. major*	1092	1241	2332
LmjF34.2530	*L. major*	511	2420	2930
LmjF34.2530	*L. major*	511	3018	3528
LmjF34.2530	*L. major*	198	3616	3813
LmjF34.2530	*L. major*	432	3883	4314
LmjF34.2530	*L. major*	67	4329	4395
LmjF34.2540	*L. major*	47	13	59
LmjF34.2540	*L. major*	423	84	506
LmjF34.2540	*L. major*	183	512	694
LmjF34.2550	*L. major*	68	54	121
LmjF34.2550	*L. major*	43	155	197
LmjF34.2550	*L. major*	196	299	494
LmjF34.2550	*L. major*	82	496	577
LmjF34.2560	*L. major*	120	53	172

LmjF34.2560	*L. major*	64	209	272
LmjF34.2560	*L. major*	2577	282	2858
LmjF34.2560	*L. major*	116	2913	3028
LmjF34.2570	*L. major*	268	56	323
LmjF34.2570	*L. major*	50	358	407
LmjF34.2570	*L. major*	44	438	481
LmjF34.2580	*L. major*	77	129	205
LmjF34.2590	*L. major*	82	339	420
LmjF34.2610	*L. major*	51	1	51
LmjF34.2610	*L. major*	59	129	187
LmjF34.2610	*L. major*	59	129	187
LmjF34.2620	*L. major*	42	327	368
LmjF34.2630	*L. major*	136	288	423
LmjF34.2630	*L. major*	57	679	735
LmjF34.2640	*L. major*	49	146	194
LmjF34.2640	*L. major*	81	734	814
LmjF34.2640	*L. major*	92	885	976
LmjF34.2640	*L. major*	380	986	1365
LmjF34.2660	*L. major*	68	289	356
LmjF34.2660	*L. major*	69	645	713
LmjF34.2660	*L. major*	99	749	847
LmjF34.2680	*L. major*	40	79	118
LmjF34.2680	*L. major*	93	280	372
LmjF34.2680	*L. major*	44	451	494
LmjF34.2690	*L. major*	251	34	284
LmjF34.2690	*L. major*	256	316	571
LmjF34.2700	*L. major*	42	1	42
LmjF34.2700	*L. major*	108	83	190
LmjF34.2710	*L. major*	99	1	99
LmjF34.2720	*L. major*	125	256	380
LmjF34.2720	*L. major*	56	629	684
LmjF34.2720	*L. major*	56	629	684
LmjF34.2730	*L. major*	51	369	419
LmjF34.2740	*L. major*	40	13	52
LmjF34.2740	*L. major*	102	83	184
LmjF34.2750	*L. major*	53	136	188
LmjF34.2750	*L. major*	123	305	427
LmjF34.2760	*L. major*	51	60	110
LmjF34.2760	*L. major*	45	257	301
LmjF34.2765	*L. major*	156	1	156
LmjF34.2765	*L. major*	160	184	343
LmjF34.2770	*L. major*	68	109	176
LmjF34.2770	*L. major*	43	279	321
LmjF34.2770	*L. major*	47	371	417
LmjF34.2790	*L. major*	62	83	144
LmjF34.2790	*L. major*	45	355	399
LmjF34.2790	*L. major*	48	553	600

LmjF34.2810	*L. major*	86	151	236
LmjF34.2810	*L. major*	64	279	342
LmjF34.2810	*L. major*	79	758	836
LmjF34.2820	*L. major*	118	36	153
LmjF34.2820	*L. major*	171	186	356
LmjF34.2820	*L. major*	59	590	648
LmjF34.2840	*L. major*	45	610	654
LmjF34.2850	*L. major*	66	126	191
LmjF34.2870	*L. major*	42	1	42
LmjF34.2870	*L. major*	87	333	419
LmjF34.2870	*L. major*	87	333	419
LmjF34.2880	*L. major*	42	1	42
LmjF34.2880	*L. major*	87	333	419
LmjF34.2880	*L. major*	87	333	419
LmjF34.2890	*L. major*	42	1	42
LmjF34.2890	*L. major*	87	333	419
LmjF34.2890	*L. major*	87	333	419
LmjF34.2900	*L. major*	42	1	42
LmjF34.2900	*L. major*	87	333	419
LmjF34.2900	*L. major*	87	333	419
LmjF34.2910	*L. major*	40	393	432
LmjF34.2930	*L. major*	57	58	114
LmjF34.2940	*L. major*	41	1	41
LmjF34.2940	*L. major*	44	199	242
LmjF34.2940	*L. major*	44	199	242
LmjF34.2960	*L. major*	54	167	220
LmjF34.2970	*L. major*	103	80	182
LmjF34.2990	*L. major*	95	106	200
LmjF34.3000	*L. major*	95	1	95
LmjF34.3000	*L. major*	111	172	282
LmjF34.3000	*L. major*	86	284	369
LmjF34.3000	*L. major*	56	375	430
LmjF34.3000	*L. major*	148	441	588
LmjF34.3010	*L. major*	41	209	249
LmjF34.3020	*L. major*	267	29	295
LmjF34.3020	*L. major*	43	328	370
LmjF34.3020	*L. major*	324	617	940
LmjF34.3030	*L. major*	148	1	148
LmjF34.3040	*L. major*	55	103	157
LmjF34.3040	*L. major*	75	195	269
LmjF34.3040	*L. major*	41	380	420
LmjF34.3060	*L. major*	79	60	138
LmjF34.3070	*L. major*	112	1	112
LmjF34.3070	*L. major*	49	180	228
LmjF34.3080	*L. major*	81	70	150
LmjF34.3080	*L. major*	168	575	742
LmjF34.3090	*L. major*	58	1	58

LmjF34.3090	*L. major*	147	75	221
LmjF34.3090	*L. major*	40	430	469
LmjF34.3110	*L. major*	49	57	105
LmjF34.3140	*L. major*	50	1	50
LmjF34.3140	*L. major*	74	182	255
LmjF34.3150	*L. major*	83	1	83
LmjF34.3160	*L. major*	63	1	63
LmjF34.3160	*L. major*	168	100	267
LmjF34.3170	*L. major*	149	94	242
LmjF34.3170	*L. major*	175	259	433
LmjF34.3170	*L. major*	175	259	433
LmjF34.3180	*L. major*	194	45	238
LmjF34.3180	*L. major*	137	268	404
LmjF34.3190	*L. major*	77	33	109
LmjF34.3190	*L. major*	54	433	486
LmjF34.3190	*L. major*	54	433	486
LmjF34.3200	*L. major*	41	171	211
LmjF34.3200	*L. major*	212	807	1018
LmjF34.3220	*L. major*	46	1	46
LmjF34.3230	*L. major*	53	47	99
LmjF34.3230	*L. major*	48	242	289
LmjF34.3230	*L. major*	117	398	514
LmjF34.3230	*L. major*	67	666	732
LmjF34.3230	*L. major*	44	868	911
LmjF34.3230	*L. major*	80	1264	1343
LmjF34.3260	*L. major*	104	281	384
LmjF34.3260	*L. major*	140	525	664
LmjF34.3260	*L. major*	140	525	664
LmjF34.3270	*L. major*	64	121	184
LmjF34.3280	*L. major*	40	100	139
LmjF34.3280	*L. major*	110	248	357
LmjF34.3280	*L. major*	60	418	477
LmjF34.3310	*L. major*	60	32	91
LmjF34.3320	*L. major*	42	1	42
LmjF34.3340	*L. major*	71	10	80
LmjF34.3340	*L. major*	74	98	171
LmjF34.3340	*L. major*	64	337	400
LmjF34.3340	*L. major*	71	465	535
LmjF34.3350	*L. major*	40	100	139
LmjF34.3350	*L. major*	116	385	500
LmjF34.3350	*L. major*	116	385	500
LmjF34.3390	*L. major*	60	223	282
LmjF34.3390	*L. major*	82	374	455
LmjF34.3390	*L. major*	44	686	729
LmjF34.3400	*L. major*	60	248	307
LmjF34.3420	*L. major*	41	403	443
LmjF34.3430	*L. major*	41	550	590

LmjF34.3440	*L. major*	178	457	634
LmjF34.3450	*L. major*	50	65	114
LmjF34.3450	*L. major*	121	311	431
LmjF34.3450	*L. major*	143	506	648
LmjF34.3450	*L. major*	91	788	878
LmjF34.3450	*L. major*	174	899	1072
LmjF34.3450	*L. major*	59	1120	1178
LmjF34.3460	*L. major*	50	319	368
LmjF34.3480	*L. major*	52	1	52
LmjF34.3490	*L. major*	43	30	72
LmjF34.3520	*L. major*	45	307	351
LmjF34.3530	*L. major*	57	1356	1412
LmjF34.3530	*L. major*	50	1896	1945
LmjF34.3530	*L. major*	50	1896	1945
LmjF34.3550	*L. major*	242	99	340
LmjF34.3560	*L. major*	148	194	341
LmjF34.3570	*L. major*	420	47	466
LmjF34.3570	*L. major*	191	505	695
LmjF34.3570	*L. major*	226	716	941
LmjF34.3580	*L. major*	67	65	131
LmjF34.3590	*L. major*	51	120	170
LmjF34.3590	*L. major*	64	260	323
LmjF34.3590	*L. major*	64	260	323
LmjF34.3640	*L. major*	78	85	162
LmjF34.3690	*L. major*	73	1	73
LmjF34.3690	*L. major*	53	222	274
LmjF34.3690	*L. major*	40	394	433
LmjF34.3690	*L. major*	80	515	594
LmjF34.3690	*L. major*	49	1015	1063
LmjF34.3690	*L. major*	52	1168	1219
LmjF34.3690	*L. major*	65	1246	1310
LmjF34.3710	*L. major*	127	46	172
LmjF34.3730	*L. major*	63	1	63
LmjF34.3745	*L. major*	60	103	162
LmjF34.3745	*L. major*	246	169	414
LmjF34.3745	*L. major*	70	430	499
LmjF34.3750	*L. major*	131	1	131
LmjF34.3750	*L. major*	48	279	326
LmjF34.3750	*L. major*	93	346	438
LmjF34.3760	*L. major*	60	47	106
LmjF34.3770	*L. major*	56	1	56
LmjF34.3770	*L. major*	95	105	199
LmjF34.3770	*L. major*	95	105	199
LmjF34.3790	*L. major*	68	642	709
LmjF34.3790	*L. major*	50	931	980
LmjF34.3790	*L. major*	44	1054	1097
LmjF34.3820	*L. major*	74	1	74

LmjF34.3820	*L. major*	50	108	157
LmjF34.3820	*L. major*	56	234	289
LmjF34.3830	*L. major*	208	26	233
LmjF34.3870	*L. major*	121	113	233
LmjF34.3870	*L. major*	89	638	726
LmjF34.3870	*L. major*	89	638	726
LmjF34.3880	*L. major*	50	2590	2639
LmjF34.3880	*L. major*	84	3194	3277
LmjF34.3880	*L. major*	65	3400	3464
LmjF34.3880	*L. major*	42	3759	3800
LmjF34.3900	*L. major*	88	120	207
LmjF34.3920	*L. major*	74	84	157
LmjF34.3920	*L. major*	59	322	380
LmjF34.3920	*L. major*	59	322	380
LmjF34.3940	*L. major*	140	2437	2576
LmjF34.3950	*L. major*	162	1	162
LmjF34.3950	*L. major*	40	231	270
LmjF34.3960	*L. major*	53	224	276
LmjF34.3960	*L. major*	45	606	650
LmjF34.3970	*L. major*	92	611	702
LmjF34.3990	*L. major*	44	235	278
LmjF34.4020	*L. major*	42	1	42
LmjF34.4040	*L. major*	44	43	86
LmjF34.4050	*L. major*	62	1	62
LmjF34.4050	*L. major*	72	112	183
LmjF34.4050	*L. major*	73	583	655
LmjF34.4070	*L. major*	75	1	75
LmjF34.4090	*L. major*	308	62	369
LmjF34.4100	*L. major*	47	21	67
LmjF34.4100	*L. major*	43	131	173
LmjF34.4100	*L. major*	43	131	173
LmjF34.4110	*L. major*	57	655	711
LmjF34.4110	*L. major*	50	964	1013
LmjF34.4110	*L. major*	43	1263	1305
LmjF34.4110	*L. major*	46	2034	2079
LmjF34.4140	*L. major*	108	1	108
LmjF34.4140	*L. major*	208	127	334
LmjF34.4140	*L. major*	87	366	452
LmjF34.4150	*L. major*	45	421	465
LmjF34.4160	*L. major*	93	999	1091
LmjF34.4160	*L. major*	109	2833	2941
LmjF34.4160	*L. major*	72	3046	3117
LmjF34.4160	*L. major*	79	3348	3426
LmjF34.4170	*L. major*	101	1	101
LmjF34.4170	*L. major*	87	128	214
LmjF34.4180	*L. major*	97	56	152
LmjF34.4210	*L. major*	281	84	364

LmjF34.4210	*L. major*	81	408	488
LmjF34.4240	*L. major*	135	111	245
LmjF34.4250	*L. major*	94	18	111
LmjF34.4250	*L. major*	66	205	270
LmjF34.4260	*L. major*	46	1	46
LmjF34.4260	*L. major*	42	286	327
LmjF34.4260	*L. major*	181	428	608
LmjF34.4260	*L. major*	167	653	819
LmjF34.4270	*L. major*	44	1	44
LmjF34.4280	*L. major*	71	292	362
LmjF34.4290	*L. major*	63	1	63
LmjF34.4290	*L. major*	107	114	220
LmjF34.4310	*L. major*	49	818	866
LmjF34.4310	*L. major*	47	1085	1131
LmjF34.4310	*L. major*	47	1085	1131
LmjF34.4530	*L. major*	88	1338	1425
LmjF34.4530	*L. major*	46	2321	2366
LmjF34.4530	*L. major*	46	2321	2366
LmjF34.4540	*L. major*	91	18	108
LmjF34.4550	*L. major*	77	1	77
LmjF34.4560	*L. major*	40	1	40
LmjF34.4560	*L. major*	146	181	326
LmjF34.4600	*L. major*	113	1	113
LmjF34.4610	*L. major*	47	332	378
LmjF34.4610	*L. major*	96	398	493
LmjF34.4610	*L. major*	96	398	493
LmjF35.0010	*L. major*	90	1	90
LmjF35.0010	*L. major*	54	133	186
LmjF35.0050	*L. major*	45	1	45
LmjF35.0060	*L. major*	497	174	670
LmjF35.0070	*L. major*	41	194	234
LmjF35.0080	*L. major*	40	1	40
LmjF35.0090	*L. major*	147	29	175
LmjF35.0090	*L. major*	68	184	251
LmjF35.0090	*L. major*	68	184	251
LmjF35.0170	*L. major*	118	52	169
LmjF35.0170	*L. major*	54	224	277
LmjF35.0170	*L. major*	54	224	277
LmjF35.0180	*L. major*	73	39	111
LmjF35.0180	*L. major*	345	124	468
LmjF35.0180	*L. major*	53	487	539
LmjF35.0180	*L. major*	219	560	778
LmjF35.0180	*L. major*	82	805	886
LmjF35.0200	*L. major*	331	69	399
LmjF35.0200	*L. major*	52	467	518
LmjF35.0200	*L. major*	53	520	572
LmjF35.0200	*L. major*	56	611	666

LmjF35.0200	*L. major*	155	733	887
LmjF35.0200	*L. major*	92	933	1024
LmjF35.0200	*L. major*	87	1214	1300
LmjF35.0200	*L. major*	181	1329	1509
LmjF35.0200	*L. major*	164	1554	1717
LmjF35.0200	*L. major*	159	1766	1924
LmjF35.0200	*L. major*	178	1946	2123
LmjF35.0220	*L. major*	104	160	263
LmjF35.0230	*L. major*	83	110	192
LmjF35.0230	*L. major*	65	283	347
LmjF35.0250	*L. major*	57	121	177
LmjF35.0250	*L. major*	139	307	445
LmjF35.0250	*L. major*	48	670	717
LmjF35.0260	*L. major*	59	233	291
LmjF35.0260	*L. major*	44	433	476
LmjF35.0260	*L. major*	44	433	476
LmjF35.0290	*L. major*	638	27	664
LmjF35.0290	*L. major*	115	672	786
LmjF35.0290	*L. major*	41	1081	1121
LmjF35.0300	*L. major*	100	36	135
LmjF35.0300	*L. major*	97	157	253
LmjF35.0310	*L. major*	64	302	365
LmjF35.0320	*L. major*	45	1	45
LmjF35.0340	*L. major*	42	162	203
LmjF35.0340	*L. major*	43	327	369
LmjF35.0340	*L. major*	43	327	369
LmjF35.0350	*L. major*	43	160	202
LmjF35.0350	*L. major*	88	438	525
LmjF35.0350	*L. major*	88	438	525
LmjF35.0370	*L. major*	59	1	59
LmjF35.0380	*L. major*	187	1	187
LmjF35.0380	*L. major*	119	229	347
LmjF35.0380	*L. major*	44	388	431
LmjF35.0380	*L. major*	40	557	596
LmjF35.0390	*L. major*	103	36	138
LmjF35.0390	*L. major*	52	161	212
LmjF35.0390	*L. major*	41	325	365
LmjF35.0390	*L. major*	60	451	510
LmjF35.0430	*L. major*	66	1	66
LmjF35.0430	*L. major*	63	103	165
LmjF35.0430	*L. major*	171	205	375
LmjF35.0440	*L. major*	56	255	310
LmjF35.0440	*L. major*	59	346	404
LmjF35.0450	*L. major*	85	64	148
LmjF35.0450	*L. major*	93	360	452
LmjF35.0460	*L. major*	222	696	917
LmjF35.0470	*L. major*	85	41	125

LmjF35.0490	*L. major*	412	277	688
LmjF35.0490	*L. major*	140	783	922
LmjF35.0490	*L. major*	98	1096	1193
LmjF35.0490	*L. major*	294	1284	1577
LmjF35.0500	*L. major*	72	161	232
LmjF35.0500	*L. major*	585	645	1229
LmjF35.0520	*L. major*	6693	296	6988
LmjF35.0540	*L. major*	11167	296	11462
LmjF35.0540	*L. major*	45	11613	11657
LmjF35.0540	*L. major*	5457	11729	17185
LmjF35.0550	*L. major*	46	489	534
LmjF35.0550	*L. major*	1703	598	2300
LmjF35.0560	*L. major*	358	1	358
LmjF35.0560	*L. major*	91	387	477
LmjF35.0560	*L. major*	159	502	660
LmjF35.0560	*L. major*	47	690	736
LmjF35.0570	*L. major*	61	147	207
LmjF35.0570	*L. major*	49	422	470
LmjF35.0570	*L. major*	67	513	579
LmjF35.0570	*L. major*	437	1344	1780
LmjF35.0570	*L. major*	143	1977	2119
LmjF35.0580	*L. major*	224	1	224
LmjF35.0590	*L. major*	94	257	350
LmjF35.0590	*L. major*	49	371	419
LmjF35.0590	*L. major*	65	623	687
LmjF35.0610	*L. major*	63	23	85
LmjF35.0610	*L. major*	108	135	242
LmjF35.0610	*L. major*	98	433	530
LmjF35.0610	*L. major*	52	638	689
LmjF35.0610	*L. major*	87	858	944
LmjF35.0610	*L. major*	43	1000	1042
LmjF35.0610	*L. major*	59	1087	1145
LmjF35.0610	*L. major*	53	1149	1201
LmjF35.0610	*L. major*	40	1290	1329
LmjF35.0610	*L. major*	51	1712	1762
LmjF35.0610	*L. major*	43	1826	1868
LmjF35.0610	*L. major*	57	2054	2110
LmjF35.0610	*L. major*	45	2141	2185
LmjF35.0610	*L. major*	58	2304	2361
LmjF35.0610	*L. major*	86	2421	2506
LmjF35.0630	*L. major*	74	1	74
LmjF35.0630	*L. major*	121	199	319
LmjF35.0630	*L. major*	41	404	444
LmjF35.0640	*L. major*	54	433	486
LmjF35.0660	*L. major*	91	1	91
LmjF35.0660	*L. major*	68	171	238
LmjF35.0660	*L. major*	260	280	539

LmjF35.0660	*L. major*	131	556	686
LmjF35.0660	*L. major*	49	741	789
LmjF35.0660	*L. major*	87	822	908
LmjF35.0660	*L. major*	100	1169	1268
LmjF35.0670	*L. major*	50	273	322
LmjF35.0670	*L. major*	48	489	536
LmjF35.0670	*L. major*	67	713	779
LmjF35.0670	*L. major*	81	818	898
LmjF35.0670	*L. major*	55	940	994
LmjF35.0670	*L. major*	43	1017	1059
LmjF35.0670	*L. major*	49	1061	1109
LmjF35.0670	*L. major*	50	1315	1364
LmjF35.0670	*L. major*	50	1706	1755
LmjF35.0670	*L. major*	107	2141	2247
LmjF35.0670	*L. major*	42	2674	2715
LmjF35.0670	*L. major*	93	2875	2967
LmjF35.0670	*L. major*	112	3259	3370
LmjF35.0670	*L. major*	42	3822	3863
LmjF35.0670	*L. major*	52	4070	4121
LmjF35.0670	*L. major*	48	4127	4174
LmjF35.0680	*L. major*	433	1	433
LmjF35.0680	*L. major*	231	454	684
LmjF35.0680	*L. major*	246	694	939
LmjF35.0680	*L. major*	155	971	1125
LmjF35.0680	*L. major*	100	1155	1254
LmjF35.0680	*L. major*	71	1265	1335
LmjF35.0680	*L. major*	76	1435	1510
LmjF35.0690	*L. major*	41	582	622
LmjF35.0700	*L. major*	63	214	276
LmjF35.0710	*L. major*	48	108	155
LmjF35.0720	*L. major*	49	385	433
LmjF35.0720	*L. major*	43	459	501
LmjF35.0720	*L. major*	43	459	501
LmjF35.0730	*L. major*	225	1	225
LmjF35.0730	*L. major*	43	256	298
LmjF35.0730	*L. major*	58	480	537
LmjF35.0730	*L. major*	71	659	729
LmjF35.0740	*L. major*	83	225	307
LmjF35.0740	*L. major*	59	388	446
LmjF35.0740	*L. major*	165	482	646
LmjF35.0740	*L. major*	107	656	762
LmjF35.0740	*L. major*	167	765	931
LmjF35.0750	*L. major*	57	114	170
LmjF35.0760	*L. major*	70	221	290
LmjF35.0770	*L. major*	97	152	248
LmjF35.0780	*L. major*	208	100	307
LmjF35.0780	*L. major*	40	359	398

LmjF35.0780	*L. major*	89	400	488
LmjF35.0780	*L. major*	268	512	779
LmjF35.0790	*L. major*	80	1	80
LmjF35.0790	*L. major*	62	125	186
LmjF35.0790	*L. major*	115	265	379
LmjF35.0800	*L. major*	105	61	165
LmjF35.0800	*L. major*	133	217	349
LmjF35.0800	*L. major*	187	392	578
LmjF35.0800	*L. major*	189	590	778
LmjF35.0800	*L. major*	79	792	870
LmjF35.0800	*L. major*	108	908	1015
LmjF35.0810	*L. major*	156	1	156
LmjF35.0810	*L. major*	212	166	377
LmjF35.0810	*L. major*	684	388	1071
LmjF35.0810	*L. major*	60	1104	1163
LmjF35.0810	*L. major*	114	1168	1281
LmjF35.0810	*L. major*	97	1286	1382
LmjF35.0810	*L. major*	81	1409	1489
LmjF35.0840	*L. major*	47	1	47
LmjF35.0840	*L. major*	67	60	126
LmjF35.0840	*L. major*	67	60	126
LmjF35.0860	*L. major*	110	52	161
LmjF35.0870	*L. major*	48	675	722
LmjF35.0880	*L. major*	52	207	258
LmjF35.0880	*L. major*	118	402	519
LmjF35.0890	*L. major*	52	56	107
LmjF35.0900	*L. major*	235	1	235
LmjF35.0910	*L. major*	206	1	206
LmjF35.0910	*L. major*	434	327	760
LmjF35.0910	*L. major*	93	797	889
LmjF35.0910	*L. major*	322	910	1231
LmjF35.0910	*L. major*	251	1233	1483
LmjF35.0910	*L. major*	140	1538	1677
LmjF35.0910	*L. major*	46	1756	1801
LmjF35.0920	*L. major*	246	94	339
LmjF35.0920	*L. major*	61	576	636
LmjF35.0920	*L. major*	41	696	736
LmjF35.0920	*L. major*	116	944	1059
LmjF35.0920	*L. major*	139	1207	1345
LmjF35.0930	*L. major*	136	1	136
LmjF35.0940	*L. major*	55	44	98
LmjF35.0950	*L. major*	141	74	214
LmjF35.0960	*L. major*	166	102	267
LmjF35.0960	*L. major*	40	278	317
LmjF35.0960	*L. major*	44	495	538
LmjF35.0960	*L. major*	78	567	644
LmjF35.0960	*L. major*	62	683	744

LmjF35.0990	*L. major*	86	42	127
LmjF35.0990	*L. major*	82	261	342
LmjF35.0990	*L. major*	46	388	433
LmjF35.0990	*L. major*	88	456	543
LmjF35.0990	*L. major*	270	588	857
LmjF35.1010	*L. major*	51	303	353
LmjF35.1020	*L. major*	80	60	139
LmjF35.1020	*L. major*	213	171	383
LmjF35.1040	*L. major*	40	1	40
LmjF35.1040	*L. major*	123	67	189
LmjF35.1040	*L. major*	88	230	317
LmjF35.1050	*L. major*	152	338	489
LmjF35.1060	*L. major*	343	1	343
LmjF35.1060	*L. major*	78	399	476
LmjF35.1060	*L. major*	70	479	548
LmjF35.1080	*L. major*	91	229	319
LmjF35.1090	*L. major*	656	1	656
LmjF35.1090	*L. major*	111	689	799
LmjF35.1090	*L. major*	71	801	871
LmjF35.1090	*L. major*	136	914	1049
LmjF35.1100	*L. major*	553	1	553
LmjF35.1100	*L. major*	108	574	681
LmjF35.1110	*L. major*	73	1	73
LmjF35.1110	*L. major*	41	130	170
LmjF35.1130	*L. major*	44	1	44
LmjF35.1130	*L. major*	45	509	553
LmjF35.1130	*L. major*	45	509	553
LmjF35.1160	*L. major*	41	1	41
LmjF35.1170	*L. major*	46	1	46
LmjF35.1170	*L. major*	152	68	219
LmjF35.1170	*L. major*	103	505	607
LmjF35.1240	*L. major*	163	1	163
LmjF35.1240	*L. major*	148	233	380
LmjF35.1240	*L. major*	149	410	558
LmjF35.1240	*L. major*	146	702	847
LmjF35.1260	*L. major*	48	314	361
LmjF35.1270	*L. major*	63	38	100
LmjF35.1270	*L. major*	99	200	298
LmjF35.1290	*L. major*	106	46	151
LmjF35.1290	*L. major*	57	193	249
LmjF35.1310	*L. major*	49	1	49
LmjF35.1320	*L. major*	281	73	353
LmjF35.1320	*L. major*	120	481	600
LmjF35.1320	*L. major*	117	669	785
LmjF35.1320	*L. major*	102	861	962
LmjF35.1320	*L. major*	69	970	1038
LmjF35.1320	*L. major*	124	1071	1194

LmjF35.1320	*L. major*	183	1288	1470
LmjF35.1320	*L. major*	50	1542	1591
LmjF35.1320	*L. major*	69	1623	1691
LmjF35.1350	*L. major*	63	159	221
LmjF35.1355	*L. major*	243	1	243
LmjF35.1355	*L. major*	49	281	329
LmjF35.1360	*L. major*	149	17	165
LmjF35.1360	*L. major*	58	245	302
LmjF35.1360	*L. major*	107	343	449
LmjF35.1390	*L. major*	52	1	52
LmjF35.1390	*L. major*	67	115	181
LmjF35.1390	*L. major*	50	315	364
LmjF35.1390	*L. major*	121	560	680
LmjF35.1400	*L. major*	113	120	232
LmjF35.1410	*L. major*	44	497	540
LmjF35.1410	*L. major*	45	595	639
LmjF35.1410	*L. major*	45	595	639
LmjF35.1420	*L. major*	325	1	325
LmjF35.1430	*L. major*	63	198	260
LmjF35.1440	*L. major*	63	198	260
LmjF35.1450	*L. major*	56	1	56
LmjF35.1450	*L. major*	113	224	336
LmjF35.1450	*L. major*	97	389	485
LmjF35.1450	*L. major*	52	539	590
LmjF35.1450	*L. major*	69	594	662
LmjF35.1450	*L. major*	49	736	784
LmjF35.1450	*L. major*	71	841	911
LmjF35.1450	*L. major*	40	950	989
LmjF35.1450	*L. major*	40	1138	1177
LmjF35.1450	*L. major*	47	1446	1492
LmjF35.1450	*L. major*	203	1706	1908
LmjF35.1450	*L. major*	101	2035	2135
LmjF35.1450	*L. major*	79	2164	2242
LmjF35.1450	*L. major*	149	2311	2459
LmjF35.1450	*L. major*	162	2511	2672
LmjF35.1450	*L. major*	280	2755	3034
LmjF35.1450	*L. major*	105	3108	3212
LmjF35.1470	*L. major*	80	1	80
LmjF35.1510	*L. major*	74	212	285
LmjF35.1510	*L. major*	291	293	583
LmjF35.1520	*L. major*	86	214	299
LmjF35.1520	*L. major*	46	479	524
LmjF35.1520	*L. major*	46	479	524
LmjF35.1530	*L. major*	87	27	113
LmjF35.1530	*L. major*	73	165	237
LmjF35.1530	*L. major*	73	165	237
LmjF35.1540	*L. major*	41	24	64

LmjF35.1550	*L. major*	161	39	199
LmjF35.1550	*L. major*	64	483	546
LmjF35.1550	*L. major*	64	483	546
LmjF35.1560	*L. major*	45	177	221
LmjF35.1560	*L. major*	153	334	486
LmjF35.1560	*L. major*	65	853	917
LmjF35.1560	*L. major*	45	1020	1064
LmjF35.1570	*L. major*	40	1	40
LmjF35.1580	*L. major*	106	330	435
LmjF35.1590	*L. major*	41	93	133
LmjF35.1600	*L. major*	45	368	412
LmjF35.1610	*L. major*	47	143	189
LmjF35.1610	*L. major*	50	892	941
LmjF35.1610	*L. major*	50	892	941
LmjF35.1620	*L. major*	83	247	329
LmjF35.1620	*L. major*	58	434	491
LmjF35.1620	*L. major*	40	863	902
LmjF35.1620	*L. major*	41	1053	1093
LmjF35.1620	*L. major*	50	1280	1329
LmjF35.1620	*L. major*	61	1891	1951
LmjF35.1630	*L. major*	156	139	294
LmjF35.1630	*L. major*	156	303	458
LmjF35.1640	*L. major*	76	90	165
LmjF35.1650	*L. major*	62	32	93
LmjF35.1650	*L. major*	419	115	533
LmjF35.1650	*L. major*	97	543	639
LmjF35.1660	*L. major*	84	77	160
LmjF35.1660	*L. major*	248	388	635
LmjF35.1670	*L. major*	40	1	40
LmjF35.1670	*L. major*	41	103	143
LmjF35.1670	*L. major*	41	103	143
LmjF35.1680	*L. major*	76	203	278
LmjF35.1690	*L. major*	205	174	378
LmjF35.1720	*L. major*	90	30	119
LmjF35.1740	*L. major*	62	685	746
LmjF35.1760	*L. major*	54	115	168
LmjF35.1770	*L. major*	93	98	190
LmjF35.1800	*L. major*	45	211	255
LmjF35.1810	*L. major*	230	64	293
LmjF35.1810	*L. major*	41	373	413
LmjF35.1810	*L. major*	41	373	413
LmjF35.1820	*L. major*	123	1	123
LmjF35.1820	*L. major*	114	160	273
LmjF35.1860	*L. major*	184	1	184
LmjF35.1870	*L. major*	162	149	310
LmjF35.1870	*L. major*	41	326	366
LmjF35.1870	*L. major*	45	410	454

LmjF35.1870	*L. major*	54	538	591
LmjF35.1870	*L. major*	49	617	665
LmjF35.1880	*L. major*	69	237	305
LmjF35.1890	*L. major*	69	260	328
LmjF35.1900	*L. major*	69	237	305
LmjF35.1910	*L. major*	51	35	85
LmjF35.1910	*L. major*	47	154	200
LmjF35.1910	*L. major*	47	154	200
LmjF35.1930	*L. major*	48	45	92
LmjF35.1940	*L. major*	75	89	163
LmjF35.1940	*L. major*	77	226	302
LmjF35.1945	*L. major*	40	31	70
LmjF35.1945	*L. major*	43	124	166
LmjF35.1945	*L. major*	393	228	620
LmjF35.1970	*L. major*	118	136	253
LmjF35.1980	*L. major*	42	280	321
LmjF35.1980	*L. major*	50	357	406
LmjF35.1980	*L. major*	163	427	589
LmjF35.1990	*L. major*	55	199	253
LmjF35.1990	*L. major*	43	391	433
LmjF35.1990	*L. major*	50	480	529
LmjF35.1990	*L. major*	77	659	735
LmjF35.1990	*L. major*	64	767	830
LmjF35.2000	*L. major*	71	554	624
LmjF35.2000	*L. major*	42	730	771
LmjF35.2000	*L. major*	43	796	838
LmjF35.2010	*L. major*	120	130	249
LmjF35.2030	*L. major*	126	133	258
LmjF35.2030	*L. major*	76	305	380
LmjF35.2030	*L. major*	59	433	491
LmjF35.2030	*L. major*	52	936	987
LmjF35.2030	*L. major*	46	1045	1090
LmjF35.2030	*L. major*	47	1146	1192
LmjF35.2030	*L. major*	94	1242	1335
LmjF35.2030	*L. major*	49	1348	1396
LmjF35.2040	*L. major*	89	147	235
LmjF35.2040	*L. major*	97	618	714
LmjF35.2040	*L. major*	67	1844	1910
LmjF35.2040	*L. major*	46	2450	2495
LmjF35.2040	*L. major*	48	2592	2639
LmjF35.2040	*L. major*	45	2687	2731
LmjF35.2040	*L. major*	65	3095	3159
LmjF35.2040	*L. major*	106	3668	3773
LmjF35.2050	*L. major*	65	1	65
LmjF35.2060	*L. major*	54	1	54
LmjF35.2060	*L. major*	48	217	264
LmjF35.2060	*L. major*	89	394	482

LmjF35.2070	*L. major*	42	47	88
LmjF35.2070	*L. major*	237	94	330
LmjF35.2075	*L. major*	55	48	102
LmjF35.2080	*L. major*	77	1	77
LmjF35.2080	*L. major*	75	1035	1109
LmjF35.2080	*L. major*	75	1035	1109
LmjF35.2090	*L. major*	76	218	293
LmjF35.2090	*L. major*	78	475	552
LmjF35.2090	*L. major*	78	475	552
LmjF35.2100	*L. major*	137	111	247
LmjF35.2100	*L. major*	61	385	445
LmjF35.2100	*L. major*	97	627	723
LmjF35.2110	*L. major*	182	97	278
LmjF35.2110	*L. major*	53	280	332
LmjF35.2110	*L. major*	61	340	400
LmjF35.2125	*L. major*	242	1	242
LmjF35.2130	*L. major*	80	68	147
LmjF35.2130	*L. major*	71	257	327
LmjF35.2140	*L. major*	56	32	87
LmjF35.2140	*L. major*	88	388	475
LmjF35.2140	*L. major*	88	388	475
LmjF35.2180	*L. major*	124	1	124
LmjF35.2180	*L. major*	53	292	344
LmjF35.2180	*L. major*	126	372	497
LmjF35.2180	*L. major*	85	624	708
LmjF35.2180	*L. major*	65	812	876
LmjF35.2180	*L. major*	59	1012	1070
LmjF35.2200	*L. major*	104	92	195
LmjF35.2240	*L. major*	81	1	81
LmjF35.2240	*L. major*	78	133	210
LmjF35.2250	*L. major*	91	56	146
LmjF35.2250	*L. major*	137	180	316
LmjF35.2250	*L. major*	40	355	394
LmjF35.2250	*L. major*	64	451	514
LmjF35.2250	*L. major*	44	795	838
LmjF35.2250	*L. major*	153	888	1040
LmjF35.2250	*L. major*	133	1201	1333
LmjF35.2250	*L. major*	52	1436	1487
LmjF35.2250	*L. major*	42	1517	1558
LmjF35.2250	*L. major*	40	1746	1785
LmjF35.2250	*L. major*	65	1843	1907
LmjF35.2250	*L. major*	86	1994	2079
LmjF35.2250	*L. major*	188	2110	2297
LmjF35.2250	*L. major*	103	2386	2488
LmjF35.2250	*L. major*	150	2538	2687
LmjF35.2270	*L. major*	118	1	118
LmjF35.2270	*L. major*	64	602	665

LmjF35.2270	*L. major*	65	1086	1150
LmjF35.2280	*L. major*	61	1	61
LmjF35.2290	*L. major*	111	60	170
LmjF35.2290	*L. major*	74	221	294
LmjF35.2290	*L. major*	71	382	452
LmjF35.2290	*L. major*	41	557	597
LmjF35.2290	*L. major*	52	686	737
LmjF35.2290	*L. major*	158	846	1003
LmjF35.2290	*L. major*	68	1023	1090
LmjF35.2290	*L. major*	124	1226	1349
LmjF35.2300	*L. major*	111	1	111
LmjF35.2300	*L. major*	51	356	406
LmjF35.2310	*L. major*	72	1	72
LmjF35.2310	*L. major*	80	106	185
LmjF35.2310	*L. major*	80	106	185
LmjF35.2320	*L. major*	112	1	112
LmjF35.2320	*L. major*	77	142	218
LmjF35.2320	*L. major*	46	453	498
LmjF35.2320	*L. major*	114	597	710
LmjF35.2320	*L. major*	145	800	944
LmjF35.2320	*L. major*	169	958	1126
LmjF35.2330	*L. major*	40	342	381
LmjF35.2360	*L. major*	187	1	187
LmjF35.2370	*L. major*	92	176	267
LmjF35.2370	*L. major*	60	312	371
LmjF35.2370	*L. major*	108	416	523
LmjF35.2370	*L. major*	108	535	642
LmjF35.2380	*L. major*	60	65	124
LmjF35.2380	*L. major*	62	391	452
LmjF35.2380	*L. major*	41	519	559
LmjF35.2380	*L. major*	50	725	774
LmjF35.2380	*L. major*	292	980	1271
LmjF35.2390	*L. major*	56	97	152
LmjF35.2400	*L. major*	45	174	218
LmjF35.2400	*L. major*	52	785	836
LmjF35.2400	*L. major*	52	785	836
LmjF35.2410	*L. major*	448	1	448
LmjF35.2420	*L. major*	52	152	203
LmjF35.2420	*L. major*	52	321	372
LmjF35.2420	*L. major*	40	378	417
LmjF35.2430	*L. major*	49	26	74
LmjF35.2430	*L. major*	49	133	181
LmjF35.2430	*L. major*	130	210	339
LmjF35.2430	*L. major*	65	355	419
LmjF35.2430	*L. major*	291	467	757
LmjF35.2450	*L. major*	59	184	242
LmjF35.2450	*L. major*	59	539	597

LmjF35.2450	*L. major*	64	1026	1089
LmjF35.2450	*L. major*	41	1092	1132
LmjF35.2450	*L. major*	125	1688	1812
LmjF35.2450	*L. major*	60	1834	1893
LmjF35.2450	*L. major*	79	2183	2261
LmjF35.2470	*L. major*	220	91	310
LmjF35.2470	*L. major*	91	405	495
LmjF35.2480	*L. major*	222	30	251
LmjF35.2480	*L. major*	49	291	339
LmjF35.2490	*L. major*	125	8	132
LmjF35.2490	*L. major*	286	842	1127
LmjF35.2490	*L. major*	273	1202	1474
LmjF35.2490	*L. major*	164	1493	1656
LmjF35.2500	*L. major*	66	181	246
LmjF35.2520	*L. major*	56	140	195
LmjF35.2520	*L. major*	88	229	316
LmjF35.2520	*L. major*	88	229	316
LmjF35.2530	*L. major*	150	1	150
LmjF35.2530	*L. major*	45	294	338
LmjF35.2550	*L. major*	160	1	160
LmjF35.2550	*L. major*	113	343	455
LmjF35.2560	*L. major*	43	33	75
LmjF35.2560	*L. major*	124	293	416
LmjF35.2570	*L. major*	148	70	217
LmjF35.2580	*L. major*	89	1	89
LmjF35.2580	*L. major*	70	122	191
LmjF35.2580	*L. major*	70	122	191
LmjF35.2610	*L. major*	201	22	222
LmjF35.2620	*L. major*	54	1	54
LmjF35.2620	*L. major*	100	166	265
LmjF35.2620	*L. major*	131	271	401
LmjF35.2630	*L. major*	85	90	174
LmjF35.2630	*L. major*	43	207	249
LmjF35.2630	*L. major*	150	395	544
LmjF35.2630	*L. major*	48	546	593
LmjF35.2660	*L. major*	63	105	167
LmjF35.2660	*L. major*	436	179	614
LmjF35.2690	*L. major*	138	1	138
LmjF35.2700	*L. major*	46	343	388
LmjF35.2700	*L. major*	71	468	538
LmjF35.2700	*L. major*	49	1054	1102
LmjF35.2700	*L. major*	93	1148	1240
LmjF35.2710	*L. major*	61	39	99
LmjF35.2710	*L. major*	66	166	231
LmjF35.2720	*L. major*	58	53	110
LmjF35.2720	*L. major*	60	155	214
LmjF35.2720	*L. major*	60	155	214

LmjF35.2725	*L. major*	153	1	153
LmjF35.2725	*L. major*	101	211	311
LmjF35.2725	*L. major*	55	340	394
LmjF35.2750	*L. major*	89	1	89
LmjF35.2750	*L. major*	226	95	320
LmjF35.2750	*L. major*	53	377	429
LmjF35.2760	*L. major*	43	1	43
LmjF35.2760	*L. major*	139	265	403
LmjF35.2760	*L. major*	59	562	620
LmjF35.2770	*L. major*	48	42	89
LmjF35.2770	*L. major*	62	323	384
LmjF35.2770	*L. major*	42	426	467
LmjF35.2770	*L. major*	67	657	723
LmjF35.2770	*L. major*	120	884	1003
LmjF35.2770	*L. major*	63	1117	1179
LmjF35.2780	*L. major*	46	525	570
LmjF35.2780	*L. major*	91	608	698
LmjF35.2810	*L. major*	40	269	308
LmjF35.2810	*L. major*	62	397	458
LmjF35.2810	*L. major*	62	397	458
LmjF35.2820	*L. major*	135	291	425
LmjF35.2830	*L. major*	88	413	500
LmjF35.2830	*L. major*	49	532	580
LmjF35.2830	*L. major*	49	532	580
LmjF35.2850	*L. major*	204	277	480
LmjF35.2860	*L. major*	76	1	76
LmjF35.2860	*L. major*	53	99	151
LmjF35.2870	*L. major*	52	121	172
LmjF35.2870	*L. major*	95	383	477
LmjF35.2870	*L. major*	102	516	617
LmjF35.2890	*L. major*	61	1	61
LmjF35.2890	*L. major*	86	284	369
LmjF35.2890	*L. major*	42	371	412
LmjF35.2890	*L. major*	104	466	569
LmjF35.2910	*L. major*	65	1	65
LmjF35.2920	*L. major*	52	39	90
LmjF35.2920	*L. major*	54	125	178
LmjF35.2920	*L. major*	156	350	505
LmjF35.2920	*L. major*	60	616	675
LmjF35.2940	*L. major*	76	1	76
LmjF35.2950	*L. major*	49	19	67
LmjF35.2950	*L. major*	65	986	1050
LmjF35.2950	*L. major*	65	986	1050
LmjF35.2960	*L. major*	46	514	559
LmjF35.2970	*L. major*	84	140	223
LmjF35.2970	*L. major*	50	332	381
LmjF35.2980	*L. major*	256	1	256

LmjF35.2980	*L. major*	86	393	478
LmjF35.2990	*L. major*	54	15	68
LmjF35.2990	*L. major*	52	361	412
LmjF35.3000	*L. major*	45	50	94
LmjF35.3050	*L. major*	120	78	197
LmjF35.3050	*L. major*	69	273	341
LmjF35.3050	*L. major*	61	350	410
LmjF35.3060	*L. major*	61	344	404
LmjF35.3060	*L. major*	53	861	913
LmjF35.3060	*L. major*	53	861	913
LmjF35.3070	*L. major*	55	1	55
LmjF35.3090	*L. major*	72	70	141
LmjF35.3090	*L. major*	99	182	280
LmjF35.3090	*L. major*	99	182	280
LmjF35.3100	*L. major*	430	1	430
LmjF35.3100	*L. major*	70	854	923
LmjF35.3120	*L. major*	81	32	112
LmjF35.3120	*L. major*	41	394	434
LmjF35.3120	*L. major*	150	762	911
LmjF35.3120	*L. major*	247	1073	1319
LmjF35.3120	*L. major*	80	1372	1451
LmjF35.3120	*L. major*	148	1477	1624
LmjF35.3130	*L. major*	58	1	58
LmjF35.3150	*L. major*	64	38	101
LmjF35.3150	*L. major*	82	121	202
LmjF35.3150	*L. major*	102	339	440
LmjF35.3170	*L. major*	51	1	51
LmjF35.3170	*L. major*	539	59	597
LmjF35.3170	*L. major*	89	782	870
LmjF35.3170	*L. major*	187	1138	1324
LmjF35.3170	*L. major*	214	1367	1580
LmjF35.3170	*L. major*	41	1730	1770
LmjF35.3180	*L. major*	47	1	47
LmjF35.3180	*L. major*	40	54	93
LmjF35.3180	*L. major*	68	125	192
LmjF35.3190	*L. major*	54	1	54
LmjF35.3190	*L. major*	64	161	224
LmjF35.3190	*L. major*	233	299	531
LmjF35.3190	*L. major*	42	561	602
LmjF35.3200	*L. major*	56	118	173
LmjF35.3200	*L. major*	319	384	702
LmjF35.3210	*L. major*	212	35	246
LmjF35.3220	*L. major*	42	1	42
LmjF35.3240	*L. major*	115	1	115
LmjF35.3240	*L. major*	621	180	800
LmjF35.3260	*L. major*	207	1	207
LmjF35.3260	*L. major*	48	227	274

LmjF35.3270	*L. major*	171	19	189
LmjF35.3280	*L. major*	104	1	104
LmjF35.3290	*L. major*	104	1	104
LmjF35.3300	*L. major*	41	348	388
LmjF35.3300	*L. major*	55	456	510
LmjF35.3300	*L. major*	47	556	602
LmjF35.3310	*L. major*	137	141	277
LmjF35.3350	*L. major*	61	1	61
LmjF35.3350	*L. major*	108	354	461
LmjF35.3360	*L. major*	52	1	52
LmjF35.3360	*L. major*	41	167	207
LmjF35.3360	*L. major*	48	604	651
LmjF35.3360	*L. major*	55	997	1051
LmjF35.3360	*L. major*	56	1206	1261
LmjF35.3360	*L. major*	48	1713	1760
LmjF35.3360	*L. major*	60	2015	2074
LmjF35.3370	*L. major*	130	1	130
LmjF35.3370	*L. major*	51	1015	1065
LmjF35.3380	*L. major*	97	442	538
LmjF35.3390	*L. major*	64	152	215
LmjF35.3400	*L. major*	44	252	295
LmjF35.3410	*L. major*	60	1	60
LmjF35.3410	*L. major*	46	96	141
LmjF35.3410	*L. major*	51	358	408
LmjF35.3420	*L. major*	122	1	122
LmjF35.3430	*L. major*	44	18	61
LmjF35.3430	*L. major*	41	165	205
LmjF35.3430	*L. major*	46	217	262
LmjF35.3430	*L. major*	60	390	449
LmjF35.3430	*L. major*	57	490	546
LmjF35.3450	*L. major*	162	1	162
LmjF35.3450	*L. major*	96	378	473
LmjF35.3460	*L. major*	99	206	304
LmjF35.3470	*L. major*	43	490	532
LmjF35.3480	*L. major*	98	139	236
LmjF35.3480	*L. major*	313	418	730
LmjF35.3490	*L. major*	225	202	426
LmjF35.3500	*L. major*	94	1	94
LmjF35.3500	*L. major*	64	122	185
LmjF35.3510	*L. major*	57	85	141
LmjF35.3510	*L. major*	357	192	548
LmjF35.3510	*L. major*	41	702	742
LmjF35.3510	*L. major*	91	864	954
LmjF35.3510	*L. major*	184	982	1165
LmjF35.3520	*L. major*	242	1	242
LmjF35.3530	*L. major*	44	53	96
LmjF35.3550	*L. major*	341	1	341

LmjF35.3550	*L. major*	369	381	749
LmjF35.3560	*L. major*	55	318	372
LmjF35.3570	*L. major*	145	1	145
LmjF35.3570	*L. major*	73	792	864
LmjF35.3570	*L. major*	94	986	1079
LmjF35.3580	*L. major*	225	480	704
LmjF35.3590	*L. major*	150	78	227
LmjF35.3590	*L. major*	330	299	628
LmjF35.3590	*L. major*	59	873	931
LmjF35.3600	*L. major*	58	1	58
LmjF35.3630	*L. major*	42	27	68
LmjF35.3630	*L. major*	46	565	610
LmjF35.3630	*L. major*	46	565	610
LmjF35.3640	*L. major*	179	23	201
LmjF35.3650	*L. major*	46	17	62
LmjF35.3650	*L. major*	445	130	574
LmjF35.3650	*L. major*	58	604	661
LmjF35.3650	*L. major*	50	728	777
LmjF35.3660	*L. major*	49	1	49
LmjF35.3660	*L. major*	40	125	164
LmjF35.3660	*L. major*	59	717	775
LmjF35.3660	*L. major*	101	812	912
LmjF35.3660	*L. major*	49	1251	1299
LmjF35.3710	*L. major*	119	1	119
LmjF35.3720	*L. major*	112	1	112
LmjF35.3720	*L. major*	147	173	319
LmjF35.3720	*L. major*	88	353	440
LmjF35.3730	*L. major*	42	168	209
LmjF35.3750	*L. major*	49	174	222
LmjF35.3750	*L. major*	64	277	340
LmjF35.3750	*L. major*	45	474	518
LmjF35.3750	*L. major*	137	544	680
LmjF35.3750	*L. major*	48	786	833
LmjF35.3760	*L. major*	47	1	47
LmjF35.3770	*L. major*	73	230	302
LmjF35.3780	*L. major*	47	1	47
LmjF35.3820	*L. major*	61	1	61
LmjF35.3820	*L. major*	61	75	135
LmjF35.3820	*L. major*	114	163	276
LmjF35.3820	*L. major*	40	281	320
LmjF35.3850	*L. major*	88	225	312
LmjF35.3860	*L. major*	49	522	570
LmjF35.3870	*L. major*	40	152	191
LmjF35.3880	*L. major*	84	361	444
LmjF35.3900	*L. major*	73	1	73
LmjF35.3920	*L. major*	43	575	617
LmjF35.3925	*L. major*	388	43	430

LmjF35.3940	*L. major*	78	1	78
LmjF35.3950	*L. major*	40	1	40
LmjF35.3950	*L. major*	52	492	543
LmjF35.3950	*L. major*	53	1884	1936
LmjF35.3950	*L. major*	47	2076	2122
LmjF35.3970	*L. major*	218	83	300
LmjF35.3970	*L. major*	122	583	704
LmjF35.3980	*L. major*	202	30	231
LmjF35.3980	*L. major*	562	235	796
LmjF35.4000	*L. major*	68	143	210
LmjF35.4000	*L. major*	61	240	300
LmjF35.4000	*L. major*	101	333	433
LmjF35.4000	*L. major*	113	467	579
LmjF35.4010	*L. major*	42	1	42
LmjF35.4020	*L. major*	64	88	151
LmjF35.4030	*L. major*	80	406	485
LmjF35.4030	*L. major*	118	573	690
LmjF35.4030	*L. major*	118	573	690
LmjF35.4040	*L. major*	45	34	78
LmjF35.4040	*L. major*	200	111	310
LmjF35.4050	*L. major*	43	238	280
LmjF35.4050	*L. major*	383	362	744
LmjF35.4050	*L. major*	160	913	1072
LmjF35.4070	*L. major*	64	139	202
LmjF35.4070	*L. major*	51	288	338
LmjF35.4080	*L. major*	71	42	112
LmjF35.4080	*L. major*	271	137	407
LmjF35.4080	*L. major*	138	434	571
LmjF35.4080	*L. major*	392	673	1064
LmjF35.4100	*L. major*	50	1	50
LmjF35.4100	*L. major*	52	211	262
LmjF35.4100	*L. major*	52	211	262
LmjF35.4110	*L. major*	80	57	136
LmjF35.4120	*L. major*	89	78	166
LmjF35.4120	*L. major*	64	213	276
LmjF35.4120	*L. major*	119	411	529
LmjF35.4120	*L. major*	149	595	743
LmjF35.4120	*L. major*	57	757	813
LmjF35.4125	*L. major*	588	1	588
LmjF35.4130	*L. major*	66	237	302
LmjF35.4130	*L. major*	169	365	533
LmjF35.4140	*L. major*	45	164	208
LmjF35.4150	*L. major*	85	1	85
LmjF35.4150	*L. major*	102	131	232
LmjF35.4170	*L. major*	195	40	234
LmjF35.4190	*L. major*	78	1	78
LmjF35.4200	*L. major*	53	342	394

LmjF35.4200	*L. major*	107	427	533
LmjF35.4200	*L. major*	107	427	533
LmjF35.4210	*L. major*	578	1	578
LmjF35.4210	*L. major*	74	628	701
LmjF35.4220	*L. major*	40	17	56
LmjF35.4220	*L. major*	335	70	404
LmjF35.4220	*L. major*	174	429	602
LmjF35.4230	*L. major*	82	144	225
LmjF35.4230	*L. major*	55	309	363
LmjF35.4230	*L. major*	61	401	461
LmjF35.4230	*L. major*	74	614	687
LmjF35.4230	*L. major*	59	718	776
LmjF35.4240	*L. major*	66	142	207
LmjF35.4250	*L. major*	45	277	321
LmjF35.4260	*L. major*	46	1	46
LmjF35.4260	*L. major*	213	65	277
LmjF35.4260	*L. major*	41	291	331
LmjF35.4270	*L. major*	72	98	169
LmjF35.4280	*L. major*	167	1	167
LmjF35.4280	*L. major*	212	304	515
LmjF35.4280	*L. major*	82	518	599
LmjF35.4280	*L. major*	97	687	783
LmjF35.4290	*L. major*	59	65	123
LmjF35.4290	*L. major*	112	166	277
LmjF35.4300	*L. major*	57	22	78
LmjF35.4300	*L. major*	45	188	232
LmjF35.4320	*L. major*	56	37	92
LmjF35.4320	*L. major*	59	124	182
LmjF35.4330	*L. major*	83	1	83
LmjF35.4330	*L. major*	40	119	158
LmjF35.4330	*L. major*	85	211	295
LmjF35.4330	*L. major*	120	418	537
LmjF35.4330	*L. major*	63	550	612
LmjF35.4360	*L. major*	101	679	779
LmjF35.4360	*L. major*	101	1265	1365
LmjF35.4360	*L. major*	41	2083	2123
LmjF35.4370	*L. major*	51	64	114
LmjF35.4380	*L. major*	470	26	495
LmjF35.4400	*L. major*	58	10	67
LmjF35.4400	*L. major*	47	85	131
LmjF35.4400	*L. major*	73	134	206
LmjF35.4440	*L. major*	42	1	42
LmjF35.4440	*L. major*	64	44	107
LmjF35.4440	*L. major*	64	44	107
LmjF35.4450	*L. major*	55	1	55
LmjF35.4450	*L. major*	41	59	99
LmjF35.4450	*L. major*	158	107	264

LmjF35.4470	*L. major*	88	112	199
LmjF35.4490	*L. major*	507	1	507
LmjF35.4490	*L. major*	40	644	683
LmjF35.4490	*L. major*	114	760	873
LmjF35.4490	*L. major*	357	1131	1487
LmjF35.4510	*L. major*	69	1	69
LmjF35.4520	*L. major*	48	216	263
LmjF35.4530	*L. major*	60	254	313
LmjF35.4530	*L. major*	64	531	594
LmjF35.4530	*L. major*	64	531	594
LmjF35.4540	*L. major*	47	1	47
LmjF35.4540	*L. major*	71	420	490
LmjF35.4540	*L. major*	71	420	490
LmjF35.4550	*L. major*	43	77	119
LmjF35.4560	*L. major*	213	99	311
LmjF35.4580	*L. major*	147	386	532
LmjF35.4600	*L. major*	88	85	172
LmjF35.4600	*L. major*	310	202	511
LmjF35.4620	*L. major*	44	245	288
LmjF35.4640	*L. major*	114	1	114
LmjF35.4650	*L. major*	66	30	95
LmjF35.4650	*L. major*	57	105	161
LmjF35.4670	*L. major*	139	293	431
LmjF35.4690	*L. major*	78	1	78
LmjF35.4690	*L. major*	58	85	142
LmjF35.4700	*L. major*	45	209	253
LmjF35.4710	*L. major*	53	671	723
LmjF35.4770	*L. major*	51	304	354
LmjF35.4790	*L. major*	41	1	41
LmjF35.4800	*L. major*	66	1	66
LmjF35.4800	*L. major*	151	1323	1473
LmjF35.4830	*L. major*	42	332	373
LmjF35.4860	*L. major*	66	1	66
LmjF35.4870	*L. major*	901	43	943
LmjF35.4890	*L. major*	43	1	43
LmjF35.4900	*L. major*	66	1	66
LmjF35.4910	*L. major*	40	274	313
LmjF35.4910	*L. major*	156	753	908
LmjF35.4930	*L. major*	64	1	64
LmjF35.4930	*L. major*	74	100	173
LmjF35.4940	*L. major*	184	27	210
LmjF35.4950	*L. major*	57	1	57
LmjF35.4950	*L. major*	596	176	771
LmjF35.4950	*L. major*	219	811	1029
LmjF35.4960	*L. major*	67	1	67
LmjF35.4960	*L. major*	46	105	150
LmjF35.4960	*L. major*	46	105	150

LmjF35.5000	*L. major*	90	1	90
LmjF35.5010	*L. major*	114	270	383
LmjF35.5020	*L. major*	45	676	720
LmjF35.5020	*L. major*	63	834	896
LmjF35.5020	*L. major*	63	834	896
LmjF35.5030	*L. major*	217	53	269
LmjF35.5030	*L. major*	91	305	395
LmjF35.5040	*L. major*	125	381	505
LmjF35.5050	*L. major*	256	61	316
LmjF35.5050	*L. major*	286	363	648
LmjF35.5060	*L. major*	140	62	201
LmjF35.5060	*L. major*	81	254	334
LmjF35.5080	*L. major*	42	94	135
LmjF35.5090	*L. major*	54	1	54
LmjF35.5110	*L. major*	42	62	103
LmjF35.5110	*L. major*	60	221	280
LmjF35.5110	*L. major*	60	221	280
LmjF35.5120	*L. major*	45	555	599
LmjF35.5130	*L. major*	103	36	138
LmjF35.5130	*L. major*	41	266	306
LmjF35.5130	*L. major*	45	326	370
LmjF35.5150	*L. major*	46	258	303
LmjF35.5170	*L. major*	51	1	51
LmjF35.5180	*L. major*	55	269	323
LmjF35.5200	*L. major*	104	154	257
LmjF35.5210	*L. major*	64	401	464
LmjF35.5220	*L. major*	67	81	147
LmjF35.5220	*L. major*	170	149	318
LmjF35.5220	*L. major*	170	149	318
LmjF35.5270	*L. major*	41	109	149
LmjF35.5290	*L. major*	48	65	112
LmjF35.5300	*L. major*	40	171	210
LmjF35.5310	*L. major*	61	1	61
LmjF35.5320	*L. major*	51	89	139
LmjF35.5320	*L. major*	83	174	256
LmjF35.5320	*L. major*	83	174	256
LmjF35.5340	*L. major*	42	163	204
LmjF35.5390	*L. major*	87	1	87
LmjF35.5390	*L. major*	67	1463	1529
LmjF35.5390	*L. major*	64	2455	2518
LmjF35.5390	*L. major*	70	5084	5153
LmjF35.5390	*L. major*	40	5941	5980
LmjF36.0010	*L. major*	87	39	125
LmjF36.0010	*L. major*	44	175	218
LmjF36.0020	*L. major*	49	1	49
LmjF36.0050	*L. major*	57	1	57
LmjF36.0050	*L. major*	125	429	553

LmjF36.0050	*L. major*	125	429	553
LmjF36.0065	*L. major*	64	12	75
LmjF36.0065	*L. major*	173	111	283
LmjF36.0070	*L. major*	49	102	150
LmjF36.0100	*L. major*	62	477	538
LmjF36.0110	*L. major*	52	234	285
LmjF36.0110	*L. major*	41	354	394
LmjF36.0110	*L. major*	41	515	555
LmjF36.0130	*L. major*	42	1	42
LmjF36.0130	*L. major*	159	98	256
LmjF36.0140	*L. major*	71	580	650
LmjF36.0160	*L. major*	48	238	285
LmjF36.0170	*L. major*	41	178	218
LmjF36.0240	*L. major*	83	1	83
LmjF36.0240	*L. major*	107	118	224
LmjF36.0240	*L. major*	107	118	224
LmjF36.0250	*L. major*	49	400	448
LmjF36.0250	*L. major*	86	548	633
LmjF36.0270	*L. major*	115	91	205
LmjF36.0270	*L. major*	50	216	265
LmjF36.0270	*L. major*	105	581	685
LmjF36.0280	*L. major*	41	27	67
LmjF36.0280	*L. major*	55	124	178
LmjF36.0280	*L. major*	61	192	252
LmjF36.0300	*L. major*	40	120	159
LmjF36.0350	*L. major*	53	1	53
LmjF36.0350	*L. major*	125	82	206
LmjF36.0350	*L. major*	47	330	376
LmjF36.0360	*L. major*	45	69	113
LmjF36.0370	*L. major*	81	1	81
LmjF36.0370	*L. major*	50	225	274
LmjF36.0370	*L. major*	49	326	374
LmjF36.0380	*L. major*	129	56	184
LmjF36.0390	*L. major*	114	1	114
LmjF36.0390	*L. major*	97	286	382
LmjF36.0400	*L. major*	65	42	106
LmjF36.0400	*L. major*	71	142	212
LmjF36.0400	*L. major*	98	280	377
LmjF36.0410	*L. major*	45	1	45
LmjF36.0430	*L. major*	62	19	80
LmjF36.0430	*L. major*	59	162	220
LmjF36.0430	*L. major*	59	162	220
LmjF36.0450	*L. major*	95	277	371
LmjF36.0450	*L. major*	411	409	819
LmjF36.0470	*L. major*	41	415	455
LmjF36.0480	*L. major*	45	15	59
LmjF36.0490	*L. major*	310	39	348

LmjF36.0500	*L. major*	44	336	379
LmjF36.0500	*L. major*	101	410	510
LmjF36.0500	*L. major*	101	410	510
LmjF36.0510	*L. major*	48	1	48
LmjF36.0510	*L. major*	43	259	301
LmjF36.0510	*L. major*	43	259	301
LmjF36.0520	*L. major*	61	42	102
LmjF36.0535	*L. major*	56	86	141
LmjF36.0540	*L. major*	214	71	284
LmjF36.0560	*L. major*	59	74	132
LmjF36.0560	*L. major*	112	170	281
LmjF36.0560	*L. major*	112	170	281
LmjF36.0570	*L. major*	53	411	463
LmjF36.0570	*L. major*	42	705	746
LmjF36.0570	*L. major*	42	705	746
LmjF36.0580	*L. major*	74	68	141
LmjF36.0600	*L. major*	57	65	121
LmjF36.0610	*L. major*	86	1	86
LmjF36.0610	*L. major*	53	113	165
LmjF36.0610	*L. major*	66	185	250
LmjF36.0620	*L. major*	64	27	90
LmjF36.0660	*L. major*	63	34	96
LmjF36.0680	*L. major*	271	98	368
LmjF36.0680	*L. major*	60	412	471
LmjF36.0680	*L. major*	230	499	728
LmjF36.0680	*L. major*	122	831	952
LmjF36.0690	*L. major*	78	1	78
LmjF36.0700	*L. major*	72	1	72
LmjF36.0700	*L. major*	50	180	229
LmjF36.0710	*L. major*	83	130	212
LmjF36.0710	*L. major*	42	218	259
LmjF36.0710	*L. major*	42	218	259
LmjF36.0720	*L. major*	127	305	431
LmjF36.0730	*L. major*	79	42	120
LmjF36.0730	*L. major*	74	335	408
LmjF36.0730	*L. major*	137	418	554
LmjF36.0740	*L. major*	201	1	201
LmjF36.0740	*L. major*	64	215	278
LmjF36.0740	*L. major*	62	482	543
LmjF36.0750	*L. major*	125	93	217
LmjF36.0750	*L. major*	261	295	555
LmjF36.0750	*L. major*	84	1111	1194
LmjF36.0750	*L. major*	63	1228	1290
LmjF36.0760	*L. major*	233	1	233
LmjF36.0760	*L. major*	75	331	405
LmjF36.0760	*L. major*	76	704	779
LmjF36.0770	*L. major*	61	82	142

LmjF36.0770	*L. major*	64	154	217
LmjF36.0770	*L. major*	194	244	437
LmjF36.0780	*L. major*	127	1	127
LmjF36.0780	*L. major*	206	165	370
LmjF36.0780	*L. major*	60	663	722
LmjF36.0780	*L. major*	54	914	967
LmjF36.0790	*L. major*	134	1	134
LmjF36.0790	*L. major*	206	232	437
LmjF36.0790	*L. major*	120	442	561
LmjF36.0800	*L. major*	54	491	544
LmjF36.0820	*L. major*	61	45	105
LmjF36.0830	*L. major*	46	411	456
LmjF36.0830	*L. major*	154	528	681
LmjF36.0830	*L. major*	56	725	780
LmjF36.0830	*L. major*	100	814	913
LmjF36.0830	*L. major*	67	1049	1115
LmjF36.0830	*L. major*	81	1548	1628
LmjF36.0830	*L. major*	88	1714	1801
LmjF36.0840	*L. major*	59	1	59
LmjF36.0840	*L. major*	69	387	455
LmjF36.0840	*L. major*	306	479	784
LmjF36.0850	*L. major*	280	25	304
LmjF36.0850	*L. major*	270	514	783
LmjF36.0860	*L. major*	74	317	390
LmjF36.0860	*L. major*	103	412	514
LmjF36.0870	*L. major*	76	121	196
LmjF36.0870	*L. major*	40	240	279
LmjF36.0870	*L. major*	45	832	876
LmjF36.0900	*L. major*	102	408	509
LmjF36.0900	*L. major*	96	557	652
LmjF36.0910	*L. major*	85	1	85
LmjF36.0910	*L. major*	79	121	199
LmjF36.0910	*L. major*	199	1080	1278
LmjF36.0920	*L. major*	49	82	130
LmjF36.0920	*L. major*	81	396	476
LmjF36.0920	*L. major*	76	507	582
LmjF36.0930	*L. major*	66	153	218
LmjF36.0940	*L. major*	66	88	153
LmjF36.0950	*L. major*	75	497	571
LmjF36.0950	*L. major*	102	2728	2829
LmjF36.0950	*L. major*	75	2948	3022
LmjF36.0960	*L. major*	66	376	441
LmjF36.0960	*L. major*	82	549	630
LmjF36.0960	*L. major*	165	659	823
LmjF36.0970	*L. major*	63	46	108
LmjF36.0980	*L. major*	77	92	168
LmjF36.0990	*L. major*	77	92	168

LmjF36.1000	*L. major*	50	626	675
LmjF36.1010	*L. major*	46	304	349
LmjF36.1010	*L. major*	46	368	413
LmjF36.1010	*L. major*	41	526	566
LmjF36.1010	*L. major*	40	586	625
LmjF36.1010	*L. major*	70	744	813
LmjF36.1010	*L. major*	45	1160	1204
LmjF36.1010	*L. major*	41	1261	1301
LmjF36.1020	*L. major*	72	87	158
LmjF36.1030	*L. major*	96	1	96
LmjF36.1030	*L. major*	121	107	227
LmjF36.1030	*L. major*	135	302	436
LmjF36.1030	*L. major*	50	470	519
LmjF36.1030	*L. major*	62	526	587
LmjF36.1030	*L. major*	72	792	863
LmjF36.1030	*L. major*	66	1123	1188
LmjF36.1030	*L. major*	42	1605	1646
LmjF36.1040	*L. major*	63	168	230
LmjF36.1040	*L. major*	49	238	286
LmjF36.1040	*L. major*	49	238	286
LmjF36.1050	*L. major*	133	1	133
LmjF36.1050	*L. major*	41	416	456
LmjF36.1050	*L. major*	47	541	587
LmjF36.1060	*L. major*	141	122	262
LmjF36.1060	*L. major*	74	392	465
LmjF36.1060	*L. major*	104	569	672
LmjF36.1060	*L. major*	126	706	831
LmjF36.1060	*L. major*	49	887	935
LmjF36.1070	*L. major*	64	61	124
LmjF36.1080	*L. major*	58	251	308
LmjF36.1100	*L. major*	64	61	124
LmjF36.1110	*L. major*	61	247	307
LmjF36.1110	*L. major*	46	322	367
LmjF36.1110	*L. major*	46	322	367
LmjF36.1120	*L. major*	66	59	124
LmjF36.1150	*L. major*	66	1	66
LmjF36.1150	*L. major*	254	82	335
LmjF36.1150	*L. major*	96	349	444
LmjF36.1150	*L. major*	110	590	699
LmjF36.1150	*L. major*	70	706	775
LmjF36.1150	*L. major*	55	824	878
LmjF36.1160	*L. major*	50	1	50
LmjF36.1170	*L. major*	53	62	114
LmjF36.1170	*L. major*	64	160	223
LmjF36.1170	*L. major*	76	285	360
LmjF36.1210	*L. major*	49	164	212
LmjF36.1220	*L. major*	101	1	101

LmjF36.1220	*L. major*	98	138	235
LmjF36.1220	*L. major*	248	249	496
LmjF36.1230	*L. major*	70	175	244
LmjF36.1240	*L. major*	82	163	244
LmjF36.1260	*L. major*	50	322	371
LmjF36.1265	*L. major*	59	76	134
LmjF36.1280	*L. major*	57	44	100
LmjF36.1280	*L. major*	44	278	321
LmjF36.1280	*L. major*	44	278	321
LmjF36.1290	*L. major*	113	174	286
LmjF36.1300	*L. major*	122	89	210
LmjF36.1300	*L. major*	84	399	482
LmjF36.1310	*L. major*	156	102	257
LmjF36.1320	*L. major*	196	1	196
LmjF36.1320	*L. major*	154	269	422
LmjF36.1330	*L. major*	41	1	41
LmjF36.1330	*L. major*	74	70	143
LmjF36.1330	*L. major*	74	70	143
LmjF36.1350	*L. major*	46	31	76
LmjF36.1350	*L. major*	82	153	234
LmjF36.1350	*L. major*	46	240	285
LmjF36.1370	*L. major*	48	265	312
LmjF36.1370	*L. major*	94	691	784
LmjF36.1370	*L. major*	94	691	784
LmjF36.1380	*L. major*	80	1	80
LmjF36.1390	*L. major*	78	67	144
LmjF36.1390	*L. major*	58	173	230
LmjF36.1390	*L. major*	119	260	378
LmjF36.1390	*L. major*	204	449	652
LmjF36.1390	*L. major*	252	838	1089
LmjF36.1395	*L. major*	47	36	82
LmjF36.1400	*L. major*	55	142	196
LmjF36.1400	*L. major*	245	199	443
LmjF36.1410	*L. major*	130	104	233
LmjF36.1410	*L. major*	56	466	521
LmjF36.1410	*L. major*	49	661	709
LmjF36.1410	*L. major*	45	809	853
LmjF36.1420	*L. major*	69	41	109
LmjF36.1420	*L. major*	47	181	227
LmjF36.1420	*L. major*	47	181	227
LmjF36.1430	*L. major*	70	59	128
LmjF36.1440	*L. major*	42	55	96
LmjF36.1440	*L. major*	69	141	209
LmjF36.1440	*L. major*	60	839	898
LmjF36.1440	*L. major*	75	920	994
LmjF36.1440	*L. major*	121	1815	1935
LmjF36.1440	*L. major*	80	2056	2135

LmjF36.1440	*L. major*	67	2214	2280
LmjF36.1440	*L. major*	79	2347	2425
LmjF36.1440	*L. major*	40	2684	2723
LmjF36.1440	*L. major*	46	3063	3108
LmjF36.1440	*L. major*	77	3245	3321
LmjF36.1440	*L. major*	59	3778	3836
LmjF36.1440	*L. major*	53	4072	4124
LmjF36.1440	*L. major*	53	4150	4202
LmjF36.1440	*L. major*	41	4550	4590
LmjF36.1440	*L. major*	41	5068	5108
LmjF36.1440	*L. major*	40	5229	5268
LmjF36.1460	*L. major*	196	1	196
LmjF36.1460	*L. major*	131	208	338
LmjF36.1460	*L. major*	316	340	655
LmjF36.1470	*L. major*	57	1	57
LmjF36.1470	*L. major*	111	385	495
LmjF36.1470	*L. major*	65	552	616
LmjF36.1470	*L. major*	49	626	674
LmjF36.1470	*L. major*	52	747	798
LmjF36.1470	*L. major*	98	834	931
LmjF36.1480	*L. major*	82	46	127
LmjF36.1480	*L. major*	44	242	285
LmjF36.1480	*L. major*	44	242	285
LmjF36.1490	*L. major*	554	1	554
LmjF36.1490	*L. major*	133	563	695
LmjF36.1490	*L. major*	365	698	1062
LmjF36.1490	*L. major*	104	1099	1202
LmjF36.1500	*L. major*	146	223	368
LmjF36.1500	*L. major*	46	554	599
LmjF36.1500	*L. major*	42	759	800
LmjF36.1500	*L. major*	116	831	946
LmjF36.1510	*L. major*	94	1	94
LmjF36.1520	*L. major*	44	324	367
LmjF36.1530	*L. major*	53	318	370
LmjF36.1540	*L. major*	488	1	488
LmjF36.1540	*L. major*	99	558	656
LmjF36.1550	*L. major*	71	1	71
LmjF36.1550	*L. major*	50	380	429
LmjF36.1550	*L. major*	47	519	565
LmjF36.1550	*L. major*	76	585	660
LmjF36.1550	*L. major*	112	737	848
LmjF36.1560	*L. major*	130	278	407
LmjF36.1570	*L. major*	437	40	476
LmjF36.1580	*L. major*	86	1	86
LmjF36.1580	*L. major*	62	179	240
LmjF36.1580	*L. major*	56	467	522
LmjF36.1580	*L. major*	74	579	652

LmjF36.1580	*L. major*	278	697	974
LmjF36.1580	*L. major*	70	1251	1320
LmjF36.1580	*L. major*	55	1419	1473
LmjF36.1580	*L. major*	106	1530	1635
LmjF36.1590	*L. major*	92	1	92
LmjF36.1590	*L. major*	129	1065	1193
LmjF36.1595	*L. major*	123	1	123
LmjF36.1610	*L. major*	115	1	115
LmjF36.1620	*L. major*	64	1	64
LmjF36.1620	*L. major*	110	66	175
LmjF36.1620	*L. major*	110	66	175
LmjF36.1630	*L. major*	60	1621	1680
LmjF36.1635	*L. major*	135	1	135
LmjF36.1640	*L. major*	271	1	271
LmjF36.1650	*L. major*	46	27	72
LmjF36.1660	*L. major*	278	18	295
LmjF36.1660	*L. major*	91	596	686
LmjF36.1670	*L. major*	41	144	184
LmjF36.1670	*L. major*	48	367	414
LmjF36.1670	*L. major*	48	367	414
LmjF36.1680	*L. major*	51	17	67
LmjF36.1680	*L. major*	56	229	284
LmjF36.1680	*L. major*	70	321	390
LmjF36.1680	*L. major*	84	512	595
LmjF36.1680	*L. major*	76	630	705
LmjF36.1690	*L. major*	46	137	182
LmjF36.1700	*L. major*	390	38	427
LmjF36.1700	*L. major*	55	457	511
LmjF36.1710	*L. major*	70	1	70
LmjF36.1720	*L. major*	115	13	127
LmjF36.1720	*L. major*	45	292	336
LmjF36.1720	*L. major*	100	390	489
LmjF36.1720	*L. major*	80	552	631
LmjF36.1720	*L. major*	42	813	854
LmjF36.1720	*L. major*	110	890	999
LmjF36.1720	*L. major*	73	1078	1150
LmjF36.1720	*L. major*	61	1458	1518
LmjF36.1720	*L. major*	40	2123	2162
LmjF36.1720	*L. major*	80	2289	2368
LmjF36.1720	*L. major*	46	2601	2646
LmjF36.1720	*L. major*	71	2875	2945
LmjF36.1720	*L. major*	60	3071	3130
LmjF36.1720	*L. major*	58	3229	3286
LmjF36.1720	*L. major*	47	3365	3411
LmjF36.1720	*L. major*	114	3968	4081
LmjF36.1730	*L. major*	60	44	103
LmjF36.1730	*L. major*	57	262	318

LmjF36.1730	*L. major*	64	704	767
LmjF36.1730	*L. major*	148	804	951
LmjF36.1740	*L. major*	67	1	67
LmjF36.1740	*L. major*	41	69	109
LmjF36.1740	*L. major*	99	223	321
LmjF36.1740	*L. major*	41	746	786
LmjF36.1740	*L. major*	48	799	846
LmjF36.1740	*L. major*	72	953	1024
LmjF36.1740	*L. major*	58	1092	1149
LmjF36.1750	*L. major*	74	167	240
LmjF36.1780	*L. major*	129	103	231
LmjF36.1780	*L. major*	45	354	398
LmjF36.1780	*L. major*	83	635	717
LmjF36.1790	*L. major*	47	91	137
LmjF36.1790	*L. major*	258	638	895
LmjF36.1800	*L. major*	184	53	236
LmjF36.1810	*L. major*	43	1	43
LmjF36.1810	*L. major*	49	169	217
LmjF36.1810	*L. major*	56	336	391
LmjF36.1810	*L. major*	77	643	719
LmjF36.1820	*L. major*	164	152	315
LmjF36.1820	*L. major*	56	345	400
LmjF36.1820	*L. major*	415	459	873
LmjF36.1830	*L. major*	92	1	92
LmjF36.1830	*L. major*	478	125	602
LmjF36.1840	*L. major*	158	1	158
LmjF36.1840	*L. major*	54	604	657
LmjF36.1850	*L. major*	53	1	53
LmjF36.1850	*L. major*	106	422	527
LmjF36.1860	*L. major*	53	358	410
LmjF36.1870	*L. major*	135	666	800
LmjF36.1880	*L. major*	41	198	238
LmjF36.1880	*L. major*	54	380	433
LmjF36.1900	*L. major*	72	1	72
LmjF36.1900	*L. major*	48	920	967
LmjF36.1900	*L. major*	335	972	1306
LmjF36.1900	*L. major*	223	1348	1570
LmjF36.1900	*L. major*	69	1587	1655
LmjF36.1905	*L. major*	255	1	255
LmjF36.1905	*L. major*	125	290	414
LmjF36.1910	*L. major*	150	66	215
LmjF36.1910	*L. major*	131	256	386
LmjF36.1920	*L. major*	40	149	188
LmjF36.1920	*L. major*	45	452	496
LmjF36.1920	*L. major*	45	452	496
LmjF36.1930	*L. major*	106	71	176
LmjF36.1930	*L. major*	50	227	276

LmjF36.1940	*L. major*	55	254	308
LmjF36.1950	*L. major*	41	554	594
LmjF36.1955	*L. major*	46	62	107
LmjF36.1955	*L. major*	40	344	383
LmjF36.1955	*L. major*	40	344	383
LmjF36.1970	*L. major*	61	1	61
LmjF36.2000	*L. major*	76	1038	1113
LmjF36.2000	*L. major*	100	1143	1242
LmjF36.2010	*L. major*	184	1	184
LmjF36.2010	*L. major*	103	186	288
LmjF36.2010	*L. major*	103	186	288
LmjF36.2020	*L. major*	40	346	385
LmjF36.2035	*L. major*	50	1	50
LmjF36.2035	*L. major*	45	93	137
LmjF36.2035	*L. major*	55	173	227
LmjF36.2035	*L. major*	52	242	293
LmjF36.2035	*L. major*	136	382	517
LmjF36.2035	*L. major*	70	527	596
LmjF36.2040	*L. major*	42	406	447
LmjF36.2050	*L. major*	146	1	146
LmjF36.2050	*L. major*	49	381	429
LmjF36.2050	*L. major*	66	504	569
LmjF36.2050	*L. major*	119	595	713
LmjF36.2060	*L. major*	48	1	48
LmjF36.2060	*L. major*	53	247	299
LmjF36.2060	*L. major*	108	399	506
LmjF36.2070	*L. major*	149	124	272
LmjF36.2080	*L. major*	420	50	469
LmjF36.2080	*L. major*	200	513	712
LmjF36.2080	*L. major*	488	801	1288
LmjF36.2090	*L. major*	49	198	246
LmjF36.2090	*L. major*	235	364	598
LmjF36.2090	*L. major*	51	626	676
LmjF36.2090	*L. major*	65	686	750
LmjF36.2100	*L. major*	74	72	145
LmjF36.2110	*L. major*	49	68	116
LmjF36.2110	*L. major*	46	227	272
LmjF36.2110	*L. major*	46	227	272
LmjF36.2115	*L. major*	237	133	369
LmjF36.2115	*L. major*	178	395	572
LmjF36.2115	*L. major*	43	617	659
LmjF36.2120	*L. major*	261	1	261
LmjF36.2120	*L. major*	46	283	328
LmjF36.2120	*L. major*	179	345	523
LmjF36.2120	*L. major*	791	560	1350
LmjF36.2120	*L. major*	128	1406	1533
LmjF36.2130	*L. major*	51	1	51

LmjF36.2130	*L. major*	83	491	573
LmjF36.2170	*L. major*	78	479	556
LmjF36.2180	*L. major*	75	1	75
LmjF36.2180	*L. major*	71	124	194
LmjF36.2180	*L. major*	42	376	417
LmjF36.2190	*L. major*	244	1	244
LmjF36.2190	*L. major*	320	267	586
LmjF36.2200	*L. major*	86	46	131
LmjF36.2200	*L. major*	187	457	643
LmjF36.2210	*L. major*	47	1	47
LmjF36.2220	*L. major*	71	161	231
LmjF36.2220	*L. major*	137	239	375
LmjF36.2230	*L. major*	41	134	174
LmjF36.2240	*L. major*	61	1	61
LmjF36.2240	*L. major*	77	218	294
LmjF36.2240	*L. major*	66	327	392
LmjF36.2240	*L. major*	47	434	480
LmjF36.2240	*L. major*	50	520	569
LmjF36.2240	*L. major*	61	808	868
LmjF36.2240	*L. major*	233	1082	1314
LmjF36.2240	*L. major*	153	1339	1491
LmjF36.2240	*L. major*	78	1530	1607
LmjF36.2240	*L. major*	133	1633	1765
LmjF36.2240	*L. major*	71	1954	2024
LmjF36.2240	*L. major*	313	2182	2494
LmjF36.2260	*L. major*	70	173	242
LmjF36.2270	*L. major*	65	201	265
LmjF36.2280	*L. major*	52	1	52
LmjF36.2280	*L. major*	55	441	495
LmjF36.2280	*L. major*	55	441	495
LmjF36.2290	*L. major*	51	19	69
LmjF36.2290	*L. major*	41	119	159
LmjF36.2290	*L. major*	86	391	476
LmjF36.2300	*L. major*	165	314	478
LmjF36.2310	*L. major*	93	233	325
LmjF36.2330	*L. major*	52	1	52
LmjF36.2340	*L. major*	100	241	340
LmjF36.2360	*L. major*	77	1	77
LmjF36.2370	*L. major*	80	1	80
LmjF36.2370	*L. major*	54	152	205
LmjF36.2370	*L. major*	41	222	262
LmjF36.2370	*L. major*	75	350	424
LmjF36.2370	*L. major*	66	455	520
LmjF36.2370	*L. major*	87	726	812
LmjF36.2380	*L. major*	47	1	47
LmjF36.2390	*L. major*	47	1	47
LmjF36.2410	*L. major*	58	131	188

LmjF36.2410	*L. major*	110	275	384
LmjF36.2410	*L. major*	99	424	522
LmjF36.2410	*L. major*	273	741	1013
LmjF36.2410	*L. major*	71	1317	1387
LmjF36.2410	*L. major*	69	1468	1536
LmjF36.2410	*L. major*	105	1548	1652
LmjF36.2410	*L. major*	103	1758	1860
LmjF36.2430	*L. major*	116	29	144
LmjF36.2440	*L. major*	173	22	194
LmjF36.2450	*L. major*	106	204	309
LmjF36.2460	*L. major*	61	267	327
LmjF36.2460	*L. major*	41	544	584
LmjF36.2460	*L. major*	41	544	584
LmjF36.2480	*L. major*	51	79	129
LmjF36.2480	*L. major*	108	152	259
LmjF36.2480	*L. major*	108	152	259
LmjF36.2490	*L. major*	206	1	206
LmjF36.2500	*L. major*	41	335	375
LmjF36.2500	*L. major*	48	440	487
LmjF36.2500	*L. major*	81	594	674
LmjF36.2510	*L. major*	102	1	102
LmjF36.2510	*L. major*	43	214	256
LmjF36.2510	*L. major*	49	701	749
LmjF36.2520	*L. major*	140	107	246
LmjF36.2520	*L. major*	43	308	350
LmjF36.2530	*L. major*	99	1	99
LmjF36.2530	*L. major*	45	422	466
LmjF36.2530	*L. major*	42	661	702
LmjF36.2550	*L. major*	42	1	42
LmjF36.2550	*L. major*	70	85	154
LmjF36.2550	*L. major*	66	271	336
LmjF36.2560	*L. major*	59	120	178
LmjF36.2560	*L. major*	62	865	926
LmjF36.2560	*L. major*	62	865	926
LmjF36.2580	*L. major*	51	242	292
LmjF36.2580	*L. major*	68	372	439
LmjF36.2580	*L. major*	49	724	772
LmjF36.2580	*L. major*	44	892	935
LmjF36.2590	*L. major*	41	465	505
LmjF36.2600	*L. major*	90	153	242
LmjF36.2600	*L. major*	40	332	371
LmjF36.2600	*L. major*	64	538	601
LmjF36.2600	*L. major*	41	662	702
LmjF36.2610	*L. major*	86	1	86
LmjF36.2620	*L. major*	201	1	201
LmjF36.2620	*L. major*	69	565	633
LmjF36.2620	*L. major*	116	707	822

LmjF36.2620	*L. major*	50	905	954
LmjF36.2620	*L. major*	44	1290	1333
LmjF36.2620	*L. major*	141	1452	1592
LmjF36.2630	*L. major*	80	1	80
LmjF36.2630	*L. major*	93	146	238
LmjF36.2630	*L. major*	139	300	438
LmjF36.2630	*L. major*	82	465	546
LmjF36.2630	*L. major*	51	624	674
LmjF36.2630	*L. major*	85	729	813
LmjF36.2630	*L. major*	108	845	952
LmjF36.2630	*L. major*	91	1015	1105
LmjF36.2630	*L. major*	77	1162	1238
LmjF36.2630	*L. major*	46	1307	1352
LmjF36.2630	*L. major*	59	1477	1535
LmjF36.2630	*L. major*	53	1698	1750
LmjF36.2630	*L. major*	41	1840	1880
LmjF36.2630	*L. major*	62	1992	2053
LmjF36.2630	*L. major*	303	2147	2449
LmjF36.2630	*L. major*	237	2453	2689
LmjF36.2630	*L. major*	54	2734	2787
LmjF36.2630	*L. major*	64	2822	2885
LmjF36.2630	*L. major*	70	2917	2986
LmjF36.2630	*L. major*	52	3252	3303
LmjF36.2630	*L. major*	67	3351	3417
LmjF36.2650	*L. major*	103	101	203
LmjF36.2660	*L. major*	132	100	231
LmjF36.2670	*L. major*	54	20	73
LmjF36.2670	*L. major*	41	140	180
LmjF36.2670	*L. major*	68	325	392
LmjF36.2685	*L. major*	114	42	155
LmjF36.2690	*L. major*	41	44	84
LmjF36.2690	*L. major*	44	152	195
LmjF36.2690	*L. major*	46	213	258
LmjF36.2690	*L. major*	62	278	339
LmjF36.2700	*L. major*	146	1	146
LmjF36.2700	*L. major*	271	378	648
LmjF36.2700	*L. major*	59	1380	1438
LmjF36.2710	*L. major*	52	520	571
LmjF36.2740	*L. major*	55	306	360
LmjF36.2740	*L. major*	43	977	1019
LmjF36.2740	*L. major*	43	977	1019
LmjF36.2760	*L. major*	50	310	359
LmjF36.2770	*L. major*	607	1	607
LmjF36.2790	*L. major*	40	506	545
LmjF36.2790	*L. major*	42	1331	1372
LmjF36.2790	*L. major*	48	2078	2125
LmjF36.2790	*L. major*	60	2531	2590

LmjF36.2800	*L. major*	51	30	80
LmjF36.2800	*L. major*	305	115	419
LmjF36.2800	*L. major*	102	502	603
LmjF36.2810	*L. major*	62	94	155
LmjF36.2810	*L. major*	98	329	426
LmjF36.2810	*L. major*	63	525	587
LmjF36.2820	*L. major*	212	380	591
LmjF36.2830	*L. major*	61	46	106
LmjF36.2830	*L. major*	251	155	405
LmjF36.2840	*L. major*	138	38	175
LmjF36.2860	*L. major*	45	93	137
LmjF36.2870	*L. major*	45	93	137
LmjF36.2880	*L. major*	77	464	540
LmjF36.2880	*L. major*	84	1527	1610
LmjF36.2880	*L. major*	46	1672	1717
LmjF36.2890	*L. major*	48	1	48
LmjF36.2890	*L. major*	78	308	385
LmjF36.2900	*L. major*	86	481	566
LmjF36.2900	*L. major*	69	599	667
LmjF36.2900	*L. major*	112	801	912
LmjF36.2910	*L. major*	56	20	75
LmjF36.2910	*L. major*	49	298	346
LmjF36.2930	*L. major*	205	346	550
LmjF36.2940	*L. major*	46	366	411
LmjF36.2940	*L. major*	58	539	596
LmjF36.2940	*L. major*	40	2233	2272
LmjF36.2940	*L. major*	138	2605	2742
LmjF36.2940	*L. major*	94	3698	3791
LmjF36.2980	*L. major*	121	113	233
LmjF36.2990	*L. major*	43	363	405
LmjF36.3000	*L. major*	47	250	296
LmjF36.3000	*L. major*	64	705	768
LmjF36.3000	*L. major*	64	705	768
LmjF36.3040	*L. major*	46	138	183
LmjF36.3090	*L. major*	294	238	531
LmjF36.3090	*L. major*	45	779	823
LmjF36.3100	*L. major*	99	1	99
LmjF36.3140	*L. major*	42	20	61
LmjF36.3150	*L. major*	294	125	418
LmjF36.3160	*L. major*	60	1	60
LmjF36.3160	*L. major*	65	94	158
LmjF36.3160	*L. major*	65	94	158
LmjF36.3180	*L. major*	48	79	126
LmjF36.3180	*L. major*	43	278	320
LmjF36.3180	*L. major*	81	1182	1262
LmjF36.3190	*L. major*	98	1	98
LmjF36.3190	*L. major*	68	140	207

LmjF36.3190	*L. major*	84	246	329
LmjF36.3200	*L. major*	85	846	930
LmjF36.3220	*L. major*	353	31	383
LmjF36.3220	*L. major*	85	409	493
LmjF36.3250	*L. major*	53	132	184
LmjF36.3260	*L. major*	79	95	173
LmjF36.3280	*L. major*	46	1	46
LmjF36.3280	*L. major*	42	134	175
LmjF36.3280	*L. major*	47	206	252
LmjF36.3280	*L. major*	65	1106	1170
LmjF36.3300	*L. major*	287	22	308
LmjF36.3300	*L. major*	64	343	406
LmjF36.3300	*L. major*	290	458	747
LmjF36.3330	*L. major*	146	187	332
LmjF36.3340	*L. major*	61	320	380
LmjF36.3340	*L. major*	77	1482	1558
LmjF36.3340	*L. major*	40	1763	1802
LmjF36.3350	*L. major*	47	45	91
LmjF36.3410	*L. major*	70	1	70
LmjF36.3430	*L. major*	77	294	370
LmjF36.3430	*L. major*	79	389	467
LmjF36.3430	*L. major*	97	632	728
LmjF36.3440	*L. major*	216	1	216
LmjF36.3480	*L. major*	155	54	208
LmjF36.3490	*L. major*	117	223	339
LmjF36.3500	*L. major*	391	1	391
LmjF36.3510	*L. major*	42	145	186
LmjF36.3520	*L. major*	66	1	66
LmjF36.3540	*L. major*	57	43	99
LmjF36.3540	*L. major*	44	191	234
LmjF36.3540	*L. major*	40	332	371
LmjF36.3550	*L. major*	291	266	556
LmjF36.3550	*L. major*	435	580	1014
LmjF36.3550	*L. major*	50	1038	1087
LmjF36.3570	*L. major*	166	103	268
LmjF36.3600	*L. major*	41	73	113
LmjF36.3600	*L. major*	119	126	244
LmjF36.3600	*L. major*	112	380	491
LmjF36.3630	*L. major*	75	127	201
LmjF36.3630	*L. major*	52	239	290
LmjF36.3630	*L. major*	52	239	290
LmjF36.3670	*L. major*	55	59	113
LmjF36.3670	*L. major*	87	254	340
LmjF36.3680	*L. major*	51	640	690
LmjF36.3690	*L. major*	42	1	42
LmjF36.3720	*L. major*	49	26	74
LmjF36.3720	*L. major*	131	125	255

LmjF36.3720	*L. major*	41	546	586
LmjF36.3740	*L. major*	51	1	51
LmjF36.3740	*L. major*	71	98	168
LmjF36.3770	*L. major*	41	1	41
LmjF36.3790	*L. major*	220	142	361
LmjF36.3790	*L. major*	62	490	551
LmjF36.3790	*L. major*	61	574	634
LmjF36.3790	*L. major*	272	672	943
LmjF36.3820	*L. major*	106	1	106
LmjF36.3830	*L. major*	41	1	41
LmjF36.3830	*L. major*	77	186	262
LmjF36.3830	*L. major*	77	186	262
LmjF36.3850	*L. major*	225	1	225
LmjF36.3870	*L. major*	77	56	132
LmjF36.3870	*L. major*	40	171	210
LmjF36.3870	*L. major*	48	276	323
LmjF36.3870	*L. major*	96	414	509
LmjF36.3870	*L. major*	131	552	682
LmjF36.3870	*L. major*	76	691	766
LmjF36.3870	*L. major*	44	782	825
LmjF36.3890	*L. major*	120	90	209
LmjF36.3900	*L. major*	100	131	230
LmjF36.3930	*L. major*	101	59	159
LmjF36.3930	*L. major*	129	228	356
LmjF36.3930	*L. major*	60	654	713
LmjF36.3930	*L. major*	168	751	918
LmjF36.3930	*L. major*	120	1332	1451
LmjF36.3940	*L. major*	130	1	130
LmjF36.3940	*L. major*	234	236	469
LmjF36.3940	*L. major*	221	505	725
LmjF36.3940	*L. major*	101	754	854
LmjF36.3940	*L. major*	123	887	1009
LmjF36.3950	*L. major*	41	56	96
LmjF36.3960	*L. major*	193	1	193
LmjF36.3960	*L. major*	321	297	617
LmjF36.3960	*L. major*	178	654	831
LmjF36.3960	*L. major*	116	902	1017
LmjF36.3970	*L. major*	127	24	150
LmjF36.3970	*L. major*	63	255	317
LmjF36.3970	*L. major*	41	592	632
LmjF36.3970	*L. major*	257	761	1017
LmjF36.3970	*L. major*	244	1111	1354
LmjF36.3980	*L. major*	69	100	168
LmjF36.4010	*L. major*	83	172	254
LmjF36.4010	*L. major*	113	317	429
LmjF36.4010	*L. major*	113	317	429
LmjF36.4020	*L. major*	150	39	188

LmjF36.4030	*L. major*	310	23	332
LmjF36.4030	*L. major*	95	376	470
LmjF36.4030	*L. major*	67	790	856
LmjF36.4030	*L. major*	118	1079	1196
LmjF36.4040	*L. major*	93	1	93
LmjF36.4040	*L. major*	120	311	430
LmjF36.4050	*L. major*	53	111	163
LmjF36.4050	*L. major*	50	245	294
LmjF36.4050	*L. major*	50	245	294
LmjF36.4060	*L. major*	73	1	73
LmjF36.4060	*L. major*	79	576	654
LmjF36.4070	*L. major*	41	141	181
LmjF36.4080	*L. major*	266	200	465
LmjF36.4090	*L. major*	84	1	84
LmjF36.4090	*L. major*	245	173	417
LmjF36.4100	*L. major*	133	261	393
LmjF36.4100	*L. major*	143	1198	1340
LmjF36.4100	*L. major*	57	1364	1420
LmjF36.4110	*L. major*	69	1	69
LmjF36.4110	*L. major*	55	76	130
LmjF36.4110	*L. major*	102	305	406
LmjF36.4110	*L. major*	73	837	909
LmjF36.4120	*L. major*	44	1	44
LmjF36.4150	*L. major*	45	81	125
LmjF36.4150	*L. major*	105	271	375
LmjF36.4150	*L. major*	86	393	478
LmjF36.4150	*L. major*	105	511	615
LmjF36.4160	*L. major*	329	1	329
LmjF36.4190	*L. major*	124	220	343
LmjF36.4200	*L. major*	238	275	512
LmjF36.4200	*L. major*	64	681	744
LmjF36.4210	*L. major*	63	694	756
LmjF36.4210	*L. major*	59	786	844
LmjF36.4210	*L. major*	59	786	844
LmjF36.4220	*L. major*	109	19	127
LmjF36.4220	*L. major*	250	228	477
LmjF36.4220	*L. major*	126	485	610
LmjF36.4240	*L. major*	88	124	211
LmjF36.4240	*L. major*	102	300	401
LmjF36.4250	*L. major*	62	145	206
LmjF36.4250	*L. major*	80	218	297
LmjF36.4250	*L. major*	144	515	658
LmjF36.4250	*L. major*	79	686	764
LmjF36.4260	*L. major*	149	1	149
LmjF36.4260	*L. major*	167	176	342
LmjF36.4270	*L. major*	71	180	250
LmjF36.4270	*L. major*	92	1142	1233

LmjF36.4270	*L. major*	50	1414	1463
LmjF36.4280	*L. major*	65	358	422
LmjF36.4280	*L. major*	42	493	534
LmjF36.4280	*L. major*	42	493	534
LmjF36.4290	*L. major*	81	346	426
LmjF36.4290	*L. major*	138	839	976
LmjF36.4290	*L. major*	66	1165	1230
LmjF36.4320	*L. major*	41	95	135
LmjF36.4320	*L. major*	52	166	217
LmjF36.4320	*L. major*	149	269	417
LmjF36.4320	*L. major*	41	1019	1059
LmjF36.4320	*L. major*	67	1676	1742
LmjF36.4320	*L. major*	46	2148	2193
LmjF36.4330	*L. major*	241	1	241
LmjF36.4330	*L. major*	43	786	828
LmjF36.4330	*L. major*	59	936	994
LmjF36.4330	*L. major*	40	1019	1058
LmjF36.4330	*L. major*	128	1239	1366
LmjF36.4330	*L. major*	80	1546	1625
LmjF36.4340	*L. major*	521	1	521
LmjF36.4350	*L. major*	61	1	61
LmjF36.4350	*L. major*	242	63	304
LmjF36.4350	*L. major*	109	389	497
LmjF36.4350	*L. major*	85	622	706
LmjF36.4350	*L. major*	168	722	889
LmjF36.4360	*L. major*	48	34	81
LmjF36.4370	*L. major*	62	437	498
LmjF36.4370	*L. major*	225	654	878
LmjF36.4370	*L. major*	41	967	1007
LmjF36.4370	*L. major*	187	1098	1284
LmjF36.4370	*L. major*	87	1387	1473
LmjF36.4370	*L. major*	84	1746	1829
LmjF36.4370	*L. major*	54	2179	2232
LmjF36.4380	*L. major*	79	1	79
LmjF36.4380	*L. major*	75	169	243
LmjF36.4380	*L. major*	69	375	443
LmjF36.4390	*L. major*	70	56	125
LmjF36.4390	*L. major*	60	360	419
LmjF36.4390	*L. major*	45	603	647
LmjF36.4400	*L. major*	183	1	183
LmjF36.4400	*L. major*	51	217	267
LmjF36.4400	*L. major*	40	292	331
LmjF36.4400	*L. major*	163	451	613
LmjF36.4400	*L. major*	52	913	964
LmjF36.4410	*L. major*	133	62	194
LmjF36.4460	*L. major*	44	117	160
LmjF36.4470	*L. major*	42	58	99

LmjF36.4470	*L. major*	113	163	275
LmjF36.4480	*L. major*	120	130	249
LmjF36.4480	*L. major*	88	562	649
LmjF36.4490	*L. major*	94	1	94
LmjF36.4490	*L. major*	40	121	160
LmjF36.4490	*L. major*	45	343	387
LmjF36.4510	*L. major*	51	148	198
LmjF36.4520	*L. major*	41	390	430
LmjF36.4520	*L. major*	40	432	471
LmjF36.4520	*L. major*	78	554	631
LmjF36.4530	*L. major*	99	1	99
LmjF36.4540	*L. major*	45	1	45
LmjF36.4540	*L. major*	60	310	369
LmjF36.4550	*L. major*	74	1	74
LmjF36.4550	*L. major*	111	734	844
LmjF36.4550	*L. major*	111	734	844
LmjF36.4560	*L. major*	263	87	349
LmjF36.4560	*L. major*	42	452	493
LmjF36.4570	*L. major*	91	1	91
LmjF36.4580	*L. major*	80	36	115
LmjF36.4580	*L. major*	50	213	262
LmjF36.4580	*L. major*	47	319	365
LmjF36.4590	*L. major*	78	51	128
LmjF36.4590	*L. major*	146	199	344
LmjF36.4590	*L. major*	58	531	588
LmjF36.4590	*L. major*	61	636	696
LmjF36.4610	*L. major*	159	1	159
LmjF36.4610	*L. major*	74	222	295
LmjF36.4610	*L. major*	86	330	415
LmjF36.4620	*L. major*	113	17	129
LmjF36.4620	*L. major*	63	401	463
LmjF36.4620	*L. major*	58	720	777
LmjF36.4620	*L. major*	41	1200	1240
LmjF36.4640	*L. major*	135	416	550
LmjF36.4660	*L. major*	51	66	116
LmjF36.4660	*L. major*	96	287	382
LmjF36.4660	*L. major*	99	691	789
LmjF36.4660	*L. major*	89	808	896
LmjF36.4670	*L. major*	128	1	128
LmjF36.4680	*L. major*	51	1	51
LmjF36.4680	*L. major*	67	173	239
LmjF36.4680	*L. major*	49	633	681
LmjF36.4680	*L. major*	79	698	776
LmjF36.4680	*L. major*	588	786	1373
LmjF36.4680	*L. major*	430	1385	1814
LmjF36.4690	*L. major*	70	59	128
LmjF36.4690	*L. major*	121	271	391

LmjF36.4690	*L. major*	88	534	621
LmjF36.4690	*L. major*	48	757	804
LmjF36.4690	*L. major*	51	838	888
LmjF36.4710	*L. major*	48	143	190
LmjF36.4710	*L. major*	284	444	727
LmjF36.4720	*L. major*	59	408	466
LmjF36.4720	*L. major*	97	664	760
LmjF36.4720	*L. major*	85	924	1008
LmjF36.4730	*L. major*	63	165	227
LmjF36.4730	*L. major*	54	443	496
LmjF36.4730	*L. major*	565	913	1477
LmjF36.4740	*L. major*	63	108	170
LmjF36.4740	*L. major*	188	481	668
LmjF36.4740	*L. major*	140	777	916
LmjF36.4740	*L. major*	250	935	1184
LmjF36.4740	*L. major*	125	1282	1406
LmjF36.4760	*L. major*	144	99	242
LmjF36.4790	*L. major*	162	1	162
LmjF36.4790	*L. major*	102	197	298
LmjF36.4790	*L. major*	48	329	376
LmjF36.4790	*L. major*	78	400	477
LmjF36.4790	*L. major*	142	485	626
LmjF36.4790	*L. major*	128	687	814
LmjF36.4790	*L. major*	82	837	918
LmjF36.4800	*L. major*	45	98	142
LmjF36.4800	*L. major*	65	226	290
LmjF36.4820	*L. major*	171	1	171
LmjF36.4840	*L. major*	46	1	46
LmjF36.4840	*L. major*	65	775	839
LmjF36.4840	*L. major*	91	893	983
LmjF36.4850	*L. major*	96	22	117
LmjF36.4850	*L. major*	130	219	348
LmjF36.4850	*L. major*	57	419	475
LmjF36.4850	*L. major*	123	487	609
LmjF36.4890	*L. major*	74	1	74
LmjF36.4890	*L. major*	57	276	332
LmjF36.4890	*L. major*	57	276	332
LmjF36.4900	*L. major*	100	1	100
LmjF36.4900	*L. major*	154	122	275
LmjF36.4900	*L. major*	74	1334	1407
LmjF36.4900	*L. major*	76	1575	1650
LmjF36.4900	*L. major*	41	1658	1698
LmjF36.4900	*L. major*	62	1722	1783
LmjF36.4900	*L. major*	86	1950	2035
LmjF36.4900	*L. major*	53	2043	2095
LmjF36.4910	*L. major*	360	1	360
LmjF36.4920	*L. major*	131	62	192

LmjF36.4920	*L. major*	238	342	579
LmjF36.4930	*L. major*	99	46	144
LmjF36.4940	*L. major*	45	97	141
LmjF36.4950	*L. major*	79	1	79
LmjF36.4950	*L. major*	106	200	305
LmjF36.4950	*L. major*	61	578	638
LmjF36.4960	*L. major*	167	246	412
LmjF36.4960	*L. major*	43	870	912
LmjF36.4970	*L. major*	77	89	165
LmjF36.4970	*L. major*	132	214	345
LmjF36.4980	*L. major*	103	55	157
LmjF36.4990	*L. major*	71	27	97
LmjF36.4990	*L. major*	60	108	167
LmjF36.4990	*L. major*	60	108	167
LmjF36.5000	*L. major*	63	294	356
LmjF36.5020	*L. major*	40	1	40
LmjF36.5020	*L. major*	233	67	299
LmjF36.5020	*L. major*	305	319	623
LmjF36.5020	*L. major*	144	668	811
LmjF36.5030	*L. major*	102	1	102
LmjF36.5030	*L. major*	46	125	170
LmjF36.5030	*L. major*	118	212	329
LmjF36.5060	*L. major*	99	155	253
LmjF36.5080	*L. major*	130	1	130
LmjF36.5080	*L. major*	55	228	282
LmjF36.5080	*L. major*	72	350	421
LmjF36.5090	*L. major*	63	212	274
LmjF36.5100	*L. major*	257	1	257
LmjF36.5100	*L. major*	93	413	505
LmjF36.5100	*L. major*	73	616	688
LmjF36.5140	*L. major*	52	164	215
LmjF36.5140	*L. major*	230	289	518
LmjF36.5150	*L. major*	263	363	625
LmjF36.5160	*L. major*	42	199	240
LmjF36.5160	*L. major*	75	291	365
LmjF36.5190	*L. major*	146	1	146
LmjF36.5190	*L. major*	71	153	223
LmjF36.5190	*L. major*	92	1054	1145
LmjF36.5200	*L. major*	45	355	399
LmjF36.5200	*L. major*	58	565	622
LmjF36.5200	*L. major*	58	565	622
LmjF36.5210	*L. major*	76	1	76
LmjF36.5210	*L. major*	53	1212	1264
LmjF36.5210	*L. major*	53	1212	1264
LmjF36.5230	*L. major*	62	1	62
LmjF36.5240	*L. major*	105	58	162
LmjF36.5240	*L. major*	71	299	369

LmjF36.5250	*L. major*	43	163	205
LmjF36.5250	*L. major*	57	263	319
LmjF36.5250	*L. major*	57	360	416
LmjF36.5250	*L. major*	67	454	520
LmjF36.5250	*L. major*	55	682	736
LmjF36.5250	*L. major*	713	908	1620
LmjF36.5250	*L. major*	76	2000	2075
LmjF36.5250	*L. major*	152	2138	2289
LmjF36.5250	*L. major*	77	2346	2422
LmjF36.5250	*L. major*	66	2548	2613
LmjF36.5250	*L. major*	53	2627	2679
LmjF36.5250	*L. major*	78	2895	2972
LmjF36.5250	*L. major*	67	3005	3071
LmjF36.5250	*L. major*	47	3516	3562
LmjF36.5260	*L. major*	61	266	326
LmjF36.5260	*L. major*	164	686	849
LmjF36.5270	*L. major*	57	51	107
LmjF36.5270	*L. major*	52	518	569
LmjF36.5270	*L. major*	72	652	723
LmjF36.5280	*L. major*	81	1	81
LmjF36.5280	*L. major*	48	111	158
LmjF36.5280	*L. major*	62	311	372
LmjF36.5280	*L. major*	51	603	653
LmjF36.5280	*L. major*	66	674	739
LmjF36.5280	*L. major*	44	905	948
LmjF36.5290	*L. major*	95	1	95
LmjF36.5290	*L. major*	51	108	158
LmjF36.5300	*L. major*	52	1	52
LmjF36.5300	*L. major*	52	89	140
LmjF36.5300	*L. major*	47	155	201
LmjF36.5300	*L. major*	206	508	713
LmjF36.5310	*L. major*	48	1	48
LmjF36.5310	*L. major*	65	93	157
LmjF36.5310	*L. major*	55	317	371
LmjF36.5320	*L. major*	109	427	535
LmjF36.5330	*L. major*	153	146	298
LmjF36.5330	*L. major*	43	888	930
LmjF36.5330	*L. major*	79	960	1038
LmjF36.5340	*L. major*	656	1	656
LmjF36.5340	*L. major*	62	781	842
LmjF36.5350	*L. major*	40	1	40
LmjF36.5350	*L. major*	81	329	409
LmjF36.5350	*L. major*	66	444	509
LmjF36.5350	*L. major*	381	618	998
LmjF36.5365	*L. major*	64	443	506
LmjF36.5370	*L. major*	183	311	493
LmjF36.5380	*L. major*	77	451	527

LmjF36.5400	*L. major*	95	238	332
LmjF36.5400	*L. major*	72	366	437
LmjF36.5400	*L. major*	72	366	437
LmjF36.5420	*L. major*	104	186	289
LmjF36.5430	*L. major*	67	178	244
LmjF36.5440	*L. major*	108	1	108
LmjF36.5440	*L. major*	175	110	284
LmjF36.5440	*L. major*	53	381	433
LmjF36.5440	*L. major*	47	478	524
LmjF36.5450	*L. major*	45	132	176
LmjF36.5460	*L. major*	48	458	505
LmjF36.5470	*L. major*	63	1	63
LmjF36.5470	*L. major*	74	95	168
LmjF36.5470	*L. major*	79	179	257
LmjF36.5470	*L. major*	111	342	452
LmjF36.5470	*L. major*	83	822	904
LmjF36.5480	*L. major*	146	21	166
LmjF36.5480	*L. major*	233	410	642
LmjF36.5490	*L. major*	170	1	170
LmjF36.5500	*L. major*	48	1	48
LmjF36.5510	*L. major*	198	1	198
LmjF36.5530	*L. major*	50	23	72
LmjF36.5540	*L. major*	51	296	346
LmjF36.5550	*L. major*	44	596	639
LmjF36.5550	*L. major*	59	1243	1301
LmjF36.5550	*L. major*	59	1243	1301
LmjF36.5560	*L. major*	50	326	375
LmjF36.5580	*L. major*	44	110	153
LmjF36.5580	*L. major*	67	306	372
LmjF36.5580	*L. major*	67	306	372
LmjF36.5590	*L. major*	43	1	43
LmjF36.5590	*L. major*	70	45	114
LmjF36.5600	*L. major*	169	25	193
LmjF36.5600	*L. major*	42	244	285
LmjF36.5600	*L. major*	63	347	409
LmjF36.5630	*L. major*	64	96	159
LmjF36.5640	*L. major*	117	378	494
LmjF36.5650	*L. major*	59	217	275
LmjF36.5650	*L. major*	53	277	329
LmjF36.5660	*L. major*	111	293	403
LmjF36.5660	*L. major*	57	527	583
LmjF36.5660	*L. major*	67	651	717
LmjF36.5670	*L. major*	45	561	605
LmjF36.5690	*L. major*	96	209	304
LmjF36.5700	*L. major*	52	410	461
LmjF36.5700	*L. major*	149	641	789
LmjF36.5730	*L. major*	50	117	166

LmjF36.5730	*L. major*	68	332	399
LmjF36.5740	*L. major*	380	54	433
LmjF36.5740	*L. major*	89	443	531
LmjF36.5740	*L. major*	172	559	730
LmjF36.5750	*L. major*	56	77	132
LmjF36.5760	*L. major*	40	58	97
LmjF36.5760	*L. major*	43	256	298
LmjF36.5760	*L. major*	89	357	445
LmjF36.5760	*L. major*	49	650	698
LmjF36.5760	*L. major*	83	1059	1141
LmjF36.5760	*L. major*	48	1454	1501
LmjF36.5760	*L. major*	56	1640	1695
LmjF36.5780	*L. major*	43	76	118
LmjF36.5780	*L. major*	50	255	304
LmjF36.5780	*L. major*	47	418	464
LmjF36.5790	*L. major*	70	300	369
LmjF36.5800	*L. major*	52	294	345
LmjF36.5800	*L. major*	85	790	874
LmjF36.5800	*L. major*	53	1094	1146
LmjF36.5810	*L. major*	46	99	144
LmjF36.5810	*L. major*	122	194	315
LmjF36.5810	*L. major*	44	354	397
LmjF36.5820	*L. major*	74	70	143
LmjF36.5820	*L. major*	187	151	337
LmjF36.5820	*L. major*	65	374	438
LmjF36.5840	*L. major*	54	29	82
LmjF36.5840	*L. major*	117	88	204
LmjF36.5840	*L. major*	129	219	347
LmjF36.5845	*L. major*	67	23	89
LmjF36.5850	*L. major*	116	38	153
LmjF36.5850	*L. major*	43	183	225
LmjF36.5850	*L. major*	289	249	537
LmjF36.5860	*L. major*	88	396	483
LmjF36.5860	*L. major*	44	628	671
LmjF36.5860	*L. major*	44	628	671
LmjF36.5870	*L. major*	43	220	262
LmjF36.5890	*L. major*	59	31	89
LmjF36.5890	*L. major*	110	385	494
LmjF36.5890	*L. major*	68	529	596
LmjF36.5890	*L. major*	66	1085	1150
LmjF36.5890	*L. major*	54	1203	1256
LmjF36.5890	*L. major*	52	1808	1859
LmjF36.5920	*L. major*	62	20	81
LmjF36.5920	*L. major*	48	114	161
LmjF36.5930	*L. major*	177	1	177
LmjF36.5930	*L. major*	92	201	292
LmjF36.5930	*L. major*	92	201	292

LmjF36.5940	*L. major*	86	47	132
LmjF36.5950	*L. major*	63	1	63
LmjF36.5950	*L. major*	116	83	198
LmjF36.5950	*L. major*	116	229	344
LmjF36.5970	*L. major*	252	387	638
LmjF36.5980	*L. major*	75	1	75
LmjF36.5980	*L. major*	68	393	460
LmjF36.5990	*L. major*	106	157	262
LmjF36.5990	*L. major*	280	378	657
LmjF36.6000	*L. major*	173	1	173
LmjF36.6000	*L. major*	130	200	329
LmjF36.6000	*L. major*	55	353	407
LmjF36.6000	*L. major*	81	418	498
LmjF36.6000	*L. major*	143	626	768
LmjF36.6000	*L. major*	72	781	852
LmjF36.6010	*L. major*	232	1	232
LmjF36.6010	*L. major*	113	246	358
LmjF36.6010	*L. major*	84	415	498
LmjF36.6010	*L. major*	50	621	670
LmjF36.6020	*L. major*	48	94	141
LmjF36.6030	*L. major*	46	318	363
LmjF36.6030	*L. major*	108	551	658
LmjF36.6030	*L. major*	41	740	780
LmjF36.6030	*L. major*	105	900	1004
LmjF36.6030	*L. major*	67	1045	1111
LmjF36.6030	*L. major*	57	1140	1196
LmjF36.6030	*L. major*	97	1321	1417
LmjF36.6030	*L. major*	120	1478	1597
LmjF36.6030	*L. major*	71	1698	1768
LmjF36.6030	*L. major*	50	1926	1975
LmjF36.6030	*L. major*	90	2257	2346
LmjF36.6040	*L. major*	68	1	68
LmjF36.6040	*L. major*	48	90	137
LmjF36.6040	*L. major*	54	277	330
LmjF36.6040	*L. major*	57	863	919
LmjF36.6060	*L. major*	43	52	94
LmjF36.6060	*L. major*	45	174	218
LmjF36.6060	*L. major*	202	331	532
LmjF36.6070	*L. major*	133	56	188
LmjF36.6080	*L. major*	87	1	87
LmjF36.6120	*L. major*	45	268	312
LmjF36.6130	*L. major*	62	1	62
LmjF36.6130	*L. major*	101	68	168
LmjF36.6130	*L. major*	102	218	319
LmjF36.6140	*L. major*	72	119	190
LmjF36.6140	*L. major*	533	234	766
LmjF36.6150	*L. major*	114	174	287

LmjF36.6160	*L. major*	204	1	204
LmjF36.6170	*L. major*	122	116	237
LmjF36.6200	*L. major*	176	1	176
LmjF36.6200	*L. major*	142	211	352
LmjF36.6200	*L. major*	54	789	842
LmjF36.6210	*L. major*	41	978	1018
LmjF36.6220	*L. major*	185	53	237
LmjF36.6220	*L. major*	207	269	475
LmjF36.6220	*L. major*	378	533	910
LmjF36.6220	*L. major*	254	930	1183
LmjF36.6220	*L. major*	43	1215	1257
LmjF36.6220	*L. major*	195	1274	1468
LmjF36.6240	*L. major*	49	33	81
LmjF36.6240	*L. major*	341	322	662
LmjF36.6240	*L. major*	95	665	759
LmjF36.6250	*L. major*	130	168	297
LmjF36.6270	*L. major*	83	31	113
LmjF36.6270	*L. major*	47	129	175
LmjF36.6270	*L. major*	47	129	175
LmjF36.6300	*L. major*	124	1	124
LmjF36.6300	*L. major*	40	206	245
LmjF36.6320	*L. major*	43	1110	1152
LmjF36.6320	*L. major*	40	1209	1248
LmjF36.6320	*L. major*	118	2409	2526
LmjF36.6320	*L. major*	47	2539	2585
LmjF36.6340	*L. major*	51	1140	1190
LmjF36.6340	*L. major*	68	1298	1365
LmjF36.6340	*L. major*	41	1884	1924
LmjF36.6340	*L. major*	147	2224	2370
LmjF36.6340	*L. major*	91	2980	3070
LmjF36.6340	*L. major*	67	3211	3277
LmjF36.6360	*L. major*	46	1	46
LmjF36.6360	*L. major*	106	159	264
LmjF36.6370	*L. major*	146	70	215
LmjF36.6380	*L. major*	45	185	229
LmjF36.6380	*L. major*	55	360	414
LmjF36.6380	*L. major*	55	360	414
LmjF36.6400	*L. major*	168	1	168
LmjF36.6410	*L. major*	125	193	317
LmjF36.6410	*L. major*	164	333	496
LmjF36.6420	*L. major*	53	154	206
LmjF36.6430	*L. major*	122	1	122
LmjF36.6430	*L. major*	49	183	231
LmjF36.6440	*L. major*	66	243	308
LmjF36.6465	*L. major*	399	64	462
LmjF36.6480	*L. major*	41	351	391
LmjF36.6490	*L. major*	141	185	325

LmjF36.6500	*L. major*	179	59	237
LmjF36.6500	*L. major*	54	503	556
LmjF36.6510	*L. major*	54	37	90
LmjF36.6520	*L. major*	88	96	183
LmjF36.6520	*L. major*	361	269	629
LmjF36.6525	*L. major*	144	1	144
LmjF36.6525	*L. major*	120	477	596
LmjF36.6530	*L. major*	168	1	168
LmjF36.6530	*L. major*	186	212	397
LmjF36.6560	*L. major*	97	93	189
LmjF36.6560	*L. major*	88	237	324
LmjF36.6570	*L. major*	316	47	362
LmjF36.6590	*L. major*	51	1	51
LmjF36.6590	*L. major*	153	127	279
LmjF36.6590	*L. major*	44	325	368
LmjF36.6590	*L. major*	49	405	453
LmjF36.6590	*L. major*	45	526	570
LmjF36.6590	*L. major*	52	597	648
LmjF36.6600	*L. major*	41	478	518
LmjF36.6600	*L. major*	43	693	735
LmjF36.6600	*L. major*	43	693	735
LmjF36.6610	*L. major*	44	1	44
LmjF36.6630	*L. major*	49	81	129
LmjF36.6670	*L. major*	40	1	40
LmjF36.6680	*L. major*	59	84	142
LmjF36.6730	*L. major*	84	392	475
LmjF36.6730	*L. major*	48	599	646
LmjF36.6730	*L. major*	59	838	896
LmjF36.6730	*L. major*	40	1037	1076
LmjF36.6770	*L. major*	48	1	48
LmjF36.6770	*L. major*	146	601	746
LmjF36.6780	*L. major*	41	252	292
LmjF36.6790	*L. major*	46	441	486
LmjF36.6800	*L. major*	53	272	324
LmjF36.6840	*L. major*	41	1	41
LmjF36.6840	*L. major*	69	82	150
LmjF36.6840	*L. major*	69	82	150
LmjF36.6850	*L. major*	83	584	666
LmjF36.6860	*L. major*	103	1	103
LmjF36.6880	*L. major*	99	185	283
LmjF36.6890	*L. major*	40	389	428
LmjF36.6890	*L. major*	41	528	568
LmjF36.6890	*L. major*	41	528	568
LmjF36.6900	*L. major*	72	154	225
LmjF36.6900	*L. major*	356	352	707
LmjF36.6930	*L. major*	59	1	59
LmjF36.6930	*L. major*	78	79	156

LmjF36.6930	*L. major*	250	262	511
LmjF36.6960	*L. major*	326	1	326
LmjF36.6980	*L. major*	55	1	55
LmjF36.6980	*L. major*	69	115	183
LmjF36.6980	*L. major*	50	682	731
LinJ01_V3.0010	*L. infantum*	47	287	333
LinJ01_V3.0020	*L. infantum*	92	1	92
LinJ01_V3.0020	*L. infantum*	50	413	462
LinJ01_V3.0030	*L. infantum*	62	70	131
LinJ01_V3.0030	*L. infantum*	43	312	354
LinJ01_V3.0030	*L. infantum*	128	448	575
LinJ01_V3.0030	*L. infantum*	44	630	673
LinJ01_V3.0060	*L. infantum*	112	138	249
LinJ01_V3.0070	*L. infantum*	68	75	142
LinJ01_V3.0070	*L. infantum*	72	221	292
LinJ01_V3.0070	*L. infantum*	93	487	579
LinJ01_V3.0090	*L. infantum*	46	187	232
LinJ01_V3.0110	*L. infantum*	74	19	92
LinJ01_V3.0120	*L. infantum*	153	78	230
LinJ01_V3.0120	*L. infantum*	48	254	301
LinJ01_V3.0120	*L. infantum*	338	320	657
LinJ01_V3.0130	*L. infantum*	46	1	46
LinJ01_V3.0130	*L. infantum*	187	178	364
LinJ01_V3.0150	*L. infantum*	42	63	104
LinJ01_V3.0170	*L. infantum*	220	1	220
LinJ01_V3.0170	*L. infantum*	110	348	457
LinJ01_V3.0180	*L. infantum*	46	725	770
LinJ01_V3.0190	*L. infantum*	69	30	98
LinJ01_V3.0190	*L. infantum*	74	134	207
LinJ01_V3.0200	*L. infantum*	93	94	186
LinJ01_V3.0210	*L. infantum*	105	1	105
LinJ01_V3.0210	*L. infantum*	159	183	341
LinJ01_V3.0210	*L. infantum*	110	372	481
LinJ01_V3.0210	*L. infantum*	55	672	726
LinJ01_V3.0220	*L. infantum*	53	54	106
LinJ01_V3.0240	*L. infantum*	51	481	531
LinJ01_V3.0240	*L. infantum*	131	569	699
LinJ01_V3.0240	*L. infantum*	131	569	699
LinJ01_V3.0250	*L. infantum*	45	1	45
LinJ01_V3.0250	*L. infantum*	184	139	322
LinJ01_V3.0250	*L. infantum*	308	348	655
LinJ01_V3.0260	*L. infantum*	281	1	281
LinJ01_V3.0280	*L. infantum*	42	13	54
LinJ01_V3.0280	*L. infantum*	40	126	165
LinJ01_V3.0280	*L. infantum*	40	126	165
LinJ01_V3.0290	*L. infantum*	54	1	54
LinJ01_V3.0360	*L. infantum*	133	1	133

LinJ01_V3.0370	*L. infantum*	221	372	592
LinJ01_V3.0370	*L. infantum*	137	612	748
LinJ01_V3.0380	*L. infantum*	61	1	61
LinJ01_V3.0380	*L. infantum*	49	66	114
LinJ01_V3.0380	*L. infantum*	45	145	189
LinJ01_V3.0400	*L. infantum*	105	660	764
LinJ01_V3.0410	*L. infantum*	91	9	99
LinJ01_V3.0410	*L. infantum*	131	282	412
LinJ01_V3.0410	*L. infantum*	78	542	619
LinJ01_V3.0420	*L. infantum*	69	1	69
LinJ01_V3.0420	*L. infantum*	58	145	202
LinJ01_V3.0420	*L. infantum*	162	290	451
LinJ01_V3.0430	*L. infantum*	42	1	42
LinJ01_V3.0440	*L. infantum*	42	1	42
LinJ01_V3.0450	*L. infantum*	72	53	124
LinJ01_V3.0450	*L. infantum*	133	239	371
LinJ01_V3.0450	*L. infantum*	84	492	575
LinJ01_V3.0450	*L. infantum*	60	577	636
LinJ01_V3.0450	*L. infantum*	64	681	744
LinJ01_V3.0460	*L. infantum*	56	269	324
LinJ01_V3.0480	*L. infantum*	341	1	341
LinJ01_V3.0480	*L. infantum*	46	772	817
LinJ01_V3.0480	*L. infantum*	220	920	1139
LinJ01_V3.0480	*L. infantum*	276	1163	1438
LinJ01_V3.0550	*L. infantum*	91	18	108
LinJ01_V3.0550	*L. infantum*	177	143	319
LinJ01_V3.0550	*L. infantum*	75	405	479
LinJ01_V3.0550	*L. infantum*	72	517	588
LinJ01_V3.0550	*L. infantum*	386	667	1052
LinJ01_V3.0550	*L. infantum*	48	1100	1147
LinJ01_V3.0550	*L. infantum*	50	1497	1546
LinJ01_V3.0550	*L. infantum*	47	1593	1639
LinJ01_V3.0560	*L. infantum*	102	1	102
LinJ01_V3.0560	*L. infantum*	114	161	274
LinJ01_V3.0560	*L. infantum*	107	349	455
LinJ01_V3.0560	*L. infantum*	183	460	642
LinJ01_V3.0570	*L. infantum*	81	87	167
LinJ01_V3.0570	*L. infantum*	92	196	287
LinJ01_V3.0580	*L. infantum*	235	1	235
LinJ01_V3.0580	*L. infantum*	80	311	390
LinJ01_V3.0600	*L. infantum*	126	1	126
LinJ01_V3.0600	*L. infantum*	76	287	362
LinJ01_V3.0600	*L. infantum*	63	508	570
LinJ01_V3.0630	*L. infantum*	48	158	205
LinJ01_V3.0640	*L. infantum*	55	1	55
LinJ01_V3.0640	*L. infantum*	50	134	183
LinJ01_V3.0640	*L. infantum*	149	245	393

LinJ01_V3.0640	*L. infantum*	41	408	448
LinJ01_V3.0650	*L. infantum*	177	1	177
LinJ01_V3.0650	*L. infantum*	56	208	263
LinJ01_V3.0650	*L. infantum*	56	265	320
LinJ01_V3.0650	*L. infantum*	561	342	902
LinJ01_V3.0660	*L. infantum*	42	1	42
LinJ01_V3.0660	*L. infantum*	78	146	223
LinJ01_V3.0660	*L. infantum*	70	321	390
LinJ01_V3.0660	*L. infantum*	93	882	974
LinJ01_V3.0680	*L. infantum*	301	1	301
LinJ01_V3.0680	*L. infantum*	109	343	451
LinJ01_V3.0680	*L. infantum*	77	510	586
LinJ01_V3.0680	*L. infantum*	68	760	827
LinJ01_V3.0680	*L. infantum*	51	882	932
LinJ01_V3.0680	*L. infantum*	209	949	1157
LinJ01_V3.0680	*L. infantum*	59	1376	1434
LinJ01_V3.0690	*L. infantum*	84	23	106
LinJ01_V3.0690	*L. infantum*	187	116	302
LinJ01_V3.0700	*L. infantum*	113	46	158
LinJ01_V3.0700	*L. infantum*	284	183	466
LinJ01_V3.0700	*L. infantum*	308	494	801
LinJ01_V3.0720	*L. infantum*	93	32	124
LinJ01_V3.0720	*L. infantum*	83	143	225
LinJ01_V3.0720	*L. infantum*	79	332	410
LinJ01_V3.0730	*L. infantum*	51	270	320
LinJ01_V3.0740	*L. infantum*	79	35	113
LinJ01_V3.0740	*L. infantum*	44	435	478
LinJ01_V3.0740	*L. infantum*	56	507	562
LinJ01_V3.0740	*L. infantum*	77	857	933
LinJ01_V3.0740	*L. infantum*	49	1149	1197
LinJ01_V3.0740	*L. infantum*	62	1242	1303
LinJ01_V3.0750	*L. infantum*	80	1	80
LinJ01_V3.0750	*L. infantum*	186	127	312
LinJ01_V3.0750	*L. infantum*	115	415	529
LinJ01_V3.0750	*L. infantum*	80	733	812
LinJ01_V3.0750	*L. infantum*	65	923	987
LinJ01_V3.0750	*L. infantum*	102	1003	1104
LinJ01_V3.0750	*L. infantum*	77	1331	1407
LinJ01_V3.0750	*L. infantum*	116	1785	1900
LinJ01_V3.0750	*L. infantum*	101	2194	2294
LinJ01_V3.0760	*L. infantum*	147	119	265
LinJ01_V3.0760	*L. infantum*	180	386	565
LinJ01_V3.0780	*L. infantum*	54	48	101
LinJ01_V3.0810	*L. infantum*	162	1	162
LinJ01_V3.0810	*L. infantum*	218	175	392
LinJ01_V3.0810	*L. infantum*	66	397	462
LinJ01_V3.0820	*L. infantum*	212	1	212

LinJ01_V3.0820	*L. infantum*	108	383	490
LinJ01_V3.0830	*L. infantum*	174	551	724
LinJ01_V3.0840	*L. infantum*	82	89	170
LinJ01_V3.0840	*L. infantum*	140	815	954
LinJ02_V3.0030	*L. infantum*	40	83	122
LinJ02_V3.0040	*L. infantum*	64	334	397
LinJ02_V3.0050	*L. infantum*	51	1	51
LinJ02_V3.0050	*L. infantum*	44	777	820
LinJ02_V3.0050	*L. infantum*	63	931	993
LinJ02_V3.0060	*L. infantum*	139	1	139
LinJ02_V3.0070	*L. infantum*	128	27	154
LinJ02_V3.0070	*L. infantum*	52	303	354
LinJ02_V3.0080	*L. infantum*	150	27	176
LinJ02_V3.0080	*L. infantum*	258	207	464
LinJ02_V3.0080	*L. infantum*	93	691	783
LinJ02_V3.0080	*L. infantum*	78	841	918
LinJ02_V3.0090	*L. infantum*	120	715	834
LinJ02_V3.0100	*L. infantum*	143	108	250
LinJ02_V3.0100	*L. infantum*	41	513	553
LinJ02_V3.0100	*L. infantum*	135	643	777
LinJ02_V3.0100	*L. infantum*	42	922	963
LinJ02_V3.0100	*L. infantum*	41	1108	1148
LinJ02_V3.0100	*L. infantum*	41	1189	1229
LinJ02_V3.0100	*L. infantum*	115	1270	1384
LinJ02_V3.0100	*L. infantum*	72	1414	1485
LinJ02_V3.0100	*L. infantum*	76	2052	2127
LinJ02_V3.0100	*L. infantum*	44	2173	2216
LinJ02_V3.0100	*L. infantum*	76	2610	2685
LinJ02_V3.0100	*L. infantum*	45	2719	2763
LinJ02_V3.0100	*L. infantum*	51	2932	2982
LinJ02_V3.0100	*L. infantum*	106	3033	3138
LinJ02_V3.0100	*L. infantum*	40	3286	3325
LinJ02_V3.0100	*L. infantum*	116	3518	3633
LinJ02_V3.0100	*L. infantum*	57	3656	3712
LinJ02_V3.0100	*L. infantum*	70	3856	3925
LinJ02_V3.0100	*L. infantum*	80	4122	4201
LinJ02_V3.0100	*L. infantum*	44	4823	4866
LinJ02_V3.0110	*L. infantum*	64	55	118
LinJ02_V3.0120	*L. infantum*	52	83	134
LinJ02_V3.0120	*L. infantum*	41	301	341
LinJ02_V3.0120	*L. infantum*	93	480	572
LinJ02_V3.0130	*L. infantum*	257	76	332
LinJ02_V3.0130	*L. infantum*	41	348	388
LinJ02_V3.0130	*L. infantum*	72	479	550
LinJ02_V3.0130	*L. infantum*	110	574	683
LinJ02_V3.0130	*L. infantum*	41	749	789
LinJ02_V3.0130	*L. infantum*	104	798	901

LinJ02_V3.0130	*L. infantum*	46	955	1000
LinJ02_V3.0130	*L. infantum*	81	1110	1190
LinJ02_V3.0130	*L. infantum*	60	1447	1506
LinJ02_V3.0130	*L. infantum*	124	1802	1925
LinJ02_V3.0140	*L. infantum*	66	34	99
LinJ02_V3.0140	*L. infantum*	45	346	390
LinJ02_V3.0140	*L. infantum*	45	346	390
LinJ02_V3.0160	*L. infantum*	45	165	209
LinJ02_V3.0180	*L. infantum*	75	46	120
LinJ02_V3.0180	*L. infantum*	45	374	418
LinJ02_V3.0180	*L. infantum*	44	588	631
LinJ02_V3.0190	*L. infantum*	47	1	47
LinJ02_V3.0190	*L. infantum*	43	427	469
LinJ02_V3.0190	*L. infantum*	43	427	469
LinJ02_V3.0200	*L. infantum*	130	1	130
LinJ02_V3.0200	*L. infantum*	53	140	192
LinJ02_V3.0200	*L. infantum*	44	340	383
LinJ02_V3.0200	*L. infantum*	52	733	784
LinJ02_V3.0210	*L. infantum*	80	100	179
LinJ02_V3.0210	*L. infantum*	57	228	284
LinJ02_V3.0210	*L. infantum*	41	334	374
LinJ02_V3.0220	*L. infantum*	47	47	93
LinJ02_V3.0220	*L. infantum*	41	606	646
LinJ02_V3.0220	*L. infantum*	105	654	758
LinJ02_V3.0220	*L. infantum*	41	955	995
LinJ02_V3.0220	*L. infantum*	43	1034	1076
LinJ02_V3.0220	*L. infantum*	48	1099	1146
LinJ02_V3.0230	*L. infantum*	88	187	274
LinJ02_V3.0250	*L. infantum*	97	73	169
LinJ02_V3.0260	*L. infantum*	121	10	130
LinJ02_V3.0260	*L. infantum*	43	452	494
LinJ02_V3.0260	*L. infantum*	43	452	494
LinJ02_V3.0270	*L. infantum*	402	1	402
LinJ02_V3.0270	*L. infantum*	84	771	854
LinJ02_V3.0270	*L. infantum*	141	1109	1249
LinJ02_V3.0270	*L. infantum*	212	1515	1726
LinJ02_V3.0270	*L. infantum*	100	2177	2276
LinJ02_V3.0270	*L. infantum*	45	2620	2664
LinJ02_V3.0280	*L. infantum*	147	1	147
LinJ02_V3.0280	*L. infantum*	92	182	273
LinJ02_V3.0280	*L. infantum*	108	401	508
LinJ02_V3.0290	*L. infantum*	85	168	252
LinJ02_V3.0300	*L. infantum*	56	1	56
LinJ02_V3.0300	*L. infantum*	102	86	187
LinJ02_V3.0300	*L. infantum*	49	469	517
LinJ02_V3.0300	*L. infantum*	80	565	644
LinJ02_V3.0300	*L. infantum*	44	989	1032

LinJ02_V3.0300	*L. infantum*	158	1192	1349
LinJ02_V3.0300	*L. infantum*	83	1378	1460
LinJ02_V3.0310	*L. infantum*	46	1	46
LinJ02_V3.0310	*L. infantum*	120	106	225
LinJ02_V3.0310	*L. infantum*	154	291	444
LinJ02_V3.0310	*L. infantum*	95	540	634
LinJ02_V3.0310	*L. infantum*	82	678	759
LinJ02_V3.0310	*L. infantum*	63	826	888
LinJ02_V3.0320	*L. infantum*	311	1	311
LinJ02_V3.0320	*L. infantum*	57	335	391
LinJ02_V3.0320	*L. infantum*	52	413	464
LinJ02_V3.0320	*L. infantum*	70	686	755
LinJ02_V3.0320	*L. infantum*	141	886	1026
LinJ02_V3.0340	*L. infantum*	55	475	529
LinJ02_V3.0350	*L. infantum*	62	263	324
LinJ02_V3.0350	*L. infantum*	73	385	457
LinJ02_V3.0380	*L. infantum*	121	51	171
LinJ02_V3.0380	*L. infantum*	56	208	263
LinJ02_V3.0380	*L. infantum*	200	265	464
LinJ02_V3.0380	*L. infantum*	141	561	701
LinJ02_V3.0380	*L. infantum*	70	735	804
LinJ02_V3.0380	*L. infantum*	74	900	973
LinJ02_V3.0390	*L. infantum*	95	203	297
LinJ02_V3.0390	*L. infantum*	67	397	463
LinJ02_V3.0390	*L. infantum*	157	515	671
LinJ02_V3.0390	*L. infantum*	48	943	990
LinJ02_V3.0400	*L. infantum*	48	87	134
LinJ02_V3.0440	*L. infantum*	294	1	294
LinJ02_V3.0440	*L. infantum*	250	320	569
LinJ02_V3.0440	*L. infantum*	384	620	1003
LinJ02_V3.0440	*L. infantum*	65	1027	1091
LinJ02_V3.0440	*L. infantum*	167	1154	1320
LinJ02_V3.0440	*L. infantum*	487	1322	1808
LinJ02_V3.0440	*L. infantum*	482	1838	2319
LinJ02_V3.0440	*L. infantum*	390	2349	2738
LinJ02_V3.0440	*L. infantum*	493	2764	3256
LinJ02_V3.0440	*L. infantum*	168	3267	3434
LinJ02_V3.0440	*L. infantum*	128	3456	3583
LinJ02_V3.0450	*L. infantum*	152	1	152
LinJ02_V3.0460	*L. infantum*	100	82	181
LinJ02_V3.0460	*L. infantum*	72	252	323
LinJ02_V3.0460	*L. infantum*	74	400	473
LinJ02_V3.0460	*L. infantum*	166	496	661
LinJ02_V3.0470	*L. infantum*	100	1	100
LinJ02_V3.0470	*L. infantum*	56	135	190
LinJ02_V3.0470	*L. infantum*	62	512	573
LinJ02_V3.0480	*L. infantum*	170	1	170

LinJ02_V3.0490	*L. infantum*	250	1	250
LinJ02_V3.0490	*L. infantum*	141	286	426
LinJ02_V3.0490	*L. infantum*	810	434	1243
LinJ02_V3.0490	*L. infantum*	601	1251	1851
LinJ02_V3.0490	*L. infantum*	206	1886	2091
LinJ02_V3.0500	*L. infantum*	131	64	194
LinJ02_V3.0500	*L. infantum*	106	225	330
LinJ02_V3.0500	*L. infantum*	46	366	411
LinJ02_V3.0500	*L. infantum*	86	459	544
LinJ02_V3.0500	*L. infantum*	174	548	721
LinJ02_V3.0500	*L. infantum*	42	728	769
LinJ02_V3.0510	*L. infantum*	44	214	257
LinJ02_V3.0520	*L. infantum*	73	33	105
LinJ02_V3.0530	*L. infantum*	65	1	65
LinJ02_V3.0530	*L. infantum*	50	324	373
LinJ02_V3.0530	*L. infantum*	84	439	522
LinJ02_V3.0530	*L. infantum*	254	594	847
LinJ02_V3.0540	*L. infantum*	59	205	263
LinJ02_V3.0540	*L. infantum*	164	272	435
LinJ02_V3.0540	*L. infantum*	46	558	603
LinJ02_V3.0540	*L. infantum*	51	627	677
LinJ02_V3.0540	*L. infantum*	70	912	981
LinJ02_V3.0560	*L. infantum*	137	1	137
LinJ02_V3.0560	*L. infantum*	90	256	345
LinJ02_V3.0560	*L. infantum*	170	355	524
LinJ02_V3.0580	*L. infantum*	69	1	69
LinJ02_V3.0580	*L. infantum*	191	106	296
LinJ02_V3.0590	*L. infantum*	126	1	126
LinJ02_V3.0590	*L. infantum*	43	268	310
LinJ02_V3.0590	*L. infantum*	43	268	310
LinJ02_V3.0600	*L. infantum*	242	1	242
LinJ02_V3.0600	*L. infantum*	45	288	332
LinJ02_V3.0610	*L. infantum*	97	83	179
LinJ02_V3.0610	*L. infantum*	160	262	421
LinJ02_V3.0610	*L. infantum*	82	536	617
LinJ02_V3.0610	*L. infantum*	60	1088	1147
LinJ02_V3.0610	*L. infantum*	49	1213	1261
LinJ02_V3.0610	*L. infantum*	46	1359	1404
LinJ02_V3.0620	*L. infantum*	51	1	51
LinJ02_V3.0620	*L. infantum*	86	122	207
LinJ02_V3.0630	*L. infantum*	239	1	239
LinJ02_V3.0630	*L. infantum*	268	252	519
LinJ02_V3.0630	*L. infantum*	140	771	910
LinJ02_V3.0660	*L. infantum*	263	1	263
LinJ02_V3.0660	*L. infantum*	40	275	314
LinJ02_V3.0660	*L. infantum*	96	345	440
LinJ02_V3.0670	*L. infantum*	53	1	53

LinJ02_V3.0670	*L. infantum*	166	112	277
LinJ02_V3.0670	*L. infantum*	56	287	342
LinJ02_V3.0670	*L. infantum*	99	350	448
LinJ02_V3.0670	*L. infantum*	120	595	714
LinJ02_V3.0670	*L. infantum*	158	779	936
LinJ02_V3.0680	*L. infantum*	99	1	99
LinJ02_V3.0680	*L. infantum*	88	335	422
LinJ02_V3.0680	*L. infantum*	88	335	422
LinJ02_V3.0700	*L. infantum*	56	97	152
LinJ02_V3.0710	*L. infantum*	45	139	183
LinJ02_V3.0710	*L. infantum*	41	277	317
LinJ02_V3.0710	*L. infantum*	41	277	317
LinJ02_V3.0720	*L. infantum*	254	103	356
LinJ02_V3.0720	*L. infantum*	40	381	420
LinJ02_V3.0740	*L. infantum*	263	1	263
LinJ02_V3.0740	*L. infantum*	40	275	314
LinJ02_V3.0740	*L. infantum*	96	345	440
LinJ03_V3.0020	*L. infantum*	64	43	106
LinJ03_V3.0080	*L. infantum*	51	86	136
LinJ03_V3.0080	*L. infantum*	46	522	567
LinJ03_V3.0080	*L. infantum*	46	522	567
LinJ03_V3.0100	*L. infantum*	349	169	517
LinJ03_V3.0100	*L. infantum*	87	597	683
LinJ03_V3.0100	*L. infantum*	52	704	755
LinJ03_V3.0110	*L. infantum*	493	202	694
LinJ03_V3.0110	*L. infantum*	59	703	761
LinJ03_V3.0110	*L. infantum*	146	780	925
LinJ03_V3.0130	*L. infantum*	94	46	139
LinJ03_V3.0150	*L. infantum*	95	91	185
LinJ03_V3.0150	*L. infantum*	42	438	479
LinJ03_V3.0150	*L. infantum*	42	438	479
LinJ03_V3.0160	*L. infantum*	61	43	103
LinJ03_V3.0160	*L. infantum*	75	117	191
LinJ03_V3.0170	*L. infantum*	317	100	416
LinJ03_V3.0170	*L. infantum*	55	578	632
LinJ03_V3.0170	*L. infantum*	59	669	727
LinJ03_V3.0180	*L. infantum*	48	163	210
LinJ03_V3.0210	*L. infantum*	45	45	89
LinJ03_V3.0250	*L. infantum*	77	36	112
LinJ03_V3.0250	*L. infantum*	79	126	204
LinJ03_V3.0250	*L. infantum*	79	126	204
LinJ03_V3.0260	*L. infantum*	69	84	152
LinJ03_V3.0260	*L. infantum*	1875	169	2043
LinJ03_V3.0260	*L. infantum*	69	2075	2143
LinJ03_V3.0270	*L. infantum*	110	1	110
LinJ03_V3.0270	*L. infantum*	164	128	291
LinJ03_V3.0270	*L. infantum*	125	339	463

LinJ03_V3.0270	*L. infantum*	87	651	737
LinJ03_V3.0270	*L. infantum*	117	795	911
LinJ03_V3.0270	*L. infantum*	56	913	968
LinJ03_V3.0300	*L. infantum*	180	1	180
LinJ03_V3.0300	*L. infantum*	451	185	635
LinJ03_V3.0300	*L. infantum*	109	768	876
LinJ03_V3.0300	*L. infantum*	163	881	1043
LinJ03_V3.0310	*L. infantum*	41	1	41
LinJ03_V3.0310	*L. infantum*	82	193	274
LinJ03_V3.0310	*L. infantum*	89	311	399
LinJ03_V3.0310	*L. infantum*	48	583	630
LinJ03_V3.0320	*L. infantum*	233	30	262
LinJ03_V3.0330	*L. infantum*	282	1	282
LinJ03_V3.0330	*L. infantum*	63	348	410
LinJ03_V3.0330	*L. infantum*	275	458	732
LinJ03_V3.0330	*L. infantum*	83	973	1055
LinJ03_V3.0330	*L. infantum*	89	1085	1173
LinJ03_V3.0330	*L. infantum*	208	1247	1454
LinJ03_V3.0350	*L. infantum*	51	1	51
LinJ03_V3.0350	*L. infantum*	71	53	123
LinJ03_V3.0350	*L. infantum*	71	53	123
LinJ03_V3.0360	*L. infantum*	75	1	75
LinJ03_V3.0360	*L. infantum*	53	78	130
LinJ03_V3.0370	*L. infantum*	57	37	93
LinJ03_V3.0370	*L. infantum*	43	206	248
LinJ03_V3.0370	*L. infantum*	43	206	248
LinJ03_V3.0390	*L. infantum*	81	1	81
LinJ03_V3.0390	*L. infantum*	108	285	392
LinJ03_V3.0390	*L. infantum*	108	285	392
LinJ03_V3.0400	*L. infantum*	168	1	168
LinJ03_V3.0400	*L. infantum*	57	184	240
LinJ03_V3.0400	*L. infantum*	293	250	542
LinJ03_V3.0400	*L. infantum*	43	576	618
LinJ03_V3.0400	*L. infantum*	56	714	769
LinJ03_V3.0400	*L. infantum*	51	906	956
LinJ03_V3.0400	*L. infantum*	267	1246	1512
LinJ03_V3.0400	*L. infantum*	80	1592	1671
LinJ03_V3.0410	*L. infantum*	67	46	112
LinJ03_V3.0420	*L. infantum*	61	51	111
LinJ03_V3.0430	*L. infantum*	52	1	52
LinJ03_V3.0430	*L. infantum*	118	104	221
LinJ03_V3.0430	*L. infantum*	102	254	355
LinJ03_V3.0430	*L. infantum*	82	411	492
LinJ03_V3.0430	*L. infantum*	46	672	717
LinJ03_V3.0430	*L. infantum*	84	794	877
LinJ03_V3.0430	*L. infantum*	198	920	1117
LinJ03_V3.0430	*L. infantum*	44	1186	1229

LinJ03_V3.0430	*L. infantum*	63	1285	1347
LinJ03_V3.0430	*L. infantum*	47	1371	1417
LinJ03_V3.0430	*L. infantum*	76	1465	1540
LinJ03_V3.0440	*L. infantum*	388	1	388
LinJ03_V3.0440	*L. infantum*	110	424	533
LinJ03_V3.0440	*L. infantum*	70	562	631
LinJ03_V3.0440	*L. infantum*	109	648	756
LinJ03_V3.0440	*L. infantum*	52	765	816
LinJ03_V3.0440	*L. infantum*	227	834	1060
LinJ03_V3.0450	*L. infantum*	106	49	154
LinJ03_V3.0450	*L. infantum*	43	231	273
LinJ03_V3.0450	*L. infantum*	197	347	543
LinJ03_V3.0450	*L. infantum*	84	874	957
LinJ03_V3.0460	*L. infantum*	40	348	387
LinJ03_V3.0470	*L. infantum*	83	11	93
LinJ03_V3.0470	*L. infantum*	437	130	566
LinJ03_V3.0470	*L. infantum*	77	612	688
LinJ03_V3.0470	*L. infantum*	151	959	1109
LinJ03_V3.0480	*L. infantum*	119	252	370
LinJ03_V3.0490	*L. infantum*	61	48	108
LinJ03_V3.0490	*L. infantum*	210	124	333
LinJ03_V3.0490	*L. infantum*	154	361	514
LinJ03_V3.0490	*L. infantum*	729	528	1256
LinJ03_V3.0490	*L. infantum*	271	1281	1551
LinJ03_V3.0490	*L. infantum*	107	1575	1681
LinJ03_V3.0490	*L. infantum*	117	1744	1860
LinJ03_V3.0490	*L. infantum*	65	1893	1957
LinJ03_V3.0490	*L. infantum*	48	1983	2030
LinJ03_V3.0490	*L. infantum*	194	2117	2310
LinJ03_V3.0490	*L. infantum*	49	2393	2441
LinJ03_V3.0490	*L. infantum*	60	2541	2600
LinJ03_V3.0500	*L. infantum*	357	1	357
LinJ03_V3.0500	*L. infantum*	49	541	589
LinJ03_V3.0500	*L. infantum*	51	661	711
LinJ03_V3.0500	*L. infantum*	89	769	857
LinJ03_V3.0510	*L. infantum*	182	1	182
LinJ03_V3.0510	*L. infantum*	43	213	255
LinJ03_V3.0510	*L. infantum*	70	257	326
LinJ03_V3.0510	*L. infantum*	72	380	451
LinJ03_V3.0510	*L. infantum*	69	551	619
LinJ03_V3.0510	*L. infantum*	41	897	937
LinJ03_V3.0510	*L. infantum*	90	1065	1154
LinJ03_V3.0510	*L. infantum*	116	1203	1318
LinJ03_V3.0510	*L. infantum*	71	1360	1430
LinJ03_V3.0510	*L. infantum*	51	1480	1530
LinJ03_V3.0510	*L. infantum*	97	1568	1664
LinJ03_V3.0510	*L. infantum*	81	1755	1835

LinJ03_V3.0510	*L. infantum*	132	1892	2023
LinJ03_V3.0520	*L. infantum*	49	1	49
LinJ03_V3.0530	*L. infantum*	125	1	125
LinJ03_V3.0530	*L. infantum*	58	271	328
LinJ03_V3.0530	*L. infantum*	72	464	535
LinJ03_V3.0530	*L. infantum*	56	570	625
LinJ03_V3.0530	*L. infantum*	341	735	1075
LinJ03_V3.0530	*L. infantum*	91	1278	1368
LinJ03_V3.0540	*L. infantum*	87	1	87
LinJ03_V3.0540	*L. infantum*	276	118	393
LinJ03_V3.0540	*L. infantum*	69	470	538
LinJ03_V3.0540	*L. infantum*	46	572	617
LinJ03_V3.0560	*L. infantum*	99	80	178
LinJ03_V3.0570	*L. infantum*	98	15	112
LinJ03_V3.0570	*L. infantum*	230	128	357
LinJ03_V3.0570	*L. infantum*	80	467	546
LinJ03_V3.0570	*L. infantum*	73	721	793
LinJ03_V3.0570	*L. infantum*	55	987	1041
LinJ03_V3.0570	*L. infantum*	76	1105	1180
LinJ03_V3.0580	*L. infantum*	58	1	58
LinJ03_V3.0600	*L. infantum*	53	136	188
LinJ03_V3.0600	*L. infantum*	44	551	594
LinJ03_V3.0600	*L. infantum*	44	551	594
LinJ03_V3.0610	*L. infantum*	82	67	148
LinJ03_V3.0620	*L. infantum*	57	55	111
LinJ03_V3.0620	*L. infantum*	55	214	268
LinJ03_V3.0630	*L. infantum*	198	1	198
LinJ03_V3.0630	*L. infantum*	65	223	287
LinJ03_V3.0630	*L. infantum*	82	569	650
LinJ03_V3.0630	*L. infantum*	80	810	889
LinJ03_V3.0640	*L. infantum*	183	10	192
LinJ03_V3.0640	*L. infantum*	145	440	584
LinJ03_V3.0670	*L. infantum*	40	29	68
LinJ03_V3.0670	*L. infantum*	60	282	341
LinJ03_V3.0670	*L. infantum*	88	683	770
LinJ03_V3.0670	*L. infantum*	52	1447	1498
LinJ03_V3.0670	*L. infantum*	66	1551	1616
LinJ03_V3.0680	*L. infantum*	232	68	299
LinJ03_V3.0680	*L. infantum*	113	407	519
LinJ03_V3.0680	*L. infantum*	43	534	576
LinJ03_V3.0680	*L. infantum*	90	599	688
LinJ03_V3.0680	*L. infantum*	77	698	774
LinJ03_V3.0680	*L. infantum*	79	901	979
LinJ03_V3.0680	*L. infantum*	68	991	1058
LinJ03_V3.0680	*L. infantum*	62	1137	1198
LinJ03_V3.0680	*L. infantum*	45	1350	1394
LinJ03_V3.0680	*L. infantum*	67	1442	1508

LinJ03_V3.0680	*L. infantum*	88	1724	1811
LinJ03_V3.0680	*L. infantum*	60	1937	1996
LinJ03_V3.0680	*L. infantum*	131	2154	2284
LinJ03_V3.0680	*L. infantum*	48	2328	2375
LinJ03_V3.0680	*L. infantum*	111	2399	2509
LinJ03_V3.0680	*L. infantum*	69	2602	2670
LinJ03_V3.0690	*L. infantum*	63	46	108
LinJ03_V3.0700	*L. infantum*	168	27	194
LinJ03_V3.0700	*L. infantum*	62	235	296
LinJ03_V3.0700	*L. infantum*	53	310	362
LinJ03_V3.0700	*L. infantum*	147	647	793
LinJ03_V3.0700	*L. infantum*	89	1011	1099
LinJ03_V3.0710	*L. infantum*	62	1	62
LinJ03_V3.0710	*L. infantum*	71	135	205
LinJ03_V3.0710	*L. infantum*	66	303	368
LinJ03_V3.0730	*L. infantum*	79	1	79
LinJ03_V3.0730	*L. infantum*	51	365	415
LinJ03_V3.0730	*L. infantum*	208	509	716
LinJ03_V3.0740	*L. infantum*	91	1	91
LinJ03_V3.0740	*L. infantum*	49	150	198
LinJ03_V3.0760	*L. infantum*	48	1	48
LinJ03_V3.0760	*L. infantum*	140	115	254
LinJ03_V3.0760	*L. infantum*	54	268	321
LinJ03_V3.0760	*L. infantum*	57	323	379
LinJ03_V3.0760	*L. infantum*	65	460	524
LinJ03_V3.0760	*L. infantum*	1103	553	1655
LinJ03_V3.0760	*L. infantum*	45	1669	1713
LinJ03_V3.0760	*L. infantum*	310	1723	2032
LinJ03_V3.0770	*L. infantum*	297	1	297
LinJ03_V3.0770	*L. infantum*	283	410	692
LinJ03_V3.0780	*L. infantum*	95	21	115
LinJ03_V3.0780	*L. infantum*	172	219	390
LinJ03_V3.0790	*L. infantum*	696	1	696
LinJ03_V3.0790	*L. infantum*	113	698	810
LinJ03_V3.0790	*L. infantum*	350	949	1298
LinJ03_V3.0790	*L. infantum*	65	1300	1364
LinJ03_V3.0790	*L. infantum*	132	1374	1505
LinJ03_V3.0790	*L. infantum*	58	1519	1576
LinJ03_V3.0790	*L. infantum*	117	1592	1708
LinJ03_V3.0790	*L. infantum*	96	2016	2111
LinJ03_V3.0790	*L. infantum*	46	2150	2195
LinJ03_V3.0790	*L. infantum*	68	2326	2393
LinJ03_V3.0800	*L. infantum*	329	1	329
LinJ03_V3.0800	*L. infantum*	55	352	406
LinJ03_V3.0800	*L. infantum*	123	567	689
LinJ03_V3.0800	*L. infantum*	70	743	812
LinJ03_V3.0800	*L. infantum*	48	898	945

LinJ03_V3.0810	*L. infantum*	624	1	624
LinJ03_V3.0810	*L. infantum*	134	634	767
LinJ03_V3.0810	*L. infantum*	890	790	1679
LinJ03_V3.0810	*L. infantum*	100	1821	1920
LinJ03_V3.0810	*L. infantum*	182	2006	2187
LinJ03_V3.0810	*L. infantum*	259	2213	2471
LinJ03_V3.0810	*L. infantum*	135	2473	2607
LinJ03_V3.0810	*L. infantum*	290	2615	2904
LinJ03_V3.0820	*L. infantum*	104	56	159
LinJ03_V3.0830	*L. infantum*	101	1	101
LinJ03_V3.0830	*L. infantum*	349	117	465
LinJ03_V3.0830	*L. infantum*	187	467	653
LinJ03_V3.0830	*L. infantum*	190	680	869
LinJ03_V3.0830	*L. infantum*	158	881	1038
LinJ03_V3.0830	*L. infantum*	74	1057	1130
LinJ03_V3.0830	*L. infantum*	78	1189	1266
LinJ03_V3.0830	*L. infantum*	90	1298	1387
LinJ03_V3.0830	*L. infantum*	476	1399	1874
LinJ03_V3.0830	*L. infantum*	55	1895	1949
LinJ03_V3.0830	*L. infantum*	71	1983	2053
LinJ03_V3.0830	*L. infantum*	298	2073	2370
LinJ03_V3.0840	*L. infantum*	103	1	103
LinJ03_V3.0840	*L. infantum*	139	132	270
LinJ03_V3.0840	*L. infantum*	101	306	406
LinJ03_V3.0840	*L. infantum*	56	470	525
LinJ03_V3.0840	*L. infantum*	49	759	807
LinJ03_V3.0840	*L. infantum*	87	809	895
LinJ03_V3.0840	*L. infantum*	43	939	981
LinJ03_V3.0840	*L. infantum*	113	1116	1228
LinJ03_V3.0850	*L. infantum*	110	1	110
LinJ03_V3.0850	*L. infantum*	555	364	918
LinJ03_V3.0850	*L. infantum*	42	920	961
LinJ03_V3.0860	*L. infantum*	124	76	199
LinJ03_V3.0860	*L. infantum*	92	323	414
LinJ03_V3.0860	*L. infantum*	104	689	792
LinJ03_V3.0860	*L. infantum*	95	866	960
LinJ03_V3.0860	*L. infantum*	97	1365	1461
LinJ03_V3.0860	*L. infantum*	45	1876	1920
LinJ03_V3.0870	*L. infantum*	83	61	143
LinJ03_V3.0870	*L. infantum*	104	180	283
LinJ03_V3.0870	*L. infantum*	121	413	533
LinJ03_V3.0870	*L. infantum*	112	600	711
LinJ03_V3.0880	*L. infantum*	87	294	380
LinJ03_V3.0920	*L. infantum*	135	10	144
LinJ03_V3.0960	*L. infantum*	60	354	413
LinJ03_V3.0970	*L. infantum*	80	481	560
LinJ04_V3.0030	*L. infantum*	76	35	110

LinJ04_V3.0030	*L. infantum*	87	471	557
LinJ04_V3.0030	*L. infantum*	60	665	724
LinJ04_V3.0040	*L. infantum*	90	44	133
LinJ04_V3.0060	*L. infantum*	200	1	200
LinJ04_V3.0060	*L. infantum*	149	227	375
LinJ04_V3.0060	*L. infantum*	121	409	529
LinJ04_V3.0070	*L. infantum*	40	1	40
LinJ04_V3.0070	*L. infantum*	145	57	201
LinJ04_V3.0090	*L. infantum*	141	18	158
LinJ04_V3.0100	*L. infantum*	58	1335	1392
LinJ04_V3.0110	*L. infantum*	45	57	101
LinJ04_V3.0110	*L. infantum*	114	207	320
LinJ04_V3.0110	*L. infantum*	104	474	577
LinJ04_V3.0120	*L. infantum*	62	83	144
LinJ04_V3.0120	*L. infantum*	115	233	347
LinJ04_V3.0140	*L. infantum*	137	22	158
LinJ04_V3.0150	*L. infantum*	82	1	82
LinJ04_V3.0150	*L. infantum*	152	122	273
LinJ04_V3.0150	*L. infantum*	55	330	384
LinJ04_V3.0160	*L. infantum*	52	49	100
LinJ04_V3.0160	*L. infantum*	60	359	418
LinJ04_V3.0160	*L. infantum*	58	437	494
LinJ04_V3.0170	*L. infantum*	71	159	229
LinJ04_V3.0170	*L. infantum*	70	352	421
LinJ04_V3.0170	*L. infantum*	70	352	421
LinJ04_V3.0180	*L. infantum*	107	385	491
LinJ04_V3.0180	*L. infantum*	70	557	626
LinJ04_V3.0180	*L. infantum*	70	557	626
LinJ04_V3.0190	*L. infantum*	107	1	107
LinJ04_V3.0200	*L. infantum*	136	114	249
LinJ04_V3.0210	*L. infantum*	62	293	354
LinJ04_V3.0220	*L. infantum*	76	104	179
LinJ04_V3.0220	*L. infantum*	74	185	258
LinJ04_V3.0230	*L. infantum*	99	1	99
LinJ04_V3.0240	*L. infantum*	99	1	99
LinJ04_V3.0240	*L. infantum*	139	362	500
LinJ04_V3.0240	*L. infantum*	58	658	715
LinJ04_V3.0240	*L. infantum*	58	794	851
LinJ04_V3.0240	*L. infantum*	44	894	937
LinJ04_V3.0240	*L. infantum*	71	1017	1087
LinJ04_V3.0240	*L. infantum*	109	1212	1320
LinJ04_V3.0250	*L. infantum*	42	1	42
LinJ04_V3.0250	*L. infantum*	71	65	135
LinJ04_V3.0250	*L. infantum*	46	223	268
LinJ04_V3.0260	*L. infantum*	91	65	155
LinJ04_V3.0260	*L. infantum*	53	235	287
LinJ04_V3.0260	*L. infantum*	53	235	287

LinJ04_V3.0270	*L. infantum*	72	70	141
LinJ04_V3.0270	*L. infantum*	45	444	488
LinJ04_V3.0270	*L. infantum*	79	799	877
LinJ04_V3.0270	*L. infantum*	81	926	1006
LinJ04_V3.0270	*L. infantum*	44	1171	1214
LinJ04_V3.0270	*L. infantum*	243	1611	1853
LinJ04_V3.0320	*L. infantum*	59	1	59
LinJ04_V3.0320	*L. infantum*	59	370	428
LinJ04_V3.0320	*L. infantum*	44	432	475
LinJ04_V3.0340	*L. infantum*	64	1	64
LinJ04_V3.0340	*L. infantum*	234	74	307
LinJ04_V3.0340	*L. infantum*	414	386	799
LinJ04_V3.0340	*L. infantum*	131	805	935
LinJ04_V3.0340	*L. infantum*	97	990	1086
LinJ04_V3.0340	*L. infantum*	203	1184	1386
LinJ04_V3.0340	*L. infantum*	52	1471	1522
LinJ04_V3.0340	*L. infantum*	108	1587	1694
LinJ04_V3.0340	*L. infantum*	46	1763	1808
LinJ04_V3.0340	*L. infantum*	51	1910	1960
LinJ04_V3.0340	*L. infantum*	50	2016	2065
LinJ04_V3.0350	*L. infantum*	106	85	190
LinJ04_V3.0370	*L. infantum*	55	188	242
LinJ04_V3.0380	*L. infantum*	50	1	50
LinJ04_V3.0380	*L. infantum*	90	176	265
LinJ04_V3.0390	*L. infantum*	483	1	483
LinJ04_V3.0390	*L. infantum*	369	522	890
LinJ04_V3.0390	*L. infantum*	77	894	970
LinJ04_V3.0390	*L. infantum*	115	985	1099
LinJ04_V3.0400	*L. infantum*	69	293	361
LinJ04_V3.0410	*L. infantum*	59	197	255
LinJ04_V3.0410	*L. infantum*	63	301	363
LinJ04_V3.0410	*L. infantum*	40	524	563
LinJ04_V3.0410	*L. infantum*	82	565	646
LinJ04_V3.0410	*L. infantum*	46	799	844
LinJ04_V3.0410	*L. infantum*	63	1067	1129
LinJ04_V3.0410	*L. infantum*	62	1275	1336
LinJ04_V3.0410	*L. infantum*	75	2902	2976
LinJ04_V3.0410	*L. infantum*	43	3022	3064
LinJ04_V3.0410	*L. infantum*	63	3757	3819
LinJ04_V3.0420	*L. infantum*	70	1	70
LinJ04_V3.0420	*L. infantum*	571	86	656
LinJ04_V3.0420	*L. infantum*	48	696	743
LinJ04_V3.0420	*L. infantum*	54	779	832
LinJ04_V3.0420	*L. infantum*	57	979	1035
LinJ04_V3.0430	*L. infantum*	144	1	144
LinJ04_V3.0440	*L. infantum*	44	1	44
LinJ04_V3.0450	*L. infantum*	43	125	167

LinJ04_V3.0450	*L. infantum*	172	242	413
LinJ04_V3.0450	*L. infantum*	65	792	856
LinJ04_V3.0450	*L. infantum*	98	880	977
LinJ04_V3.0460	*L. infantum*	50	139	188
LinJ04_V3.0480	*L. infantum*	234	90	323
LinJ04_V3.0490	*L. infantum*	573	1	573
LinJ04_V3.0490	*L. infantum*	464	577	1040
LinJ04_V3.0490	*L. infantum*	260	1125	1384
LinJ04_V3.0490	*L. infantum*	200	1424	1623
LinJ04_V3.0490	*L. infantum*	56	1747	1802
LinJ04_V3.0490	*L. infantum*	48	1823	1870
LinJ04_V3.0490	*L. infantum*	275	1908	2182
LinJ04_V3.0490	*L. infantum*	516	2308	2823
LinJ04_V3.0490	*L. infantum*	168	2835	3002
LinJ04_V3.0500	*L. infantum*	103	1	103
LinJ04_V3.0500	*L. infantum*	99	110	208
LinJ04_V3.0500	*L. infantum*	284	212	495
LinJ04_V3.0500	*L. infantum*	217	597	813
LinJ04_V3.0500	*L. infantum*	116	873	988
LinJ04_V3.0500	*L. infantum*	180	1152	1331
LinJ04_V3.0510	*L. infantum*	62	92	153
LinJ04_V3.0510	*L. infantum*	85	286	370
LinJ04_V3.0510	*L. infantum*	117	887	1003
LinJ04_V3.0520	*L. infantum*	97	1	97
LinJ04_V3.0520	*L. infantum*	112	154	265
LinJ04_V3.0520	*L. infantum*	77	271	347
LinJ04_V3.0530	*L. infantum*	140	116	255
LinJ04_V3.0530	*L. infantum*	182	411	592
LinJ04_V3.0540	*L. infantum*	71	1274	1344
LinJ04_V3.0550	*L. infantum*	112	198	309
LinJ04_V3.0560	*L. infantum*	59	32	90
LinJ04_V3.0560	*L. infantum*	47	499	545
LinJ04_V3.0560	*L. infantum*	40	591	630
LinJ04_V3.0560	*L. infantum*	44	685	728
LinJ04_V3.0560	*L. infantum*	81	731	811
LinJ04_V3.0560	*L. infantum*	63	1011	1073
LinJ04_V3.0560	*L. infantum*	186	1327	1512
LinJ04_V3.0560	*L. infantum*	52	1517	1568
LinJ04_V3.0580	*L. infantum*	58	116	173
LinJ04_V3.0590	*L. infantum*	238	239	476
LinJ04_V3.0590	*L. infantum*	67	591	657
LinJ04_V3.0590	*L. infantum*	58	833	890
LinJ04_V3.0590	*L. infantum*	188	913	1100
LinJ04_V3.0590	*L. infantum*	138	1275	1412
LinJ04_V3.0590	*L. infantum*	75	1497	1571
LinJ04_V3.0590	*L. infantum*	136	1637	1772
LinJ04_V3.0590	*L. infantum*	61	1829	1889

LinJ04_V3.0590	*L. infantum*	48	1905	1952
LinJ04_V3.0590	*L. infantum*	285	2004	2288
LinJ04_V3.0590	*L. infantum*	75	2300	2374
LinJ04_V3.0590	*L. infantum*	180	2427	2606
LinJ04_V3.0590	*L. infantum*	65	2666	2730
LinJ04_V3.0590	*L. infantum*	313	2745	3057
LinJ04_V3.0600	*L. infantum*	167	1	167
LinJ04_V3.0610	*L. infantum*	41	1	41
LinJ04_V3.0610	*L. infantum*	379	103	481
LinJ04_V3.0610	*L. infantum*	775	515	1289
LinJ04_V3.0620	*L. infantum*	144	195	338
LinJ04_V3.0650	*L. infantum*	60	1	60
LinJ04_V3.0650	*L. infantum*	45	139	183
LinJ04_V3.0650	*L. infantum*	69	219	287
LinJ04_V3.0650	*L. infantum*	81	617	697
LinJ04_V3.0650	*L. infantum*	49	744	792
LinJ04_V3.0650	*L. infantum*	135	1024	1158
LinJ04_V3.0660	*L. infantum*	41	1	41
LinJ04_V3.0670	*L. infantum*	53	1	53
LinJ04_V3.0690	*L. infantum*	85	1	85
LinJ04_V3.0690	*L. infantum*	124	118	241
LinJ04_V3.0690	*L. infantum*	48	300	347
LinJ04_V3.0690	*L. infantum*	44	492	535
LinJ04_V3.0690	*L. infantum*	94	629	722
LinJ04_V3.0710	*L. infantum*	263	72	334
LinJ04_V3.0710	*L. infantum*	117	350	466
LinJ04_V3.0710	*L. infantum*	93	595	687
LinJ04_V3.0720	*L. infantum*	125	1	125
LinJ04_V3.0720	*L. infantum*	501	144	644
LinJ04_V3.0730	*L. infantum*	46	1	46
LinJ04_V3.0730	*L. infantum*	46	74	119
LinJ04_V3.0730	*L. infantum*	61	420	480
LinJ04_V3.0740	*L. infantum*	233	115	347
LinJ04_V3.0740	*L. infantum*	374	437	810
LinJ04_V3.0760	*L. infantum*	69	74	142
LinJ04_V3.0760	*L. infantum*	68	184	251
LinJ04_V3.0760	*L. infantum*	68	184	251
LinJ04_V3.0770	*L. infantum*	48	1	48
LinJ04_V3.0770	*L. infantum*	68	90	157
LinJ04_V3.0770	*L. infantum*	68	90	157
LinJ04_V3.0780	*L. infantum*	49	230	278
LinJ04_V3.0780	*L. infantum*	40	375	414
LinJ04_V3.0780	*L. infantum*	81	496	576
LinJ04_V3.0790	*L. infantum*	77	344	420
LinJ04_V3.0790	*L. infantum*	693	425	1117
LinJ04_V3.0800	*L. infantum*	142	27	168
LinJ04_V3.0800	*L. infantum*	90	202	291

LinJ04_V3.0810	*L. infantum*	61	22	82
LinJ04_V3.0810	*L. infantum*	212	227	438
LinJ04_V3.0820	*L. infantum*	43	59	101
LinJ04_V3.0820	*L. infantum*	55	339	393
LinJ04_V3.0820	*L. infantum*	55	339	393
LinJ04_V3.0830	*L. infantum*	625	1	625
LinJ04_V3.0830	*L. infantum*	208	661	868
LinJ04_V3.0830	*L. infantum*	410	880	1289
LinJ04_V3.0830	*L. infantum*	97	1291	1387
LinJ04_V3.0840	*L. infantum*	193	1	193
LinJ04_V3.0840	*L. infantum*	83	382	464
LinJ04_V3.0840	*L. infantum*	206	487	692
LinJ04_V3.0850	*L. infantum*	92	1	92
LinJ04_V3.0850	*L. infantum*	58	128	185
LinJ04_V3.0860	*L. infantum*	65	202	266
LinJ04_V3.0870	*L. infantum*	454	1	454
LinJ04_V3.0870	*L. infantum*	717	468	1184
LinJ04_V3.0880	*L. infantum*	174	1	174
LinJ04_V3.0890	*L. infantum*	105	1	105
LinJ04_V3.0890	*L. infantum*	80	244	323
LinJ04_V3.0890	*L. infantum*	490	476	965
LinJ04_V3.0890	*L. infantum*	305	970	1274
LinJ04_V3.0900	*L. infantum*	966	1	966
LinJ04_V3.0900	*L. infantum*	59	1017	1075
LinJ04_V3.0900	*L. infantum*	88	1336	1423
LinJ04_V3.0900	*L. infantum*	84	1623	1706
LinJ04_V3.0900	*L. infantum*	49	1824	1872
LinJ04_V3.0910	*L. infantum*	130	65	194
LinJ04_V3.0920	*L. infantum*	85	1	85
LinJ04_V3.0920	*L. infantum*	150	141	290
LinJ04_V3.0920	*L. infantum*	72	305	376
LinJ04_V3.0940	*L. infantum*	93	1	93
LinJ04_V3.0940	*L. infantum*	132	105	236
LinJ04_V3.0940	*L. infantum*	91	271	361
LinJ04_V3.0970	*L. infantum*	303	1	303
LinJ04_V3.0970	*L. infantum*	49	440	488
LinJ04_V3.0980	*L. infantum*	83	30	112
LinJ04_V3.0980	*L. infantum*	91	167	257
LinJ04_V3.0980	*L. infantum*	167	262	428
LinJ04_V3.0990	*L. infantum*	90	1	90
LinJ04_V3.0990	*L. infantum*	162	94	255
LinJ04_V3.0990	*L. infantum*	53	291	343
LinJ04_V3.0990	*L. infantum*	55	447	501
LinJ04_V3.0990	*L. infantum*	105	747	851
LinJ04_V3.0990	*L. infantum*	57	996	1052
LinJ04_V3.0990	*L. infantum*	54	1121	1174
LinJ04_V3.1000	*L. infantum*	111	67	177

LinJ04_V3.1000	*L. infantum*	60	226	285
LinJ04_V3.1010	*L. infantum*	47	73	119
LinJ04_V3.1010	*L. infantum*	70	708	777
LinJ04_V3.1010	*L. infantum*	223	956	1178
LinJ04_V3.1020	*L. infantum*	222	1	222
LinJ04_V3.1030	*L. infantum*	55	140	194
LinJ04_V3.1050	*L. infantum*	54	303	356
LinJ04_V3.1060	*L. infantum*	93	54	146
LinJ04_V3.1060	*L. infantum*	278	208	485
LinJ04_V3.1070	*L. infantum*	56	1	56
LinJ04_V3.1080	*L. infantum*	231	1	231
LinJ04_V3.1080	*L. infantum*	41	694	734
LinJ04_V3.1080	*L. infantum*	64	813	876
LinJ04_V3.1090	*L. infantum*	76	1	76
LinJ04_V3.1100	*L. infantum*	183	1	183
LinJ04_V3.1100	*L. infantum*	697	191	887
LinJ04_V3.1100	*L. infantum*	100	939	1038
LinJ04_V3.1100	*L. infantum*	203	1105	1307
LinJ04_V3.1120	*L. infantum*	51	893	943
LinJ04_V3.1130	*L. infantum*	157	58	214
LinJ04_V3.1130	*L. infantum*	72	229	300
LinJ04_V3.1130	*L. infantum*	340	343	682
LinJ04_V3.1150	*L. infantum*	300	1	300
LinJ04_V3.1160	*L. infantum*	47	122	168
LinJ04_V3.1180	*L. infantum*	165	1	165
LinJ04_V3.1180	*L. infantum*	48	277	324
LinJ04_V3.1180	*L. infantum*	74	344	417
LinJ04_V3.1190	*L. infantum*	90	1	90
LinJ04_V3.1190	*L. infantum*	46	266	311
LinJ04_V3.1200	*L. infantum*	54	1086	1139
LinJ04_V3.1200	*L. infantum*	329	1250	1578
LinJ04_V3.1200	*L. infantum*	204	1617	1820
LinJ04_V3.1200	*L. infantum*	515	1880	2394
LinJ04_V3.1230	*L. infantum*	61	308	368
LinJ04_V3.1240	*L. infantum*	50	1	50
LinJ04_V3.1240	*L. infantum*	377	107	483
LinJ04_V3.1240	*L. infantum*	83	951	1033
LinJ05_V3.0010	*L. infantum*	78	1	78
LinJ05_V3.0010	*L. infantum*	109	80	188
LinJ05_V3.0010	*L. infantum*	248	226	473
LinJ05_V3.0010	*L. infantum*	86	526	611
LinJ05_V3.0010	*L. infantum*	147	689	835
LinJ05_V3.0020	*L. infantum*	42	18	59
LinJ05_V3.0040	*L. infantum*	368	1	368
LinJ05_V3.0040	*L. infantum*	94	380	473
LinJ05_V3.0040	*L. infantum*	48	534	581
LinJ05_V3.0050	*L. infantum*	107	45	151

LinJ05_V3.0050	*L. infantum*	83	183	265
LinJ05_V3.0050	*L. infantum*	70	364	433
LinJ05_V3.0050	*L. infantum*	79	755	833
LinJ05_V3.0060	*L. infantum*	41	69	109
LinJ05_V3.0060	*L. infantum*	157	618	774
LinJ05_V3.0060	*L. infantum*	157	618	774
LinJ05_V3.0080	*L. infantum*	115	1	115
LinJ05_V3.0080	*L. infantum*	43	159	201
LinJ05_V3.0080	*L. infantum*	43	159	201
LinJ05_V3.0100	*L. infantum*	53	226	278
LinJ05_V3.0130	*L. infantum*	78	355	432
LinJ05_V3.0140	*L. infantum*	88	1	88
LinJ05_V3.0140	*L. infantum*	63	616	678
LinJ05_V3.0160	*L. infantum*	230	11	240
LinJ05_V3.0160	*L. infantum*	230	284	513
LinJ05_V3.0170	*L. infantum*	222	1	222
LinJ05_V3.0170	*L. infantum*	529	231	759
LinJ05_V3.0180	*L. infantum*	55	121	175
LinJ05_V3.0180	*L. infantum*	74	189	262
LinJ05_V3.0180	*L. infantum*	74	189	262
LinJ05_V3.0190	*L. infantum*	44	1	44
LinJ05_V3.0190	*L. infantum*	90	117	206
LinJ05_V3.0190	*L. infantum*	145	217	361
LinJ05_V3.0200	*L. infantum*	50	1	50
LinJ05_V3.0200	*L. infantum*	125	255	379
LinJ05_V3.0200	*L. infantum*	43	583	625
LinJ05_V3.0210	*L. infantum*	45	1	45
LinJ05_V3.0210	*L. infantum*	57	59	115
LinJ05_V3.0210	*L. infantum*	54	166	219
LinJ05_V3.0210	*L. infantum*	41	406	446
LinJ05_V3.0210	*L. infantum*	50	620	669
LinJ05_V3.0210	*L. infantum*	67	1057	1123
LinJ05_V3.0210	*L. infantum*	171	1304	1474
LinJ05_V3.0220	*L. infantum*	46	1	46
LinJ05_V3.0220	*L. infantum*	464	208	671
LinJ05_V3.0230	*L. infantum*	44	88	131
LinJ05_V3.0230	*L. infantum*	46	157	202
LinJ05_V3.0230	*L. infantum*	77	366	442
LinJ05_V3.0230	*L. infantum*	53	529	581
LinJ05_V3.0240	*L. infantum*	149	217	365
LinJ05_V3.0240	*L. infantum*	482	373	854
LinJ05_V3.0260	*L. infantum*	45	100	144
LinJ05_V3.0260	*L. infantum*	110	277	386
LinJ05_V3.0270	*L. infantum*	195	1	195
LinJ05_V3.0270	*L. infantum*	108	292	399
LinJ05_V3.0270	*L. infantum*	108	292	399
LinJ05_V3.0290	*L. infantum*	92	1	92

LinJ05_V3.0300	*L. infantum*	51	1	51
LinJ05_V3.0310	*L. infantum*	193	1	193
LinJ05_V3.0310	*L. infantum*	88	376	463
LinJ05_V3.0310	*L. infantum*	48	501	548
LinJ05_V3.0310	*L. infantum*	50	554	603
LinJ05_V3.0310	*L. infantum*	45	696	740
LinJ05_V3.0320	*L. infantum*	79	1	79
LinJ05_V3.0330	*L. infantum*	52	308	359
LinJ05_V3.0330	*L. infantum*	67	389	455
LinJ05_V3.0330	*L. infantum*	52	462	513
LinJ05_V3.0330	*L. infantum*	40	819	858
LinJ05_V3.0340	*L. infantum*	44	1	44
LinJ05_V3.0340	*L. infantum*	60	52	111
LinJ05_V3.0340	*L. infantum*	49	140	188
LinJ05_V3.0360	*L. infantum*	63	183	245
LinJ05_V3.0360	*L. infantum*	148	478	625
LinJ05_V3.0370	*L. infantum*	47	81	127
LinJ05_V3.0370	*L. infantum*	167	162	328
LinJ05_V3.0380	*L. infantum*	1463	4	1466
LinJ05_V3.0390	*L. infantum*	159	1	159
LinJ05_V3.0390	*L. infantum*	115	379	493
LinJ05_V3.0400	*L. infantum*	318	177	494
LinJ05_V3.0400	*L. infantum*	210	730	939
LinJ05_V3.0400	*L. infantum*	40	979	1018
LinJ05_V3.0410	*L. infantum*	134	1	134
LinJ05_V3.0410	*L. infantum*	43	222	264
LinJ05_V3.0410	*L. infantum*	41	316	356
LinJ05_V3.0410	*L. infantum*	96	428	523
LinJ05_V3.0410	*L. infantum*	63	560	622
LinJ05_V3.0430	*L. infantum*	60	41	100
LinJ05_V3.0430	*L. infantum*	73	181	253
LinJ05_V3.0430	*L. infantum*	73	181	253
LinJ05_V3.0440	*L. infantum*	72	84	155
LinJ05_V3.0460	*L. infantum*	69	1	69
LinJ05_V3.0460	*L. infantum*	43	112	154
LinJ05_V3.0460	*L. infantum*	153	475	627
LinJ05_V3.0470	*L. infantum*	154	89	242
LinJ05_V3.0480	*L. infantum*	127	211	337
LinJ05_V3.0490	*L. infantum*	41	56	96
LinJ05_V3.0560	*L. infantum*	58	67	124
LinJ05_V3.0560	*L. infantum*	146	193	338
LinJ05_V3.0570	*L. infantum*	59	192	250
LinJ05_V3.0580	*L. infantum*	129	1	129
LinJ05_V3.0580	*L. infantum*	51	332	382
LinJ05_V3.0580	*L. infantum*	233	547	779
LinJ05_V3.0590	*L. infantum*	194	17	210
LinJ05_V3.0590	*L. infantum*	47	569	615

LinJ05_V3.0590	*L. infantum*	51	680	730
LinJ05_V3.0590	*L. infantum*	139	805	943
LinJ05_V3.0600	*L. infantum*	42	148	189
LinJ05_V3.0600	*L. infantum*	83	483	565
LinJ05_V3.0600	*L. infantum*	45	630	674
LinJ05_V3.0600	*L. infantum*	40	867	906
LinJ05_V3.0610	*L. infantum*	99	1	99
LinJ05_V3.0610	*L. infantum*	94	123	216
LinJ05_V3.0610	*L. infantum*	157	324	480
LinJ05_V3.0610	*L. infantum*	96	654	749
LinJ05_V3.0610	*L. infantum*	117	752	868
LinJ05_V3.0610	*L. infantum*	42	891	932
LinJ05_V3.0620	*L. infantum*	149	95	243
LinJ05_V3.0620	*L. infantum*	93	256	348
LinJ05_V3.0620	*L. infantum*	93	256	348
LinJ05_V3.0630	*L. infantum*	46	37	82
LinJ05_V3.0630	*L. infantum*	42	395	436
LinJ05_V3.0630	*L. infantum*	143	499	641
LinJ05_V3.0630	*L. infantum*	304	766	1069
LinJ05_V3.0640	*L. infantum*	57	1	57
LinJ05_V3.0640	*L. infantum*	93	156	248
LinJ05_V3.0640	*L. infantum*	45	512	556
LinJ05_V3.0640	*L. infantum*	91	617	707
LinJ05_V3.0640	*L. infantum*	77	888	964
LinJ05_V3.0640	*L. infantum*	72	966	1037
LinJ05_V3.0640	*L. infantum*	194	1153	1346
LinJ05_V3.0640	*L. infantum*	118	1488	1605
LinJ05_V3.0650	*L. infantum*	78	32	109
LinJ05_V3.0650	*L. infantum*	51	298	348
LinJ05_V3.0650	*L. infantum*	189	390	578
LinJ05_V3.0650	*L. infantum*	129	673	801
LinJ05_V3.0660	*L. infantum*	79	1	79
LinJ05_V3.0670	*L. infantum*	1562	1	1562
LinJ05_V3.0670	*L. infantum*	117	1606	1722
LinJ05_V3.0670	*L. infantum*	519	1768	2286
LinJ05_V3.0670	*L. infantum*	176	2317	2492
LinJ05_V3.0680	*L. infantum*	52	1	52
LinJ05_V3.0690	*L. infantum*	47	39	85
LinJ05_V3.0690	*L. infantum*	389	116	504
LinJ05_V3.0690	*L. infantum*	308	549	856
LinJ05_V3.0690	*L. infantum*	130	1025	1154
LinJ05_V3.0690	*L. infantum*	114	1248	1361
LinJ05_V3.0690	*L. infantum*	319	1402	1720
LinJ05_V3.0690	*L. infantum*	130	1794	1923
LinJ05_V3.0690	*L. infantum*	127	1927	2053
LinJ05_V3.0690	*L. infantum*	272	2074	2345
LinJ05_V3.0700	*L. infantum*	986	1	986

LinJ05_V3.0700	*L. infantum*	76	998	1073
LinJ05_V3.0700	*L. infantum*	157	1078	1234
LinJ05_V3.0700	*L. infantum*	189	1265	1453
LinJ05_V3.0700	*L. infantum*	111	1477	1587
LinJ05_V3.0700	*L. infantum*	117	1609	1725
LinJ05_V3.0710	*L. infantum*	650	1	650
LinJ05_V3.0710	*L. infantum*	44	820	863
LinJ05_V3.0710	*L. infantum*	196	911	1106
LinJ05_V3.0710	*L. infantum*	290	1111	1400
LinJ05_V3.0720	*L. infantum*	340	63	402
LinJ05_V3.0720	*L. infantum*	257	408	664
LinJ05_V3.0720	*L. infantum*	189	750	938
LinJ05_V3.0720	*L. infantum*	393	1006	1398
LinJ05_V3.0730	*L. infantum*	609	1	609
LinJ05_V3.0740	*L. infantum*	176	154	329
LinJ05_V3.0750	*L. infantum*	47	53	99
LinJ05_V3.0750	*L. infantum*	137	282	418
LinJ05_V3.0750	*L. infantum*	137	282	418
LinJ05_V3.0760	*L. infantum*	254	1	254
LinJ05_V3.0760	*L. infantum*	67	276	342
LinJ05_V3.0760	*L. infantum*	149	356	504
LinJ05_V3.0760	*L. infantum*	179	658	836
LinJ05_V3.0760	*L. infantum*	70	916	985
LinJ05_V3.0760	*L. infantum*	149	1097	1245
LinJ05_V3.0760	*L. infantum*	136	1282	1417
LinJ05_V3.0760	*L. infantum*	59	1450	1508
LinJ05_V3.0800	*L. infantum*	73	1	73
LinJ05_V3.0800	*L. infantum*	42	107	148
LinJ05_V3.0800	*L. infantum*	78	230	307
LinJ05_V3.0820	*L. infantum*	63	186	248
LinJ05_V3.0820	*L. infantum*	43	357	399
LinJ05_V3.0820	*L. infantum*	45	592	636
LinJ05_V3.0840	*L. infantum*	155	1	155
LinJ05_V3.0840	*L. infantum*	472	166	637
LinJ05_V3.0840	*L. infantum*	763	659	1421
LinJ05_V3.0850	*L. infantum*	114	1	114
LinJ05_V3.0850	*L. infantum*	355	158	512
LinJ05_V3.0850	*L. infantum*	153	514	666
LinJ05_V3.0860	*L. infantum*	95	61	155
LinJ05_V3.0870	*L. infantum*	62	1	62
LinJ05_V3.0870	*L. infantum*	145	194	338
LinJ05_V3.0870	*L. infantum*	138	424	561
LinJ05_V3.0870	*L. infantum*	1313	628	1940
LinJ05_V3.0880	*L. infantum*	59	1	59
LinJ05_V3.0890	*L. infantum*	64	249	312
LinJ05_V3.0890	*L. infantum*	190	624	813
LinJ05_V3.0890	*L. infantum*	57	858	914

LinJ05_V3.0890	*L. infantum*	52	1072	1123
LinJ05_V3.0890	*L. infantum*	57	1152	1208
LinJ05_V3.0890	*L. infantum*	53	1284	1336
LinJ05_V3.0890	*L. infantum*	40	1364	1403
LinJ05_V3.0890	*L. infantum*	132	1552	1683
LinJ05_V3.0910	*L. infantum*	57	1	57
LinJ05_V3.0910	*L. infantum*	72	104	175
LinJ05_V3.0910	*L. infantum*	70	514	583
LinJ05_V3.0910	*L. infantum*	47	625	671
LinJ05_V3.0910	*L. infantum*	97	732	828
LinJ05_V3.0910	*L. infantum*	57	986	1042
LinJ05_V3.0920	*L. infantum*	44	1	44
LinJ05_V3.0920	*L. infantum*	365	50	414
LinJ05_V3.0920	*L. infantum*	59	523	581
LinJ05_V3.0920	*L. infantum*	56	620	675
LinJ05_V3.0920	*L. infantum*	268	821	1088
LinJ05_V3.0930	*L. infantum*	250	162	411
LinJ05_V3.0940	*L. infantum*	158	125	282
LinJ05_V3.0940	*L. infantum*	333	292	624
LinJ05_V3.0940	*L. infantum*	192	706	897
LinJ05_V3.0940	*L. infantum*	217	979	1195
LinJ05_V3.0940	*L. infantum*	41	1240	1280
LinJ05_V3.0950	*L. infantum*	54	237	290
LinJ05_V3.0950	*L. infantum*	46	334	379
LinJ05_V3.0950	*L. infantum*	42	411	452
LinJ05_V3.1000	*L. infantum*	54	1	54
LinJ05_V3.1010	*L. infantum*	131	484	614
LinJ05_V3.1020	*L. infantum*	67	179	245
LinJ05_V3.1020	*L. infantum*	60	307	366
LinJ05_V3.1020	*L. infantum*	54	455	508
LinJ05_V3.1020	*L. infantum*	44	668	711
LinJ05_V3.1020	*L. infantum*	53	736	788
LinJ05_V3.1020	*L. infantum*	42	811	852
LinJ05_V3.1030	*L. infantum*	74	1	74
LinJ05_V3.1040	*L. infantum*	47	8	54
LinJ05_V3.1040	*L. infantum*	61	212	272
LinJ05_V3.1040	*L. infantum*	61	212	272
LinJ05_V3.1050	*L. infantum*	69	1	69
LinJ05_V3.1050	*L. infantum*	41	139	179
LinJ05_V3.1060	*L. infantum*	41	145	185
LinJ05_V3.1060	*L. infantum*	46	404	449
LinJ05_V3.1060	*L. infantum*	46	404	449
LinJ05_V3.1070	*L. infantum*	97	158	254
LinJ05_V3.1070	*L. infantum*	44	314	357
LinJ05_V3.1070	*L. infantum*	44	314	357
LinJ05_V3.1080	*L. infantum*	62	1	62
LinJ05_V3.1080	*L. infantum*	63	137	199

LinJ05_V3.1080	*L. infantum*	55	534	588
LinJ05_V3.1080	*L. infantum*	70	656	725
LinJ05_V3.1090	*L. infantum*	199	188	386
LinJ05_V3.1090	*L. infantum*	130	390	519
LinJ05_V3.1110	*L. infantum*	140	1	140
LinJ05_V3.1110	*L. infantum*	100	237	336
LinJ05_V3.1110	*L. infantum*	82	432	513
LinJ05_V3.1110	*L. infantum*	222	707	928
LinJ05_V3.1110	*L. infantum*	67	1087	1153
LinJ05_V3.1130	*L. infantum*	44	465	508
LinJ05_V3.1150	*L. infantum*	49	43	91
LinJ05_V3.1150	*L. infantum*	40	163	202
LinJ05_V3.1150	*L. infantum*	153	207	359
LinJ05_V3.1150	*L. infantum*	148	436	583
LinJ05_V3.1150	*L. infantum*	295	622	916
LinJ05_V3.1170	*L. infantum*	67	36	102
LinJ05_V3.1170	*L. infantum*	48	248	295
LinJ05_V3.1170	*L. infantum*	135	324	458
LinJ05_V3.1180	*L. infantum*	72	1	72
LinJ05_V3.1180	*L. infantum*	59	224	282
LinJ05_V3.1180	*L. infantum*	59	353	411
LinJ05_V3.1180	*L. infantum*	122	551	672
LinJ05_V3.1180	*L. infantum*	50	731	780
LinJ05_V3.1180	*L. infantum*	71	844	914
LinJ05_V3.1180	*L. infantum*	83	957	1039
LinJ05_V3.1180	*L. infantum*	104	1209	1312
LinJ05_V3.1180	*L. infantum*	98	1369	1466
LinJ05_V3.1180	*L. infantum*	140	1470	1609
LinJ05_V3.1180	*L. infantum*	327	1633	1959
LinJ05_V3.1190	*L. infantum*	55	1	55
LinJ05_V3.1190	*L. infantum*	44	92	135
LinJ05_V3.1190	*L. infantum*	44	92	135
LinJ05_V3.1200	*L. infantum*	63	1	63
LinJ06_V3.0010	*L. infantum*	46	1	46
LinJ06_V3.0030	*L. infantum*	63	52	114
LinJ06_V3.0030	*L. infantum*	121	135	255
LinJ06_V3.0030	*L. infantum*	41	329	369
LinJ06_V3.0050	*L. infantum*	164	1	164
LinJ06_V3.0060	*L. infantum*	54	60	113
LinJ06_V3.0060	*L. infantum*	107	216	322
LinJ06_V3.0080	*L. infantum*	47	1	47
LinJ06_V3.0090	*L. infantum*	43	1	43
LinJ06_V3.0110	*L. infantum*	99	103	201
LinJ06_V3.0110	*L. infantum*	46	205	250
LinJ06_V3.0110	*L. infantum*	101	342	442
LinJ06_V3.0140	*L. infantum*	40	1	40
LinJ06_V3.0180	*L. infantum*	52	120	171

LinJ06_V3.0180	*L. infantum*	141	234	374
LinJ06_V3.0180	*L. infantum*	146	450	595
LinJ06_V3.0190	*L. infantum*	76	100	175
LinJ06_V3.0190	*L. infantum*	63	222	284
LinJ06_V3.0190	*L. infantum*	174	319	492
LinJ06_V3.0190	*L. infantum*	51	537	587
LinJ06_V3.0200	*L. infantum*	333	1	333
LinJ06_V3.0210	*L. infantum*	74	59	132
LinJ06_V3.0210	*L. infantum*	75	202	276
LinJ06_V3.0210	*L. infantum*	84	292	375
LinJ06_V3.0210	*L. infantum*	110	393	502
LinJ06_V3.0210	*L. infantum*	55	562	616
LinJ06_V3.0220	*L. infantum*	74	213	286
LinJ06_V3.0240	*L. infantum*	53	549	601
LinJ06_V3.0240	*L. infantum*	40	652	691
LinJ06_V3.0240	*L. infantum*	98	703	800
LinJ06_V3.0250	*L. infantum*	144	113	256
LinJ06_V3.0260	*L. infantum*	50	1061	1110
LinJ06_V3.0260	*L. infantum*	149	1178	1326
LinJ06_V3.0270	*L. infantum*	40	1	40
LinJ06_V3.0270	*L. infantum*	64	103	166
LinJ06_V3.0270	*L. infantum*	43	189	231
LinJ06_V3.0270	*L. infantum*	50	263	312
LinJ06_V3.0280	*L. infantum*	76	79	154
LinJ06_V3.0280	*L. infantum*	63	217	279
LinJ06_V3.0280	*L. infantum*	45	350	394
LinJ06_V3.0280	*L. infantum*	112	529	640
LinJ06_V3.0280	*L. infantum*	67	662	728
LinJ06_V3.0280	*L. infantum*	102	741	842
LinJ06_V3.0310	*L. infantum*	49	1	49
LinJ06_V3.0310	*L. infantum*	51	287	337
LinJ06_V3.0310	*L. infantum*	51	287	337
LinJ06_V3.0320	*L. infantum*	101	15	115
LinJ06_V3.0320	*L. infantum*	131	162	292
LinJ06_V3.0320	*L. infantum*	574	298	871
LinJ06_V3.0320	*L. infantum*	55	883	937
LinJ06_V3.0330	*L. infantum*	146	311	456
LinJ06_V3.0330	*L. infantum*	79	790	868
LinJ06_V3.0330	*L. infantum*	176	890	1065
LinJ06_V3.0340	*L. infantum*	51	32	82
LinJ06_V3.0340	*L. infantum*	49	857	905
LinJ06_V3.0340	*L. infantum*	49	857	905
LinJ06_V3.0380	*L. infantum*	43	30	72
LinJ06_V3.0380	*L. infantum*	70	311	380
LinJ06_V3.0380	*L. infantum*	70	311	380
LinJ06_V3.0390	*L. infantum*	52	373	424
LinJ06_V3.0390	*L. infantum*	42	475	516

LinJ06_V3.0390	*L. infantum*	101	644	744
LinJ06_V3.0390	*L. infantum*	59	779	837
LinJ06_V3.0390	*L. infantum*	69	839	907
LinJ06_V3.0410	*L. infantum*	48	54	101
LinJ06_V3.0410	*L. infantum*	113	133	245
LinJ06_V3.0420	*L. infantum*	131	35	165
LinJ06_V3.0430	*L. infantum*	48	54	101
LinJ06_V3.0430	*L. infantum*	106	133	238
LinJ06_V3.0440	*L. infantum*	69	1	69
LinJ06_V3.0440	*L. infantum*	313	138	450
LinJ06_V3.0440	*L. infantum*	73	500	572
LinJ06_V3.0450	*L. infantum*	114	1	114
LinJ06_V3.0450	*L. infantum*	59	172	230
LinJ06_V3.0450	*L. infantum*	214	478	691
LinJ06_V3.0460	*L. infantum*	117	24	140
LinJ06_V3.0460	*L. infantum*	44	291	334
LinJ06_V3.0460	*L. infantum*	321	417	737
LinJ06_V3.0460	*L. infantum*	75	743	817
LinJ06_V3.0460	*L. infantum*	94	899	992
LinJ06_V3.0470	*L. infantum*	198	1	198
LinJ06_V3.0470	*L. infantum*	109	232	340
LinJ06_V3.0480	*L. infantum*	157	32	188
LinJ06_V3.0480	*L. infantum*	177	307	483
LinJ06_V3.0480	*L. infantum*	367	498	864
LinJ06_V3.0480	*L. infantum*	71	929	999
LinJ06_V3.0490	*L. infantum*	107	1	107
LinJ06_V3.0490	*L. infantum*	85	173	257
LinJ06_V3.0490	*L. infantum*	57	298	354
LinJ06_V3.0490	*L. infantum*	211	381	591
LinJ06_V3.0490	*L. infantum*	102	597	698
LinJ06_V3.0490	*L. infantum*	178	789	966
LinJ06_V3.0490	*L. infantum*	88	1011	1098
LinJ06_V3.0500	*L. infantum*	52	274	325
LinJ06_V3.0520	*L. infantum*	57	48	104
LinJ06_V3.0520	*L. infantum*	51	123	173
LinJ06_V3.0520	*L. infantum*	70	324	393
LinJ06_V3.0520	*L. infantum*	76	430	505
LinJ06_V3.0530	*L. infantum*	53	72	124
LinJ06_V3.0530	*L. infantum*	93	285	377
LinJ06_V3.0530	*L. infantum*	76	507	582
LinJ06_V3.0530	*L. infantum*	42	638	679
LinJ06_V3.0540	*L. infantum*	42	1	42
LinJ06_V3.0540	*L. infantum*	330	84	413
LinJ06_V3.0540	*L. infantum*	108	443	550
LinJ06_V3.0540	*L. infantum*	91	568	658
LinJ06_V3.0540	*L. infantum*	141	685	825
LinJ06_V3.0540	*L. infantum*	255	858	1112

LinJ06_V3.0540	*L. infantum*	80	1239	1318
LinJ06_V3.0540	*L. infantum*	74	1376	1449
LinJ06_V3.0550	*L. infantum*	83	97	179
LinJ06_V3.0560	*L. infantum*	238	1	238
LinJ06_V3.0560	*L. infantum*	142	240	381
LinJ06_V3.0560	*L. infantum*	297	630	926
LinJ06_V3.0560	*L. infantum*	44	933	976
LinJ06_V3.0570	*L. infantum*	148	1	148
LinJ06_V3.0570	*L. infantum*	157	213	369
LinJ06_V3.0570	*L. infantum*	60	378	437
LinJ06_V3.0570	*L. infantum*	61	580	640
LinJ06_V3.0570	*L. infantum*	60	754	813
LinJ06_V3.0570	*L. infantum*	96	840	935
LinJ06_V3.0570	*L. infantum*	115	1150	1264
LinJ06_V3.0570	*L. infantum*	57	1570	1626
LinJ06_V3.0570	*L. infantum*	50	1684	1733
LinJ06_V3.0590	*L. infantum*	58	1	58
LinJ06_V3.0610	*L. infantum*	69	85	153
LinJ06_V3.0610	*L. infantum*	65	243	307
LinJ06_V3.0610	*L. infantum*	106	441	546
LinJ06_V3.0610	*L. infantum*	680	556	1235
LinJ06_V3.0610	*L. infantum*	254	1254	1507
LinJ06_V3.0620	*L. infantum*	69	107	175
LinJ06_V3.0620	*L. infantum*	213	196	408
LinJ06_V3.0620	*L. infantum*	51	412	462
LinJ06_V3.0620	*L. infantum*	84	529	612
LinJ06_V3.0620	*L. infantum*	62	807	868
LinJ06_V3.0620	*L. infantum*	100	883	982
LinJ06_V3.0620	*L. infantum*	126	1065	1190
LinJ06_V3.0620	*L. infantum*	92	1203	1294
LinJ06_V3.0630	*L. infantum*	51	1	51
LinJ06_V3.0640	*L. infantum*	49	509	557
LinJ06_V3.0650	*L. infantum*	90	1	90
LinJ06_V3.0650	*L. infantum*	134	173	306
LinJ06_V3.0650	*L. infantum*	40	358	397
LinJ06_V3.0650	*L. infantum*	81	590	670
LinJ06_V3.0650	*L. infantum*	132	686	817
LinJ06_V3.0650	*L. infantum*	302	963	1264
LinJ06_V3.0650	*L. infantum*	157	1287	1443
LinJ06_V3.0650	*L. infantum*	219	1597	1815
LinJ06_V3.0660	*L. infantum*	186	1	186
LinJ06_V3.0660	*L. infantum*	45	507	551
LinJ06_V3.0660	*L. infantum*	287	605	891
LinJ06_V3.0660	*L. infantum*	82	958	1039
LinJ06_V3.0660	*L. infantum*	123	1174	1296
LinJ06_V3.0660	*L. infantum*	145	1338	1482
LinJ06_V3.0660	*L. infantum*	167	1505	1671

LinJ06_V3.0660	*L. infantum*	129	1673	1801
LinJ06_V3.0660	*L. infantum*	622	1840	2461
LinJ06_V3.0660	*L. infantum*	51	2496	2546
LinJ06_V3.0660	*L. infantum*	114	2599	2712
LinJ06_V3.0660	*L. infantum*	158	2992	3149
LinJ06_V3.0670	*L. infantum*	43	1	43
LinJ06_V3.0680	*L. infantum*	373	1	373
LinJ06_V3.0680	*L. infantum*	68	376	443
LinJ06_V3.0680	*L. infantum*	187	480	666
LinJ06_V3.0690	*L. infantum*	145	1	145
LinJ06_V3.0690	*L. infantum*	204	151	354
LinJ06_V3.0690	*L. infantum*	380	376	755
LinJ06_V3.0690	*L. infantum*	56	757	812
LinJ06_V3.0690	*L. infantum*	212	848	1059
LinJ06_V3.0700	*L. infantum*	103	1	103
LinJ06_V3.0710	*L. infantum*	165	25	189
LinJ06_V3.0710	*L. infantum*	78	203	280
LinJ06_V3.0710	*L. infantum*	81	294	374
LinJ06_V3.0720	*L. infantum*	100	1	100
LinJ06_V3.0720	*L. infantum*	105	166	270
LinJ06_V3.0720	*L. infantum*	93	309	401
LinJ06_V3.0720	*L. infantum*	56	555	610
LinJ06_V3.0720	*L. infantum*	46	647	692
LinJ06_V3.0720	*L. infantum*	58	837	894
LinJ06_V3.0720	*L. infantum*	58	1140	1197
LinJ06_V3.0720	*L. infantum*	56	1255	1310
LinJ06_V3.0720	*L. infantum*	74	1547	1620
LinJ06_V3.0720	*L. infantum*	45	1684	1728
LinJ06_V3.0720	*L. infantum*	43	1903	1945
LinJ06_V3.0720	*L. infantum*	80	2306	2385
LinJ06_V3.0720	*L. infantum*	213	2419	2631
LinJ06_V3.0730	*L. infantum*	467	131	597
LinJ06_V3.0750	*L. infantum*	62	1	62
LinJ06_V3.0750	*L. infantum*	544	85	628
LinJ06_V3.0750	*L. infantum*	157	631	787
LinJ06_V3.0750	*L. infantum*	84	837	920
LinJ06_V3.0750	*L. infantum*	52	1000	1051
LinJ06_V3.0750	*L. infantum*	197	1076	1272
LinJ06_V3.0750	*L. infantum*	86	1339	1424
LinJ06_V3.0750	*L. infantum*	47	1480	1526
LinJ06_V3.0750	*L. infantum*	73	1610	1682
LinJ06_V3.0750	*L. infantum*	40	1689	1728
LinJ06_V3.0770	*L. infantum*	549	1	549
LinJ06_V3.0770	*L. infantum*	93	565	657
LinJ06_V3.0770	*L. infantum*	129	679	807
LinJ06_V3.0770	*L. infantum*	51	841	891
LinJ06_V3.0780	*L. infantum*	126	130	255

LinJ06_V3.0780	*L. infantum*	40	386	425
LinJ06_V3.0780	*L. infantum*	78	529	606
LinJ06_V3.0790	*L. infantum*	141	1	141
LinJ06_V3.0790	*L. infantum*	108	173	280
LinJ06_V3.0790	*L. infantum*	79	310	388
LinJ06_V3.0790	*L. infantum*	559	434	992
LinJ06_V3.0790	*L. infantum*	166	1019	1184
LinJ06_V3.0800	*L. infantum*	107	1	107
LinJ06_V3.0800	*L. infantum*	610	119	728
LinJ06_V3.0800	*L. infantum*	314	793	1106
LinJ06_V3.0800	*L. infantum*	81	1185	1265
LinJ06_V3.0800	*L. infantum*	141	1354	1494
LinJ06_V3.0800	*L. infantum*	286	1531	1816
LinJ06_V3.0810	*L. infantum*	203	1	203
LinJ06_V3.0810	*L. infantum*	338	206	543
LinJ06_V3.0810	*L. infantum*	826	576	1401
LinJ06_V3.0810	*L. infantum*	689	1412	2100
LinJ06_V3.0810	*L. infantum*	57	2121	2177
LinJ06_V3.0810	*L. infantum*	65	2216	2280
LinJ06_V3.0810	*L. infantum*	216	2285	2500
LinJ06_V3.0810	*L. infantum*	367	2531	2897
LinJ06_V3.0810	*L. infantum*	235	2909	3143
LinJ06_V3.0810	*L. infantum*	163	3178	3340
LinJ06_V3.0820	*L. infantum*	192	67	258
LinJ06_V3.0820	*L. infantum*	257	260	516
LinJ06_V3.0820	*L. infantum*	296	520	815
LinJ06_V3.0820	*L. infantum*	154	830	983
LinJ06_V3.0820	*L. infantum*	75	1000	1074
LinJ06_V3.0830	*L. infantum*	228	1	228
LinJ06_V3.0830	*L. infantum*	222	274	495
LinJ06_V3.0830	*L. infantum*	173	605	777
LinJ06_V3.0830	*L. infantum*	220	786	1005
LinJ06_V3.0830	*L. infantum*	113	1023	1135
LinJ06_V3.0830	*L. infantum*	73	1209	1281
LinJ06_V3.0830	*L. infantum*	50	1429	1478
LinJ06_V3.0830	*L. infantum*	47	1480	1526
LinJ06_V3.0830	*L. infantum*	40	1556	1595
LinJ06_V3.0840	*L. infantum*	199	49	247
LinJ06_V3.0840	*L. infantum*	82	292	373
LinJ06_V3.0840	*L. infantum*	54	424	477
LinJ06_V3.0840	*L. infantum*	48	484	531
LinJ06_V3.0840	*L. infantum*	197	675	871
LinJ06_V3.0840	*L. infantum*	67	977	1043
LinJ06_V3.0840	*L. infantum*	62	1106	1167
LinJ06_V3.0840	*L. infantum*	86	1431	1516
LinJ06_V3.0840	*L. infantum*	64	1536	1599
LinJ06_V3.0840	*L. infantum*	47	1646	1692

LinJ06_V3.0840	*L. infantum*	47	1932	1978
LinJ06_V3.0840	*L. infantum*	41	2112	2152
LinJ06_V3.0840	*L. infantum*	65	2328	2392
LinJ06_V3.0840	*L. infantum*	59	2703	2761
LinJ06_V3.0840	*L. infantum*	101	2846	2946
LinJ06_V3.0840	*L. infantum*	52	2981	3032
LinJ06_V3.0840	*L. infantum*	42	3087	3128
LinJ06_V3.0840	*L. infantum*	102	3262	3363
LinJ06_V3.0840	*L. infantum*	41	3455	3495
LinJ06_V3.0840	*L. infantum*	52	3507	3558
LinJ06_V3.0850	*L. infantum*	58	1	58
LinJ06_V3.0850	*L. infantum*	126	63	188
LinJ06_V3.0850	*L. infantum*	86	227	312
LinJ06_V3.0860	*L. infantum*	59	88	146
LinJ06_V3.0860	*L. infantum*	295	149	443
LinJ06_V3.0860	*L. infantum*	118	488	605
LinJ06_V3.0860	*L. infantum*	240	839	1078
LinJ06_V3.0860	*L. infantum*	310	1080	1389
LinJ06_V3.0870	*L. infantum*	161	72	232
LinJ06_V3.0880	*L. infantum*	42	1	42
LinJ06_V3.0880	*L. infantum*	442	49	490
LinJ06_V3.0880	*L. infantum*	276	529	804
LinJ06_V3.0880	*L. infantum*	44	886	929
LinJ06_V3.0880	*L. infantum*	87	1001	1087
LinJ06_V3.0880	*L. infantum*	42	1126	1167
LinJ06_V3.0880	*L. infantum*	183	1173	1355
LinJ06_V3.0880	*L. infantum*	81	1384	1464
LinJ06_V3.0880	*L. infantum*	84	1514	1597
LinJ06_V3.0880	*L. infantum*	102	1678	1779
LinJ06_V3.0880	*L. infantum*	566	1796	2361
LinJ06_V3.0880	*L. infantum*	200	2394	2593
LinJ06_V3.0880	*L. infantum*	44	2612	2655
LinJ06_V3.0880	*L. infantum*	144	2712	2855
LinJ06_V3.0880	*L. infantum*	45	2925	2969
LinJ06_V3.0880	*L. infantum*	199	3167	3365
LinJ06_V3.0900	*L. infantum*	72	1	72
LinJ06_V3.0900	*L. infantum*	44	395	438
LinJ06_V3.0900	*L. infantum*	44	395	438
LinJ06_V3.0910	*L. infantum*	40	1	40
LinJ06_V3.0920	*L. infantum*	65	22	86
LinJ06_V3.0920	*L. infantum*	49	92	140
LinJ06_V3.0920	*L. infantum*	102	226	327
LinJ06_V3.0920	*L. infantum*	63	427	489
LinJ06_V3.0920	*L. infantum*	209	527	735
LinJ06_V3.0930	*L. infantum*	186	1	186
LinJ06_V3.0930	*L. infantum*	668	206	873
LinJ06_V3.0930	*L. infantum*	121	889	1009

LinJ06_V3.0930	*L. infantum*	146	1020	1165
LinJ06_V3.0930	*L. infantum*	58	1205	1262
LinJ06_V3.0930	*L. infantum*	70	1314	1383
LinJ06_V3.0930	*L. infantum*	183	1467	1649
LinJ06_V3.0930	*L. infantum*	114	1701	1814
LinJ06_V3.0950	*L. infantum*	42	1	42
LinJ06_V3.0950	*L. infantum*	160	246	405
LinJ06_V3.0970	*L. infantum*	54	148	201
LinJ06_V3.0970	*L. infantum*	44	251	294
LinJ06_V3.0970	*L. infantum*	80	314	393
LinJ06_V3.0970	*L. infantum*	58	531	588
LinJ06_V3.1000	*L. infantum*	51	282	332
LinJ06_V3.1000	*L. infantum*	97	436	532
LinJ06_V3.1000	*L. infantum*	108	546	653
LinJ06_V3.1000	*L. infantum*	47	730	776
LinJ06_V3.1000	*L. infantum*	124	1217	1340
LinJ06_V3.1000	*L. infantum*	132	1368	1499
LinJ06_V3.1010	*L. infantum*	52	1	52
LinJ06_V3.1010	*L. infantum*	52	296	347
LinJ06_V3.1010	*L. infantum*	52	296	347
LinJ06_V3.1020	*L. infantum*	107	1	107
LinJ06_V3.1020	*L. infantum*	83	168	250
LinJ06_V3.1020	*L. infantum*	102	760	861
LinJ06_V3.1020	*L. infantum*	163	875	1037
LinJ06_V3.1030	*L. infantum*	46	69	114
LinJ06_V3.1040	*L. infantum*	93	79	171
LinJ06_V3.1040	*L. infantum*	43	223	265
LinJ06_V3.1040	*L. infantum*	43	223	265
LinJ06_V3.1050	*L. infantum*	56	1	56
LinJ06_V3.1050	*L. infantum*	69	117	185
LinJ06_V3.1050	*L. infantum*	49	286	334
LinJ06_V3.1050	*L. infantum*	50	552	601
LinJ06_V3.1060	*L. infantum*	151	80	230
LinJ06_V3.1070	*L. infantum*	48	153	200
LinJ06_V3.1070	*L. infantum*	81	381	461
LinJ06_V3.1070	*L. infantum*	210	494	703
LinJ06_V3.1070	*L. infantum*	123	714	836
LinJ06_V3.1080	*L. infantum*	214	1	214
LinJ06_V3.1080	*L. infantum*	137	275	411
LinJ06_V3.1080	*L. infantum*	86	482	567
LinJ06_V3.1080	*L. infantum*	72	612	683
LinJ06_V3.1100	*L. infantum*	44	1	44
LinJ06_V3.1130	*L. infantum*	147	1	147
LinJ06_V3.1130	*L. infantum*	78	185	262
LinJ06_V3.1130	*L. infantum*	104	282	385
LinJ06_V3.1130	*L. infantum*	133	463	595
LinJ06_V3.1150	*L. infantum*	130	1	130

LinJ06_V3.1150	*L. infantum*	716	204	919
LinJ06_V3.1150	*L. infantum*	69	983	1051
LinJ06_V3.1150	*L. infantum*	48	1060	1107
LinJ06_V3.1180	*L. infantum*	67	1	67
LinJ06_V3.1180	*L. infantum*	44	130	173
LinJ06_V3.1180	*L. infantum*	149	322	470
LinJ06_V3.1180	*L. infantum*	54	699	752
LinJ06_V3.1190	*L. infantum*	98	110	207
LinJ06_V3.1190	*L. infantum*	92	259	350
LinJ06_V3.1190	*L. infantum*	63	459	521
LinJ06_V3.1220	*L. infantum*	56	220	275
LinJ06_V3.1220	*L. infantum*	49	407	455
LinJ06_V3.1220	*L. infantum*	49	407	455
LinJ06_V3.1250	*L. infantum*	64	21	84
LinJ06_V3.1250	*L. infantum*	56	151	206
LinJ06_V3.1250	*L. infantum*	156	225	380
LinJ06_V3.1260	*L. infantum*	52	453	504
LinJ06_V3.1270	*L. infantum*	67	89	155
LinJ06_V3.1270	*L. infantum*	44	269	312
LinJ06_V3.1270	*L. infantum*	325	379	703
LinJ06_V3.1270	*L. infantum*	83	709	791
LinJ06_V3.1280	*L. infantum*	44	1	44
LinJ06_V3.1290	*L. infantum*	57	1	57
LinJ06_V3.1310	*L. infantum*	52	1	52
LinJ06_V3.1360	*L. infantum*	74	504	577
LinJ07_V3.0020	*L. infantum*	49	29	77
LinJ07_V3.0020	*L. infantum*	64	158	221
LinJ07_V3.0050	*L. infantum*	48	36	83
LinJ07_V3.0050	*L. infantum*	76	239	314
LinJ07_V3.0050	*L. infantum*	76	239	314
LinJ07_V3.0060	*L. infantum*	53	644	696
LinJ07_V3.0070	*L. infantum*	192	127	318
LinJ07_V3.0080	*L. infantum*	42	63	104
LinJ07_V3.0080	*L. infantum*	95	126	220
LinJ07_V3.0080	*L. infantum*	76	465	540
LinJ07_V3.0080	*L. infantum*	43	886	928
LinJ07_V3.0090	*L. infantum*	56	122	177
LinJ07_V3.0090	*L. infantum*	43	216	258
LinJ07_V3.0090	*L. infantum*	76	306	381
LinJ07_V3.0090	*L. infantum*	46	401	446
LinJ07_V3.0090	*L. infantum*	91	545	635
LinJ07_V3.0090	*L. infantum*	85	773	857
LinJ07_V3.0090	*L. infantum*	80	1003	1082
LinJ07_V3.0090	*L. infantum*	64	1117	1180
LinJ07_V3.0090	*L. infantum*	41	1218	1258
LinJ07_V3.0090	*L. infantum*	43	1352	1394
LinJ07_V3.0090	*L. infantum*	50	1636	1685

LinJ07_V3.0090	*L. infantum*	81	1730	1810
LinJ07_V3.0090	*L. infantum*	97	2263	2359
LinJ07_V3.0090	*L. infantum*	116	2406	2521
LinJ07_V3.0090	*L. infantum*	99	2675	2773
LinJ07_V3.0090	*L. infantum*	51	2900	2950
LinJ07_V3.0090	*L. infantum*	60	3021	3080
LinJ07_V3.0090	*L. infantum*	45	3279	3323
LinJ07_V3.0090	*L. infantum*	49	3423	3471
LinJ07_V3.0090	*L. infantum*	50	3630	3679
LinJ07_V3.0100	*L. infantum*	376	1	376
LinJ07_V3.0100	*L. infantum*	50	463	512
LinJ07_V3.0100	*L. infantum*	162	634	795
LinJ07_V3.0120	*L. infantum*	274	242	515
LinJ07_V3.0120	*L. infantum*	54	577	630
LinJ07_V3.0130	*L. infantum*	75	1	75
LinJ07_V3.0190	*L. infantum*	51	59	109
LinJ07_V3.0210	*L. infantum*	49	69	117
LinJ07_V3.0220	*L. infantum*	60	13	72
LinJ07_V3.0230	*L. infantum*	56	399	454
LinJ07_V3.0240	*L. infantum*	40	633	672
LinJ07_V3.0250	*L. infantum*	73	322	394
LinJ07_V3.0250	*L. infantum*	79	495	573
LinJ07_V3.0250	*L. infantum*	79	495	573
LinJ07_V3.0270	*L. infantum*	137	32	168
LinJ07_V3.0270	*L. infantum*	69	648	716
LinJ07_V3.0270	*L. infantum*	58	970	1027
LinJ07_V3.0270	*L. infantum*	43	1479	1521
LinJ07_V3.0270	*L. infantum*	40	1784	1823
LinJ07_V3.0270	*L. infantum*	53	1983	2035
LinJ07_V3.0270	*L. infantum*	94	2132	2225
LinJ07_V3.0280	*L. infantum*	58	275	332
LinJ07_V3.0290	*L. infantum*	47	180	226
LinJ07_V3.0300	*L. infantum*	90	263	352
LinJ07_V3.0310	*L. infantum*	57	20	76
LinJ07_V3.0310	*L. infantum*	45	205	249
LinJ07_V3.0310	*L. infantum*	87	503	589
LinJ07_V3.0320	*L. infantum*	602	247	848
LinJ07_V3.0330	*L. infantum*	123	196	318
LinJ07_V3.0330	*L. infantum*	58	456	513
LinJ07_V3.0330	*L. infantum*	213	538	750
LinJ07_V3.0330	*L. infantum*	159	820	978
LinJ07_V3.0340	*L. infantum*	43	1	43
LinJ07_V3.0340	*L. infantum*	133	78	210
LinJ07_V3.0340	*L. infantum*	166	230	395
LinJ07_V3.0340	*L. infantum*	40	435	474
LinJ07_V3.0340	*L. infantum*	69	495	563
LinJ07_V3.0340	*L. infantum*	487	729	1215

LinJ07_V3.0340	*L. infantum*	63	1306	1368
LinJ07_V3.0340	*L. infantum*	67	1375	1441
LinJ07_V3.0340	*L. infantum*	46	1475	1520
LinJ07_V3.0340	*L. infantum*	99	1591	1689
LinJ07_V3.0340	*L. infantum*	78	1709	1786
LinJ07_V3.0340	*L. infantum*	88	1859	1946
LinJ07_V3.0340	*L. infantum*	47	1982	2028
LinJ07_V3.0360	*L. infantum*	43	41	83
LinJ07_V3.0360	*L. infantum*	69	204	272
LinJ07_V3.0360	*L. infantum*	76	376	451
LinJ07_V3.0370	*L. infantum*	41	1	41
LinJ07_V3.0370	*L. infantum*	49	184	232
LinJ07_V3.0370	*L. infantum*	49	184	232
LinJ07_V3.0380	*L. infantum*	87	48	134
LinJ07_V3.0380	*L. infantum*	52	176	227
LinJ07_V3.0380	*L. infantum*	40	596	635
LinJ07_V3.0390	*L. infantum*	91	1	91
LinJ07_V3.0400	*L. infantum*	80	149	228
LinJ07_V3.0400	*L. infantum*	115	373	487
LinJ07_V3.0420	*L. infantum*	47	261	307
LinJ07_V3.0440	*L. infantum*	99	1	99
LinJ07_V3.0440	*L. infantum*	157	167	323
LinJ07_V3.0440	*L. infantum*	53	341	393
LinJ07_V3.0440	*L. infantum*	41	461	501
LinJ07_V3.0440	*L. infantum*	149	633	781
LinJ07_V3.0440	*L. infantum*	44	785	828
LinJ07_V3.0440	*L. infantum*	51	874	924
LinJ07_V3.0440	*L. infantum*	148	1118	1265
LinJ07_V3.0440	*L. infantum*	61	1319	1379
LinJ07_V3.0440	*L. infantum*	44	1460	1503
LinJ07_V3.0440	*L. infantum*	96	1575	1670
LinJ07_V3.0440	*L. infantum*	56	1792	1847
LinJ07_V3.0440	*L. infantum*	167	1855	2021
LinJ07_V3.0440	*L. infantum*	91	2093	2183
LinJ07_V3.0440	*L. infantum*	125	2388	2512
LinJ07_V3.0440	*L. infantum*	42	2628	2669
LinJ07_V3.0440	*L. infantum*	248	2778	3025
LinJ07_V3.0440	*L. infantum*	85	3051	3135
LinJ07_V3.0440	*L. infantum*	92	3286	3377
LinJ07_V3.0440	*L. infantum*	44	3438	3481
LinJ07_V3.0440	*L. infantum*	49	3536	3584
LinJ07_V3.0440	*L. infantum*	118	3662	3779
LinJ07_V3.0440	*L. infantum*	82	3832	3913
LinJ07_V3.0440	*L. infantum*	420	3933	4352
LinJ07_V3.0440	*L. infantum*	187	4377	4563
LinJ07_V3.0440	*L. infantum*	105	4569	4673
LinJ07_V3.0440	*L. infantum*	92	4723	4814

LinJ07_V3.0440	*L. infantum*	151	4872	5022
LinJ07_V3.0440	*L. infantum*	61	5096	5156
LinJ07_V3.0440	*L. infantum*	49	5388	5436
LinJ07_V3.0440	*L. infantum*	81	5604	5684
LinJ07_V3.0450	*L. infantum*	45	34	78
LinJ07_V3.0460	*L. infantum*	246	57	302
LinJ07_V3.0460	*L. infantum*	58	431	488
LinJ07_V3.0460	*L. infantum*	92	545	636
LinJ07_V3.0460	*L. infantum*	40	811	850
LinJ07_V3.0470	*L. infantum*	100	1	100
LinJ07_V3.0470	*L. infantum*	60	238	297
LinJ07_V3.0470	*L. infantum*	46	300	345
LinJ07_V3.0470	*L. infantum*	56	393	448
LinJ07_V3.0470	*L. infantum*	57	574	630
LinJ07_V3.0470	*L. infantum*	84	655	738
LinJ07_V3.0480	*L. infantum*	74	61	134
LinJ07_V3.0480	*L. infantum*	48	512	559
LinJ07_V3.0480	*L. infantum*	220	571	790
LinJ07_V3.0480	*L. infantum*	79	817	895
LinJ07_V3.0490	*L. infantum*	58	141	198
LinJ07_V3.0490	*L. infantum*	83	260	342
LinJ07_V3.0490	*L. infantum*	158	436	593
LinJ07_V3.0500	*L. infantum*	60	1	60
LinJ07_V3.0500	*L. infantum*	76	73	148
LinJ07_V3.0500	*L. infantum*	72	181	252
LinJ07_V3.0500	*L. infantum*	53	435	487
LinJ07_V3.0500	*L. infantum*	52	559	610
LinJ07_V3.0500	*L. infantum*	56	690	745
LinJ07_V3.0500	*L. infantum*	125	747	871
LinJ07_V3.0510	*L. infantum*	43	199	241
LinJ07_V3.0530	*L. infantum*	47	2280	2326
LinJ07_V3.0530	*L. infantum*	61	2350	2410
LinJ07_V3.0530	*L. infantum*	41	2420	2460
LinJ07_V3.0530	*L. infantum*	41	2597	2637
LinJ07_V3.0530	*L. infantum*	47	2687	2733
LinJ07_V3.0530	*L. infantum*	94	2793	2886
LinJ07_V3.0530	*L. infantum*	73	3586	3658
LinJ07_V3.0530	*L. infantum*	78	3695	3772
LinJ07_V3.0530	*L. infantum*	49	3981	4029
LinJ07_V3.0530	*L. infantum*	40	4110	4149
LinJ07_V3.0530	*L. infantum*	82	4191	4272
LinJ07_V3.0530	*L. infantum*	79	4392	4470
LinJ07_V3.0530	*L. infantum*	50	4517	4566
LinJ07_V3.0530	*L. infantum*	57	4698	4754
LinJ07_V3.0540	*L. infantum*	53	1	53
LinJ07_V3.0540	*L. infantum*	89	98	186
LinJ07_V3.0540	*L. infantum*	91	194	284

LinJ07_V3.0550	*L. infantum*	63	156	218
LinJ07_V3.0560	*L. infantum*	76	20	95
LinJ07_V3.0560	*L. infantum*	63	141	203
LinJ07_V3.0560	*L. infantum*	63	141	203
LinJ07_V3.0570	*L. infantum*	145	28	172
LinJ07_V3.0580	*L. infantum*	68	1	68
LinJ07_V3.0590	*L. infantum*	93	1	93
LinJ07_V3.0590	*L. infantum*	386	131	516
LinJ07_V3.0590	*L. infantum*	78	591	668
LinJ07_V3.0590	*L. infantum*	245	671	915
LinJ07_V3.0600	*L. infantum*	385	27	411
LinJ07_V3.0600	*L. infantum*	60	448	507
LinJ07_V3.0600	*L. infantum*	97	541	637
LinJ07_V3.0600	*L. infantum*	164	706	869
LinJ07_V3.0600	*L. infantum*	67	900	966
LinJ07_V3.0600	*L. infantum*	112	983	1094
LinJ07_V3.0600	*L. infantum*	45	1224	1268
LinJ07_V3.0600	*L. infantum*	76	1310	1385
LinJ07_V3.0600	*L. infantum*	64	1417	1480
LinJ07_V3.0600	*L. infantum*	80	1562	1641
LinJ07_V3.0600	*L. infantum*	201	1655	1855
LinJ07_V3.0610	*L. infantum*	57	169	225
LinJ07_V3.0620	*L. infantum*	138	1	138
LinJ07_V3.0620	*L. infantum*	242	225	466
LinJ07_V3.0630	*L. infantum*	164	57	220
LinJ07_V3.0630	*L. infantum*	449	236	684
LinJ07_V3.0630	*L. infantum*	94	754	847
LinJ07_V3.0630	*L. infantum*	64	896	959
LinJ07_V3.0630	*L. infantum*	101	1148	1248
LinJ07_V3.0630	*L. infantum*	93	1278	1370
LinJ07_V3.0640	*L. infantum*	556	225	780
LinJ07_V3.0650	*L. infantum*	50	49	98
LinJ07_V3.0650	*L. infantum*	84	326	409
LinJ07_V3.0650	*L. infantum*	54	716	769
LinJ07_V3.0670	*L. infantum*	96	100	195
LinJ07_V3.0680	*L. infantum*	52	113	164
LinJ07_V3.0680	*L. infantum*	42	191	232
LinJ07_V3.0680	*L. infantum*	42	191	232
LinJ07_V3.0690	*L. infantum*	135	1	135
LinJ07_V3.0690	*L. infantum*	139	156	294
LinJ07_V3.0690	*L. infantum*	226	330	555
LinJ07_V3.0690	*L. infantum*	80	557	636
LinJ07_V3.0700	*L. infantum*	48	1	48
LinJ07_V3.0700	*L. infantum*	43	431	473
LinJ07_V3.0700	*L. infantum*	43	431	473
LinJ07_V3.0740	*L. infantum*	100	22	121
LinJ07_V3.0750	*L. infantum*	111	1	111

LinJ07_V3.0770	*L. infantum*	259	1	259
LinJ07_V3.0770	*L. infantum*	104	288	391
LinJ07_V3.0770	*L. infantum*	399	405	803
LinJ07_V3.0770	*L. infantum*	352	833	1184
LinJ07_V3.0770	*L. infantum*	179	1295	1473
LinJ07_V3.0770	*L. infantum*	41	1521	1561
LinJ07_V3.0770	*L. infantum*	139	1663	1801
LinJ07_V3.0780	*L. infantum*	99	1	99
LinJ07_V3.0780	*L. infantum*	209	111	319
LinJ07_V3.0780	*L. infantum*	56	325	380
LinJ07_V3.0780	*L. infantum*	57	705	761
LinJ07_V3.0780	*L. infantum*	111	807	917
LinJ07_V3.0790	*L. infantum*	40	1	40
LinJ07_V3.0800	*L. infantum*	138	15	152
LinJ07_V3.0800	*L. infantum*	244	161	404
LinJ07_V3.0800	*L. infantum*	176	406	581
LinJ07_V3.0810	*L. infantum*	96	861	956
LinJ07_V3.0810	*L. infantum*	45	1217	1261
LinJ07_V3.0820	*L. infantum*	97	1	97
LinJ07_V3.0820	*L. infantum*	53	306	358
LinJ07_V3.0820	*L. infantum*	58	631	688
LinJ07_V3.0830	*L. infantum*	109	1	109
LinJ07_V3.0830	*L. infantum*	59	223	281
LinJ07_V3.0830	*L. infantum*	103	357	459
LinJ07_V3.0830	*L. infantum*	89	870	958
LinJ07_V3.0830	*L. infantum*	77	1024	1100
LinJ07_V3.0830	*L. infantum*	53	1140	1192
LinJ07_V3.0830	*L. infantum*	48	1336	1383
LinJ07_V3.0830	*L. infantum*	50	1443	1492
LinJ07_V3.0830	*L. infantum*	157	1605	1761
LinJ07_V3.0830	*L. infantum*	47	1844	1890
LinJ07_V3.0830	*L. infantum*	105	1997	2101
LinJ07_V3.0830	*L. infantum*	43	2148	2190
LinJ07_V3.0830	*L. infantum*	68	2245	2312
LinJ07_V3.0830	*L. infantum*	40	2420	2459
LinJ07_V3.0830	*L. infantum*	65	2537	2601
LinJ07_V3.0830	*L. infantum*	123	2611	2733
LinJ07_V3.0830	*L. infantum*	49	2779	2827
LinJ07_V3.0830	*L. infantum*	120	2938	3057
LinJ07_V3.0830	*L. infantum*	93	3122	3214
LinJ07_V3.0830	*L. infantum*	52	3236	3287
LinJ07_V3.0830	*L. infantum*	49	3321	3369
LinJ07_V3.0830	*L. infantum*	312	3494	3805
LinJ07_V3.0840	*L. infantum*	150	1	150
LinJ07_V3.0860	*L. infantum*	176	48	223
LinJ07_V3.0870	*L. infantum*	68	1	68
LinJ07_V3.0870	*L. infantum*	57	109	165

LinJ07_V3.0870	*L. infantum*	143	257	399
LinJ07_V3.0870	*L. infantum*	286	459	744
LinJ07_V3.0870	*L. infantum*	72	855	926
LinJ07_V3.0870	*L. infantum*	60	1004	1063
LinJ07_V3.0880	*L. infantum*	130	1	130
LinJ07_V3.0880	*L. infantum*	141	192	332
LinJ07_V3.0890	*L. infantum*	83	69	151
LinJ07_V3.0890	*L. infantum*	132	180	311
LinJ07_V3.0890	*L. infantum*	192	358	549
LinJ07_V3.0900	*L. infantum*	79	1	79
LinJ07_V3.0900	*L. infantum*	167	108	274
LinJ07_V3.0910	*L. infantum*	54	279	332
LinJ07_V3.0910	*L. infantum*	42	452	493
LinJ07_V3.0910	*L. infantum*	42	452	493
LinJ07_V3.0920	*L. infantum*	130	71	200
LinJ07_V3.0920	*L. infantum*	113	240	352
LinJ07_V3.0950	*L. infantum*	59	437	495
LinJ07_V3.0950	*L. infantum*	49	532	580
LinJ07_V3.0950	*L. infantum*	65	736	800
LinJ07_V3.0970	*L. infantum*	47	425	471
LinJ07_V3.0980	*L. infantum*	64	16	79
LinJ07_V3.0980	*L. infantum*	358	81	438
LinJ07_V3.0990	*L. infantum*	49	1410	1458
LinJ07_V3.0990	*L. infantum*	510	1831	2340
LinJ07_V3.1000	*L. infantum*	53	1	53
LinJ07_V3.1010	*L. infantum*	1173	23	1195
LinJ07_V3.1010	*L. infantum*	126	1218	1343
LinJ07_V3.1010	*L. infantum*	101	1375	1475
LinJ07_V3.1020	*L. infantum*	83	1	83
LinJ07_V3.1020	*L. infantum*	41	145	185
LinJ07_V3.1020	*L. infantum*	125	251	375
LinJ07_V3.1040	*L. infantum*	586	1	586
LinJ07_V3.1040	*L. infantum*	55	778	832
LinJ07_V3.1040	*L. infantum*	64	906	969
LinJ07_V3.1040	*L. infantum*	111	1228	1338
LinJ07_V3.1040	*L. infantum*	76	1375	1450
LinJ07_V3.1040	*L. infantum*	152	1676	1827
LinJ07_V3.1050	*L. infantum*	108	29	136
LinJ07_V3.1050	*L. infantum*	502	138	639
LinJ07_V3.1050	*L. infantum*	126	653	778
LinJ07_V3.1050	*L. infantum*	84	890	973
LinJ07_V3.1050	*L. infantum*	50	977	1026
LinJ07_V3.1050	*L. infantum*	41	1176	1216
LinJ07_V3.1050	*L. infantum*	43	1314	1356
LinJ07_V3.1060	*L. infantum*	635	1	635
LinJ07_V3.1070	*L. infantum*	250	46	295
LinJ07_V3.1070	*L. infantum*	83	418	500

LinJ07_V3.1070	*L. infantum*	90	630	719
LinJ07_V3.1070	*L. infantum*	92	915	1006
LinJ07_V3.1070	*L. infantum*	140	1054	1193
LinJ07_V3.1070	*L. infantum*	103	1232	1334
LinJ07_V3.1070	*L. infantum*	45	1346	1390
LinJ07_V3.1070	*L. infantum*	91	1435	1525
LinJ07_V3.1070	*L. infantum*	88	1587	1674
LinJ07_V3.1070	*L. infantum*	285	1732	2016
LinJ07_V3.1080	*L. infantum*	80	110	189
LinJ07_V3.1100	*L. infantum*	125	1	125
LinJ07_V3.1100	*L. infantum*	376	199	574
LinJ07_V3.1100	*L. infantum*	133	582	714
LinJ07_V3.1100	*L. infantum*	76	738	813
LinJ07_V3.1100	*L. infantum*	144	912	1055
LinJ07_V3.1100	*L. infantum*	131	1182	1312
LinJ07_V3.1100	*L. infantum*	78	1345	1422
LinJ07_V3.1100	*L. infantum*	221	1673	1893
LinJ07_V3.1110	*L. infantum*	733	1	733
LinJ07_V3.1120	*L. infantum*	63	45	107
LinJ07_V3.1120	*L. infantum*	116	203	318
LinJ07_V3.1120	*L. infantum*	140	670	809
LinJ07_V3.1140	*L. infantum*	116	88	203
LinJ07_V3.1150	*L. infantum*	266	99	364
LinJ07_V3.1160	*L. infantum*	44	1	44
LinJ07_V3.1170	*L. infantum*	260	1	260
LinJ07_V3.1170	*L. infantum*	60	475	534
LinJ07_V3.1170	*L. infantum*	134	815	948
LinJ07_V3.1180	*L. infantum*	61	12	72
LinJ07_V3.1200	*L. infantum*	41	1	41
LinJ07_V3.1210	*L. infantum*	123	871	993
LinJ07_V3.1220	*L. infantum*	46	1	46
LinJ07_V3.1240	*L. infantum*	128	89	216
LinJ07_V3.1240	*L. infantum*	85	237	321
LinJ07_V3.1250	*L. infantum*	231	307	537
LinJ07_V3.1270	*L. infantum*	83	1	83
LinJ07_V3.1270	*L. infantum*	146	159	304
LinJ07_V3.1290	*L. infantum*	75	230	304
LinJ07_V3.1300	*L. infantum*	158	188	345
LinJ07_V3.1330	*L. infantum*	146	41	186
LinJ07_V3.1330	*L. infantum*	66	1094	1159
LinJ07_V3.1330	*L. infantum*	51	1273	1323
LinJ07_V3.1330	*L. infantum*	42	1425	1466
LinJ07_V3.1330	*L. infantum*	53	1550	1602
LinJ07_V3.1340	*L. infantum*	77	1	77
LinJ08_V3.0010	*L. infantum*	116	176	291
LinJ08_V3.0010	*L. infantum*	202	303	504
LinJ08_V3.0010	*L. infantum*	166	674	839

LinJ08_V3.0010	*L. infantum*	84	854	937
LinJ08_V3.0040	*L. infantum*	83	45	127
LinJ08_V3.0070	*L. infantum*	79	72	150
LinJ08_V3.0070	*L. infantum*	81	200	280
LinJ08_V3.0070	*L. infantum*	173	296	468
LinJ08_V3.0080	*L. infantum*	162	418	579
LinJ08_V3.0090	*L. infantum*	56	545	600
LinJ08_V3.0090	*L. infantum*	193	664	856
LinJ08_V3.0090	*L. infantum*	193	664	856
LinJ08_V3.0100	*L. infantum*	47	1	47
LinJ08_V3.0100	*L. infantum*	78	254	331
LinJ08_V3.0100	*L. infantum*	72	380	451
LinJ08_V3.0110	*L. infantum*	220	53	272
LinJ08_V3.0110	*L. infantum*	266	378	643
LinJ08_V3.0120	*L. infantum*	46	197	242
LinJ08_V3.0130	*L. infantum*	59	20	78
LinJ08_V3.0130	*L. infantum*	185	137	321
LinJ08_V3.0130	*L. infantum*	812	339	1150
LinJ08_V3.0140	*L. infantum*	138	212	349
LinJ08_V3.0150	*L. infantum*	85	303	387
LinJ08_V3.0150	*L. infantum*	61	523	583
LinJ08_V3.0150	*L. infantum*	232	686	917
LinJ08_V3.0150	*L. infantum*	61	958	1018
LinJ08_V3.0160	*L. infantum*	71	1	71
LinJ08_V3.0160	*L. infantum*	66	130	195
LinJ08_V3.0160	*L. infantum*	48	263	310
LinJ08_V3.0160	*L. infantum*	56	444	499
LinJ08_V3.0170	*L. infantum*	82	21	102
LinJ08_V3.0180	*L. infantum*	54	1	54
LinJ08_V3.0180	*L. infantum*	108	237	344
LinJ08_V3.0180	*L. infantum*	108	237	344
LinJ08_V3.0190	*L. infantum*	60	235	294
LinJ08_V3.0210	*L. infantum*	58	538	595
LinJ08_V3.0220	*L. infantum*	157	1	157
LinJ08_V3.0220	*L. infantum*	234	162	395
LinJ08_V3.0220	*L. infantum*	59	413	471
LinJ08_V3.0230	*L. infantum*	40	24	63
LinJ08_V3.0230	*L. infantum*	116	70	185
LinJ08_V3.0230	*L. infantum*	114	478	591
LinJ08_V3.0230	*L. infantum*	75	731	805
LinJ08_V3.0230	*L. infantum*	98	987	1084
LinJ08_V3.0250	*L. infantum*	46	1	46
LinJ08_V3.0250	*L. infantum*	50	213	262
LinJ08_V3.0250	*L. infantum*	50	213	262
LinJ08_V3.0260	*L. infantum*	73	1	73
LinJ08_V3.0260	*L. infantum*	41	77	117
LinJ08_V3.0270	*L. infantum*	438	1	438

LinJ08_V3.0280	*L. infantum*	144	1	144
LinJ08_V3.0280	*L. infantum*	58	229	286
LinJ08_V3.0280	*L. infantum*	64	353	416
LinJ08_V3.0280	*L. infantum*	158	418	575
LinJ08_V3.0280	*L. infantum*	161	587	747
LinJ08_V3.0290	*L. infantum*	41	285	325
LinJ08_V3.0320	*L. infantum*	52	12	63
LinJ08_V3.0320	*L. infantum*	54	256	309
LinJ08_V3.0320	*L. infantum*	54	256	309
LinJ08_V3.0350	*L. infantum*	147	1	147
LinJ08_V3.0350	*L. infantum*	40	426	465
LinJ08_V3.0350	*L. infantum*	55	544	598
LinJ08_V3.0350	*L. infantum*	80	652	731
LinJ08_V3.0350	*L. infantum*	66	815	880
LinJ08_V3.0350	*L. infantum*	41	1139	1179
LinJ08_V3.0350	*L. infantum*	82	1218	1299
LinJ08_V3.0350	*L. infantum*	44	1307	1350
LinJ08_V3.0350	*L. infantum*	131	1383	1513
LinJ08_V3.0350	*L. infantum*	46	1744	1789
LinJ08_V3.0350	*L. infantum*	91	1852	1942
LinJ08_V3.0350	*L. infantum*	63	2157	2219
LinJ08_V3.0350	*L. infantum*	74	2302	2375
LinJ08_V3.0350	*L. infantum*	50	2443	2492
LinJ08_V3.0350	*L. infantum*	41	2582	2622
LinJ08_V3.0350	*L. infantum*	172	2633	2804
LinJ08_V3.0350	*L. infantum*	87	3036	3122
LinJ08_V3.0350	*L. infantum*	78	3170	3247
LinJ08_V3.0350	*L. infantum*	138	3269	3406
LinJ08_V3.0350	*L. infantum*	46	3455	3500
LinJ08_V3.0350	*L. infantum*	126	3618	3743
LinJ08_V3.0350	*L. infantum*	57	4183	4239
LinJ08_V3.0350	*L. infantum*	48	4248	4295
LinJ08_V3.0350	*L. infantum*	58	4380	4437
LinJ08_V3.0350	*L. infantum*	80	4472	4551
LinJ08_V3.0350	*L. infantum*	136	4635	4770
LinJ08_V3.0360	*L. infantum*	87	1	87
LinJ08_V3.0360	*L. infantum*	59	96	154
LinJ08_V3.0360	*L. infantum*	786	194	979
LinJ08_V3.0360	*L. infantum*	139	1079	1217
LinJ08_V3.0370	*L. infantum*	142	52	193
LinJ08_V3.0370	*L. infantum*	159	251	409
LinJ08_V3.0370	*L. infantum*	68	416	483
LinJ08_V3.0380	*L. infantum*	108	1	108
LinJ08_V3.0400	*L. infantum*	73	16	88
LinJ08_V3.0400	*L. infantum*	372	107	478
LinJ08_V3.0400	*L. infantum*	108	502	609
LinJ08_V3.0400	*L. infantum*	154	644	797

LinJ08_V3.0400	*L. infantum*	138	826	963
LinJ08_V3.0400	*L. infantum*	111	1033	1143
LinJ08_V3.0400	*L. infantum*	58	1270	1327
LinJ08_V3.0400	*L. infantum*	96	1336	1431
LinJ08_V3.0400	*L. infantum*	76	1437	1512
LinJ08_V3.0400	*L. infantum*	347	1540	1886
LinJ08_V3.0420	*L. infantum*	45	21	65
LinJ08_V3.0420	*L. infantum*	69	161	229
LinJ08_V3.0420	*L. infantum*	52	354	405
LinJ08_V3.0420	*L. infantum*	40	976	1015
LinJ08_V3.0430	*L. infantum*	172	1	172
LinJ08_V3.0430	*L. infantum*	118	424	541
LinJ08_V3.0430	*L. infantum*	280	558	837
LinJ08_V3.0430	*L. infantum*	148	840	987
LinJ08_V3.0430	*L. infantum*	57	1086	1142
LinJ08_V3.0430	*L. infantum*	198	1493	1690
LinJ08_V3.0430	*L. infantum*	106	1879	1984
LinJ08_V3.0440	*L. infantum*	196	81	276
LinJ08_V3.0440	*L. infantum*	68	329	396
LinJ08_V3.0440	*L. infantum*	91	399	489
LinJ08_V3.0440	*L. infantum*	87	516	602
LinJ08_V3.0440	*L. infantum*	142	637	778
LinJ08_V3.0450	*L. infantum*	44	157	200
LinJ08_V3.0450	*L. infantum*	46	236	281
LinJ08_V3.0450	*L. infantum*	186	460	645
LinJ08_V3.0450	*L. infantum*	62	756	817
LinJ08_V3.0450	*L. infantum*	102	926	1027
LinJ08_V3.0450	*L. infantum*	77	1423	1499
LinJ08_V3.0450	*L. infantum*	62	1634	1695
LinJ08_V3.0450	*L. infantum*	69	1755	1823
LinJ08_V3.0450	*L. infantum*	63	2207	2269
LinJ08_V3.0450	*L. infantum*	48	2363	2410
LinJ08_V3.0490	*L. infantum*	42	75	116
LinJ08_V3.0490	*L. infantum*	111	172	282
LinJ08_V3.0490	*L. infantum*	111	172	282
LinJ08_V3.0500	*L. infantum*	238	1	238
LinJ08_V3.0510	*L. infantum*	103	1	103
LinJ08_V3.0510	*L. infantum*	82	105	186
LinJ08_V3.0510	*L. infantum*	126	205	330
LinJ08_V3.0520	*L. infantum*	68	63	130
LinJ08_V3.0520	*L. infantum*	74	567	640
LinJ08_V3.0520	*L. infantum*	59	653	711
LinJ08_V3.0530	*L. infantum*	87	351	437
LinJ08_V3.0530	*L. infantum*	109	523	631
LinJ08_V3.0530	*L. infantum*	57	662	718
LinJ08_V3.0530	*L. infantum*	54	916	969
LinJ08_V3.0530	*L. infantum*	77	1026	1102

LinJ08_V3.0530	*L. infantum*	126	1138	1263
LinJ08_V3.0530	*L. infantum*	76	1298	1373
LinJ08_V3.0530	*L. infantum*	59	1511	1569
LinJ08_V3.0530	*L. infantum*	63	1587	1649
LinJ08_V3.0530	*L. infantum*	132	1758	1889
LinJ08_V3.0530	*L. infantum*	152	1906	2057
LinJ08_V3.0530	*L. infantum*	47	2090	2136
LinJ08_V3.0530	*L. infantum*	43	2169	2211
LinJ08_V3.0530	*L. infantum*	60	2269	2328
LinJ08_V3.0540	*L. infantum*	128	1	128
LinJ08_V3.0540	*L. infantum*	210	199	408
LinJ08_V3.0540	*L. infantum*	645	497	1141
LinJ08_V3.0540	*L. infantum*	309	1159	1467
LinJ08_V3.0540	*L. infantum*	192	1470	1661
LinJ08_V3.0540	*L. infantum*	115	1678	1792
LinJ08_V3.0540	*L. infantum*	56	1807	1862
LinJ08_V3.0550	*L. infantum*	66	49	114
LinJ08_V3.0550	*L. infantum*	64	125	188
LinJ08_V3.0550	*L. infantum*	85	268	352
LinJ08_V3.0560	*L. infantum*	48	1	48
LinJ08_V3.0560	*L. infantum*	44	50	93
LinJ08_V3.0560	*L. infantum*	44	50	93
LinJ08_V3.0570	*L. infantum*	210	1	210
LinJ08_V3.0580	*L. infantum*	42	1	42
LinJ08_V3.0580	*L. infantum*	53	92	144
LinJ08_V3.0580	*L. infantum*	53	92	144
LinJ08_V3.0590	*L. infantum*	63	117	179
LinJ08_V3.0600	*L. infantum*	60	89	148
LinJ08_V3.0610	*L. infantum*	292	69	360
LinJ08_V3.0610	*L. infantum*	111	406	516
LinJ08_V3.0620	*L. infantum*	52	92	143
LinJ08_V3.0630	*L. infantum*	119	66	184
LinJ08_V3.0630	*L. infantum*	48	200	247
LinJ08_V3.0630	*L. infantum*	151	305	455
LinJ08_V3.0630	*L. infantum*	253	475	727
LinJ08_V3.0630	*L. infantum*	71	904	974
LinJ08_V3.0630	*L. infantum*	95	1054	1148
LinJ08_V3.0630	*L. infantum*	57	1177	1233
LinJ08_V3.0630	*L. infantum*	1525	1312	2836
LinJ08_V3.0640	*L. infantum*	120	178	297
LinJ08_V3.0640	*L. infantum*	40	320	359
LinJ08_V3.0640	*L. infantum*	150	388	537
LinJ08_V3.0640	*L. infantum*	421	692	1112
LinJ08_V3.0640	*L. infantum*	43	1425	1467
LinJ08_V3.0640	*L. infantum*	337	1546	1882
LinJ08_V3.0640	*L. infantum*	251	1921	2171
LinJ08_V3.0660	*L. infantum*	224	60	283

LinJ08_V3.0670	*L. infantum*	62	1	62
LinJ08_V3.0670	*L. infantum*	48	586	633
LinJ08_V3.0670	*L. infantum*	72	921	992
LinJ08_V3.0740	*L. infantum*	54	1	54
LinJ08_V3.0740	*L. infantum*	43	224	266
LinJ08_V3.0740	*L. infantum*	43	224	266
LinJ08_V3.0750	*L. infantum*	76	168	243
LinJ08_V3.0750	*L. infantum*	93	250	342
LinJ08_V3.0750	*L. infantum*	93	250	342
LinJ08_V3.0800	*L. infantum*	290	1	290
LinJ08_V3.0810	*L. infantum*	62	73	134
LinJ08_V3.0810	*L. infantum*	47	184	230
LinJ08_V3.0810	*L. infantum*	111	309	419
LinJ08_V3.0820	*L. infantum*	60	77	136
LinJ08_V3.0820	*L. infantum*	58	269	326
LinJ08_V3.0820	*L. infantum*	49	400	448
LinJ08_V3.0830	*L. infantum*	58	39	96
LinJ08_V3.0830	*L. infantum*	44	333	376
LinJ08_V3.0830	*L. infantum*	44	333	376
LinJ08_V3.0840	*L. infantum*	645	36	680
LinJ08_V3.0840	*L. infantum*	252	701	952
LinJ08_V3.0840	*L. infantum*	46	1003	1048
LinJ08_V3.0840	*L. infantum*	85	1379	1463
LinJ08_V3.0850	*L. infantum*	128	27	154
LinJ08_V3.0850	*L. infantum*	80	260	339
LinJ08_V3.0850	*L. infantum*	114	401	514
LinJ08_V3.0850	*L. infantum*	59	547	605
LinJ08_V3.0850	*L. infantum*	47	623	669
LinJ08_V3.0850	*L. infantum*	54	697	750
LinJ08_V3.0850	*L. infantum*	101	808	908
LinJ08_V3.0850	*L. infantum*	59	955	1013
LinJ08_V3.0850	*L. infantum*	190	1031	1220
LinJ08_V3.0850	*L. infantum*	175	1268	1442
LinJ08_V3.0850	*L. infantum*	95	1536	1630
LinJ08_V3.0850	*L. infantum*	42	1632	1673
LinJ08_V3.0850	*L. infantum*	68	1716	1783
LinJ08_V3.0850	*L. infantum*	56	1801	1856
LinJ08_V3.0850	*L. infantum*	96	1922	2017
LinJ08_V3.0850	*L. infantum*	73	2049	2121
LinJ08_V3.0860	*L. infantum*	47	158	204
LinJ08_V3.0870	*L. infantum*	317	1	317
LinJ08_V3.0870	*L. infantum*	136	422	557
LinJ08_V3.0880	*L. infantum*	116	121	236
LinJ08_V3.0890	*L. infantum*	146	1	146
LinJ08_V3.0900	*L. infantum*	152	42	193
LinJ08_V3.0900	*L. infantum*	47	196	242
LinJ08_V3.0900	*L. infantum*	44	318	361

LinJ08_V3.0900	*L. infantum*	55	379	433
LinJ08_V3.0900	*L. infantum*	50	470	519
LinJ08_V3.0910	*L. infantum*	359	26	384
LinJ08_V3.0910	*L. infantum*	213	438	650
LinJ08_V3.0910	*L. infantum*	58	776	833
LinJ08_V3.0920	*L. infantum*	95	1	95
LinJ08_V3.0920	*L. infantum*	926	115	1040
LinJ08_V3.0930	*L. infantum*	51	86	136
LinJ08_V3.0930	*L. infantum*	117	210	326
LinJ08_V3.0930	*L. infantum*	117	210	326
LinJ08_V3.0960	*L. infantum*	67	339	405
LinJ08_V3.1000	*L. infantum*	58	1	58
LinJ08_V3.1000	*L. infantum*	66	561	626
LinJ08_V3.1010	*L. infantum*	45	54	98
LinJ08_V3.1010	*L. infantum*	69	297	365
LinJ08_V3.1010	*L. infantum*	69	297	365
LinJ08_V3.1020	*L. infantum*	60	184	243
LinJ08_V3.1020	*L. infantum*	43	334	376
LinJ08_V3.1020	*L. infantum*	53	469	521
LinJ08_V3.1030	*L. infantum*	62	42	103
LinJ08_V3.1030	*L. infantum*	57	426	482
LinJ08_V3.1030	*L. infantum*	46	709	754
LinJ08_V3.1050	*L. infantum*	328	381	708
LinJ08_V3.1050	*L. infantum*	62	733	794
LinJ08_V3.1060	*L. infantum*	57	221	277
LinJ08_V3.1060	*L. infantum*	127	469	595
LinJ08_V3.1060	*L. infantum*	145	631	775
LinJ08_V3.1070	*L. infantum*	88	126	213
LinJ08_V3.1090	*L. infantum*	45	106	150
LinJ08_V3.1090	*L. infantum*	104	265	368
LinJ08_V3.1090	*L. infantum*	104	265	368
LinJ08_V3.1100	*L. infantum*	94	1060	1153
LinJ08_V3.1120	*L. infantum*	75	135	209
LinJ08_V3.1120	*L. infantum*	48	233	280
LinJ08_V3.1130	*L. infantum*	50	118	167
LinJ08_V3.1130	*L. infantum*	101	169	269
LinJ08_V3.1130	*L. infantum*	151	356	506
LinJ08_V3.1160	*L. infantum*	255	1	255
LinJ08_V3.1160	*L. infantum*	122	352	473
LinJ08_V3.1250	*L. infantum*	89	193	281
LinJ08_V3.1250	*L. infantum*	44	506	549
LinJ08_V3.1250	*L. infantum*	44	506	549
LinJ08_V3.1280	*L. infantum*	40	404	443
LinJ08_V3.1290	*L. infantum*	40	404	443
LinJ08_V3.1300	*L. infantum*	60	42	101
LinJ08_V3.1300	*L. infantum*	156	313	468
LinJ08_V3.1310	*L. infantum*	189	51	239

LinJ09_V3.0010	*L. infantum*	67	1	67
LinJ09_V3.0040	*L. infantum*	85	479	563
LinJ09_V3.0050	*L. infantum*	55	196	250
LinJ09_V3.0080	*L. infantum*	46	86	131
LinJ09_V3.0090	*L. infantum*	125	209	333
LinJ09_V3.0090	*L. infantum*	43	527	569
LinJ09_V3.0090	*L. infantum*	43	527	569
LinJ09_V3.0100	*L. infantum*	78	107	184
LinJ09_V3.0110	*L. infantum*	62	38	99
LinJ09_V3.0110	*L. infantum*	90	178	267
LinJ09_V3.0110	*L. infantum*	90	178	267
LinJ09_V3.0120	*L. infantum*	87	92	178
LinJ09_V3.0120	*L. infantum*	322	261	582
LinJ09_V3.0130	*L. infantum*	77	1	77
LinJ09_V3.0140	*L. infantum*	225	26	250
LinJ09_V3.0140	*L. infantum*	205	265	469
LinJ09_V3.0140	*L. infantum*	203	509	711
LinJ09_V3.0150	*L. infantum*	44	174	217
LinJ09_V3.0150	*L. infantum*	40	286	325
LinJ09_V3.0150	*L. infantum*	134	440	573
LinJ09_V3.0150	*L. infantum*	240	637	876
LinJ09_V3.0150	*L. infantum*	165	920	1084
LinJ09_V3.0160	*L. infantum*	64	244	307
LinJ09_V3.0160	*L. infantum*	43	321	363
LinJ09_V3.0160	*L. infantum*	43	321	363
LinJ09_V3.0230	*L. infantum*	78	52	129
LinJ09_V3.0230	*L. infantum*	107	171	277
LinJ09_V3.0230	*L. infantum*	107	171	277
LinJ09_V3.0240	*L. infantum*	72	133	204
LinJ09_V3.0240	*L. infantum*	183	257	439
LinJ09_V3.0240	*L. infantum*	42	476	517
LinJ09_V3.0240	*L. infantum*	119	603	721
LinJ09_V3.0250	*L. infantum*	78	1	78
LinJ09_V3.0250	*L. infantum*	128	243	370
LinJ09_V3.0250	*L. infantum*	192	428	619
LinJ09_V3.0250	*L. infantum*	488	656	1143
LinJ09_V3.0250	*L. infantum*	72	1185	1256
LinJ09_V3.0260	*L. infantum*	58	54	111
LinJ09_V3.0260	*L. infantum*	74	132	205
LinJ09_V3.0260	*L. infantum*	74	132	205
LinJ09_V3.0270	*L. infantum*	45	1	45
LinJ09_V3.0270	*L. infantum*	42	290	331
LinJ09_V3.0270	*L. infantum*	42	290	331
LinJ09_V3.0280	*L. infantum*	68	1	68
LinJ09_V3.0280	*L. infantum*	95	678	772
LinJ09_V3.0290	*L. infantum*	175	534	708
LinJ09_V3.0300	*L. infantum*	227	515	741

LinJ09_V3.0300	*L. infantum*	94	767	860
LinJ09_V3.0310	*L. infantum*	47	1	47
LinJ09_V3.0310	*L. infantum*	60	109	168
LinJ09_V3.0310	*L. infantum*	62	190	251
LinJ09_V3.0320	*L. infantum*	611	1	611
LinJ09_V3.0320	*L. infantum*	173	661	833
LinJ09_V3.0330	*L. infantum*	126	1	126
LinJ09_V3.0330	*L. infantum*	45	179	223
LinJ09_V3.0340	*L. infantum*	82	1	82
LinJ09_V3.0360	*L. infantum*	88	1	88
LinJ09_V3.0380	*L. infantum*	59	169	227
LinJ09_V3.0390	*L. infantum*	166	1	166
LinJ09_V3.0390	*L. infantum*	124	278	401
LinJ09_V3.0390	*L. infantum*	71	753	823
LinJ09_V3.0400	*L. infantum*	72	609	680
LinJ09_V3.0410	*L. infantum*	109	1	109
LinJ09_V3.0420	*L. infantum*	72	273	344
LinJ09_V3.0420	*L. infantum*	63	559	621
LinJ09_V3.0420	*L. infantum*	63	559	621
LinJ09_V3.0440	*L. infantum*	46	1	46
LinJ09_V3.0440	*L. infantum*	290	76	365
LinJ09_V3.0440	*L. infantum*	221	497	717
LinJ09_V3.0440	*L. infantum*	67	749	815
LinJ09_V3.0440	*L. infantum*	61	923	983
LinJ09_V3.0440	*L. infantum*	118	1036	1153
LinJ09_V3.0440	*L. infantum*	160	1162	1321
LinJ09_V3.0440	*L. infantum*	48	1464	1511
LinJ09_V3.0440	*L. infantum*	182	1572	1753
LinJ09_V3.0440	*L. infantum*	81	1835	1915
LinJ09_V3.0450	*L. infantum*	105	1	105
LinJ09_V3.0460	*L. infantum*	92	25	116
LinJ09_V3.0470	*L. infantum*	79	1	79
LinJ09_V3.0470	*L. infantum*	62	223	284
LinJ09_V3.0470	*L. infantum*	88	477	564
LinJ09_V3.0470	*L. infantum*	130	642	771
LinJ09_V3.0470	*L. infantum*	92	777	868
LinJ09_V3.0470	*L. infantum*	241	893	1133
LinJ09_V3.0480	*L. infantum*	237	138	374
LinJ09_V3.0490	*L. infantum*	71	112	182
LinJ09_V3.0490	*L. infantum*	43	251	293
LinJ09_V3.0490	*L. infantum*	43	251	293
LinJ09_V3.0500	*L. infantum*	103	110	212
LinJ09_V3.0500	*L. infantum*	258	280	537
LinJ09_V3.0500	*L. infantum*	59	571	629
LinJ09_V3.0500	*L. infantum*	75	642	716
LinJ09_V3.0500	*L. infantum*	68	748	815
LinJ09_V3.0500	*L. infantum*	104	831	934

LinJ09_V3.0520	*L. infantum*	114	1	114
LinJ09_V3.0520	*L. infantum*	113	139	251
LinJ09_V3.0530	*L. infantum*	55	39	93
LinJ09_V3.0530	*L. infantum*	289	298	586
LinJ09_V3.0550	*L. infantum*	75	1	75
LinJ09_V3.0550	*L. infantum*	68	208	275
LinJ09_V3.0550	*L. infantum*	51	314	364
LinJ09_V3.0550	*L. infantum*	75	391	465
LinJ09_V3.0550	*L. infantum*	92	478	569
LinJ09_V3.0550	*L. infantum*	100	922	1021
LinJ09_V3.0550	*L. infantum*	54	1100	1153
LinJ09_V3.0550	*L. infantum*	43	1207	1249
LinJ09_V3.0550	*L. infantum*	220	1286	1505
LinJ09_V3.0560	*L. infantum*	153	165	317
LinJ09_V3.0570	*L. infantum*	50	73	122
LinJ09_V3.0570	*L. infantum*	65	164	228
LinJ09_V3.0570	*L. infantum*	98	600	697
LinJ09_V3.0570	*L. infantum*	61	710	770
LinJ09_V3.0580	*L. infantum*	125	1	125
LinJ09_V3.0580	*L. infantum*	48	131	178
LinJ09_V3.0580	*L. infantum*	58	480	537
LinJ09_V3.0580	*L. infantum*	247	751	997
LinJ09_V3.0590	*L. infantum*	64	1	64
LinJ09_V3.0590	*L. infantum*	419	86	504
LinJ09_V3.0590	*L. infantum*	249	515	763
LinJ09_V3.0590	*L. infantum*	183	818	1000
LinJ09_V3.0590	*L. infantum*	52	1058	1109
LinJ09_V3.0600	*L. infantum*	89	42	130
LinJ09_V3.0600	*L. infantum*	121	137	257
LinJ09_V3.0600	*L. infantum*	79	267	345
LinJ09_V3.0610	*L. infantum*	45	133	177
LinJ09_V3.0620	*L. infantum*	75	85	159
LinJ09_V3.0620	*L. infantum*	139	211	349
LinJ09_V3.0640	*L. infantum*	220	596	815
LinJ09_V3.0650	*L. infantum*	48	288	335
LinJ09_V3.0660	*L. infantum*	88	1	88
LinJ09_V3.0660	*L. infantum*	136	159	294
LinJ09_V3.0660	*L. infantum*	49	813	861
LinJ09_V3.0670	*L. infantum*	88	1	88
LinJ09_V3.0670	*L. infantum*	115	125	239
LinJ09_V3.0670	*L. infantum*	65	383	447
LinJ09_V3.0670	*L. infantum*	43	522	564
LinJ09_V3.0680	*L. infantum*	108	53	160
LinJ09_V3.0680	*L. infantum*	42	206	247
LinJ09_V3.0680	*L. infantum*	62	343	404
LinJ09_V3.0680	*L. infantum*	157	537	693
LinJ09_V3.0680	*L. infantum*	279	697	975

LinJ09_V3.0690	*L. infantum*	52	141	192
LinJ09_V3.0690	*L. infantum*	56	408	463
LinJ09_V3.0690	*L. infantum*	56	408	463
LinJ09_V3.0700	*L. infantum*	79	474	552
LinJ09_V3.0710	*L. infantum*	68	1	68
LinJ09_V3.0720	*L. infantum*	48	1	48
LinJ09_V3.0720	*L. infantum*	44	95	138
LinJ09_V3.0730	*L. infantum*	72	59	130
LinJ09_V3.0730	*L. infantum*	45	182	226
LinJ09_V3.0730	*L. infantum*	81	258	338
LinJ09_V3.0750	*L. infantum*	86	1	86
LinJ09_V3.0750	*L. infantum*	88	107	194
LinJ09_V3.0750	*L. infantum*	236	280	515
LinJ09_V3.0760	*L. infantum*	84	1	84
LinJ09_V3.0760	*L. infantum*	48	105	152
LinJ09_V3.0760	*L. infantum*	102	217	318
LinJ09_V3.0760	*L. infantum*	59	338	396
LinJ09_V3.0760	*L. infantum*	52	428	479
LinJ09_V3.0770	*L. infantum*	48	1	48
LinJ09_V3.0770	*L. infantum*	129	208	336
LinJ09_V3.0780	*L. infantum*	155	415	569
LinJ09_V3.0790	*L. infantum*	45	279	323
LinJ09_V3.0790	*L. infantum*	65	894	958
LinJ09_V3.0790	*L. infantum*	41	1049	1089
LinJ09_V3.0790	*L. infantum*	45	1245	1289
LinJ09_V3.0790	*L. infantum*	56	1335	1390
LinJ09_V3.0790	*L. infantum*	83	1544	1626
LinJ09_V3.0790	*L. infantum*	44	1735	1778
LinJ09_V3.0800	*L. infantum*	53	216	268
LinJ09_V3.0800	*L. infantum*	53	429	481
LinJ09_V3.0800	*L. infantum*	75	787	861
LinJ09_V3.0810	*L. infantum*	73	24	96
LinJ09_V3.0810	*L. infantum*	104	116	219
LinJ09_V3.0810	*L. infantum*	78	273	350
LinJ09_V3.0810	*L. infantum*	86	689	774
LinJ09_V3.0810	*L. infantum*	107	813	919
LinJ09_V3.0810	*L. infantum*	65	949	1013
LinJ09_V3.0810	*L. infantum*	57	1227	1283
LinJ09_V3.0810	*L. infantum*	96	1386	1481
LinJ09_V3.0810	*L. infantum*	57	1570	1626
LinJ09_V3.0810	*L. infantum*	65	1736	1800
LinJ09_V3.0810	*L. infantum*	42	1816	1857
LinJ09_V3.0810	*L. infantum*	75	1996	2070
LinJ09_V3.0810	*L. infantum*	65	2181	2245
LinJ09_V3.0810	*L. infantum*	65	2257	2321
LinJ09_V3.0810	*L. infantum*	82	2352	2433
LinJ09_V3.0810	*L. infantum*	52	2519	2570

LinJ09_V3.0810	*L. infantum*	75	2626	2700
LinJ09_V3.0810	*L. infantum*	50	2785	2834
LinJ09_V3.0810	*L. infantum*	57	2968	3024
LinJ09_V3.0810	*L. infantum*	49	3105	3153
LinJ09_V3.0810	*L. infantum*	88	3213	3300
LinJ09_V3.0810	*L. infantum*	64	3579	3642
LinJ09_V3.0810	*L. infantum*	79	3720	3798
LinJ09_V3.0810	*L. infantum*	78	3821	3898
LinJ09_V3.0810	*L. infantum*	44	3943	3986
LinJ09_V3.0810	*L. infantum*	40	4160	4199
LinJ09_V3.0810	*L. infantum*	74	4221	4294
LinJ09_V3.0810	*L. infantum*	44	4483	4526
LinJ09_V3.0810	*L. infantum*	93	4538	4630
LinJ09_V3.0810	*L. infantum*	60	4688	4747
LinJ09_V3.0810	*L. infantum*	78	4869	4946
LinJ09_V3.0810	*L. infantum*	99	4965	5063
LinJ09_V3.0810	*L. infantum*	261	5102	5362
LinJ09_V3.0810	*L. infantum*	56	5405	5460
LinJ09_V3.0840	*L. infantum*	79	1	79
LinJ09_V3.0840	*L. infantum*	143	96	238
LinJ09_V3.0840	*L. infantum*	96	244	339
LinJ09_V3.0840	*L. infantum*	68	377	444
LinJ09_V3.0840	*L. infantum*	70	469	538
LinJ09_V3.0840	*L. infantum*	72	1079	1150
LinJ09_V3.0850	*L. infantum*	193	10	202
LinJ09_V3.0850	*L. infantum*	180	310	489
LinJ09_V3.0850	*L. infantum*	78	526	603
LinJ09_V3.0850	*L. infantum*	306	681	986
LinJ09_V3.0850	*L. infantum*	212	1030	1241
LinJ09_V3.0860	*L. infantum*	44	83	126
LinJ09_V3.0870	*L. infantum*	41	24	64
LinJ09_V3.0870	*L. infantum*	88	257	344
LinJ09_V3.0870	*L. infantum*	42	489	530
LinJ09_V3.0880	*L. infantum*	51	1	51
LinJ09_V3.0880	*L. infantum*	60	410	469
LinJ09_V3.0880	*L. infantum*	57	720	776
LinJ09_V3.0900	*L. infantum*	78	153	230
LinJ09_V3.0920	*L. infantum*	83	1	83
LinJ09_V3.0920	*L. infantum*	74	104	177
LinJ09_V3.0920	*L. infantum*	63	191	253
LinJ09_V3.0920	*L. infantum*	165	255	419
LinJ09_V3.0930	*L. infantum*	76	1	76
LinJ09_V3.0930	*L. infantum*	375	116	490
LinJ09_V3.0940	*L. infantum*	131	163	293
LinJ09_V3.0940	*L. infantum*	310	425	734
LinJ09_V3.0940	*L. infantum*	130	995	1124
LinJ09_V3.0940	*L. infantum*	99	1217	1315

LinJ09_V3.0940	*L. infantum*	64	1341	1404
LinJ09_V3.0940	*L. infantum*	63	1440	1502
LinJ09_V3.0940	*L. infantum*	223	1586	1808
LinJ09_V3.0940	*L. infantum*	88	1975	2062
LinJ09_V3.0940	*L. infantum*	64	2465	2528
LinJ09_V3.0960	*L. infantum*	62	105	166
LinJ09_V3.0970	*L. infantum*	89	1	89
LinJ09_V3.0980	*L. infantum*	89	1	89
LinJ09_V3.0990	*L. infantum*	50	526	575
LinJ09_V3.0990	*L. infantum*	355	737	1091
LinJ09_V3.0990	*L. infantum*	65	1139	1203
LinJ09_V3.1000	*L. infantum*	53	159	211
LinJ09_V3.1000	*L. infantum*	42	246	287
LinJ09_V3.1000	*L. infantum*	40	445	484
LinJ09_V3.1010	*L. infantum*	46	33	78
LinJ09_V3.1010	*L. infantum*	57	170	226
LinJ09_V3.1010	*L. infantum*	83	458	540
LinJ09_V3.1020	*L. infantum*	53	1	53
LinJ09_V3.1020	*L. infantum*	60	252	311
LinJ09_V3.1020	*L. infantum*	60	252	311
LinJ09_V3.1030	*L. infantum*	60	204	263
LinJ09_V3.1040	*L. infantum*	268	21	288
LinJ09_V3.1040	*L. infantum*	65	307	371
LinJ09_V3.1040	*L. infantum*	46	380	425
LinJ09_V3.1040	*L. infantum*	101	438	538
LinJ09_V3.1040	*L. infantum*	392	560	951
LinJ09_V3.1050	*L. infantum*	48	879	926
LinJ09_V3.1050	*L. infantum*	65	1042	1106
LinJ09_V3.1050	*L. infantum*	131	1166	1296
LinJ09_V3.1050	*L. infantum*	89	1728	1816
LinJ09_V3.1050	*L. infantum*	61	1894	1954
LinJ09_V3.1050	*L. infantum*	134	1971	2104
LinJ09_V3.1050	*L. infantum*	83	2147	2229
LinJ09_V3.1050	*L. infantum*	80	2318	2397
LinJ09_V3.1050	*L. infantum*	97	2600	2696
LinJ09_V3.1050	*L. infantum*	192	2818	3009
LinJ09_V3.1050	*L. infantum*	174	3130	3303
LinJ09_V3.1050	*L. infantum*	179	3526	3704
LinJ09_V3.1050	*L. infantum*	43	4196	4238
LinJ09_V3.1050	*L. infantum*	84	4680	4763
LinJ09_V3.1050	*L. infantum*	206	4853	5058
LinJ09_V3.1050	*L. infantum*	112	5073	5184
LinJ09_V3.1050	*L. infantum*	70	5294	5363
LinJ09_V3.1060	*L. infantum*	56	128	183
LinJ09_V3.1070	*L. infantum*	122	28	149
LinJ09_V3.1080	*L. infantum*	106	1	106
LinJ09_V3.1090	*L. infantum*	264	1	264

LinJ09_V3.1100	*L. infantum*	94	1	94
LinJ09_V3.1110	*L. infantum*	71	136	206
LinJ09_V3.1120	*L. infantum*	49	178	226
LinJ09_V3.1140	*L. infantum*	59	392	450
LinJ09_V3.1160	*L. infantum*	40	1	40
LinJ09_V3.1160	*L. infantum*	44	44	87
LinJ09_V3.1160	*L. infantum*	117	110	226
LinJ09_V3.1170	*L. infantum*	185	1	185
LinJ09_V3.1170	*L. infantum*	241	218	458
LinJ09_V3.1180	*L. infantum*	45	34	78
LinJ09_V3.1200	*L. infantum*	57	1	57
LinJ09_V3.1200	*L. infantum*	41	142	182
LinJ09_V3.1210	*L. infantum*	58	1	58
LinJ09_V3.1210	*L. infantum*	52	129	180
LinJ09_V3.1210	*L. infantum*	68	218	285
LinJ09_V3.1220	*L. infantum*	89	1	89
LinJ09_V3.1220	*L. infantum*	73	227	299
LinJ09_V3.1220	*L. infantum*	59	446	504
LinJ09_V3.1220	*L. infantum*	48	538	585
LinJ09_V3.1220	*L. infantum*	47	693	739
LinJ09_V3.1230	*L. infantum*	40	39	78
LinJ09_V3.1240	*L. infantum*	371	1	371
LinJ09_V3.1240	*L. infantum*	44	379	422
LinJ09_V3.1240	*L. infantum*	132	443	574
LinJ09_V3.1250	*L. infantum*	43	1	43
LinJ09_V3.1250	*L. infantum*	85	117	201
LinJ09_V3.1250	*L. infantum*	61	377	437
LinJ09_V3.1250	*L. infantum*	80	444	523
LinJ09_V3.1260	*L. infantum*	114	53	166
LinJ09_V3.1260	*L. infantum*	91	203	293
LinJ09_V3.1260	*L. infantum*	75	627	701
LinJ09_V3.1260	*L. infantum*	50	890	939
LinJ09_V3.1260	*L. infantum*	81	966	1046
LinJ09_V3.1260	*L. infantum*	60	1070	1129
LinJ09_V3.1260	*L. infantum*	62	1223	1284
LinJ09_V3.1260	*L. infantum*	99	1423	1521
LinJ09_V3.1270	*L. infantum*	187	234	420
LinJ09_V3.1280	*L. infantum*	49	80	128
LinJ09_V3.1280	*L. infantum*	75	334	408
LinJ09_V3.1280	*L. infantum*	141	423	563
LinJ09_V3.1290	*L. infantum*	74	1	74
LinJ09_V3.1290	*L. infantum*	53	360	412
LinJ09_V3.1300	*L. infantum*	72	114	185
LinJ09_V3.1300	*L. infantum*	51	566	616
LinJ09_V3.1300	*L. infantum*	48	1069	1116
LinJ09_V3.1300	*L. infantum*	256	1192	1447
LinJ09_V3.1300	*L. infantum*	50	1557	1606

LinJ09_V3.1300	*L. infantum*	71	1608	1678
LinJ09_V3.1300	*L. infantum*	53	2021	2073
LinJ09_V3.1300	*L. infantum*	47	2202	2248
LinJ09_V3.1300	*L. infantum*	80	2403	2482
LinJ09_V3.1320	*L. infantum*	58	127	184
LinJ09_V3.1320	*L. infantum*	172	298	469
LinJ09_V3.1320	*L. infantum*	172	298	469
LinJ09_V3.1340	*L. infantum*	49	1	49
LinJ09_V3.1340	*L. infantum*	78	77	154
LinJ09_V3.1340	*L. infantum*	96	211	306
LinJ09_V3.1350	*L. infantum*	64	85	148
LinJ09_V3.1350	*L. infantum*	111	194	304
LinJ09_V3.1350	*L. infantum*	58	318	375
LinJ09_V3.1370	*L. infantum*	53	202	254
LinJ09_V3.1390	*L. infantum*	109	247	355
LinJ09_V3.1390	*L. infantum*	66	540	605
LinJ09_V3.1390	*L. infantum*	66	540	605
LinJ09_V3.1400	*L. infantum*	46	179	224
LinJ09_V3.1440	*L. infantum*	73	1	73
LinJ09_V3.1440	*L. infantum*	131	138	268
LinJ09_V3.1450	*L. infantum*	168	23	190
LinJ09_V3.1450	*L. infantum*	57	476	532
LinJ09_V3.1450	*L. infantum*	57	476	532
LinJ09_V3.1460	*L. infantum*	73	22	94
LinJ09_V3.1470	*L. infantum*	73	131	203
LinJ09_V3.1480	*L. infantum*	53	70	122
LinJ09_V3.1480	*L. infantum*	125	147	271
LinJ09_V3.1480	*L. infantum*	40	451	490
LinJ09_V3.1480	*L. infantum*	45	529	573
LinJ09_V3.1490	*L. infantum*	108	32	139
LinJ09_V3.1520	*L. infantum*	164	57	220
LinJ09_V3.1520	*L. infantum*	165	249	413
LinJ09_V3.1560	*L. infantum*	43	350	392
LinJ09_V3.1560	*L. infantum*	41	528	568
LinJ09_V3.1560	*L. infantum*	41	528	568
LinJ09_V3.1590	*L. infantum*	48	1	48
LinJ09_V3.1600	*L. infantum*	71	136	206
LinJ09_V3.1600	*L. infantum*	93	1069	1161
LinJ09_V3.1620	*L. infantum*	51	55	105
LinJ09_V3.1630	*L. infantum*	485	1	485
LinJ09_V3.1630	*L. infantum*	110	498	607
LinJ09_V3.1630	*L. infantum*	110	498	607
LinJ09_V3.1640	*L. infantum*	70	1	70
LinJ10_V3.0010	*L. infantum*	58	258	315
LinJ10_V3.0080	*L. infantum*	238	1	238
LinJ10_V3.0100	*L. infantum*	57	1	57
LinJ10_V3.0100	*L. infantum*	70	67	136

LinJ10_V3.0100	*L. infantum*	60	202	261
LinJ10_V3.0110	*L. infantum*	59	161	219
LinJ10_V3.0110	*L. infantum*	116	265	380
LinJ10_V3.0110	*L. infantum*	247	398	644
LinJ10_V3.0110	*L. infantum*	74	664	737
LinJ10_V3.0120	*L. infantum*	83	1	83
LinJ10_V3.0120	*L. infantum*	127	123	249
LinJ10_V3.0120	*L. infantum*	40	356	395
LinJ10_V3.0130	*L. infantum*	50	1	50
LinJ10_V3.0130	*L. infantum*	262	55	316
LinJ10_V3.0130	*L. infantum*	47	323	369
LinJ10_V3.0140	*L. infantum*	458	48	505
LinJ10_V3.0150	*L. infantum*	107	411	517
LinJ10_V3.0180	*L. infantum*	52	44	95
LinJ10_V3.0180	*L. infantum*	108	109	216
LinJ10_V3.0180	*L. infantum*	301	274	574
LinJ10_V3.0190	*L. infantum*	89	1	89
LinJ10_V3.0200	*L. infantum*	50	358	407
LinJ10_V3.0210	*L. infantum*	54	420	473
LinJ10_V3.0220	*L. infantum*	42	70	111
LinJ10_V3.0220	*L. infantum*	48	168	215
LinJ10_V3.0220	*L. infantum*	48	168	215
LinJ10_V3.0230	*L. infantum*	53	226	278
LinJ10_V3.0240	*L. infantum*	79	22	100
LinJ10_V3.0250	*L. infantum*	44	1	44
LinJ10_V3.0260	*L. infantum*	374	114	487
LinJ10_V3.0260	*L. infantum*	129	540	668
LinJ10_V3.0260	*L. infantum*	42	695	736
LinJ10_V3.0270	*L. infantum*	73	36	108
LinJ10_V3.0270	*L. infantum*	48	212	259
LinJ10_V3.0270	*L. infantum*	48	212	259
LinJ10_V3.0290	*L. infantum*	92	1	92
LinJ10_V3.0290	*L. infantum*	52	154	205
LinJ10_V3.0290	*L. infantum*	115	239	353
LinJ10_V3.0290	*L. infantum*	68	378	445
LinJ10_V3.0290	*L. infantum*	85	660	744
LinJ10_V3.0290	*L. infantum*	48	761	808
LinJ10_V3.0290	*L. infantum*	41	857	897
LinJ10_V3.0300	*L. infantum*	75	106	180
LinJ10_V3.0300	*L. infantum*	86	192	277
LinJ10_V3.0300	*L. infantum*	432	279	710
LinJ10_V3.0300	*L. infantum*	52	737	788
LinJ10_V3.0300	*L. infantum*	98	970	1067
LinJ10_V3.0300	*L. infantum*	45	1127	1171
LinJ10_V3.0300	*L. infantum*	121	1253	1373
LinJ10_V3.0300	*L. infantum*	47	1461	1507
LinJ10_V3.0300	*L. infantum*	123	1535	1657

LinJ10_V3.0300	*L. infantum*	173	1806	1978
LinJ10_V3.0320	*L. infantum*	263	1	263
LinJ10_V3.0320	*L. infantum*	328	276	603
LinJ10_V3.0330	*L. infantum*	51	484	534
LinJ10_V3.0340	*L. infantum*	109	89	197
LinJ10_V3.0340	*L. infantum*	178	245	422
LinJ10_V3.0340	*L. infantum*	133	432	564
LinJ10_V3.0340	*L. infantum*	180	678	857
LinJ10_V3.0340	*L. infantum*	318	880	1197
LinJ10_V3.0340	*L. infantum*	102	1264	1365
LinJ10_V3.0350	*L. infantum*	202	234	435
LinJ10_V3.0360	*L. infantum*	52	1	52
LinJ10_V3.0360	*L. infantum*	91	298	388
LinJ10_V3.0360	*L. infantum*	91	298	388
LinJ10_V3.0370	*L. infantum*	76	270	345
LinJ10_V3.0370	*L. infantum*	44	648	691
LinJ10_V3.0370	*L. infantum*	44	648	691
LinJ10_V3.0380	*L. infantum*	78	273	350
LinJ10_V3.0390	*L. infantum*	78	271	348
LinJ10_V3.0390	*L. infantum*	44	657	700
LinJ10_V3.0390	*L. infantum*	44	657	700
LinJ10_V3.0400	*L. infantum*	78	271	348
LinJ10_V3.0400	*L. infantum*	49	656	704
LinJ10_V3.0400	*L. infantum*	49	656	704
LinJ10_V3.0410	*L. infantum*	78	271	348
LinJ10_V3.0410	*L. infantum*	43	656	698
LinJ10_V3.0410	*L. infantum*	43	656	698
LinJ10_V3.0420	*L. infantum*	63	253	315
LinJ10_V3.0440	*L. infantum*	211	1	211
LinJ10_V3.0440	*L. infantum*	171	240	410
LinJ10_V3.0440	*L. infantum*	137	499	635
LinJ10_V3.0450	*L. infantum*	41	62	102
LinJ10_V3.0460	*L. infantum*	44	54	97
LinJ10_V3.0520	*L. infantum*	183	1	183
LinJ10_V3.0550	*L. infantum*	93	443	535
LinJ10_V3.0550	*L. infantum*	41	717	757
LinJ10_V3.0550	*L. infantum*	42	1114	1155
LinJ10_V3.0550	*L. infantum*	44	1424	1467
LinJ10_V3.0550	*L. infantum*	66	2380	2445
LinJ10_V3.0550	*L. infantum*	61	2479	2539
LinJ10_V3.0550	*L. infantum*	64	5591	5654
LinJ10_V3.0550	*L. infantum*	53	6228	6280
LinJ10_V3.0550	*L. infantum*	308	6495	6802
LinJ10_V3.0570	*L. infantum*	78	23	100
LinJ10_V3.0570	*L. infantum*	43	181	223
LinJ10_V3.0570	*L. infantum*	106	352	457
LinJ10_V3.0570	*L. infantum*	154	753	906

LinJ10_V3.0580	*L. infantum*	195	1	195
LinJ10_V3.0580	*L. infantum*	121	222	342
LinJ10_V3.0580	*L. infantum*	182	364	545
LinJ10_V3.0580	*L. infantum*	120	569	688
LinJ10_V3.0580	*L. infantum*	67	697	763
LinJ10_V3.0580	*L. infantum*	379	777	1155
LinJ10_V3.0590	*L. infantum*	64	235	298
LinJ10_V3.0590	*L. infantum*	121	518	638
LinJ10_V3.0590	*L. infantum*	167	762	928
LinJ10_V3.0590	*L. infantum*	129	961	1089
LinJ10_V3.0600	*L. infantum*	59	1	59
LinJ10_V3.0600	*L. infantum*	85	347	431
LinJ10_V3.0620	*L. infantum*	49	430	478
LinJ10_V3.0630	*L. infantum*	214	1	214
LinJ10_V3.0630	*L. infantum*	58	231	288
LinJ10_V3.0630	*L. infantum*	75	394	468
LinJ10_V3.0630	*L. infantum*	51	506	556
LinJ10_V3.0630	*L. infantum*	166	605	770
LinJ10_V3.0660	*L. infantum*	45	47	91
LinJ10_V3.0660	*L. infantum*	86	143	228
LinJ10_V3.0660	*L. infantum*	45	258	302
LinJ10_V3.0680	*L. infantum*	54	355	408
LinJ10_V3.0690	*L. infantum*	76	102	177
LinJ10_V3.0700	*L. infantum*	129	68	196
LinJ10_V3.0710	*L. infantum*	195	1	195
LinJ10_V3.0710	*L. infantum*	124	204	327
LinJ10_V3.0710	*L. infantum*	675	348	1022
LinJ10_V3.0710	*L. infantum*	156	1060	1215
LinJ10_V3.0710	*L. infantum*	459	1220	1678
LinJ10_V3.0720	*L. infantum*	118	1	118
LinJ10_V3.0730	*L. infantum*	67	1	67
LinJ10_V3.0730	*L. infantum*	431	75	505
LinJ10_V3.0740	*L. infantum*	41	114	154
LinJ10_V3.0740	*L. infantum*	99	215	313
LinJ10_V3.0740	*L. infantum*	45	359	403
LinJ10_V3.0740	*L. infantum*	41	710	750
LinJ10_V3.0740	*L. infantum*	105	845	949
LinJ10_V3.0740	*L. infantum*	57	1155	1211
LinJ10_V3.0740	*L. infantum*	67	1223	1289
LinJ10_V3.0740	*L. infantum*	44	1330	1373
LinJ10_V3.0740	*L. infantum*	45	1435	1479
LinJ10_V3.0750	*L. infantum*	58	13	70
LinJ10_V3.0750	*L. infantum*	254	114	367
LinJ10_V3.0750	*L. infantum*	64	445	508
LinJ10_V3.0750	*L. infantum*	93	782	874
LinJ10_V3.0760	*L. infantum*	70	1	70
LinJ10_V3.0770	*L. infantum*	70	1	70

LinJ10_V3.0780	*L. infantum*	44	103	146
LinJ10_V3.0780	*L. infantum*	41	195	235
LinJ10_V3.0780	*L. infantum*	41	195	235
LinJ10_V3.0790	*L. infantum*	75	51	125
LinJ10_V3.0800	*L. infantum*	372	22	393
LinJ10_V3.0800	*L. infantum*	207	419	625
LinJ10_V3.0800	*L. infantum*	372	663	1034
LinJ10_V3.0800	*L. infantum*	78	1037	1114
LinJ10_V3.0800	*L. infantum*	264	1148	1411
LinJ10_V3.0800	*L. infantum*	92	1461	1552
LinJ10_V3.0800	*L. infantum*	135	1587	1721
LinJ10_V3.0800	*L. infantum*	173	1803	1975
LinJ10_V3.0810	*L. infantum*	130	137	266
LinJ10_V3.0810	*L. infantum*	61	325	385
LinJ10_V3.0810	*L. infantum*	56	466	521
LinJ10_V3.0820	*L. infantum*	48	192	239
LinJ10_V3.0820	*L. infantum*	89	284	372
LinJ10_V3.0820	*L. infantum*	47	549	595
LinJ10_V3.0840	*L. infantum*	221	1	221
LinJ10_V3.0850	*L. infantum*	65	73	137
LinJ10_V3.0850	*L. infantum*	270	292	561
LinJ10_V3.0850	*L. infantum*	388	887	1274
LinJ10_V3.0860	*L. infantum*	191	61	251
LinJ10_V3.0860	*L. infantum*	78	434	511
LinJ10_V3.0860	*L. infantum*	80	629	708
LinJ10_V3.0860	*L. infantum*	84	939	1022
LinJ10_V3.0860	*L. infantum*	64	1251	1314
LinJ10_V3.0860	*L. infantum*	161	1646	1806
LinJ10_V3.0860	*L. infantum*	254	1820	2073
LinJ10_V3.0870	*L. infantum*	86	1	86
LinJ10_V3.0870	*L. infantum*	64	88	151
LinJ10_V3.0880	*L. infantum*	1278	1	1278
LinJ10_V3.0880	*L. infantum*	76	1396	1471
LinJ10_V3.0890	*L. infantum*	72	315	386
LinJ10_V3.0890	*L. infantum*	63	498	560
LinJ10_V3.0890	*L. infantum*	63	498	560
LinJ10_V3.0910	*L. infantum*	54	67	120
LinJ10_V3.0920	*L. infantum*	53	1	53
LinJ10_V3.0930	*L. infantum*	192	1	192
LinJ10_V3.0930	*L. infantum*	464	221	684
LinJ10_V3.0930	*L. infantum*	319	688	1006
LinJ10_V3.0930	*L. infantum*	96	1025	1120
LinJ10_V3.0940	*L. infantum*	41	37	77
LinJ10_V3.0950	*L. infantum*	42	1	42
LinJ10_V3.0950	*L. infantum*	61	75	135
LinJ10_V3.0950	*L. infantum*	57	158	214
LinJ10_V3.0950	*L. infantum*	372	567	938

LinJ10_V3.0950	*L. infantum*	66	951	1016
LinJ10_V3.0950	*L. infantum*	54	1065	1118
LinJ10_V3.0950	*L. infantum*	59	1154	1212
LinJ10_V3.0950	*L. infantum*	81	1231	1311
LinJ10_V3.0950	*L. infantum*	44	1348	1391
LinJ10_V3.0950	*L. infantum*	94	1393	1486
LinJ10_V3.0990	*L. infantum*	154	1	154
LinJ10_V3.0990	*L. infantum*	81	178	258
LinJ10_V3.1010	*L. infantum*	50	1	50
LinJ10_V3.1010	*L. infantum*	250	359	608
LinJ10_V3.1010	*L. infantum*	89	630	718
LinJ10_V3.1010	*L. infantum*	188	720	907
LinJ10_V3.1010	*L. infantum*	163	1080	1242
LinJ10_V3.1030	*L. infantum*	124	128	251
LinJ10_V3.1040	*L. infantum*	159	1	159
LinJ10_V3.1040	*L. infantum*	92	265	356
LinJ10_V3.1040	*L. infantum*	40	538	577
LinJ10_V3.1040	*L. infantum*	88	591	678
LinJ10_V3.1040	*L. infantum*	126	681	806
LinJ10_V3.1060	*L. infantum*	41	126	166
LinJ10_V3.1060	*L. infantum*	92	231	322
LinJ10_V3.1060	*L. infantum*	47	397	443
LinJ10_V3.1060	*L. infantum*	107	485	591
LinJ10_V3.1070	*L. infantum*	53	1	53
LinJ10_V3.1080	*L. infantum*	82	27	108
LinJ10_V3.1100	*L. infantum*	83	1	83
LinJ10_V3.1110	*L. infantum*	172	1	172
LinJ10_V3.1110	*L. infantum*	59	324	382
LinJ10_V3.1120	*L. infantum*	154	216	369
LinJ10_V3.1120	*L. infantum*	78	619	696
LinJ10_V3.1120	*L. infantum*	57	798	854
LinJ10_V3.1120	*L. infantum*	50	895	944
LinJ10_V3.1120	*L. infantum*	43	1015	1057
LinJ10_V3.1130	*L. infantum*	47	102	148
LinJ10_V3.1140	*L. infantum*	45	52	96
LinJ10_V3.1140	*L. infantum*	57	237	293
LinJ10_V3.1150	*L. infantum*	180	1	180
LinJ10_V3.1150	*L. infantum*	287	204	490
LinJ10_V3.1150	*L. infantum*	63	509	571
LinJ10_V3.1160	*L. infantum*	103	1	103
LinJ10_V3.1160	*L. infantum*	49	125	173
LinJ10_V3.1160	*L. infantum*	69	397	465
LinJ10_V3.1170	*L. infantum*	42	57	98
LinJ10_V3.1170	*L. infantum*	181	210	390
LinJ10_V3.1170	*L. infantum*	71	544	614
LinJ10_V3.1190	*L. infantum*	40	314	353
LinJ10_V3.1190	*L. infantum*	50	375	424

LinJ10_V3.1200	*L. infantum*	150	161	310
LinJ10_V3.1200	*L. infantum*	186	338	523
LinJ10_V3.1210	*L. infantum*	40	69	108
LinJ10_V3.1220	*L. infantum*	195	1	195
LinJ10_V3.1220	*L. infantum*	44	214	257
LinJ10_V3.1220	*L. infantum*	106	890	995
LinJ10_V3.1220	*L. infantum*	124	1274	1397
LinJ10_V3.1230	*L. infantum*	58	204	261
LinJ10_V3.1240	*L. infantum*	427	54	480
LinJ10_V3.1250	*L. infantum*	42	175	216
LinJ10_V3.1280	*L. infantum*	379	1	379
LinJ10_V3.1290	*L. infantum*	128	62	189
LinJ10_V3.1310	*L. infantum*	95	1	95
LinJ10_V3.1310	*L. infantum*	204	295	498
LinJ10_V3.1440	*L. infantum*	58	258	315
LinJ11_V3.0010	*L. infantum*	131	1	131
LinJ11_V3.0020	*L. infantum*	41	111	151
LinJ11_V3.0020	*L. infantum*	46	244	289
LinJ11_V3.0020	*L. infantum*	46	244	289
LinJ11_V3.0030	*L. infantum*	51	257	307
LinJ11_V3.0040	*L. infantum*	66	236	301
LinJ11_V3.0050	*L. infantum*	43	585	627
LinJ11_V3.0060	*L. infantum*	64	12	75
LinJ11_V3.0060	*L. infantum*	100	252	351
LinJ11_V3.0070	*L. infantum*	51	1	51
LinJ11_V3.0070	*L. infantum*	232	113	344
LinJ11_V3.0070	*L. infantum*	675	361	1035
LinJ11_V3.0070	*L. infantum*	114	1096	1209
LinJ11_V3.0080	*L. infantum*	86	1	86
LinJ11_V3.0080	*L. infantum*	63	163	225
LinJ11_V3.0080	*L. infantum*	63	163	225
LinJ11_V3.0090	*L. infantum*	55	151	205
LinJ11_V3.0090	*L. infantum*	51	439	489
LinJ11_V3.0090	*L. infantum*	51	439	489
LinJ11_V3.0100	*L. infantum*	82	49	130
LinJ11_V3.0140	*L. infantum*	106	39	144
LinJ11_V3.0150	*L. infantum*	634	1	634
LinJ11_V3.0150	*L. infantum*	219	662	880
LinJ11_V3.0160	*L. infantum*	70	143	212
LinJ11_V3.0170	*L. infantum*	157	122	278
LinJ11_V3.0170	*L. infantum*	61	295	355
LinJ11_V3.0170	*L. infantum*	94	368	461
LinJ11_V3.0190	*L. infantum*	43	54	96
LinJ11_V3.0190	*L. infantum*	42	420	461
LinJ11_V3.0190	*L. infantum*	55	555	609
LinJ11_V3.0200	*L. infantum*	133	1	133
LinJ11_V3.0210	*L. infantum*	46	40	85

LinJ11_V3.0230	*L. infantum*	90	53	142
LinJ11_V3.0230	*L. infantum*	42	399	440
LinJ11_V3.0230	*L. infantum*	47	450	496
LinJ11_V3.0250	*L. infantum*	234	1	234
LinJ11_V3.0250	*L. infantum*	52	239	290
LinJ11_V3.0250	*L. infantum*	175	307	481
LinJ11_V3.0250	*L. infantum*	80	540	619
LinJ11_V3.0250	*L. infantum*	46	749	794
LinJ11_V3.0250	*L. infantum*	133	852	984
LinJ11_V3.0260	*L. infantum*	43	439	481
LinJ11_V3.0290	*L. infantum*	183	1	183
LinJ11_V3.0290	*L. infantum*	66	185	250
LinJ11_V3.0290	*L. infantum*	119	256	374
LinJ11_V3.0290	*L. infantum*	61	403	463
LinJ11_V3.0300	*L. infantum*	41	1	41
LinJ11_V3.0300	*L. infantum*	155	69	223
LinJ11_V3.0310	*L. infantum*	61	1	61
LinJ11_V3.0310	*L. infantum*	74	92	165
LinJ11_V3.0310	*L. infantum*	181	377	557
LinJ11_V3.0310	*L. infantum*	55	560	614
LinJ11_V3.0310	*L. infantum*	62	643	704
LinJ11_V3.0310	*L. infantum*	95	724	818
LinJ11_V3.0310	*L. infantum*	56	961	1016
LinJ11_V3.0310	*L. infantum*	44	1336	1379
LinJ11_V3.0310	*L. infantum*	57	1396	1452
LinJ11_V3.0310	*L. infantum*	44	1494	1537
LinJ11_V3.0310	*L. infantum*	50	1756	1805
LinJ11_V3.0320	*L. infantum*	152	56	207
LinJ11_V3.0320	*L. infantum*	53	228	280
LinJ11_V3.0320	*L. infantum*	71	288	358
LinJ11_V3.0320	*L. infantum*	158	442	599
LinJ11_V3.0320	*L. infantum*	53	712	764
LinJ11_V3.0320	*L. infantum*	93	943	1035
LinJ11_V3.0320	*L. infantum*	75	1441	1515
LinJ11_V3.0330	*L. infantum*	73	1	73
LinJ11_V3.0330	*L. infantum*	48	163	210
LinJ11_V3.0330	*L. infantum*	54	414	467
LinJ11_V3.0330	*L. infantum*	83	785	867
LinJ11_V3.0330	*L. infantum*	99	896	994
LinJ11_V3.0340	*L. infantum*	82	67	148
LinJ11_V3.0340	*L. infantum*	101	337	437
LinJ11_V3.0350	*L. infantum*	49	205	253
LinJ11_V3.0360	*L. infantum*	100	60	159
LinJ11_V3.0360	*L. infantum*	44	246	289
LinJ11_V3.0360	*L. infantum*	55	301	355
LinJ11_V3.0370	*L. infantum*	136	1	136
LinJ11_V3.0370	*L. infantum*	404	168	571

LinJ11_V3.0380	*L. infantum*	165	1	165
LinJ11_V3.0380	*L. infantum*	43	427	469
LinJ11_V3.0380	*L. infantum*	67	505	571
LinJ11_V3.0380	*L. infantum*	66	581	646
LinJ11_V3.0380	*L. infantum*	61	835	895
LinJ11_V3.0390	*L. infantum*	97	66	162
LinJ11_V3.0390	*L. infantum*	54	246	299
LinJ11_V3.0390	*L. infantum*	55	319	373
LinJ11_V3.0390	*L. infantum*	106	376	481
LinJ11_V3.0400	*L. infantum*	118	1	118
LinJ11_V3.0400	*L. infantum*	48	158	205
LinJ11_V3.0400	*L. infantum*	76	559	634
LinJ11_V3.0400	*L. infantum*	438	664	1101
LinJ11_V3.0410	*L. infantum*	122	1	122
LinJ11_V3.0410	*L. infantum*	113	249	361
LinJ11_V3.0410	*L. infantum*	42	541	582
LinJ11_V3.0420	*L. infantum*	41	529	569
LinJ11_V3.0420	*L. infantum*	40	688	727
LinJ11_V3.0420	*L. infantum*	40	688	727
LinJ11_V3.0430	*L. infantum*	94	28	121
LinJ11_V3.0430	*L. infantum*	40	292	331
LinJ11_V3.0430	*L. infantum*	55	398	452
LinJ11_V3.0430	*L. infantum*	45	599	643
LinJ11_V3.0440	*L. infantum*	399	182	580
LinJ11_V3.0450	*L. infantum*	40	150	189
LinJ11_V3.0450	*L. infantum*	51	235	285
LinJ11_V3.0450	*L. infantum*	49	324	372
LinJ11_V3.0450	*L. infantum*	51	436	486
LinJ11_V3.0450	*L. infantum*	70	559	628
LinJ11_V3.0450	*L. infantum*	40	667	706
LinJ11_V3.0460	*L. infantum*	47	1	47
LinJ11_V3.0460	*L. infantum*	57	76	132
LinJ11_V3.0460	*L. infantum*	44	165	208
LinJ11_V3.0460	*L. infantum*	46	312	357
LinJ11_V3.0470	*L. infantum*	53	1	53
LinJ11_V3.0470	*L. infantum*	91	85	175
LinJ11_V3.0470	*L. infantum*	49	292	340
LinJ11_V3.0490	*L. infantum*	56	59	114
LinJ11_V3.0510	*L. infantum*	112	1	112
LinJ11_V3.0510	*L. infantum*	59	254	312
LinJ11_V3.0510	*L. infantum*	75	718	792
LinJ11_V3.0510	*L. infantum*	41	875	915
LinJ11_V3.0510	*L. infantum*	163	920	1082
LinJ11_V3.0520	*L. infantum*	64	307	370
LinJ11_V3.0530	*L. infantum*	48	111	158
LinJ11_V3.0530	*L. infantum*	70	324	393
LinJ11_V3.0530	*L. infantum*	70	324	393

LinJ11_V3.0540	*L. infantum*	96	31	126
LinJ11_V3.0540	*L. infantum*	48	271	318
LinJ11_V3.0540	*L. infantum*	48	271	318
LinJ11_V3.0560	*L. infantum*	110	58	167
LinJ11_V3.0580	*L. infantum*	56	1	56
LinJ11_V3.0580	*L. infantum*	87	224	310
LinJ11_V3.0590	*L. infantum*	40	1021	1060
LinJ11_V3.0610	*L. infantum*	222	61	282
LinJ11_V3.0610	*L. infantum*	211	388	598
LinJ11_V3.0650	*L. infantum*	80	13	92
LinJ11_V3.0650	*L. infantum*	125	239	363
LinJ11_V3.0650	*L. infantum*	56	412	467
LinJ11_V3.0650	*L. infantum*	61	502	562
LinJ11_V3.0650	*L. infantum*	54	598	651
LinJ11_V3.0650	*L. infantum*	109	751	859
LinJ11_V3.0650	*L. infantum*	45	898	942
LinJ11_V3.0650	*L. infantum*	54	1112	1165
LinJ11_V3.0650	*L. infantum*	310	1208	1517
LinJ11_V3.0650	*L. infantum*	40	1551	1590
LinJ11_V3.0660	*L. infantum*	57	308	364
LinJ11_V3.0670	*L. infantum*	65	313	377
LinJ11_V3.0670	*L. infantum*	45	655	699
LinJ11_V3.0680	*L. infantum*	53	570	622
LinJ11_V3.0690	*L. infantum*	66	520	585
LinJ11_V3.0710	*L. infantum*	89	43	131
LinJ11_V3.0710	*L. infantum*	55	217	271
LinJ11_V3.0710	*L. infantum*	55	217	271
LinJ11_V3.0720	*L. infantum*	75	1	75
LinJ11_V3.0720	*L. infantum*	190	109	298
LinJ11_V3.0720	*L. infantum*	43	339	381
LinJ11_V3.0720	*L. infantum*	133	424	556
LinJ11_V3.0730	*L. infantum*	72	251	322
LinJ11_V3.0730	*L. infantum*	127	333	459
LinJ11_V3.0730	*L. infantum*	156	498	653
LinJ11_V3.0740	*L. infantum*	137	1	137
LinJ11_V3.0740	*L. infantum*	105	196	300
LinJ11_V3.0740	*L. infantum*	40	342	381
LinJ11_V3.0740	*L. infantum*	85	403	487
LinJ11_V3.0740	*L. infantum*	92	599	690
LinJ11_V3.0740	*L. infantum*	171	740	910
LinJ11_V3.0740	*L. infantum*	100	928	1027
LinJ11_V3.0740	*L. infantum*	53	1063	1115
LinJ11_V3.0750	*L. infantum*	59	1	59
LinJ11_V3.0750	*L. infantum*	74	253	326
LinJ11_V3.0750	*L. infantum*	87	358	444
LinJ11_V3.0760	*L. infantum*	92	114	205
LinJ11_V3.0770	*L. infantum*	96	69	164

LinJ11_V3.0780	*L. infantum*	103	191	293
LinJ11_V3.0810	*L. infantum*	85	75	159
LinJ11_V3.0810	*L. infantum*	54	477	530
LinJ11_V3.0810	*L. infantum*	54	477	530
LinJ11_V3.0820	*L. infantum*	267	71	337
LinJ11_V3.0830	*L. infantum*	73	1	73
LinJ11_V3.0830	*L. infantum*	199	131	329
LinJ11_V3.0830	*L. infantum*	102	348	449
LinJ11_V3.0830	*L. infantum*	628	777	1404
LinJ11_V3.0850	*L. infantum*	50	111	160
LinJ11_V3.0860	*L. infantum*	63	61	123
LinJ11_V3.0860	*L. infantum*	62	318	379
LinJ11_V3.0860	*L. infantum*	97	643	739
LinJ11_V3.0860	*L. infantum*	58	990	1047
LinJ11_V3.0860	*L. infantum*	140	1117	1256
LinJ11_V3.0860	*L. infantum*	140	1535	1674
LinJ11_V3.0870	*L. infantum*	243	23	265
LinJ11_V3.0870	*L. infantum*	65	296	360
LinJ11_V3.0870	*L. infantum*	606	442	1047
LinJ11_V3.0880	*L. infantum*	47	69	115
LinJ11_V3.0890	*L. infantum*	95	1	95
LinJ11_V3.0890	*L. infantum*	55	452	506
LinJ11_V3.0890	*L. infantum*	41	616	656
LinJ11_V3.0890	*L. infantum*	40	971	1010
LinJ11_V3.0900	*L. infantum*	103	88	190
LinJ11_V3.0910	*L. infantum*	345	1	345
LinJ11_V3.0910	*L. infantum*	63	764	826
LinJ11_V3.0910	*L. infantum*	66	915	980
LinJ11_V3.0910	*L. infantum*	53	1017	1069
LinJ11_V3.0910	*L. infantum*	53	1113	1165
LinJ11_V3.0910	*L. infantum*	153	1301	1453
LinJ11_V3.0920	*L. infantum*	126	48	173
LinJ11_V3.0920	*L. infantum*	58	301	358
LinJ11_V3.0920	*L. infantum*	169	452	620
LinJ11_V3.0930	*L. infantum*	181	83	263
LinJ11_V3.0930	*L. infantum*	106	272	377
LinJ11_V3.0940	*L. infantum*	77	65	141
LinJ11_V3.0940	*L. infantum*	76	182	257
LinJ11_V3.0940	*L. infantum*	618	264	881
LinJ11_V3.0940	*L. infantum*	271	891	1161
LinJ11_V3.0940	*L. infantum*	117	1281	1397
LinJ11_V3.0950	*L. infantum*	48	1	48
LinJ11_V3.0950	*L. infantum*	54	94	147
LinJ11_V3.0950	*L. infantum*	67	179	245
LinJ11_V3.0950	*L. infantum*	150	264	413
LinJ11_V3.0980	*L. infantum*	61	43	103
LinJ11_V3.0980	*L. infantum*	139	124	262

LinJ11_V3.0980	*L. infantum*	86	355	440
LinJ11_V3.0990	*L. infantum*	51	241	291
LinJ11_V3.0990	*L. infantum*	81	681	761
LinJ11_V3.0990	*L. infantum*	81	681	761
LinJ11_V3.1000	*L. infantum*	53	304	356
LinJ11_V3.1000	*L. infantum*	47	380	426
LinJ11_V3.1000	*L. infantum*	47	380	426
LinJ11_V3.1010	*L. infantum*	46	129	174
LinJ11_V3.1010	*L. infantum*	67	228	294
LinJ11_V3.1010	*L. infantum*	87	518	604
LinJ11_V3.1020	*L. infantum*	55	1	55
LinJ11_V3.1020	*L. infantum*	415	75	489
LinJ11_V3.1020	*L. infantum*	160	493	652
LinJ11_V3.1020	*L. infantum*	157	681	837
LinJ11_V3.1020	*L. infantum*	50	1112	1161
LinJ11_V3.1020	*L. infantum*	188	1354	1541
LinJ11_V3.1020	*L. infantum*	163	1571	1733
LinJ11_V3.1040	*L. infantum*	160	1	160
LinJ11_V3.1040	*L. infantum*	62	240	301
LinJ11_V3.1040	*L. infantum*	165	315	479
LinJ11_V3.1040	*L. infantum*	263	482	744
LinJ11_V3.1070	*L. infantum*	505	1	505
LinJ11_V3.1080	*L. infantum*	76	1	76
LinJ11_V3.1080	*L. infantum*	75	119	193
LinJ11_V3.1080	*L. infantum*	93	201	293
LinJ11_V3.1090	*L. infantum*	112	1	112
LinJ11_V3.1110	*L. infantum*	81	67	147
LinJ11_V3.1120	*L. infantum*	42	104	145
LinJ11_V3.1120	*L. infantum*	85	193	277
LinJ11_V3.1120	*L. infantum*	85	193	277
LinJ11_V3.1130	*L. infantum*	81	67	147
LinJ11_V3.1140	*L. infantum*	54	401	454
LinJ11_V3.1140	*L. infantum*	42	456	497
LinJ11_V3.1140	*L. infantum*	42	456	497
LinJ11_V3.1160	*L. infantum*	318	1	318
LinJ11_V3.1170	*L. infantum*	41	1	41
LinJ11_V3.1170	*L. infantum*	71	371	441
LinJ11_V3.1170	*L. infantum*	71	371	441
LinJ11_V3.1190	*L. infantum*	48	356	403
LinJ11_V3.1190	*L. infantum*	76	551	626
LinJ11_V3.1190	*L. infantum*	50	643	692
LinJ11_V3.1210	*L. infantum*	40	921	960
LinJ11_V3.1210	*L. infantum*	46	1399	1444
LinJ11_V3.1210	*L. infantum*	90	1687	1776
LinJ11_V3.1230	*L. infantum*	100	1	100
LinJ11_V3.1240	*L. infantum*	140	1	140
LinJ11_V3.1240	*L. infantum*	41	1041	1081

LinJ11_V3.1240	*L. infantum*	43	1520	1562
LinJ11_V3.1240	*L. infantum*	49	1849	1897
LinJ11_V3.1280	*L. infantum*	183	1679	1861
LinJ11_V3.1290	*L. infantum*	112	1	112
LinJ11_V3.1320	*L. infantum*	103	70	172
LinJ11_V3.1320	*L. infantum*	110	202	311
LinJ11_V3.1320	*L. infantum*	47	366	412
LinJ11_V3.1320	*L. infantum*	84	462	545
LinJ11_V3.1320	*L. infantum*	116	621	736
LinJ11_V3.1320	*L. infantum*	65	859	923
LinJ11_V3.1320	*L. infantum*	124	1377	1500
LinJ11_V3.1320	*L. infantum*	89	1535	1623
LinJ11_V3.1330	*L. infantum*	83	325	407
LinJ11_V3.1340	*L. infantum*	42	1	42
LinJ11_V3.1340	*L. infantum*	99	79	177
LinJ11_V3.1340	*L. infantum*	99	79	177
LinJ11_V3.1350	*L. infantum*	71	1	71
LinJ11_V3.1370	*L. infantum*	55	122	176
LinJ11_V3.1370	*L. infantum*	41	215	255
LinJ11_V3.1370	*L. infantum*	51	300	350
LinJ12_V3.0050	*L. infantum*	107	127	233
LinJ12_V3.0050	*L. infantum*	251	340	590
LinJ12_V3.0060	*L. infantum*	232	351	582
LinJ12_V3.0060	*L. infantum*	371	991	1361
LinJ12_V3.0060	*L. infantum*	47	1364	1410
LinJ12_V3.0070	*L. infantum*	140	1	140
LinJ12_V3.0070	*L. infantum*	744	160	903
LinJ12_V3.0100	*L. infantum*	98	31	128
LinJ12_V3.0140	*L. infantum*	63	340	402
LinJ12_V3.0140	*L. infantum*	49	473	521
LinJ12_V3.0140	*L. infantum*	59	658	716
LinJ12_V3.0150	*L. infantum*	139	1	139
LinJ12_V3.0150	*L. infantum*	178	153	330
LinJ12_V3.0160	*L. infantum*	96	120	215
LinJ12_V3.0160	*L. infantum*	66	363	428
LinJ12_V3.0160	*L. infantum*	66	441	506
LinJ12_V3.0170	*L. infantum*	53	1	53
LinJ12_V3.0170	*L. infantum*	99	99	197
LinJ12_V3.0170	*L. infantum*	44	234	277
LinJ12_V3.0170	*L. infantum*	83	357	439
LinJ12_V3.0170	*L. infantum*	88	602	689
LinJ12_V3.0170	*L. infantum*	122	865	986
LinJ12_V3.0170	*L. infantum*	91	1012	1102
LinJ12_V3.0170	*L. infantum*	57	1158	1214
LinJ12_V3.0170	*L. infantum*	54	1312	1365
LinJ12_V3.0170	*L. infantum*	63	1488	1550
LinJ12_V3.0170	*L. infantum*	76	1570	1645

LinJ12_V3.0180	*L. infantum*	194	1	194
LinJ12_V3.0180	*L. infantum*	238	196	433
LinJ12_V3.0180	*L. infantum*	614	481	1094
LinJ12_V3.0190	*L. infantum*	50	1	50
LinJ12_V3.0210	*L. infantum*	92	1	92
LinJ12_V3.0210	*L. infantum*	57	139	195
LinJ12_V3.0210	*L. infantum*	89	252	340
LinJ12_V3.0210	*L. infantum*	236	369	604
LinJ12_V3.0210	*L. infantum*	62	653	714
LinJ12_V3.0210	*L. infantum*	74	895	968
LinJ12_V3.0210	*L. infantum*	77	1030	1106
LinJ12_V3.0210	*L. infantum*	49	1163	1211
LinJ12_V3.0210	*L. infantum*	43	1239	1281
LinJ12_V3.0210	*L. infantum*	85	1461	1545
LinJ12_V3.0220	*L. infantum*	44	205	248
LinJ12_V3.0220	*L. infantum*	126	573	698
LinJ12_V3.0240	*L. infantum*	55	1	55
LinJ12_V3.0240	*L. infantum*	828	74	901
LinJ12_V3.0240	*L. infantum*	207	905	1111
LinJ12_V3.0240	*L. infantum*	58	1143	1200
LinJ12_V3.0240	*L. infantum*	143	1406	1548
LinJ12_V3.0250	*L. infantum*	122	544	665
LinJ12_V3.0250	*L. infantum*	476	691	1166
LinJ12_V3.0250	*L. infantum*	59	1236	1294
LinJ12_V3.0250	*L. infantum*	103	1335	1437
LinJ12_V3.0250	*L. infantum*	62	1579	1640
LinJ12_V3.0260	*L. infantum*	140	91	230
LinJ12_V3.0260	*L. infantum*	46	359	404
LinJ12_V3.0260	*L. infantum*	44	464	507
LinJ12_V3.0260	*L. infantum*	60	583	642
LinJ12_V3.0270	*L. infantum*	190	100	289
LinJ12_V3.0270	*L. infantum*	56	302	357
LinJ12_V3.0270	*L. infantum*	51	451	501
LinJ12_V3.0270	*L. infantum*	95	536	630
LinJ12_V3.0270	*L. infantum*	48	726	773
LinJ12_V3.0270	*L. infantum*	475	814	1288
LinJ12_V3.0270	*L. infantum*	60	1328	1387
LinJ12_V3.0270	*L. infantum*	91	1404	1494
LinJ12_V3.0270	*L. infantum*	78	1523	1600
LinJ12_V3.0270	*L. infantum*	93	1636	1728
LinJ12_V3.0270	*L. infantum*	41	1831	1871
LinJ12_V3.0270	*L. infantum*	99	2119	2217
LinJ12_V3.0270	*L. infantum*	42	2303	2344
LinJ12_V3.0270	*L. infantum*	201	2423	2623
LinJ12_V3.0270	*L. infantum*	215	2699	2913
LinJ12_V3.0270	*L. infantum*	72	2924	2995
LinJ12_V3.0270	*L. infantum*	174	3057	3230

LinJ12_V3.0280	*L. infantum*	277	1	277
LinJ12_V3.0280	*L. infantum*	125	311	435
LinJ12_V3.0280	*L. infantum*	73	651	723
LinJ12_V3.0280	*L. infantum*	52	866	917
LinJ12_V3.0280	*L. infantum*	47	952	998
LinJ12_V3.0280	*L. infantum*	145	1021	1165
LinJ12_V3.0280	*L. infantum*	53	1191	1243
LinJ12_V3.0290	*L. infantum*	451	1	451
LinJ12_V3.0290	*L. infantum*	171	477	647
LinJ12_V3.0290	*L. infantum*	594	671	1264
LinJ12_V3.0290	*L. infantum*	110	1302	1411
LinJ12_V3.0290	*L. infantum*	58	1442	1499
LinJ12_V3.0290	*L. infantum*	50	1569	1618
LinJ12_V3.0290	*L. infantum*	289	1654	1942
LinJ12_V3.0300	*L. infantum*	343	1	343
LinJ12_V3.0300	*L. infantum*	58	504	561
LinJ12_V3.0310	*L. infantum*	99	199	297
LinJ12_V3.0320	*L. infantum*	132	155	286
LinJ12_V3.0330	*L. infantum*	114	1	114
LinJ12_V3.0330	*L. infantum*	125	177	301
LinJ12_V3.0330	*L. infantum*	99	326	424
LinJ12_V3.0330	*L. infantum*	552	430	981
LinJ12_V3.0330	*L. infantum*	69	1014	1082
LinJ12_V3.0340	*L. infantum*	49	330	378
LinJ12_V3.0360	*L. infantum*	333	1	333
LinJ12_V3.0390	*L. infantum*	57	38	94
LinJ12_V3.0390	*L. infantum*	213	206	418
LinJ12_V3.0390	*L. infantum*	104	507	610
LinJ12_V3.0400	*L. infantum*	100	189	288
LinJ12_V3.0400	*L. infantum*	161	308	468
LinJ12_V3.0400	*L. infantum*	161	491	651
LinJ12_V3.0410	*L. infantum*	366	1	366
LinJ12_V3.0410	*L. infantum*	46	375	420
LinJ12_V3.0410	*L. infantum*	113	447	559
LinJ12_V3.0410	*L. infantum*	426	664	1089
LinJ12_V3.0420	*L. infantum*	525	1	525
LinJ12_V3.0420	*L. infantum*	60	555	614
LinJ12_V3.0420	*L. infantum*	167	618	784
LinJ12_V3.0430	*L. infantum*	414	1	414
LinJ12_V3.0430	*L. infantum*	158	439	596
LinJ12_V3.0430	*L. infantum*	65	670	734
LinJ12_V3.0440	*L. infantum*	325	1	325
LinJ12_V3.0440	*L. infantum*	163	447	609
LinJ12_V3.0450	*L. infantum*	40	1	40
LinJ12_V3.0450	*L. infantum*	143	62	204
LinJ12_V3.0450	*L. infantum*	61	240	300
LinJ12_V3.0460	*L. infantum*	119	42	160

LinJ12_V3.0460	*L. infantum*	68	181	248
LinJ12_V3.0460	*L. infantum*	206	285	490
LinJ12_V3.0460	*L. infantum*	175	538	712
LinJ12_V3.0460	*L. infantum*	60	755	814
LinJ12_V3.0460	*L. infantum*	233	816	1048
LinJ12_V3.0460	*L. infantum*	59	1062	1120
LinJ12_V3.0460	*L. infantum*	63	1135	1197
LinJ12_V3.0460	*L. infantum*	51	1228	1278
LinJ12_V3.0460	*L. infantum*	65	1322	1386
LinJ12_V3.0460	*L. infantum*	67	1407	1473
LinJ12_V3.0470	*L. infantum*	713	62	774
LinJ12_V3.0470	*L. infantum*	54	777	830
LinJ12_V3.0470	*L. infantum*	77	893	969
LinJ12_V3.0500	*L. infantum*	76	1	76
LinJ12_V3.0510	*L. infantum*	94	41	134
LinJ12_V3.0510	*L. infantum*	241	166	406
LinJ12_V3.0510	*L. infantum*	86	414	499
LinJ12_V3.0520	*L. infantum*	46	1	46
LinJ12_V3.0520	*L. infantum*	122	219	340
LinJ12_V3.0520	*L. infantum*	47	496	542
LinJ12_V3.0520	*L. infantum*	423	624	1046
LinJ12_V3.0530	*L. infantum*	161	331	491
LinJ12_V3.0530	*L. infantum*	122	513	634
LinJ12_V3.0530	*L. infantum*	215	651	865
LinJ12_V3.0530	*L. infantum*	187	867	1053
LinJ12_V3.0540	*L. infantum*	289	1	289
LinJ12_V3.0540	*L. infantum*	43	913	955
LinJ12_V3.0540	*L. infantum*	60	1138	1197
LinJ12_V3.0540	*L. infantum*	47	1275	1321
LinJ12_V3.0540	*L. infantum*	142	1351	1492
LinJ12_V3.0540	*L. infantum*	206	1507	1712
LinJ12_V3.0540	*L. infantum*	72	1752	1823
LinJ12_V3.0550	*L. infantum*	54	1	54
LinJ12_V3.0550	*L. infantum*	42	180	221
LinJ12_V3.0550	*L. infantum*	57	254	310
LinJ12_V3.0560	*L. infantum*	178	179	356
LinJ12_V3.0560	*L. infantum*	65	451	515
LinJ12_V3.0560	*L. infantum*	153	792	944
LinJ12_V3.0560	*L. infantum*	53	961	1013
LinJ12_V3.0570	*L. infantum*	183	1	183
LinJ12_V3.0570	*L. infantum*	645	195	839
LinJ12_V3.0570	*L. infantum*	369	865	1233
LinJ12_V3.0570	*L. infantum*	221	1244	1464
LinJ12_V3.0590	*L. infantum*	86	177	262
LinJ12_V3.0590	*L. infantum*	52	309	360
LinJ12_V3.0600	*L. infantum*	617	1	617
LinJ12_V3.0610	*L. infantum*	70	1	70

LinJ12_V3.0620	*L. infantum*	85	241	325
LinJ12_V3.0630	*L. infantum*	42	135	176
LinJ12_V3.0630	*L. infantum*	55	328	382
LinJ12_V3.0640	*L. infantum*	105	60	164
LinJ12_V3.0650	*L. infantum*	55	119	173
LinJ12_V3.0650	*L. infantum*	55	230	284
LinJ12_V3.0650	*L. infantum*	65	402	466
LinJ12_V3.0650	*L. infantum*	173	678	850
LinJ12_V3.0690	*L. infantum*	105	150	254
LinJ12_V3.0690	*L. infantum*	55	283	337
LinJ12_V3.0690	*L. infantum*	55	283	337
LinJ12_V3.0700	*L. infantum*	43	1	43
LinJ12_V3.0700	*L. infantum*	95	128	222
LinJ12_V3.0700	*L. infantum*	60	261	320
LinJ12_V3.0700	*L. infantum*	356	372	727
LinJ12_V3.0700	*L. infantum*	61	751	811
LinJ12_V3.0700	*L. infantum*	50	829	878
LinJ12_V3.0700	*L. infantum*	225	913	1137
LinJ12_V3.0700	*L. infantum*	154	1431	1584
LinJ12_V3.0710	*L. infantum*	293	32	324
LinJ12_V3.0710	*L. infantum*	174	336	509
LinJ12_V3.0710	*L. infantum*	52	557	608
LinJ12_V3.0720	*L. infantum*	299	152	450
LinJ12_V3.0720	*L. infantum*	120	464	583
LinJ12_V3.0720	*L. infantum*	262	718	979
LinJ12_V3.0720	*L. infantum*	108	981	1088
LinJ12_V3.0740	*L. infantum*	76	375	450
LinJ12_V3.0740	*L. infantum*	47	782	828
LinJ12_V3.0740	*L. infantum*	47	782	828
LinJ12_V3.0750	*L. infantum*	181	46	226
LinJ12_V3.0750	*L. infantum*	415	281	695
LinJ12_V3.0750	*L. infantum*	163	780	942
LinJ12_V3.0750	*L. infantum*	41	960	1000
LinJ12_V3.0760	*L. infantum*	113	198	310
LinJ12_V3.0760	*L. infantum*	106	326	431
LinJ12_V3.0760	*L. infantum*	260	688	947
LinJ12_V3.0770	*L. infantum*	58	176	233
LinJ12_V3.0770	*L. infantum*	46	239	284
LinJ12_V3.0770	*L. infantum*	178	523	700
LinJ12_V3.0780	*L. infantum*	366	30	395
LinJ12_V3.0780	*L. infantum*	65	471	535
LinJ12_V3.0780	*L. infantum*	59	655	713
LinJ12_V3.0780	*L. infantum*	52	813	864
LinJ12_V3.0790	*L. infantum*	50	1	50
LinJ12_V3.0790	*L. infantum*	63	155	217
LinJ12_V3.0790	*L. infantum*	49	277	325
LinJ12_V3.0790	*L. infantum*	92	498	589

LinJ12_V3.0790	*L. infantum*	58	1041	1098
LinJ12_V3.0790	*L. infantum*	102	1138	1239
LinJ12_V3.0790	*L. infantum*	52	1275	1326
LinJ12_V3.0790	*L. infantum*	40	1337	1376
LinJ12_V3.0800	*L. infantum*	196	238	433
LinJ12_V3.0820	*L. infantum*	80	1	80
LinJ12_V3.0820	*L. infantum*	70	107	176
LinJ12_V3.0820	*L. infantum*	66	286	351
LinJ12_V3.0820	*L. infantum*	107	413	519
LinJ12_V3.0820	*L. infantum*	83	638	720
LinJ12_V3.0830	*L. infantum*	44	237	280
LinJ12_V3.0830	*L. infantum*	54	667	720
LinJ12_V3.0830	*L. infantum*	177	900	1076
LinJ12_V3.0830	*L. infantum*	47	1257	1303
LinJ12_V3.0840	*L. infantum*	116	68	183
LinJ12_V3.0840	*L. infantum*	43	397	439
LinJ12_V3.0860	*L. infantum*	623	1	623
LinJ12_V3.0870	*L. infantum*	62	152	213
LinJ12_V3.0880	*L. infantum*	63	226	288
LinJ12_V3.0920	*L. infantum*	50	492	541
LinJ12_V3.0930	*L. infantum*	239	103	341
LinJ12_V3.0930	*L. infantum*	49	390	438
LinJ12_v4.0070	*L. infantum*	56	61	116
LinJ12_v4.0070	*L. infantum*	426	150	575
LinJ12_v4.0080	*L. infantum*	78	1	78
LinJ12_v4.0100	*L. infantum*	67	136	202
LinJ12_v4.0110	*L. infantum*	169	79	247
LinJ12_v4.0110	*L. infantum*	468	276	743
LinJ12_v4.0110	*L. infantum*	108	793	900
LinJ12_v4.0120	*L. infantum*	44	57	100
LinJ12_v4.0120	*L. infantum*	243	121	363
LinJ12_v4.0120	*L. infantum*	969	402	1370
LinJ12_v4.0130	*L. infantum*	101	254	354
LinJ12_v4.0130	*L. infantum*	59	473	531
LinJ12_v4.0140	*L. infantum*	44	1	44
LinJ12_v4.0140	*L. infantum*	129	455	583
LinJ12_v4.0140	*L. infantum*	129	455	583
LinJ12_v4.0150	*L. infantum*	307	1	307
LinJ12_v4.0150	*L. infantum*	207	476	682
LinJ12_v4.0150	*L. infantum*	82	707	788
LinJ12_v4.0150	*L. infantum*	71	832	902
LinJ12_v4.0150	*L. infantum*	181	1182	1362
LinJ12_v4.0255	*L. infantum*	50	146	195
LinJ12_v4.0255	*L. infantum*	57	303	359
LinJ12_v4.0255	*L. infantum*	57	303	359
LinJ12_v4.0260	*L. infantum*	228	61	288
LinJ12_v4.0260	*L. infantum*	54	408	461

LinJ12_v4.0260	*L. infantum*	45	650	694
LinJ12_v4.0260	*L. infantum*	48	775	822
LinJ12_v4.0260	*L. infantum*	63	867	929
LinJ12_v4.0260	*L. infantum*	56	1048	1103
LinJ12_v4.0260	*L. infantum*	41	1177	1217
LinJ12_v4.0260	*L. infantum*	75	1242	1316
LinJ12_v4.0260	*L. infantum*	45	1448	1492
LinJ12_v4.0260	*L. infantum*	47	1584	1630
LinJ12_v4.0260	*L. infantum*	44	1744	1787
LinJ12_v4.0260	*L. infantum*	86	1807	1892
LinJ12_v4.0260	*L. infantum*	235	1927	2161
LinJ12_v4.0260	*L. infantum*	57	2193	2249
LinJ12_v4.0260	*L. infantum*	97	2422	2518
LinJ12_v4.0260	*L. infantum*	49	2612	2660
LinJ12_v4.0260	*L. infantum*	58	2764	2821
LinJ12_v4.0260	*L. infantum*	83	2848	2930
LinJ12_v4.0270	*L. infantum*	112	63	174
LinJ12_v4.0270	*L. infantum*	76	232	307
LinJ12_v4.0270	*L. infantum*	64	402	465
LinJ12_v4.0661	*L. infantum*	93	219	311
LinJ12_v4.0661	*L. infantum*	112	328	439
LinJ12_v4.0662	*L. infantum*	41	18	58
LinJ12_v4.0662	*L. infantum*	95	106	200
LinJ12_v4.0662	*L. infantum*	95	106	200
LinJ12_v4.0663	*L. infantum*	47	366	412
LinJ12_v4.0663	*L. infantum*	159	458	616
LinJ12_v4.0664	*L. infantum*	93	219	311
LinJ12_v4.0664	*L. infantum*	112	328	439
LinJ12_v4.0665	*L. infantum*	147	136	282
LinJ12_v4.0665	*L. infantum*	55	362	416
LinJ12_v4.0665	*L. infantum*	55	362	416
LinJ12_v4.0666	*L. infantum*	109	145	253
LinJ12_v4.0666	*L. infantum*	94	287	380
LinJ12_v4.0667	*L. infantum*	213	45	257
LinJ12_v4.0668	*L. infantum*	40	138	177
LinJ12_v4.0668	*L. infantum*	91	304	394
LinJ12_v4.0668	*L. infantum*	163	422	584
LinJ12_v4.0669	*L. infantum*	92	219	310
LinJ12_v4.0669	*L. infantum*	112	328	439
LinJ12_v4.0670	*L. infantum*	41	195	235
LinJ12_v4.0670	*L. infantum*	87	317	403
LinJ12_v4.0671	*L. infantum*	107	315	421
LinJ12_v4.0671	*L. infantum*	42	500	541
LinJ12_v4.0671	*L. infantum*	42	500	541
LinJ13_V3.0010	*L. infantum*	48	76	123
LinJ13_V3.0020	*L. infantum*	40	286	325
LinJ13_V3.0040	*L. infantum*	156	1	156

LinJ13_V3.0060	*L. infantum*	104	14	117
LinJ13_V3.0060	*L. infantum*	97	148	244
LinJ13_V3.0060	*L. infantum*	148	247	394
LinJ13_V3.0060	*L. infantum*	123	397	519
LinJ13_V3.0070	*L. infantum*	223	68	290
LinJ13_V3.0080	*L. infantum*	42	1	42
LinJ13_V3.0080	*L. infantum*	62	1369	1430
LinJ13_V3.0080	*L. infantum*	62	1369	1430
LinJ13_V3.0100	*L. infantum*	50	20	69
LinJ13_V3.0100	*L. infantum*	237	200	436
LinJ13_V3.0110	*L. infantum*	43	33	75
LinJ13_V3.0110	*L. infantum*	43	310	352
LinJ13_V3.0110	*L. infantum*	43	310	352
LinJ13_V3.0120	*L. infantum*	57	1	57
LinJ13_V3.0130	*L. infantum*	67	1	67
LinJ13_V3.0130	*L. infantum*	55	240	294
LinJ13_V3.0130	*L. infantum*	77	390	466
LinJ13_V3.0130	*L. infantum*	135	515	649
LinJ13_V3.0140	*L. infantum*	44	61	104
LinJ13_V3.0150	*L. infantum*	146	69	214
LinJ13_V3.0170	*L. infantum*	46	135	180
LinJ13_V3.0170	*L. infantum*	82	495	576
LinJ13_V3.0180	*L. infantum*	63	39	101
LinJ13_V3.0180	*L. infantum*	60	178	237
LinJ13_V3.0180	*L. infantum*	60	178	237
LinJ13_V3.0190	*L. infantum*	75	21	95
LinJ13_V3.0190	*L. infantum*	62	128	189
LinJ13_V3.0200	*L. infantum*	107	1	107
LinJ13_V3.0200	*L. infantum*	43	188	230
LinJ13_V3.0200	*L. infantum*	56	627	682
LinJ13_V3.0210	*L. infantum*	192	392	583
LinJ13_V3.0230	*L. infantum*	327	93	419
LinJ13_V3.0240	*L. infantum*	58	1	58
LinJ13_V3.0240	*L. infantum*	57	64	120
LinJ13_V3.0240	*L. infantum*	76	153	228
LinJ13_V3.0250	*L. infantum*	63	65	127
LinJ13_V3.0260	*L. infantum*	99	162	260
LinJ13_V3.0290	*L. infantum*	68	290	357
LinJ13_V3.0290	*L. infantum*	130	366	495
LinJ13_V3.0290	*L. infantum*	61	538	598
LinJ13_V3.0310	*L. infantum*	42	37	78
LinJ13_V3.0310	*L. infantum*	75	661	735
LinJ13_V3.0310	*L. infantum*	61	867	927
LinJ13_V3.0320	*L. infantum*	245	19	263
LinJ13_V3.0320	*L. infantum*	100	1397	1496
LinJ13_V3.0320	*L. infantum*	181	1791	1971
LinJ13_V3.0340	*L. infantum*	70	118	187

LinJ13_V3.0340	*L. infantum*	179	287	465
LinJ13_V3.0340	*L. infantum*	62	520	581
LinJ13_V3.0340	*L. infantum*	67	653	719
LinJ13_V3.0340	*L. infantum*	94	956	1049
LinJ13_V3.0360	*L. infantum*	100	393	492
LinJ13_V3.0370	*L. infantum*	113	1	113
LinJ13_V3.0370	*L. infantum*	145	139	283
LinJ13_V3.0370	*L. infantum*	128	299	426
LinJ13_V3.0380	*L. infantum*	67	1	67
LinJ13_V3.0380	*L. infantum*	54	267	320
LinJ13_V3.0380	*L. infantum*	42	435	476
LinJ13_V3.0390	*L. infantum*	138	1	138
LinJ13_V3.0390	*L. infantum*	72	158	229
LinJ13_V3.0410	*L. infantum*	186	1	186
LinJ13_V3.0420	*L. infantum*	253	1	253
LinJ13_V3.0420	*L. infantum*	110	255	364
LinJ13_V3.0420	*L. infantum*	48	381	428
LinJ13_V3.0420	*L. infantum*	214	521	734
LinJ13_V3.0420	*L. infantum*	135	777	911
LinJ13_V3.0420	*L. infantum*	285	936	1220
LinJ13_V3.0440	*L. infantum*	60	87	146
LinJ13_V3.0440	*L. infantum*	157	202	358
LinJ13_V3.0440	*L. infantum*	91	528	618
LinJ13_V3.0450	*L. infantum*	51	148	198
LinJ13_V3.0470	*L. infantum*	212	1	212
LinJ13_V3.0470	*L. infantum*	96	271	366
LinJ13_V3.0470	*L. infantum*	75	617	691
LinJ13_V3.0470	*L. infantum*	158	819	976
LinJ13_V3.0470	*L. infantum*	73	987	1059
LinJ13_V3.0470	*L. infantum*	92	1074	1165
LinJ13_V3.0470	*L. infantum*	42	1200	1241
LinJ13_V3.0470	*L. infantum*	54	1340	1393
LinJ13_V3.0470	*L. infantum*	42	1433	1474
LinJ13_V3.0470	*L. infantum*	62	1517	1578
LinJ13_V3.0470	*L. infantum*	49	1601	1649
LinJ13_V3.0490	*L. infantum*	88	1	88
LinJ13_V3.0490	*L. infantum*	45	111	155
LinJ13_V3.0490	*L. infantum*	45	111	155
LinJ13_V3.0500	*L. infantum*	69	123	191
LinJ13_V3.0510	*L. infantum*	349	41	389
LinJ13_V3.0530	*L. infantum*	41	776	816
LinJ13_V3.0530	*L. infantum*	153	1119	1271
LinJ13_V3.0540	*L. infantum*	155	98	252
LinJ13_V3.0540	*L. infantum*	56	413	468
LinJ13_V3.0540	*L. infantum*	84	524	607
LinJ13_V3.0560	*L. infantum*	196	253	448
LinJ13_V3.0570	*L. infantum*	162	1	162

LinJ13_V3.0570	*L. infantum*	71	504	574
LinJ13_V3.0580	*L. infantum*	149	43	191
LinJ13_V3.0580	*L. infantum*	558	213	770
LinJ13_V3.0580	*L. infantum*	42	820	861
LinJ13_V3.0590	*L. infantum*	49	1	49
LinJ13_V3.0590	*L. infantum*	463	99	561
LinJ13_V3.0590	*L. infantum*	68	623	690
LinJ13_V3.0590	*L. infantum*	134	780	913
LinJ13_V3.0590	*L. infantum*	124	991	1114
LinJ13_V3.0590	*L. infantum*	43	1209	1251
LinJ13_V3.0600	*L. infantum*	300	132	431
LinJ13_V3.0620	*L. infantum*	368	73	440
LinJ13_V3.0620	*L. infantum*	65	465	529
LinJ13_V3.0620	*L. infantum*	248	600	847
LinJ13_V3.0630	*L. infantum*	116	1	116
LinJ13_V3.0630	*L. infantum*	48	730	777
LinJ13_V3.0630	*L. infantum*	48	730	777
LinJ13_V3.0640	*L. infantum*	138	1	138
LinJ13_V3.0650	*L. infantum*	221	149	369
LinJ13_V3.0650	*L. infantum*	295	517	811
LinJ13_V3.0650	*L. infantum*	291	1087	1377
LinJ13_V3.0650	*L. infantum*	78	1453	1530
LinJ13_V3.0650	*L. infantum*	69	1595	1663
LinJ13_V3.0650	*L. infantum*	50	1686	1735
LinJ13_V3.0650	*L. infantum*	108	1788	1895
LinJ13_V3.0670	*L. infantum*	97	312	408
LinJ13_V3.0680	*L. infantum*	107	71	177
LinJ13_V3.0680	*L. infantum*	86	181	266
LinJ13_V3.0680	*L. infantum*	68	307	374
LinJ13_V3.0680	*L. infantum*	49	498	546
LinJ13_V3.0680	*L. infantum*	54	581	634
LinJ13_V3.0690	*L. infantum*	40	1	40
LinJ13_V3.0690	*L. infantum*	128	96	223
LinJ13_V3.0690	*L. infantum*	128	96	223
LinJ13_V3.0700	*L. infantum*	185	1	185
LinJ13_V3.0700	*L. infantum*	393	214	606
LinJ13_V3.0700	*L. infantum*	183	628	810
LinJ13_V3.0700	*L. infantum*	118	860	977
LinJ13_V3.0700	*L. infantum*	131	1000	1130
LinJ13_V3.0700	*L. infantum*	98	1232	1329
LinJ13_V3.0700	*L. infantum*	138	1346	1483
LinJ13_V3.0700	*L. infantum*	240	1493	1732
LinJ13_V3.0700	*L. infantum*	114	1796	1909
LinJ13_V3.0700	*L. infantum*	77	1955	2031
LinJ13_V3.0700	*L. infantum*	52	2318	2369
LinJ13_V3.0700	*L. infantum*	148	2405	2552
LinJ13_V3.0720	*L. infantum*	124	1	124

LinJ13_V3.0720	*L. infantum*	167	136	302
LinJ13_V3.0720	*L. infantum*	113	433	545
LinJ13_V3.0720	*L. infantum*	192	582	773
LinJ13_V3.0720	*L. infantum*	54	1183	1236
LinJ13_V3.0730	*L. infantum*	85	1	85
LinJ13_V3.0730	*L. infantum*	104	125	228
LinJ13_V3.0730	*L. infantum*	56	268	323
LinJ13_V3.0730	*L. infantum*	176	352	527
LinJ13_V3.0730	*L. infantum*	59	554	612
LinJ13_V3.0730	*L. infantum*	232	670	901
LinJ13_V3.0730	*L. infantum*	263	903	1165
LinJ13_V3.0740	*L. infantum*	502	1	502
LinJ13_V3.0740	*L. infantum*	112	587	698
LinJ13_V3.0740	*L. infantum*	123	732	854
LinJ13_V3.0740	*L. infantum*	162	882	1043
LinJ13_V3.0750	*L. infantum*	98	15	112
LinJ13_V3.0750	*L. infantum*	193	138	330
LinJ13_V3.0750	*L. infantum*	50	343	392
LinJ13_V3.0770	*L. infantum*	278	79	356
LinJ13_V3.0770	*L. infantum*	242	453	694
LinJ13_V3.0780	*L. infantum*	58	1	58
LinJ13_V3.0780	*L. infantum*	75	105	179
LinJ13_V3.0780	*L. infantum*	93	234	326
LinJ13_V3.0790	*L. infantum*	46	26	71
LinJ13_V3.0790	*L. infantum*	150	86	235
LinJ13_V3.0790	*L. infantum*	143	307	449
LinJ13_V3.0790	*L. infantum*	127	490	616
LinJ13_V3.0800	*L. infantum*	50	49	98
LinJ13_V3.0800	*L. infantum*	254	178	431
LinJ13_V3.0800	*L. infantum*	69	442	510
LinJ13_V3.0800	*L. infantum*	40	691	730
LinJ13_V3.0800	*L. infantum*	75	747	821
LinJ13_V3.0810	*L. infantum*	166	1	166
LinJ13_V3.0810	*L. infantum*	44	182	225
LinJ13_V3.0810	*L. infantum*	40	320	359
LinJ13_V3.0810	*L. infantum*	97	420	516
LinJ13_V3.0810	*L. infantum*	108	542	649
LinJ13_V3.0810	*L. infantum*	63	801	863
LinJ13_V3.0810	*L. infantum*	63	910	972
LinJ13_V3.0810	*L. infantum*	100	1042	1141
LinJ13_V3.0810	*L. infantum*	193	1263	1455
LinJ13_V3.0810	*L. infantum*	63	1460	1522
LinJ13_V3.0830	*L. infantum*	40	21	60
LinJ13_V3.0830	*L. infantum*	132	371	502
LinJ13_V3.0840	*L. infantum*	73	229	301
LinJ13_V3.0860	*L. infantum*	134	1	134
LinJ13_V3.0860	*L. infantum*	47	208	254

LinJ13_V3.0860	*L. infantum*	159	345	503
LinJ13_V3.0870	*L. infantum*	51	121	171
LinJ13_V3.0870	*L. infantum*	57	260	316
LinJ13_V3.0870	*L. infantum*	45	330	374
LinJ13_V3.0870	*L. infantum*	71	505	575
LinJ13_V3.0870	*L. infantum*	61	817	877
LinJ13_V3.0870	*L. infantum*	148	942	1089
LinJ13_V3.0870	*L. infantum*	70	1587	1656
LinJ13_V3.0880	*L. infantum*	490	1	490
LinJ13_V3.0890	*L. infantum*	73	1	73
LinJ13_V3.0890	*L. infantum*	66	134	199
LinJ13_V3.0890	*L. infantum*	152	221	372
LinJ13_V3.0900	*L. infantum*	40	190	229
LinJ13_V3.0910	*L. infantum*	74	117	190
LinJ13_V3.0910	*L. infantum*	52	222	273
LinJ13_V3.0910	*L. infantum*	51	465	515
LinJ13_V3.0910	*L. infantum*	44	600	643
LinJ13_V3.0910	*L. infantum*	44	1052	1095
LinJ13_V3.0910	*L. infantum*	142	1151	1292
LinJ13_V3.0920	*L. infantum*	80	1	80
LinJ13_V3.0920	*L. infantum*	41	191	231
LinJ13_V3.0920	*L. infantum*	41	191	231
LinJ13_V3.0930	*L. infantum*	41	218	258
LinJ13_V3.0940	*L. infantum*	56	558	613
LinJ13_V3.0940	*L. infantum*	74	683	756
LinJ13_V3.0940	*L. infantum*	93	789	881
LinJ13_V3.0940	*L. infantum*	64	1129	1192
LinJ13_V3.0970	*L. infantum*	42	14	55
LinJ13_V3.0970	*L. infantum*	204	150	353
LinJ13_V3.0970	*L. infantum*	47	396	442
LinJ13_V3.0970	*L. infantum*	84	665	748
LinJ13_V3.0970	*L. infantum*	83	913	995
LinJ13_V3.0970	*L. infantum*	118	1142	1259
LinJ13_V3.0970	*L. infantum*	83	1618	1700
LinJ13_V3.0970	*L. infantum*	104	1921	2024
LinJ13_V3.0970	*L. infantum*	205	2037	2241
LinJ13_V3.0980	*L. infantum*	105	87	191
LinJ13_V3.0980	*L. infantum*	95	632	726
LinJ13_V3.0980	*L. infantum*	117	817	933
LinJ13_V3.0980	*L. infantum*	60	1219	1278
LinJ13_V3.0980	*L. infantum*	40	1331	1370
LinJ13_V3.0990	*L. infantum*	45	1	45
LinJ13_V3.1000	*L. infantum*	49	102	150
LinJ13_V3.1010	*L. infantum*	452	43	494
LinJ13_V3.1030	*L. infantum*	107	226	332
LinJ13_V3.1060	*L. infantum*	134	1	134
LinJ13_V3.1070	*L. infantum*	137	1	137

LinJ13_V3.1070	*L. infantum*	98	180	277
LinJ13_V3.1070	*L. infantum*	74	393	466
LinJ13_V3.1090	*L. infantum*	135	1	135
LinJ13_V3.1100	*L. infantum*	74	1	74
LinJ13_V3.1110	*L. infantum*	99	235	333
LinJ13_V3.1130	*L. infantum*	61	161	221
LinJ13_V3.1140	*L. infantum*	70	167	236
LinJ13_V3.1140	*L. infantum*	43	306	348
LinJ13_V3.1140	*L. infantum*	44	500	543
LinJ13_V3.1150	*L. infantum*	81	70	150
LinJ13_V3.1160	*L. infantum*	65	128	192
LinJ13_V3.1170	*L. infantum*	86	62	147
LinJ13_V3.1170	*L. infantum*	55	260	314
LinJ13_V3.1170	*L. infantum*	115	489	603
LinJ13_V3.1170	*L. infantum*	64	663	726
LinJ13_V3.1170	*L. infantum*	89	760	848
LinJ13_V3.1190	*L. infantum*	443	1	443
LinJ13_V3.1190	*L. infantum*	83	709	791
LinJ13_V3.1210	*L. infantum*	109	312	420
LinJ13_V3.1220	*L. infantum*	59	310	368
LinJ13_V3.1220	*L. infantum*	81	385	465
LinJ13_V3.1230	*L. infantum*	48	93	140
LinJ13_V3.1230	*L. infantum*	65	363	427
LinJ13_V3.1240	*L. infantum*	161	1	161
LinJ13_V3.1240	*L. infantum*	47	199	245
LinJ13_V3.1240	*L. infantum*	42	335	376
LinJ13_V3.1240	*L. infantum*	155	433	587
LinJ13_V3.1240	*L. infantum*	135	633	767
LinJ13_V3.1240	*L. infantum*	111	793	903
LinJ13_V3.1240	*L. infantum*	161	919	1079
LinJ13_V3.1240	*L. infantum*	52	1087	1138
LinJ13_V3.1240	*L. infantum*	253	1140	1392
LinJ13_V3.1250	*L. infantum*	84	44	127
LinJ13_V3.1250	*L. infantum*	101	193	293
LinJ13_V3.1250	*L. infantum*	115	564	678
LinJ13_V3.1250	*L. infantum*	62	726	787
LinJ13_V3.1250	*L. infantum*	70	819	888
LinJ13_V3.1250	*L. infantum*	47	966	1012
LinJ13_V3.1250	*L. infantum*	74	1023	1096
LinJ13_V3.1260	*L. infantum*	143	382	524
LinJ13_V3.1260	*L. infantum*	68	616	683
LinJ13_V3.1260	*L. infantum*	68	616	683
LinJ13_V3.1270	*L. infantum*	54	58	111
LinJ13_V3.1270	*L. infantum*	117	159	275
LinJ13_V3.1270	*L. infantum*	66	472	537
LinJ13_V3.1300	*L. infantum*	40	276	315
LinJ13_V3.1300	*L. infantum*	49	540	588

LinJ13_V3.1300	*L. infantum*	49	540	588
LinJ13_V3.1310	*L. infantum*	262	1	262
LinJ13_V3.1310	*L. infantum*	269	280	548
LinJ13_V3.1310	*L. infantum*	254	609	862
LinJ13_V3.1310	*L. infantum*	136	864	999
LinJ13_V3.1330	*L. infantum*	621	1	621
LinJ13_V3.1340	*L. infantum*	85	96	180
LinJ13_V3.1340	*L. infantum*	99	206	304
LinJ13_V3.1340	*L. infantum*	163	332	494
LinJ13_V3.1340	*L. infantum*	65	517	581
LinJ13_V3.1340	*L. infantum*	344	618	961
LinJ13_V3.1340	*L. infantum*	48	997	1044
LinJ13_V3.1340	*L. infantum*	55	1065	1119
LinJ13_V3.1350	*L. infantum*	143	63	205
LinJ13_V3.1350	*L. infantum*	159	512	670
LinJ13_V3.1370	*L. infantum*	40	984	1023
LinJ13_V3.1390	*L. infantum*	46	1	46
LinJ13_V3.1390	*L. infantum*	55	980	1034
LinJ13_V3.1390	*L. infantum*	85	1179	1263
LinJ13_V3.1390	*L. infantum*	90	3145	3234
LinJ13_V3.1390	*L. infantum*	62	3359	3420
LinJ13_V3.1390	*L. infantum*	76	4076	4151
LinJ13_V3.1390	*L. infantum*	60	4381	4440
LinJ13_V3.1410	*L. infantum*	49	30	78
LinJ13_V3.1450	*L. infantum*	82	1	82
LinJ13_V3.1450	*L. infantum*	43	244	286
LinJ13_V3.1450	*L. infantum*	43	244	286
LinJ13_V3.1470	*L. infantum*	78	32	109
LinJ13_V3.1470	*L. infantum*	174	291	464
LinJ13_V3.1470	*L. infantum*	132	494	625
LinJ13_V3.1480	*L. infantum*	289	1	289
LinJ13_V3.1480	*L. infantum*	120	697	816
LinJ13_V3.1480	*L. infantum*	82	932	1013
LinJ13_V3.1480	*L. infantum*	54	1112	1165
LinJ13_V3.1500	*L. infantum*	68	1	68
LinJ13_V3.1510	*L. infantum*	111	1	111
LinJ13_V3.1520	*L. infantum*	62	1	62
LinJ13_V3.1520	*L. infantum*	66	258	323
LinJ13_V3.1520	*L. infantum*	49	747	795
LinJ13_V3.1520	*L. infantum*	40	1051	1090
LinJ13_V3.1520	*L. infantum*	93	1195	1287
LinJ13_V3.1520	*L. infantum*	75	1403	1477
LinJ13_V3.1520	*L. infantum*	45	1526	1570
LinJ13_V3.1520	*L. infantum*	56	1672	1727
LinJ13_V3.1520	*L. infantum*	48	1926	1973
LinJ13_V3.1520	*L. infantum*	63	2070	2132
LinJ13_V3.1520	*L. infantum*	50	2168	2217

LinJ13_V3.1520	*L. infantum*	121	2387	2507
LinJ13_V3.1520	*L. infantum*	40	2816	2855
LinJ13_V3.1520	*L. infantum*	41	2905	2945
LinJ13_V3.1520	*L. infantum*	123	3100	3222
LinJ13_V3.1520	*L. infantum*	51	3232	3282
LinJ13_V3.1520	*L. infantum*	196	4103	4298
LinJ13_V3.1520	*L. infantum*	46	4639	4684
LinJ13_V3.1520	*L. infantum*	45	4745	4789
LinJ13_V3.1540	*L. infantum*	47	383	429
LinJ13_V3.1540	*L. infantum*	199	574	772
LinJ13_V3.1550	*L. infantum*	657	1	657
LinJ13_V3.1550	*L. infantum*	209	666	874
LinJ13_V3.1560	*L. infantum*	77	13	89
LinJ13_V3.1560	*L. infantum*	67	134	200
LinJ13_V3.1560	*L. infantum*	67	134	200
LinJ13_V3.1570	*L. infantum*	68	1	68
LinJ13_V3.1580	*L. infantum*	63	162	224
LinJ13_V3.1580	*L. infantum*	52	285	336
LinJ13_V3.1580	*L. infantum*	105	356	460
LinJ13_V3.1580	*L. infantum*	86	494	579
LinJ13_V3.1600	*L. infantum*	104	48	151
LinJ13_V3.1600	*L. infantum*	64	337	400
LinJ13_V3.1600	*L. infantum*	64	337	400
LinJ13_V3.1610	*L. infantum*	406	765	1170
LinJ13_V3.1630	*L. infantum*	159	80	238
LinJ14_V3.0020	*L. infantum*	53	1	53
LinJ14_V3.0020	*L. infantum*	42	166	207
LinJ14_V3.0020	*L. infantum*	42	166	207
LinJ14_V3.0030	*L. infantum*	49	28	76
LinJ14_V3.0030	*L. infantum*	141	130	270
LinJ14_V3.0040	*L. infantum*	89	89	177
LinJ14_V3.0050	*L. infantum*	44	509	552
LinJ14_V3.0050	*L. infantum*	146	587	732
LinJ14_V3.0090	*L. infantum*	260	89	348
LinJ14_V3.0100	*L. infantum*	56	1	56
LinJ14_V3.0100	*L. infantum*	51	318	368
LinJ14_V3.0100	*L. infantum*	43	399	441
LinJ14_V3.0100	*L. infantum*	46	558	603
LinJ14_V3.0110	*L. infantum*	65	379	443
LinJ14_V3.0110	*L. infantum*	57	467	523
LinJ14_V3.0120	*L. infantum*	148	1	148
LinJ14_V3.0120	*L. infantum*	114	214	327
LinJ14_V3.0140	*L. infantum*	85	57	141
LinJ14_V3.0140	*L. infantum*	56	184	239
LinJ14_V3.0150	*L. infantum*	48	76	123
LinJ14_V3.0160	*L. infantum*	83	58	140
LinJ14_V3.0170	*L. infantum*	125	188	312

LinJ14_V3.0170	*L. infantum*	56	630	685
LinJ14_V3.0190	*L. infantum*	43	157	199
LinJ14_V3.0200	*L. infantum*	96	78	173
LinJ14_V3.0210	*L. infantum*	43	1	43
LinJ14_V3.0210	*L. infantum*	69	149	217
LinJ14_V3.0210	*L. infantum*	59	232	290
LinJ14_V3.0210	*L. infantum*	53	338	390
LinJ14_V3.0220	*L. infantum*	72	157	228
LinJ14_V3.0230	*L. infantum*	112	151	262
LinJ14_V3.0230	*L. infantum*	48	325	372
LinJ14_V3.0230	*L. infantum*	70	448	517
LinJ14_V3.0230	*L. infantum*	47	557	603
LinJ14_V3.0230	*L. infantum*	44	643	686
LinJ14_V3.0230	*L. infantum*	83	820	902
LinJ14_V3.0230	*L. infantum*	71	1144	1214
LinJ14_V3.0230	*L. infantum*	292	1545	1836
LinJ14_V3.0260	*L. infantum*	41	363	403
LinJ14_V3.0260	*L. infantum*	43	590	632
LinJ14_V3.0260	*L. infantum*	43	590	632
LinJ14_V3.0270	*L. infantum*	52	1	52
LinJ14_V3.0280	*L. infantum*	128	836	963
LinJ14_V3.0290	*L. infantum*	45	1	45
LinJ14_V3.0300	*L. infantum*	97	61	157
LinJ14_V3.0310	*L. infantum*	51	235	285
LinJ14_V3.0320	*L. infantum*	155	649	803
LinJ14_V3.0330	*L. infantum*	202	1	202
LinJ14_V3.0340	*L. infantum*	377	1	377
LinJ14_V3.0340	*L. infantum*	124	408	531
LinJ14_V3.0340	*L. infantum*	75	545	619
LinJ14_V3.0340	*L. infantum*	43	641	683
LinJ14_V3.0340	*L. infantum*	118	718	835
LinJ14_V3.0340	*L. infantum*	47	863	909
LinJ14_V3.0340	*L. infantum*	45	933	977
LinJ14_V3.0350	*L. infantum*	53	624	676
LinJ14_V3.0360	*L. infantum*	210	62	271
LinJ14_V3.0360	*L. infantum*	61	409	469
LinJ14_V3.0360	*L. infantum*	135	602	736
LinJ14_V3.0360	*L. infantum*	270	772	1041
LinJ14_V3.0370	*L. infantum*	445	1	445
LinJ14_V3.0370	*L. infantum*	380	459	838
LinJ14_V3.0370	*L. infantum*	106	844	949
LinJ14_V3.0370	*L. infantum*	867	970	1836
LinJ14_V3.0370	*L. infantum*	168	1840	2007
LinJ14_V3.0370	*L. infantum*	380	2044	2423
LinJ14_V3.0370	*L. infantum*	292	2428	2719
LinJ14_V3.0370	*L. infantum*	64	2729	2792
LinJ14_V3.0370	*L. infantum*	69	2843	2911

LinJ14_V3.0380	*L. infantum*	60	43	102
LinJ14_V3.0410	*L. infantum*	104	54	157
LinJ14_V3.0420	*L. infantum*	342	1	342
LinJ14_V3.0420	*L. infantum*	101	566	666
LinJ14_V3.0430	*L. infantum*	160	170	329
LinJ14_V3.0430	*L. infantum*	479	342	820
LinJ14_V3.0450	*L. infantum*	44	250	293
LinJ14_V3.0460	*L. infantum*	115	40	154
LinJ14_V3.0460	*L. infantum*	64	259	322
LinJ14_V3.0470	*L. infantum*	59	1	59
LinJ14_V3.0510	*L. infantum*	150	1	150
LinJ14_V3.0510	*L. infantum*	195	160	354
LinJ14_V3.0510	*L. infantum*	96	402	497
LinJ14_V3.0530	*L. infantum*	70	1	70
LinJ14_V3.0540	*L. infantum*	165	340	504
LinJ14_V3.0560	*L. infantum*	41	324	364
LinJ14_V3.0570	*L. infantum*	176	313	488
LinJ14_V3.0580	*L. infantum*	48	28	75
LinJ14_V3.0580	*L. infantum*	83	411	493
LinJ14_V3.0580	*L. infantum*	45	722	766
LinJ14_V3.0580	*L. infantum*	57	934	990
LinJ14_V3.0580	*L. infantum*	44	1082	1125
LinJ14_V3.0600	*L. infantum*	58	329	386
LinJ14_V3.0600	*L. infantum*	43	436	478
LinJ14_V3.0600	*L. infantum*	118	679	796
LinJ14_V3.0600	*L. infantum*	89	888	976
LinJ14_V3.0600	*L. infantum*	178	1043	1220
LinJ14_V3.0600	*L. infantum*	122	1262	1383
LinJ14_V3.0610	*L. infantum*	75	1	75
LinJ14_V3.0610	*L. infantum*	61	128	188
LinJ14_V3.0620	*L. infantum*	163	1	163
LinJ14_V3.0620	*L. infantum*	45	179	223
LinJ14_V3.0620	*L. infantum*	47	277	323
LinJ14_V3.0620	*L. infantum*	109	355	463
LinJ14_V3.0630	*L. infantum*	48	13	60
LinJ14_V3.0630	*L. infantum*	161	146	306
LinJ14_V3.0630	*L. infantum*	240	352	591
LinJ14_V3.0640	*L. infantum*	156	71	226
LinJ14_V3.0740	*L. infantum*	62	274	335
LinJ14_V3.0800	*L. infantum*	40	264	303
LinJ14_V3.0810	*L. infantum*	72	1	72
LinJ14_V3.0810	*L. infantum*	159	513	671
LinJ14_V3.0810	*L. infantum*	89	683	771
LinJ14_V3.0810	*L. infantum*	79	786	864
LinJ14_V3.0810	*L. infantum*	51	907	957
LinJ14_V3.0820	*L. infantum*	80	1	80
LinJ14_V3.0820	*L. infantum*	111	307	417

LinJ14_V3.0820	*L. infantum*	111	307	417
LinJ14_V3.0830	*L. infantum*	42	1	42
LinJ14_V3.0830	*L. infantum*	111	80	190
LinJ14_V3.0830	*L. infantum*	152	204	355
LinJ14_V3.0850	*L. infantum*	74	1	74
LinJ14_V3.0850	*L. infantum*	44	94	137
LinJ14_V3.0850	*L. infantum*	201	167	367
LinJ14_V3.0850	*L. infantum*	64	388	451
LinJ14_V3.0850	*L. infantum*	84	621	704
LinJ14_V3.0850	*L. infantum*	84	727	810
LinJ14_V3.0850	*L. infantum*	56	857	912
LinJ14_V3.0850	*L. infantum*	101	942	1042
LinJ14_V3.0850	*L. infantum*	50	1052	1101
LinJ14_V3.0850	*L. infantum*	151	1168	1318
LinJ14_V3.0850	*L. infantum*	44	1410	1453
LinJ14_V3.0850	*L. infantum*	71	1505	1575
LinJ14_V3.0850	*L. infantum*	209	1690	1898
LinJ14_V3.0850	*L. infantum*	105	1911	2015
LinJ14_V3.0850	*L. infantum*	96	2078	2173
LinJ14_V3.0850	*L. infantum*	107	2220	2326
LinJ14_V3.0850	*L. infantum*	102	2333	2434
LinJ14_V3.0850	*L. infantum*	263	2473	2735
LinJ14_V3.0850	*L. infantum*	154	2738	2891
LinJ14_V3.0850	*L. infantum*	1099	2895	3993
LinJ14_V3.0850	*L. infantum*	79	4017	4095
LinJ14_V3.0850	*L. infantum*	96	4136	4231
LinJ14_V3.0860	*L. infantum*	429	1	429
LinJ14_V3.0870	*L. infantum*	48	1	48
LinJ14_V3.0870	*L. infantum*	87	76	162
LinJ14_V3.0870	*L. infantum*	59	201	259
LinJ14_V3.0870	*L. infantum*	221	606	826
LinJ14_V3.0870	*L. infantum*	379	837	1215
LinJ14_V3.0870	*L. infantum*	61	1247	1307
LinJ14_V3.0880	*L. infantum*	432	146	577
LinJ14_V3.0890	*L. infantum*	125	211	335
LinJ14_V3.0890	*L. infantum*	98	422	519
LinJ14_V3.0890	*L. infantum*	40	554	593
LinJ14_V3.0900	*L. infantum*	229	1	229
LinJ14_V3.0900	*L. infantum*	228	231	458
LinJ14_V3.0900	*L. infantum*	117	1053	1169
LinJ14_V3.0900	*L. infantum*	82	1180	1261
LinJ14_V3.0910	*L. infantum*	47	69	115
LinJ14_V3.0920	*L. infantum*	47	69	115
LinJ14_V3.0940	*L. infantum*	71	1	71
LinJ14_V3.0940	*L. infantum*	95	102	196
LinJ14_V3.0940	*L. infantum*	188	395	582
LinJ14_V3.0940	*L. infantum*	230	638	867

LinJ14_V3.0940	*L. infantum*	46	923	968
LinJ14_V3.0950	*L. infantum*	63	160	222
LinJ14_V3.0950	*L. infantum*	275	271	545
LinJ14_V3.0950	*L. infantum*	67	591	657
LinJ14_V3.0960	*L. infantum*	42	1	42
LinJ14_V3.0960	*L. infantum*	362	60	421
LinJ14_V3.0960	*L. infantum*	99	430	528
LinJ14_V3.0960	*L. infantum*	47	754	800
LinJ14_V3.0960	*L. infantum*	58	913	970
LinJ14_V3.0990	*L. infantum*	41	164	204
LinJ14_V3.0990	*L. infantum*	108	366	473
LinJ14_V3.0990	*L. infantum*	105	622	726
LinJ14_V3.1000	*L. infantum*	43	1	43
LinJ14_V3.1000	*L. infantum*	510	80	589
LinJ14_V3.1000	*L. infantum*	68	630	697
LinJ14_V3.1000	*L. infantum*	109	770	878
LinJ14_V3.1020	*L. infantum*	214	1	214
LinJ14_V3.1020	*L. infantum*	59	396	454
LinJ14_V3.1020	*L. infantum*	51	713	763
LinJ14_V3.1020	*L. infantum*	60	815	874
LinJ14_V3.1020	*L. infantum*	51	936	986
LinJ14_V3.1020	*L. infantum*	81	1034	1114
LinJ14_V3.1040	*L. infantum*	99	548	646
LinJ14_V3.1050	*L. infantum*	40	1	40
LinJ14_V3.1060	*L. infantum*	50	1	50
LinJ14_V3.1070	*L. infantum*	88	1	88
LinJ14_V3.1070	*L. infantum*	89	91	179
LinJ14_V3.1070	*L. infantum*	115	419	533
LinJ14_V3.1070	*L. infantum*	51	826	876
LinJ14_V3.1080	*L. infantum*	97	58	154
LinJ14_V3.1080	*L. infantum*	49	266	314
LinJ14_V3.1130	*L. infantum*	62	1574	1635
LinJ14_V3.1130	*L. infantum*	50	1850	1899
LinJ14_V3.1130	*L. infantum*	49	2034	2082
LinJ14_V3.1130	*L. infantum*	118	2628	2745
LinJ14_V3.1130	*L. infantum*	46	2852	2897
LinJ14_V3.1130	*L. infantum*	71	3282	3352
LinJ14_V3.1130	*L. infantum*	66	3826	3891
LinJ14_V3.1130	*L. infantum*	80	3915	3994
LinJ14_V3.1140	*L. infantum*	199	31	229
LinJ14_V3.1140	*L. infantum*	77	239	315
LinJ14_V3.1140	*L. infantum*	54	375	428
LinJ14_V3.1140	*L. infantum*	79	487	565
LinJ14_V3.1140	*L. infantum*	62	799	860
LinJ14_V3.1150	*L. infantum*	49	148	196
LinJ14_V3.1170	*L. infantum*	43	129	171
LinJ14_V3.1170	*L. infantum*	47	355	401

LinJ14_V3.1170	*L. infantum*	47	355	401
LinJ14_V3.1180	*L. infantum*	232	62	293
LinJ14_V3.1180	*L. infantum*	2949	331	3279
LinJ14_V3.1190	*L. infantum*	108	1	108
LinJ14_V3.1190	*L. infantum*	64	280	343
LinJ14_V3.1190	*L. infantum*	165	428	592
LinJ14_V3.1190	*L. infantum*	205	636	840
LinJ14_V3.1190	*L. infantum*	1982	914	2895
LinJ14_V3.1200	*L. infantum*	209	1	209
LinJ14_V3.1200	*L. infantum*	91	326	416
LinJ14_V3.1200	*L. infantum*	79	669	747
LinJ14_V3.1200	*L. infantum*	42	856	897
LinJ14_V3.1210	*L. infantum*	59	149	207
LinJ14_V3.1210	*L. infantum*	49	428	476
LinJ14_V3.1220	*L. infantum*	190	1	190
LinJ14_V3.1220	*L. infantum*	68	347	414
LinJ14_V3.1230	*L. infantum*	60	1	60
LinJ14_V3.1230	*L. infantum*	190	119	308
LinJ14_V3.1250	*L. infantum*	143	141	283
LinJ14_V3.1250	*L. infantum*	62	326	387
LinJ14_V3.1260	*L. infantum*	64	432	495
LinJ14_V3.1260	*L. infantum*	152	558	709
LinJ14_V3.1280	*L. infantum*	81	1	81
LinJ14_V3.1290	*L. infantum*	62	86	147
LinJ14_V3.1300	*L. infantum*	140	56	195
LinJ14_V3.1300	*L. infantum*	166	221	386
LinJ14_V3.1300	*L. infantum*	82	414	495
LinJ14_V3.1300	*L. infantum*	174	569	742
LinJ14_V3.1300	*L. infantum*	91	841	931
LinJ14_V3.1310	*L. infantum*	48	297	344
LinJ14_V3.1310	*L. infantum*	56	406	461
LinJ14_V3.1310	*L. infantum*	75	479	553
LinJ14_V3.1320	*L. infantum*	73	122	194
LinJ14_V3.1320	*L. infantum*	57	246	302
LinJ14_V3.1350	*L. infantum*	56	66	121
LinJ14_V3.1360	*L. infantum*	40	67	106
LinJ14_V3.1360	*L. infantum*	44	201	244
LinJ14_V3.1360	*L. infantum*	132	283	414
LinJ14_V3.1360	*L. infantum*	48	450	497
LinJ14_V3.1370	*L. infantum*	93	14	106
LinJ14_V3.1370	*L. infantum*	564	238	801
LinJ14_V3.1380	*L. infantum*	146	1	146
LinJ14_V3.1380	*L. infantum*	204	464	667
LinJ14_V3.1380	*L. infantum*	116	684	799
LinJ14_V3.1390	*L. infantum*	175	15	189
LinJ14_V3.1390	*L. infantum*	53	273	325
LinJ14_V3.1390	*L. infantum*	53	273	325

LinJ14_V3.1410	*L. infantum*	174	401	574
LinJ14_V3.1430	*L. infantum*	87	1	87
LinJ14_V3.1430	*L. infantum*	59	110	168
LinJ14_V3.1430	*L. infantum*	53	322	374
LinJ14_V3.1430	*L. infantum*	43	533	575
LinJ14_V3.1460	*L. infantum*	49	216	264
LinJ14_V3.1470	*L. infantum*	48	270	317
LinJ14_V3.1500	*L. infantum*	61	1	61
LinJ14_V3.1500	*L. infantum*	175	99	273
LinJ14_V3.1500	*L. infantum*	117	312	428
LinJ14_V3.1500	*L. infantum*	71	882	952
LinJ14_V3.1500	*L. infantum*	59	1232	1290
LinJ14_V3.1510	*L. infantum*	264	292	555
LinJ14_V3.1520	*L. infantum*	45	494	538
LinJ14_V3.1530	*L. infantum*	113	422	534
LinJ14_V3.1530	*L. infantum*	67	582	648
LinJ14_V3.1530	*L. infantum*	48	652	699
LinJ14_V3.1530	*L. infantum*	161	785	945
LinJ14_V3.1530	*L. infantum*	167	1041	1207
LinJ14_V3.1540	*L. infantum*	100	1	100
LinJ14_V3.1540	*L. infantum*	483	524	1006
LinJ14_V3.1550	*L. infantum*	47	1	47
LinJ14_V3.1570	*L. infantum*	161	26	186
LinJ14_V3.1570	*L. infantum*	66	237	302
LinJ14_V3.1570	*L. infantum*	63	343	405
LinJ14_V3.1570	*L. infantum*	75	410	484
LinJ14_V3.1580	*L. infantum*	40	228	267
LinJ14_V3.1590	*L. infantum*	65	34	98
LinJ14_V3.1590	*L. infantum*	43	728	770
LinJ14_V3.1590	*L. infantum*	43	728	770
LinJ15_V3.0010	*L. infantum*	49	1	49
LinJ15_V3.0050	*L. infantum*	42	1	42
LinJ15_V3.0050	*L. infantum*	42	238	279
LinJ15_V3.0050	*L. infantum*	42	238	279
LinJ15_V3.0060	*L. infantum*	103	1	103
LinJ15_V3.0060	*L. infantum*	43	168	210
LinJ15_V3.0060	*L. infantum*	64	264	327
LinJ15_V3.0060	*L. infantum*	162	431	592
LinJ15_V3.0070	*L. infantum*	304	120	423
LinJ15_V3.0080	*L. infantum*	132	190	321
LinJ15_V3.0090	*L. infantum*	45	106	150
LinJ15_V3.0090	*L. infantum*	45	155	199
LinJ15_V3.0090	*L. infantum*	45	155	199
LinJ15_V3.0110	*L. infantum*	40	1	40
LinJ15_V3.0110	*L. infantum*	47	71	117
LinJ15_V3.0110	*L. infantum*	52	200	251
LinJ15_V3.0110	*L. infantum*	64	319	382

LinJ15_V3.0110	*L. infantum*	59	655	713
LinJ15_V3.0130	*L. infantum*	61	244	304
LinJ15_V3.0130	*L. infantum*	168	483	650
LinJ15_V3.0140	*L. infantum*	156	60	215
LinJ15_V3.0150	*L. infantum*	43	35	77
LinJ15_V3.0150	*L. infantum*	70	113	182
LinJ15_V3.0150	*L. infantum*	70	113	182
LinJ15_V3.0160	*L. infantum*	229	1	229
LinJ15_V3.0160	*L. infantum*	314	241	554
LinJ15_V3.0160	*L. infantum*	77	587	663
LinJ15_V3.0160	*L. infantum*	64	665	728
LinJ15_V3.0160	*L. infantum*	50	758	807
LinJ15_V3.0170	*L. infantum*	107	1	107
LinJ15_V3.0180	*L. infantum*	54	1	54
LinJ15_V3.0180	*L. infantum*	241	63	303
LinJ15_V3.0180	*L. infantum*	90	656	745
LinJ15_V3.0190	*L. infantum*	160	27	186
LinJ15_V3.0190	*L. infantum*	294	192	485
LinJ15_V3.0190	*L. infantum*	82	504	585
LinJ15_V3.0210	*L. infantum*	69	70	138
LinJ15_V3.0210	*L. infantum*	42	187	228
LinJ15_V3.0210	*L. infantum*	42	187	228
LinJ15_V3.0220	*L. infantum*	46	177	222
LinJ15_V3.0230	*L. infantum*	78	212	289
LinJ15_V3.0230	*L. infantum*	98	363	460
LinJ15_V3.0250	*L. infantum*	60	156	215
LinJ15_V3.0270	*L. infantum*	65	1	65
LinJ15_V3.0290	*L. infantum*	122	57	178
LinJ15_V3.0290	*L. infantum*	112	257	368
LinJ15_V3.0290	*L. infantum*	59	410	468
LinJ15_V3.0290	*L. infantum*	80	504	583
LinJ15_V3.0290	*L. infantum*	82	665	746
LinJ15_V3.0290	*L. infantum*	129	782	910
LinJ15_V3.0290	*L. infantum*	68	932	999
LinJ15_V3.0290	*L. infantum*	52	1348	1399
LinJ15_V3.0290	*L. infantum*	57	1551	1607
LinJ15_V3.0300	*L. infantum*	176	44	219
LinJ15_V3.0300	*L. infantum*	54	291	344
LinJ15_V3.0300	*L. infantum*	93	375	467
LinJ15_V3.0340	*L. infantum*	50	66	115
LinJ15_V3.0340	*L. infantum*	219	582	800
LinJ15_V3.0360	*L. infantum*	93	1	93
LinJ15_V3.0360	*L. infantum*	213	160	372
LinJ15_V3.0360	*L. infantum*	61	390	450
LinJ15_V3.0360	*L. infantum*	163	459	621
LinJ15_V3.0370	*L. infantum*	47	142	188
LinJ15_V3.0370	*L. infantum*	63	225	287

LinJ15_V3.0370	*L. infantum*	43	310	352
LinJ15_V3.0370	*L. infantum*	67	662	728
LinJ15_V3.0380	*L. infantum*	408	85	492
LinJ15_V3.0380	*L. infantum*	84	735	818
LinJ15_V3.0380	*L. infantum*	43	861	903
LinJ15_V3.0380	*L. infantum*	46	947	992
LinJ15_V3.0390	*L. infantum*	173	1	173
LinJ15_V3.0390	*L. infantum*	111	209	319
LinJ15_V3.0390	*L. infantum*	116	324	439
LinJ15_V3.0400	*L. infantum*	47	257	303
LinJ15_V3.0410	*L. infantum*	81	1	81
LinJ15_V3.0410	*L. infantum*	111	110	220
LinJ15_V3.0410	*L. infantum*	101	251	351
LinJ15_V3.0410	*L. infantum*	56	409	464
LinJ15_V3.0420	*L. infantum*	51	58	108
LinJ15_V3.0430	*L. infantum*	51	58	108
LinJ15_V3.0450	*L. infantum*	54	30	83
LinJ15_V3.0450	*L. infantum*	44	286	329
LinJ15_V3.0450	*L. infantum*	98	365	462
LinJ15_V3.0460	*L. infantum*	62	409	470
LinJ15_V3.0470	*L. infantum*	50	1	50
LinJ15_V3.0480	*L. infantum*	62	46	107
LinJ15_V3.0480	*L. infantum*	151	138	288
LinJ15_V3.0500	*L. infantum*	116	1	116
LinJ15_V3.0500	*L. infantum*	83	132	214
LinJ15_V3.0500	*L. infantum*	40	236	275
LinJ15_V3.0500	*L. infantum*	139	313	451
LinJ15_V3.0500	*L. infantum*	40	610	649
LinJ15_V3.0500	*L. infantum*	92	907	998
LinJ15_V3.0500	*L. infantum*	68	1107	1174
LinJ15_V3.0500	*L. infantum*	131	1484	1614
LinJ15_V3.0500	*L. infantum*	482	1620	2101
LinJ15_V3.0500	*L. infantum*	69	2142	2210
LinJ15_V3.0500	*L. infantum*	129	2277	2405
LinJ15_V3.0500	*L. infantum*	167	2486	2652
LinJ15_V3.0510	*L. infantum*	549	1	549
LinJ15_V3.0510	*L. infantum*	41	728	768
LinJ15_V3.0520	*L. infantum*	45	50	94
LinJ15_V3.0520	*L. infantum*	471	177	647
LinJ15_V3.0520	*L. infantum*	99	822	920
LinJ15_V3.0520	*L. infantum*	43	922	964
LinJ15_V3.0540	*L. infantum*	241	138	378
LinJ15_V3.0550	*L. infantum*	562	1	562
LinJ15_V3.0550	*L. infantum*	61	648	708
LinJ15_V3.0550	*L. infantum*	218	754	971
LinJ15_V3.0550	*L. infantum*	232	1033	1264
LinJ15_V3.0550	*L. infantum*	54	1607	1660

LinJ15_V3.0550	*L. infantum*	46	1677	1722
LinJ15_V3.0560	*L. infantum*	41	1	41
LinJ15_V3.0560	*L. infantum*	132	63	194
LinJ15_V3.0560	*L. infantum*	126	288	413
LinJ15_V3.0570	*L. infantum*	40	1	40
LinJ15_V3.0570	*L. infantum*	139	58	196
LinJ15_V3.0570	*L. infantum*	41	337	377
LinJ15_V3.0570	*L. infantum*	64	388	451
LinJ15_V3.0570	*L. infantum*	209	616	824
LinJ15_V3.0570	*L. infantum*	105	853	957
LinJ15_V3.0570	*L. infantum*	104	989	1092
LinJ15_V3.0570	*L. infantum*	88	1160	1247
LinJ15_V3.0570	*L. infantum*	116	1332	1447
LinJ15_V3.0570	*L. infantum*	100	1597	1696
LinJ15_V3.0580	*L. infantum*	104	1	104
LinJ15_V3.0580	*L. infantum*	216	124	339
LinJ15_V3.0580	*L. infantum*	78	374	451
LinJ15_V3.0580	*L. infantum*	69	474	542
LinJ15_V3.0590	*L. infantum*	67	103	169
LinJ15_V3.0600	*L. infantum*	40	76	115
LinJ15_V3.0600	*L. infantum*	47	187	233
LinJ15_V3.0600	*L. infantum*	47	187	233
LinJ15_V3.0610	*L. infantum*	121	103	223
LinJ15_V3.0620	*L. infantum*	648	1	648
LinJ15_V3.0620	*L. infantum*	201	696	896
LinJ15_V3.0620	*L. infantum*	421	1127	1547
LinJ15_V3.0620	*L. infantum*	170	1598	1767
LinJ15_V3.0630	*L. infantum*	107	17	123
LinJ15_V3.0630	*L. infantum*	42	149	190
LinJ15_V3.0630	*L. infantum*	334	223	556
LinJ15_V3.0630	*L. infantum*	330	728	1057
LinJ15_V3.0630	*L. infantum*	93	1093	1185
LinJ15_V3.0650	*L. infantum*	43	1	43
LinJ15_V3.0650	*L. infantum*	105	257	361
LinJ15_V3.0650	*L. infantum*	105	257	361
LinJ15_V3.0660	*L. infantum*	223	1	223
LinJ15_V3.0660	*L. infantum*	276	239	514
LinJ15_V3.0660	*L. infantum*	222	542	763
LinJ15_V3.0660	*L. infantum*	171	785	955
LinJ15_V3.0670	*L. infantum*	160	1	160
LinJ15_V3.0670	*L. infantum*	110	579	688
LinJ15_V3.0670	*L. infantum*	75	740	814
LinJ15_V3.0670	*L. infantum*	76	880	955
LinJ15_V3.0670	*L. infantum*	175	975	1149
LinJ15_V3.0680	*L. infantum*	393	1	393
LinJ15_V3.0680	*L. infantum*	145	492	636
LinJ15_V3.0680	*L. infantum*	60	640	699

LinJ15_V3.0690	*L. infantum*	119	1	119
LinJ15_V3.0690	*L. infantum*	55	123	177
LinJ15_V3.0690	*L. infantum*	54	216	269
LinJ15_V3.0690	*L. infantum*	179	284	462
LinJ15_V3.0710	*L. infantum*	60	1	60
LinJ15_V3.0710	*L. infantum*	42	160	201
LinJ15_V3.0710	*L. infantum*	42	160	201
LinJ15_V3.0730	*L. infantum*	195	43	237
LinJ15_V3.0730	*L. infantum*	201	270	470
LinJ15_V3.0730	*L. infantum*	95	499	593
LinJ15_V3.0730	*L. infantum*	40	619	658
LinJ15_V3.0730	*L. infantum*	195	696	890
LinJ15_V3.0730	*L. infantum*	201	923	1123
LinJ15_V3.0730	*L. infantum*	95	1152	1246
LinJ15_V3.0730	*L. infantum*	40	1272	1311
LinJ15_V3.0730	*L. infantum*	64	1349	1412
LinJ15_V3.0740	*L. infantum*	88	89	176
LinJ15_V3.0740	*L. infantum*	66	198	263
LinJ15_V3.0740	*L. infantum*	99	309	407
LinJ15_V3.0740	*L. infantum*	102	513	614
LinJ15_V3.0750	*L. infantum*	85	72	156
LinJ15_V3.0750	*L. infantum*	56	210	265
LinJ15_V3.0750	*L. infantum*	53	384	436
LinJ15_V3.0750	*L. infantum*	41	650	690
LinJ15_V3.0750	*L. infantum*	63	740	802
LinJ15_V3.0750	*L. infantum*	50	867	916
LinJ15_V3.0750	*L. infantum*	67	1125	1191
LinJ15_V3.0750	*L. infantum*	45	1222	1266
LinJ15_V3.0750	*L. infantum*	228	1924	2151
LinJ15_V3.0750	*L. infantum*	68	2181	2248
LinJ15_V3.0750	*L. infantum*	63	2627	2689
LinJ15_V3.0760	*L. infantum*	95	91	185
LinJ15_V3.0760	*L. infantum*	48	303	350
LinJ15_V3.0770	*L. infantum*	65	1	65
LinJ15_V3.0770	*L. infantum*	80	75	154
LinJ15_V3.0770	*L. infantum*	60	203	262
LinJ15_V3.0790	*L. infantum*	333	1	333
LinJ15_V3.0790	*L. infantum*	119	354	472
LinJ15_V3.0790	*L. infantum*	92	574	665
LinJ15_V3.0810	*L. infantum*	297	364	660
LinJ15_V3.0820	*L. infantum*	87	569	655
LinJ15_V3.0830	*L. infantum*	61	522	582
LinJ15_V3.0830	*L. infantum*	54	629	682
LinJ15_V3.0830	*L. infantum*	77	1321	1397
LinJ15_V3.0830	*L. infantum*	40	1481	1520
LinJ15_V3.0830	*L. infantum*	44	1612	1655
LinJ15_V3.0830	*L. infantum*	51	1751	1801

LinJ15_V3.0830	*L. infantum*	226	1874	2099
LinJ15_V3.0840	*L. infantum*	289	1	289
LinJ15_V3.0840	*L. infantum*	128	307	434
LinJ15_V3.0840	*L. infantum*	139	498	636
LinJ15_V3.0840	*L. infantum*	198	660	857
LinJ15_V3.0840	*L. infantum*	339	863	1201
LinJ15_V3.0840	*L. infantum*	151	1219	1369
LinJ15_V3.0840	*L. infantum*	303	1376	1678
LinJ15_V3.0840	*L. infantum*	94	1710	1803
LinJ15_V3.0840	*L. infantum*	137	1858	1994
LinJ15_V3.0840	*L. infantum*	68	2015	2082
LinJ15_V3.0850	*L. infantum*	41	254	294
LinJ15_V3.0850	*L. infantum*	46	342	387
LinJ15_V3.0850	*L. infantum*	44	449	492
LinJ15_V3.0880	*L. infantum*	44	1	44
LinJ15_V3.0880	*L. infantum*	52	80	131
LinJ15_V3.0880	*L. infantum*	271	143	413
LinJ15_V3.0890	*L. infantum*	100	1	100
LinJ15_V3.0890	*L. infantum*	189	167	355
LinJ15_V3.0890	*L. infantum*	50	358	407
LinJ15_V3.0890	*L. infantum*	127	434	560
LinJ15_V3.0890	*L. infantum*	215	589	803
LinJ15_V3.0900	*L. infantum*	124	1	124
LinJ15_V3.0910	*L. infantum*	70	12	81
LinJ15_V3.0910	*L. infantum*	61	110	170
LinJ15_V3.0910	*L. infantum*	58	220	277
LinJ15_V3.0910	*L. infantum*	118	449	566
LinJ15_V3.0920	*L. infantum*	245	62	306
LinJ15_V3.0930	*L. infantum*	51	1	51
LinJ15_V3.0930	*L. infantum*	146	247	392
LinJ15_V3.0930	*L. infantum*	146	247	392
LinJ15_V3.0940	*L. infantum*	100	190	289
LinJ15_V3.0950	*L. infantum*	42	1	42
LinJ15_V3.0950	*L. infantum*	87	655	741
LinJ15_V3.0960	*L. infantum*	64	59	122
LinJ15_V3.0960	*L. infantum*	69	210	278
LinJ15_V3.0960	*L. infantum*	69	210	278
LinJ15_V3.0970	*L. infantum*	42	1	42
LinJ15_V3.0970	*L. infantum*	129	54	182
LinJ15_V3.0970	*L. infantum*	52	222	273
LinJ15_V3.0970	*L. infantum*	197	311	507
LinJ15_V3.0970	*L. infantum*	154	541	694
LinJ15_V3.0970	*L. infantum*	51	725	775
LinJ15_V3.0970	*L. infantum*	122	859	980
LinJ15_V3.0970	*L. infantum*	431	1280	1710
LinJ15_V3.0970	*L. infantum*	86	1818	1903
LinJ15_V3.0970	*L. infantum*	106	2030	2135

LinJ15_V3.0970	*L. infantum*	135	2183	2317
LinJ15_V3.0980	*L. infantum*	129	1	129
LinJ15_V3.0980	*L. infantum*	70	541	610
LinJ15_V3.1000	*L. infantum*	59	117	175
LinJ15_V3.1000	*L. infantum*	86	189	274
LinJ15_V3.1000	*L. infantum*	106	304	409
LinJ15_V3.1000	*L. infantum*	84	418	501
LinJ15_V3.1000	*L. infantum*	58	565	622
LinJ15_V3.1000	*L. infantum*	62	649	710
LinJ15_V3.1000	*L. infantum*	66	782	847
LinJ15_V3.1000	*L. infantum*	41	889	929
LinJ15_V3.1000	*L. infantum*	242	964	1205
LinJ15_V3.1020	*L. infantum*	61	123	183
LinJ15_V3.1020	*L. infantum*	91	201	291
LinJ15_V3.1030	*L. infantum*	44	278	321
LinJ15_V3.1040	*L. infantum*	46	1	46
LinJ15_V3.1040	*L. infantum*	69	76	144
LinJ15_V3.1040	*L. infantum*	145	361	505
LinJ15_V3.1040	*L. infantum*	255	607	861
LinJ15_V3.1050	*L. infantum*	40	158	197
LinJ15_V3.1060	*L. infantum*	72	92	163
LinJ15_V3.1070	*L. infantum*	46	597	642
LinJ15_V3.1070	*L. infantum*	74	659	732
LinJ15_V3.1070	*L. infantum*	74	659	732
LinJ15_V3.1080	*L. infantum*	51	309	359
LinJ15_V3.1090	*L. infantum*	135	1	135
LinJ15_V3.1090	*L. infantum*	264	164	427
LinJ15_V3.1110	*L. infantum*	55	62	116
LinJ15_V3.1130	*L. infantum*	55	62	116
LinJ15_V3.1150	*L. infantum*	60	66	125
LinJ15_V3.1150	*L. infantum*	56	282	337
LinJ15_V3.1150	*L. infantum*	66	363	428
LinJ15_V3.1160	*L. infantum*	102	186	287
LinJ15_V3.1160	*L. infantum*	47	364	410
LinJ15_V3.1160	*L. infantum*	79	442	520
LinJ15_V3.1160	*L. infantum*	58	709	766
LinJ15_V3.1160	*L. infantum*	40	985	1024
LinJ15_V3.1160	*L. infantum*	57	1080	1136
LinJ15_V3.1160	*L. infantum*	93	1657	1749
LinJ15_V3.1160	*L. infantum*	82	1765	1846
LinJ15_V3.1160	*L. infantum*	40	2151	2190
LinJ15_V3.1160	*L. infantum*	105	2205	2309
LinJ15_V3.1160	*L. infantum*	211	2432	2642
LinJ15_V3.1160	*L. infantum*	469	2664	3132
LinJ15_V3.1170	*L. infantum*	131	1	131
LinJ15_V3.1170	*L. infantum*	173	139	311
LinJ15_V3.1170	*L. infantum*	46	411	456

LinJ15_V3.1170	*L. infantum*	106	938	1043
LinJ15_V3.1180	*L. infantum*	101	1	101
LinJ15_V3.1180	*L. infantum*	232	139	370
LinJ15_V3.1180	*L. infantum*	57	478	534
LinJ15_V3.1180	*L. infantum*	40	658	697
LinJ15_V3.1180	*L. infantum*	154	989	1142
LinJ15_V3.1180	*L. infantum*	327	1593	1919
LinJ15_V3.1180	*L. infantum*	68	1931	1998
LinJ15_V3.1180	*L. infantum*	254	2043	2296
LinJ15_V3.1180	*L. infantum*	143	2322	2464
LinJ15_V3.1180	*L. infantum*	175	2484	2658
LinJ15_V3.1190	*L. infantum*	54	58	111
LinJ15_V3.1200	*L. infantum*	54	58	111
LinJ15_V3.1210	*L. infantum*	60	366	425
LinJ15_V3.1220	*L. infantum*	41	14	54
LinJ15_V3.1220	*L. infantum*	70	57	126
LinJ15_V3.1220	*L. infantum*	43	146	188
LinJ15_V3.1220	*L. infantum*	44	218	261
LinJ15_V3.1230	*L. infantum*	56	258	313
LinJ15_V3.1240	*L. infantum*	56	258	313
LinJ15_V3.1250	*L. infantum*	56	258	313
LinJ15_V3.1260	*L. infantum*	56	258	313
LinJ15_V3.1280	*L. infantum*	71	337	407
LinJ15_V3.1290	*L. infantum*	63	1	63
LinJ15_V3.1290	*L. infantum*	90	252	341
LinJ15_V3.1290	*L. infantum*	107	438	544
LinJ15_V3.1300b	*L. infantum*	89	66	154
LinJ15_V3.1300b	*L. infantum*	97	205	301
LinJ15_V3.1300b	*L. infantum*	431	388	818
LinJ15_V3.1300b	*L. infantum*	212	837	1048
LinJ15_V3.1300b	*L. infantum*	67	1069	1135
LinJ15_V3.1300b	*L. infantum*	62	1193	1254
LinJ15_V3.1300b	*L. infantum*	40	1320	1359
LinJ15_V3.1300b	*L. infantum*	82	1373	1454
LinJ15_V3.1300b	*L. infantum*	97	1466	1562
LinJ15_V3.1310	*L. infantum*	73	1144	1216
LinJ15_V3.1320	*L. infantum*	113	1	113
LinJ15_V3.1320	*L. infantum*	148	907	1054
LinJ15_V3.1330	*L. infantum*	215	1	215
LinJ15_V3.1330	*L. infantum*	100	253	352
LinJ15_V3.1330	*L. infantum*	70	415	484
LinJ15_V3.1340	*L. infantum*	251	62	312
LinJ15_V3.1340	*L. infantum*	75	349	423
LinJ15_V3.1340	*L. infantum*	49	558	606
LinJ15_V3.1340	*L. infantum*	129	717	845
LinJ15_V3.1340	*L. infantum*	270	1186	1455
LinJ15_V3.1350	*L. infantum*	51	240	290

LinJ15_V3.1360	*L. infantum*	80	159	238
LinJ15_V3.1380	*L. infantum*	303	1	303
LinJ15_V3.1390	*L. infantum*	55	1	55
LinJ15_V3.1390	*L. infantum*	66	107	172
LinJ15_V3.1400	*L. infantum*	46	152	197
LinJ15_V3.1430	*L. infantum*	91	355	445
LinJ15_V3.1450	*L. infantum*	82	214	295
LinJ15_V3.1460	*L. infantum*	142	337	478
LinJ15_V3.1490	*L. infantum*	47	1	47
LinJ15_V3.1500	*L. infantum*	59	166	224
LinJ15_V3.1550	*L. infantum*	41	54	94
LinJ15_V3.1560	*L. infantum*	47	1	47
LinJ15_V3.1560	*L. infantum*	68	114	181
LinJ15_V3.1560	*L. infantum*	289	378	666
LinJ15_V3.1590	*L. infantum*	57	53	109
LinJ15_V3.1610	*L. infantum*	48	369	416
LinJ15_V3.1620	*L. infantum*	54	1	54
LinJ15_V3.1620	*L. infantum*	139	84	222
LinJ15_V3.1620	*L. infantum*	139	84	222
LinJ15_V3.1640	*L. infantum*	42	152	193
LinJ15_V3.1640	*L. infantum*	56	870	925
LinJ15_V3.1640	*L. infantum*	48	1209	1256
LinJ15_V3.1650	*L. infantum*	148	305	452
LinJ15_V3.1650	*L. infantum*	54	490	543
LinJ16_V30010	*L. infantum*	43	143	185
LinJ16_V30060	*L. infantum*	61	181	241
LinJ16_V30060	*L. infantum*	48	536	583
LinJ16_V30060	*L. infantum*	48	536	583
LinJ16_V30080	*L. infantum*	56	55	110
LinJ16_V30090	*L. infantum*	75	1	75
LinJ16_V30090	*L. infantum*	63	384	446
LinJ16_V30090	*L. infantum*	82	508	589
LinJ16_V30090	*L. infantum*	115	621	735
LinJ16_V30110	*L. infantum*	70	168	237
LinJ16_V30130	*L. infantum*	50	1	50
LinJ16_V30130	*L. infantum*	44	62	105
LinJ16_V30130	*L. infantum*	44	422	465
LinJ16_V30130	*L. infantum*	46	670	715
LinJ16_V30140	*L. infantum*	69	34	102
LinJ16_V30140	*L. infantum*	83	104	186
LinJ16_V30140	*L. infantum*	83	104	186
LinJ16_V30150	*L. infantum*	40	1	40
LinJ16_V30150	*L. infantum*	53	116	168
LinJ16_V30150	*L. infantum*	53	116	168
LinJ16_V30160	*L. infantum*	129	83	211
LinJ16_V30170	*L. infantum*	41	1	41
LinJ16_V30170	*L. infantum*	58	248	305

LinJ16_V30170	*L. infantum*	58	248	305
LinJ16_V30180	*L. infantum*	87	144	230
LinJ16_V30180	*L. infantum*	48	302	349
LinJ16_V30180	*L. infantum*	69	1180	1248
LinJ16_V30180	*L. infantum*	69	1283	1351
LinJ16_V30180	*L. infantum*	312	1507	1818
LinJ16_V30180	*L. infantum*	62	1918	1979
LinJ16_V30180	*L. infantum*	51	2310	2360
LinJ16_V30180	*L. infantum*	65	2400	2464
LinJ16_V30180	*L. infantum*	112	2732	2843
LinJ16_V30180	*L. infantum*	50	2906	2955
LinJ16_V30180	*L. infantum*	62	3045	3106
LinJ16_V30180	*L. infantum*	72	3338	3409
LinJ16_V30180	*L. infantum*	79	3446	3524
LinJ16_V30180	*L. infantum*	49	3548	3596
LinJ16_V30180	*L. infantum*	68	4272	4339
LinJ16_V30180	*L. infantum*	48	4393	4440
LinJ16_V30190	*L. infantum*	45	1	45
LinJ16_V30190	*L. infantum*	96	57	152
LinJ16_V30200	*L. infantum*	66	1	66
LinJ16_V30200	*L. infantum*	122	232	353
LinJ16_V30220	*L. infantum*	55	110	164
LinJ16_V30220	*L. infantum*	45	339	383
LinJ16_V30230	*L. infantum*	50	82	131
LinJ16_V30250	*L. infantum*	56	1	56
LinJ16_V30250	*L. infantum*	43	117	159
LinJ16_V30250	*L. infantum*	43	657	699
LinJ16_V30270	*L. infantum*	64	68	131
LinJ16_V30280	*L. infantum*	43	51	93
LinJ16_V30280	*L. infantum*	57	153	209
LinJ16_V30280	*L. infantum*	42	297	338
LinJ16_V30280	*L. infantum*	68	396	463
LinJ16_V30280	*L. infantum*	46	942	987
LinJ16_V30290	*L. infantum*	45	452	496
LinJ16_V30290	*L. infantum*	40	724	763
LinJ16_V30290	*L. infantum*	88	783	870
LinJ16_V30290	*L. infantum*	50	2133	2182
LinJ16_V30290	*L. infantum*	94	2781	2874
LinJ16_V30300	*L. infantum*	47	110	156
LinJ16_V30310	*L. infantum*	139	257	395
LinJ16_V30310	*L. infantum*	106	427	532
LinJ16_V30310	*L. infantum*	106	427	532
LinJ16_V30320	*L. infantum*	41	89	129
LinJ16_V30320	*L. infantum*	73	179	251
LinJ16_V30320	*L. infantum*	40	650	689
LinJ16_V30330	*L. infantum*	488	384	871
LinJ16_V30330	*L. infantum*	225	897	1121

LinJ16_V30340	*L. infantum*	66	1	66
LinJ16_V30340	*L. infantum*	47	142	188
LinJ16_V30340	*L. infantum*	47	142	188
LinJ16_V30360	*L. infantum*	72	1	72
LinJ16_V30370	*L. infantum*	70	133	202
LinJ16_V30370	*L. infantum*	41	210	250
LinJ16_V30370	*L. infantum*	41	360	400
LinJ16_V30380	*L. infantum*	115	64	178
LinJ16_V30390	*L. infantum*	191	1	191
LinJ16_V30390	*L. infantum*	51	197	247
LinJ16_V30390	*L. infantum*	61	267	327
LinJ16_V30390	*L. infantum*	85	515	599
LinJ16_V30400	*L. infantum*	62	41	102
LinJ16_V30400	*L. infantum*	65	251	315
LinJ16_V30400	*L. infantum*	291	710	1000
LinJ16_V30420	*L. infantum*	40	92	131
LinJ16_V30420	*L. infantum*	246	330	575
LinJ16_V30430	*L. infantum*	63	1	63
LinJ16_V30440	*L. infantum*	185	1	185
LinJ16_V30450	*L. infantum*	42	683	724
LinJ16_V30450	*L. infantum*	50	743	792
LinJ16_V30450	*L. infantum*	50	743	792
LinJ16_V30490	*L. infantum*	42	683	724
LinJ16_V30490	*L. infantum*	48	745	792
LinJ16_V30490	*L. infantum*	48	745	792
LinJ16_V30520	*L. infantum*	46	33	78
LinJ16_V30520	*L. infantum*	107	144	250
LinJ16_V30520	*L. infantum*	92	297	388
LinJ16_V30520	*L. infantum*	373	432	804
LinJ16_V30530	*L. infantum*	43	286	328
LinJ16_V30570	*L. infantum*	84	148	231
LinJ16_V30570	*L. infantum*	104	373	476
LinJ16_V30570	*L. infantum*	120	492	611
LinJ16_V30580	*L. infantum*	60	157	216
LinJ16_V30600	*L. infantum*	53	1	53
LinJ16_V30610	*L. infantum*	53	1	53
LinJ16_V30620	*L. infantum*	221	58	278
LinJ16_V30620	*L. infantum*	84	317	400
LinJ16_V30620	*L. infantum*	61	432	492
LinJ16_V30620	*L. infantum*	106	527	632
LinJ16_V30620	*L. infantum*	53	784	836
LinJ16_V30620	*L. infantum*	120	861	980
LinJ16_V30620	*L. infantum*	53	1052	1104
LinJ16_V30630	*L. infantum*	73	33	105
LinJ16_V30630	*L. infantum*	69	121	189
LinJ16_V30640	*L. infantum*	114	163	276
LinJ16_V30640	*L. infantum*	77	487	563

LinJ16_V30640	*L. infantum*	77	487	563
LinJ16_V30660	*L. infantum*	163	65	227
LinJ16_V30660	*L. infantum*	312	255	566
LinJ16_V30690	*L. infantum*	65	487	551
LinJ16_V30700	*L. infantum*	68	21	88
LinJ16_V30700	*L. infantum*	639	116	754
LinJ16_V30700	*L. infantum*	71	779	849
LinJ16_V30710	*L. infantum*	103	1	103
LinJ16_V30710	*L. infantum*	345	121	465
LinJ16_V30710	*L. infantum*	95	501	595
LinJ16_V30710	*L. infantum*	353	620	972
LinJ16_V30720	*L. infantum*	62	55	116
LinJ16_V30720	*L. infantum*	41	168	208
LinJ16_V30720	*L. infantum*	43	216	258
LinJ16_V30730	*L. infantum*	52	82	133
LinJ16_V30730	*L. infantum*	53	191	243
LinJ16_V30730	*L. infantum*	44	595	638
LinJ16_V30730	*L. infantum*	64	730	793
LinJ16_V30730	*L. infantum*	44	864	907
LinJ16_V30730	*L. infantum*	73	923	995
LinJ16_V30730	*L. infantum*	66	1028	1093
LinJ16_V30730	*L. infantum*	40	1166	1205
LinJ16_V30730	*L. infantum*	103	1244	1346
LinJ16_V30730	*L. infantum*	81	1505	1585
LinJ16_V30730	*L. infantum*	49	1719	1767
LinJ16_V30740	*L. infantum*	88	33	120
LinJ16_V30740	*L. infantum*	92	153	244
LinJ16_V30740	*L. infantum*	64	278	341
LinJ16_V30740	*L. infantum*	48	348	395
LinJ16_V30740	*L. infantum*	135	468	602
LinJ16_V30740	*L. infantum*	64	677	740
LinJ16_V30750	*L. infantum*	44	174	217
LinJ16_V30750	*L. infantum*	44	361	404
LinJ16_V30750	*L. infantum*	141	462	602
LinJ16_V30770	*L. infantum*	340	53	392
LinJ16_V30770	*L. infantum*	43	541	583
LinJ16_V30780	*L. infantum*	40	48	87
LinJ16_V30780	*L. infantum*	77	120	196
LinJ16_V30780	*L. infantum*	107	477	583
LinJ16_V30780	*L. infantum*	67	699	765
LinJ16_V30780	*L. infantum*	49	886	934
LinJ16_V30780	*L. infantum*	141	1029	1169
LinJ16_V30780	*L. infantum*	74	1267	1340
LinJ16_V30780	*L. infantum*	82	1432	1513
LinJ16_V30780	*L. infantum*	43	1583	1625
LinJ16_V30780	*L. infantum*	78	1655	1732
LinJ16_V30780	*L. infantum*	81	1770	1850

LinJ16_V30780	*L. infantum*	69	1917	1985
LinJ16_V30780	*L. infantum*	78	2121	2198
LinJ16_V30780	*L. infantum*	60	2397	2456
LinJ16_V30780	*L. infantum*	66	2494	2559
LinJ16_V30780	*L. infantum*	45	2604	2648
LinJ16_V30780	*L. infantum*	50	2879	2928
LinJ16_V30780	*L. infantum*	112	3042	3153
LinJ16_V30780	*L. infantum*	59	3425	3483
LinJ16_V30780	*L. infantum*	57	3782	3838
LinJ16_V30780	*L. infantum*	48	4433	4480
LinJ16_V30780	*L. infantum*	91	5280	5370
LinJ16_V30780	*L. infantum*	178	5394	5571
LinJ16_V30800	*L. infantum*	43	116	158
LinJ16_V30800	*L. infantum*	88	285	372
LinJ16_V30800	*L. infantum*	43	589	631
LinJ16_V30810	*L. infantum*	40	114	153
LinJ16_V30810	*L. infantum*	70	178	247
LinJ16_V30820	*L. infantum*	232	176	407
LinJ16_V30820	*L. infantum*	51	558	608
LinJ16_V30820	*L. infantum*	60	618	677
LinJ16_V30830	*L. infantum*	139	1	139
LinJ16_V30850	*L. infantum*	52	211	262
LinJ16_V30850	*L. infantum*	63	326	388
LinJ16_V30850	*L. infantum*	46	429	474
LinJ16_V30860	*L. infantum*	72	1	72
LinJ16_V30860	*L. infantum*	110	96	205
LinJ16_V30860	*L. infantum*	110	96	205
LinJ16_V30870	*L. infantum*	201	1	201
LinJ16_V30870	*L. infantum*	52	366	417
LinJ16_V30890	*L. infantum*	75	95	169
LinJ16_V30890	*L. infantum*	54	291	344
LinJ16_V30890	*L. infantum*	130	533	662
LinJ16_V30900	*L. infantum*	76	44	119
LinJ16_V30900	*L. infantum*	150	148	297
LinJ16_V30910	*L. infantum*	41	156	196
LinJ16_V30910	*L. infantum*	56	227	282
LinJ16_V30910	*L. infantum*	41	1093	1133
LinJ16_V30910	*L. infantum*	57	1502	1558
LinJ16_V30930	*L. infantum*	46	1	46
LinJ16_V30940	*L. infantum*	61	1	61
LinJ16_V30940	*L. infantum*	91	130	220
LinJ16_V30970	*L. infantum*	79	268	346
LinJ16_V30980	*L. infantum*	89	170	258
LinJ16_V30980	*L. infantum*	45	293	337
LinJ16_V30980	*L. infantum*	63	600	662
LinJ16_V30990	*L. infantum*	296	1	296
LinJ16_V30990	*L. infantum*	642	306	947

LinJ16_V31000	*L. infantum*	59	148	206
LinJ16_V31000	*L. infantum*	88	296	383
LinJ16_V31000	*L. infantum*	88	296	383
LinJ16_V31020	*L. infantum*	100	253	352
LinJ16_V31030	*L. infantum*	173	61	233
LinJ16_V31030	*L. infantum*	80	825	904
LinJ16_V31040	*L. infantum*	45	1	45
LinJ16_V31040	*L. infantum*	84	84	167
LinJ16_V31040	*L. infantum*	72	228	299
LinJ16_V31040	*L. infantum*	100	788	887
LinJ16_V31050	*L. infantum*	121	40	160
LinJ16_V31050	*L. infantum*	100	741	840
LinJ16_V31060	*L. infantum*	47	1	47
LinJ16_V31060	*L. infantum*	106	83	188
LinJ16_V31060	*L. infantum*	100	764	863
LinJ16_V31070	*L. infantum*	100	443	542
LinJ16_V31080	*L. infantum*	71	77	147
LinJ16_V31080	*L. infantum*	100	853	952
LinJ16_V31080	*L. infantum*	100	853	952
LinJ16_V31090	*L. infantum*	41	58	98
LinJ16_V31090	*L. infantum*	61	405	465
LinJ16_V31090	*L. infantum*	61	405	465
LinJ16_V31110	*L. infantum*	72	153	224
LinJ16_V31110	*L. infantum*	48	442	489
LinJ16_V31110	*L. infantum*	48	442	489
LinJ16_V31120	*L. infantum*	44	1	44
LinJ16_V31120	*L. infantum*	467	88	554
LinJ16_V31120	*L. infantum*	72	562	633
LinJ16_V31130	*L. infantum*	103	1	103
LinJ16_V31130	*L. infantum*	50	120	169
LinJ16_V31130	*L. infantum*	116	212	327
LinJ16_V31130	*L. infantum*	166	343	508
LinJ16_V31130	*L. infantum*	537	525	1061
LinJ16_V31130	*L. infantum*	91	1093	1183
LinJ16_V31130	*L. infantum*	90	1186	1275
LinJ16_V31130	*L. infantum*	563	1287	1849
LinJ16_V31130	*L. infantum*	45	1880	1924
LinJ16_V31140	*L. infantum*	56	65	120
LinJ16_V31140	*L. infantum*	399	161	559
LinJ16_V31150	*L. infantum*	125	1	125
LinJ16_V31180	*L. infantum*	165	215	379
LinJ16_V31180	*L. infantum*	106	405	510
LinJ16_V31180	*L. infantum*	48	513	560
LinJ16_V31180	*L. infantum*	74	562	635
LinJ16_V31210	*L. infantum*	92	127	218
LinJ16_V31210	*L. infantum*	68	272	339
LinJ16_V31230	*L. infantum*	145	139	283

LinJ16_V31240	*L. infantum*	102	144	245
LinJ16_V31240	*L. infantum*	56	427	482
LinJ16_V31240	*L. infantum*	80	538	617
LinJ16_V31240	*L. infantum*	95	1050	1144
LinJ16_V31240	*L. infantum*	218	1223	1440
LinJ16_V31250	*L. infantum*	123	244	366
LinJ16_V31260	*L. infantum*	169	53	221
LinJ16_V31260	*L. infantum*	52	265	316
LinJ16_V31260	*L. infantum*	154	332	485
LinJ16_V31260	*L. infantum*	48	872	919
LinJ16_V31260	*L. infantum*	72	982	1053
LinJ16_V31260	*L. infantum*	74	1203	1276
LinJ16_V31270	*L. infantum*	158	1	158
LinJ16_V31270	*L. infantum*	300	251	550
LinJ16_V31270	*L. infantum*	249	553	801
LinJ16_V31270	*L. infantum*	246	902	1147
LinJ16_V31270	*L. infantum*	59	1303	1361
LinJ16_V31270	*L. infantum*	180	1386	1565
LinJ16_V31270	*L. infantum*	40	1674	1713
LinJ16_V31270	*L. infantum*	53	2059	2111
LinJ16_V31280	*L. infantum*	77	1	77
LinJ16_V31280	*L. infantum*	42	442	483
LinJ16_V31280	*L. infantum*	43	540	582
LinJ16_V31280	*L. infantum*	57	1320	1376
LinJ16_V31280	*L. infantum*	170	1645	1814
LinJ16_V31290	*L. infantum*	143	64	206
LinJ16_V31290	*L. infantum*	119	238	356
LinJ16_V31290	*L. infantum*	272	390	661
LinJ16_V31290	*L. infantum*	48	770	817
LinJ16_V31290	*L. infantum*	115	915	1029
LinJ16_V31290	*L. infantum*	56	1134	1189
LinJ16_V31290	*L. infantum*	401	1221	1621
LinJ16_V31290	*L. infantum*	51	1859	1909
LinJ16_V31290	*L. infantum*	116	2015	2130
LinJ16_V31310	*L. infantum*	217	10	226
LinJ16_V31310	*L. infantum*	46	239	284
LinJ16_V31310	*L. infantum*	40	597	636
LinJ16_V31310	*L. infantum*	61	671	731
LinJ16_V31310	*L. infantum*	107	755	861
LinJ16_V31310	*L. infantum*	52	978	1029
LinJ16_V31310	*L. infantum*	102	1267	1368
LinJ16_V31310	*L. infantum*	58	1415	1472
LinJ16_V31310	*L. infantum*	46	1605	1650
LinJ16_V31320	*L. infantum*	248	1	248
LinJ16_V31320	*L. infantum*	194	281	474
LinJ16_V31320	*L. infantum*	144	625	768
LinJ16_V31320	*L. infantum*	40	937	976

LinJ16_V31320	*L. infantum*	584	1063	1646
LinJ16_V31320	*L. infantum*	59	1758	1816
LinJ16_V31320	*L. infantum*	162	1920	2081
LinJ16_V31320	*L. infantum*	75	2101	2175
LinJ16_V31320	*L. infantum*	40	2194	2233
LinJ16_V31340	*L. infantum*	145	1	145
LinJ16_V31340	*L. infantum*	49	373	421
LinJ16_V31340	*L. infantum*	43	676	718
LinJ16_V31360	*L. infantum*	186	50	235
LinJ16_V31360	*L. infantum*	89	286	374
LinJ16_V31360	*L. infantum*	109	504	612
LinJ16_V31360	*L. infantum*	43	798	840
LinJ16_V31360	*L. infantum*	67	870	936
LinJ16_V31380	*L. infantum*	97	1	97
LinJ16_V31400	*L. infantum*	715	1	715
LinJ16_V31400	*L. infantum*	86	1144	1229
LinJ16_V31400	*L. infantum*	70	1334	1403
LinJ16_V31420	*L. infantum*	49	218	266
LinJ16_V31420	*L. infantum*	40	884	923
LinJ16_V31420	*L. infantum*	54	1109	1162
LinJ16_V31420	*L. infantum*	167	1385	1551
LinJ16_V31460	*L. infantum*	131	1	131
LinJ16_V31460	*L. infantum*	235	140	374
LinJ16_V31460	*L. infantum*	148	511	658
LinJ16_V31460	*L. infantum*	180	688	867
LinJ16_V31470	*L. infantum*	41	186	226
LinJ16_V31490	*L. infantum*	77	64	140
LinJ16_V31490	*L. infantum*	154	329	482
LinJ16_V31500	*L. infantum*	72	89	160
LinJ16_V31510	*L. infantum*	120	259	378
LinJ16_V31510	*L. infantum*	44	485	528
LinJ16_V31510	*L. infantum*	44	485	528
LinJ16_V31520	*L. infantum*	120	104	223
LinJ16_V31520	*L. infantum*	44	330	373
LinJ16_V31520	*L. infantum*	44	330	373
LinJ16_V31530	*L. infantum*	55	16	70
LinJ16_V31530	*L. infantum*	59	350	408
LinJ16_V31530	*L. infantum*	77	604	680
LinJ16_V31530	*L. infantum*	51	748	798
LinJ16_V31530	*L. infantum*	150	995	1144
LinJ16_V31530	*L. infantum*	60	1258	1317
LinJ16_V31540	*L. infantum*	70	44	113
LinJ16_V31540	*L. infantum*	132	115	246
LinJ16_V31550	*L. infantum*	114	1	114
LinJ16_V31550	*L. infantum*	2098	561	2658
LinJ16_V31550	*L. infantum*	50	2762	2811
LinJ16_V31570	*L. infantum*	194	455	648

LinJ16_V31570	*L. infantum*	42	752	793
LinJ16_V31580	*L. infantum*	77	1	77
LinJ16_V31580	*L. infantum*	93	271	363
LinJ16_V31580	*L. infantum*	93	271	363
LinJ16_V31590	*L. infantum*	59	268	326
LinJ16_V31600	*L. infantum*	40	24	63
LinJ16_V31600	*L. infantum*	42	156	197
LinJ16_V31600	*L. infantum*	74	340	413
LinJ16_V31600	*L. infantum*	41	530	570
LinJ16_V31620	*L. infantum*	79	240	318
LinJ16_V31620	*L. infantum*	139	698	836
LinJ16_V31620	*L. infantum*	154	997	1150
LinJ16_V31640	*L. infantum*	214	1	214
LinJ16_V31650	*L. infantum*	449	706	1154
LinJ16_V31660	*L. infantum*	57	285	341
LinJ16_V31660	*L. infantum*	78	615	692
LinJ16_V31660	*L. infantum*	78	615	692
LinJ16_V31670	*L. infantum*	135	142	276
LinJ16_V31680	*L. infantum*	45	243	287
LinJ16_V31680	*L. infantum*	110	476	585
LinJ16_V31680	*L. infantum*	99	648	746
LinJ16_V31690	*L. infantum*	78	1	78
LinJ16_V31690	*L. infantum*	68	156	223
LinJ16_V31690	*L. infantum*	41	326	366
LinJ16_V31700	*L. infantum*	42	149	190
LinJ16_V31700	*L. infantum*	134	259	392
LinJ16_V31720	*L. infantum*	53	200	252
LinJ16_V31720	*L. infantum*	45	320	364
LinJ16_V31720	*L. infantum*	45	320	364
LinJ16_V31730	*L. infantum*	51	71	121
LinJ16_V31730	*L. infantum*	88	152	239
LinJ16_V31730	*L. infantum*	88	152	239
LinJ16_V31740	*L. infantum*	40	292	331
LinJ16_V31760	*L. infantum*	63	1	63
LinJ16_V31760	*L. infantum*	41	843	883
LinJ16_V31760	*L. infantum*	51	1119	1169
LinJ16_V31760	*L. infantum*	47	1454	1500
LinJ16_V31760	*L. infantum*	202	1625	1826
LinJ16_V31760	*L. infantum*	40	2154	2193
LinJ16_V31760	*L. infantum*	40	2227	2266
LinJ16_V31760	*L. infantum*	360	2496	2855
LinJ17_V3.0010	*L. infantum*	111	516	626
LinJ17_V3.0010	*L. infantum*	100	675	774
LinJ17_V3.0010	*L. infantum*	100	675	774
LinJ17_V3.0040	*L. infantum*	68	120	187
LinJ17_V3.0060	*L. infantum*	46	158	203
LinJ17_V3.0070	*L. infantum*	74	480	553

LinJ17_V3.0110	*L. infantum*	57	429	485
LinJ17_V3.0120	*L. infantum*	118	1248	1365
LinJ17_V3.0130	*L. infantum*	125	1198	1322
LinJ17_V3.0140	*L. infantum*	57	1283	1339
LinJ17_V3.0140	*L. infantum*	50	1350	1399
LinJ17_V3.0140	*L. infantum*	50	1350	1399
LinJ17_V3.0180	*L. infantum*	74	162	235
LinJ17_V3.0210	*L. infantum*	56	1	56
LinJ17_V3.0210	*L. infantum*	43	469	511
LinJ17_V3.0210	*L. infantum*	92	520	611
LinJ17_V3.0220	*L. infantum*	134	1	134
LinJ17_V3.0220	*L. infantum*	368	159	526
LinJ17_V3.0240	*L. infantum*	51	37	87
LinJ17_V3.0240	*L. infantum*	184	655	838
LinJ17_V3.0260	*L. infantum*	54	1	54
LinJ17_V3.0260	*L. infantum*	55	87	141
LinJ17_V3.0260	*L. infantum*	55	87	141
LinJ17_V3.0270	*L. infantum*	40	48	87
LinJ17_V3.0270	*L. infantum*	59	213	271
LinJ17_V3.0270	*L. infantum*	85	332	416
LinJ17_V3.0270	*L. infantum*	126	454	579
LinJ17_V3.0270	*L. infantum*	304	630	933
LinJ17_V3.0270	*L. infantum*	53	983	1035
LinJ17_V3.0280	*L. infantum*	159	1	159
LinJ17_V3.0290	*L. infantum*	131	1	131
LinJ17_V3.0290	*L. infantum*	209	186	394
LinJ17_V3.0290	*L. infantum*	92	875	966
LinJ17_V3.0290	*L. infantum*	157	1016	1172
LinJ17_V3.0310	*L. infantum*	178	29	206
LinJ17_V3.0310	*L. infantum*	546	240	785
LinJ17_V3.0320	*L. infantum*	51	223	273
LinJ17_V3.0330	*L. infantum*	75	1	75
LinJ17_V3.0340	*L. infantum*	282	60	341
LinJ17_V3.0340	*L. infantum*	41	685	725
LinJ17_V3.0350	*L. infantum*	41	1	41
LinJ17_V3.0350	*L. infantum*	60	144	203
LinJ17_V3.0350	*L. infantum*	60	144	203
LinJ17_V3.0360	*L. infantum*	145	1	145
LinJ17_V3.0360	*L. infantum*	61	213	273
LinJ17_V3.0360	*L. infantum*	975	375	1349
LinJ17_V3.0370	*L. infantum*	82	1	82
LinJ17_V3.0370	*L. infantum*	153	175	327
LinJ17_V3.0380	*L. infantum*	53	319	371
LinJ17_V3.0380	*L. infantum*	63	749	811
LinJ17_V3.0380	*L. infantum*	49	850	898
LinJ17_V3.0380	*L. infantum*	44	1084	1127
LinJ17_V3.0380	*L. infantum*	48	1334	1381

LinJ17_V3.0400	*L. infantum*	230	1	230
LinJ17_V3.0420	*L. infantum*	91	1	91
LinJ17_V3.0420	*L. infantum*	51	188	238
LinJ17_V3.0420	*L. infantum*	166	245	410
LinJ17_V3.0420	*L. infantum*	140	417	556
LinJ17_V3.0420	*L. infantum*	72	565	636
LinJ17_V3.0430	*L. infantum*	45	23	67
LinJ17_V3.0430	*L. infantum*	147	87	233
LinJ17_V3.0440	*L. infantum*	56	40	95
LinJ17_V3.0440	*L. infantum*	42	214	255
LinJ17_V3.0440	*L. infantum*	82	537	618
LinJ17_V3.0440	*L. infantum*	105	691	795
LinJ17_V3.0440	*L. infantum*	52	1066	1117
LinJ17_V3.0450	*L. infantum*	207	39	245
LinJ17_V3.0450	*L. infantum*	68	307	374
LinJ17_V3.0450	*L. infantum*	85	481	565
LinJ17_V3.0460	*L. infantum*	129	136	264
LinJ17_V3.0460	*L. infantum*	390	380	769
LinJ17_V3.0470	*L. infantum*	55	229	283
LinJ17_V3.0470	*L. infantum*	126	358	483
LinJ17_V3.0470	*L. infantum*	126	358	483
LinJ17_V3.0480	*L. infantum*	933	1	933
LinJ17_V3.0480	*L. infantum*	142	985	1126
LinJ17_V3.0480	*L. infantum*	58	1175	1232
LinJ17_V3.0490	*L. infantum*	183	40	222
LinJ17_V3.0490	*L. infantum*	57	274	330
LinJ17_V3.0490	*L. infantum*	281	385	665
LinJ17_V3.0490	*L. infantum*	559	687	1245
LinJ17_V3.0490	*L. infantum*	194	1319	1512
LinJ17_V3.0490	*L. infantum*	88	1664	1751
LinJ17_V3.0490	*L. infantum*	59	1805	1863
LinJ17_V3.0500	*L. infantum*	58	1	58
LinJ17_V3.0500	*L. infantum*	162	93	254
LinJ17_V3.0500	*L. infantum*	72	357	428
LinJ17_V3.0500	*L. infantum*	71	471	541
LinJ17_V3.0500	*L. infantum*	64	593	656
LinJ17_V3.0500	*L. infantum*	228	736	963
LinJ17_V3.0500	*L. infantum*	70	1311	1380
LinJ17_V3.0510	*L. infantum*	134	1	134
LinJ17_V3.0530	*L. infantum*	57	136	192
LinJ17_V3.0530	*L. infantum*	40	241	280
LinJ17_V3.0530	*L. infantum*	147	307	453
LinJ17_V3.0530	*L. infantum*	66	484	549
LinJ17_V3.0530	*L. infantum*	58	690	747
LinJ17_V3.0530	*L. infantum*	75	830	904
LinJ17_V3.0530	*L. infantum*	105	1204	1308
LinJ17_V3.0530	*L. infantum*	49	1496	1544

LinJ17_V3.0530	*L. infantum*	68	1973	2040
LinJ17_V3.0530	*L. infantum*	52	2082	2133
LinJ17_V3.0540	*L. infantum*	42	45	86
LinJ17_V3.0540	*L. infantum*	112	119	230
LinJ17_V3.0550	*L. infantum*	259	1	259
LinJ17_V3.0550	*L. infantum*	102	332	433
LinJ17_V3.0550	*L. infantum*	69	450	518
LinJ17_V3.0550	*L. infantum*	46	530	575
LinJ17_V3.0550	*L. infantum*	40	746	785
LinJ17_V3.0560	*L. infantum*	364	1	364
LinJ17_V3.0560	*L. infantum*	245	400	644
LinJ17_V3.0560	*L. infantum*	68	680	747
LinJ17_V3.0560	*L. infantum*	100	815	914
LinJ17_V3.0560	*L. infantum*	237	922	1158
LinJ17_V3.0560	*L. infantum*	82	1212	1293
LinJ17_V3.0560	*L. infantum*	128	1299	1426
LinJ17_V3.0570	*L. infantum*	741	1	741
LinJ17_V3.0570	*L. infantum*	264	767	1030
LinJ17_V3.0580	*L. infantum*	580	1	580
LinJ17_V3.0580	*L. infantum*	121	588	708
LinJ17_V3.0580	*L. infantum*	121	726	846
LinJ17_V3.0590	*L. infantum*	96	1	96
LinJ17_V3.0590	*L. infantum*	48	114	161
LinJ17_V3.0590	*L. infantum*	89	169	257
LinJ17_V3.0590	*L. infantum*	289	304	592
LinJ17_V3.0590	*L. infantum*	258	614	871
LinJ17_V3.0590	*L. infantum*	74	902	975
LinJ17_V3.0600	*L. infantum*	119	1	119
LinJ17_V3.0600	*L. infantum*	196	133	328
LinJ17_V3.0600	*L. infantum*	63	444	506
LinJ17_V3.0600	*L. infantum*	70	595	664
LinJ17_V3.0600	*L. infantum*	50	709	758
LinJ17_V3.0600	*L. infantum*	72	915	986
LinJ17_V3.0600	*L. infantum*	269	1002	1270
LinJ17_V3.0610	*L. infantum*	162	55	216
LinJ17_V3.0620	*L. infantum*	56	65	120
LinJ17_V3.0630	*L. infantum*	151	17	167
LinJ17_V3.0630	*L. infantum*	626	210	835
LinJ17_V3.0630	*L. infantum*	57	860	916
LinJ17_V3.0630	*L. infantum*	40	948	987
LinJ17_V3.0630	*L. infantum*	121	1025	1145
LinJ17_V3.0640	*L. infantum*	63	190	252
LinJ17_V3.0650	*L. infantum*	70	94	163
LinJ17_V3.0650	*L. infantum*	47	318	364
LinJ17_V3.0650	*L. infantum*	56	385	440
LinJ17_V3.0650	*L. infantum*	91	571	661
LinJ17_V3.0650	*L. infantum*	175	707	881

LinJ17_V3.0650	*L. infantum*	44	894	937
LinJ17_V3.0650	*L. infantum*	45	1003	1047
LinJ17_V3.0650	*L. infantum*	415	1080	1494
LinJ17_V3.0650	*L. infantum*	135	1503	1637
LinJ17_V3.0660	*L. infantum*	59	1	59
LinJ17_V3.0670	*L. infantum*	240	260	499
LinJ17_V3.0710	*L. infantum*	95	63	157
LinJ17_V3.0710	*L. infantum*	60	200	259
LinJ17_V3.0710	*L. infantum*	61	373	433
LinJ17_V3.0720	*L. infantum*	46	277	322
LinJ17_V3.0730	*L. infantum*	130	41	170
LinJ17_V3.0730	*L. infantum*	362	213	574
LinJ17_V3.0740	*L. infantum*	41	211	251
LinJ17_V3.0750	*L. infantum*	115	82	196
LinJ17_V3.0750	*L. infantum*	51	302	352
LinJ17_V3.0760	*L. infantum*	164	1	164
LinJ17_V3.0770	*L. infantum*	43	1	43
LinJ17_V3.0770	*L. infantum*	219	272	490
LinJ17_V3.0790	*L. infantum*	106	1	106
LinJ17_V3.0790	*L. infantum*	147	208	354
LinJ17_V3.0790	*L. infantum*	51	586	636
LinJ17_V3.0790	*L. infantum*	58	659	716
LinJ17_V3.0790	*L. infantum*	76	820	895
LinJ17_V3.0790	*L. infantum*	94	1042	1135
LinJ17_V3.0800	*L. infantum*	218	368	585
LinJ17_V3.0800	*L. infantum*	66	666	731
LinJ17_V3.0800	*L. infantum*	132	793	924
LinJ17_V3.0800	*L. infantum*	49	1032	1080
LinJ17_V3.0800	*L. infantum*	40	1188	1227
LinJ17_V3.0810	*L. infantum*	460	1	460
LinJ17_V3.0810	*L. infantum*	238	476	713
LinJ17_V3.0810	*L. infantum*	41	751	791
LinJ17_V3.0810	*L. infantum*	289	1034	1322
LinJ17_V3.0810	*L. infantum*	51	1423	1473
LinJ17_V3.0810	*L. infantum*	47	1490	1536
LinJ17_V3.0810	*L. infantum*	137	1608	1744
LinJ17_V3.0810	*L. infantum*	49	1790	1838
LinJ17_V3.0820	*L. infantum*	93	352	444
LinJ17_V3.0830	*L. infantum*	72	97	168
LinJ17_V3.0830	*L. infantum*	233	183	415
LinJ17_V3.0830	*L. infantum*	89	417	505
LinJ17_V3.0840	*L. infantum*	73	404	476
LinJ17_V3.0850	*L. infantum*	443	112	554
LinJ17_V3.0850	*L. infantum*	74	574	647
LinJ17_V3.0850	*L. infantum*	54	755	808
LinJ17_V3.0860	*L. infantum*	113	1	113
LinJ17_V3.0860	*L. infantum*	46	265	310

LinJ17_V3.0860	*L. infantum*	158	373	530
LinJ17_V3.0860	*L. infantum*	56	592	647
LinJ17_V3.0890	*L. infantum*	68	197	264
LinJ17_V3.0890	*L. infantum*	142	315	456
LinJ17_V3.0890	*L. infantum*	76	494	569
LinJ17_V3.0890	*L. infantum*	348	593	940
LinJ17_V3.0900	*L. infantum*	104	1	104
LinJ17_V3.0900	*L. infantum*	98	175	272
LinJ17_V3.0900	*L. infantum*	74	314	387
LinJ17_V3.0900	*L. infantum*	51	521	571
LinJ17_V3.0910	*L. infantum*	183	25	207
LinJ17_V3.0920	*L. infantum*	152	1	152
LinJ17_V3.0920	*L. infantum*	58	157	214
LinJ17_V3.0920	*L. infantum*	113	236	348
LinJ17_V3.0920	*L. infantum*	180	366	545
LinJ17_V3.0930	*L. infantum*	45	1	45
LinJ17_V3.0930	*L. infantum*	185	437	621
LinJ17_V3.0940	*L. infantum*	118	1	118
LinJ17_V3.0940	*L. infantum*	58	177	234
LinJ17_V3.0950	*L. infantum*	77	1	77
LinJ17_V3.0960	*L. infantum*	120	25	144
LinJ17_V3.1000	*L. infantum*	51	1	51
LinJ17_V3.1000	*L. infantum*	153	449	601
LinJ17_V3.1000	*L. infantum*	40	635	674
LinJ17_V3.1020	*L. infantum*	49	51	99
LinJ17_V3.1030	*L. infantum*	42	1174	1215
LinJ17_V3.1040	*L. infantum*	125	1	125
LinJ17_V3.1040	*L. infantum*	117	224	340
LinJ17_V3.1040	*L. infantum*	495	372	866
LinJ17_V3.1040	*L. infantum*	261	1176	1436
LinJ17_V3.1050	*L. infantum*	83	46	128
LinJ17_V3.1050	*L. infantum*	59	145	203
LinJ17_V3.1050	*L. infantum*	77	246	322
LinJ17_V3.1050	*L. infantum*	49	428	476
LinJ17_V3.1050	*L. infantum*	79	539	617
LinJ17_V3.1050	*L. infantum*	150	635	784
LinJ17_V3.1050	*L. infantum*	48	854	901
LinJ17_V3.1050	*L. infantum*	51	943	993
LinJ17_V3.1060	*L. infantum*	454	1	454
LinJ17_V3.1060	*L. infantum*	290	484	773
LinJ17_V3.1060	*L. infantum*	129	815	943
LinJ17_V3.1060	*L. infantum*	154	1296	1449
LinJ17_V3.1070	*L. infantum*	547	1	547
LinJ17_V3.1070	*L. infantum*	65	603	667
LinJ17_V3.1070	*L. infantum*	400	855	1254
LinJ17_V3.1070	*L. infantum*	53	1368	1420
LinJ17_V3.1080	*L. infantum*	47	155	201

LinJ17_V3.1080	*L. infantum*	57	633	689
LinJ17_V3.1080	*L. infantum*	57	633	689
LinJ17_V3.1090	*L. infantum*	265	17	281
LinJ17_V3.1090	*L. infantum*	229	322	550
LinJ17_V3.1090	*L. infantum*	65	600	664
LinJ17_V3.1090	*L. infantum*	97	700	796
LinJ17_V3.1090	*L. infantum*	385	1029	1413
LinJ17_V3.1090	*L. infantum*	64	1476	1539
LinJ17_V3.1090	*L. infantum*	183	1551	1733
LinJ17_V3.1090	*L. infantum*	130	1778	1907
LinJ17_V3.1090	*L. infantum*	101	1949	2049
LinJ17_V3.1090	*L. infantum*	40	2124	2163
LinJ17_V3.1090	*L. infantum*	117	2245	2361
LinJ17_V3.1090	*L. infantum*	115	2464	2578
LinJ17_V3.1090	*L. infantum*	58	2868	2925
LinJ17_V3.1090	*L. infantum*	100	3123	3222
LinJ17_V3.1090	*L. infantum*	57	3257	3313
LinJ17_V3.1090	*L. infantum*	104	3370	3473
LinJ17_V3.1090	*L. infantum*	85	3658	3742
LinJ17_V3.1090	*L. infantum*	565	3757	4321
LinJ17_V3.1090	*L. infantum*	51	4516	4566
LinJ17_V3.1090	*L. infantum*	46	4587	4632
LinJ17_V3.1090	*L. infantum*	41	4665	4705
LinJ17_V3.1090	*L. infantum*	66	4744	4809
LinJ17_V3.1090	*L. infantum*	57	5063	5119
LinJ17_V3.1090	*L. infantum*	166	5148	5313
LinJ17_V3.1090	*L. infantum*	141	5446	5586
LinJ17_V3.1090	*L. infantum*	171	5656	5826
LinJ17_V3.1090	*L. infantum*	64	5863	5926
LinJ17_V3.1090	*L. infantum*	113	5962	6074
LinJ17_V3.1090	*L. infantum*	53	6120	6172
LinJ17_V3.1090	*L. infantum*	71	6392	6462
LinJ17_V3.1090	*L. infantum*	58	6484	6541
LinJ17_V3.1090	*L. infantum*	261	6719	6979
LinJ17_V3.1100	*L. infantum*	66	1	66
LinJ17_V3.1100	*L. infantum*	118	241	358
LinJ17_V3.1100	*L. infantum*	63	501	563
LinJ17_V3.1120	*L. infantum*	44	292	335
LinJ17_V3.1130	*L. infantum*	299	1	299
LinJ17_V3.1130	*L. infantum*	178	334	511
LinJ17_V3.1130	*L. infantum*	41	772	812
LinJ17_V3.1140	*L. infantum*	41	201	241
LinJ17_V3.1150	*L. infantum*	52	1	52
LinJ17_V3.1160	*L. infantum*	76	1	76
LinJ17_V3.1160	*L. infantum*	66	375	440
LinJ17_V3.1160	*L. infantum*	152	493	644
LinJ17_V3.1160	*L. infantum*	123	672	794

LinJ17_V3.1170	*L. infantum*	110	1	110
LinJ17_V3.1170	*L. infantum*	47	113	159
LinJ17_V3.1170	*L. infantum*	102	274	375
LinJ17_V3.1180	*L. infantum*	143	1	143
LinJ17_V3.1180	*L. infantum*	61	145	205
LinJ17_V3.1180	*L. infantum*	45	346	390
LinJ17_V3.1180	*L. infantum*	65	556	620
LinJ17_V3.1180	*L. infantum*	62	671	732
LinJ17_V3.1190	*L. infantum*	85	76	160
LinJ17_V3.1190	*L. infantum*	64	200	263
LinJ17_V3.1190	*L. infantum*	50	345	394
LinJ17_V3.1190	*L. infantum*	59	472	530
LinJ17_V3.1200	*L. infantum*	44	360	403
LinJ17_V3.1210	*L. infantum*	65	365	429
LinJ17_V3.1210	*L. infantum*	228	1151	1378
LinJ17_V3.1230	*L. infantum*	79	1	79
LinJ17_V3.1230	*L. infantum*	132	135	266
LinJ17_V3.1230	*L. infantum*	40	612	651
LinJ17_V3.1230	*L. infantum*	57	861	917
LinJ17_V3.1230	*L. infantum*	100	1076	1175
LinJ17_V3.1240	*L. infantum*	832	93	924
LinJ17_V3.1240	*L. infantum*	422	976	1397
LinJ17_V3.1250	*L. infantum*	55	167	221
LinJ17_V3.1250	*L. infantum*	100	352	451
LinJ17_V3.1250	*L. infantum*	54	508	561
LinJ17_V3.1250	*L. infantum*	40	741	780
LinJ17_V3.1250	*L. infantum*	104	825	928
LinJ17_V3.1250	*L. infantum*	67	946	1012
LinJ17_V3.1270	*L. infantum*	57	35	91
LinJ17_V3.1270	*L. infantum*	104	136	239
LinJ17_V3.1270	*L. infantum*	178	264	441
LinJ17_V3.1270	*L. infantum*	107	492	598
LinJ17_V3.1280	*L. infantum*	62	1	62
LinJ17_V3.1280	*L. infantum*	343	202	544
LinJ17_V3.1280	*L. infantum*	209	557	765
LinJ17_V3.1290	*L. infantum*	53	1	53
LinJ17_V3.1290	*L. infantum*	192	90	281
LinJ17_V3.1290	*L. infantum*	124	333	456
LinJ17_V3.1300	*L. infantum*	283	1	283
LinJ17_V3.1300	*L. infantum*	112	300	411
LinJ17_V3.1300	*L. infantum*	427	429	855
LinJ17_V3.1310	*L. infantum*	45	1	45
LinJ17_V3.1310	*L. infantum*	56	189	244
LinJ17_V3.1310	*L. infantum*	156	300	455
LinJ17_V3.1320	*L. infantum*	41	1	41
LinJ17_V3.1330	*L. infantum*	43	56	98
LinJ17_V3.1330	*L. infantum*	96	503	598

LinJ17_V3.1330	*L. infantum*	180	778	957
LinJ17_V3.1340	*L. infantum*	47	101	147
LinJ17_V3.1340	*L. infantum*	68	278	345
LinJ17_V3.1340	*L. infantum*	43	730	772
LinJ17_V3.1340	*L. infantum*	42	859	900
LinJ17_V3.1350	*L. infantum*	42	422	463
LinJ17_V3.1350	*L. infantum*	48	660	707
LinJ17_V3.1350	*L. infantum*	50	956	1005
LinJ17_V3.1360	*L. infantum*	43	284	326
LinJ17_V3.1360	*L. infantum*	94	358	451
LinJ17_V3.1390	*L. infantum*	73	600	672
LinJ17_V3.1410	*L. infantum*	50	102	151
LinJ17_V3.1410	*L. infantum*	44	163	206
LinJ17_V3.1410	*L. infantum*	124	523	646
LinJ17_V3.1410	*L. infantum*	45	812	856
LinJ17_V3.1420	*L. infantum*	44	77	120
LinJ17_V3.1430	*L. infantum*	310	1	310
LinJ17_V3.1460	*L. infantum*	46	40	85
LinJ17_V3.1470	*L. infantum*	48	236	283
LinJ17_V3.1480	*L. infantum*	71	229	299
LinJ17_V3.1480	*L. infantum*	382	493	874
LinJ17_V3.1500	*L. infantum*	69	106	174
LinJ17_V3.1500	*L. infantum*	45	192	236
LinJ17_V3.1500	*L. infantum*	78	283	360
LinJ17_V3.1500	*L. infantum*	75	366	440
LinJ17_V3.1500	*L. infantum*	54	730	783
LinJ17_V3.1530	*L. infantum*	43	1	43
LinJ17_V3.1530	*L. infantum*	73	425	497
LinJ17_V3.1540	*L. infantum*	210	48	257
LinJ17_V3.1550	*L. infantum*	58	1	58
LinJ17_V3.1560	*L. infantum*	65	202	266
LinJ17_V3.1560	*L. infantum*	57	565	621
LinJ17_V3.1570	*L. infantum*	72	71	142
LinJ17_V3.1590	*L. infantum*	193	51	243
LinJ18_V3.0010	*L. infantum*	60	682	741
LinJ18_V3.0030	*L. infantum*	66	87	152
LinJ18_V3.0050	*L. infantum*	42	228	269
LinJ18_V3.0050	*L. infantum*	72	706	777
LinJ18_V3.0050	*L. infantum*	72	706	777
LinJ18_V3.0060	*L. infantum*	43	115	157
LinJ18_V3.0070	*L. infantum*	53	109	161
LinJ18_V3.0070	*L. infantum*	157	186	342
LinJ18_V3.0070	*L. infantum*	117	352	468
LinJ18_V3.0070	*L. infantum*	231	494	724
LinJ18_V3.0080	*L. infantum*	40	404	443
LinJ18_V3.0100	*L. infantum*	89	126	214
LinJ18_V3.0110	*L. infantum*	45	544	588

LinJ18_V3.0130	*L. infantum*	64	356	419
LinJ18_V3.0130	*L. infantum*	54	650	703
LinJ18_V3.0140	*L. infantum*	45	544	588
LinJ18_V3.0140	*L. infantum*	49	697	745
LinJ18_V3.0140	*L. infantum*	49	697	745
LinJ18_V3.0170	*L. infantum*	141	207	347
LinJ18_V3.0180	*L. infantum*	72	215	286
LinJ18_V3.0190	*L. infantum*	43	197	239
LinJ18_V3.0190	*L. infantum*	46	254	299
LinJ18_V3.0190	*L. infantum*	46	254	299
LinJ18_V3.0210	*L. infantum*	66	168	233
LinJ18_V3.0220	*L. infantum*	329	81	409
LinJ18_V3.0230	*L. infantum*	112	1	112
LinJ18_V3.0240	*L. infantum*	48	279	326
LinJ18_V3.0250	*L. infantum*	67	1	67
LinJ18_V3.0250	*L. infantum*	45	98	142
LinJ18_V3.0250	*L. infantum*	45	98	142
LinJ18_V3.0270	*L. infantum*	43	1	43
LinJ18_V3.0280	*L. infantum*	47	1	47
LinJ18_V3.0280	*L. infantum*	55	237	291
LinJ18_V3.0280	*L. infantum*	55	237	291
LinJ18_V3.0290	*L. infantum*	40	26	65
LinJ18_V3.0300	*L. infantum*	141	135	275
LinJ18_V3.0330	*L. infantum*	78	100	177
LinJ18_V3.0340	*L. infantum*	48	101	148
LinJ18_V3.0340	*L. infantum*	65	289	353
LinJ18_V3.0340	*L. infantum*	65	289	353
LinJ18_V3.0350	*L. infantum*	43	1	43
LinJ18_V3.0370	*L. infantum*	44	167	210
LinJ18_V3.0380	*L. infantum*	72	304	375
LinJ18_V3.0380	*L. infantum*	43	846	888
LinJ18_V3.0380	*L. infantum*	43	846	888
LinJ18_V3.0390	*L. infantum*	124	47	170
LinJ18_V3.0390	*L. infantum*	205	212	416
LinJ18_V3.0390	*L. infantum*	304	418	721
LinJ18_V3.0390	*L. infantum*	471	725	1195
LinJ18_V3.0390	*L. infantum*	170	1224	1393
LinJ18_V3.0390	*L. infantum*	187	1560	1746
LinJ18_V3.0400	*L. infantum*	114	104	217
LinJ18_V3.0400	*L. infantum*	40	339	378
LinJ18_V3.0410	*L. infantum*	88	57	144
LinJ18_V3.0420	*L. infantum*	56	43	98
LinJ18_V3.0430	*L. infantum*	88	282	369
LinJ18_V3.0440	*L. infantum*	124	107	230
LinJ18_V3.0460	*L. infantum*	60	95	154
LinJ18_V3.0470	*L. infantum*	63	1	63
LinJ18_V3.0470	*L. infantum*	207	127	333

LinJ18_V3.0480	*L. infantum*	149	4	152
LinJ18_V3.0490	*L. infantum*	81	151	231
LinJ18_V3.0500	*L. infantum*	92	1	92
LinJ18_V3.0500	*L. infantum*	46	150	195
LinJ18_V3.0500	*L. infantum*	80	301	380
LinJ18_V3.0510	*L. infantum*	50	384	433
LinJ18_V3.0520	*L. infantum*	368	1	368
LinJ18_V3.0520	*L. infantum*	366	380	745
LinJ18_V3.0530	*L. infantum*	50	127	176
LinJ18_V3.0530	*L. infantum*	135	391	525
LinJ18_V3.0540	*L. infantum*	95	419	513
LinJ18_V3.0550	*L. infantum*	131	200	330
LinJ18_V3.0570	*L. infantum*	50	558	607
LinJ18_V3.0570	*L. infantum*	47	638	684
LinJ18_V3.0570	*L. infantum*	47	638	684
LinJ18_V3.0590	*L. infantum*	170	1	170
LinJ18_V3.0590	*L. infantum*	175	247	421
LinJ18_V3.0610	*L. infantum*	324	1	324
LinJ18_V3.0610	*L. infantum*	211	352	562
LinJ18_V3.0640	*L. infantum*	512	1	512
LinJ18_V3.0660	*L. infantum*	106	58	163
LinJ18_V3.0660	*L. infantum*	55	187	241
LinJ18_V3.0660	*L. infantum*	62	255	316
LinJ18_V3.0660	*L. infantum*	64	364	427
LinJ18_V3.0660	*L. infantum*	62	469	530
LinJ18_V3.0660	*L. infantum*	98	612	709
LinJ18_V3.0660	*L. infantum*	85	813	897
LinJ18_V3.0660	*L. infantum*	60	1000	1059
LinJ18_V3.0660	*L. infantum*	80	1194	1273
LinJ18_V3.0670	*L. infantum*	117	595	711
LinJ18_V3.0680	*L. infantum*	211	1	211
LinJ18_V3.0680	*L. infantum*	46	324	369
LinJ18_V3.0680	*L. infantum*	124	461	584
LinJ18_V3.0680	*L. infantum*	73	600	672
LinJ18_V3.0690	*L. infantum*	50	1	50
LinJ18_V3.0710	*L. infantum*	100	103	202
LinJ18_V3.0710	*L. infantum*	42	373	414
LinJ18_V3.0710	*L. infantum*	44	455	498
LinJ18_V3.0710	*L. infantum*	120	537	656
LinJ18_V3.0710	*L. infantum*	117	827	943
LinJ18_V3.0710	*L. infantum*	87	1001	1087
LinJ18_V3.0710	*L. infantum*	127	1163	1289
LinJ18_V3.0710	*L. infantum*	190	1291	1480
LinJ18_V3.0710	*L. infantum*	68	1489	1556
LinJ18_V3.0720	*L. infantum*	102	146	247
LinJ18_V3.0720	*L. infantum*	141	381	521
LinJ18_V3.0720	*L. infantum*	43	537	579

LinJ18_V3.0720	*L. infantum*	41	680	720
LinJ18_V3.0730	*L. infantum*	112	1	112
LinJ18_V3.0750	*L. infantum*	214	63	276
LinJ18_V3.0750	*L. infantum*	73	389	461
LinJ18_V3.0770	*L. infantum*	62	64	125
LinJ18_V3.0770	*L. infantum*	283	205	487
LinJ18_V3.0770	*L. infantum*	109	503	611
LinJ18_V3.0770	*L. infantum*	70	718	787
LinJ18_V3.0770	*L. infantum*	50	819	868
LinJ18_V3.0770	*L. infantum*	183	996	1178
LinJ18_V3.0770	*L. infantum*	242	1219	1460
LinJ18_V3.0770	*L. infantum*	93	1610	1702
LinJ18_V3.0780	*L. infantum*	41	146	186
LinJ18_V3.0790	*L. infantum*	53	65	117
LinJ18_V3.0790	*L. infantum*	64	134	197
LinJ18_V3.0800	*L. infantum*	52	68	119
LinJ18_V3.0820	*L. infantum*	78	30	107
LinJ18_V3.0820	*L. infantum*	57	156	212
LinJ18_V3.0820	*L. infantum*	62	446	507
LinJ18_V3.0850	*L. infantum*	166	24	189
LinJ18_V3.0850	*L. infantum*	62	261	322
LinJ18_V3.0850	*L. infantum*	102	359	460
LinJ18_V3.0850	*L. infantum*	86	641	726
LinJ18_V3.0850	*L. infantum*	55	771	825
LinJ18_V3.0850	*L. infantum*	299	845	1143
LinJ18_V3.0850	*L. infantum*	52	1229	1280
LinJ18_V3.0850	*L. infantum*	189	1449	1637
LinJ18_V3.0850	*L. infantum*	332	1655	1986
LinJ18_V3.0850	*L. infantum*	82	2119	2200
LinJ18_V3.0850	*L. infantum*	138	2234	2371
LinJ18_V3.0850	*L. infantum*	115	2440	2554
LinJ18_V3.0850	*L. infantum*	129	2663	2791
LinJ18_V3.0860	*L. infantum*	89	55	143
LinJ18_V3.0860	*L. infantum*	43	195	237
LinJ18_V3.0860	*L. infantum*	68	276	343
LinJ18_V3.0860	*L. infantum*	75	581	655
LinJ18_V3.0860	*L. infantum*	126	731	856
LinJ18_V3.0870	*L. infantum*	59	93	151
LinJ18_V3.0870	*L. infantum*	41	194	234
LinJ18_V3.0870	*L. infantum*	154	313	466
LinJ18_V3.0880	*L. infantum*	83	79	161
LinJ18_V3.0880	*L. infantum*	62	507	568
LinJ18_V3.0880	*L. infantum*	154	699	852
LinJ18_V3.0900	*L. infantum*	94	36	129
LinJ18_V3.0900	*L. infantum*	41	467	507
LinJ18_V3.0910	*L. infantum*	80	56	135
LinJ18_V3.0910	*L. infantum*	49	169	217

LinJ18_V3.0910	*L. infantum*	51	299	349
LinJ18_V3.0910	*L. infantum*	210	898	1107
LinJ18_V3.0920	*L. infantum*	59	1	59
LinJ18_V3.0920	*L. infantum*	84	410	493
LinJ18_V3.0920	*L. infantum*	58	517	574
LinJ18_V3.0930	*L. infantum*	116	86	201
LinJ18_V3.0930	*L. infantum*	623	277	899
LinJ18_V3.0940	*L. infantum*	44	145	188
LinJ18_V3.0940	*L. infantum*	122	1136	1257
LinJ18_V3.0950	*L. infantum*	215	28	242
LinJ18_V3.0950	*L. infantum*	63	292	354
LinJ18_V3.0950	*L. infantum*	63	292	354
LinJ18_V3.0960	*L. infantum*	40	202	241
LinJ18_V3.0960	*L. infantum*	45	337	381
LinJ18_V3.0960	*L. infantum*	45	337	381
LinJ18_V3.0970	*L. infantum*	929	1	929
LinJ18_V3.0970	*L. infantum*	129	1128	1256
LinJ18_V3.0970	*L. infantum*	94	1346	1439
LinJ18_V3.0970	*L. infantum*	114	1461	1574
LinJ18_V3.0970	*L. infantum*	401	1594	1994
LinJ18_V3.0980	*L. infantum*	191	1	191
LinJ18_V3.1000	*L. infantum*	41	1	41
LinJ18_V3.1020	*L. infantum*	52	1	52
LinJ18_V3.1020	*L. infantum*	126	72	197
LinJ18_V3.1020	*L. infantum*	201	210	410
LinJ18_V3.1020	*L. infantum*	69	423	491
LinJ18_V3.1020	*L. infantum*	56	505	560
LinJ18_V3.1020	*L. infantum*	176	568	743
LinJ18_V3.1020	*L. infantum*	347	860	1206
LinJ18_V3.1020	*L. infantum*	101	1236	1336
LinJ18_V3.1030	*L. infantum*	247	112	358
LinJ18_V3.1040	*L. infantum*	74	156	229
LinJ18_V3.1050	*L. infantum*	45	662	706
LinJ18_V3.1060	*L. infantum*	86	1	86
LinJ18_V3.1060	*L. infantum*	54	423	476
LinJ18_V3.1060	*L. infantum*	50	629	678
LinJ18_V3.1060	*L. infantum*	44	1134	1177
LinJ18_V3.1060	*L. infantum*	53	1381	1433
LinJ18_V3.1070	*L. infantum*	40	501	540
LinJ18_V3.1100	*L. infantum*	74	175	248
LinJ18_V3.1110	*L. infantum*	56	44	99
LinJ18_V3.1120	*L. infantum*	57	164	220
LinJ18_V3.1130	*L. infantum*	96	93	188
LinJ18_V3.1150	*L. infantum*	947	1	947
LinJ18_V3.1160	*L. infantum*	117	1	117
LinJ18_V3.1160	*L. infantum*	41	163	203
LinJ18_V3.1190	*L. infantum*	117	1	117

LinJ18_V3.1190	*L. infantum*	319	154	472
LinJ18_V3.1190	*L. infantum*	200	562	761
LinJ18_V3.1190	*L. infantum*	488	784	1271
LinJ18_V3.1200	*L. infantum*	52	108	159
LinJ18_V3.1200	*L. infantum*	55	177	231
LinJ18_V3.1200	*L. infantum*	56	471	526
LinJ18_V3.1200	*L. infantum*	50	990	1039
LinJ18_V3.1210	*L. infantum*	198	44	241
LinJ18_V3.1220	*L. infantum*	89	1	89
LinJ18_V3.1220	*L. infantum*	42	261	302
LinJ18_V3.1220	*L. infantum*	46	742	787
LinJ18_V3.1230	*L. infantum*	43	174	216
LinJ18_V3.1230	*L. infantum*	46	654	699
LinJ18_V3.1230	*L. infantum*	46	654	699
LinJ18_V3.1240	*L. infantum*	158	32	189
LinJ18_V3.1240	*L. infantum*	72	480	551
LinJ18_V3.1250	*L. infantum*	101	1	101
LinJ18_V3.1250	*L. infantum*	63	156	218
LinJ18_V3.1250	*L. infantum*	96	273	368
LinJ18_V3.1250	*L. infantum*	206	463	668
LinJ18_V3.1250	*L. infantum*	76	681	756
LinJ18_V3.1260	*L. infantum*	54	1	54
LinJ18_V3.1260	*L. infantum*	94	122	215
LinJ18_V3.1260	*L. infantum*	94	220	313
LinJ18_V3.1260	*L. infantum*	66	322	387
LinJ18_V3.1260	*L. infantum*	92	420	511
LinJ18_V3.1260	*L. infantum*	163	531	693
LinJ18_V3.1260	*L. infantum*	56	742	797
LinJ18_V3.1290	*L. infantum*	125	57	181
LinJ18_V3.1290	*L. infantum*	93	240	332
LinJ18_V3.1290	*L. infantum*	66	427	492
LinJ18_V3.1290	*L. infantum*	129	603	731
LinJ18_V3.1300	*L. infantum*	417	1	417
LinJ18_V3.1300	*L. infantum*	61	544	604
LinJ18_V3.1310	*L. infantum*	42	115	156
LinJ18_V3.1320	*L. infantum*	216	1	216
LinJ18_V3.1320	*L. infantum*	191	219	409
LinJ18_V3.1320	*L. infantum*	85	559	643
LinJ18_V3.1320	*L. infantum*	103	665	767
LinJ18_V3.1330	*L. infantum*	64	18	81
LinJ18_V3.1330	*L. infantum*	179	105	283
LinJ18_V3.1330	*L. infantum*	143	439	581
LinJ18_V3.1340	*L. infantum*	299	1	299
LinJ18_V3.1340	*L. infantum*	611	351	961
LinJ18_V3.1360	*L. infantum*	48	1	48
LinJ18_V3.1360	*L. infantum*	73	282	354
LinJ18_V3.1360	*L. infantum*	73	282	354

LinJ18_V3.1370	*L. infantum*	55	1	55
LinJ18_V3.1380	*L. infantum*	51	1	51
LinJ18_V3.1380	*L. infantum*	71	98	168
LinJ18_V3.1390	*L. infantum*	564	68	631
LinJ18_V3.1390	*L. infantum*	113	644	756
LinJ18_V3.1400	*L. infantum*	83	88	170
LinJ18_V3.1400	*L. infantum*	355	541	895
LinJ18_V3.1410	*L. infantum*	258	80	337
LinJ18_V3.1410	*L. infantum*	312	349	660
LinJ18_V3.1410	*L. infantum*	403	719	1121
LinJ18_V3.1430	*L. infantum*	866	1	866
LinJ18_V3.1430	*L. infantum*	65	953	1017
LinJ18_V3.1430	*L. infantum*	117	1153	1269
LinJ18_V3.1430	*L. infantum*	68	1363	1430
LinJ18_V3.1430	*L. infantum*	106	1486	1591
LinJ18_V3.1430	*L. infantum*	388	1660	2047
LinJ18_V3.1440	*L. infantum*	260	1	260
LinJ18_V3.1440	*L. infantum*	107	297	403
LinJ18_V3.1450	*L. infantum*	42	154	195
LinJ18_V3.1480	*L. infantum*	85	15	99
LinJ18_V3.1480	*L. infantum*	52	210	261
LinJ18_V3.1480	*L. infantum*	140	379	518
LinJ18_V3.1480	*L. infantum*	132	613	744
LinJ18_V3.1500	*L. infantum*	82	1	82
LinJ18_V3.1500	*L. infantum*	61	904	964
LinJ18_V3.1510	*L. infantum*	82	1	82
LinJ18_V3.1510	*L. infantum*	71	904	974
LinJ18_V3.1520	*L. infantum*	190	1	190
LinJ18_V3.1520	*L. infantum*	70	300	369
LinJ18_V3.1520	*L. infantum*	134	605	738
LinJ18_V3.1520	*L. infantum*	340	902	1241
LinJ18_V3.1540	*L. infantum*	65	213	277
LinJ18_V3.1550	*L. infantum*	45	170	214
LinJ18_V3.1590	*L. infantum*	42	1	42
LinJ18_V3.1590	*L. infantum*	85	324	408
LinJ18_V3.1590	*L. infantum*	219	578	796
LinJ18_V3.1600	*L. infantum*	154	1	154
LinJ18_V3.1600	*L. infantum*	45	610	654
LinJ18_V3.1630	*L. infantum*	72	49	120
LinJ18_V3.1650	*L. infantum*	62	76	137
LinJ18_V3.1650	*L. infantum*	47	468	514
LinJ18_V3.1650	*L. infantum*	47	468	514
LinJ18_V3.1670	*L. infantum*	59	35	93
LinJ18_V3.1670	*L. infantum*	180	128	307
LinJ18_V3.1670	*L. infantum*	69	402	470
LinJ18_V3.1670	*L. infantum*	58	492	549
LinJ19_V3.0010	*L. infantum*	200	1	200

LinJ19_V3.0020	*L. infantum*	87	1	87
LinJ19_V3.0020	*L. infantum*	154	104	257
LinJ19_V3.0030	*L. infantum*	54	1	54
LinJ19_V3.0050	*L. infantum*	48	1	48
LinJ19_V3.0060	*L. infantum*	163	1	163
LinJ19_V3.0060	*L. infantum*	56	276	331
LinJ19_V3.0070	*L. infantum*	375	58	432
LinJ19_V3.0080	*L. infantum*	64	1	64
LinJ19_V3.0080	*L. infantum*	54	154	207
LinJ19_V3.0080	*L. infantum*	52	243	294
LinJ19_V3.0090	*L. infantum*	60	1	60
LinJ19_V3.0100	*L. infantum*	247	1	247
LinJ19_V3.0110	*L. infantum*	144	55	198
LinJ19_V3.0120	*L. infantum*	41	213	253
LinJ19_V3.0130	*L. infantum*	211	351	561
LinJ19_V3.0160	*L. infantum*	90	122	211
LinJ19_V3.0160	*L. infantum*	50	461	510
LinJ19_V3.0160	*L. infantum*	100	1392	1491
LinJ19_V3.0170	*L. infantum*	118	290	407
LinJ19_V3.0180	*L. infantum*	87	1	87
LinJ19_V3.0180	*L. infantum*	46	118	163
LinJ19_V3.0220	*L. infantum*	55	1	55
LinJ19_V3.0220	*L. infantum*	44	203	246
LinJ19_V3.0230	*L. infantum*	109	1	109
LinJ19_V3.0230	*L. infantum*	48	446	493
LinJ19_V3.0230	*L. infantum*	59	614	672
LinJ19_V3.0240	*L. infantum*	102	244	345
LinJ19_V3.0240	*L. infantum*	74	657	730
LinJ19_V3.0250	*L. infantum*	40	1	40
LinJ19_V3.0250	*L. infantum*	49	145	193
LinJ19_V3.0250	*L. infantum*	158	261	418
LinJ19_V3.0260	*L. infantum*	432	1	432
LinJ19_V3.0260	*L. infantum*	309	491	799
LinJ19_V3.0270	*L. infantum*	197	435	631
LinJ19_V3.0280	*L. infantum*	49	37	85
LinJ19_V3.0280	*L. infantum*	143	141	283
LinJ19_V3.0280	*L. infantum*	55	467	521
LinJ19_V3.0280	*L. infantum*	52	552	603
LinJ19_V3.0280	*L. infantum*	87	661	747
LinJ19_V3.0280	*L. infantum*	44	939	982
LinJ19_V3.0280	*L. infantum*	59	1002	1060
LinJ19_V3.0280	*L. infantum*	48	1068	1115
LinJ19_V3.0280	*L. infantum*	150	1193	1342
LinJ19_V3.0290	*L. infantum*	96	124	219
LinJ19_V3.0300	*L. infantum*	59	1	59
LinJ19_V3.0300	*L. infantum*	77	169	245
LinJ19_V3.0300	*L. infantum*	101	292	392

LinJ19_V3.0310	*L. infantum*	40	99	138
LinJ19_V3.0320	*L. infantum*	42	1	42
LinJ19_V3.0320	*L. infantum*	60	181	240
LinJ19_V3.0320	*L. infantum*	57	402	458
LinJ19_V3.0340	*L. infantum*	108	84	191
LinJ19_V3.0350	*L. infantum*	44	1	44
LinJ19_V3.0350	*L. infantum*	86	52	137
LinJ19_V3.0350	*L. infantum*	48	273	320
LinJ19_V3.0360	*L. infantum*	110	71	180
LinJ19_V3.0360	*L. infantum*	201	214	414
LinJ19_V3.0360	*L. infantum*	49	426	474
LinJ19_V3.0360	*L. infantum*	78	479	556
LinJ19_V3.0360	*L. infantum*	284	572	855
LinJ19_V3.0360	*L. infantum*	253	860	1112
LinJ19_V3.0360	*L. infantum*	220	1381	1600
LinJ19_V3.0360	*L. infantum*	49	1705	1753
LinJ19_V3.0360	*L. infantum*	42	1776	1817
LinJ19_V3.0370	*L. infantum*	65	1	65
LinJ19_V3.0370	*L. infantum*	392	71	462
LinJ19_V3.0370	*L. infantum*	206	480	685
LinJ19_V3.0370	*L. infantum*	413	740	1152
LinJ19_V3.0390	*L. infantum*	56	1	56
LinJ19_V3.0400	*L. infantum*	46	154	199
LinJ19_V3.0410	*L. infantum*	44	1	44
LinJ19_V3.0420	*L. infantum*	58	58	115
LinJ19_V3.0430	*L. infantum*	51	44	94
LinJ19_V3.0430	*L. infantum*	140	155	294
LinJ19_V3.0430	*L. infantum*	73	303	375
LinJ19_V3.0430	*L. infantum*	52	470	521
LinJ19_V3.0430	*L. infantum*	51	1080	1130
LinJ19_V3.0440	*L. infantum*	50	1	50
LinJ19_V3.0440	*L. infantum*	68	216	283
LinJ19_V3.0440	*L. infantum*	53	298	350
LinJ19_V3.0450	*L. infantum*	591	55	645
LinJ19_V3.0450	*L. infantum*	87	699	785
LinJ19_V3.0450	*L. infantum*	60	817	876
LinJ19_V3.0460	*L. infantum*	188	53	240
LinJ19_V3.0470	*L. infantum*	106	91	196
LinJ19_V3.0470	*L. infantum*	96	264	359
LinJ19_V3.0480	*L. infantum*	91	1	91
LinJ19_V3.0490	*L. infantum*	81	1	81
LinJ19_V3.0500	*L. infantum*	106	27	132
LinJ19_V3.0500	*L. infantum*	140	136	275
LinJ19_V3.0500	*L. infantum*	140	136	275
LinJ19_V3.0510	*L. infantum*	152	1	152
LinJ19_V3.0510	*L. infantum*	109	193	301
LinJ19_V3.0510	*L. infantum*	209	320	528

LinJ19_V3.0510	*L. infantum*	129	607	735
LinJ19_V3.0510	*L. infantum*	119	822	940
LinJ19_V3.0510	*L. infantum*	48	969	1016
LinJ19_V3.0510	*L. infantum*	132	1026	1157
LinJ19_V3.0510	*L. infantum*	151	1176	1326
LinJ19_V3.0510	*L. infantum*	101	1505	1605
LinJ19_V3.0520	*L. infantum*	50	42	91
LinJ19_V3.0520	*L. infantum*	98	195	292
LinJ19_V3.0520	*L. infantum*	98	195	292
LinJ19_V3.0530	*L. infantum*	165	1	165
LinJ19_V3.0530	*L. infantum*	41	457	497
LinJ19_V3.0530	*L. infantum*	124	512	635
LinJ19_V3.0530	*L. infantum*	101	642	742
LinJ19_V3.0540	*L. infantum*	67	65	131
LinJ19_V3.0550	*L. infantum*	94	126	219
LinJ19_V3.0550	*L. infantum*	204	229	432
LinJ19_V3.0550	*L. infantum*	115	583	697
LinJ19_V3.0550	*L. infantum*	94	1071	1164
LinJ19_V3.0550	*L. infantum*	65	1204	1268
LinJ19_V3.0590	*L. infantum*	276	1	276
LinJ19_V3.0590	*L. infantum*	256	312	567
LinJ19_V3.0590	*L. infantum*	121	583	703
LinJ19_V3.0590	*L. infantum*	198	921	1118
LinJ19_V3.0590	*L. infantum*	128	1243	1370
LinJ19_V3.0590	*L. infantum*	42	1382	1423
LinJ19_V3.0590	*L. infantum*	107	1503	1609
LinJ19_V3.0600	*L. infantum*	449	1	449
LinJ19_V3.0600	*L. infantum*	59	451	509
LinJ19_V3.0600	*L. infantum*	69	517	585
LinJ19_V3.0600	*L. infantum*	55	723	777
LinJ19_V3.0620	*L. infantum*	64	236	299
LinJ19_V3.0620	*L. infantum*	51	430	480
LinJ19_V3.0620	*L. infantum*	47	608	654
LinJ19_V3.0630	*L. infantum*	57	1	57
LinJ19_V3.0650	*L. infantum*	40	299	338
LinJ19_V3.0650	*L. infantum*	55	899	953
LinJ19_V3.0650	*L. infantum*	42	983	1024
LinJ19_V3.0650	*L. infantum*	42	1205	1246
LinJ19_V3.0650	*L. infantum*	43	1278	1320
LinJ19_V3.0650	*L. infantum*	257	1478	1734
LinJ19_V3.0650	*L. infantum*	132	1800	1931
LinJ19_V3.0660	*L. infantum*	129	1	129
LinJ19_V3.0670	*L. infantum*	186	1	186
LinJ19_V3.0680	*L. infantum*	88	1	88
LinJ19_V3.0680	*L. infantum*	55	161	215
LinJ19_V3.0680	*L. infantum*	49	267	315
LinJ19_V3.0680	*L. infantum*	88	620	707

LinJ19_V3.0680	*L. infantum*	211	776	986
LinJ19_V3.0680	*L. infantum*	97	1031	1127
LinJ19_V3.0690	*L. infantum*	85	1	85
LinJ19_V3.0690	*L. infantum*	54	163	216
LinJ19_V3.0690	*L. infantum*	49	275	323
LinJ19_V3.0690	*L. infantum*	63	643	705
LinJ19_V3.0690	*L. infantum*	218	760	977
LinJ19_V3.0690	*L. infantum*	69	1031	1099
LinJ19_V3.0700	*L. infantum*	90	214	303
LinJ19_V3.0700	*L. infantum*	47	339	385
LinJ19_V3.0700	*L. infantum*	340	568	907
LinJ19_V3.0700	*L. infantum*	1338	943	2280
LinJ19_V3.0720	*L. infantum*	47	33	79
LinJ19_V3.0720	*L. infantum*	152	96	247
LinJ19_V3.0730	*L. infantum*	92	294	385
LinJ19_V3.0740	*L. infantum*	113	125	237
LinJ19_V3.0740	*L. infantum*	57	349	405
LinJ19_V3.0740	*L. infantum*	57	349	405
LinJ19_V3.0750	*L. infantum*	62	35	96
LinJ19_V3.0750	*L. infantum*	47	119	165
LinJ19_V3.0750	*L. infantum*	57	468	524
LinJ19_V3.0760	*L. infantum*	108	167	274
LinJ19_V3.0760	*L. infantum*	46	329	374
LinJ19_V3.0760	*L. infantum*	46	329	374
LinJ19_V3.0770	*L. infantum*	41	59	99
LinJ19_V3.0770	*L. infantum*	49	141	189
LinJ19_V3.0770	*L. infantum*	49	141	189
LinJ19_V3.0790	*L. infantum*	78	370	447
LinJ19_V3.0790	*L. infantum*	124	618	741
LinJ19_V3.0800	*L. infantum*	63	1	63
LinJ19_V3.0810	*L. infantum*	45	47	91
LinJ19_V3.0810	*L. infantum*	138	732	869
LinJ19_V3.0810	*L. infantum*	138	732	869
LinJ19_V3.0860	*L. infantum*	61	1	61
LinJ19_V3.0870	*L. infantum*	73	278	350
LinJ19_V3.0880	*L. infantum*	63	75	137
LinJ19_V3.0880	*L. infantum*	85	263	347
LinJ19_V3.0880	*L. infantum*	70	383	452
LinJ19_V3.0880	*L. infantum*	232	529	760
LinJ19_V3.0880	*L. infantum*	48	861	908
LinJ19_V3.0890	*L. infantum*	484	96	579
LinJ19_V3.0900	*L. infantum*	152	117	268
LinJ19_V3.0900	*L. infantum*	101	270	370
LinJ19_V3.0900	*L. infantum*	72	373	444
LinJ19_V3.0900	*L. infantum*	208	480	687
LinJ19_V3.0930	*L. infantum*	393	24	416
LinJ19_V3.0930	*L. infantum*	67	469	535

LinJ19_V3.0930	*L. infantum*	88	676	763
LinJ19_V3.0950	*L. infantum*	42	540	581
LinJ19_V3.0970	*L. infantum*	89	515	603
LinJ19_V3.0980	*L. infantum*	85	67	151
LinJ19_V3.0980	*L. infantum*	53	242	294
LinJ19_V3.0980	*L. infantum*	98	343	440
LinJ19_V3.0980	*L. infantum*	88	445	532
LinJ19_V3.0980	*L. infantum*	160	582	741
LinJ19_V3.0990	*L. infantum*	73	1	73
LinJ19_V3.0990	*L. infantum*	56	321	376
LinJ19_V3.0990	*L. infantum*	56	321	376
LinJ19_V3.1000	*L. infantum*	295	1	295
LinJ19_V3.1010	*L. infantum*	46	304	349
LinJ19_V3.1020	*L. infantum*	50	132	181
LinJ19_V3.1020	*L. infantum*	482	230	711
LinJ19_V3.1020	*L. infantum*	280	833	1112
LinJ19_V3.1020	*L. infantum*	225	1145	1369
LinJ19_V3.1030	*L. infantum*	111	14	124
LinJ19_V3.1030	*L. infantum*	259	208	466
LinJ19_V3.1030	*L. infantum*	68	519	586
LinJ19_V3.1030	*L. infantum*	86	647	732
LinJ19_V3.1030	*L. infantum*	54	743	796
LinJ19_V3.1030	*L. infantum*	178	835	1012
LinJ19_V3.1030	*L. infantum*	218	1039	1256
LinJ19_V3.1040	*L. infantum*	80	74	153
LinJ19_V3.1040	*L. infantum*	82	172	253
LinJ19_V3.1040	*L. infantum*	47	342	388
LinJ19_V3.1040	*L. infantum*	250	472	721
LinJ19_V3.1040	*L. infantum*	79	900	978
LinJ19_V3.1050	*L. infantum*	55	17	71
LinJ19_V3.1050	*L. infantum*	140	88	227
LinJ19_V3.1050	*L. infantum*	49	248	296
LinJ19_V3.1050	*L. infantum*	165	355	519
LinJ19_V3.1060	*L. infantum*	624	1	624
LinJ19_V3.1060	*L. infantum*	266	636	901
LinJ19_V3.1060	*L. infantum*	147	948	1094
LinJ19_V3.1060	*L. infantum*	76	1395	1470
LinJ19_V3.1060	*L. infantum*	211	1484	1694
LinJ19_V3.1060	*L. infantum*	97	1738	1834
LinJ19_V3.1060	*L. infantum*	60	2103	2162
LinJ19_V3.1060	*L. infantum*	42	2340	2381
LinJ19_V3.1060	*L. infantum*	90	2395	2484
LinJ19_V3.1070	*L. infantum*	55	142	196
LinJ19_V3.1080	*L. infantum*	758	1	758
LinJ19_V3.1080	*L. infantum*	93	779	871
LinJ19_V3.1080	*L. infantum*	81	917	997
LinJ19_V3.1080	*L. infantum*	178	1012	1189

LinJ19_V3.1080	*L. infantum*	105	1205	1309
LinJ19_V3.1110	*L. infantum*	44	42	85
LinJ19_V3.1110	*L. infantum*	163	284	446
LinJ19_V3.1110	*L. infantum*	43	619	661
LinJ19_V3.1110	*L. infantum*	78	687	764
LinJ19_V3.1110	*L. infantum*	58	805	862
LinJ19_V3.1110	*L. infantum*	63	928	990
LinJ19_V3.1110	*L. infantum*	62	1069	1130
LinJ19_V3.1110	*L. infantum*	42	1261	1302
LinJ19_V3.1110	*L. infantum*	52	1355	1406
LinJ19_V3.1110	*L. infantum*	59	1410	1468
LinJ19_V3.1110	*L. infantum*	153	1515	1667
LinJ19_V3.1110	*L. infantum*	98	1694	1791
LinJ19_V3.1110	*L. infantum*	55	1803	1857
LinJ19_V3.1110	*L. infantum*	45	2106	2150
LinJ19_V3.1110	*L. infantum*	82	2291	2372
LinJ19_V3.1110	*L. infantum*	41	2558	2598
LinJ19_V3.1110	*L. infantum*	57	2643	2699
LinJ19_V3.1110	*L. infantum*	116	2797	2912
LinJ19_V3.1110	*L. infantum*	64	2960	3023
LinJ19_V3.1110	*L. infantum*	41	3338	3378
LinJ19_V3.1110	*L. infantum*	101	3398	3498
LinJ19_V3.1110	*L. infantum*	51	3561	3611
LinJ19_V3.1110	*L. infantum*	135	3659	3793
LinJ19_V3.1110	*L. infantum*	44	3814	3857
LinJ19_V3.1110	*L. infantum*	60	4005	4064
LinJ19_V3.1110	*L. infantum*	56	4074	4129
LinJ19_V3.1110	*L. infantum*	199	4275	4473
LinJ19_V3.1110	*L. infantum*	130	4486	4615
LinJ19_V3.1110	*L. infantum*	74	4735	4808
LinJ19_V3.1110	*L. infantum*	113	4937	5049
LinJ19_V3.1110	*L. infantum*	43	5153	5195
LinJ19_V3.1110	*L. infantum*	104	5202	5305
LinJ19_V3.1110	*L. infantum*	158	5332	5489
LinJ19_V3.1110	*L. infantum*	122	5569	5690
LinJ19_V3.1120	*L. infantum*	189	47	235
LinJ19_V3.1130	*L. infantum*	248	71	318
LinJ19_V3.1130	*L. infantum*	52	322	373
LinJ19_V3.1130	*L. infantum*	97	474	570
LinJ19_V3.1130	*L. infantum*	42	604	645
LinJ19_V3.1130	*L. infantum*	66	691	756
LinJ19_V3.1140	*L. infantum*	691	82	772
LinJ19_V3.1140	*L. infantum*	125	777	901
LinJ19_V3.1140	*L. infantum*	645	995	1639
LinJ19_V3.1140	*L. infantum*	744	1648	2391
LinJ19_V3.1140	*L. infantum*	148	2397	2544
LinJ19_V3.1140	*L. infantum*	81	2598	2678

LinJ19_V3.1150	*L. infantum*	103	56	158
LinJ19_V3.1150	*L. infantum*	71	301	371
LinJ19_V3.1160	*L. infantum*	611	1	611
LinJ19_V3.1160	*L. infantum*	271	618	888
LinJ19_V3.1160	*L. infantum*	68	923	990
LinJ19_V3.1170	*L. infantum*	133	1	133
LinJ19_V3.1170	*L. infantum*	527	143	669
LinJ19_V3.1170	*L. infantum*	125	736	860
LinJ19_V3.1170	*L. infantum*	47	976	1022
LinJ19_V3.1170	*L. infantum*	203	1025	1227
LinJ19_V3.1170	*L. infantum*	141	1250	1390
LinJ19_V3.1170	*L. infantum*	330	1407	1736
LinJ19_V3.1200	*L. infantum*	48	1	48
LinJ19_V3.1210	*L. infantum*	95	138	232
LinJ19_V3.1220	*L. infantum*	54	57	110
LinJ19_V3.1220	*L. infantum*	164	159	322
LinJ19_V3.1230	*L. infantum*	137	111	247
LinJ19_V3.1230	*L. infantum*	56	353	408
LinJ19_V3.1230	*L. infantum*	64	622	685
LinJ19_V3.1230	*L. infantum*	69	874	942
LinJ19_V3.1230	*L. infantum*	100	1161	1260
LinJ19_V3.1230	*L. infantum*	202	1326	1527
LinJ19_V3.1230	*L. infantum*	88	1653	1740
LinJ19_V3.1240	*L. infantum*	55	87	141
LinJ19_V3.1240	*L. infantum*	47	156	202
LinJ19_V3.1240	*L. infantum*	40	368	407
LinJ19_V3.1250	*L. infantum*	65	47	111
LinJ19_V3.1260	*L. infantum*	106	122	227
LinJ19_V3.1290	*L. infantum*	40	104	143
LinJ19_V3.1290	*L. infantum*	41	159	199
LinJ19_V3.1290	*L. infantum*	41	159	199
LinJ19_V3.1300	*L. infantum*	241	31	271
LinJ19_V3.1300	*L. infantum*	279	293	571
LinJ19_V3.1300	*L. infantum*	180	589	768
LinJ19_V3.1300	*L. infantum*	75	874	948
LinJ19_V3.1320	*L. infantum*	113	1	113
LinJ19_V3.1320	*L. infantum*	40	686	725
LinJ19_V3.1320	*L. infantum*	40	686	725
LinJ19_V3.1330	*L. infantum*	59	75	133
LinJ19_V3.1340	*L. infantum*	59	75	133
LinJ19_V3.1350	*L. infantum*	52	1	52
LinJ19_V3.1350	*L. infantum*	59	168	226
LinJ19_V3.1350	*L. infantum*	59	168	226
LinJ19_V3.1360	*L. infantum*	52	1	52
LinJ19_V3.1360	*L. infantum*	59	168	226
LinJ19_V3.1360	*L. infantum*	59	168	226
LinJ19_V3.1370	*L. infantum*	48	145	192

LinJ19_V3.1370	*L. infantum*	56	446	501
LinJ19_V3.1370	*L. infantum*	56	446	501
LinJ19_V3.1380	*L. infantum*	71	323	393
LinJ19_V3.1400	*L. infantum*	52	23	74
LinJ19_V3.1400	*L. infantum*	43	122	164
LinJ19_V3.1400	*L. infantum*	43	122	164
LinJ19_V3.1410	*L. infantum*	284	101	384
LinJ19_V3.1410	*L. infantum*	70	631	700
LinJ19_V3.1420	*L. infantum*	42	1	42
LinJ19_V3.1420	*L. infantum*	46	172	217
LinJ19_V3.1420	*L. infantum*	64	460	523
LinJ19_V3.1420	*L. infantum*	62	552	613
LinJ19_V3.1450	*L. infantum*	70	164	233
LinJ19_V3.1450	*L. infantum*	74	704	777
LinJ19_V3.1450	*L. infantum*	74	704	777
LinJ19_V3.1470	*L. infantum*	76	211	286
LinJ19_V3.1490	*L. infantum*	62	205	266
LinJ19_V3.1490	*L. infantum*	46	324	369
LinJ19_V3.1490	*L. infantum*	46	324	369
LinJ19_V3.1510	*L. infantum*	42	1	42
LinJ19_V3.1510	*L. infantum*	46	344	389
LinJ19_V3.1510	*L. infantum*	46	344	389
LinJ19_V3.1520	*L. infantum*	53	191	243
LinJ19_V3.1550	*L. infantum*	154	1	154
LinJ19_V3.1550	*L. infantum*	93	175	267
LinJ19_V3.1550	*L. infantum*	45	384	428
LinJ19_V3.1550	*L. infantum*	44	578	621
LinJ19_V3.1550	*L. infantum*	109	677	785
LinJ19_V3.1610	*L. infantum*	65	174	238
LinJ19_V3.1610	*L. infantum*	40	250	289
LinJ19_V3.1610	*L. infantum*	40	250	289
LinJ19_V3.1620	*L. infantum*	41	73	113
LinJ19_V3.1620	*L. infantum*	55	477	531
LinJ19_V3.1620	*L. infantum*	49	550	598
LinJ19_V3.1630	*L. infantum*	49	1	49
LinJ19_V3.1650	*L. infantum*	48	261	308
LinJ19_V3.1670	*L. infantum*	91	439	529
LinJ19_V3.1680	*L. infantum*	1360	136	1495
LinJ19_V3.1680	*L. infantum*	147	1527	1673
LinJ19_V3.1680	*L. infantum*	40	1700	1739
LinJ19_V3.1690	*L. infantum*	1415	139	1553
LinJ20_V3.0020	*L. infantum*	59	354	412
LinJ20_V3.0030	*L. infantum*	50	1	50
LinJ20_V3.0040	*L. infantum*	85	265	349
LinJ20_V3.0050	*L. infantum*	53	51	103
LinJ20_V3.0050	*L. infantum*	47	114	160
LinJ20_V3.0050	*L. infantum*	47	114	160

LinJ20_V3.0070	*L. infantum*	263	23	285
LinJ20_V3.0070	*L. infantum*	56	570	625
LinJ20_V3.0080	*L. infantum*	40	1	40
LinJ20_V3.0100	*L. infantum*	61	39	99
LinJ20_V3.0140	*L. infantum*	157	288	444
LinJ20_V3.0150	*L. infantum*	138	1	138
LinJ20_V3.0150	*L. infantum*	73	635	707
LinJ20_V3.0170	*L. infantum*	40	712	751
LinJ20_V3.0180	*L. infantum*	129	539	667
LinJ20_V3.0200	*L. infantum*	75	53	127
LinJ20_V3.0200	*L. infantum*	49	171	219
LinJ20_V3.0200	*L. infantum*	191	273	463
LinJ20_V3.0210	*L. infantum*	40	32	71
LinJ20_V3.0210	*L. infantum*	61	98	158
LinJ20_V3.0210	*L. infantum*	61	98	158
LinJ20_V3.0220	*L. infantum*	69	1	69
LinJ20_V3.0220	*L. infantum*	76	243	318
LinJ20_V3.0220	*L. infantum*	76	243	318
LinJ20_V3.0240	*L. infantum*	44	373	416
LinJ20_V3.0250	*L. infantum*	66	1	66
LinJ20_V3.0270	*L. infantum*	73	1	73
LinJ20_V3.0280	*L. infantum*	42	1418	1459
LinJ20_V3.0280	*L. infantum*	42	1880	1921
LinJ20_V3.0280	*L. infantum*	99	2012	2110
LinJ20_V3.0280	*L. infantum*	44	2923	2966
LinJ20_V3.0280	*L. infantum*	53	3380	3432
LinJ20_V3.0280	*L. infantum*	54	3440	3493
LinJ20_V3.0280	*L. infantum*	92	3748	3839
LinJ20_V3.0280	*L. infantum*	547	3948	4494
LinJ20_V3.0280	*L. infantum*	45	4716	4760
LinJ20_V3.0290	*L. infantum*	60	94	153
LinJ20_V3.0290	*L. infantum*	42	221	262
LinJ20_V3.0290	*L. infantum*	42	221	262
LinJ20_V3.0300	*L. infantum*	67	1	67
LinJ20_V3.0300	*L. infantum*	98	107	204
LinJ20_V3.0300	*L. infantum*	63	211	273
LinJ20_V3.0310	*L. infantum*	52	1	52
LinJ20_V3.0320	*L. infantum*	40	164	203
LinJ20_V3.0320	*L. infantum*	69	408	476
LinJ20_V3.0320	*L. infantum*	57	718	774
LinJ20_V3.0350	*L. infantum*	97	65	161
LinJ20_V3.0350	*L. infantum*	183	302	484
LinJ20_V3.0360	*L. infantum*	74	29	102
LinJ20_V3.0360	*L. infantum*	91	104	194
LinJ20_V3.0370	*L. infantum*	75	169	243
LinJ20_V3.0370	*L. infantum*	84	306	389
LinJ20_V3.0370	*L. infantum*	84	306	389

LinJ20_V3.0380	*L. infantum*	57	71	127
LinJ20_V3.0400	*L. infantum*	108	212	319
LinJ20_V3.0400	*L. infantum*	130	603	732
LinJ20_V3.0410	*L. infantum*	273	37	309
LinJ20_V3.0410	*L. infantum*	137	328	464
LinJ20_V3.0410	*L. infantum*	137	328	464
LinJ20_V3.0430	*L. infantum*	53	296	348
LinJ20_V3.0430	*L. infantum*	45	433	477
LinJ20_V3.0430	*L. infantum*	45	433	477
LinJ20_V3.0440	*L. infantum*	68	319	386
LinJ20_V3.0440	*L. infantum*	73	459	531
LinJ20_V3.0440	*L. infantum*	151	581	731
LinJ20_V3.0440	*L. infantum*	57	1342	1398
LinJ20_V3.0450	*L. infantum*	47	220	266
LinJ20_V3.0450	*L. infantum*	63	274	336
LinJ20_V3.0450	*L. infantum*	63	274	336
LinJ20_V3.0470	*L. infantum*	55	160	214
LinJ20_V3.0470	*L. infantum*	51	298	348
LinJ20_V3.0470	*L. infantum*	60	416	475
LinJ20_V3.0470	*L. infantum*	84	485	568
LinJ20_V3.0480	*L. infantum*	472	47	518
LinJ20_V3.0480	*L. infantum*	806	538	1343
LinJ20_V3.0490	*L. infantum*	142	1	142
LinJ20_V3.0500	*L. infantum*	42	183	224
LinJ20_V3.0500	*L. infantum*	92	326	417
LinJ20_V3.0500	*L. infantum*	92	326	417
LinJ20_V3.0510	*L. infantum*	50	168	217
LinJ20_V3.0540	*L. infantum*	56	103	158
LinJ20_V3.0540	*L. infantum*	116	387	502
LinJ20_V3.0550	*L. infantum*	70	1	70
LinJ20_V3.0560	*L. infantum*	76	1	76
LinJ20_V3.0570	*L. infantum*	68	69	136
LinJ20_V3.0570	*L. infantum*	74	210	283
LinJ20_V3.0570	*L. infantum*	74	210	283
LinJ20_V3.0580	*L. infantum*	52	14	65
LinJ20_V3.0580	*L. infantum*	62	329	390
LinJ20_V3.0580	*L. infantum*	62	329	390
LinJ20_V3.0590	*L. infantum*	359	117	475
LinJ20_V3.0590	*L. infantum*	86	502	587
LinJ20_V3.0590	*L. infantum*	57	702	758
LinJ20_V3.0610	*L. infantum*	44	1	44
LinJ20_V3.0610	*L. infantum*	122	111	232
LinJ20_V3.0610	*L. infantum*	122	111	232
LinJ20_V3.0620	*L. infantum*	43	71	113
LinJ20_V3.0620	*L. infantum*	66	167	232
LinJ20_V3.0620	*L. infantum*	43	486	528
LinJ20_V3.0640	*L. infantum*	85	104	188

LinJ20_V3.0640	*L. infantum*	44	293	336
LinJ20_V3.0640	*L. infantum*	273	456	728
LinJ20_V3.0640	*L. infantum*	71	762	832
LinJ20_V3.0650	*L. infantum*	146	1	146
LinJ20_V3.0650	*L. infantum*	120	312	431
LinJ20_V3.0660	*L. infantum*	67	63	129
LinJ20_V3.0660	*L. infantum*	211	179	389
LinJ20_V3.0660	*L. infantum*	71	507	577
LinJ20_V3.0680	*L. infantum*	91	69	159
LinJ20_V3.0680	*L. infantum*	61	228	288
LinJ20_V3.0680	*L. infantum*	194	316	509
LinJ20_V3.0680	*L. infantum*	448	540	987
LinJ20_V3.0680	*L. infantum*	212	1011	1222
LinJ20_V3.0680	*L. infantum*	103	1388	1490
LinJ20_V3.0690	*L. infantum*	314	1	314
LinJ20_V3.0690	*L. infantum*	153	320	472
LinJ20_V3.0690	*L. infantum*	703	517	1219
LinJ20_V3.0700	*L. infantum*	160	1	160
LinJ20_V3.0700	*L. infantum*	60	182	241
LinJ20_V3.0700	*L. infantum*	86	524	609
LinJ20_V3.0700	*L. infantum*	343	709	1051
LinJ20_V3.0710	*L. infantum*	65	143	207
LinJ20_V3.0710	*L. infantum*	63	389	451
LinJ20_V3.0720	*L. infantum*	45	106	150
LinJ20_V3.0720	*L. infantum*	40	560	599
LinJ20_V3.0720	*L. infantum*	40	560	599
LinJ20_V3.0730	*L. infantum*	41	143	183
LinJ20_V3.0750	*L. infantum*	61	69	129
LinJ20_V3.0750	*L. infantum*	146	160	305
LinJ20_V3.0750	*L. infantum*	177	438	614
LinJ20_V3.0750	*L. infantum*	174	666	839
LinJ20_V3.0750	*L. infantum*	498	841	1338
LinJ20_V3.0760	*L. infantum*	184	117	300
LinJ20_V3.0760	*L. infantum*	448	621	1068
LinJ20_V3.0770	*L. infantum*	160	53	212
LinJ20_V3.0770	*L. infantum*	139	440	578
LinJ20_V3.0770	*L. infantum*	58	641	698
LinJ20_V3.0770	*L. infantum*	477	826	1302
LinJ20_V3.0770	*L. infantum*	316	1308	1623
LinJ20_V3.0780	*L. infantum*	239	1	239
LinJ20_V3.0780	*L. infantum*	485	282	766
LinJ20_V3.0780	*L. infantum*	150	791	940
[truncated: 353,191 more chars]
